# Leaf Proteome Analysis Reveals Prospective Drought and Heat Stress Response Mechanisms in Soybean

**DOI:** 10.1155/2016/6021047

**Published:** 2016-03-13

**Authors:** Aayudh Das, Moustafa Eldakak, Bimal Paudel, Dea-Wook Kim, Homa Hemmati, Chhandak Basu, Jai S. Rohila

**Affiliations:** ^1^Department of Biology & Microbiology, South Dakota State University, Brookings, SD 57007, USA; ^2^Department of Biochemistry and Biophysics, Texas A&M University, College Station, TX 77840, USA; ^3^National Institute of Crop Science, Rural Development Administration (RDA), Wanju-gun, Jeollabuk-do 55365, Republic of Korea; ^4^Department of Biology, California State University Northridge, Northridge, CA 91330, USA

## Abstract

Drought and heat are among the major abiotic stresses that affect soybean crops worldwide. During the current investigation, the effect of drought, heat, and drought plus heat stresses was compared in the leaves of two soybean varieties, Surge and Davison, combining 2D-DIGE proteomic data with physiology and biochemical analyses. We demonstrated how 25 differentially expressed photosynthesis-related proteins affect RuBisCO regulation, electron transport, Calvin cycle, and carbon fixation during drought and heat stress. We also observed higher abundance of heat stress-induced EF-Tu protein in Surge. It is possible that EF-Tu might have activated heat tolerance mechanisms in the soybean. Higher level expressions of heat shock-related protein seem to be regulating the heat tolerance mechanisms. This study identifies the differential expression of various abiotic stress-responsive proteins that regulate various molecular processes and signaling cascades. One inevitable outcome from the biochemical and proteomics assays of this study is that increase of ROS levels during drought stress does not show significant changes at the phenotypic level in Davison and this seems to be due to a higher amount of carbonic anhydrase accumulation in the cell which aids the cell to become more resistant to cytotoxic concentrations of H_2_O_2_.

## 1. Introduction

Soybeans are one of the most important legume crops and have a major impact on the US and global economies. International markets use about half of the soybeans produced in the US, and its production is expected to alleviate the global demand for human consumption, biofuel production, and high-protein meal for animal feed [1]. In 2011, the total soybean production in the US was 83.29 × 10^12^ Kg with a total trade value of $40.2 billion [2, 3]. Heat and drought are the predominant abiotic stress factors that limit the growth and development of soybean plants by causing a reduction in carbon fixation by the photosynthetic apparatus of the plants, resulting in net yield losses [4]. At the cellular level, plants exhibit a variety of responses related to their physiology and biochemistry to overcome the stress. Drought stress causes reduced carbon assimilation due to stomatal closure, membrane damage, and distressed activity of various CO_2_ fixation enzymes [5]. Heat stress increases membrane damage and impairs metabolic functions [6–8]. As a result, combination of drought and heat stress causes enormous economic losses for farmers [9].

Proteomic evaluation for the purpose of differentially regulated proteins identification in response to the drought, heat, and the combined stress has been monitored in various plants in context of the morphological and physiological changes [10–12]. Particularly, changes in proteomic expression during drought stress have been observed in major crops like rice and wheat which shows differential regulation of various proteins [13–15]. Earlier studies using proteomic and transcriptomic approaches also implicated the drought stress response mechanisms including alteration in the signal transduction pathway and plant's metabolism [16–19]. Moreover, in response to heat stress, many stress proteins were originally identified as HSP which indicates plant's tolerance threshold [20]. To improve the understanding of the mechanisms underlying soybean responses to drought and heat stress, in the present investigation we have used a global proteomic approach combined with physiological and computational analysis [10, 21, 22]. The two major soybean varieties, Surge (yield 3,272 Kg/ha) and Davison (yield 4,074 Kg/ha), showing distinct physiological characteristics were used in this study. Proteomic analysis revealed differentially expressed proteins related to key biological processes (such as photosynthesis and respiration), various metabolic pathways (nitrogen metabolism and carbohydrate metabolism), and several other molecular processes (protein biosynthesis and ATP synthesis). Computational analysis predicted a protein network displaying the likely interactions between protein abundances and plant stress responses. These analyses will enable the development of plant molecular improvement programs to produce more effective strategies contributing to greater food security in the coming years. Since the proteins are the translated version of mRNA (proteomics approach may have advantage over transcriptomics approach [4]) several research groups have utilized the power of proteomic evaluations and combined it with physiological studies to create a link between plant physiology and the molecular signatory responses [10, 23]. The primary aim of this study was to investigate differential protein expression between two contrasting genotypes for drought and heat stress responses, and to reveal the drought and heat stress-responsive mechanisms in soybean at early stage of growth. Based on physiological analyses, we found that drought and heat stress decrease photosynthesis and reduce stomatal conductance and transpiration rates, which ultimately alter the net CO_2_ concentrations in leaves. The proteomic analyses facilitated the characterization of a set of proteins whose expressions were altered under heat and drought stresses. We found that, in Surge, more proteins were downregulated during drought stress than during heat stress, but the combined stress conditions exerted a drastic effect at both the molecular and the phenotypic levels. In this study, we have identified genes involved in photosynthesis that were differentially expressed during drought and heat stress conditions. This differential expression is most likely the result of blocked interactions between RuBisCO, RuBisCO activase, electron transport chain, and downregulation of various Calvin cycle enzymes, which ultimately results in a net reduced level of photosynthesis [24]. This study identified greater accumulations of EF-Tu protein during heat stress in Surge. The results also indicated the accumulation of a 70 kDa stromal HSP, which we assume is crucial for plant survival during heat stress by activating heat tolerance responses [25]. Previously, it has been reported that drought and heat stresses may trigger the formation of reactive oxygen species (ROS) [26, 27]. Likewise, we also examined the drought and heat stress-induced changes in the production of ROS and found that, during drought stress, soybeans exhibited a significant increase in the level of hydrogen peroxide and an enhanced ROS detoxification capacity via carbonic anhydrase, which protects the plant against oxidative damage [28]. Plants use ABA as a signaling molecule while maintaining a positive water balance throughout the system, as physiological studies have shown [29–31]. Elevated ABA levels during heat stress are shown to protect the maize plant against heat-induced oxidative damage [32]. We quantified the ABA levels in soybean leaves during all the stress conditions and have discussed it in drought and heat stress contexts for soybean crop. At the level of computational analysis, we have constructed a protein-protein network to predict the interactions between the differentially expressed proteins [17].

## 2. Materials and Methods 

### 2.1. Plant Growth Conditions

Soybean (*Glycine max* L. cultivars: Surge and Davison) seeds were planted in pots filled with Metromix 360 (Sun Gro Horticulture, Floodwood, Minnesota, USA). A total of eight, 6.0-liter size pots, each containing 4-5 individual soybean plants (biological replications), were placed in a growth chamber (Conviron, Canada) illuminated with white fluorescent light and incandescent bulbs (500 *µ*moles m^−2^ s^−1^, 12 h photoperiod) at 25°C and 70% RH for 3 weeks. At this stage, 8 pots from each cultivar were separated into 4 groups (control, drought, heat, and drought plus heat; Figure S1 in Supplementary Material available online at http://dx.doi.org/10.1155/2016/6021047). Watering was done daily with 150 mL tap water per pot until the second trifoliate leaves emerged, after which various stress treatments were carried out. The first group was the control, and it was maintained under the conditions described above. For the second group, the plants were exposed to drought stress (no watering for 7 days). The third group contained plants that were exposed to heat stress (42°C daytime/35°C night time, 50% humidity) with normal watering schedules. The fourth group contained plants that were exposed to drought plus heat stress. Soil moisture sensors (SM 100, Spectrum Technologies) were inserted into all of the pots on the day the plants were exposed to abiotic stress to measure the % VWC for each treatment. The % VWC value was measured throughout the entire experimental period using a microstation data logger [33].

### 2.2. Measurement of Photosynthesis, Stomatal Conductance, Transpiration, and Leaf Water Potential

Photosynthesis, stomatal conductance, and the transpiration rate were measured, every day at noon, on the first and second trifoliate stage leaves for all the plants in each group and with three replications (following the rationale from previous published drought stress time point studies [34, 35]). We used CI-510 Photosynthesis System (CID Bio-Science) with the following parameters: leaf chamber area: 6.25 cm^2^; flow rate: 0.3; time interval: 1 second; delay: 2 seconds; system: open; temperature: 25°C, as per the manufacturer's recommendations. Leaf water potential was measured using a pressure chamber (PMS Instrument) following the manufacturer's recommendations.

### 2.3. Sample Preparation for 2D-DIGE

Leaf samples collected from all treatments were snap-frozen under liquid nitrogen and stored in −80°C freezer until further use. Total protein was isolated according to the procedure of Hurkman and Tanaka [36]. Ground soybean leaf tissue (500 mg) was homogenized in 5 mL of 2D cell lysis buffer (30 mM Tris-HCl, pH 8.8, 0.9 M sucrose, 10 mM EDTA, 0.4% 2-mercaptoethanol, 7 M urea, 2 M thiourea, and 4% CHAPS) and an equal volume of Tris-saturated phenol, vortexed for 30 seconds and incubated for 30 min at 4°C. Following homogenization and mixing, centrifugation was carried out for 15 min at 4°C at 6,000 rpm, and the phenol phase was collected in a fresh tube. Now, the proteins in the collected phase were precipitated overnight by adding 5 volumes of ice-cold 0.1 M ammonium acetate in 100% methanol at −20°C. Next day, after 20 min of centrifugation at 10,000 rpm, the protein pellet was washed in 5 mL of 0.1 M ammonium acetate in 100% methanol followed by a wash in 5 mL of ice-cold 80% acetone, and then a final wash in 4 mL of 70% ethanol. The protein pellet was air-dried and stored at −80°C until downstream experiments. Protein quantification assays were performed using the Bio-Rad reagent (catalog# 500-0006) on the Bio-Rad SmartSpec Plus spectrophotometer.

### 2.4. 2D-DIGE, Trypsin Digestion, and MALDI-TOF MS Analysis

2D-DIGE analysis was performed by Applied Biomics (Hayward, CA, USA). For each sample set (either Surge or Davison with respective treatments, Figure S1), 30 *μ*g of sample protein was mixed with 1.0 *μ*L of diluted Cy3 or Cy5 dye (1 : 5 dilution in a 1 nmol *μ*L^−1^ DMF stock) for labeling. A pooled protein sample containing equal amounts of all samples in the experiment was labeled with Cy2 using the same protocol. After vortexing, the tubes were incubated in the dark for 30 min on ice. Next, 1.0 *μ*L of 10 mM lysine was added to each sample. After that, the samples were vortexed and incubated in the dark for 15 min on ice. Next, the Cy2, Cy3, and Cy5 labeled samples were mixed by adding 2X 2D sample buffer (Bio-Rad), followed by the addition of 100 *μ*L of DeStreak solution and a rehydration buffer (Bio-Rad) up to 350 *μ*L for the 13 cm gradient (pH 4–7) IPG strip. Upon completion of IEF, the IPG strips were incubated in freshly made equilibration buffers I and II (Bio-Rad) for 15 minutes with gentle shaking. The IPG strips were rinsed in Tris-glycine-SDS running buffer (Bio-Rad) and then transferred to 12% SDS-polyacrylamide gels, followed by sealing with 0.5% agarose solution for second-dimension electrophoresis. The SDS gels were electrophoresed at 175 volts at 15°C for 8 hrs. Gel images were scanned using Typhoon TRIO (GE Healthcare, PA, USA) and were analyzed using Image QuantTL software (version 6.0, GE Healthcare, USA) and then subjected to in-gel analysis and cross-gel analysis using DeCyder software, version 6.5 (GE Healthcare). The differential expression of the proteins was obtained from in-gel DeCyder software analysis [37]. This way, a total of 12 gels (Table S1) were run comprising protein samples from 8 different sets of treatments (Figure S1) and 3 replications each. The spots of interest were selected based on the in-gel analysis and statistical analyses (Table S2), *p* value of ≤0.1 and cut-off value of 1.5-fold, and were picked up using the Ettan Spot Picker (GE Healthcare). The picked gel spots were washed and digested in-gel using modified porcine trypsin protease (Trypsin Gold, Promega). The digested tryptic peptides were desalted using Zip-tip C18 (Millipore) and spotted on the MALDI plate. MALDI-TOF MS and TOF/TOF tandem MS were performed on a 5800 mass spectrometer (AB Sciex). The MALDI-TOF mass spectra were generated in the reflectron positive ion mode, and TOF/TOF tandem MS fragmentation spectra were acquired for each sample. On average, 4,000 laser shots per fragmentation spectrum were applied to each of the 5–10 most abundant ions present in each sample, excluding the trypsin autolytic peptides and other known background ions [38]. Both the resulting peptide mass and the associated fragmentation spectra were submitted to the MASCOT (version 2.4, Matrix Sciences, UK) in October 2014 to search the SwissProt database with 546,790 sequences. The searches were performed following the details of Poschmann et al. [39], for example, without constraining the protein MW or the pI value and with variable carbamidomethylation of cysteine and oxidation of methionine residues, a mass tolerance of 100 ppm, and allowing only one missed and/or nonspecific cleavage. Proteins with a protein score > 62 were considered to be assigned correctly. Candidates with either a protein score of CI % or an ion score of CI % greater than 95 were considered to be statistically significant. The protein peptide summary for all identified spots is listed in Supplementary Material Table S4.

### 2.5. ROS Measurement (Hydrogen Peroxide Quantification)

The levels of H_2_O_2_ in leaves were measured using the Amplex® Red Hydrogen Peroxide/Peroxidase Assay Kit (Molecular Probes, catalog# A-22188) [40]. For this assay, manufacturer's protocol was followed. Briefly, 50 mg of the ground leaf powder was mixed with reaction buffer (pH 7.4), vortexed, and incubated on ice for 15 min. Then, the samples were centrifuged at 10,000 rpm for 10 min at 4°C, and the supernatant was collected. Next, 50 *µ*L of the samples was loaded into a multiwell plate (clear bottom, black sides) followed by the addition of 50 *µ*L of 100 *μ*M Amplex® Red reagent and 0.2 U mL^−1^ HRP. The samples were incubated in the dark for 30 min at room temperature. Afterward, the fluorescence was measured at an emission wavelength of 620 nm using a Synergy 2 multimode microplate reader (BioTek); the blank value (buffer only) was subtracted from the sample's florescence values. Then, based on the standard curve, the H_2_O_2_ concentration was calculated in terms of nmol *µ*L^−1^ sample [41].

### 2.6. Quantification of Abscisic Acid

ABA quantification was performed as outlined by Kim et al. [42]. Two hundred mg of ground leaf powder was suspended in 1 mL of sterile deionized water and incubated overnight at 4°C with constant shaking. The solution was centrifuged at 4,000 rpm for 20 min. Then, the supernatant was transferred to a clean microfuge tube ensuring that no leaf pieces were transferred. The solution then was dried completely using a vacuum concentrator. The dried precipitate was resuspended with 60 *µ*L sterile deionized water and a 1 : 1000 dilution of the sample was made with TBS. ABA concentration was determined using a Phytodetek® ABA Test Kit (Agdia, USA) following the manufacturer's recommendations. A standard curve was generated using ABA standards (100, 20, 4, 0.8, 0.16, and 0.32 pmoles mL^−1^). 100 *µ*L of diluted sample per standard was loaded onto an ABA-antibody coated multiwell plate, and the ABA concentration was determined according to manufacturer's guidelines. The absorbance values at 405 nm were read using Spectromax M3 (Molecular Devices). The ABA concentration was calculated using the slope of the standard curve based on the manufacturer's recommendations.

### 2.7. Computational Analysis and Protein-Protein Interaction Predictions

First, we mapped protein ID (using UniProt database) to the* Arabidopsis *accession numbers and performed a multiple sequence alignment of* Arabidopsis *homologs using CLUSTALO [43] to obtain the protein identities. To search the variety of functional connections between the 44 differentially expressed proteins, we used the online database resource STRING (http://string-db.org/) version 9.1 [44]. Then, after obtaining the primary interactions in STRING, we portrayed our different protein networks using Cytoscape software, version 3.0.1 [45].

### 2.8. Clustering and Statistical Analyses

For the clustering data, the log_2_-transformed expression values of the protein spots were used. Hierarchical clustering of the proteins was performed using Gene Cluster 3.0 [46] with Euclidean distance similarity metrics and the complete linkage method. The clusters were visualized using JAVA TREEVIEW [47]. The statistical significance of the results was evaluated using Student's *t*-test and a level of significance of *p* ≤ 0.05 for the two group comparisons. Data analyses and graphical representations were performed using Microsoft Excel 2013.

## 3. Results 

Understanding the mechanisms by which plants respond to drought, heat, and cooccurring drought and heat stresses plays a major role in optimizing crop performance under drought and high temperature conditions [16]. Figure S2 shows the phenotypic changes between soybean plants on the sixth day of the stress experiment.

### 3.1. Soil Moisture Content and Physiological Responses of Plants to Different Abiotic Stresses

**Figure 1:**
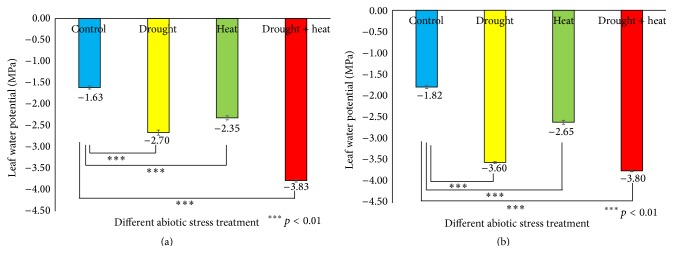
Bar diagrams showing the effect of various abiotic stresses on leaf water potential in two soybean cultivars Surge (a) and Davison (b). *x*-axis defines various abiotic stress treatments (drought, heat, and drought + heat) including control and *y*-axis defines leaf water potential in MPa (megapascal unit). Each value represents the mean ± SE of three replicates and the asterisks designate the significance of changes from their corresponding control (^*∗∗∗*^ *p* < 0.01).

To keep track of the soil moisture levels during the stress experiments, the soil water content was measured [48]. Figure S3 shows the soil moisture levels from Day 0 (prestress) to the sixth day of stress treatment. The leaf water potential is considered as a reliable parameter for quantifying the plant water stress response [49]. It was found (Figure 1) that, in Surge, stress reduced the leaf water potential from −1.63 MPa (control plants) to −2.70 MPa in drought-stressed plants, −2.35 MPa in heat-stressed plants, and −3.83 MPa in drought plus heat-stressed plants on the sixth day of stress. In Davison, we found that stress reduced the leaf water potential from −1.82 MPa (control plants) to −3.60 MPa in drought-stressed plants, −2.65 MPa in heat-stressed plants, and −3.80 MPa in drought plus heat-stressed plants, on the sixth day of stress.

**Figure 2:**
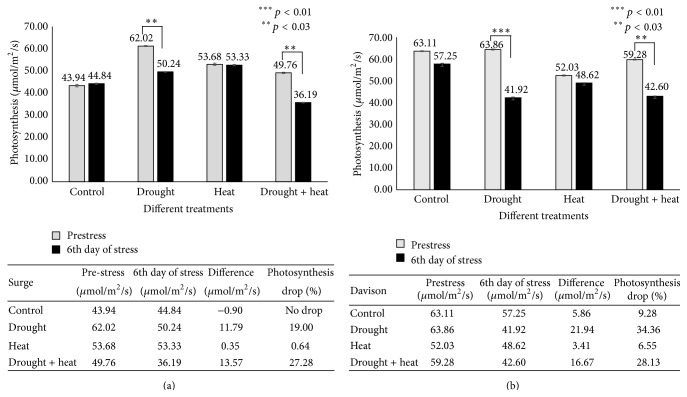
Bar diagram showing the effect of various abiotic stresses on photosynthesis in two soybean cultivars Surge (a) and Davison (b). *x*-axis defines various abiotic stress treatments (drought, heat, and drought + heat) including control and *y*-axis defines measurement of net photosynthesis (*μ*mol/m^2^/s). Each value represents the mean ± SE of three replicates and the asterisks designate the significance of changes from their resultant control (^*∗∗∗*^ *p* < 0.01, ^*∗∗*^ *p* < 0.03).

Photosynthesis is among the primary processes affected by drought and heat stresses [50, 51]. In Surge control plants (Figure 2), it was found that there was no decrease in photosynthesis, whereas in drought-stressed plants a 19% decrease in photosynthesis was detected on the sixth day of stress. Interestingly, in heat-stressed plants, we found that the level of photosynthesis was identical to prestress condition; and in drought plus heat-stressed plants, we detected a 27.28% decrease in photosynthesis on the sixth day of stress compared to prestress (Day 0) conditions. In the Davison control plants, there was a 9% decrease in photosynthesis most likely due to a developmental effect and the water use efficiency of the plant on that particular day; in drought-stressed plants, there was a 34.36% decrease in photosynthesis; in heat-stressed plants, photosynthesis decreased to 6.55%; and in drought plus heat-stressed plants, we detected a huge decrease (28.13%) in photosynthesis on the sixth day of stress compared to prestress (Day 0) conditions.

**Figure 3:**
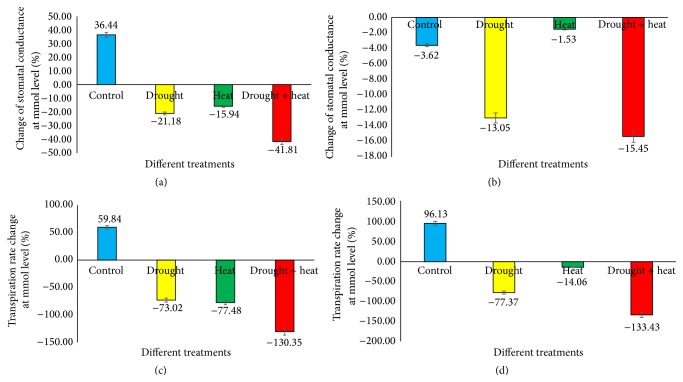
Bar diagram showing the comparison of stomatal conductance and transpiration rate profiles between Day 0 (prestress) and Day 6 under various stress conditions in two soybean cultivars Surge ((a), (c)) and Davison ((b), (d)). *x*-axis defines various abiotic stress treatments (drought, heat, and drought + heat) including control and *y*-axis defines percentage change of stomatal conductance at mmol level. Each value represents the mean ± SE of three replicates.

Several studies have suggested changes in the transpiration rate or stomatal conductance in response to different stress conditions. Altering stomatal conductance causes a fluctuation in the leaf water potential by shifting the transpiration rate [52]. The results (Figure 3) revealed that, in Surge control plants, stomatal conductance was increased by 36.44% (at the same time, the transpiration rate increased by 59.84%), whereas in drought-stressed plants stomatal conductance was decreased by 21.18% (while the transpiration rate decreased by 73.02%) on the sixth day of stress compared to prestress (Day 0) conditions. In heat-stressed plants, stomatal conductance decreased by 15.94% (the transpiration rate decreased by 77.48%); and in drought plus heat-stressed plants, stomatal conductance decreased by 41.81% (the transpiration rate decreased by 130.35%) on the sixth day of stress compared to prestress (Day 0) conditions. In Davison control plants, we found that stomatal conductance decreased by 3.62% (the transpiration rate increased by 96.13%); in drought-stressed plants, stomatal conductance decreased by 13.05% (the transpiration rate decreased by 77.37%); in heat-stressed plants, stomatal conductance decreased by 1.53% (the transpiration rate decreased by 14.06%); and in cooccurring drought plus heat-stressed plants, stomatal conductance decreased by 15.45% (the transpiration rate decreased by 133.43%) on the sixth day of stress compared to prestress (Day 0) conditions.

**Figure 4:**
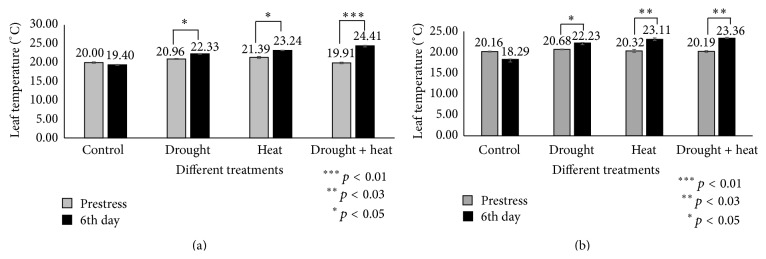
Bar diagrams showing temperature profiles of leaves between Day 0 (prestress) and Day 6 in two soybean cultivars Surge (a) and Davison (b). *x*-axis defines various abiotic stress treatments (drought, heat, and drought + heat) including control and *y*-axis defines measurement of leaf temperature OC. Each value represents the mean ± SE of three replicates and the asterisks designate the significance of changes from their subsequent control (^*∗∗∗*^ *p* < 0.01, ^*∗∗*^ *p* < 0.03, and ^*∗*^ *p* < 0.05).

Stomatal conductance also depends on the leaf temperature via the transpiration rates [52]. The analysis of present investigation indicated that leaf temperature increased in a nonproportional manner with stomatal conductance (Figure 4). We found that stomatal conductance reduced under all conditions in which the leaf temperature increased.

### 3.2. 2D-DIGE Analysis Followed by MALDI-TOF MS to Identify the Differentially Expressed Proteins in Response to Drought and Heat Stress

**Figure 5:**
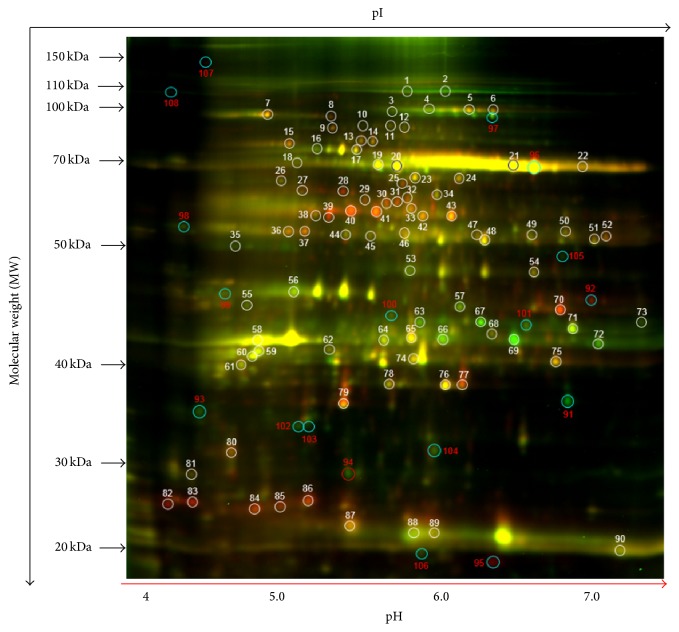
A representative figure showing 2D-DIGE analysis of differentially expressed soybean leaf proteins in response to heat, drought, and drought plus heat stresses. For each sample set, 30 *μ*g of sample protein was mixed with 1.0 *μ*L of diluted Cy3 or Cy5 dye for labeling. A pooled protein sample containing equal amounts of all samples was labeled with Cy2. Labeled samples were subjected to isoelectric focusing (pH 4–7) followed by 12% SDS-polyacrylamide gels. Gel images were scanned using Typhoon TRIO (GE Healthcare, PA, USA) and were analyzed using Image QuantTL software (version 6.0, GE Healthcare, USA) and then subjected to in-gel analysis and cross-gel analysis using DeCyder software, version 6.5 (GE Healthcare). The spot numbering on the merged 2D-DIGE image indicates the protein spots that were found statistically significant between various treatments for mass spectrometric identification.

The comparison of the leaf proteins from the two soybean cultivars among the control, heat, drought, and drought plus heat stress conditions via 2D-DIGE analysis revealed a broad distribution in the pI range and in the molecular weight range.  Figure 5 is a representative image of master gel, and supplemental Figures S4 and S5 show the Cy2/Cy3/Cy5 overlay images of all 12 gels run during the experiment. The protein spots were selected from the preparative gels for protein identification. Among the control and three treated groups, based on in-gel analysis, on average a total of 2600 spots were detected using DeCyder software. Of these, on average a total of 1,900–2,000 protein spots were matched among all other gels (Table SI); from here, a total of 108 spots were selected based on statistical analyses (*p* values; Table S2), and 92 differential spots were successfully identified using a threshold of significance of *p* ≤ 0.1, and with a cut-off value of 1.5-fold increase/decrease in protein expressions. Of these identified spots, we determined the protein identity of 88 spots with high confidence (Tables S3 and S4), corresponding to 44 nonredundant differentially expressed proteins in both soybean varieties exposed to different stress conditions.

#### 3.2.1. Genotypic Comparison at Molecular Levels for Different Stresses

**Figure 6:**
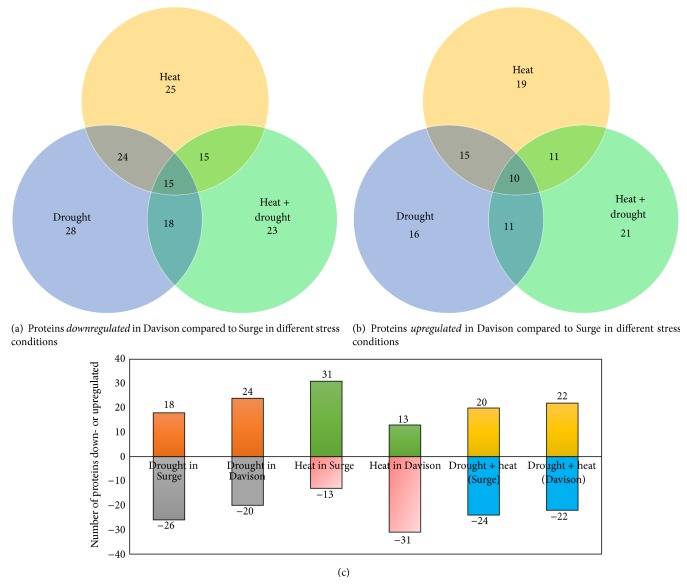
Venn diagram showing the proteins which are unique to respective genotype and stress. The number of downregulated (a) and upregulated (b) proteins and comparison of two genotypes in relation to number of proteins that get affected as a result of various stresses (c).

For the genotypic comparisons between Surge and Davison soybean varieties, we examined three different stress conditions (heat, drought, and drought plus heat) and a control condition. Under drought stress conditions, out of the 44 differentially expressed proteins in Davison, 16 proteins were upregulated and 28 proteins were downregulated compared to Surge. Under heat stress conditions, 19 proteins were upregulated and 25 proteins were downregulated in Davison compared to Surge. Furthermore, when the soybean leaves were exposed to combined drought plus heat stress, 21 proteins were upregulated and 23 proteins were downregulated in Davison compared to Surge. To determine how these soybean leaf proteins vary under specific stress conditions, Venn diagram analysis of the number of differentially expressed leaf proteins was conducted (Figures 6(a) and 6(b)).

**Figure 7:**
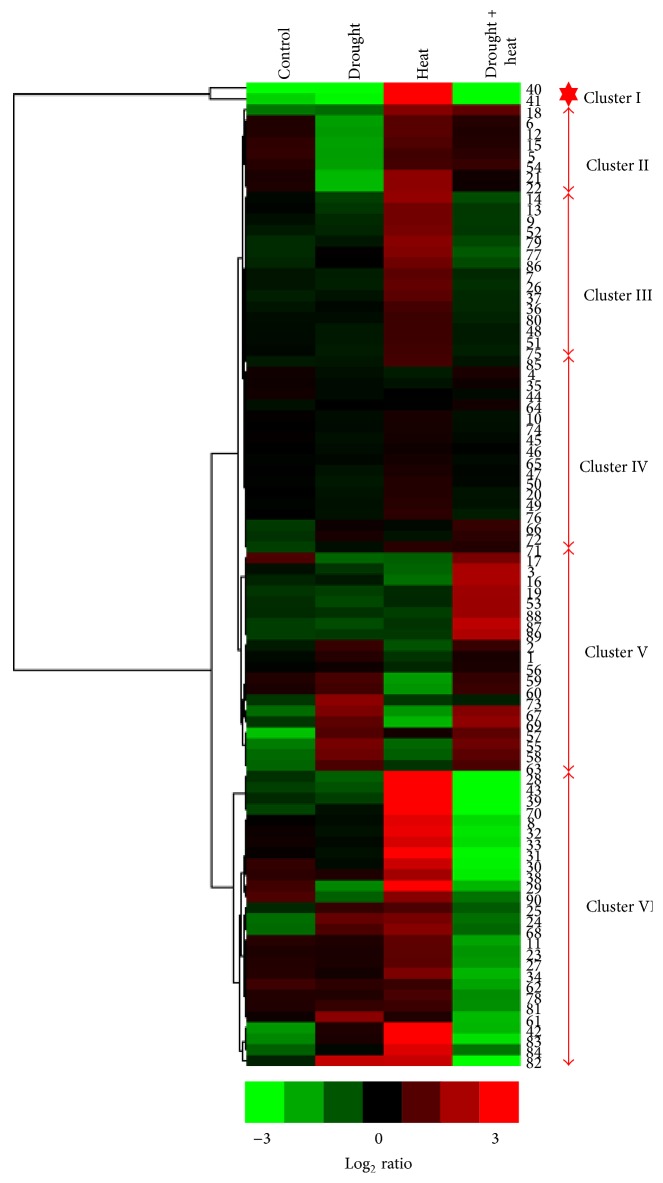
Heat map: Euclidean distance similarity metric and complete linkage method. Heat maps of proteins associated with soybean plants (pooled lines) exposed to different stress conditions. Hierarchical clustering was done based on the log_2_-transformed expression ratios of protein spots using Gene Cluster 3.0 software with the Euclidean distance similarity metric and complete linkage method. Clusters were visualized in JAVA TREEVIEW software.

Hierarchical clustering analysis was performed on the expression data for the 92 differentially expressed protein spots in Surge compared to Davison under control, heat stress, drought stress, and drought plus heat stress conditions to identify trends (Figure 7). Clustering analysis revealed the assignment of the 92 protein spots into 6 prominent clusters (I–VI). Cluster I contained two proteins that were downregulated in leaves when exposed to drought, and drought plus heat conditions, but were upregulated under heat stress conditions. Cluster II was comprised of leaf proteins that were downregulated under drought conditions but were not altered under the other conditions. Cluster III consisted of proteins that were upregulated in response to heat stress to a lesser extent than those in cluster I. Cluster IV included proteins that were not significantly altered in response to stress. The expression of protein spots in cluster V displayed remarkable variability; under heat stress conditions, these proteins were downregulated, but under combined drought and heat stress conditions, these proteins were upregulated. The proteins included in cluster VI displayed prominent downregulation under heat stress conditions but upregulation under combined drought and heat stress conditions.

#### 3.2.2. Comparison of the Effects of Stress on Different Genotypes

To examine the molecular changes that occur under stress conditions, the leaf proteomes of both varieties were analyzed (Figure 6(c)). Under drought stress conditions, it was found that 11 proteins (stromal 70 kDa heat shock-related protein, ATP-dependent zinc metalloprotease, RuBisCO large subunit-binding protein subunit beta, elongation factor Tu, glutamine synthetase, photosystem II stability/assembly factor HCF136, malate dehydrogenase, fructose-bisphosphate aldolase, ferredoxin-NADP reductase, and chlorophyll a-b binding protein 6A) were upregulated in Davison but were downregulated in Surge.

Under heat stress conditions, 13 proteins were downregulated in Surge, whereas 31 proteins were downregulated in Davison compared to the control condition. The results revealed significant downregulation of 20 proteins in Davison. The most downregulated proteins include stromal 70 kDa heat shock-related protein, ATP-dependent zinc metalloprotease FTSH 8, elongation factor Tu, and ribulose bisphosphate carboxylase large chain, but all of these proteins were upregulated in Surge.

When the plants were exposed to drought plus heat stress, it was found that 22 proteins were downregulated and 22 proteins were upregulated in Davison, whereas 24 proteins were downregulated and 20 proteins were upregulated in Surge compared to plants under the control conditions. Under the drought plus heat stress conditions, 9 proteins were downregulated in Surge (the most downregulated proteins include stromal 70 kDa heat shock-related protein, ATP-dependent zinc metalloprotease FTSH 8, ribulose bisphosphate carboxylase small chain, and carbonic anhydrase 1). However, these proteins were upregulated in Davison.

### 3.3. Functional Correlation among the Identified Proteins under Drought and Heat Stress

**Figure 8:**
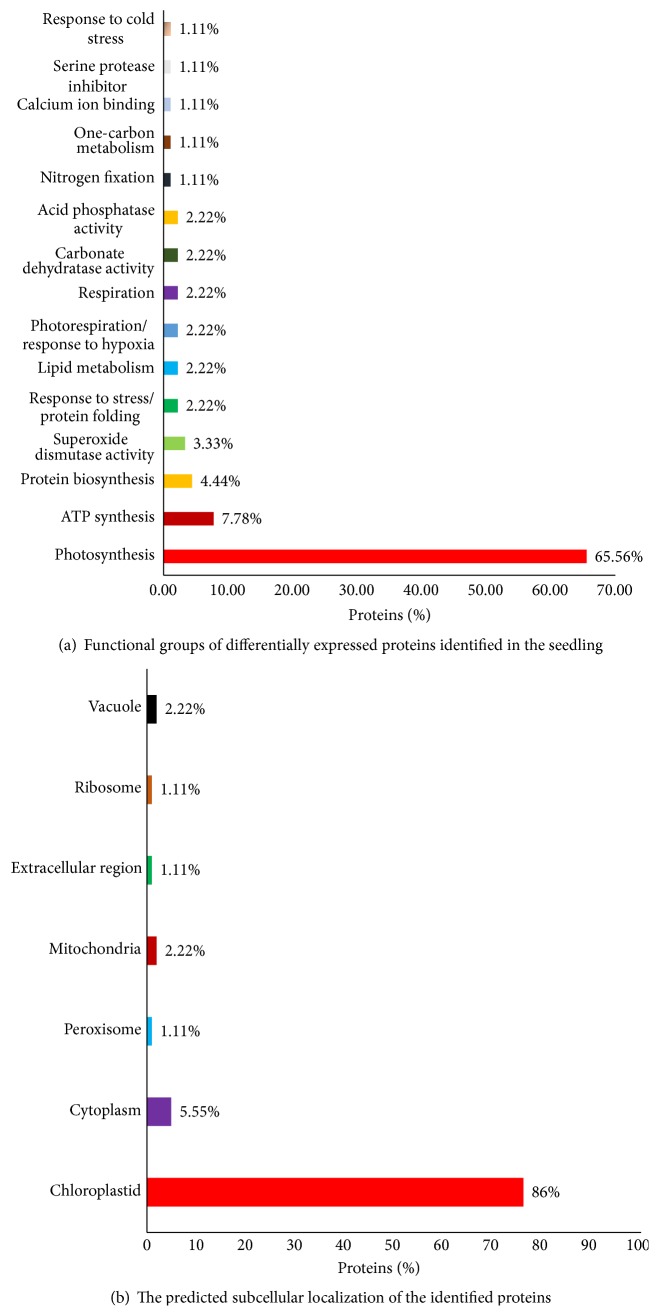
Graphical representation of functional categories of identified proteins. *x*-axis defines various functional groups and *y*-axis indicates % of proteins. B-Mapping for the subcellular localization of the identified proteins. *x*-axis defines various subcellular organelles and *y*-axis indicates % of proteins. Numerical values symbolize the percentage of proteins in each functional class.

To attain a better understanding of the early proteomic responses, a majority of the differentially expressed proteins identified were classified into various functional categories. This analysis (Figure 8(a)) indicated that the 92 significantly altered protein spots identified were found to be involved in different metabolic pathways and processes, including photosynthesis (65.56%), ATP synthesis (7.78%), protein biosynthesis (4.44%), superoxide dismutase activity (3.33%), protein folding (2.22%), lipid metabolism (2.22%), photorespiration/response to hypoxia (2.22%), respiration (2.22%), carbonate dehydratase activity (2.22%), acid phosphatase activity (2.22%), nitrogen fixation (1.11%), one-carbon metabolism (1.11%), calcium ion binding (1.11%), serine protease inhibitor (1.11%), and response to cold stress (1.11%).

An analysis was performed to elucidate the subcellular localization of the differentially expressed proteins (Figure 8(b)). A large portion of the differentially expressed proteins identified were predicted to be chloroplastic (86%), whereas most of the remaining proteins were predicted to be localized to the cytoplasm (5.55%), mitochondria (2.22%), vacuoles (2.22%), peroxisomes (1.11%), or ribosomes (1.11%).

### 3.4. Identified Proteins Affecting Photosynthesis under Drought and Heat Stress Conditions

**Figure 9:**
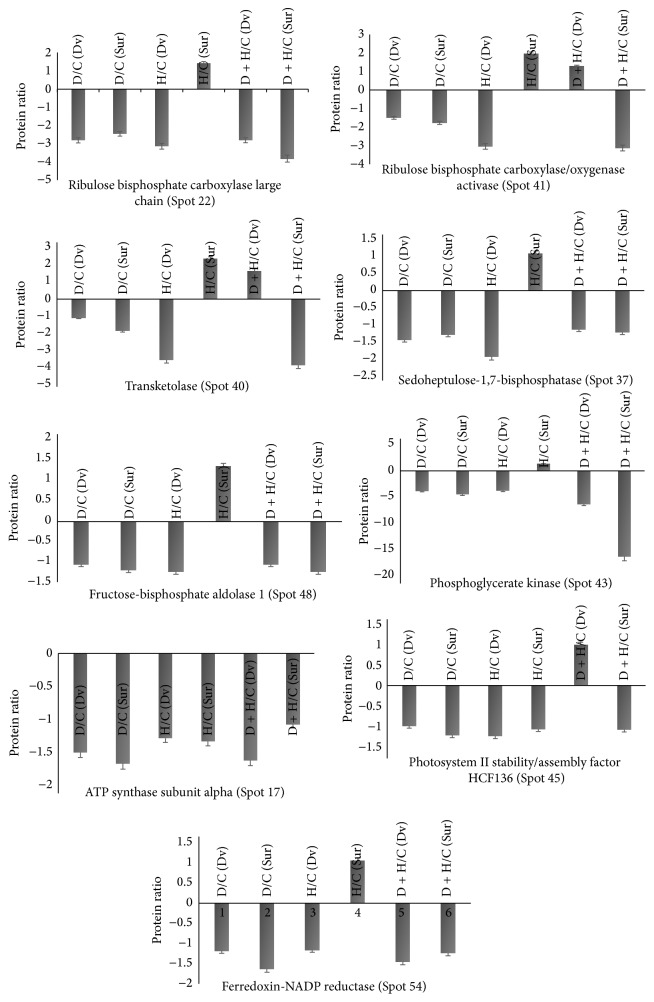
Bar diagrams showing differential regulation of photosynthesis-related proteins. This response is to various stress treatments in two soybean cultivars Surge and Davison. Dv = Davison; Sur = Surge; D = drought, H = heat, and D + H = drought plus heat stress. The protein abundance is presented as protein ratio (*y*-axis) compared to control in response to various abiotic stress treatments (drought, heat, and drought + heat) on *x*-axis.

In this study, 25 proteins that are directly or indirectly involved in photosynthesis were discovered as differentially expressed in soybeans in response to various stress conditions. Protein upregulation and downregulation in Davison compared to Surge are presented in Figure 9. The proteomic analysis revealed that, under drought stress, photosynthesis-related proteins, including ribulose bisphosphate carboxylase large chain (Spot 22), ribulose bisphosphate carboxylase/oxygenase activase (Spot 41), transketolase (Spot 40), sedoheptulose-1,7-bisphosphatase (Spot 37), fructose-bisphosphate aldolase 1 (Spot 48), phosphoglycerate kinase (Spot 43), oxygen-evolving enhancer protein 2-1 (Spot 74), chlorophyll a-b binding protein 3 (Spot 75), ATP-dependent zinc metalloprotease (Spot 9), ATP synthase subunit alpha (Spot 17), ATP synthase subunit beta (Spot 19), photosystem II stability/assembly factor HCF136 (Spot 45), and ferredoxin-NADP reductase (Spot 54), were downregulated in both soybean varieties (Figure 9). This indicates that drought stress favors a reduction in the activities of photosynthetic carbon reduction cycle enzymes, specifically ribulose-1,5-bisphosphate (RuBP) regeneration and inhibition of RuBisCO activity [53–55]. These findings also support the current physiological analysis of the photosynthesis.

The photosynthesis rate in higher plants depends on the activity of RuBisCO and the regeneration of RuBP. It has been reported that drought stress results in a rapid decrease in the abundance of RuBisCO small subunit (rbcS) in tomato [56]. Similarly, our analysis also revealed a decreased (2–4-fold) synthesis of RuBisCO large and small chain enzyme under drought stress which may have direct effect on photosynthesis. The literature suggests that this inhibition of RuBisCO activity was due to the binding of inhibitors such as 2-carboxyarabinitol 1 phosphate (CA1P) to RuBisCO [57]. However, the interactions between RuBisCO and inhibitors are known to be prevented by ATP hydrolysis and RuBisCO activase, which regulates the active site conformation of RuBisCO, removes the inhibitors and the bound inactive RuBP, and allows RuBisCO to undergo rapid carboxylation [58]. Furthermore, we found that, in response to drought stress, RuBisCO activase is also downregulated by 2-fold under stress conditions compared to the control conditions. This result suggests that, under drought stress, RuBisCO activase cannot prevent the interaction between RuBisCO and its inhibitors and cannot remove the bound RuBP to activate RuBisCO. Along with the downregulation of RuBisCO and RuBisCO activase, we found that, under drought stress, other key Calvin cycle enzymes that play a crucial role in carboxylation were downregulated, including transketolase (2-fold compared to the control condition), sedoheptulose-1,7-bisphosphatase (1–2.4-fold), fructose-bisphosphate aldolase 1 (1.5-fold), and phosphoglycerate kinase (4–17-fold). This result suggests that carboxylation is also reduced due to the inhibition of these enzymes, thereby decreasing the level of photosynthesis and ultimately affecting the growth and development of these soybean plants.

We found that, due to heat stress, crucial proteins that regulate electron transport activity were also downregulated including ATP synthase subunit alpha (2.18-fold in Surge compared to Davison), ATP synthase subunit beta (1.74-fold in Davison compared to Surge), photosystem II stability/assembly factor HCF136 (1.15-fold in in Davison compared to Surge), oxygen-evolving enhancer protein 2-1 (1.8-fold in Surge compared to Davison), and ferredoxin-NADP reductase (1.57-fold in Davison compared to Surge). These results suggest that, in both soybean cultivars, heat stress negatively affects electron transport activity, including PSII downregulation, thereby reducing the level of photosynthesis [59].

### 3.5. Upregulation of EF-Tu during Heat Stress in Surge and the Role of EF-Tu in Protecting the Photosynthesis Machinery

2D-DIGE analysis revealed that the chloroplast protein synthesis elongation factor, EF-Tu protein (Spot 31), was upregulated by 4.6-fold in Surge compared to Davison under heat stress. Additionally, we found that, in Surge, under heat stress, fewer (13) proteins were downregulated than under drought stress, whereas 31 proteins were downregulated in Davison under heat stress. Chloroplast EF-Tu is a protein that is involved in the elongation of polypeptides during the translational process, and it belongs to a nuclear-encoded multigene family [60, 61]. The heat stress-induced accumulation of EF-Tu in mature plants is thought to protect the photosynthesis machinery [62]. It has been reported that maize EF-Tu plays a crucial role in heat tolerance by acting as a molecular chaperone, and it has been found to protect heat-labile citrate synthase, RuBisCO activase, and malate dehydrogenase from thermal accumulations [63, 64]. The results indicate that, upon heat stress, EF-Tu assembles in the cytosol in Surge (as seen by an increase in the protein levels of Spots 29–31). Therefore, based on these results, EF-Tu could serve as a biomarker of heat stress in soybeans.

Bioinformatics analysis of protein-protein interactions of this study revealed that the EF-Tu protein undergoes chaperone-mediated interaction with all photosynthesis-related enzymes, including RuBisCO activase, malate dehydrogenase, phosphoglycerate kinase, and sedoheptulose-1,7-bisphosphatase (Figure 12). An interaction between cpn60*β* and RuBisCO activase has been reported in* Arabidopsis* for acclimating photosynthesis to heat stress [65]. Consequently, it was found that, in Surge variety under heat stress, the expression of most proteins related to photosynthesis was conserved, that is, either upregulated or unchanged; in particular, RuBisCO activase (Spot 41) was upregulated 8.71-fold in Surge compared to Davison. We propose that EF-Tu is synthesized upon heat stress and protects the heat-induced degradation of all photosynthesis-related proteins and enzymes, especially RuBisCO activase, maintaining the photosynthesis levels.

### 3.6. Role of Stromal Heat Shock-Related Protein during Severe Heat Stress

Based on 2D-DIGE analysis, we found that the stromal 70 kDa heat shock-related protein (Spot 8) was upregulated in Surge by 4.15-fold compared to Davison (Figure S6), and, compared to the control conditions, it was upregulated by 2.33-fold in Surge under heat stress conditions. This molecular chaperone maintains cellular homeostasis in cells under adverse abiotic stress conditions. It has been found that stromal 70 kDa heat shock-related protein of* Arabidopsis thaliana* is responsible for protein folding and can assist in protein refolding under heat stress conditions [66]. The results also indicate that, under heat stress, proteins related to the photosynthesis machinery are protected. Taken together, the analysis indicates that stromal 70 kDa heat shock-related protein plays a role in the maintenance or biogenesis of chloroplast proteins, thereby maintaining the level of photosynthesis [20, 67].

A further bioinformatics analysis of protein-protein interactions revealed that this HSP protein undergoes two-way interactions, one with a molecular chaperone (GrpE) and one directly with phosphoglycerate kinase. As discussed above, under heat stress, most proteins related to photosynthesis are more abundant in Surge than in Davison. Thus, we propose that stromal 70 kDa heat shock-related protein might activate a multiprotein interaction cascade that maintains the biogenesis of chloroplast proteins under heat stress and protects against the heat-induced degradation of all photosynthesis-related proteins and enzymes, preserving the photosynthesis levels.

### 3.7. Quantification of ROS and the Role of Carbonic Anhydrase in Plant Protection

**Figure 10:**
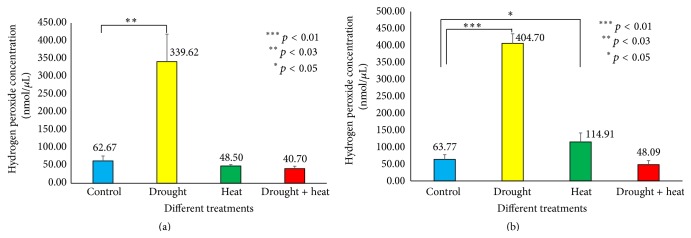
Bar diagram showing hydrogen peroxide levels under various stress treatments among Surge (a) and Davison (b). *x*-axis defines various abiotic stress treatments (drought, heat, and drought + heat) including control and the *y*-axis defines measurement of hydrogen peroxide concentration in nmol/*µ*L. Each value represents the mean ± SE of five replicates and the asterisks designate the significance of changes from their subsequent control (^*∗∗∗*^ *p* < 0.01, ^*∗∗*^ *p* < 0.03, and ^*∗*^ *p* < 0.05).

Studies have shown that increasing levels of water stress in* Vigna* plants may increase ROS levels by means of hydrogen peroxide [28]. Results of present study (Figure 10) indicate a 5.4-fold elevation in the H_2_O_2_ level under drought stress compared to the control conditions in Surge (62.67 nmol *µ*L^−1^ to 339.62 nmol *µ*L^−1^) and a nearly 6.3-fold elevation in Davison (63.77 nmol *µ*L^−1^ to 404.70 nmol *µ*L^−1^) on the sixth day of stress. Under heat stress, we found no significant difference in the H_2_O_2_ level in Surge, but in Davison, the H_2_O_2_ level was increased by 1.8-fold.

The proteomic analysis shows that carbonic anhydrase 1 (Spot 72) is upregulated by 1.8-fold due to drought stress and by 1.6-fold due to combined drought plus heat stress in Davison (Figure S7). Carbonic anhydrase is a zinc-containing metalloenzyme, and the specific association between carbonic anhydrase and RuBisCO enables CO_2_ to interact with RuBisCO and maintains the functional machinery of RuBisCO [68]. Under drought stress conditions, when plants detect a limitation of water availability, stomatal closure is triggered, which limits the entrance of CO_2_, resulting in a net reduction of photosynthesis. This confers increased ROS accumulation with oxidative stress [69]. It has been reported that higher expression of carbonic anhydrase in the cell increases its resistance to cytotoxic concentrations of H_2_O_2_ [70]. Thus, it is possible that, under drought stress conditions, the upregulated expression of carbonic anhydrase in Davison principally makes the plant cells resistant to toxic H_2_O_2_ levels and protects the plant from oxidative stress. Although we detected a higher level of hydrogen peroxide in drought-stressed Davison plants, no significant phenotypic changes were detected on the sixth day of stress.

### 3.8. Quantification of ABA under Different Abiotic Stress Conditions

**Figure 11:**
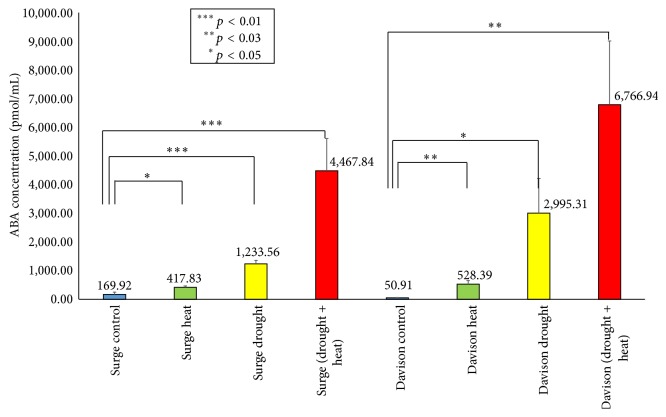
Bar diagram showing quantification of ABA in different abiotic stress conditions in Surge and Davison. *x*-axis defines various abiotic stress treatments (drought, heat, and drought + heat) including control and the *y*-axis defines measurement of ABA concentration (pmol/mL). Each value represents the mean ± SE of five replicates and the asterisks designate the significance of changes from their subsequent control (^*∗∗∗*^ *p* < 0.01, ^*∗∗*^ *p* < 0.03, and ^*∗*^ *p* < 0.05).

ABA is a phytohormone critical for plant growth and plays a key role in integrating various stress signals [71]. Several studies suggested that, in response to the water and high temperature stress, stomatal movement is controlled by ABA signaling [72]. In this study, we quantified the ABA concentration in the leaf under all the stress conditions (Figure 11). We found that, compared to the control plants, the highest level of accumulation of ABA occurred during the drought plus heat stress conditions. In Surge, it was 26-fold higher, and in Davison, it was 132-fold higher compared to the control conditions. In the drought-stressed leaves, we found ABA levels to be 7-fold higher in Surge and 58-fold higher in Davison compared to the control plants. Under heat stress, ABA is upregulated by 2-fold in Surge and 10-fold in Davison compared to the normal plants. Through investigating the change of abscisic acid (ABA) levels, we found that drought plus heat stress has the highest level of ABA accumulation, which also correlates with the stomatal conductance measurements.

### 3.9. The Identified Protein “Glutamine Synthetase” and Its Association with Nitrogen Metabolism

Based on the analysis of the soybean leaf proteome, we found that cytosolic glutamine synthetase leaf isozyme (Spot 33) was downregulated by 4-fold in Davison under drought stress conditions compared to Surge. This enzyme catalyzes a major reaction of nitrogen metabolism, that is, the assimilation of ammonium to glutamine using the substrate glutamic acid [73]. Reduction of glutamine synthetase expression under drought conditions has been reported as a protective mechanism for plants because the intermediate nitric oxide is an active radical. Thus, we predict that the same protective mechanism also occurs in soybean leaves [21, 74]. Additionally, it has been reported that the overexpression of cytosolic glutamine synthetase in poplar enhances the photorespiration under drought stress and that it also contributes to the protection of photosynthesis [75, 76].

### 3.10. Protein-Protein Interaction Analysis

**Figure 12:**
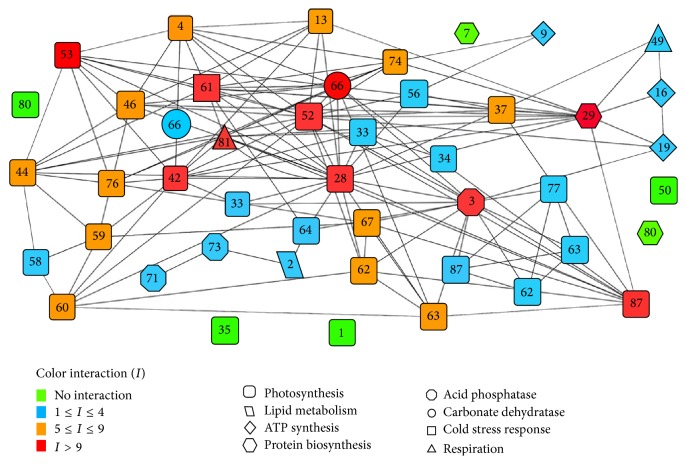
Protein-protein interaction network predictions made by bioinformatics analysis. The prediction is between differentially expressed proteins due to different stress conditions and identified by proteomic study. The network comprises 46 nodes and 139 edges. Symbol color corresponds to degree of interactions (color code on the left side). Symbol shapes indicate specific protein function. Node legends indicate the spot number corresponding to the particular protein spot from 2D-DIGE gel. Follow Table 1 for protein names for corresponding spot number [note: duplicate insertion of few spot numbers (Spot numbers 33, 62, 63, 66, and 87) is incorporated to avoid overcrowding of the network. Spot number 80 appears two times with two different shapes indicating that the same protein is counted for two different functions].

For any systems-level understanding of cellular functions, it is crucial to appropriately elucidate all functional interactions between the proteins in the cell at a given time. We searched for a variety of functional protein-protein interactions between the differentially expressed proteins in soybeans in response to abiotic stress to obtain an improved understanding of these protein-protein interactions, including stable complexes, metabolic pathways, and a wide range of regulatory interactions [44] (Figure 12 and Table 1). From the network, we discovered 10 major proteins (most significantly, ribulose bisphosphate carboxylase, ribulose bisphosphate carboxylase/oxygenase activase, ATP synthase subunit alpha/beta, sedoheptulose-1,7-bisphosphatase, photosystem II stability/assembly factor HCF136, ferredoxin-NADP reductase, elongation factor Tu, and carbonic anhydrase 1) having more than 9 interactions that are considered to be at the central body of the network. Drought or heat stress mediated downregulation or upregulation of those central body proteins of the network may also affect their predicted partners, which ultimately could affect the molecular signaling by collapsing the whole cellular network.

## 4. Discussion 

Drought and high temperature stress conditions cause extensive losses to crops', including soybean, production worldwide [77, 78]. Individually, the effect of drought or heat stress conditions on soybeans has been the subject of intense research [79, 80], but to the best of our knowledge, no such detailed proteomics-based study attempt has been made to date. The cooccurrence of drought plus heat stress is of special interest because they occur together in the field. Recent studies have revealed that the responses of plants to two different abiotic stresses that occur simultaneously are exclusive and cannot be directly inferred from the response of plants to each of the different stresses applied independently [81]. Thus, to emphasize the molecular, physiological, and proteomic aspects of stress combination and to unravel the underlying mechanisms of soybean responses to drought, heat stress, and cooccurring stresses, we performed a proteomic study combined with physiological and computational analysis to facilitate the discovery of genes that can enhance the tolerance capabilities of soybean crops to the stress conditions [82].

Reduced stomatal conductance is one of the most sensitive responses to drought stress, observed in kidney beans and various C3 plants [48, 83]. At the level of soybean physiology, we concluded that drought stress, heat stress, and drought plus heat stress reduce stomatal conductance, which is caused by decreasing the leaf water potential via transpiration rate alterations in both varieties. Stomatal conductance is also reliant on leaf temperature via transpiration rate, and we observed that the increase of leaf temperature is correlated to the stomatal conductance and transpiration rates. Photosynthesis is among the primary processes affected by the drought and heat stresses [16, 84, 85]. As soil moisture decreases during drought stress and cooccurring drought and heat stress, the water content of mesophyll tissue also reduces, ultimately affecting the photosynthetic physiology, principally carbon assimilation and energy use of the plant. Surprisingly, there was no such significant effect observed during heat stress alone, which actually establishes the fact that consequences of water deficiency have an enormous impact on altering the photosynthetic machinery [86].

**Figure 13:**
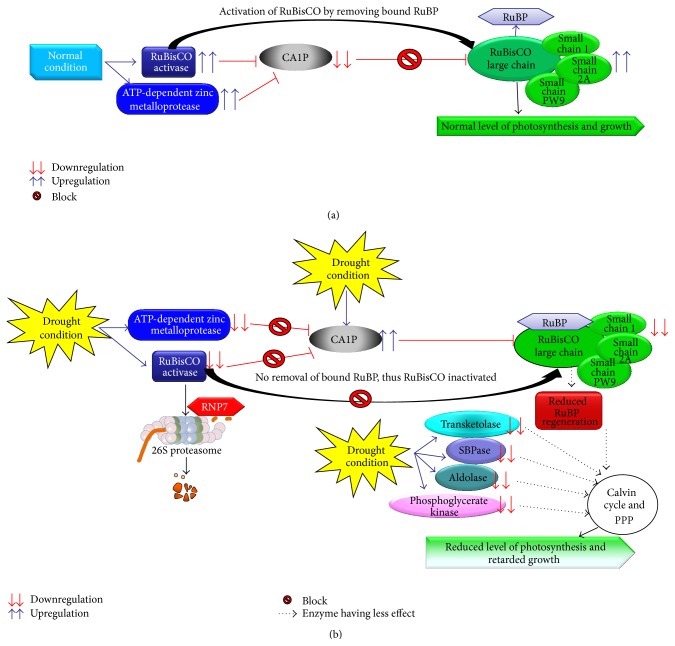
Predicted analysis of the mechanism by which the photosynthesis rate is reduced. (a) In normal condition, RuBisCO activase removes the RuBP from RuBisCO and photosynthesis is not affected. (b) During drought stress, RuBisCO activase and ATP-dependent zinc metalloprotease are downregulated and CA1P (2-carboxyarabinitol 1 phosphate, a potent inhibitor of RuBisCO) is upregulated; thus no removal of RuBP from RuBisCO occurs, and with that the Calvin cycle and pentose phosphate pathway (PPP) enzymes are downregulated which ultimately results in reduced level photosynthesis and retarded growth.

**Figure 14:**
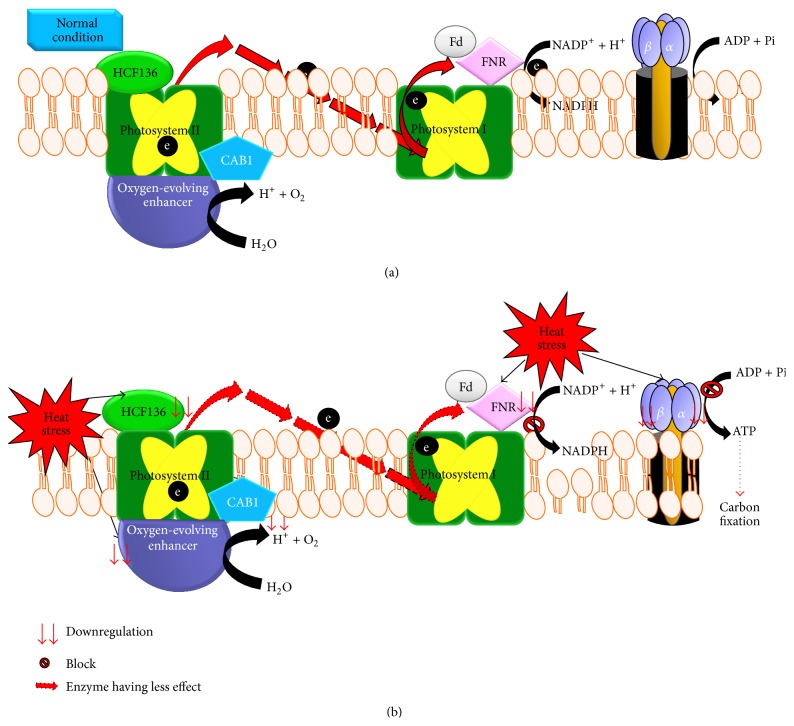
Predicted heat stress and electron transport block mechanism. Predicted analysis of irreversible inhibition of photosynthesis during heat stress and the protein-protein interplay that negatively affects the electron flow. (a) In normal condition, there is normal electron flow and no reduction in carbon fixation. (b) Due to heat stress effect, all key proteins that mediate electron flow are downregulated and block the electron flow, and as a result downregulation of ATP synthase reduces the production of ATP and as a result carbon fixation process slows down.

Gel-based proteomic separation is widely used in various plants' studies to decipher abiotic stress-responsive mechanisms for its high precision exclusively for comparative proteomics [87]. Likewise, we used a gel-based proteomic approach to elucidate the mechanisms underlying the early responses of soybeans to various abiotic stresses. To propose a functional relationship among proteins in response to abiotic stress, we performed functional categorization and subcellular localization of various differentially expressed proteins [88, 89]. Our proteomic analysis revealed that a total of 44 proteins were significantly changed in soybean leaves after 6 days of different stress exposures (drought, heat, and drought plus heat), and their functional categorization showed that most of the differentially changed proteins were related to photosynthesis, ATP synthesis, and protein biosynthesis. The subcellular localization study reveals that most of the proteins are actively synthesized in chloroplast and cytoplasm. We found that 25 proteins related to photosynthesis were downregulated during stress conditions in both the soybean varieties. Most importantly, downregulation of RuBisCO and RuBisCO activase enzymes during drought stress reduces the carboxylation process because the limitation of the RuBisCO activase prevents the reactivation of RuBisCO molecules [53, 90]. Previous reports on* Phaseolus vulgaris* and drought stress showed that the Calvin cycle enzymes' activities are affected significantly [91]. Similarly, our study also indicates that, under drought stress, key enzymes of Calvin cycle—such as transketolase, sedoheptulose-1,7-bisphosphatase, fructose-bisphosphate aldolase 1, and phosphoglycerate kinase—that play major roles in carboxylation are also downregulated, resulting in reduced levels of photosynthesis, thus affecting the development and growth of the soybean plant. Figures 13 and 14 show models for how the photosynthesis level could be reduced after drought/heat stress via alternated protein expressions and protein-protein interactions. It was found that salt stress-induced effects in cowpea plants negatively affect nitrogen assimilation and overexpressed glutamine synthetase gene in rice modifies nitrogen metabolism during abiotic stress [92, 93]. Surprisingly, our soybean leaf proteome analysis revealed that cytosolic glutamine synthetase leaf isozyme, which is responsible for assimilation of ammonium to glutamine using substrate glutamic acid, was highly downregulated in Davison as compared to Surge during drought stress conditions, resulting in negatively affected nitrogen assimilation.

Studies have shown that heat stress reduces yields by limiting electron transport activity in cotton [94] and* Arabidopsis* [95]. The physiological and proteomic data of our study reveal that the PSII could be damaged during heat stress, and this damage reduces net photosynthesis levels in soybeans. Crucial proteins—for example, ATP synthase, HCF136, oxygen-evolving enhancer protein 2-1, and ferredoxin-NADP reductase that regulate the electron transport activity—were found to be downregulated during heat stress.  Figure 14 shows the predicted analysis of irreversible inhibition of photosynthesis during heat stress and the protein-protein interplay that negatively affects the electron flow. During heat stress, we observed that the EF-Tu protein is highly upregulated in Surge, which is consistent with the earlier findings in heat tolerant maize [96]. So we predict that high levels of heat stress-induced expression of EF-Tu in Surge activate the heat stress tolerance mechanisms, which might actually be a safeguard for the heat-labile proteins such as citrate synthase, RuBisCO activase, and malate dehydrogenase from thermal degradation; and finally, this action at the cellular level enables the protection of photosynthetic machinery. In support of the heat tolerance mechanism in Surge, we found a higher accumulation of a stromal 70 kDa HSP, which we predict might regulate functionally vital processes crucial for the plant's survival. This finding is relevant to the earlier reports reporting that HSP family of chaperons can promote the protein refolding to maintain protein homeostasis under heat stress conditions [97]. The physiological study also indicates that, in Surge, there is no significant change of photosynthesis levels due to heat stress.

One of the inevitable outcomes from the biochemical assays is the increase of ROS level (hydrogen peroxide) during drought stress in both Surge and Davison. We also found higher amounts of carbonic anhydrase accumulation in the cell which probably aid the cell in becoming more resistant to cytotoxic concentrations of H_2_O_2_ in drought-stressed Davison plants; thus, significant changes at the phenotypic level were not noticed at the sixth day of the stress [70]. While investigating the changes in ABA levels during stress, we found that the cooccurrence of drought and heat stresses causes the highest accumulation of ABA in soybeans compared to control plants, which is consistent with earlier findings in brassica and rice [98, 99]. This observation also correlates with the stomatal conductance measurements, statistical analysis by scatter plot of ABA concentration, and stomatal conductance measurement that shows a linear relationship with *R* ^2^ > 0.95, which indicates that an increase in ABA levels and a decrease in stomatal conductance have a kind of cause and effect relationship. While comparing the ABA measurement analysis and stomatal conductance measurement, we found that, in cooccurring drought and heat stress, stomatal conductance value is lowest while ABA levels are at the highest compared to drought-stressed and heat-stressed plants. After correlating the two observations, we concluded that the more severe the stress is, the more the ABA synthesis ultimately results in the stomatal closure, that is, lesser stomatal conductance. In response to the drought, heat, and cooccurring drought plus heat stresses, calcium levels elevate as probable calcium binding protein CML33 accumulates at higher levels [100]. These consequences activate the phosphoprotein cascade, which ultimately activates the transcription of ABA biosynthesis precursors and finally synthesizes ABA that aids in stomatal closure to reduce the transpiration levels, CO_2_ assimilation, and photosynthesis for the sake of survival of the plant under adverse environmental conditions [71].

Finally, for the better understanding of the crosstalk between complex sets of abiotic stresses responsive proteins, we applied system biology approaches to determine the holistic outlook of the molecular responses [17, 101]. Our computational analysis of protein-protein interaction networks identifies 10 major proteinsthat havemore than 9 interactions considered to be at the central body of the network system. This protein network forecasts that drought or heat stress mediated downregulation or upregulation of those central body proteins of the network may also affect their predicted partners, which ultimately could affect the molecular signaling by collapsing the whole cellular network. This systems biology study will assist in the breeding of more abiotic stress tolerant soybean varieties having high nutritional value.

Proteomics study on rice by Kim et al. has shown that proteomic data can be used for crop improvement which eventually leads to food security [42]. A clearer understanding of differential expression of various stress-related proteins and inclusion of this knowledge to breed better varieties may trigger a speedy improvement of crop plants [26, 42]. The extensive application of such quantitative proteomic approaches together with mapping of posttranslational modifications will provide us with comprehensive insights of the regulation of various stress-responsive proteins in complex biological systems [102]. The agronomical perspective of the current study will support the soybean breeders to develop heat and drought tolerant soybean varieties.

Taken together, our study results identify the differential expression of a number of drought-, heat-, and cooccurring drought and heat-responsive proteins. The observed changes in leaf protein expressions suggest that the regulation of various molecular processes and signaling alters due to drought and heat stresses.

## 5. Conclusions

This study shows how drought, heat, and cooccurring drought and heat stresses alter the soybean leaf proteome. Differential expression of 44 abiotic stress-responsive proteins suggests that various signaling cascades and molecular processes are affected due to drought and heat stresses. Furthermore, the result suggests that many differentially expressed photosynthesis-related proteins perturb RuBisCO regulation, electron transport, and Calvin cycle during abiotic stress. These findings, as well as upregulation of EF-Tu and higher level expressions of HSP, will help in better understanding of the heat tolerance mechanisms in the soybean varieties. The biochemical and proteomic assays also explain how higher amount of carbonic anhydrase accumulation in the cell alleviates cytotoxic concentrations of H_2_O_2_ during drought stress. Moreover, we found that cooccurring drought and heat stresses have the highest level of ABA accumulations in Davison, which also correlates with our stomatal conductance measurements. Taken together, the results presented provide a proteomic level explanation for the abiotic stress effects on physiological processes of soybean plants during abiotic stress conditions.

## Supplementary Material

In the supplementary section we provide work flow diagram (Figure S1) followed by phenotypic changes observed on 6th day of stress among various group of soybean plants that were exposed to four different treatments (Figure S2). Soil moisture content measurement is very necessary to monitor the perfect drought condition which is described in Figure S3. In addition to the merged 2D-DIGE gel image in the main article, we also provided the overlaying gel images in Figure S4-S5 for better understanding of the reader. We presented the DeCyder software analysis of stromal heat shock protein and carbonic anhydrase-1 in Figure S6 and Figure S7, respectively. In Table S1 we provide the details of samples and spot map information. Moreover, Table S2 indicates all the p values and average ratios of all the spots identified in this study. For better prediction of the readers we included Table S3 that encompasses protein ID report and Table S4 that included protein peptide summary. We encourage readers to see Table S5 while predicting the protein-protein network.

## Figures and Tables

**Table 1:** Data used to predict protein-protein network. Protein spot numbers are corresponding to the 2D-DIGE gel and respective mass spectrometry based protein identifications are given as UniProt ID.

Spot number	Protein name	UniProt accession number
1	Acyl-[acyl-carrier-protein] desaturase 6	Q0J7E4
2	Lipoxygenase	Q43440
3	Ribulose bisphosphate carboxylase large chain	Q01873
4	Transketolase, chloroplastic	O20250
7	Stromal 70 kDa heat shock-related protein	Q02028
9	ATP-dependent zinc metalloprotease FTSH 8	Q8W585
13	RuBisCO large subunit-binding protein subunit beta	P08927
16	ATP synthase subunit alpha	Q2PMS8
19	ATP synthase subunit beta	Q2PMV0
28	Ribulose bisphosphate carboxylase/oxygenase activase	Q40281
29	Elongation factor Tu, chloroplastic	Q43467
33	Glutamine synthetase leaf isozyme	P15102
34	S-adenosylmethionine synthase 4	A7PRJ6
35	Probable calcium binding protein CML33	Q9SRP4
37	Sedoheptulose-1,7-bisphosphatase	O20252
42	Phosphoglycerate kinase	P12782
44	Photosystem II stability/assembly factor HCF136	O82660
46	Fructose-bisphosphate aldolase 1	Q01516
49	Malate dehydrogenase 1	Q9ZP06
50	Trypsin inhibitor 1	Q43667
52	Chloroplast stem-loop binding protein of 41 kDa b	Q9SA52
53	Ferredoxin-NADP reductase, leaf isozyme 1	Q9FKW6-1
56	Oxygen-evolving enhancer protein 1	P14226
58	Chlorophyll a-b binding protein of LHCII type I	P08221
59	Chlorophyll a-b binding protein	Q10HD0
60	Chlorophyll a-b binding protein 13	P27489
61	2-Cys peroxiredoxin BAS1-like	Q9C5R8
62	Ribulose bisphosphate carboxylase small chain PW9	P26667
63	Ribulose bisphosphate carboxylase small chain 2A	P26575
64	Superoxide dismutase [Fe]	P28759
66	Carbonic anhydrase 1	P46512
67	RuBisCO-associated protein	P39657
71	Stem 31 kDa glycoprotein	P10743
73	Stem 28 kDa glycoprotein	P15490
74	Oxygen-evolving enhancer protein 2-1	Q7DM39
76	Chlorophyll a-b binding protein 6A	P12360
77	Ribulose bisphosphate carboxylase large chain (fragment)	P28416
80	Ribosomal protein L7/L12	Q4BZ06
81	Glycine cleavage system H protein 1	P25855
87	Ribulose bisphosphate carboxylase small chain 1	P00865

## Abbreviations

% VWC: Percentage volume water content
2D-DIGE: Two-dimensional differential in-gel electrophoresis
ABA: Abscisic acid
CA1P: 2-Carboxyarabinitol 1 phosphate
CAB1: Chlorophyll a-b binding protein 1
CML33: Probable calcium binding protein
Cy2/Cy3/Cy5: Cyanine dye 2, 3, or 5
DMF: N, N-Dimethylformamide
EF-Tu: Elongation factor Tu
Fd: Ferredoxin
FNR: Ferredoxin-NADP^+^ oxidoreductase
HRP: Horseradish peroxidase
HSP: Heat shock-related proteins
PPP: Pentose phosphate pathway
RH: Relative humidity
RNP7: Protein RNP-7
ROS: Reactive oxygen species
RuBisCO: Ribulose bisphosphate carboxylase oxygenase
STRING: Search tool for the retrieval of interacting genes.
