# Supplementary material for: Leaf Proteome Analysis Reveals Prospective Drought and Heat Stress Response Mechanisms in Soybean
Source: Biomed Res Int. 2016 Mar 13;2016:6021047. doi: 10.1155/2016/6021047 (PMC4808539; doi:10.1155/2016/6021047)
Supplement: Supplementary file 1 — In the supplementary section we provide work flow diagram (Figure S1) followed by phenotypic changes observed on 6th day of stress among various group of soybean plants that were exposed to four different treatments (Figure S2). Soil moisture content measurement is very necessary to monitor the perfect drought condition which is described in Figure S3. In addition to the merged 2D-DIGE gel image in the main article, we also provided the overlaying gel images in Figure S4-S5 for better understanding of the reader. We presented the DeCyder software analysis of stromal heat shock protein and carbonic anhydrase-1 in Figure S6 and Figure S7, respectively. In Table S1 we provide the details of samples and spot map information. Moreover, Table S2 indicates all the p values and average ratios of all the spots identified in this study. For better prediction of the readers we included Table S3 that encompasses protein ID report and Table S4 that included protein peptide summary. We encourage readers to see Table S5 while predicting the protein-protein network. [file 6021047.f1.pdf]

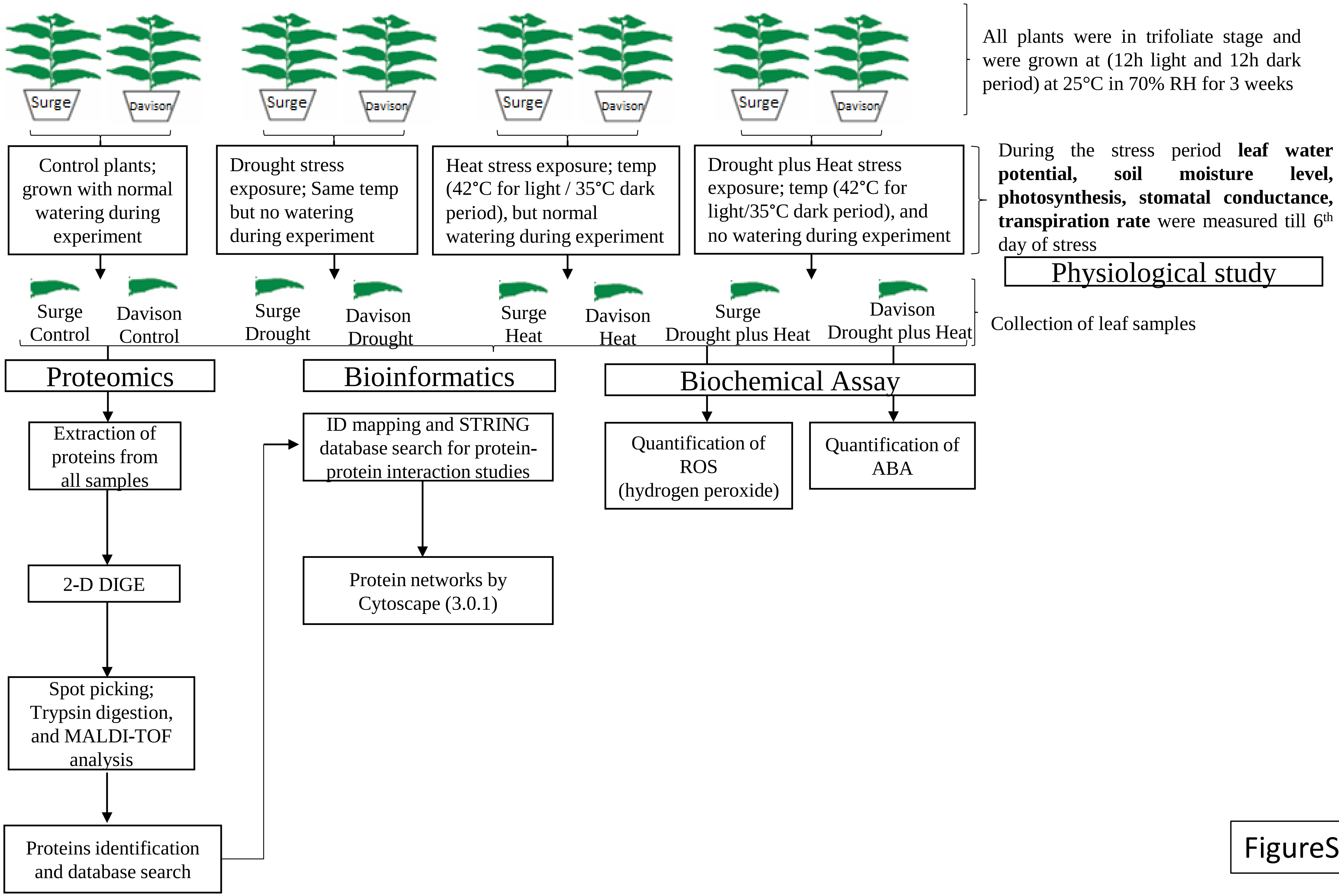
Surge
Davison
All plants were in trifoliate stage and were grown at (12h light and 12h dark period) at 25°C in 70% RH for 3 weeks
Control plants; grown with normal watering during experiment
Drought stress exposure; Same temp but no watering during experiment
Heat stress exposure; temp (42°C for light / 35°C dark period), but normal watering during experiment
Drought plus Heat stress exposure; temp (42°C for light/35°C dark period), and no watering during experiment
During the stress period leaf water potential, soil moisture level, photosynthesis, stomatal conductance, transpiration rate were measured till 6th day of stress
Physiological study
Surge Control
Davison Control
Surge Drought
Davison Drought
Surge Heat
Davison Heat
Surge Drought plus Heat
Davison Drought plus Heat
Collection of leaf samples
Proteomics
Bioinformatics
Biochemical Assay
Extraction of proteins from all samples
2-D DIGE
Spot picking; Trypsin digestion, and MALDI-TOF analysis
Proteins identification and database search
ID mapping and STRING database search for protein-protein interaction studies
Protein networks by Cytoscape (3.0.1)
Quantification of ROS (hydrogen peroxide)
Quantification of ABA

FigureS1

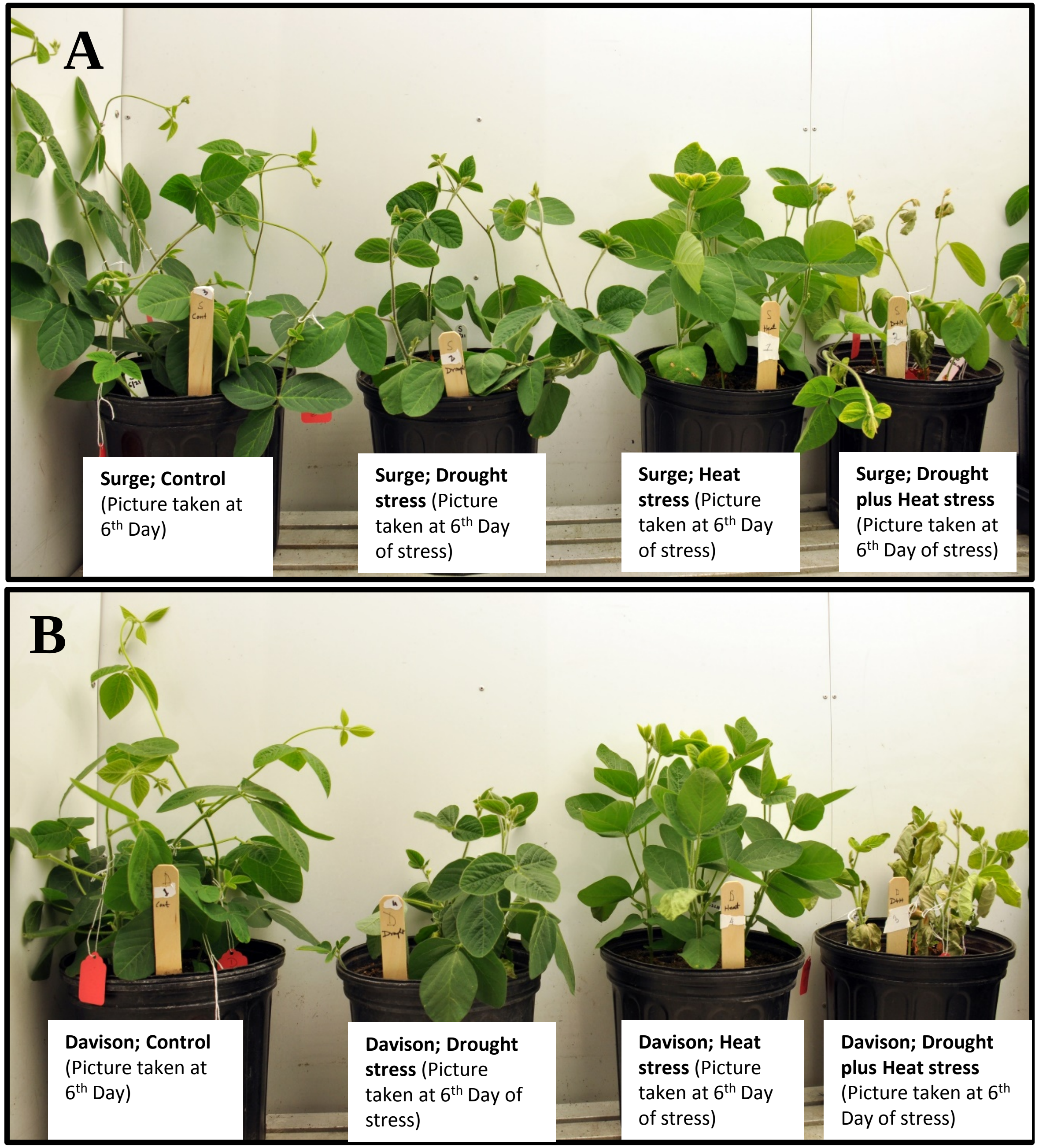
A
Surge; Control (Picture taken at 6th Day)
Surge; Drought stress (Picture taken at 6th Day of stress)
Surge; Heat stress (Picture taken at 6th Day of stress)
Surge; Drought plus Heat stress (Picture taken at 6th Day of stress)
B
Davison; Control (Picture taken at 6th Day)
Davison; Drought stress (Picture taken at 6th Day of stress)
Davison; Heat stress (Picture taken at 6th Day of stress)
Davison; Drought plus Heat stress (Picture taken at 6th Day of stress)

Figure S2

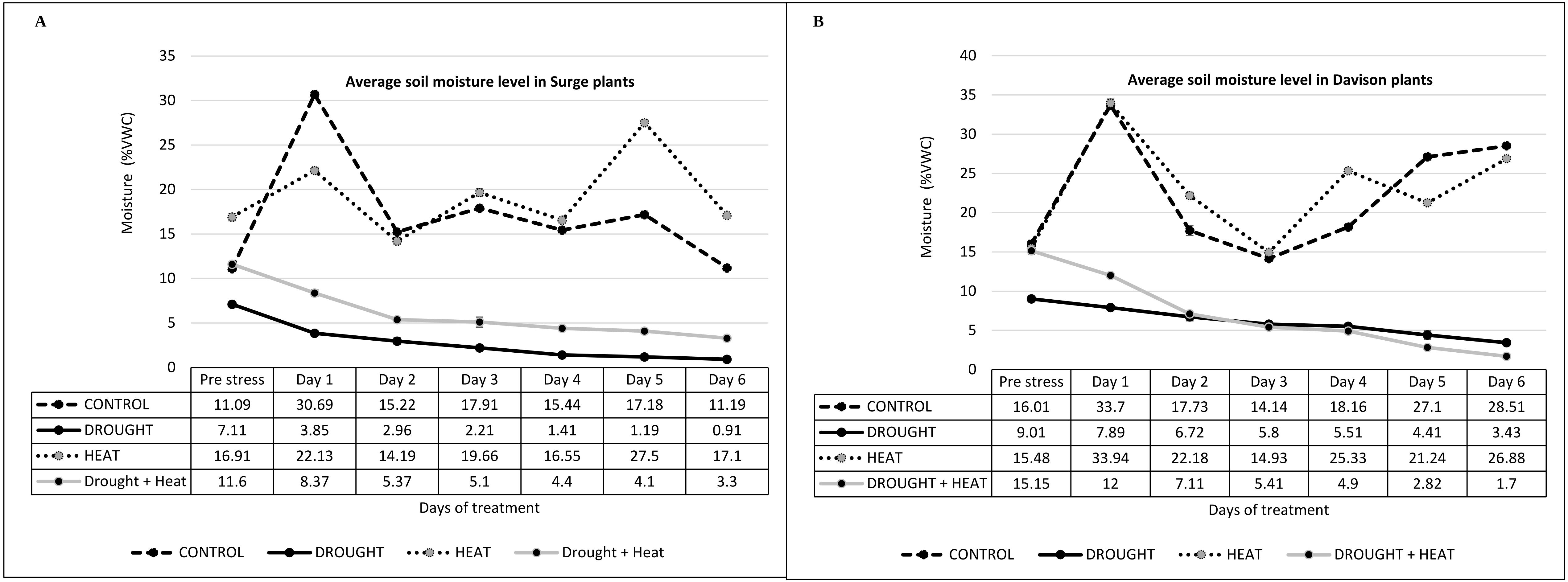

A

	Pre stress	Day 1	Day 2	Day 3	Day 4	Day 5	Day 6
CONTROL	11.09	30.69	15.22	17.91	15.44	17.18	11.19
DROUGHT	7.11	3.85	2.96	2.21	1.41	1.19	0.91
HEAT	16.91	22.13	14.19	19.66	16.55	27.5	17.1
Drought + Heat	11.6	8.37	5.37	5.1	4.4	4.1	3.3

B

	Pre stress	Day 1	Day 2	Day 3	Day 4	Day 5	Day 6
CONTROL	16.01	33.7	17.73	14.14	18.16	27.1	28.51
DROUGHT	9.01	7.89	6.72	5.8	5.51	4.41	3.43
HEAT	15.48	33.94	22.18	14.93	25.33	21.24	26.88
DROUGHT + HEAT	15.15	12	7.11	5.41	4.9	2.82	1.7

Figure S3

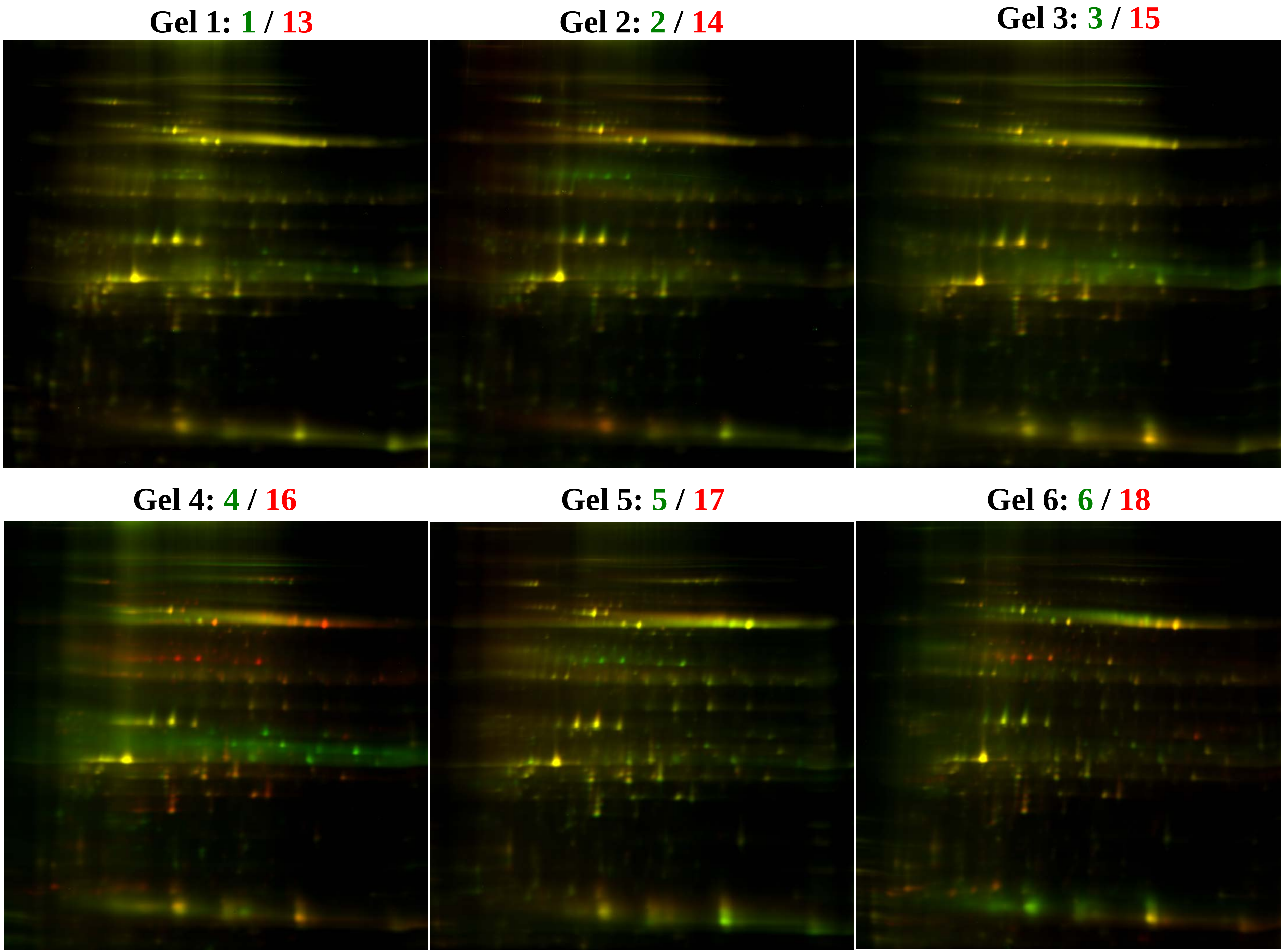

Figure S4

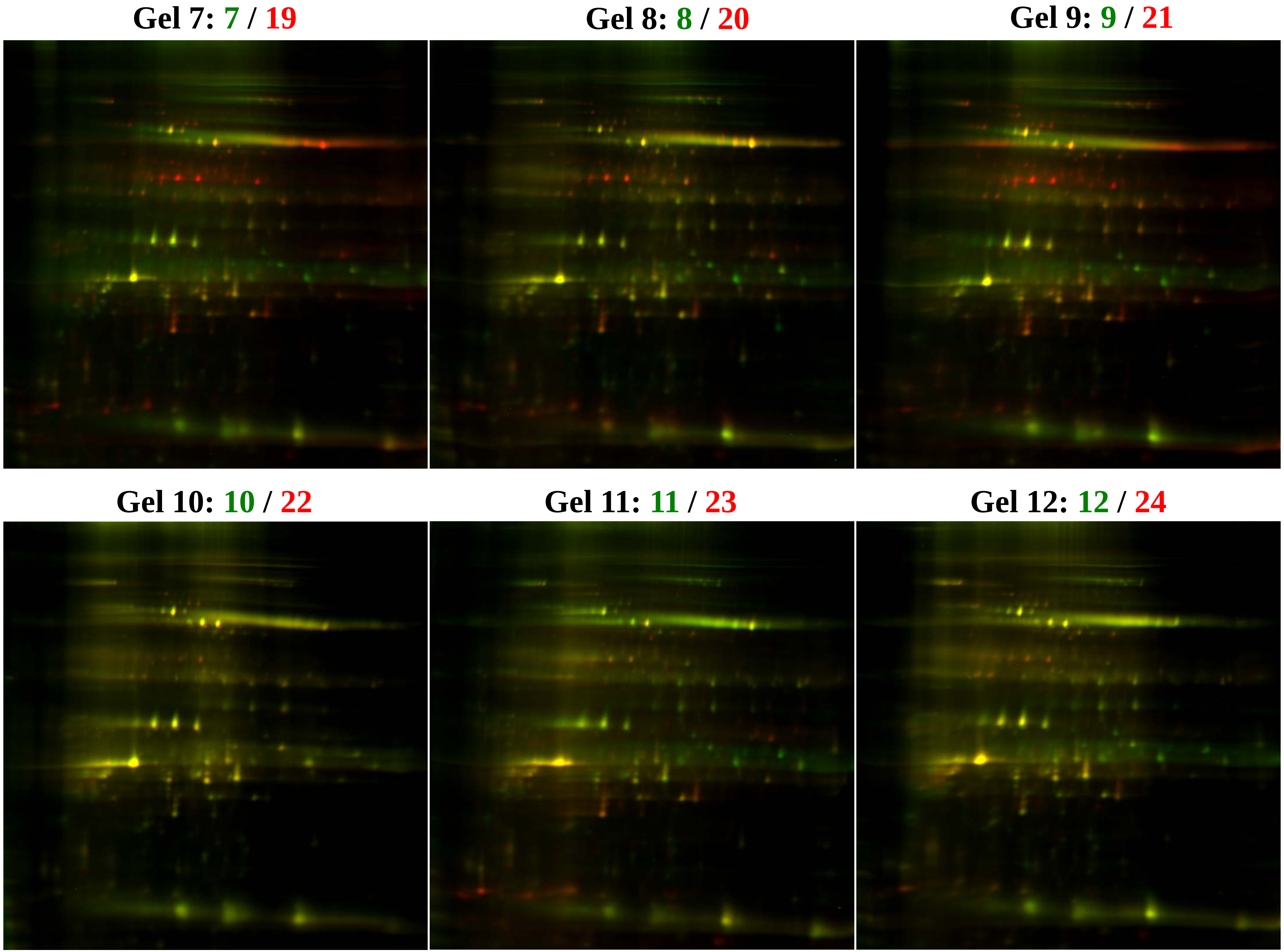

Figure S5

A

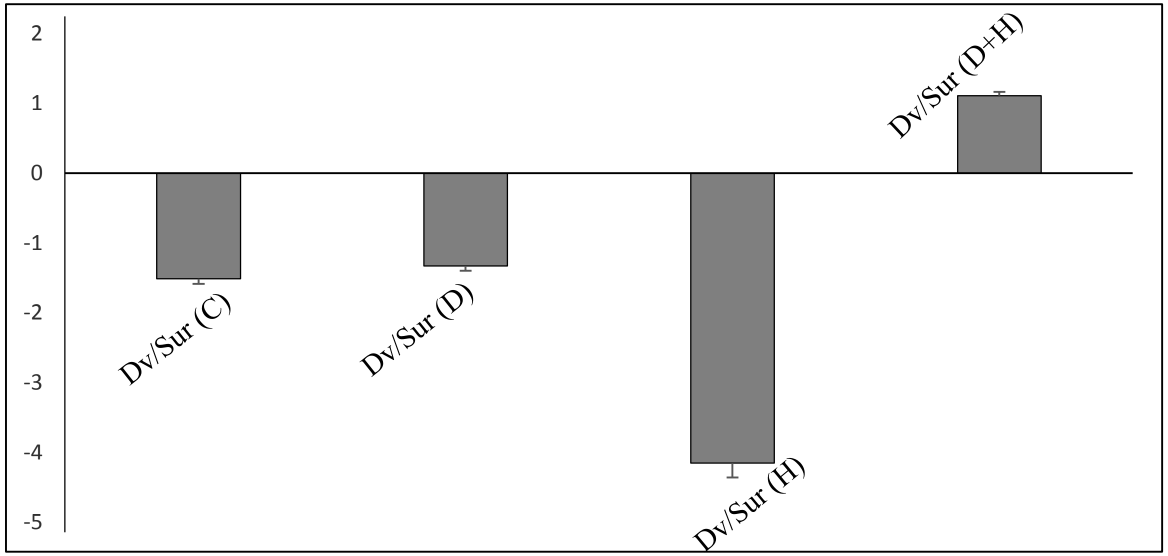

Stromal Heat shock protein in heat stress condition

B

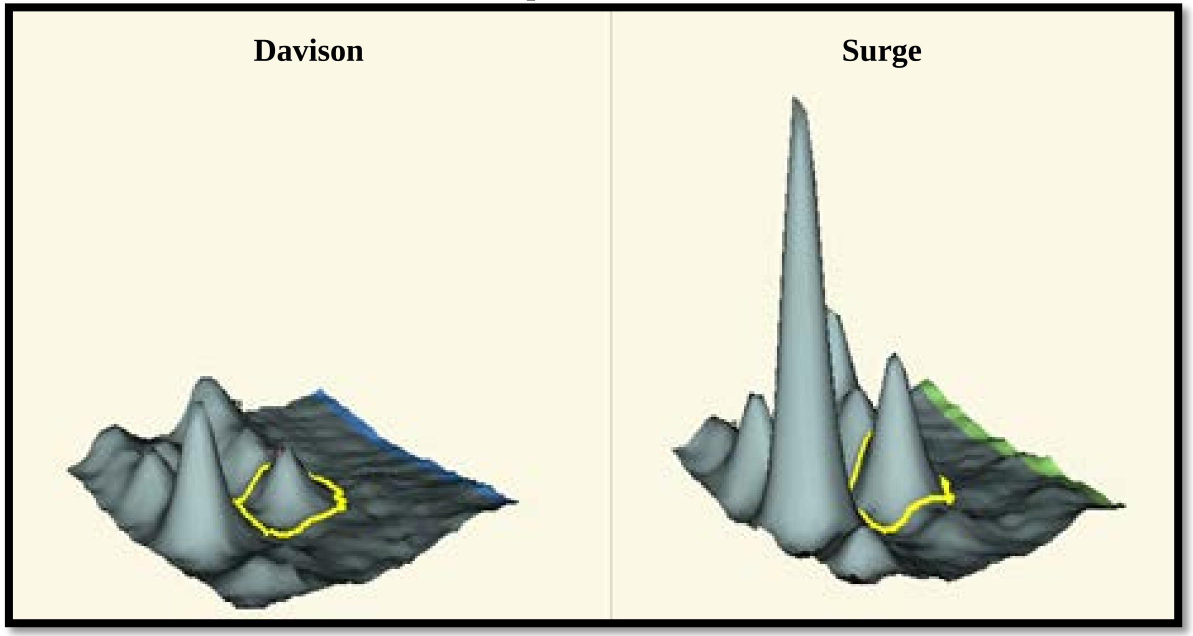

Figure S6

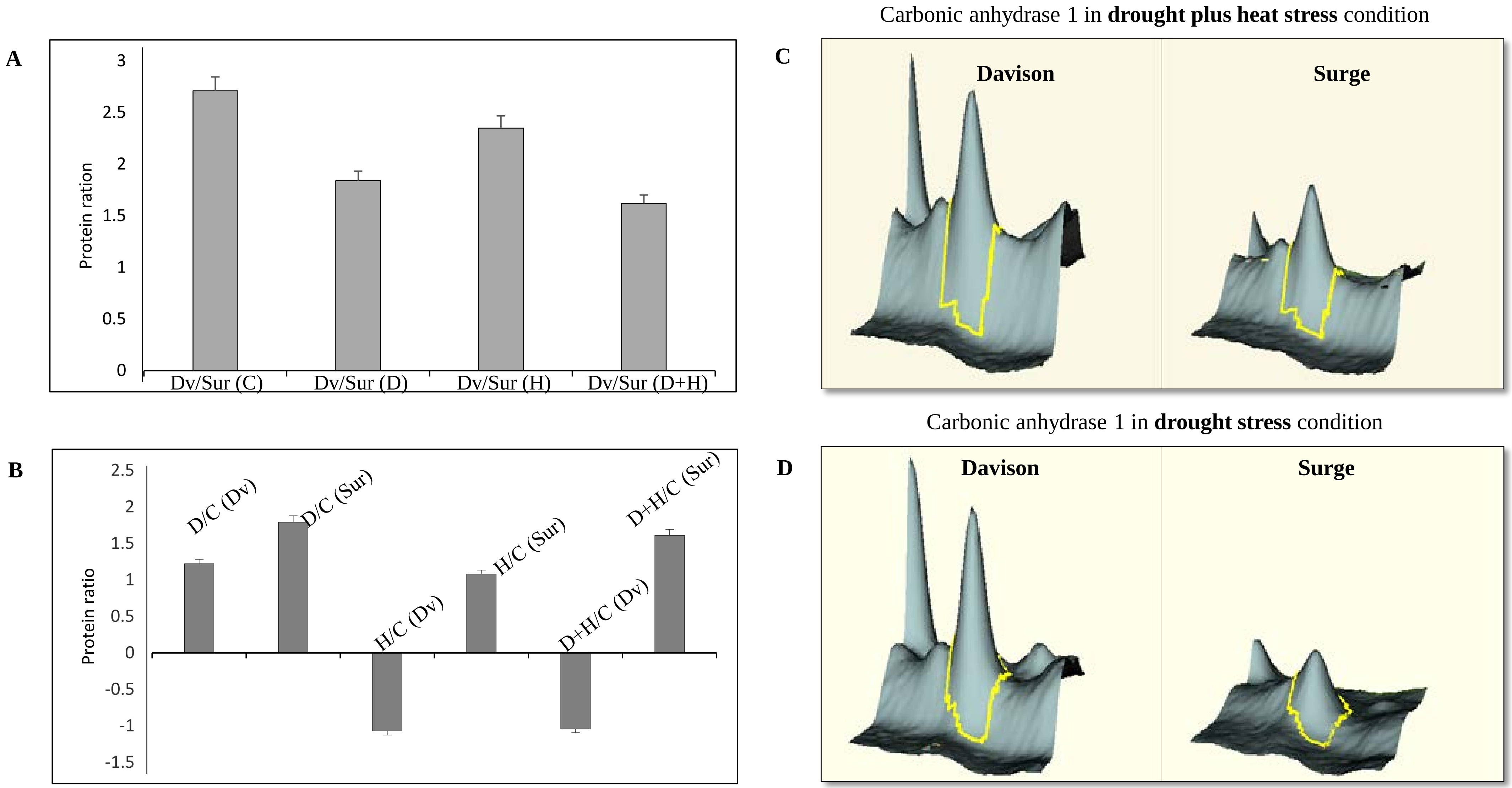
A
Protein ration
3
2.5
2
1.5
1
0.5
0
Dv/Sur (C)
Dv/Sur (D)
Dv/Sur (H)
Dv/Sur (D+H)
B
Protein ratio
2.5
2
1.5
1
0.5
0
-0.5
-1
-1.5
D/C (Dv)
D/C (Sur)
H/C (Dv)
H/C (Sur)
D+H/C (Dv)
D+H/C (Sur)
Carbonic anhydrase 1 in **drought plus heat stress** condition
C
Davison
Surge
Carbonic anhydrase 1 in **drought stress** condition
D
Davison
Surge

Figure S7

Table S1

Spot Map Table: ROJA130919

Gel#	Image name	Gel ID	Type	Label	No.of Spots	Matched	Group	Details of Group (Reference Figure 1)
Gel 1	ROJA130919 Gel-1 (sample 1) Cy3.gel	87216	DIGE Min	Cy3	2660	1997	Condition 1	Davison- D+H - replication 1
	ROJA130919 Gel-1 (sample 13) Cy5.gel	87216	DIGE Min	Cy5	2660	1997	Condition 2	Surge- D+H - replication 1
	ROJA130919 Gel-1 STANDARD CY2.gel	87216	DIGE Min	Cy2	2660	1997	Internal Standard	
Gel 2	ROJA130919 Gel-2 (sample 14) Cy5.gel	95353	DIGE Min	Cy5	2603	1980	Condition 2	Surge- D+H - replication 2
	ROJA130919 Gel-2 (sample 2) Cy3.gel	95353	DIGE Min	Cy3	2603	1980	Condition 1	Davison- D+H - replication 2
	ROJA130919 Gel-2 STANDARD CY2.gel	95353	DIGE Min	Cy2	2603	1980	Internal Standard	
Gel 3	ROJA130919 Gel-3 (sample 15) Cy5.gel	15276	DIGE Min	Cy5	2597	1943	Condition 2	Surge- D+H - replication 3
	ROJA130919 Gel-3 (sample 3) Cy3.gel	15276	DIGE Min	Cy3	2597	1943	Condition 1	Davison- D+H - replication 3
	ROJA130919 Gel-3 STANDARD CY2.gel	15276	DIGE Min	Cy2	2597	1943	Internal Standard	
Gel 4	ROJA130919 Gel-4 (sample 16) Cy5.gel	78820	DIGE Min	Cy5	2577	1951	Condition 4	Surge- Control - replication 1
	ROJA130919 Gel-4 (sample 4) Cy3.gel	78820	DIGE Min	Cy3	2577	1951	Condition 3	Davison- Control - replication 1
	ROJA130919 Gel-4 STANDARD CY2.gel	78820	DIGE Min	Cy2	2577	1951	Internal Standard	
Gel 5	ROJA130919 Gel-5 (sample 17) Cy5.gel	77001	DIGE Min	Cy5	2625	2024	Condition 4	Surge- Control - replication 2
	ROJA130919 Gel-5 (sample 5) Cy3.gel	77001	DIGE Min	Cy3	2625	2024	Condition 3	Davison- Control - replication 2
	ROJA130919 Gel-5 STANDARD CY2.gel	77001	DIGE Min	Cy2	2625	2024	Internal Standard	
Gel 6	ROJA130919 Gel-6 (sample 18) Cy5.gel	49317	DIGE Min	Cy5	2698	2080	Condition 4	Surge- Control - replication 3
	ROJA130919 Gel-6 (sample 6) Cy3.gel	49317	DIGE Min	Cy3	2698	2080	Condition 3	Davison- Control - replication 3
	ROJA130919 Gel-6 STANDARD CY2.gel	49317	DIGE Min	Cy2	2698	2080	Internal Standard	
Gel 7	ROJA130919 Gel-7 (sample 19) Cy5.gel	76213	DIGE Min	Cy5	2799	2799	Condition 6	Surge- Heat - replication 1
	ROJA130919 Gel-7 (sample 7) Cy3.gel	76213	DIGE Min	Cy3	2799	2799	Condition 5	Davison- Heat - replication 1
	ROJA130919 Gel-7 STANDARD CY2.gel	76213	DIGE Min	Cy2	2799	2799	Internal Standard	
Gel 8	ROJA130919 Gel-8 (sample 20) Cy5.gel	74003	DIGE Min	Cy5	2671	2067	Condition 6	Surge- Heat - replication 2
	ROJA130919 Gel-8 (sample 8) Cy3.gel	74003	DIGE Min	Cy3	2671	2067	Condition 5	Davison- Heat - replication 2
	ROJA130919 Gel-8 STANDARD CY2.gel	74003	DIGE Min	Cy2	2671	2067	Internal Standard	
Gel 9	ROJA130919 Gel-9 (sample 21) Cy5.gel	45487	DIGE Min	Cy5	2601	2090	Condition 6	Surge- Heat - replication 3
	ROJA130919 Gel-9 (sample 9) Cy3.gel	45487	DIGE Min	Cy3	2601	2090	Condition 5	Davison- Heat - replication 3
	ROJA130919 Gel-9 STANDARD CY2.gel	45487	DIGE Min	Cy2	2601	2090	Internal Standard	
Gel 10	ROJA130919 Gel-10 (sample 10) Cy3.gel	57557	DIGE Min	Cy3	2666	2007	Condition 7	Davison- Drought - replication 1
	ROJA130919 Gel-10 (sample 22) Cy5.gel	57557	DIGE Min	Cy5	2666	2007	Condition 8	Surge- Drought - replication 1
	ROJA130919 Gel-10 STANDARD CY2.gel	57557	DIGE Min	Cy2	2666	2007	Internal Standard	
Gel 11	ROJA130919 Gel-11 (sample 11) Cy3.gel	54236	DIGE Min	Cy3	2607	1989	Condition 7	Davison- Drought - replication 2
	ROJA130919 Gel-11 (sample 23) Cy5.gel	54236	DIGE Min	Cy5	2607	1989	Condition 8	Surge- Drought - replication 2
	ROJA130919 Gel-11 STANDARD CY2.gel	54236	DIGE Min	Cy2	2607	1989	Internal Standard	
Gel 12	ROJA130919 Gel-12 (sample 12) Cy3.gel	15200	DIGE Min	Cy3	2482	1987	Condition 7	Davison- Drought - replication 3
	ROJA130919 Gel-12 (sample 24) Cy5.gel	15200	DIGE Min	Cy5	2482	1987	Condition 8	Surge- Drought - replication 3
	ROJA130919 Gel-12 STANDARD CY2.gel	15200	DIGE Min	Cy2	2482	1987	Internal Standard	

Table S2

ROJA130919 Protein Ratio
Cut off : 1.5 fold, P value <= 0.1

Assigned ID	Original No.	Appearance	Genotypes comparison for different stresses								Stress Comparison on different genotypes											
			Surge/Davison; Control		Surge/Davison; Drought		Surge/Davison; Heat		Surge/Davison; D+H		Drought/Control; Davison		Drought/Control; Surge		Heat/Control; Davison		Heat/Control; Surge		D+H/C; Davison		D+H/C; Surge	
			**Condition 4 / Condition 3**		**Condition 8 / Condition 7**		**Condition 6 / Condition 5**		**Condition 2 / Condition 1**		**Condition 7 / Condition 3**		**Condition 8 / Condition 4**		**Condition 5 / Condition 3**		**Condition 6 / Condition 4**		**Condition 1 / Condition 3**		**Condition 2 / Condition 4**	
			P-value	Av. Ratio	P-value	Av. Ratio	P-value	Av. Ratio	P-value	Av. Ratio	P-value	Av. Ratio	P-value	Av. Ratio	P-value	Av. Ratio	P-value	Av. Ratio	P-value	Av. Ratio	P-value	Av. Ratio
1	375	33 (36)	0.47	-1.59	0.7	-1.04	0.00088	-2.09	0.23	-1.19	0.71	-1.23	0.78	1.24	0.58	1.11	0.42	-1.18	0.78	1.01	0.26	1.35
2	368	36 (36)	0.11	-2.14	0.47	-1.18	0.0036	-2.8	0.32	-1.18	0.72	1.02	0.26	1.85	0.23	1.42	0.75	1.08	0.45	1.17	0.018	2.12
3	487	33 (36)	0.76	-1.08	0.15	-1.52	0.0064	-2.09	0.58	1.1	0.17	-1.2	0.25	-1.69	0.8	-1.04	0.048	-2.02	0.11	-1.36	0.62	-1.16
4	490	36 (36)	0.33	-1.18	0.05	-1.55	0.0013	-1.71	0.84	-1.04	0.74	-1.02	0.3	-1.34	0.29	1.04	0.18	-1.39	0.32	-1.13	0.84	1.01
5	495	36 (36)	0.41	1.23	0.26	-1.24	0.091	1.39	0.35	1.14	0.42	-1.3	0.017	-1.99	0.63	-1.18	0.82	-1.04	0.24	-1.47	0.03	-1.58
6	500	36 (36)	0.63	1.12	0.27	-1.16	0.061	1.76	0.26	1.17	0.032	-1.7	0.015	-2.2	0.23	-1.41	0.52	1.11	0.019	-2.05	0.017	-1.96
7	511	36 (36)	0.15	1.47	0.038	1.36	0.0069	2.79	0.16	1.23	0.79	1.02	0.67	-1.06	0.21	-1.57	0.12	1.21	0.51	1.17	0.92	-1.02
8	533	36 (36)	0.13	1.51	0.31	1.33	0.01	4.15	0.38	-1.11	0.93	1.01	0.55	-1.12	0.58	-1.18	0.0015	2.33	0.85	1	0.022	-1.66
9	601	36 (36)	0.11	1.82	0.018	1.5	0.0013	3.34	0.0088	1.31	0.7	1.05	0.1	-1.15	0.39	-1.56	0.0015	1.18	0.49	1.18	0.0057	-1.18
10	572	36 (36)	0.15	1.3	0.47	1.09	0.011	1.53	0.57	1.04	0.6	-1.08	0.13	-1.29	0.045	-1.39	0.28	-1.18	0.22	1.2	0.8	-1.04
11	576	36 (36)	0.065	1.29	0.036	1.19	0.023	1.94	0.11	-1.11	0.054	-1.35	0.0016	-1.47	0.24	-1.29	0.15	1.16	0.51	1.06	0.0092	-1.35
12	593	36 (36)	0.2	1.11	0.46	-1.11	0.0077	1.76	0.49	1.08	0.086	-1.26	0.009	-1.55	0.063	-1.34	0.074	1.18	0.22	-1.2	0.015	-1.24
13	684	36 (36)	0.11	1.74	0.49	1.14	0.0068	3.14	0.17	1.11	0.42	1.23	0.2	-1.24	0.54	-1.32	0.024	1.37	0.21	1.45	0.43	-1.08
14	689	36 (36)	0.08	1.94	0.2	1.27	0.0054	3.77	0.22	1.12	0.58	1.14	0.043	-1.34	0.61	-1.29	0.02	1.5	0.28	1.38	0.03	-1.25
15	704	33 (36)	0.18	1.35	0.63	-1.1	0.0059	1.74	0.34	1.21	0.49	1.18	0.19	-1.25	0.9	-1	0.0033	1.28	0.76	1.09	0.67	-1.03
16	727	36 (36)	0.24	-1.3	0.39	-1.17	0.022	-2.18	0.46	1.15	0.44	1.08	0.54	1.2	0.32	1.24	0.31	-1.35	0.83	-1.02	0.17	1.46
17	733	36 (36)	0.99	1.03	0.64	-1.08	0.91	-1.01	0.07	1.54	0.0069	-1.52	0.14	-1.69	0.0096	-1.3	0.2	-1.35	0.012	-1.64	0.73	-1.09
18	781	36 (36)	0.53	-1.34	0.45	-1.22	0.077	1.62	0.6	1.16	0.71	-1.22	0.58	-1.11	0.18	-1.8	0.39	1.2	0.6	-1.29	0.47	1.21
19	779	36 (36)	0.36	-1.19	0.3	-1.31	0.53	-1.07	0.072	1.27	0.042	1.18	0.91	1.07	0.031	-1.22	0.82	-1.09	0.5	1.06	0.077	1.61
20	796	36 (36)	0.27	1.34	0.18	1.11	0.0066	1.74	0.46	1.11	0.69	-1.21	0.00068	-1.47	0.73	-1.19	0.16	1.1	0.61	-1.25	0.0017	-1.51
21	802	36 (36)	0.45	1.22	0.16	-1.28	0.025	2.6	0.25	1.11	0.85	-1.03	0.0015	-1.6	0.45	-1.56	0.0038	1.37	0.52	-1.49	0.0015	-1.64
22	813	36 (36)	0.32	1.36	0.36	-1.18	0.026	2.68	0.081	1.18	0.98	-1.11	0.0021	-1.78	0.44	-1.57	0.0041	1.25	0.45	-1.57	0.0003	-1.83
23	835	36 (36)	0.61	1.08	0.76	1.04	0.031	1.83	1	-1.02	0.54	1.12	0.51	1.09	0.69	-1.14	0.00039	1.47	0.63	1.12	0.84	1.01
24	843	36 (36)	0.98	-1.04	0.063	1.45	0.02	1.65	0.65	-1.07	0.57	-1.16	0.14	1.29	0.49	-1.21	0.0064	1.41	0.8	-1.09	0.11	-1.12
25	888	36 (36)	0.42	1.94	0.064	3.04	0.00052	3.33	0.36	1.35	0.23	1.35	0.26	2.12	0.34	-1.4	0.47	1.23	0.64	1.16	0.95	-1.24
26	857	33 (36)	0.13	1.4	0.099	1.27	0.022	2.8	0.57	1.09	0.39	-1.28	0.082	-1.41	0.15	-1.72	0.2	1.16	0.25	-1.4	0.0039	-1.8
27	941	33 (36)	0.33	1.2	0.26	1.12	0.025	1.88	0.7	-1.02	0.83	1.02	0.53	-1.04	0.22	-1.46	0.24	1.07	0.88	-1	0.025	-1.23
28	940	36 (36)	0.26	2.04	0.17	1.47	0.0003	8.13	0.18	-1.25	0.85	-1.05	0.54	-1.45	0.38	-1.81	0.075	2.21	0.42	1.27	0.21	-2.01
29	988	36 (36)	0.65	1.34	0.83	-1.01	0.016	3.59	0.05	-1.59	0.75	1.04	0.7	-1.31	0.49	-1.51	0.16	1.77	0.49	1.22	0.39	-1.74
30	1001	36 (36)	0.34	1.99	0.56	1.26	0.0071	3.78	0.048	-1.49	0.14	1.73	0.72	1.09	0.77	1.14	0.11	2.16	0.071	1.85	0.57	-1.61
31	997	36 (36)	0.32	1.63	0.37	1.35	0.0018	4.59	0.11	-1.37	0.85	-1	0.69	-1.21	0.48	-1.48	0.074	1.89	0.52	1.2	0.14	-1.88
32	981	36 (36)	0.31	1.57	0.55	1.22	0.0042	4.2	0.13	-1.29	0.32	1.41	0.76	1.1	0.75	-1.22	0.033	2.19	0.23	1.51	0.44	-1.34
33	1031	36 (36)	0.34	1.62	0.29	1.31	0.011	3.93	0.12	-1.22	0.52	1.2	0.95	-1.04	0.89	-1.07	0.047	2.26	0.42	1.24	0.29	-1.6
34	956	36 (36)	0.43	1.32	0.52	1.1	0.077	2.34	0.28	-1.27	0.056	-1.86	0.034	-2.24	0.42	-1.32	0.22	1.34	0.057	-1.93	0.014	-3.23
35	1206	36 (36)	0.3	-1.17	0.027	-1.47	0.0015	-1.57	0.21	-1.16	0.073	1.26	0.96	1.01	0.024	1.4	0.65	1.05	0.12	1.21	0.23	1.22
36	1132	36 (36)	0.31	1.29	0.039	1.47	0.0013	2.35	0.27	1.08	0.32	-1.28	0.56	-1.12	0.036	-1.99	0.63	-1.09	0.77	1.03	0.4	-1.15
37	1133	36 (36)	0.14	1.31	0.076	1.47	0.00032	2.76	0.078	1.21	0.026	-1.44	0.23	-1.29	0.0058	-1.94	0.48	1.08	0.32	-1.14	0.19	-1.23
38	1047	33 (36)	0.72	1.45	0.36	1.28	0.0094	2.84	0.066	-1.89	0.79	-1.08	0.87	-1.23	0.41	-1.36	0.31	1.44	0.56	1.15	0.23	-2.4
39	1081	36 (36)	0.42	2.36	0.25	2.11	6.80E-06	8.71	1.90E-01	-1.79	6.80E-01	-1.14	9.00E-01	-1.28	2.80E-01	-1.47	1.30E-01	2.51	9.60E-01	-1.01	1.50E-01	-4.28
40	1041	36 (36)	0.42	3.47	0.23	2.07	0.0071	30.38	0.2	-1.91	0.78	-1.12	0.94	-1.88	0.32	-3.64	0.21	2.41	0.29	1.67	0.44	-3.96
41	1049	36 (36)	0.35	2.87	0.1	2.43	0.0066	17.56	0.28	-1.44	0.97	-1.53	0.76	-1.81	0.42	-3.1	0.23	1.98	0.42	1.29	0.34	-3.18
42	1076	36 (36)	0.71	-1.05	0.85	1.1	0.024	4.28	0.24	-1.43	0.36	-2.82	0.044	-2.46	0.28	-3.15	0.19	1.42	0.36	-2.81	0.006	-3.83
43	1079	36 (36)	0.48	1.43	0.8	1.22	0.015	7.89	0.095	-1.8	0.38	-4.05	0.054	-4.75	0.34	-4	0.33	1.38	0.22	-6.7	0.0043	-17.27
44	1147	36 (36)	0.083	1.35	0.93	1.01	0.02	1.15	0.89	1.02	0.78	1.01	0.23	-1.32	0.029	1.12	0.83	-1.04	0.007	1.53	0.35	1.15
45	1151	33 (36)	0.1	1.34	0.26	1.1	0.0083	1.54	0.023	1.18	0.78	-1.02	0.098	-1.25	0.098	-1.27	0.35	-1.1	0.83	1.02	0.12	-1.11
46	1170	36 (36)	0.35	1.19	0.7	1.04	0.00054	1.37	0.36	1.16	0.32	-1.31	0.0076	-1.5	0.23	-1.38	0.011	-1.2	0.26	-1.41	0.0092	-1.44
47	1169	36 (36)	0.35	1.23	0.92	1.02	0.0077	1.56	0.083	1.19	0.42	-1.28	0.036	-1.54	0.3	-1.37	0.51	-1.08	0.32	-1.35	0.029	-1.39
48	1187	36 (36)	0.38	1.24	0.35	1.1	0.021	2.09	0.62	1.06	0.94	-1.05	0.2	-1.19	0.64	-1.23	0.024	1.36	0.93	-1.05	0.15	-1.23
49	1185	36 (36)	0.39	1.15	0.99	1.01	0.052	1.67	0.94	1	0.57	1.11	0.79	-1.03	0.89	-1.03	0.0097	1.41	0.32	1.2	0.62	1.04
50	1152	33 (36)	0.26	1.26	0.82	1.02	0.047	1.71	0.52	1.23	0.38	1.19	0.78	-1.04	0.78	-1.06	0.078	1.28	0.89	-1.03	0.75	-1.06
51	1194	36 (36)	0.29	1.34	0.27	1.18	0.043	2.19	0.28	1.16	0.7	1.05	0.61	-1.08	0.88	-1.06	0.017	1.55	0.7	1.05	0.5	-1.1
52	1171	36 (36)	0.21	1.59	0.16	1.46	0.012	3.32	0.24	1.25	0.74	-1.33	0.13	-1.45	0.38	-1.73	0.18	1.2	0.57	-1.48	0.0056	-1.88
53	1348	36 (36)	0.054	-1.34	0.0012	-1.66	0.079	-1.3	0.78	1.02	0.21	1.13	0.37	-1.1	0.4	1.09	0.44	1.12	0.29	-1.29	0.56	1.06
54	1359	36 (36)	0.28	1.23	0.17	-1.12	0.077	1.57	0.074	1.44	0.48	-1.21	0.00019	-1.67	0.54	-1.19	0.73	1.07	0.2	-1.49	0.0041	-1.27
55	1490	36 (36)	0.038	-1.77	0.62	1.11	0.071	-1.5	0.91	1	0.53	1.13	0.005	2.22	0.65	1.11	0.013	1.31	0.61	1.16	0.0026	2.05
56	1423	36 (36)	0.025	-1.38	0.36	-1.17	0.0058	-1.79	0.76	-1.02	0.092	1.24	0.11	1.46	0.037	1.47	0.15	1.13	0.044	1.28	0.0018	1.73
57	1501	36 (36)	0.083	-5.24	0.045	-1.99	0.003	-2.7	0.23	-1.84	0.48	-2.1	0.51	1.26	0.64	-1.8	0.67	1.08	0.63	-1.75	0.26	1.62
58	1635	36 (36)	0.07	-1.21	0.0027	1.31	0.53	-1.07	0.55	1.1	0.37	1.06	0.0012	1.68	0.95	1	0.44	1.13	0.84	-1.01	0.025	1.32
59	1689	36 (36)	0.36	1.09	0.047	1.52	0.061	-1.14	0.062	1.3	0.14	1.2	0.02	1.68	0.02	1.43	0.007	1.16	0.16	1.27	0.00026	1.51
60	1712	36 (36)	0.62	1.04	0.026	1.49	0.67	-1.03	0.037	1.41	0.29	1.18	0.00078	1.69	0.088	1.22	0.14	1.14	0.83	-1.03	0.0044	1.32
61	1745	36 (36)	0.55	1.25	0.0012	2.7	0.44	1.45	0.6	-1.14	0.31	1.14	0.023	2.48	0.62	1.44	0.3	1.68	0.0068	2.87	0.045	2.02
62	1677	36 (36)	0.1	1.46	0.41	1.26	0.0093	1.4	0.2	-1.22	0.53	-1.18	0.16	-1.37	0.94	-1.05	0.2	-1.09	0.83	1.01	0.015	-1.76
63	1565	36 (36)	0.059	-3.5	0.2	-1.42	0.00071	-2.92	0.09	-1.43	0.64	-1.46	0.17	1.69	0.79	-1.33	0.46	-1.11	0.68	-1.42	0.023	1.73
64	1653	36 (36)	0.00065	-1.47	0.057	-1.28	0.052	-1.29	0.56	-1.06	0.024	-1.32	0.081	-1.15	0.012	-1.5	0.0005	-1.31	0.0033	-1.69	0.01	-1.22
65	1633	36 (36)	0.37	1.11	0.29	1.09	0.054	1.42	0.64	1.04	0.054	-1.29	0.032	-1.31	0.083	-1.37	0.51	-1.07	0.0067	-1.41	0.019	-1.5
66	1634	36 (36)	0.079	-3.14	0.019	-2.31	0.0013	-2.56	0.0056	-1.85	0.9	-1.25	0.85	1.09	0.98	-1.16	0.58	1.06	0.81	-1.31	0.013	1.3
67	1578	36 (36)	0.064	-4.01	0.4	-1.24	0.00062	-4.5	0.57	-1.16	0.67	-1.53	0.19	2.11	0.92	-1.3	0.11	-1.46	0.45	-1.81	0.019	1.91
68	1619	36 (36)	0.83	-1.06	0.66	1.09	0.06	1.79	0.98	-1.01	0.26	-1.29	0.44	-1.12	0.18	-1.51	0.13	1.26	0.41	-1.23	0.34	-1.17
69	1639	36 (36)	0.07	-3.83	0.058	-2.09	0.00082	-5.31	0.017	-1.48	0.65	1.01	0.18	1.84	0.58	1.06	0.21	-1.31	0.98	-1.23	0.0019	2.11
70	1522	36 (36)	0.2	3.02	0.26	3.64	0.0035	10	0.58	-1.37	0.36	-1.62	0.61	-1.35	0.2	-2.14	0.29	1.55	0.57	-1.35	0.052	-5.57
71	1605	36 (36)	0.3	-3.12	0.047	-2.55	0.00049	-1.89	0.32	-1.97	0.62	-1.08	0.99	1.13	0.99	-1.51	0.54	1.09	0.7	-1.75	0.67	-1.1
72	1670	36 (36)	0.12	-2.71	0.019	-1.84	0.0011	-2.35	0.022	-1.62	0.43	1.22	0.062	1.79	0.83	-1.07	0.61	1.08	0.79	-1.04	0.059	1.61
73	1579	36 (36)	0.094	-1.3	0.88	1.01	0.062	-1.31	0.76	-1.03	0.36	1.2	0.0051	1.59	0.55	-1.09	0.38	-1.1	0.15	1.22	0.03	1.55
74	1720	36 (36)	0.13	1.35	0.09	1.2	0.034	1.6	0.23	1.16	0.79	-1.07	0.1	-1.21	0.62	-1.13	0.45	1.05	0.75	1.04	0.24	-1.12
75	1737	36 (36)	0.23	1.43	0.58	1.18	0.0084	2.27	0.44	1.13	0.84	-1.13	0.21	-1.37	0.57	-1.27	0.14	1.24	0.23	-1.63	0.0053	-2.08
76	1838	36 (36)	0.25	1.43	0.67	1.22	0.019	1.95	0.66	1.09	0.65	1.08	0.68	-1.08	0.97	-1.02	0.11	1.33	0.32	-1.49	0.025	-1.95
77	1839	36 (36)	0.29	1.86	0.12	2.39	0.00049	3.9	0.38	1.31	0.94	-1.13	0.84	1.14	0.23	-1.8	0.52	1.17	0.5	-1.42	0.21	-2.01
78	1835	36 (36)	0.83	1.02	0.85	1.01	0.017	1.47	0.92	-1.04	0.89	1	0.81	-1.01	0.42	-1.17	0.1	1.22	0.15	-1.45	0.015	-1.53
79	1950	36 (36)	0.25	1.62	0.35	1.87	0.0042	3.75	0.17	1.29	0.81	-1.17	0.79	-1.01	0.23	-1.75	0.28	1.32	0.36	-1.5	0.083	-1.89
80	2196	36 (36)	0.12	1.54	0.017	1.54	0.0012	2.4	0.068	1.29	0.72	-1.14	0.4	-1.13	0.23	-1.46	0.46	1.07	0.94	-1.06	0.14	-1.27
81	2332	36 (36)	0.74	1.05	0.4	1.28	0.088	1.36	0.88	-1.03	0.2	1.31	0.11	1.59	0.36	1.19	0.018	1.54	0.098	1.38	0.14	1.28
82	2516	36 (36)	0.41	2.04	0.16	4.74	0.014	4.79	0.5	-1.8	0.99	-1.01	0.48	2.29	0.2	-1.56	0.39	1.51	0.57	1.73	0.37	-2.13
83	2482	36 (36)	0.32	2.37	0.15	4.29	0.00078	7.91	0.55	1.33	0.93	-1.11	0.67	1.63	0.069	-2.39	0.39	1.4	0.4	-1.44	0.28	-2.57
84	2512	36 (36)	0.57	1.48	0.34	2.54	0.00047	5.22	0.051	1.24	0.06	-1.54	0.92	1.11	0.0026	-2.49	0.33	1.41	0.0067	-2.08	0.13	-2.48
85	2503	36 (36)	0.55	1.25	0.65	1.29	0.027	2.36	0.065	1.31	0.27	-1.3	0.53	-1.26	0.051	-2.06	0.88	-1.09	0.018	-1.93	0.098	-1.83
86	2484	36 (36)	0.27	1.65	0.29	2.11	0.0012	3.42	0.0046	1.21	0.064	-1.35	0.78	-1.06	0.0047	-1.93	0.68	1.07	0.0064	-1.7	0.075	-2.32
87	2593	36 (36)	0.44	-1.27	0.24	-1.45	0.88	-1.15	0.056	1.68	0.11	-1.7	0.12	-1.94	0.062	-2.45	0.082	-2.21	0.012	-2.75	0.55	-1.28
88	2610	36 (36)	0.29	-1.27	0.081	-1.36	0.006	-1.51	0.4	1.07	0.1	-1.44	0.067	-1.54	0.32	-1.18	0.13	-1.41	0.044	-1.5	0.66	-1.11
89	2616	36 (36)	0.091	-1.52	0.15	-1.48	0.16	-1.46	0.1	1.27	0.045	-1.75	0.075	-1.7	0.27	-1.29	0.37	-1.23	0.0043	-2.36	0.39	-1.22
90	2705	36 (36)	0.92	1.02	1	-1.02	0.11	1.72	0.31	-1.24	0.23	1.56	0.27	1.49	0.62	1.29	0.055	2.17	0.068	1.77	0.29	1.39

**Assigned ID**	Unique spot number that was manually assigned (1-90)
**Original No.**	Unique spot number assigned by DeCyder software
**Appearance**	# of images the spots can be detected (12 gels x 3 images (Cy2, 3, 5)) = 36
**P value**	Student t test
**Av. Ratio: 4/3**	Positive value means increased ratio (up-regulated in 4)
	Negative value means decreased ratio (down-regulated in 4)
**Red Font**	Spots that meet a 1.5 fold change and p-value <= 0.1 in each comparison

Table S 3

Project Code: ROJA131014

Spot number	MALDI well number	Match Quality	Top Ranked Protein Name [S	Accession No.	E-value	# Un-matched peptide	% Coverage	Protein MW	Protein PI	Pep.Count	Protein Score	Protein Score C.I.%	Total Ion Score	Total Ion C.I.%	Comments
B1	1		Acyl-[acyl-carrier-protein] desaturase 6, chloroplastic OS=Oryza sativa subsp. japonica (	STAD6_ORYSJ	26	64	14	46,071	7.65	8	31				
B2	2		lipoxygenase [Glycine max]	gi|351722222	8.60E-23	39	40	96,241	6.29	21	246	100	123	100	
B3	3		Ribulose bisphosphate carboxylase large chain OS=Liquidambar styraciflua GN=rbcL P	RBL_LIQST	9.80E-05	60	30	52,626	6.04	15	86	100	10		
B4	4		Transketolase, chloroplastic OS=Spinacia oleracea PE=1 SV=1	TKTC_SPIOL	0.0035	68	10	80,231	6.20	5	70	100	61	100	
B5	5		Transketolase, chloroplastic OS=Spinacia oleracea PE=1 SV=1	TKTC_SPIOL	0.009	64	14	80,231	6.20	7	66	99	50	100	
B6	6		Transketolase, chloroplastic OS=Spinacia oleracea PE=1 SV=1	TKTC_SPIOL	1.8-008	63	12	80,231	6.20	7	123	100	108	100	
B7	7		Stromal 70 kDa heat shock-related protein, chloroplastic OS=Pisum sativum GN=HSP7	HSP7S_PEA	1.40E-50	50	25	75,469	5.22	16	544	100	468	100	
B8	8		Stromal 70 kDa heat shock-related protein, chloroplastic OS=Pisum sativum GN=HSP7	HSP7S_PEA	7.10E-09	62	17	75,469	5.22	10	127	100	99	100	could also contain hit#2: Ribulose bisphosphate carboxylase large chain
B9	9		ATP-dependent zinc metalloprotease FTSH 8, chloroplastic OS=Arabidopsis thaliana G	FTSH8_ARATH	0.003	62	19	73,153	5.72	9	71	100	45	100	
B10	10		Ribulose bisphosphate carboxylase large chain OS=Phaseolus vulgaris GN=rbcL PE=3	RBL_PHAVU	2.80E-13	57	33	52,768	5.96	13	171	100	102	100	
B11	11		Ribulose bisphosphate carboxylase large chain OS=Sesbania sesban GN=rbcL PE=3 S	RBL_SESSE	3.60E-13	54	41	52,358	6.09	16	170	100	73	100	
B12	12		Ribulose bisphosphate carboxylase large chain OS=Hyophorbe lagenicaulis GN=rbcL P	RBL_HYOLA	0.0031	61	40	52,929	6.13	13	71	100	6		
B13	13		RuBisCO large subunit-binding protein subunit beta, chloroplastic OS=Pisum sativum P	RUBB_PEA	3.60E-25	53	32	62,945	5.85	15	290	100	216	100	
B14	14		RuBisCO large subunit-binding protein subunit beta, chloroplastic OS=Pisum sativum P	RUBB_PEA	9.00E-23	53	32	62,945	5.85	15	266	100	192	100	
B15	15		RuBisCO large subunit-binding protein subunit alpha (Fragment) OS=Ricinus communis	RUBA_RICCO	9.00E-25	53	40	52,347	4.77	14	286	100	210	100	
B16	16		ATP synthase subunit alpha, chloroplastic OS=Glycine max GN=atpA PE=3 SV=1	ATPA_SOYBN	1.80E-63	38	53	55,719	5.15	22	673	100	502	100	
B17	17		ATP synthase subunit alpha, chloroplastic OS=Glycine max GN=atpA PE=3 SV=1	ATPA_SOYBN	2.80E-71	37	55	55,719	5.15	23	751	100	567	100	
B18	18		Ribulose bisphosphate carboxylase large chain OS=Glycine max GN=rbcL PE=1 SV=3	RBL_SOYBN	4.50E-89	35	59	52,576	6.00	25	929	100	722	100	
B19	19		ATP synthase subunit beta, chloroplastic OS=Glycine max GN=atpB PE=3 SV=1	ATPB_SOYBN	7.10E-84	29	77	53,721	5.29	28	877	100	614	100	
B20	20		ATP synthase subunit beta, chloroplastic OS=Glycine max GN=atpB PE=3 SV=1	ATPB_SOYBN	1.40E-94	26	81	53,721	5.29	30	984	100	689	100	
B21	21		Ribulose bisphosphate carboxylase large chain OS=Glycine max GN=rbcL PE=1 SV=3	RBL_SOYBN	1.10E-85	32	59	52,576	6.00	26	895	100	671	100	
B22	22		Ribulose bisphosphate carboxylase large chain OS=Glycine max GN=rbcL PE=1 SV=3	RBL_SOYBN	2.80E-92	34	59	52,576	6.00	25	961	100	752	100	
B23	23		Glutamate--glyoxylate aminotransferase 1 OS=Arabidopsis thaliana GN=GGAT1 PE=1 S	GGT1_ARATH	1.80E-16	57	25	53,267	6.49	8	203	100	173	100	
B24	24		Glutamate--glyoxylate aminotransferase 1 OS=Arabidopsis thaliana GN=GGAT1 PE=1 S	GGT1_ARATH	5.70E-14	56	26	53,267	6.49	10	178	100	133	100	
C1	25		ATP synthase subunit alpha, chloroplastic OS=Glycine max GN=atpA PE=3 SV=1	ATPA_SOYBN	9.00E-25	52	37	55,719	5.15	15	286	100	202	100	
C2	26		Ribulose bisphosphate carboxylase large chain (Fragment) OS=Fragaria ananassa GN=	RBL_FRAAN	0.0024	61	34	51,629	6.04	13	72	100	4		fragment?
C3	27		Ribulose bisphosphate carboxylase large chain OS=Cajanus cajan GN=rbcL PE=3 SV=	RBL_CAJCA	0.0027	58	22	52,359	6.45	13	71	100	4		
C4	28		Ribulose bisphosphate carboxylase/oxygenase activase, chloroplastic OS=Malus domes	RCA_MALDO	2.80E-41	54	36	48,046	8.20	12	451	100	392	100	C-terminus?
C5	29		Elongation factor Tu, chloroplastic OS=Glycine max GN=TUFA PE=3 SV=1	EFTU1_SOYBN	4.50E-08	65	24	52,063	6.21	8	119	100	91	100	see detail hits for other possibilities
C6	30		Elongation factor Tu, chloroplastic OS=Glycine max GN=TUFA PE=3 SV=1	EFTU1_SOYBN	2.30E-14	59	32	52,063	6.21	11	182	100	132	100	see detail hits for other possibilities
C7	31		Elongation factor Tu, chloroplastic OS=Glycine max GN=TUFA PE=3 SV=1	EFTU1_SOYBN	2.80E-30	56	34	52,063	6.21	11	341	100	290	100	
C8	32		Ribulose bisphosphate carboxylase large chain (Fragment) OS=Streptopus lanceolatus	RBL_STRLC	0.01	61	26	50,649	6.18	13	66	99			
C9	33		Glutamine synthetase leaf isozyme, chloroplastic OS=Phaseolus vulgaris PE=2 SV=1	GLNA4_PHAVU	2.80E-15	63	28	47,217	6.77	8	191	100	158	100	
C10	34		S-adenosylmethionine synthase 4 OS=Vitis vinifera GN=METK4 PE=3 SV=1	METK4_VITVI	3.60E-44	50	54	42,904	5.50	17	480	100	356	100	
C11	35		Probable calcium-binding protein CML33	CML33_ARATH	5.7	67	45	15,058	4.40	6	38	0			could be hit#2, 3 or 4
C12	36		Ribulose bisphosphate carboxylase large chain OS=Liquidambar styraciflua GN=rbcL P	RBL_LIQST	7.1e-007	59	45	52,626	6.04	15	107	100	26	60	
C13	37		Sedoheptulose-1,7-bisphosphatase, chloroplastic OS=Spinacia oleracea PE=2 SV=1	S17P_SPIOL	5.70E-08	66	17	42,054	5.87	6	118	100	100	100	
C14	38		Ribulose bisphosphate carboxylase/oxygenase activase, chloroplastic OS=Vigna radiata	RCA_VIGRR	9.00E-24	62	25	47,871	7.57	9	276	100	243	100	
C15	39		Ribulose bisphosphate carboxylase/oxygenase activase, chloroplastic OS=Vigna radiata	RCA_VIGRR	2.30E-18	58	28	47,871	7.57	9	222	100	186	100	
C16	40		Ribulose bisphosphate carboxylase/oxygenase activase, chloroplastic OS=Vigna radiata	RCA_VIGRR	1.8e-059	48	39	47,871	7.57	16	633	100	535	100	
C17	41		Ribulose bisphosphate carboxylase/oxygenase activase, chloroplastic OS=Vigna radiata	RCA_VIGRR	2.80E-58	50	37	47,871	7.57	13	621	100	551	100	
C18	42		Phosphoglycerate kinase, chloroplastic OS=Triticum aestivum PE=2 SV=1	PGKH_WHEAT	2.80E-29	58	26	49,809	6.58	11	331	100	280	100	
C19	43		Phosphoglycerate kinase, chloroplastic OS=Triticum aestivum PE=2 SV=1	PGKH_WHEAT	1.10E-29	58	34	49,809	6.58	11	335	100	283	100	
C20	44		Photosystem II stability/assembly factor HCF136, chloroplastic OS=Arabidopsis thaliana	P2SAF_ARATH	5.70E-25	53	46	44,076	6.79	15	288	100	192	100	
C21	45		Photosystem II stability/assembly factor HCF136, chloroplastic OS=Oryza sativa subsp.	P2SAF_ORYSJ	1.10E-12	61	31	45,441	9.02	10	165	100	118	100	
C22	46		Fructose-bisphosphate aldolase 1, chloroplastic (Fragment) OS=Pisum sativum PE=1 S	ALFC1_PEA	7.10E-12	68	15	38,633	5.83	4	157	100	145	100	
C23	47		Fructose-bisphosphate aldolase 1, chloroplastic (Fragment) OS=Pisum sativum PE=1 S	ALFC1_PEA	2.80E-25	60	26	38,633	5.83	8	291	100	253	100	
C24	48		Fructose-bisphosphate aldolase 1, chloroplastic (Fragment) OS=Pisum sativum PE=1 S	ALFC1_PEA	1.80E-12	64	21	38,633	5.83	6	163	100	140	100	
D1	49		Malate dehydrogenase 1, mitochondrial OS=Arabidopsis thaliana GN=At1g53240 PE=1	MDHM1_ARATH	0.0023	70	14	35,782	8.54	3	72	100	63	100	see detail hits
D2	50		Trypsin inhibitor 1 OS=Trichosanthes kirilowii PE=3 SV=1	ITR1_TRIKI	1.1	63	66	6,872	4.61	6	45				could be hit#3: ATP-dependent Clp protease proteolytic subunit-related protein 1
D3	51		Fructose-bisphosphate aldolase 1, chloroplastic (Fragment) OS=Pisum sativum PE=1 S	ALFC1_PEA	7.1e-016	66	21	38,633	5.83	6	197	100	174	100	
D4	52		Chloroplast stem-loop binding protein of 41 kDa b, chloroplastic OS=Arabidopsis thalian	CP41B_ARATH	3.60E-13	65	21	42,593	8.19	7	170	100	143	100	
D5	53		Ferredoxin--NADP reductase, leaf isozyme 1, chloroplastic OS=Arabidopsis thaliana GN	FNRL1_ARATH	3.6e-024	57	23	40,301	8.32	9	280	100	244	100	
D6	54		Ferredoxin--NADP reductase, leaf isozyme 1, chloroplastic OS=Arabidopsis thaliana GN	FNRL1_ARATH	4.50E-21	56	23	40,301	8.32	9	249	100	213	100	
D7	55		ATP synthase subunit beta, chloroplastic OS=Glycine max GN=atpB PE=3 SV=1	ATPB_SOYBN	9.00E-15	49	43	53,721	5.29	13	186	100	112	100	fragment?
D8	56		Oxygen-evolving enhancer protein 1, chloroplastic OS=Pisum sativum GN=PSBO PE=2	PSBO_PEA	1.80E-39	57	32	34,872	6.25	10	433	100	375	100	
D9	57		Ribulose bisphosphate carboxylase large chain (Fragment) OS=Prunus domestica GN=	RBL_PRUDO	0.00016	53	32	50,915	6.23	14	84	100	7		C-terminus?
D10	58		Chlorophyll a-b binding protein of LHCII type I, chloroplastic (Fragment) OS=Cucumis sa	CB21_CUCSA	7.10E-27	59	58	27,218	5.14	8	307	100	254	100	
D11	59		Chlorophyll a-b binding protein, chloroplastic OS=Oryza sativa subsp. japonica GN=RCA	CB23_ORYSJ	2.30E-09	66	12	28,477	5.62	4	132	100	117	100	
D12	60		Chlorophyll a-b binding protein 13, chloroplastic OS=Solanum lycopersicum GN=CAB13	CB23_SOLLC	1.10E-21	63	27	28,604	5.09	6	255	100	226	100	
D13	61		2-Cys peroxiredoxin BAS1-like, chloroplastic OS=Arabidopsis thaliana GN=At5g06290 F	BAS1B_ARATH	4.50E-12	65	20	29,761	5.55	5	159	100	137	100	could also contain hit#2: Protein P21
D14	62		Ribulose bisphosphate carboxylase small chain PW9, chloroplastic OS=Triticum aestivu	RBS2_WHEAT	3.4	66	63	19,442	8.52	7	40	0			
D15	63		Ribulose bisphosphate carboxylase small chain 2A, chloroplastic OS=Solanum tuberosu	RBSA_SOLTU	0.039	64	50	20,276	7.60	9	60	96			
D16	64		Superoxide dismutase [Fe], chloroplastic OS=Glycine max GN=SODB PE=2 SV=1	SODF_SOYBN	2.30E-33	58	41	27,824	5.60	8	372	100	322	100	
D17	65		Superoxide dismutase [Fe], chloroplastic OS=Glycine max GN=SODB PE=2 SV=1	SODF_SOYBN	2.80E-30	57	49	27,824	5.60	9	341	100	282	100	
D18	66		Carbonic anhydrase 1 OS=Flaveria linearis PE=2 SV=1	CAH1_FLALI	2.80E-06	68	25	35,552	5.70	5	101	100	84	100	
D19	67		RuBisCO-associated protein OS=Glycine max PE=2 SV=1	RUAP_SOYBN	9.00E-10	57	42	31,239	6.00	8	136	100	88	100	
D20	68		Ribulose bisphosphate carboxylase large chain (Fragment) OS=Byrsonima crassifolia G	RBL_BYRCR	0.0033	61	29	51,555	6.09	13	70	100	6		
D21	69		Ribulose bisphosphate carboxylase large chain (Fragment) OS=Manilkara zapota GN=rl	RBL_MANZA	0.00071	60	25	51,550	6.04	10	77	100	33	95	
D22	70		Ribulose bisphosphate carboxylase large chain OS=Glycine max GN=rbcL PE=1 SV=3	RBL_SOYBN	3.60E-59	35	40	52,576	6.00	20	630	100	484	100	fragment?
D23	71		Stem 31 kDa glycoprotein OS=Glycine max GN=VSPB PE=2 SV=1	VSPB_SOYBN	1.10E-57	49	55	29,262	6.72	14	615	100	499	100	
D24	72		Carbonic anhydrase OS=Flaveria bidentis PE=2 SV=2	CAHX_FLABI	0.0035	66	19	35,523	5.85	4	70	100	57	100	
E1	73		Stem 28 kDa glycoprotein OS=Glycine max GN=VSPA PE=2 SV=1	VSPA_SOYBN	0.00017	62	33	29,047	8.75	7	83	100	45	100	see detail hit#2
E2	74		Oxygen-evolving enhancer protein 2-1, chloroplastic OS=Nicotiana tabacum GN=PSBP	PSBP1_TOBAC	1.40E-07	64	32	28,634	6.84	6	114	100	86	100	
E3	75		Chlorophyll a-b binding protein 3, chloroplastic OS=Pisum sativum GN=lhca3 PE=1 SV=	CB23_PEA	0.23	67	7	29,588	8.84	3	51	73	41	99	
E4	76		Chlorophyll a-b binding protein 6A, chloroplastic OS=Solanum lycopersicum GN=CAB6A	CB11_SOLLC	1.80E-19	63	26	26,557	5.82	5	233	100	209	100	
E5	77		Ribulose bisphosphate carboxylase large chain (Fragment) OS=Garrya elliptica GN=rbc	RBL_GAREL	2.80E-46	58	27	50,885	6.34	9	501	100	462	100	
E6	78		Chlorophyll a-b binding protein 6, chloroplastic OS=Arabidopsis thaliana GN=LHCA1 PE	CAB6_ARATH	2.30E-11	65	9	25,979	6.21	4	152	100	135	100	
E7	79		Ribulose bisphosphate carboxylase large chain OS=Glycine max GN=rbcL PE=1 SV=3	RBL_SOYBN	2.80E-49	61	18	52,576	6.00	6	531	100	511	100	fragment?
E8	80		Ribosomal protein L7/L12 [Crocosphaera watsonii WH 8501]	gi|67924345	0.088	69	17	13,803	4.73	2	80	91	71	100	hit#8 is the correct one
E9	81		Glycine cleavage system H protein 1, mitochondrial OS=Arabidopsis thaliana GN=GDC	GCSH1_ARATH	0.0053	64	30	17,936	5.24	4	68	100	46	100	
E10	82		Ribulose bisphosphate carboxylase large chain OS=Sesbania sesban GN=rbcL PE=3 S	RBL_SESSE	4.50E-18	60	22	52,358	6.09	8	219	100	187	100	N-terminal fragment
E11	83		Ribulose bisphosphate carboxylase large chain OS=Sesbania sesban GN=rbcL PE=3 S	RBL_SESSE	1.80E-29	60	23	52,358	6.09	9	333	100	295	100	N-terminal fragment
E12	84		Ribulose bisphosphate carboxylase large chain OS=Glycine max GN=rbcL PE=1 SV=3	RBL_SOYBN	1.80E-34	53	21	52,576	6.00	11	383	100	330	100	C-terminus
E13	85		Ribulose bisphosphate carboxylase large chain OS=Glycine max GN=rbcL PE=1 SV=3	RBL_SOYBN	9.00E-24	55	24	52,576	6.00	11	276	100	222	100	C-terminus
E14	86		Ribulose bisphosphate carboxylase large chain OS=Glycine max GN=rbcL PE=1 SV=3	RBL_SOYBN	5.70E-26	55	19	52,576	6.00	10	298	100	253	100	C-terminus
E15	87		Ribulose bisphosphate carboxylase small chain 1, chloroplastic OS=Glycine max GN=R	RBS1_SOYBN	2.80E-48	54	61	20,060	8.87	12	521	100	429	100	
E16	88		Ribulose bisphosphate carboxylase small chain 1, chloroplastic OS=Glycine max GN=R	RBS1_SOYBN	2.80E-52	53	61	20,060	8.87	12	561	100	468	100	
E17	89		Ribulose bisphosphate carboxylase small chain 1, chloroplastic OS=Glycine max GN=R	RBS1_SOYBN	5.70E-44	57	61	20,060	8.87	11	478	100	398	100	
E18	90		Ribulose bisphosphate carboxylase small chain 1, chloroplastic OS=Glycine max GN=R	RBS1_SOYBN	3.60E-48	52	69	20,060	8.87	13	520	100	415	100	
E19	91		Metalloendoproteinase 1 OS=Glycine max PE=1 SV=2	MEP1_SOYBN	4.50E-08	59	36	34,023	6.29	9	119	100	68	100	
E20	92		Ribulose bisphosphate carboxylase large chain OS=Glycine max GN=rbcL PE=1 SV=3	RBL_SOYBN	2.80E-41	36	45	52,576	6.00	22	451	100	279	100	

Control

1 fmol	E21		beta-D-galactosidase [Escherichia coli IAI39]	gi|218698747	1.70E-29	44	23	116583.2	5.15	21	357	100	262	100
1 fmol	E22		beta-D-galactosidase [Escherichia coli IAI39]	gi|218698747	4.30E-40	41	24	116583.2	5.15	22	463	100	357	100
1 fmol	E23		beta-D-galactosidase [Escherichia coli IAI39]	gi|218698747	2.20E-26	45	22	116583.2	5.15	20	326	100	240	100
1 fmol	E24		beta-D-galactosidase [Escherichia coli IAI39]	gi|218698747	8.60E-23	44	22	116583.2	5.15	21	290	100	196	100

high confidence
low confidence
no confidence

1. Name of peaklist-generating software and release version (number or date): MASCOT version 2.4
2. Name of the search engine and release version(number or date): MASCOT version 2.4
3. Name of sequence database/spectral library searched: SwissProt (updated weekly)
4. # of entries in the database (or subset of database) actually searched: 546790 sequences
5. Specificity of all proteases used to generate peptides: Arg-C, Lys-C
6. # of missed and/or non-specific cleavages permitted: 1
7. List of all fixed modification(s) (including residue specificity) considered: Carbamidomethyl (C), Oxidation (M)
8. List of all variable modification(s) (including residue specificity) considered:
9. Mass tolerance for precursor ions: 100ppm
10. Mass tolerance for fragment ions (not required for PMFdata): 0.5 Dalton
11. Known contaminants excluded (primarily for PMF data): Trypsin peaks
12. Threshold score/Expectation value for accepting individual spectra: Column G
13. Justification of the threshold score/expectation value employed: 95% Confidence interval
14. Precursor charge and mass/charge (m/z) for each assignment: +1
15. # of matched and unmatched masses (for PMFdata): Column G and L
16. Peptide Identification Score(s): see table
17. For all protein identifications:
    i. Accession # (and database from which it is derived): Yes
    ii. # of distinct peptides assigned for each protein: Column L
    iii. % coverage of each protein assigned (or derived protein identification probability): Column I

Table S4

2013_10
ROJA131014

Analysis Information

**Report Type**	Protein-Peptide Summary by Spot	**Analysis Type**	Combined (MS+MS/MS)
**Sample Set Name**	ROJA131014	**Database**	
**Analysis Name**		**Creation Date**	
**Reported By**		**Last Modified**	

MS Acq. : Proc. Methods (Unspecified) : (Unspecified)
Interpretation Method (Unspecified)

**Gel Idx/Pos**	28/B1	**Instr./Gel Origin**	BA2060/131017A	**Process Sta**	Analysis Succeeded
**Plate [#] Name**	[1] 31496	**Instrument Sample Name**		**Spectra**	11

Rank	Protein Name	Accession No	Protein MW	Protein PI	Pep. Count	Protein Score	Protein Score C. I. %	Total Ion Score	Total Ion C. I. %
1	Acyl-[acyl-carrier-protein] desaturase 6, chloropla OS=Oryza sativa subsp. japonica GN=Os08g019	STAD6 ORYS	46071.3	7.65	8	31	0		

Peptide Information

Calc. Mass	Obsrv. Mass	± da	± ppm	Start Seq.	End Seq.	Sequence	Ion Score	C. I. %	Modification	Rank	Result Type
937.3567	937.3203	-0.0364	-39	66	72	EEDEEMR					Mascot
996.4454	996.4606	0.0152	15	206	213	FDMAEVER					Mascot
1094.5187	1094.4124	-0.1063	-97	374	382	DYVCELPQK					Mascot
1094.5187	1094.4124	-0.1063	-97	374	382	DYVCELPQK					Mascot
1334.6117	1334.5129	-0.0988	-74	17	28	CKPNTNSSSPSR			Carbamidomethyl (C)[1]		Mascot
1378.6783	1378.5092	-0.1691	-123	372	382	ARDYVCELPQK			Carbamidomethyl (C)[6]		Mascot
1465.696	1465.5332	-0.1628	-111	1	14	MAATATMAMPLANR			Oxidation (M)[1]		Mascot
1465.696	1465.5332	-0.1628	-111	1	14	MAATATMAMPLANR			Oxidation (M)[1]		Mascot
1587.8458	1587.627	-0.2188	-138	2	16	AATATMAMPLANRLR					Mascot
1587.8458	1587.627	-0.2188	-138	2	16	AATATMAMPLANRLR					Mascot
1866.8513	1866.7742	-0.0771	-41	199	213	YMYLSGRFDMAEVER					Mascot

Rank	Protein Name	Accession No	Protein MW	Protein PI	Pep. Count	Protein Score	Protein Score C. I. %	Total Ion Score	Total Ion C. I. %
2	Protein GLUTAMINE DUMPER 4 OS=Arabidopsis thaliana GN=GDU4 PE=2 SV=1	GDU4_ARATH	16840.3	5.4	5	29	0		

Peptide Information

Calc. Mass	Obsrv. Mass	± da	± ppm	Start Seq.	End Seq.	Sequence	Ion Score	C. I. %	Modification	Rank	Result Type
Calc. Mass	Obsrv. Mass	± da	± ppm	Seq.	Seq.	Sequence	Score	C. I. %	Modification	Rank	Result Type
937.3745	937.3203	-0.0542	-58	149	156	ENEETTSQ					Mascot
1006.3651	1006.2161	-0.149	-148	118	125	CMCGHEGR			Carbamidomethyl (C)[1,3]		Mascot
1638.8043	1638.5771	-0.2272	-139	126	140	MVIFKEDGIGAGEEK			Oxidation (M)[1]		Mascot
1640.7164	1640.627	-0.0894	-54	118	130	CMCGHEGRMVIFK			Carbamidomethyl (C)[1,3], Oxidation (M)[2]		Mascot
1821.8193	1821.6693	-0.15	-82	109	125	FLATPAANKCMCGHEGR			Oxidation (M)[11]		Mascot

Rank	Protein Name	Accession No	Protein MW	Protein PI	Pep. Count	Protein Score	Protein Score C. I. %	Total Ion Score	Total Ion C. I. %
3	Acyl-[acyl-carrier-protein] desaturase 6, chloropla OS=Oryza sativa subsp. indica GN=OsI_28171	STAD6_ORYS	46535.5	7.16	8	29	0		

Peptide Information

Calc. Mass	Obsrv. Mass	± da	± ppm	Start Seq.	End Seq.	Sequence	Ion Score	C. I. %	Modification	Rank	Result Type
937.3567	937.3203	-0.0364	-39	69	75	EEDEEMR					Mascot
996.4454	996.4606	0.0152	15	209	216	FDMAEVER					Mascot
1094.5187	1094.4124	-0.1063	-97	377	385	DYVCELPQK					Mascot
1094.5187	1094.4124	-0.1063	-97	377	385	DYVCELPQK					Mascot
1334.6117	1334.5129	-0.0988	-74	17	28	CKPNTNSSSPSR			Carbamidomethyl (C)[1]		Mascot
1378.6783	1378.5092	-0.1691	-123	375	385	ARDYVCELPQK			Carbamidomethyl (C)[6]		Mascot
1465.696	1465.5332	-0.1628	-111	1	14	MAATATMAMPLANR			Oxidation (M)[1]		Mascot
1465.696	1465.5332	-0.1628	-111	1	14	MAATATMAMPLANR			Oxidation (M)[1]		Mascot
1587.8458	1587.627	-0.2188	-138	2	16	AATATMAMPLANRLR					Mascot
1587.8458	1587.627	-0.2188	-138	2	16	AATATMAMPLANRLR					Mascot
1866.8513	1866.7742	-0.0771	-41	202	216	YMYLSGRFDMAEVER					Mascot

Rank	Protein Name	Accession No	Protein MW	Protein PI	Pep. Count	Protein Score	Protein Score C. I. %	Total Ion Score	Total Ion C. I. %
4	Probable phospholipid hydroperoxide glutathione peroxidase OS=Spinacia oleracea PE=2 SV=1	GPX4_SPIOL	19035.7	5.92	5	28	0		

Peptide Information

Calc. Mass	Obsrv. Mass	± da	± ppm	Start Seq.	End Seq.	Sequence	Ion Score	C. I. %	Modification	Rank	Result Type
863.462	863.3549	-0.1071	-124	124	132	GGLFGDGLK					Mascot
864.4461	864.3329	-0.1132	-131	150	157	YAPTTSPK					Mascot
1021.4619	1021.374	-0.0879	-86	1	10	MASDSSAQPK					Mascot
1165.6211	1165.606	-0.0151	-13	121	132	SSKGGLFGDGLK					Mascot
1465.7281	1465.5332	-0.1949	-133	19	31	DARGNDVDLSIYK					Mascot
1465.7281	1465.5332	-0.1949	-133	19	31	DARGNDVDLSIYK					Mascot

Rank	Protein Name	Accession No	Protein MW	Protein PI	Pep. Count	Protein Score	Protein Score C. I. %	Total Ion Score	Total Ion C. I. %
5	GATA transcription factor 18 OS=Arabidopsis tha GN=GATA18 PE=2 SV=2	GAT18_ARAT	31811.4	8.22	6	26	0		

Peptide Information

Calc. Mass	Obsrv. Mass	± da	± ppm	Start Seq.	End Seq.	Sequence	Ion Score	C. I. %	Modification	Rank	Result Type
1021.4618	1021.374	-0.0878	-86	80	87	LCEEDEKR					Mascot
1045.5425	1045.4403	-0.1022	-98	287	295	ASLVHDFTR					Mascot
1275.615	1275.5452	-0.0698	-55	242	252	VPCNYPANEIR					Mascot
1275.6296	1275.5452	-0.0844	-66	171	182	GPKSLCNACGIR			Carbamidomethyl (C)[6]		Mascot
1662.7247	1662.5811	-0.1436	-86	132	152	GCSGGGGGGGGGGGG DSLLAR			Carbamidomethyl (C)[2]		Mascot
2297.0979	2296.8704	-0.2275	-99	89	109	RSTSSGASSCISNFWDLI HTK					Mascot

Rank	Protein Name	Accession No	Protein MW	Protein PI	Pep. Count	Protein Score	Protein Score C. I. %	Total Ion Score	Total Ion C. I. %
6	Em protein OS=Triticum aestivum GN=EM PE=2 S	EM1_WHEAT	9956.8	5.55	4	24	0		

Peptide Information

Calc. Mass	Obsrv. Mass	± da	± ppm	Start Seq.	End Seq.	Sequence	Ion Score	C. I. %	Modification	Rank	Result Type
1005.4734	1005.4672	-0.0062	-6	80	88	EGIDIDESK					Mascot
1187.5902	1187.4879	-0.1023	-86	17	29	EGETVVPGGTGGK					Mascot
1517.5995	1517.4476	-0.1519	-100	50	62	EOMGEEGYSOMGR			Oxidation (M)[3]		Mascot
1559.7772	1559.5607	-0.2165	-139	30	43	SLEAQENLAEGRSR					Mascot

Rank	Protein Name	Accession No	Protein MW	Protein PI	Pep. Count	Protein Score	Protein Score C. I. %	Total Ion Score	Total Ion C. I. %
7	Embryonic abundant protein 1 OS=Oryza sativa su japonica GN=EMP1 PE=2 SV=1	EMP1_ORYSJ	10158.9	5.57	4	24	0		

Peptide Information

Calc. Mass	Obsrv. Mass	± da	± ppm	Start Seq.	End Seq.	Sequence	Ion Score	C. I. %	Modification	Rank	Result Type
1005.4734	1005.4672	-0.0062	-6	82	90	EGIDIDESK					Mascot
1349.6292	1349.4504	-0.1788	-132	65	78	KGGLSTGDESGGER					Mascot
1559.7521	1559.5607	-0.1914	-123	2	15	ASGQQQQGRSELDR					Mascot
1587.6526	1587.627	-0.0256	-16	52	64	EQMGEEGYREMGR			Oxidation (M)[3]		Mascot
1587.6526	1587.627	-0.0256	-16	52	64	EQMGEEGYREMGR			Oxidation (M)[3]		Mascot

Rank	Protein Name	Accession No	Protein MW	Protein PI	Pep. Count	Protein Score	Protein Score C. I. %	Total Ion Score	Total Ion C. I. %
8	Putative E3 ubiquitin-protein ligase SINAT1 OS=Arabidopsis thaliana GN=SINAT1 PE=3 SV=1	SINA1_ARAT	34534.7	7.85	6	23	0		

Peptide Information

Calc. Mass	Obsrv. Mass	± da	± ppm	Start Seq.	End Seq.	Sequence	Ion Score	C. I. %	Modification	Rank	Result Type
864.4573	864.3329	-0.1244	-144	94	100	YELGNIR					Mascot
870.454	870.4096	-0.0444	-51	260	266	SIRDSHR					Mascot
870.454	870.4096	-0.0444	-51	260	266	SIRDSHR					Mascot
	870.4096										Mascot
1021.4553	1021.374	-0.0813	-80	85	93	VQNTCPTCR					Mascot
1640.7649	1640.627	-0.1379	-84	10	24	EALESNSTGVDYEVK					Mascot
1821.8159	1821.6693	-0.1466	-80	171	185	VDMHDGCTFNHRYVK					Mascot
1866.8949	1866.7742	-0.1207	-65	85	100	VQNTCPTCRYELGNIR					Mascot

Rank	Protein Name	Accession No	Protein MW	Protein PI	Pep. Count	Protein Score	Protein Score C. I. %	Total Ion Score	Total Ion C. I. %
9	Putative cytochrome c oxidase subunit II PS17 (Fragments) OS=Pinus strobus PE=1 SV=1	PS17_PINST	1707	9.63	2	22	0		

Peptide Information

Calc. Mass	Obsrv. Mass	± da	± ppm	Start Seq.	End Seq.	Sequence	Ion Score	C. I. %	Modification	Rank	Result Type
856.525	856.3995	-0.1255	-147	1	8	SPTVIALR					Mascot
856.525	856.3995	-0.1255	-147	1	8	SPTVIALR					Mascot
870.5043	870.4096	-0.0947	-109	9	16	VVEALSPR					Mascot
870.5043	870.4096	-0.0947	-109	9	16	VVEALSPR					Mascot

Rank	Protein Name	Accession No	Protein MW	Protein PI	Pep. Count	Protein Score	Protein Score C. I. %	Total Ion Score	Total Ion C. I. %
10	Stigma/stylar cysteine-rich adhesin OS=Lilium longiflorum GN=SCA PE=1 SV=1	SCA_LILLO	11678	9.13	4	22	0		

Peptide Information

Calc. Mass	Obsrv. Mass	± da	± ppm	Start Seq.	End Seq.	Sequence	Ion Score	C. I. %	Modification	Rank	Result Type
937.423	937.3203	-0.1027	-110	67	74	QTACNCLK			Carbamidomethyl (C)[4]		Mascot
1094.5082	1094.4124	-0.0958	-88	105	113	MQTDCNKVR					Mascot
1094.5082	1094.4124	-0.0958	-88	105	113	MQTDCNKVR					Mascot
1185.5867	1185.4775	-0.1092	-92	42	54	GGVIPPGCCAGVR					Mascot
1313.6816	1313.496	-0.1856	-141	41	54	KGGVIPPGCCAGVR					Mascot

Gel Idx/Pos	29/B2	Instr./Gel Origin	BA2060/131017A	Process Sta	Analysis Succeeded
Plate [#] Name	[1] 31496	Instrument Sample Name		Spectra	11

Rank	Protein Name	Accession No	Protein MW	Protein PI	Pep. Count	Protein Score	Protein Score C. I. %	Total Ion Score	Total Ion C. I. %
1	lipoxygenase [Glycine max]	gi|351722222	96240.7	6.29	21	246	100	123	100

Peptide Information

Calc. Mass	Obsrv. Mass	± da	± ppm	Start Seq.	End Seq.	Sequence	Ion Score	C. I. %	Modification	Rank	Result Type
856.3804	856.405	0.0246	29	1	8	MTGGMFGR					Mascot
956.4988	956.3636	-0.1352	-141	683	689	DKPWWPK					Mascot
1014.573	1014.4357	-0.1373	-135	387	394	RLQEFPPK					Mascot
1175.6896	1175.5212	-0.1684	-143	95	105	HLPTLPTLGAR	6	0			Mascot
1175.6896	1175.5212	-0.1684	-143	95	105	HLPTLPTLGAR					Mascot
1274.6124	1274.4368	-0.1756	-138	778	788	HASDEVYLGQR					Mascot
1274.6124	1274.4368	-0.1756	-138	778	788	HASDEVYLGQR	5	0			Mascot
1312.7041	1312.517	-0.1871	-143	375	386	EMIAGVNPNVIR					Mascot
1314.542	1314.3601	-0.1819	-138	364	374	SGWMTDDEFAR	7	0			Mascot
1314.542	1314.3601	-0.1819	-138	364	374	SGWMTDDEFAR					Mascot
1330.5369	1330.3544	-0.1825	-137	364	374	SGWMTDDEFAR			Oxidation (M)[4]		Mascot
1334.7063	1334.5189	-0.1874	-140	189	198	YRQEELEVLR					Mascot
1334.7063	1334.5189	-0.1874	-140	189	198	YRQEELEVLR					Mascot
1418.7386	1418.5353	-0.2033	-143	610	623	GLAVEDPSAPHGLR					Mascot
1517.6754	1517.4586	-0.2168	-143	304	316	VTSSEFDSFDEVR	52	85.657			Mascot
1517.6754	1517.4586	-0.2168	-143	304	316	VTSSEFDSFDEVR					Mascot
1530.7758	1530.6329	-0.1429	-93	191	204	QEELEVLRGDGTGK					Mascot
1576.7716	1576.5399	-0.2317	-147	129	141	NFMTDEFFLVSVK					Mascot
1592.7665	1592.5316	-0.2349	-147	129	141	NFMTDEFFLVSVK			Oxidation (M)[3]		Mascot
1600.7965	1600.5631	-0.2334	-146	254	267	DPNSEKPGEIYVPR					Mascot
1716.8955	1716.6516	-0.2439	-142	594	608	NWVFTDQALPADLVK					Mascot
1728.8915	1728.6425	-0.249	-144	253	267	KDPNSEKPGEIYVPR					Mascot
1733.0571	1732.8051	-0.252	-145	325	340	LPTNILSQISPLPVLK					Mascot
1747.984	1747.7255	-0.2585	-148	763	777	FETLIDISVIEILSR	12	0			Mascot
1747.984	1747.7255	-0.2585	-148	763	777	FETLIDISVIEILSR					Mascot
1798.8528	1798.5892	-0.2636	-147	843	859	SSEEGMSFKGIPNSISI			Oxidation (M)[6]		Mascot
1820.929	1820.6606	-0.2684	-147	345	360	TDGENTLQFPPPHVIR	46	39.098			Mascot
1820.929	1820.6606	-0.2684	-147	345	360	TDGENTLQFPPPHVIR					Mascot
2164.1357	2163.8157	-0.32	-148	412	431	QQLEINLGGVTVEEAISAHR					Mascot
2164.1357	2163.8157	-0.32	-148	412	431	QQLEINLGGVTVEEAISAHR	14	0			Mascot
2322.1904	2321.8796	-0.3108	-134	624	643	LVIEDYPYAVDGLEIWDAIK					Mascot

Rank	Protein Name	Accession No	Protein MW	Protein PI	Pep. Count	Protein Score	Protein Score C. I. %	Total Ion Score	Total Ion C. I. %
2	Chain B, Refined 1.8 Angstroms Resolution Cryst Structure Of Porcine Epsilon-Trypsin	gi|999627	8813.5	6.67	4	71	28.376	40	0

Peptide Information

Calc. Mass	Obsrv. Mass	± da	± ppm	Start Seq.	End Seq.	Sequence	Ion Score	C. I. %	Modification	Rank	Result Type
842.5094	842.3881	-0.1213	-144	57	64	VATVSLPR					Mascot
1045.5636	1045.4087	-0.1549	-148	47	56	LSSPATLNSR					Mascot
2211.104	2210.7822	-0.3218	-146	7	26	LGEHNIDVLEGNEQFINAAK					Mascot
2211.104	2210.7822	-0.3218	-146	7	26	LGEHNIDVLEGNEQFINAAK	40	0			Mascot
2299.175	2298.8611	-0.3139	-137	27	46	IITHPNFNGNTLDNDIMLIK			Oxidation (M)[17]		Mascot

Rank	Protein Name	Accession No	Protein MW	Protein PI	Pep. Count	Protein Score	Protein Score C. I. %	Total Ion Score	Total Ion C. I. %
3	Chain A, Crystal Structure Of The First Active Autolysate Form Of The Porcine Alpha Trypsin	gi|1942351	13283.6	7.83	4	65	0	40	0

Peptide Information

Calc. Mass	Obsrv. Mass	± da	± ppm	Start Seq.	End Seq.	Sequence	Ion Score	C. I. %	Modification	Rank	Result Type
842.5094	842.3881	-0.1213	-144	100	107	VATVSLPR					Mascot
1045.5636	1045.4087	-0.1549	-148	90	99	LSSPATLNSR					Mascot
2211.104	2210.7822	-0.3218	-146	50	69	LGEHNIDVLEGNEQFINAAK					Mascot
2211.104	2210.7822	-0.3218	-146	50	69	LGEHNIDVLEGNEQFINAAK	40	0			Mascot
2299.175	2298.8611	-0.3139	-137	70	89	IITHPNFNGNTLDNDIMLIK			Oxidation (M)[17]		Mascot

Rank	Protein Name	Accession No	Protein MW	Protein PI	Pep. Count	Protein Score	Protein Score C. I. %	Total Ion Score	Total Ion C. I. %
4	Chain A, The Refined 1.6 Angstroms Resolution Structure Of The Complex Formed Between Porci	gi|494360	23458.5	8.6	5	64	0	40	0

Peptide Information

Calc. Mass	Obsrv. Mass	± da	± ppm	Start Seq.	End Seq.	Sequence	Ion Score	C. I. %	Modification	Rank	Result Type
842.5094	842.3881	-0.1213	-144	100	107	VATVSLPR					Mascot
1045.5636	1045.4087	-0.1549	-148	90	99	LSSPATLNSR					Mascot
2211.104	2210.7822	-0.3218	-146	50	69	LGEHNIDVLEGNEQFINAAK					Mascot
2211.104	2210.7822	-0.3218	-146	50	69	LGEHNIDVLEGNEQFINAAK	40	0			Mascot
2230.063	2229.8757	-0.1873	-84	150	170	SSYPGQITGNMICVGFLQGGK			Carbamidomethyl (C)[13], Oxidation (M)[11]		Mascot
2299.175	2298.8611	-0.3139	-137	70	89	IITHPNFNGNTLDNDIMLIK			Oxidation (M)[17]		Mascot

Rank	Protein Name	Accession No	Protein MW	Protein PI	Pep. Count	Protein Score	Protein Score C. I. %	Total Ion Score	Total Ion C. I. %
5	hypothetical protein BRAFLDRAFT_86434 [Branchiostoma floridae]	gi|260814494	59341.1	9.11	18	58	0		

Peptide Information

Calc. Mass	Obsrv. Mass	± da	± ppm	Start Seq.	End Seq.	Sequence	Ion Score	C. I. %	Modification	Rank	Result Type
842.3824	842.3881	0.0057	7	131	137	CDYRSAK					Mascot
856.4886	856.405	-0.0836	-98	307	314	ATLSSHIK					Mascot
870.396	870.4242	0.0282	32	292	297	KPYMCR			Carbamidomethyl (C)[5], Oxidation (M)[4]		Mascot
910.4563	910.3483	-0.108	-119	389	396	SHLASHMK					Mascot
1014.3767	1014.4357	0.059	58	72	78	CEQCDFR			Carbamidomethyl (C)[1,4]		Mascot
1045.4983	1045.4087	-0.0896	-86	223	231	ADLDTHMVK			Oxidation (M)[7]		Mascot
1245.6586	1245.5129	-0.1457	-117	202	211	KTHTENKPYK					Mascot
1418.6377	1418.5353	-0.1024	-72	292	302	KPYMCRECGFK			Carbamidomethyl (C)[5]		Mascot
1531.6304	1531.4719	-0.1585	-103	16	27	CDQCDFSSAWKK			Carbamidomethyl (C)[1,4]		Mascot
1728.7931	1728.6425	-0.1506	-87	324	337	CDLCDFDIKSNLTK			Carbamidomethyl (C)[1,4]		Mascot
1732.6941	1732.8051	0.111	64	36	50	HTGENLYTCGECDYK					Mascot
1747.8076	1747.7255	-0.0821	-47	478	492	HMVQHTGVKPYMCGK			Oxidation (M)[2,12]		Mascot
1747.8076	1747.7255	-0.0821	-47	478	492	HMVQHTGVKPYMCGK			Oxidation (M)[2,12]		Mascot
	1747.7255										Mascot
2169.9692	2169.6523	-0.3169	-146	176	194	HRGDKPYICVECGYDTADK					Mascot
2211.0144	2210.7822	-0.2322	-105	259	277	VHSGEKPYKCPLCDFCTAR			Carbamidomethyl (C)[10]		Mascot
2211.0144	2210.7822	-0.2322	-105	259	277	VHSGEKPYKCPLCDFCTAR			Carbamidomethyl (C)[10]		Mascot
2297.0662	2296.8606	-0.2056	-90	453	471	IHTGELHHRCQLCDYSTAR			Carbamidomethyl (C)[10]		Mascot
2667.2112	2666.8474	-0.3638	-136	361	384	STLDTHMAKHTGEKPYVCGECGFR					Mascot
2692.2581	2691.9246	-0.3335	-124	418	440	DLDAHMAKHTGVKPYLCWQCGYR					Mascot
3337.4646	3337.2502	-0.2144	-64	139	166	SHLNQHMKTHTGEKPYACDLCDFCTAWK			Carbamidomethyl (C)[18], Oxidation (M)[7]		Mascot

Rank	Protein Name	Accession No	Protein MW	Protein PI	Pep. Count	Protein Score	Protein Score C. I. %	Total Ion Score	Total Ion C. I. %
6	Chain A, Trypsin In Complex With Borate	gi|110590762	23458.3	8.22	4	57	0	40	0

Peptide Information

Calc. Mass	Obsrv. Mass	± da	± ppm	Start Seq.	End Seq.	Sequence	Ion Score	C. I. %	Modification	Rank	Result Type
842.5094	842.3881	-0.1213	-144	100	107	VATVSLPR					Mascot
1045.5636	1045.4087	-0.1549	-148	90	99	LSSPATLNSR					Mascot
2211.104	2210.7822	-0.3218	-146	50	69	LGEHNIDVLEGNEQFINAAK					Mascot
2211.104	2210.7822	-0.3218	-146	50	69	LGEHNIDVLEGNEQFINAAK	40	0			Mascot
2299.175	2298.8611	-0.3139	-137	70	89	IITHPNFNGNTLDNDIMLIK			Oxidation (M)[17]		Mascot

Rank	Protein Name	Accession No	Protein MW	Protein PI	Pep. Count	Protein Score	Protein Score C. I. %	Total Ion Score	Total Ion C. I. %
7	Chain E, Complex Of Eeti-Ii With Porcine Trypsin	gi|157878102	24156.7	8.26	4	56	0	40	0

Peptide Information

Calc. Mass	Obsrv. Mass	± da	± ppm	Start Seq.	End Seq.	Sequence	Ion Score	C. I. %	Modification	Rank	Result Type
842.5094	842.3881	-0.1213	-144	103	110	VATVSLPR					Mascot
1045.5636	1045.4087	-0.1549	-148	93	102	LSSPATLNSR					Mascot
2211.104	2210.7822	-0.3218	-146	53	72	LGEHNIDVLEGNEQFINAAK					Mascot
2211.104	2210.7822	-0.3218	-146	53	72	LGEHNIDVLEGNEQFINAAK	40	0			Mascot
2299.175	2298.8611	-0.3139	-137	73	92	IITHPNFNGNTLDNDIMLIK			Oxidation (M)[17]		Mascot

Rank	Protein Name	Accession No	Protein MW	Protein PI	Pep. Count	Protein Score	Protein Score C. I. %	Total Ion Score	Total Ion C. I. %
8	metallothionein [Tamarix androssowii]	gi|47933905	7090.8	4.72	7	52	0		

Peptide Information

Calc. Mass	Obsrv. Mass	± da	± ppm	Start Seq.	End Seq.	Sequence	Ion Score	C. I. %	Modification	Rank	Result Type
842.313	842.3881	0.0751	89	2	9	SSCGGSCK			Carbamidomethyl (C)[3,7]		Mascot
1334.3574	1334.5189	0.1615	121	61	72	CGDGCSCDPCTC			Carbamidomethyl (C)[1,5,7]		Mascot
1334.3574	1334.5189	0.1615	121	61	72	CGDGCSCDPCTC			Carbamidomethyl (C)[1,5,7]		Mascot
	1334.5189										Mascot
1342.5249	1342.3821	-0.1428	-106	49	60	SSEMNVEGEGCK			Carbamidomethyl (C)[11], Oxidation (M)[4]		Mascot
1465.4381	1465.5469	0.1088	74	10	22	CSGCSCGDSCNCR			Carbamidomethyl (C)[1,4,6]		Mascot
1798.7946	1798.5892	-0.2054	-114	44	60	TVSGKSSEMNVEGEGCK			Carbamidomethyl (C)[16]		Mascot
2003.6261	2003.7007	0.0746	37	2	22	SSCGGSCKCSGCSCGDSCNCR					Mascot
2321.7258	2321.8796	0.1538	66	1	22	MSSCGGSCKCSGCSCGDSCNCR			Carbamidomethyl (C)[4,8,10], Oxidation (M)[1]		Mascot

Rank	Protein Name	Accession No	Protein MW	Protein PI	Pep. Count	Protein Score	Protein Score C. I. %	Total Ion Score	Total Ion C. I. %
9	Chain E, Leech-Derived Tryptase InhibitorTRYPS COMPLEX	gi|3318722	23457.4	8.26	3	51	0	40	0

Peptide Information

Calc. Mass	Obsrv. Mass	± da	± ppm	Start Seq.	End Seq.	Sequence	Ion Score	C. I. %	Modification	Rank	Result Type
842.5094	842.3881	-0.1213	-144	100	107	VATVSLPR					Mascot
2211.104	2210.7822	-0.3218	-146	50	69	LGEHNIDVLEGNEQFINAAK					Mascot
2211.104	2210.7822	-0.3218	-146	50	69	LGEHNIDVLEGNEQFINAAK	40	0			Mascot
2299.175	2298.8611	-0.3139	-137	70	89	IITHPNFNGNTLDNDIMLIK			Oxidation (M)[17]		Mascot

Rank	Protein Name	Accession No	Protein MW	Protein PI	Pep. Count	Protein Score	Protein Score C. I. %	Total Ion Score	Total Ion C. I. %
10	Chain A, Crystal Structure Of Human Pank1 Alpha Catalytic Core Domain In Complex With Accoa	gi|122920553	40105.9	5.66	11	50	0		

Peptide Information

Calc. Mass	Obsrv. Mass	± da	± ppm	Start Seq.	End Seq.	Sequence	Ion Score	C. I. %	Modification	Rank	Result Type
842.3679	842.3881	0.0202	24	113	118	FEEDFR					Mascot
856.4787	856.405	-0.0737	-86	72	78	GNLHFIR					Mascot
1175.6266	1175.5212	-0.1054	-90	233	243	GDSTNVDKLVK					Mascot
1175.6266	1175.5212	-0.1054	-90	233	243	GDSTNVDKLVK					Mascot
1245.6473	1245.5129	-0.1344	-108	42	52	KYLTSNTAYGK					Mascot
1886.0026	1885.7526	-0.25	-133	309	324	VVFVGNFLRINMVSMK			Oxidation (M)[12,15]		Mascot
1891.9371	1891.6857	-0.2514	-133	113	127	FEEDFRMIADLQLHK					Mascot
1925.882	1925.7927	-0.0893	-46	79	94	FPSCAMHRFIQMGSEK			Carbamidomethyl (C)[4]		Mascot
2164.064	2163.8157	-0.2483	-115	318	335	INMVSMKLLAYAMDFWSK			Oxidation (M)[3]		Mascot
2164.064	2163.8157	-0.2483	-115	318	335	INMVSMKLLAYAMDFWSK			Oxidation (M)[3]		Mascot
2283.1372	2282.8228	-0.3144	-138	299	317	MCALNENIDRVVFVGNFLR			Carbamidomethyl (C)[2], Oxidation (M)[1]		Mascot
2692.2458	2691.9246	-0.3212	-119	95	118	NFSSLHTTLCATGGGAFKFEEDFR			Carbamidomethyl (C)[10]		Mascot
3338.5662	3338.2307	-0.3355	-100	200	232	VTGTSLGGGTFLGLCCLLTGCETFEEALEMAAK			Oxidation (M)[30]		Mascot

Gel Idx/Pos	30/B3	Instr./Gel Origin	BA2060/131017A	Process Sta	Analysis Succeeded
Plate [#] Name	[1] 31496	Instrument Sample Name		Spectra	11

Rank	Protein Name	Accession No	Protein MW	Protein PI	Pep. Count	Protein Score	Protein Score C. I. %	Total Ion Score	Total Ion C. I. %
1	Ribulose bisphosphate carboxylase large chain OS=Liquidambar styraciflua GN=rbcL PE=3 SV=1	RBL_LIQST	52625.5	6.04	15	86	99.991	10	0

Peptide Information

Calc. Mass	Obsrv. Mass	± da	± ppm	Start Seq.	End Seq.	Sequence	Ion Score	C. I. %	Modification	Rank	Result Type
830.4366	830.3157	-0.1209	-146	440	446	EGNEIIR					Mascot
910.4451	910.3124	-0.1327	-146	188	194	AVYECLR			Carbamidomethyl (C)[5]		Mascot
914.4049	914.2761	-0.1288	-141	306	312	NHGMHFR			Oxidation (M)[4]		Mascot
921.4346	921.3711	-0.0635	-69	1	8	MSPQTETK					Mascot
928.4669	928.3415	-0.1254	-135	296	303	AMHAVIDR			Oxidation (M)[2]		Mascot
1021.5312	1021.3972	-0.134	-131	33	41	DTDILAAFR					Mascot
1170.5585	1170.4802	-0.0783	-67	304	312	QKNHGMHFR			Oxidation (M)[6]		Mascot
1187.6643	1187.5074	-0.1569	-132	286	295	DNGLLLHIHR					Mascot
1261.6285	1261.4791	-0.1494	-118	218	227	FLFCAEAIYK			Carbamidomethyl (C)[4]		Mascot
1275.7307	1275.5529	-0.1778	-139	340	350	EITLGFVDLLR					Mascot
1451.6219	1451.4252	-0.1967	-136	202	213	DDENVNSQPFMR					Mascot
1465.7216	1465.5526	-0.169	-115	320	334	MSGGDHIHAGTVVGK					Mascot
1465.7546	1465.5526	-0.202	-138	147	159	TFQGPPHGIQVER	11	0			Mascot
1488.6926	1488.5504	-0.1422	-96	464	475	EIKFEFEAMDTL			Oxidation (M)[9]		Mascot
1502.8512	1502.6278	-0.2234	-149	165	177	YGRPLLGCTIKPK			Carbamidomethyl (C)[8]		Mascot

Rank	Protein Name	Accession No	Protein MW	Protein PI	Pep. Count	Protein Score	Protein Score C. I. %	Total Ion Score	Total Ion C. I. %
2	Ribulose bisphosphate carboxylase large chain OS=Sesbania sesban GN=rbcL PE=3 SV=1	RBL_SESSE	52358.4	6.09	14	83	99.982	11	0

Peptide Information

Calc. Mass	Obsrv. Mass	± da	± ppm	Start Seq.	End Seq.	Sequence	Ion Score	C. I. %	Modification	Rank	Result Type
830.4366	830.3157	-0.1209	-146	440	446	EGNEIIR					Mascot
856.3546	856.4133	0.0587	69	467	473	FEFPAMD					Mascot
856.3546	856.4133	0.0587	69	467	473	FEFPAMD					Mascot
910.4451	910.3124	-0.1327	-146	188	194	AVYECLR			Carbamidomethyl (C)[5]		Mascot
914.4049	914.2761	-0.1288	-141	306	312	NHGMHFR			Oxidation (M)[4]		Mascot
921.4346	921.3711	-0.0635	-69	1	8	MSPQTETK					Mascot
928.4669	928.3415	-0.1254	-135	296	303	AMHAVIDR			Oxidation (M)[2]		Mascot
1021.5312	1021.3972	-0.134	-131	33	41	DTDILAAFR					Mascot
1039.4691	1039.3811	-0.088	-85	351	358	DDYVEKDR					Mascot
1170.5585	1170.4802	-0.0783	-67	304	312	QKNHGMHFR			Oxidation (M)[6]		Mascot
1187.6643	1187.5074	-0.1569	-132	286	295	DNGLLLHIHR					Mascot
1275.7307	1275.5529	-0.1778	-139	340	350	EITLGFVDLLR					Mascot
1451.6219	1451.4252	-0.1967	-136	202	213	DDENVNSQPFMR					Mascot
1465.7546	1465.5526	-0.202	-138	147	159	TFQGPPHGIQVER					Mascot
1465.7546	1465.5526	-0.202	-138	147	159	TFQGPPHGIQVER	11	0			Mascot
1502.8512	1502.6278	-0.2234	-149	165	177	YGRPLLGCTIKPK			Carbamidomethyl (C)[8]		Mascot

Rank	Protein Name	Accession No	Protein MW	Protein PI	Pep. Count	Protein Score	Protein Score C. I. %	Total Ion Score	Total Ion C. I. %
3	Ribulose bisphosphate carboxylase large chain (Fragment) OS=Sinapis alba GN=rbcL PE=3 SV=1	RBL_SINAL	52405.4	6.23	14	81	99.973	11	0

Peptide Information

Calc. Mass	Obsrv. Mass	± da	± ppm	Start Seq.	End Seq.	Sequence	Ion Score	C. I. %	Modification	Rank	Result Type
830.4366	830.3157	-0.1209	-146	440	446	EGNEIIR					Mascot
856.3546	856.4133	0.0587	69	467	473	FEFPAMD					Mascot
856.3546	856.4133	0.0587	69	467	473	FEFPAMD					Mascot
910.4451	910.3124	-0.1327	-146	188	194	AVYECLR			Carbamidomethyl (C)[5]		Mascot
914.4049	914.2761	-0.1288	-141	306	312	NHGMHFR			Oxidation (M)[4]		Mascot
921.4346	921.3711	-0.0635	-69	1	8	MSPQTETK					Mascot
928.4669	928.3415	-0.1254	-135	296	303	AMHAVIDR			Oxidation (M)[2]		Mascot
1021.5312	1021.3972	-0.134	-131	33	41	DTDILAAFR					Mascot
1170.5585	1170.4802	-0.0783	-67	304	312	QKNHGMHFR			Oxidation (M)[6]		Mascot
1187.6643	1187.5074	-0.1569	-132	286	295	DNGLLLHIHR					Mascot
1261.6285	1261.4791	-0.1494	-118	218	227	FLFCAEAIYK			Carbamidomethyl (C)[4]		Mascot
1275.7307	1275.5529	-0.1778	-139	340	350	EITLGFVDLLR					Mascot
1451.6219	1451.4252	-0.1967	-136	202	213	DDENVNSQPFMR					Mascot
1465.7546	1465.5526	-0.202	-138	147	159	TFQGPPHGIQVER					Mascot
1465.7546	1465.5526	-0.202	-138	147	159	TFQGPPHGIQVER	11	0			Mascot
1502.8512	1502.6278	-0.2234	-149	165	177	YGRPLLGCTIKPK			Carbamidomethyl (C)[8]		Mascot

Rank	Protein Name	Accession No	Protein MW	Protein PI	Pep. Count	Protein Score	Protein Score C. I. %	Total Ion Score	Total Ion C. I. %
4	Ribulose bisphosphate carboxylase large chain (Fragment) OS=Hamamelis mollis GN=rbcL PE=3 SV=1	RBL_HAMMO	51542.9	6.05	14	80	99.969	10	0

Peptide Information

Calc. Mass	Obsrv. Mass	± da	± ppm	Start Seq.	End Seq.	Sequence	Ion Score	C. I. %	Modification	Rank	Result Type
830.4366	830.3157	-0.1209	-146	430	436	EGNEIIR					Mascot
910.4451	910.3124	-0.1327	-146	178	184	AVYECLR			Carbamidomethyl (C)[5]		Mascot
914.4049	914.2761	-0.1288	-141	296	302	NHGMHFR			Oxidation (M)[4]		Mascot
928.4669	928.3415	-0.1254	-135	286	293	AMHAVIDR			Oxidation (M)[2]		Mascot
1021.5312	1021.3972	-0.134	-131	23	31	DTDILAAFR					Mascot
1170.5585	1170.4802	-0.0783	-67	294	302	QKNHGMHFR			Oxidation (M)[6]		Mascot
1187.6643	1187.5074	-0.1569	-132	276	285	DNGLLLHIHR					Mascot
1261.6285	1261.4791	-0.1494	-118	208	217	FLFCAEAIYK			Carbamidomethyl (C)[4]		Mascot
1275.7307	1275.5529	-0.1778	-139	330	340	EITLGFVDLLR					Mascot
1451.6219	1451.4252	-0.1967	-136	192	203	DDENVNSQPFMR					Mascot
1465.7216	1465.5526	-0.169	-115	310	324	MSGGDHIHAGTVVGK					Mascot
1465.7546	1465.5526	-0.202	-138	137	149	TFQGPPHGIQVER	11	0			Mascot
1488.6926	1488.5504	-0.1422	-96	454	465	EIKFEFEAMDTL			Oxidation (M)[9]		Mascot
1502.8512	1502.6278	-0.2234	-149	155	167	YGRPLLGCTIKPK			Carbamidomethyl (C)[8]		Mascot

Rank	Protein Name	Accession No	Protein MW	Protein PI	Pep. Count	Protein Score	Protein Score C. I. %	Total Ion Score	Total Ion C. I. %
5	Ribulose bisphosphate carboxylase large chain OS=Panax ginseng GN=rbcL PE=3 SV=1	RBL_PANGI	52883.7	6	14	80	99.965	11	0

Peptide Information

Calc. Mass	Obsrv. Mass	± da	± ppm	Start Seq.	End Seq.	Sequence	Ion Score	C. I. %	Modification	Rank	Result Type
830.4366	830.3157	-0.1209	-146	440	446	EGNEIIR					Mascot
910.4451	910.3124	-0.1327	-146	188	194	AVYECLR			Carbamidomethyl (C)[5]		Mascot
914.4049	914.2761	-0.1288	-141	306	312	NHGMHFR			Oxidation (M)[4]		Mascot
921.4346	921.3711	-0.0635	-69	1	8	MSPQTETK					Mascot
928.4669	928.3415	-0.1254	-135	296	303	AMHAVIDR			Oxidation (M)[2]		Mascot
1021.5312	1021.3972	-0.134	-131	33	41	DTDILAAFR					Mascot
1170.5585	1170.4802	-0.0783	-67	304	312	QKNHGMHFR			Oxidation (M)[6]		Mascot
1187.6643	1187.5074	-0.1569	-132	286	295	DNGLLLHIHR					Mascot
1261.6285	1261.4791	-0.1494	-118	218	227	FLFCAEALYK			Carbamidomethyl (C)[4]		Mascot
1375.6416	1375.4806	-0.161	-117	22	32	LTYYTPDYDPK					Mascot
1451.6219	1451.4252	-0.1967	-136	202	213	DDENVNSQPFMR					Mascot
1465.7546	1465.5526	-0.202	-138	147	159	TFQGPPHGIQVER					Mascot
1465.7546	1465.5526	-0.202	-138	147	159	TFQGPPHGIQVER	11	0			Mascot
1502.8512	1502.6278	-0.2234	-149	165	177	YGRPLLGCTIKPK			Carbamidomethyl (C)[8]		Mascot
1781.8269	1781.5623	-0.2646	-148	19	32	DYKLTYYTPDYDPK					Mascot

Rank	Protein Name	Accession No	Protein MW	Protein PI	Pep. Count	Protein Score	Protein Score C. I. %	Total Ion Score	Total Ion C. I. %
6	Ribulose bisphosphate carboxylase large chain OS=Ipomoea purpurea GN=rbcL PE=3 SV=1	RBL_IPOPU	53353.9	6.41	14	80	99.962	11	0

Peptide Information

Calc. Mass	Obsrv. Mass	± da	± ppm	Start Seq.	End Seq.	Sequence	Ion Score	C. I. %	Modification	Rank	Result Type
830.4366	830.3157	-0.1209	-146	440	446	EGNEIIR					Mascot
910.4451	910.3124	-0.1327	-146	188	194	AVYECLR			Carbamidomethyl (C)[5]		Mascot
914.4049	914.2761	-0.1288	-141	306	312	NHGMHFR			Oxidation (M)[4]		Mascot
921.4346	921.3711	-0.0635	-69	1	8	MSPQTETK					Mascot
928.4669	928.3415	-0.1254	-135	296	303	AMHAVIDR			Oxidation (M)[2]		Mascot
1021.5312	1021.3972	-0.134	-131	33	41	DTDILAAFR					Mascot
1170.5585	1170.4802	-0.0783	-67	304	312	QKNHGMHFR			Oxidation (M)[6]		Mascot
1187.6643	1187.5074	-0.1569	-132	286	295	DNGLLLHIHR					Mascot
1261.6285	1261.4791	-0.1494	-118	218	227	FLFCAEAIYK			Carbamidomethyl (C)[4]		Mascot
1275.7307	1275.5529	-0.1778	-139	340	350	EITLGFVDLLR					Mascot
1317.658	1317.4707	-0.1873	-142	440	450	EGNEIIRQACK			Carbamidomethyl (C)[10]		Mascot
1317.658	1317.4707	-0.1873	-142	440	450	EGNEIIRQACK			Carbamidomethyl (C)[10]		Mascot
1451.6219	1451.4252	-0.1967	-136	202	213	DDENVNSQPFMR					Mascot
1465.7546	1465.5526	-0.202	-138	147	159	TFQGPPHGIQVER					Mascot
1465.7546	1465.5526	-0.202	-138	147	159	TFQGPPHGIQVER	11	0			Mascot
1502.8512	1502.6278	-0.2234	-149	165	177	YGRPLLGCTIKPK			Carbamidomethyl (C)[8]		Mascot

Rank	Protein Name	Accession No	Protein MW	Protein PI	Pep. Count	Protein Score	Protein Score C. I. %	Total Ion Score	Total Ion C. I. %
7	Ribulose bisphosphate carboxylase large chain OS=Platanus occidentalis GN=rbcL PE=3 SV=2	RBL_PLAOC	52652.5	6.04	14	79	99.961	10	0

Peptide Information

Calc. Mass	Obsrv. Mass	± da	± ppm	Start Seq.	End Seq.	Sequence	Ion Score	C. I. %	Modification	Rank	Result Type
830.4366	830.3157	-0.1209	-146	440	446	EGNEIIR					Mascot
880.4536	880.3522	-0.1014	-115	306	312	NHGIHFR					Mascot
910.4451	910.3124	-0.1327	-146	188	194	AVYECLR			Carbamidomethyl (C)[5]		Mascot
921.4346	921.3711	-0.0635	-69	1	8	MSPQTETK					Mascot
928.4669	928.3415	-0.1254	-135	296	303	AMHAVIDR			Oxidation (M)[2]		Mascot
1021.5312	1021.3972	-0.134	-131	33	41	DTDILAAFR					Mascot
1187.6643	1187.5074	-0.1569	-132	286	295	DNGLLLHIHR					Mascot
1261.6205	1261.4791	-0.1414	-112	440	450	EGNEIIREACK					Mascot
1275.7307	1275.5529	-0.1778	-139	340	350	EITLGFVDLLR					Mascot
1451.6219	1451.4252	-0.1967	-136	202	213	DDENVNSQPFMR					Mascot
1465.7216	1465.5526	-0.169	-115	320	334	MSGGDHIHAGTVVGK					Mascot
	1465.5526										Mascot
1465.7546	1465.5526	-0.202	-138	147	159	TFQGPPHGIQVER	11	0			Mascot
1488.6926	1488.5504	-0.1422	-96	464	475	EIKFEFEAMDTL			Oxidation (M)[9]		Mascot
1502.8512	1502.6278	-0.2234	-149	165	177	YGRPLLGCTIKPK			Carbamidomethyl (C)[8]		Mascot

Rank	Protein Name	Accession No	Protein MW	Protein PI	Pep. Count	Protein Score	Protein Score C. I. %	Total Ion Score	Total Ion C. I. %
8	Ribulose bisphosphate carboxylase large chain (Fragment) OS=Saururus cernuus GN=rbcL PE=3 SV=1	RBL_SAUCE	51663	6	14	78	99.952	10	0

Peptide Information

Calc. Mass	Obsrv. Mass	± da	± ppm	Start Seq.	End Seq.	Sequence	Ion Score	C. I. %	Modification	Rank	Result Type	
830.4366	830.3157	-0.1209	-146	431	437	EGNEIIR					Mascot	
910.4451	910.3124	-0.1327	-146	179	185	AVYECLR			Carbamidomethyl (C)[5]		Mascot	Mascot
914.4049	914.2761	-0.1288	-141	297	303	NHGMHFR			Oxidation (M)[4]		Mascot	
928.4669	928.3415	-0.1254	-135	287	294	AMHAVIDR			Oxidation (M)[2]		Mascot	
1021.5312	1021.3972	-0.134	-131	24	32	DTDILAAFR					Mascot	
1170.5585	1170.4802	-0.0783	-67	295	303	QKNHGMHFR			Oxidation (M)[6]		Mascot	
1187.6643	1187.5074	-0.1569	-132	277	286	DNGLLLHIHR					Mascot	
1261.6285	1261.4791	-0.1494	-118	209	218	FLFCAEAIYK			Carbamidomethyl (C)[4]		Mascot	
1275.7307	1275.5529	-0.1778	-139	331	341	EITLGFVDLLR					Mascot	
1451.6219	1451.4252	-0.1967	-136	193	204	DDENVNSQPFMR					Mascot	
1465.7216	1465.5526	-0.169	-115	311	325	MSGGDHIHAGTVVGK					Mascot	
1465.7546	1465.5526	-0.202	-138	138	150	TFQGPPHGIQVER	11	0			Mascot	
1488.6926	1488.5504	-0.1422	-96	455	466	EIKFEFEAMDTL			Oxidation (M)[9]		Mascot	
1502.8512	1502.6278	-0.2234	-149	156	168	YGRPLLGCTIKPK			Carbamidomethyl (C)[8]		Mascot	

Rank	Protein Name	Accession No	Protein MW	Protein PI	Pep. Count	Protein Score	Protein Score C. I. %	Total Ion Score	Total Ion C. I. %
9	Ribulose bisphosphate carboxylase large chain OS=Cucumis sativus GN=rbcL PE=1 SV=5	RBL_CUCSA	52608.4	6	14	78	99.945	10	0

Peptide Information

Calc. Mass	Obsrv. Mass	± da	± ppm	Start Seq.	End Seq.	Sequence	Ion Score	C. I. %	Modification	Rank	Result Type
830.4366	830.3157	-0.1209	-146	440	446	EGNEIIR					Mascot
910.4451	910.3124	-0.1327	-146	188	194	AVYECLR			Carbamidomethyl (C)[5]		Mascot
914.4049	914.2761	-0.1288	-141	306	312	NHGMHFR			Oxidation (M)[4]		Mascot
921.4346	921.3711	-0.0635	-69	1	8	MSPQTETK					Mascot
928.4669	928.3415	-0.1254	-135	296	303	AMHAVIDR			Oxidation (M)[2]		Mascot
1021.5312	1021.3972	-0.134	-131	33	41	DTDILAAFR					Mascot
1170.5585	1170.4802	-0.0783	-67	304	312	QKNHGMHFR			Oxidation (M)[6]		Mascot
1187.6643	1187.5074	-0.1569	-132	286	295	DNGLLLHIHR					Mascot
1275.7307	1275.5529	-0.1778	-139	340	350	EITLGFVDLLR					Mascot
1451.6219	1451.4252	-0.1967	-136	202	213	DDENVNSQPFMR					Mascot
1465.7216	1465.5526	-0.169	-115	320	334	MSGGDHIHAGTVVGK					Mascot
1465.7546	1465.5526	-0.202	-138	147	159	TFQGPPHGIQVER	11	0			Mascot
1488.6926	1488.5504	-0.1422	-96	464	475	EIKFEFEAMDTL			Oxidation (M)[9]		Mascot
1502.8512	1502.6278	-0.2234	-149	165	177	YGRPLLGCTIKPK			Carbamidomethyl (C)[8]		Mascot

Rank	Protein Name	Accession No	Protein MW	Protein PI	Pep. Count	Protein Score	Protein Score C. I. %	Total Ion Score	Total Ion C. I. %
10	Ribulose bisphosphate carboxylase large chain (Fragment) OS=Glycyrrhiza echinata GN=rbcL PE=3 SV=1	RBL_GLYEC	48689.7	6.54	13	77	99.93	11	0

Peptide Information

Calc. Mass	Obsrv. Mass	± da	± ppm	Start Seq.	End Seq.	Sequence	Ion Score	C. I. %	Modification	Rank	Result Type
830.4366	830.3157	-0.1209	-146	431	437	EGNEIIR					Mascot
910.4451	910.3124	-0.1327	-146	179	185	AVYECLR			Carbamidomethyl (C)[5]		Mascot
914.4049	914.2761	-0.1288	-141	297	303	NHGMHFR			Oxidation (M)[4]		Mascot
928.4669	928.3415	-0.1254	-135	287	294	AMHAVIDR			Oxidation (M)[2]		Mascot
1021.5312	1021.3972	-0.134	-131	24	32	DTDILAAFR					Mascot
1170.5585	1170.4802	-0.0783	-67	295	303	QKNHGMHFR			Oxidation (M)[6]		Mascot
1187.6643	1187.5074	-0.1569	-132	277	286	DNGLLLHIHR					Mascot
1261.6285	1261.4791	-0.1494	-118	209	218	FLFCAEAIYK			Carbamidomethyl (C)[4]		Mascot
1275.7307	1275.5529	-0.1778	-139	331	341	EITLGFVDLLR					Mascot
1317.658	1317.4707	-0.1873	-142	431	441	EGNEIIRQACK			Carbamidomethyl (C)[10]		Mascot
1317.658	1317.4707	-0.1873	-142	431	441	EGNEIIRQACK			Carbamidomethyl (C)[10]		Mascot
1451.6219	1451.4252	-0.1967	-136	193	204	DDENVNSQPFMR					Mascot
1465.7546	1465.5526	-0.202	-138	138	150	TFQGPPHGIQVER					Mascot
1465.7546	1465.5526	-0.202	-138	138	150	TFQGPPHGIQVER	11	0			Mascot
1502.8512	1502.6278	-0.2234	-149	156	168	YGRPLLGCTIKPK			Carbamidomethyl (C)[8]		Mascot

Gel Idx/Pos	31/B4	Instr./Gel Origin	BA2060/131017A	Process Sta	Analysis Succeeded
Plate [#] Name	[1] 31496	Instrument Sample Name		Spectra	11

Rank	Protein Name	Accession No	Protein MW	Protein PI	Pep. Count	Protein Score	Protein Score C. I. %	Total Ion Score	Total Ion C. I. %
1	Transketolase, chloroplastic OS=Spinacia olerac PE=1 SV=1	TKTC SPIOL	80230.6	6.2	5	70	99.671	61	99.993

Peptide Information

Calc. Mass	Obsrv. Mass	± da	± ppm	Start Seq.	End Seq.	Sequence	Ion Score	C. I. %	Modification	Rank	Result Type
996.4686	996.4751	0.0065	7	128	134	NPYWFNR					Mascot
1005.5615	1005.4698	-0.0917	-91	90	98	FLAIDAVEK					Mascot
1484.8048	1484.5999	-0.2049	-138	283	294	FEALGWHVIWVK	41	99.381			Mascot
1484.8048	1484.5999	-0.2049	-138	283	294	FEALGWHVIWVK					Mascot
1589.7806	1589.568	-0.2126	-134	420	434	ALPTYTPETPGDATR					Mascot
1589.7806	1589.568	-0.2126	-134	420	434	ALPTYTPETPGDATR	20	21.383			Mascot
3854.7043	3854.2932	-0.4111	-107	488	520	EHGMGAICNGICLHSPGF VPYCATFFVFTDYMR			Carbamidomethyl (C)[8,12,22]		Mascot

Rank	Protein Name	Accession No	Protein MW	Protein PI	Pep. Count	Protein Score	Protein Score C. I. %	Total Ion Score	Total Ion C. I. %
2	Transketolase-2, chloroplastic OS=Arabidopsis tl GN=TKL-2 PE=1 SV=1	TKTC2 ARAT	79872.1	6.12	5	50	64.771	41	99.381

Peptide Information

Calc. Mass	Obsrv. Mass	± da	± ppm	Start Seq.	End Seq.	Sequence	Ion Score	C. I. %	Modification	Rank	Result Type
856.4562	856.4089	-0.0473	-55	391	397	FAAYEKK					Mascot
996.4686	996.4751	0.0065	7	128	134	NPYWFNR					Mascot
1005.5615	1005.4698	-0.0917	-91	90	98	FLAIDAVEK					Mascot
1484.8048	1484.5999	-0.2049	-138	283	294	FEALGWHVIWVK	41	99.381			Mascot
1484.8048	1484.5999	-0.2049	-138	283	294	FEALGWHVIWVK					Mascot
3077.3987	3077.0303	-0.3684	-120	101	127	SGHPGLPMGCAPMSHIL YDEVMKYNPK			Carbamidomethyl (C)[10], Oxidation (M)[8,13,22]		Mascot
3077.3987	3077.0303	-0.3684	-120	101	127	SGHPGLPMGCAPMSHIL YDEVMKYNPK			Carbamidomethyl (C)[10], Oxidation (M)[8,13,22]		Mascot

Rank	Protein Name	Accession No	Protein MW	Protein PI	Pep. Count	Protein Score	Protein Score C. I. %	Total Ion Score	Total Ion C. I. %
3	Transketolase-1, chloroplastic OS=Arabidopsis tl GN=TKL-1 PE=1 SV=1	TKTC1 ARAT	79918.2	5.94	4	47	37.354	41	99.381

Peptide Information

Calc. Mass	Obsrv. Mass	± da	± ppm	Start Seq.	End Seq.	Sequence	Ion Score	C. I. %	Modification	Rank	Result Type
856.4562	856.4089	-0.0473	-55	391	397	FAAYEKK					Mascot
996.4686	996.4751	0.0065	7	128	134	NPYWFNR					Mascot
1005.5615	1005.4698	-0.0917	-91	90	98	FLAIDAVEK					Mascot
1484.8048	1484.5999	-0.2049	-138	283	294	FEALGWHVIWVK	41	99.381			Mascot
1484.8048	1484.5999	-0.2049	-138	283	294	FEALGWHVIWVK					Mascot

Rank	Protein Name	Accession No	Protein MW	Protein PI	Pep. Count	Protein Score	Protein Score C. I. %	Total Ion Score	Total Ion C. I. %
4	Transketolase, chloroplastic (Fragment) OS=Craterostigma plantagineum GN=TKT3 PE=1 SV=1	TKTC CRAPI	56152.5	5.8	1	41	0	41	99.381

Peptide Information

Calc. Mass	Obsrv. Mass	± da	± ppm	Start Seq.	End Seq.	Sequence	Ion Score	C. I. %	Modification	Rank	Result Type
1484.8048	1484.5999	-0.2049	-138	61	72	FEALGWHVIWVK	41	99.381			Mascot
1484.8048	1484.5999	-0.2049	-138	61	72	FEALGWHVIWVK					Mascot

Rank	Protein Name	Accession No	Protein MW	Protein PI	Pep. Count	Protein Score	Protein Score C. I. %	Total Ion Score	Total Ion C. I. %
5	Pentatricopeptide repeat-containing protein At4g3 OS=Arabidopsis thaliana GN=PCMP-E45 PE=3 SV=1	PP355 ARAT	62393.1	8.52	11	39	0		

Peptide Information

Calc. Mass	Obsrv. Mass	± da	± ppm	Start Seq.	End Seq.	Sequence	Ion Score	C. I. %	Modification	Rank	Result Type
850.5508	850.4332	-0.1176	-138	225	232	GIHGLILK					Mascot
961.4335	961.3574	-0.0761	-79	394	400	YFEEMVK			Oxidation (M)[5]		Mascot
996.5294	996.4751	-0.0543	-54	16	23	CSSLRVFK			Carbamidomethyl (C)[1]		Mascot
1012.5607	1012.498	-0.0627	-62	469	477	AMPVKPDVR					Mascot
1052.45	1052.4036	-0.0464	-44	252	260	CEQLSDAMR					Mascot
1638.7437	1638.6395	-0.1042	-64	444	456	LEHYGCMIDLLCR			Carbamidomethyl (C)[6], Oxidation (M)[7]		Mascot
1638.7437	1638.6395	-0.1042	-64	444	456	LEHYGCMIDLLCR			Carbamidomethyl (C)[6], Oxidation (M)[7]		Mascot
2872.2705	2871.9834	-0.2871	-100	135	158	MGFYDDIYVQNSLVHFY GVCGESR			Carbamidomethyl (C)[20], Oxidation (M)[1]		Mascot
3316.6921	3317.1375	0.4454	134	67	95	SVLSSFSYNTLLSSYAVC DKPRVTIFAYK			Carbamidomethyl (C)[18]		Mascot
3345.4761	3345.165	-0.3111	-93	135	162	MGFYDDIYVQNSLVHFY GVCGESRNACK			Carbamidomethyl (C)[20,27], Oxidation (M)[1]		Mascot
3345.7	3346.1611	0.4611	138	498	526	EILDSFLDIEFEDSGVYVL LSNIFAANRR					Mascot
3732.7937	3732.3027	-0.491	-132	127	158	QIHGIVTKMGFYDDIYVQ NSLVHFYGVCGESR			Carbamidomethyl (C)[28]		Mascot

Rank	Protein Name	Accession No	Protein MW	Protein PI	Pep. Count	Protein Score	Protein Score C. I. %	Total Ion Score	Total Ion C. I. %
6	UPF0496 protein At5g66675 OS=Arabidopsis tha GN=At5g66675 PE=2 SV=1	U496H ARAT	46341.7	7.56	8	35	0		

Peptide Information

Calc. Mass	Obsrv. Mass	± da	± ppm	Start Seq.	End Seq.	Sequence	Ion Score	C. I. %	Modification	Rank	Result Type
882.4753	882.4543	-0.021	-24	306	313	IGAYVTMK					Mascot
960.4681	960.3466	-0.1215	-127	1	8	MFLGMFSK					Mascot
1005.5509	1005.4698	-0.0811	-81	377	385	CSKNITLAR					Mascot
1465.6702	1465.5507	-0.1195	-82	2	13	FLGMFSKTMEDK			Oxidation (M)[4,9]		Mascot
2054.8794	2054.7441	-0.1353	-66	32	50	TDMGSLYSADLTAYNSA CR			Oxidation (M)[3]		Mascot
3317.5525	3317.1375	-0.415	-125	21	50	TNNTPLPQQIRTDMGSLY SADLTAYNSACR			Oxidation (M)[14]		Mascot
3487.6799	3487.302	-0.3779	-108	190	219	ASGDPFDGDFFMLLESV YEQQVMLLEILHK			Oxidation (M)[12]		Mascot
3743.8335	3744.3506	0.5171	138	190	221	ASGDPFDGDFFMLLESV YEQQVMLLEILHKQK			Oxidation (M)[12]		Mascot

Rank	Protein Name	Accession No	Protein MW	Protein PI	Pep. Count	Protein Score	Protein Score C. I. %	Total Ion Score	Total Ion C. I. %
7	Ribulose bisphosphate carboxylase small chain (512 (Fragment) OS=Triticum aestivum PE=3 SV=1	RBS3 WHEA	13046.5	5.84	5	32	0		

Peptide Information

Calc. Mass	Obsrv. Mass	± da	± ppm	Start Seq.	End Seq.	Sequence	Ion Score	C. I. %	Modification	Rank	Result Type
1012.4734	1012.498	0.0246	24	77	84	EYPDAYVR					Mascot
1165.571	1165.5916	0.0206	18	23	31	WVPCLEFSK			Carbamidomethyl (C)[4]		Mascot
2211.0571	2210.781	-0.2761	-125	93	112	QVQCVSFIAFKPPGCEES GK			Carbamidomethyl (C)[4]		Mascot
2211.0571	2210.781	-0.2761	-125	93	112	QVQCVSFIAFKPPGCEES GK			Carbamidomethyl (C)[4]		Mascot
2225.073	2224.8062	-0.2668	-120	93	113	QVQCVSFIAFKPPGCEES GKA					Mascot
2239.062	2238.7808	-0.2812	-126	56	75	LPMFGCTDATQVINEVEE VK			Oxidation (M)[3]		Mascot

Rank	Protein Name	Accession No	Protein MW	Protein PI	Pep. Count	Protein Score	Protein Score C. I. %	Total Ion Score	Total Ion C. I. %
8	Ras-related protein RABA5b OS=Arabidopsis tha GN=RABA5B PE=2 SV=1	RAA5B ARAT	24305.1	4.74	6	31	0		

Peptide Information

Calc. Mass	Obsrv. Mass	± da	± ppm	Start Seq.	End Seq.	Sequence	Ion Score	C. I. %	Modification	Rank	Result Type
850.3723	850.4332	0.0609	72	1	7	MGKEDDR					Mascot
882.4203	882.4543	0.034	39	97	104	GDTFESVK					Mascot
1038.5215	1038.3868	-0.1347	-130	97	105	GDTFESVKR					Mascot
1051.5782	1051.5796	0.0014	1	180	188	KLLNSDAYK					Mascot
2501.1753	2500.8855	-0.2898	-116	142	164	ALAEEEGLFFMETSALDA TNVDK					Mascot
2501.1753	2500.8855	-0.2898	-116	142	164	ALAEEEGLFFMETSALDA TNVDK					Mascot
3345.5659	3345.165	-0.4009	-120	106	133	WLQELNTHCDTAVAQML VGNKCDLEDIR			Carbamidomethyl (C)[9,22], Oxidation (M)[16]		Mascot

Rank	Protein Name	Accession No	Protein MW	Protein PI	Pep. Count	Protein Score	Protein Score C. I. %	Total Ion Score	Total Ion C. I. %
9	Phospho-2-dehydro-3-deoxyheptonate aldolase 1, chloroplastic OS=Solanum tuberosum GN=SHKA PE=1 SV=2	AROF SOLTU	59630.3	8.94	9	30	0		

Peptide Information

Calc. Mass	Obsrv. Mass	± da	± ppm	Start Seq.	End Seq.	Sequence	Ion Score	C. I. %	Modification	Rank	Result Type
882.5406	882.4543	-0.0863	-98	366	374	GIANPLGIK					Mascot
1005.5298	1005.4698	-0.06	-60	193	201	MAGQFAKPR					Mascot
1021.5247	1021.4026	-0.1221	-120	193	201	MAGQFAKPR			Oxidation (M)[1]		Mascot
1051.5023	1051.5796	0.0773	74	406	414	MGAENMRVK			Oxidation (M)[1]		Mascot
1493.6979	1493.5299	-0.168	-112	218	231	GDNVNGDAFDVKSR					Mascot
1496.724	1496.5012	-0.2228	-149	159	170	EFNANNIRDTFR					Mascot
1638.8698	1638.6395	-0.2303	-141	69	86	SSPPAATATTAPAPAVTK					Mascot
1638.8698	1638.6395	-0.2303	-141	69	86	SSPPAATATTAPAPAVTK					Mascot
2149.9719	2149.793	-0.1789	-83	494	511	TVTFDDLSSRYHTHCDP R					Mascot
2501.1877	2500.8855	-0.3022	-121	145	166	AFLLQGGDCAESFKEFN ANNIR			Carbamidomethyl (C)[9]		Mascot
2501.1877	2500.8855	-0.3022	-121	145	166	AFLLQGGDCAESFKEFN ANNIR			Carbamidomethyl (C)[9]		Mascot
3076.7	3077.0303	0.3303	107	2	30	ALSSTSTTNSLLPNRSLV QNQPLLPSPLK					Mascot
3076.7	3077.0303	0.3303	107	2	30	ALSSTSTTNSLLPNRSLV QNQPLLPSPLK					Mascot

Rank	Protein Name	Accession No	Protein MW	Protein PI	Pep. Count	Protein Score	Protein Score C. I. %	Total Ion Score	Total Ion C. I. %
10	Probably inactive receptor-like protein kinase At2g46850 OS=Arabidopsis thaliana GN=At2g46850 PE=3	Y2685_ARAT	70121.3	6.44	9	29	0		

Peptide Information

Calc. Mass	Obsrv. Mass	± da	± ppm	Start Seq.	End Seq.	Sequence	Ion Score	C. I. %	Modification	Rank	Result Type
882.4138	882.4543	0.0405	46	580	587	FGMVDAAR			Oxidation (M)[3]		Mascot
1005.5839	1005.4698	-0.1141	-113	492	500	VTGFGLQRK					Mascot
1012.4767	1012.498	0.0213	21	268	275	ELYGDKCK			Carbamidomethyl (C)[7]		Mascot
1926.949	1926.6631	-0.2859	-148	210	225	LEWAIPRNSPEAICDR			Carbamidomethyl (C)[14]		Mascot
2297.0654	2296.8572	-0.2082	-91	43	64	CGNFSVSFPFQLSSSSS AAAFR					Mascot
2584.0352	2583.8342	-0.201	-78	154	176	EEDETSGGDIGCCYPLS DHSAWR			Carbamidomethyl (C)[12]		Mascot
3077.4746	3077.0303	-0.4443	-144	414	440	IIGFCMDIGYNPLVVYEYP VNGSLGDR			Carbamidomethyl (C)[5], Oxidation (M)[6]		Mascot
3077.4746	3077.0303	-0.4443	-144	414	440	IIGFCMDIGYNPLVVYEYP VNGSLGDR			Carbamidomethyl (C)[5], Oxidation (M)[6]		Mascot
3346.6597	3346.1611	-0.4986	-149	414	442	IIGFCMDIGYNPLVVYEYP VNGSLGDRLR			Carbamidomethyl (C)[5], Oxidation (M)[6]		Mascot
3487.8188	3487.302	-0.5168	-148	380	409	VIVHKVLCENQIEFMEISS QIDHLSAVLHR					Mascot

Gel Idx/Pos	32/B5	Instr./Gel Origin	BA2060/131017A	Process Sta	Analysis Succeeded
Plate [#] Name	[1] 31496	Instrument Sample Name		Spectra	8

Rank	Protein Name	Accession No	Protein MW	Protein PI	Pep. Count	Protein Score	Protein Score C. I. %	Total Ion Score	Total Ion C. I. %
1	Transketolase, chloroplastic OS=Spinacia olerac PE=1 SV=1	TKTC_SPIOL	80230.6	6.2	7	66	99.155	50	99.931

Peptide Information

Calc. Mass	Obsrv. Mass	± da	± ppm	Start Seq.	End Seq.	Sequence	Ion Score	C. I. %	Modification	Rank	Result Type
996.4686	996.4653	-0.0033	-3	128	134	NPYWFNR					Mascot
1005.5615	1005.4517	-0.1098	-109	90	98	FLAIDAVEK					Mascot
1138.5123	1138.3672	-0.1451	-127	295	304	NGNTGYDEIR					Mascot
1484.8048	1484.6161	-0.1887	-127	283	294	FEALGWHVIWVK	50	99.931			Mascot
1484.8048	1484.6161	-0.1887	-127	283	294	FEALGWHVIWVK					Mascot
1589.7806	1589.5785	-0.2021	-127	420	434	ALPTYTPETPGDATR					Mascot
2724.2512	2723.8984	-0.3528	-130	99	123	ANSGHPGLPMGCAPMGHILYDEIMR			Carbamidomethyl (C)[12]		Mascot
3854.7043	3855.2732	0.5689	148	488	520	EHGMGAICNGICLHSPGFVPYCATFFVFTDYMR			Carbamidomethyl (C)[8,12,22]		Mascot

Rank	Protein Name	Accession No	Protein MW	Protein PI	Pep. Count	Protein Score	Protein Score C. I. %	Total Ion Score	Total Ion C. I. %
2	Transketolase-1, chloroplastic OS=Arabidopsis tl GN=TKL-1 PE=1 SV=1	TKTC1_ARAT	79918.2	5.94	7	66	99.155	50	99.931

Peptide Information

Calc. Mass	Obsrv. Mass	± da	± ppm	Start Seq.	End Seq.	Sequence	Ion Score	C. I. %	Modification	Rank	Result Type
856.4562	856.4161	-0.0401	-47	391	397	FAAYEKK					Mascot
856.4562	856.4161	-0.0401	-47	391	397	FAAYEKK					Mascot
996.4686	996.4653	-0.0033	-3	128	134	NPYWFNR					Mascot
1005.5615	1005.4517	-0.1098	-109	90	98	FLAIDAVEK					Mascot
1138.5123	1138.3672	-0.1451	-127	295	304	NGNTGYDEIR					Mascot
1484.8048	1484.6161	-0.1887	-127	283	294	FEALGWHVIWVK	50	99.931			Mascot
1484.8048	1484.6161	-0.1887	-127	283	294	FEALGWHVIWVK					Mascot
2280.0164	2279.7837	-0.2327	-102	658	675	VVSFVCWELFDEQSDEYK			Carbamidomethyl (C)[6]		Mascot
3376.7603	3377.114	0.3537	105	435	467	NLSQQCLNALAKVVPGFLGGSADLASSNMTLLK			Oxidation (M)[29]		Mascot

Rank	Protein Name	Accession No	Protein MW	Protein PI	Pep. Count	Protein Score	Protein Score C. I. %	Total Ion Score	Total Ion C. I. %
3	Transketolase-2, chloroplastic OS=Arabidopsis tl GN=TKL-2 PE=1 SV=1	TKTC2_ARAT	79872.1	6.12	6	63	98.314	50	99.931

Peptide Information

Calc. Mass	Obsrv. Mass	± da	± ppm	Start Seq.	End Seq.	Sequence	Ion Score	C. I. %	Modification	Rank	Result Type
856.4562	856.4161	-0.0401	-47	391	397	FAAYEKK					Mascot
856.4562	856.4161	-0.0401	-47	391	397	FAAYEKK					Mascot
996.4686	996.4653	-0.0033	-3	128	134	NPYWFNR					Mascot
1005.5615	1005.4517	-0.1098	-109	90	98	FLAIDAVEK					Mascot
1484.8048	1484.6161	-0.1887	-127	283	294	FEALGWHVIWVK	50	99.931			Mascot
1484.8048	1484.6161	-0.1887	-127	283	294	FEALGWHVIWVK					Mascot
3077.3987	3077.042	-0.3567	-116	101	127	SGHPGLPMGCAPMSHILYDEVMKYNPK			Carbamidomethyl (C)[10], Oxidation (M)[8,13,22]		Mascot
3408.6714	3409.0442	0.3728	109	137	166	FVLSAGHGCMLQYALLHLAGYDSVREEDLK			Carbamidomethyl (C)[9], Oxidation (M)[10]		Mascot

Rank	Protein Name	Accession No	Protein MW	Protein PI	Pep. Count	Protein Score	Protein Score C. I. %	Total Ion Score	Total Ion C. I. %
4	Transketolase, chloroplastic (Fragment) OS=Craterostigma plantagineum GN=TKT3 PE=1 SV=1	TKTC_CRAPI	56152.5	5.8	1	50	68.603	50	99.931

Peptide Information

Calc. Mass	Obsrv. Mass	± da	± ppm	Start Seq.	End Seq.	Sequence	Ion Score	C. I. %	Modification	Rank	Result Type
1484.8048	1484.6161	-0.1887	-127	61	72	FEALGWHVIWVK	50	99.931			Mascot
1484.8048	1484.6161	-0.1887	-127	61	72	FEALGWHVIWVK					Mascot

Rank	Protein Name	Accession No	Protein MW	Protein PI	Pep. Count	Protein Score	Protein Score C. I. %	Total Ion Score	Total Ion C. I. %
5	Ribulose bisphosphate carboxylase small chain (512 (Fragment) OS=Triticum aestivum PE=3 SV=1	RBS3_WHEA	13046.5	5.84	6	43	0		

Peptide Information

Calc. Mass	Obsrv. Mass	± da	± ppm	Start Seq.	End Seq.	Sequence	Ion Score	C. I. %	Modification	Rank	Result Type
914.4229	914.288	-0.1349	-148	50	55	YWTMWK					Mascot
1012.4734	1012.5026	0.0292	29	77	84	EYPDAYVR					Mascot
1165.571	1165.5961	0.0251	22	23	31	WVPCLEFSK			Carbamidomethyl (C)[4]		Mascot
2211.0571	2210.8064	-0.2507	-113	93	112	QVQCVSFIAFKPPGCEESGK			Carbamidomethyl (C)[4]		Mascot
2211.0571	2210.8064	-0.2507	-113	93	112	QVQCVSFIAFKPPGCEESGK			Carbamidomethyl (C)[4]		Mascot
2225.073	2224.8245	-0.2485	-112	93	113	QVQCVSFIAFKPPGCEESGKA					Mascot
2239.062	2238.8145	-0.2475	-111	56	75	LPMFGCTDATQVINEVEEVK			Oxidation (M)[3]		Mascot
2280.0886	2279.7837	-0.3049	-134	56	75	LPMFGCTDATQVINEVEEVK			Carbamidomethyl (C)[6]		Mascot

Rank	Protein Name	Accession No	Protein MW	Protein PI	Pep. Count	Protein Score	Protein Score C. I. %	Total Ion Score	Total Ion C. I. %
6	Serine acetyltransferase 5 OS=Arabidopsis thalia GN=SAT5 PE=1 SV=1	SAT5_ARATH	32749.9	6.7	7	36	0		

Peptide Information

Calc. Mass	Obsrv. Mass	± da	± ppm	Start Seq.	End Seq.	Sequence	Ion Score	C. I. %	Modification	Rank	Result Type
842.4478	842.4016	-0.0462	-55	270	278	GTAVGNPAR					Mascot
870.4501	870.4277	-0.0224	-26	1	8	MPPAGELR					Mascot
870.4501	870.4277	-0.0224	-26	1	8	MPPAGELR					Mascot
1012.5686	1012.5026	-0.066	-65	143	151	GFLAIQAHR					Mascot
1384.7616	1384.569	-0.1926	-139	256	269	VGAGSVVLIDVPCR					Mascot
2779.3896	2779.5635	0.1739	63	55	79	RDAEAEPALASYLYSTILSHSSLER					Mascot
3395.4719	3395.0464	-0.4255	-125	284	312	EKPTIHDEECPGESMDHTSFISEWSDYII					Mascot
3731.8357	3732.2585	0.4228	113	17	54	LSSVTQSDEAEAASAAIS					Mascot
	3732.2585					AAAADAEAAGLWTQIKAEAR					Mascot

Rank	Protein Name	Accession No	Protein MW	Protein PI	Pep. Count	Protein Score	Protein Score C. I. %	Total Ion Score	Total Ion C. I. %
7	UPF0496 protein At5g66675 OS=Arabidopsis tha GN=At5g66675 PE=2 SV=1	U496H_ARAT	46341.7	7.56	8	34	0		

Peptide Information

Calc. Mass	Obsrv. Mass	± da	± ppm	Start Seq.	End Seq.	Sequence	Ion Score	C. I. %	Modification	Rank	Result Type
882.4753	882.4539	-0.0214	-24	306	313	IGAYVTMK					Mascot
960.4681	960.3606	-0.1075	-112	1	8	MFLGMFSK					Mascot
1005.5509	1005.4517	-0.0992	-99	377	385	CSKNITLAR					Mascot
1465.6702	1465.5675	-0.1027	-70	2	13	FLGMFSKTMEDK			Oxidation (M)[4,9]		Mascot
2054.8794	2054.7661	-0.1133	-55	32	50	TDMGSLYSADLTAYNSACR			Oxidation (M)[3]		Mascot
2054.8794	2054.7661	-0.1133	-55	32	50	TDMGSLYSADLTAYNSACR			Oxidation (M)[3]		Mascot
3317.5525	3317.1152	-0.4373	-132	21	50	TNNTPLPQQIRTDMGSLYSADLTAYNSACR			Oxidation (M)[14]		Mascot
3743.8335	3744.3882	0.5547	148	190	221	ASGDPFDGDFFMLLESVYEQQVMLLEILHKQK			Oxidation (M)[12]		Mascot
3793.6704	3794.2065	0.5361	141	32	65	TDMGSLYSADLTAYNSACREDPDLQSFDSSLHQR					Mascot

Rank	Protein Name	Accession No	Protein MW	Protein PI	Pep. Count	Protein Score	Protein Score C. I. %	Total Ion Score	Total Ion C. I. %
8	Acyl carrier protein 2, chloroplastic OS=Spinacia oleracea GN=ACL1.2 PE=1 SV=1	ACP2_SPIOL	14089.1	4.78	5	33	0		

Peptide Information

Calc. Mass	Obsrv. Mass	± da	± ppm	Start Seq.	End Seq.	Sequence	Ion Score	C. I. %	Modification	Rank	Result Type
856.4821	856.4161	-0.066	-77	36	42	RTPVMPR					Mascot
856.4821	856.4161	-0.066	-77	36	42	RTPVMPR					Mascot
882.4468	882.4539	0.0071	8	29	35	SSVTFWR					Mascot
1038.548	1038.3975	-0.1505	-145	29	36	SSVTFWRR					Mascot
1045.5524	1045.4353	-0.1171	-112	64	73	SQLALAEDAK					Mascot
1471.7692	1471.6162	-0.153	-104	24	35	NYALKSSVTFWR					Mascot

Rank	Protein Name	Accession No	Protein MW	Protein PI	Pep. Count	Protein Score	Protein Score C. I. %	Total Ion Score	Total Ion C. I. %
9	Ribulose bisphosphate carboxylase small chain SSU40A, chloroplastic OS=Lemna gibba GN=SSU40A PE=3 S	RBS3_LEMGI	19788.7	8.24	6	33	0		

Peptide Information

Calc. Mass	Obsrv. Mass	± da	± ppm	Start Seq.	End Seq.	Sequence	Ion Score	C. I. %	Modification	Rank	Result Type
914.4229	914.288	-0.1349	-148	122	127	YWTMWK					Mascot
1384.6927	1384.569	-0.1237	-89	41	54	ANNNLSTLPGNGGR					Mascot
1471.6702	1471.6162	-0.054	-37	1	15	MASSMMASTAAAVAR			Oxidation (M)[1]		Mascot
	1471.6162										Mascot
2211.0308	2210.8064	-0.2244	-101	128	147	LPMFGCTDASQVIAEVEEAK			Carbamidomethyl (C)[6], Oxidation (M)[3]		Mascot
2211.0308	2210.8064	-0.2244	-101	128	147	LPMFGCTDASQVIAEVEEAK			Carbamidomethyl (C)[6], Oxidation (M)[3]		Mascot
2283.1113	2282.8508	-0.2605	-114	86	103	EVDYLLRNDWVPCIEFSK			Carbamidomethyl (C)[13]		Mascot
3077.373	3077.042	-0.331	-108	1	30	MASSMMASTAAAVARAGPAQSSMVPFNACR			Carbamidomethyl (C)[29], Oxidation (M)[1,5,6]		Mascot

Rank	Protein Name	Accession No	Protein MW	Protein PI	Pep. Count	Protein Score	Protein Score C. I. %	Total Ion Score	Total Ion C. I. %
10	E3 ubiquitin-protein ligase makorin OS=Arabidops thaliana GN=MKRN PE=2 SV=1	C3H35_ARAT	36538.5	7.16	8	32	0		

Peptide Information

Calc. Mass	Obsrv. Mass	± da	± ppm	Start Seq.	End Seq.	Sequence	Ion Score	C. I. %	Modification	Rank	Result Type
904.4491	904.3279	-0.1212	-134	223	229	ACPICRK			Carbamidomethyl (C)[2,5]		Mascot
1005.4683	1005.4517	-0.0166	-17	49	55	CRYDHVR			Carbamidomethyl (C)[1]		Mascot
1038.5071	1038.3975	-0.1096	-106	1	8	MSDRILCK			Carbamidomethyl (C)[7], Oxidation (M)[1]		Mascot
2056.8567	2056.7874	-0.0693	-34	118	135	GNQCPHMHGDLCNTCGKK			Carbamidomethyl (C)[4,12]		Mascot
2237.0471	2236.7803	-0.2668	-119	208	228	SSAPVSGMDVNSTLRACPICR			Carbamidomethyl (C)[17], Oxidation (M)[8]		Mascot
2298.0706	2297.8828	-0.1878	-82	72	92	SISTTPSRHLQQQGDNNDGDK					Mascot
3347.6614	3348.1633	0.5019	150	283	312	HAYSDGHLEEVVLRHLGSQEGETVITDSIR					Mascot
3395.394	3395.0464	-0.3476	-102	103	134	EYPICSFAAAGDCPRGNQCPHMHGDLCNTCGK					Mascot

Gel Idx/Pos	33/B6	Instr./Gel Origin	BA2060/131017A	Process Sta	Analysis Succeeded
Plate [#] Name	[1] 31496	Instrument Sample Name		Spectra	8

Rank	Protein Name	Accession No	Protein MW	Protein PI	Pep. Count	Protein Score	Protein Score C. I. %	Total Ion Score	Total Ion C. I. %
1	Transketolase, chloroplastic OS=Spinacia olerac PE=1 SV=1	TKTC_SPIOL	80230.6	6.2	7	123	100	108	100

Peptide Information

Calc. Mass	Obsrv. Mass	± da	± ppm	Start Seq.	End Seq.	Sequence	Ion Score	C. I. %	Modification	Rank	Result Type
996.4686	996.4643	-0.0043	-4	128	134	NPYWFNR					Mascot
1005.5615	1005.4487	-0.1128	-112	90	98	FLAIDAVEK					Mascot
1138.5123	1138.3624	-0.1499	-132	295	304	NGNTGYDEIR					Mascot
1418.7063	1418.5148	-0.1915	-135	687	699	VSIEAGSTFGWHK					Mascot
1484.8048	1484.6034	-0.2014	-136	283	294	FEALGWHVIWVK	47	99.845			Mascot
1484.8048	1484.6034	-0.2014	-136	283	294	FEALGWHVIWVK					Mascot
1589.7806	1589.5652	-0.2154	-135	420	434	ALPTYTPETPGDATR	61	99.995			Mascot
1589.7806	1589.5652	-0.2154	-135	420	434	ALPTYTPETPGDATR					Mascot
2724.2512	2723.8821	-0.3691	-135	99	123	ANSGHPGLPMGCAPMG HILYDEIMR			Carbamidomethyl (C)[12]		Mascot

Rank	Protein Name	Accession No	Protein MW	Protein PI	Pep. Count	Protein Score	Protein Score C. I. %	Total Ion Score	Total Ion C. I. %
2	Transketolase, chloroplastic OS=Solanum tuberc PE=2 SV=1	TKTC_SOLTU	79942.2	5.94	6	91	99.998	79	100

Peptide Information

Calc. Mass	Obsrv. Mass	± da	± ppm	Start Seq.	End Seq.	Sequence	Ion Score	C. I. %	Modification	Rank	Result Type
996.4686	996.4643	-0.0043	-4	128	134	NPYWFNR					Mascot
1005.5615	1005.4487	-0.1128	-112	90	98	FLAIDAVEK					Mascot
1138.5123	1138.3624	-0.1499	-132	295	304	NGNTGYDEIR					Mascot
1589.7806	1589.5652	-0.2154	-135	420	434	ALPTYTPESPADATR	79	100			Mascot
1589.7806	1589.5652	-0.2154	-135	420	434	ALPTYTPESPADATR					Mascot
2299.0845	2298.8669	-0.2176	-95	562	582	AMPNILMFRPADGNETA GAYK			Oxidation (M)[2,7]		Mascot
3793.7632	3794.2458	0.4826	127	488	520	EHGMGAICNGIALHSLGLI PYCATFFVFTDYMR			Carbamidomethyl (C)[8,22], Oxidation (M)[4,32]		Mascot

Rank	Protein Name	Accession No	Protein MW	Protein PI	Pep. Count	Protein Score	Protein Score C. I. %	Total Ion Score	Total Ion C. I. %
3	Transketolase (Fragment) OS=Populus euphratica PE=1 SV=1	TKT_POPEU	1588.8	4.38	1	84	99.986	79	100

Peptide Information

Calc. Mass	Obsrv. Mass	± da	± ppm	Start Seq.	End Seq.	Sequence	Ion Score	C. I. %	Modification	Rank	Result Type
1589.7806	1589.5652	-0.2154	-135	1	15	ALPTYTPESPADATR	79	100			Mascot
1589.7806	1589.5652	-0.2154	-135	1	15	ALPTYTPESPADATR					Mascot

Rank	Protein Name	Accession No	Protein MW	Protein PI	Pep. Count	Protein Score	Protein Score C. I. %	Total Ion Score	Total Ion C. I. %
4	Transketolase-2, chloroplastic OS=Arabidopsis tl GN=TKL-2 PE=1 SV=1	TKTC2_ARAT	79872.1	6.12	7	65	98.936	47	99.845

Peptide Information

Calc. Mass	Obsrv. Mass	± da	± ppm	Start Seq.	End Seq.	Sequence	Ion Score	C. I. %	Modification	Rank	Result Type
856.4562	856.4155	-0.0407	-48	391	397	FAAYEKK					Mascot
996.4686	996.4643	-0.0043	-4	128	134	NPYWFNR					Mascot
1005.5615	1005.4487	-0.1128	-112	90	98	FLAIDAVEK					Mascot
1484.8048	1484.6034	-0.2014	-136	283	294	FEALGWHVIWVK	47	99.845			Mascot
1484.8048	1484.6034	-0.2014	-136	283	294	FEALGWHVIWVK					Mascot
3077.3987	3077.0571	-0.3416	-111	101	127	SGHPGLPMGCAPMSHIL YDEVMKYNPK			Carbamidomethyl (C)[10], Oxidation (M)[8,13,22]		Mascot
3408.6714	3409.083	0.4116	121	137	166	FVLSAGHGCMLQYALLH LAGYDSVREEDLK			Carbamidomethyl (C)[9], Oxidation (M)[10]		Mascot
3732.9048	3732.3582	-0.5466	-146	1	36	MASTSSLALSQALLTRAI SHNGSENCVSIPAFSALK			Carbamidomethyl (C)[26]		Mascot

Rank	Protein Name	Accession No	Protein MW	Protein PI	Pep. Count	Protein Score	Protein Score C. I. %	Total Ion Score	Total Ion C. I. %
5	Transketolase-1, chloroplastic OS=Arabidopsis tl GN=TKL-1 PE=1 SV=1	TKTC1_ARAT	79918.2	5.94	7	61	97.202	47	99.845

Peptide Information

Calc. Mass	Obsrv. Mass	± da	± ppm	Start Seq.	End Seq.	Sequence	Ion Score	C. I. %	Modification	Rank	Result Type
856.4562	856.4155	-0.0407	-48	391	397	FAAYEKK					Mascot
996.4686	996.4643	-0.0043	-4	128	134	NPYWFNR					Mascot
1005.5615	1005.4487	-0.1128	-112	90	98	FLAIDAVEK					Mascot
1138.5123	1138.3624	-0.1499	-132	295	304	NGNTGYDEIR					Mascot
1484.8048	1484.6034	-0.2014	-136	283	294	FEALGWHVIWVK	47	99.845			Mascot
1484.8048	1484.6034	-0.2014	-136	283	294	FEALGWHVIWVK					Mascot
1832.9124	1832.6887	-0.2237	-122	66	83	AAAVETVEPTTDSSIVDK					Mascot
2280.0164	2279.7949	-0.2215	-97	658	675	VVSFVCWELFDEQSDEY K			Carbamidomethyl (C)[6]		Mascot

Rank	Protein Name	Accession No	Protein MW	Protein PI	Pep. Count	Protein Score	Protein Score C. I. %	Total Ion Score	Total Ion C. I. %
6	Transketolase, chloroplastic (Fragment) OS=Craterostigma plantagineum GN=TKT3 PE=1 SV=1	TKTC_CRAPL	56152.5	5.8	1	47	28.073	47	99.845

Peptide Information

Calc. Mass	Obsrv. Mass	± da	± ppm	Start Seq.	End Seq.	Sequence	Ion Score	C. I. %	Modification	Rank	Result Type
1484.8048	1484.6034	-0.2014	-136	61	72	FEALGWHVIWVK	47	99.845			Mascot
1484.8048	1484.6034	-0.2014	-136	61	72	FEALGWHVIWVK					Mascot

Rank	Protein Name	Accession No	Protein MW	Protein PI	Pep. Count	Protein Score	Protein Score C. I. %	Total Ion Score	Total Ion C. I. %
7	Putative cytochrome c oxidase subunit II PS17 (Fragments) OS=Pinus strobus PE=1 SV=1	PS17_PINST	1707	9.63	2	35	0	12	0

Peptide Information

Calc. Mass	Obsrv. Mass	± da	± ppm	Start Seq.	End Seq.	Sequence	Ion Score	C. I. %	Modification	Rank	Result Type
856.525	856.4155	-0.1095	-128	1	8	SPTVIALR					Mascot
870.5043	870.4279	-0.0764	-88	9	16	VVEALSPR					Mascot
870.5043	870.4279	-0.0764	-88	9	16	VVEALSPR	12	0			Mascot

Rank	Protein Name	Accession No	Protein MW	Protein PI	Pep. Count	Protein Score	Protein Score C. I. %	Total Ion Score	Total Ion C. I. %
8	Ribulose bisphosphate carboxylase small chain, chloroplastic OS=Glycine tomentella GN=RBCS PE=3 SV=	RBS_GLYTO	19936	8.87	6	33	0		

Peptide Information

Calc. Mass	Obsrv. Mass	± da	± ppm	Start Seq.	End Seq.	Sequence	Ion Score	C. I. %	Modification	Rank	Result Type
882.4138	882.4516	0.0378	43	30	37	SMAGFPTR			Oxidation (M)[2]		Mascot
914.4002	914.2845	-0.1157	-127	113	120	SPGYYDGR					Mascot
933.5151	933.3911	-0.124	-133	156	163	IIGFDNVR					Mascot
1038.5367	1038.397	-0.1397	-135	147	155	TAYPNGFIR					Mascot
2280.127	2279.7949	-0.3321	-146	91	108	KGWIPCLEFELEHGFVYR			Carbamidomethyl (C)[6]		Mascot
2748.3191	2747.9255	-0.3936	-143	39	64	TNNDIASIASNGGRVQCM QVWPTTGK					Mascot

Rank	Protein Name	Accession No	Protein MW	Protein PI	Pep. Count	Protein Score	Protein Score C. I. %	Total Ion Score	Total Ion C. I. %
9	Acyl carrier protein 2, chloroplastic OS=Spinacia oleracea GN=ACL1.2 PE=1 SV=1	ACP2_SPIOL	14089.1	4.78	5	33	0		

Peptide Information

Calc. Mass	Obsrv. Mass	± da	± ppm	Start Seq.	End Seq.	Sequence	Ion Score	C. I. %	Modification	Rank	Result Type
856.4821	856.4155	-0.0666	-78	36	42	RTPVMPR					Mascot
882.4468	882.4516	0.0048	5	29	35	SSVTFWR					Mascot
1038.548	1038.397	-0.151	-145	29	36	SSVTFWRR					Mascot
1045.5524	1045.4269	-0.1255	-120	64	73	SQLALAEDAK					Mascot
1471.7692	1471.5973	-0.1719	-117	24	35	NYALKSSVTFWR					Mascot

Rank	Protein Name	Accession No	Protein MW	Protein PI	Pep. Count	Protein Score	Protein Score C. I. %	Total Ion Score	Total Ion C. I. %
10	Ribulose bisphosphate carboxylase small chain (512 (Fragment) OS=Triticum aestivum PE=3 SV=1	RBS3_WHEAT	13046.5	5.84	5	33	0		

Peptide Information

Calc. Mass	Obsrv. Mass	± da	± ppm	Start Seq.	End Seq.	Sequence	Ion Score	C. I. %	Modification	Rank	Result Type
1012.4734	1012.4941	0.0207	20	77	84	EYPDAYVR					Mascot
1165.571	1165.5907	0.0197	17	23	31	WVPCLEFSK			Carbamidomethyl (C)[4]		Mascot
2211.0571	2210.7971	-0.26	-118	93	112	QVQCVSFIAFKPPGCEES GK			Carbamidomethyl (C)[4]		Mascot
2211.0571	2210.7971	-0.26	-118	93	112	QVQCVSFIAFKPPGCEES GK			Carbamidomethyl (C)[4]		Mascot
2225.073	2224.812	-0.261	-117	93	113	QVQCVSFIAFKPPGCEES GKA					Mascot
2239.062	2238.7949	-0.2671	-119	56	75	LPMFGCTDATQVINEVEE VK			Oxidation (M)[3]		Mascot
2280.0886	2279.7949	-0.2937	-129	56	75	LPMFGCTDATQVINEVEE VK			Carbamidomethyl (C)[6]		Mascot

Gel Idx/Pos	34/B7	Instr./Gel Origin	BA2060/131017A	Process Sta	Analysis Succeeded
Plate [#] Name	[1] 31496	Instrument Sample Name		Spectra	8

Rank	Protein Name	Accession No	Protein MW	Protein PI	Pep Count	Protein Score	Protein Score C. I. %	Total Ion Score	Total Ion C. I. %
1	Stromal 70 kDa heat shock-related protein, chloro OS=Pisum sativum GN=HSP70 PE=2 SV=1	HSP7S_PEA	75469.3	5.22	16	544	100	468	100

Peptide Information

Calc. Mass	Obsrv. Mass	± da	± ppm	Start Seq.	End Seq.	Sequence	Ion Score	C. I. %	Modification	Rank	Result Type
870.5406	870.4306	-0.11	-126	226	233	IAGLEVLR	12	0			Mascot
870.5406	870.4306	-0.11	-126	226	233	IAGLEVLR					Mascot
915.4894	915.374	-0.1154	-126	379	386	TPVENSLR					Mascot
935.5308	935.4103	-0.1205	-129	520	528	LDGIPPAPR					Mascot
970.5316	970.4083	-0.1233	-127	356	363	HIETTLTR					Mascot
996.6088	996.4786	-0.1302	-131	407	415	IPAVQELVK					Mascot
1197.5304	1197.5126	-0.0178	-15	142	151	KMSEVDEESK			Oxidation (M)[2]		Mascot
1199.5725	1199.496	-0.0765	-64	576	585	MVSEAERFSK			Oxidation (M)[1]		Mascot
1373.7271	1373.5525	-0.1746	-127	394	406	DIDEVILVGGSTR	115	100			Mascot
1373.7271	1373.5525	-0.1746	-127	394	406	DIDEVILVGGSTR					Mascot
1381.6594	1381.4764	-0.183	-132	598	609	NQADSVVYQTEK					Mascot
1396.6412	1396.4613	-0.1799	-129	366	376	FEELCSDLLDR			Carbamidomethyl (C)[5]		Mascot
1566.791	1566.5835	-0.2075	-132	204	217	AVVTVPAYFNDSQR	71	100			Mascot
1566.791	1566.5835	-0.2075	-132	204	217	AVVTVPAYFNDSQR					Mascot
1579.8114	1579.599	-0.2124	-134	123	136	QAVVNPENTFFSVK					Mascot
1595.7733	1595.5574	-0.2159	-135	364	376	AKFEELCSDLLDR			Carbamidomethyl (C)[7]		Mascot
1595.7733	1595.5574	-0.2159	-135	364	376	AKFEELCSDLLDR	60	99.993	Carbamidomethyl (C)[7]		Mascot
1735.9126	1735.6774	-0.2352	-135	123	137	QAVVNPENTFFSVKR					Mascot
2437.1841	2436.8462	-0.3379	-139	484	506	SEVFSTAADGQTSVEINVLQGER					Mascot
2437.1841	2436.8462	-0.3379	-139	484	506	SEVFSTAADGQTSVEINVLQGER	210	100			Mascot
3070.5837	3070.1418	-0.4419	-144	70	100	VVGIDLGTTNSAVAAMEGGKPTIITNAEGQR					Mascot
3086.5786	3086.1509	-0.4277	-139	70	100	VVGIDLGTTNSAVAAMEGGKPTIITNAEGQR			Oxidation (M)[16]		Mascot

Rank	Protein Name	Accession No	Protein MW	Protein PI	Pep Count	Protein Score	Protein Score C. I. %	Total Ion Score	Total Ion C. I. %
2	Heat shock 70 kDa protein 6, chloroplastic OS=Arabidopsis thaliana GN=HSP70-6 PE=1 SV=1	HSP7F_ARAT	76461.4	5.07	13	520	100	471	100

Peptide Information

Calc. Mass	Obsrv. Mass	± da	± ppm	Start Seq.	End Seq.	Sequence	Ion Score	C. I. %	Modification	Rank	Result Type
870.5406	870.4306	-0.11	-126	236	243	IAGLEVLR	12	0			Mascot
870.5406	870.4306	-0.11	-126	236	243	IAGLEVLR					Mascot
915.4894	915.374	-0.1154	-126	389	396	TPVENSLR					Mascot
935.5308	935.4103	-0.1205	-129	530	538	LDGIPPAPR					Mascot
970.5316	970.4083	-0.1233	-127	366	373	HIETTLTR					Mascot
1373.7271	1373.5525	-0.1746	-127	404	416	DIDEVILVGGSTR	115	100			Mascot
1373.7271	1373.5525	-0.1746	-127	404	416	DIDEVILVGGSTR					Mascot
1381.6594	1381.4764	-0.183	-132	608	619	NQADSVVYQTEK					Mascot
1396.6412	1396.4613	-0.1799	-129	376	386	FEELCSDLLDR			Carbamidomethyl (C)[5]		Mascot
1461.7695	1461.5776	-0.1919	-131	185	197	QFAAEEISAQVLR					Mascot
1461.7695	1461.5776	-0.1919	-131	185	197	QFAAEEISAQVLR	73	100			Mascot
1579.8114	1579.599	-0.2124	-134	133	146	QAVVNPENTFFSVK					Mascot
1595.7733	1595.5574	-0.2159	-135	374	386	AKFEELCSDLLDR			Carbamidomethyl (C)[7]		Mascot
1595.7733	1595.5574	-0.2159	-135	374	386	AKFEELCSDLLDR	60	99.993	Carbamidomethyl (C)[7]		Mascot
1735.9126	1735.6774	-0.2352	-135	133	147	QAVVNPENTFFSVKR					Mascot
1849.0065	1848.754	-0.2525	-137	400	416	LSFKDIDEVILVGGSTR					Mascot
2437.1841	2436.8462	-0.3379	-139	494	516	SEVFSTAADGQTSVEINVLQGER					Mascot
2437.1841	2436.8462	-0.3379	-139	494	516	SEVFSTAADGQTSVEINVLQGER	210	100			Mascot

Rank	Protein Name	Accession No	Protein MW	Protein PI	Pep Count	Protein Score	Protein Score C. I. %	Total Ion Score	Total Ion C. I. %
3	Heat shock 70 kDa protein 7, chloroplastic OS=Arabidopsis thaliana GN=HSP70-7 PE=1 SV=1	HSP7G_ARA	76949.7	5.17	13	459	100	410	100

Peptide Information

Calc. Mass	Obsrv. Mass	± da	± ppm	Start Seq.	End Seq.	Sequence	Ion Score	C. I. %	Modification	Rank	Result Type
870.5406	870.4306	-0.11	-126	236	243	IAGLEVLR	12	0			Mascot
870.5406	870.4306	-0.11	-126	236	243	IAGLEVLR					Mascot
915.4894	915.374	-0.1154	-126	389	396	TPVENSLR					Mascot
935.5308	935.4103	-0.1205	-129	530	538	LDGIPPAPR					Mascot
970.5316	970.4083	-0.1233	-127	366	373	HIETTLTR					Mascot
1192.5626	1192.4845	-0.0781	-65	152	161	RMNEVAEESK					Mascot
1373.7271	1373.5525	-0.1746	-127	404	416	DIDEVILVGGSTR	115	100			Mascot
1373.7271	1373.5525	-0.1746	-127	404	416	DIDEVILVGGSTR					Mascot
1381.6594	1381.4764	-0.183	-132	608	619	NQADSVVYQTEK					Mascot
1396.6412	1396.4613	-0.1799	-129	376	386	FEELCSDLLDR			Carbamidomethyl (C)[5]		Mascot
1461.7695	1461.5776	-0.1919	-131	185	197	QFAAEEISAQVLR					Mascot
1461.7695	1461.5776	-0.1919	-131	185	197	QFAAEEISAQVLR	73	100			Mascot
1579.8114	1579.599	-0.2124	-134	133	146	QAVVNPENTFFSVK					Mascot
1735.9126	1735.6774	-0.2352	-135	133	147	QAVVNPENTFFSVKR					Mascot
1849.0065	1848.754	-0.2525	-137	400	416	LSFKDIDEVILVGGSTR					Mascot
2437.1841	2436.8462	-0.3379	-139	494	516	SEVFSTAADGQTSVEINVLQGER					Mascot
2437.1841	2436.8462	-0.3379	-139	494	516	SEVFSTAADGQTSVEINVLQGER	210	100			Mascot

Rank	Protein Name	Accession No	Protein MW	Protein PI	Pep Count	Protein Score	Protein Score C. I. %	Total Ion Score	Total Ion C. I. %
4	Stromal 70 kDa heat shock-related protein, chloro (Fragment) OS=Spinacia oleracea GN=CHSP70	HSP7S_SPIO	64860.7	4.87	10	402	100	367	100

Peptide Information

Calc. Mass	Obsrv. Mass	± da	± ppm	Start Seq.	End Seq.	Sequence	Ion Score	C. I. %	Modification	Rank	Result Type
870.5406	870.4306	-0.11	-126	106	113	IAGLEVLR	12	0			Mascot
870.5406	870.4306	-0.11	-126	106	113	IAGLEVLR					Mascot
915.4894	915.374	-0.1154	-126	259	266	TPVENSLR					Mascot
935.5308	935.4103	-0.1205	-129	400	408	LDGIPPAPR					Mascot
970.5316	970.4083	-0.1233	-127	236	243	HIETTLTR					Mascot
1381.6594	1381.4764	-0.183	-132	478	489	NQADSVVYQTEK					Mascot
1461.7695	1461.5776	-0.1919	-131	55	67	QFAAEEISAQVLR					Mascot
1461.7695	1461.5776	-0.1919	-131	55	67	QFAAEEISAQVLR	73	100			Mascot
1566.791	1566.5835	-0.2075	-132	84	97	AVVTVPAYFNDSQR	71	100			Mascot
1566.791	1566.5835	-0.2075	-132	84	97	AVVTVPAYFNDSQR					Mascot
1691.8751	1691.6543	-0.2208	-131	246	258	FEELWSDLLDRLR					Mascot
1723.8901	1723.6547	-0.2354	-137	114	129	IINEPTAASLAYGFEK					Mascot
2437.1841	2436.8462	-0.3379	-139	364	386	SEVFSTAADGQTSVEINVLQGER					Mascot
2437.1841	2436.8462	-0.3379	-139	364	386	SEVFSTAADGQTSVEINVLQGER	210	100			Mascot

Rank	Protein Name	Accession No	Protein MW	Protein PI	Pep Count	Protein Score	Protein Score C. I. %	Total Ion Score	Total Ion C. I. %
5	Heat shock 70 kDa protein OS=Chlamydomonas reinhardtii GN=HSP70 PE=2 SV=2	HSP70_CHLR	70953.2	5.2	6	87	99.994	71	100

Peptide Information

Calc. Mass	Obsrv. Mass	± da	± ppm	Start Seq.	End Seq.	Sequence	Ion Score	C. I. %	Modification	Rank	Result Type
949.4481	949.4258	-0.0223	-23	318	325	KCMDPVEK					Mascot
1197.6626	1197.5126	-0.15	-125	465	475	FELTGIPPAPR					Mascot
1487.7013	1487.5131	-0.1882	-127	38	50	TTPSYVAFTDTER					Mascot
1566.791	1566.5835	-0.2075	-132	146	159	AVVTVPAYFNDSQR	71	100			Mascot
1566.791	1566.5835	-0.2075	-132	146	159	AVVTVPAYFNDSQR					Mascot
2658.2681	2657.8882	-0.3799	-143	431	453	EQVFSTYSDNQPGVLIQVYEGER					Mascot
3854.8547	3854.334	-0.5207	-135	603	645	ELEGLCNPIITRLYQGGAGAGGMPGGGAGAGAAPSGGSGAGPK			Carbamidomethyl (C)[6], Oxidation (M)[23]		Mascot

Rank	Protein Name	Accession No	Protein MW	Protein PI	Pep Count	Protein Score	Protein Score C. I. %	Total Ion Score	Total Ion C. I. %
6	Heat shock 70 kDa protein 3 OS=Arabidopsis thal GN=HSP70-3 PE=1 SV=1	HSP7C_ARA	71103.2	4.97	11	41	0		

Peptide Information

Calc. Mass	Obsrv. Mass	± da	± ppm	Start Seq.	End Seq.	Sequence	Ion Score	C. I. %	Modification	Rank	Result Type
1045.5524	1045.4291	-0.1233	-118	564	573	IGEKLAGDDK					Mascot
1278.63	1278.4618	-0.1682	-132	243	252	MVNHFVQEFK					Mascot
1329.7083	1329.5499	-0.1584	-119	604	615	ELESICNPIIAK					Mascot
1358.6157	1358.4391	-0.1766	-130	546	556	NALENYAYNMR					Mascot
1358.6157	1358.5466	-0.0691	-51	546	556	NALENYAYNMR					Mascot
1473.6857	1473.5342	-0.1515	-103	40	52	TTPSYVAFTDSER					Mascot
1675.7307	1675.5057	-0.225	-134	227	242	ATAGDTHLGGEDFDNR					Mascot
1680.834	1680.6094	-0.2246	-134	145	159	NAVVTVPAYFNDSQR					Mascot
2588.2197	2587.8804	-0.3393	-131	5	28	GEGPAIGIDLGTTYSCVGVWQHDR			Carbamidomethyl (C)[16]		Mascot
2658.2681	2657.8882	-0.3799	-143	431	453	EQVFSTYSDNQPGVLIQVYEGER					Mascot
2663.1348	2662.9316	-0.2032	-76	616	644	MYQGGEAGGPAAGGMDEDVPPSAGGAGPK			Oxidation (M)[1,15]		Mascot
3326.5078	3326.2393	-0.2685	-81	576	603	IEDSIEAAIEWLEANQLAECDEFEDKMK			Carbamidomethyl (C)[20]		Mascot
3342.5027	3342.2671	-0.2356	-70	576	603	IEDSIEAAIEWLEANQLAECDEFEDKMK			Carbamidomethyl (C)[20], Oxidation (M)[27]		Mascot

Rank	Protein Name	Accession No	Protein MW	Protein PI	Pep Count	Protein Score	Protein Score C. I. %	Total Ion Score	Total Ion C. I. %
7	Serine/threonine-protein phosphatase BSL1 hom OS=Oryza sativa subsp. japonica GN=BSL1 PE=2 SV=1	BSL1_ORYSJ	96221.2	5.9	12	38	0		

Peptide Information

Calc. Mass	Obsrv. Mass	± da	± ppm	Start Seq.	End Seq.	Sequence	Ion Score	C. I. %	Modification	Rank	Result Type
870.4138	870.4306	0.0168	19	210	217	MYATASAR					Mascot
870.4138	870.4306	0.0168	19	210	217	MYATASAR					Mascot
882.4501	882.4512	0.0011	1	768	774	VTEFCKR					Mascot
1192.5813	1192.4845	-0.0968	-81	218	228	TDGMLLLCGGR			Carbamidomethyl (C)[8]		Mascot
1197.597	1197.5126	-0.0844	-70	199	209	LNPDGDRPSAR					Mascot
1461.6427	1461.5776	-0.0651	-45	504	516	MHPSSNDQSYPAK					Mascot
1461.6427	1461.5776	-0.0651	-45	504	516	MHPSSNDQSYPAK					Mascot
1465.6918	1465.5708	-0.121	-83	491	502	QLSLDQFENESR					Mascot
1595.7521	1595.5574	-0.1947	-122	317	330	SSHDHDASSDLLRR					Mascot
1595.7521	1595.5574	-0.1947	-122	317	330	SSHDHDASSDLLRR	2	0			Mascot
1707.7245	1707.5343	-0.1902	-111	18	33	EVEGWEGAGDDSPGFR					Mascot
2059.9722	2059.7676	-0.2046	-99	210	228	MYATASARTDGMLLLCGGR			Carbamidomethyl (C)[16], Oxidation (M)[1]		Mascot
2059.9722	2059.7676	-0.2046	-99	210	228	MYATASARTDGMLLLCGGR			Carbamidomethyl (C)[16], Oxidation (M)[1]		Mascot
2089.1111	2088.8787	-0.2324	-111	51	72	LILFGGATAIEAGASSGMPGIR					Mascot
2225.0115	2224.8076	-0.2039	-92	666	683	LECIERMGESDGIWAWTR			Carbamidomethyl (C)[3], Oxidation (M)[7]		Mascot
3326.6118	3326.2393	-0.3725	-112	849	877	AMDATWMQELNIQRPPTPTRGRPQSASDR			Oxidation (M)[2]		Mascot
3342.6067	3342.2671	-0.3396	-102	849	877	AMDATWMQELNIQRPPTPTRGRPQSASDR			Oxidation (M)[2,7]		Mascot

Rank	Protein Name	Accession No	Protein MW	Protein PI	Pep Count	Protein Score	Protein Score C. I. %	Total Ion Score	Total Ion C. I. %
8	Putative cytochrome c oxidase subunit II PS17 (Fragments) OS=Pinus strobus PE=1 SV=1	PS17_PINST	1707	9.63	2	38	0	15	0

Peptide Information

Calc. Mass	Obsrv. Mass	± da	± ppm	Start Seq.	End Seq.	Sequence	Ion Score	C. I. %	Modification	Rank	Result Type
856.525	856.4169	-0.1081	-126	1	8	SPTVIALR					Mascot
870.5043	870.4306	-0.0737	-85	9	16	VVEALSPR	15	0			Mascot
870.5043	870.4306	-0.0737	-85	9	16	VVEALSPR					Mascot

Rank	Protein Name	Accession No	Protein MW	Protein PI	Pep Count	Protein Score	Protein Score C. I. %	Total Ion Score	Total Ion C. I. %
9	Stellacyanin OS=Toxicodendron vernicifluum PE SV=1	STEL_TOXVE	12288.1	9.17	5	37	0		

Peptide Information

Calc. Mass	Obsrv. Mass	± da	± ppm	Start Seq.	End Seq.	Sequence	Ion Score	C. I. %	Modification	Rank	Result Type
915.4794	915.374	-0.1054	-115	44	50	RFHNVDK					Mascot
1275.7096	1275.5702	-0.1394	-109	30	40	TFHIGDVLVFK					Mascot
1487.6842	1487.5131	-0.1711	-115	13	24	VPFFGDVDYDWK					Mascot
1723.8295	1723.6547	-0.1748	-101	84	98	YYICGVPKHCDLGQK					Mascot
2691.1951	2690.9263	-0.2688	-100	51	74	VTQKNYQSCNDTTPIASYNTGBBR					Mascot

Rank	Protein Name	Accession No	Protein MW	Protein PI	Pep Count	Protein Score	Protein Score C. I. %	Total Ion Score	Total Ion C. I. %
10	Zinc finger CCCH domain-containing protein 34 OS=Oryza sativa subsp. japonica GN=Os05g0176400 PE=2	C3H34_ORYS	55597.1	6.04	10	36	0		

Peptide Information

Calc. Mass	Obsrv. Mass	± da	± ppm	Start Seq.	End Seq.	Sequence	Ion Score	C. I. %	Modification	Rank	Result Type
842.3824	842.4041	0.0217	26	33	39	GSKCEYR					Mascot
1197.5139	1197.5126	-0.0013	-1	57	67	GNCVNPSCTFR					Mascot
1373.6332	1373.5525	-0.0807	-59	428	440	SSEHPTADFTGPK					Mascot
1373.6332	1373.5525	-0.0807	-59	428	440	SSEHPTADFTGPK					Mascot
1443.6611	1443.5625	-0.0986	-68	2	14	AAAAEGDEDPRWR					Mascot
1675.7415	1675.5057	-0.2358	-141	152	166	DTITNPCQDTSCHIK					Mascot
1691.813	1691.6543	-0.1587	-94	379	392	NSTQQIESMPTRQR			Oxidation (M)[9]		Mascot
2224.9514	2224.8076	-0.1438	-65	50	67	NCWYWFKGNCVNPSCTFR					Mascot
2297.0913	2296.8767	-0.2146	-93	57	76	GNCVNPSCTFRHPPLENLNK			Carbamidomethyl (C)[3]		Mascot
3086.3386	3086.1509	-0.1877	-61	140	166	SYVGDEMVEESKDTITNPCQDTSCHIK			Carbamidomethyl (C)[19]		Mascot
3327.5459	3327.2449	-0.301	-90	167	197	EVPVSINPEFGEAEAVSGALETSTDIDEYMK					Mascot

Gel Idx/Pos	35/B8	Instr./Gel Origin	BA2060/131017A	Process Sta	Analysis Succeeded
Plate [#] Name	[1] 31496	Instrument Sample Name		Spectra	11

Rank	Protein Name	Accession No	Protein MW	Protein PI	Pep. Count	Protein Score	Protein Score C. I. %	Total Ion Score	Total Ion C. I. %
1	Stromal 70 kDa heat shock-related protein, chloro OS=Pisum sativum GN=HSP70 PE=2 SV=1	HSP7S_PEA	75469.3	5.22	10	127	100	99	100

Peptide Information

Calc. Mass	Obsrv. Mass	± da	± ppm	Start Seq.	End Seq.	Sequence	Ion Score	C. I. %	Modification	Rank	Result Type
870.5406	870.4269	-0.1137	-131	226	233	IAGLEVLR					Mascot
870.5406	870.4269	-0.1137	-131	226	233	IAGLEVLR					Mascot
996.6088	996.4672	-0.1416	-142	407	415	IPAVQELVK					Mascot
1170.6589	1170.4855	-0.1734	-148	111	121	NGDRLVGQIAK					Mascot
1197.5304	1197.5048	-0.0256	-21	142	151	KMSEVDEESK			Oxidation (M)[2]		Mascot
1373.7271	1373.5461	-0.181	-132	394	406	DIDEVILVGGSTR	82	100			Mascot
1373.7271	1373.5461	-0.181	-132	394	406	DIDEVILVGGSTR					Mascot
1566.791	1566.5809	-0.2101	-134	204	217	AVVTVPAYFNDSQR	16	0			Mascot
1566.791	1566.5809	-0.2101	-134	204	217	AVVTVPAYFNDSQR					Mascot
1595.7733	1595.559	-0.2143	-134	364	376	AKFEELCSDLLDR			Carbamidomethyl (C)[7]		Mascot
1686.7639	1686.5826	-0.1813	-107	143	156	MSEVDEESKQVSYR					Mascot
1892.9535	1892.6868	-0.2667	-141	1	19	MASSAQIHGLGTASFSSLK					Mascot
2437.1841	2436.8403	-0.3438	-141	484	506	SEVFSTAADGQTSVEINVLQGER					Mascot

Rank	Protein Name	Accession No	Protein MW	Protein PI	Pep. Count	Protein Score	Protein Score C. I. %	Total Ion Score	Total Ion C. I. %
2	Ribulose bisphosphate carboxylase large chain (Fragment) OS=Manilkara zapota GN=rbcL PE=3 SV=2	RBL_MANZA	51550	6.04	14	104	100	25	67.202

Peptide Information

Calc. Mass	Obsrv. Mass	± da	± ppm	Start Seq.	End Seq.	Sequence	Ion Score	C. I. %	Modification	Rank	Result Type
898.41	898.2976	-0.1124	-125	296	302	NHGMHFR					Mascot
910.4451	910.3295	-0.1156	-127	178	184	AVYECLR			Carbamidomethyl (C)[5]		Mascot
914.4049	914.2867	-0.1182	-129	296	302	NHGMHFR			Oxidation (M)[4]		Mascot
914.4049	914.2867	-0.1182	-129	296	302	NHGMHFR			Oxidation (M)[4]		Mascot
1021.5312	1021.4	-0.1312	-128	23	31	DTDILAAFR					Mascot
1116.583	1116.4341	-0.1489	-133	412	421	VALEACVQAR			Carbamidomethyl (C)[6]		Mascot
1170.5585	1170.4855	-0.073	-62	294	302	QKNHGMHFR			Oxidation (M)[6]		Mascot
1187.6643	1187.5093	-0.155	-131	276	285	DNGLLLHIHR					Mascot
1245.6433	1245.4746	-0.1687	-135	430	440	EGNEIIREASK					Mascot
1275.7307	1275.5624	-0.1683	-132	330	340	EITLGFVDLLR	25	67.202			Mascot
1275.7307	1275.5624	-0.1683	-132	330	340	EITLGFVDLLR					Mascot
1461.7195	1461.5762	-0.1433	-98	206	217	DRFVFCAEAIYK					Mascot
1465.7546	1465.561	-0.1936	-132	137	149	TFQGPPHGIQVER					Mascot
1502.8512	1502.6508	-0.2004	-133	155	167	YGRPLLGCTIKPK			Carbamidomethyl (C)[8]		Mascot
2169.9868	2169.6931	-0.2937	-135	185	203	GGLDFTKDDENVNSQPFMR					Mascot
2185.9819	2185.6792	-0.3027	-138	185	203	GGLDFTKDDENVNSQPFMR			Oxidation (M)[18]		Mascot
3022.4548	3022.0188	-0.436	-144	249	275	ELGVPIVMHDYLTGGFTANTSLAHYCR			Carbamidomethyl (C)[26]		Mascot
3854.8718	3854.3113	-0.5605	-145	32	69	VTPQPGVPPEEAGAAVAAESSTGTWTTVWTDGLTSLDR					Mascot

Rank	Protein Name	Accession No	Protein MW	Protein PI	Pep. Count	Protein Score	Protein Score C. I. %	Total Ion Score	Total Ion C. I. %
3	Ribulose bisphosphate carboxylase large chain (Fragment) OS=Sinapis alba GN=rbcL PE=3 SV=1	RBL_SINAL	52405.4	6.23	14	101	100	25	67.202

Peptide Information

Calc. Mass	Obsrv. Mass	± da	± ppm	Start Seq.	End Seq.	Sequence	Ion Score	C. I. %	Modification	Rank	Result Type
856.3546	856.4155	0.0609	71	467	473	FEFPAMD					Mascot
898.41	898.2976	-0.1124	-125	306	312	NHGMHFR					Mascot
910.4451	910.3295	-0.1156	-127	188	194	AVYECLR			Carbamidomethyl (C)[5]		Mascot
914.4049	914.2867	-0.1182	-129	306	312	NHGMHFR			Oxidation (M)[4]		Mascot
914.4049	914.2867	-0.1182	-129	306	312	NHGMHFR			Oxidation (M)[4]		Mascot
1021.5312	1021.4	-0.1312	-128	33	41	DTDILAAFR					Mascot
1116.583	1116.4341	-0.1489	-133	422	431	VALEACVQAR			Carbamidomethyl (C)[6]		Mascot
1170.5585	1170.4855	-0.073	-62	304	312	QKNHGMHFR			Oxidation (M)[6]		Mascot
1187.6643	1187.5093	-0.155	-131	286	295	DNGLLLHIHR					Mascot
1245.6433	1245.4746	-0.1687	-135	440	450	EGNEIIREASK					Mascot
1275.7307	1275.5624	-0.1683	-132	340	350	EITLGFVDLLR	25	67.202			Mascot
1275.7307	1275.5624	-0.1683	-132	340	350	EITLGFVDLLR					Mascot
1465.7546	1465.561	-0.1936	-132	147	159	TFQGPPHGIQVER					Mascot
1502.8512	1502.6508	-0.2004	-133	165	177	YGRPLLGCTIKPK			Carbamidomethyl (C)[8]		Mascot
2169.9868	2169.6931	-0.2937	-135	195	213	GGLDFTKDDENVNSQPFMR					Mascot
2185.9819	2185.6792	-0.3027	-138	195	213	GGLDFTKDDENVNSQPFMR			Oxidation (M)[18]		Mascot
3022.4548	3022.0188	-0.436	-144	259	285	ELGVPIVMHDYLTGGFTANTSLAHYCR			Carbamidomethyl (C)[26]		Mascot
3854.8718	3854.3113	-0.5605	-145	42	79	VTPQPGVPPEEAGAAVAAESSTGTWTTVWTDGLTSLDR					Mascot

Rank	Protein Name	Accession No	Protein MW	Protein PI	Pep. Count	Protein Score	Protein Score C. I. %	Total Ion Score	Total Ion C. I. %
4	Ribulose bisphosphate carboxylase large chain OS=Sesbania sesban GN=rbcL PE=3 SV=1	RBL_SESSE	52358.4	6.09	14	99	100	25	67.202

Peptide Information

Calc. Mass	Obsrv. Mass	± da	± ppm	Start Seq.	End Seq.	Sequence	Ion Score	C. I. %	Modification	Rank	Result Type
856.3546	856.4155	0.0609	71	467	473	FEFPAMD					Mascot
898.41	898.2976	-0.1124	-125	306	312	NHGMHFR					Mascot
910.4451	910.3295	-0.1156	-127	188	194	AVYECLR			Carbamidomethyl (C)[5]		Mascot
914.4049	914.2867	-0.1182	-129	306	312	NHGMHFR			Oxidation (M)[4]		Mascot
914.4049	914.2867	-0.1182	-129	306	312	NHGMHFR			Oxidation (M)[4]		Mascot
1021.5312	1021.4	-0.1312	-128	33	41	DTDILAAFR					Mascot
1116.583	1116.4341	-0.1489	-133	422	431	VALEACVQAR			Carbamidomethyl (C)[6]		Mascot
1170.5585	1170.4855	-0.073	-62	304	312	QKNHGMHFR			Oxidation (M)[6]		Mascot
1187.6643	1187.5093	-0.155	-131	286	295	DNGLLLHIHR					Mascot
1245.6337	1245.4746	-0.1591	-128	218	227	FLFCAEAIFK			Carbamidomethyl (C)[4]		Mascot
1275.7307	1275.5624	-0.1683	-132	340	350	EITLGFVDLLR	25	67.202			Mascot
1275.7307	1275.5624	-0.1683	-132	340	350	EITLGFVDLLR					Mascot
1465.7546	1465.561	-0.1936	-132	147	159	TFQGPPHGIQVER					Mascot
1502.8512	1502.6508	-0.2004	-133	165	177	YGRPLLGCTIKPK			Carbamidomethyl (C)[8]		Mascot
2169.9868	2169.6931	-0.2937	-135	195	213	GGLDFTKDDENVNSQPFMR					Mascot
2185.9819	2185.6792	-0.3027	-138	195	213	GGLDFTKDDENVNSQPFMR			Oxidation (M)[18]		Mascot
3022.4548	3022.0188	-0.436	-144	259	285	ELGVPIVMHDYLTGGFTANTSLAHYCR			Carbamidomethyl (C)[26]		Mascot
3854.8718	3854.3113	-0.5605	-145	42	79	VTPQPGVPPEEAGAAVAAESSTGTWTTVWTDGLTSLDR					Mascot

Rank	Protein Name	Accession No	Protein MW	Protein PI	Pep. Count	Protein Score	Protein Score C. I. %	Total Ion Score	Total Ion C. I. %
5	Ribulose bisphosphate carboxylase large chain (Fragment) OS=Geum quellyon GN=rbcL PE=3 SV=1	RBL_GEUQU	50910.8	6.23	13	96	100	25	67.202

Peptide Information

Calc. Mass	Obsrv. Mass	± da	± ppm	Start Seq.	End Seq.	Sequence	Ion Score	C. I. %	Modification	Rank	Result Type
898.41	898.2976	-0.1124	-125	296	302	NHGMHFR					Mascot
910.4451	910.3295	-0.1156	-127	178	184	AVYECLR			Carbamidomethyl (C)[5]		Mascot
914.4049	914.2867	-0.1182	-129	296	302	NHGMHFR			Oxidation (M)[4]		Mascot
914.4049	914.2867	-0.1182	-129	296	302	NHGMHFR			Oxidation (M)[4]		Mascot
1021.5312	1021.4	-0.1312	-128	23	31	DTDILAAFR					Mascot
1116.583	1116.4341	-0.1489	-133	412	421	VALEACVQAR			Carbamidomethyl (C)[6]		Mascot
1170.5585	1170.4855	-0.073	-62	294	302	QKNHGMHFR			Oxidation (M)[6]		Mascot
1187.6643	1187.5093	-0.155	-131	276	285	DNGLLLHIHR					Mascot
1245.6337	1245.4746	-0.1591	-128	208	217	FLFCAEALFK			Carbamidomethyl (C)[4]		Mascot
1275.7307	1275.5624	-0.1683	-132	330	340	EITLGFVDLLR	25	67.202			Mascot
1275.7307	1275.5624	-0.1683	-132	330	340	EITLGFVDLLR					Mascot
1465.7216	1465.561	-0.1606	-110	310	324	MSGGDHIHAGTVVGK					Mascot
1502.8512	1502.6508	-0.2004	-133	155	167	YGRPLLGCTIKPK			Carbamidomethyl (C)[8]		Mascot
2169.9868	2169.6931	-0.2937	-135	185	203	GGLDFTKDDENVNSQPFMR					Mascot
2185.9819	2185.6792	-0.3027	-138	185	203	GGLDFTKDDENVNSQPFMR			Oxidation (M)[18]		Mascot
3022.4548	3022.0188	-0.436	-144	249	275	ELGVPIVMHDYLTGGFTANTSLAHYCR			Carbamidomethyl (C)[26]		Mascot
3854.8718	3854.3113	-0.5605	-145	32	69	VTPQPGVPPEEAGAAVAAESSTGTWTTVWTDGLTSLDR					Mascot

Rank	Protein Name	Accession No	Protein MW	Protein PI	Pep. Count	Protein Score	Protein Score C. I. %	Total Ion Score	Total Ion C. I. %
6	Ribulose bisphosphate carboxylase large chain (Fragment) OS=Prunus domestica GN=rbcL PE=3 SV=1	RBL_PRUDO	50914.6	6.23	13	96	100	25	67.202

Peptide Information

Calc. Mass	Obsrv. Mass	± da	± ppm	Start Seq.	End Seq.	Sequence	Ion Score	C. I. %	Modification	Rank	Result Type
898.41	898.2976	-0.1124	-125	296	302	NHGMHFR					Mascot
910.4451	910.3295	-0.1156	-127	178	184	AVYECLR			Carbamidomethyl (C)[5]		Mascot
914.4049	914.2867	-0.1182	-129	296	302	NHGMHFR			Oxidation (M)[4]		Mascot
914.4049	914.2867	-0.1182	-129	296	302	NHGMHFR			Oxidation (M)[4]		Mascot
1021.5312	1021.4	-0.1312	-128	23	31	DTDILAAFR					Mascot
1116.583	1116.4341	-0.1489	-133	412	421	VALEACVQAR			Carbamidomethyl (C)[6]		Mascot
1170.5585	1170.4855	-0.073	-62	294	302	QKNHGMHFR			Oxidation (M)[6]		Mascot
1187.6643	1187.5093	-0.155	-131	276	285	DNGLLLHIHR					Mascot
1245.6433	1245.4746	-0.1687	-135	430	440	EGNEIIREASK					Mascot
1275.7307	1275.5624	-0.1683	-132	330	340	EITLGFVDLLR	25	67.202			Mascot
1275.7307	1275.5624	-0.1683	-132	330	340	EITLGFVDLLR					Mascot
1465.7216	1465.561	-0.1606	-110	310	324	MSGGDHIHAGTVVGK					Mascot
1502.8512	1502.6508	-0.2004	-133	155	167	YGRPLLGCTIKPK			Carbamidomethyl (C)[8]		Mascot
2169.9868	2169.6931	-0.2937	-135	185	203	GGLDFTKDDENVNSQPFMR					Mascot
2185.9819	2185.6792	-0.3027	-138	185	203	GGLDFTKDDENVNSQPFMR			Oxidation (M)[18]		Mascot
3022.4548	3022.0188	-0.436	-144	249	275	ELGVPIVMHDYLTGGFTANTSLAHYCR			Carbamidomethyl (C)[26]		Mascot
3854.8718	3854.3113	-0.5605	-145	32	69	VTPQPGVPPEEAGAAVAAESSTGTWTTVWTDGLTSLDR					Mascot

Rank	Protein Name	Accession No	Protein MW	Protein PI	Pep. Count	Protein Score	Protein Score C. I. %	Total Ion Score	Total Ion C. I. %
7	Ribulose bisphosphate carboxylase large chain (Fragment) OS=Cornus obliqua GN=rbcL PE=3 SV=2	RBL_COROB	51536.8	6	13	95	100	25	67.202

Peptide Information

Calc. Mass	Obsrv. Mass	± da	± ppm	Start Seq.	End Seq.	Sequence	Ion Score	C. I. %	Modification	Rank	Result Type
898.41	898.2976	-0.1124	-125	296	302	NHGMHFR					Mascot
910.4451	910.3295	-0.1156	-127	178	184	AVYECLR			Carbamidomethyl (C)[5]		Mascot
914.4049	914.2867	-0.1182	-129	296	302	NHGMHFR			Oxidation (M)[4]		Mascot
914.4049	914.2867	-0.1182	-129	296	302	NHGMHFR			Oxidation (M)[4]		Mascot
1021.5312	1021.4	-0.1312	-128	23	31	DTDILAAFR					Mascot
1116.583	1116.4341	-0.1489	-133	412	421	VALEACVQAR			Carbamidomethyl (C)[6]		Mascot
1170.5585	1170.4855	-0.073	-62	294	302	QKNHGMHFR			Oxidation (M)[6]		Mascot
1187.6643	1187.5093	-0.155	-131	276	285	DNGLLLHIHR					Mascot
1245.6433	1245.4746	-0.1687	-135	430	440	EGNEIIREASK					Mascot
1275.7307	1275.5624	-0.1683	-132	330	340	EITLGFVDLLR	25	67.202			Mascot
1275.7307	1275.5624	-0.1683	-132	330	340	EITLGFVDLLR					Mascot
1465.7546	1465.561	-0.1936	-132	137	149	TFQGPPHGIQVER					Mascot
1502.8512	1502.6508	-0.2004	-133	155	167	YGRPLLGCTIKPK			Carbamidomethyl (C)[8]		Mascot
2169.9868	2169.6931	-0.2937	-135	185	203	GGLDFTKDDENVNSQPFMR					Mascot
2185.9819	2185.6792	-0.3027	-138	185	203	GGLDFTKDDENVNSQPFMR			Oxidation (M)[18]		Mascot
3022.4548	3022.0188	-0.436	-144	249	275	ELGVPIVMHDYLTGGFTANTSLAHYCR			Carbamidomethyl (C)[26]		Mascot
3854.8718	3854.3113	-0.5605	-145	32	69	VTPQPGVPPEEAGAAVAAESSTGTWTTVWTDGLTSLDR					Mascot

Rank	Protein Name	Accession No	Protein MW	Protein PI	Pep. Count	Protein Score	Protein Score C. I. %	Total Ion Score	Total Ion C. I. %
8	Ribulose bisphosphate carboxylase large chain (Fragment) OS=Tamarindus indica GN=rbcL PE=3 SV=1	RBL_TAMIN	50497.5	6.13	13	94	99.999	25	67.202

Peptide Information

Calc. Mass	Obsrv. Mass	± da	± ppm	Start Seq.	End Seq.	Sequence	Ion Score	C. I. %	Modification	Rank	Result Type
898.41	898.2976	-0.1124	-125	297	303	NHGMHFR					Mascot
910.4451	910.3295	-0.1156	-127	179	185	AVYECLR			Carbamidomethyl (C)[5]		Mascot
914.4049	914.2867	-0.1182	-129	297	303	NHGMHFR			Oxidation (M)[4]		Mascot
914.4049	914.2867	-0.1182	-129	297	303	NHGMHFR			Oxidation (M)[4]		Mascot
1021.5312	1021.4	-0.1312	-128	24	32	DTDILAAFR					Mascot
1116.583	1116.4341	-0.1489	-133	413	422	VALEACVQAR			Carbamidomethyl (C)[6]		Mascot
1170.5585	1170.4855	-0.073	-62	295	303	QKNHGMHFR			Oxidation (M)[6]		Mascot
1187.6643	1187.5093	-0.155	-131	277	286	DNGLLLHIHR					Mascot
1245.6433	1245.4746	-0.1687	-135	431	441	EGNEIIREASK					Mascot
1275.7307	1275.5624	-0.1683	-132	331	341	EITLGFVDLLR	25	67.202			Mascot
1275.7307	1275.5624	-0.1683	-132	331	341	EITLGFVDLLR					Mascot
1465.7546	1465.561	-0.1936	-132	138	150	TFQGPPHGIQVER					Mascot
1502.8512	1502.6508	-0.2004	-133	156	168	YGRPLLGCTIKPK			Carbamidomethyl (C)[8]		Mascot
2169.9868	2169.6931	-0.2937	-135	186	204	GGLDFTKDDENVNSQPFMR					Mascot
2185.9819	2185.6792	-0.3027	-138	186	204	GGLDFTKDDENVNSQPFMR			Oxidation (M)[18]		Mascot
2748.3298	2747.9343	-0.3955	-144	219	243	AQAETGEIKGHYLNATAGTWEEMIK					Mascot
3022.4548	3022.0188	-0.436	-144	250	276	ELGVPIVMHDYLTGGFTANTSLAHYCR			Carbamidomethyl (C)[26]		Mascot

Rank	Protein Name	Accession No	Protein MW	Protein PI	Pep. Count	Protein Score	Protein Score C. I. %	Total Ion Score	Total Ion C. I. %
9	Heat shock 70 kDa protein 7, chloroplastic OS=Arabidopsis thaliana GN=HSP70-7 PE=1 SV=1	HSP7G_ARAT	76949.7	5.17	6	94	99.998	82	100

Peptide Information

Calc. Mass	Obsrv. Mass	± da	± ppm	Start Seq.	End Seq.	Sequence	Ion Score	C. I. %	Modification	Rank	Result Type
870.5406	870.4269	-0.1137	-131	236	243	IAGLEVLR					Mascot
870.5406	870.4269	-0.1137	-131	236	243	IAGLEVLR					Mascot
1155.6481	1155.4912	-0.1569	-136	366	375	HIETTLTRGK					Mascot
1170.6589	1170.4855	-0.1734	-148	387	396	VRTPVENSLR					Mascot
1373.7271	1373.5461	-0.181	-132	404	416	DIDEVILVGGSTR	82	100			Mascot
1373.7271	1373.5461	-0.181	-132	404	416	DIDEVILVGGSTR					Mascot
1461.7695	1461.5762	-0.1933	-132	185	197	QFAAEEISAQVLR					Mascot
2437.1841	2436.8403	-0.3438	-141	494	516	SEVFSTAADGQTSVEINVLQGER					Mascot

Rank	Protein Name	Accession No	Protein MW	Protein PI	Pep. Count	Protein Score	Protein Score C. I. %	Total Ion Score	Total Ion C. I. %
10	Heat shock 70 kDa protein 6, chloroplastic OS=Arabidopsis thaliana GN=HSP70-6 PE=1 SV=1	HSP7F_ARAT	76461.4	5.07	6	93	99.998	82	100

Peptide Information

Calc. Mass	Obsrv. Mass	± da	± ppm	Start Seq.	End Seq.	Sequence	Ion Score	C. I. %	Modification	Rank	Result Type
870.5406	870.4269	-0.1137	-131	236	243	IAGLEVLR					Mascot
870.5406	870.4269	-0.1137	-131	236	243	IAGLEVLR					Mascot
1170.6589	1170.4855	-0.1734	-148	387	396	VRTPVENSLR					Mascot
1373.7271	1373.5461	-0.181	-132	404	416	DIDEVILVGGSTR	82	100			Mascot
1373.7271	1373.5461	-0.181	-132	404	416	DIDEVILVGGSTR					Mascot
1461.7695	1461.5762	-0.1933	-132	185	197	QFAAEEISAQVLR					Mascot
1595.7733	1595.559	-0.2143	-134	374	386	AKFEELCSDLLDR			Carbamidomethyl (C)[7]		Mascot
2437.1841	2436.8403	-0.3438	-141	494	516	SEVFSTAADGQTSVEINVLQGER					Mascot

Gel Idx/Pos	36/B9	Instr./Gel Origin	BA2060/131017A	Process Sta	Analysis Succeeded
Plate [#] Name	[1] 31496	Instrument Sample Name		Spectra	8

Rank	Protein Name	Accession No	Protein MW	Protein PI	Pep. Count	Protein Score	Protein Score C. I. %	Total Ion Score	Total Ion C. I. %
1	ATP-dependent zinc metalloprotease FTSH 8, chloroplastic OS=Arabidopsis thaliana GN=FTSH8 PE=1 SV=1	FTSH8_ARAT	73152.9	5.72	9	71	99.72	45	99.706

Peptide Information

Calc. Mass	Obsrv. Mass	± da	± ppm	Start Seq.	End Seq.	Sequence	Ion Score	C. I. %	Modification	Rank	Result Type
962.4425	962.3291	-0.1134	-118	452	459	EIDDSIDR					Mascot
1032.4705	1032.3341	-0.1364	-132	329	339	GTGIGGGNDER					Mascot
1094.6567	1094.5186	-0.1381	-126	255	266	GVLLVGPPGTGK					Mascot
1396.7906	1396.6195	-0.1711	-122	368	380	ADILDSALLRPGR					Mascot
1903.9218	1903.6501	-0.2717	-143	310	326	ENAPCIVFVDEIDAVGR			Carbamidomethyl (C)[5]		Mascot
2103.054	2102.7466	-0.3074	-146	308	326	AKENAPCIVFVDEIDAVGR			Carbamidomethyl (C)[7]		Mascot
2171.0947	2170.8359	-0.2588	-119	1	21	MAASSACLLGNGLSVYTTKQR					Mascot
2171.1455	2170.8359	-0.3096	-143	421	442	TPGFSGADLANLLNEAAILAGR	24	65.918			Mascot
2660.325	2659.9272	-0.3978	-150	476	500	SLVAYHEVGHAICGTLTPGHDAVQK			Carbamidomethyl (C)[13]		Mascot
2660.325	2659.9272	-0.3978	-150	476	500	SLVAYHEVGHAICGTLTPGHDAVQK	22	45.359	Carbamidomethyl (C)[13]		Mascot

Rank	Protein Name	Accession No	Protein MW	Protein PI	Pep. Count	Protein Score	Protein Score C. I. %	Total Ion Score	Total Ion C. I. %
2	ATP-dependent zinc metalloprotease FTSH 2, chloroplastic OS=Oryza sativa subsp. japonica GN=FTSH2 P	FTSH2_ORYS	72492.8	5.54	9	50	63.951	24	65.918

Peptide Information

Calc. Mass	Obsrv. Mass	± da	± ppm	Start Seq.	End Seq.	Sequence	Ion Score	C. I. %	Modification	Rank	Result Type
962.4425	962.3291	-0.1134	-118	446	453	EIDDSIDR					Mascot
1032.4705	1032.3341	-0.1364	-132	323	333	GTGIGGGNDER					Mascot
1094.6567	1094.5186	-0.1381	-126	249	260	GVLLVGPPGTGK					Mascot
1107.5537	1107.4089	-0.1448	-131	1	11	MAPTSMSLAAK					Mascot
1198.6102	1198.4651	-0.1451	-121	84	92	FLEYLDKDR					Mascot
1396.7906	1396.6195	-0.1711	-122	362	374	ADILDSALLRPGR					Mascot
1903.9218	1903.6501	-0.2717	-143	304	320	ENAPCIVFVDEIDAVGR			Carbamidomethyl (C)[5]		Mascot
2103.054	2102.7466	-0.3074	-146	302	320	AKENAPCIVFVDEIDAVGR			Carbamidomethyl (C)[7]		Mascot
2171.1455	2170.8359	-0.3096	-143	415	436	TPGFSGADLANLLNEAAILAGR	24	65.918			Mascot
2171.1455	2170.8359	-0.3096	-143	415	436	TPGFSGADLANLLNEAAILAGR					Mascot

Rank	Protein Name	Accession No	Protein MW	Protein PI	Pep. Count	Protein Score	Protein Score C. I. %	Total Ion Score	Total Ion C. I. %
3	Ubiquinol oxidase 1a, mitochondrial OS=Arabidoı thaliana GN=AOX1A PE=1 SV=2	AOX1A_ARAT	39954.3	8.56	9	42	0		

Peptide Information

Calc. Mass	Obsrv. Mass	± da	± ppm	Start Seq.	End Seq.	Sequence	Ion Score	C. I. %	Modification	Rank	Result Type
856.4886	856.4136	-0.075	-88	307	314	LPADATLR					Mascot
996.4964	996.4716	-0.0248	-25	1	9	MMITRGGAK			Oxidation (M)[1,2]		Mascot
1051.5208	1051.5938	0.073	69	205	213	FEQSGGWIK					Mascot
1594.7791	1594.5774	-0.2017	-126	224	236	MHLMTFMEVAKPK			Oxidation (M)[1,4]		Mascot
1658.767	1658.6141	-0.1529	-92	329	342	DVNHFASDIHYQGR					Mascot
1658.767	1658.6141	-0.1529	-92	329	342	DVNHFASDIHYQGR					Mascot
1821.7845	1821.6149	-0.1696	-93	84	102	TENESTGGDAAGGNNKGDK					Mascot
2332.1355	2331.8186	-0.3169	-136	179	200	AMMLETVAAVPGMVGGMLLHCK			Carbamidomethyl (C)[21], Oxidation (M)[2]		Mascot
2660.28	2659.9272	-0.3528	-133	268	289	MVGYLEEEAIHSYTEFLKELDK			Oxidation (M)[1]		Mascot
2660.28	2659.9272	-0.3528	-133	268	289	MVGYLEEEAIHSYTEFLKELDK			Oxidation (M)[1]		Mascot
2859.3152	2859.0071	-0.3081	-108	175	200	YGCRAMMLETVAAVPGMVGGMLLHCK			Carbamidomethyl (C)[3], Oxidation (M)[6,7,17,21]		Mascot

Rank	Protein Name	Accession No	Protein MW	Protein PI	Pep. Count	Protein Score	Protein Score C. I. %	Total Ion Score	Total Ion C. I. %
4	ATP-dependent zinc metalloprotease FTSH, chloroplastic (Fragments) OS=Populus euphratica PE=1 SV=1	FTSH_POPEU	5194.8	6.29	2	42	0	24	65.918

Peptide Information

Calc. Mass	Obsrv. Mass	± da	± ppm	Start Seq.	End Seq.	Sequence	Ion Score	C. I. %	Modification	Rank	Result Type
1198.6102	1198.4651	-0.1451	-121	1	9	FLEYLDKDR					Mascot
2171.1455	2170.8359	-0.3096	-143	25	46	TPGFSGADLANLLNEAAILAGR	24	65.918			Mascot
2171.1455	2170.8359	-0.3096	-143	25	46	TPGFSGADLANLLNEAAILAGR					Mascot

Rank	Protein Name	Accession No	Protein MW	Protein PI	Pep. Count	Protein Score	Protein Score C. I. %	Total Ion Score	Total Ion C. I. %
5	ATP-dependent zinc metalloprotease FTSH 2, chloroplastic OS=Arabidopsis thaliana GN=FTSH2 PE=1 SV=1	FTSH2_ARAT	74111.4	6	7	40	0	24	65.918

Peptide Information

Calc. Mass	Obsrv. Mass	± da	± ppm	Start Seq.	End Seq.	Sequence	Ion Score	C. I. %	Modification	Rank	Result Type
962.4425	962.3291	-0.1134	-118	459	466	EIDDSIDR					Mascot
1032.4705	1032.3341	-0.1364	-132	336	346	GTGIGGGNDER					Mascot
1198.6102	1198.4651	-0.1451	-121	97	105	FLEYLDKDR					Mascot
1396.7906	1396.6195	-0.1711	-122	375	387	ADILDSALLRPGR					Mascot
1903.9218	1903.6501	-0.2717	-143	317	333	ENAPCIVFVDEIDAVGR			Carbamidomethyl (C)[5]		Mascot
2103.054	2102.7466	-0.3074	-146	315	333	AKENAPCIVFVDEIDAVGR			Carbamidomethyl (C)[7]		Mascot
2171.1455	2170.8359	-0.3096	-143	428	449	TPGFSGADLANLLNEAAILAGR	24	65.918			Mascot
2171.1455	2170.8359	-0.3096	-143	428	449	TPGFSGADLANLLNEAAILAGR					Mascot

Rank	Protein Name	Accession No	Protein MW	Protein PI	Pep. Count	Protein Score	Protein Score C. I. %	Total Ion Score	Total Ion C. I. %
6	Putative E3 ubiquitin-protein ligase SINAT1 OS=Arabidopsis thaliana GN=SINAT1 PE=3 SV=1	SINA1_ARAT	34534.7	7.85	8	32	0		

Peptide Information

Calc. Mass	Obsrv. Mass	± da	± ppm	Start Seq.	End Seq.	Sequence	Ion Score	C. I. %	Modification	Rank	Result Type
870.454	870.4351	-0.0189	-22	260	266	SIRDSHR					Mascot
870.454	870.4351	-0.0189	-22	260	266	SIRDSHR					Mascot
973.5312	973.3942	-0.137	-141	28	36	VEANSKPTK					Mascot
1021.4553	1021.4041	-0.0512	-50	85	93	VQNTCPTCR					Mascot
1107.5476	1107.4089	-0.1387	-125	132	139	LKHEQHCR			Carbamidomethyl (C)[7]		Mascot
1214.6051	1214.536	-0.0691	-57	280	290	NLALYFSGSDK					Mascot
1816.9296	1816.7183	-0.2113	-116	101	116	CLALEKVAESLEVPCR			Carbamidomethyl (C)[1]		Mascot
1821.8159	1821.6149	-0.201	-110	171	185	VDMHDGCTFNHRYVK					Mascot
1838.8418	1838.6709	-0.1709	-93	117	131	YQNLGCQDIFPYYSK					Mascot

Rank	Protein Name	Accession No	Protein MW	Protein PI	Pep. Count	Protein Score	Protein Score C. I. %	Total Ion Score	Total Ion C. I. %
7	Cysteine synthase OS=Oryza sativa subsp. japoni GN=RCS1 PE=2 SV=2	CYSK1_ORYS	33816.7	5.39	7	32	0		

Peptide Information

Calc. Mass	Obsrv. Mass	± da	± ppm	Start Seq.	End Seq.	Sequence	Ion Score	C. I. %	Modification	Rank	Result Type
1141.5959	1141.4812	-0.1147	-100	280	289	LAQRPENEGK					Mascot
1141.5959	1141.4812	-0.1147	-100	280	289	LAQRPENEGK					Mascot
1214.674	1214.536	-0.138	-114	127	138	GAVQKAEELAAK					Mascot
1275.6692	1275.5641	-0.1051	-82	192	201	YLREQNPDIK					Mascot
1275.6692	1275.5641	-0.1051	-82	192	201	YLREQNPDIK					Mascot
1396.7623	1396.6195	-0.1428	-102	290	301	LFVVVFPSFGER					Mascot
1465.7212	1465.5618	-0.1594	-109	93	105	LVLTMPASMSMER					Mascot
1594.7815	1594.5774	-0.2041	-128	32	46	VAAKLESMEPCSSVK			Oxidation (M)[8]		Mascot
1816.9803	1816.7183	-0.262	-144	8	23	DVTELIGNTPLVYLNR					Mascot

Rank	Protein Name	Accession No	Protein MW	Protein PI	Pep. Count	Protein Score	Protein Score C. I. %	Total Ion Score	Total Ion C. I. %
8	Ribulose bisphosphate carboxylase small chain 4, chloroplastic OS=Mesembryanthemum crystallinum GN=	RBS4_MESCR	20299	6.73	5	28	0		

Peptide Information

Calc. Mass	Obsrv. Mass	± da	± ppm	Start Seq.	End Seq.	Sequence	Ion Score	C. I. %	Modification	Rank	Result Type
914.4229	914.2894	-0.1335	-146	124	129	YWTMWK					Mascot
933.5151	933.3799	-0.1352	-145	159	166	IIGFDNVR					Mascot
1094.5992	1094.5186	-0.0806	-74	150	158	KAYPEAFIR					Mascot
1644.8423	1644.609	-0.2333	-142	56	69	VQCMQVWPPLGMKK					Mascot
2859.3699	2859.0071	-0.3628	-127	43	68	NNDITSVASNGGRVQCMQVWPPLGMK			Carbamidomethyl (C)[16]		Mascot

Rank	Protein Name	Accession No	Protein MW	Protein PI	Pep. Count	Protein Score	Protein Score C. I. %	Total Ion Score	Total Ion C. I. %
9	Ubiquinol oxidase 1, mitochondrial OS=Glycine m GN=AOX1 PE=1 SV=1	AOX1_SOYBN	36413.3	8.57	8	28	0		

Peptide Information

Calc. Mass	Obsrv. Mass	± da	± ppm	Start Seq.	End Seq.	Sequence	Ion Score	C. I. %	Modification	Rank	Result Type
882.4542	882.4451	-0.0091	-10	122	128	MAFWTVK					Mascot
933.3483	933.3799	0.0316	34	1	7	MMMMMSR			Oxidation (M)[1]		Mascot
1051.5604	1051.5938	0.0334	32	13	22	VANTAMFVAK					Mascot
1094.5054	1094.5186	0.0132	12	172	180	FEHSGGWFK					Mascot
1275.6538	1275.5641	-0.0897	-70	57	69	VGLSSAGGNKEEK					Mascot
1275.6538	1275.5641	-0.0897	-70	57	69	VGLSSAGGNKEEK					Mascot
1594.7791	1594.5774	-0.2017	-126	191	203	MHLMTFMEVAKPK			Oxidation (M)[1,4]		Mascot
1658.767	1658.6141	-0.1529	-92	296	309	DVNHFASDIHYQGR					Mascot
1658.767	1658.6141	-0.1529	-92	296	309	DVNHFASDIHYQGR					Mascot
2171.1052	2170.8359	-0.2693	-124	103	121	ADLSIDLEKHMPPTTFLDK					Mascot
2171.1052	2170.8359	-0.2693	-124	103	121	ADLSIDLEKHMPPTTFLDK					Mascot

Rank	Protein Name	Accession No	Protein MW	Protein PI	Pep. Count	Protein Score	Protein Score C. I. %	Total Ion Score	Total Ion C. I. %
10	Putative pentatricopeptide repeat-containing prote At3g47840 OS=Arabidopsis thaliana GN=PCMP-E43	PP268_ARAT	78003	8.33	10	27	0		

Peptide Information

Calc. Mass	Obsrv. Mass	± da	± ppm	Start Seq.	End Seq.	Sequence	Ion Score	C. I. %	Modification	Rank	Result Type
848.4042	848.4305	0.0263	31	7	13	NCGTIQR			Carbamidomethyl (C)[2]		Mascot
870.4355	870.4351	-0.0004	0	699	706	AFGVNLYS					Mascot
870.4355	870.4351	-0.0004	0	699	706	AFGVNLYS					Mascot
882.4501	882.4451	-0.005	-6	692	698	FDCTLKR					Mascot
1058.5187	1058.4237	-0.095	-90	450	458	SSLINMYSK			Oxidation (M)[6]		Mascot
1094.6027	1094.5186	-0.0841	-77	294	302	AVETFIKMR					Mascot
1198.5594	1198.4651	-0.0943	-79	570	578	MINEMSWKK			Oxidation (M)[1,5]		Mascot
1396.689	1396.6195	-0.0695	-50	656	668	IKDCVSAFVSGDR					Mascot
1587.7869	1587.6292	-0.1577	-99	450	463	SSLINMYSKCGSIK			Carbamidomethyl (C)[10]		Mascot
1587.7869	1587.6292	-0.1577	-99	450	463	SSLINMYSKCGSIK			Carbamidomethyl (C)[10]		Mascot
1816.8203	1816.7183	-0.102	-56	459	474	CGSIKEASMIFGETDR			Carbamidomethyl (C)[1], Oxidation (M)[9]		Mascot
2171.0913	2170.8359	-0.2554	-118	80	98	RYVTANNSDEALILFSAMR					Mascot
2171.0913	2170.8359	-0.2554	-118	80	98	RYVTANNSDEALILFSAMR					Mascot

Gel Idx/Pos	37/B10	Instr./Gel Origin	BA2060/131017A	Process Sta	Analysis Succeeded
Plate [#] Name	[1] 31496	Instrument Sample Name		Spectra	11

Rank	Protein Name	Accession No	Protein MW	Protein PI	Pep. Count	Protein Score	Protein Score C. I. %	Total Ion Score	Total Ion C. I. %
1	Ribulose bisphosphate carboxylase large chain OS=Phaseolus vulgaris GN=rbcL PE=3 SV=1	RBL_PHAVU	52767.6	5.96	13	171	100	102	100

Peptide Information

Calc. Mass	Obsrv. Mass	± da	± ppm	Start Seq.	End Seq.	Sequence	Ion Score	C. I. %	Modification	Rank	Result Type
910.4451	910.341	-0.1041	-114	188	194	AVYECLR			Carbamidomethyl (C)[5]		Mascot
914.4049	914.2946	-0.1103	-121	306	312	NHGMHFR			Oxidation (M)[4]		Mascot
914.4049	914.2946	-0.1103	-121	306	312	NHGMHFR			Oxidation (M)[4]		Mascot
921.4346	921.3848	-0.0498	-54	1	8	MSPQTETK					Mascot
1021.5312	1021.4078	-0.1234	-121	33	41	DTDILAAFR					Mascot
1170.5585	1170.5005	-0.058	-50	304	312	QKNHGMHFR			Oxidation (M)[6]		Mascot
1187.6643	1187.495	-0.1693	-143	286	295	DNGLLLHIHR					Mascot
1245.6433	1245.4811	-0.1622	-130	440	450	EGNEIIREASK					Mascot
1275.7307	1275.5682	-0.1625	-127	340	350	EITLGFVDLLR	42	99.222			Mascot
1275.7307	1275.5682	-0.1625	-127	340	350	EITLGFVDLLR					Mascot
1465.7546	1465.5735	-0.1811	-124	147	159	TFQGPPHGIQVER					Mascot
1465.7546	1465.5735	-0.1811	-124	147	159	TFQGPPHGIQVER	25	59.532			Mascot
1546.7358	1546.5408	-0.195	-126	451	463	WSPELAAACEVWK			Carbamidomethyl (C)[9]		Mascot
1587.7247	1587.6307	-0.094	-59	464	476	EIKFEFEAMDTLD					Mascot
1587.7247	1587.6307	-0.094	-59	464	476	EIKFEFEAMDTLD					Mascot
2185.9819	2185.6965	-0.2854	-131	195	213	GGLDFTKDDENVNSQPFMR			Oxidation (M)[18]		Mascot
3854.8718	3854.3477	-0.5241	-136	42	79	VTPQPGVPPEEAGAAVAAESSTGTWTTVWTDGLTSLDR	35	95.603			Mascot
3854.8718	3854.3477	-0.5241	-136	42	79	VTPQPGVPPEEAGAAVAAESSTGTWTTVWTDGLTSLDR					Mascot

Rank	Protein Name	Accession No	Protein MW	Protein PI	Pep. Count	Protein Score	Protein Score C. I. %	Total Ion Score	Total Ion C. I. %
2	Ribulose bisphosphate carboxylase large chain OS=Liquidambar styraciflua GN=rbcL PE=3 SV=1	RBL_LIQST	52625.5	6.04	13	168	100	101	100

Peptide Information

Calc. Mass	Obsrv. Mass	± da	± ppm	Start Seq.	End Seq.	Sequence	Ion Score	C. I. %	Modification	Rank	Result Type
910.4451	910.341	-0.1041	-114	188	194	AVYECLR			Carbamidomethyl (C)[5]		Mascot
914.4049	914.2946	-0.1103	-121	306	312	NHGMHFR			Oxidation (M)[4]		Mascot
914.4049	914.2946	-0.1103	-121	306	312	NHGMHFR			Oxidation (M)[4]		Mascot
921.4346	921.3848	-0.0498	-54	1	8	MSPQTETK					Mascot
1021.5312	1021.4078	-0.1234	-121	33	41	DTDILAAFR					Mascot
1170.5585	1170.5005	-0.058	-50	304	312	QKNHGMHFR			Oxidation (M)[6]		Mascot
1187.6643	1187.495	-0.1693	-143	286	295	DNGLLLHIHR					Mascot
1245.6433	1245.4811	-0.1622	-130	440	450	EGNEIIREASK					Mascot
1275.7307	1275.5682	-0.1625	-127	340	350	EITLGFVDLLR	42	99.222			Mascot
1275.7307	1275.5682	-0.1625	-127	340	350	EITLGFVDLLR					Mascot
1465.7216	1465.5735	-0.1481	-101	320	334	MSGGDHIHAGTVVGK					Mascot
1465.7546	1465.5735	-0.1811	-124	147	159	TFQGPPHGIQVER	25	59.532			Mascot
1546.7358	1546.5408	-0.195	-126	451	463	WSPELAAACEVWK			Carbamidomethyl (C)[9]		Mascot
2185.9819	2185.6965	-0.2854	-131	195	213	GGLDFTKDDENVNSQPFMR			Oxidation (M)[18]		Mascot
3854.8718	3854.3477	-0.5241	-136	42	79	VTPQPGVPPEEAGAAVAAESSTGTWTTVWTDGLTSLDR	35	95.603			Mascot
3854.8718	3854.3477	-0.5241	-136	42	79	VTPQPGVPPEEAGAAVAAESSTGTWTTVWTDGLTSLDR					Mascot

Rank	Protein Name	Accession No	Protein MW	Protein PI	Pep. Count	Protein Score	Protein Score C. I. %	Total Ion Score	Total Ion C. I. %
3	Ribulose bisphosphate carboxylase large chain OS=Cucumis sativus GN=rbcL PE=1 SV=5	RBL_CUCSA	52608.4	6	13	165	100	101	100

Peptide Information

Calc. Mass	Obsrv. Mass	± da	± ppm	Start Seq.	End Seq.	Sequence	Ion Score	C. I. %	Modification	Rank	Result Type
910.4451	910.341	-0.1041	-114	188	194	AVYECLR			Carbamidomethyl (C)[5]		Mascot
914.4049	914.2946	-0.1103	-121	306	312	NHGMHFR			Oxidation (M)[4]		Mascot
914.4049	914.2946	-0.1103	-121	306	312	NHGMHFR			Oxidation (M)[4]		Mascot
921.4346	921.3848	-0.0498	-54	1	8	MSPQTETK					Mascot
1021.5312	1021.4078	-0.1234	-121	33	41	DTDILAAFR					Mascot
1170.5585	1170.5005	-0.058	-50	304	312	QKNHGMHFR			Oxidation (M)[6]		Mascot
1187.6643	1187.495	-0.1693	-143	286	295	DNGLLLHIHR					Mascot
1245.6337	1245.4811	-0.1526	-123	218	227	FLFCAEAIFK			Carbamidomethyl (C)[4]		Mascot
1275.7307	1275.5682	-0.1625	-127	340	350	EITLGFVDLLR	42	99.222			Mascot
1275.7307	1275.5682	-0.1625	-127	340	350	EITLGFVDLLR					Mascot
1465.7216	1465.5735	-0.1481	-101	320	334	MSGGDHIHAGTVVGK					Mascot
1465.7546	1465.5735	-0.1811	-124	147	159	TFQGPPHGIQVER	25	59.532			Mascot
1546.7358	1546.5408	-0.195	-126	451	463	WSPELAAACEVWK			Carbamidomethyl (C)[9]		Mascot
2185.9819	2185.6965	-0.2854	-131	195	213	GGLDFTKDDENVNSQPFMR			Oxidation (M)[18]		Mascot
3854.8718	3854.3477	-0.5241	-136	42	79	VTPQPGVPPEEAGAAVAAESSTGTWTTVWTDGLTSLDR	35	95.603			Mascot
3854.8718	3854.3477	-0.5241	-136	42	79	VTPQPGVPPEEAGAAVAAESSTGTWTTVWTDGLTSLDR					Mascot

Rank	Protein Name	Accession No	Protein MW	Protein PI	Pep. Count	Protein Score	Protein Score C. I. %	Total Ion Score	Total Ion C. I. %
4	Ribulose bisphosphate carboxylase large chain (Fragment) OS=Sinapis alba GN=rbcL PE=3 SV=1	RBL_SINAL	52405.4	6.23	12	163	100	102	100

Peptide Information

Calc. Mass	Obsrv. Mass	± da	± ppm	Start Seq.	End Seq.	Sequence	Ion Score	C. I. %	Modification	Rank	Result Type
910.4451	910.341	-0.1041	-114	188	194	AVYECLR			Carbamidomethyl (C)[5]		Mascot
914.4049	914.2946	-0.1103	-121	306	312	NHGMHFR			Oxidation (M)[4]		Mascot
914.4049	914.2946	-0.1103	-121	306	312	NHGMHFR			Oxidation (M)[4]		Mascot
921.4346	921.3848	-0.0498	-54	1	8	MSPQTETK					Mascot
1021.5312	1021.4078	-0.1234	-121	33	41	DTDILAAFR					Mascot
1170.5585	1170.5005	-0.058	-50	304	312	QKNHGMHFR			Oxidation (M)[6]		Mascot
1187.6643	1187.495	-0.1693	-143	286	295	DNGLLLHIHR					Mascot
1245.6433	1245.4811	-0.1622	-130	440	450	EGNEIIREASK					Mascot
1275.7307	1275.5682	-0.1625	-127	340	350	EITLGFVDLLR	42	99.222			Mascot
1275.7307	1275.5682	-0.1625	-127	340	350	EITLGFVDLLR					Mascot
1465.7546	1465.5735	-0.1811	-124	147	159	TFQGPPHGIQVER					Mascot
1465.7546	1465.5735	-0.1811	-124	147	159	TFQGPPHGIQVER	25	59.532			Mascot
1546.7358	1546.5408	-0.195	-126	451	463	WSPELAAACEVWK			Carbamidomethyl (C)[9]		Mascot
2185.9819	2185.6965	-0.2854	-131	195	213	GGLDFTKDDENVNSQPFMR			Oxidation (M)[18]		Mascot
3854.8718	3854.3477	-0.5241	-136	42	79	VTPQPGVPPEEAGAAVAAESSTGTWTTVWTDGLTSLDR	35	95.603			Mascot
3854.8718	3854.3477	-0.5241	-136	42	79	VTPQPGVPPEEAGAAVAAESSTGTWTTVWTDGLTSLDR					Mascot

Rank	Protein Name	Accession No	Protein MW	Protein PI	Pep. Count	Protein Score	Protein Score C. I. %	Total Ion Score	Total Ion C. I. %
5	Ribulose bisphosphate carboxylase large chain OS=Ipomoea purpurea GN=rbcL PE=3 SV=1	RBL_IPOPU	53353.9	6.41	12	162	100	102	100

Peptide Information

Calc. Mass	Obsrv. Mass	± da	± ppm	Start Seq.	End Seq.	Sequence	Ion Score	C. I. %	Modification	Rank	Result Type
910.4451	910.341	-0.1041	-114	188	194	AVYECLR			Carbamidomethyl (C)[5]		Mascot
914.4049	914.2946	-0.1103	-121	306	312	NHGMHFR			Oxidation (M)[4]		Mascot
914.4049	914.2946	-0.1103	-121	306	312	NHGMHFR			Oxidation (M)[4]		Mascot
921.4346	921.3848	-0.0498	-54	1	8	MSPQTETK					Mascot
1021.5312	1021.4078	-0.1234	-121	33	41	DTDILAAFR					Mascot
1170.5585	1170.5005	-0.058	-50	304	312	QKNHGMHFR			Oxidation (M)[6]		Mascot
1187.6643	1187.495	-0.1693	-143	286	295	DNGLLLHIHR					Mascot
1275.7307	1275.5682	-0.1625	-127	340	350	EITLGFVDLLR	42	99.222			Mascot
1275.7307	1275.5682	-0.1625	-127	340	350	EITLGFVDLLR					Mascot
1465.7546	1465.5735	-0.1811	-124	147	159	TFQGPPHGIQVER					Mascot
1465.7546	1465.5735	-0.1811	-124	147	159	TFQGPPHGIQVER	25	59.532			Mascot
1546.7358	1546.5408	-0.195	-126	451	463	WSPELAAACEVWK			Carbamidomethyl (C)[9]		Mascot
1593.7432	1593.625	-0.1182	-74	467	480	FEFKPVDTLDPDGN					Mascot
2185.9819	2185.6965	-0.2854	-131	195	213	GGLDFTKDDENVNSQPFMR			Oxidation (M)[18]		Mascot
3854.8718	3854.3477	-0.5241	-136	42	79	VTPQPGVPPEEAGAAVAAESSTGTWTTVWTDGLTSLDR	35	95.603			Mascot
3854.8718	3854.3477	-0.5241	-136	42	79	VTPQPGVPPEEAGAAVAAESSTGTWTTVWTDGLTSLDR					Mascot

Rank	Protein Name	Accession No	Protein MW	Protein PI	Pep. Count	Protein Score	Protein Score C. I. %	Total Ion Score	Total Ion C. I. %
6	Ribulose bisphosphate carboxylase large chain (Fragment) OS=Geum quellyon GN=rbcL PE=3 SV=1	RBL_GEUQU	50910.8	6.23	12	161	100	101	100

Peptide Information

Calc. Mass	Obsrv. Mass	± da	± ppm	Start Seq.	End Seq.	Sequence	Ion Score	C. I. %	Modification	Rank	Result Type
910.4451	910.341	-0.1041	-114	178	184	AVYECLR			Carbamidomethyl (C)[5]		Mascot
914.4049	914.2946	-0.1103	-121	296	302	NHGMHFR			Oxidation (M)[4]		Mascot
914.4049	914.2946	-0.1103	-121	296	302	NHGMHFR			Oxidation (M)[4]		Mascot
1021.5312	1021.4078	-0.1234	-121	23	31	DTDILAAFR					Mascot
1170.5585	1170.5005	-0.058	-50	294	302	QKNHGMHFR			Oxidation (M)[6]		Mascot
1187.6643	1187.495	-0.1693	-143	276	285	DNGLLLHIHR					Mascot
1245.6337	1245.4811	-0.1526	-123	208	217	FLFCAEALFK			Carbamidomethyl (C)[4]		Mascot
1275.7307	1275.5682	-0.1625	-127	330	340	EITLGFVDLLR	42	99.222			Mascot
1275.7307	1275.5682	-0.1625	-127	330	340	EITLGFVDLLR					Mascot
1465.7216	1465.5735	-0.1481	-101	310	324	MSGGDHIHAGTVVGK					Mascot
1465.7546	1465.5735	-0.1811	-124	137	149	TFQGPPHGIQVER	25	59.532			Mascot
1546.7358	1546.5408	-0.195	-126	441	453	WSPELAAACEVWK			Carbamidomethyl (C)[9]		Mascot
2185.9819	2185.6965	-0.2854	-131	185	203	GGLDFTKDDENVNSQPFMR			Oxidation (M)[18]		Mascot
3854.8718	3854.3477	-0.5241	-136	32	69	VTPQPGVPPEEAGAAVAAESSTGTWTTVWTDGLTSLDR	35	95.603			Mascot
3854.8718	3854.3477	-0.5241	-136	32	69	VTPQPGVPPEEAGAAVAAESSTGTWTTVWTDGLTSLDR					Mascot

Rank	Protein Name	Accession No	Protein MW	Protein PI	Pep. Count	Protein Score	Protein Score C. I. %	Total Ion Score	Total Ion C. I. %
7	Ribulose bisphosphate carboxylase large chain (Fragment) OS=Prunus domestica GN=rbcL PE=3 SV=1	RBL_PRUDO	50914.6	6.23	12	161	100	101	100

Peptide Information

Calc. Mass	Obsrv. Mass	± da	± ppm	Start Seq.	End Seq.	Sequence	Ion Score	C. I. %	Modification	Rank	Result Type
910.4451	910.341	-0.1041	-114	178	184	AVYECLR			Carbamidomethyl (C)[5]		Mascot
914.4049	914.2946	-0.1103	-121	296	302	NHGMHFR			Oxidation (M)[4]		Mascot
914.4049	914.2946	-0.1103	-121	296	302	NHGMHFR			Oxidation (M)[4]		Mascot
1021.5312	1021.4078	-0.1234	-121	23	31	DTDILAAFR					Mascot
1170.5585	1170.5005	-0.058	-50	294	302	QKNHGMHFR			Oxidation (M)[6]		Mascot
1187.6643	1187.495	-0.1693	-143	276	285	DNGLLLHIHR					Mascot
1245.6433	1245.4811	-0.1622	-130	430	440	EGNEIIREASK					Mascot
1275.7307	1275.5682	-0.1625	-127	330	340	EITLGFVDLLR	42	99.222			Mascot
1275.7307	1275.5682	-0.1625	-127	330	340	EITLGFVDLLR					Mascot
1465.7216	1465.5735	-0.1481	-101	310	324	MSGGDHIHAGTVVGK					Mascot
1465.7546	1465.5735	-0.1811	-124	137	149	TFQGPPHGIQVER	25	59.532			Mascot
1546.7358	1546.5408	-0.195	-126	441	453	WSPELAAACEVWK			Carbamidomethyl (C)[9]		Mascot
2185.9819	2185.6965	-0.2854	-131	185	203	GGLDFTKDDENVNSQPFMR			Oxidation (M)[18]		Mascot
3854.8718	3854.3477	-0.5241	-136	32	69	VTPQPGVPPEEAGAAVAAESSTGTWTTVWTDGLTSLDR	35	95.603			Mascot
3854.8718	3854.3477	-0.5241	-136	32	69	VTPQPGVPPEEAGAAVAAESSTGTWTTVWTDGLTSLDR					Mascot

Rank	Protein Name	Accession No	Protein MW	Protein PI	Pep. Count	Protein Score	Protein Score C. I. %	Total Ion Score	Total Ion C. I. %
8	Ribulose bisphosphate carboxylase large chain OS=Vitis vinifera GN=rbcL PE=3 SV=2	RBL_VITVI	52485.5	6.33	12	161	100	102	100

Peptide Information

Calc. Mass	Obsrv. Mass	± da	± ppm	Start Seq.	End Seq.	Sequence	Ion Score	C. I. %	Modification	Rank	Result Type
910.4451	910.341	-0.1041	-114	188	194	AVYECLR			Carbamidomethyl (C)[5]		Mascot
914.4049	914.2946	-0.1103	-121	306	312	NHGMHFR			Oxidation (M)[4]		Mascot
914.4049	914.2946	-0.1103	-121	306	312	NHGMHFR			Oxidation (M)[4]		Mascot
921.4346	921.3848	-0.0498	-54	1	8	MSPQTETK					Mascot
1170.5585	1170.5005	-0.058	-50	304	312	QKNHGMHFR			Oxidation (M)[6]		Mascot
1187.6378	1187.495	-0.1428	-120	440	450	EGNEIIRAASK					Mascot
1245.6337	1245.4811	-0.1526	-123	218	227	FLFCAEAIFK			Carbamidomethyl (C)[4]		Mascot
1275.7307	1275.5682	-0.1625	-127	340	350	EITLGFVDLLR	42	99.222			Mascot
1275.7307	1275.5682	-0.1625	-127	340	350	EITLGFVDLLR					Mascot
1465.7546	1465.5735	-0.1811	-124	147	159	TFQGPPHGIQVER					Mascot
1465.7546	1465.5735	-0.1811	-124	147	159	TFQGPPHGIQVER	25	59.532			Mascot
1546.7358	1546.5408	-0.195	-126	451	463	WSPELAAACEVWK			Carbamidomethyl (C)[9]		Mascot
1903.937	1903.6753	-0.2617	-137	447	463	AASKWSPELAAACEVWK			Carbamidomethyl (C)[13]		Mascot
1903.937	1903.6753	-0.2617	-137	447	463	AASKWSPELAAACEVWK			Carbamidomethyl (C)[13]		Mascot
2185.9819	2185.6965	-0.2854	-131	195	213	GGLDFTKDDENVNSQPFMR			Oxidation (M)[18]		Mascot
3854.8718	3854.3477	-0.5241	-136	42	79	VTPQPGVPPEEAGAAVAAESSTGTWTTVWTDGLTSLDR	35	95.603			Mascot
3854.8718	3854.3477	-0.5241	-136	42	79	VTPQPGVPPEEAGAAVAAESSTGTWTTVWTDGLTSLDR					Mascot

Rank	Protein Name	Accession No	Protein MW	Protein PI	Pep. Count	Protein Score	Protein Score C. I. %	Total Ion Score	Total Ion C. I. %
9	Ribulose bisphosphate carboxylase large chain OS=Cajanus cajan GN=rbcL PE=3 SV=1	RBL_CAJCA	52358.5	6.45	12	161	100	102	100

Peptide Information

Calc. Mass	Obsrv. Mass	± da	± ppm	Start Seq.	End Seq.	Sequence	Ion Score	C. I. %	Modification	Rank	Result Type
910.4451	910.341	-0.1041	-114	188	194	AVYECLR			Carbamidomethyl (C)[5]		Mascot
914.4049	914.2946	-0.1103	-121	306	312	NHGMHFR			Oxidation (M)[4]		Mascot
914.4049	914.2946	-0.1103	-121	306	312	NHGMHFR			Oxidation (M)[4]		Mascot
921.4346	921.3848	-0.0498	-54	1	8	MSPQTETK					Mascot
1021.5312	1021.4078	-0.1234	-121	33	41	DTDILAAFR					Mascot
1170.5585	1170.5005	-0.058	-50	304	312	QKNHGMHFR			Oxidation (M)[6]		Mascot
1187.6643	1187.495	-0.1693	-143	286	295	DNGLLLHIHR					Mascot
1245.6337	1245.4811	-0.1526	-123	218	227	FLFCAEALFK			Carbamidomethyl (C)[4]		Mascot
1275.7307	1275.5682	-0.1625	-127	340	350	EITLGFVDLLR	42	99.222			Mascot
1275.7307	1275.5682	-0.1625	-127	340	350	EITLGFVDLLR					Mascot
1465.7546	1465.5735	-0.1811	-124	147	159	TFQGPPHGIQVER					Mascot
1465.7546	1465.5735	-0.1811	-124	147	159	TFQGPPHGIQVER	25	59.532			Mascot
1546.7358	1546.5408	-0.195	-126	451	463	WSPELAAACEVWK			Carbamidomethyl (C)[9]		Mascot
2185.9819	2185.6965	-0.2854	-131	195	213	GGLDFTKDDENVNSQPFMR			Oxidation (M)[18]		Mascot
3854.8718	3854.3477	-0.5241	-136	42	79	VTPQPGVPPEEAGAAVAAESSTGTWTTVWTDGLTSLDR	35	95.603			Mascot
3854.8718	3854.3477	-0.5241	-136	42	79	VTPQPGVPPEEAGAAVAAESSTGTWTTVWTDGLTSLDR					Mascot

Rank	Protein Name	Accession No	Protein MW	Protein PI	Pep. Count	Protein Score	Protein Score C. I. %	Total Ion Score	Total Ion C. I. %
10	Ribulose bisphosphate carboxylase large chain OS=Sesbania sesban GN=rbcL PE=3 SV=1	RBL_SESSE	52358.4	6.09	12	161	100	102	100

Peptide Information

Calc. Mass	Obsrv. Mass	± da	± ppm	Start Seq.	End Seq.	Sequence	Ion Score	C. I. %	Modification	Rank	Result Type
910.4451	910.341	-0.1041	-114	188	194	AVYECLR			Carbamidomethyl (C)[5]		Mascot
914.4049	914.2946	-0.1103	-121	306	312	NHGMHFR			Oxidation (M)[4]		Mascot
914.4049	914.2946	-0.1103	-121	306	312	NHGMHFR			Oxidation (M)[4]		Mascot
921.4346	921.3848	-0.0498	-54	1	8	MSPQTETK					Mascot
1021.5312	1021.4078	-0.1234	-121	33	41	DTDILAAFR					Mascot
1170.5585	1170.5005	-0.058	-50	304	312	QKNHGMHFR			Oxidation (M)[6]		Mascot
1187.6643	1187.495	-0.1693	-143	286	295	DNGLLLHIHR					Mascot
1245.6337	1245.4811	-0.1526	-123	218	227	FLFCAEAIFK			Carbamidomethyl (C)[4]		Mascot
1275.7307	1275.5682	-0.1625	-127	340	350	EITLGFVDLLR	42	99.222			Mascot
1275.7307	1275.5682	-0.1625	-127	340	350	EITLGFVDLLR					Mascot
1465.7546	1465.5735	-0.1811	-124	147	159	TFQGPPHGIQVER					Mascot
1465.7546	1465.5735	-0.1811	-124	147	159	TFQGPPHGIQVER	25	59.532			Mascot
1546.7358	1546.5408	-0.195	-126	451	463	WSPELAAACEVWK			Carbamidomethyl (C)[9]		Mascot
2185.9819	2185.6965	-0.2854	-131	195	213	GGLDFTKDDENVNSQPFMR			Oxidation (M)[18]		Mascot
3854.8718	3854.3477	-0.5241	-136	42	79	VTPQPGVPPEEAGAAVAAESSTGTWTTVWTDGLTSLDR	35	95.603			Mascot
3854.8718	3854.3477	-0.5241	-136	42	79	VTPQPGVPPEEAGAAVAAESSTGTWTTVWTDGLTSLDR					Mascot

Gel Idx/Pos	38/B11	Instr./Gel Origin	BA2060/131017A	Process Sta	Analysis Succeeded
Plate [#] Name	[1] 31496	Instrument Sample Name		Spectra	11

Rank	Protein Name	Accession No	Protein MW	Protein PI	Pep. Count	Protein Score	Protein Score C. I. %	Total Ion Score	Total Ion C. I. %
1	Ribulose bisphosphate carboxylase large chain OS=Sesbania sesban GN=rbcL PE=3 SV=1	RBL_SESSE	52358.4	6.09	16	170	100	73	100

Peptide Information

Calc. Mass	Obsrv. Mass	± da	± ppm	Start Seq.	End Seq.	Sequence	Ion Score	C. I. %	Modification	Rank	Result Type
856.3546	856.426	0.0714	83	467	473	FEFPAMD					Mascot
910.4451	910.3304	-0.1147	-126	188	194	AVYECLR			Carbamidomethyl (C)[5]		Mascot
914.4049	914.2973	-0.1076	-118	306	312	NHGMHFR			Oxidation (M)[4]		Mascot
914.4049	914.2973	-0.1076	-118	306	312	NHGMHFR			Oxidation (M)[4]		Mascot
921.4346	921.3987	-0.0359	-39	1	8	MSPQTETK					Mascot
1021.5312	1021.409	-0.1222	-120	33	41	DTDILAAFR					Mascot
1039.4691	1039.4015	-0.0676	-65	351	358	DDYVEKDR					Mascot
1116.583	1116.4622	-0.1208	-108	422	431	VALEACVQAR			Carbamidomethyl (C)[6]		Mascot
1187.6643	1187.4926	-0.1717	-145	286	295	DNGLLLHIHR					Mascot
1245.6337	1245.4839	-0.1498	-120	218	227	FLFCAEAIFK			Carbamidomethyl (C)[4]		Mascot
1275.7307	1275.5717	-0.159	-125	340	350	EITLGFVDLLR					Mascot
1275.7307	1275.5717	-0.159	-125	340	350	EITLGFVDLLR	28	79.474			Mascot
1393.6522	1393.4941	-0.1581	-113	22	32	LTYYTPDYETK					Mascot
1465.7546	1465.5778	-0.1768	-121	147	159	TFQGPPHGIQVER	22	20.144			Mascot
1465.7546	1465.5778	-0.1768	-121	147	159	TFQGPPHGIQVER					Mascot
1546.7358	1546.5294	-0.2064	-133	451	463	WSPELAAACEVWK			Carbamidomethyl (C)[9]		Mascot
1794.8149	1794.6844	-0.1305	-73	237	252	GHYLNATAGTCEEMIK			Carbamidomethyl (C)[11]		Mascot
2169.9868	2169.7053	-0.2815	-130	195	213	GGLDFTKDDENVNSQPFMR					Mascot
2185.9819	2185.6948	-0.2871	-131	195	213	GGLDFTKDDENVNSQPFMR			Oxidation (M)[18]		Mascot
3854.8718	3854.314	-0.5578	-145	42	79	VTPQPGVPPEEAGAAVAAESSTGTWTTVWTDGLTSLDR	23	46.506			Mascot
3854.8718	3854.314	-0.5578	-145	42	79	VTPQPGVPPEEAGAAVAAESSTGTWTTVWTDGLTSLDR					Mascot

2	Ribulose bisphosphate carboxylase large chain (Fragment) OS=Manilkara zapota GN=rbcL PE=3 SV=2	RBL_MANZA	51550	6.04	15	161	100	73	100

Peptide Information

Calc. Mass	Obsrv. Mass	± da	± ppm	Start Seq.	End Seq.	Sequence	Ion Score	C. I. %	Modification	Rank	Result Type
910.4451	910.3304	-0.1147	-126	178	184	AVYECLR			Carbamidomethyl (C)[5]		Mascot
914.4049	914.2973	-0.1076	-118	296	302	NHGMHFR			Oxidation (M)[4]		Mascot
914.4049	914.2973	-0.1076	-118	296	302	NHGMHFR			Oxidation (M)[4]		Mascot
1021.5312	1021.409	-0.1222	-120	23	31	DTDILAAFR					Mascot
1039.4691	1039.4015	-0.0676	-65	341	348	DDYVEKDR					Mascot
1116.583	1116.4622	-0.1208	-108	412	421	VALEACVQAR			Carbamidomethyl (C)[6]		Mascot
1187.6643	1187.4926	-0.1717	-145	276	285	DNGLLLHIHR					Mascot
1245.6433	1245.4839	-0.1594	-128	430	440	EGNEIIREASK					Mascot
1275.7307	1275.5717	-0.159	-125	330	340	EITLGFVDLLR					Mascot
1275.7307	1275.5717	-0.159	-125	330	340	EITLGFVDLLR	28	79.474			Mascot
1393.6522	1393.4941	-0.1581	-113	12	22	LTYYTPDYETK					Mascot
1433.7495	1433.5691	-0.1804	-126	310	324	LSGGDHVHAGTVVGK					Mascot
1465.7546	1465.5778	-0.1768	-121	137	149	TFQGPPHGIQVER	22	20.144			Mascot
1465.7546	1465.5778	-0.1768	-121	137	149	TFQGPPHGIQVER					Mascot
1546.7358	1546.5294	-0.2064	-133	441	453	WSPELAAACEVWK			Carbamidomethyl (C)[9]		Mascot
1794.8149	1794.6844	-0.1305	-73	227	242	GHYLNATAGTCEEMIK			Carbamidomethyl (C)[11]		Mascot
2169.9868	2169.7053	-0.2815	-130	185	203	GGLDFTKDDENVNSQPFMR					Mascot
2185.9819	2185.6948	-0.2871	-131	185	203	GGLDFTKDDENVNSQPFMR			Oxidation (M)[18]		Mascot
3854.8718	3854.314	-0.5578	-145	32	69	VTPQPGVPPEEAGAAVAAESSTGTWTTVWTDGLTSLDR	23	46.506			Mascot
3854.8718	3854.314	-0.5578	-145	32	69	VTPQPGVPPEEAGAAVAAESSTGTWTTVWTDGLTSLDR					Mascot

3	Ribulose bisphosphate carboxylase large chain OS=Phaseolus vulgaris GN=rbcL PE=3 SV=1	RBL_PHAVU	52767.6	5.96	15	159	100	73	100

Peptide Information

Calc. Mass	Obsrv. Mass	± da	± ppm	Start Seq.	End Seq.	Sequence	Ion Score	C. I. %	Modification	Rank	Result Type
910.4451	910.3304	-0.1147	-126	188	194	AVYECLR			Carbamidomethyl (C)[5]		Mascot
914.4049	914.2973	-0.1076	-118	306	312	NHGMHFR			Oxidation (M)[4]		Mascot
914.4049	914.2973	-0.1076	-118	306	312	NHGMHFR			Oxidation (M)[4]		Mascot
921.4346	921.3987	-0.0359	-39	1	8	MSPQTETK					Mascot
1021.5312	1021.409	-0.1222	-120	33	41	DTDILAAFR					Mascot
1116.583	1116.4622	-0.1208	-108	422	431	VALEACVQAR			Carbamidomethyl (C)[6]		Mascot
1187.6643	1187.4926	-0.1717	-145	286	295	DNGLLLHIHR					Mascot
1245.6433	1245.4839	-0.1594	-128	440	450	EGNEIIREASK					Mascot
1275.7307	1275.5717	-0.159	-125	340	350	EITLGFVDLLR					Mascot
1275.7307	1275.5717	-0.159	-125	340	350	EITLGFVDLLR	28	79.474			Mascot
1393.6522	1393.4941	-0.1581	-113	22	32	LTYYTPDYETK					Mascot
1465.7546	1465.5778	-0.1768	-121	147	159	TFQGPPHGIQVER	22	20.144			Mascot
1465.7546	1465.5778	-0.1768	-121	147	159	TFQGPPHGIQVER					Mascot
1475.7351	1475.5702	-0.1649	-112	216	227	DRFLFCAEAIYK					Mascot
1546.7358	1546.5294	-0.2064	-133	451	463	WSPELAAACEVWK			Carbamidomethyl (C)[9]		Mascot
1794.8149	1794.6844	-0.1305	-73	237	252	GHYLNATAGTCEEMIK			Carbamidomethyl (C)[11]		Mascot
2169.9868	2169.7053	-0.2815	-130	195	213	GGLDFTKDDENVNSQPFMR					Mascot
2185.9819	2185.6948	-0.2871	-131	195	213	GGLDFTKDDENVNSQPFMR			Oxidation (M)[18]		Mascot
3854.8718	3854.314	-0.5578	-145	42	79	VTPQPGVPPEEAGAAVAAESSTGTWTTVWTDGLTSLDR	23	46.506			Mascot
3854.8718	3854.314	-0.5578	-145	42	79	VTPQPGVPPEEAGAAVAAESSTGTWTTVWTDGLTSLDR					Mascot

4	Ribulose bisphosphate carboxylase large chain (Fragment) OS=Sinapis alba GN=rbcL PE=3 SV=1	RBL_SINAL	52405.4	6.23	15	159	100	73	100

Peptide Information

Calc. Mass	Obsrv. Mass	± da	± ppm	Start Seq.	End Seq.	Sequence	Ion Score	C. I. %	Modification	Rank	Result Type
856.3546	856.426	0.0714	83	467	473	FEFPAMD					Mascot
910.4451	910.3304	-0.1147	-126	188	194	AVYECLR			Carbamidomethyl (C)[5]		Mascot
914.4049	914.2973	-0.1076	-118	306	312	NHGMHFR			Oxidation (M)[4]		Mascot
914.4049	914.2973	-0.1076	-118	306	312	NHGMHFR			Oxidation (M)[4]		Mascot
921.4346	921.3987	-0.0359	-39	1	8	MSPQTETK					Mascot
1021.5312	1021.409	-0.1222	-120	33	41	DTDILAAFR					Mascot
1116.583	1116.4622	-0.1208	-108	422	431	VALEACVQAR			Carbamidomethyl (C)[6]		Mascot
1187.6643	1187.4926	-0.1717	-145	286	295	DNGLLLHIHR					Mascot
1245.6433	1245.4839	-0.1594	-128	440	450	EGNEIIREASK					Mascot
1275.7307	1275.5717	-0.159	-125	340	350	EITLGFVDLLR					Mascot
1275.7307	1275.5717	-0.159	-125	340	350	EITLGFVDLLR	28	79.474			Mascot
1465.7546	1465.5778	-0.1768	-121	147	159	TFQGPPHGIQVER	22	20.144			Mascot
1465.7546	1465.5778	-0.1768	-121	147	159	TFQGPPHGIQVER					Mascot
1475.7351	1475.5702	-0.1649	-112	216	227	DRFLFCAEAIYK					Mascot
1546.7358	1546.5294	-0.2064	-133	451	463	WSPELAAACEVWK			Carbamidomethyl (C)[9]		Mascot
1794.8149	1794.6844	-0.1305	-73	237	252	GHYLNATAGTCEEMIK			Carbamidomethyl (C)[11]		Mascot
2169.9868	2169.7053	-0.2815	-130	195	213	GGLDFTKDDENVNSQPFMR					Mascot
2185.9819	2185.6948	-0.2871	-131	195	213	GGLDFTKDDENVNSQPFMR			Oxidation (M)[18]		Mascot
3854.8718	3854.314	-0.5578	-145	42	79	VTPQPGVPPEEAGAAVAAESSTGTWTTVWTDGLTSLDR	23	46.506			Mascot
3854.8718	3854.314	-0.5578	-145	42	79	VTPQPGVPPEEAGAAVAAESSTGTWTTVWTDGLTSLDR					Mascot

5	Ribulose bisphosphate carboxylase large chain OS=Cajanus cajan GN=rbcL PE=3 SV=1	RBL_CAJCA	52358.5	6.45	15	159	100	73	100

Peptide Information

Calc. Mass	Obsrv. Mass	± da	± ppm	Start Seq.	End Seq.	Sequence	Ion Score	C. I. %	Modification	Rank	Result Type
856.3546	856.426	0.0714	83	467	473	FEFPAMD					Mascot
910.4451	910.3304	-0.1147	-126	188	194	AVYECLR			Carbamidomethyl (C)[5]		Mascot
914.4049	914.2973	-0.1076	-118	306	312	NHGMHFR			Oxidation (M)[4]		Mascot
914.4049	914.2973	-0.1076	-118	306	312	NHGMHFR			Oxidation (M)[4]		Mascot
921.4346	921.3987	-0.0359	-39	1	8	MSPQTETK					Mascot
1021.5312	1021.409	-0.1222	-120	33	41	DTDILAAFR					Mascot
1116.583	1116.4622	-0.1208	-108	422	431	VALEACVQAR			Carbamidomethyl (C)[6]		Mascot
1187.6643	1187.4926	-0.1717	-145	286	295	DNGLLLHIHR					Mascot
1245.6337	1245.4839	-0.1498	-120	218	227	FLFCAEALFK			Carbamidomethyl (C)[4]		Mascot
1275.7307	1275.5717	-0.159	-125	340	350	EITLGFVDLLR					Mascot
1275.7307	1275.5717	-0.159	-125	340	350	EITLGFVDLLR	28	79.474			Mascot
1465.7546	1465.5778	-0.1768	-121	147	159	TFQGPPHGIQVER	22	20.144			Mascot
1465.7546	1465.5778	-0.1768	-121	147	159	TFQGPPHGIQVER					Mascot
1546.7358	1546.5294	-0.2064	-133	451	463	WSPELAAACEVWK			Carbamidomethyl (C)[9]		Mascot
1794.8149	1794.6844	-0.1305	-73	237	252	GHYLNATAGTCEEMIK			Carbamidomethyl (C)[11]		Mascot
1801.9305	1801.7034	-0.2271	-126	451	466	WSPELAAACEVWKAIK					Mascot
2169.9868	2169.7053	-0.2815	-130	195	213	GGLDFTKDDENVNSQPFMR					Mascot
2185.9819	2185.6948	-0.2871	-131	195	213	GGLDFTKDDENVNSQPFMR			Oxidation (M)[18]		Mascot
3854.8718	3854.314	-0.5578	-145	42	79	VTPQPGVPPEEAGAAVAAESSTGTWTTVWTDGLTSLDR	23	46.506			Mascot
3854.8718	3854.314	-0.5578	-145	42	79	VTPQPGVPPEEAGAAVAAESSTGTWTTVWTDGLTSLDR					Mascot

6	Ribulose bisphosphate carboxylase large chain OS=Corylus cornuta GN=rbcL PE=3 SV=1	RBL_CORCO	52521.5	6.22	15	157	100	71	100

Peptide Information

Calc. Mass	Obsrv. Mass	± da	± ppm	Start Seq.	End Seq.	Sequence	Ion Score	C. I. %	Modification	Rank	Result Type
910.4451	910.3304	-0.1147	-126	188	194	AVYECLR			Carbamidomethyl (C)[5]		Mascot
914.4049	914.2973	-0.1076	-118	306	312	NHGMHFR			Oxidation (M)[4]		Mascot
914.4049	914.2973	-0.1076	-118	306	312	NHGMHFR			Oxidation (M)[4]		Mascot
921.4346	921.3987	-0.0359	-39	1	8	MSPQTETK					Mascot
1021.5312	1021.409	-0.1222	-120	33	41	DTDILAAFR					Mascot
1116.583	1116.4622	-0.1208	-108	422	431	VALEACVQAR			Carbamidomethyl (C)[6]		Mascot
1187.6643	1187.4926	-0.1717	-145	286	295	DNGLLLHIHR					Mascot
1275.7307	1275.5717	-0.159	-125	340	350	EITLGFVDLLR					Mascot
1275.7307	1275.5717	-0.159	-125	340	350	EITLGFVDLLR	28	79.474			Mascot
1393.6522	1393.4941	-0.1581	-113	22	32	LTYYTPDYETK					Mascot
1465.7216	1465.5778	-0.1438	-98	320	334	MSGGDHIHAGTVVGK					Mascot
1465.7546	1465.5778	-0.1768	-121	147	159	TFQGPPHGIQVER	22	20.144			Mascot
1475.7351	1475.5702	-0.1649	-112	216	227	DRFLFCAEAIYK					Mascot
1546.7358	1546.5294	-0.2064	-133	451	463	WSPELAAACEVWK			Carbamidomethyl (C)[9]		Mascot
1794.8149	1794.6844	-0.1305	-73	237	252	GHYLNATAGTCEEMIK			Carbamidomethyl (C)[11]		Mascot
2169.9868	2169.7053	-0.2815	-130	195	213	GGLDFTKDDENVNSQPFMR					Mascot
2185.9819	2185.6948	-0.2871	-131	195	213	GGLDFTKDDENVNSQPFMR			Oxidation (M)[18]		Mascot
3854.8718	3854.314	-0.5578	-145	42	79	VTPQPGVPPEEAGAAVAAESSTGTWTTVWTDGLTSLDR	23	46.506			Mascot
3854.8718	3854.314	-0.5578	-145	42	79	VTPQPGVPPEEAGAAVAAESSTGTWTTVWTDGLTSLDR					Mascot

7	Ribulose bisphosphate carboxylase large chain OS=Liquidambar styraciflua GN=rbcL PE=3 SV=1	RBL_LIQST	52625.5	6.04	15	155	100	71	100

Peptide Information

Calc. Mass	Obsrv. Mass	± da	± ppm	Start Seq.	End Seq.	Sequence	Ion Score	C. I. %	Modification	Rank	Result Type
910.4451	910.3304	-0.1147	-126	188	194	AVYECLR			Carbamidomethyl (C)[5]		Mascot
914.4049	914.2973	-0.1076	-118	306	312	NHGMHFR			Oxidation (M)[4]		Mascot
914.4049	914.2973	-0.1076	-118	306	312	NHGMHFR			Oxidation (M)[4]		Mascot
921.4346	921.3987	-0.0359	-39	1	8	MSPQTETK					Mascot
1021.5312	1021.409	-0.1222	-120	33	41	DTDILAAFR					Mascot
1116.583	1116.4622	-0.1208	-108	422	431	VALEACVQAR			Carbamidomethyl (C)[6]		Mascot
1187.6643	1187.4926	-0.1717	-145	286	295	DNGLLLHIHR					Mascot
1245.6433	1245.4839	-0.1594	-128	440	450	EGNEIIREASK					Mascot
1275.7307	1275.5717	-0.159	-125	340	350	EITLGFVDLLR					Mascot
1275.7307	1275.5717	-0.159	-125	340	350	EITLGFVDLLR	28	79.474			Mascot
1465.7216	1465.5778	-0.1438	-98	320	334	MSGGDHIHAGTVVGK					Mascot
1465.7546	1465.5778	-0.1768	-121	147	159	TFQGPPHGIQVER	22	20.144			Mascot
1475.7351	1475.5702	-0.1649	-112	216	227	DRFLFCAEAIYK					Mascot
1546.7358	1546.5294	-0.2064	-133	451	463	WSPELAAACEVWK			Carbamidomethyl (C)[9]		Mascot
1794.8149	1794.6844	-0.1305	-73	237	252	GHYLNATAGTCEEMIK			Carbamidomethyl (C)[11]		Mascot
2169.9868	2169.7053	-0.2815	-130	195	213	GGLDFTKDDENVNSQPFMR					Mascot
2185.9819	2185.6948	-0.2871	-131	195	213	GGLDFTKDDENVNSQPFMR			Oxidation (M)[18]		Mascot
3854.8718	3854.314	-0.5578	-145	42	79	VTPQPGVPPEEAGAAVAAESSTGTWTTVWTDGLTSLDR	23	46.506			Mascot
3854.8718	3854.314	-0.5578	-145	42	79	VTPQPGVPPEEAGAAVAAESSTGTWTTVWTDGLTSLDR					Mascot

8	Ribulose bisphosphate carboxylase large chain (Fragment) OS=Erythrina crista-galli GN=rbcL PE=3 SV=	RBL_ERYCG	50384.5	6.22	14	152	100	73	100

Peptide Information

Calc. Mass	Obsrv. Mass	± da	± ppm	Start Seq.	End Seq.	Sequence	Ion Score	C. I. %	Modification	Rank	Result Type
910.4451	910.3304	-0.1147	-126	179	185	AVYECLR			Carbamidomethyl (C)[5]		Mascot
914.4049	914.2973	-0.1076	-118	297	303	NHGMHFR			Oxidation (M)[4]		Mascot
914.4049	914.2973	-0.1076	-118	297	303	NHGMHFR			Oxidation (M)[4]		Mascot
1021.5312	1021.409	-0.1222	-120	24	32	DTDILAAFR					Mascot
1116.583	1116.4622	-0.1208	-108	413	422	VALEACVQAR			Carbamidomethyl (C)[6]		Mascot
1187.6643	1187.4926	-0.1717	-145	277	286	DNGLLLHIHR					Mascot
1245.6433	1245.4839	-0.1594	-128	431	441	EGNEIIREASK					Mascot
1275.7307	1275.5717	-0.159	-125	331	341	EITLGFVDLIR					Mascot
1275.7307	1275.5717	-0.159	-125	331	341	EITLGFVDLIR	28	79.474			Mascot
1465.7546	1465.5778	-0.1768	-121	138	150	TFQGPPHGIQVER	22	20.144			Mascot
1465.7546	1465.5778	-0.1768	-121	138	150	TFQGPPHGIQVER					Mascot
1475.7351	1475.5702	-0.1649	-112	207	218	DRFLFCAEALYK					Mascot
1546.7358	1546.5294	-0.2064	-133	442	454	WSPELAAACEVWK			Carbamidomethyl (C)[9]		Mascot
1675.7784	1675.5927	-0.1857	-111	442	455	WSPELAAACEVWKE			Carbamidomethyl (C)[9]		Mascot
1794.8149	1794.6844	-0.1305	-73	228	243	GHYLNATAGTCEEMIK			Carbamidomethyl (C)[11]		Mascot
2169.9868	2169.7053	-0.2815	-130	186	204	GGLDFTKDDENVNSQPFMR					Mascot
2185.9819	2185.6948	-0.2871	-131	186	204	GGLDFTKDDENVNSQPFMR			Oxidation (M)[18]		Mascot
3854.8718	3854.314	-0.5578	-145	33	70	VTPQPGVPPEEAGAAVAAESSTGTWTTVWTDGLTSLDR	23	46.506			Mascot
3854.8718	3854.314	-0.5578	-145	33	70	VTPQPGVPPEEAGAAVAAESSTGTWTTVWTDGLTSLDR					Mascot

9	Ribulose bisphosphate carboxylase large chain (Fragment) OS=Lupinus albus GN=rbcL PE=3 SV=1	RBL_LUPAL	50283.4	6.22	14	152	100	73	100

Peptide Information

Calc. Mass	Obsrv. Mass	± da	± ppm	Start Seq.	End Seq.	Sequence	Ion Score	C. I. %	Modification	Rank	Result Type
910.4451	910.3304	-0.1147	-126	179	185	AVYECLR			Carbamidomethyl (C)[5]		Mascot
914.4049	914.2973	-0.1076	-118	297	303	NHGMHFR			Oxidation (M)[4]		Mascot
914.4049	914.2973	-0.1076	-118	297	303	NHGMHFR			Oxidation (M)[4]		Mascot
1021.5312	1021.409	-0.1222	-120	24	32	DTDILAAFR					Mascot
1116.583	1116.4622	-0.1208	-108	413	422	VALEACVQAR			Carbamidomethyl (C)[6]		Mascot
1187.6643	1187.4926	-0.1717	-145	277	286	DNGLLLHIHR					Mascot
1275.7307	1275.5717	-0.159	-125	331	341	EITLGFVDLLR					Mascot
1275.7307	1275.5717	-0.159	-125	331	341	EITLGFVDLLR	28	79.474			Mascot
1393.6522	1393.4941	-0.1581	-113	13	23	LTYYTPDYETK					Mascot
1465.7546	1465.5778	-0.1768	-121	138	150	TFQGPPHGIQVER	22	20.144			Mascot
1465.7546	1465.5778	-0.1768	-121	138	150	TFQGPPHGIQVER					Mascot
1475.7351	1475.5702	-0.1649	-112	207	218	DRFLFCAEALYK					Mascot
1546.7358	1546.5294	-0.2064	-133	442	454	WSPELAAACEVWK			Carbamidomethyl (C)[9]		Mascot
1675.7784	1675.5927	-0.1857	-111	442	455	WSPELAAACEVWKE			Carbamidomethyl (C)[9]		Mascot
1794.8149	1794.6844	-0.1305	-73	228	243	GHYLNATAGTCEEMIK			Carbamidomethyl (C)[11]		Mascot
2169.9868	2169.7053	-0.2815	-130	186	204	GGLDFTKDDENVNSQPFMR					Mascot
2185.9819	2185.6948	-0.2871	-131	186	204	GGLDFTKDDENVNSQPFMR			Oxidation (M)[18]		Mascot
3854.8718	3854.314	-0.5578	-145	33	70	VTPQPGVPPEEAGAAVAAESSTGTWTTVWTDGLTSLDR	23	46.506			Mascot
3854.8718	3854.314	-0.5578	-145	33	70	VTPQPGVPPEEAGAAVAAESSTGTWTTVWTDGLTSLDR					Mascot

10	Ribulose bisphosphate carboxylase large chain (Fragment) OS=Clitoria ternatea GN=rbcL PE=3 SV=1	RBL_CLITE	51460	6.23	14	151	100	73	100

Peptide Information

Calc. Mass	Obsrv. Mass	± da	± ppm	Start Seq.	End Seq.	Sequence	Ion Score	C. I. %	Modification	Rank	Result Type
910.4451	910.3304	-0.1147	-126	178	184	AVYECLR			Carbamidomethyl (C)[5]		Mascot
914.4049	914.2973	-0.1076	-118	296	302	NHGMHFR			Oxidation (M)[4]		Mascot
914.4049	914.2973	-0.1076	-118	296	302	NHGMHFR			Oxidation (M)[4]		Mascot
1021.5312	1021.409	-0.1222	-120	23	31	DTDILAAFR					Mascot
1116.583	1116.4622	-0.1208	-108	412	421	VALEACVQAR			Carbamidomethyl (C)[6]		Mascot
1187.6643	1187.4926	-0.1717	-145	276	285	DNGLLLHIHR					Mascot
1245.6433	1245.4839	-0.1594	-128	430	440	EGNEIIREASK					Mascot
1275.7307	1275.5717	-0.159	-125	330	340	EITLGFVDLLR					Mascot
1275.7307	1275.5717	-0.159	-125	330	340	EITLGFVDLLR	28	77.795			Mascot
1465.7546	1465.5778	-0.1768	-121	137	149	TFQGPPHGIQVER	22	13.613			Mascot
1465.7546	1465.5778	-0.1768	-121	137	149	TFQGPPHGIQVER					Mascot
1475.7351	1475.5702	-0.1649	-112	206	217	DRFLFCAEAIYK					Mascot
1546.7358	1546.5294	-0.2064	-133	441	453	WSPELAAACEVWK			Carbamidomethyl (C)[9]		Mascot
1794.8149	1794.6844	-0.1305	-73	227	242	GHYLNATAGTCEEMIK			Carbamidomethyl (C)[11]		Mascot
1801.9305	1801.7034	-0.2271	-126	441	456	WSPELAAACEVWKAIK					Mascot
2169.9868	2169.7053	-0.2815	-130	185	203	GGLDFTKDDENVNSQPFMR					Mascot
2185.9819	2185.6948	-0.2871	-131	185	203	GGLDFTKDDENVNSQPFMR			Oxidation (M)[18]		Mascot
3854.8718	3854.314	-0.5578	-145	32	69	VTPQPGVPPEEAGAAVAAESSTGTWTTVWTDGLTSLDR	23	42.13			Mascot
3854.8718	3854.314	-0.5578	-145	32	69	VTPQPGVPPEEAGAAVAAESSTGTWTTVWTDGLTSLDR					Mascot

Gel Idx/Pos	39/B12	Instr./Gel Origin	BA2060/131017A	Process State	Analysis Succeeded
Plate [#] Name	[1] 31496	Instrument Sample Name		Spectra	11

Rank	Protein Name	Accession No.	Protein MW	Protein PI	Pep. Count	Protein Score	Protein Score C. I. %	Total Ion Score	Total Ion C. I. %
1	Ribulose bisphosphate carboxylase large chain OS=Hyophorbe lagenicaulis GN=rbcL PE=3 SV=1	RBL_HYOLA	52928.5	6.13	13	71	99.707	6	0

Peptide Information

Calc. Mass	Obsrv. Mass	± da	± ppm	Start Seq.	End Seq.	Sequence	Ion Score	C. I. %	Modification	Rank	Result Type
910.4451	910.3491	-0.096	-105	188	194	AVYECLR			Carbamidomethyl (C)[5]		Mascot
914.4049	914.3074	-0.0975	-107	306	312	NHGMHFR			Oxidation (M)[4]		Mascot
921.4346	921.4014	-0.0332	-36	1	8	MSPQTETK					Mascot
996.4672	996.4875	0.0203	20	467	474	FEFDPVDK					Mascot
1021.5312	1021.4232	-0.108	-106	33	41	DTDILAAFR					Mascot
1245.6433	1245.4983	-0.145	-116	440	450	EGNEIIREASK					Mascot
1417.7686	1417.5901	-0.1785	-126	135	146	LEDLRIPPSYSK					Mascot
1465.7216	1465.5858	-0.1358	-93	320	334	MSGGDHIHAGTVVGK					Mascot
1465.7546	1465.5858	-0.1688	-115	147	159	TFQGPPHGIQVER					Mascot
2057.0259	2056.7896	-0.2363	-115	340	356	EMTLGFVDLLRDDFIEK			Oxidation (M)[2]		Mascot
2169.9868	2169.741	-0.2458	-113	195	213	GGLDFTKDDENVNSQPFMR					Mascot
3022.4548	3022.0471	-0.4077	-135	259	285	ELGVPIVMHDYLTGGFTANTSLAHYCR			Carbamidomethyl (C)[26]		Mascot
3854.8718	3854.3772	-0.4946	-128	42	79	VTPQPGVPPEEAGAAVAESSTGTWTTVWTDGLTSLDR	6	0			Mascot
3854.8718	3854.3772	-0.4946	-128	42	79	VTPQPGVPPEEAGAAVAESSTGTWTTVWTDGLTSLDR					Mascot

Rank	Protein Name	Accession No.	Protein MW	Protein PI	Pep. Count	Protein Score	Protein Score C. I. %	Total Ion Score	Total Ion C. I. %
2	Ribulose bisphosphate carboxylase large chain (Fragment) OS=Sinapis alba GN=rbcL PE=3 SV=1	RBL_SINAL	52405.4	6.23	11	66	99.193	13	0

Peptide Information

Calc. Mass	Obsrv. Mass	± da	± ppm	Start Seq.	End Seq.	Sequence	Ion Score	C. I. %	Modification	Rank	Result Type
856.3546	856.4416	0.087	102	467	473	FEFPAMD					Mascot
910.4451	910.3491	-0.096	-105	188	194	AVYECLR			Carbamidomethyl (C)[5]		Mascot
914.4049	914.3074	-0.0975	-107	306	312	NHGMHFR			Oxidation (M)[4]		Mascot
921.4346	921.4014	-0.0332	-36	1	8	MSPQTETK					Mascot
1021.5312	1021.4232	-0.108	-106	33	41	DTDILAAFR					Mascot
1245.6433	1245.4983	-0.145	-116	440	450	EGNEIIREASK					Mascot
1275.7307	1275.5874	-0.1433	-112	340	350	EITLGFVDLLR	7	0			Mascot
1275.7307	1275.5874	-0.1433	-112	340	350	EITLGFVDLLR					Mascot
1465.7546	1465.5858	-0.1688	-115	147	159	TFQGPPHGIQVER					Mascot
1465.7546	1465.5858	-0.1688	-115	147	159	TFQGPPHGIQVER					Mascot
2169.9868	2169.741	-0.2458	-113	195	213	GGLDFTKDDENVNSQPFMR					Mascot
3022.4548	3022.0471	-0.4077	-135	259	285	ELGVPIVMHDYLTGGFTANTSLAHYCR			Carbamidomethyl (C)[26]		Mascot
3854.8718	3854.3772	-0.4946	-128	42	79	VTPQPGVPPEEAGAAVAESSTGTWTTVWTDGLTSLDR	6	0			Mascot
3854.8718	3854.3772	-0.4946	-128	42	79	VTPQPGVPPEEAGAAVAESSTGTWTTVWTDGLTSLDR					Mascot

Rank	Protein Name	Accession No.	Protein MW	Protein PI	Pep. Count	Protein Score	Protein Score C. I. %	Total Ion Score	Total Ion C. I. %
3	Ribulose bisphosphate carboxylase large chain OS=Liquidambar styraciflua GN=rbcL PE=3 SV=1	RBL_LIQST	52625.5	6.04	11	65	98.984	13	0

Peptide Information

Calc. Mass	Obsrv. Mass	± da	± ppm	Start Seq.	End Seq.	Sequence	Ion Score	C. I. %	Modification	Rank	Result Type
910.4451	910.3491	-0.096	-105	188	194	AVYECLR			Carbamidomethyl (C)[5]		Mascot
914.4049	914.3074	-0.0975	-107	306	312	NHGMHFR			Oxidation (M)[4]		Mascot
921.4346	921.4014	-0.0332	-36	1	8	MSPQTETK					Mascot
1021.5312	1021.4232	-0.108	-106	33	41	DTDILAAFR					Mascot
1245.6433	1245.4983	-0.145	-116	440	450	EGNEIIREASK					Mascot
1275.7307	1275.5874	-0.1433	-112	340	350	EITLGFVDLLR	7	0			Mascot
1275.7307	1275.5874	-0.1433	-112	340	350	EITLGFVDLLR					Mascot
1465.7216	1465.5858	-0.1358	-93	320	334	MSGGDHIHAGTVVGK					Mascot
1465.7546	1465.5858	-0.1688	-115	147	159	TFQGPPHGIQVER					Mascot
2169.9868	2169.741	-0.2458	-113	195	213	GGLDFTKDDENVNSQPFMR					Mascot
3022.4548	3022.0471	-0.4077	-135	259	285	ELGVPIVMHDYLTGGFTANTSLAHYCR			Carbamidomethyl (C)[26]		Mascot
3854.8718	3854.3772	-0.4946	-128	42	79	VTPQPGVPPEEAGAAVAESSTGTWTTVWTDGLTSLDR	6	0			Mascot
3854.8718	3854.3772	-0.4946	-128	42	79	VTPQPGVPPEEAGAAVAESSTGTWTTVWTDGLTSLDR					Mascot

Rank	Protein Name	Accession No.	Protein MW	Protein PI	Pep. Count	Protein Score	Protein Score C. I. %	Total Ion Score	Total Ion C. I. %
4	Ribulose bisphosphate carboxylase large chain OS=Sesbania sesban GN=rbcL PE=3 SV=1	RBL_SESSE	52358.4	6.09	11	65	98.936	13	0

Peptide Information

Calc. Mass	Obsrv. Mass	± da	± ppm	Start Seq.	End Seq.	Sequence	Ion Score	C. I. %	Modification	Rank	Result Type
856.3546	856.4416	0.087	102	467	473	FEFPAMD					Mascot
910.4451	910.3491	-0.096	-105	188	194	AVYECLR			Carbamidomethyl (C)[5]		Mascot
914.4049	914.3074	-0.0975	-107	306	312	NHGMHFR			Oxidation (M)[4]		Mascot
921.4346	921.4014	-0.0332	-36	1	8	MSPQTETK					Mascot
1021.5312	1021.4232	-0.108	-106	33	41	DTDILAAFR					Mascot
1245.6337	1245.4983	-0.1354	-109	218	227	FLFCAEAIFK			Carbamidomethyl (C)[4]		Mascot
1275.7307	1275.5874	-0.1433	-112	340	350	EITLGFVDLLR	7	0			Mascot
1275.7307	1275.5874	-0.1433	-112	340	350	EITLGFVDLLR					Mascot
1465.7546	1465.5858	-0.1688	-115	147	159	TFQGPPHGIQVER					Mascot
1465.7546	1465.5858	-0.1688	-115	147	159	TFQGPPHGIQVER					Mascot
2169.9868	2169.741	-0.2458	-113	195	213	GGLDFTKDDENVNSQPFMR					Mascot
3022.4548	3022.0471	-0.4077	-135	259	285	ELGVPIVMHDYLTGGFTANTSLAHYCR			Carbamidomethyl (C)[26]		Mascot
3854.8718	3854.3772	-0.4946	-128	42	79	VTPQPGVPPEEAGAAVAESSTGTWTTVWTDGLTSLDR	6	0			Mascot
3854.8718	3854.3772	-0.4946	-128	42	79	VTPQPGVPPEEAGAAVAESSTGTWTTVWTDGLTSLDR					Mascot

Rank	Protein Name	Accession No.	Protein MW	Protein PI	Pep. Count	Protein Score	Protein Score C. I. %	Total Ion Score	Total Ion C. I. %
5	Ribulose bisphosphate carboxylase large chain (Fragment) OS=Drymophloeus subdistichus GN=rbcL PE=3	RBL_DRYSU	52254.1	6.33	12	65	98.86	6	0

Peptide Information

Calc. Mass	Obsrv. Mass	± da	± ppm	Start Seq.	End Seq.	Sequence	Ion Score	C. I. %	Modification	Rank	Result Type
910.4451	910.3491	-0.096	-105	188	194	AVYECLR			Carbamidomethyl (C)[5]		Mascot
914.4049	914.3074	-0.0975	-107	306	312	NHGMHFR			Oxidation (M)[4]		Mascot
921.4346	921.4014	-0.0332	-36	1	8	MSPQTETK					Mascot
1021.5312	1021.4232	-0.108	-106	33	41	DTDILAAFR					Mascot
1245.6433	1245.4983	-0.145	-116	440	450	EGNEIIREASK					Mascot
1465.7216	1465.5858	-0.1358	-93	320	334	MSGGDHIHAGTVVGK					Mascot
1465.7546	1465.5858	-0.1688	-115	147	159	TFQGPPHGIQVER					Mascot
1801.9305	1801.7186	-0.2119	-118	451	466	WSPELAAACEVWKAIK					Mascot
1801.9305	1801.7186	-0.2119	-118	451	466	WSPELAAACEVWKAIK					Mascot
2057.0259	2056.7896	-0.2363	-115	340	356	EMTLGFVDLLRDDFIEK			Oxidation (M)[2]		Mascot
2169.9868	2169.741	-0.2458	-113	195	213	GGLDFTKDDENVNSQPFMR					Mascot
3022.4548	3022.0471	-0.4077	-135	259	285	ELGVPIVMHDYLTGGFTANTSLAHYCR			Carbamidomethyl (C)[26]		Mascot
3854.8718	3854.3772	-0.4946	-128	42	79	VTPQPGVPPEEAGAAVAESSTGTWTTVWTDGLTSLDR	6	0			Mascot
3854.8718	3854.3772	-0.4946	-128	42	79	VTPQPGVPPEEAGAAVAESSTGTWTTVWTDGLTSLDR					Mascot

Rank	Protein Name	Accession No.	Protein MW	Protein PI	Pep. Count	Protein Score	Protein Score C. I. %	Total Ion Score	Total Ion C. I. %
6	Ribulose bisphosphate carboxylase large chain OS=Cajanus cajan GN=rbcL PE=3 SV=1	RBL_CAJCA	52358.5	6.45	11	65	98.833	13	0

Peptide Information

Calc. Mass	Obsrv. Mass	± da	± ppm	Start Seq.	End Seq.	Sequence	Ion Score	C. I. %	Modification	Rank	Result Type
856.3546	856.4416	0.087	102	467	473	FEFPAMD					Mascot
910.4451	910.3491	-0.096	-105	188	194	AVYECLR			Carbamidomethyl (C)[5]		Mascot
914.4049	914.3074	-0.0975	-107	306	312	NHGMHFR			Oxidation (M)[4]		Mascot
921.4346	921.4014	-0.0332	-36	1	8	MSPQTETK					Mascot
1021.5312	1021.4232	-0.108	-106	33	41	DTDILAAFR					Mascot
1245.6337	1245.4983	-0.1354	-109	218	227	FLFCAEALFK			Carbamidomethyl (C)[4]		Mascot
1275.7307	1275.5874	-0.1433	-112	340	350	EITLGFVDLLR	7	0			Mascot
1275.7307	1275.5874	-0.1433	-112	340	350	EITLGFVDLLR					Mascot
1465.7546	1465.5858	-0.1688	-115	147	159	TFQGPPHGIQVER					Mascot
1465.7546	1465.5858	-0.1688	-115	147	159	TFQGPPHGIQVER					Mascot
1801.9305	1801.7186	-0.2119	-118	451	466	WSPELAAACEVWKAIK					Mascot
1801.9305	1801.7186	-0.2119	-118	451	466	WSPELAAACEVWKAIK					Mascot
2169.9868	2169.741	-0.2458	-113	195	213	GGLDFTKDDENVNSQPFMR					Mascot
3854.8718	3854.3772	-0.4946	-128	42	79	VTPQPGVPPEEAGAAVAESSTGTWTTVWTDGLTSLDR	6	0			Mascot
3854.8718	3854.3772	-0.4946	-128	42	79	VTPQPGVPPEEAGAAVAESSTGTWTTVWTDGLTSLDR					Mascot

Rank	Protein Name	Accession No.	Protein MW	Protein PI	Pep. Count	Protein Score	Protein Score C. I. %	Total Ion Score	Total Ion C. I. %
7	Ribulose bisphosphate carboxylase large chain (Fragment) OS=Phoenix reclinata GN=rbcL PE=3 SV=1	RBL_PHORE	51737.9	6.44	12	64	98.598	6	0

Peptide Information

Calc. Mass	Obsrv. Mass	± da	± ppm	Start Seq.	End Seq.	Sequence	Ion Score	C. I. %	Modification	Rank	Result Type
910.4451	910.3491	-0.096	-105	188	194	AVYECLR			Carbamidomethyl (C)[5]		Mascot
914.4049	914.3074	-0.0975	-107	306	312	NHGMHFR			Oxidation (M)[4]		Mascot
921.4346	921.4014	-0.0332	-36	1	8	MSPQTETK					Mascot
1021.5312	1021.4232	-0.108	-106	33	41	DTDILAAFR					Mascot
1245.6433	1245.4983	-0.145	-116	440	450	EGNEIIREASK					Mascot
1465.7216	1465.5858	-0.1358	-93	320	334	MSGGDHIHAGTVVGK					Mascot
1465.7546	1465.5858	-0.1688	-115	147	159	TFQGPPHGIQVER					Mascot
1801.9305	1801.7186	-0.2119	-118	451	466	WSPELAAACEVWKAIK					Mascot
1801.9305	1801.7186	-0.2119	-118	451	466	WSPELAAACEVWKAIK					Mascot
2057.0259	2056.7896	-0.2363	-115	340	356	EMTLGFVDLLRDDFIEK			Oxidation (M)[2]		Mascot
2169.9868	2169.741	-0.2458	-113	195	213	GGLDFTKDDENVNSQPFMR					Mascot
3022.4548	3022.0471	-0.4077	-135	259	285	ELGVPIVMHDYLTGGFTANTSLAHYCR			Carbamidomethyl (C)[26]		Mascot
3854.8718	3854.3772	-0.4946	-128	42	79	VTPQPGVPPEEAGAAVAESSTGTWTTVWTDGLTSLDR	6	0			Mascot
3854.8718	3854.3772	-0.4946	-128	42	79	VTPQPGVPPEEAGAAVAESSTGTWTTVWTDGLTSLDR					Mascot

Rank	Protein Name	Accession No.	Protein MW	Protein PI	Pep. Count	Protein Score	Protein Score C. I. %	Total Ion Score	Total Ion C. I. %
8	Ribulose bisphosphate carboxylase large chain (Fragment) OS=Cornus obliqua GN=rbcL PE=3 SV=2	RBL_COROB	51536.8	6	10	60	96.311	13	0

Peptide Information

Calc. Mass	Obsrv. Mass	± da	± ppm	Start Seq.	End Seq.	Sequence	Ion Score	C. I. %	Modification	Rank	Result Type
910.4451	910.3491	-0.096	-105	178	184	AVYECLR			Carbamidomethyl (C)[5]		Mascot
914.4049	914.3074	-0.0975	-107	296	302	NHGMHFR			Oxidation (M)[4]		Mascot
1021.5312	1021.4232	-0.108	-106	23	31	DTDILAAFR					Mascot
1245.6433	1245.4983	-0.145	-116	430	440	EGNEIIREASK					Mascot
1275.7307	1275.5874	-0.1433	-112	330	340	EITLGFVDLLR	7	0			Mascot
1275.7307	1275.5874	-0.1433	-112	330	340	EITLGFVDLLR					Mascot
1465.7546	1465.5858	-0.1688	-115	137	149	TFQGPPHGIQVER					Mascot
1465.7546	1465.5858	-0.1688	-115	137	149	TFQGPPHGIQVER					Mascot
1801.8643	1801.7186	-0.1457	-81	9	22	EYKLTYHTPEYETK					Mascot
1801.8643	1801.7186	-0.1457	-81	9	22	EYKLTYHTPEYETK					Mascot
2169.9868	2169.741	-0.2458	-113	185	203	GGLDFTKDDENVNSQPFMR					Mascot
3022.4548	3022.0471	-0.4077	-135	249	275	ELGVPIVMHDYLTGGFTANTSLAHYCR			Carbamidomethyl (C)[26]		Mascot
3854.8718	3854.3772	-0.4946	-128	32	69	VTPQPGVPPEEAGAAVAESSTGTWTTVWTDGLTSLDR	6	0			Mascot
3854.8718	3854.3772	-0.4946	-128	32	69	VTPQPGVPPEEAGAAVAESSTGTWTTVWTDGLTSLDR					Mascot

Rank	Protein Name	Accession No.	Protein MW	Protein PI	Pep. Count	Protein Score	Protein Score C. I. %	Total Ion Score	Total Ion C. I. %
9	Ribulose bisphosphate carboxylase large chain (Fragment) OS=Prunus domestica GN=rbcL PE=3 SV=1	RBL_PRUDO	50914.6	6.23	10	59	95.666	13	0

Peptide Information

Calc. Mass	Obsrv. Mass	± da	± ppm	Start Seq.	End Seq.	Sequence	Ion Score	C. I. %	Modification	Rank	Result Type
910.4451	910.3491	-0.096	-105	178	184	AVYECLR			Carbamidomethyl (C)[5]		Mascot
914.4049	914.3074	-0.0975	-107	296	302	NHGMHFR			Oxidation (M)[4]		Mascot
1021.5312	1021.4232	-0.108	-106	23	31	DTDILAAFR					Mascot
1245.6433	1245.4983	-0.145	-116	430	440	EGNEIIREASK					Mascot
1275.7307	1275.5874	-0.1433	-112	330	340	EITLGFVDLLR	7	0			Mascot
1275.7307	1275.5874	-0.1433	-112	330	340	EITLGFVDLLR					Mascot
1465.7216	1465.5858	-0.1358	-93	310	324	MSGGDHIHAGTVVGK					Mascot
1465.7546	1465.5858	-0.1688	-115	137	149	TFQGPPHGIQVER					Mascot
2169.9868	2169.741	-0.2458	-113	185	203	GGLDFTKDDENVNSQPFMR					Mascot
3022.4548	3022.0471	-0.4077	-135	249	275	ELGVPIVMHDYLTGGFTANTSLAHYCR			Carbamidomethyl (C)[26]		Mascot
3854.8718	3854.3772	-0.4946	-128	32	69	VTPQPGVPPEEAGAAVAESSTGTWTTVWTDGLTSLDR	6	0			Mascot
3854.8718	3854.3772	-0.4946	-128	32	69	VTPQPGVPPEEAGAAVAESSTGTWTTVWTDGLTSLDR					Mascot

Rank	Protein Name	Accession No.	Protein MW	Protein PI	Pep. Count	Protein Score	Protein Score C. I. %	Total Ion Score	Total Ion C. I. %
10	Ribulose bisphosphate carboxylase large chain (Fragment) OS=Geum quellyon GN=rbcL PE=3 SV=1	RBL_GEUQU	50910.8	6.23	10	59	95.565	13	0

Peptide Information

Calc. Mass	Obsrv. Mass	± da	± ppm	Start Seq.	End Seq.	Sequence	Ion Score	C. I. %	Modification	Rank	Result Type
910.4451	910.3491	-0.096	-105	178	184	AVYECLR			Carbamidomethyl (C)[5]		Mascot
914.4049	914.3074	-0.0975	-107	296	302	NHGMHFR			Oxidation (M)[4]		Mascot
1021.5312	1021.4232	-0.108	-106	23	31	DTDILAAFR					Mascot
1245.6337	1245.4983	-0.1354	-109	208	217	FLFCAEALFK			Carbamidomethyl (C)[4]		Mascot
1275.7307	1275.5874	-0.1433	-112	330	340	EITLGFVDLLR	7	0			Mascot
1275.7307	1275.5874	-0.1433	-112	330	340	EITLGFVDLLR					Mascot
1465.7216	1465.5858	-0.1358	-93	310	324	MSGGDHIHAGTVVGK					Mascot
1465.7546	1465.5858	-0.1688	-115	137	149	TFQGPPHGIQVER					Mascot
2169.9868	2169.741	-0.2458	-113	185	203	GGLDFTKDDENVNSQPFMR					Mascot
3022.4548	3022.0471	-0.4077	-135	249	275	ELGVPIVMHDYLTGGFTANTSLAHYCR			Carbamidomethyl (C)[26]		Mascot
3854.8718	3854.3772	-0.4946	-128	32	69	VTPQPGVPPEEAGAAVAESSTGTWTTVWTDGLTSLDR	6	0			Mascot
3854.8718	3854.3772	-0.4946	-128	32	69	VTPQPGVPPEEAGAAVAESSTGTWTTVWTDGLTSLDR					Mascot

Gel Idx/Pos	40/B13	Instr./Gel Origin	BA2060/131017A	Process Sta	Analysis Succeeded
Plate [#] Name	[1] 31496	Instrument Sample Name		Spectra	8

Rank	Protein Name	Accession No	Protein MW	Protein PI	Pep. Count	Protein Score	Protein Score C. I. %	Total Ion Score	Total Ion C. I. %
1	RuBisCO large subunit-binding protein subunit be chloroplastic OS=Pisum sativum PE=1 SV=2	RUBB_PEA	62945.3	5.85	15	290	100	216	100

Peptide Information

Calc. Mass	Obsrv. Mass	± da	± ppm	Start Seq.	End Seq.	Sequence	Ion Score	C. I. %	Modification	Rank	Result Type
856.5363	856.4373	-0.099	-116	122	129	LVRQAAAK					Mascot
1116.47	1116.4625	-0.0075	-7	259	267	MTVEFENCK			Oxidation (M)[1]		Mascot
1182.7092	1182.5812	-0.128	-108	72	83	LADLVGVTLGPK					Mascot
1280.7684	1280.6305	-0.1379	-108	155	167	VVAAGANPVLITR					Mascot
1284.7158	1284.577	-0.1388	-108	280	290	DLINILEDAIR	69	99.999			Mascot
1284.7158	1284.577	-0.1388	-108	280	290	DLINILEDAIR					Mascot
1501.7614	1501.6364	-0.125	-83	550	563	VVRCCLEHASSVAK					Mascot
1505.7158	1505.547	-0.1688	-112	246	258	GYISPYFVTDSEK					Mascot
1541.7693	1541.599	-0.1703	-110	108	121	EVELEDPVENIGAK					Mascot
1700.8999	1700.7122	-0.1877	-110	453	469	AAVEEGIVVGGGCTLLR			Carbamidomethyl (C)[13]		Mascot
1700.8999	1700.7122	-0.1877	-110	453	469	AAVEEGIVVGGGCTLLR	46	99.718	Carbamidomethyl (C)[13]		Mascot
1834.9908	1834.7856	-0.2052	-112	334	350	SQYLDDIAILTGGTVIR	75	100			Mascot
1834.9908	1834.7856	-0.2052	-112	334	350	SQYLDDIAILTGGTVIR					Mascot
1891.9355	1891.724	-0.2115	-112	376	393	DTTTIVGDGSTQEAVNKR					Mascot
1900.0386	1899.8256	-0.213	-112	420	438	LSGGVAVIQVGAQTETELK					Mascot
1963.0858	1962.8594	-0.2264	-115	333	350	KSQYLDDIAILTGGTVIR	26	73.332			Mascot
1963.0858	1962.8594	-0.2264	-115	333	350	KSQYLDDIAILTGGTVIR					Mascot
2157.176	2156.9324	-0.2436	-113	420	440	LSGGVAVIQVGAQTETELKEK					Mascot
2430.2722	2429.9951	-0.2771	-114	130	154	TNDLAGDGTTTSVVLAQGLIAEGVK					Mascot

Rank	Protein Name	Accession No	Protein MW	Protein PI	Pep. Count	Protein Score	Protein Score C. I. %	Total Ion Score	Total Ion C. I. %
2	Ribulose bisphosphate carboxylase large chain OS=Sesbania sesban GN=rbcL PE=3 SV=1	RBL_SESSE	52358.4	6.09	16	160	100	63	99.994

Peptide Information

Calc. Mass	Obsrv. Mass	± da	± ppm	Start Seq.	End Seq.	Sequence	Ion Score	C. I. %	Modification	Rank	Result Type
856.3546	856.4373	0.0827	97	467	473	FEFPAMD					Mascot
898.41	898.3174	-0.0926	-103	306	312	NHGMHFR					Mascot
910.4451	910.3486	-0.0965	-106	188	194	AVYECLR			Carbamidomethyl (C)[5]		Mascot
914.4049	914.3092	-0.0957	-105	306	312	NHGMHFR			Oxidation (M)[4]		Mascot
921.4346	921.4083	-0.0263	-29	1	8	MSPQTETK					Mascot
1021.5312	1021.4243	-0.1069	-105	33	41	DTDILAAFR					Mascot
1039.4691	1039.415	-0.0541	-52	351	358	DDYVEKDR					Mascot
1116.583	1116.4625	-0.1205	-108	422	431	VALEACVQAR			Carbamidomethyl (C)[6]		Mascot
1170.5585	1170.5106	-0.0479	-41	304	312	QKNHGMHFR			Oxidation (M)[6]		Mascot
1187.6643	1187.532	-0.1323	-111	286	295	DNGLLLHIHR					Mascot
1245.6337	1245.496	-0.1377	-111	218	227	FLFCAEAIFK			Carbamidomethyl (C)[4]		Mascot
1275.7307	1275.5936	-0.1371	-107	340	350	EITLGFVDLLR					Mascot
1393.6522	1393.504	-0.1482	-106	22	32	LTYYTPDYETK					Mascot
1465.7546	1465.5961	-0.1585	-108	147	159	TFQGPPHGIQVER					Mascot
1465.7546	1465.5961	-0.1585	-108	147	159	TFQGPPHGIQVER	63	99.994			Mascot
1502.8512	1502.6777	-0.1735	-115	165	177	YGRPLLGCTIKPK			Carbamidomethyl (C)[8]		Mascot
2169.9868	2169.7493	-0.2375	-109	195	213	GGLDFTKDDENVNSQPFMR					Mascot
2185.9819	2185.7402	-0.2417	-111	195	213	GGLDFTKDDENVNSQPFMR			Oxidation (M)[18]		Mascot
3854.8718	3854.4333	-0.4385	-114	42	79	VTPQPGVPPEEAGAAVAAESSTGTWTTVWTDGLTSLDR					Mascot

Rank	Protein Name	Accession No	Protein MW	Protein PI	Pep. Count	Protein Score	Protein Score C. I. %	Total Ion Score	Total Ion C. I. %
3	Ribulose bisphosphate carboxylase large chain (Fragment) OS=Prunus domestica GN=rbcL PE=3 SV=1	RBL_PRUDO	50914.6	6.23	15	149	100	61	99.992

Peptide Information

Calc. Mass	Obsrv. Mass	± da	± ppm	Start Seq.	End Seq.	Sequence	Ion Score	C. I. %	Modification	Rank	Result Type
898.41	898.3174	-0.0926	-103	296	302	NHGMHFR					Mascot
910.4451	910.3486	-0.0965	-106	178	184	AVYECLR			Carbamidomethyl (C)[5]		Mascot
914.4049	914.3092	-0.0957	-105	296	302	NHGMHFR			Oxidation (M)[4]		Mascot
1021.5312	1021.4243	-0.1069	-105	23	31	DTDILAAFR					Mascot
1116.583	1116.4625	-0.1205	-108	412	421	VALEACVQAR			Carbamidomethyl (C)[6]		Mascot
1170.5585	1170.5106	-0.0479	-41	294	302	QKNHGMHFR			Oxidation (M)[6]		Mascot
1187.6643	1187.532	-0.1323	-111	276	285	DNGLLLHIHR					Mascot
1245.6433	1245.496	-0.1473	-118	430	440	EGNEIIREASK					Mascot
1275.7307	1275.5936	-0.1371	-107	330	340	EITLGFVDLLR					Mascot
1393.6522	1393.504	-0.1482	-106	12	22	LTYYTPDYETK					Mascot
1465.7216	1465.5961	-0.1255	-86	310	324	MSGGDHIHAGTVVGK					Mascot
1465.7546	1465.5961	-0.1585	-108	137	149	TFQGPPHGIQVER	63	99.994			Mascot
1502.8512	1502.6777	-0.1735	-115	155	167	YGRPLLGCTIKPK			Carbamidomethyl (C)[8]		Mascot
1830.7455	1830.6284	-0.1171	-64	227	242	GHYLNATAGTCEDMMK			Carbamidomethyl (C)[11], Oxidation (M)[14,15]		Mascot
2169.9868	2169.7493	-0.2375	-109	185	203	GGLDFTKDDENVNSQPFMR					Mascot
2185.9819	2185.7402	-0.2417	-111	185	203	GGLDFTKDDENVNSQPFMR			Oxidation (M)[18]		Mascot
3854.8718	3854.4333	-0.4385	-114	32	69	VTPQPGVPPEEAGAAVAAESSTGTWTTVWTDGLTSLDR					Mascot

Rank	Protein Name	Accession No	Protein MW	Protein PI	Pep. Count	Protein Score	Protein Score C. I. %	Total Ion Score	Total Ion C. I. %
4	Ribulose bisphosphate carboxylase large chain (Fragment) OS=Serenoa repens GN=rbcL PE=3 SV=1	RBL_SERRE	51794	6.33	15	148	100	61	99.992

Peptide Information

Calc. Mass	Obsrv. Mass	± da	± ppm	Start Seq.	End Seq.	Sequence	Ion Score	C. I. %	Modification	Rank	Result Type
898.41	898.3174	-0.0926	-103	306	312	NHGMHFR					Mascot
910.4451	910.3486	-0.0965	-106	188	194	AVYECLR			Carbamidomethyl (C)[5]		Mascot
914.4049	914.3092	-0.0957	-105	306	312	NHGMHFR			Oxidation (M)[4]		Mascot
921.4346	921.4083	-0.0263	-29	1	8	MSPQTETK					Mascot
1021.5312	1021.4243	-0.1069	-105	33	41	DTDILAAFR					Mascot
1116.583	1116.4625	-0.1205	-108	422	431	VALEACVQAR			Carbamidomethyl (C)[6]		Mascot
1170.5585	1170.5106	-0.0479	-41	304	312	QKNHGMHFR			Oxidation (M)[6]		Mascot
1187.6643	1187.532	-0.1323	-111	286	295	DNGLLLHIHR					Mascot
1245.6433	1245.496	-0.1473	-118	440	450	EGNEIIREASK					Mascot
1393.6522	1393.504	-0.1482	-106	22	32	LTYYTPDYETK					Mascot
1465.7216	1465.5961	-0.1255	-86	320	334	MSGGDHIHAGTVVGK					Mascot
1465.7546	1465.5961	-0.1585	-108	147	159	TFQGPPHGIQVER	63	99.994			Mascot
1502.8512	1502.6777	-0.1735	-115	165	177	YGRPLLGCTIKPK			Carbamidomethyl (C)[8]		Mascot
1505.7094	1505.547	-0.1624	-108	451	463	WSPELASACEVWK					Mascot
2169.9868	2169.7493	-0.2375	-109	195	213	GGLDFTKDDENVNSQPFMR					Mascot
2185.9819	2185.7402	-0.2417	-111	195	213	GGLDFTKDDENVNSQPFMR			Oxidation (M)[18]		Mascot
3854.8718	3854.4333	-0.4385	-114	42	79	VTPQPGVPPEEAGAAVAAESSTGTWTTVWTDGLTSLDR					Mascot

Rank	Protein Name	Accession No	Protein MW	Protein PI	Pep. Count	Protein Score	Protein Score C. I. %	Total Ion Score	Total Ion C. I. %
5	Ribulose bisphosphate carboxylase large chain (Fragment) OS=Salacia pallescens GN=rbcL PE=3 SV=2	RBL_SALPL	49365.3	6.25	12	147	100	84	100

Peptide Information

Calc. Mass	Obsrv. Mass	± da	± ppm	Start Seq.	End Seq.	Sequence	Ion Score	C. I. %	Modification	Rank	Result Type
898.41	898.3174	-0.0926	-103	306	312	NHGMHFR					Mascot
910.4451	910.3486	-0.0965	-106	188	194	AVYECLR			Carbamidomethyl (C)[5]		Mascot
914.4049	914.3092	-0.0957	-105	306	312	NHGMHFR			Oxidation (M)[4]		Mascot
921.4346	921.4083	-0.0263	-29	1	8	MSPQTETK					Mascot
1021.5312	1021.4243	-0.1069	-105	33	41	DTDILAAFR					Mascot
1045.5459	1045.4561	-0.0898	-86	422	431	VAVEACVQAR					Mascot
1284.7019	1284.577	-0.1249	-97	436	446	DLAREGNXIIR	21	11.285			Mascot
1393.6522	1393.504	-0.1482	-106	22	32	LTYYTPDYETK					Mascot
1465.7546	1465.5961	-0.1585	-108	147	159	TFQGPPHGIQVER					Mascot
1465.7546	1465.5961	-0.1585	-108	147	159	TFQGPPHGIQVER	63	99.994			Mascot
1501.7539	1501.6364	-0.1175	-78	422	435	VAVEACVQARNEGR					Mascot
1502.8512	1502.6777	-0.1735	-115	165	177	YGRPLLGCTIKPK			Carbamidomethyl (C)[8]		Mascot
2169.9868	2169.7493	-0.2375	-109	195	213	GGLDFTKDDENVNSQPFMR					Mascot
2185.9819	2185.7402	-0.2417	-111	195	213	GGLDFTKDDENVNSQPFMR			Oxidation (M)[18]		Mascot
3854.8718	3854.4333	-0.4385	-114	42	79	VTPQPGVPPEEAGAAVAAESSTGTWTTVWTDGLTSLDR					Mascot

Rank	Protein Name	Accession No	Protein MW	Protein PI	Pep. Count	Protein Score	Protein Score C. I. %	Total Ion Score	Total Ion C. I. %
6	Ribulose bisphosphate carboxylase large chain OS=Lotus japonicus GN=rbcL PE=3 SV=1	RBL_LOTJA	52739.5	6.04	15	145	100	61	99.992

Peptide Information

Calc. Mass	Obsrv. Mass	± da	± ppm	Start Seq.	End Seq.	Sequence	Ion Score	C. I. %	Modification	Rank	Result Type
898.41	898.3174	-0.0926	-103	306	312	NHGMHFR					Mascot
910.4451	910.3486	-0.0965	-106	188	194	AVYECLR			Carbamidomethyl (C)[5]		Mascot
914.4049	914.3092	-0.0957	-105	306	312	NHGMHFR			Oxidation (M)[4]		Mascot
921.4346	921.4083	-0.0263	-29	1	8	MSPQTETK					Mascot
1021.5312	1021.4243	-0.1069	-105	33	41	DTDILAAFR					Mascot
1116.583	1116.4625	-0.1205	-108	422	431	VALEACVQAR			Carbamidomethyl (C)[6]		Mascot
1170.5585	1170.5106	-0.0479	-41	304	312	QKNHGMHFR			Oxidation (M)[6]		Mascot
1187.6643	1187.532	-0.1323	-111	286	295	DNGLLLHIHR					Mascot
1245.6337	1245.496	-0.1377	-111	218	227	FLFCAEALFK			Carbamidomethyl (C)[4]		Mascot
1275.7307	1275.5936	-0.1371	-107	340	350	EITLGFVDLLR					Mascot
1393.6522	1393.504	-0.1482	-106	22	32	LTYYTPDYETK					Mascot
1465.7216	1465.5961	-0.1255	-86	320	334	MSGGDHIHAGTVVGK					Mascot
1465.7546	1465.5961	-0.1585	-108	147	159	TFQGPPHGIQVER	63	99.994			Mascot
1502.8512	1502.6777	-0.1735	-115	165	177	YGRPLLGCTIKPK			Carbamidomethyl (C)[8]		Mascot
2169.9868	2169.7493	-0.2375	-109	195	213	GGLDFTKDDENVNSQPFMR					Mascot
2185.9819	2185.7402	-0.2417	-111	195	213	GGLDFTKDDENVNSQPFMR			Oxidation (M)[18]		Mascot
3854.8718	3854.4333	-0.4385	-114	42	79	VTPQPGVPPEEAGAAVAAESSTGTWTTVWTDGLTSLDR					Mascot

Rank	Protein Name	Accession No	Protein MW	Protein PI	Pep. Count	Protein Score	Protein Score C. I. %	Total Ion Score	Total Ion C. I. %
7	Ribulose bisphosphate carboxylase large chain (Fragment) OS=Drymophloeus subdistichus GN=rbcL PE=3	RBL_DRYSU	52254.1	6.33	15	145	100	61	99.992

Peptide Information

Calc. Mass	Obsrv. Mass	± da	± ppm	Start Seq.	End Seq.	Sequence	Ion Score	C. I. %	Modification	Rank	Result Type
898.41	898.3174	-0.0926	-103	306	312	NHGMHFR					Mascot
910.4451	910.3486	-0.0965	-106	188	194	AVYECLR			Carbamidomethyl (C)[5]		Mascot
914.4049	914.3092	-0.0957	-105	306	312	NHGMHFR			Oxidation (M)[4]		Mascot
921.4346	921.4083	-0.0263	-29	1	8	MSPQTETK					Mascot
1021.5312	1021.4243	-0.1069	-105	33	41	DTDILAAFR					Mascot
1116.583	1116.4625	-0.1205	-108	422	431	VALEACVQAR			Carbamidomethyl (C)[6]		Mascot
1170.5585	1170.5106	-0.0479	-41	304	312	QKNHGMHFR			Oxidation (M)[6]		Mascot
1187.6643	1187.532	-0.1323	-111	286	295	DNGLLLHIHR					Mascot
1245.6433	1245.496	-0.1473	-118	440	450	EGNEIIREASK					Mascot
1393.6522	1393.504	-0.1482	-106	22	32	LTYYTPDYETK					Mascot
1465.7216	1465.5961	-0.1255	-86	320	334	MSGGDHIHAGTVVGK					Mascot
1465.7546	1465.5961	-0.1585	-108	147	159	TFQGPPHGIQVER	63	99.994			Mascot
1502.8512	1502.6777	-0.1735	-115	165	177	YGRPLLGCTIKPK			Carbamidomethyl (C)[8]		Mascot
1505.7457	1505.547	-0.1987	-132	216	227	DRFLFCTEALYK					Mascot
2169.9868	2169.7493	-0.2375	-109	195	213	GGLDFTKDDENVNSQPFMR					Mascot
2185.9819	2185.7402	-0.2417	-111	195	213	GGLDFTKDDENVNSQPFMR			Oxidation (M)[18]		Mascot
3854.8718	3854.4333	-0.4385	-114	42	79	VTPQPGVPPEEAGAAVAAESSTGTWTTVWTDGLTSLDR					Mascot

Rank	Protein Name	Accession No	Protein MW	Protein PI	Pep. Count	Protein Score	Protein Score C. I. %	Total Ion Score	Total Ion C. I. %
8	Ribulose bisphosphate carboxylase large chain (Fragment) OS=Manilkara zapota GN=rbcL PE=3 SV=2	RBL_MANZA	51550	6.04	14	143	100	63	99.994

Peptide Information

Calc. Mass	Obsrv. Mass	± da	± ppm	Start Seq.	End Seq.	Sequence	Ion Score	C. I. %	Modification	Rank	Result Type
898.41	898.3174	-0.0926	-103	296	302	NHGMHFR					Mascot
910.4451	910.3486	-0.0965	-106	178	184	AVYECLR			Carbamidomethyl (C)[5]		Mascot
914.4049	914.3092	-0.0957	-105	296	302	NHGMHFR			Oxidation (M)[4]		Mascot
1021.5312	1021.4243	-0.1069	-105	23	31	DTDILAAFR					Mascot
1039.4691	1039.415	-0.0541	-52	341	348	DDYVEKDR					Mascot
1116.583	1116.4625	-0.1205	-108	412	421	VALEACVQAR			Carbamidomethyl (C)[6]		Mascot
1170.5585	1170.5106	-0.0479	-41	294	302	QKNHGMHFR			Oxidation (M)[6]		Mascot
1187.6643	1187.532	-0.1323	-111	276	285	DNGLLLHIHR					Mascot
1245.6433	1245.496	-0.1473	-118	430	440	EGNEIIREASK					Mascot
1275.7307	1275.5936	-0.1371	-107	330	340	EITLGFVDLLR					Mascot
1393.6522	1393.504	-0.1482	-106	12	22	LTYYTPDYETK					Mascot
1465.7546	1465.5961	-0.1585	-108	137	149	TFQGPPHGIQVER					Mascot
1465.7546	1465.5961	-0.1585	-108	137	149	TFQGPPHGIQVER	63	99.994			Mascot
1502.8512	1502.6777	-0.1735	-115	155	167	YGRPLLGCTIKPK			Carbamidomethyl (C)[8]		Mascot
2169.9868	2169.7493	-0.2375	-109	185	203	GGLDFTKDDENVNSQPFMR					Mascot
2185.9819	2185.7402	-0.2417	-111	185	203	GGLDFTKDDENVNSQPFMR			Oxidation (M)[18]		Mascot
3854.8718	3854.4333	-0.4385	-114	32	69	VTPQPGVPPEEAGAAVAAESSTGTWTTVWTDGLTSLDR					Mascot

Rank	Protein Name	Accession No	Protein MW	Protein PI	Pep. Count	Protein Score	Protein Score C. I. %	Total Ion Score	Total Ion C. I. %
9	Ribulose bisphosphate carboxylase large chain OS=Phaseolus vulgaris GN=rbcL PE=3 SV=1	RBL_PHAVU	52767.6	5.96	14	141	100	63	99.994

Peptide Information

Calc. Mass	Obsrv. Mass	± da	± ppm	Start Seq.	End Seq.	Sequence	Ion Score	C. I. %	Modification	Rank	Result Type
898.41	898.3174	-0.0926	-103	306	312	NHGMHFR					Mascot
910.4451	910.3486	-0.0965	-106	188	194	AVYECLR			Carbamidomethyl (C)[5]		Mascot
914.4049	914.3092	-0.0957	-105	306	312	NHGMHFR			Oxidation (M)[4]		Mascot
921.4346	921.4083	-0.0263	-29	1	8	MSPQTETK					Mascot
1021.5312	1021.4243	-0.1069	-105	33	41	DTDILAAFR					Mascot
1116.583	1116.4625	-0.1205	-108	422	431	VALEACVQAR			Carbamidomethyl (C)[6]		Mascot
1170.5585	1170.5106	-0.0479	-41	304	312	QKNHGMHFR			Oxidation (M)[6]		Mascot
1187.6643	1187.532	-0.1323	-111	286	295	DNGLLLHIHR					Mascot
1245.6433	1245.496	-0.1473	-118	440	450	EGNEIIREASK					Mascot
1275.7307	1275.5936	-0.1371	-107	340	350	EITLGFVDLLR					Mascot
1393.6522	1393.504	-0.1482	-106	22	32	LTYYTPDYETK					Mascot
1465.7546	1465.5961	-0.1585	-108	147	159	TFQGPPHGIQVER					Mascot
1465.7546	1465.5961	-0.1585	-108	147	159	TFQGPPHGIQVER	63	99.994			Mascot
1502.8512	1502.6777	-0.1735	-115	165	177	YGRPLLGCTIKPK			Carbamidomethyl (C)[8]		Mascot
2169.9868	2169.7493	-0.2375	-109	195	213	GGLDFTKDDENVNSQPFMR					Mascot
2185.9819	2185.7402	-0.2417	-111	195	213	GGLDFTKDDENVNSQPFMR			Oxidation (M)[18]		Mascot
3854.8718	3854.4333	-0.4385	-114	42	79	VTPQPGVPPEEAGAAVAAESSTGTWTTVWTDGLTSLDR					Mascot

Rank	Protein Name	Accession No	Protein MW	Protein PI	Pep. Count	Protein Score	Protein Score C. I. %	Total Ion Score	Total Ion C. I. %
10	Ribulose bisphosphate carboxylase large chain (Fragment) OS=Sinapis alba GN=rbcL PE=3 SV=1	RBL_SINAL	52405.4	6.23	14	141	100	63	99.994

Peptide Information

Calc. Mass	Obsrv. Mass	± da	± ppm	Start Seq.	End Seq.	Sequence	Ion Score	C. I. %	Modification	Rank	Result Type
856.3546	856.4373	0.0827	97	467	473	FEFPAMD					Mascot
898.41	898.3174	-0.0926	-103	306	312	NHGMHFR					Mascot
910.4451	910.3486	-0.0965	-106	188	194	AVYECLR			Carbamidomethyl (C)[5]		Mascot
914.4049	914.3092	-0.0957	-105	306	312	NHGMHFR			Oxidation (M)[4]		Mascot
921.4346	921.4083	-0.0263	-29	1	8	MSPQTETK					Mascot
1021.5312	1021.4243	-0.1069	-105	33	41	DTDILAAFR					Mascot
1116.583	1116.4625	-0.1205	-108	422	431	VALEACVQAR			Carbamidomethyl (C)[6]		Mascot
1170.5585	1170.5106	-0.0479	-41	304	312	QKNHGMHFR			Oxidation (M)[6]		Mascot
1187.6643	1187.532	-0.1323	-111	286	295	DNGLLLHIHR					Mascot
1245.6433	1245.496	-0.1473	-118	440	450	EGNEIIREASK					Mascot
1275.7307	1275.5936	-0.1371	-107	340	350	EITLGFVDLLR					Mascot
1465.7546	1465.5961	-0.1585	-108	147	159	TFQGPPHGIQVER					Mascot
1465.7546	1465.5961	-0.1585	-108	147	159	TFQGPPHGIQVER	63	99.994			Mascot
	1465.5961										Mascot
1502.8512	1502.6777	-0.1735	-115	165	177	YGRPLLGCTIKPK			Carbamidomethyl (C)[8]		Mascot
2169.9868	2169.7493	-0.2375	-109	195	213	GGLDFTKDDENVNSQPFMR					Mascot
2185.9819	2185.7402	-0.2417	-111	195	213	GGLDFTKDDENVNSQPFMR			Oxidation (M)[18]		Mascot
3854.8718	3854.4333	-0.4385	-114	42	79	VTPQPGVPPEEAGAAVAAESSTGTWTTVWTDGLTSLDR					Mascot

Gel Idx/Pos	41/B14	Instr./Gel Origin	BA2060/131017A	Process Sta	Analysis Succeeded
Plate [#] Name	[1] 31496	Instrument Sample Name		Spectra	8

Rank	Protein Name	Accession No	Protein MW	Protein PI	Pep Count	Protein Score	Protein Score C. I. %	Total Ion Score	Total Ion C. I. %
1	RuBisCO large subunit-binding protein subunit be chloroplastic OS=Pisum sativum PE=1 SV=2	RUBB_PEA	62945.3	5.85	15	266	100	192	100

Peptide Information

Calc. Mass	Obsrv. Mass	± da	± ppm	Start Seq.	End Seq.	Sequence	Ion Score	C. I. %	Modification	Rank	Result Type
856.5363	856.4414	-0.0949	-111	122	129	LVRQAAAK					Mascot
1116.47	1116.4644	-0.0056	-5	259	267	MTVEFENCK			Oxidation (M)[1]		Mascot
1182.7092	1182.5869	-0.1223	-103	72	83	LADLVGVTLGPK					Mascot
1280.7684	1280.6357	-0.1327	-104	155	167	VVAAGANPVLITR					Mascot
1284.7158	1284.5839	-0.1319	-103	280	290	DLINILEDAIR	39	98.655			Mascot
1284.7158	1284.5839	-0.1319	-103	280	290	DLINILEDAIR					Mascot
1501.7614	1501.6444	-0.117	-78	550	563	VVRCCLEHASSVAK					Mascot
1505.7158	1505.5579	-0.1579	-105	246	258	GYISPYFVTDSEK					Mascot
1541.7693	1541.6017	-0.1676	-109	108	121	EVELEDPVENIGAK					Mascot
1700.8999	1700.7186	-0.1813	-107	453	469	AAVEEGIVVGGGCTLLR			Carbamidomethyl (C)[13]		Mascot
1700.8999	1700.7186	-0.1813	-107	453	469	AAVEEGIVVGGGCTLLR	53	99.95	Carbamidomethyl (C)[13]		Mascot
1834.9908	1834.7954	-0.1954	-106	334	350	SQYLDDIAILTGGTVIR					Mascot
1834.9908	1834.7954	-0.1954	-106	334	350	SQYLDDIAILTGGTVIR	65	99.997			Mascot
1891.9355	1891.7303	-0.2052	-108	376	393	DTTTIVGDGSTQEAVNKR					Mascot
1900.0386	1899.8325	-0.2061	-108	420	438	LSGGVAVIQVGAQTETELK					Mascot
1963.0858	1962.873	-0.2128	-108	333	350	KSQYLDDIAILTGGTVIR	35	96.868			Mascot
1963.0858	1962.873	-0.2128	-108	333	350	KSQYLDDIAILTGGTVIR					Mascot
2157.176	2156.946	-0.23	-107	420	440	LSGGVAVIQVGAQTETELKEK					Mascot
2430.2722	2430.002	-0.2702	-111	130	154	TNDLAGDGTTTSVVLAQGLIAEGVK					Mascot

Rank	Protein Name	Accession No	Protein MW	Protein PI	Pep Count	Protein Score	Protein Score C. I. %	Total Ion Score	Total Ion C. I. %
2	Ribulose bisphosphate carboxylase large chain OS=Sesbania sesban GN=rbcL PE=3 SV=1	RBL_SESSE	52358.4	6.09	16	115	100	19	0

Peptide Information

Calc. Mass	Obsrv. Mass	± da	± ppm	Start Seq.	End Seq.	Sequence	Ion Score	C. I. %	Modification	Rank	Result Type
856.3546	856.4414	0.0868	101	467	473	FEFPAMD					Mascot
898.41	898.3185	-0.0915	-102	306	312	NHGMHFR					Mascot
910.4451	910.3561	-0.089	-98	188	194	AVYECLR			Carbamidomethyl (C)[5]		Mascot
914.4049	914.3116	-0.0933	-102	306	312	NHGMHFR			Oxidation (M)[4]		Mascot
921.4346	921.4125	-0.0221	-24	1	8	MSPQTETK					Mascot
1021.5312	1021.43	-0.1012	-99	33	41	DTDILAAFR					Mascot
1039.4691	1039.4187	-0.0504	-48	351	358	DDYVEKDR					Mascot
1116.583	1116.4644	-0.1186	-106	422	431	VALEACVQAR			Carbamidomethyl (C)[6]		Mascot
1170.5585	1170.5168	-0.0417	-36	304	312	QKNHGMHFR			Oxidation (M)[6]		Mascot
1187.6643	1187.5428	-0.1215	-102	286	295	DNGLLLHIHR					Mascot
1245.6337	1245.4967	-0.137	-110	218	227	FLFCAEAIFK			Carbamidomethyl (C)[4]		Mascot
1275.7307	1275.6016	-0.1291	-101	340	350	EITLGFVDLLR					Mascot
1393.6522	1393.5057	-0.1465	-105	22	32	LTYYTPDYETK					Mascot
1465.7546	1465.5997	-0.1549	-106	147	159	TFQGPPHGIQVER	19	0			Mascot
1465.7546	1465.5997	-0.1549	-106	147	159	TFQGPPHGIQVER					Mascot
1502.8512	1502.6748	-0.1764	-117	165	177	YGRPLLGCTIKPK			Carbamidomethyl (C)[8]		Mascot
2169.9868	2169.7502	-0.2366	-109	195	213	GGLDFTKDDENVNSQPFMR					Mascot
3854.8718	3854.4534	-0.4184	-109	42	79	VTPQPGVPPEEAGAAVAAESSTGTWTTVWTDGLTSLDR					Mascot

Rank	Protein Name	Accession No	Protein MW	Protein PI	Pep Count	Protein Score	Protein Score C. I. %	Total Ion Score	Total Ion C. I. %
3	Ribulose bisphosphate carboxylase large chain (Fragment) OS=Prunus domestica GN=rbcL PE=3 SV=1	RBL_PRUDO	50914.6	6.23	15	105	100	18	0

Peptide Information

Calc. Mass	Obsrv. Mass	± da	± ppm	Start Seq.	End Seq.	Sequence	Ion Score	C. I. %	Modification	Rank	Result Type
898.41	898.3185	-0.0915	-102	296	302	NHGMHFR					Mascot
910.4451	910.3561	-0.089	-98	178	184	AVYECLR			Carbamidomethyl (C)[5]		Mascot
914.4049	914.3116	-0.0933	-102	296	302	NHGMHFR			Oxidation (M)[4]		Mascot
1021.5312	1021.43	-0.1012	-99	23	31	DTDILAAFR					Mascot
1116.583	1116.4644	-0.1186	-106	412	421	VALEACVQAR			Carbamidomethyl (C)[6]		Mascot
1170.5585	1170.5168	-0.0417	-36	294	302	QKNHGMHFR			Oxidation (M)[6]		Mascot
1187.6643	1187.5428	-0.1215	-102	276	285	DNGLLLHIHR					Mascot
1245.6433	1245.4967	-0.1466	-118	430	440	EGNEIIREASK					Mascot
1275.7307	1275.6016	-0.1291	-101	330	340	EITLGFVDLLR					Mascot
1393.6522	1393.5057	-0.1465	-105	12	22	LTYYTPDYETK					Mascot
1465.7216	1465.5997	-0.1219	-83	310	324	MSGGDHIHAGTVVGK					Mascot
1465.7546	1465.5997	-0.1549	-106	137	149	TFQGPPHGIQVER	19	0			Mascot
1502.8512	1502.6748	-0.1764	-117	155	167	YGRPLLGCTIKPK			Carbamidomethyl (C)[8]		Mascot
1830.7455	1830.6361	-0.1094	-60	227	242	GHYLNATAGTCEDMMK			Carbamidomethyl (C)[11], Oxidation (M)[14,15]		Mascot
2169.9868	2169.7502	-0.2366	-109	185	203	GGLDFTKDDENVNSQPFMR					Mascot
3854.8718	3854.4534	-0.4184	-109	32	69	VTPQPGVPPEEAGAAVAAESSTGTWTTVWTDGLTSLDR					Mascot

Rank	Protein Name	Accession No	Protein MW	Protein PI	Pep Count	Protein Score	Protein Score C. I. %	Total Ion Score	Total Ion C. I. %
4	Ribulose bisphosphate carboxylase large chain (Fragment) OS=Serenoa repens GN=rbcL PE=3 SV=1	RBL_SERRE	51794	6.33	15	104	100	18	0

Peptide Information

Calc. Mass	Obsrv. Mass	± da	± ppm	Start Seq.	End Seq.	Sequence	Ion Score	C. I. %	Modification	Rank	Result Type
898.41	898.3185	-0.0915	-102	306	312	NHGMHFR					Mascot
910.4451	910.3561	-0.089	-98	188	194	AVYECLR			Carbamidomethyl (C)[5]		Mascot
914.4049	914.3116	-0.0933	-102	306	312	NHGMHFR			Oxidation (M)[4]		Mascot
921.4346	921.4125	-0.0221	-24	1	8	MSPQTETK					Mascot
1021.5312	1021.43	-0.1012	-99	33	41	DTDILAAFR					Mascot
1116.583	1116.4644	-0.1186	-106	422	431	VALEACVQAR			Carbamidomethyl (C)[6]		Mascot
1170.5585	1170.5168	-0.0417	-36	304	312	QKNHGMHFR			Oxidation (M)[6]		Mascot
1187.6643	1187.5428	-0.1215	-102	286	295	DNGLLLHIHR					Mascot
1245.6433	1245.4967	-0.1466	-118	440	450	EGNEIIREASK					Mascot
1393.6522	1393.5057	-0.1465	-105	22	32	LTYYTPDYETK					Mascot
1465.7216	1465.5997	-0.1219	-83	320	334	MSGGDHIHAGTVVGK					Mascot
1465.7546	1465.5997	-0.1549	-106	147	159	TFQGPPHGIQVER	19	0			Mascot
1502.8512	1502.6748	-0.1764	-117	165	177	YGRPLLGCTIKPK			Carbamidomethyl (C)[8]		Mascot
1505.7094	1505.5579	-0.1515	-101	451	463	WSPELASACEVWK					Mascot
2169.9868	2169.7502	-0.2366	-109	195	213	GGLDFTKDDENVNSQPFMR					Mascot
3854.8718	3854.4534	-0.4184	-109	42	79	VTPQPGVPPEEAGAAVAAESSTGTWTTVWTDGLTSLDR					Mascot

Rank	Protein Name	Accession No	Protein MW	Protein PI	Pep Count	Protein Score	Protein Score C. I. %	Total Ion Score	Total Ion C. I. %
5	Ribulose bisphosphate carboxylase large chain OS=Lotus japonicus GN=rbcL PE=3 SV=1	RBL_LOTJA	52739.5	6.04	15	101	100	18	0

Peptide Information

Calc. Mass	Obsrv. Mass	± da	± ppm	Start Seq.	End Seq.	Sequence	Ion Score	C. I. %	Modification	Rank	Result Type
898.41	898.3185	-0.0915	-102	306	312	NHGMHFR					Mascot
910.4451	910.3561	-0.089	-98	188	194	AVYECLR			Carbamidomethyl (C)[5]		Mascot
914.4049	914.3116	-0.0933	-102	306	312	NHGMHFR			Oxidation (M)[4]		Mascot
921.4346	921.4125	-0.0221	-24	1	8	MSPQTETK					Mascot
1021.5312	1021.43	-0.1012	-99	33	41	DTDILAAFR					Mascot
1116.583	1116.4644	-0.1186	-106	422	431	VALEACVQAR			Carbamidomethyl (C)[6]		Mascot
1170.5585	1170.5168	-0.0417	-36	304	312	QKNHGMHFR			Oxidation (M)[6]		Mascot
1187.6643	1187.5428	-0.1215	-102	286	295	DNGLLLHIHR					Mascot
1245.6337	1245.4967	-0.137	-110	218	227	FLFCAEALFK			Carbamidomethyl (C)[4]		Mascot
1275.7307	1275.6016	-0.1291	-101	340	350	EITLGFVDLLR					Mascot
1393.6522	1393.5057	-0.1465	-105	22	32	LTYYTPDYETK					Mascot
1465.7216	1465.5997	-0.1219	-83	320	334	MSGGDHIHAGTVVGK					Mascot
1465.7546	1465.5997	-0.1549	-106	147	159	TFQGPPHGIQVER	19	0			Mascot
1502.8512	1502.6748	-0.1764	-117	165	177	YGRPLLGCTIKPK			Carbamidomethyl (C)[8]		Mascot
2169.9868	2169.7502	-0.2366	-109	195	213	GGLDFTKDDENVNSQPFMR					Mascot
3854.8718	3854.4534	-0.4184	-109	42	79	VTPQPGVPPEEAGAAVAAESSTGTWTTVWTDGLTSLDR					Mascot

Rank	Protein Name	Accession No	Protein MW	Protein PI	Pep Count	Protein Score	Protein Score C. I. %	Total Ion Score	Total Ion C. I. %
6	Ribulose bisphosphate carboxylase large chain (Fragment) OS=Drymophloeus subdistichus GN=rbcL PE=3	RBL_DRYSU	52254.1	6.33	15	101	100	18	0

Peptide Information

Calc. Mass	Obsrv. Mass	± da	± ppm	Start Seq.	End Seq.	Sequence	Ion Score	C. I. %	Modification	Rank	Result Type
898.41	898.3185	-0.0915	-102	306	312	NHGMHFR					Mascot
910.4451	910.3561	-0.089	-98	188	194	AVYECLR			Carbamidomethyl (C)[5]		Mascot
914.4049	914.3116	-0.0933	-102	306	312	NHGMHFR			Oxidation (M)[4]		Mascot
921.4346	921.4125	-0.0221	-24	1	8	MSPQTETK					Mascot
1021.5312	1021.43	-0.1012	-99	33	41	DTDILAAFR					Mascot
1116.583	1116.4644	-0.1186	-106	422	431	VALEACVQAR			Carbamidomethyl (C)[6]		Mascot
1170.5585	1170.5168	-0.0417	-36	304	312	QKNHGMHFR			Oxidation (M)[6]		Mascot
1187.6643	1187.5428	-0.1215	-102	286	295	DNGLLLHIHR					Mascot
1245.6433	1245.4967	-0.1466	-118	440	450	EGNEIIREASK					Mascot
1393.6522	1393.5057	-0.1465	-105	22	32	LTYYTPDYETK					Mascot
1465.7216	1465.5997	-0.1219	-83	320	334	MSGGDHIHAGTVVGK					Mascot
1465.7546	1465.5997	-0.1549	-106	147	159	TFQGPPHGIQVER	19	0			Mascot
1502.8512	1502.6748	-0.1764	-117	165	177	YGRPLLGCTIKPK			Carbamidomethyl (C)[8]		Mascot
1505.7457	1505.5579	-0.1878	-125	216	227	DRFLFCTEALYK					Mascot
2169.9868	2169.7502	-0.2366	-109	195	213	GGLDFTKDDENVNSQPFMR					Mascot
3854.8718	3854.4534	-0.4184	-109	42	79	VTPQPGVPPEEAGAAVAAESSTGTWTTVWTDGLTSLDR					Mascot

Rank	Protein Name	Accession No	Protein MW	Protein PI	Pep Count	Protein Score	Protein Score C. I. %	Total Ion Score	Total Ion C. I. %
7	Ribulose bisphosphate carboxylase large chain (Fragment) OS=Manilkara zapota GN=rbcL PE=3 SV=2	RBL_MANZA	51550	6.04	14	98	100	19	0

Peptide Information

Calc. Mass	Obsrv. Mass	± da	± ppm	Start Seq.	End Seq.	Sequence	Ion Score	C. I. %	Modification	Rank	Result Type
898.41	898.3185	-0.0915	-102	296	302	NHGMHFR					Mascot
910.4451	910.3561	-0.089	-98	178	184	AVYECLR			Carbamidomethyl (C)[5]		Mascot
914.4049	914.3116	-0.0933	-102	296	302	NHGMHFR			Oxidation (M)[4]		Mascot
1021.5312	1021.43	-0.1012	-99	23	31	DTDILAAFR					Mascot
1039.4691	1039.4187	-0.0504	-48	341	348	DDYVEKDR					Mascot
1116.583	1116.4644	-0.1186	-106	412	421	VALEACVQAR			Carbamidomethyl (C)[6]		Mascot
1170.5585	1170.5168	-0.0417	-36	294	302	QKNHGMHFR			Oxidation (M)[6]		Mascot
1187.6643	1187.5428	-0.1215	-102	276	285	DNGLLLHIHR					Mascot
1245.6433	1245.4967	-0.1466	-118	430	440	EGNEIIREASK					Mascot
1275.7307	1275.6016	-0.1291	-101	330	340	EITLGFVDLLR					Mascot
1393.6522	1393.5057	-0.1465	-105	12	22	LTYYTPDYETK					Mascot
1465.7546	1465.5997	-0.1549	-106	137	149	TFQGPPHGIQVER	19	0			Mascot
1465.7546	1465.5997	-0.1549	-106	137	149	TFQGPPHGIQVER					Mascot
1502.8512	1502.6748	-0.1764	-117	155	167	YGRPLLGCTIKPK			Carbamidomethyl (C)[8]		Mascot
2169.9868	2169.7502	-0.2366	-109	185	203	GGLDFTKDDENVNSQPFMR					Mascot
3854.8718	3854.4534	-0.4184	-109	32	69	VTPQPGVPPEEAGAAVAAESSTGTWTTVWTDGLTSLDR					Mascot

Rank	Protein Name	Accession No	Protein MW	Protein PI	Pep Count	Protein Score	Protein Score C. I. %	Total Ion Score	Total Ion C. I. %
8	RuBisCO large subunit-binding protein subunit be chloroplastic (Fragment) OS=Secale cereale GN=C	RUBB_SECCI	53379.1	4.88	7	97	100	75	100

Peptide Information

Calc. Mass	Obsrv. Mass	± da	± ppm	Start Seq.	End Seq.	Sequence	Ion Score	C. I. %	Modification	Rank	Result Type
856.5363	856.4414	-0.0949	-111	27	34	LVRQAAAK					Mascot
1245.5714	1245.4967	-0.0747	-60	458	468	CCLEHAASVAK			Carbamidomethyl (C)[1,2]		Mascot
1284.7158	1284.5839	-0.1319	-103	185	195	DLINVLEEAIR	22	32.263			Mascot
1284.7158	1284.5839	-0.1319	-103	185	195	DLINVLEEAIR					Mascot
1505.7158	1505.5579	-0.1579	-105	151	163	GYISPYFVTDSEK					Mascot
1541.7693	1541.6017	-0.1676	-109	13	26	EVELEDPVENIGAK					Mascot
1700.8999	1700.7186	-0.1813	-107	358	374	AAVEEGIVVGGGCTLLR			Carbamidomethyl (C)[13]		Mascot
1700.8999	1700.7186	-0.1813	-107	358	374	AAVEEGIVVGGGCTLLR	53	99.95	Carbamidomethyl (C)[13]		Mascot
2430.2722	2430.002	-0.2702	-111	35	59	TNDLAGDGTTTSVVLAQGLIAEGVK					Mascot

Rank	Protein Name	Accession No	Protein MW	Protein PI	Pep Count	Protein Score	Protein Score C. I. %	Total Ion Score	Total Ion C. I. %
9	Ribulose bisphosphate carboxylase large chain OS=Phaseolus vulgaris GN=rbcL PE=3 SV=1	RBL_PHAVU	52767.6	5.96	14	97	100	19	0

Peptide Information

Calc. Mass	Obsrv. Mass	± da	± ppm	Start Seq.	End Seq.	Sequence	Ion Score	C. I. %	Modification	Rank	Result Type
898.41	898.3185	-0.0915	-102	306	312	NHGMHFR					Mascot
910.4451	910.3561	-0.089	-98	188	194	AVYECLR			Carbamidomethyl (C)[5]		Mascot
914.4049	914.3116	-0.0933	-102	306	312	NHGMHFR			Oxidation (M)[4]		Mascot
921.4346	921.4125	-0.0221	-24	1	8	MSPQTETK					Mascot
1021.5312	1021.43	-0.1012	-99	33	41	DTDILAAFR					Mascot
1116.583	1116.4644	-0.1186	-106	422	431	VALEACVQAR			Carbamidomethyl (C)[6]		Mascot
1170.5585	1170.5168	-0.0417	-36	304	312	QKNHGMHFR			Oxidation (M)[6]		Mascot
1187.6643	1187.5428	-0.1215	-102	286	295	DNGLLLHIHR					Mascot
1245.6433	1245.4967	-0.1466	-118	440	450	EGNEIIREASK					Mascot
1275.7307	1275.6016	-0.1291	-101	340	350	EITLGFVDLLR					Mascot
1393.6522	1393.5057	-0.1465	-105	22	32	LTYYTPDYETK					Mascot
1465.7546	1465.5997	-0.1549	-106	147	159	TFQGPPHGIQVER	19	0			Mascot
1465.7546	1465.5997	-0.1549	-106	147	159	TFQGPPHGIQVER					Mascot
1502.8512	1502.6748	-0.1764	-117	165	177	YGRPLLGCTIKPK			Carbamidomethyl (C)[8]		Mascot
2169.9868	2169.7502	-0.2366	-109	195	213	GGLDFTKDDENVNSQPFMR					Mascot
3854.8718	3854.4534	-0.4184	-109	42	79	VTPQPGVPPEEAGAAVAAESSTGTWTTVWTDGLTSLDR					Mascot

Rank	Protein Name	Accession No	Protein MW	Protein PI	Pep Count	Protein Score	Protein Score C. I. %	Total Ion Score	Total Ion C. I. %
10	Ribulose bisphosphate carboxylase large chain (Fragment) OS=Sinapis alba GN=rbcL PE=3 SV=1	RBL_SINAL	52405.4	6.23	14	97	100	19	0

Peptide Information

Calc. Mass	Obsrv. Mass	± da	± ppm	Start Seq.	End Seq.	Sequence	Ion Score	C. I. %	Modification	Rank	Result Type
856.3546	856.4414	0.0868	101	467	473	FEFPAMD					Mascot
898.41	898.3185	-0.0915	-102	306	312	NHGMHFR					Mascot
910.4451	910.3561	-0.089	-98	188	194	AVYECLR			Carbamidomethyl (C)[5]		Mascot
914.4049	914.3116	-0.0933	-102	306	312	NHGMHFR			Oxidation (M)[4]		Mascot
921.4346	921.4125	-0.0221	-24	1	8	MSPQTETK					Mascot
1021.5312	1021.43	-0.1012	-99	33	41	DTDILAAFR					Mascot
1116.583	1116.4644	-0.1186	-106	422	431	VALEACVQAR			Carbamidomethyl (C)[6]		Mascot
1170.5585	1170.5168	-0.0417	-36	304	312	QKNHGMHFR			Oxidation (M)[6]		Mascot
1187.6643	1187.5428	-0.1215	-102	286	295	DNGLLLHIHR					Mascot
1245.6433	1245.4967	-0.1466	-118	440	450	EGNEIIREASK					Mascot
1275.7307	1275.6016	-0.1291	-101	340	350	EITLGFVDLLR					Mascot
1465.7546	1465.5997	-0.1549	-106	147	159	TFQGPPHGIQVER	19	0			Mascot
1465.7546	1465.5997	-0.1549	-106	147	159	TFQGPPHGIQVER					Mascot
1502.8512	1502.6748	-0.1764	-117	165	177	YGRPLLGCTIKPK			Carbamidomethyl (C)[8]		Mascot
2169.9868	2169.7502	-0.2366	-109	195	213	GGLDFTKDDENVNSQPFMR					Mascot
3854.8718	3854.4534	-0.4184	-109	42	79	VTPQPGVPPEEAGAAVAAESSTGTWTTVWTDGLTSLDR					Mascot

Gel Idx/Pos	42/B15	Instr./Gel Origin	BA2060/131017A	Process Sta	Analysis Succeeded
Plate [#] Name	[1] 31496	Instrument Sample Name		Spectra	8

Rank	Protein Name	Accession No	Protein MW	Protein PI	Pep. Count	Protein Score	Protein Score C. I. %	Total Ion Score	Total Ion C. I. %
1	RuBisCO large subunit-binding protein subunit al (Fragment) OS=Ricinus communis PE=2 SV=1	RUBA_RICC	52346.9	4.77	14	286	100	210	100

Peptide Information

Calc. Mass	Obsrv. Mass	± da	± ppm	Start Seq.	End Seq.	Sequence	Ion Score	C. I. %	Modification	Rank	Result Type
898.572	898.4874	-0.0846	-94	256	264	GILNVAAIK					Mascot
1182.6841	1182.5729	-0.1112	-94	11	22	LADAVGLTLGPR					Mascot
1182.6841	1182.5729	-0.1112	-94	11	22	LADAVGLTLGPR	69	99.999			Mascot
1204.6208	1204.502	-0.1188	-99	25	35	NVVLDEFGSPK					Mascot
1290.6172	1290.4935	-0.1237	-96	368	379	VGAATETELEDR					Mascot
1479.7478	1479.6031	-0.1447	-98	185	197	GYISPQFVTNPEK					Mascot
1479.7478	1479.6031	-0.1447	-98	185	197	GYISPQFVTNPEK	47	99.817			Mascot
1517.7845	1517.6279	-0.1566	-103	474	487	YENLVEAGVIDPAK					Mascot
1555.9054	1555.7405	-0.1649	-106	89	104	LGLLSVTSGANPVSIK					Mascot
1612.9169	1612.7373	-0.1796	-111	256	271	GILNVAAIKAPGFGER					Mascot
1636.7772	1636.6196	-0.1576	-96	68	84	TNDSAGDGTTTASVLAR					Mascot
1712.0065	1711.8313	-0.1752	-102	89	105	LGLLSVTSGANPVSIKR					Mascot
1780.9261	1780.7455	-0.1806	-101	46	62	AIELPDPMENAGAALIR					Mascot
1780.9261	1780.7455	-0.1806	-101	46	62	AIELPDPMENAGAALIR	63	99.996			Mascot
1796.921	1796.7388	-0.1822	-101	46	62	AIELPDPMENAGAALIR			Oxidation (M)[8]		Mascot
1904.9559	1904.7562	-0.1997	-105	315	332	DSTTLIADAASKDELQAR					Mascot
2250.2954	2250.061	-0.2344	-104	232	253	APLLIIAEDVTGEALATLVVNK					Mascot
3098.4358	3098.095	-0.3408	-110	156	184	VGPDGVLSIESSSSFETTVEVEEGMEIDR	30	91.089			Mascot
3098.4358	3098.095	-0.3408	-110	156	184	VGPDGVLSIESSSSFETTVEVEEGMEIDR					Mascot

Rank	Protein Name	Accession No	Protein MW	Protein PI	Pep. Count	Protein Score	Protein Score C. I. %	Total Ion Score	Total Ion C. I. %
2	RuBisCO large subunit-binding protein subunit al chloroplastic (Fragment) OS=Brassica napus PE=	RUB1_BRAN	57656.8	4.84	14	275	100	203	100

Peptide Information

Calc. Mass	Obsrv. Mass	± da	± ppm	Start Seq.	End Seq.	Sequence	Ion Score	C. I. %	Modification	Rank	Result Type
898.572	898.4874	-0.0846	-94	273	281	GVLNVVAVK					Mascot
1043.5844	1043.4858	-0.0986	-94	53	62	VVNDGVTIAR					Mascot
1090.5891	1090.4858	-0.1033	-95	215	223	LLVEFENAR					Mascot
1090.5891	1090.4858	-0.1033	-95	215	223	LLVEFENAR	57	99.982			Mascot
1182.6841	1182.5729	-0.1112	-94	28	39	LADAVGLTLGPR					Mascot
1182.6841	1182.5729	-0.1112	-94	28	39	LADAVGLTLGPR	69	99.999			Mascot
1204.6208	1204.502	-0.1188	-99	42	52	NVVLDEFGSPK					Mascot
1290.6172	1290.4935	-0.1237	-96	385	396	VGAATETELEDR					Mascot
1479.7478	1479.6031	-0.1447	-98	202	214	GYISPQFVTNPEK					Mascot
1479.7478	1479.6031	-0.1447	-98	202	214	GYISPQFVTNPEK	47	99.817			Mascot
1501.6541	1501.6433	-0.0108	-7	356	368	ELSETDSVYDSEK					Mascot
1612.9169	1612.7373	-0.1796	-111	273	288	GVLNVVAVKAPGFGER					Mascot
1636.7772	1636.6196	-0.1576	-96	85	101	TNDSAGDGTTTASVLAR					Mascot
1904.9559	1904.7562	-0.1997	-105	332	349	DSTTLIADAASKDELQAR					Mascot
2250.2954	2250.061	-0.2344	-104	249	270	APLLIIAEDVTGEALATLVVNK					Mascot
3098.4358	3098.095	-0.3408	-110	173	201	VGPDGVLSIESSSSFETTVEVEEGMEIDR	30	91.089			Mascot
3098.4358	3098.095	-0.3408	-110	173	201	VGPDGVLSIESSSSFETTVEVEEGMEIDR					Mascot
3808.8083	3808.4446	-0.3637	-95	475	507	IMFSEWEIGYNAMTDTYENLLEAGVIDPAKVTR			Oxidation (M)[2,13]		Mascot

Rank	Protein Name	Accession No	Protein MW	Protein PI	Pep. Count	Protein Score	Protein Score C. I. %	Total Ion Score	Total Ion C. I. %
3	RuBisCO large subunit-binding protein subunit al chloroplastic OS=Brassica napus PE=2 SV=1	RUB2_BRAN	61567.8	5.14	13	234	100	173	100

Peptide Information

Calc. Mass	Obsrv. Mass	± da	± ppm	Start Seq.	End Seq.	Sequence	Ion Score	C. I. %	Modification	Rank	Result Type
856.5111	856.4473	-0.0638	-74	178	185	SRPVKGGR					Mascot
898.572	898.4874	-0.0846	-94	312	320	GVLNVVAVK					Mascot
1043.5844	1043.4858	-0.0986	-94	92	101	VVNDGVTIAR					Mascot
1090.5891	1090.4858	-0.1033	-95	254	262	LLVEFENAR					Mascot
1090.5891	1090.4858	-0.1033	-95	254	262	LLVEFENAR	57	99.982			Mascot
1182.6841	1182.5729	-0.1112	-94	67	78	LADAVGLTLGPR					Mascot
1182.6841	1182.5729	-0.1112	-94	67	78	LADAVGLTLGPR	69	99.999			Mascot
1204.6208	1204.502	-0.1188	-99	81	91	NVVLDEFGSPK					Mascot
1290.6172	1290.4935	-0.1237	-96	423	434	VGAATETELEDR					Mascot
1479.7478	1479.6031	-0.1447	-98	241	253	GYISPQFVTNPEK					Mascot
1479.7478	1479.6031	-0.1447	-98	241	253	GYISPQFVTNPEK	47	99.817			Mascot
1612.9169	1612.7373	-0.1796	-111	312	327	GVLNVVAVKAPGFGER					Mascot
1636.7772	1636.6196	-0.1576	-96	124	140	TNDSAGDGTTTASVLAR					Mascot
2250.2954	2250.061	-0.2344	-104	288	309	APLLIIAEDVTGEALATLVVNK					Mascot
3098.3994	3098.095	-0.3044	-98	212	240	VGPDGVSPIESSSSFETTVEVEEGMEIDR			Oxidation (M)[25]		Mascot
3098.3994	3098.095	-0.3044	-98	212	240	VGPDGVSPIESSSSFETTVEVEEGMEIDR			Oxidation (M)[25]		Mascot
3808.8083	3808.4446	-0.3637	-95	512	544	IMFSEWELGYNAMTDTYENLLEAGVIDPAKVTR			Oxidation (M)[2,13]		Mascot

Rank	Protein Name	Accession No	Protein MW	Protein PI	Pep. Count	Protein Score	Protein Score C. I. %	Total Ion Score	Total Ion C. I. %
4	RuBisCO large subunit-binding protein subunit al chloroplastic OS=Pisum sativum PE=1 SV=2	RUBA_PEA	61941.1	5.15	8	203	100	180	100

Peptide Information

Calc. Mass	Obsrv. Mass	± da	± ppm	Start Seq.	End Seq.	Sequence	Ion Score	C. I. %	Modification	Rank	Result Type
898.572	898.4874	-0.0846	-94	314	322	GILNVAAIK					Mascot
1043.5844	1043.4858	-0.0986	-94	94	103	VVNDGVTIAR					Mascot
1182.6841	1182.5729	-0.1112	-94	69	80	LADAVGLTLGPR					Mascot
1182.6841	1182.5729	-0.1112	-94	69	80	LADAVGLTLGPR	69	99.999			Mascot
1204.6208	1204.502	-0.1188	-99	83	93	NVVLDEFGSPK					Mascot
1290.6172	1290.4935	-0.1237	-96	426	437	VGAATETELEDR					Mascot
1479.7478	1479.6031	-0.1447	-98	243	255	GYISPQFVTNPEK					Mascot
1479.7478	1479.6031	-0.1447	-98	243	255	GYISPQFVTNPEK	47	99.817			Mascot
1612.9169	1612.7373	-0.1796	-111	314	329	GILNVAAIKAPGFGER					Mascot
1780.9261	1780.7455	-0.1806	-101	104	120	AIELPDPMENAGAALIR					Mascot
1780.9261	1780.7455	-0.1806	-101	104	120	AIELPDPMENAGAALIR	63	99.996			Mascot
1796.921	1796.7388	-0.1822	-101	104	120	AIELPDPMENAGAALIR			Oxidation (M)[8]		Mascot

Rank	Protein Name	Accession No	Protein MW	Protein PI	Pep. Count	Protein Score	Protein Score C. I. %	Total Ion Score	Total Ion C. I. %
5	Chaperonin 60 subunit alpha 1, chloroplastic OS=Arabidopsis thaliana GN=CPN60A1 PE=1 SV=2	CPNA1_ARAT	62033.8	5.09	12	165	100	112	100

Peptide Information

Calc. Mass	Obsrv. Mass	± da	± ppm	Start Seq.	End Seq.	Sequence	Ion Score	C. I. %	Modification	Rank	Result Type
898.572	898.4874	-0.0846	-94	313	321	GVLNVVAVK					Mascot
1043.5844	1043.4858	-0.0986	-94	93	102	VVNDGVTIAR					Mascot
1102.5276	1102.422	-0.1056	-96	50	58	EIAFDQHSR	35	97.13			Mascot
1102.5276	1102.422	-0.1056	-96	50	58	EIAFDQHSR					Mascot
1204.6208	1204.502	-0.1188	-99	82	92	NVVLDEFGSPK					Mascot
1290.6172	1290.4935	-0.1237	-96	425	436	VGAATETELEDR					Mascot
1479.7478	1479.6031	-0.1447	-98	242	254	GYISPQFVTNPEK					Mascot
1479.7478	1479.6031	-0.1447	-98	242	254	GYISPQFVTNPEK	47	99.817			Mascot
1612.9169	1612.7373	-0.1796	-111	313	328	GVLNVVAVKAPGFGER					Mascot
1711.8102	1711.8313	0.0211	12	1	17	MASANALSSASVLCSSR			Carbamidomethyl (C)[14]		Mascot
1904.9559	1904.7562	-0.1997	-105	372	389	DSTTLIADAASKDELQAR					Mascot
2250.2954	2250.061	-0.2344	-104	289	310	APLLIIAEDVTGEALATLVVNK					Mascot
3098.4358	3098.095	-0.3408	-110	213	241	VGPDGVLSIESSSSFETTVEVEEGMEIDR	30	91.089			Mascot
3098.4358	3098.095	-0.3408	-110	213	241	VGPDGVLSIESSSSFETTVEVEEGMEIDR					Mascot
3795.9216	3795.3962	-0.5254	-138	331	365	AMLQDIAILTGAEYLAMDMSLLVENATIDQLGIAR			Oxidation (M)[2,17]		Mascot

Rank	Protein Name	Accession No	Protein MW	Protein PI	Pep. Count	Protein Score	Protein Score C. I. %	Total Ion Score	Total Ion C. I. %
6	Ribulose bisphosphate carboxylase large chain (Fragment) OS=Cornus obliqua GN=rbcL PE=3 SV=2	RBL_COROB	51536.8	6	11	54	85.649		

Peptide Information

Calc. Mass	Obsrv. Mass	± da	± ppm	Start Seq.	End Seq.	Sequence	Ion Score	C. I. %	Modification	Rank	Result Type
898.41	898.4874	0.0774	86	296	302	NHGMHFR					Mascot
1021.5312	1021.436	-0.0952	-93	23	31	DTDILAAFR					Mascot
1102.4762	1102.422	-0.0542	-49	457	465	FEFEAMDTL					Mascot
1102.4762	1102.422	-0.0542	-49	457	465	FEFEAMDTL					Mascot
1116.583	1116.4811	-0.1019	-91	412	421	VALEACVQAR			Carbamidomethyl (C)[6]		Mascot
1204.6071	1204.502	-0.1051	-87	208	217	FLFCAEAIYK					Mascot
1275.7307	1275.6091	-0.1216	-95	330	340	EITLGFVDLLR					Mascot
1465.7546	1465.6122	-0.1424	-97	137	149	TFQGPPHGIQVER					Mascot
1918.9368	1918.772	-0.1648	-86	437	453	EASKWSPELAAACEIWK					Mascot
2169.9868	2169.7664	-0.2204	-102	185	203	GGLDFTKDDENVNSQPFMR					Mascot
3022.4548	3022.1521	-0.3027	-100	249	275	ELGVPIVMHDYLTGGFTANTSLAHYCR			Carbamidomethyl (C)[26]		Mascot
3854.8718	3854.4412	-0.4306	-112	32	69	VTPQPGVPPEEAGAAVAAESSTGTWTTVWTDGLTSLDR					Mascot

Rank	Protein Name	Accession No	Protein MW	Protein PI	Pep. Count	Protein Score	Protein Score C. I. %	Total Ion Score	Total Ion C. I. %
7	Ribulose bisphosphate carboxylase large chain OS=Liquidambar styraciflua GN=rbcL PE=3 SV=1	RBL_LIQST	52625.5	6.04	11	53	82.344		

Peptide Information

Calc. Mass	Obsrv. Mass	± da	± ppm	Start Seq.	End Seq.	Sequence	Ion Score	C. I. %	Modification	Rank	Result Type
898.41	898.4874	0.0774	86	306	312	NHGMHFR					Mascot
1021.5312	1021.436	-0.0952	-93	33	41	DTDILAAFR					Mascot
1102.4762	1102.422	-0.0542	-49	467	475	FEFEAMDTL					Mascot
1102.4762	1102.422	-0.0542	-49	467	475	FEFEAMDTL					Mascot
1116.583	1116.4811	-0.1019	-91	422	431	VALEACVQAR			Carbamidomethyl (C)[6]		Mascot
1204.6071	1204.502	-0.1051	-87	218	227	FLFCAEAIYK					Mascot
1275.7307	1275.6091	-0.1216	-95	340	350	EITLGFVDLLR					Mascot
1465.7216	1465.6122	-0.1094	-75	320	334	MSGGDHIHAGTVVGK					Mascot
1904.921	1904.7562	-0.1648	-87	447	463	EASKWSPELAAACEVWK					Mascot
2169.9868	2169.7664	-0.2204	-102	195	213	GGLDFTKDDENVNSQPFMR					Mascot
3022.4548	3022.1521	-0.3027	-100	259	285	ELGVPIVMHDYLTGGFTANTSLAHYCR			Carbamidomethyl (C)[26]		Mascot
3854.8718	3854.4412	-0.4306	-112	42	79	VTPQPGVPPEEAGAAVAAESSTGTWTTVWTDGLTSLDR					Mascot

Rank	Protein Name	Accession No	Protein MW	Protein PI	Pep. Count	Protein Score	Protein Score C. I. %	Total Ion Score	Total Ion C. I. %
8	Ribulose bisphosphate carboxylase large chain (Fragment) OS=Sinapis alba GN=rbcL PE=3 SV=1	RBL_SINAL	52405.4	6.23	11	52	80.19		

Peptide Information

Calc. Mass	Obsrv. Mass	± da	± ppm	Start Seq.	End Seq.	Sequence	Ion Score	C. I. %	Modification	Rank	Result Type
856.3546	856.4473	0.0927	108	467	473	FEFPAMD					Mascot
898.41	898.4874	0.0774	86	306	312	NHGMHFR					Mascot
1021.5312	1021.436	-0.0952	-93	33	41	DTDILAAFR					Mascot
1116.583	1116.4811	-0.1019	-91	422	431	VALEACVQAR			Carbamidomethyl (C)[6]		Mascot
1204.6071	1204.502	-0.1051	-87	218	227	FLFCAEAIYK					Mascot
1275.7307	1275.6091	-0.1216	-95	340	350	EITLGFVDLLR					Mascot
1465.7546	1465.6122	-0.1424	-97	147	159	TFQGPPHGIQVER					Mascot
1904.921	1904.7562	-0.1648	-87	447	463	EASKWSPELAAACEVWK					Mascot
2169.9868	2169.7664	-0.2204	-102	195	213	GGLDFTKDDENVNSQPFMR					Mascot
3022.4548	3022.1521	-0.3027	-100	259	285	ELGVPIVMHDYLTGGFTANTSLAHYCR			Carbamidomethyl (C)[26]		Mascot
3854.8718	3854.4412	-0.4306	-112	42	79	VTPQPGVPPEEAGAAVAAESSTGTWTTVWTDGLTSLDR					Mascot

Rank	Protein Name	Accession No	Protein MW	Protein PI	Pep. Count	Protein Score	Protein Score C. I. %	Total Ion Score	Total Ion C. I. %
9	Ribulose bisphosphate carboxylase large chain (Fragment) OS=Salacia pallescens GN=rbcL PE=3 SV=2	RBL_SALPL	49365.3	6.25	10	47	34.401		

Peptide Information

Calc. Mass	Obsrv. Mass	± da	± ppm	Start Seq.	End Seq.	Sequence	Ion Score	C. I. %	Modification	Rank	Result Type
898.41	898.4874	0.0774	86	306	312	NHGMHFR					Mascot
1021.5312	1021.436	-0.0952	-93	33	41	DTDILAAFR					Mascot
1045.5459	1045.4717	-0.0742	-71	422	431	VAVEACVQAR					Mascot
1102.5487	1102.422	-0.1267	-115	440	449	EGNXIIREAS	2	0			Mascot
1102.5674	1102.422	-0.1454	-132	422	431	VAVEACVQAR			Carbamidomethyl (C)[6]		Mascot
1204.6071	1204.502	-0.1051	-87	218	227	FLFCAEALYK					Mascot
1465.7546	1465.6122	-0.1424	-97	147	159	TFQGPPHGIQVER					Mascot
1501.7539	1501.6433	-0.1106	-74	422	435	VAVEACVQARNEGR					Mascot
2169.9868	2169.7664	-0.2204	-102	195	213	GGLDFTKDDENVNSQPFMR					Mascot
3022.4548	3022.1521	-0.3027	-100	259	285	ELGVPIVMHDYLTGGFTANTSLAHYCR			Carbamidomethyl (C)[26]		Mascot
3854.8718	3854.4412	-0.4306	-112	42	79	VTPQPGVPPEEAGAAVAAESSTGTWTTVWTDGLTSLDR					Mascot

Rank	Protein Name	Accession No	Protein MW	Protein PI	Pep. Count	Protein Score	Protein Score C. I. %	Total Ion Score	Total Ion C. I. %
10	Ribulose bisphosphate carboxylase large chain (Fragment) OS=Aphelandra sinclairiana GN=rbcL PE=3 SV	RBL_APHSI	51555	6	10	46	24.683		

Peptide Information

Calc. Mass	Obsrv. Mass	± da	± ppm	Start Seq.	End Seq.	Sequence	Ion Score	C. I. %	Modification	Rank	Result Type
898.41	898.4874	0.0774	86	296	302	NHGMHFR					Mascot
1021.5312	1021.436	-0.0952	-93	23	31	DTDILAAFR					Mascot
1045.5459	1045.4717	-0.0742	-71	412	421	VAVEACVQAR					Mascot
1102.5674	1102.422	-0.1454	-132	412	421	VAVEACVQAR			Carbamidomethyl (C)[6]		Mascot
1102.5674	1102.422	-0.1454	-132	412	421	VAVEACVQAR			Carbamidomethyl (C)[6]		Mascot
1204.6071	1204.502	-0.1051	-87	208	217	FLFCAEAIYK					Mascot
1465.7546	1465.6122	-0.1424	-97	137	149	TFQGPPHGIQVER					Mascot
1501.7539	1501.6433	-0.1106	-74	412	425	VAVEACVQARNEGR					Mascot
1904.921	1904.7562	-0.1648	-87	437	453	EASKWSPELAAACEVWK					Mascot
2169.9868	2169.7664	-0.2204	-102	185	203	GGLDFTKDDENVNSQPFMR					Mascot
3022.4548	3022.1521	-0.3027	-100	249	275	ELGVPIVMHDYLTGGFTANTSLAHYCR			Carbamidomethyl (C)[26]		Mascot
3854.8718	3854.4412	-0.4306	-112	32	69	VTPQPGVPPEEAGAAVAAESSTGTWTTVWTDGLTSLDR					Mascot

Gel Idx/Pos	43/B16	Instr./Gel Origin	BA2060/131017A	Process Sta	Analysis Succeeded
Plate [#] Name	[1] 31496	Instrument Sample Name		Spectra	8

Rank	Protein Name	Accession No	Protein MW	Protein PI	Pep. Count	Protein Score	Protein Score C. I. %	Total Ion Score	Total Ion C. I. %
1	ATP synthase subunit alpha, chloroplastic OS=G max GN=atpA PE=3 SV=1	ATPA SOYBI	55719.3	5.15	22	673	100	502	100

Peptide Information

Calc. Mass	Obsrv. Mass	± da	± ppm	Start Seq.	End Seq.	Sequence	Ion Score	C. I. %	Modification	Rank	Result Type
815.4621	815.3939	-0.0682	-84	166	172	ELIIGDR					Mascot
860.5022	860.4275	-0.0747	-87	273	279	QMSLLLR					Mascot
876.4971	876.4198	-0.0773	-88	273	279	QMSLLLR			Oxidation (M)[2]		Mascot
901.485	901.4083	-0.0767	-85	406	413	ATQNQLAR					Mascot
904.5614	904.4821	-0.0793	-88	456	462	KFLVELR					Mascot
1123.563	1123.4662	-0.0968	-86	17	25	IEQYNTEVK					Mascot
1207.6569	1207.5474	-0.1095	-91	481	491	TFTGEAEVLLK					Mascot
1252.726	1252.6146	-0.1114	-89	129	140	LIESPAPGIISR					Mascot
1252.726	1252.6146	-0.1114	-89	129	140	LIESPAPGIISR	58	99.982			Mascot
1266.7528	1266.637	-0.1158	-91	108	119	VINALAKPIDGR					Mascot
1317.705	1317.5841	-0.1209	-92	256	266	HTLIIYDDLSK					Mascot
1323.7056	1323.5865	-0.1191	-90	463	473	AYLNTNKPQFK					Mascot
1408.7067	1408.5785	-0.1282	-91	15	25	ERIEQYNTEVK					Mascot
1416.7845	1416.6559	-0.1286	-91	95	107	IAQIPVSEAYLGR	98	100			Mascot
1416.7845	1416.6559	-0.1286	-91	95	107	IAQIPVSEAYLGR					Mascot
1501.7969	1501.6606	-0.1363	-91	203	216	ASSVAQVVNTLQER					Mascot
1501.7969	1501.6606	-0.1363	-91	203	216	ASSVAQVVNTLQER	40	98.745			Mascot
1553.7383	1553.5946	-0.1437	-92	285	297	EAYPGDVFYLHSR	96	100			Mascot
1553.7383	1553.5946	-0.1437	-92	285	297	EAYPGDVFYLHSR					Mascot
1612.9016	1612.7528	-0.1488	-92	26	41	IVNTGTVLQVGDGIAR	91	100			Mascot
1612.9016	1612.7528	-0.1488	-92	26	41	IVNTGTVLQVGDGIAR					Mascot
2033.0405	2032.8477	-0.1928	-95	71	90	NVGVVLMGDGLMIQEGSSVK					Mascot
2049.0354	2048.8467	-0.1887	-92	71	90	NVGVVLMGDGLMIQEGSSVK			Oxidation (M)[7]		Mascot
2065.0303	2064.8408	-0.1895	-92	71	90	NVGVVLMGDGLMIQEGSSVK			Oxidation (M)[7,12]		Mascot
2205.1108	2204.8987	-0.2121	-96	492	509	EAIQEQMELFLLQEQVEK					Mascot
2221.1057	2220.9019	-0.2038	-92	492	509	EAIQEQMELFLLQEQVEK			Oxidation (M)[7]		Mascot
2273.2209	2273.0005	-0.2204	-97	142	162	SVYEPLQTGLIAIDSMIPIGR	120	100			Mascot
2273.2209	2273.0005	-0.2204	-97	142	162	SVYEPLQTGLIAIDSMIPIGR					Mascot
2289.2158	2288.9995	-0.2163	-94	142	162	SVYEPLQTGLIAIDSMIPIGR			Oxidation (M)[16]		Mascot
2319.1536	2318.9309	-0.2227	-96	492	510	EAIQEQMELFLLQEQVEKN					Mascot
2335.1484	2334.9282	-0.2202	-94	492	510	EAIQEQMELFLLQEQVEKN			Oxidation (M)[7]		Mascot
2429.322	2429.0889	-0.2331	-96	141	162	RSVYEPLQTGLIAIDSMIPIGR					Mascot
2804.4609	2804.2092	-0.2517	-90	177	202	TAVATDTILNQQGQNVICVYVAIGQK			Carbamidomethyl (C)[18]		Mascot
3623.8438	3623.4707	-0.3731	-103	423	455	QSQSAPLTVEEQIITIYTGTNGYLDSLEIGQVR					Mascot

Rank	Protein Name	Accession No	Protein MW	Protein PI	Pep. Count	Protein Score	Protein Score C. I. %	Total Ion Score	Total Ion C. I. %
2	ATP synthase subunit alpha, chloroplastic OS=Eucalyptus globulus subsp. globulus GN=atpA PE=3 SV=1	ATPA EUCGI	55478.1	5.15	16	599	100	502	100

Peptide Information

Calc. Mass	Obsrv. Mass	± da	± ppm	Start Seq.	End Seq.	Sequence	Ion Score	C. I. %	Modification	Rank	Result Type
815.4621	815.3939	-0.0682	-84	166	172	ELIIGDR					Mascot
860.5022	860.4275	-0.0747	-87	273	279	QMSLLLR					Mascot
876.4971	876.4198	-0.0773	-88	273	279	QMSLLLR			Oxidation (M)[2]		Mascot
901.485	901.4083	-0.0767	-85	406	413	ATQNQLAR					Mascot
904.5614	904.4821	-0.0793	-88	456	462	KFLVELR					Mascot
1252.726	1252.6146	-0.1114	-89	129	140	LIESPAPGIISR					Mascot
1252.726	1252.6146	-0.1114	-89	129	140	LIESPAPGIISR	58	99.982			Mascot
1266.7528	1266.637	-0.1158	-91	108	119	VINALAKPIDGR					Mascot
1317.705	1317.5841	-0.1209	-92	256	266	HTLIIYDDLSK					Mascot
1416.7845	1416.6559	-0.1286	-91	95	107	IAQIPVSEAYLGR	98	100			Mascot
1416.7845	1416.6559	-0.1286	-91	95	107	IAQIPVSEAYLGR					Mascot
1501.7969	1501.6606	-0.1363	-91	203	216	ASSVAQVVNTLQER					Mascot
1501.7969	1501.6606	-0.1363	-91	203	216	ASSVAQVVNTLQER	40	98.745			Mascot
1553.7383	1553.5946	-0.1437	-92	285	297	EAYPGDVFYLHSR	96	100			Mascot
1553.7383	1553.5946	-0.1437	-92	285	297	EAYPGDVFYLHSR					Mascot
1612.9016	1612.7528	-0.1488	-92	26	41	IVNTGTVLQVGDGIAR	91	100			Mascot
1612.9016	1612.7528	-0.1488	-92	26	41	IVNTGTVLQVGDGIAR					Mascot
2065.05	2064.8408	-0.2092	-101	285	301	EAYPGDVFYLHSRLLER					Mascot
2273.2209	2273.0005	-0.2204	-97	142	162	SVYEPLQTGLIAIDSMIPIGR	120	100			Mascot
2273.2209	2273.0005	-0.2204	-97	142	162	SVYEPLQTGLIAIDSMIPIGR					Mascot
2289.2158	2288.9995	-0.2163	-94	142	162	SVYEPLQTGLIAIDSMIPIGR			Oxidation (M)[16]		Mascot
2356.1707	2355.9639	-0.2068	-88	385	405	LELAQFAELEAFAQFASDLDK					Mascot
2429.322	2429.0889	-0.2331	-96	141	162	RSVYEPLQTGLIAIDSMIPIGR					Mascot
2804.4609	2804.2092	-0.2517	-90	177	202	TAVATDTILNQQGQNVICVYVAIGQK			Carbamidomethyl (C)[18]		Mascot

Rank	Protein Name	Accession No	Protein MW	Protein PI	Pep. Count	Protein Score	Protein Score C. I. %	Total Ion Score	Total Ion C. I. %
3	ATP synthase subunit alpha, chloroplastic OS=L sativa GN=atpA PE=3 SV=1	ATPA LACSA	55484.2	5.27	16	559	100	462	100

Peptide Information

Calc. Mass	Obsrv. Mass	± da	± ppm	Start Seq.	End Seq.	Sequence	Ion Score	C. I. %	Modification	Rank	Result Type
815.4621	815.3939	-0.0682	-84	166	172	ELIIGDR					Mascot
860.5022	860.4275	-0.0747	-87	273	279	QMSLLLR					Mascot
876.4971	876.4198	-0.0773	-88	273	279	QMSLLLR			Oxidation (M)[2]		Mascot
901.485	901.4083	-0.0767	-85	406	413	ATQNQLAR					Mascot
904.5614	904.4821	-0.0793	-88	456	462	KFLVELR					Mascot
1252.726	1252.6146	-0.1114	-89	129	140	LIESPAPGIISR					Mascot
1252.726	1252.6146	-0.1114	-89	129	140	LIESPAPGIISR	58	99.982			Mascot
1266.7528	1266.637	-0.1158	-91	108	119	VINALAKPIDGR					Mascot
1275.6216	1275.6167	-0.0049	-4	256	266	HTSIIYDDPSK					Mascot
1275.6216	1275.6167	-0.0049	-4	256	266	HTSIIYDDPSK					Mascot
1416.7845	1416.6559	-0.1286	-91	95	107	IAQIPVSEAYLGR	98	100			Mascot
1416.7845	1416.6559	-0.1286	-91	95	107	IAQIPVSEAYLGR					Mascot
1535.7812	1535.5864	-0.1948	-127	203	216	ASSVAQVVTNFQER					Mascot
1553.7383	1553.5946	-0.1437	-92	285	297	EAYPGDVFYLHSR	96	100			Mascot
1553.7383	1553.5946	-0.1437	-92	285	297	EAYPGDVFYLHSR					Mascot
1612.9016	1612.7528	-0.1488	-92	26	41	IVNTGTVLQVGDGIAR	91	100			Mascot
1612.9016	1612.7528	-0.1488	-92	26	41	IVNTGTVLQVGDGIAR					Mascot
2065.05	2064.8408	-0.2092	-101	285	301	EAYPGDVFYLHSRLLER					Mascot
2273.2209	2273.0005	-0.2204	-97	142	162	SVYEPLQTGLIAIDSMIPIGR	120	100			Mascot
2273.2209	2273.0005	-0.2204	-97	142	162	SVYEPLQTGLIAIDSMIPIGR					Mascot
2289.2158	2288.9995	-0.2163	-94	142	162	SVYEPLQTGLIAIDSMIPIGR			Oxidation (M)[16]		Mascot
2356.1707	2355.9639	-0.2068	-88	385	405	LELAQFAELEAFAQFASDLDK					Mascot
2429.322	2429.0889	-0.2331	-96	141	162	RSVYEPLQTGLIAIDSMIPIGR					Mascot
2804.4973	2804.2092	-0.2881	-103	177	202	TAVATDTILNQQGKNVICVYVAIGQK			Carbamidomethyl (C)[18]		Mascot

Rank	Protein Name	Accession No	Protein MW	Protein PI	Pep. Count	Protein Score	Protein Score C. I. %	Total Ion Score	Total Ion C. I. %
4	ATP synthase subunit alpha, chloroplastic OS=Gossypium hirsutum GN=atpA PE=3 SV=1	ATPA GOSHI	55379.9	5.25	16	559	100	462	100

Peptide Information

Calc. Mass	Obsrv. Mass	± da	± ppm	Start Seq.	End Seq.	Sequence	Ion Score	C. I. %	Modification	Rank	Result Type
815.4621	815.3939	-0.0682	-84	166	172	ELIIGDR					Mascot
860.5022	860.4275	-0.0747	-87	273	279	QMSLLLR					Mascot
876.4971	876.4198	-0.0773	-88	273	279	QMSLLLR			Oxidation (M)[2]		Mascot
901.485	901.4083	-0.0767	-85	406	413	ATQNQLAR					Mascot
904.5614	904.4821	-0.0793	-88	456	462	KFLVELR					Mascot
1252.726	1252.6146	-0.1114	-89	129	140	LIESPAPGIISR					Mascot
1252.726	1252.6146	-0.1114	-89	129	140	LIESPAPGIISR	58	99.982			Mascot
1266.7528	1266.637	-0.1158	-91	108	119	VINALAKPIDGR					Mascot
1317.705	1317.5841	-0.1209	-92	256	266	HTLIIYDDLSK					Mascot
1408.6638	1408.5785	-0.0853	-61	492	502	DAIQDQMERFR					Mascot
1416.7845	1416.6559	-0.1286	-91	95	107	IAQIPVSEAYLGR	98	100			Mascot
1416.7845	1416.6559	-0.1286	-91	95	107	IAQIPVSEAYLGR					Mascot
1553.7383	1553.5946	-0.1437	-92	285	297	EAYPGDVFYLHSR	96	100			Mascot
1553.7383	1553.5946	-0.1437	-92	285	297	EAYPGDVFYLHSR					Mascot
1612.9016	1612.7528	-0.1488	-92	26	41	IVNTGTVLQVGDGIAR	91	100			Mascot
1612.9016	1612.7528	-0.1488	-92	26	41	IVNTGTVLQVGDGIAR					Mascot
2065.05	2064.8408	-0.2092	-101	285	301	EAYPGDVFYLHSRLLER					Mascot
2273.2209	2273.0005	-0.2204	-97	142	162	SVYEPLQTGLIAIDSMIPIGR	120	100			Mascot
2273.2209	2273.0005	-0.2204	-97	142	162	SVYEPLQTGLIAIDSMIPIGR					Mascot
2289.2158	2288.9995	-0.2163	-94	142	162	SVYEPLQTGLIAIDSMIPIGR			Oxidation (M)[16]		Mascot
2356.1707	2355.9639	-0.2068	-88	385	405	LELAQFAELEAFAQFASDLDK					Mascot
2429.322	2429.0889	-0.2331	-96	141	162	RSVYEPLQTGLIAIDSMIPIGR					Mascot
2804.4609	2804.2092	-0.2517	-90	177	202	TAVATDTILNQQGQNVICVYVAIGQK			Carbamidomethyl (C)[18]		Mascot

Rank	Protein Name	Accession No	Protein MW	Protein PI	Pep. Count	Protein Score	Protein Score C. I. %	Total Ion Score	Total Ion C. I. %
5	ATP synthase subunit alpha, chloroplastic OS=P trichocarpa GN=atpA PE=3 SV=1	ATPA POPTI	55288	5.2	16	558	100	462	100

Peptide Information

Calc. Mass	Obsrv. Mass	± da	± ppm	Start Seq.	End Seq.	Sequence	Ion Score	C. I. %	Modification	Rank	Result Type
815.4621	815.3939	-0.0682	-84	166	172	ELIIGDR					Mascot
860.5022	860.4275	-0.0747	-87	273	279	QMSLLLR					Mascot
876.4825	876.4198	-0.0627	-72	501	507	FLLQEQV					Mascot
901.485	901.4083	-0.0767	-85	406	413	ATQNQLAR					Mascot
904.5614	904.4821	-0.0793	-88	456	462	KFLVELR					Mascot
1252.726	1252.6146	-0.1114	-89	129	140	LIESPAPGIISR					Mascot
1252.726	1252.6146	-0.1114	-89	129	140	LIESPAPGIISR	58	99.982			Mascot
1266.7528	1266.637	-0.1158	-91	108	119	VINALAKPIDGR					Mascot
1317.705	1317.5841	-0.1209	-92	256	266	HTLIIYDDLSK					Mascot
1416.7845	1416.6559	-0.1286	-91	95	107	IAQIPVSEAYLGR	98	100			Mascot
1416.7845	1416.6559	-0.1286	-91	95	107	IAQIPVSEAYLGR					Mascot
1553.7383	1553.5946	-0.1437	-92	285	297	EAYPGDVFYLHSR	96	100			Mascot
1553.7383	1553.5946	-0.1437	-92	285	297	EAYPGDVFYLHSR					Mascot
1612.9016	1612.7528	-0.1488	-92	26	41	IVNTGTVLQVGDGIAR	91	100			Mascot
1612.9016	1612.7528	-0.1488	-92	26	41	IVNTGTVLQVGDGIAR					Mascot
2065.05	2064.8408	-0.2092	-101	285	301	EAYPGDVFYLHSRLLER					Mascot
2273.2209	2273.0005	-0.2204	-97	142	162	SVYEPLQTGLIAIDSMIPIGR	120	100			Mascot
2273.2209	2273.0005	-0.2204	-97	142	162	SVYEPLQTGLIAIDSMIPIGR					Mascot
2289.2158	2288.9995	-0.2163	-94	142	162	SVYEPLQTGLIAIDSMIPIGR			Oxidation (M)[16]		Mascot
2356.1707	2355.9639	-0.2068	-88	385	405	LELAQFAELEAFAQFASDLDK					Mascot
2429.322	2429.0889	-0.2331	-96	141	162	RSVYEPLQTGLIAIDSMIPIGR					Mascot
2804.4609	2804.2092	-0.2517	-90	177	202	TAVATDTILNQQGQNVICVYVAIGQK			Carbamidomethyl (C)[18]		Mascot

Rank	Protein Name	Accession No	Protein MW	Protein PI	Pep. Count	Protein Score	Protein Score C. I. %	Total Ion Score	Total Ion C. I. %
6	ATP synthase subunit alpha, chloroplastic OS=Helianthus annuus GN=atpA PE=3 SV=1	ATPA HELAN	55482.2	5.2	15	549	100	462	100

Peptide Information

Calc. Mass	Obsrv. Mass	± da	± ppm	Start Seq.	End Seq.	Sequence	Ion Score	C. I. %	Modification	Rank	Result Type
815.4621	815.3939	-0.0682	-84	166	172	ELIIGDR					Mascot
860.5022	860.4275	-0.0747	-87	273	279	QMSLLLR					Mascot
876.4971	876.4198	-0.0773	-88	273	279	QMSLLLR			Oxidation (M)[2]		Mascot
901.485	901.4083	-0.0767	-85	406	413	ATQNQLAR					Mascot
904.5614	904.4821	-0.0793	-88	456	462	KFLVELR					Mascot
1252.726	1252.6146	-0.1114	-89	129	140	LIESPAPGIISR					Mascot
1252.726	1252.6146	-0.1114	-89	129	140	LIESPAPGIISR	58	99.982			Mascot
1266.7528	1266.637	-0.1158	-91	108	119	VINALAKPIDGR					Mascot
1317.705	1317.5841	-0.1209	-92	256	266	HTLIIYDDLSK					Mascot
1416.7845	1416.6559	-0.1286	-91	95	107	IAQIPVSEAYLGR	98	100			Mascot
1416.7845	1416.6559	-0.1286	-91	95	107	IAQIPVSEAYLGR					Mascot
1553.7383	1553.5946	-0.1437	-92	285	297	EAYPGDVFYLHSR	96	100			Mascot
1553.7383	1553.5946	-0.1437	-92	285	297	EAYPGDVFYLHSR					Mascot
1612.9016	1612.7528	-0.1488	-92	26	41	IVNTGTVLQVGDGIAR	91	100			Mascot
1612.9016	1612.7528	-0.1488	-92	26	41	IVNTGTVLQVGDGIAR					Mascot
2065.05	2064.8408	-0.2092	-101	285	301	EAYPGDVFYLHSRLLER					Mascot
2273.2209	2273.0005	-0.2204	-97	142	162	SVYEPLQTGLIAIDSMIPIGR	120	100			Mascot
2273.2209	2273.0005	-0.2204	-97	142	162	SVYEPLQTGLIAIDSMIPIGR					Mascot
2289.2158	2288.9995	-0.2163	-94	142	162	SVYEPLQTGLIAIDSMIPIGR			Oxidation (M)[16]		Mascot
2356.1707	2355.9639	-0.2068	-88	385	405	LELAQFAELEAFAQFASDLDK					Mascot
2429.322	2429.0889	-0.2331	-96	141	162	RSVYEPLQTGLIAIDSMIPIGR					Mascot
2804.4609	2804.2092	-0.2517	-90	177	202	TAVATDTILNQQGQNVICVYVAIGQK			Carbamidomethyl (C)[18]		Mascot

Rank	Protein Name	Accession No	Protein MW	Protein PI	Pep. Count	Protein Score	Protein Score C. I. %	Total Ion Score	Total Ion C. I. %
7	ATP synthase subunit alpha, chloroplastic OS=D carota GN=atpA PE=3 SV=1	ATPA DAUCA	55451.2	5.34	15	546	100	460	100

Peptide Information

Calc. Mass	Obsrv. Mass	± da	± ppm	Start Seq.	End Seq.	Sequence	Ion Score	C. I. %	Modification	Rank	Result Type
815.4621	815.3939	-0.0682	-84	166	172	ELIIGDR					Mascot
860.5022	860.4275	-0.0747	-87	273	279	QMSLLLR					Mascot
876.4825	876.4198	-0.0627	-72	501	507	FLLQEQV					Mascot
901.485	901.4083	-0.0767	-85	406	413	ATQNQLAR					Mascot
904.5614	904.4821	-0.0793	-88	456	462	KFLVELR					Mascot
1252.726	1252.6146	-0.1114	-89	129	140	LIESPAPGIISR					Mascot
1252.726	1252.6146	-0.1114	-89	129	140	LIESPAPGIISR	58	99.982			Mascot
1416.7845	1416.6559	-0.1286	-91	95	107	IAQIPVSEAYLGR	98	100			Mascot
1416.7845	1416.6559	-0.1286	-91	95	107	IAQIPVSEAYLGR					Mascot
1535.7812	1535.5864	-0.1948	-127	203	216	ASSVAQVVTNFQER					Mascot
1553.7383	1553.5946	-0.1437	-92	285	297	EAYPGDVFYLHSR	96	100			Mascot
1553.7383	1553.5946	-0.1437	-92	285	297	EAYPGDVFYLHSR					Mascot
1612.9016	1612.7528	-0.1488	-92	26	41	IVNTGTVLQVGDGIAR	91	100			Mascot
1612.9016	1612.7528	-0.1488	-92	26	41	IVNTGTVLQVGDGIAR					Mascot
2065.05	2064.8408	-0.2092	-101	285	301	EAYPGDVFYLHSRLLER					Mascot
2273.2209	2273.0005	-0.2204	-97	142	162	SVYEPLQTGLIAIDSMIPIGR	120	100			Mascot
2273.2209	2273.0005	-0.2204	-97	142	162	SVYEPLQTGLIAIDSMIPIGR					Mascot
2289.2158	2288.9995	-0.2163	-94	142	162	SVYEPLQTGLIAIDSMIPIGR			Oxidation (M)[16]		Mascot
2356.1707	2355.9639	-0.2068	-88	385	405	LELAQFAELEAFAQFASDLDK					Mascot
2429.322	2429.0889	-0.2331	-96	141	162	RSVYEPLQTGLIAIDSMIPIGR					Mascot
2804.4609	2804.2092	-0.2517	-90	177	202	TAVATDTILNQQGQNVICVYVAIGQK			Carbamidomethyl (C)[18]		Mascot

Rank	Protein Name	Accession No	Protein MW	Protein PI	Pep. Count	Protein Score	Protein Score C. I. %	Total Ion Score	Total Ion C. I. %
8	ATP synthase subunit alpha, chloroplastic OS=Amborella trichopoda GN=atpA PE=3 SV=1	ATPA AMBTC	55329	5.25	14	540	100	462	100

Peptide Information

Calc. Mass	Obsrv. Mass	± da	± ppm	Start Seq.	End Seq.	Sequence	Ion Score	C. I. %	Modification	Rank	Result Type
815.4621	815.3939	-0.0682	-84	166	172	ELIIGDR					Mascot
860.5022	860.4275	-0.0747	-87	273	279	QMSLLLR					Mascot
876.4971	876.4198	-0.0773	-88	273	279	QMSLLLR			Oxidation (M)[2]		Mascot
901.485	901.4083	-0.0767	-85	406	413	ATQNQLAR					Mascot
1252.726	1252.6146	-0.1114	-89	129	140	LIESPAPGIISR					Mascot
1252.726	1252.6146	-0.1114	-89	129	140	LIESPAPGIISR	58	99.982			Mascot
1266.7528	1266.637	-0.1158	-91	108	119	VINALAKPIDGR					Mascot
1275.6692	1275.6167	-0.0525	-41	256	266	HTLIIHDDPSK					Mascot
1275.6692	1275.6167	-0.0525	-41	256	266	HTLIIHDDPSK					Mascot
1416.7845	1416.6559	-0.1286	-91	95	107	IAQIPVSEAYLGR	98	100			Mascot
1416.7845	1416.6559	-0.1286	-91	95	107	IAQIPVSEAYLGR					Mascot
1553.7383	1553.5946	-0.1437	-92	285	297	EAYPGDVFYLHSR	96	100			Mascot
1553.7383	1553.5946	-0.1437	-92	285	297	EAYPGDVFYLHSR					Mascot
1612.9016	1612.7528	-0.1488	-92	26	41	IVNTGTVLQVGDGIAR	91	100			Mascot
1612.9016	1612.7528	-0.1488	-92	26	41	IVNTGTVLQVGDGIAR					Mascot
2065.05	2064.8408	-0.2092	-101	285	301	EAYPGDVFYLHSRLLER					Mascot
2273.2209	2273.0005	-0.2204	-97	142	162	SVYEPLQTGLIAIDSMIPIGR	120	100			Mascot
2273.2209	2273.0005	-0.2204	-97	142	162	SVYEPLQTGLIAIDSMIPIGR					Mascot
2289.2158	2288.9995	-0.2163	-94	142	162	SVYEPLQTGLIAIDSMIPIGR			Oxidation (M)[16]		Mascot
2356.1707	2355.9639	-0.2068	-88	385	405	LELAQFAELEAFAQFASDLDK					Mascot
2429.322	2429.0889	-0.2331	-96	141	162	RSVYEPLQTGLIAIDSMIPIGR					Mascot
2804.4973	2804.2092	-0.2881	-103	177	202	TAVATDTILNQKGQNVICVYVAIGQK			Carbamidomethyl (C)[18]		Mascot

Rank	Protein Name	Accession No	Protein MW	Protein PI	Pep. Count	Protein Score	Protein Score C. I. %	Total Ion Score	Total Ion C. I. %
9	ATP synthase subunit alpha, chloroplastic OS=C arabica GN=atpA PE=3 SV=1	ATPA COFAI	55233.8	5.19	14	540	100	462	100

Peptide Information

Calc. Mass	Obsrv. Mass	± da	± ppm	Start Seq.	End Seq.	Sequence	Ion Score	C. I. %	Modification	Rank	Result Type
815.4621	815.3939	-0.0682	-84	166	172	ELIIGDR					Mascot
860.5022	860.4275	-0.0747	-87	273	279	QMSLLLR					Mascot
876.4971	876.4198	-0.0773	-88	273	279	QMSLLLR			Oxidation (M)[2]		Mascot
901.485	901.4083	-0.0767	-85	406	413	ATQNQLAR					Mascot
1252.726	1252.6146	-0.1114	-89	129	140	LIESPAPGIISR					Mascot
1252.726	1252.6146	-0.1114	-89	129	140	LIESPAPGIISR	58	99.982			Mascot
1266.7528	1266.637	-0.1158	-91	108	119	VINALAKPIDGR					Mascot
1275.6216	1275.6167	-0.0049	-4	256	266	HTSIIYDDPSK					Mascot
1275.6216	1275.6167	-0.0049	-4	256	266	HTSIIYDDPSK					Mascot
1416.7845	1416.6559	-0.1286	-91	95	107	IAQIPVSEAYLGR	98	100			Mascot
1416.7845	1416.6559	-0.1286	-91	95	107	IAQIPVSEAYLGR					Mascot
1553.7383	1553.5946	-0.1437	-92	285	297	EAYPGDVFYLHSR	96	100			Mascot
1553.7383	1553.5946	-0.1437	-92	285	297	EAYPGDVFYLHSR					Mascot
1612.9016	1612.7528	-0.1488	-92	26	41	IVNTGTVLQVGDGIAR	91	100			Mascot
1612.9016	1612.7528	-0.1488	-92	26	41	IVNTGTVLQVGDGIAR					Mascot
2065.05	2064.8408	-0.2092	-101	285	301	EAYPGDVFYLHSRLLER					Mascot
2273.2209	2273.0005	-0.2204	-97	142	162	SVYEPLQTGLIAIDSMIPIGR	120	100			Mascot
2273.2209	2273.0005	-0.2204	-97	142	162	SVYEPLQTGLIAIDSMIPIGR					Mascot
2289.2158	2288.9995	-0.2163	-94	142	162	SVYEPLQTGLIAIDSMIPIGR			Oxidation (M)[16]		Mascot
2356.1707	2355.9639	-0.2068	-88	385	405	LELAQFAELEAFAQFASDLDK					Mascot
2429.322	2429.0889	-0.2331	-96	141	162	RSVYEPLQTGLIAIDSMIPIGR					Mascot
2804.4609	2804.2092	-0.2517	-90	177	202	TAVATDTILNQQGQNVICVYVAIGQK			Carbamidomethyl (C)[18]		Mascot

Rank	Protein Name	Accession No	Protein MW	Protein PI	Pep. Count	Protein Score	Protein Score C. I. %	Total Ion Score	Total Ion C. I. %
10	ATP synthase subunit alpha, chloroplastic OS=Gossypium barbadense GN=atpA PE=3 SV=1	ATPA GOSBA	55443.9	5.19	14	539	100	462	100

Peptide Information

Calc. Mass	Obsrv. Mass	± da	± ppm	Start Seq.	End Seq.	Sequence	Ion Score	C. I. %	Modification	Rank	Result Type
860.5022	860.4275	-0.0747	-87	273	279	QMSLLLR					Mascot
876.4971	876.4198	-0.0773	-88	273	279	QMSLLLR			Oxidation (M)[2]		Mascot
901.485	901.4083	-0.0767	-85	406	413	ATQNQLAR					Mascot
904.5614	904.4821	-0.0793	-88	456	462	KFLVELR					Mascot
1252.726	1252.6146	-0.1114	-89	129	140	LIESPAPGIISR					Mascot
1252.726	1252.6146	-0.1114	-89	129	140	LIESPAPGIISR	58	99.981			Mascot
1317.705	1317.5841	-0.1209	-92	256	266	HTLIIYDDLSK					Mascot
1408.6638	1408.5785	-0.0853	-61	492	502	DAIQDQMERFR					Mascot
1416.7845	1416.6559	-0.1286	-91	95	107	IAQIPVSEAYLGR	98	100			Mascot
1416.7845	1416.6559	-0.1286	-91	95	107	IAQIPVSEAYLGR					Mascot
1553.7383	1553.5946	-0.1437	-92	285	297	EAYPGDVFYLHSR	96	100			Mascot
1553.7383	1553.5946	-0.1437	-92	285	297	EAYPGDVFYLHSR					Mascot
1612.9016	1612.7528	-0.1488	-92	26	41	IVNTGTVLQVGDGIAR	91	100			Mascot
1612.9016	1612.7528	-0.1488	-92	26	41	IVNTGTVLQVGDGIAR					Mascot
2065.05	2064.8408	-0.2092	-101	285	301	EAYPGDVFYLHSRLLER					Mascot
2273.2209	2273.0005	-0.2204	-97	142	162	SVYEPLQTGLIAIDSMIPIGR	120	100			Mascot
2273.2209	2273.0005	-0.2204	-97	142	162	SVYEPLQTGLIAIDSMIPIGR					Mascot
2289.2158	2288.9995	-0.2163	-94	142	162	SVYEPLQTGLIAIDSMIPIGR			Oxidation (M)[16]		Mascot
2356.1707	2355.9639	-0.2068	-88	385	405	LELAQFAELEAFAQFASDLDK					Mascot
2429.322	2429.0889	-0.2331	-96	141	162	RSVYEPLQTGLIAIDSMIPIGR					Mascot
2804.4609	2804.2092	-0.2517	-90	177	202	TAVATDTILNQQGQNVICVYVAIGQK			Carbamidomethyl (C)[18]		Mascot

Gel Idx/Pos	44/B17	Instr./Gel Origin	BA2060/131017A	Process Sta	Analysis Succeeded
Plate [#] Name	[1] 31496	Instrument Sample Name		Spectra	8

Rank	Protein Name	Accession No	Protein MW	Protein PI	Pep. Count	Protein Score	Protein Score C. I. %	Total Ion Score	Total Ion C. I. %
1	ATP synthase subunit alpha, chloroplastic OS=G max GN=atpA PE=3 SV=1	ATPA_SOYBI	55719.3	5.15	23	751	100	567	100

Peptide Information

Calc. Mass	Obsrv. Mass	± da	± ppm	Start Seq.	End Seq.	Sequence	Ion Score	C. I. %	Modification	Rank	Result Type
815.4621	815.4028	-0.0593	-73	166	172	ELIIGDR					Mascot
860.5022	860.4364	-0.0658	-76	273	279	QMSLLLR					Mascot
876.4971	876.4275	-0.0696	-79	273	279	QMSLLLR			Oxidation (M)[2]		Mascot
901.485	901.4178	-0.0672	-75	406	413	ATQNQLAR					Mascot
904.5614	904.4937	-0.0677	-75	456	462	KFLVELR					Mascot
935.4428	935.377	-0.0658	-70	120	128	GEISASESR					Mascot
1123.563	1123.4763	-0.0867	-77	17	25	IEQYNTEVK					Mascot
1207.6569	1207.5582	-0.0987	-82	481	491	TFTGEAEVLLK					Mascot
1252.726	1252.6292	-0.0968	-77	129	140	LIESPAPGIISR	51	99.892			Mascot
1252.726	1252.6292	-0.0968	-77	129	140	LIESPAPGIISR					Mascot
1266.7528	1266.6536	-0.0992	-78	108	119	VINALAKPIDGR					Mascot
1317.705	1317.5979	-0.1071	-81	256	266	HTLIIYDDLSK					Mascot
1323.7056	1323.6002	-0.1054	-80	463	473	AYLNTNKPQFK					Mascot
1408.7067	1408.5916	-0.1151	-82	15	25	ERIEQYNTEVK					Mascot
1416.7845	1416.6735	-0.111	-78	95	107	IAQIPVSEAYLGR					Mascot
1416.7845	1416.6735	-0.111	-78	95	107	IAQIPVSEAYLGR	112	100			Mascot
1501.7969	1501.6777	-0.1192	-79	203	216	ASSVAQVVNTLQER					Mascot
1501.7969	1501.6777	-0.1192	-79	203	216	ASSVAQVVNTLQER	58	99.981			Mascot
1553.7383	1553.6136	-0.1247	-80	285	297	EAYPGDVFYLHSR					Mascot
1553.7383	1553.6136	-0.1247	-80	285	297	EAYPGDVFYLHSR	79	100			Mascot
1612.9016	1612.771	-0.1306	-81	26	41	IVNTGTVLQVGDGIAR					Mascot
1612.9016	1612.771	-0.1306	-81	26	41	IVNTGTVLQVGDGIAR	139	100			Mascot
2033.0405	2032.8656	-0.1749	-86	71	90	NVGVVLMGDGLMIQEGSSVK					Mascot
2049.0354	2048.8589	-0.1765	-86	71	90	NVGVVLMGDGLMIQEGSSVK			Oxidation (M)[7]		Mascot
2065.0303	2064.8547	-0.1756	-85	71	90	NVGVVLMGDGLMIQEGSSVK			Oxidation (M)[7,12]		Mascot
2205.1108	2204.9272	-0.1836	-83	492	509	EAIQEQMELFLLQEQVEK					Mascot
2221.1057	2220.9172	-0.1885	-85	492	509	EAIQEQMELFLLQEQVEK			Oxidation (M)[7]		Mascot
2273.2209	2273.0266	-0.1943	-85	142	162	SVYEPLQTGLIAIDSMIPIGR					Mascot
2273.2209	2273.0266	-0.1943	-85	142	162	SVYEPLQTGLIAIDSMIPIGR	129	100			Mascot
2289.2158	2289.0222	-0.1936	-85	142	162	SVYEPLQTGLIAIDSMIPIGR			Oxidation (M)[16]		Mascot
2319.1536	2318.9539	-0.1997	-86	492	510	EAIQEQMELFLLQEQVEKN					Mascot
2335.1484	2334.9512	-0.1972	-84	492	510	EAIQEQMELFLLQEQVEKN			Oxidation (M)[7]		Mascot
2445.3169	2445.0981	-0.2188	-89	141	162	RSVYEPLQTGLIAIDSMIPIGR			Oxidation (M)[17]		Mascot
2804.4609	2804.2231	-0.2378	-85	177	202	TAVATDTILNQQGQNVICVYVAIGQK			Carbamidomethyl (C)[18]		Mascot
3623.8438	3623.5171	-0.3267	-90	423	455	QSQSAPLTVEEQIITIYTGTNGYLDSLEIGQVR					Mascot

Rank	Protein Name	Accession No	Protein MW	Protein PI	Pep. Count	Protein Score	Protein Score C. I. %	Total Ion Score	Total Ion C. I. %
2	ATP synthase subunit alpha, chloroplastic OS=Eucalyptus globulus subsp. globulus GN=atpA PE=3 SV=1	ATPA_EUCG(	55478.1	5.15	17	675	100	567	100

Peptide Information

Calc. Mass	Obsrv. Mass	± da	± ppm	Start Seq.	End Seq.	Sequence	Ion Score	C. I. %	Modification	Rank	Result Type
815.4621	815.4028	-0.0593	-73	166	172	ELIIGDR					Mascot
860.5022	860.4364	-0.0658	-76	273	279	QMSLLLR					Mascot
	860.4364										Mascot
876.4971	876.4275	-0.0696	-79	273	279	QMSLLLR			Oxidation (M)[2]		Mascot
901.485	901.4178	-0.0672	-75	406	413	ATQNQLAR					Mascot
904.5614	904.4937	-0.0677	-75	456	462	KFLVELR					Mascot
1252.726	1252.6292	-0.0968	-77	129	140	LIESPAPGIISR	51	99.892			Mascot
1252.726	1252.6292	-0.0968	-77	129	140	LIESPAPGIISR					Mascot
1266.7528	1266.6536	-0.0992	-78	108	119	VINALAKPIDGR					Mascot
1317.705	1317.5979	-0.1071	-81	256	266	HTLIIYDDLSK					Mascot
1416.7845	1416.6735	-0.111	-78	95	107	IAQIPVSEAYLGR					Mascot
1416.7845	1416.6735	-0.111	-78	95	107	IAQIPVSEAYLGR	112	100			Mascot
1501.7969	1501.6777	-0.1192	-79	203	216	ASSVAQVVNTLQER					Mascot
1501.7969	1501.6777	-0.1192	-79	203	216	ASSVAQVVNTLQER	58	99.981			Mascot
1553.7383	1553.6136	-0.1247	-80	285	297	EAYPGDVFYLHSR					Mascot
1553.7383	1553.6136	-0.1247	-80	285	297	EAYPGDVFYLHSR	79	100			Mascot
1612.9016	1612.771	-0.1306	-81	26	41	IVNTGTVLQVGDGIAR					Mascot
1612.9016	1612.771	-0.1306	-81	26	41	IVNTGTVLQVGDGIAR	139	100			Mascot
2065.05	2064.8547	-0.1953	-95	285	301	EAYPGDVFYLHSRLLER					Mascot
2273.2209	2273.0266	-0.1943	-85	142	162	SVYEPLQTGLIAIDSMIPIGR					Mascot
2273.2209	2273.0266	-0.1943	-85	142	162	SVYEPLQTGLIAIDSMIPIGR	129	100			Mascot
2289.2158	2289.0222	-0.1936	-85	142	162	SVYEPLQTGLIAIDSMIPIGR			Oxidation (M)[16]		Mascot
2356.1707	2355.9753	-0.1954	-83	385	405	LELAQFAELEAFAQFASDLDK					Mascot
2445.3169	2445.0981	-0.2188	-89	141	162	RSVYEPLQTGLIAIDSMIPIGR			Oxidation (M)[17]		Mascot
2597.3496	2597.1206	-0.229	-88	383	405	LKLELAQFAELEAFAQFASDLDK					Mascot
2804.4609	2804.2231	-0.2378	-85	177	202	TAVATDTILNQQGQNVICVYVAIGQK			Carbamidomethyl (C)[18]		Mascot

Rank	Protein Name	Accession No	Protein MW	Protein PI	Pep. Count	Protein Score	Protein Score C. I. %	Total Ion Score	Total Ion C. I. %
3	ATP synthase subunit alpha, chloroplastic OS=L sativa GN=atpA PE=3 SV=1	ATPA_LACS/	55484.2	5.27	17	616	100	509	100

Peptide Information

Calc. Mass	Obsrv. Mass	± da	± ppm	Start Seq.	End Seq.	Sequence	Ion Score	C. I. %	Modification	Rank	Result Type
815.4621	815.4028	-0.0593	-73	166	172	ELIIGDR					Mascot
860.5022	860.4364	-0.0658	-76	273	279	QMSLLLR					Mascot
876.4971	876.4275	-0.0696	-79	273	279	QMSLLLR			Oxidation (M)[2]		Mascot
901.485	901.4178	-0.0672	-75	406	413	ATQNQLAR					Mascot
904.5614	904.4937	-0.0677	-75	456	462	KFLVELR					Mascot
1252.726	1252.6292	-0.0968	-77	129	140	LIESPAPGIISR	51	99.892			Mascot
1252.726	1252.6292	-0.0968	-77	129	140	LIESPAPGIISR					Mascot
1266.7528	1266.6536	-0.0992	-78	108	119	VINALAKPIDGR					Mascot
1275.6216	1275.6307	0.0091	7	256	266	HTSIIYDDPSK					Mascot
1416.7845	1416.6735	-0.111	-78	95	107	IAQIPVSEAYLGR					Mascot
1416.7845	1416.6735	-0.111	-78	95	107	IAQIPVSEAYLGR	112	100			Mascot
1535.7812	1535.6039	-0.1773	-115	203	216	ASSVAQVVTNFQER					Mascot
1553.7383	1553.6136	-0.1247	-80	285	297	EAYPGDVFYLHSR					Mascot
1553.7383	1553.6136	-0.1247	-80	285	297	EAYPGDVFYLHSR	79	100			Mascot
1612.9016	1612.771	-0.1306	-81	26	41	IVNTGTVLQVGDGIAR					Mascot
1612.9016	1612.771	-0.1306	-81	26	41	IVNTGTVLQVGDGIAR	139	100			Mascot
2065.05	2064.8547	-0.1953	-95	285	301	EAYPGDVFYLHSRLLER					Mascot
2273.2209	2273.0266	-0.1943	-85	142	162	SVYEPLQTGLIAIDSMIPIGR					Mascot
2273.2209	2273.0266	-0.1943	-85	142	162	SVYEPLQTGLIAIDSMIPIGR	129	100			Mascot
2289.2158	2289.0222	-0.1936	-85	142	162	SVYEPLQTGLIAIDSMIPIGR			Oxidation (M)[16]		Mascot
2356.1707	2355.9753	-0.1954	-83	385	405	LELAQFAELEAFAQFASDLDK					Mascot
2445.3169	2445.0981	-0.2188	-89	141	162	RSVYEPLQTGLIAIDSMIPIGR			Oxidation (M)[17]		Mascot
2597.3496	2597.1206	-0.229	-88	383	405	LKLELAQFAELEAFAQFASDLDK					Mascot
2804.4973	2804.2231	-0.2742	-98	177	202	TAVATDTILNQQGKNVICVYVAIGQK			Carbamidomethyl (C)[18]		Mascot

Rank	Protein Name	Accession No	Protein MW	Protein PI	Pep. Count	Protein Score	Protein Score C. I. %	Total Ion Score	Total Ion C. I. %
4	ATP synthase subunit alpha, chloroplastic OS=Gossypium hirsutum GN=atpA PE=3 SV=1	ATPA_GOSHI	55379.9	5.25	17	616	100	509	100

Peptide Information

Calc. Mass	Obsrv. Mass	± da	± ppm	Start Seq.	End Seq.	Sequence	Ion Score	C. I. %	Modification	Rank	Result Type
815.4621	815.4028	-0.0593	-73	166	172	ELIIGDR					Mascot
860.5022	860.4364	-0.0658	-76	273	279	QMSLLLR					Mascot
876.4971	876.4275	-0.0696	-79	273	279	QMSLLLR			Oxidation (M)[2]		Mascot
901.485	901.4178	-0.0672	-75	406	413	ATQNQLAR					Mascot
904.5614	904.4937	-0.0677	-75	456	462	KFLVELR					Mascot
935.4428	935.377	-0.0658	-70	120	128	GEISASESR					Mascot
1252.726	1252.6292	-0.0968	-77	129	140	LIESPAPGIISR	51	99.892			Mascot
1252.726	1252.6292	-0.0968	-77	129	140	LIESPAPGIISR					Mascot
1266.7528	1266.6536	-0.0992	-78	108	119	VINALAKPIDGR					Mascot
1317.705	1317.5979	-0.1071	-81	256	266	HTLIIYDDLSK					Mascot
1408.6638	1408.5916	-0.0722	-51	492	502	DAIQDQMERFR					Mascot
1416.7845	1416.6735	-0.111	-78	95	107	IAQIPVSEAYLGR					Mascot
1416.7845	1416.6735	-0.111	-78	95	107	IAQIPVSEAYLGR	112	100			Mascot
1553.7383	1553.6136	-0.1247	-80	285	297	EAYPGDVFYLHSR					Mascot
1553.7383	1553.6136	-0.1247	-80	285	297	EAYPGDVFYLHSR	79	100			Mascot
1612.9016	1612.771	-0.1306	-81	26	41	IVNTGTVLQVGDGIAR					Mascot
1612.9016	1612.771	-0.1306	-81	26	41	IVNTGTVLQVGDGIAR	139	100			Mascot
2065.05	2064.8547	-0.1953	-95	285	301	EAYPGDVFYLHSRLLER					Mascot
2273.2209	2273.0266	-0.1943	-85	142	162	SVYEPLQTGLIAIDSMIPIGR					Mascot
2273.2209	2273.0266	-0.1943	-85	142	162	SVYEPLQTGLIAIDSMIPIGR	129	100			Mascot
2289.2158	2289.0222	-0.1936	-85	142	162	SVYEPLQTGLIAIDSMIPIGR			Oxidation (M)[16]		Mascot
2356.1707	2355.9753	-0.1954	-83	385	405	LELAQFAELEAFAQFASDLDK					Mascot
2445.3169	2445.0981	-0.2188	-89	141	162	RSVYEPLQTGLIAIDSMIPIGR			Oxidation (M)[17]		Mascot
2804.4609	2804.2231	-0.2378	-85	177	202	TAVATDTILNQQGQNVICVYVAIGQK			Carbamidomethyl (C)[18]		Mascot

Rank	Protein Name	Accession No	Protein MW	Protein PI	Pep. Count	Protein Score	Protein Score C. I. %	Total Ion Score	Total Ion C. I. %
5	ATP synthase subunit alpha, chloroplastic OS=P trichocarpa GN=atpA PE=3 SV=1	ATPA_POPTI	55288	5.2	17	615	100	509	100

Peptide Information

Calc. Mass	Obsrv. Mass	± da	± ppm	Start Seq.	End Seq.	Sequence	Ion Score	C. I. %	Modification	Rank	Result Type
815.4621	815.4028	-0.0593	-73	166	172	ELIIGDR					Mascot
860.5022	860.4364	-0.0658	-76	273	279	QMSLLLR					Mascot
876.4825	876.4275	-0.055	-63	501	507	FLLQEQV					Mascot
901.485	901.4178	-0.0672	-75	406	413	ATQNQLAR					Mascot
904.5614	904.4937	-0.0677	-75	456	462	KFLVELR					Mascot
1252.726	1252.6292	-0.0968	-77	129	140	LIESPAPGIISR	51	99.892			Mascot
1252.726	1252.6292	-0.0968	-77	129	140	LIESPAPGIISR					Mascot
1266.7528	1266.6536	-0.0992	-78	108	119	VINALAKPIDGR					Mascot
1317.705	1317.5979	-0.1071	-81	256	266	HTLIIYDDLSK					Mascot
1416.7845	1416.6735	-0.111	-78	95	107	IAQIPVSEAYLGR					Mascot
1416.7845	1416.6735	-0.111	-78	95	107	IAQIPVSEAYLGR	112	100			Mascot
1553.7383	1553.6136	-0.1247	-80	285	297	EAYPGDVFYLHSR					Mascot
1553.7383	1553.6136	-0.1247	-80	285	297	EAYPGDVFYLHSR	79	100			Mascot
1612.9016	1612.771	-0.1306	-81	26	41	IVNTGTVLQVGDGIAR					Mascot
1612.9016	1612.771	-0.1306	-81	26	41	IVNTGTVLQVGDGIAR	139	100			Mascot
2065.05	2064.8547	-0.1953	-95	285	301	EAYPGDVFYLHSRLLER					Mascot
2273.2209	2273.0266	-0.1943	-85	142	162	SVYEPLQTGLIAIDSMIPIGR					Mascot
2273.2209	2273.0266	-0.1943	-85	142	162	SVYEPLQTGLIAIDSMIPIGR	129	100			Mascot
2289.2158	2289.0222	-0.1936	-85	142	162	SVYEPLQTGLIAIDSMIPIGR			Oxidation (M)[16]		Mascot
2356.1707	2355.9753	-0.1954	-83	385	405	LELAQFAELEAFAQFASDLDK					Mascot
2445.3169	2445.0981	-0.2188	-89	141	162	RSVYEPLQTGLIAIDSMIPIGR			Oxidation (M)[17]		Mascot
2597.3496	2597.1206	-0.229	-88	383	405	LKLELAQFAELEAFAQFASDLDK					Mascot
2804.4609	2804.2231	-0.2378	-85	177	202	TAVATDTILNQQGQNVICVYVAIGQK			Carbamidomethyl (C)[18]		Mascot

Rank	Protein Name	Accession No	Protein MW	Protein PI	Pep. Count	Protein Score	Protein Score C. I. %	Total Ion Score	Total Ion C. I. %
6	ATP synthase subunit alpha, chloroplastic OS=Helianthus annuus GN=atpA PE=3 SV=1	ATPA_HELAN	55482.2	5.2	16	606	100	509	100

Peptide Information

Calc. Mass	Obsrv. Mass	± da	± ppm	Start Seq.	End Seq.	Sequence	Ion Score	C. I. %	Modification	Rank	Result Type
815.4621	815.4028	-0.0593	-73	166	172	ELIIGDR					Mascot
860.5022	860.4364	-0.0658	-76	273	279	QMSLLLR					Mascot
876.4971	876.4275	-0.0696	-79	273	279	QMSLLLR			Oxidation (M)[2]		Mascot
901.485	901.4178	-0.0672	-75	406	413	ATQNQLAR					Mascot
904.5614	904.4937	-0.0677	-75	456	462	KFLVELR					Mascot
1252.726	1252.6292	-0.0968	-77	129	140	LIESPAPGIISR	51	99.892			Mascot
1252.726	1252.6292	-0.0968	-77	129	140	LIESPAPGIISR					Mascot
1266.7528	1266.6536	-0.0992	-78	108	119	VINALAKPIDGR					Mascot
1317.705	1317.5979	-0.1071	-81	256	266	HTLIIYDDLSK					Mascot
1416.7845	1416.6735	-0.111	-78	95	107	IAQIPVSEAYLGR					Mascot
1416.7845	1416.6735	-0.111	-78	95	107	IAQIPVSEAYLGR	112	100			Mascot
1553.7383	1553.6136	-0.1247	-80	285	297	EAYPGDVFYLHSR					Mascot
1553.7383	1553.6136	-0.1247	-80	285	297	EAYPGDVFYLHSR	79	100			Mascot
1612.9016	1612.771	-0.1306	-81	26	41	IVNTGTVLQVGDGIAR					Mascot
1612.9016	1612.771	-0.1306	-81	26	41	IVNTGTVLQVGDGIAR	139	100			Mascot
2065.05	2064.8547	-0.1953	-95	285	301	EAYPGDVFYLHSRLLER					Mascot
2273.2209	2273.0266	-0.1943	-85	142	162	SVYEPLQTGLIAIDSMIPIGR					Mascot
2273.2209	2273.0266	-0.1943	-85	142	162	SVYEPLQTGLIAIDSMIPIGR	129	100			Mascot
2289.2158	2289.0222	-0.1936	-85	142	162	SVYEPLQTGLIAIDSMIPIGR			Oxidation (M)[16]		Mascot
2356.1707	2355.9753	-0.1954	-83	385	405	LELAQFAELEAFAQFASDLDK					Mascot
2445.3169	2445.0981	-0.2188	-89	141	162	RSVYEPLQTGLIAIDSMIPIGR			Oxidation (M)[17]		Mascot
2597.3496	2597.1206	-0.229	-88	383	405	LKLELAQFAELEAFAQFASDLDK					Mascot
2804.4609	2804.2231	-0.2378	-85	177	202	TAVATDTILNQQGQNVICVYVAIGQK			Carbamidomethyl (C)[18]		Mascot
	2804.2231										Mascot

Rank	Protein Name	Accession No	Protein MW	Protein PI	Pep. Count	Protein Score	Protein Score C. I. %	Total Ion Score	Total Ion C. I. %
7	ATP synthase subunit alpha, chloroplastic OS=C arabica GN=atpA PE=3 SV=1	ATPA_COFAI	55233.8	5.19	16	606	100	509	100

Peptide Information

Calc. Mass	Obsrv. Mass	± da	± ppm	Start Seq.	End Seq.	Sequence	Ion Score	C. I. %	Modification	Rank	Result Type
815.4621	815.4028	-0.0593	-73	166	172	ELIIGDR					Mascot
860.5022	860.4364	-0.0658	-76	273	279	QMSLLLR					Mascot
876.4971	876.4275	-0.0696	-79	273	279	QMSLLLR			Oxidation (M)[2]		Mascot
901.485	901.4178	-0.0672	-75	406	413	ATQNQLAR					Mascot
935.4428	935.377	-0.0658	-70	120	128	GEISASESR					Mascot
1252.726	1252.6292	-0.0968	-77	129	140	LIESPAPGIISR	51	99.892			Mascot
1252.726	1252.6292	-0.0968	-77	129	140	LIESPAPGIISR					Mascot
1266.7528	1266.6536	-0.0992	-78	108	119	VINALAKPIDGR					Mascot
1275.6216	1275.6307	0.0091	7	256	266	HTSIIYDDPSK					Mascot
1416.7845	1416.6735	-0.111	-78	95	107	IAQIPVSEAYLGR					Mascot
1416.7845	1416.6735	-0.111	-78	95	107	IAQIPVSEAYLGR	112	100			Mascot
1553.7383	1553.6136	-0.1247	-80	285	297	EAYPGDVFYLHSR					Mascot
1553.7383	1553.6136	-0.1247	-80	285	297	EAYPGDVFYLHSR	79	100			Mascot
1612.9016	1612.771	-0.1306	-81	26	41	IVNTGTVLQVGDGIAR					Mascot
1612.9016	1612.771	-0.1306	-81	26	41	IVNTGTVLQVGDGIAR	139	100			Mascot
2065.05	2064.8547	-0.1953	-95	285	301	EAYPGDVFYLHSRLLER					Mascot
2273.2209	2273.0266	-0.1943	-85	142	162	SVYEPLQTGLIAIDSMIPIGR					Mascot
2273.2209	2273.0266	-0.1943	-85	142	162	SVYEPLQTGLIAIDSMIPIGR	129	100			Mascot
2289.2158	2289.0222	-0.1936	-85	142	162	SVYEPLQTGLIAIDSMIPIGR			Oxidation (M)[16]		Mascot
2356.1707	2355.9753	-0.1954	-83	385	405	LELAQFAELEAFAQFASDLDK					Mascot
2445.3169	2445.0981	-0.2188	-89	141	162	RSVYEPLQTGLIAIDSMIPIGR			Oxidation (M)[17]		Mascot
2597.3496	2597.1206	-0.229	-88	383	405	LKLELAQFAELEAFAQFASDLDK					Mascot
2804.4609	2804.2231	-0.2378	-85	177	202	TAVATDTILNQQGQNVICVYVAIGQK			Carbamidomethyl (C)[18]		Mascot

Rank	Protein Name	Accession No	Protein MW	Protein PI	Pep. Count	Protein Score	Protein Score C. I. %	Total Ion Score	Total Ion C. I. %
8	ATP synthase subunit alpha, chloroplastic OS=D carota GN=atpA PE=3 SV=1	ATPA_DAUC/	55451.2	5.34	16	604	100	507	100

Peptide Information

Calc. Mass	Obsrv. Mass	± da	± ppm	Start Seq.	End Seq.	Sequence	Ion Score	C. I. %	Modification	Rank	Result Type
815.4621	815.4028	-0.0593	-73	166	172	ELIIGDR					Mascot
860.5022	860.4364	-0.0658	-76	273	279	QMSLLLR					Mascot
876.4825	876.4275	-0.055	-63	501	507	FILQEQV					Mascot
901.485	901.4178	-0.0672	-75	406	413	ATQNQLAR					Mascot
904.5614	904.4937	-0.0677	-75	456	462	KFLVELR					Mascot
1252.726	1252.6292	-0.0968	-77	129	140	LIESPAPGIISR	51	99.892			Mascot
1252.726	1252.6292	-0.0968	-77	129	140	LIESPAPGIISR					Mascot
1416.7845	1416.6735	-0.111	-78	95	107	IAQIPVSEAYLGR					Mascot
1416.7845	1416.6735	-0.111	-78	95	107	IAQIPVSEAYLGR	112	100			Mascot
1535.7812	1535.6039	-0.1773	-115	203	216	ASSVAQVVTNFQER					Mascot
1553.7383	1553.6136	-0.1247	-80	285	297	EAYPGDVFYLHSR					Mascot
1553.7383	1553.6136	-0.1247	-80	285	297	EAYPGDVFYLHSR	79	100			Mascot
1612.9016	1612.771	-0.1306	-81	26	41	IVNTGTVLQVGDGIAR					Mascot
1612.9016	1612.771	-0.1306	-81	26	41	IVNTGTVLQVGDGIAR	139	100			Mascot
2065.05	2064.8547	-0.1953	-95	285	301	EAYPGDVFYLHSRLLER					Mascot
2273.2209	2273.0266	-0.1943	-85	142	162	SVYEPLQTGLIAIDSMIPIGR					Mascot
2273.2209	2273.0266	-0.1943	-85	142	162	SVYEPLQTGLIAIDSMIPIGR	129	100			Mascot
2289.2158	2289.0222	-0.1936	-85	142	162	SVYEPLQTGLIAIDSMIPIGR			Oxidation (M)[16]		Mascot
2356.1707	2355.9753	-0.1954	-83	385	405	LELAQFAELEAFAQFASDLDK					Mascot
2445.3169	2445.0981	-0.2188	-89	141	162	RSVYEPLQTGLIAIDSMIPIGR			Oxidation (M)[17]		Mascot
2597.3496	2597.1206	-0.229	-88	383	405	LKLELAQFAELEAFAQFASDLDK					Mascot
2804.4609	2804.2231	-0.2378	-85	177	202	TAVATDTILNQQGQNVICVYVAIGQK			Carbamidomethyl (C)[18]		Mascot

Mascot

Rank	Protein Name	Accession No	Protein MW	Protein PI	Pep. Count	Protein Score	Protein Score C. I. %	Total Ion Score	Total Ion C. I. %
9	ATP synthase subunit alpha, chloroplastic OS=Nymphaea alba GN=atpA PE=3 SV=1	ATPA_NYMAL	55482.2	5.58	15	596	100	509	100

Peptide Information

Calc. Mass	Obsrv. Mass	± da	± ppm	Start Seq.	End Seq.	Sequence	Ion Score	C. I. %	Modification	Rank	Result Type
815.4621	815.4028	-0.0593	-73	166	172	ELIIGDR					Mascot
860.5022	860.4364	-0.0658	-76	273	279	QMSLLLR					Mascot
876.4971	876.4275	-0.0696	-79	273	279	QMSLLLR			Oxidation (M)[2]		Mascot
901.485	901.4178	-0.0672	-75	406	413	ATQNQLAR					Mascot
1252.726	1252.6292	-0.0968	-77	129	140	LIESPAPGIISR	51	99.892			Mascot
1252.726	1252.6292	-0.0968	-77	129	140	LIESPAPGIISR					Mascot
1266.7528	1266.6536	-0.0992	-78	108	119	VINALAKPIDGR					Mascot
1317.705	1317.5979	-0.1071	-81	256	266	HTLIIYDDLSK					Mascot
1416.7845	1416.6735	-0.111	-78	95	107	IAQIPVSEAYLGR					Mascot
1416.7845	1416.6735	-0.111	-78	95	107	IAQIPVSEAYLGR	112	100			Mascot
1553.7383	1553.6136	-0.1247	-80	285	297	EAYPGDVFYLHSR					Mascot
1553.7383	1553.6136	-0.1247	-80	285	297	EAYPGDVFYLHSR	79	100			Mascot
1612.9016	1612.771	-0.1306	-81	26	41	IVNTGTVLQVGDGIAR					Mascot
1612.9016	1612.771	-0.1306	-81	26	41	IVNTGTVLQVGDGIAR	139	100			Mascot
2065.05	2064.8547	-0.1953	-95	285	301	EAYPGDVFYLHSRLLER					Mascot
2273.2209	2273.0266	-0.1943	-85	142	162	SVYEPLQTGLIAIDSMIPIGR					Mascot
2273.2209	2273.0266	-0.1943	-85	142	162	SVYEPLQTGLIAIDSMIPIGR	129	100			Mascot
2289.2158	2289.0222	-0.1936	-85	142	162	SVYEPLQTGLIAIDSMIPIGR			Oxidation (M)[16]		Mascot
2356.1707	2355.9753	-0.1954	-83	385	405	LELAQFAELEAFAQFASDLDK					Mascot
2445.3169	2445.0981	-0.2188	-89	141	162	RSVYEPLQTGLIAIDSMIPIGR			Oxidation (M)[17]		Mascot
2597.3496	2597.1206	-0.229	-88	383	405	LKLELAQFAELEAFAQFASDLDK					Mascot
2804.4973	2804.2231	-0.2742	-98	177	202	TAVATDTILNQKGQNVICVYVAIGQK			Carbamidomethyl (C)[18]		Mascot

Rank	Protein Name	Accession No	Protein MW	Protein PI	Pep. Count	Protein Score	Protein Score C. I. %	Total Ion Score	Total Ion C. I. %
10	ATP synthase subunit alpha, chloroplastic OS=M esculenta GN=atpA PE=3 SV=1	ATPA_MANES	55564.2	5.22	15	596	100	509	100

Peptide Information

Calc. Mass	Obsrv. Mass	± da	± ppm	Start Seq.	End Seq.	Sequence	Ion Score	C. I. %	Modification	Rank	Result Type
815.4621	815.4028	-0.0593	-73	166	172	ELIIGDR					Mascot
860.5022	860.4364	-0.0658	-76	273	279	QMSLLLR					Mascot
876.4971	876.4275	-0.0696	-79	273	279	QMSLLLR			Oxidation (M)[2]		Mascot
901.485	901.4178	-0.0672	-75	406	413	ATQNQLAR					Mascot
904.5614	904.4937	-0.0677	-75	456	462	KFLVELR					Mascot
935.4428	935.377	-0.0658	-70	120	128	GEISASESR					Mascot
1252.726	1252.6292	-0.0968	-77	129	140	LIESPAPGIISR	51	99.892			Mascot
1252.726	1252.6292	-0.0968	-77	129	140	LIESPAPGIISR					Mascot
1266.7528	1266.6536	-0.0992	-78	108	119	VINALAKPIDGR					Mascot
1317.705	1317.5979	-0.1071	-81	256	266	HTLIIYDDLSK					Mascot
1416.7845	1416.6735	-0.111	-78	95	107	IAQIPVSEAYLGR					Mascot
1416.7845	1416.6735	-0.111	-78	95	107	IAQIPVSEAYLGR	112	100			Mascot
1553.7383	1553.6136	-0.1247	-80	285	297	EAYPGDVFYLHSR					Mascot
1553.7383	1553.6136	-0.1247	-80	285	297	EAYPGDVFYLHSR	79	100			Mascot
1612.9016	1612.771	-0.1306	-81	26	41	IVNTGTVLQVGDGIAR					Mascot
1612.9016	1612.771	-0.1306	-81	26	41	IVNTGTVLQVGDGIAR	139	100			Mascot
2065.05	2064.8547	-0.1953	-95	285	301	EAYPGDVFYLHSRLLER					Mascot
2273.2209	2273.0266	-0.1943	-85	142	162	SVYEPLQTGLIAIDSMIPIGR					Mascot
2273.2209	2273.0266	-0.1943	-85	142	162	SVYEPLQTGLIAIDSMIPIGR	129	100			Mascot
2289.2158	2289.0222	-0.1936	-85	142	162	SVYEPLQTGLIAIDSMIPIGR			Oxidation (M)[16]		Mascot
2445.3169	2445.0981	-0.2188	-89	141	162	RSVYEPLQTGLIAIDSMIPIGR			Oxidation (M)[17]		Mascot
2804.4609	2804.2231	-0.2378	-85	177	202	TAVATDTILNQQGQNVICVYVAIGQK			Carbamidomethyl (C)[18]		Mascot

Gel Idx/Pos	45/B18	Instr./Gel Origin	BA2060/131017A	Process Sta	Analysis Succeeded
Plate [#] Name	[1] 31496	Instrument Sample Name		Spectra	8

Rank	Protein Name	Accession No	Protein MW	Protein PI	Pep. Count	Protein Score	Protein Score C. I. %	Total Ion Score	Total Ion C. I. %
1	Ribulose bisphosphate carboxylase large chain OS=Glycine max GN=rbcL PE=1 SV=3	RBL_SOYBN	52576.4	6	25	929	100	722	100

Peptide Information

Calc. Mass	Obsrv. Mass	± da	± ppm	Start Seq.	End Seq.	Sequence	Ion Score	C. I. %	Modification	Rank	Result Type	
805.4818	805.4279	-0.0539	-67	140	146	IPTAYIK					Mascot	
830.4366	830.3821	-0.0545	-66	440	446	EGNEIIR					Mascot	
898.41	898.352	-0.058	-65	306	312	NHGMHFR					Mascot	
910.4451	910.3861	-0.059	-65	188	194	AVYECLR			Carbamidomethyl (C)[5]		Mascot	
912.4719	912.4047	-0.0672	-74	296	303	AMHAVIDR					Mascot	
914.4049	914.3503	-0.0546	-60	306	312	NHGMHFR			Oxidation (M)[4]		Mascot	
928.4669	928.4054	-0.0615	-66	296	303	AMHAVIDR			Oxidation (M)[2]		Mascot	
962.4788	962.4172	-0.0616	-64	228	236	SQAETGEIK					Mascot	Mascot
1021.5312	1021.4648	-0.0664	-65	33	41	DTDILAAFR					Mascot	
1116.583	1116.5121	-0.0709	-63	422	431	VALEACVQAR			Carbamidomethyl (C)[6]		Mascot	
1170.5585	1170.5605	0.002	2	304	312	QKNHGMHFR			Oxidation (M)[6]		Mascot	
1187.6643	1187.5884	-0.0759	-64	286	295	DNGLLLHIHR					Mascot	
1245.6337	1245.5505	-0.0832	-67	218	227	FLFCAEAIFK	62	99.991	Carbamidomethyl (C)[4]		Mascot	
1245.6337	1245.5505	-0.0832	-67	218	227	FLFCAEAIFK			Carbamidomethyl (C)[4]		Mascot	
1275.7307	1275.6484	-0.0823	-65	340	350	EITLGFVDLLR	88	100			Mascot	
1275.7307	1275.6484	-0.0823	-65	340	350	EITLGFVDLLR					Mascot	
1393.6522	1393.5598	-0.0924	-66	22	32	LTYYTPDYETK					Mascot	
1433.7495	1433.6456	-0.1039	-72	320	334	LSGGDHVHAGTVVGK					Mascot	
1451.6219	1451.5237	-0.0982	-68	202	213	DDENVNSQPFMR					Mascot	
1465.7546	1465.6561	-0.0985	-67	147	159	TFQGPPHGIQVER					Mascot	
1465.7546	1465.6561	-0.0985	-67	147	159	TFQGPPHGIQVER	99	100			Mascot	
1472.6978	1472.5931	-0.1047	-71	464	475	EIKFEFEAMDTL					Mascot	
1488.6926	1488.599	-0.0936	-63	464	475	EIKFEFEAMDTL			Oxidation (M)[9]		Mascot	
1502.8512	1502.7471	-0.1041	-69	165	177	YGRPLLGCTIKPK			Carbamidomethyl (C)[8]		Mascot	
1546.7358	1546.6277	-0.1081	-70	451	463	WSPELAAACEVWK			Carbamidomethyl (C)[9]		Mascot	
1812.7714	1812.6377	-0.1337	-74	237	252	GHYLNATAGTCEEMMK			Carbamidomethyl (C)[11]		Mascot	
1828.7662	1828.6273	-0.1389	-76	237	252	GHYLNATAGTCEEMMK			Carbamidomethyl (C)[11], Oxidation (M)[14]		Mascot	
1984.8673	1984.7363	-0.131	-66	237	253	GHYLNATAGTCEEMMKR			Carbamidomethyl (C)[11], Oxidation (M)[14]		Mascot	
2009.059	2008.9052	-0.1538	-77	340	356	EITLGFVDLLRDDFVEK					Mascot	
2169.9868	2169.8264	-0.1604	-74	195	213	GGLDFTKDDENVNSQPFMR					Mascot	
2169.9868	2169.8264	-0.1604	-74	195	213	GGLDFTKDDENVNSQPFMR	107	100			Mascot	
2185.9819	2185.8154	-0.1665	-76	195	213	GGLDFTKDDENVNSQPFMR			Oxidation (M)[18]		Mascot	
3022.4548	3022.2112	-0.2436	-81	259	285	ELGVPIVMHDYLTGGFTANTSLAHYCR			Carbamidomethyl (C)[26]		Mascot	
3022.4548	3022.2112	-0.2436	-81	259	285	ELGVPIVMHDYLTGGFTANTSLAHYCR	110	100	Carbamidomethyl (C)[26]		Mascot	
3038.45	3038.2051	-0.2449	-81	259	285	ELGVPIVMHDYLTGGFTANTSLAHYCR			Carbamidomethyl (C)[26], Oxidation (M)[8]		Mascot	
3854.8718	3854.5588	-0.313	-81	42	79	VTPQPGVPPEEAGAAVAAESSTGTWTTVWTDGLTSLDR	258	100			Mascot	
3854.8718	3854.5588	-0.313	-81	42	79	VTPQPGVPPEEAGAAVAAESSTGTWTTVWTDGLTSLDR					Mascot	

Rank	Protein Name	Accession No	Protein MW	Protein PI	Pep. Count	Protein Score	Protein Score C. I. %	Total Ion Score	Total Ion C. I. %
2	Ribulose bisphosphate carboxylase large chain (Fragment) OS=Geum quellyon GN=rbcL PE=3 SV=1	RBL_GEUQU	50910.8	6.23	21	887	100	722	100

Peptide Information

Calc. Mass	Obsrv. Mass	± da	± ppm	Start Seq.	End Seq.	Sequence	Ion Score	C. I. %	Modification	Rank	Result Type
805.4929	805.4279	-0.065	-81	1	8	VGFKAGVK					Mascot
898.41	898.352	-0.058	-65	296	302	NHGMHFR					Mascot
910.4451	910.3861	-0.059	-65	178	184	AVYECLR			Carbamidomethyl (C)[5]		Mascot
912.4719	912.4047	-0.0672	-74	286	293	AMHAVIDR					Mascot
914.4049	914.3503	-0.0546	-60	296	302	NHGMHFR			Oxidation (M)[4]		Mascot
928.4669	928.4054	-0.0615	-66	286	293	AMHAVIDR			Oxidation (M)[2]		Mascot
1021.5312	1021.4648	-0.0664	-65	23	31	DTDILAAFR					Mascot
1116.583	1116.5121	-0.0709	-63	412	421	VALEACVQAR			Carbamidomethyl (C)[6]		Mascot
1170.5585	1170.5605	0.002	2	294	302	QKNHGMHFR			Oxidation (M)[6]		Mascot
1187.6643	1187.5884	-0.0759	-64	276	285	DNGLLLHIHR					Mascot
1245.6337	1245.5505	-0.0832	-67	208	217	FLFCAEAIFK	62	99.991	Carbamidomethyl (C)[4]		Mascot
1245.6337	1245.5505	-0.0832	-67	208	217	FLFCAEAIFK			Carbamidomethyl (C)[4]		Mascot
1275.7307	1275.6484	-0.0823	-65	330	340	EITLGFVDLLR	88	100			Mascot
1275.7307	1275.6484	-0.0823	-65	330	340	EITLGFVDLLR					Mascot
1393.6522	1393.5598	-0.0924	-66	12	22	LTYYTPDYETK					Mascot
1451.6219	1451.5237	-0.0982	-68	192	203	DDENVNSQPFMR					Mascot
1465.7216	1465.6561	-0.0655	-45	310	324	MSGGDHIHAGTVVGK					Mascot
1465.7546	1465.6561	-0.0985	-67	137	149	TFQGPPHGIQVER	99	100			Mascot
1502.8512	1502.7471	-0.1041	-69	155	167	YGRPLLGCTIKPK			Carbamidomethyl (C)[8]		Mascot
1546.7358	1546.6277	-0.1081	-70	441	453	WSPELAAACEVWK			Carbamidomethyl (C)[9]		Mascot
1812.7714	1812.6377	-0.1337	-74	227	242	GHYLNATAGTCEEMMK			Carbamidomethyl (C)[11]		Mascot
1828.7662	1828.6273	-0.1389	-76	227	242	GHYLNATAGTCEEMMK			Carbamidomethyl (C)[11], Oxidation (M)[14]		Mascot
1984.8673	1984.7363	-0.131	-66	227	243	GHYLNATAGTCEEMMKR			Carbamidomethyl (C)[11], Oxidation (M)[14]		Mascot
2169.9868	2169.8264	-0.1604	-74	185	203	GGLDFTKDDENVNSQPFMR					Mascot
2169.9868	2169.8264	-0.1604	-74	185	203	GGLDFTKDDENVNSQPFMR	107	100			Mascot
2185.9819	2185.8154	-0.1665	-76	185	203	GGLDFTKDDENVNSQPFMR			Oxidation (M)[18]		Mascot
3022.4548	3022.2112	-0.2436	-81	249	275	ELGVPIVMHDYLTGGFTANTSLAHYCR			Carbamidomethyl (C)[26]		Mascot
3022.4548	3022.2112	-0.2436	-81	249	275	ELGVPIVMHDYLTGGFTANTSLAHYCR	110	100	Carbamidomethyl (C)[26]		Mascot
3038.45	3038.2051	-0.2449	-81	249	275	ELGVPIVMHDYLTGGFTANTSLAHYCR			Carbamidomethyl (C)[26], Oxidation (M)[8]		Mascot
3854.8718	3854.5588	-0.313	-81	32	69	VTPQPGVPPEEAGAAVAAESSTGTWTTVWTDGLTSLDR	258	100			Mascot
3854.8718	3854.5588	-0.313	-81	32	69	VTPQPGVPPEEAGAAVAAESSTGTWTTVWTDGLTSLDR					Mascot

Rank	Protein Name	Accession No	Protein MW	Protein PI	Pep. Count	Protein Score	Protein Score C. I. %	Total Ion Score	Total Ion C. I. %
3	Ribulose bisphosphate carboxylase large chain OS=Sesbania sesban GN=rbcL PE=3 SV=1	RBL_SESSE	52358.4	6.09	19	855	100	722	100

Peptide Information

Calc. Mass	Obsrv. Mass	± da	± ppm	Start Seq.	End Seq.	Sequence	Ion Score	C. I. %	Modification	Rank	Result Type
830.4366	830.3821	-0.0545	-66	440	446	EGNEIIR					Mascot
898.41	898.352	-0.058	-65	306	312	NHGMHFR					Mascot
910.4451	910.3861	-0.059	-65	188	194	AVYECLR			Carbamidomethyl (C)[5]		Mascot
912.4719	912.4047	-0.0672	-74	296	303	AMHAVIDR					Mascot
914.4049	914.3503	-0.0546	-60	306	312	NHGMHFR			Oxidation (M)[4]		Mascot
928.4669	928.4054	-0.0615	-66	296	303	AMHAVIDR			Oxidation (M)[2]		Mascot
962.4788	962.4172	-0.0616	-64	228	236	SQAETGEIK					Mascot
1021.5312	1021.4648	-0.0664	-65	33	41	DTDILAAFR					Mascot
1116.583	1116.5121	-0.0709	-63	422	431	VALEACVQAR			Carbamidomethyl (C)[6]		Mascot
1170.5585	1170.5605	0.002	2	304	312	QKNHGMHFR			Oxidation (M)[6]		Mascot
1187.6643	1187.5884	-0.0759	-64	286	295	DNGLLLHIHR					Mascot
1245.6337	1245.5505	-0.0832	-67	218	227	FLFCAEAIFK	62	99.991	Carbamidomethyl (C)[4]		Mascot
1245.6337	1245.5505	-0.0832	-67	218	227	FLFCAEAIFK			Carbamidomethyl (C)[4]		Mascot
1275.7307	1275.6484	-0.0823	-65	340	350	EITLGFVDLLR	88	100			Mascot
1275.7307	1275.6484	-0.0823	-65	340	350	EITLGFVDLLR					Mascot
1393.6522	1393.5598	-0.0924	-66	22	32	LTYYTPDYETK					Mascot
1451.6219	1451.5237	-0.0982	-68	202	213	DDENVNSQPFMR					Mascot
1465.7546	1465.6561	-0.0985	-67	147	159	TFQGPPHGIQVER					Mascot
1465.7546	1465.6561	-0.0985	-67	147	159	TFQGPPHGIQVER	99	100			Mascot
1502.8512	1502.7471	-0.1041	-69	165	177	YGRPLLGCTIKPK			Carbamidomethyl (C)[8]		Mascot
1546.7358	1546.6277	-0.1081	-70	451	463	WSPELAAACEVWK			Carbamidomethyl (C)[9]		Mascot
2169.9868	2169.8264	-0.1604	-74	195	213	GGLDFTKDDENVNSQPFMR					Mascot
2169.9868	2169.8264	-0.1604	-74	195	213	GGLDFTKDDENVNSQPFMR	107	100			Mascot
2185.9819	2185.8154	-0.1665	-76	195	213	GGLDFTKDDENVNSQPFMR			Oxidation (M)[18]		Mascot
3022.4548	3022.2112	-0.2436	-81	259	285	ELGVPIVMHDYLTGGFTANTSLAHYCR			Carbamidomethyl (C)[26]		Mascot
3022.4548	3022.2112	-0.2436	-81	259	285	ELGVPIVMHDYLTGGFTANTSLAHYCR	110	100	Carbamidomethyl (C)[26]		Mascot
3038.45	3038.2051	-0.2449	-81	259	285	ELGVPIVMHDYLTGGFTANTSLAHYCR			Carbamidomethyl (C)[26], Oxidation (M)[8]		Mascot
3854.8718	3854.5588	-0.313	-81	42	79	VTPQPGVPPEEAGAAVAAESSTGTWTTVWTDGLTSLDR	258	100			Mascot
3854.8718	3854.5588	-0.313	-81	42	79	VTPQPGVPPEEAGAAVAAESSTGTWTTVWTDGLTSLDR					Mascot

Rank	Protein Name	Accession No	Protein MW	Protein PI	Pep. Count	Protein Score	Protein Score C. I. %	Total Ion Score	Total Ion C. I. %	
4	Ribulose bisphosphate carboxylase large chain OS=Vitis vinifera GN=rbcL PE=3 SV=2	RBL_VITVI	52485.5	6.33	18	842	100	724	100	Mascot

Peptide Information

Calc. Mass	Obsrv. Mass	± da	± ppm	Start Seq.	End Seq.	Sequence	Ion Score	C. I. %	Modification	Rank	Result Type
830.4366	830.3821	-0.0545	-66	440	446	EGNEIIR					Mascot
898.41	898.352	-0.058	-65	306	312	NHGMHFR					Mascot
910.4451	910.3861	-0.059	-65	188	194	AVYECLR			Carbamidomethyl (C)[5]		Mascot
912.4719	912.4047	-0.0672	-74	296	303	AMHAVIDR					Mascot
914.4049	914.3503	-0.0546	-60	306	312	NHGMHFR			Oxidation (M)[4]		Mascot
928.4669	928.4054	-0.0615	-66	296	303	AMHAVIDR			Oxidation (M)[2]		Mascot
962.4788	962.4172	-0.0616	-64	228	236	SQAETGEIK					Mascot
1116.583	1116.5121	-0.0709	-63	422	431	VALEACVQAR			Carbamidomethyl (C)[6]		Mascot
1170.5585	1170.5605	0.002	2	304	312	QKNHGMHFR			Oxidation (M)[6]		Mascot
1187.6378	1187.5884	-0.0494	-42	440	450	EGNEIIRAASK					Mascot
1245.6337	1245.5505	-0.0832	-67	218	227	FLFCAEAIFK	62	99.991	Carbamidomethyl (C)[4]		Mascot
1245.6337	1245.5505	-0.0832	-67	218	227	FLFCAEAIFK			Carbamidomethyl (C)[4]		Mascot
1275.7307	1275.6484	-0.0823	-65	340	350	EITLGFVDLLR	88	100			Mascot
1275.7307	1275.6484	-0.0823	-65	340	350	EITLGFVDLLR					Mascot
1451.6219	1451.5237	-0.0982	-68	202	213	DDENVNSQPFMR					Mascot
1465.7546	1465.6561	-0.0985	-67	147	159	TFQGPPHGIQVER					Mascot
1465.7546	1465.6561	-0.0985	-67	147	159	TFQGPPHGIQVER	99	100			Mascot
1502.8512	1502.7471	-0.1041	-69	165	177	YGRPLLGCTIKPK			Carbamidomethyl (C)[8]		Mascot
1546.7358	1546.6277	-0.1081	-70	451	463	WSPELAAACEVWK			Carbamidomethyl (C)[9]		Mascot
2009.059	2008.9052	-0.1538	-77	340	356	EITLGFVDLLRDDFVEK					Mascot
2169.9868	2169.8264	-0.1604	-74	195	213	GGLDFTKDDENVNSQPFMR					Mascot
2169.9868	2169.8264	-0.1604	-74	195	213	GGLDFTKDDENVNSQPFMR	107	100			Mascot
2185.9819	2185.8154	-0.1665	-76	195	213	GGLDFTKDDENVNSQPFMR			Oxidation (M)[18]		Mascot
3022.4548	3022.2112	-0.2436	-81	259	285	ELGVPIVMHDYLTGGFTANTSLAHYCR			Carbamidomethyl (C)[26]		Mascot
3022.4548	3022.2112	-0.2436	-81	259	285	ELGVPIVMHDYLTGGFTANTSLAHYCR	110	100	Carbamidomethyl (C)[26]		Mascot
3038.45	3038.2051	-0.2449	-81	259	285	ELGVPIVMHDYLTGGFTANTSLAHYCR			Carbamidomethyl (C)[26], Oxidation (M)[8]		Mascot
3854.8718	3854.5588	-0.313	-81	42	79	VTPQPGVPPEEAGAAVAAESSTGTWTTVWTDGLTSLDR	258	100			Mascot
3854.8718	3854.5588	-0.313	-81	42	79	VTPQPGVPPEEAGAAVAAESSTGTWTTVWTDGLTSLDR					Mascot

Rank	Protein Name	Accession No	Protein MW	Protein PI	Pep. Count	Protein Score	Protein Score C. I. %	Total Ion Score	Total Ion C. I. %
5	Ribulose bisphosphate carboxylase large chain OS=Lactuca sativa GN=rbcL PE=3 SV=2	RBL_LACSA	52861.6	5.91	17	830	100	724	100

Peptide Information

Calc. Mass	Obsrv. Mass	± da	± ppm	Start Seq.	End Seq.	Sequence	Ion Score	C. I. %	Modification	Rank	Result Type
910.4451	910.3861	-0.059	-65	188	194	AVYECLR			Carbamidomethyl (C)[5]		Mascot
912.4719	912.4047	-0.0672	-74	296	303	AMHAVIDR					Mascot
928.4669	928.4054	-0.0615	-66	296	303	AMHAVIDR			Oxidation (M)[2]		Mascot
962.4788	962.4172	-0.0616	-64	228	236	SQAETGEIK					Mascot
1021.5312	1021.4648	-0.0664	-65	33	41	DTDILAAFR					Mascot
1116.583	1116.5121	-0.0709	-63	422	431	VALEACVQAR			Carbamidomethyl (C)[6]		Mascot
1187.6643	1187.5884	-0.0759	-64	286	295	DNGLLLHIHR					Mascot
1245.6337	1245.5505	-0.0832	-67	218	227	FLFCAEAIFK	62	99.991	Carbamidomethyl (C)[4]		Mascot
1245.6337	1245.5505	-0.0832	-67	218	227	FLFCAEAIFK			Carbamidomethyl (C)[4]		Mascot
1275.7307	1275.6484	-0.0823	-65	340	350	EITLGFVDLLR	88	100			Mascot
1275.7307	1275.6484	-0.0823	-65	340	350	EITLGFVDLLR					Mascot
1451.6219	1451.5237	-0.0982	-68	202	213	DDENVNSQPFMR					Mascot
1465.7546	1465.6561	-0.0985	-67	147	159	TFQGPPHGIQVER					Mascot
1465.7546	1465.6561	-0.0985	-67	147	159	TFQGPPHGIQVER	99	100			Mascot
1502.8512	1502.7471	-0.1041	-69	165	177	YGRPLLGCTIKPK			Carbamidomethyl (C)[8]		Mascot
1546.7358	1546.6277	-0.1081	-70	451	463	WSPELAAACEVWK			Carbamidomethyl (C)[9]		Mascot
1812.7714	1812.6377	-0.1337	-74	237	252	GHYLNATAGTCEEMMK			Carbamidomethyl (C)[11]		Mascot
1828.7662	1828.6273	-0.1389	-76	237	252	GHYLNATAGTCEEMMK			Carbamidomethyl (C)[11], Oxidation (M)[14]		Mascot
1984.8673	1984.7363	-0.131	-66	237	253	GHYLNATAGTCEEMMKR			Carbamidomethyl (C)[11], Oxidation (M)[14]		Mascot
2169.9868	2169.8264	-0.1604	-74	195	213	GGLDFTKDDENVNSQPFMR					Mascot
2169.9868	2169.8264	-0.1604	-74	195	213	GGLDFTKDDENVNSQPFMR	107	100			Mascot
2185.9819	2185.8154	-0.1665	-76	195	213	GGLDFTKDDENVNSQPFMR			Oxidation (M)[18]		Mascot
3022.4548	3022.2112	-0.2436	-81	259	285	ELGVPIVMHDYLTGGFTANTSLAHYCR			Carbamidomethyl (C)[26]		Mascot
3022.4548	3022.2112	-0.2436	-81	259	285	ELGVPIVMHDYLTGGFTANTSLAHYCR	110	100	Carbamidomethyl (C)[26]		Mascot
3038.45	3038.2051	-0.2449	-81	259	285	ELGVPIVMHDYLTGGFTANTSLAHYCR			Carbamidomethyl (C)[26], Oxidation (M)[8]		Mascot
3854.8718	3854.5588	-0.313	-81	42	79	VTPQPGVPPEEAGAAVAAESSTGTWTTVWTDGLTSLDR	258	100			Mascot
3854.8718	3854.5588	-0.313	-81	42	79	VTPQPGVPPEEAGAAVAAESSTGTWTTVWTDGLTSLDR					Mascot

Rank	Protein Name	Accession No	Protein MW	Protein PI	Pep. Count	Protein Score	Protein Score C. I. %	Total Ion Score	Total Ion C. I. %	
6	Ribulose bisphosphate carboxylase large chain OS=Ipomoea purpurea GN=rbcL PE=3 SV=1	RBL_IPOPU	53353.9	6.41	22	829	100	662	100	Mascot

Peptide Information

Calc. Mass	Obsrv. Mass	± da	± ppm	Start Seq.	End Seq.	Sequence	Ion Score	C. I. %	Modification	Rank	Result Type
805.4818	805.4279	-0.0539	-67	140	146	IPTAYIK					Mascot
830.4366	830.3821	-0.0545	-66	440	446	EGNEIIR					Mascot
898.41	898.352	-0.058	-65	306	312	NHGMHFR					Mascot
910.4451	910.3861	-0.059	-65	188	194	AVYECLR			Carbamidomethyl (C)[5]		Mascot
912.4719	912.4047	-0.0672	-74	296	303	AMHAVIDR					Mascot
914.4049	914.3503	-0.0546	-60	306	312	NHGMHFR			Oxidation (M)[4]		Mascot
928.4669	928.4054	-0.0615	-66	296	303	AMHAVIDR			Oxidation (M)[2]		Mascot
962.4788	962.4172	-0.0616	-64	228	236	SQAETGEIK					Mascot
1021.5312	1021.4648	-0.0664	-65	33	41	DTDILAAFR					Mascot
1116.583	1116.5121	-0.0709	-63	422	431	VALEACVQAR			Carbamidomethyl (C)[6]		Mascot
1170.5585	1170.5605	0.002	2	304	312	QKNHGMHFR			Oxidation (M)[6]		Mascot
1187.6643	1187.5884	-0.0759	-64	286	295	DNGLLLHIHR					Mascot
1275.7307	1275.6484	-0.0823	-65	340	350	EITLGFVDLLR	88	100			Mascot
1275.7307	1275.6484	-0.0823	-65	340	350	EITLGFVDLLR					Mascot
1451.6219	1451.5237	-0.0982	-68	202	213	DDENVNSQPFMR					Mascot
1465.7546	1465.6561	-0.0985	-67	147	159	TFQGPPHGIQVER					Mascot
1465.7546	1465.6561	-0.0985	-67	147	159	TFQGPPHGIQVER	99	100			Mascot
1502.8512	1502.7471	-0.1041	-69	165	177	YGRPLLGCTIKPK			Carbamidomethyl (C)[8]		Mascot
1546.7358	1546.6277	-0.1081	-70	451	463	WSPELAAACEVWK			Carbamidomethyl (C)[9]		Mascot
1812.7714	1812.6377	-0.1337	-74	237	252	GHYLNATAGTCEEMMK			Carbamidomethyl (C)[11]		Mascot
1828.7662	1828.6273	-0.1389	-76	237	252	GHYLNATAGTCEEMMK			Carbamidomethyl (C)[11], Oxidation (M)[14]		Mascot
1984.8673	1984.7363	-0.131	-66	237	253	GHYLNATAGTCEEMMKR			Carbamidomethyl (C)[11], Oxidation (M)[14]		Mascot
2033.9572	2033.85	-0.1072	-53	447	463	QACKWSPELAAACEVWK			Carbamidomethyl (C)[3,13]		Mascot
2169.9868	2169.8264	-0.1604	-74	195	213	GGLDFTKDDENVNSQPFMR					Mascot
2169.9868	2169.8264	-0.1604	-74	195	213	GGLDFTKDDENVNSQPFMR	107	100			Mascot
2185.9819	2185.8154	-0.1665	-76	195	213	GGLDFTKDDENVNSQPFMR			Oxidation (M)[18]		Mascot
2280.1506	2280.0146	-0.136	-60	340	358	EITLGFVDLLRDDFVEQDR					Mascot
3022.4548	3022.2112	-0.2436	-81	259	285	ELGVPIVMHDYLTGGFTANTSLAHYCR			Carbamidomethyl (C)[26]		Mascot
3022.4548	3022.2112	-0.2436	-81	259	285	ELGVPIVMHDYLTGGFTANTSLAHYCR	110	100	Carbamidomethyl (C)[26]		Mascot
3038.45	3038.2051	-0.2449	-81	259	285	ELGVPIVMHDYLTGGFTANTSLAHYCR			Carbamidomethyl (C)[26], Oxidation (M)[8]		Mascot
3854.8718	3854.5588	-0.313	-81	42	79	VTPQPGVPPEEAGAAVAAESSTGTWTTVWTDGLTSLDR	258	100			Mascot
3854.8718	3854.5588	-0.313	-81	42	79	VTPQPGVPPEEAGAAVAAESSTGTWTTVWTDGLTSLDR					Mascot

Rank	Protein Name	Accession No	Protein MW	Protein PI	Pep. Count	Protein Score	Protein Score C. I. %	Total Ion Score	Total Ion C. I. %
7	Ribulose bisphosphate carboxylase large chain (Fragment) OS=Manilkara zapota GN=rbcL PE=3 SV=2	RBL_MANZA	51550	6.04	21	828	100	662	100

Peptide Information

Calc. Mass	Obsrv. Mass	± da	± ppm	Start Seq.	End Seq.	Sequence	Ion Score	C. I. %	Modification	Rank	Result Type
805.4929	805.4279	-0.065	-81	1	8	VGFKAGVK					Mascot
830.4366	830.3821	-0.0545	-66	430	436	EGNEIIR					Mascot
898.41	898.352	-0.058	-65	296	302	NHGMHFR					Mascot
910.4451	910.3861	-0.059	-65	178	184	AVYECLR			Carbamidomethyl (C)[5]		Mascot
912.4719	912.4047	-0.0672	-74	286	293	AMHAVIDR					Mascot
914.4049	914.3503	-0.0546	-60	296	302	NHGMHFR			Oxidation (M)[4]		Mascot
928.4669	928.4054	-0.0615	-66	286	293	AMHAVIDR			Oxidation (M)[2]		Mascot
1021.5312	1021.4648	-0.0664	-65	23	31	DTDILAAFR					Mascot
1116.583	1116.5121	-0.0709	-63	412	421	VALEACVQAR			Carbamidomethyl (C)[6]		Mascot
1170.5585	1170.5605	0.002	2	294	302	QKNHGMHFR			Oxidation (M)[6]		Mascot
1187.6643	1187.5884	-0.0759	-64	276	285	DNGLLLHIHR					Mascot
1245.6433	1245.5505	-0.0928	-74	430	440	EGNEIIREASK					Mascot
1245.6433	1245.5505	-0.0928	-74	430	440	EGNEIIREASK					Mascot
1275.7307	1275.6484	-0.0823	-65	330	340	EITLGFVDLLR	88	100			Mascot
1275.7307	1275.6484	-0.0823	-65	330	340	EITLGFVDLLR					Mascot
1393.6522	1393.5598	-0.0924	-66	12	22	LTYYTPDYETK					Mascot
1433.7495	1433.6456	-0.1039	-72	310	324	LSGGDHVHAGTVVGK					Mascot
1451.6219	1451.5237	-0.0982	-68	192	203	DDENVNSQPFMR					Mascot
1465.7546	1465.6561	-0.0985	-67	137	149	TFQGPPHGIQVER					Mascot
1465.7546	1465.6561	-0.0985	-67	137	149	TFQGPPHGIQVER	99	100			Mascot
1472.6978	1472.5931	-0.1047	-71	454	465	EIKFEFEAMDTL					Mascot
1488.6926	1488.599	-0.0936	-63	454	465	EIKFEFEAMDTL			Oxidation (M)[9]		Mascot
1502.8512	1502.7471	-0.1041	-69	155	167	YGRPLLGCTIKPK			Carbamidomethyl (C)[8]		Mascot
1546.7358	1546.6277	-0.1081	-70	441	453	WSPELAAACEVWK			Carbamidomethyl (C)[9]		Mascot
2169.9868	2169.8264	-0.1604	-74	185	203	GGLDFTKDDENVNSQPFMR					Mascot
2169.9868	2169.8264	-0.1604	-74	185	203	GGLDFTKDDENVNSQPFMR	107	100			Mascot
2185.9819	2185.8154	-0.1665	-76	185	203	GGLDFTKDDENVNSQPFMR			Oxidation (M)[18]		Mascot
3022.4548	3022.2112	-0.2436	-81	249	275	ELGVPIVMHDYLTGGFTANTSLAHYCR			Carbamidomethyl (C)[26]		Mascot
3022.4548	3022.2112	-0.2436	-81	249	275	ELGVPIVMHDYLTGGFTANTSLAHYCR	110	100	Carbamidomethyl (C)[26]		Mascot
3038.45	3038.2051	-0.2449	-81	249	275	ELGVPIVMHDYLTGGFTANTSLAHYCR			Carbamidomethyl (C)[26], Oxidation (M)[8]		Mascot
3854.8718	3854.5588	-0.313	-81	32	69	VTPQPGVPPEEAGAAVAAESSTGTWTTVWTDGLTSLDR	258	100			Mascot
3854.8718	3854.5588	-0.313	-81	32	69	VTPQPGVPPEEAGAAVAAESSTGTWTTVWTDGLTSLDR					Mascot

Rank	Protein Name	Accession No	Protein MW	Protein PI	Pep. Count	Protein Score	Protein Score C. I. %	Total Ion Score	Total Ion C. I. %
8	Ribulose bisphosphate carboxylase large chain (Fragment) OS=Oxalis dillenii GN=rbcL PE=3 SV=2	RBL_OXADI	51653.9	5.91	16	826	100	721	100

Peptide Information

Calc. Mass	Obsrv. Mass	± da	± ppm	Start Seq.	End Seq.	Sequence	Ion Score	C. I. %	Modification	Rank	Result Type
830.4366	830.3821	-0.0545	-66	431	437	EGNEIIR					Mascot
910.4451	910.3861	-0.059	-65	179	185	AVYECLR			Carbamidomethyl (C)[5]		Mascot
912.4719	912.4047	-0.0672	-74	287	294	AMHAVIDR					Mascot
928.4669	928.4054	-0.0615	-66	287	294	AMHAVIDR			Oxidation (M)[2]		Mascot
1021.5312	1021.4648	-0.0664	-65	24	32	DTDILAAFR					Mascot
1116.583	1116.5121	-0.0709	-63	413	422	VALEACVQAR			Carbamidomethyl (C)[6]		Mascot
1187.6643	1187.5884	-0.0759	-64	277	286	DNGLLLHIHR					Mascot
1245.6337	1245.5505	-0.0832	-67	209	218	FLFCAEAIFK	62	99.991	Carbamidomethyl (C)[4]		Mascot
1245.6337	1245.5505	-0.0832	-67	209	218	FLFCAEAIFK			Carbamidomethyl (C)[4]		Mascot
1275.7307	1275.6484	-0.0823	-65	331	341	EITLGFVDLLR	88	100			Mascot
1275.7307	1275.6484	-0.0823	-65	331	341	EITLGFVDLLR					Mascot
1451.6219	1451.5237	-0.0982	-68	193	204	DDENVNSQPFMR					Mascot
1465.7546	1465.6561	-0.0985	-67	138	150	TFQGPPHGIQVER					Mascot
1465.7546	1465.6561	-0.0985	-67	138	150	TFQGPPHGIQVER	99	100			Mascot
1502.8512	1502.7471	-0.1041	-69	156	168	YGRPLLGCTIKPK			Carbamidomethyl (C)[8]		Mascot
1546.7358	1546.6277	-0.1081	-70	442	454	WSPELAAACEVWK			Carbamidomethyl (C)[9]		Mascot
2009.059	2008.9052	-0.1538	-77	331	347	EITLGFVDLLRDDFVEK					Mascot
2169.9868	2169.8264	-0.1604	-74	186	204	GGLDFTKDDENVNSQPFMR					Mascot
2169.9868	2169.8264	-0.1604	-74	186	204	GGLDFTKDDENVNSQPFMR	107	100			Mascot
2185.9819	2185.8154	-0.1665	-76	186	204	GGLDFTKDDENVNSQPFMR			Oxidation (M)[18]		Mascot
3022.4548	3022.2112	-0.2436	-81	250	276	ELGVPIVMHDYLTGGFTANTSLAHYCR			Carbamidomethyl (C)[26]		Mascot
3022.4548	3022.2112	-0.2436	-81	250	276	ELGVPIVMHDYLTGGFTANTSLAHYCR	110	100	Carbamidomethyl (C)[26]		Mascot
3038.45	3038.2051	-0.2449	-81	250	276	ELGVPIVMHDYLTGGFTANTSLAHYCR			Carbamidomethyl (C)[26], Oxidation (M)[8]		Mascot
3854.8718	3854.5588	-0.313	-81	33	70	VTPQPGVPPEEAGAAVAAESSTGTWTTVWTDGLTSLDR	258	100			Mascot
3854.8718	3854.5588	-0.313	-81	33	70	VTPQPGVPPEEAGAAVAAESSTGTWTTVWTDGLTSLDR					Mascot

Rank	Protein Name	Accession No	Protein MW	Protein PI	Pep. Count	Protein Score	Protein Score C. I. %	Total Ion Score	Total Ion C. I. %
9	Ribulose bisphosphate carboxylase large chain (Fragment) OS=Prunus domestica GN=rbcL PE=3 SV=1	RBL_PRUDO	50914.6	6.23	21	824	100	661	100

Peptide Information

Calc. Mass	Obsrv. Mass	± da	± ppm	Start Seq.	End Seq.	Sequence	Ion Score	C. I. %	Modification	Rank	Result Type
805.4929	805.4279	-0.065	-81	1	8	VGFKAGVK					Mascot
830.4366	830.3821	-0.0545	-66	430	436	EGNEIIR					Mascot
898.41	898.352	-0.058	-65	296	302	NHGMHFR					Mascot
910.4451	910.3861	-0.059	-65	178	184	AVYECLR			Carbamidomethyl (C)[5]		Mascot
912.4719	912.4047	-0.0672	-74	286	293	AMHAVIDR					Mascot
914.4049	914.3503	-0.0546	-60	296	302	NHGMHFR			Oxidation (M)[4]		Mascot
928.4669	928.4054	-0.0615	-66	286	293	AMHAVIDR			Oxidation (M)[2]		Mascot
1021.5312	1021.4648	-0.0664	-65	23	31	DTDILAAFR					Mascot
1116.583	1116.5121	-0.0709	-63	412	421	VALEACVQAR			Carbamidomethyl (C)[6]		Mascot
1170.5585	1170.5605	0.002	2	294	302	QKNHGMHFR			Oxidation (M)[6]		Mascot
1187.6643	1187.5884	-0.0759	-64	276	285	DNGLLLHIHR					Mascot
1245.6433	1245.5505	-0.0928	-74	430	440	EGNEIIREASK					Mascot
1245.6433	1245.5505	-0.0928	-74	430	440	EGNEIIREASK					Mascot
1275.7307	1275.6484	-0.0823	-65	330	340	EITLGFVDLLR	88	100			Mascot
1275.7307	1275.6484	-0.0823	-65	330	340	EITLGFVDLLR					Mascot
1393.6522	1393.5598	-0.0924	-66	12	22	LTYYTPDYETK					Mascot
1451.6219	1451.5237	-0.0982	-68	192	203	DDENVNSQPFMR					Mascot
1465.7216	1465.6561	-0.0655	-45	310	324	MSGGDHIHAGTVVGK					Mascot
1465.7546	1465.6561	-0.0985	-67	137	149	TFQGPPHGIQVER	99	100			Mascot
1502.8512	1502.7471	-0.1041	-69	155	167	YGRPLLGCTIKPK			Carbamidomethyl (C)[8]		Mascot
1546.7358	1546.6277	-0.1081	-70	441	453	WSPELAAACEVWK			Carbamidomethyl (C)[9]		Mascot
2009.059	2008.9052	-0.1538	-77	330	346	EITLGFVDLLRDDFVEK					Mascot
2169.9868	2169.8264	-0.1604	-74	185	203	GGLDFTKDDENVNSQPFMR					Mascot
2169.9868	2169.8264	-0.1604	-74	185	203	GGLDFTKDDENVNSQPFMR	107	100			Mascot
2185.9819	2185.8154	-0.1665	-76	185	203	GGLDFTKDDENVNSQPFMR			Oxidation (M)[18]		Mascot
3022.4548	3022.2112	-0.2436	-81	249	275	ELGVPIVMHDYLTGGFTANTSLAHYCR			Carbamidomethyl (C)[26]		Mascot
3022.4548	3022.2112	-0.2436	-81	249	275	ELGVPIVMHDYLTGGFTANTSLAHYCR	110	100	Carbamidomethyl (C)[26]		Mascot
3038.45	3038.2051	-0.2449	-81	249	275	ELGVPIVMHDYLTGGFTANTSLAHYCR			Carbamidomethyl (C)[26], Oxidation (M)[8]		Mascot
3854.8718	3854.5588	-0.313	-81	32	69	VTPQPGVPPEEAGAAVAAESSTGTWTTVWTDGLTSLDR	258	100			Mascot
3854.8718	3854.5588	-0.313	-81	32	69	VTPQPGVPPEEAGAAVAAESSTGTWTTVWTDGLTSLDR					Mascot

Rank	Protein Name	Accession No	Protein MW	Protein PI	Pep. Count	Protein Score	Protein Score C. I. %	Total Ion Score	Total Ion C. I. %
10	Ribulose bisphosphate carboxylase large chain OS=Phaseolus vulgaris GN=rbcL PE=3 SV=1	RBL_PHAVU	52767.6	5.96	20	809	100	662	100

Peptide Information

Calc. Mass	Obsrv. Mass	± da	± ppm	Start Seq.	End Seq.	Sequence	Ion Score	C. I. %	Modification	Rank	Result Type
805.4818	805.4279	-0.0539	-67	140	146	IPTAYIK					Mascot
830.4366	830.3821	-0.0545	-66	440	446	EGNEIIR					Mascot
898.41	898.352	-0.058	-65	306	312	NHGMHFR					Mascot
910.4451	910.3861	-0.059	-65	188	194	AVYECLR			Carbamidomethyl (C)[5]		Mascot
912.4719	912.4047	-0.0672	-74	296	303	AMHAVIDR					Mascot
914.4049	914.3503	-0.0546	-60	306	312	NHGMHFR			Oxidation (M)[4]		Mascot
928.4669	928.4054	-0.0615	-66	296	303	AMHAVIDR			Oxidation (M)[2]		Mascot
1021.5312	1021.4648	-0.0664	-65	33	41	DTDILAAFR					Mascot
1116.583	1116.5121	-0.0709	-63	422	431	VALEACVQAR			Carbamidomethyl (C)[6]		Mascot
1170.5585	1170.5605	0.002	2	304	312	QKNHGMHFR			Oxidation (M)[6]		Mascot
1187.6643	1187.5884	-0.0759	-64	286	295	DNGLLLHIHR					Mascot
1245.6433	1245.5505	-0.0928	-74	440	450	EGNEIIREASK					Mascot
1245.6433	1245.5505	-0.0928	-74	440	450	EGNEIIREASK					Mascot
1275.7307	1275.6484	-0.0823	-65	340	350	EITLGFVDLLR	88	100			Mascot
1275.7307	1275.6484	-0.0823	-65	340	350	EITLGFVDLLR					Mascot
1393.6522	1393.5598	-0.0924	-66	22	32	LTYYTPDYETK					Mascot
1451.6219	1451.5237	-0.0982	-68	202	213	DDENVNSQPFMR					Mascot
1465.7546	1465.6561	-0.0985	-67	147	159	TFQGPPHGIQVER					Mascot
1465.7546	1465.6561	-0.0985	-67	147	159	TFQGPPHGIQVER	99	100			Mascot
1502.8512	1502.7471	-0.1041	-69	165	177	YGRPLLGCTIKPK			Carbamidomethyl (C)[8]		Mascot
1546.7358	1546.6277	-0.1081	-70	451	463	WSPELAAACEVWK			Carbamidomethyl (C)[9]		Mascot
2034.0363	2033.85	-0.1863	-92	320	339	LSGGDHVHSGTVVGKLEGER					Mascot
2169.9868	2169.8264	-0.1604	-74	195	213	GGLDFTKDDENVNSQPFMR					Mascot
2169.9868	2169.8264	-0.1604	-74	195	213	GGLDFTKDDENVNSQPFMR	107	100			Mascot
2185.9819	2185.8154	-0.1665	-76	195	213	GGLDFTKDDENVNSQPFMR			Oxidation (M)[18]		Mascot
3022.4548	3022.2112	-0.2436	-81	259	285	ELGVPIVMHDYLTGGFTANTSLAHYCR			Carbamidomethyl (C)[26]		Mascot
3022.4548	3022.2112	-0.2436	-81	259	285	ELGVPIVMHDYLTGGFTANTSLAHYCR	110	100	Carbamidomethyl (C)[26]		Mascot
3038.45	3038.2051	-0.2449	-81	259	285	ELGVPIVMHDYLTGGFTANTSLAHYCR			Carbamidomethyl (C)[26], Oxidation (M)[8]		Mascot
3854.8718	3854.5588	-0.313	-81	42	79	VTPQPGVPPEEAGAAVAAESSTGTWTTVWTDGLTSLDR	258	100			Mascot
3854.8718	3854.5588	-0.313	-81	42	79	VTPQPGVPPEEAGAAVAAESSTGTWTTVWTDGLTSLDR					Mascot

Gel Idx/Pos	46/B19	Instr./Gel Origin	BA2060/131017A	Process Sta	Analysis Succeeded
Plate [#] Name	[1] 31496	Instrument Sample Name		Spectra	8

Rank	Protein Name	Accession No	Protein MW	Protein PI	Pep. Count	Protein Score	Protein Score C. I. %	Total Ion Score	Total Ion C. I. %
1	ATP synthase subunit beta, chloroplastic OS=Gl max GN=atpB PE=3 SV=1	ATPB_SOYBI	53721.1	5.29	28	877	100	614	100

Peptide Information

Calc. Mass	Obsrv. Mass	± da	± ppm	Start Seq.	End Seq.	Sequence	Ion Score	C. I. %	Modification	Rank	Result Type
811.4421	811.3904	-0.0517	-64	128	134	TTSPIHR					Mascot
975.5621	975.5021	-0.06	-62	168	178	IGLFGGAGVGK					Mascot
991.5822	991.5178	-0.0644	-65	146	154	LAIFETGIK					Mascot
1045.604	1045.5377	-0.0663	-63	155	163	VVDLLAPYR					Mascot
1190.6416	1190.5685	-0.0731	-61	135	145	SAPAFIQLDTK					Mascot
1236.6075	1236.5315	-0.076	-61	76	87	AVAMSATEGLMR					Mascot
1245.5126	1245.5527	0.0401	32	208	217	EGNDLYMEMK			Oxidation (M)[7]		Mascot
1252.6024	1252.5243	-0.0781	-62	76	87	AVAMSATEGLMR			Oxidation (M)[4]		Mascot
1275.7129	1275.6504	-0.0625	-49	40	50	MPNIYNALIVK					Mascot
1275.7129	1275.6504	-0.0625	-49	40	50	MPNIYNALIVK	13	0			Mascot
1328.6705	1328.5869	-0.0836	-63	192	205	AHGGVSVFGGVGER					Mascot
1393.6991	1393.5603	-0.1388	-100	487	498	ATNLEMESNLKK			Oxidation (M)[6]		Mascot
1431.6863	1431.594	-0.0923	-64	379	390	IVGEEHYETAQR					Mascot
1433.7747	1433.6796	-0.0951	-66	278	291	FVQAGSEVSALLGR	76	100			Mascot
1433.7747	1433.6796	-0.0951	-66	278	291	FVQAGSEVSALLGR					Mascot
1471.7614	1471.668	-0.0934	-63	249	261	VGLTALTMAEYFR					Mascot
1471.7614	1471.668	-0.0934	-63	249	261	VGLTALTMAEYFR	45	99.665			Mascot
1486.6665	1486.5713	-0.0952	-64	206	217	TREGNDLYMEMK					Mascot
1487.7562	1487.6564	-0.0998	-67	249	261	VGLTALTMAEYFR			Oxidation (M)[8]		Mascot
1502.6614	1502.7509	0.0895	60	206	217	TREGNDLYMEMK			Oxidation (M)[9]		Mascot
1601.8104	1601.7039	-0.1065	-66	232	246	VALVYGQMNEPPGAR	80	100			Mascot
1601.8104	1601.7039	-0.1065	-66	232	246	VALVYGQMNEPPGAR					Mascot
1617.8053	1617.6959	-0.1094	-68	232	246	VALVYGQMNEPPGAR			Oxidation (M)[8]		Mascot
1735.0153	1734.8967	-0.1186	-68	23	39	IAQIIGPVLDVAFPPGK					Mascot
1949.9967	1949.8654	-0.1313	-67	262	277	DVNEQDVLLFIDNIFR					Mascot
1949.9967	1949.8654	-0.1313	-67	262	277	DVNEQDVLLFIDNIFR	158	100			Mascot
1969.0388	1968.9038	-0.135	-69	110	127	IFNVLGEPIDNLGPVDTR					Mascot
2061.0322	2060.8909	-0.1413	-69	360	378	GIYPAVDPLDSTSTMLQPR					Mascot
2071.0852	2070.9404	-0.1448	-70	88	109	GMEVIDTGAALSVPVGGATLGR					Mascot
2071.0852	2070.9404	-0.1448	-70	88	109	GMEVIDTGAALSVPVGGATLGR	100	100			Mascot
2077.0271	2076.8875	-0.1396	-67	360	378	GIYPAVDPLDSTSTMLQPR			Oxidation (M)[15]		Mascot
2087.0801	2086.9346	-0.1455	-70	88	109	GMEVIDTGAALSVPVGGATLGR			Oxidation (M)[2]		Mascot
2171.1077	2170.8914	-0.2163	-100	400	418	ELQDIIAILGLDELSEEDR					Mascot
2282.0791	2281.9192	-0.1599	-70	292	312	MPSAVGYQPTLSTEMGSLQER					Mascot
2298.074	2297.9141	-0.1599	-70	292	312	MPSAVGYQPTLSTEMGSLQER			Oxidation (M)[1]		Mascot
2314.0688	2313.9443	-0.1245	-54	292	312	MPSAVGYQPTLSTEMGSLQER			Oxidation (M)[1,15]		Mascot
2386.178	2386.009	-0.169	-71	53	73	DTVGQQINVTCEVQQLLGNNR	144	100	Carbamidomethyl (C)[11]		Mascot
2386.178	2386.009	-0.169	-71	53	73	DTVGQQINVTCEVQQLLGNNR			Carbamidomethyl (C)[11]		Mascot
2462.2661	2462.0957	-0.1704	-69	398	418	YKELQDIIAILGLDELSEEDR					Mascot
2599.3005	2599.1221	-0.1784	-69	51	73	GRDTVGQQINVTCEVQQLLGNNR			Carbamidomethyl (C)[13]		Mascot
2711.446	2711.252	-0.194	-72	400	423	ELQDIIAILGLDELSEEDRLTVAR					Mascot
2876.4927	2876.2935	-0.1992	-69	460	486	LILSGELDGLPEQAFYLVGNIDEATAK					Mascot
3843.9287	3843.6577	-0.271	-71	318	354	EGSITSIQAVYVPADDLTDPAPATTFAHLDATTVLSR					Mascot

Rank	Protein Name	Accession No	Protein MW	Protein PI	Pep. Count	Protein Score	Protein Score C. I. %	Total Ion Score	Total Ion C. I. %
2	ATP synthase subunit beta, chloroplastic OS=Ot siberica GN=atpB PE=3 SV=1	ATPB_OTHSI	53309.8	5.22	22	778	100	602	100

Peptide Information

Calc. Mass	Obsrv. Mass	± da	± ppm	Start Seq.	End Seq.	Sequence	Ion Score	C. I. %	Modification	Rank	Result Type
811.4421	811.3904	-0.0517	-64	128	134	TTSPIHR					Mascot
975.5621	975.5021	-0.06	-62	168	178	IGLFGGAGVGK					Mascot
1021.5676	1021.4698	-0.0978	-96	448	456	YVGLAETIR					Mascot
1045.604	1045.5377	-0.0663	-63	155	163	VVDLLAPYR					Mascot
1190.6416	1190.5685	-0.0731	-61	135	145	SAPAFIQLDTK					Mascot
1245.5126	1245.5527	0.0401	32	208	217	EGNDLYMEMK			Oxidation (M)[7]		Mascot
1328.6705	1328.5869	-0.0836	-63	192	205	AHGGVSVFGGVGER					Mascot
1431.6863	1431.594	-0.0923	-64	379	390	IVGEEHYETAQR					Mascot
1433.7747	1433.6796	-0.0951	-66	278	291	FVQAGSEVSALLGR	76	100			Mascot
1433.7747	1433.6796	-0.0951	-66	278	291	FVQAGSEVSALLGR					Mascot
1471.7614	1471.668	-0.0934	-63	249	261	VGLTALTMAEYFR					Mascot
1471.7614	1471.668	-0.0934	-63	249	261	VGLTALTMAEYFR	45	99.665			Mascot
1486.6665	1486.5713	-0.0952	-64	206	217	TREGNDLYMEMK					Mascot
1487.7562	1487.6564	-0.0998	-67	249	261	VGLTALTMAEYFR			Oxidation (M)[8]		Mascot
1502.6614	1502.7509	0.0895	60	206	217	TREGNDLYMEMK			Oxidation (M)[9]		Mascot
1601.8104	1601.7039	-0.1065	-66	232	246	VALVYGQMNEPPGAR	80	100			Mascot
1601.8104	1601.7039	-0.1065	-66	232	246	VALVYGQMNEPPGAR					Mascot Mascot
1617.8053	1617.6959	-0.1094	-68	232	246	VALVYGQMNEPPGAR			Oxidation (M)[8]		Mascot
1949.9967	1949.8654	-0.1313	-67	262	277	DVNEQDVLLFIDNIFR					Mascot
1949.9967	1949.8654	-0.1313	-67	262	277	DVNEQDVLLFIDNIFR	158	100			Mascot
2061.0322	2060.8909	-0.1413	-69	360	378	GIYPAVDPLDSTSTMLQPR					Mascot
2071.0852	2070.9404	-0.1448	-70	88	109	GMEVIDTGAALSVPVGGATLGR					Mascot
2071.0852	2070.9404	-0.1448	-70	88	109	GMEVIDTGAALSVPVGGATLGR	100	100			Mascot
2077.0271	2076.8875	-0.1396	-67	360	378	GIYPAVDPLDSTSTMLQPR			Oxidation (M)[15]		Mascot
2087.0801	2086.9346	-0.1455	-70	88	109	GMEVIDTGAALSVPVGGATLGR			Oxidation (M)[2]		Mascot
2171.1077	2170.8914	-0.2163	-100	400	418	ELQDIIAILGLDELSEEDR					Mascot
2282.0791	2281.9192	-0.1599	-70	292	312	MPSAVGYQPTLSTEMGSLQER					Mascot
2298.074	2297.9141	-0.1599	-70	292	312	MPSAVGYQPTLSTEMGSLQER			Oxidation (M)[1]		Mascot
2314.0688	2313.9443	-0.1245	-54	292	312	MPSAVGYQPTLSTEMGSLQER			Oxidation (M)[1,15]		Mascot
2386.178	2386.009	-0.169	-71	53	73	DTVGQQINVTCEVQQLLGNNR	144	100	Carbamidomethyl (C)[11]		Mascot
2386.178	2386.009	-0.169	-71	53	73	DTVGQQINVTCEVQQLLGNNR			Carbamidomethyl (C)[11]		Mascot
2462.2661	2462.0957	-0.1704	-69	398	418	YKELQDIIAILGLDELSEEDR					Mascot
2599.3005	2599.1221	-0.1784	-69	51	73	GRDTVGQQINVTCEVQQLLGNNR			Carbamidomethyl (C)[13]		Mascot
2711.446	2711.252	-0.194	-72	400	423	ELQDIIAILGLDELSEEDRLTVAR					Mascot
3843.9287	3843.6577	-0.271	-71	318	354	EGSITSIQAVYVPADDLTDPAPATTFAHLDATTVLSR					Mascot

Rank	Protein Name	Accession No	Protein MW	Protein PI	Pep. Count	Protein Score	Protein Score C. I. %	Total Ion Score	Total Ion C. I. %
3	ATP synthase subunit beta, chloroplastic OS=Lachenalia pusilla GN=atpB PE=3 SV=1	ATPB_LACPU	53030.6	5.15	21	766	100	602	100

Peptide Information

Calc. Mass	Obsrv. Mass	± da	± ppm	Start Seq.	End Seq.	Sequence	Ion Score	C. I. %	Modification	Rank	Result Type
811.4421	811.3904	-0.0517	-64	126	132	TTSPIHR					Mascot
975.5621	975.5021	-0.06	-62	166	176	IGLFGGAGVGK					Mascot
1045.604	1045.5377	-0.0663	-63	153	161	VVDLLAPYR					Mascot
1190.6416	1190.5685	-0.0731	-61	133	143	SAPAFIQLDTK					Mascot
1245.5126	1245.5527	0.0401	32	206	215	EGNDLYMEMK			Oxidation (M)[7]		Mascot
1328.6705	1328.5869	-0.0836	-63	190	203	AHGGVSVFGGVGER					Mascot
1431.6863	1431.594	-0.0923	-64	377	388	IVGEEHYETAQR					Mascot
1433.7747	1433.6796	-0.0951	-66	276	289	FVQAGSEVSALLGR	76	100			Mascot
1433.7747	1433.6796	-0.0951	-66	276	289	FVQAGSEVSALLGR					Mascot
1471.7614	1471.668	-0.0934	-63	247	259	VGLTALTMAEYFR					Mascot
1471.7614	1471.668	-0.0934	-63	247	259	VGLTALTMAEYFR	45	99.665			Mascot
1486.6665	1486.5713	-0.0952	-64	204	215	TREGNDLYMEMK					Mascot
1487.7562	1487.6564	-0.0998	-67	247	259	VGLTALTMAEYFR			Oxidation (M)[8]		Mascot
1502.6614	1502.7509	0.0895	60	204	215	TREGNDLYMEMK			Oxidation (M)[9]		Mascot
1601.8104	1601.7039	-0.1065	-66	230	244	VALVYGQMNEPPGAR	80	100			Mascot
1601.8104	1601.7039	-0.1065	-66	230	244	VALVYGQMNEPPGAR					Mascot
1617.8053	1617.6959	-0.1094	-68	230	244	VALVYGQMNEPPGAR			Oxidation (M)[8]		Mascot
1949.9967	1949.8654	-0.1313	-67	260	275	DVNEQDVLLFIDNIFR					Mascot
1949.9967	1949.8654	-0.1313	-67	260	275	DVNEQDVLLFIDNIFR	158	100			Mascot
2061.0322	2060.8909	-0.1413	-69	358	376	GIYPAVDPLDSTSTMLQPR					Mascot
2071.0852	2070.9404	-0.1448	-70	86	107	GMEVIDTGAALSVPVGGATLGR					Mascot
2071.0852	2070.9404	-0.1448	-70	86	107	GMEVIDTGAALSVPVGGATLGR	100	100			Mascot
2077.0271	2076.8875	-0.1396	-67	358	376	GIYPAVDPLDSTSTMLQPR			Oxidation (M)[15]		Mascot
2087.0801	2086.9346	-0.1455	-70	86	107	GMEVIDTGAALSVPVGGATLGR			Oxidation (M)[2]		Mascot
2171.1077	2170.8914	-0.2163	-100	398	416	ELQDIIAILGLDELSEEDR					Mascot
2282.0791	2281.9192	-0.1599	-70	290	310	MPSAVGYQPTLSTEMGSLQER					Mascot
2298.074	2297.9141	-0.1599	-70	290	310	MPSAVGYQPTLSTEMGSLQER			Oxidation (M)[1]		Mascot
2314.0688	2313.9443	-0.1245	-54	290	310	MPSAVGYQPTLSTEMGSLQER			Oxidation (M)[1,15]		Mascot
2386.178	2386.009	-0.169	-71	51	71	DTVGQQINVTCEVQQLLGNNR	144	100	Carbamidomethyl (C)[11]		Mascot
2386.178	2386.009	-0.169	-71	51	71	DTVGQQINVTCEVQQLLGNNR			Carbamidomethyl (C)[11]		Mascot
2462.2661	2462.0957	-0.1704	-69	396	416	YKELQDIIAILGLDELSEEDR					Mascot
2599.3005	2599.1221	-0.1784	-69	49	71	GRDTVGQQINVTCEVQQLLGNNR			Carbamidomethyl (C)[13]		Mascot
2711.446	2711.252	-0.194	-72	398	421	ELQDIIAILGLDELSEEDRLTVAR					Mascot
3843.9287	3843.6577	-0.271	-71	316	352	EGSITSIQAVYVPADDLTDPAPATTFAHLDATTVLSR					Mascot

Rank	Protein Name	Accession No	Protein MW	Protein PI	Pep. Count	Protein Score	Protein Score C. I. %	Total Ion Score	Total Ion C. I. %
4	ATP synthase subunit beta, chloroplastic OS=Eu montana GN=atpB PE=3 SV=1	ATPB_EUCM	53317.8	5.16	22	739	100	563	100

Peptide Information

Calc. Mass	Obsrv. Mass	± da	± ppm	Start Seq.	End Seq.	Sequence	Ion Score	C. I. %	Modification	Rank	Result Type
811.4421	811.3904	-0.0517	-64	128	134	TTSPIHR					Mascot
975.5621	975.5021	-0.06	-62	168	178	IGLFGGAGVGK					Mascot
1045.604	1045.5377	-0.0663	-63	155	163	VVDLLAPYR					Mascot
1190.6416	1190.5685	-0.0731	-61	135	145	SAPAFIQLDTK					Mascot
1245.5126	1245.5527	0.0401	32	208	217	EGNDLYMEMK			Oxidation (M)[7]		Mascot
1328.6705	1328.5869	-0.0836	-63	192	205	AHGGVSVFGGVGER					Mascot
1431.6863	1431.594	-0.0923	-64	379	390	IVGEEHYETAQR					Mascot
1433.7747	1433.6796	-0.0951	-66	278	291	FVQAGSEVSALLGR	76	100			Mascot
1433.7747	1433.6796	-0.0951	-66	278	291	FVQAGSEVSALLGR					Mascot
1471.8552	1471.668	-0.1872	-127	179	191	TVLIMELINNIAK					Mascot
1471.8552	1471.668	-0.1872	-127	179	191	TVLIMELINNIAK	5	0			Mascot
1486.6665	1486.5713	-0.0952	-64	206	217	TREGNDLYMEMK					Mascot
1487.8501	1487.6564	-0.1937	-130	179	191	TVLIMELINNIAK			Oxidation (M)[5]		Mascot
1502.6614	1502.7509	0.0895	60	206	217	TREGNDLYMEMK			Oxidation (M)[9]		Mascot
1601.8104	1601.7039	-0.1065	-66	232	246	VALVYGQMNEPPGAR	80	100			Mascot
1601.8104	1601.7039	-0.1065	-66	232	246	VALVYGQMNEPPGAR					Mascot
1617.8053	1617.6959	-0.1094	-68	232	246	VALVYGQMNEPPGAR			Oxidation (M)[8]		Mascot
1740.9136	1740.7834	-0.1302	-75	247	261	MRVGLTALTMAEYIR			Oxidation (M)[1]		Mascot
1949.9967	1949.8654	-0.1313	-67	262	277	DVNEQDVLLFIDNIFR					Mascot
1949.9967	1949.8654	-0.1313	-67	262	277	DVNEQDVLLFIDNIFR	158	100			Mascot
2061.0322	2060.8909	-0.1413	-69	360	378	GIYPAVDPLDSTSTMLQPR					Mascot
2071.0852	2070.9404	-0.1448	-70	88	109	GMEVIDTGAALSVPVGGATLGR					Mascot
2071.0852	2070.9404	-0.1448	-70	88	109	GMEVIDTGAALSVPVGGATLGR	100	100			Mascot
2077.0271	2076.8875	-0.1396	-67	360	378	GIYPAVDPLDSTSTMLQPR			Oxidation (M)[15]		Mascot
2087.0801	2086.9346	-0.1455	-70	88	109	GMEVIDTGAALSVPVGGATLGR			Oxidation (M)[2]		Mascot
2171.1077	2170.8914	-0.2163	-100	400	418	ELQDIIAILGLDELSEEDR					Mascot
2282.0791	2281.9192	-0.1599	-70	292	312	MPSAVGYQPTLSTEMGSLQER					Mascot
2298.074	2297.9141	-0.1599	-70	292	312	MPSAVGYQPTLSTEMGSLQER			Oxidation (M)[1]		Mascot
2314.0688	2313.9443	-0.1245	-54	292	312	MPSAVGYQPTLSTEMGSLQER			Oxidation (M)[1,15]		Mascot
2386.178	2386.009	-0.169	-71	53	73	DTVGQQINVTCEVQQLLGNNR	144	100	Carbamidomethyl (C)[11]		Mascot
2386.178	2386.009	-0.169	-71	53	73	DTVGQQINVTCEVQQLLGNNR			Carbamidomethyl (C)[11]		Mascot
2462.2661	2462.0957	-0.1704	-69	398	418	YKELQDIIAILGLDELSEEDR					Mascot
2599.3005	2599.1221	-0.1784	-69	51	73	GRDTVGQQINVTCEVQQLLGNNR			Carbamidomethyl (C)[13]		Mascot
2711.446	2711.252	-0.194	-72	400	423	ELQDIIAILGLDELSEEDRLTVAR					Mascot
3843.9287	3843.6577	-0.271	-71	318	354	EGSITSIQAVYVPADDLTDPAPATTFAHLDATTVLSR					Mascot

Rank	Protein Name	Accession No	Protein MW	Protein PI	Pep. Count	Protein Score	Protein Score C. I. %	Total Ion Score	Total Ion C. I. %
5	ATP synthase subunit beta, chloroplastic OS=Pi sativum GN=atpB PE=3 SV=1	ATPB_PEA	53089.6	5.13	19	737	100	600	100

Peptide Information

Calc. Mass	Obsrv. Mass	± da	± ppm	Start Seq.	End Seq.	Sequence	Ion Score	C. I. %	Modification	Rank	Result Type
811.4421	811.3904	-0.0517	-64	128	134	TTSPIHR					Mascot
975.5621	975.5021	-0.06	-62	168	178	IGLFGGAGVGK					Mascot
1021.5676	1021.4698	-0.0978	-96	448	456	YVGLAETIR					Mascot
1045.604	1045.5377	-0.0663	-63	155	163	VVDLLAPYR					Mascot
1245.5126	1245.5527	0.0401	32	208	217	EGNDLYMEMK			Oxidation (M)[7]		Mascot
1328.6705	1328.5869	-0.0836	-63	192	205	AHGGVSVFGGVGER					Mascot
1431.6863	1431.594	-0.0923	-64	379	390	IVGEEHYETAQR					Mascot
1433.7747	1433.6796	-0.0951	-66	278	291	FVQAGSEVSALLGR	76	100			Mascot
1433.7747	1433.6796	-0.0951	-66	278	291	FVQAGSEVSALLGR					Mascot
1471.7614	1471.668	-0.0934	-63	249	261	VGLTALTMAEYFR					Mascot
1471.7614	1471.668	-0.0934	-63	249	261	VGLTALTMAEYFR	45	99.665			Mascot
1486.6665	1486.5713	-0.0952	-64	206	217	TREGNDLYMEMK					Mascot
1487.7562	1487.6564	-0.0998	-67	249	261	VGLTALTMAEYFR			Oxidation (M)[8]		Mascot
1502.6614	1502.7509	0.0895	60	206	217	TREGNDLYMEMK			Oxidation (M)[9]		Mascot
1601.6934	1601.7039	0.0105	7	208	220	EGNDLYMEMKESR					Mascot
1601.8104	1601.7039	-0.1065	-66	232	246	VALVYGQMNEPPGAR	80	100			Mascot
1617.6884	1617.6959	0.0075	5	208	220	EGNDLYMEMKESR			Oxidation (M)[7]		Mascot
1949.9967	1949.8654	-0.1313	-67	262	277	DVNEQDVLLFIDNIFR					Mascot
1949.9967	1949.8654	-0.1313	-67	262	277	DVNEQDVLLFIDNIFR	158	100			Mascot
1969.0388	1968.9038	-0.135	-69	110	127	IFNVLGEPIDNLGPVDTR					Mascot
2061.0322	2060.8909	-0.1413	-69	360	378	GIYPAVDPLDSTSTMLQPR					Mascot
2071.0852	2070.9404	-0.1448	-70	88	109	GMEVIDTGAALSVPVGGATLGR					Mascot
2071.0852	2070.9404	-0.1448	-70	88	109	GMEVIDTGAALSVPVGGATLGR	100	100			Mascot
2077.0271	2076.8875	-0.1396	-67	360	378	GIYPAVDPLDSTSTMLQPR			Oxidation (M)[15]		Mascot
2087.0801	2086.9346	-0.1455	-70	88	109	GMEVIDTGAALSVPVGGATLGR			Oxidation (M)[2]		Mascot
2282.0791	2281.9192	-0.1599	-70	292	312	MPSAVGYQPTLGTEMGTLQER			Oxidation (M)[1]		Mascot
2298.074	2297.9141	-0.1599	-70	292	312	MPSAVGYQPTLGTEMGTLQER			Oxidation (M)[1,15]		Mascot
2386.2144	2386.009	-0.2054	-86	53	73	DTVGKQINVTCEVQQLLGNNR	144	100	Carbamidomethyl (C)[11]		Mascot
2386.2144	2386.009	-0.2054	-86	53	73	DTVGKQINVTCEVQQLLGNNR			Carbamidomethyl (C)[11]		Mascot
3843.9287	3843.6577	-0.271	-71	318	354	EGSITSIQAVYVPADDLTDPAPATTFAHLDATTVLSR					Mascot

Rank	Protein Name	Accession No	Protein MW	Protein PI	Pep. Count	Protein Score	Protein Score C. I. %	Total Ion Score	Total Ion C. I. %
6	ATP synthase subunit beta, chloroplastic OS=Lo japonicus GN=atpB PE=3 SV=1	ATPB_LOTJA	53779.2	5.37	23	701	100	514	100

Peptide Information

Calc. Mass	Obsrv. Mass	± da	± ppm	Start Seq.	End Seq.	Sequence	Ion Score	C. I. %	Modification	Rank	Result Type
811.4421	811.3904	-0.0517	-64	128	134	TTSPIHR					Mascot
975.5621	975.5021	-0.06	-62	168	178	IGLFGGAGVGK					Mascot
1021.5676	1021.4698	-0.0978	-96	448	456	YVGLAETIR					Mascot
1045.604	1045.5377	-0.0663	-63	155	163	VVDLLAPYR					Mascot
1190.6416	1190.5685	-0.0731	-61	135	145	SAPAFIQLDTK					Mascot
1245.5126	1245.5527	0.0401	32	208	217	EGNDLYMEMK			Oxidation (M)[7]		Mascot
1275.7129	1275.6504	-0.0625	-49	40	50	MPNIYNALIVK					Mascot
1275.7129	1275.6504	-0.0625	-49	40	50	MPNIYNALIVK	13	0			Mascot
1328.6705	1328.5869	-0.0836	-63	192	205	AHGGVSVFGGVGER					Mascot
1431.6863	1431.594	-0.0923	-64	379	390	IVGEEHYETAQR					Mascot
1433.7747	1433.6796	-0.0951	-66	278	291	FVQAGSEVSALLGR	76	100			Mascot
1433.7747	1433.6796	-0.0951	-66	278	291	FVQAGSEVSALLGR					Mascot
1471.7614	1471.668	-0.0934	-63	249	261	VGLTALTMAEYFR					Mascot
1471.7614	1471.668	-0.0934	-63	249	261	VGLTALTMAEYFR	45	99.665			Mascot
1486.6665	1486.5713	-0.0952	-64	206	217	TREGNDLYMEMK					Mascot
1487.7562	1487.6564	-0.0998	-67	249	261	VGLTALTMAEYFR			Oxidation (M)[8]		Mascot
1502.6614	1502.7509	0.0895	60	206	217	TREGNDLYMEMK			Oxidation (M)[9]		Mascot
1601.8104	1601.7039	-0.1065	-66	232	246	VALVYGQMNEPPGAR	80	100			Mascot
1601.8104	1601.7039	-0.1065	-66	232	246	VALVYGQMNEPPGAR					Mascot
1617.8053	1617.6959	-0.1094	-68	232	246	VALVYGQMNEPPGAR			Oxidation (M)[8]		Mascot
1949.9967	1949.8654	-0.1313	-67	262	277	DVNEQDVLLFIDNIFR					Mascot
1949.9967	1949.8654	-0.1313	-67	262	277	DVNEQDVLLFIDNIFR	158	100			Mascot
1969.0388	1968.9038	-0.135	-69	110	127	IFNVLGEPIDNLGPVDTR					Mascot
2061.0322	2060.8909	-0.1413	-69	360	378	GIYPAVDPLDSTSTMLQPR					Mascot
2077.0271	2076.8875	-0.1396	-67	360	378	GIYPAVDPLDSTSTMLQPR			Oxidation (M)[15]		Mascot
2171.1077	2170.8914	-0.2163	-100	400	418	ELQDIIAILGLDELSEEDR					Mascot
2282.0791	2281.9192	-0.1599	-70	292	312	MPSAVGYQPTLSTEMGSLQER					Mascot
2298.074	2297.9141	-0.1599	-70	292	312	MPSAVGYQPTLSTEMGSLQER			Oxidation (M)[1]		Mascot
2314.0688	2313.9443	-0.1245	-54	292	312	MPSAVGYQPTLSTEMGSLQER			Oxidation (M)[1,15]		Mascot
2386.178	2386.009	-0.169	-71	53	73	DTVGQQINVTCEVQQLLGNNR	144	100	Carbamidomethyl (C)[11]		Mascot
2386.178	2386.009	-0.169	-71	53	73	DTVGQQINVTCEVQQLLGNNR			Carbamidomethyl (C)[11]		Mascot
2462.2661	2462.0957	-0.1704	-69	398	418	YKELQDIIAILGLDELSEEDR					Mascot
2599.3005	2599.1221	-0.1784	-69	51	73	GRDTVGQQINVTCEVQQLLGNNR			Carbamidomethyl (C)[13]		Mascot
2711.446	2711.252	-0.194	-72	400	423	ELQDIIAILGLDELSEEDRLTVAR					Mascot
3843.9287	3843.6577	-0.271	-71	318	354	EGSITSIQAVYVPADDLTDPAPATTFAHLDATTVLSR					Mascot

Rank	Protein Name	Accession No	Protein MW	Protein PI	Pep. Count	Protein Score	Protein Score C. I. %	Total Ion Score	Total Ion C. I. %
7	ATP synthase subunit beta, chloroplastic OS=Mo indica GN=atpB PE=3 SV=1	ATPB_MORIN	53758.1	5.46	24	700	100	502	100

Peptide Information

Calc. Mass	Obsrv. Mass	± da	± ppm	Start Seq.	End Seq.	Sequence	Ion Score	C. I. %	Modification	Rank	Result Type
811.4421	811.3904	-0.0517	-64	128	134	TTSPIHR					Mascot
975.5621	975.5021	-0.06	-62	168	178	IGLFGGAGVGK					Mascot
1021.5676	1021.4698	-0.0978	-96	448	456	YVGLAETIR					Mascot
1045.604	1045.5377	-0.0663	-63	155	163	VVDLLAPYR					Mascot
1190.6416	1190.5685	-0.0731	-61	135	145	SAPAFIQLDTK					Mascot
1245.5126	1245.5527	0.0401	32	208	217	EGNDLYMEMK			Oxidation (M)[7]		Mascot
1328.6705	1328.5869	-0.0836	-63	192	205	AHGGVSVFGGVGER					Mascot
1431.6863	1431.594	-0.0923	-64	379	390	IVGEEHYETAQR					Mascot
1433.7747	1433.6796	-0.0951	-66	278	291	FVQAGSEVSALLGR	76	100			Mascot
1433.7747	1433.6796	-0.0951	-66	278	291	FVQAGSEVSALLGR					Mascot
1471.7614	1471.668	-0.0934	-63	249	261	VGLTALTMAEYFR					Mascot
1471.7614	1471.668	-0.0934	-63	249	261	VGLTALTMAEYFR	45	99.665			Mascot
1486.6665	1486.5713	-0.0952	-64	206	217	TREGNDLYMEMK					Mascot
1487.7562	1487.6564	-0.0998	-67	249	261	VGLTALTMAEYFR			Oxidation (M)[8]		Mascot
1502.6614	1502.7509	0.0895	60	206	217	TREGNDLYMEMK			Oxidation (M)[9]		Mascot
1601.8104	1601.7039	-0.1065	-66	232	246	VALVYGQMNEPPGAR	80	100			Mascot
1601.8104	1601.7039	-0.1065	-66	232	246	VALVYGQMNEPPGAR					Mascot
1617.8053	1617.6959	-0.1094	-68	232	246	VALVYGQMNEPPGAR			Oxidation (M)[8]		Mascot
1735.0153	1734.8967	-0.1186	-68	23	39	IAQIIGPVLDVAFPPGK					Mascot
1949.9967	1949.8654	-0.1313	-67	262	277	DVNEQDVLLFIDNIFR					Mascot
1949.9967	1949.8654	-0.1313	-67	262	277	DVNEQDVLLFIDNIFR	158	100			Mascot
1969.0388	1968.9038	-0.135	-69	110	127	IFNVLGEPIDNLGPVDTR					Mascot
2061.0322	2060.8909	-0.1413	-69	360	378	GIYPAVDPLDSTSTMLQPR					Mascot
2077.0271	2076.8875	-0.1396	-67	360	378	GIYPAVDPLDSTSTMLQPR			Oxidation (M)[15]		Mascot
2171.1077	2170.8914	-0.2163	-100	400	418	ELQDIIAILGLDELSEEDR					Mascot
2282.0791	2281.9192	-0.1599	-70	292	312	MPSAVGYQPTLSTEMGSLQER					Mascot
2298.074	2297.9141	-0.1599	-70	292	312	MPSAVGYQPTLSTEMGSLQER			Oxidation (M)[1]		Mascot
2314.0688	2313.9443	-0.1245	-54	292	312	MPSAVGYQPTLSTEMGSLQER			Oxidation (M)[1,15]		Mascot
2386.178	2386.009	-0.169	-71	53	73	DTVGQQINVTCEVQQLLGNNR	144	100	Carbamidomethyl (C)[11]		Mascot
2386.178	2386.009	-0.169	-71	53	73	DTVGQQINVTCEVQQLLGNNR			Carbamidomethyl (C)[11]		Mascot
2462.2661	2462.0957	-0.1704	-69	398	418	YKELQDIIAILGLDELSEEDR					Mascot
2599.3005	2599.1221	-0.1784	-69	51	73	GRDTVGQQINVTCEVQQLLGNNR			Carbamidomethyl (C)[13]		Mascot
2711.446	2711.252	-0.194	-72	400	423	ELQDIIAILGLDELSEEDRLTVAR					Mascot
2876.4927	2876.2935	-0.1992	-69	460	486	LILSGELDGLPEQAFYLVGNIDEATAK					Mascot
3843.9287	3843.6577	-0.271	-71	318	354	EGSITSIQAVYVPADDLTDPAPATTFAHLDATTVLSR					Mascot

Rank	Protein Name	Accession No	Protein MW	Protein PI	Pep. Count	Protein Score	Protein Score C. I. %	Total Ion Score	Total Ion C. I. %
8	ATP synthase subunit beta, chloroplastic OS=Galbulimima belgraveana GN=atpB PE=3 SV=1	ATPB_GALBE	53610.9	5.44	23	686	100	502	100

Peptide Information

Calc. Mass	Obsrv. Mass	± da	± ppm	Start Seq.	End Seq.	Sequence	Ion Score	C. I. %	Modification	Rank	Result Type
811.4421	811.3904	-0.0517	-64	128	134	TTSPIHR					Mascot
910.4741	910.3899	-0.0842	-92	393	399	QTSQRYK					Mascot
975.5621	975.5021	-0.06	-62	168	178	IGLFGGAGVGK					Mascot
1021.5676	1021.4698	-0.0978	-96	448	456	YVGLAETIR					Mascot
1045.604	1045.5377	-0.0663	-63	155	163	VVDLLAPYR					Mascot
1190.6416	1190.5685	-0.0731	-61	135	145	SAPAFIQLDTK					Mascot
1245.5126	1245.5527	0.0401	32	208	217	EGNDLYMEMK			Oxidation (M)[7]		Mascot
1328.6705	1328.5869	-0.0836	-63	192	205	AHGGVSVFGGVGER					Mascot
1431.6863	1431.594	-0.0923	-64	379	390	IVGEEHYETAQR					Mascot
1433.7747	1433.6796	-0.0951	-66	278	291	FVQAGSEVSALLGR	76	100			Mascot
1433.7747	1433.6796	-0.0951	-66	278	291	FVQAGSEVSALLGR					Mascot
1471.7614	1471.668	-0.0934	-63	249	261	VGLTALTMAEYFR					Mascot
1471.7614	1471.668	-0.0934	-63	249	261	VGLTALTMAEYFR	45	99.665			Mascot
1486.6665	1486.5713	-0.0952	-64	206	217	TREGNDLYMEMK					Mascot
1487.7562	1487.6564	-0.0998	-67	249	261	VGLTALTMAEYFR			Oxidation (M)[8]		Mascot
1502.6614	1502.7509	0.0895	60	206	217	TREGNDLYMEMK			Oxidation (M)[9]		Mascot
1601.8104	1601.7039	-0.1065	-66	232	246	VALVYGQMNEPPGAR	80	100			Mascot
1601.8104	1601.7039	-0.1065	-66	232	246	VALVYGQMNEPPGAR					Mascot
1617.8053	1617.6959	-0.1094	-68	232	246	VALVYGQMNEPPGAR			Oxidation (M)[8]		Mascot
1735.0153	1734.8967	-0.1186	-68	23	39	IAQIIGPVLDVAFPPGK					Mascot
1949.9967	1949.8654	-0.1313	-67	262	277	DVNEQDVLLFIDNIFR					Mascot
1949.9967	1949.8654	-0.1313	-67	262	277	DVNEQDVLLFIDNIFR	158	100			Mascot
2061.0322	2060.8909	-0.1413	-69	360	378	GIYPAVDPLDSTSTMLQPR					Mascot
2077.0271	2076.8875	-0.1396	-67	360	378	GIYPAVDPLDSTSTMLQPR			Oxidation (M)[15]		Mascot
2171.1077	2170.8914	-0.2163	-100	400	418	ELQDIIAILGLDELSEEDR					Mascot
2282.0791	2281.9192	-0.1599	-70	292	312	MPSAVGYQPTLSTEMGSLQER					Mascot
2298.074	2297.9141	-0.1599	-70	292	312	MPSAVGYQPTLSTEMGSLQER			Oxidation (M)[1]		Mascot
2314.0688	2313.9443	-0.1245	-54	292	312	MPSAVGYQPTLSTEMGSLQER			Oxidation (M)[1,15]		Mascot
2386.178	2386.009	-0.169	-71	53	73	DTVGQQINVTCEVQQLLGNNR	144	100	Carbamidomethyl (C)[11]		Mascot
2386.178	2386.009	-0.169	-71	53	73	DTVGQQINVTCEVQQLLGNNR			Carbamidomethyl (C)[11]		Mascot
2462.2661	2462.0957	-0.1704	-69	398	418	YKELQDIIAILGLDELSEEDR					Mascot
2599.3005	2599.1221	-0.1784	-69	51	73	GRDTVGQQINVTCEVQQLLGNNR			Carbamidomethyl (C)[13]		Mascot
2711.446	2711.252	-0.194	-72	400	423	ELQDIIAILGLDELSEEDRLTVAR					Mascot
3843.9287	3843.6577	-0.271	-71	318	354	EGSITSIQAVYVPADDLTDPAPATTFAHLDATTVLSR					Mascot

Rank	Protein Name	Accession No	Protein MW	Protein PI	Pep. Count	Protein Score	Protein Score C. I. %	Total Ion Score	Total Ion C. I. %
9	ATP synthase subunit beta, chloroplastic OS=Sa zalacca GN=atpB PE=3 SV=1	ATPB_SALZA	53753	5.16	22	676	100	502	100

Peptide Information

Calc. Mass	Obsrv. Mass	± da	± ppm	Start Seq.	End Seq.	Sequence	Ion Score	C. I. %	Modification	Rank	Result Type
811.4421	811.3904	-0.0517	-64	128	134	TTSPIHR					Mascot
910.4741	910.3899	-0.0842	-92	393	399	QTSQRYK					Mascot
975.5621	975.5021	-0.06	-62	168	178	IGLFGGAGVGK					Mascot
1021.5676	1021.4698	-0.0978	-96	448	456	YVGLAETIR					Mascot
1045.604	1045.5377	-0.0663	-63	155	163	VVDLLAPYR					Mascot
1190.6416	1190.5685	-0.0731	-61	135	145	SAPAFIQLDTK					Mascot
1245.5126	1245.5527	0.0401	32	208	217	EGNDLYMEMK			Oxidation (M)[7]		Mascot
1328.6705	1328.5869	-0.0836	-63	192	205	AHGGVSVFGGVGER					Mascot
1431.6863	1431.594	-0.0923	-64	379	390	IVGEEHYETAQR					Mascot
1433.7747	1433.6796	-0.0951	-66	278	291	FVQAGSEVSALLGR	76	100			Mascot
1433.7747	1433.6796	-0.0951	-66	278	291	FVQAGSEVSALLGR					Mascot
1471.7614	1471.668	-0.0934	-63	249	261	VGLTALTMAEYFR					Mascot
1471.7614	1471.668	-0.0934	-63	249	261	VGLTALTMAEYFR	45	99.665			Mascot
1486.6665	1486.5713	-0.0952	-64	206	217	TREGNDLYMEMK					Mascot
1487.7562	1487.6564	-0.0998	-67	249	261	VGLTALTMAEYFR			Oxidation (M)[8]		Mascot
1502.6614	1502.7509	0.0895	60	206	217	TREGNDLYMEMK			Oxidation (M)[9]		Mascot
1601.8104	1601.7039	-0.1065	-66	232	246	VALVYGQMNEPPGAR	80	100			Mascot
1601.8104	1601.7039	-0.1065	-66	232	246	VALVYGQMNEPPGAR					Mascot
1617.8053	1617.6959	-0.1094	-68	232	246	VALVYGQMNEPPGAR			Oxidation (M)[8]		Mascot
1949.9967	1949.8654	-0.1313	-67	262	277	DVNEQDVLLFIDNIFR					Mascot
1949.9967	1949.8654	-0.1313	-67	262	277	DVNEQDVLLFIDNIFR	158	100			Mascot
2061.0322	2060.8909	-0.1413	-69	360	378	GIYPAVDPLDSTSTMLQPR					Mascot
2077.0271	2076.8875	-0.1396	-67	360	378	GIYPAVDPLDSTSTMLQPR			Oxidation (M)[15]		Mascot
2171.1077	2170.8914	-0.2163	-100	400	418	ELQDIIAILGLDELSEEDR					Mascot
2282.0791	2281.9192	-0.1599	-70	292	312	MPSAVGYQPTLSTEMGSLQER					Mascot
2298.074	2297.9141	-0.1599	-70	292	312	MPSAVGYQPTLSTEMGSLQER			Oxidation (M)[1]		Mascot
2314.0688	2313.9443	-0.1245	-54	292	312	MPSAVGYQPTLSTEMGSLQER			Oxidation (M)[1,15]		Mascot
2386.178	2386.009	-0.169	-71	53	73	DTVGQQINVTCEVQQLLGNNR	144	100	Carbamidomethyl (C)[11]		Mascot
2386.178	2386.009	-0.169	-71	53	73	DTVGQQINVTCEVQQLLGNNR			Carbamidomethyl (C)[11]		Mascot
2462.2661	2462.0957	-0.1704	-69	398	418	YKELQDIIAILGLDELSEEDR					Mascot
2711.446	2711.252	-0.194	-72	400	423	ELQDIIAILGLDELSEEDRLTVAR					Mascot
3842.9275	3842.6252	-0.3023	-79	40	73	MPNIYNALVVEGRDTVGQQINVTCEVQQLLGNNR			Carbamidomethyl (C)[24]		Mascot
3843.9287	3843.6577	-0.271	-71	318	354	EGSITSIQAVYVPADDLTDPAPATTFAHLDATTVLSR					Mascot

Rank	Protein Name	Accession No	Protein MW	Protein PI	Pep. Count	Protein Score	Protein Score C. I. %	Total Ion Score	Total Ion C. I. %
10	ATP synthase subunit beta, chloroplastic OS=Ca papaya GN=atpB PE=3 SV=1	ATPB_CARP	53609	5.46	22	675	100	502	100

Peptide Information

Calc. Mass	Obsrv. Mass	± da	± ppm	Start Seq.	End Seq.	Sequence	Ion Score	C. I. %	Modification	Rank	Result Type
975.5621	975.5021	-0.06	-62	168	178	IGLFGGAGVGK					Mascot
1021.5676	1021.4698	-0.0978	-96	448	456	YVGLAETIR					Mascot
1045.604	1045.5377	-0.0663	-63	155	163	VVDLLAPYR					Mascot
1190.6416	1190.5685	-0.0731	-61	135	145	SAPAFIQLDTK					Mascot
1245.5126	1245.5527	0.0401	32	208	217	EGNDLYMEMK			Oxidation (M)[7]		Mascot
1328.6705	1328.5869	-0.0836	-63	192	205	AHGGVSVFGGVGER					Mascot
1431.6863	1431.594	-0.0923	-64	379	390	IVGEEHYETAQR					Mascot
1433.7747	1433.6796	-0.0951	-66	278	291	FVQAGSEVSALLGR	76	100			Mascot
1433.7747	1433.6796	-0.0951	-66	278	291	FVQAGSEVSALLGR					Mascot
1471.7614	1471.668	-0.0934	-63	249	261	VGLTALTMAEYFR					Mascot
1471.7614	1471.668	-0.0934	-63	249	261	VGLTALTMAEYFR	45	99.683			Mascot
1486.6665	1486.5713	-0.0952	-64	206	217	TREGNDLYMEMK					Mascot
1487.7562	1487.6564	-0.0998	-67	249	261	VGLTALTMAEYFR			Oxidation (M)[8]		Mascot
1502.6614	1502.7509	0.0895	60	206	217	TREGNDLYMEMK			Oxidation (M)[9]		Mascot
1601.8104	1601.7039	-0.1065	-66	232	246	VALVYGQMNEPPGAR	80	100			Mascot
1601.8104	1601.7039	-0.1065	-66	232	246	VALVYGQMNEPPGAR					Mascot
1617.8053	1617.6959	-0.1094	-68	232	246	VALVYGQMNEPPGAR			Oxidation (M)[8]		Mascot
1735.0153	1734.8967	-0.1186	-68	23	39	IAQIIGPVLDVAFPPGK					Mascot
1949.9967	1949.8654	-0.1313	-67	262	277	DVNEQDVLLFIDNIFR					Mascot
1949.9967	1949.8654	-0.1313	-67	262	277	DVNEQDVLLFIDNIFR	158	100			Mascot
2061.0322	2060.8909	-0.1413	-69	360	378	GIYPAVDPLDSTSTMLQPR					Mascot
2077.0271	2076.8875	-0.1396	-67	360	378	GIYPAVDPLDSTSTMLQPR			Oxidation (M)[15]		Mascot
2171.1077	2170.8914	-0.2163	-100	400	418	ELQDIIAILGLDELSEEDR					Mascot
2282.0791	2281.9192	-0.1599	-70	292	312	MPSAVGYQPTLSTEMGSLQER					Mascot
2298.074	2297.9141	-0.1599	-70	292	312	MPSAVGYQPTLSTEMGSLQER			Oxidation (M)[1]		Mascot
2314.0688	2313.9443	-0.1245	-54	292	312	MPSAVGYQPTLSTEMGSLQER			Oxidation (M)[1,15]		Mascot
2386.178	2386.009	-0.169	-71	53	73	DTVGQQINVTCEVQQLLGNNR	144	100	Carbamidomethyl (C)[11]		Mascot
2386.178	2386.009	-0.169	-71	53	73	DTVGQQINVTCEVQQLLGNNR			Carbamidomethyl (C)[11]		Mascot
2462.2661	2462.0957	-0.1704	-69	398	418	YKELQDIIAILGLDELSEEDR					Mascot
2599.3005	2599.1221	-0.1784	-69	51	73	GRDTVGQQINVTCEVQQLLGNNR			Carbamidomethyl (C)[13]		Mascot
2711.446	2711.252	-0.194	-72	400	423	ELQDIIAILGLDELSEEDRLTVAR					Mascot
2876.4927	2876.2935	-0.1992	-69	460	486	LILSGELDGLPEQAFYLVGNIDEATAK					Mascot
3843.9287	3843.6577	-0.271	-71	318	354	EGSITSIQAVYVPADDLTDPAPATTFAHLDATTVLSR					Mascot

Gel Idx/Pos	47/B20	Instr./Gel Origin	BA2060/131017A	Process Sta	Analysis Succeeded
Plate [#] Name	[1] 31496	Instrument Sample Name		Spectra	8

Rank	Protein Name	Accession No	Protein MW	Protein PI	Pep. Count	Protein Score	Protein Score C. I. %	Total Ion Score	Total Ion C. I. %
1	ATP synthase subunit beta, chloroplastic OS=Gl max GN=atpB PE=3 SV=1	ATPB_SOYBI	53721.1	5.29	30	984	100	689	100

Peptide Information

Calc. Mass	Obsrv. Mass	± da	± ppm	Start Seq.	End Seq.	Sequence	Ion Score	C. I. %	Modification	Rank	Result Type
811.4421	811.4009	-0.0412	-51	128	134	TTSPIHR					Mascot
975.5621	975.5026	-0.0595	-61	168	178	IGLFGGAGVGK					Mascot
991.5822	991.527	-0.0552	-56	146	154	LAIFETGIK					Mascot
1045.604	1045.5453	-0.0587	-56	155	163	VVDLLAPYR					Mascot
1190.6416	1190.5757	-0.0659	-55	135	145	SAPAFIQLDTK					Mascot
1201.7052	1201.6393	-0.0659	-55	155	164	VVDLLAPYRR					Mascot
1229.5177	1229.4539	-0.0638	-52	208	217	EGNDLYMEMK					Mascot
1236.6075	1236.5376	-0.0699	-57	76	87	AVAMSATEGLMR					Mascot
1245.5126	1245.5599	0.0473	38	208	217	EGNDLYMEMK			Oxidation (M)[7]		Mascot
1252.6024	1252.5287	-0.0737	-59	76	87	AVAMSATEGLMR			Oxidation (M)[4]		Mascot
1275.7129	1275.6541	-0.0588	-46	40	50	MPNIYNALIVK					Mascot
1328.6705	1328.5929	-0.0776	-58	192	205	AHGGVSVFGGVGER					Mascot
1431.6863	1431.6031	-0.0832	-58	379	390	IVGEEHYETAQR					Mascot
1433.7747	1433.6885	-0.0862	-60	278	291	FVQAGSEVSALLGR	51	99.907			Mascot
1433.7747	1433.6885	-0.0862	-60	278	291	FVQAGSEVSALLGR					Mascot
1471.7614	1471.6768	-0.0846	-57	249	261	VGLTALTMAEYFR					Mascot
1471.7614	1471.6768	-0.0846	-57	249	261	VGLTALTMAEYFR	53	99.94			Mascot
1486.6665	1486.5858	-0.0807	-54	206	217	TREGNDLYMEMK					Mascot
1487.7562	1487.6637	-0.0925	-62	249	261	VGLTALTMAEYFR			Oxidation (M)[8]		Mascot
1502.6614	1502.7535	0.0921	61	206	217	TREGNDLYMEMK			Oxidation (M)[9]		Mascot
1517.7441	1517.6451	-0.099	-65	218	231	ESGVINEQNIAESK					Mascot
1542.8009	1542.7048	-0.0961	-62	3	17	INPTTSGPEVSALEK					Mascot
1601.8104	1601.7146	-0.0958	-60	232	246	VALVYGQMNEPPGAR	72	100			Mascot
1601.8104	1601.7146	-0.0958	-60	232	246	VALVYGQMNEPPGAR					Mascot
1617.8053	1617.7068	-0.0985	-61	232	246	VALVYGQMNEPPGAR			Oxidation (M)[8]		Mascot
1735.0153	1734.9052	-0.1101	-63	23	39	IAQIIGPVLDVAFPPGK					Mascot
1949.9967	1949.8717	-0.125	-64	262	277	DVNEQDVLLFIDNIFR					Mascot
1949.9967	1949.8717	-0.125	-64	262	277	DVNEQDVLLFIDNIFR	152	100			Mascot
1969.0388	1968.9137	-0.1251	-64	110	127	IFNVLGEPIDNLGPVDTR					Mascot
2061.0322	2060.9009	-0.1313	-64	360	378	GIYPAVDPLDSTSTMLQPR					Mascot
2071.0852	2070.9541	-0.1311	-63	88	109	GMEVIDTGAALSVPVGGATLGR	88	100			Mascot
2071.0852	2070.9541	-0.1311	-63	88	109	GMEVIDTGAALSVPVGGATLGR					Mascot
2077.0271	2076.8945	-0.1326	-64	360	378	GIYPAVDPLDSTSTMLQPR			Oxidation (M)[15]		Mascot
2087.0801	2086.9463	-0.1338	-64	88	109	GMEVIDTGAALSVPVGGATLGR			Oxidation (M)[2]		Mascot
2171.1077	2170.9673	-0.1404	-65	400	418	ELQDIIAILGLDELSEEDR					Mascot
2282.0791	2281.9272	-0.1519	-67	292	312	MPSAVGYQPTLSTEMGSLQER					Mascot
2282.0791	2281.9272	-0.1519	-67	292	312	MPSAVGYQPTLSTEMGSLQER	125	100			Mascot
2298.074	2297.9253	-0.1487	-65	292	312	MPSAVGYQPTLSTEMGSLQER			Oxidation (M)[1]		Mascot
2314.0688	2313.9287	-0.1401	-61	292	312	MPSAVGYQPTLSTEMGSLQER			Oxidation (M)[1,15]		Mascot
2386.178	2386.022	-0.156	-65	53	73	DTVGQQINVTCEVQQLLGNNR	152	100	Carbamidomethyl (C)[11]		Mascot
2386.178	2386.022	-0.156	-65	53	73	DTVGQQINVTCEVQQLLGNNR			Carbamidomethyl (C)[11]		Mascot
2462.2661	2462.1001	-0.166	-67	398	418	YKELQDIIAILGLDELSEEDR					Mascot
2599.3005	2599.1211	-0.1794	-69	51	73	GRDTVGQQINVTCEVQQLLGNNR			Carbamidomethyl (C)[13]		Mascot
2711.446	2711.2666	-0.1794	-66	400	423	ELQDIIAILGLDELSEEDRLTVAR					Mascot
2876.4927	2876.3005	-0.1922	-67	460	486	LILSGELDGLPEQAFYLVGNIDEATAK					Mascot
3843.9287	3843.6604	-0.2683	-70	318	354	EGSITSIQAVYVPADDLTDPAPATTFAHLDATTVLSR					Mascot

Rank	Protein Name	Accession No	Protein MW	Protein PI	Pep. Count	Protein Score	Protein Score C. I. %	Total Ion Score	Total Ion C. I. %
2	ATP synthase subunit beta, chloroplastic OS=Ot siberica GN=atpB PE=3 SV=1	ATPB_OTHSI	53309.8	5.22	24	896	100	689	100

Peptide Information

Calc. Mass	Obsrv. Mass	± da	± ppm	Start Seq.	End Seq.	Sequence	Ion Score	C. I. %	Modification	Rank	Result Type
811.4421	811.4009	-0.0412	-51	128	134	TTSPIHR					Mascot
975.5621	975.5026	-0.0595	-61	168	178	IGLFGGAGVGK					Mascot
1021.5676	1021.4725	-0.0951	-93	448	456	YVGLAETIR					Mascot
1045.604	1045.5453	-0.0587	-56	155	163	VVDLLAPYR					Mascot
1190.6416	1190.5757	-0.0659	-55	135	145	SAPAFIQLDTK					Mascot
1201.7052	1201.6393	-0.0659	-55	155	164	VVDLLAPYRR					Mascot
1229.5177	1229.4539	-0.0638	-52	208	217	EGNDLYMEMK					Mascot
1245.5126	1245.5599	0.0473	38	208	217	EGNDLYMEMK			Oxidation (M)[7]		Mascot
1328.6705	1328.5929	-0.0776	-58	192	205	AHGGVSVFGGVGER					Mascot
1431.6863	1431.6031	-0.0832	-58	379	390	IVGEEHYETAQR					Mascot
1433.7747	1433.6885	-0.0862	-60	278	291	FVQAGSEVSALLGR	51	99.907			Mascot
1433.7747	1433.6885	-0.0862	-60	278	291	FVQAGSEVSALLGR					Mascot
1471.7614	1471.6768	-0.0846	-57	249	261	VGLTALTMAEYFR					Mascot
1471.7614	1471.6768	-0.0846	-57	249	261	VGLTALTMAEYFR	53	99.94			Mascot
1486.6665	1486.5858	-0.0807	-54	206	217	TREGNDLYMEMK					Mascot
1487.7562	1487.6637	-0.0925	-62	249	261	VGLTALTMAEYFR			Oxidation (M)[8]		Mascot
1502.6614	1502.7535	0.0921	61	206	217	TREGNDLYMEMK			Oxidation (M)[9]		Mascot
1517.7805	1517.6451	-0.1354	-89	218	231	ESGVINEKNIAESK					Mascot
1601.8104	1601.7146	-0.0958	-60	232	246	VALVYGQMNEPPGAR	72	100			Mascot
1601.8104	1601.7146	-0.0958	-60	232	246	VALVYGQMNEPPGAR					Mascot
1617.8053	1617.7068	-0.0985	-61	232	246	VALVYGQMNEPPGAR			Oxidation (M)[8]		Mascot
1949.9967	1949.8717	-0.125	-64	262	277	DVNEQDVLLFIDNIFR					Mascot
1949.9967	1949.8717	-0.125	-64	262	277	DVNEQDVLLFIDNIFR	152	100			Mascot
2061.0322	2060.9009	-0.1313	-64	360	378	GIYPAVDPLDSTSTMLQPR					Mascot
2071.0852	2070.9541	-0.1311	-63	88	109	GMEVIDTGAALSVPVGGATLGR	88	100			Mascot
2071.0852	2070.9541	-0.1311	-63	88	109	GMEVIDTGAALSVPVGGATLGR					Mascot
2077.0271	2076.8945	-0.1326	-64	360	378	GIYPAVDPLDSTSTMLQPR			Oxidation (M)[15]		Mascot
2087.0801	2086.9463	-0.1338	-64	88	109	GMEVIDTGAALSVPVGGATLGR			Oxidation (M)[2]		Mascot
2171.1077	2170.9673	-0.1404	-65	400	418	ELQDIIAILGLDELSEEDR					Mascot
2282.0791	2281.9272	-0.1519	-67	292	312	MPSAVGYQPTLSTEMGSLQER					Mascot
2282.0791	2281.9272	-0.1519	-67	292	312	MPSAVGYQPTLSTEMGSLQER	125	100			Mascot
2298.074	2297.9253	-0.1487	-65	292	312	MPSAVGYQPTLSTEMGSLQER			Oxidation (M)[1]		Mascot
2314.0688	2313.9287	-0.1401	-61	292	312	MPSAVGYQPTLSTEMGSLQER			Oxidation (M)[1,15]		Mascot
2386.178	2386.022	-0.156	-65	53	73	DTVGQQINVTCEVQQLLGNNR	152	100	Carbamidomethyl (C)[11]		Mascot
2386.178	2386.022	-0.156	-65	53	73	DTVGQQINVTCEVQQLLGNNR			Carbamidomethyl (C)[11]		Mascot
2462.2661	2462.1001	-0.166	-67	398	418	YKELQDIIAILGLDELSEEDR					Mascot
2599.3005	2599.1211	-0.1794	-69	51	73	GRDTVGQQINVTCEVQQLLGNNR			Carbamidomethyl (C)[13]		Mascot
2711.446	2711.2666	-0.1794	-66	400	423	ELQDIIAILGLDELSEEDRLTVAR					Mascot
3843.9287	3843.6604	-0.2683	-70	318	354	EGSITSIQAVYVPADDLTDPAPATTFAHLDATTVLSR					Mascot

Rank	Protein Name	Accession No	Protein MW	Protein PI	Pep. Count	Protein Score	Protein Score C. I. %	Total Ion Score	Total Ion C. I. %
3	ATP synthase subunit beta, chloroplastic OS=Lachenalia pusilla GN=atpB PE=3 SV=1	ATPB_LACPU	53030.6	5.15	23	883	100	689	100

Peptide Information

Calc. Mass	Obsrv. Mass	± da	± ppm	Start Seq.	End Seq.	Sequence	Ion Score	C. I. %	Modification	Rank	Result Type
811.4421	811.4009	-0.0412	-51	126	132	TTSPIHR					Mascot
975.5621	975.5026	-0.0595	-61	166	176	IGLFGGAGVGK					Mascot
1045.604	1045.5453	-0.0587	-56	153	161	VVDLLAPYR					Mascot
1190.6416	1190.5757	-0.0659	-55	133	143	SAPAFIQLDTK					Mascot
1201.7052	1201.6393	-0.0659	-55	153	162	VVDLLAPYRR					Mascot
1229.5177	1229.4539	-0.0638	-52	206	215	EGNDLYMEMK					Mascot
1245.5126	1245.5599	0.0473	38	206	215	EGNDLYMEMK			Oxidation (M)[7]		Mascot
1328.6705	1328.5929	-0.0776	-58	190	203	AHGGVSVFGGVGER					Mascot
1431.6863	1431.6031	-0.0832	-58	377	388	IVGEEHYETAQR					Mascot
1433.7747	1433.6885	-0.0862	-60	276	289	FVQAGSEVSALLGR	51	99.907			Mascot
1433.7747	1433.6885	-0.0862	-60	276	289	FVQAGSEVSALLGR					Mascot
1471.7614	1471.6768	-0.0846	-57	247	259	VGLTALTMAEYFR					Mascot
1471.7614	1471.6768	-0.0846	-57	247	259	VGLTALTMAEYFR	53	99.94			Mascot
1486.6665	1486.5858	-0.0807	-54	204	215	TREGNDLYMEMK					Mascot
1487.7562	1487.6637	-0.0925	-62	247	259	VGLTALTMAEYFR			Oxidation (M)[8]		Mascot
1502.6614	1502.7535	0.0921	61	204	215	TREGNDLYMEMK			Oxidation (M)[9]		Mascot
1517.7805	1517.6451	-0.1354	-89	216	229	ESGVINEKNIAESK					Mascot
1601.8104	1601.7146	-0.0958	-60	230	244	VALVYGQMNEPPGAR	72	100			Mascot
1601.8104	1601.7146	-0.0958	-60	230	244	VALVYGQMNEPPGAR					Mascot
1617.8053	1617.7068	-0.0985	-61	230	244	VALVYGQMNEPPGAR			Oxidation (M)[8]		Mascot
1949.9967	1949.8717	-0.125	-64	260	275	DVNEQDVLLFIDNIFR					Mascot
1949.9967	1949.8717	-0.125	-64	260	275	DVNEQDVLLFIDNIFR	152	100			Mascot
2061.0322	2060.9009	-0.1313	-64	358	376	GIYPAVDPLDSTSTMLQPR					Mascot
2071.0852	2070.9541	-0.1311	-63	86	107	GMEVIDTGAALSVPVGGATLGR	88	100			Mascot
2071.0852	2070.9541	-0.1311	-63	86	107	GMEVIDTGAALSVPVGGATLGR					Mascot
2077.0271	2076.8945	-0.1326	-64	358	376	GIYPAVDPLDSTSTMLQPR			Oxidation (M)[15]		Mascot
2087.0801	2086.9463	-0.1338	-64	86	107	GMEVIDTGAALSVPVGGATLGR			Oxidation (M)[2]		Mascot
2171.1077	2170.9673	-0.1404	-65	398	416	ELQDIIAILGLDELSEEDR					Mascot
2282.0791	2281.9272	-0.1519	-67	290	310	MPSAVGYQPTLSTEMGSLQER					Mascot
2282.0791	2281.9272	-0.1519	-67	290	310	MPSAVGYQPTLSTEMGSLQER	125	100			Mascot
2298.074	2297.9253	-0.1487	-65	290	310	MPSAVGYQPTLSTEMGSLQER			Oxidation (M)[1]		Mascot
2314.0688	2313.9287	-0.1401	-61	290	310	MPSAVGYQPTLSTEMGSLQER			Oxidation (M)[1,15]		Mascot
2386.178	2386.022	-0.156	-65	51	71	DTVGQQINVTCEVQQLLGNNR	152	100	Carbamidomethyl (C)[11]		Mascot
2386.178	2386.022	-0.156	-65	51	71	DTVGQQINVTCEVQQLLGNNR			Carbamidomethyl (C)[11]		Mascot
2462.2661	2462.1001	-0.166	-67	396	416	YKELQDIIAILGLDELSEEDR					Mascot
2599.3005	2599.1211	-0.1794	-69	49	71	GRDTVGQQINVTCEVQQLLGNNR			Carbamidomethyl (C)[13]		Mascot
2711.446	2711.2666	-0.1794	-66	398	421	ELQDIIAILGLDELSEEDRLTVAR					Mascot
3843.9287	3843.6604	-0.2683	-70	316	352	EGSITSIQAVYVPADDLTDPAPATTFAHLDATTVLSR					Mascot

Rank	Protein Name	Accession No	Protein MW	Protein PI	Pep. Count	Protein Score	Protein Score C. I. %	Total Ion Score	Total Ion C. I. %
4	ATP synthase subunit beta, chloroplastic OS=Eu montana GN=atpB PE=3 SV=1	ATPB_EUCM	53317.8	5.16	23	840	100	647	100

Peptide Information

Calc. Mass	Obsrv. Mass	± da	± ppm	Start Seq.	End Seq.	Sequence	Ion Score	C. I. %	Modification	Rank	Result Type
811.4421	811.4009	-0.0412	-51	128	134	TTSPIHR					Mascot
975.5621	975.5026	-0.0595	-61	168	178	IGLFGGAGVGK					Mascot
1045.604	1045.5453	-0.0587	-56	155	163	VVDLLAPYR					Mascot
1190.6416	1190.5757	-0.0659	-55	135	145	SAPAFIQLDTK					Mascot
1201.7052	1201.6393	-0.0659	-55	155	164	VVDLLAPYRR					Mascot
1229.5177	1229.4539	-0.0638	-52	208	217	EGNDLYMEMK					Mascot
1245.5126	1245.5599	0.0473	38	208	217	EGNDLYMEMK			Oxidation (M)[7]		Mascot
1328.6705	1328.5929	-0.0776	-58	192	205	AHGGVSVFGGVGER					Mascot
1431.6863	1431.6031	-0.0832	-58	379	390	IVGEEHYETAQR					Mascot
1433.7747	1433.6885	-0.0862	-60	278	291	FVQAGSEVSALLGR	51	99.907			Mascot
1433.7747	1433.6885	-0.0862	-60	278	291	FVQAGSEVSALLGR					Mascot
1471.8552	1471.6768	-0.1784	-121	179	191	TVLIMELINNIAK					Mascot
1471.8552	1471.6768	-0.1784	-121	179	191	TVLIMELINNIAK	9	0			Mascot
1486.6665	1486.5858	-0.0807	-54	206	217	TREGNDLYMEMK					Mascot
1487.8501	1487.6637	-0.1864	-125	179	191	TVLIMELINNIAK			Oxidation (M)[5]		Mascot
1502.6614	1502.7535	0.0921	61	206	217	TREGNDLYMEMK			Oxidation (M)[9]		Mascot
1517.7805	1517.6451	-0.1354	-89	218	231	ESGVINEKNIAESK					Mascot
1601.8104	1601.7146	-0.0958	-60	232	246	VALVYGQMNEPPGAR	72	100			Mascot
1601.8104	1601.7146	-0.0958	-60	232	246	VALVYGQMNEPPGAR					Mascot
1617.8053	1617.7068	-0.0985	-61	232	246	VALVYGQMNEPPGAR			Oxidation (M)[8]		Mascot
1949.9967	1949.8717	-0.125	-64	262	277	DVNEQDVLLFIDNIFR					Mascot
1949.9967	1949.8717	-0.125	-64	262	277	DVNEQDVLLFIDNIFR	152	100			Mascot
2061.0322	2060.9009	-0.1313	-64	360	378	GIYPAVDPLDSTSTMLQPR					Mascot
2071.0852	2070.9541	-0.1311	-63	88	109	GMEVIDTGAALSVPVGGATLGR	88	100			Mascot
2071.0852	2070.9541	-0.1311	-63	88	109	GMEVIDTGAALSVPVGGATLGR					Mascot
2077.0271	2076.8945	-0.1326	-64	360	378	GIYPAVDPLDSTSTMLQPR			Oxidation (M)[15]		Mascot
2087.0801	2086.9463	-0.1338	-64	88	109	GMEVIDTGAALSVPVGGATLGR			Oxidation (M)[2]		Mascot
2171.1077	2170.9673	-0.1404	-65	400	418	ELQDIIAILGLDELSEEDR					Mascot
2282.0791	2281.9272	-0.1519	-67	292	312	MPSAVGYQPTLSTEMGSLQER					Mascot
2282.0791	2281.9272	-0.1519	-67	292	312	MPSAVGYQPTLSTEMGSLQER	125	100			Mascot
2298.074	2297.9253	-0.1487	-65	292	312	MPSAVGYQPTLSTEMGSLQER			Oxidation (M)[1]		Mascot
2314.0688	2313.9287	-0.1401	-61	292	312	MPSAVGYQPTLSTEMGSLQER			Oxidation (M)[1,15]		Mascot
2386.178	2386.022	-0.156	-65	53	73	DTVGQQINVTCEVQQLLGNNR	152	100	Carbamidomethyl (C)[11]		Mascot
2386.178	2386.022	-0.156	-65	53	73	DTVGQQINVTCEVQQLLGNNR			Carbamidomethyl (C)[11]		Mascot
2462.2661	2462.1001	-0.166	-67	398	418	YKELQDIIAILGLDELSEEDR					Mascot
2599.3005	2599.1211	-0.1794	-69	51	73	GRDTVGQQINVTCEVQQLLGNNR			Carbamidomethyl (C)[13]		Mascot
2711.446	2711.2666	-0.1794	-66	400	423	ELQDIIAILGLDELSEEDRLTVAR					Mascot
3843.9287	3843.6604	-0.2683	-70	318	354	EGSITSIQAVYVPADDLTDPAPATTFAHLDATTVLSR					Mascot

Rank	Protein Name	Accession No	Protein MW	Protein PI	Pep. Count	Protein Score	Protein Score C. I. %	Total Ion Score	Total Ion C. I. %
5	ATP synthase subunit beta, chloroplastic OS=Mo indica GN=atpB PE=3 SV=1	ATPB_MORIN	53758.1	5.46	26	830	100	602	100

Peptide Information

Calc. Mass	Obsrv. Mass	± da	± ppm	Start Seq.	End Seq.	Sequence	Ion Score	C. I. %	Modification	Rank	Result Type
811.4421	811.4009	-0.0412	-51	128	134	TTSPIHR					Mascot
975.5621	975.5026	-0.0595	-61	168	178	IGLFGGAGVGK					Mascot
1021.5676	1021.4725	-0.0951	-93	448	456	YVGLAETIR					Mascot
1045.604	1045.5453	-0.0587	-56	155	163	VVDLLAPYR					Mascot
1190.6416	1190.5757	-0.0659	-55	135	145	SAPAFIQLDTK					Mascot
1201.7052	1201.6393	-0.0659	-55	155	164	VVDLLAPYRR					Mascot
1229.5177	1229.4539	-0.0638	-52	208	217	EGNDLYMEMK					Mascot
1245.5126	1245.5599	0.0473	38	208	217	EGNDLYMEMK			Oxidation (M)[7]		Mascot
1328.6705	1328.5929	-0.0776	-58	192	205	AHGGVSVFGGVGER					Mascot
1431.6863	1431.6031	-0.0832	-58	379	390	IVGEEHYETAQR					Mascot
1433.7747	1433.6885	-0.0862	-60	278	291	FVQAGSEVSALLGR	51	99.907			Mascot
1433.7747	1433.6885	-0.0862	-60	278	291	FVQAGSEVSALLGR					Mascot
1471.7614	1471.6768	-0.0846	-57	249	261	VGLTALTMAEYFR					Mascot
1471.7614	1471.6768	-0.0846	-57	249	261	VGLTALTMAEYFR	53	99.94			Mascot
1486.6665	1486.5858	-0.0807	-54	206	217	TREGNDLYMEMK					Mascot
1487.7562	1487.6637	-0.0925	-62	249	261	VGLTALTMAEYFR			Oxidation (M)[8]		Mascot
1502.6614	1502.7535	0.0921	61	206	217	TREGNDLYMEMK			Oxidation (M)[9]		Mascot
1517.7441	1517.6451	-0.099	-65	218	231	ESGVINEQNIAESK					Mascot
1601.8104	1601.7146	-0.0958	-60	232	246	VALVYGQMNEPPGAR	72	100			Mascot
1601.8104	1601.7146	-0.0958	-60	232	246	VALVYGQMNEPPGAR					Mascot
1617.8053	1617.7068	-0.0985	-61	232	246	VALVYGQMNEPPGAR			Oxidation (M)[8]		Mascot
1735.0153	1734.9052	-0.1101	-63	23	39	IAQIIGPVLDVAFPPGK					Mascot
1949.9967	1949.8717	-0.125	-64	262	277	DVNEQDVLLFIDNIFR					Mascot
1949.9967	1949.8717	-0.125	-64	262	277	DVNEQDVLLFIDNIFR	152	100			Mascot
1969.0388	1968.9137	-0.1251	-64	110	127	IFNVLGEPIDNLGPVDTR					Mascot
2061.0322	2060.9009	-0.1313	-64	360	378	GIYPAVDPLDSTSTMLQPR					Mascot
2077.0271	2076.8945	-0.1326	-64	360	378	GIYPAVDPLDSTSTMLQPR			Oxidation (M)[15]		Mascot
2171.1077	2170.9673	-0.1404	-65	400	418	ELQDIIAILGLDELSEEDR					Mascot
2282.0791	2281.9272	-0.1519	-67	292	312	MPSAVGYQPTLSTEMGSLQER					Mascot
2282.0791	2281.9272	-0.1519	-67	292	312	MPSAVGYQPTLSTEMGSLQER	125	100			Mascot
2298.074	2297.9253	-0.1487	-65	292	312	MPSAVGYQPTLSTEMGSLQER			Oxidation (M)[1]		Mascot
2314.0688	2313.9287	-0.1401	-61	292	312	MPSAVGYQPTLSTEMGSLQER			Oxidation (M)[1,15]		Mascot
2386.178	2386.022	-0.156	-65	53	73	DTVGQQINVTCEVQQLLGNNR	152	100	Carbamidomethyl (C)[11]		Mascot
2386.178	2386.022	-0.156	-65	53	73	DTVGQQINVTCEVQQLLGNNR			Carbamidomethyl (C)[11]		Mascot
2462.2661	2462.1001	-0.166	-67	398	418	YKELQDIIAILGLDELSEEDR					Mascot
2599.3005	2599.1211	-0.1794	-69	51	73	GRDTVGQQINVTCEVQQLLGNNR			Carbamidomethyl (C)[13]		Mascot
2711.446	2711.2666	-0.1794	-66	400	423	ELQDIIAILGLDELSEEDRLTVAR					Mascot
2876.4927	2876.3005	-0.1922	-67	460	486	LILSGELDGLPEQAFYLVGNIDEATAK					Mascot
3843.9287	3843.6604	-0.2683	-70	318	354	EGSITSIQAVYVPADDLTDPAPATTFAHLDATTVLSR					Mascot

Rank	Protein Name	Accession No	Protein MW	Protein PI	Pep. Count	Protein Score	Protein Score C. I. %	Total Ion Score	Total Ion C. I. %
6	ATP synthase subunit beta, chloroplastic OS=Galbulimima belgraveana GN=atpB PE=3 SV=1	ATPB_GALBE	53610.9	5.44	25	816	100	602	100

Peptide Information

Calc. Mass	Obsrv. Mass	± da	± ppm	Start Seq.	End Seq.	Sequence	Ion Score	C. I. %	Modification	Rank	Result Type
811.4421	811.4009	-0.0412	-51	128	134	TTSPIHR					Mascot
910.4741	910.3912	-0.0829	-91	393	399	QTSQRYK					Mascot
975.5621	975.5026	-0.0595	-61	168	178	IGLFGGAGVGK					Mascot
1021.5676	1021.4725	-0.0951	-93	448	456	YVGLAETIR					Mascot
1045.604	1045.5453	-0.0587	-56	155	163	VVDLLAPYR					Mascot
1190.6416	1190.5757	-0.0659	-55	135	145	SAPAFIQLDTK					Mascot
1201.7052	1201.6393	-0.0659	-55	155	164	VVDLLAPYRR					Mascot
1229.5177	1229.4539	-0.0638	-52	208	217	EGNDLYMEMK					Mascot
1245.5126	1245.5599	0.0473	38	208	217	EGNDLYMEMK			Oxidation (M)[7]		Mascot
1328.6705	1328.5929	-0.0776	-58	192	205	AHGGVSVFGGVGER					Mascot
1431.6863	1431.6031	-0.0832	-58	379	390	IVGEEHYETAQR					Mascot
1433.7747	1433.6885	-0.0862	-60	278	291	FVQAGSEVSALLGR	51	99.907			Mascot
1433.7747	1433.6885	-0.0862	-60	278	291	FVQAGSEVSALLGR					Mascot
1471.7614	1471.6768	-0.0846	-57	249	261	VGLTALTMAEYFR					Mascot
1471.7614	1471.6768	-0.0846	-57	249	261	VGLTALTMAEYFR	53	99.94			Mascot
1486.6665	1486.5858	-0.0807	-54	206	217	TREGNDLYMEMK					Mascot
1487.7562	1487.6637	-0.0925	-62	249	261	VGLTALTMAEYFR			Oxidation (M)[8]		Mascot
1502.6614	1502.7535	0.0921	61	206	217	TREGNDLYMEMK			Oxidation (M)[9]		Mascot
1517.7441	1517.6451	-0.099	-65	218	231	ESGVINEQNIAESK					Mascot
1601.8104	1601.7146	-0.0958	-60	232	246	VALVYGQMNEPPGAR	72	100			Mascot
1601.8104	1601.7146	-0.0958	-60	232	246	VALVYGQMNEPPGAR					Mascot
1617.8053	1617.7068	-0.0985	-61	232	246	VALVYGQMNEPPGAR			Oxidation (M)[8]		Mascot
1735.0153	1734.9052	-0.1101	-63	23	39	IAQIIGPVLDVAFPPGK					Mascot
1949.9967	1949.8717	-0.125	-64	262	277	DVNEQDVLLFIDNIFR					Mascot
1949.9967	1949.8717	-0.125	-64	262	277	DVNEQDVLLFIDNIFR	152	100			Mascot
2061.0322	2060.9009	-0.1313	-64	360	378	GIYPAVDPLDSTSTMLQPR					Mascot
2077.0271	2076.8945	-0.1326	-64	360	378	GIYPAVDPLDSTSTMLQPR			Oxidation (M)[15]		Mascot
2171.1077	2170.9673	-0.1404	-65	400	418	ELQDIIAILGLDELSEEDR					Mascot
2282.0791	2281.9272	-0.1519	-67	292	312	MPSAVGYQPTLSTEMGSLQER					Mascot
2282.0791	2281.9272	-0.1519	-67	292	312	MPSAVGYQPTLSTEMGSLQER	125	100			Mascot
2298.074	2297.9253	-0.1487	-65	292	312	MPSAVGYQPTLSTEMGSLQER			Oxidation (M)[1]		Mascot
2314.0688	2313.9287	-0.1401	-61	292	312	MPSAVGYQPTLSTEMGSLQER			Oxidation (M)[1,15]		Mascot
2386.178	2386.022	-0.156	-65	53	73	DTVGQQINVTCEVQQLLGNNR	152	100	Carbamidomethyl (C)[11]		Mascot
2386.178	2386.022	-0.156	-65	53	73	DTVGQQINVTCEVQQLLGNNR			Carbamidomethyl (C)[11]		Mascot
2462.2661	2462.1001	-0.166	-67	398	418	YKELQDIIAILGLDELSEEDR					Mascot
2599.3005	2599.1211	-0.1794	-69	51	73	GRDTVGQQINVTCEVQQLLGNNR			Carbamidomethyl (C)[13]		Mascot
2711.446	2711.2666	-0.1794	-66	400	423	ELQDIIAILGLDELSEEDRLTVAR					Mascot
3843.9287	3843.6604	-0.2683	-70	318	354	EGSITSIQAVYVPADDLTDPAPATTFAHLDATTVLSR					Mascot

Rank	Protein Name	Accession No	Protein MW	Protein PI	Pep. Count	Protein Score	Protein Score C. I. %	Total Ion Score	Total Ion C. I. %
7	ATP synthase subunit beta, chloroplastic OS=Lo japonicus GN=atpB PE=3 SV=1	ATPB_LOTJA	53779.2	5.37	25	816	100	602	100

Peptide Information

Calc. Mass	Obsrv. Mass	± da	± ppm	Start Seq.	End Seq.	Sequence	Ion Score	C. I. %	Modification	Rank	Result Type
811.4421	811.4009	-0.0412	-51	128	134	TTSPIHR					Mascot
975.5621	975.5026	-0.0595	-61	168	178	IGLFGGAGVGK					Mascot
1021.5676	1021.4725	-0.0951	-93	448	456	YVGLAETIR					Mascot
1045.604	1045.5453	-0.0587	-56	155	163	VVDLLAPYR					Mascot
1190.6416	1190.5757	-0.0659	-55	135	145	SAPAFIQLDTK					Mascot
1201.7052	1201.6393	-0.0659	-55	155	164	VVDLLAPYRR					Mascot
1229.5177	1229.4539	-0.0638	-52	208	217	EGNDLYMEMK					Mascot
1245.5126	1245.5599	0.0473	38	208	217	EGNDLYMEMK			Oxidation (M)[7]		Mascot
1275.7129	1275.6541	-0.0588	-46	40	50	MPNIYNALIVK					Mascot
1328.6705	1328.5929	-0.0776	-58	192	205	AHGGVSVFGGVGER					Mascot
1431.6863	1431.6031	-0.0832	-58	379	390	IVGEEHYETAQR					Mascot
1433.7747	1433.6885	-0.0862	-60	278	291	FVQAGSEVSALLGR	51	99.907			Mascot
1433.7747	1433.6885	-0.0862	-60	278	291	FVQAGSEVSALLGR					Mascot
1471.7614	1471.6768	-0.0846	-57	249	261	VGLTALTMAEYFR					Mascot
1471.7614	1471.6768	-0.0846	-57	249	261	VGLTALTMAEYFR	53	99.94			Mascot
1486.6665	1486.5858	-0.0807	-54	206	217	TREGNDLYMEMK					Mascot
1487.7562	1487.6637	-0.0925	-62	249	261	VGLTALTMAEYFR			Oxidation (M)[8]		Mascot
1502.6614	1502.7535	0.0921	61	206	217	TREGNDLYMEMK			Oxidation (M)[9]		Mascot
1517.7805	1517.6451	-0.1354	-89	218	231	ESGVINEKNIAESK					Mascot
1601.8104	1601.7146	-0.0958	-60	232	246	VALVYGQMNEPPGAR	72	100			Mascot
1601.8104	1601.7146	-0.0958	-60	232	246	VALVYGQMNEPPGAR					Mascot
1617.8053	1617.7068	-0.0985	-61	232	246	VALVYGQMNEPPGAR			Oxidation (M)[8]		Mascot
1949.9967	1949.8717	-0.125	-64	262	277	DVNEQDVLLFIDNIFR					Mascot
1949.9967	1949.8717	-0.125	-64	262	277	DVNEQDVLLFIDNIFR	152	100			Mascot
1969.0388	1968.9137	-0.1251	-64	110	127	IFNVLGEPIDNLGPVDTR					Mascot
2061.0322	2060.9009	-0.1313	-64	360	378	GIYPAVDPLDSTSTMLQPR					Mascot
2077.0271	2076.8945	-0.1326	-64	360	378	GIYPAVDPLDSTSTMLQPR			Oxidation (M)[15]		Mascot
2171.1077	2170.9673	-0.1404	-65	400	418	ELQDIIAILGLDELSEEDR					Mascot
2282.0791	2281.9272	-0.1519	-67	292	312	MPSAVGYQPTLSTEMGSLQER					Mascot
2282.0791	2281.9272	-0.1519	-67	292	312	MPSAVGYQPTLSTEMGSLQER	125	100			Mascot
2298.074	2297.9253	-0.1487	-65	292	312	MPSAVGYQPTLSTEMGSLQER			Oxidation (M)[1]		Mascot
2314.0688	2313.9287	-0.1401	-61	292	312	MPSAVGYQPTLSTEMGSLQER			Oxidation (M)[1,15]		Mascot
2386.178	2386.022	-0.156	-65	53	73	DTVGQQINVTCEVQQLLGNNR	152	100	Carbamidomethyl (C)[11]		Mascot
2386.178	2386.022	-0.156	-65	53	73	DTVGQQINVTCEVQQLLGNNR			Carbamidomethyl (C)[11]		Mascot
2462.2661	2462.1001	-0.166	-67	398	418	YKELQDIIAILGLDELSEEDR					Mascot
2599.3005	2599.1211	-0.1794	-69	51	73	GRDTVGQQINVTCEVQQLLGNNR			Carbamidomethyl (C)[13]		Mascot
2711.446	2711.2666	-0.1794	-66	400	423	ELQDIIAILGLDELSEEDRLTVAR					Mascot
3843.9287	3843.6604	-0.2683	-70	318	354	EGSITSIQAVYVPADDLTDPAPATTFAHLDATTVLSR					Mascot

Rank	Protein Name	Accession No	Protein MW	Protein PI	Pep. Count	Protein Score	Protein Score C. I. %	Total Ion Score	Total Ion C. I. %
8	ATP synthase subunit beta, chloroplastic OS=Ca papaya GN=atpB PE=3 SV=1	ATPB_CARPA	53609	5.46	24	803	100	602	100

Peptide Information

Calc. Mass	Obsrv. Mass	± da	± ppm	Start Seq.	End Seq.	Sequence	Ion Score	C. I. %	Modification	Rank	Result Type
975.5621	975.5026	-0.0595	-61	168	178	IGLFGGAGVGK					Mascot
1021.5676	1021.4725	-0.0951	-93	448	456	YVGLAETIR					Mascot
1045.604	1045.5453	-0.0587	-56	155	163	VVDLLAPYR					Mascot
1190.6416	1190.5757	-0.0659	-55	135	145	SAPAFIQLDTK					Mascot
1201.7052	1201.6393	-0.0659	-55	155	164	VVDLLAPYRR					Mascot
1229.5177	1229.4539	-0.0638	-52	208	217	EGNDLYMEMK					Mascot
1245.5126	1245.5599	0.0473	38	208	217	EGNDLYMEMK			Oxidation (M)[7]		Mascot
1328.6705	1328.5929	-0.0776	-58	192	205	AHGGVSVFGGVGER					Mascot
1431.6863	1431.6031	-0.0832	-58	379	390	IVGEEHYETAQR					Mascot
1433.7747	1433.6885	-0.0862	-60	278	291	FVQAGSEVSALLGR	51	99.907			Mascot
1433.7747	1433.6885	-0.0862	-60	278	291	FVQAGSEVSALLGR					Mascot
1471.7614	1471.6768	-0.0846	-57	249	261	VGLTALTMAEYFR					Mascot
1471.7614	1471.6768	-0.0846	-57	249	261	VGLTALTMAEYFR	53	99.94			Mascot
1486.6665	1486.5858	-0.0807	-54	206	217	TREGNDLYMEMK					Mascot
1487.7562	1487.6637	-0.0925	-62	249	261	VGLTALTMAEYFR			Oxidation (M)[8]		Mascot
1502.6614	1502.7535	0.0921	61	206	217	TREGNDLYMEMK			Oxidation (M)[9]		Mascot
1517.7441	1517.6451	-0.099	-65	218	231	ESGVINEQNLAESK					Mascot
1601.8104	1601.7146	-0.0958	-60	232	246	VALVYGQMNEPPGAR	72	100			Mascot
1601.8104	1601.7146	-0.0958	-60	232	246	VALVYGQMNEPPGAR					Mascot
1617.8053	1617.7068	-0.0985	-61	232	246	VALVYGQMNEPPGAR			Oxidation (M)[8]		Mascot
1735.0153	1734.9052	-0.1101	-63	23	39	IAQIIGPVLDVAFPPGK					Mascot
1949.9967	1949.8717	-0.125	-64	262	277	DVNEQDVLLFIDNIFR					Mascot
1949.9967	1949.8717	-0.125	-64	262	277	DVNEQDVLLFIDNIFR	152	100			Mascot
2061.0322	2060.9009	-0.1313	-64	360	378	GIYPAVDPLDSTSTMLQPR					Mascot
2077.0271	2076.8945	-0.1326	-64	360	378	GIYPAVDPLDSTSTMLQPR			Oxidation (M)[15]		Mascot
2171.1077	2170.9673	-0.1404	-65	400	418	ELQDIIAILGLDELSEEDR					Mascot
2282.0791	2281.9272	-0.1519	-67	292	312	MPSAVGYQPTLSTEMGSLQER					Mascot
2282.0791	2281.9272	-0.1519	-67	292	312	MPSAVGYQPTLSTEMGSLQER	125	100			Mascot
2298.074	2297.9253	-0.1487	-65	292	312	MPSAVGYQPTLSTEMGSLQER			Oxidation (M)[1]		Mascot
2314.0688	2313.9287	-0.1401	-61	292	312	MPSAVGYQPTLSTEMGSLQER			Oxidation (M)[1,15]		Mascot
2386.178	2386.022	-0.156	-65	53	73	DTVGQQINVTCEVQQLLGNNR	152	100	Carbamidomethyl (C)[11]		Mascot
2386.178	2386.022	-0.156	-65	53	73	DTVGQQINVTCEVQQLLGNNR			Carbamidomethyl (C)[11]		Mascot
2462.2661	2462.1001	-0.166	-67	398	418	YKELQDIIAILGLDELSEEDR					Mascot
2599.3005	2599.1211	-0.1794	-69	51	73	GRDTVGQQINVTCEVQQLLGNNR			Carbamidomethyl (C)[13]		Mascot
2711.446	2711.2666	-0.1794	-66	400	423	ELQDIIAILGLDELSEEDRLTVAR					Mascot
2876.4927	2876.3005	-0.1922	-67	460	486	LILSGELDGLPEQAFYLVGNIDEATAK					Mascot
3843.9287	3843.6604	-0.2683	-70	318	354	EGSITSIQAVYVPADDLTDPAPATTFAHLDATTVLSR					Mascot

Rank	Protein Name	Accession No	Protein MW	Protein PI	Pep. Count	Protein Score	Protein Score C. I. %	Total Ion Score	Total Ion C. I. %
9	ATP synthase subunit beta, chloroplastic OS=Ba seemannii GN=atpB PE=3 SV=1	ATPB_BALSE	53764.1	5.29	24	803	100	602	100

Peptide Information

Calc. Mass	Obsrv. Mass	± da	± ppm	Start Seq.	End Seq.	Sequence	Ion Score	C. I. %	Modification	Rank	Result Type
811.4421	811.4009	-0.0412	-51	128	134	TTSPIHR					Mascot
910.4741	910.3912	-0.0829	-91	393	399	QTSQRYK					Mascot
975.5621	975.5026	-0.0595	-61	168	178	IGLFGGAGVGK					Mascot
1021.5676	1021.4725	-0.0951	-93	448	456	YVGLAETIR					Mascot
1045.604	1045.5453	-0.0587	-56	155	163	VVDLLAPYR					Mascot
1190.6416	1190.5757	-0.0659	-55	135	145	SAPAFIQLDTK					Mascot
1201.7052	1201.6393	-0.0659	-55	155	164	VVDLLAPYRR					Mascot
1229.5177	1229.4539	-0.0638	-52	208	217	EGNDLYMEMK					Mascot
1245.5126	1245.5599	0.0473	38	208	217	EGNDLYMEMK			Oxidation (M)[7]		Mascot
1328.6705	1328.5929	-0.0776	-58	192	205	AHGGVSVFGGVGER					Mascot
1431.6863	1431.6031	-0.0832	-58	379	390	IVGEEHYETAQR					Mascot
1433.7747	1433.6885	-0.0862	-60	278	291	FVQAGSEVSALLGR	51	99.907			Mascot
1433.7747	1433.6885	-0.0862	-60	278	291	FVQAGSEVSALLGR					Mascot
1471.7614	1471.6768	-0.0846	-57	249	261	VGLTALTMAEYFR					Mascot
1471.7614	1471.6768	-0.0846	-57	249	261	VGLTALTMAEYFR	53	99.94			Mascot
1486.6665	1486.5858	-0.0807	-54	206	217	TREGNDLYMEMK					Mascot
1487.7562	1487.6637	-0.0925	-62	249	261	VGLTALTMAEYFR			Oxidation (M)[8]		Mascot
1502.6614	1502.7535	0.0921	61	206	217	TREGNDLYMEMK			Oxidation (M)[9]		Mascot
1517.7805	1517.6451	-0.1354	-89	218	231	ESGVINEKNIAESK					Mascot
1601.8104	1601.7146	-0.0958	-60	232	246	VALVYGQMNEPPGAR	72	100			Mascot
1601.8104	1601.7146	-0.0958	-60	232	246	VALVYGQMNEPPGAR					Mascot
1617.8053	1617.7068	-0.0985	-61	232	246	VALVYGQMNEPPGAR			Oxidation (M)[8]		Mascot
1949.9967	1949.8717	-0.125	-64	262	277	DVNEQDVLLFIDNIFR					Mascot
1949.9967	1949.8717	-0.125	-64	262	277	DVNEQDVLLFIDNIFR	152	100			Mascot
2061.0322	2060.9009	-0.1313	-64	360	378	GIYPAVDPLDSTSTMLQPR					Mascot
2077.0271	2076.8945	-0.1326	-64	360	378	GIYPAVDPLDSTSTMLQPR			Oxidation (M)[15]		Mascot
2171.1077	2170.9673	-0.1404	-65	400	418	ELQDIIAILGLDELSEEDR					Mascot
2282.0791	2281.9272	-0.1519	-67	292	312	MPSAVGYQPTLSTEMGSLQER					Mascot
2282.0791	2281.9272	-0.1519	-67	292	312	MPSAVGYQPTLSTEMGSLQER	125	100			Mascot
2298.074	2297.9253	-0.1487	-65	292	312	MPSAVGYQPTLSTEMGSLQER			Oxidation (M)[1]		Mascot
2314.0688	2313.9287	-0.1401	-61	292	312	MPSAVGYQPTLSTEMGSLQER			Oxidation (M)[1,15]		Mascot
2386.178	2386.022	-0.156	-65	53	73	DTVGQQINVTCEVQQLLGNNR	152	100	Carbamidomethyl (C)[11]		Mascot
2386.178	2386.022	-0.156	-65	53	73	DTVGQQINVTCEVQQLLGNNR			Carbamidomethyl (C)[11]		Mascot
2462.2661	2462.1001	-0.166	-67	398	418	YKELQDIIAILGLDELSEEDR					Mascot
2599.3005	2599.1211	-0.1794	-69	51	73	GRDTVGQQINVTCEVQQLLGNNR			Carbamidomethyl (C)[13]		Mascot
2711.446	2711.2666	-0.1794	-66	400	423	ELQDIIAILGLDELSEEDRLTVAR					Mascot
3843.9287	3843.6604	-0.2683	-70	318	354	EGSITSIQAVYVPADDLTDPAPATTFAHLDATTVLSR					Mascot

Rank	Protein Name	Accession No	Protein MW	Protein PI	Pep. Count	Protein Score	Protein Score C. I. %	Total Ion Score	Total Ion C. I. %
10	ATP synthase subunit beta, chloroplastic OS=My fragrans GN=atpB PE=3 SV=1	ATPB_MYRF	53535.7	5.09	24	803	100	602	100

Peptide Information

Calc. Mass	Obsrv. Mass	± da	± ppm	Start Seq.	End Seq.	Sequence	Ion Score	C. I. %	Modification	Rank	Result Type
811.4421	811.4009	-0.0412	-51	128	134	TTSPIHR					Mascot
910.4741	910.3912	-0.0829	-91	393	399	QTSQRYK					Mascot
975.5621	975.5026	-0.0595	-61	168	178	IGLFGGAGVGK					Mascot
1021.5676	1021.4725	-0.0951	-93	448	456	YVGLAETIR					Mascot
1045.604	1045.5453	-0.0587	-56	155	163	VVDLLAPYR					Mascot
1190.6416	1190.5757	-0.0659	-55	135	145	SAPAFIQLDTK					Mascot
1201.7052	1201.6393	-0.0659	-55	155	164	VVDLLAPYRR					Mascot
1229.5177	1229.4539	-0.0638	-52	208	217	EGNDLYMEMK					Mascot
1245.5126	1245.5599	0.0473	38	208	217	EGNDLYMEMK			Oxidation (M)[7]		Mascot
1328.6705	1328.5929	-0.0776	-58	192	205	AHGGVSVFGGVGER					Mascot
1431.6863	1431.6031	-0.0832	-58	379	390	IVGEEHYETAQR					Mascot
1433.7747	1433.6885	-0.0862	-60	278	291	FVQAGSEVSALLGR	51	99.885			Mascot
1433.7747	1433.6885	-0.0862	-60	278	291	FVQAGSEVSALLGR					Mascot
1471.7614	1471.6768	-0.0846	-57	249	261	VGLTALTMAEYFR					Mascot
1471.7614	1471.6768	-0.0846	-57	249	261	VGLTALTMAEYFR	53	99.927			Mascot
1486.6665	1486.5858	-0.0807	-54	206	217	TREGNDLYMEMK					Mascot
1487.7562	1487.6637	-0.0925	-62	249	261	VGLTALTMAEYFR			Oxidation (M)[8]		Mascot
1502.6614	1502.7535	0.0921	61	206	217	TREGNDLYMEMK			Oxidation (M)[9]		Mascot
1517.7441	1517.6451	-0.099	-65	218	231	ESGVINEQNIAESK					Mascot
1601.8104	1601.7146	-0.0958	-60	232	246	VALVYGQMNEPPGAR	72	100			Mascot
1601.8104	1601.7146	-0.0958	-60	232	246	VALVYGQMNEPPGAR					Mascot
1617.8053	1617.7068	-0.0985	-61	232	246	VALVYGQMNEPPGAR			Oxidation (M)[8]		Mascot
1949.9967	1949.8717	-0.125	-64	262	277	DVNEQDVLLFIDNIFR					Mascot
1949.9967	1949.8717	-0.125	-64	262	277	DVNEQDVLLFIDNIFR	152	100			Mascot
2061.0322	2060.9009	-0.1313	-64	360	378	GIYPAVDPLDSTSTMLQPR					Mascot
2077.0271	2076.8945	-0.1326	-64	360	378	GIYPAVDPLDSTSTMLQPR			Oxidation (M)[15]		Mascot
2171.1077	2170.9673	-0.1404	-65	400	418	ELQDIIAILGLDELSEEDR					Mascot
2282.0791	2281.9272	-0.1519	-67	292	312	MPSAVGYQPTLSTEMGSLQER					Mascot
2282.0791	2281.9272	-0.1519	-67	292	312	MPSAVGYQPTLSTEMGSLQER	125	100			Mascot
2298.074	2297.9253	-0.1487	-65	292	312	MPSAVGYQPTLSTEMGSLQER			Oxidation (M)[1]		Mascot
2314.0688	2313.9287	-0.1401	-61	292	312	MPSAVGYQPTLSTEMGSLQER			Oxidation (M)[1,15]		Mascot
2386.178	2386.022	-0.156	-65	53	73	DTVGQQINVTCEVQQLLGNNR	152	100	Carbamidomethyl (C)[11]		Mascot
2386.178	2386.022	-0.156	-65	53	73	DTVGQQINVTCEVQQLLGNNR			Carbamidomethyl (C)[11]		Mascot
2462.2661	2462.1001	-0.166	-67	398	418	YKELQDIIAILGLDELSEEDR					Mascot
2599.3005	2599.1211	-0.1794	-69	51	73	GRDTVGQQINVTCEVQQLLGNNR			Carbamidomethyl (C)[13]		Mascot
2711.446	2711.2666	-0.1794	-66	400	423	ELQDIIAILGLDELSEEDRLTVAR					Mascot
3843.9287	3843.6604	-0.2683	-70	318	354	EGSITSIQAVYVPADDLTDPAPATTFAHLDATTVLSR					Mascot

Mascot

Gel Idx/Pos	48/B21	Instr./Gel Origin	BA2060/131017A	Process Sta	Analysis Succeeded
Plate [#] Name	[1] 31496	Instrument Sample Name		Spectra	8

Rank	Protein Name	Accession Nc	Protein MW	Protein PI	Pep. Count	Protein Score	Protein Score C. I. %	Total Ion Score	Total Ion C. I. %
1	Ribulose bisphosphate carboxylase large chain OS=Glycine max GN=rbcL PE=1 SV=3	RBL_SOYBN	52576.4	6	26	895	100	671	100

Peptide Information

Calc. Mass	Obsrv. Mass	± da	± ppm	Start Seq.	End Seq.	Sequence	Ion Score	C. I. %	Modification	Rank	Result Type
805.4818	805.4423	-0.0395	-49	140	146	IPTAYIK					Mascot
830.4366	830.399	-0.0376	-45	440	446	EGNEIIR					Mascot
853.4236	853.3833	-0.0403	-47	188	194	AVYECLR					Mascot
898.41	898.3663	-0.0437	-49	306	312	NHGMHFR					Mascot
910.4451	910.4037	-0.0414	-45	188	194	AVYECLR			Carbamidomethyl (C)[5]		Mascot
912.4719	912.4191	-0.0528	-58	296	303	AMHAVIDR					Mascot
914.4049	914.3641	-0.0408	-45	306	312	NHGMHFR			Oxidation (M)[4]		Mascot
928.4669	928.4246	-0.0423	-46	296	303	AMHAVIDR			Oxidation (M)[2]		Mascot
962.4788	962.4345	-0.0443	-46	228	236	SQAETGEIK					Mascot
1021.5312	1021.4844	-0.0468	-46	33	41	DTDILAAFR	63	99.992			Mascot
1021.5312	1021.4844	-0.0468	-46	33	41	DTDILAAFR					Mascot
1116.583	1116.5306	-0.0524	-47	422	431	VALEACVQAR			Carbamidomethyl (C)[6]		Mascot
1170.5585	1170.5833	0.0248	21	304	312	QKNHGMHFR			Oxidation (M)[6]		Mascot
1187.6643	1187.6108	-0.0535	-45	286	295	DNGLLLHIHR					Mascot
1245.6337	1245.5757	-0.058	-47	218	227	FLFCAEAIFK			Carbamidomethyl (C)[4]		Mascot
1275.7307	1275.6705	-0.0602	-47	340	350	EITLGFVDLLR	92	100			Mascot
1275.7307	1275.6705	-0.0602	-47	340	350	EITLGFVDLLR					Mascot
1393.6522	1393.5815	-0.0707	-51	22	32	LTYYTPDYETK					Mascot
1433.7495	1433.6879	-0.0616	-43	320	334	LSGGDHVHAGTVVGK					Mascot
1451.6219	1451.551	-0.0709	-49	202	213	DDENVNSQPFMR					Mascot
1465.7546	1465.6815	-0.0731	-50	147	159	TFQGPPHGIQVER					Mascot
1465.7546	1465.6815	-0.0731	-50	147	159	TFQGPPHGIQVER	89	100			Mascot
1472.6978	1472.6267	-0.0711	-48	464	475	EIKFEFEAMDTL					Mascot
1488.6926	1488.6224	-0.0702	-47	464	475	EIKFEFEAMDTL			Oxidation (M)[9]		Mascot
1502.8512	1502.7758	-0.0754	-50	165	177	YGRPLLGCTIKPK			Carbamidomethyl (C)[8]		Mascot
1516.7616	1516.7593	-0.0023	-2	216	227	DRFLFCAEAIFK			Carbamidomethyl (C)[6]		Mascot
1546.7358	1546.6554	-0.0804	-52	451	463	WSPELAAACEVWK			Carbamidomethyl (C)[9]		Mascot
1812.7714	1812.6749	-0.0965	-53	237	252	GHYLNATAGTCEEMMK			Carbamidomethyl (C)[11]		Mascot
1828.7662	1828.6735	-0.0927	-51	237	252	GHYLNATAGTCEEMMK			Carbamidomethyl (C)[11], Oxidation (M)[14]		Mascot
1968.8724	1968.9298	0.0574	29	237	253	GHYLNATAGTCEEMMKR			Carbamidomethyl (C)[11]		Mascot
1984.8673	1984.7522	-0.1151	-58	237	253	GHYLNATAGTCEEMMKR			Carbamidomethyl (C)[11], Oxidation (M)[14]		Mascot
2009.059	2008.9478	-0.1112	-55	340	356	EITLGFVDLLRDDFVEK					Mascot
2169.9868	2169.8689	-0.1179	-54	195	213	GGLDFTKDDENVNSQPFMR					Mascot
2169.9868	2169.8689	-0.1179	-54	195	213	GGLDFTKDDENVNSQPFMR	104	100			Mascot
2185.9819	2185.8633	-0.1186	-54	195	213	GGLDFTKDDENVNSQPFMR			Oxidation (M)[18]		Mascot
3022.4548	3022.283	-0.1718	-57	259	285	ELGVPIVMHDYLTGGFTANTSLAHYCR	114	100	Carbamidomethyl (C)[26]		Mascot
3022.4548	3022.283	-0.1718	-57	259	285	ELGVPIVMHDYLTGGFTANTSLAHYCR			Carbamidomethyl (C)[26]		Mascot
3038.45	3038.2795	-0.1705	-56	259	285	ELGVPIVMHDYLTGGFTANTSLAHYCR			Carbamidomethyl (C)[26], Oxidation (M)[8]		Mascot
3854.8718	3854.6453	-0.2265	-59	42	79	VTPQPGVPPEEAGAAVAAESSTGTWTTVWTDGLTSLDR					Mascot
3854.8718	3854.6453	-0.2265	-59	42	79	VTPQPGVPPEEAGAAVAAESSTGTWTTVWTDGLTSLDR	210	100			Mascot

Rank	Protein Name	Accession Nc	Protein MW	Protein PI	Pep. Count	Protein Score	Protein Score C. I. %	Total Ion Score	Total Ion C. I. %
2	Ribulose bisphosphate carboxylase large chain (Fragment) OS=Geum quellyon GN=rbcL PE=3 SV=1	RBL_GEUQU	50910.8	6.23	23	863	100	670	100

Peptide Information

Calc. Mass	Obsrv. Mass	± da	± ppm	Start Seq.	End Seq.	Sequence	Ion Score	C. I. %	Modification	Rank	Result Type
805.4929	805.4423	-0.0506	-63	1	8	VGFKAGVK					Mascot
853.4236	853.3833	-0.0403	-47	178	184	AVYECLR					Mascot
898.41	898.3663	-0.0437	-49	296	302	NHGMHFR					Mascot
910.4451	910.4037	-0.0414	-45	178	184	AVYECLR			Carbamidomethyl (C)[5]		Mascot
912.4719	912.4191	-0.0528	-58	286	293	AMHAVIDR					Mascot
914.4049	914.3641	-0.0408	-45	296	302	NHGMHFR			Oxidation (M)[4]		Mascot
928.4669	928.4246	-0.0423	-46	286	293	AMHAVIDR			Oxidation (M)[2]		Mascot
1021.5312	1021.4844	-0.0468	-46	23	31	DTDILAAFR	63	99.992			Mascot
1021.5312	1021.4844	-0.0468	-46	23	31	DTDILAAFR					Mascot
1116.583	1116.5306	-0.0524	-47	412	421	VALEACVQAR			Carbamidomethyl (C)[6]		Mascot
1170.5585	1170.5833	0.0248	21	294	302	QKNHGMHFR			Oxidation (M)[6]		Mascot
1187.6643	1187.6108	-0.0535	-45	276	285	DNGLLLHIHR					Mascot
1245.6337	1245.5757	-0.058	-47	208	217	FLFCAEALFK			Carbamidomethyl (C)[4]		Mascot
1275.7307	1275.6705	-0.0602	-47	330	340	EITLGFVDLLR	92	100			Mascot
1275.7307	1275.6705	-0.0602	-47	330	340	EITLGFVDLLR					Mascot
1393.6522	1393.5815	-0.0707	-51	12	22	LTYYTPDYETK					Mascot
1451.6219	1451.551	-0.0709	-49	192	203	DDENVNSQPFMR					Mascot
1465.7216	1465.6815	-0.0401	-27	310	324	MSGGDHIHAGTVVGK					Mascot
1465.7546	1465.6815	-0.0731	-50	137	149	TFQGPPHGIQVER	89	100			Mascot
1502.8512	1502.7758	-0.0754	-50	155	167	YGRPLLGCTIKPK			Carbamidomethyl (C)[8]		Mascot
1516.7616	1516.7593	-0.0023	-2	206	217	DRFLFCAEALFK			Carbamidomethyl (C)[6]		Mascot
1546.7358	1546.6554	-0.0804	-52	441	453	WSPELAAACEVWK			Carbamidomethyl (C)[9]		Mascot
1812.7714	1812.6749	-0.0965	-53	227	242	GHYLNATAGTCEEMMK			Carbamidomethyl (C)[11]		Mascot
1828.7662	1828.6735	-0.0927	-51	227	242	GHYLNATAGTCEEMMK			Carbamidomethyl (C)[11], Oxidation (M)[14]		Mascot
1968.8724	1968.9298	0.0574	29	227	243	GHYLNATAGTCEEMMKR			Carbamidomethyl (C)[11]		Mascot
1984.8673	1984.7522	-0.1151	-58	227	243	GHYLNATAGTCEEMMKR			Carbamidomethyl (C)[11], Oxidation (M)[14]		Mascot
2050.0134	2049.8823	-0.1311	-64	310	329	MSGGDHIHAGTVVGKLEGER					Mascot
2169.9868	2169.8689	-0.1179	-54	185	203	GGLDFTKDDENVNSQPFMR					Mascot
2169.9868	2169.8689	-0.1179	-54	185	203	GGLDFTKDDENVNSQPFMR	104	100			Mascot
2185.9819	2185.8633	-0.1186	-54	185	203	GGLDFTKDDENVNSQPFMR			Oxidation (M)[18]		Mascot
3022.4548	3022.283	-0.1718	-57	249	275	ELGVPIVMHDYLTGGFTANTSLAHYCR	114	100	Carbamidomethyl (C)[26]		Mascot
3022.4548	3022.283	-0.1718	-57	249	275	ELGVPIVMHDYLTGGFTANTSLAHYCR			Carbamidomethyl (C)[26]		Mascot
3038.45	3038.2795	-0.1705	-56	249	275	ELGVPIVMHDYLTGGFTANTSLAHYCR			Carbamidomethyl (C)[26], Oxidation (M)[8]		Mascot
3854.8718	3854.6453	-0.2265	-59	32	69	VTPQPGVPPEEAGAAVAAESSTGTWTTVWTDGLTSLDR					Mascot
3854.8718	3854.6453	-0.2265	-59	32	69	VTPQPGVPPEEAGAAVAAESSTGTWTTVWTDGLTSLDR	210	100			Mascot

Rank	Protein Name	Accession Nc	Protein MW	Protein PI	Pep. Count	Protein Score	Protein Score C. I. %	Total Ion Score	Total Ion C. I. %
3	Ribulose bisphosphate carboxylase large chain (Fragment) OS=Manilkara zapota GN=rbcL PE=3 SV=2	RBL_MANZA	51550	6.04	22	851	100	671	100

Peptide Information

Calc. Mass	Obsrv. Mass	± da	± ppm	Start Seq.	End Seq.	Sequence	Ion Score	C. I. %	Modification	Rank	Result Type	
805.4929	805.4423	-0.0506	-63	1	8	VGFKAGVK					Mascot	
830.4366	830.399	-0.0376	-45	430	436	EGNEIIR					Mascot	
853.4236	853.3833	-0.0403	-47	178	184	AVYECLR					Mascot	
898.41	898.3663	-0.0437	-49	296	302	NHGMHFR					Mascot	
910.4451	910.4037	-0.0414	-45	178	184	AVYECLR			Carbamidomethyl (C)[5]		Mascot	
912.4719	912.4191	-0.0528	-58	286	293	AMHAVIDR					Mascot	
914.4049	914.3641	-0.0408	-45	296	302	NHGMHFR			Oxidation (M)[4]		Mascot	
928.4669	928.4246	-0.0423	-46	286	293	AMHAVIDR			Oxidation (M)[2]		Mascot	
1021.5312	1021.4844	-0.0468	-46	23	31	DTDILAAFR	63	99.992			Mascot	
1021.5312	1021.4844	-0.0468	-46	23	31	DTDILAAFR					Mascot	
1116.583	1116.5306	-0.0524	-47	412	421	VALEACVQAR			Carbamidomethyl (C)[6]		Mascot	
1170.5585	1170.5833	0.0248	21	294	302	QKNHGMHFR			Oxidation (M)[6]		Mascot	
1187.6643	1187.6108	-0.0535	-45	276	285	DNGLLLHIHR					Mascot	
1245.6433	1245.5757	-0.0676	-54	430	440	EGNEIIREASK					Mascot	
1275.7307	1275.6705	-0.0602	-47	330	340	EITLGFVDLLR	92	100			Mascot	
1275.7307	1275.6705	-0.0602	-47	330	340	EITLGFVDLLR					Mascot	
1393.6522	1393.5815	-0.0707	-51	12	22	LTYYTPDYETK					Mascot	
1433.7495	1433.6879	-0.0616	-43	310	324	LSGGDHVHAGTVVGK					Mascot	
1451.6219	1451.551	-0.0709	-49	192	203	DDENVNSQPFMR					Mascot	
1465.7546	1465.6815	-0.0731	-50	137	149	TFQGPPHGIQVER					Mascot	
1465.7546	1465.6815	-0.0731	-50	137	149	TFQGPPHGIQVER	89	100			Mascot	
1472.6978	1472.6267	-0.0711	-48	454	465	EIKFEFEAMDTL					Mascot	
1488.6926	1488.6224	-0.0702	-47	454	465	EIKFEFEAMDTL			Oxidation (M)[9]		Mascot	
1502.8512	1502.7758	-0.0754	-50	155	167	YGRPLLGCTIKPK			Carbamidomethyl (C)[8]		Mascot	
1546.7358	1546.6554	-0.0804	-52	441	453	WSPELAAACEVWK			Carbamidomethyl (C)[9]		Mascot	
1794.8149	1794.7255	-0.0894	-50	227	242	GHYLNATAGTCEEMIK			Carbamidomethyl (C)[11]		Mascot	
2169.9868	2169.8689	-0.1179	-54	185	203	GGLDFTKDDENVNSQPFMR					Mascot	
2169.9868	2169.8689	-0.1179	-54	185	203	GGLDFTKDDENVNSQPFMR	104	100			Mascot	
2185.9819	2185.8633	-0.1186	-54	185	203	GGLDFTKDDENVNSQPFMR			Oxidation (M)[18]		Mascot	
3022.4548	3022.283	-0.1718	-57	249	275	ELGVPIVMHDYLTGGFTANTSLAHYCR	114	100	Carbamidomethyl (C)[26]		Mascot	
3022.4548	3022.283	-0.1718	-57	249	275	ELGVPIVMHDYLTGGFTANTSLAHYCR			Carbamidomethyl (C)[26]		Mascot	
3038.45	3038.2795	-0.1705	-56	249	275	ELGVPIVMHDYLTGGFTANTSLAHYCR			Carbamidomethyl (C)[26], Oxidation (M)[8]		Mascot	Mascot
3854.8718	3854.6453	-0.2265	-59	32	69	VTPQPGVPPEEAGAAVAAESSTGTWTTVWTDGLTSLDR					Mascot	
3854.8718	3854.6453	-0.2265	-59	32	69	VTPQPGVPPEEAGAAVAAESSTGTWTTVWTDGLTSLDR	210	100			Mascot	

Rank	Protein Name	Accession Nc	Protein MW	Protein PI	Pep. Count	Protein Score	Protein Score C. I. %	Total Ion Score	Total Ion C. I. %
4	Ribulose bisphosphate carboxylase large chain (Fragment) OS=Betula nigra GN=rbcL PE=3 SV=1	RBL_BETNI	51666	6.13	22	848	100	670	100

Peptide Information

Calc. Mass	Obsrv. Mass	± da	± ppm	Start Seq.	End Seq.	Sequence	Ion Score	C. I. %	Modification	Rank	Result Type
830.4366	830.399	-0.0376	-45	431	437	EGNEIIR					Mascot
853.4236	853.3833	-0.0403	-47	179	185	AVYECLR					Mascot
898.41	898.3663	-0.0437	-49	297	303	NHGMHFR					Mascot
910.4451	910.4037	-0.0414	-45	179	185	AVYECLR			Carbamidomethyl (C)[5]		Mascot
912.4719	912.4191	-0.0528	-58	287	294	AMHAVIDR					Mascot
914.4049	914.3641	-0.0408	-45	297	303	NHGMHFR			Oxidation (M)[4]		Mascot
928.4669	928.4246	-0.0423	-46	287	294	AMHAVIDR			Oxidation (M)[2]		Mascot
1021.5312	1021.4844	-0.0468	-46	24	32	DTDILAAFR	63	99.992			Mascot
1021.5312	1021.4844	-0.0468	-46	24	32	DTDILAAFR					Mascot
1116.583	1116.5306	-0.0524	-47	413	422	VALEACVQAR			Carbamidomethyl (C)[6]		Mascot
1170.5585	1170.5833	0.0248	21	295	303	QKNHGMHFR			Oxidation (M)[6]		Mascot
1187.6643	1187.6108	-0.0535	-45	277	286	DNGLLLHIHR					Mascot
1275.7307	1275.6705	-0.0602	-47	331	341	EITLGFVDLLR	92	100			Mascot
1275.7307	1275.6705	-0.0602	-47	331	341	EITLGFVDLLR					Mascot
1393.6522	1393.5815	-0.0707	-51	13	23	LTYYTPDYETK					Mascot
1451.6219	1451.551	-0.0709	-49	193	204	DDENVNSQPFMR					Mascot
1465.7216	1465.6815	-0.0401	-27	311	325	MSGGDHIHAGTVVGK					Mascot
1465.7546	1465.6815	-0.0731	-50	138	150	TFQGPPHGIQVER	89	100			Mascot
1502.8512	1502.7758	-0.0754	-50	156	168	YGRPLLGCTIKPK			Carbamidomethyl (C)[8]		Mascot
1546.7358	1546.6554	-0.0804	-52	442	454	WSPELAAACEVWK			Carbamidomethyl (C)[9]		Mascot
1812.7714	1812.6749	-0.0965	-53	228	243	GHYLNATAGTCEEMMK			Carbamidomethyl (C)[11]		Mascot
1828.7662	1828.6735	-0.0927	-51	228	243	GHYLNATAGTCEEMMK			Carbamidomethyl (C)[11], Oxidation (M)[14]		Mascot
1968.8724	1968.9298	0.0574	29	228	244	GHYLNATAGTCEEMMKR			Carbamidomethyl (C)[11]		Mascot
1984.8673	1984.7522	-0.1151	-58	228	244	GHYLNATAGTCEEMMKR			Carbamidomethyl (C)[11], Oxidation (M)[14]		Mascot
2039.0696	2038.9001	-0.1695	-83	331	347	EITLGFVDLLRDDYIEK					Mascot
2050.0134	2049.8823	-0.1311	-64	311	330	MSGGDHIHAGTVVGKLEGER					Mascot
2169.9868	2169.8689	-0.1179	-54	186	204	GGLDFTKDDENVNSQPFMR					Mascot
2169.9868	2169.8689	-0.1179	-54	186	204	GGLDFTKDDENVNSQPFMR	104	100			Mascot
2185.9819	2185.8633	-0.1186	-54	186	204	GGLDFTKDDENVNSQPFMR			Oxidation (M)[18]		Mascot
3022.4548	3022.283	-0.1718	-57	250	276	ELGVPIVMHDYLTGGFTANTSLAHYCR	114	100	Carbamidomethyl (C)[26]		Mascot
3022.4548	3022.283	-0.1718	-57	250	276	ELGVPIVMHDYLTGGFTANTSLAHYCR			Carbamidomethyl (C)[26]		Mascot
3038.45	3038.2795	-0.1705	-56	250	276	ELGVPIVMHDYLTGGFTANTSLAHYCR			Carbamidomethyl (C)[26], Oxidation (M)[8]		Mascot
3854.8718	3854.6453	-0.2265	-59	33	70	VTPQPGVPPEEAGAAVAAESSTGTWTTVWTDGLTSLDR					Mascot
3854.8718	3854.6453	-0.2265	-59	33	70	VTPQPGVPPEEAGAAVAAESSTGTWTTVWTDGLTSLDR	210	100			Mascot

Rank	Protein Name	Accession Nc	Protein MW	Protein PI	Pep. Count	Protein Score	Protein Score C. I. %	Total Ion Score	Total Ion C. I. %
5	Ribulose bisphosphate carboxylase large chain (Fragment) OS=Prunus domestica GN=rbcL PE=3 SV=1	RBL_PRUDO	50914.6	6.23	22	847	100	670	100

Peptide Information

Calc. Mass	Obsrv. Mass	± da	± ppm	Start Seq.	End Seq.	Sequence	Ion Score	C. I. %	Modification	Rank	Result Type
805.4929	805.4423	-0.0506	-63	1	8	VGFKAGVK					Mascot
830.4366	830.399	-0.0376	-45	430	436	EGNEIIR					Mascot
853.4236	853.3833	-0.0403	-47	178	184	AVYECLR					Mascot
898.41	898.3663	-0.0437	-49	296	302	NHGMHFR					Mascot
910.4451	910.4037	-0.0414	-45	178	184	AVYECLR			Carbamidomethyl (C)[5]		Mascot
912.4719	912.4191	-0.0528	-58	286	293	AMHAVIDR					Mascot
914.4049	914.3641	-0.0408	-45	296	302	NHGMHFR			Oxidation (M)[4]		Mascot
928.4669	928.4246	-0.0423	-46	286	293	AMHAVIDR			Oxidation (M)[2]		Mascot
1021.5312	1021.4844	-0.0468	-46	23	31	DTDILAAFR	63	99.992			Mascot
1021.5312	1021.4844	-0.0468	-46	23	31	DTDILAAFR					Mascot
1116.583	1116.5306	-0.0524	-47	412	421	VALEACVQAR			Carbamidomethyl (C)[6]		Mascot
1170.5585	1170.5833	0.0248	21	294	302	QKNHGMHFR			Oxidation (M)[6]		Mascot
1187.6643	1187.6108	-0.0535	-45	276	285	DNGLLLHIHR					Mascot
1245.6433	1245.5757	-0.0676	-54	430	440	EGNEIIREASK					Mascot
1275.7307	1275.6705	-0.0602	-47	330	340	EITLGFVDLLR	92	100			Mascot
1275.7307	1275.6705	-0.0602	-47	330	340	EITLGFVDLLR					Mascot
1393.6522	1393.5815	-0.0707	-51	12	22	LTYYTPDYETK					Mascot
1451.6219	1451.551	-0.0709	-49	192	203	DDENVNSQPFMR					Mascot
1465.7216	1465.6815	-0.0401	-27	310	324	MSGGDHIHAGTVVGK					Mascot
1465.7546	1465.6815	-0.0731	-50	137	149	TFQGPPHGIQVER	89	100			Mascot
1502.8512	1502.7758	-0.0754	-50	155	167	YGRPLLGCTIKPK			Carbamidomethyl (C)[8]		Mascot
1546.7358	1546.6554	-0.0804	-52	441	453	WSPELAAACEVWK			Carbamidomethyl (C)[9]		Mascot
2009.059	2008.9478	-0.1112	-55	330	346	EITLGFVDLLRDDFVEK					Mascot
2050.0134	2049.8823	-0.1311	-64	310	329	MSGGDHIHAGTVVGKLEGER					Mascot
2169.9868	2169.8689	-0.1179	-54	185	203	GGLDFTKDDENVNSQPFMR					Mascot
2169.9868	2169.8689	-0.1179	-54	185	203	GGLDFTKDDENVNSQPFMR	104	100			Mascot
2185.9819	2185.8633	-0.1186	-54	185	203	GGLDFTKDDENVNSQPFMR			Oxidation (M)[18]		Mascot
3022.4548	3022.283	-0.1718	-57	249	275	ELGVPIVMHDYLTGGFTANTSLAHYCR	114	100	Carbamidomethyl (C)[26]		Mascot
3022.4548	3022.283	-0.1718	-57	249	275	ELGVPIVMHDYLTGGFTANTSLAHYCR			Carbamidomethyl (C)[26]		Mascot
3038.45	3038.2795	-0.1705	-56	249	275	ELGVPIVMHDYLTGGFTANTSLAHYCR			Carbamidomethyl (C)[26], Oxidation (M)[8]		Mascot
3854.8718	3854.6453	-0.2265	-59	32	69	VTPQPGVPPEEAGAAVAAESSTGTWTTVWTDGLTSLDR					Mascot
3854.8718	3854.6453	-0.2265	-59	32	69	VTPQPGVPPEEAGAAVAAESSTGTWTTVWTDGLTSLDR	210	100			Mascot

Rank	Protein Name	Accession Nc	Protein MW	Protein PI	Pep. Count	Protein Score	Protein Score C. I. %	Total Ion Score	Total Ion C. I. %
6	Ribulose bisphosphate carboxylase large chain (Fragment) OS=Saururus cernuus GN=rbcL PE=3 SV=1	RBL_SAUCE	51663	6	22	845	100	670	100

Peptide Information

Calc. Mass	Obsrv. Mass	± da	± ppm	Start Seq.	End Seq.	Sequence	Ion Score	C. I. %	Modification	Rank	Result Type
830.4366	830.399	-0.0376	-45	431	437	EGNEIIR					Mascot
853.4236	853.3833	-0.0403	-47	179	185	AVYECLR					Mascot
898.41	898.3663	-0.0437	-49	297	303	NHGMHFR					Mascot
910.4451	910.4037	-0.0414	-45	179	185	AVYECLR			Carbamidomethyl (C)[5]		Mascot
912.4719	912.4191	-0.0528	-58	287	294	AMHAVIDR					Mascot
914.4049	914.3641	-0.0408	-45	297	303	NHGMHFR			Oxidation (M)[4]		Mascot
928.4669	928.4246	-0.0423	-46	287	294	AMHAVIDR			Oxidation (M)[2]		Mascot
962.4788	962.4345	-0.0443	-46	219	227	SQAETGEIK					Mascot
1021.5312	1021.4844	-0.0468	-46	24	32	DTDILAAFR	63	99.992			Mascot
1021.5312	1021.4844	-0.0468	-46	24	32	DTDILAAFR					Mascot
1116.583	1116.5306	-0.0524	-47	413	422	VALEACVQAR			Carbamidomethyl (C)[6]		Mascot
1170.5585	1170.5833	0.0248	21	295	303	QKNHGMHFR			Oxidation (M)[6]		Mascot
1187.6643	1187.6108	-0.0535	-45	277	286	DNGLLLHIHR					Mascot
1275.7307	1275.6705	-0.0602	-47	331	341	EITLGFVDLLR	92	100			Mascot
1275.7307	1275.6705	-0.0602	-47	331	341	EITLGFVDLLR					Mascot
1393.6522	1393.5815	-0.0707	-51	13	23	LTYYTPDYETK					Mascot
1451.6219	1451.551	-0.0709	-49	193	204	DDENVNSQPFMR					Mascot
1465.7216	1465.6815	-0.0401	-27	311	325	MSGGDHIHAGTVVGK					Mascot
1465.7546	1465.6815	-0.0731	-50	138	150	TFQGPPHGIQVER	89	100			Mascot
1472.6978	1472.6267	-0.0711	-48	455	466	EIKFEFEAMDTL					Mascot
1488.6926	1488.6224	-0.0702	-47	455	466	EIKFEFEAMDTL			Oxidation (M)[9]		Mascot
1502.8512	1502.7758	-0.0754	-50	156	168	YGRPLLGCTIKPK			Carbamidomethyl (C)[8]		Mascot
1546.7358	1546.6554	-0.0804	-52	442	454	WSPELAAACEVWK			Carbamidomethyl (C)[9]		Mascot
1794.8149	1794.7255	-0.0894	-50	228	243	GHYLNATAGTCEEMIK			Carbamidomethyl (C)[11]		Mascot
2050.0134	2049.8823	-0.1311	-64	311	330	MSGGDHIHAGTVVGKLEGER					Mascot
2169.9868	2169.8689	-0.1179	-54	186	204	GGLDFTKDDENVNSQPFMR					Mascot
2169.9868	2169.8689	-0.1179	-54	186	204	GGLDFTKDDENVNSQPFMR	104	100			Mascot
2185.9819	2185.8633	-0.1186	-54	186	204	GGLDFTKDDENVNSQPFMR			Oxidation (M)[18]		Mascot
3022.4548	3022.283	-0.1718	-57	250	276	ELGVPIVMHDYLTGGFTANTSLAHYCR	114	100	Carbamidomethyl (C)[26]		Mascot
3022.4548	3022.283	-0.1718	-57	250	276	ELGVPIVMHDYLTGGFTANTSLAHYCR			Carbamidomethyl (C)[26]		Mascot
3038.45	3038.2795	-0.1705	-56	250	276	ELGVPIVMHDYLTGGFTANTSLAHYCR			Carbamidomethyl (C)[26], Oxidation (M)[8]		Mascot
3854.8718	3854.6453	-0.2265	-59	33	70	VTPQPGVPPEEAGAAVAAESSTGTWTTVWTDGLTSLDR					Mascot
3854.8718	3854.6453	-0.2265	-59	33	70	VTPQPGVPPEEAGAAVAAESSTGTWTTVWTDGLTSLDR	210	100			Mascot

Rank	Protein Name	Accession Nc	Protein MW	Protein PI	Pep. Count	Protein Score	Protein Score C. I. %	Total Ion Score	Total Ion C. I. %
7	Ribulose bisphosphate carboxylase large chain OS=Ipomoea purpurea GN=rbcL PE=3 SV=1	RBL_IPOPU	53353.9	6.41	22	844	100	671	100

Peptide Information

Calc. Mass	Obsrv. Mass	± da	± ppm	Start Seq.	End Seq.	Sequence	Ion Score	C. I. %	Modification	Rank	Result Type
805.4818	805.4423	-0.0395	-49	140	146	IPTAYIK					Mascot
830.4366	830.399	-0.0376	-45	440	446	EGNEIIR					Mascot
853.4236	853.3833	-0.0403	-47	188	194	AVYECLR					Mascot
898.41	898.3663	-0.0437	-49	306	312	NHGMHFR					Mascot
910.4451	910.4037	-0.0414	-45	188	194	AVYECLR			Carbamidomethyl (C)[5]		Mascot
912.4719	912.4191	-0.0528	-58	296	303	AMHAVIDR					Mascot
914.4049	914.3641	-0.0408	-45	306	312	NHGMHFR			Oxidation (M)[4]		Mascot
928.4669	928.4246	-0.0423	-46	296	303	AMHAVIDR			Oxidation (M)[2]		Mascot
962.4788	962.4345	-0.0443	-46	228	236	SQAETGEIK					Mascot
1021.5312	1021.4844	-0.0468	-46	33	41	DTDILAAFR	63	99.992			Mascot
1021.5312	1021.4844	-0.0468	-46	33	41	DTDILAAFR					Mascot
1116.583	1116.5306	-0.0524	-47	422	431	VALEACVQAR			Carbamidomethyl (C)[6]		Mascot
1170.5585	1170.5833	0.0248	21	304	312	QKNHGMHFR			Oxidation (M)[6]		Mascot
1187.6643	1187.6108	-0.0535	-45	286	295	DNGLLLHIHR					Mascot
1275.7307	1275.6705	-0.0602	-47	340	350	EITLGFVDLLR	92	100			Mascot
1275.7307	1275.6705	-0.0602	-47	340	350	EITLGFVDLLR					Mascot
1451.6219	1451.551	-0.0709	-49	202	213	DDENVNSQPFMR					Mascot
1465.7546	1465.6815	-0.0731	-50	147	159	TFQGPPHGIQVER					Mascot
1465.7546	1465.6815	-0.0731	-50	147	159	TFQGPPHGIQVER	89	100			Mascot
1502.8512	1502.7758	-0.0754	-50	165	177	YGRPLLGCTIKPK			Carbamidomethyl (C)[8]		Mascot
1546.7358	1546.6554	-0.0804	-52	451	463	WSPELAAACEVWK			Carbamidomethyl (C)[9]		Mascot
1812.7714	1812.6749	-0.0965	-53	237	252	GHYLNATAGTCEEMMK			Carbamidomethyl (C)[11]		Mascot
1828.7662	1828.6735	-0.0927	-51	237	252	GHYLNATAGTCEEMMK			Carbamidomethyl (C)[11], Oxidation (M)[14]		Mascot
1968.8724	1968.9298	0.0574	29	237	253	GHYLNATAGTCEEMMKR			Carbamidomethyl (C)[11]		Mascot
1984.8673	1984.7522	-0.1151	-58	237	253	GHYLNATAGTCEEMMKR			Carbamidomethyl (C)[11], Oxidation (M)[14]		Mascot
2033.9572	2033.8898	-0.0674	-33	447	463	QACKWSPELAAACEVWK			Carbamidomethyl (C)[3,13]		Mascot
2169.9868	2169.8689	-0.1179	-54	195	213	GGLDFTKDDENVNSQPFMR					Mascot
2169.9868	2169.8689	-0.1179	-54	195	213	GGLDFTKDDENVNSQPFMR	104	100			Mascot
2185.9819	2185.8633	-0.1186	-54	195	213	GGLDFTKDDENVNSQPFMR			Oxidation (M)[18]		Mascot
2280.1506	2280.0625	-0.0881	-39	340	358	EITLGFVDLLRDDFVEQDR					Mascot
3022.4548	3022.283	-0.1718	-57	259	285	ELGVPIVMHDYLTGGFTANTSLAHYCR	114	100	Carbamidomethyl (C)[26]		Mascot
3022.4548	3022.283	-0.1718	-57	259	285	ELGVPIVMHDYLTGGFTANTSLAHYCR			Carbamidomethyl (C)[26]		Mascot
3038.45	3038.2795	-0.1705	-56	259	285	ELGVPIVMHDYLTGGFTANTSLAHYCR			Carbamidomethyl (C)[26], Oxidation (M)[8]		Mascot
3854.8718	3854.6453	-0.2265	-59	42	79	VTPQPGVPPEEAGAAVAAESSTGTWTTVWTDGLTSLDR					Mascot
3854.8718	3854.6453	-0.2265	-59	42	79	VTPQPGVPPEEAGAAVAAESSTGTWTTVWTDGLTSLDR	210	100			Mascot

Rank	Protein Name	Accession Nc	Protein MW	Protein PI	Pep. Count	Protein Score	Protein Score C. I. %	Total Ion Score	Total Ion C. I. %
8	Ribulose bisphosphate carboxylase large chain (Fragment) OS=Sanguinaria canadensis GN=rbcL PE=3 SV=	RBL_SANCA	48270.4	6.46	21	839	100	670	100

Peptide Information

Calc. Mass	Obsrv. Mass	± da	± ppm	Start Seq.	End Seq.	Sequence	Ion Score	C. I. %	Modification	Rank	Result Type
805.4929	805.4423	-0.0506	-63	1	8	VGFKAGVK					Mascot
830.4366	830.399	-0.0376	-45	430	436	EGNEIIR					Mascot
853.4236	853.3833	-0.0403	-47	178	184	AVYECLR					Mascot
898.41	898.3663	-0.0437	-49	296	302	NHGMHFR					Mascot
910.4451	910.4037	-0.0414	-45	178	184	AVYECLR			Carbamidomethyl (C)[5]		Mascot
912.4719	912.4191	-0.0528	-58	286	293	AMHAVIDR					Mascot
914.4049	914.3641	-0.0408	-45	296	302	NHGMHFR			Oxidation (M)[4]		Mascot
928.4669	928.4246	-0.0423	-46	286	293	AMHAVIDR			Oxidation (M)[2]		Mascot
1021.5312	1021.4844	-0.0468	-46	23	31	DTDILAAFR	63	99.992			Mascot
1021.5312	1021.4844	-0.0468	-46	23	31	DTDILAAFR					Mascot
1116.583	1116.5306	-0.0524	-47	412	421	VALEACVQAR			Carbamidomethyl (C)[6]		Mascot
1170.5585	1170.5833	0.0248	21	294	302	QKNHGMHFR			Oxidation (M)[6]		Mascot
1187.6643	1187.6108	-0.0535	-45	276	285	DNGLLLHIHR					Mascot
1275.7307	1275.6705	-0.0602	-47	330	340	EITLGFVDLLR	92	100			Mascot
1275.7307	1275.6705	-0.0602	-47	330	340	EITLGFVDLLR					Mascot
1393.6522	1393.5815	-0.0707	-51	12	22	LTYYTPDYETK					Mascot
1451.6219	1451.551	-0.0709	-49	192	203	DDENVNSQPFMR					Mascot
1465.7216	1465.6815	-0.0401	-27	310	324	MSGGDHIHAGTVVGK					Mascot
1465.7546	1465.6815	-0.0731	-50	137	149	TFQGPPHGIQVER	89	100			Mascot
1502.8512	1502.7758	-0.0754	-50	155	167	YGRPLLGCTIKPK			Carbamidomethyl (C)[8]		Mascot
1794.8149	1794.7255	-0.0894	-50	227	242	GHYLNATAGTCEEMIK			Carbamidomethyl (C)[11]		Mascot
2009.059	2008.9478	-0.1112	-55	330	346	EITLGFVDLLRDDFVEK					Mascot
2050.0134	2049.8823	-0.1311	-64	310	329	MSGGDHIHAGTVVGKLEGER					Mascot
2169.9868	2169.8689	-0.1179	-54	185	203	GGLDFTKDDENVNSQPFMR					Mascot
2169.9868	2169.8689	-0.1179	-54	185	203	GGLDFTKDDENVNSQPFMR	104	100			Mascot
2185.9819	2185.8633	-0.1186	-54	185	203	GGLDFTKDDENVNSQPFMR			Oxidation (M)[18]		Mascot
3022.4548	3022.283	-0.1718	-57	249	275	ELGVPIVMHDYLTGGFTANTSLAHYCR	114	100	Carbamidomethyl (C)[26]		Mascot
3022.4548	3022.283	-0.1718	-57	249	275	ELGVPIVMHDYLTGGFTANTSLAHYCR			Carbamidomethyl (C)[26]		Mascot
3038.45	3038.2795	-0.1705	-56	249	275	ELGVPIVMHDYLTGGFTANTSLAHYCR			Carbamidomethyl (C)[26], Oxidation (M)[8]		Mascot
3854.8718	3854.6453	-0.2265	-59	32	69	VTPQPGVPPEEAGAAVAAESSTGTWTTVWTDGLTSLDR					Mascot
3854.8718	3854.6453	-0.2265	-59	32	69	VTPQPGVPPEEAGAAVAAESSTGTWTTVWTDGLTSLDR	210	100			Mascot

Rank	Protein Name	Accession Nc	Protein MW	Protein PI	Pep. Count	Protein Score	Protein Score C. I. %	Total Ion Score	Total Ion C. I. %
9	Ribulose bisphosphate carboxylase large chain OS=Phaseolus vulgaris GN=rbcL PE=3 SV=1	RBL_PHAVU	52767.6	5.96	21	832	100	671	100

Peptide Information

Calc. Mass	Obsrv. Mass	± da	± ppm	Start Seq.	End Seq.	Sequence	Ion Score	C. I. %	Modification	Rank	Result Type
805.4818	805.4423	-0.0395	-49	140	146	IPTAYIK					Mascot
830.4366	830.399	-0.0376	-45	440	446	EGNEIIR					Mascot
853.4236	853.3833	-0.0403	-47	188	194	AVYECLR					Mascot
898.41	898.3663	-0.0437	-49	306	312	NHGMHFR					Mascot
910.4451	910.4037	-0.0414	-45	188	194	AVYECLR			Carbamidomethyl (C)[5]		Mascot
912.4719	912.4191	-0.0528	-58	296	303	AMHAVIDR					Mascot
914.4049	914.3641	-0.0408	-45	306	312	NHGMHFR			Oxidation (M)[4]		Mascot
928.4669	928.4246	-0.0423	-46	296	303	AMHAVIDR			Oxidation (M)[2]		Mascot
1021.5312	1021.4844	-0.0468	-46	33	41	DTDILAAFR	63	99.992			Mascot
1021.5312	1021.4844	-0.0468	-46	33	41	DTDILAAFR					Mascot
1116.583	1116.5306	-0.0524	-47	422	431	VALEACVQAR			Carbamidomethyl (C)[6]		Mascot
1170.5585	1170.5833	0.0248	21	304	312	QKNHGMHFR			Oxidation (M)[6]		Mascot
1187.6643	1187.6108	-0.0535	-45	286	295	DNGLLLHIHR					Mascot
1245.6433	1245.5757	-0.0676	-54	440	450	EGNEIIREASK					Mascot
1275.7307	1275.6705	-0.0602	-47	340	350	EITLGFVDLLR	92	100			Mascot
1275.7307	1275.6705	-0.0602	-47	340	350	EITLGFVDLLR					Mascot
1393.6522	1393.5815	-0.0707	-51	22	32	LTYYTPDYETK					Mascot
1451.6219	1451.551	-0.0709	-49	202	213	DDENVNSQPFMR					Mascot
1465.7546	1465.6815	-0.0731	-50	147	159	TFQGPPHGIQVER					Mascot
1465.7546	1465.6815	-0.0731	-50	147	159	TFQGPPHGIQVER	89	100			Mascot
1502.8512	1502.7758	-0.0754	-50	165	177	YGRPLLGCTIKPK			Carbamidomethyl (C)[8]		Mascot
1546.7358	1546.6554	-0.0804	-52	451	463	WSPELAAACEVWK			Carbamidomethyl (C)[9]		Mascot
1794.8149	1794.7255	-0.0894	-50	237	252	GHYLNATAGTCEEMIK			Carbamidomethyl (C)[11]		Mascot
2034.0363	2033.8898	-0.1465	-72	320	339	LSGGDHVHSGTVVGKLEGER					Mascot
2169.9868	2169.8689	-0.1179	-54	195	213	GGLDFTKDDENVNSQPFMR					Mascot
2169.9868	2169.8689	-0.1179	-54	195	213	GGLDFTKDDENVNSQPFMR	104	100			Mascot
2185.9819	2185.8633	-0.1186	-54	195	213	GGLDFTKDDENVNSQPFMR			Oxidation (M)[18]		Mascot
3022.4548	3022.283	-0.1718	-57	259	285	ELGVPIVMHDYLTGGFTANTSLAHYCR	114	100	Carbamidomethyl (C)[26]		Mascot
3022.4548	3022.283	-0.1718	-57	259	285	ELGVPIVMHDYLTGGFTANTSLAHYCR			Carbamidomethyl (C)[26]		Mascot
3038.45	3038.2795	-0.1705	-56	259	285	ELGVPIVMHDYLTGGFTANTSLAHYCR			Carbamidomethyl (C)[26], Oxidation (M)[8]		Mascot
3854.8718	3854.6453	-0.2265	-59	42	79	VTPQPGVPPEEAGAAVAAESSTGTWTTVWTDGLTSLDR					Mascot
3854.8718	3854.6453	-0.2265	-59	42	79	VTPQPGVPPEEAGAAVAAESSTGTWTTVWTDGLTSLDR	210	100			Mascot

Rank	Protein Name	Accession Nc	Protein MW	Protein PI	Pep. Count	Protein Score	Protein Score C. I. %	Total Ion Score	Total Ion C. I. %
10	Ribulose bisphosphate carboxylase large chain OS=Liquidambar styraciflua GN=rbcL PE=3 SV=1	RBL_LIQST	52625.5	6.04	21	829	100	670	100

Peptide Information

Calc. Mass	Obsrv. Mass	± da	± ppm	Start Seq.	End Seq.	Sequence	Ion Score	C. I. %	Modification	Rank	Result Type
830.4366	830.399	-0.0376	-45	440	446	EGNEIIR					Mascot
853.4236	853.3833	-0.0403	-47	188	194	AVYECLR					Mascot
898.41	898.3663	-0.0437	-49	306	312	NHGMHFR					Mascot
910.4451	910.4037	-0.0414	-45	188	194	AVYECLR			Carbamidomethyl (C)[5]		Mascot
912.4719	912.4191	-0.0528	-58	296	303	AMHAVIDR					Mascot
914.4049	914.3641	-0.0408	-45	306	312	NHGMHFR			Oxidation (M)[4]		Mascot
928.4669	928.4246	-0.0423	-46	296	303	AMHAVIDR			Oxidation (M)[2]		Mascot
1021.5312	1021.4844	-0.0468	-46	33	41	DTDILAAFR	63	99.992			Mascot
1021.5312	1021.4844	-0.0468	-46	33	41	DTDILAAFR					Mascot
1116.583	1116.5306	-0.0524	-47	422	431	VALEACVQAR			Carbamidomethyl (C)[6]		Mascot
1170.5585	1170.5833	0.0248	21	304	312	QKNHGMHFR			Oxidation (M)[6]		Mascot
1187.6643	1187.6108	-0.0535	-45	286	295	DNGLLLHIHR					Mascot
1245.6433	1245.5757	-0.0676	-54	440	450	EGNEIIREASK					Mascot
1275.7307	1275.6705	-0.0602	-47	340	350	EITLGFVDLLR	92	100			Mascot
1275.7307	1275.6705	-0.0602	-47	340	350	EITLGFVDLLR					Mascot
1451.6219	1451.551	-0.0709	-49	202	213	DDENVNSQPFMR					Mascot
1465.7216	1465.6815	-0.0401	-27	320	334	MSGGDHIHAGTVVGK					Mascot
1465.7546	1465.6815	-0.0731	-50	147	159	TFQGPPHGIQVER	89	100			Mascot
1472.6978	1472.6267	-0.0711	-48	464	475	EIKFEFEAMDTL					Mascot
1488.6926	1488.6224	-0.0702	-47	464	475	EIKFEFEAMDTL			Oxidation (M)[9]		Mascot
1502.8512	1502.7758	-0.0754	-50	165	177	YGRPLLGCTIKPK			Carbamidomethyl (C)[8]		Mascot
1546.7358	1546.6554	-0.0804	-52	451	463	WSPELAAACEVWK			Carbamidomethyl (C)[9]		Mascot
1794.8149	1794.7255	-0.0894	-50	237	252	GHYLNATAGTCEEMIK			Carbamidomethyl (C)[11]		Mascot
2050.0134	2049.8823	-0.1311	-64	320	339	MSGGDHIHAGTVVGKLEGER					Mascot
2169.9868	2169.8689	-0.1179	-54	195	213	GGLDFTKDDENVNSQPFMR					Mascot
2169.9868	2169.8689	-0.1179	-54	195	213	GGLDFTKDDENVNSQPFMR	104	100			Mascot
2185.9819	2185.8633	-0.1186	-54	195	213	GGLDFTKDDENVNSQPFMR			Oxidation (M)[18]		Mascot
3022.4548	3022.283	-0.1718	-57	259	285	ELGVPIVMHDYLTGGFTANTSLAHYCR	114	100	Carbamidomethyl (C)[26]		Mascot
3022.4548	3022.283	-0.1718	-57	259	285	ELGVPIVMHDYLTGGFTANTSLAHYCR			Carbamidomethyl (C)[26]		Mascot
3038.45	3038.2795	-0.1705	-56	259	285	ELGVPIVMHDYLTGGFTANTSLAHYCR			Carbamidomethyl (C)[26], Oxidation (M)[8]		Mascot
3854.8718	3854.6453	-0.2265	-59	42	79	VTPQPGVPPEEAGAAVAAESSTGTWTTVWTDGLTSLDR					Mascot
3854.8718	3854.6453	-0.2265	-59	42	79	VTPQPGVPPEEAGAAVAAESSTGTWTTVWTDGLTSLDR	210	100			Mascot

Gel Idx/Pos	49/B22	Instr./Gel Origin	BA2060/131017A	Process Sta	Analysis Succeeded
Plate [#] Name	[1] 31496	Instrument Sample Name		Spectra	8

Rank	Protein Name	Accession No	Protein MW	Protein PI	Pep. Count	Protein Score	Protein Score C. I. %	Total Ion Score	Total Ion C. I. %
1	Ribulose bisphosphate carboxylase large chain OS=Glycine max GN=rbcL PE=1 SV=3	RBL_SOYBN	52576.4	6	25	961	100	752	100

Peptide Information

Calc. Mass	Obsrv. Mass	± da	± ppm	Start Seq.	End Seq.	Sequence	Ion Score	C. I. %	Modification	Rank	Result Type
805.4818	805.4519	-0.0299	-37	140	146	IPTAYIK					Mascot
830.4366	830.4059	-0.0307	-37	440	446	EGNEIIR					Mascot
898.41	898.3764	-0.0336	-37	306	312	NHGMHFR					Mascot
910.4451	910.4108	-0.0343	-38	188	194	AVYECLR			Carbamidomethyl (C)[5]		Mascot
912.4719	912.422	-0.0499	-55	296	303	AMHAVIDR					Mascot
914.4049	914.373	-0.0319	-35	306	312	NHGMHFR			Oxidation (M)[4]		Mascot
928.4669	928.4342	-0.0327	-35	296	303	AMHAVIDR			Oxidation (M)[2]		Mascot
962.4788	962.4403	-0.0385	-40	228	236	SQAETGEIK					Mascot
1021.5312	1021.4927	-0.0385	-38	33	41	DTDILAAFR					Mascot
1116.583	1116.5388	-0.0442	-40	422	431	VALEACVQAR			Carbamidomethyl (C)[6]		Mascot
1170.5585	1170.5935	0.035	30	304	312	QKNHGMHFR			Oxidation (M)[6]		Mascot
1187.6643	1187.6178	-0.0465	-39	286	295	DNGLLLHIHR					Mascot
1245.6337	1245.5841	-0.0496	-40	218	227	FLFCAEAIFK			Carbamidomethyl (C)[4]		Mascot
1245.6337	1245.5841	-0.0496	-40	218	227	FLFCAEAIFK	60	99.987	Carbamidomethyl (C)[4]		Mascot
1275.7307	1275.6815	-0.0492	-39	340	350	EITLGFVDLLR	85	100			Mascot
1275.7307	1275.6815	-0.0492	-39	340	350	EITLGFVDLLR					Mascot
1393.6522	1393.5959	-0.0563	-40	22	32	LTYYTPDYETK					Mascot
1433.7495	1433.6858	-0.0637	-44	320	334	LSGGDHVHAGTVVGK					Mascot
1451.6219	1451.562	-0.0599	-41	202	213	DDENVNSQPFMR					Mascot
1465.7546	1465.6945	-0.0601	-41	147	159	TFQGPPHGIQVER	96	100			Mascot
1465.7546	1465.6945	-0.0601	-41	147	159	TFQGPPHGIQVER					Mascot
1472.6978	1472.6348	-0.063	-43	464	475	EIKFEFEAMDTL					Mascot
1488.6926	1488.6345	-0.0581	-39	464	475	EIKFEFEAMDTL			Oxidation (M)[9]		Mascot
1502.8512	1502.787	-0.0642	-43	165	177	YGRPLLGCTIKPK			Carbamidomethyl (C)[8]		Mascot
1546.7358	1546.6692	-0.0666	-43	451	463	WSPELAAACEVWK			Carbamidomethyl (C)[9]		Mascot
1812.7714	1812.6936	-0.0778	-43	237	252	GHYLNATAGTCEEMMK			Carbamidomethyl (C)[11]		Mascot
1828.7662	1828.682	-0.0842	-46	237	252	GHYLNATAGTCEEMMK			Carbamidomethyl (C)[11], Oxidation (M)[14]		Mascot
1984.8673	1984.7759	-0.0914	-46	237	253	GHYLNATAGTCEEMMKR			Carbamidomethyl (C)[11], Oxidation (M)[14]		Mascot
2000.8623	2000.7673	-0.095	-47	237	253	GHYLNATAGTCEEMMKR			Carbamidomethyl (C)[11], Oxidation (M)[14,15]		Mascot
2009.059	2008.9644	-0.0946	-47	340	356	EITLGFVDLLRDDFVEK					Mascot
2169.9868	2169.8865	-0.1003	-46	195	213	GGLDFTKDDENVNSQPFMR					Mascot
2169.9868	2169.8865	-0.1003	-46	195	213	GGLDFTKDDENVNSQPFMR	130	100			Mascot
2185.9819	2185.8806	-0.1013	-46	195	213	GGLDFTKDDENVNSQPFMR			Oxidation (M)[18]		Mascot
3022.4548	3022.2957	-0.1591	-53	259	285	ELGVPIVMHDYLTGGFTANTSLAHYCR			Carbamidomethyl (C)[26]		Mascot
3022.4548	3022.2957	-0.1591	-53	259	285	ELGVPIVMHDYLTGGFTANTSLAHYCR	146	100	Carbamidomethyl (C)[26]		Mascot
3038.45	3038.301	-0.149	-49	259	285	ELGVPIVMHDYLTGGFTANTSLAHYCR			Carbamidomethyl (C)[26], Oxidation (M)[8]		Mascot
3854.8718	3854.666	-0.2058	-53	42	79	VTPQPGVPPEEAGAAVAAESSTGTWTTVWTDGLTSLDR	238	100			Mascot
3854.8718	3854.666	-0.2058	-53	42	79	VTPQPGVPPEEAGAAVAAESSTGTWTTVWTDGLTSLDR					Mascot

Rank	Protein Name	Accession No	Protein MW	Protein PI	Pep. Count	Protein Score	Protein Score C. I. %	Total Ion Score	Total Ion C. I. %
2	Ribulose bisphosphate carboxylase large chain (Fragment) OS=Geum quellyon GN=rbcL PE=3 SV=1	RBL_GEUQU	50910.8	6.23	22	932	100	752	100

Peptide Information

Calc. Mass	Obsrv. Mass	± da	± ppm	Start Seq.	End Seq.	Sequence	Ion Score	C. I. %	Modification	Rank	Result Type
805.4929	805.4519	-0.041	-51	1	8	VGFKAGVK					Mascot
898.41	898.3764	-0.0336	-37	296	302	NHGMHFR					Mascot
910.4451	910.4108	-0.0343	-38	178	184	AVYECLR			Carbamidomethyl (C)[5]		Mascot
912.4719	912.422	-0.0499	-55	286	293	AMHAVIDR					Mascot
914.4049	914.373	-0.0319	-35	296	302	NHGMHFR			Oxidation (M)[4]		Mascot
928.4669	928.4342	-0.0327	-35	286	293	AMHAVIDR			Oxidation (M)[2]		Mascot
1021.5312	1021.4927	-0.0385	-38	23	31	DTDILAAFR					Mascot
1116.583	1116.5388	-0.0442	-40	412	421	VALEACVQAR			Carbamidomethyl (C)[6]		Mascot
1170.5585	1170.5935	0.035	30	294	302	QKNHGMHFR			Oxidation (M)[6]		Mascot
1187.6643	1187.6178	-0.0465	-39	276	285	DNGLLLHIHR					Mascot
1245.6337	1245.5841	-0.0496	-40	208	217	FLFCAEALFK			Carbamidomethyl (C)[4]		Mascot
1245.6337	1245.5841	-0.0496	-40	208	217	FLFCAEALFK	60	99.987	Carbamidomethyl (C)[4]		Mascot
1275.7307	1275.6815	-0.0492	-39	330	340	EITLGFVDLLR	85	100			Mascot
1275.7307	1275.6815	-0.0492	-39	330	340	EITLGFVDLLR					Mascot
1393.6522	1393.5959	-0.0563	-40	12	22	LTYYTPDYETK					Mascot
1451.6219	1451.562	-0.0599	-41	192	203	DDENVNSQPFMR					Mascot
1465.7216	1465.6945	-0.0271	-18	310	324	MSGGDHIHAGTVVGK					Mascot
1465.7546	1465.6945	-0.0601	-41	137	149	TFQGPPHGIQVER	96	100			Mascot
1502.8512	1502.787	-0.0642	-43	155	167	YGRPLLGCTIKPK			Carbamidomethyl (C)[8]		Mascot
1546.7358	1546.6692	-0.0666	-43	441	453	WSPELAAACEVWK			Carbamidomethyl (C)[9]		Mascot
1812.7714	1812.6936	-0.0778	-43	227	242	GHYLNATAGTCEEMMK			Carbamidomethyl (C)[11]		Mascot
1828.7662	1828.682	-0.0842	-46	227	242	GHYLNATAGTCEEMMK			Carbamidomethyl (C)[11], Oxidation (M)[14]		Mascot
1984.8673	1984.7759	-0.0914	-46	227	243	GHYLNATAGTCEEMMKR			Carbamidomethyl (C)[11], Oxidation (M)[14]		Mascot
2000.8623	2000.7673	-0.095	-47	227	243	GHYLNATAGTCEEMMKR			Carbamidomethyl (C)[11], Oxidation (M)[14,15]		Mascot
2050.0134	2049.9011	-0.1123	-55	310	329	MSGGDHIHAGTVVGKLEGER					Mascot
2169.9868	2169.8865	-0.1003	-46	185	203	GGLDFTKDDENVNSQPFMR					Mascot
2169.9868	2169.8865	-0.1003	-46	185	203	GGLDFTKDDENVNSQPFMR	130	100			Mascot
2185.9819	2185.8806	-0.1013	-46	185	203	GGLDFTKDDENVNSQPFMR			Oxidation (M)[18]		Mascot
3022.4548	3022.2957	-0.1591	-53	249	275	ELGVPIVMHDYLTGGFTANTSLAHYCR			Carbamidomethyl (C)[26]		Mascot
3022.4548	3022.2957	-0.1591	-53	249	275	ELGVPIVMHDYLTGGFTANTSLAHYCR	146	100	Carbamidomethyl (C)[26]		Mascot
3038.45	3038.301	-0.149	-49	249	275	ELGVPIVMHDYLTGGFTANTSLAHYCR			Carbamidomethyl (C)[26], Oxidation (M)[8]		Mascot
3854.8718	3854.666	-0.2058	-53	32	69	VTPQPGVPPEEAGAAVAAESSTGTWTTVWTDGLTSLDR	238	100			Mascot
3854.8718	3854.666	-0.2058	-53	32	69	VTPQPGVPPEEAGAAVAAESSTGTWTTVWTDGLTSLDR					Mascot

Rank	Protein Name	Accession No	Protein MW	Protein PI	Pep. Count	Protein Score	Protein Score C. I. %	Total Ion Score	Total Ion C. I. %
3	Ribulose bisphosphate carboxylase large chain OS=Sesbania sesban GN=rbcL PE=3 SV=1	RBL_SESSE	52358.4	6.09	19	886	100	752	100

Peptide Information

Calc. Mass	Obsrv. Mass	± da	± ppm	Start Seq.	End Seq.	Sequence	Ion Score	C. I. %	Modification	Rank	Result Type
830.4366	830.4059	-0.0307	-37	440	446	EGNEIIR					Mascot
898.41	898.3764	-0.0336	-37	306	312	NHGMHFR					Mascot
910.4451	910.4108	-0.0343	-38	188	194	AVYECLR			Carbamidomethyl (C)[5]		Mascot
912.4719	912.422	-0.0499	-55	296	303	AMHAVIDR					Mascot
914.4049	914.373	-0.0319	-35	306	312	NHGMHFR			Oxidation (M)[4]		Mascot
928.4669	928.4342	-0.0327	-35	296	303	AMHAVIDR			Oxidation (M)[2]		Mascot
962.4788	962.4403	-0.0385	-40	228	236	SQAETGEIK					Mascot
1021.5312	1021.4927	-0.0385	-38	33	41	DTDILAAFR					Mascot
1116.583	1116.5388	-0.0442	-40	422	431	VALEACVQAR			Carbamidomethyl (C)[6]		Mascot
1170.5585	1170.5935	0.035	30	304	312	QKNHGMHFR			Oxidation (M)[6]		Mascot
1187.6643	1187.6178	-0.0465	-39	286	295	DNGLLLHIHR					Mascot
1245.6337	1245.5841	-0.0496	-40	218	227	FLFCAEAIFK			Carbamidomethyl (C)[4]		Mascot
1245.6337	1245.5841	-0.0496	-40	218	227	FLFCAEAIFK	60	99.987	Carbamidomethyl (C)[4]		Mascot
1275.7307	1275.6815	-0.0492	-39	340	350	EITLGFVDLLR	85	100			Mascot
1275.7307	1275.6815	-0.0492	-39	340	350	EITLGFVDLLR					Mascot
1393.6522	1393.5959	-0.0563	-40	22	32	LTYYTPDYETK					Mascot
1451.6219	1451.562	-0.0599	-41	202	213	DDENVNSQPFMR					Mascot
1465.7546	1465.6945	-0.0601	-41	147	159	TFQGPPHGIQVER	96	100			Mascot
1465.7546	1465.6945	-0.0601	-41	147	159	TFQGPPHGIQVER					Mascot
1502.8512	1502.787	-0.0642	-43	165	177	YGRPLLGCTIKPK			Carbamidomethyl (C)[8]		Mascot
1546.7358	1546.6692	-0.0666	-43	451	463	WSPELAAACEVWK			Carbamidomethyl (C)[9]		Mascot
2169.9868	2169.8865	-0.1003	-46	195	213	GGLDFTKDDENVNSQPFMR					Mascot
2169.9868	2169.8865	-0.1003	-46	195	213	GGLDFTKDDENVNSQPFMR	130	100			Mascot
2185.9819	2185.8806	-0.1013	-46	195	213	GGLDFTKDDENVNSQPFMR			Oxidation (M)[18]		Mascot
3022.4548	3022.2957	-0.1591	-53	259	285	ELGVPIVMHDYLTGGFTANTSLAHYCR			Carbamidomethyl (C)[26]		Mascot
3022.4548	3022.2957	-0.1591	-53	259	285	ELGVPIVMHDYLTGGFTANTSLAHYCR	146	100	Carbamidomethyl (C)[26]		Mascot
3038.45	3038.301	-0.149	-49	259	285	ELGVPIVMHDYLTGGFTANTSLAHYCR			Carbamidomethyl (C)[26], Oxidation (M)[8]		Mascot
3854.8718	3854.666	-0.2058	-53	42	79	VTPQPGVPPEEAGAAVAAESSTGTWTTVWTDGLTSLDR	238	100			Mascot
3854.8718	3854.666	-0.2058	-53	42	79	VTPQPGVPPEEAGAAVAAESSTGTWTTVWTDGLTSLDR					Mascot

Rank	Protein Name	Accession No	Protein MW	Protein PI	Pep. Count	Protein Score	Protein Score C. I. %	Total Ion Score	Total Ion C. I. %
4	Ribulose bisphosphate carboxylase large chain OS=Lactuca sativa GN=rbcL PE=3 SV=2	RBL_LACSA	52861.6	5.91	18	873	100	754	100

Peptide Information

Calc. Mass	Obsrv. Mass	± da	± ppm	Start Seq.	End Seq.	Sequence	Ion Score	C. I. %	Modification	Rank	Result Type
910.4451	910.4108	-0.0343	-38	188	194	AVYECLR			Carbamidomethyl (C)[5]		Mascot
912.4719	912.422	-0.0499	-55	296	303	AMHAVIDR					Mascot
928.4669	928.4342	-0.0327	-35	296	303	AMHAVIDR			Oxidation (M)[2]		Mascot
962.4788	962.4403	-0.0385	-40	228	236	SQAETGEIK					Mascot
1021.5312	1021.4927	-0.0385	-38	33	41	DTDILAAFR					Mascot
1116.583	1116.5388	-0.0442	-40	422	431	VALEACVQAR			Carbamidomethyl (C)[6]		Mascot
1187.6643	1187.6178	-0.0465	-39	286	295	DNGLLLHIHR					Mascot
1245.6337	1245.5841	-0.0496	-40	218	227	FLFCAEAIFK			Carbamidomethyl (C)[4]		Mascot
1245.6337	1245.5841	-0.0496	-40	218	227	FLFCAEAIFK	60	99.987	Carbamidomethyl (C)[4]		Mascot
1275.7307	1275.6815	-0.0492	-39	340	350	EITLGFVDLLR	85	100			Mascot
1275.7307	1275.6815	-0.0492	-39	340	350	EITLGFVDLLR					Mascot
1451.6219	1451.562	-0.0599	-41	202	213	DDENVNSQPFMR					Mascot
1465.7546	1465.6945	-0.0601	-41	147	159	TFQGPPHGIQVER	96	100			Mascot
1465.7546	1465.6945	-0.0601	-41	147	159	TFQGPPHGIQVER					Mascot
1497.7114	1497.6539	-0.0575	-38	320	334	MSGGDHIHSGTVVGK			Oxidation (M)[1]		Mascot
1502.8512	1502.787	-0.0642	-43	165	177	YGRPLLGCTIKPK			Carbamidomethyl (C)[8]		Mascot
1546.7358	1546.6692	-0.0666	-43	451	463	WSPELAAACEVWK			Carbamidomethyl (C)[9]		Mascot
1812.7714	1812.6936	-0.0778	-43	237	252	GHYLNATAGTCEEMMK			Carbamidomethyl (C)[11]		Mascot
1828.7662	1828.682	-0.0842	-46	237	252	GHYLNATAGTCEEMMK			Carbamidomethyl (C)[11], Oxidation (M)[14]		Mascot
1984.8673	1984.7759	-0.0914	-46	237	253	GHYLNATAGTCEEMMKR			Carbamidomethyl (C)[11], Oxidation (M)[14]		Mascot
2000.8623	2000.7673	-0.095	-47	237	253	GHYLNATAGTCEEMMKR			Carbamidomethyl (C)[11], Oxidation (M)[14,15]		Mascot
2169.9868	2169.8865	-0.1003	-46	195	213	GGLDFTKDDENVNSQPFMR					Mascot
2169.9868	2169.8865	-0.1003	-46	195	213	GGLDFTKDDENVNSQPFMR	130	100			Mascot
2185.9819	2185.8806	-0.1013	-46	195	213	GGLDFTKDDENVNSQPFMR			Oxidation (M)[18]		Mascot
3022.4548	3022.2957	-0.1591	-53	259	285	ELGVPIVMHDYLTGGFTANTSLAHYCR			Carbamidomethyl (C)[26]		Mascot
3022.4548	3022.2957	-0.1591	-53	259	285	ELGVPIVMHDYLTGGFTANTSLAHYCR	146	100	Carbamidomethyl (C)[26]		Mascot
3038.45	3038.301	-0.149	-49	259	285	ELGVPIVMHDYLTGGFTANTSLAHYCR			Carbamidomethyl (C)[26], Oxidation (M)[8]		Mascot
3854.8718	3854.666	-0.2058	-53	42	79	VTPQPGVPPEEAGAAVAAESSTGTWTTVWTDGLTSLDR	238	100			Mascot
3854.8718	3854.666	-0.2058	-53	42	79	VTPQPGVPPEEAGAAVAAESSTGTWTTVWTDGLTSLDR					Mascot

Rank	Protein Name	Accession No	Protein MW	Protein PI	Pep. Count	Protein Score	Protein Score C. I. %	Total Ion Score	Total Ion C. I. %
5	Ribulose bisphosphate carboxylase large chain OS=Vitis vinifera GN=rbcL PE=3 SV=2	RBL_VITVI	52485.5	6.33	18	872	100	754	100

Peptide Information

Calc. Mass	Obsrv. Mass	± da	± ppm	Start Seq.	End Seq.	Sequence	Ion Score	C. I. %	Modification	Rank	Result Type
830.4366	830.4059	-0.0307	-37	440	446	EGNEIIR					Mascot
898.41	898.3764	-0.0336	-37	306	312	NHGMHFR					Mascot
910.4451	910.4108	-0.0343	-38	188	194	AVYECLR			Carbamidomethyl (C)[5]		Mascot
912.4719	912.422	-0.0499	-55	296	303	AMHAVIDR					Mascot
914.4049	914.373	-0.0319	-35	306	312	NHGMHFR			Oxidation (M)[4]		Mascot
928.4669	928.4342	-0.0327	-35	296	303	AMHAVIDR			Oxidation (M)[2]		Mascot
962.4788	962.4403	-0.0385	-40	228	236	SQAETGEIK					Mascot
1116.583	1116.5388	-0.0442	-40	422	431	VALEACVQAR			Carbamidomethyl (C)[6]		Mascot
1170.5585	1170.5935	0.035	30	304	312	QKNHGMHFR			Oxidation (M)[6]		Mascot
1187.6378	1187.6178	-0.02	-17	440	450	EGNEIIRAASK					Mascot
1245.6337	1245.5841	-0.0496	-40	218	227	FLFCAEAIFK			Carbamidomethyl (C)[4]		Mascot
1245.6337	1245.5841	-0.0496	-40	218	227	FLFCAEAIFK	60	99.987	Carbamidomethyl (C)[4]		Mascot
1275.7307	1275.6815	-0.0492	-39	340	350	EITLGFVDLLR	85	100			Mascot
1275.7307	1275.6815	-0.0492	-39	340	350	EITLGFVDLLR					Mascot
1451.6219	1451.562	-0.0599	-41	202	213	DDENVNSQPFMR					Mascot
1465.7546	1465.6945	-0.0601	-41	147	159	TFQGPPHGIQVER	96	100			Mascot
1465.7546	1465.6945	-0.0601	-41	147	159	TFQGPPHGIQVER					Mascot
1502.8512	1502.787	-0.0642	-43	165	177	YGRPLLGCTIKPK			Carbamidomethyl (C)[8]		Mascot
1546.7358	1546.6692	-0.0666	-43	451	463	WSPELAAACEVWK			Carbamidomethyl (C)[9]		Mascot
2009.059	2008.9644	-0.0946	-47	340	356	EITLGFVDLLRDDFVEK					Mascot
2169.9868	2169.8865	-0.1003	-46	195	213	GGLDFTKDDENVNSQPFMR					Mascot
2169.9868	2169.8865	-0.1003	-46	195	213	GGLDFTKDDENVNSQPFMR	130	100			Mascot
2185.9819	2185.8806	-0.1013	-46	195	213	GGLDFTKDDENVNSQPFMR			Oxidation (M)[18]		Mascot
3022.4548	3022.2957	-0.1591	-53	259	285	ELGVPIVMHDYLTGGFTANTSLAHYCR			Carbamidomethyl (C)[26]		Mascot
3022.4548	3022.2957	-0.1591	-53	259	285	ELGVPIVMHDYLTGGFTANTSLAHYCR	146	100	Carbamidomethyl (C)[26]		Mascot
3038.45	3038.301	-0.149	-49	259	285	ELGVPIVMHDYLTGGFTANTSLAHYCR			Carbamidomethyl (C)[26], Oxidation (M)[8]		Mascot
3854.8718	3854.666	-0.2058	-53	42	79	VTPQPGVPPEEAGAAVAAESSTGTWTTVWTDGLTSLDR	238	100			Mascot
3854.8718	3854.666	-0.2058	-53	42	79	VTPQPGVPPEEAGAAVAAESSTGTWTTVWTDGLTSLDR					Mascot

Rank	Protein Name	Accession No	Protein MW	Protein PI	Pep. Count	Protein Score	Protein Score C. I. %	Total Ion Score	Total Ion C. I. %
6	Ribulose bisphosphate carboxylase large chain (Fragment) OS=Prunus domestica GN=rbcL PE=3 SV=1	RBL_PRUDO	50914.6	6.23	22	868	100	692	100

Peptide Information

Calc. Mass	Obsrv. Mass	± da	± ppm	Start Seq.	End Seq.	Sequence	Ion Score	C. I. %	Modification	Rank	Result Type	
805.4929	805.4519	-0.041	-51	1	8	VGFKAGVK					Mascot	
830.4366	830.4059	-0.0307	-37	430	436	EGNEIIR					Mascot	
898.41	898.3764	-0.0336	-37	296	302	NHGMHFR					Mascot	
910.4451	910.4108	-0.0343	-38	178	184	AVYECLR			Carbamidomethyl (C)[5]		Mascot	
912.4719	912.422	-0.0499	-55	286	293	AMHAVIDR					Mascot	
914.4049	914.373	-0.0319	-35	296	302	NHGMHFR			Oxidation (M)[4]		Mascot	
928.4669	928.4342	-0.0327	-35	286	293	AMHAVIDR			Oxidation (M)[2]		Mascot	
1021.5312	1021.4927	-0.0385	-38	23	31	DTDILAAFR					Mascot	
1116.583	1116.5388	-0.0442	-40	412	421	VALEACVQAR			Carbamidomethyl (C)[6]		Mascot	
1170.5585	1170.5935	0.035	30	294	302	QKNHGMHFR			Oxidation (M)[6]		Mascot	
1187.6643	1187.6178	-0.0465	-39	276	285	DNGLLLHIHR					Mascot	
1245.6433	1245.5841	-0.0592	-48	430	440	EGNEIIREASK					Mascot	Mascot
1245.6433	1245.5841	-0.0592	-48	430	440	EGNEIIREASK					Mascot	
1275.7307	1275.6815	-0.0492	-39	330	340	EITLGFVDLLR	85	100			Mascot	
1275.7307	1275.6815	-0.0492	-39	330	340	EITLGFVDLLR					Mascot	
1393.6522	1393.5959	-0.0563	-40	12	22	LTYYTPDYETK					Mascot	
1451.6219	1451.562	-0.0599	-41	192	203	DDENVNSQPFMR					Mascot	
1465.7216	1465.6945	-0.0271	-18	310	324	MSGGDHIHAGTVVGK					Mascot	
1465.7546	1465.6945	-0.0601	-41	137	149	TFQGPPHGIQVER	96	100			Mascot	
1502.8512	1502.787	-0.0642	-43	155	167	YGRPLLGCTIKPK			Carbamidomethyl (C)[8]		Mascot	
1546.7358	1546.6692	-0.0666	-43	441	453	WSPELAAACEVWK			Carbamidomethyl (C)[9]		Mascot	
2009.059	2008.9644	-0.0946	-47	330	346	EITLGFVDLLRDDFVEK					Mascot	
2050.0134	2049.9011	-0.1123	-55	310	329	MSGGDHIHAGTVVGKLEGER					Mascot	
2169.9868	2169.8865	-0.1003	-46	185	203	GGLDFTKDDENVNSQPFMR					Mascot	
2169.9868	2169.8865	-0.1003	-46	185	203	GGLDFTKDDENVNSQPFMR	130	100			Mascot	
2185.9819	2185.8806	-0.1013	-46	185	203	GGLDFTKDDENVNSQPFMR			Oxidation (M)[18]		Mascot	
3022.4548	3022.2957	-0.1591	-53	249	275	ELGVPIVMHDYLTGGFTANTSLAHYCR			Carbamidomethyl (C)[26]		Mascot	
3022.4548	3022.2957	-0.1591	-53	249	275	ELGVPIVMHDYLTGGFTANTSLAHYCR	146	100	Carbamidomethyl (C)[26]		Mascot	
3038.45	3038.301	-0.149	-49	249	275	ELGVPIVMHDYLTGGFTANTSLAHYCR			Carbamidomethyl (C)[26], Oxidation (M)[8]		Mascot	
3854.8718	3854.666	-0.2058	-53	32	69	VTPQPGVPPEEAGAAVAAESSTGTWTTVWTDGLTSLDR	238	100			Mascot	
3854.8718	3854.666	-0.2058	-53	32	69	VTPQPGVPPEEAGAAVAAESSTGTWTTVWTDGLTSLDR					Mascot	

Rank	Protein Name	Accession No	Protein MW	Protein PI	Pep. Count	Protein Score	Protein Score C. I. %	Total Ion Score	Total Ion C. I. %
7	Ribulose bisphosphate carboxylase large chain (Fragment) OS=Oxalis dillenii GN=rbcL PE=3 SV=2	RBL_OXADI	51653.9	5.91	17	867	100	751	100

Peptide Information

Calc. Mass	Obsrv. Mass	± da	± ppm	Start Seq.	End Seq.	Sequence	Ion Score	C. I. %	Modification	Rank	Result Type
830.4366	830.4059	-0.0307	-37	431	437	EGNEIIR					Mascot
910.4451	910.4108	-0.0343	-38	179	185	AVYECLR			Carbamidomethyl (C)[5]		Mascot
912.4719	912.422	-0.0499	-55	287	294	AMHAVIDR					Mascot
928.4669	928.4342	-0.0327	-35	287	294	AMHAVIDR			Oxidation (M)[2]		Mascot
1021.5312	1021.4927	-0.0385	-38	24	32	DTDILAAFR					Mascot
1116.583	1116.5388	-0.0442	-40	413	422	VALEACVQAR			Carbamidomethyl (C)[6]		Mascot
1187.6643	1187.6178	-0.0465	-39	277	286	DNGLLLHIHR					Mascot
1245.6337	1245.5841	-0.0496	-40	209	218	FLFCAEAIFK			Carbamidomethyl (C)[4]		Mascot
1245.6337	1245.5841	-0.0496	-40	209	218	FLFCAEAIFK	60	99.987	Carbamidomethyl (C)[4]		Mascot
1275.7307	1275.6815	-0.0492	-39	331	341	EITLGFVDLLR	85	100			Mascot
1275.7307	1275.6815	-0.0492	-39	331	341	EITLGFVDLLR					Mascot
1451.6219	1451.562	-0.0599	-41	193	204	DDENVNSQPFMR					Mascot
1465.7546	1465.6945	-0.0601	-41	138	150	TFQGPPHGIQVER	96	100			Mascot
1465.7546	1465.6945	-0.0601	-41	138	150	TFQGPPHGIQVER					Mascot
1497.7114	1497.6539	-0.0575	-38	311	325	MSGGDHIHSGTVVGK			Oxidation (M)[1]		Mascot
1502.8512	1502.787	-0.0642	-43	156	168	YGRPLLGCTIKPK			Carbamidomethyl (C)[8]		Mascot
1546.7358	1546.6692	-0.0666	-43	442	454	WSPELAAACEVWK			Carbamidomethyl (C)[9]		Mascot
	1546.6692										Mascot
2009.059	2008.9644	-0.0946	-47	331	347	EITLGFVDLLRDDFVEK					Mascot
2169.9868	2169.8865	-0.1003	-46	186	204	GGLDFTKDDENVNSQPFMR					Mascot
2169.9868	2169.8865	-0.1003	-46	186	204	GGLDFTKDDENVNSQPFMR	130	100			Mascot
2185.9819	2185.8806	-0.1013	-46	186	204	GGLDFTKDDENVNSQPFMR			Oxidation (M)[18]		Mascot
3022.4548	3022.2957	-0.1591	-53	250	276	ELGVPIVMHDYLTGGFTANTSLAHYCR			Carbamidomethyl (C)[26]		Mascot
3022.4548	3022.2957	-0.1591	-53	250	276	ELGVPIVMHDYLTGGFTANTSLAHYCR	146	100	Carbamidomethyl (C)[26]		Mascot
3038.45	3038.301	-0.149	-49	250	276	ELGVPIVMHDYLTGGFTANTSLAHYCR			Carbamidomethyl (C)[26], Oxidation (M)[8]		Mascot
3854.8718	3854.666	-0.2058	-53	33	70	VTPQPGVPPEEAGAAVAAESSTGTWTTVWTDGLTSLDR	238	100			Mascot
3854.8718	3854.666	-0.2058	-53	33	70	VTPQPGVPPEEAGAAVAAESSTGTWTTVWTDGLTSLDR					Mascot

Rank	Protein Name	Accession No	Protein MW	Protein PI	Pep. Count	Protein Score	Protein Score C. I. %	Total Ion Score	Total Ion C. I. %
8	Ribulose bisphosphate carboxylase large chain OS=Ipomoea purpurea GN=rbcL PE=3 SV=1	RBL_IPOPU	53353.9	6.41	22	863	100	694	100

Peptide Information

Calc. Mass	Obsrv. Mass	± da	± ppm	Start Seq.	End Seq.	Sequence	Ion Score	C. I. %	Modification	Rank	Result Type
805.4818	805.4519	-0.0299	-37	140	146	IPTAYIK					Mascot
830.4366	830.4059	-0.0307	-37	440	446	EGNEIIR					Mascot
898.41	898.3764	-0.0336	-37	306	312	NHGMHFR					Mascot
910.4451	910.4108	-0.0343	-38	188	194	AVYECLR			Carbamidomethyl (C)[5]		Mascot
912.4719	912.422	-0.0499	-55	296	303	AMHAVIDR					Mascot
914.4049	914.373	-0.0319	-35	306	312	NHGMHFR			Oxidation (M)[4]		Mascot
928.4669	928.4342	-0.0327	-35	296	303	AMHAVIDR			Oxidation (M)[2]		Mascot
962.4788	962.4403	-0.0385	-40	228	236	SQAETGEIK					Mascot
1021.5312	1021.4927	-0.0385	-38	33	41	DTDILAAFR					Mascot
1116.583	1116.5388	-0.0442	-40	422	431	VALEACVQAR			Carbamidomethyl (C)[6]		Mascot
1170.5585	1170.5935	0.035	30	304	312	QKNHGMHFR			Oxidation (M)[6]		Mascot
1187.6643	1187.6178	-0.0465	-39	286	295	DNGLLLHIHR					Mascot
1275.7307	1275.6815	-0.0492	-39	340	350	EITLGFVDLLR	85	100			Mascot
1275.7307	1275.6815	-0.0492	-39	340	350	EITLGFVDLLR					Mascot
1451.6219	1451.562	-0.0599	-41	202	213	DDENVNSQPFMR					Mascot
1465.7546	1465.6945	-0.0601	-41	147	159	TFQGPPHGIQVER	96	100			Mascot
1465.7546	1465.6945	-0.0601	-41	147	159	TFQGPPHGIQVER					Mascot
1502.8512	1502.787	-0.0642	-43	165	177	YGRPLLGCTIKPK			Carbamidomethyl (C)[8]		Mascot
1546.7358	1546.6692	-0.0666	-43	451	463	WSPELAAACEVWK			Carbamidomethyl (C)[9]		Mascot
1812.7714	1812.6936	-0.0778	-43	237	252	GHYLNATAGTCEEMMK			Carbamidomethyl (C)[11]		Mascot
1828.7662	1828.682	-0.0842	-46	237	252	GHYLNATAGTCEEMMK			Carbamidomethyl (C)[11], Oxidation (M)[14]		Mascot
1984.8673	1984.7759	-0.0914	-46	237	253	GHYLNATAGTCEEMMKR			Carbamidomethyl (C)[11], Oxidation (M)[14]		Mascot
2000.8623	2000.7673	-0.095	-47	237	253	GHYLNATAGTCEEMMKR			Carbamidomethyl (C)[11], Oxidation (M)[14,15]		Mascot
2033.9572	2033.9081	-0.0491	-24	447	463	QACKWSPELAAACEVWK			Carbamidomethyl (C)[3,13]		Mascot
2169.9868	2169.8865	-0.1003	-46	195	213	GGLDFTKDDENVNSQPFMR					Mascot
2169.9868	2169.8865	-0.1003	-46	195	213	GGLDFTKDDENVNSQPFMR	130	100			Mascot
2185.9819	2185.8806	-0.1013	-46	195	213	GGLDFTKDDENVNSQPFMR			Oxidation (M)[18]		Mascot
2280.1506	2280.0735	-0.0771	-34	340	358	EITLGFVDLLRDDFVEQDR					Mascot
3022.4548	3022.2957	-0.1591	-53	259	285	ELGVPIVMHDYLTGGFTANTSLAHYCR			Carbamidomethyl (C)[26]		Mascot
3022.4548	3022.2957	-0.1591	-53	259	285	ELGVPIVMHDYLTGGFTANTSLAHYCR	146	100	Carbamidomethyl (C)[26]		Mascot
3038.45	3038.301	-0.149	-49	259	285	ELGVPIVMHDYLTGGFTANTSLAHYCR			Carbamidomethyl (C)[26], Oxidation (M)[8]		Mascot
3854.8718	3854.666	-0.2058	-53	42	79	VTPQPGVPPEEAGAAVAAESSTGTWTTVWTDGLTSLDR	238	100			Mascot
3854.8718	3854.666	-0.2058	-53	42	79	VTPQPGVPPEEAGAAVAAESSTGTWTTVWTDGLTSLDR					Mascot

Rank	Protein Name	Accession No	Protein MW	Protein PI	Pep. Count	Protein Score	Protein Score C. I. %	Total Ion Score	Total Ion C. I. %
9	Ribulose bisphosphate carboxylase large chain (Fragment) OS=Manilkara zapota GN=rbcL PE=3 SV=2	RBL_MANZA	51550	6.04	21	860	100	694	100

Peptide Information

Calc. Mass	Obsrv. Mass	± da	± ppm	Start Seq.	End Seq.	Sequence	Ion Score	C. I. %	Modification	Rank	Result Type
805.4929	805.4519	-0.041	-51	1	8	VGFKAGVK					Mascot
830.4366	830.4059	-0.0307	-37	430	436	EGNEIIR					Mascot
898.41	898.3764	-0.0336	-37	296	302	NHGMHFR					Mascot
910.4451	910.4108	-0.0343	-38	178	184	AVYECLR			Carbamidomethyl (C)[5]		Mascot
912.4719	912.422	-0.0499	-55	286	293	AMHAVIDR					Mascot
914.4049	914.373	-0.0319	-35	296	302	NHGMHFR			Oxidation (M)[4]		Mascot
928.4669	928.4342	-0.0327	-35	286	293	AMHAVIDR			Oxidation (M)[2]		Mascot
1021.5312	1021.4927	-0.0385	-38	23	31	DTDILAAFR					Mascot
1116.583	1116.5388	-0.0442	-40	412	421	VALEACVQAR			Carbamidomethyl (C)[6]		Mascot
1170.5585	1170.5935	0.035	30	294	302	QKNHGMHFR			Oxidation (M)[6]		Mascot
1187.6643	1187.6178	-0.0465	-39	276	285	DNGLLLHIHR					Mascot
1245.6433	1245.5841	-0.0592	-48	430	440	EGNEIIREASK					Mascot
1245.6433	1245.5841	-0.0592	-48	430	440	EGNEIIREASK					Mascot
1275.7307	1275.6815	-0.0492	-39	330	340	EITLGFVDLLR	85	100			Mascot
1275.7307	1275.6815	-0.0492	-39	330	340	EITLGFVDLLR					Mascot
1393.6522	1393.5959	-0.0563	-40	12	22	LTYYTPDYETK					Mascot
1433.7495	1433.6858	-0.0637	-44	310	324	LSGGDHVHAGTVVGK					Mascot
1451.6219	1451.562	-0.0599	-41	192	203	DDENVNSQPFMR					Mascot
1465.7546	1465.6945	-0.0601	-41	137	149	TFQGPPHGIQVER	96	100			Mascot
1465.7546	1465.6945	-0.0601	-41	137	149	TFQGPPHGIQVER					Mascot
1472.6978	1472.6348	-0.063	-43	454	465	EIKFEFEAMDTL					Mascot
1488.6926	1488.6345	-0.0581	-39	454	465	EIKFEFEAMDTL			Oxidation (M)[9]		Mascot
1502.8512	1502.787	-0.0642	-43	155	167	YGRPLLGCTIKPK			Carbamidomethyl (C)[8]		Mascot
1546.7358	1546.6692	-0.0666	-43	441	453	WSPELAAACEVWK			Carbamidomethyl (C)[9]		Mascot
2169.9868	2169.8865	-0.1003	-46	185	203	GGLDFTKDDENVNSQPFMR					Mascot
2169.9868	2169.8865	-0.1003	-46	185	203	GGLDFTKDDENVNSQPFMR	130	100			Mascot
2185.9819	2185.8806	-0.1013	-46	185	203	GGLDFTKDDENVNSQPFMR			Oxidation (M)[18]		Mascot
3022.4548	3022.2957	-0.1591	-53	249	275	ELGVPIVMHDYLTGGFTANTSLAHYCR			Carbamidomethyl (C)[26]		Mascot
3022.4548	3022.2957	-0.1591	-53	249	275	ELGVPIVMHDYLTGGFTANTSLAHYCR	146	100	Carbamidomethyl (C)[26]		Mascot
3038.45	3038.301	-0.149	-49	249	275	ELGVPIVMHDYLTGGFTANTSLAHYCR			Carbamidomethyl (C)[26], Oxidation (M)[8]		Mascot
3854.8718	3854.666	-0.2058	-53	32	69	VTPQPGVPPEEAGAAVAAESSTGTWTTVWTDGLTSLDR	238	100			Mascot
3854.8718	3854.666	-0.2058	-53	32	69	VTPQPGVPPEEAGAAVAAESSTGTWTTVWTDGLTSLDR					Mascot

Rank	Protein Name	Accession No	Protein MW	Protein PI	Pep. Count	Protein Score	Protein Score C. I. %	Total Ion Score	Total Ion C. I. %
10	Ribulose bisphosphate carboxylase large chain (Fragment) OS=Betula nigra GN=rbcL PE=3 SV=1	RBL_BETNI	51666	6.13	21	855	100	692	100

Peptide Information

Calc. Mass	Obsrv. Mass	± da	± ppm	Start Seq.	End Seq.	Sequence	Ion Score	C. I. %	Modification	Rank	Result Type
830.4366	830.4059	-0.0307	-37	431	437	EGNEIIR					Mascot
898.41	898.3764	-0.0336	-37	297	303	NHGMHFR					Mascot
910.4451	910.4108	-0.0343	-38	179	185	AVYECLR			Carbamidomethyl (C)[5]		Mascot
912.4719	912.422	-0.0499	-55	287	294	AMHAVIDR					Mascot
914.4049	914.373	-0.0319	-35	297	303	NHGMHFR			Oxidation (M)[4]		Mascot
928.4669	928.4342	-0.0327	-35	287	294	AMHAVIDR			Oxidation (M)[2]		Mascot
1021.5312	1021.4927	-0.0385	-38	24	32	DTDILAAFR					Mascot
1116.583	1116.5388	-0.0442	-40	413	422	VALEACVQAR			Carbamidomethyl (C)[6]		Mascot
1170.5585	1170.5935	0.035	30	295	303	QKNHGMHFR			Oxidation (M)[6]		Mascot
1187.6643	1187.6178	-0.0465	-39	277	286	DNGLLLHIHR					Mascot
1275.7307	1275.6815	-0.0492	-39	331	341	EITLGFVDLLR	85	100			Mascot
1275.7307	1275.6815	-0.0492	-39	331	341	EITLGFVDLLR					Mascot
1393.6522	1393.5959	-0.0563	-40	13	23	LTYYTPDYETK					Mascot
1451.6219	1451.562	-0.0599	-41	193	204	DDENVNSQPFMR					Mascot
1465.7216	1465.6945	-0.0271	-18	311	325	MSGGDHIHAGTVVGK					Mascot
1465.7546	1465.6945	-0.0601	-41	138	150	TFQGPPHGIQVER	96	100			Mascot
1502.8512	1502.787	-0.0642	-43	156	168	YGRPLLGCTIKPK			Carbamidomethyl (C)[8]		Mascot
1546.7358	1546.6692	-0.0666	-43	442	454	WSPELAAACEVWK			Carbamidomethyl (C)[9]		Mascot
1812.7714	1812.6936	-0.0778	-43	228	243	GHYLNATAGTCEEMMK			Carbamidomethyl (C)[11]		Mascot
1828.7662	1828.682	-0.0842	-46	228	243	GHYLNATAGTCEEMMK			Carbamidomethyl (C)[11], Oxidation (M)[14]		Mascot
1984.8673	1984.7759	-0.0914	-46	228	244	GHYLNATAGTCEEMMKR			Carbamidomethyl (C)[11], Oxidation (M)[14]		Mascot
2000.8623	2000.7673	-0.095	-47	228	244	GHYLNATAGTCEEMMKR			Carbamidomethyl (C)[11], Oxidation (M)[14,15]		Mascot
2050.0134	2049.9011	-0.1123	-55	311	330	MSGGDHIHAGTVVGKLEGER					Mascot
2169.9868	2169.8865	-0.1003	-46	186	204	GGLDFTKDDENVNSQPFMR					Mascot
2169.9868	2169.8865	-0.1003	-46	186	204	GGLDFTKDDENVNSQPFMR	130	100			Mascot
2185.9819	2185.8806	-0.1013	-46	186	204	GGLDFTKDDENVNSQPFMR			Oxidation (M)[18]		Mascot
3022.4548	3022.2957	-0.1591	-53	250	276	ELGVPIVMHDYLTGGFTANTSLAHYCR			Carbamidomethyl (C)[26]		Mascot
3022.4548	3022.2957	-0.1591	-53	250	276	ELGVPIVMHDYLTGGFTANTSLAHYCR	146	100	Carbamidomethyl (C)[26]		Mascot
3038.45	3038.301	-0.149	-49	250	276	ELGVPIVMHDYLTGGFTANTSLAHYCR			Carbamidomethyl (C)[26], Oxidation (M)[8]		Mascot
3854.8718	3854.666	-0.2058	-53	33	70	VTPQPGVPPEEAGAAVAAESSTGTWTTVWTDGLTSLDR	238	100			Mascot
3854.8718	3854.666	-0.2058	-53	33	70	VTPQPGVPPEEAGAAVAAESSTGTWTTVWTDGLTSLDR					Mascot

Gel Idx/Pos	50/B23	Instr./Gel Origin	BA2060/131017A	Process Status	Analysis Succeeded
Plate [#] Name	[1] 31496	Instrument Sample Name		Spectra	8

Rank	Protein Name	Accession No	Protein MW	Protein PI	Pep. Count	Protein Score	Protein Score C. I. %	Total Ion Score	Total Ion C. I. %
1	Glutamate--glyoxylate aminotransferase 1 OS=Arabidopsis thaliana GN=GGAT1 PE=1 SV=1	GGT1_ARATH	53267	6.49	8	203	100	173	100

Peptide Information

Calc. Mass	Obsrv. Mass	± da	± ppm	Start Seq.	End Seq.	Sequence	Ion Score	C. I. %	Modification	Rank	Result Type
857.4628	857.4369	-0.0259	-30	438	444	EGVFHLR	15	0			Mascot
857.4628	857.4369	-0.0259	-30	438	444	EGVFHLR					Mascot
1257.5853	1257.6913	0.106	84	269	279	VLMEMGSPFSK			Oxidation (M)[3,5]		Mascot
1257.5853	1257.6913	0.106	84	269	279	VLMEMGSPFSK			Oxidation (M)[3,5]		Mascot
1373.7423	1373.6855	-0.0568	-41	280	291	EVQLVSFHTVSK					Mascot
1761.9381	1761.8733	-0.0648	-37	420	437	LLEATGISTVPGSGFGQK					Mascot
1783.861	1783.7861	-0.0749	-42	91	107	HYLSLTSGGLGAYSDSR	92	100			Mascot
1783.861	1783.7861	-0.0749	-42	91	107	HYLSLTSGGLGAYSDSR					Mascot
2132.9893	2132.8931	-0.0962	-45	379	396	NVVCNFTEGAMYSFPQIR			Carbamidomethyl (C)[4]		Mascot
2132.9893	2132.8931	-0.0962	-45	379	396	NVVCNFTEGAMYSFPQIR	21	4.922	Carbamidomethyl (C)[4]		Mascot
2148.9841	2148.8931	-0.091	-42	379	396	NVVCNFTEGAMYSFPQIR			Carbamidomethyl (C)[4], Oxidation (M)[11]		Mascot
2211.0559	2210.9893	-0.0666	-30	445	463	TTILPAEDEMPEIMDSFKK			Oxidation (M)[10]		Mascot
2255.127	2255.0105	-0.1165	-52	213	233	AMVIINPGNPTGQCLSEANIR			Carbamidomethyl (C)[14]		Mascot
2255.127	2255.0105	-0.1165	-52	213	233	AMVIINPGNPTGQCLSEANIR	46	99.692	Carbamidomethyl (C)[14]		Mascot
2271.1221	2271.0137	-0.1084	-48	213	233	AMVIINPGNPTGQCLSEANIR			Carbamidomethyl (C)[14], Oxidation (M)[2]		Mascot

Rank	Protein Name	Accession No	Protein MW	Protein PI	Pep. Count	Protein Score	Protein Score C. I. %	Total Ion Score	Total Ion C. I. %
2	Glutamate--glyoxylate aminotransferase 2 OS=Arabidopsis thaliana GN=GGAT2 PE=1 SV=1	GGT2_ARATH	53410	6.21	5	164	100	152	100

Peptide Information

Calc. Mass	Obsrv. Mass	± da	± ppm	Start Seq.	End Seq.	Sequence	Ion Score	C. I. %	Modification	Rank	Result Type
857.4628	857.4369	-0.0259	-30	438	444	EGVFHLR	15	0			Mascot
857.4628	857.4369	-0.0259	-30	438	444	EGVFHLR					Mascot
1761.9381	1761.8733	-0.0648	-37	420	437	LLEATGISTVPGSGFGQK					Mascot
1783.861	1783.7861	-0.0749	-42	91	107	HYLSLTSGGLGAYSDSR	92	100			Mascot
1783.861	1783.7861	-0.0749	-42	91	107	HYLSLTSGGLGAYSDSR					Mascot
2253.8853	2253.9854	0.1001	44	464	481	FNDEFMSQYADNFGYSRM			Oxidation (M)[6,18]		Mascot
2255.127	2255.0105	-0.1165	-52	213	233	AMVIINPGNPTGQCLSEANIR			Carbamidomethyl (C)[14]		Mascot
2255.127	2255.0105	-0.1165	-52	213	233	AMVIINPGNPTGQCLSEANIR	46	99.692	Carbamidomethyl (C)[14]		Mascot
2271.1221	2271.0137	-0.1084	-48	213	233	AMVIINPGNPTGQCLSEANIR			Carbamidomethyl (C)[14], Oxidation (M)[2]		Mascot

Rank	Protein Name	Accession No	Protein MW	Protein PI	Pep. Count	Protein Score	Protein Score C. I. %	Total Ion Score	Total Ion C. I. %
3	Kinesin-5 OS=Arabidopsis thaliana GN=ATK5 PE SV=1	ATK5_ARATH	89138.7	7.25	13	48	46.679	14	0

Peptide Information

Calc. Mass	Obsrv. Mass	± da	± ppm	Start Seq.	End Seq.	Sequence	Ion Score	C. I. %	Modification	Rank	Result Type
864.4825	864.455	-0.0275	-32	539	545	SLEQIFK					Mascot
976.488	976.5126	0.0246	25	182	189	DCRVVAEK			Carbamidomethyl (C)[2]		Mascot
976.488	976.5126	0.0246	25	182	189	DCRVVAEK			Carbamidomethyl (C)[2]		Mascot
1089.4742	1089.4039	-0.0703	-65	639	647	THMNEQSSR					Mascot
1104.5645	1104.5649	0.0004	0	270	279	SLQDQLASSR					Mascot
1104.5645	1104.5649	0.0004	0	270	279	SLQDQLASSR					Mascot
1216.5198	1216.4658	-0.054	-44	392	400	QCMHELQDR			Carbamidomethyl (C)[2]		Mascot
1257.6144	1257.6913	0.0769	61	212	221	VTSLEDMYKR			Oxidation (M)[7]		Mascot
1257.6144	1257.6913	0.0769	61	212	221	VTSLEDMYKR	14	0	Oxidation (M)[7]		Mascot
1275.6978	1275.6904	-0.0074	-6	87	97	GKIEQMTDIIK					Mascot
1370.7638	1370.6227	-0.1411	-103	68	79	DEVLALLNERAK					Mascot
1465.7203	1465.7054	-0.0149	-10	142	153	EEELRATITEMK			Oxidation (M)[11]		Mascot
1789.9442	1789.8718	-0.0724	-40	401	415	LADTERQLFEGELLR					Mascot
2133.0181	2132.8931	-0.125	-59	101	117	VCVRWYQQVDETHVQDK					Mascot
2133.0181	2132.8931	-0.125	-59	101	117	VCVRWYQQVDETHVQDK					Mascot
2200.9663	2200.7761	-0.1902	-86	48	67	QDANSDVGSTEECGKVEFTK			Carbamidomethyl (C)[13]		Mascot
2205.1155	2204.9329	-0.1826	-83	520	538	TYTMMGRPETPEQKGLIPR					Mascot

Rank	Protein Name	Accession No	Protein MW	Protein PI	Pep. Count	Protein Score	Protein Score C. I. %	Total Ion Score	Total Ion C. I. %
4	Heat shock 22 kDa protein, mitochondrial OS=Gly max GN=HSP23.9 PE=2 SV=1	HS22M_SOYB	23912.1	6.34	7	42	0		

Peptide Information

Calc. Mass	Obsrv. Mass	± da	± ppm	Start Seq.	End Seq.	Sequence	Ion Score	C. I. %	Modification	Rank	Result Type
976.5607	976.5126	-0.0481	-49	1	9	MASSLIAKR					Mascot
976.5607	976.5126	-0.0481	-49	1	9	MASSLIAKR					Mascot
1104.63	1104.5649	-0.0651	-59	169	177	IDLPDKLYK					Mascot
1104.63	1104.5649	-0.0651	-59	169	177	IDLPDKLYK					Mascot
1165.6688	1165.7058	0.037	32	9	18	RFLSSSLLSR					Mascot
1257.7413	1257.6913	-0.05	-40	139	149	ISVEQNTLIIK					Mascot
1257.7413	1257.6913	-0.05	-40	139	149	ISVEQNTLIIK					Mascot
1274.6237	1274.6912	0.0675	53	54	64	HSERSFPSTAR					Mascot
2204.0989	2203.8687	-0.2302	-104	19	38	SLLRPAASASHRSFDTNAMR			Oxidation (M)[19]		Mascot
3353.5603	3353.5237	-0.0366	-11	72	103	CVGSIFSDSEFEPGSEHDGPGHGQSVPLRVAR					Mascot

Rank	Protein Name	Accession No	Protein MW	Protein PI	Pep. Count	Protein Score	Protein Score C. I. %	Total Ion Score	Total Ion C. I. %
5	Protein argonaute 5 OS=Arabidopsis thaliana GN=AGO5 PE=1 SV=2	AGO5_ARATH	111019	9.51	13	37	0		

Peptide Information

Calc. Mass	Obsrv. Mass	± da	± ppm	Start Seq.	End Seq.	Sequence	Ion Score	C. I. %	Modification	Rank	Result Type
842.4518	842.4797	0.0279	33	439	444	YNYRVK					Mascot
864.3265	864.455	0.1285	149	992	997	EVMFYC			Carbamidomethyl (C)[6], Oxidation (M)[3]		Mascot
870.4791	870.5117	0.0326	37	794	801	ATGQIPQR					Mascot
1051.5275	1051.6761	0.1486	141	594	602	QLIGMCVSK			Carbamidomethyl (C)[6], Oxidation (M)[5]		Mascot
1104.5507	1104.5649	0.0142	13	555	563	LGQWNMIDK					Mascot
1104.5507	1104.5649	0.0142	13	555	563	LGQWNMIDK					Mascot
1257.681	1257.6913	0.0103	8	750	760	YRGLVSAQAHR					Mascot
1257.681	1257.6913	0.0103	8	750	760	YRGLVSAQAHR					Mascot
1373.6519	1373.6855	0.0336	24	571	582	VTSWTCVSFSTR					Mascot
1921.9171	1921.8481	-0.069	-36	862	879	DMTDKSGNIQPGTVVDTK			Oxidation (M)[2]		Mascot
2069.0662	2068.884	-0.1822	-88	260	276	NVTSTDLYQLQQFLDRK					Mascot
2149.0056	2148.8931	-0.1125	-52	26	45	QDQSSGQVAWPGLQQSYGGR					Mascot
2217.0969	2216.7788	-0.3181	-143	807	826	DGVSEGQFSQVLLHEMTAIR					Mascot
3338.5979	3338.5422	-0.0557	-17	113	143	NTEVSETMSNLQITSTETKPEMTSLPPASSK					Mascot
3408.6562	3408.4509	-0.2053	-60	445	474	YQALPAIQTGSDTRPVYLPMELCQIDEGQR			Oxidation (M)[20]		Mascot

Rank	Protein Name	Accession No	Protein MW	Protein PI	Pep. Count	Protein Score	Protein Score C. I. %	Total Ion Score	Total Ion C. I. %
6	Glutathione S-transferase T2 OS=Arabidopsis tha GN=GSTT2 PE=2 SV=1	GSTT2_ARAT	67647	9.25	10	37	0		

Peptide Information

Calc. Mass	Obsrv. Mass	± da	± ppm	Start Seq.	End Seq.	Sequence	Ion Score	C. I. %	Modification	Rank	Result Type
850.4855	850.5154	0.0299	35	16	22	AVLIFCK			Carbamidomethyl (C)[6]		Mascot
864.4937	864.455	-0.0387	-45	3	9	LKVYADR					Mascot
976.5098	976.5126	0.0028	3	39	46	QQLSPEFK					Mascot
976.5098	976.5126	0.0028	3	39	46	QQLSPEFK					Mascot
1213.7303	1213.6617	-0.0686	-57	521	530	LGNLWLLKEK					Mascot
1275.6692	1275.6904	0.0212	17	197	206	VEQWIESTRK					Mascot
1761.884	1761.8733	-0.0107	-6	39	53	QQLSPEFKEINPMGK			Oxidation (M)[13]		Mascot
1783.8431	1783.7861	-0.057	-32	207	221	ATMPHSDEVHEVLFR			Oxidation (M)[3]		Mascot
1783.8431	1783.7861	-0.057	-32	207	221	ATMPHSDEVHEVLFR			Oxidation (M)[3]		Mascot
2306.0691	2306.1516	0.0825	36	337	355	INDYVCKFVGCYDQALNQR			Carbamidomethyl (C)[6]		Mascot
2787.4121	2787.8281	0.416	149	474	497	LENLWVLDEEEQVMDLPLGVKSSK			Oxidation (M)[14]		Mascot
3338.6731	3338.5422	-0.1309	-39	64	92	LFESHAILIYLSSAYASVVDHWYPNDLSK					Mascot

Rank	Protein Name	Accession No	Protein MW	Protein PI	Pep. Count	Protein Score	Protein Score C. I. %	Total Ion Score	Total Ion C. I. %
7	Cyanate hydratase OS=Sorghum bicolor GN=CYN PE=3 SV=1	CYNS_SORBI	18648.4	5.29	6	36	0		

Peptide Information

Calc. Mass	Obsrv. Mass	± da	± ppm	Start Seq.	End Seq.	Sequence	Ion Score	C. I. %	Modification	Rank	Result Type
842.473	842.4797	0.0067	8	51	58	ADTVPALR					Mascot
864.4825	864.455	-0.0275	-32	138	145	VVVTFDGK					Mascot
1165.5769	1165.7058	0.1289	111	14	23	LMEVKEESGK			Oxidation (M)[2]		Mascot
1275.6473	1275.6904	0.0431	34	1	12	MEASGERAAVVR					Mascot
2254.0745	2253.9854	-0.0891	-40	146	163	YLPYTEQKSEHMMSRPTR					Mascot
2271.1875	2271.0137	-0.1738	-77	59	78	AALPTLTDELVQLMMQPPFR					Mascot

Rank	Protein Name	Accession No	Protein MW	Protein PI	Pep. Count	Protein Score	Protein Score C. I. %	Total Ion Score	Total Ion C. I. %
8	Protein LURP-one-related 1 OS=Arabidopsis thali GN=At1g33840 PE=2 SV=2	LOR1_ARATH	25434.4	8.57	7	35	0		

Peptide Information

Calc. Mass	Obsrv. Mass	± da	± ppm	Start Seq.	End Seq.	Sequence	Ion Score	C. I. %	Modification	Rank	Result Type
1104.5289	1104.5649	0.036	33	93	101	GKIMTMHDR			Oxidation (M)[4]		Mascot
1104.5289	1104.5649	0.036	33	93	101	GKIMTMHDR			Oxidation (M)[4]		Mascot
1275.6481	1275.6904	0.0423	33	69	78	DPFFSLHEKR					Mascot
1510.7635	1510.6846	-0.0789	-52	107	120	GGSTEEVDLLYTVK					Mascot
1670.9047	1670.8003	-0.1044	-62	32	45	YCSLHPVDLAIVRK			Carbamidomethyl (C)[2]		Mascot
2212.1648	2211.99	-0.1748	-79	102	120	WLVFRGGSTEEVDLLYTVK					Mascot
2306.2754	2306.1516	-0.1238	-54	46	66	VLKITDGNFVITNAEGNLLFK					Mascot
3353.564	3353.5237	-0.0403	-12	144	172	CDYRLEGVWLETSCFVYAGDSDIILAQMR					Mascot

Rank	Protein Name	Accession No	Protein MW	Protein PI	Pep. Count	Protein Score	Protein Score C. I. %	Total Ion Score	Total Ion C. I. %
9	40S ribosomal protein S15a-1 OS=Arabidopsis th GN=RPS15AA PE=2 SV=2	R15A1_ARAT	14794.9	9.9	6	34	0		

Peptide Information

Calc. Mass	Obsrv. Mass	± da	± ppm	Start Seq.	End Seq.	Sequence	Ion Score	C. I. %	Modification	Rank	Result Type
842.3712	842.4797	0.1085	129	13	19	SMYNAEK					Mascot
1045.5823	1045.5303	-0.052	-50	24	32	QVMIRPSSK					Mascot
1200.6411	1200.4733	-0.1678	-140	120	130	NVGGKVLGFFY					Mascot
1736.7662	1736.6027	-0.1635	-94	44	57	HGYIGEFEYVDDHR					Mascot
1795.9258	1795.7716	-0.1542	-86	4	19	ISVLNDALKSMYNAEK					Mascot
2254.0808	2253.9854	-0.0954	-42	98	117	QFGYIVLTTSAGIMDHEEAR			Oxidation (M)[14]		Mascot

Rank	Protein Name	Accession No	Protein MW	Protein PI	Pep. Count	Protein Score	Protein Score C. I. %	Total Ion Score	Total Ion C. I. %
10	40S ribosomal protein S15a OS=Brassica napus GN=RPS15A PE=2 SV=3	RS15A_BRAN	14778.9	9.94	6	34	0		

Peptide Information

Calc. Mass	Obsrv. Mass	± da	± ppm	Start Seq.	End Seq.	Sequence	Ion Score	C. I. %	Modification	Rank	Result Type
842.3712	842.4797	0.1085	129	13	19	SMFNAEK			Oxidation (M)[2]		Mascot
1045.5823	1045.5303	-0.052	-50	24	32	QVMIRPSSK					Mascot
1200.6411	1200.4733	-0.1678	-140	120	130	NVGGKVLGFFY					Mascot
1736.7662	1736.6027	-0.1635	-94	44	57	HGYIGEFEYVDDHR					Mascot
1795.9258	1795.7716	-0.1542	-86	4	19	ISVLNDALKSMFNAEK			Oxidation (M)[11]		Mascot
2254.0808	2253.9854	-0.0954	-42	98	117	QFGYIVLTTSAGIMDHEEAR			Oxidation (M)[14]		Mascot

Gel Idx/Pos	51/B24	Instr./Gel Origin	BA2060/131017A	Process Status	Analysis Succeeded
Plate [#] Name	[1] 31496	Instrument Sample Name		Spectra	8

Rank	Protein Name	Accession No	Protein MW	Protein PI	Pep. Count	Protein Score	Protein Score C. I. %	Total Ion Score	Total Ion C. I. %
1	Glutamate--glyoxylate aminotransferase 1 OS=Arabidopsis thaliana GN=GGAT1 PE=1 SV=1	GGT1_ARATH	53267	6.49	10	178	100	133	100

Peptide Information

Calc. Mass	Obsrv. Mass	± da	± ppm	Start Seq.	End Seq.	Sequence	Ion Score	C. I. %	Modification	Rank	Result Type
857.4628	857.4426	-0.0202	-24	438	444	EGVFHLR	11	0			Mascot
857.4628	857.4426	-0.0202	-24	438	444	EGVFHLR					Mascot
1140.5758	1140.5422	-0.0336	-29	411	419	VPDVFYCLK			Carbamidomethyl (C)[7]		Mascot
1245.6807	1245.5972	-0.0835	-67	142	152	GVMQILNCVIR					Mascot
1257.5853	1257.6997	0.1144	91	269	279	VLMEMGSPFSK			Oxidation (M)[3,5]		Mascot
1257.5853	1257.6997	0.1144	91	269	279	VLMEMGSPFSK			Oxidation (M)[3,5]		Mascot
1373.7423	1373.6993	-0.043	-31	280	291	EVQLVSFHTVSK					Mascot
1783.861	1783.8037	-0.0573	-32	91	107	HYLSLTSGGLGAYSDSR					Mascot
1982.993	1982.9325	-0.0605	-31	89	107	AKHYLSLTSGGLGAYSDSR					Mascot
2132.9893	2132.9133	-0.076	-36	379	396	NVVCNFTEGAMYSFPQIR	58	99.98	Carbamidomethyl (C)[4]		Mascot
2132.9893	2132.9133	-0.076	-36	379	396	NVVCNFTEGAMYSFPQIR			Carbamidomethyl (C)[4]		Mascot
2148.9841	2148.9138	-0.0703	-33	379	396	NVVCNFTEGAMYSFPQIR			Carbamidomethyl (C)[4], Oxidation (M)[11]		Mascot
2211.0559	2211.0344	-0.0215	-10	445	463	TTILPAEDEMPEIMDSFKK			Oxidation (M)[10]		Mascot
2255.127	2255.0537	-0.0733	-33	213	233	AMVIINPGNPTGQCLSEANIR	64	99.996	Carbamidomethyl (C)[14]		Mascot
2255.127	2255.0537	-0.0733	-33	213	233	AMVIINPGNPTGQCLSEANIR			Carbamidomethyl (C)[14]		Mascot
2271.1221	2271.0452	-0.0769	-34	213	233	AMVIINPGNPTGQCLSEANIR			Carbamidomethyl (C)[14], Oxidation (M)[2]		Mascot

Rank	Protein Name	Accession No	Protein MW	Protein PI	Pep. Count	Protein Score	Protein Score C. I. %	Total Ion Score	Total Ion C. I. %
2	Glutamate--glyoxylate aminotransferase 2 OS=Arabidopsis thaliana GN=GGAT2 PE=1 SV=1	GGT2_ARATH	53410	6.21	9	110	100	75	100

Peptide Information

Calc. Mass	Obsrv. Mass	± da	± ppm	Start Seq.	End Seq.	Sequence	Ion Score	C. I. %	Modification	Rank	Result Type
857.4628	857.4426	-0.0202	-24	438	444	EGVFHLR	11	0			Mascot
857.4628	857.4426	-0.0202	-24	438	444	EGVFHLR					Mascot
1140.5758	1140.5422	-0.0336	-29	411	419	VPDVFYCLK			Carbamidomethyl (C)[7]		Mascot
1245.6807	1245.5972	-0.0835	-67	142	152	GVMQILNCVIR					Mascot
1433.597	1433.7102	0.1132	79	367	378	ARMMTDGFNSCK			Carbamidomethyl (C)[11], Oxidation (M)[3]		Mascot
1783.861	1783.8037	-0.0573	-32	91	107	HYLSLTSGGLGAYSDSR					Mascot
1982.993	1982.9325	-0.0605	-31	89	107	AKHYLSLTSGGLGAYSDSR					Mascot
2218.9497	2218.9456	-0.0041	-2	463	480	KFNDEFMSQYADNFGYSR					Mascot
2225.0715	2225.05	-0.0215	-10	445	463	TTILPAEEEMPEIMDSFKK			Oxidation (M)[10]		Mascot
2255.127	2255.0537	-0.0733	-33	213	233	AMVIINPGNPTGQCLSEANIR	64	99.996	Carbamidomethyl (C)[14]		Mascot
2255.127	2255.0537	-0.0733	-33	213	233	AMVIINPGNPTGQCLSEANIR			Carbamidomethyl (C)[14]		Mascot
2271.1221	2271.0452	-0.0769	-34	213	233	AMVIINPGNPTGQCLSEANIR			Carbamidomethyl (C)[14], Oxidation (M)[2]		Mascot

Rank	Protein Name	Accession No	Protein MW	Protein PI	Pep. Count	Protein Score	Protein Score C. I. %	Total Ion Score	Total Ion C. I. %
3	Ribulose bisphosphate carboxylase large chain (Fragment) OS=Salacia pallescens GN=rbcL PE=3 SV=2	RBL_SALPL	49365.3	6.25	9	61	97.07	14	0

Peptide Information

Calc. Mass	Obsrv. Mass	± da	± ppm	Start Seq.	End Seq.	Sequence	Ion Score	C. I. %	Modification	Rank	Result Type
857.4951	857.4426	-0.0525	-61	440	446	EGNXIIR	8	0			Mascot
914.4049	914.384	-0.0209	-23	306	312	NHGMHFR			Oxidation (M)[4]		Mascot
1021.5312	1021.5099	-0.0213	-21	33	41	DTDILAAFR					Mascot
1045.5459	1045.5491	0.0032	3	422	431	VAVEACVQAR					Mascot
1257.6909	1257.6997	0.0088	7	436	446	DLAREGNXIIR	14	0			Mascot
1465.7546	1465.7053	-0.0493	-34	147	159	TFQGPPHGIQVER					Mascot
1909.8894	1909.9747	0.0853	45	237	253	GHYLNATAGTCEEMIKR			Oxidation (M)[14]		Mascot
2169.9868	2169.9177	-0.0691	-32	195	213	GGLDFTKDDENVNSQPFMR					Mascot
3854.8718	3854.7671	-0.1047	-27	42	79	VTPQPGVPPEEAGAAVAAESSTGTWTTVWTDGLTSLDR					Mascot

Rank	Protein Name	Accession No	Protein MW	Protein PI	Pep. Count	Protein Score	Protein Score C. I. %	Total Ion Score	Total Ion C. I. %
4	ATP synthase subunit beta, chloroplastic OS=Idiospermum australiense GN=atpB PE=3 SV=1	ATPB_IDIAU	53596.9	5.43	12	56	92.468		

Peptide Information

Calc. Mass	Obsrv. Mass	± da	± ppm	Start Seq.	End Seq.	Sequence	Ion Score	C. I. %	Modification	Rank	Result Type
1021.5676	1021.5099	-0.0577	-56	448	456	YVGLAETIR					Mascot
1045.604	1045.5491	-0.0549	-53	155	163	VVDLLAPYR					Mascot
1245.5126	1245.5972	0.0846	68	208	217	EGNDLYMEMK			Oxidation (M)[7]		Mascot
1275.6978	1275.6917	-0.0061	-5	487	497	AMKLELESNLK					Mascot
1328.6705	1328.644	-0.0265	-20	192	205	AHGGVSVFGGVGER					Mascot
1431.6863	1431.6429	-0.0434	-30	379	390	IVGEEHYETAQR					Mascot
1433.7747	1433.7102	-0.0645	-45	278	291	FVQAGSEVSALLGR					Mascot
1471.7614	1471.7233	-0.0381	-26	249	261	VGLTALTMAEYFR					Mascot
1955.0232	1954.8705	-0.1527	-78	110	127	IFNVLGEPVDNLGPVDTR					Mascot
1983.0658	1982.9325	-0.1333	-67	128	145	TTSPIHRSAPAFIQLDTK					Mascot
2298.074	2298.1199	0.0459	20	292	312	MPSAVGYQPTLSTEMGSLQER			Oxidation (M)[1]		Mascot
3348.6594	3348.5371	-0.1223	-37	76	109	AVAMSATDGLMRGMEVIDTGAPLSVPVGGATLGR			Oxidation (M)[4,11,14]		Mascot

Rank	Protein Name	Accession No	Protein MW	Protein PI	Pep. Count	Protein Score	Protein Score C. I. %	Total Ion Score	Total Ion C. I. %
5	ATP synthase subunit beta, chloroplastic OS=Ba scilloides GN=atpB PE=3 SV=2	ATPB_BARS	53265.8	5.22	12	56	91.151		

Peptide Information

Calc. Mass	Obsrv. Mass	± da	± ppm	Start Seq.	End Seq.	Sequence	Ion Score	C. I. %	Modification	Rank	Result Type
1021.5676	1021.5099	-0.0577	-56	448	456	YVGLAETIR					Mascot
1045.604	1045.5491	-0.0549	-53	155	163	VVDLLAPYR					Mascot
1245.5126	1245.5972	0.0846	68	208	217	EGNDLYMEMK			Oxidation (M)[7]		Mascot
1328.6705	1328.644	-0.0265	-20	192	205	AHGGVSVFGGVGER					Mascot
1431.6863	1431.6429	-0.0434	-30	379	390	IVGEEHYETAQR					Mascot
1433.7747	1433.7102	-0.0645	-45	278	291	FVQAGSEVSALLGR					Mascot
1471.7614	1471.7233	-0.0381	-26	249	261	VGLTALTMAEYFR					Mascot
1789.9291	1789.8892	-0.0399	-22	2	18	RINPTTSGSAVSTLEEK					Mascot
1789.9291	1789.8892	-0.0399	-22	2	18	RINPTTSGSAVSTLEEK					Mascot
1955.0232	1954.8705	-0.1527	-78	110	127	IFNVLGEPVDNLGPVDTR					Mascot
1983.0658	1982.9325	-0.1333	-67	128	145	TTSPIHRSAPAFIQLDTK					Mascot
2071.0852	2071.0195	-0.0657	-32	88	109	GMEVLDTGAALSVPVGGATLGR					Mascot
2298.074	2298.1199	0.0459	20	292	312	MPSAVGYQPTLSTEMGSLQER			Oxidation (M)[1]		Mascot

Rank	Protein Name	Accession No	Protein MW	Protein PI	Pep. Count	Protein Score	Protein Score C. I. %	Total Ion Score	Total Ion C. I. %
6	ATP synthase subunit beta, chloroplastic OS=Hyacinthoides non-scripta GN=atpB PE=3 SV=1	ATPB_HYAN	53325.8	5.22	12	56	91.151		

Peptide Information

Calc. Mass	Obsrv. Mass	± da	± ppm	Start Seq.	End Seq.	Sequence	Ion Score	C. I. %	Modification	Rank	Result Type
1021.5676	1021.5099	-0.0577	-56	448	456	YVGLAETIR					Mascot
1045.604	1045.5491	-0.0549	-53	155	163	VVDLLAPYR					Mascot
1245.5126	1245.5972	0.0846	68	208	217	EGNDLYMEMK			Oxidation (M)[7]		Mascot
1328.6705	1328.644	-0.0265	-20	192	205	AHGGVSVFGGVGER					Mascot
1431.6863	1431.6429	-0.0434	-30	379	390	IVGEEHYETAQR					Mascot
1433.7747	1433.7102	-0.0645	-45	278	291	FVQAGSEVSALLGR					Mascot
1471.7614	1471.7233	-0.0381	-26	249	261	VGLTALTMAEYFR					Mascot
1789.9291	1789.8892	-0.0399	-22	2	18	RINPTTSGSAVSTLEEK					Mascot
1789.9291	1789.8892	-0.0399	-22	2	18	RINPTTSGSAVSTLEEK					Mascot
1969.0388	1968.9807	-0.0581	-30	110	127	IFNVLGEPIDNLGPVDTR					Mascot
1983.0658	1982.9325	-0.1333	-67	128	145	TTSPIHRSAPAFIQLDTK					Mascot
2071.0852	2071.0195	-0.0657	-32	88	109	GMEVIDTGAALSVPVGGATLGR					Mascot
2298.074	2298.1199	0.0459	20	292	312	MPSAVGYQPTLSTEMGSLQER			Oxidation (M)[1]		Mascot

Rank	Protein Name	Accession No	Protein MW	Protein PI	Pep. Count	Protein Score	Protein Score C. I. %	Total Ion Score	Total Ion C. I. %
7	ATP synthase subunit beta, chloroplastic OS=Gl max GN=atpB PE=3 SV=1	ATPB_SOYBN	53721.1	5.29	12	55	88.86		

Peptide Information

Calc. Mass	Obsrv. Mass	± da	± ppm	Start Seq.	End Seq.	Sequence	Ion Score	C. I. %	Modification	Rank	Result Type
1045.604	1045.5491	-0.0549	-53	155	163	VVDLLAPYR					Mascot
1245.5126	1245.5972	0.0846	68	208	217	EGNDLYMEMK			Oxidation (M)[7]		Mascot
1275.7129	1275.6917	-0.0212	-17	40	50	MPNIYNALIVK					Mascot
1328.6705	1328.644	-0.0265	-20	192	205	AHGGVSVFGGVGER					Mascot
1431.6863	1431.6429	-0.0434	-30	379	390	IVGEEHYETAQR					Mascot
1433.7747	1433.7102	-0.0645	-45	278	291	FVQAGSEVSALLGR					Mascot
1471.7614	1471.7233	-0.0381	-26	249	261	VGLTALTMAEYFR					Mascot
1670.8959	1670.8207	-0.0752	-45	3	18	INPTTSGPEVSALEKK					Mascot
1969.0388	1968.9807	-0.0581	-30	110	127	IFNVLGEPIDNLGPVDTR					Mascot
1983.0658	1982.9325	-0.1333	-67	128	145	TTSPIHRSAPAFIQLDTK					Mascot
2071.0852	2071.0195	-0.0657	-32	88	109	GMEVIDTGAALSVPVGGATLGR					Mascot
2298.074	2298.1199	0.0459	20	292	312	MPSAVGYQPTLSTEMGSLQER			Oxidation (M)[1]		Mascot

Rank	Protein Name	Accession No	Protein MW	Protein PI	Pep. Count	Protein Score	Protein Score C. I. %	Total Ion Score	Total Ion C. I. %
8	Ribulose bisphosphate carboxylase large chain (Fragment) OS=Bulnesia arborea GN=rbcL PE=3 SV=1	RBL_BULAR	51566	6.18	9	52	78.278	14	0

Peptide Information

Calc. Mass	Obsrv. Mass	± da	± ppm	Start Seq.	End Seq.	Sequence	Ion Score	C. I. %	Modification	Rank	Result Type
914.4049	914.384	-0.0209	-23	297	303	NHGMHFR			Oxidation (M)[4]		Mascot
1021.5312	1021.5099	-0.0213	-21	24	32	DTDILAAFR					Mascot
1187.6643	1187.6287	-0.0356	-30	277	286	DNGLLLHIHR					Mascot
1245.6337	1245.5972	-0.0365	-29	209	218	FLFCAEALFK			Carbamidomethyl (C)[4]		Mascot
1257.6909	1257.6997	0.0088	7	427	437	DLAREGNTIIR	14	0			Mascot
1257.6909	1257.6997	0.0088	7	427	437	DLAREGNTIIR					Mascot
1275.7307	1275.6917	-0.039	-31	331	341	EITLGFVDLLR					Mascot
1909.8894	1909.9747	0.0853	45	228	244	GHYLNATAGTCEEMIKR			Oxidation (M)[14]		Mascot
2169.9868	2169.9177	-0.0691	-32	186	204	GGLDFTKDDENVNSQPFMR					Mascot
3854.8718	3854.7671	-0.1047	-27	33	70	VTPQPGVPPEEAGAAVAAESSTGTWTTVWTDGLTSLDR					Mascot

Rank	Protein Name	Accession No	Protein MW	Protein PI	Pep. Count	Protein Score	Protein Score C. I. %	Total Ion Score	Total Ion C. I. %
9	Ribulose bisphosphate carboxylase large chain (Fragment) OS=Sinapis alba GN=rbcL PE=3 SV=1	RBL_SINAL	52405.4	6.23	11	51	73.885		

Peptide Information

Calc. Mass	Obsrv. Mass	± da	± ppm	Start Seq.	End Seq.	Sequence	Ion Score	C. I. %	Modification	Rank	Result Type
914.4049	914.384	-0.0209	-23	306	312	NHGMHFR			Oxidation (M)[4]		Mascot
1021.5312	1021.5099	-0.0213	-21	33	41	DTDILAAFR					Mascot
1187.6643	1187.6287	-0.0356	-30	286	295	DNGLLLHIHR					Mascot
1245.6433	1245.5972	-0.0461	-37	440	450	EGNEIIREASK					Mascot
1275.7307	1275.6917	-0.039	-31	340	350	EITLGFVDLLR					Mascot
1431.8206	1431.6429	-0.1777	-124	135	146	LEDLRIPTAYIK					Mascot
1465.7546	1465.7053	-0.0493	-34	147	159	TFQGPPHGIQVER					Mascot
1789.9667	1789.8892	-0.0775	-43	317	334	ALRLSGGDHVHSGTVVGK					Mascot
1789.9667	1789.8892	-0.0775	-43	317	334	ALRLSGGDHVHSGTVVGK					Mascot
1909.8894	1909.9747	0.0853	45	237	253	GHYLNATAGTCEEMIKR			Oxidation (M)[14]		Mascot
2169.9868	2169.9177	-0.0691	-32	195	213	GGLDFTKDDENVNSQPFMR					Mascot
3854.8718	3854.7671	-0.1047	-27	42	79	VTPQPGVPPEEAGAAVAAESSTGTWTTVWTDGLTSLDR					Mascot

Rank	Protein Name	Accession No	Protein MW	Protein PI	Pep. Count	Protein Score	Protein Score C. I. %	Total Ion Score	Total Ion C. I. %
10	Ribulose bisphosphate carboxylase large chain OS=Phaseolus vulgaris GN=rbcL PE=3 SV=1	RBL_PHAVU	52767.6	5.96	11	51	73.276		

Peptide Information

Calc. Mass	Obsrv. Mass	± da	± ppm	Start Seq.	End Seq.	Sequence	Ion Score	C. I. %	Modification	Rank	Result Type
914.4049	914.384	-0.0209	-23	306	312	NHGMHFR			Oxidation (M)[4]		Mascot
1021.5312	1021.5099	-0.0213	-21	33	41	DTDILAAFR					Mascot
1187.6643	1187.6287	-0.0356	-30	286	295	DNGLLLHIHR					Mascot
1245.6433	1245.5972	-0.0461	-37	440	450	EGNEIIREASK					Mascot
1275.7307	1275.6917	-0.039	-31	340	350	EITLGFVDLLR					Mascot
1431.8206	1431.6429	-0.1777	-124	135	146	LEDLRIPTAYIK					Mascot
1465.7546	1465.7053	-0.0493	-34	147	159	TFQGPPHGIQVER					Mascot
1789.9667	1789.8892	-0.0775	-43	317	334	ALRLSGGDHVHSGTVVGK					Mascot
1789.9667	1789.8892	-0.0775	-43	317	334	ALRLSGGDHVHSGTVVGK					Mascot
1909.8894	1909.9747	0.0853	45	237	253	GHYLNATAGTCEEMIKR			Oxidation (M)[14]		Mascot
2169.9868	2169.9177	-0.0691	-32	195	213	GGLDFTKDDENVNSQPFMR					Mascot
3854.8718	3854.7671	-0.1047	-27	42	79	VTPQPGVPPEEAGAAVAAESSTGTWTTVWTDGLTSLDR					Mascot

Gel Idx/Pos	52/C1	Instr./Gel Origin	BA2060/131017A	Process Sta	Analysis Succeeded
Plate [#] Name	[1] 31496	Instrument Sample Name		Spectra	11

Rank	Protein Name	Accession No	Protein MW	Protein PI	Pep. Count	Protein Score	Protein Score C. I. %	Total Ion Score	Total Ion C. I. %
1	ATP synthase subunit alpha, chloroplastic OS=G max GN=atpA PE=3 SV=1	ATPA_SOYBI	55719.3	5.15	15	286	100	202	100

Peptide Information

Calc. Mass	Obsrv. Mass	± da	± ppm	Start Seq.	End Seq.	Sequence	Ion Score	C. I. %	Modification	Rank	Result Type
815.4621	815.3492	-0.1129	-138	166	172	ELIIGDR					Mascot
860.5022	860.3828	-0.1194	-139	273	279	QMSLLLR					Mascot
860.5022	860.3828	-0.1194	-139	273	279	QMSLLLR					Mascot
876.4971	876.3705	-0.1266	-144	273	279	QMSLLLR			Oxidation (M)[2]		Mascot
901.485	901.3577	-0.1273	-141	406	413	ATQNQLAR					Mascot
1123.563	1123.4006	-0.1624	-145	17	25	IEQYNTEVK					Mascot
1252.726	1252.5487	-0.1773	-142	129	140	LIESPAPGIISR					Mascot
1252.726	1252.5487	-0.1773	-142	129	140	LIESPAPGIISR	28	76.018			Mascot
1266.7528	1266.5808	-0.172	-136	108	119	VINALAKPIDGR					Mascot
1317.705	1317.5104	-0.1946	-148	256	266	HTLIIYDDLSK					Mascot
1408.7067	1408.5052	-0.2015	-143	15	25	ERIEQYNTEVK					Mascot
1416.7845	1416.5833	-0.2012	-142	95	107	IAQIPVSEAYLGR					Mascot
1416.7845	1416.5833	-0.2012	-142	95	107	IAQIPVSEAYLGR	45	99.579			Mascot
1501.7969	1501.5812	-0.2157	-144	203	216	ASSVAQVVNTLQER					Mascot
1501.7969	1501.5812	-0.2157	-144	203	216	ASSVAQVVNTLQER	34	94.753			Mascot
1553.7383	1553.5084	-0.2299	-148	285	297	EAYPGDVFYLHSR					Mascot
1553.7383	1553.5084	-0.2299	-148	285	297	EAYPGDVFYLHSR	57	99.971			Mascot
1612.9016	1612.6653	-0.2363	-147	26	41	IVNTGTVLQVGDGIAR	38	98.046			Mascot
1612.9016	1612.6653	-0.2363	-147	26	41	IVNTGTVLQVGDGIAR					Mascot
2033.0405	2032.7415	-0.299	-147	71	90	NVGVVLMGDGLMIQEGSSVK					Mascot
2049.0354	2048.7322	-0.3032	-148	71	90	NVGVVLMGDGLMIQEGSSVK			Oxidation (M)[7]		Mascot
2289.2158	2288.8872	-0.3286	-144	142	162	SVYEPLQTGLIAIDSMIPIGR			Oxidation (M)[16]		Mascot
2804.4609	2804.041	-0.4199	-150	177	202	TAVATDTILNQQGQNVICVYVAIGQK			Carbamidomethyl (C)[18]		Mascot

Rank	Protein Name	Accession No	Protein MW	Protein PI	Pep. Count	Protein Score	Protein Score C. I. %	Total Ion Score	Total Ion C. I. %
2	ATP synthase subunit alpha, chloroplastic OS=Eucalyptus globulus subsp. globulus GN=atpA PE=3 SV=1	ATPA_EUCG	55478.1	5.15	12	259	100	202	100

Peptide Information

Calc. Mass	Obsrv. Mass	± da	± ppm	Start Seq.	End Seq.	Sequence	Ion Score	C. I. %	Modification	Rank	Result Type
815.4621	815.3492	-0.1129	-138	166	172	ELIIGDR					Mascot
860.5022	860.3828	-0.1194	-139	273	279	QMSLLLR					Mascot
860.5022	860.3828	-0.1194	-139	273	279	QMSLLLR					Mascot
876.4971	876.3705	-0.1266	-144	273	279	QMSLLLR			Oxidation (M)[2]		Mascot
901.485	901.3577	-0.1273	-141	406	413	ATQNQLAR					Mascot
1252.726	1252.5487	-0.1773	-142	129	140	LIESPAPGIISR					Mascot
1252.726	1252.5487	-0.1773	-142	129	140	LIESPAPGIISR	28	76.018			Mascot
1266.7528	1266.5808	-0.172	-136	108	119	VINALAKPIDGR					Mascot
1317.705	1317.5104	-0.1946	-148	256	266	HTLIIYDDLSK					Mascot
1416.7845	1416.5833	-0.2012	-142	95	107	IAQIPVSEAYLGR					Mascot
1416.7845	1416.5833	-0.2012	-142	95	107	IAQIPVSEAYLGR	45	99.579			Mascot
1501.7969	1501.5812	-0.2157	-144	203	216	ASSVAQVVNTLQER					Mascot
1501.7969	1501.5812	-0.2157	-144	203	216	ASSVAQVVNTLQER	34	94.753			Mascot
1553.7383	1553.5084	-0.2299	-148	285	297	EAYPGDVFYLHSR					Mascot
1553.7383	1553.5084	-0.2299	-148	285	297	EAYPGDVFYLHSR	57	99.971			Mascot
1612.9016	1612.6653	-0.2363	-147	26	41	IVNTGTVLQVGDGIAR	38	98.046			Mascot
1612.9016	1612.6653	-0.2363	-147	26	41	IVNTGTVLQVGDGIAR					Mascot
2289.2158	2288.8872	-0.3286	-144	142	162	SVYEPLQTGLIAIDSMIPIGR			Oxidation (M)[16]		Mascot
2804.4609	2804.041	-0.4199	-150	177	202	TAVATDTILNQQGQNVICVYVAIGQK			Carbamidomethyl (C)[18]		Mascot

Rank	Protein Name	Accession No	Protein MW	Protein PI	Pep. Count	Protein Score	Protein Score C. I. %	Total Ion Score	Total Ion C. I. %
3	ATP synthase subunit alpha, chloroplastic OS=Gossypium hirsutum GN=atpA PE=3 SV=1	ATPA_GOSHI	55379.9	5.25	13	233	100	168	100

Peptide Information

Calc. Mass	Obsrv. Mass	± da	± ppm	Start Seq.	End Seq.	Sequence	Ion Score	C. I. %	Modification	Rank	Result Type
815.4621	815.3492	-0.1129	-138	166	172	ELIIGDR					Mascot
860.5022	860.3828	-0.1194	-139	273	279	QMSLLLR					Mascot
860.5022	860.3828	-0.1194	-139	273	279	QMSLLLR					Mascot
876.4971	876.3705	-0.1266	-144	273	279	QMSLLLR			Oxidation (M)[2]		Mascot
901.485	901.3577	-0.1273	-141	406	413	ATQNQLAR					Mascot
933.5152	933.3774	-0.1378	-148	501	507	FRLQEQL					Mascot
1252.726	1252.5487	-0.1773	-142	129	140	LIESPAPGIISR					Mascot
1252.726	1252.5487	-0.1773	-142	129	140	LIESPAPGIISR	28	76.018			Mascot
1266.7528	1266.5808	-0.172	-136	108	119	VINALAKPIDGR					Mascot
1317.705	1317.5104	-0.1946	-148	256	266	HTLIIYDDLSK					Mascot
1408.6638	1408.5052	-0.1586	-113	492	502	DAIQDQMERFR					Mascot
1416.7845	1416.5833	-0.2012	-142	95	107	IAQIPVSEAYLGR					Mascot
1416.7845	1416.5833	-0.2012	-142	95	107	IAQIPVSEAYLGR	45	99.579			Mascot
1553.7383	1553.5084	-0.2299	-148	285	297	EAYPGDVFYLHSR					Mascot
1553.7383	1553.5084	-0.2299	-148	285	297	EAYPGDVFYLHSR	57	99.971			Mascot
1612.9016	1612.6653	-0.2363	-147	26	41	IVNTGTVLQVGDGIAR	38	98.046			Mascot
1612.9016	1612.6653	-0.2363	-147	26	41	IVNTGTVLQVGDGIAR					Mascot
2289.2158	2288.8872	-0.3286	-144	142	162	SVYEPLQTGLIAIDSMIPIGR			Oxidation (M)[16]		Mascot
2804.4609	2804.041	-0.4199	-150	177	202	TAVATDTILNQQGQNVICVYVAIGQK			Carbamidomethyl (C)[18]		Mascot

Rank	Protein Name	Accession No	Protein MW	Protein PI	Pep. Count	Protein Score	Protein Score C. I. %	Total Ion Score	Total Ion C. I. %
4	ATP synthase subunit alpha, chloroplastic OS=D carota GN=atpA PE=3 SV=1	ATPA_DAUC	55451.2	5.34	12	224	100	168	100

Peptide Information

Calc. Mass	Obsrv. Mass	± da	± ppm	Start Seq.	End Seq.	Sequence	Ion Score	C. I. %	Modification	Rank	Result Type
815.4621	815.3492	-0.1129	-138	166	172	ELIIGDR					Mascot
860.5022	860.3828	-0.1194	-139	273	279	QMSLLLR					Mascot
860.5022	860.3828	-0.1194	-139	273	279	QMSLLLR					Mascot
876.4825	876.3705	-0.112	-128	501	507	FILQEQV					Mascot
901.485	901.3577	-0.1273	-141	406	413	ATQNQLAR					Mascot
1179.6005	1179.4351	-0.1654	-140	17	25	IEEYNREVK					Mascot
1252.726	1252.5487	-0.1773	-142	129	140	LIESPAPGIISR					Mascot
1252.726	1252.5487	-0.1773	-142	129	140	LIESPAPGIISR	28	76.018			Mascot
1416.7845	1416.5833	-0.2012	-142	95	107	IAQIPVSEAYLGR					Mascot
1416.7845	1416.5833	-0.2012	-142	95	107	IAQIPVSEAYLGR	45	99.579			Mascot
1535.7812	1535.5581	-0.2231	-145	203	216	ASSVAQVVTNFQER					Mascot
1553.7383	1553.5084	-0.2299	-148	285	297	EAYPGDVFYLHSR					Mascot
1553.7383	1553.5084	-0.2299	-148	285	297	EAYPGDVFYLHSR	57	99.971			Mascot
1612.9016	1612.6653	-0.2363	-147	26	41	IVNTGTVLQVGDGIAR	38	98.046			Mascot
1612.9016	1612.6653	-0.2363	-147	26	41	IVNTGTVLQVGDGIAR					Mascot
2289.2158	2288.8872	-0.3286	-144	142	162	SVYEPLQTGLIAIDSMIPIGR			Oxidation (M)[16]		Mascot
2804.4609	2804.041	-0.4199	-150	177	202	TAVATDTILNQQGQNVICVYVAIGQK			Carbamidomethyl (C)[18]		Mascot

Rank	Protein Name	Accession No	Protein MW	Protein PI	Pep. Count	Protein Score	Protein Score C. I. %	Total Ion Score	Total Ion C. I. %
5	ATP synthase subunit alpha, chloroplastic OS=P trichocarpa GN=atpA PE=3 SV=1	ATPA_POPTI	55288	5.2	12	224	100	168	100

Peptide Information

Calc. Mass	Obsrv. Mass	± da	± ppm	Start Seq.	End Seq.	Sequence	Ion Score	C. I. %	Modification	Rank	Result Type
815.4621	815.3492	-0.1129	-138	166	172	ELIIGDR					Mascot
860.5022	860.3828	-0.1194	-139	273	279	QMSLLLR					Mascot
860.5022	860.3828	-0.1194	-139	273	279	QMSLLLR					Mascot
876.4825	876.3705	-0.112	-128	501	507	FLLQEQV					Mascot
901.485	901.3577	-0.1273	-141	406	413	ATQNQLAR					Mascot
1252.726	1252.5487	-0.1773	-142	129	140	LIESPAPGIISR					Mascot
1252.726	1252.5487	-0.1773	-142	129	140	LIESPAPGIISR	28	76.018			Mascot
1266.7528	1266.5808	-0.172	-136	108	119	VINALAKPIDGR					Mascot
1317.705	1317.5104	-0.1946	-148	256	266	HTLIIYDDLSK					Mascot
1416.7845	1416.5833	-0.2012	-142	95	107	IAQIPVSEAYLGR					Mascot
1416.7845	1416.5833	-0.2012	-142	95	107	IAQIPVSEAYLGR	45	99.579			Mascot
1553.7383	1553.5084	-0.2299	-148	285	297	EAYPGDVFYLHSR					Mascot
1553.7383	1553.5084	-0.2299	-148	285	297	EAYPGDVFYLHSR	57	99.971			Mascot
1612.9016	1612.6653	-0.2363	-147	26	41	IVNTGTVLQVGDGIAR	38	98.046			Mascot
1612.9016	1612.6653	-0.2363	-147	26	41	IVNTGTVLQVGDGIAR					Mascot
2289.2158	2288.8872	-0.3286	-144	142	162	SVYEPLQTGLIAIDSMIPIGR			Oxidation (M)[16]		Mascot
2804.4609	2804.041	-0.4199	-150	177	202	TAVATDTILNQQGQNVIC			Carbamidomethyl (C)[18]		Mascot
	2804.041					VYVAIGQK					Mascot

Rank	Protein Name	Accession No	Protein MW	Protein PI	Pep. Count	Protein Score	Protein Score C. I. %	Total Ion Score	Total Ion C. I. %
6	ATP synthase subunit alpha, chloroplastic OS=V vinifera GN=atpA PE=3 SV=1	ATPA_VITVI	55409.9	5.26	11	217	100	168	100

Peptide Information

Calc. Mass	Obsrv. Mass	± da	± ppm	Start Seq.	End Seq.	Sequence	Ion Score	C. I. %	Modification	Rank	Result Type
815.4621	815.3492	-0.1129	-138	166	172	ELIIGDR					Mascot
860.5022	860.3828	-0.1194	-139	273	279	QMSLLLR					Mascot
860.5022	860.3828	-0.1194	-139	273	279	QMSLLLR					Mascot
876.4971	876.3705	-0.1266	-144	273	279	QMSLLLR			Oxidation (M)[2]		Mascot
901.485	901.3577	-0.1273	-141	406	413	ATQNQLAR					Mascot
1179.531	1179.4351	-0.0959	-81	492	500	ETIQEQMER			Oxidation (M)[7]		Mascot
1252.726	1252.5487	-0.1773	-142	129	140	LIESPAPGIISR					Mascot
1252.726	1252.5487	-0.1773	-142	129	140	LIESPAPGIISR	28	76.018			Mascot
1266.7205	1266.5808	-0.1397	-110	457	466	FLAELRNYLK					Mascot
1416.7845	1416.5833	-0.2012	-142	95	107	IAQIPVSEAYLGR					Mascot
1416.7845	1416.5833	-0.2012	-142	95	107	IAQIPVSEAYLGR	45	99.579			Mascot
1553.7383	1553.5084	-0.2299	-148	285	297	EAYPGDVFYLHSR					Mascot
1553.7383	1553.5084	-0.2299	-148	285	297	EAYPGDVFYLHSR	57	99.971			Mascot
1612.9016	1612.6653	-0.2363	-147	26	41	IVNTGTVLQVGDGIAR	38	98.046			Mascot
1612.9016	1612.6653	-0.2363	-147	26	41	IVNTGTVLQVGDGIAR					Mascot
2289.2158	2288.8872	-0.3286	-144	142	162	SVYEPLQTGLIAIDSMIPIGR			Oxidation (M)[16]		Mascot
2804.4609	2804.041	-0.4199	-150	177	202	TAVATDTILNQQGQNVICVYVAIGQK			Carbamidomethyl (C)[18]		Mascot

Rank	Protein Name	Accession No	Protein MW	Protein PI	Pep. Count	Protein Score	Protein Score C. I. %	Total Ion Score	Total Ion C. I. %
7	ATP synthase subunit alpha, chloroplastic OS=Gossypium barbadense GN=atpA PE=3 SV=1	ATPA_GOSB	55443.9	5.19	11	217	100	168	100

Peptide Information

Calc. Mass	Obsrv. Mass	± da	± ppm	Start Seq.	End Seq.	Sequence	Ion Score	C. I. %	Modification	Rank	Result Type
860.5022	860.3828	-0.1194	-139	273	279	QMSLLLR					Mascot
860.5022	860.3828	-0.1194	-139	273	279	QMSLLLR					Mascot
876.4971	876.3705	-0.1266	-144	273	279	QMSLLLR			Oxidation (M)[2]		Mascot
901.485	901.3577	-0.1273	-141	406	413	ATQNQLAR					Mascot
933.5152	933.3774	-0.1378	-148	501	507	FRLQEQL					Mascot
1252.726	1252.5487	-0.1773	-142	129	140	LIESPAPGIISR					Mascot
1252.726	1252.5487	-0.1773	-142	129	140	LIESPAPGIISR	28	76.018			Mascot
1317.705	1317.5104	-0.1946	-148	256	266	HTLIIYDDLSK					Mascot
1408.6638	1408.5052	-0.1586	-113	492	502	DAIQDQMERFR					Mascot
1416.7845	1416.5833	-0.2012	-142	95	107	IAQIPVSEAYLGR					Mascot
1416.7845	1416.5833	-0.2012	-142	95	107	IAQIPVSEAYLGR	45	99.579			Mascot
1553.7383	1553.5084	-0.2299	-148	285	297	EAYPGDVFYLHSR					Mascot
1553.7383	1553.5084	-0.2299	-148	285	297	EAYPGDVFYLHSR	57	99.971			Mascot
1612.9016	1612.6653	-0.2363	-147	26	41	IVNTGTVLQVGDGIAR	38	98.046			Mascot
1612.9016	1612.6653	-0.2363	-147	26	41	IVNTGTVLQVGDGIAR					Mascot
2289.2158	2288.8872	-0.3286	-144	142	162	SVYEPLQTGLIAIDSMIPIGR			Oxidation (M)[16]		Mascot
2804.4609	2804.041	-0.4199	-150	177	202	TAVATDTILNQQGQNVICVYVAIGQK			Carbamidomethyl (C)[18]		Mascot

Rank	Protein Name	Accession No	Protein MW	Protein PI	Pep. Count	Protein Score	Protein Score C. I. %	Total Ion Score	Total Ion C. I. %
8	ATP synthase subunit alpha, chloroplastic OS=C arabica GN=atpA PE=3 SV=1	ATPA_COFAI	55233.8	5.19	11	217	100	168	100

Peptide Information

Calc. Mass	Obsrv. Mass	± da	± ppm	Start Seq.	End Seq.	Sequence	Ion Score	C. I. %	Modification	Rank	Result Type
815.4621	815.3492	-0.1129	-138	166	172	ELIIGDR					Mascot
860.5022	860.3828	-0.1194	-139	273	279	QMSLLLR					Mascot
860.5022	860.3828	-0.1194	-139	273	279	QMSLLLR					Mascot
876.4971	876.3705	-0.1266	-144	273	279	QMSLLLR			Oxidation (M)[2]		Mascot
901.485	901.3577	-0.1273	-141	406	413	ATQNQLAR					Mascot
1252.726	1252.5487	-0.1773	-142	129	140	LIESPAPGIISR					Mascot
1252.726	1252.5487	-0.1773	-142	129	140	LIESPAPGIISR	28	76.018			Mascot
1266.7528	1266.5808	-0.172	-136	108	119	VINALAKPIDGR					Mascot
1275.6216	1275.5392	-0.0824	-65	256	266	HTSIIYDDPSK					Mascot
1416.7845	1416.5833	-0.2012	-142	95	107	IAQIPVSEAYLGR					Mascot
1416.7845	1416.5833	-0.2012	-142	95	107	IAQIPVSEAYLGR	45	99.579			Mascot
1553.7383	1553.5084	-0.2299	-148	285	297	EAYPGDVFYLHSR					Mascot
1553.7383	1553.5084	-0.2299	-148	285	297	EAYPGDVFYLHSR	57	99.971			Mascot
1612.9016	1612.6653	-0.2363	-147	26	41	IVNTGTVLQVGDGIAR	38	98.046			Mascot
1612.9016	1612.6653	-0.2363	-147	26	41	IVNTGTVLQVGDGIAR					Mascot
2289.2158	2288.8872	-0.3286	-144	142	162	SVYEPLQTGLIAIDSMIPIGR			Oxidation (M)[16]		Mascot
2804.4609	2804.041	-0.4199	-150	177	202	TAVATDTILNQQGQNVICVYVAIGQK			Carbamidomethyl (C)[18]		Mascot

Rank	Protein Name	Accession No	Protein MW	Protein PI	Pep. Count	Protein Score	Protein Score C. I. %	Total Ion Score	Total Ion C. I. %
9	ATP synthase subunit alpha, chloroplastic OS=Helianthus annuus GN=atpA PE=3 SV=1	ATPA_HELAN	55482.2	5.2	11	217	100	168	100

Peptide Information

Calc. Mass	Obsrv. Mass	± da	± ppm	Start Seq.	End Seq.	Sequence	Ion Score	C. I. %	Modification	Rank	Result Type
815.4621	815.3492	-0.1129	-138	166	172	ELIIGDR					Mascot
860.5022	860.3828	-0.1194	-139	273	279	QMSLLLR					Mascot
860.5022	860.3828	-0.1194	-139	273	279	QMSLLLR					Mascot
876.4971	876.3705	-0.1266	-144	273	279	QMSLLLR			Oxidation (M)[2]		Mascot
901.485	901.3577	-0.1273	-141	406	413	ATQNQLAR					Mascot
1252.726	1252.5487	-0.1773	-142	129	140	LIESPAPGIISR					Mascot
1252.726	1252.5487	-0.1773	-142	129	140	LIESPAPGIISR	28	76.018			Mascot
1266.7528	1266.5808	-0.172	-136	108	119	VINALAKPIDGR					Mascot
1317.705	1317.5104	-0.1946	-148	256	266	HTLIIYDDLSK					Mascot
1416.7845	1416.5833	-0.2012	-142	95	107	IAQIPVSEAYLGR					Mascot
1416.7845	1416.5833	-0.2012	-142	95	107	IAQIPVSEAYLGR	45	99.579			Mascot
1553.7383	1553.5084	-0.2299	-148	285	297	EAYPGDVFYLHSR					Mascot
1553.7383	1553.5084	-0.2299	-148	285	297	EAYPGDVFYLHSR	57	99.971			Mascot
1612.9016	1612.6653	-0.2363	-147	26	41	IVNTGTVLQVGDGIAR	38	98.046			Mascot
1612.9016	1612.6653	-0.2363	-147	26	41	IVNTGTVLQVGDGIAR					Mascot
2289.2158	2288.8872	-0.3286	-144	142	162	SVYEPLQTGLIAIDSMIPIGR			Oxidation (M)[16]		Mascot
2804.4609	2804.041	-0.4199	-150	177	202	TAVATDTILNQQGQNVICVYVAIGQK			Carbamidomethyl (C)[18]		Mascot

Rank	Protein Name	Accession No	Protein MW	Protein PI	Pep. Count	Protein Score	Protein Score C. I. %	Total Ion Score	Total Ion C. I. %
10	ATP synthase subunit alpha, chloroplastic OS=L sativa GN=atpA PE=3 SV=1	ATPA_LACS	55484.2	5.27	11	216	100	168	100

Peptide Information

Calc. Mass	Obsrv. Mass	± da	± ppm	Start Seq.	End Seq.	Sequence	Ion Score	C. I. %	Modification	Rank	Result Type
815.4621	815.3492	-0.1129	-138	166	172	ELIIGDR					Mascot
860.5022	860.3828	-0.1194	-139	273	279	QMSLLLR					Mascot
860.5022	860.3828	-0.1194	-139	273	279	QMSLLLR					Mascot
876.4971	876.3705	-0.1266	-144	273	279	QMSLLLR			Oxidation (M)[2]		Mascot
901.485	901.3577	-0.1273	-141	406	413	ATQNQLAR					Mascot
1252.726	1252.5487	-0.1773	-142	129	140	LIESPAPGIISR					Mascot
1252.726	1252.5487	-0.1773	-142	129	140	LIESPAPGIISR	28	76.018			Mascot
1266.7528	1266.5808	-0.172	-136	108	119	VINALAKPIDGR					Mascot
1275.6216	1275.5392	-0.0824	-65	256	266	HTSIIYDDPSK					Mascot
1416.7845	1416.5833	-0.2012	-142	95	107	IAQIPVSEAYLGR					Mascot
1416.7845	1416.5833	-0.2012	-142	95	107	IAQIPVSEAYLGR	45	99.579			Mascot
1535.7812	1535.5581	-0.2231	-145	203	216	ASSVAQVVTNFQER					Mascot
1553.7383	1553.5084	-0.2299	-148	285	297	EAYPGDVFYLHSR					Mascot
1553.7383	1553.5084	-0.2299	-148	285	297	EAYPGDVFYLHSR	57	99.975			Mascot
1612.9016	1612.6653	-0.2363	-147	26	41	IVNTGTVLQVGDGIAR	38	98.333			Mascot
1612.9016	1612.6653	-0.2363	-147	26	41	IVNTGTVLQVGDGIAR					Mascot
2289.2158	2288.8872	-0.3286	-144	142	162	SVYEPLQTGLIAIDSMIPIGR			Oxidation (M)[16]		Mascot

Gel Idx/Pos	53/C2	Instr./Gel Origin	BA2060/131017A	Process Sta	Analysis Succeeded
Plate [#] Name	[1] 31496	Instrument Sample Name		Spectra	11

Rank	Protein Name	Accession No	Protein MW	Protein PI	Pep. Count	Protein Score	Protein Score C. I. %	Total Ion Score	Total Ion C. I. %
1	Ribulose bisphosphate carboxylase large chain (Fragment) OS=Fragaria ananassa GN=rbcL PE=3 SV=1	RBL_FRAAN	51628.9	6.04	13	72	99.773	4	0

Peptide Information

Calc. Mass	Obsrv. Mass	± da	± ppm	Start Seq.	End Seq.	Sequence	Ion Score	C. I. %	Modification	Rank	Result Type
914.4049	914.2725	-0.1324	-145	296	302	NHGMHFR			Oxidation (M)[4]		Mascot
1021.5312	1021.3925	-0.1387	-136	23	31	DTDILAAFR					Mascot
1102.4762	1102.3827	-0.0935	-85	457	465	FEFEAMDTL					Mascot
1170.5585	1170.4706	-0.0879	-75	294	302	QKNHGMHFR			Oxidation (M)[6]		Mascot
1187.6643	1187.4915	-0.1728	-145	276	285	DNGLLLHIHR					Mascot
1275.7307	1275.5498	-0.1809	-142	330	340	EITLGFVDLLR					Mascot
1275.7307	1275.5498	-0.1809	-142	330	340	EITLGFVDLLR					Mascot
1465.7216	1465.5438	-0.1778	-121	310	324	MSGGDHIHAGTVVGK					Mascot
1465.7546	1465.5438	-0.2108	-144	137	149	TFQGPPHGIQVER	6	0			Mascot
1475.7351	1475.5553	-0.1798	-122	206	217	DRFLFCAEAIYK					Mascot
1546.7358	1546.5176	-0.2182	-141	441	453	WSPELAAACEVWK			Carbamidomethyl (C)[9]		Mascot
1920.8982	1920.7629	-0.1353	-70	437	453	EACKWSPELAAACEVWK					Mascot
2034.9412	2034.754	-0.1872	-92	437	453	EACKWSPELAAACEVWK			Carbamidomethyl (C)[3,13]		Mascot
2169.9868	2169.6792	-0.3076	-142	185	203	GGLDFTKDDENVNSQPFMR					Mascot
3854.8718	3854.3123	-0.5595	-145	32	69	VTPQPGVPPEEAGAAVAAESSTGTWTTVWTDGLTSLDR					Mascot

Rank	Protein Name	Accession No	Protein MW	Protein PI	Pep. Count	Protein Score	Protein Score C. I. %	Total Ion Score	Total Ion C. I. %
2	Ribulose bisphosphate carboxylase large chain (Fragment) OS=Hamamelis mollis GN=rbcL PE=3 SV=1	RBL_HAMMO	51542.9	6.05	13	71	99.727	4	0

Peptide Information

Calc. Mass	Obsrv. Mass	± da	± ppm	Start Seq.	End Seq.	Sequence	Ion Score	C. I. %	Modification	Rank	Result Type
914.4049	914.2725	-0.1324	-145	296	302	NHGMHFR			Oxidation (M)[4]		Mascot
1021.5312	1021.3925	-0.1387	-136	23	31	DTDILAAFR					Mascot
1102.4762	1102.3827	-0.0935	-85	457	465	FEFEAMDTL					Mascot
1170.5585	1170.4706	-0.0879	-75	294	302	QKNHGMHFR			Oxidation (M)[6]		Mascot
1187.6643	1187.4915	-0.1728	-145	276	285	DNGLLLHIHR					Mascot
1245.6433	1245.4615	-0.1818	-146	430	440	EGNEIIREASK					Mascot
1275.7307	1275.5498	-0.1809	-142	330	340	EITLGFVDLLR					Mascot
1275.7307	1275.5498	-0.1809	-142	330	340	EITLGFVDLLR					Mascot
1465.7216	1465.5438	-0.1778	-121	310	324	MSGGDHIHAGTVVGK					Mascot
1465.7546	1465.5438	-0.2108	-144	137	149	TFQGPPHGIQVER	6	0			Mascot
1475.7351	1475.5553	-0.1798	-122	206	217	DRFLFCAEAIYK					Mascot
1546.7358	1546.5176	-0.2182	-141	441	453	WSPELAAACEVWK			Carbamidomethyl (C)[9]		Mascot
2169.9868	2169.6792	-0.3076	-142	185	203	GGLDFTKDDENVNSQPFMR					Mascot
3854.8718	3854.3123	-0.5595	-145	32	69	VTPQPGVPPEEAGAAVAAESSTGTWTTVWTDGLTSLDR					Mascot

Rank	Protein Name	Accession No	Protein MW	Protein PI	Pep. Count	Protein Score	Protein Score C. I. %	Total Ion Score	Total Ion C. I. %
3	Ribulose bisphosphate carboxylase large chain OS=Liquidambar styraciflua GN=rbcL PE=3 SV=1	RBL_LIQST	52625.5	6.04	13	69	99.536	4	0

Peptide Information

Calc. Mass	Obsrv. Mass	± da	± ppm	Start Seq.	End Seq.	Sequence	Ion Score	C. I. %	Modification	Rank	Result Type
914.4049	914.2725	-0.1324	-145	306	312	NHGMHFR			Oxidation (M)[4]		Mascot
1021.5312	1021.3925	-0.1387	-136	33	41	DTDILAAFR					Mascot
1102.4762	1102.3827	-0.0935	-85	467	475	FEFEAMDTL					Mascot
1170.5585	1170.4706	-0.0879	-75	304	312	QKNHGMHFR			Oxidation (M)[6]		Mascot
1187.6643	1187.4915	-0.1728	-145	286	295	DNGLLLHIHR					Mascot
1245.6433	1245.4615	-0.1818	-146	440	450	EGNEIIREASK					Mascot
1275.7307	1275.5498	-0.1809	-142	340	350	EITLGFVDLLR					Mascot
1275.7307	1275.5498	-0.1809	-142	340	350	EITLGFVDLLR					Mascot
1465.7216	1465.5438	-0.1778	-121	320	334	MSGGDHIHAGTVVGK					Mascot
1465.7546	1465.5438	-0.2108	-144	147	159	TFQGPPHGIQVER	6	0			Mascot
1475.7351	1475.5553	-0.1798	-122	216	227	DRFLFCAEAIYK					Mascot
1546.7358	1546.5176	-0.2182	-141	451	463	WSPELAAACEVWK			Carbamidomethyl (C)[9]		Mascot
2169.9868	2169.6792	-0.3076	-142	195	213	GGLDFTKDDENVNSQPFMR					Mascot
3854.8718	3854.3123	-0.5595	-145	42	79	VTPQPGVPPEEAGAAVAAESSTGTWTTVWTDGLTSLDR					Mascot

Rank	Protein Name	Accession No	Protein MW	Protein PI	Pep. Count	Protein Score	Protein Score C. I. %	Total Ion Score	Total Ion C. I. %
4	Ribulose bisphosphate carboxylase large chain (Fragment) OS=Sinapis alba GN=rbcL PE=3 SV=1	RBL_SINAL	52405.4	6.23	12	64	98.531	6	0

Peptide Information

Calc. Mass	Obsrv. Mass	± da	± ppm	Start Seq.	End Seq.	Sequence	Ion Score	C. I. %	Modification	Rank	Result Type
856.3546	856.4097	0.0551	64	467	473	FEFPAMD					Mascot
914.4049	914.2725	-0.1324	-145	306	312	NHGMHFR			Oxidation (M)[4]		Mascot
1021.5312	1021.3925	-0.1387	-136	33	41	DTDILAAFR					Mascot
1170.5585	1170.4706	-0.0879	-75	304	312	QKNHGMHFR			Oxidation (M)[6]		Mascot
1187.6643	1187.4915	-0.1728	-145	286	295	DNGLLLHIHR					Mascot
1245.6433	1245.4615	-0.1818	-146	440	450	EGNEIIREASK					Mascot
1275.7307	1275.5498	-0.1809	-142	340	350	EITLGFVDLLR					Mascot
1275.7307	1275.5498	-0.1809	-142	340	350	EITLGFVDLLR					Mascot
1465.7546	1465.5438	-0.2108	-144	147	159	TFQGPPHGIQVER	6	0			Mascot
1465.7546	1465.5438	-0.2108	-144	147	159	TFQGPPHGIQVER					Mascot
1475.7351	1475.5553	-0.1798	-122	216	227	DRFLFCAEAIYK					Mascot
1546.7358	1546.5176	-0.2182	-141	451	463	WSPELAAACEVWK			Carbamidomethyl (C)[9]		Mascot
2169.9868	2169.6792	-0.3076	-142	195	213	GGLDFTKDDENVNSQPFMR					Mascot
3854.8718	3854.3123	-0.5595	-145	42	79	VTPQPGVPPEEAGAAVAAESSTGTWTTVWTDGLTSLDR					Mascot

Rank	Protein Name	Accession No	Protein MW	Protein PI	Pep. Count	Protein Score	Protein Score C. I. %	Total Ion Score	Total Ion C. I. %
5	Ribulose bisphosphate carboxylase large chain (Fragment) OS=Prunus domestica GN=rbcL PE=3 SV=1	RBL_PRUDO	50914.6	6.23	12	63	98.39	4	0

Peptide Information

Calc. Mass	Obsrv. Mass	± da	± ppm	Start Seq.	End Seq.	Sequence	Ion Score	C. I. %	Modification	Rank	Result Type
914.4049	914.2725	-0.1324	-145	296	302	NHGMHFR			Oxidation (M)[4]		Mascot
1021.5312	1021.3925	-0.1387	-136	23	31	DTDILAAFR					Mascot
1170.5585	1170.4706	-0.0879	-75	294	302	QKNHGMHFR			Oxidation (M)[6]		Mascot
1187.6643	1187.4915	-0.1728	-145	276	285	DNGLLLHIHR					Mascot
1245.6433	1245.4615	-0.1818	-146	430	440	EGNEIIREASK					Mascot
1275.7307	1275.5498	-0.1809	-142	330	340	EITLGFVDLLR					Mascot
1275.7307	1275.5498	-0.1809	-142	330	340	EITLGFVDLLR					Mascot
1465.7216	1465.5438	-0.1778	-121	310	324	MSGGDHIHAGTVVGK					Mascot
1465.7546	1465.5438	-0.2108	-144	137	149	TFQGPPHGIQVER	6	0			Mascot
1475.7351	1475.5553	-0.1798	-122	206	217	DRFLFCAEAIYK					Mascot
1546.7358	1546.5176	-0.2182	-141	441	453	WSPELAAACEVWK			Carbamidomethyl (C)[9]		Mascot
2169.9868	2169.6792	-0.3076	-142	185	203	GGLDFTKDDENVNSQPFMR					Mascot
3854.8718	3854.3123	-0.5595	-145	32	69	VTPQPGVPPEEAGAAVAAESSTGTWTTVWTDGLTSLDR					Mascot

Rank	Protein Name	Accession No	Protein MW	Protein PI	Pep. Count	Protein Score	Protein Score C. I. %	Total Ion Score	Total Ion C. I. %
6	Ribulose bisphosphate carboxylase large chain (Fragment) OS=Ajuga chamaepitys GN=rbcL PE=3 SV=1	RBL_AJUCH	52421.4	6.23	12	63	98.234	6	0

Peptide Information

Calc. Mass	Obsrv. Mass	± da	± ppm	Start Seq.	End Seq.	Sequence	Ion Score	C. I. %	Modification	Rank	Result Type
856.3546	856.4097	0.0551	64	467	473	FEFPAMD					Mascot
914.4049	914.2725	-0.1324	-145	306	312	NHGMHFR			Oxidation (M)[4]		Mascot
1021.5312	1021.3925	-0.1387	-136	33	41	DTDILAAFR					Mascot
1170.5585	1170.4706	-0.0879	-75	304	312	QKNHGMHFR			Oxidation (M)[6]		Mascot
1187.6643	1187.4915	-0.1728	-145	286	295	DNGLLLHIHR					Mascot
1275.7307	1275.5498	-0.1809	-142	340	350	EITLGFVDLLR					Mascot
1275.7307	1275.5498	-0.1809	-142	340	350	EITLGFVDLLR					Mascot
1465.7546	1465.5438	-0.2108	-144	147	159	TFQGPPHGIQVER	6	0			Mascot
1465.7546	1465.5438	-0.2108	-144	147	159	TFQGPPHGIQVER					Mascot
1475.7351	1475.5553	-0.1798	-122	216	227	DRFLFCAEAIYK					Mascot
1546.7358	1546.5176	-0.2182	-141	451	463	WSPELAAACEVWK			Carbamidomethyl (C)[9]		Mascot
1920.8982	1920.7629	-0.1353	-70	447	463	EACKWSPELAAACEVWK					Mascot
2034.9412	2034.754	-0.1872	-92	447	463	EACKWSPELAAACEVWK			Carbamidomethyl (C)[3,13]		Mascot
2169.9868	2169.6792	-0.3076	-142	195	213	GGLDFTKDDENVNSQPFMR					Mascot
3854.8718	3854.3123	-0.5595	-145	42	79	VTPQPGVPPEEAGAAVAAESSTGTWTTVWTDGLTSLDR					Mascot

Rank	Protein Name	Accession No	Protein MW	Protein PI	Pep. Count	Protein Score	Protein Score C. I. %	Total Ion Score	Total Ion C. I. %
7	Ribulose bisphosphate carboxylase large chain (Fragment) OS=Morus rubra GN=rbcL PE=3 SV=1	RBL_MORRU	51574	6	12	63	98.234	4	0

Peptide Information

Calc. Mass	Obsrv. Mass	± da	± ppm	Start Seq.	End Seq.	Sequence	Ion Score	C. I. %	Modification	Rank	Result Type
914.4049	914.2725	-0.1324	-145	296	302	NHGMHFR			Oxidation (M)[4]		Mascot
1021.5312	1021.3925	-0.1387	-136	23	31	DTDILAAFR					Mascot
1102.4762	1102.3827	-0.0935	-85	457	465	FEFEAMDTL					Mascot
1170.5585	1170.4706	-0.0879	-75	294	302	QKNHGMHFR			Oxidation (M)[6]		Mascot
1187.6643	1187.4915	-0.1728	-145	276	285	DNGLLLHIHR					Mascot
1275.7307	1275.5498	-0.1809	-142	330	340	EITLGFVDLLR					Mascot
1275.7307	1275.5498	-0.1809	-142	330	340	EITLGFVDLLR					Mascot
1465.7216	1465.5438	-0.1778	-121	310	324	MSGGDHIHAGTVVGK					Mascot
1465.7546	1465.5438	-0.2108	-144	137	149	TFQGPPHGIQVER	6	0			Mascot
1475.7351	1475.5553	-0.1798	-122	206	217	DRFLFCAEAIYK					Mascot
1546.7358	1546.5176	-0.2182	-141	441	453	WSPELAAACEVWK			Carbamidomethyl (C)[9]		Mascot
2169.9868	2169.6792	-0.3076	-142	185	203	GGLDFTKDDENVNSQPFMR					Mascot
3854.8718	3854.3123	-0.5595	-145	32	69	VTPQPGVPPEEAGAAVAAESSTGTWTTVWTDGLTSLDR					Mascot

Rank	Protein Name	Accession No	Protein MW	Protein PI	Pep. Count	Protein Score	Protein Score C. I. %	Total Ion Score	Total Ion C. I. %
8	Ribulose bisphosphate carboxylase large chain (Fragment) OS=Byrsonima crassifolia GN=rbcL PE=3 SV=1	RBL_BYRCR	51554.9	6.09	12	63	98.193	4	0

Peptide Information

Calc. Mass	Obsrv. Mass	± da	± ppm	Start Seq.	End Seq.	Sequence	Ion Score	C. I. %	Modification	Rank	Result Type
914.4049	914.2725	-0.1324	-145	296	302	NHGMHFR			Oxidation (M)[4]		Mascot
1021.5312	1021.3925	-0.1387	-136	23	31	DTDILAAFR					Mascot
1170.5585	1170.4706	-0.0879	-75	294	302	QKNHGMHFR			Oxidation (M)[6]		Mascot
1187.6643	1187.4915	-0.1728	-145	276	285	DNGLLLHIHR					Mascot
1245.6433	1245.4615	-0.1818	-146	430	440	EGNEIIREASK					Mascot
1275.7307	1275.5498	-0.1809	-142	330	340	EITLGFVDLLR					Mascot
1275.7307	1275.5498	-0.1809	-142	330	340	EITLGFVDLLR					Mascot
1465.7216	1465.5438	-0.1778	-121	310	324	MSGGDHIHAGTVVGK					Mascot
1465.7546	1465.5438	-0.2108	-144	137	149	TFQGPPHGIQVER	6	0			Mascot
1475.7351	1475.5553	-0.1798	-122	206	217	DRFLFCAEAIYK					Mascot
1546.7358	1546.5176	-0.2182	-141	441	453	WSPELAAACEVWK			Carbamidomethyl (C)[9]		Mascot
2169.9868	2169.6792	-0.3076	-142	185	203	GGLDFTKDDENVNSQPFMR					Mascot
3854.8718	3854.3123	-0.5595	-145	32	69	VTPQPGVPPEEAGAAVAAESSTGTWTTVWTDGLTSLDR					Mascot

Rank	Protein Name	Accession No	Protein MW	Protein PI	Pep. Count	Protein Score	Protein Score C. I. %	Total Ion Score	Total Ion C. I. %
9	Ribulose bisphosphate carboxylase large chain (Fragment) OS=Saururus cernuus GN=rbcL PE=3 SV=1	RBL_SAUCE	51663	6	12	63	98.193	4	0

Peptide Information

Calc. Mass	Obsrv. Mass	± da	± ppm	Start Seq.	End Seq.	Sequence	Ion Score	C. I. %	Modification	Rank	Result Type
914.4049	914.2725	-0.1324	-145	297	303	NHGMHFR			Oxidation (M)[4]		Mascot
1021.5312	1021.3925	-0.1387	-136	24	32	DTDILAAFR					Mascot
1102.4762	1102.3827	-0.0935	-85	458	466	FEFEAMDTL					Mascot
1170.5585	1170.4706	-0.0879	-75	295	303	QKNHGMHFR			Oxidation (M)[6]		Mascot
1187.6643	1187.4915	-0.1728	-145	277	286	DNGLLLHIHR					Mascot
1275.7307	1275.5498	-0.1809	-142	331	341	EITLGFVDLLR					Mascot
1275.7307	1275.5498	-0.1809	-142	331	341	EITLGFVDLLR					Mascot
1465.7216	1465.5438	-0.1778	-121	311	325	MSGGDHIHAGTVVGK					Mascot
1465.7546	1465.5438	-0.2108	-144	138	150	TFQGPPHGIQVER	6	0			Mascot
1475.7351	1475.5553	-0.1798	-122	207	218	DRFLFCAEAIYK					Mascot
1546.7358	1546.5176	-0.2182	-141	442	454	WSPELAAACEVWK			Carbamidomethyl (C)[9]		Mascot
2169.9868	2169.6792	-0.3076	-142	186	204	GGLDFTKDDENVNSQPFMR					Mascot
3854.8718	3854.3123	-0.5595	-145	33	70	VTPQPGVPPEEAGAAVAAESSTGTWTTVWTDGLTSLDR					Mascot

Rank	Protein Name	Accession No	Protein MW	Protein PI	Pep. Count	Protein Score	Protein Score C. I. %	Total Ion Score	Total Ion C. I. %
10	Ribulose bisphosphate carboxylase large chain OS=Morus indica GN=rbcL PE=3 SV=1	RBL_MORIN	52634.5	6	12	59	96.047	4	0

Peptide Information

Calc. Mass	Obsrv. Mass	± da	± ppm	Start Seq.	End Seq.	Sequence	Ion Score	C. I. %	Modification	Rank	Result Type
914.4049	914.2725	-0.1324	-145	306	312	NHGMHFR			Oxidation (M)[4]		Mascot
1021.5312	1021.3925	-0.1387	-136	33	41	DTDILAAFR					Mascot
1102.4762	1102.3827	-0.0935	-85	467	475	FEFEAMDTL					Mascot
1170.5585	1170.4706	-0.0879	-75	304	312	QKNHGMHFR			Oxidation (M)[6]		Mascot
1187.6643	1187.4915	-0.1728	-145	286	295	DNGLLLHIHR					Mascot
1275.7307	1275.5498	-0.1809	-142	340	350	EITLGFVDLLR					Mascot
1275.7307	1275.5498	-0.1809	-142	340	350	EITLGFVDLLR					Mascot
1465.7216	1465.5438	-0.1778	-121	320	334	MSGGDHIHAGTVVGK					Mascot
1465.7546	1465.5438	-0.2108	-144	147	159	TFQGPPHGIQVER	6	0			Mascot
1475.7351	1475.5553	-0.1798	-122	216	227	DRFLFCAEAIYK					Mascot
1546.7358	1546.5176	-0.2182	-141	451	463	WSPELAAACEVWK			Carbamidomethyl (C)[9]		Mascot
2169.9868	2169.6792	-0.3076	-142	195	213	GGLDFTKDDENVNSQPFMR					Mascot
3854.8718	3854.3123	-0.5595	-145	42	79	VTPQPGVPPEEAGAAVAAESSTGTWTTVWTDGLTSLDR					Mascot

Gel Idx/Pos	54/C3	Instr./Gel Origin	BA2060/131017A	Process Sta	Analysis Succeeded
Plate [#] Name	[1] 31496	Instrument Sample Name		Spectra	11

Rank	Protein Name	Accession No	Protein MW	Protein PI	Pep. Count	Protein Score	Protein Score C. I. %	Total Ion Score	Total Ion C. I. %
1	Ribulose bisphosphate carboxylase large chain OS=Cajanus cajan GN=rbcL PE=3 SV=1	RBL_CAJCA	52358.5	6.45	13	71	99.745	4	0

Peptide Information

Calc. Mass	Obsrv. Mass	± da	± ppm	Start Seq.	End Seq.	Sequence	Ion Score	C. I. %	Modification	Rank	Result Type
830.4366	830.327	-0.1096	-132	440	446	EGNEIIR					Mascot
856.3546	856.4129	0.0583	68	467	473	FEFPAMD					Mascot
856.3546	856.4129	0.0583	68	467	473	FEFPAMD					Mascot
910.4451	910.3222	-0.1229	-135	188	194	AVYECLR			Carbamidomethyl (C)[5]		Mascot
912.4719	912.3467	-0.1252	-137	296	303	AMHAVIDR					Mascot
914.4049	914.279	-0.1259	-138	306	312	NHGMHFR			Oxidation (M)[4]		Mascot
914.4049	914.4119	0.007	8	306	312	NHGMHFR			Oxidation (M)[4]		Mascot
921.4346	921.3864	-0.0482	-52	1	8	MSPQTETK					Mascot
928.4669	928.3407	-0.1262	-136	296	303	AMHAVIDR			Oxidation (M)[2]		Mascot
1021.5312	1021.3923	-0.1389	-136	33	41	DTDILAAFR					Mascot
1023.4741	1023.3705	-0.1036	-101	351	358	DDFVEKDR					Mascot
1116.583	1116.4167	-0.1663	-149	422	431	VALEACVQAR			Carbamidomethyl (C)[6]		Mascot
1170.5585	1170.4843	-0.0742	-63	304	312	QKNHGMHFR			Oxidation (M)[6]		Mascot
1187.6643	1187.5024	-0.1619	-136	286	295	DNGLLLHIHR					Mascot
1275.7307	1275.5546	-0.1761	-138	340	350	EITLGFVDLLR					Mascot
1465.7546	1465.5499	-0.2047	-140	147	159	TFQGPPHGIQVER	4	0			Mascot
1465.7546	1465.5499	-0.2047	-140	147	159	TFQGPPHGIQVER					Mascot

Rank	Protein Name	Accession No	Protein MW	Protein PI	Pep. Count	Protein Score	Protein Score C. I. %	Total Ion Score	Total Ion C. I. %
2	Ribulose bisphosphate carboxylase large chain OS=Glycine max GN=rbcL PE=1 SV=3	RBL_SOYBN	52576.4	6	13	70	99.664	4	0

Peptide Information

Calc. Mass	Obsrv. Mass	± da	± ppm	Start Seq.	End Seq.	Sequence	Ion Score	C. I. %	Modification	Rank	Result Type
830.4366	830.327	-0.1096	-132	440	446	EGNEIIR					Mascot
910.4451	910.3222	-0.1229	-135	188	194	AVYECLR			Carbamidomethyl (C)[5]		Mascot
912.4719	912.3467	-0.1252	-137	296	303	AMHAVIDR					Mascot
914.4049	914.279	-0.1259	-138	306	312	NHGMHFR			Oxidation (M)[4]		Mascot
914.4049	914.4119	0.007	8	306	312	NHGMHFR			Oxidation (M)[4]		Mascot
921.4346	921.3864	-0.0482	-52	1	8	MSPQTETK					Mascot
928.4669	928.3407	-0.1262	-136	296	303	AMHAVIDR			Oxidation (M)[2]		Mascot
1021.5312	1021.3923	-0.1389	-136	33	41	DTDILAAFR					Mascot
1023.4741	1023.3705	-0.1036	-101	351	358	DDFVEKDR					Mascot
1116.583	1116.4167	-0.1663	-149	422	431	VALEACVQAR			Carbamidomethyl (C)[6]		Mascot
1170.5585	1170.4843	-0.0742	-63	304	312	QKNHGMHFR			Oxidation (M)[6]		Mascot
1187.6643	1187.5024	-0.1619	-136	286	295	DNGLLLHIHR					Mascot
1275.7307	1275.5546	-0.1761	-138	340	350	EITLGFVDLLR					Mascot
1433.7495	1433.5551	-0.1944	-136	320	334	LSGGDHVHAGTVVGK					Mascot
1465.7546	1465.5499	-0.2047	-140	147	159	TFQGPPHGIQVER	4	0			Mascot
1465.7546	1465.5499	-0.2047	-140	147	159	TFQGPPHGIQVER					Mascot

Rank	Protein Name	Accession No	Protein MW	Protein PI	Pep. Count	Protein Score	Protein Score C. I. %	Total Ion Score	Total Ion C. I. %
3	Ribulose bisphosphate carboxylase/oxygenase a(2, chloroplastic OS=Nicotiana tabacum GN=RCA P	RCA2_TOBA(	48312.5	8.14	8	69	99.576	41	98.993

Peptide Information

Calc. Mass	Obsrv. Mass	± da	± ppm	Start Seq.	End Seq.	Sequence	Ion Score	C. I. %	Modification	Rank	Result Type
895.4155	895.2964	-0.1191	-133	356	362	VYDDEVR					Mascot
940.4675	940.3447	-0.1228	-131	307	313	FYWAPTR	17	0			Mascot
940.4675	940.3447	-0.1228	-131	307	313	FYWAPTR					Mascot
976.488	976.3574	-0.1306	-134	314	321	EDRIGVCK			Carbamidomethyl (C)[7]		Mascot
1023.5105	1023.3705	-0.14	-137	356	363	VYDDEVRK					Mascot
1083.5219	1083.4474	-0.0745	-69	69	76	QTYSDRWK					Mascot
1083.5219	1083.4474	-0.0745	-69	69	76	QTYSDRWK					Mascot
1882.9697	1882.7028	-0.2669	-142	337	353	IVDTFPGQSIDFFGALR					Mascot
1882.9697	1882.7028	-0.2669	-142	337	353	IVDTFPGQSIDFFGALR	24	47.037			Mascot
2063.0591	2062.7734	-0.2857	-138	397	413	LLEYGNMLVQEQENVKR					Mascot
2079.054	2078.7671	-0.2869	-138	397	413	LLEYGNMLVQEQENVKR			Oxidation (M)[7]		Mascot
2089.1692	2088.8682	-0.301	-144	282	300	VPIIVTGNDFSTLYAPLIR					Mascot
2089.1692	2088.8682	-0.301	-144	282	300	VPIIVTGNDFSTLYAPLIR					Mascot

Rank	Protein Name	Accession No	Protein MW	Protein PI	Pep. Count	Protein Score	Protein Score C. I. %	Total Ion Score	Total Ion C. I. %
4	Ribulose bisphosphate carboxylase large chain OS=Lotus japonicus GN=rbcL PE=3 SV=1	RBL_LOTJA	52739.5	6.04	13	67	99.329	3	0

Peptide Information

Calc. Mass	Obsrv. Mass	± da	± ppm	Start Seq.	End Seq.	Sequence	Ion Score	C. I. %	Modification	Rank	Result Type
830.4366	830.327	-0.1096	-132	440	446	EGNEIIR					Mascot
910.4451	910.3222	-0.1229	-135	188	194	AVYECLR			Carbamidomethyl (C)[5]		Mascot
912.4719	912.3467	-0.1252	-137	296	303	AMHAVIDR					Mascot
914.4049	914.279	-0.1259	-138	306	312	NHGMHFR			Oxidation (M)[4]		Mascot
914.4049	914.4119	0.007	8	306	312	NHGMHFR			Oxidation (M)[4]		Mascot
921.4346	921.3864	-0.0482	-52	1	8	MSPQTETK					Mascot
928.4669	928.3407	-0.1262	-136	296	303	AMHAVIDR			Oxidation (M)[2]		Mascot
1021.5312	1021.3923	-0.1389	-136	33	41	DTDILAAFR					Mascot
1023.4741	1023.3705	-0.1036	-101	351	358	DDFVEKDR					Mascot
1116.583	1116.4167	-0.1663	-149	422	431	VALEACVQAR			Carbamidomethyl (C)[6]		Mascot
1170.5585	1170.4843	-0.0742	-63	304	312	QKNHGMHFR			Oxidation (M)[6]		Mascot
1187.6643	1187.5024	-0.1619	-136	286	295	DNGLLLHIHR					Mascot
1275.7307	1275.5546	-0.1761	-138	340	350	EITLGFVDLLR					Mascot
1465.7216	1465.5499	-0.1717	-117	320	334	MSGGDHIHAGTVVGK					Mascot
1465.7546	1465.5499	-0.2047	-140	147	159	TFQGPPHGIQVER	4	0			Mascot

Rank	Protein Name	Accession No	Protein MW	Protein PI	Pep. Count	Protein Score	Protein Score C. I. %	Total Ion Score	Total Ion C. I. %
5	Ribulose bisphosphate carboxylase/oxygenase activase, chloroplastic OS=Vigna radiata var. radiata G	RCA_VIGRR	47871.3	7.57	8	66	99.211	41	98.993

Peptide Information

Calc. Mass	Obsrv. Mass	± da	± ppm	Start Seq.	End Seq.	Sequence	Ion Score	C. I. %	Modification	Rank	Result Type
895.4155	895.2964	-0.1191	-133	354	360	VYDDEVR					Mascot
940.4675	940.3447	-0.1228	-131	305	311	FYWAPTR	17	0			Mascot
940.4675	940.3447	-0.1228	-131	305	311	FYWAPTR					Mascot
1023.5105	1023.3705	-0.14	-137	354	361	VYDDEVRK					Mascot
1115.5367	1115.3959	-0.1408	-126	380	389	EGPPTFDQPK					Mascot
1623.7617	1623.5529	-0.2088	-129	224	238	MCALFINDLDAGAGR			Carbamidomethyl (C)[2]		Mascot
1639.7567	1639.5397	-0.217	-132	224	238	MCALFINDLDAGAGR			Carbamidomethyl (C)[2], Oxidation (M)[1]		Mascot
1709.7977	1709.5845	-0.2132	-125	77	91	GLAYDISDDQQDITR					Mascot
1882.9697	1882.7028	-0.2669	-142	335	351	LVDTFPGQSIDFFGALR					Mascot
1882.9697	1882.7028	-0.2669	-142	335	351	LVDTFPGQSIDFFGALR	24	47.037			Mascot
2089.1692	2088.8682	-0.301	-144	280	298	VPIIVTGNDFSTLYAPLIR					Mascot
2089.1692	2088.8682	-0.301	-144	280	298	VPIIVTGNDFSTLYAPLIR					Mascot

Rank	Protein Name	Accession No	Protein MW	Protein PI	Pep. Count	Protein Score	Protein Score C. I. %	Total Ion Score	Total Ion C. I. %
6	Ribulose bisphosphate carboxylase large chain (Fragment) OS=Sanguinaria canadensis GN=rbcL PE=3 SV=	RBL_SANCA	48270.4	6.46	12	63	98.314	3	0

Peptide Information

Calc. Mass	Obsrv. Mass	± da	± ppm	Start Seq.	End Seq.	Sequence	Ion Score	C. I. %	Modification	Rank	Result Type
Calc. Mass	Obsrv. Mass	± da	± ppm	Start Seq.	End Seq.	Sequence	Ion Score	C. I. %	Modification	Rank	Result Type
830.4366	830.327	-0.1096	-132	430	436	EGNEIIR					Mascot
910.4451	910.3222	-0.1229	-135	178	184	AVYECLR			Carbamidomethyl (C)[5]		Mascot
912.4719	912.3467	-0.1252	-137	286	293	AMHAVIDR					Mascot
914.4049	914.279	-0.1259	-138	296	302	NHGMHFR			Oxidation (M)[4]		Mascot
914.4049	914.4119	0.007	8	296	302	NHGMHFR			Oxidation (M)[4]		Mascot
928.4669	928.3407	-0.1262	-136	286	293	AMHAVIDR			Oxidation (M)[2]		Mascot
1021.5312	1021.3923	-0.1389	-136	23	31	DTDILAAFR					Mascot
1023.4741	1023.3705	-0.1036	-101	341	348	DDFVEKDR					Mascot
1116.583	1116.4167	-0.1663	-149	412	421	VALEACVQAR			Carbamidomethyl (C)[6]		Mascot
1170.5585	1170.4843	-0.0742	-63	294	302	QKNHGMHFR			Oxidation (M)[6]		Mascot
1187.6643	1187.5024	-0.1619	-136	276	285	DNGLLLHIHR					Mascot
1275.7307	1275.5546	-0.1761	-138	330	340	EITLGFVDLLR					Mascot
1465.7216	1465.5499	-0.1717	-117	310	324	MSGGDHIHAGTVVGK					Mascot
1465.7546	1465.5499	-0.2047	-140	137	149	TFQGPPHGIQVER	4	0			Mascot

Rank	Protein Name	Accession No	Protein MW	Protein PI	Pep. Count	Protein Score	Protein Score C. I. %	Total Ion Score	Total Ion C. I. %
7	Ribulose bisphosphate carboxylase large chain (Fragment) OS=Sinapis alba GN=rbcL PE=3 SV=1	RBL_SINAL	52405.4	6.23	12	63	98.193	4	0

Peptide Information

Calc. Mass	Obsrv. Mass	± da	± ppm	Start Seq.	End Seq.	Sequence	Ion Score	C. I. %	Modification	Rank	Result Type
830.4366	830.327	-0.1096	-132	440	446	EGNEIIR					Mascot
856.3546	856.4129	0.0583	68	467	473	FEFPAMD					Mascot
856.3546	856.4129	0.0583	68	467	473	FEFPAMD					Mascot
910.4451	910.3222	-0.1229	-135	188	194	AVYECLR			Carbamidomethyl (C)[5]		Mascot
912.4719	912.3467	-0.1252	-137	296	303	AMHAVIDR					Mascot
914.4049	914.279	-0.1259	-138	306	312	NHGMHFR			Oxidation (M)[4]		Mascot
914.4049	914.4119	0.007	8	306	312	NHGMHFR			Oxidation (M)[4]		Mascot
921.4346	921.3864	-0.0482	-52	1	8	MSPQTETK					Mascot
928.4669	928.3407	-0.1262	-136	296	303	AMHAVIDR			Oxidation (M)[2]		Mascot
1021.5312	1021.3923	-0.1389	-136	33	41	DTDILAAFR					Mascot
1116.583	1116.4167	-0.1663	-149	422	431	VALEACVQAR			Carbamidomethyl (C)[6]		Mascot
1170.5585	1170.4843	-0.0742	-63	304	312	QKNHGMHFR			Oxidation (M)[6]		Mascot
1187.6643	1187.5024	-0.1619	-136	286	295	DNGLLLHIHR					Mascot
1275.7307	1275.5546	-0.1761	-138	340	350	EITLGFVDLLR					Mascot
1465.7546	1465.5499	-0.2047	-140	147	159	TFQGPPHGIQVER	4	0			Mascot
1465.7546	1465.5499	-0.2047	-140	147	159	TFQGPPHGIQVER					Mascot

Rank	Protein Name	Accession No	Protein MW	Protein PI	Pep. Count	Protein Score	Protein Score C. I. %	Total Ion Score	Total Ion C. I. %
8	Ribulose bisphosphate carboxylase large chain OS=Gnetum parvifolium GN=rbcL PE=3 SV=1	RBL_GNEPA	52469.5	6.18	12	62	97.672	4	0

Peptide Information

Calc. Mass	Obsrv. Mass	± da	± ppm	Start Seq.	End Seq.	Sequence	Ion Score	C. I. %	Modification	Rank	Result Type
910.4451	910.3222	-0.1229	-135	188	194	AVYECLR			Carbamidomethyl (C)[5]		Mascot
912.4719	912.3467	-0.1252	-137	296	303	AMHAVIDR					Mascot
914.4049	914.279	-0.1259	-138	306	312	NHGMHFR			Oxidation (M)[4]		Mascot
914.4049	914.4119	0.007	8	306	312	NHGMHFR			Oxidation (M)[4]		Mascot
921.4709	921.3864	-0.0845	-92	1	8	MSPKTETK					Mascot
928.4669	928.3407	-0.1262	-136	296	303	AMHAVIDR			Oxidation (M)[2]		Mascot
1021.5312	1021.3923	-0.1389	-136	33	41	DTDILAAFR					Mascot
1023.4741	1023.3705	-0.1036	-101	351	358	DDFVEKDR					Mascot
1116.583	1116.4167	-0.1663	-149	422	431	VALEACVQAR			Carbamidomethyl (C)[6]		Mascot
1170.5585	1170.4843	-0.0742	-63	304	312	QKNHGMHFR			Oxidation (M)[6]		Mascot
1187.6643	1187.5024	-0.1619	-136	286	295	DNGLLLHIHR					Mascot
1275.7307	1275.5546	-0.1761	-138	340	350	EITLGFVDLLR					Mascot
1433.7495	1433.5551	-0.1944	-136	320	334	LSGGDHIHGGTVVGK					Mascot
1465.7546	1465.5499	-0.2047	-140	147	159	TFQGPPHGIQVER	4	0			Mascot
1465.7546	1465.5499	-0.2047	-140	147	159	TFQGPPHGIQVER					Mascot

Rank	Protein Name	Accession No	Protein MW	Protein PI	Pep. Count	Protein Score	Protein Score C. I. %	Total Ion Score	Total Ion C. I. %
9	Ribulose bisphosphate carboxylase large chain OS=Ipomoea purpurea GN=rbcL PE=3 SV=1	RBL_IPOPU	53353.9	6.41	12	61	97.563	4	0

Peptide Information

Calc. Mass	Obsrv. Mass	± da	± ppm	Start Seq.	End Seq.	Sequence	Ion Score	C. I. %	Modification	Rank	Result Type
830.4366	830.327	-0.1096	-132	440	446	EGNEIIR					Mascot
910.4451	910.3222	-0.1229	-135	188	194	AVYECLR			Carbamidomethyl (C)[5]		Mascot
912.4719	912.3467	-0.1252	-137	296	303	AMHAVIDR					Mascot
914.4049	914.279	-0.1259	-138	306	312	NHGMHFR			Oxidation (M)[4]		Mascot
914.4049	914.4119	0.007	8	306	312	NHGMHFR			Oxidation (M)[4]		Mascot
921.4346	921.3864	-0.0482	-52	1	8	MSPQTETK					Mascot
928.4669	928.3407	-0.1262	-136	296	303	AMHAVIDR			Oxidation (M)[2]		Mascot
1021.5312	1021.3923	-0.1389	-136	33	41	DTDILAAFR					Mascot
1023.4377	1023.3705	-0.0672	-66	351	358	DDFVEQDR					Mascot
1116.583	1116.4167	-0.1663	-149	422	431	VALEACVQAR			Carbamidomethyl (C)[6]		Mascot
1170.5585	1170.4843	-0.0742	-63	304	312	QKNHGMHFR			Oxidation (M)[6]		Mascot
1187.6643	1187.5024	-0.1619	-136	286	295	DNGLLLHIHR					Mascot
1275.7307	1275.5546	-0.1761	-138	340	350	EITLGFVDLLR					Mascot
1465.7546	1465.5499	-0.2047	-140	147	159	TFQGPPHGIQVER	4	0			Mascot
1465.7546	1465.5499	-0.2047	-140	147	159	TFQGPPHGIQVER					Mascot

Rank	Protein Name	Accession No	Protein MW	Protein PI	Pep. Count	Protein Score	Protein Score C. I. %	Total Ion Score	Total Ion C. I. %
10	Ribulose bisphosphate carboxylase large chain (Fragment) OS=Ajuga chamaepitys GN=rbcL PE=3 SV=1	RBL_AJUCH	52421.4	6.23	12	61	97.328	4	0

Peptide Information

Calc. Mass	Obsrv. Mass	± da	± ppm	Start Seq.	End Seq.	Sequence	Ion Score	C. I. %	Modification	Rank	Result Type
856.3546	856.4129	0.0583	68	467	473	FEFPAMD					Mascot
856.3546	856.4129	0.0583	68	467	473	FEFPAMD					Mascot
910.4451	910.3222	-0.1229	-135	188	194	AVYECLR			Carbamidomethyl (C)[5]		Mascot
912.4719	912.3467	-0.1252	-137	296	303	AMHAVIDR					Mascot
914.4049	914.279	-0.1259	-138	306	312	NHGMHFR			Oxidation (M)[4]		Mascot
914.4049	914.4119	0.007	8	306	312	NHGMHFR			Oxidation (M)[4]		Mascot
921.4346	921.3864	-0.0482	-52	1	8	MSPQTETK					Mascot
928.4669	928.3407	-0.1262	-136	296	303	AMHAVIDR			Oxidation (M)[2]		Mascot
1021.5312	1021.3923	-0.1389	-136	33	41	DTDILAAFR					Mascot
1023.4741	1023.3705	-0.1036	-101	351	358	DDFVEKDR					Mascot
1116.583	1116.4167	-0.1663	-149	422	431	VALEACVQAR			Carbamidomethyl (C)[6]		Mascot
1170.5585	1170.4843	-0.0742	-63	304	312	QKNHGMHFR			Oxidation (M)[6]		Mascot
1187.6643	1187.5024	-0.1619	-136	286	295	DNGLLLHIHR					Mascot
1275.7307	1275.5546	-0.1761	-138	340	350	EITLGFVDLLR					Mascot
1465.7546	1465.5499	-0.2047	-140	147	159	TFQGPPHGIQVER	4	0			Mascot
1465.7546	1465.5499	-0.2047	-140	147	159	TFQGPPHGIQVER					Mascot

Gel Idx/Pos	55/C4	Instr./Gel Origin	BA2060/131017A	Process Sta	Analysis Succeeded
Plate [#] Name	[1] 31496	Instrument Sample Name		Spectra	11

Rank	Protein Name	Accession No	Protein MW	Protein PI	Pep. Count	Protein Score	Protein Score C. I. %	Total Ion Score	Total Ion C. I. %
1	Ribulose bisphosphate carboxylase/oxygenase activase, chloroplastic OS=Malus domestica GN=RCA PE=2	RCA_MALDO	48046.2	8.2	12	451	100	392	100

Peptide Information

Calc. Mass	Obsrv. Mass	± da	± ppm	Start Seq.	End Seq.	Sequence	Ion Score	C. I. %	Modification	Rank	Result Type
895.4155	895.3007	-0.1148	-128	352	358	VYDDEVR					Mascot
940.4675	940.3427	-0.1248	-133	303	309	FYWAPTR					Mascot
940.4675	940.3427	-0.1248	-133	303	309	FYWAPTR	20	24.215			Mascot
1152.7139	1152.5609	-0.153	-133	157	167	VPLILGIWGGK					Mascot
1152.7139	1152.5609	-0.153	-133	157	167	VPLILGIWGGK	43	99.61			Mascot
1228.603	1228.4341	-0.1689	-137	172	181	SFQCELVFAK	10	0	Carbamidomethyl (C)[4]		Mascot
1228.603	1228.4341	-0.1689	-137	172	181	SFQCELVFAK			Carbamidomethyl (C)[4]		Mascot
1623.7617	1623.5353	-0.2264	-139	222	236	MCALFINDLDAGAGR	24	69.968	Carbamidomethyl (C)[2]		Mascot
1623.7617	1623.5353	-0.2264	-139	222	236	MCALFINDLDAGAGR			Carbamidomethyl (C)[2]		Mascot
1639.7567	1639.5339	-0.2228	-136	222	236	MCALFINDLDAGAGR			Carbamidomethyl (C)[2], Oxidation (M)[1]		Mascot
1681.7664	1681.7115	-0.0549	-33	73	87	GLAFDTSDDQQDITR					Mascot
1882.9697	1882.7045	-0.2652	-141	333	349	LVDTFPGQSIDFFGALR	138	100			Mascot
1882.9697	1882.7045	-0.2652	-141	333	349	LVDTFPGQSIDFFGALR					Mascot
1906.9579	1906.6924	-0.2655	-139	393	408	LLEYGNMLVQEQENVK					Mascot
2063.0591	2062.7734	-0.2857	-138	393	409	LLEYGNMLVQEQENVKR	15	0			Mascot
2063.0591	2062.7734	-0.2857	-138	393	409	LLEYGNMLVQEQENVKR					Mascot
2079.054	2078.7571	-0.2969	-143	393	409	LLEYGNMLVQEQENVKR			Oxidation (M)[7]		Mascot
2088.9939	2088.8755	-0.1184	-57	184	204	ISPIMMSAGELESGNAGE PAK					Mascot
2089.1692	2088.8755	-0.2937	-141	278	296	VPIIVTGNDFSTLYAPLIR	145	100			Mascot
2120.9839	2120.8303	-0.1536	-72	184	204	ISPIMMSAGELESGNAGE PAK			Oxidation (M)[5,6]		Mascot
2283.9709	2283.8625	-0.1084	-47	416	437	YLSEAALGDANSDAMNT GTFYG			Oxidation (M)[15]		Mascot

Rank	Protein Name	Accession No	Protein MW	Protein PI	Pep. Count	Protein Score	Protein Score C. I. %	Total Ion Score	Total Ion C. I. %
2	Ribulose bisphosphate carboxylase/oxygenase activase, chloroplastic OS=Vigna radiata var. radiata G	RCA_VIGRR	47871.3	7.57	10	421	100	379	100

Peptide Information

Calc. Mass	Obsrv. Mass	± da	± ppm	Start Seq.	End Seq.	Sequence	Ion Score	C. I. %	Modification	Rank	Result Type
895.4155	895.3007	-0.1148	-128	354	360	VYDDEVR					Mascot
940.4675	940.3427	-0.1248	-133	305	311	FYWAPTR					Mascot
940.4675	940.3427	-0.1248	-133	305	311	FYWAPTR	20	24.215			Mascot
1115.5367	1115.3832	-0.1535	-138	380	389	EGPPTFDQPK					Mascot
1152.7139	1152.5609	-0.153	-133	159	169	VPLILGIWGGK					Mascot
1152.7139	1152.5609	-0.153	-133	159	169	VPLILGIWGGK	43	99.61			Mascot
1228.603	1228.4341	-0.1689	-137	174	183	SFQCELVFAK	10	0	Carbamidomethyl (C)[4]		Mascot
1228.603	1228.4341	-0.1689	-137	174	183	SFQCELVFAK			Carbamidomethyl (C)[4]		Mascot
1623.7617	1623.5353	-0.2264	-139	224	238	MCALFINDLDAGAGR	24	69.968	Carbamidomethyl (C)[2]		Mascot
1623.7617	1623.5353	-0.2264	-139	224	238	MCALFINDLDAGAGR			Carbamidomethyl (C)[2]		Mascot
1639.7567	1639.5339	-0.2228	-136	224	238	MCALFINDLDAGAGR			Carbamidomethyl (C)[2], Oxidation (M)[1]		Mascot
1709.7977	1709.5604	-0.2373	-139	77	91	GLAYDISDDQQDITR					Mascot
1882.9697	1882.7045	-0.2652	-141	335	351	LVDTFPGQSIDFFGALR	138	100			Mascot
1882.9697	1882.7045	-0.2652	-141	335	351	LVDTFPGQSIDFFGALR					Mascot
2089.1692	2088.8755	-0.2937	-141	280	298	VPIIVTGNDFSTLYAPLIR					Mascot
2089.1692	2088.8755	-0.2937	-141	280	298	VPIIVTGNDFSTLYAPLIR	145	100			Mascot
2304.0669	2303.7485	-0.3184	-138	184	206	MGINPIMMSAGELESGN AGEPAK					Mascot

Rank	Protein Name	Accession No	Protein MW	Protein PI	Pep. Count	Protein Score	Protein Score C. I. %	Total Ion Score	Total Ion C. I. %
3	Ribulose bisphosphate carboxylase/oxygenase a B, chloroplastic OS=Hordeum vulgare GN=RCAB PE	RCAB_HORVL	47197.8	7.59	9	385	100	355	100

Peptide Information

Calc. Mass	Obsrv. Mass	± da	± ppm	Start Seq.	End Seq.	Sequence	Ion Score	C. I. %	Modification	Rank	Result Type
895.4155	895.3007	-0.1148	-128	339	345	VYDDEVR					Mascot
940.4675	940.3427	-0.1248	-133	290	296	FYWAPTR					Mascot
940.4675	940.3427	-0.1248	-133	290	296	FYWAPTR	20	24.215			Mascot
1115.5367	1115.3832	-0.1535	-138	365	374	EGPPTFDQPK					Mascot
1152.7139	1152.5609	-0.153	-133	145	155	VPLILGIWGGK					Mascot
1152.7139	1152.5609	-0.153	-133	145	155	VPLILGIWGGK	43	99.61			Mascot
1228.603	1228.4341	-0.1689	-137	160	169	SFQCELVFAK	10	0	Carbamidomethyl (C)[4]		Mascot
1228.603	1228.4341	-0.1689	-137	160	169	SFQCELVFAK			Carbamidomethyl (C)[4]		Mascot
1709.7977	1709.5604	-0.2373	-139	61	75	GLAYDISDDQQDITR					Mascot
1712.7552	1712.5194	-0.2358	-138	209	223	MCCLFINDLDAGAGR			Carbamidomethyl (C)[2,3]		Mascot
1882.9697	1882.7045	-0.2652	-141	320	336	LVDTFPGQSIDFFGALR	138	100			Mascot
1882.9697	1882.7045	-0.2652	-141	320	336	LVDTFPGQSIDFFGALR					Mascot
2089.1692	2088.8755	-0.2937	-141	265	283	VPIIVTGNDFSTLYAPLIR					Mascot
2089.1692	2088.8755	-0.2937	-141	265	283	VPIIVTGNDFSTLYAPLIR	145	100			Mascot

Rank	Protein Name	Accession No	Protein MW	Protein PI	Pep. Count	Protein Score	Protein Score C. I. %	Total Ion Score	Total Ion C. I. %
4	Ribulose bisphosphate carboxylase/oxygenase activase, chloroplastic (Fragments) OS=Arachis hypogaea	RCA_ARAHY	15036.4	4.35	7	378	100	325	100

Peptide Information

Calc. Mass	Obsrv. Mass	± da	± ppm	Start Seq.	End Seq.	Sequence	Ion Score	C. I. %	Modification	Rank	Result Type
965.5236	965.4003	-0.1233	-128	115	123	IGVCTGIFR					Mascot
1152.7139	1152.5609	-0.153	-133	47	57	VPLILGIWGGK					Mascot
1152.7139	1152.5609	-0.153	-133	47	57	VPLILGIWGGK	43	99.61			Mascot
1709.7977	1709.5604	-0.2373	-139	1	15	GLAYDISDDQQDITR					Mascot
1712.7552	1712.5194	-0.2358	-138	81	95	MCCLFINDLDAGAGR			Carbamidomethyl (C)[2,3]		Mascot
1882.9697	1882.7045	-0.2652	-141	124	140	LVDTFPGQSIDFFGALR	138	100			Mascot
1882.9697	1882.7045	-0.2652	-141	124	140	LVDTFPGQSIDFFGALR					Mascot
2089.1692	2088.8755	-0.2937	-141	96	114	VPIIVTGNDFSTLYAPLIR					Mascot
2089.1692	2088.8755	-0.2937	-141	96	114	VPIIVTGNDFSTLYAPLIR	145	100			Mascot
2304.0669	2303.7485	-0.3184	-138	58	80	MGINPIMMSAGELESGN AGEPAK					Mascot

Rank	Protein Name	Accession No	Protein MW	Protein PI	Pep. Count	Protein Score	Protein Score C. I. %	Total Ion Score	Total Ion C. I. %
5	Ribulose bisphosphate carboxylase/oxygenase a 1, chloroplastic OS=Larrea tridentata GN=RCA1	RCA1_LARTR	52108	6.38	10	349	100	312	100

Peptide Information

Calc. Mass	Obsrv. Mass	± da	± ppm	Start Seq.	End Seq.	Sequence	Ion Score	C. I. %	Modification	Rank	Result Type
940.4675	940.3427	-0.1248	-133	303	309	FYWAPTR					Mascot
940.4675	940.3427	-0.1248	-133	303	309	FYWAPTR	20	24.215			Mascot
1115.5367	1115.3832	-0.1535	-138	378	387	EGPPSFEQPK					Mascot
1228.603	1228.4341	-0.1689	-137	172	181	SFQCELVFAK	10	0	Carbamidomethyl (C)[4]		Mascot
1228.603	1228.4341	-0.1689	-137	172	181	SFQCELVFAK			Carbamidomethyl (C)[4]		Mascot
1703.8309	1703.6069	-0.224	-131	378	392	EGPPSFEQPKMTIDK					Mascot
1709.7977	1709.5604	-0.2373	-139	73	87	GLAYDISDDQQDITR					Mascot
1712.7552	1712.5194	-0.2358	-138	222	236	MCCLFINDLDAGAGR			Carbamidomethyl (C)[2,3]		Mascot
1882.9697	1882.7045	-0.2652	-141	333	349	LVDTFPGQSIDFFGALR	138	100			Mascot
1882.9697	1882.7045	-0.2652	-141	333	349	LVDTFPGQSIDFFGALR					Mascot
2089.1692	2088.8755	-0.2937	-141	278	296	VPIIVTGNDFSTLYAPLIR					Mascot
2089.1692	2088.8755	-0.2937	-141	278	296	VPIIVTGNDFSTLYAPLIR	145	100			Mascot
2304.0669	2303.7485	-0.3184	-138	182	204	MGINPIMMSAGELESGN AGEPAK					Mascot
2367.042	2366.7007	-0.3413	-144	121	140	QYNLDNNMDGFYIAPAF MDK					Mascot
2383.0369	2382.7102	-0.3267	-137	121	140	QYNLDNNMDGFYIAPAF MDK			Oxidation (M)[8]		Mascot

Rank	Protein Name	Accession No	Protein MW	Protein PI	Pep. Count	Protein Score	Protein Score C. I. %	Total Ion Score	Total Ion C. I. %
6	Ribulose bisphosphate carboxylase/oxygenase activase, chloroplastic OS=Cucumis sativus PE=2 SV=1	RCA_CUCSA	45681.2	7.57	9	345	100	312	100

Peptide Information

Calc. Mass	Obsrv. Mass	± da	± ppm	Start Seq.	End Seq.	Sequence	Ion Score	C. I. %	Modification	Rank	Result Type
895.4155	895.3007	-0.1148	-128	349	355	VYDDEVR					Mascot
940.4675	940.3427	-0.1248	-133	300	306	FYWAPTR					Mascot
940.4675	940.3427	-0.1248	-133	300	306	FYWAPTR	20	24.215			Mascot
1145.5474	1145.5137	-0.0337	-29	375	384	ESPPTFDQPK					Mascot
1228.603	1228.4341	-0.1689	-137	169	178	SFQCELVFAK	10	0	Carbamidomethyl (C)[4]		Mascot
1228.603	1228.4341	-0.1689	-137	169	178	SFQCELVFAK			Carbamidomethyl (C)[4]		Mascot
1681.7664	1681.7115	-0.0549	-33	70	84	GLAFDTSDDQQDITR					Mascot
1712.7552	1712.5194	-0.2358	-138	219	233	MCCLFINDLDAGAGR			Carbamidomethyl (C)[2,3]		Mascot
1882.9697	1882.7045	-0.2652	-141	330	346	LVDTFPGQSIDFFGALR	138	100			Mascot
1882.9697	1882.7045	-0.2652	-141	330	346	LVDTFPGQSIDFFGALR					Mascot
2089.1692	2088.8755	-0.2937	-141	275	293	VPIIVTGNDFSTLYAPLIR					Mascot
2089.1692	2088.8755	-0.2937	-141	275	293	VPIIVTGNDFSTLYAPLIR	145	100			Mascot
2304.0669	2303.7485	-0.3184	-138	179	201	MGINPIMMSAGELESGN AGEPAK					Mascot

Rank	Protein Name	Accession No	Protein MW	Protein PI	Pep. Count	Protein Score	Protein Score C. I. %	Total Ion Score	Total Ion C. I. %
7	Ribulose bisphosphate carboxylase/oxygenase a 2, chloroplastic OS=Nicotiana tabacum GN=RCA P	RCA2_TOBAC	48312.5	8.14	7	339	100	318	100

Peptide Information

Calc. Mass	Obsrv. Mass	± da	± ppm	Start Seq.	End Seq.	Sequence	Ion Score	C. I. %	Modification	Rank	Result Type
895.4155	895.3007	-0.1148	-128	356	362	VYDDEVR					Mascot
940.4675	940.3427	-0.1248	-133	307	313	FYWAPTR					Mascot
940.4675	940.3427	-0.1248	-133	307	313	FYWAPTR	20	24.215			Mascot
1882.9697	1882.7045	-0.2652	-141	337	353	IVDTFPGQSIDFFGALR	138	100			Mascot
1882.9697	1882.7045	-0.2652	-141	337	353	IVDTFPGQSIDFFGALR					Mascot
1906.9579	1906.6924	-0.2655	-139	397	412	LLEYGNMLVQEQENVK					Mascot
2063.0591	2062.7734	-0.2857	-138	397	413	LLEYGNMLVQEQENVKR	15	0			Mascot
2063.0591	2062.7734	-0.2857	-138	397	413	LLEYGNMLVQEQENVKR					Mascot
2079.054	2078.7571	-0.2969	-143	397	413	LLEYGNMLVQEQENVKR			Oxidation (M)[7]		Mascot
2089.1692	2088.8755	-0.2937	-141	282	300	VPIIVTGNDFSTLYAPLIR					Mascot
2089.1692	2088.8755	-0.2937	-141	282	300	VPIIVTGNDFSTLYAPLIR	145	100			Mascot
2304.0669	2303.7485	-0.3184	-138	186	208	MGINPIMMSAGELESGN AGEPAK					Mascot

Rank	Protein Name	Accession No	Protein MW	Protein PI	Pep. Count	Protein Score	Protein Score C. I. %	Total Ion Score	Total Ion C. I. %
8	Ribulose bisphosphate carboxylase/oxygenase activase, chloroplastic OS=Phaseolus vulgaris GN=RCA1 P	RCA_PHAVU	48170.6	8.19	9	261	100	228	100

Peptide Information

Calc. Mass	Obsrv. Mass	± da	± ppm	Start Seq.	End Seq.	Sequence	Ion Score	C. I. %	Modification	Rank	Result Type
895.4155	895.3007	-0.1148	-128	356	362	VYDDEVR					Mascot
940.4675	940.3427	-0.1248	-133	307	313	FYWAPTR					Mascot
940.4675	940.3427	-0.1248	-133	307	313	FYWAPTR	20	24.215			Mascot
1115.5367	1115.3832	-0.1535	-138	382	391	EGPPTFDQPK					Mascot
1145.6273	1145.5137	-0.1136	-99	2	13	AASLSTVGAVNR					Mascot
1228.603	1228.4341	-0.1689	-137	176	185	SFQCELVFAK	10	0	Carbamidomethyl (C)[4]		Mascot
1228.603	1228.4341	-0.1689	-137	176	185	SFQCELVFAK			Carbamidomethyl (C)[4]		Mascot
1695.782	1695.5465	-0.2355	-139	77	91	GLAYDVSDDQQDITR	54	99.967			Mascot
1695.782	1695.5465	-0.2355	-139	77	91	GLAYDVSDDQQDITR					Mascot
1703.8309	1703.6069	-0.224	-131	382	396	EGPPTFDQPKMTLDK					Mascot
2089.1692	2088.8755	-0.2937	-141	282	300	VPIIVTGNDFSTLYAPLIR					Mascot
2089.1692	2088.8755	-0.2937	-141	282	300	VPIIVTGNDFSTLYAPLIR	145	100			Mascot
2304.0669	2303.7485	-0.3184	-138	186	208	MGINPIMMSAGELESGN AGEPAK					Mascot

Rank	Protein Name	Accession No	Protein MW	Protein PI	Pep. Count	Protein Score	Protein Score C. I. %	Total Ion Score	Total Ion C. I. %
9	Ribulose bisphosphate carboxylase/oxygenase activase, chloroplastic OS=Oryza sativa subsp. japonica	RCA_ORYSJ	51421.4	5.43	11	255	100	217	100

Peptide Information

Calc. Mass	Obsrv. Mass	± da	± ppm	Start Seq.	End Seq.	Sequence	Ion Score	C. I. %	Modification	Rank	Result Type
895.4155	895.3007	-0.1148	-128	343	349	VYDDEVR					Mascot
940.4675	940.3427	-0.1248	-133	294	300	FYWAPTR					Mascot
940.4675	940.3427	-0.1248	-133	294	300	FYWAPTR	20	24.215			Mascot
965.5162	965.4003	-0.1159	-120	38	45	SSNINRFK					Mascot
1152.7139	1152.5609	-0.153	-133	148	158	VPLILGIWGGK					Mascot
1152.7139	1152.5609	-0.153	-133	148	158	VPLILGIWGGK	43	99.61			Mascot
1157.5474	1157.4974	-0.05	-43	369	378	EGPPEFEQPK					Mascot
1228.603	1228.4341	-0.1689	-137	163	172	SFQCELVFAK	10	0	Carbamidomethyl (C)[4]		Mascot
1228.603	1228.4341	-0.1689	-137	163	172	SFQCELVFAK			Carbamidomethyl (C)[4]		Mascot
1709.7977	1709.5604	-0.2373	-139	64	78	GLAYDISDDQQDITR					Mascot
1712.7552	1712.5194	-0.2358	-138	213	227	MCCLFINDLDAGAGR			Carbamidomethyl (C)[2,3]		Mascot
1775.8521	1775.7141	-0.138	-78	369	383	EGPPEFEQPKMTIEK			Oxidation (M)[11]		Mascot
2089.1692	2088.8755	-0.2937	-141	269	287	VPIIVTGNDFSTLYAPLIR					Mascot
2089.1692	2088.8755	-0.2937	-141	269	287	VPIIVTGNDFSTLYAPLIR	145	100			Mascot
2303.9988	2303.7485	-0.2503	-109	112	131	TYDFDNTMGGFYIAPAFM DK					Mascot

Rank	Protein Name	Accession No	Protein MW	Protein PI	Pep. Count	Protein Score	Protein Score C. I. %	Total Ion Score	Total Ion C. I. %
10	Ribulose bisphosphate carboxylase/oxygenase a 1, chloroplastic OS=Nicotiana tabacum PE=1 SV=	RCA1_TOBAC	48722.8	8.44	9	254	100	223	100

Peptide Information

Calc. Mass	Obsrv. Mass	± da	± ppm	Start Seq.	End Seq.	Sequence	Ion Score	C. I. %	Modification	Rank	Result Type
895.4155	895.3007	-0.1148	-128	356	362	VYDDEVR					Mascot
940.4675	940.3427	-0.1248	-133	307	313	FYWAPTR					Mascot
940.4675	940.3427	-0.1248	-133	307	313	FYWAPTR	20	0			Mascot
965.5236	965.4003	-0.1233	-128	317	325	IGVCTGIFR					Mascot
1152.7139	1152.5609	-0.153	-133	161	171	VPLILGIWGGK					Mascot
1152.7139	1152.5609	-0.153	-133	161	171	VPLILGIWGGK	43	99.477			Mascot
1487.7239	1487.6195	-0.1044	-70	132	144	LDGFYIAPAFMDK					Mascot
1906.9579	1906.6924	-0.2655	-139	397	412	LLEYGNMLVQEQENVK					Mascot
2063.0591	2062.7734	-0.2857	-138	397	413	LLEYGNMLVQEQENVKR	15	0			Mascot
2063.0591	2062.7734	-0.2857	-138	397	413	LLEYGNMLVQEQENVKR					Mascot
2079.054	2078.7571	-0.2969	-143	397	413	LLEYGNMLVQEQENVKR			Oxidation (M)[7]		Mascot
2089.1692	2088.8755	-0.2937	-141	282	300	VPIIVTGNDFSTLYAPLIR					Mascot
2089.1692	2088.8755	-0.2937	-141	282	300	VPIIVTGNDFSTLYAPLIR	145	100			Mascot
2304.0669	2303.7485	-0.3184	-138	186	208	MGINPIMMSAGELESGN AGEPAK					Mascot

Gel Idx/Pos	56/C5	Instr./Gel Origin	BA2060/131017A	Process Sta	Analysis Succeeded
Plate [#] Name	[1] 31496	Instrument Sample Name		Spectra	11

Rank	Protein Name	Accession No	Protein MW	Protein PI	Pep. Count	Protein Score	Protein Score C. I. %	Total Ion Score	Total Ion C. I. %
1	Elongation factor Tu, chloroplastic OS=Glycine m GN=TUFA PE=3 SV=1	EFTU1_SOYE	52062.9	6.21	8	119	100	91	100

Peptide Information

Calc. Mass	Obsrv. Mass	± da	± ppm	Start Seq.	End Seq.	Sequence	Ion Score	C. I. %	Modification	Rank	Result Type
1045.5748	1045.4539	-0.1209	-116	305	314	GTVATGRVER					Mascot
1157.6525	1157.4958	-0.1567	-135	319	329	VGETVDLVGLR					Mascot
1435.6699	1435.4771	-0.1928	-134	116	127	KYDEIDAAPEER					Mascot
1435.6699	1435.4771	-0.1928	-134	116	127	KYDEIDAAPEER	35	96.514			Mascot
1681.9482	1681.7139	-0.2343	-139	345	360	ILDEALAGDNVGLLLR					Mascot
1810.8817	1810.6301	-0.2516	-139	130	145	GITINTATVEYETENR					Mascot
1830.9275	1830.665	-0.2625	-143	444	459	MVVELIVPVACEQGMR			Carbamidomethyl (C)[11]		Mascot
2121.1226	2120.8311	-0.2915	-137	286	304	QTDLPFLLAVEDVFSITGR					Mascot
2170.0874	2169.7817	-0.3057	-141	208	225	QDQVDDEELLQLVEIEVR	57	99.978			Mascot
2170.0874	2169.7817	-0.3057	-141	208	225	QDQVDDEELLQLVEIEVR					Mascot

Rank	Protein Name	Accession No	Protein MW	Protein PI	Pep. Count	Protein Score	Protein Score C. I. %	Total Ion Score	Total Ion C. I. %
2	Elongation factor Tu, chloroplastic OS=Nicotiana tabacum GN=TUFA PE=3 SV=1	EFTU_TOBA(	51923.7	6.34	6	108	100	91	100

Peptide Information

Calc. Mass	Obsrv. Mass	± da	± ppm	Start Seq.	End Seq.	Sequence	Ion Score	C. I. %	Modification	Rank	Result Type
1045.5748	1045.4539	-0.1209	-116	304	313	GTVATGRVER					Mascot
1435.6699	1435.4771	-0.1928	-134	115	126	KYDEIDAAPEER					Mascot
1435.6699	1435.4771	-0.1928	-134	115	126	KYDEIDAAPEER	35	96.514			Mascot
1810.8817	1810.6301	-0.2516	-139	129	144	GITINTATVEYETENR					Mascot
1950.0035	1949.7181	-0.2854	-146	95	114	TTLTAALTMALASMGNSAPK					Mascot
2170.0874	2169.7817	-0.3057	-141	207	224	QDQVDDEELLQLVELEVR	57	99.978			Mascot
2170.0874	2169.7817	-0.3057	-141	207	224	QDQVDDEELLQLVELEVR					Mascot
2183.1052	2182.7781	-0.3271	-150	285	303	QTELPFLMAIEDVFSITGR			Oxidation (M)[8]		Mascot

Rank	Protein Name	Accession No	Protein MW	Protein PI	Pep. Count	Protein Score	Protein Score C. I. %	Total Ion Score	Total Ion C. I. %
3	Actin-1 OS=Sorghum bicolor GN=AC1 PE=2 SV=	ACT1_SORBI	41835.9	5.44	10	107	100	60	99.99

Peptide Information

Calc. Mass	Obsrv. Mass	± da	± ppm	Start Seq.	End Seq.	Sequence	Ion Score	C. I. %	Modification	Rank	Result Type
976.4482	976.3551	-0.0931	-95	21	30	AGFAGDDAPR					Mascot
1198.7054	1198.5471	-0.1583	-132	31	41	AVFPSIVGRPR					Mascot
1459.6812	1459.4788	-0.2024	-139	362	374	AEYDESGPSIVHR					Mascot
1515.7491	1515.5427	-0.2064	-136	87	97	IWHHTFYNELR	60	99.99			Mascot
1515.7491	1515.5427	-0.2064	-136	87	97	IWHHTFYNELR					Mascot
1616.7454	1616.5388	-0.2066	-128	315	328	MRQGNHCLAPSSMK			Carbamidomethyl (C)[7]		Mascot
1747.8861	1747.6422	-0.2439	-140	241	256	SYELPDGQVITIAADR					Mascot
1932.8796	1932.6024	-0.2772	-143	71	86	YPIEHGIVSNWDDMEK					Mascot
1954.0643	1953.7786	-0.2857	-146	98	115	VAPEEHPVLLTEAPLNPK					Mascot
2608.2334	2607.9441	-0.2893	-111	218	240	LAYIALDYDQEMETAKTSSSVEK			Oxidation (M)[12]		Mascot
3151.6421	3151.1814	-0.4607	-146	150	179	TTGIVLDSGDGVSHTVPIYEGYALPHAILR					Mascot

Rank	Protein Name	Accession No	Protein MW	Protein PI	Pep. Count	Protein Score	Protein Score C. I. %	Total Ion Score	Total Ion C. I. %
4	Actin-104 (Fragment) OS=Nicotiana tabacum PE= SV=1	ACT7_TOBA(	37120.9	5.57	9	106	100	60	99.99

Peptide Information

Calc. Mass	Obsrv. Mass	± da	± ppm	Start Seq.	End Seq.	Sequence	Ion Score	C. I. %	Modification	Rank	Result Type
976.4482	976.3551	-0.0931	-95	1	10	AGFAGDDAPR					Mascot
1198.7054	1198.5471	-0.1583	-132	11	21	AVFPSIVGRPR					Mascot
1515.7491	1515.5427	-0.2064	-136	67	77	IWHHTFYNELR	60	99.99			Mascot
1515.7491	1515.5427	-0.2064	-136	67	77	IWHHTFYNELR					Mascot
1747.8861	1747.6422	-0.2439	-140	221	236	SYELPDGQVITIGAER					Mascot
1774.8793	1774.6245	-0.2548	-144	174	188	ILTERGYMFTTSAER					Mascot
1932.8796	1932.6024	-0.2772	-143	51	66	YPIEHGIVSNWDDMEK					Mascot
1954.0643	1953.7786	-0.2857	-146	78	95	VAPEEHPVLLTEAPLNPK					Mascot
2183.0801	2182.7781	-0.302	-138	274	294	DLYGNIVLSGGSTMFPGIADR					Mascot
3151.6421	3151.1814	-0.4607	-146	130	159	TTGIVLDSGDGVSHTVPIYEGYALPHAILR					Mascot

Rank	Protein Name	Accession No	Protein MW	Protein PI	Pep. Count	Protein Score	Protein Score C. I. %	Total Ion Score	Total Ion C. I. %
5	Glutamine synthetase leaf isozyme, chloroplastic OS=Phaseolus vulgaris PE=2 SV=1	GLNA4_PHAV	47216.9	6.77	4	104	100	94	100

Peptide Information

Calc. Mass	Obsrv. Mass	± da	± ppm	Start Seq.	End Seq.	Sequence	Ion Score	C. I. %	Modification	Rank	Result Type
899.5672	899.4464	-0.1208	-134	326	333	AILNLSLR					Mascot
899.5672	899.4464	-0.1208	-134	326	333	AILNLSLR	15	0			Mascot
1688.9105	1688.6748	-0.2357	-140	61	75	LEGLLNLDITPFTDK					Mascot
1775.969	1775.7189	-0.2501	-141	76	91	IIAEYIWIGGTGIDVR					Mascot
1789.8616	1789.6101	-0.2515	-141	353	368	HETASINTFSWGVANR					Mascot
1789.8616	1789.6101	-0.2515	-141	353	368	HETASINTFSWGVANR	79	100			Mascot

Rank	Protein Name	Accession No	Protein MW	Protein PI	Pep. Count	Protein Score	Protein Score C. I. %	Total Ion Score	Total Ion C. I. %
6	Elongation factor Tu, chloroplastic OS=Glycine m GN=TUFB1 PE=3 SV=1	EFTU2_SOYE	52476.2	6.33	5	103	100	91	100

Peptide Information

Calc. Mass	Obsrv. Mass	± da	± ppm	Start Seq.	End Seq.	Sequence	Ion Score	C. I. %	Modification	Rank	Result Type
1045.5748	1045.4539	-0.1209	-116	305	314	GTVATGRVER					Mascot
1435.6699	1435.4771	-0.1928	-134	116	127	KYDEIDAAPEER					Mascot
1435.6699	1435.4771	-0.1928	-134	116	127	KYDEIDAAPEER	35	96.514			Mascot
1681.9482	1681.7139	-0.2343	-139	345	360	ILDEALAGDNVGLLLR					Mascot
1810.8817	1810.6301	-0.2516	-139	130	145	GITINTATVEYETENR					Mascot
2170.0874	2169.7817	-0.3057	-141	208	225	QDQVDDEELLQLVELEVR	57	99.978			Mascot
2170.0874	2169.7817	-0.3057	-141	208	225	QDQVDDEELLQLVELEVR					Mascot

Rank	Protein Name	Accession No	Protein MW	Protein PI	Pep. Count	Protein Score	Protein Score C. I. %	Total Ion Score	Total Ion C. I. %
7	Actin-3 OS=Pisum sativum PE=2 SV=1	ACT3_PEA	41607.9	5.31	9	101	100	60	99.99

Peptide Information

Calc. Mass	Obsrv. Mass	± da	± ppm	Start Seq.	End Seq.	Sequence	Ion Score	C. I. %	Modification	Rank	Result Type
976.4482	976.3551	-0.0931	-95	21	30	AGFAGDDAPR					Mascot
1198.7054	1198.5471	-0.1583	-132	31	41	AVFPSIVGRPR					Mascot
1459.6812	1459.4788	-0.2024	-139	362	374	AEYDESGPSIVHR					Mascot
1515.7491	1515.5427	-0.2064	-136	87	97	IWHHTFYNELR	60	99.99			Mascot
1515.7491	1515.5427	-0.2064	-136	87	97	IWHHTFYNELR					Mascot
1932.8796	1932.6024	-0.2772	-143	71	86	YPIEHGIVSNWDDMEK					Mascot
1954.0643	1953.7786	-0.2857	-146	98	115	VAPEEHPVLLTEAPLNPK					Mascot
2120.9661	2120.8311	-0.135	-64	1	20	MAEAEDIQPLVCDNGTGMVK					Mascot
2183.0801	2182.7781	-0.302	-138	294	314	DLYGNIVLSGGSTMFPGIADR					Mascot
3151.4128	3151.1814	-0.2314	-73	1	30	MAEAEDIQPLVCDNGTGMVKAGFAGDDAPR			Carbamidomethyl (C)[12], Oxidation (M)[1]		Mascot

Rank	Protein Name	Accession No	Protein MW	Protein PI	Pep. Count	Protein Score	Protein Score C. I. %	Total Ion Score	Total Ion C. I. %
8	Actin-1 OS=Oryza sativa subsp. japonica GN=AC PE=2 SV=1	ACT1_ORYS	41785.9	5.3	9	101	100	60	99.99

Peptide Information

Calc. Mass	Obsrv. Mass	± da	± ppm	Start Seq.	End Seq.	Sequence	Ion Score	C. I. %	Modification	Rank	Result Type
976.4482	976.3551	-0.0931	-95	21	30	AGFAGDDAPR					Mascot
1198.7054	1198.5471	-0.1583	-132	31	41	AVFPSIVGRPR					Mascot
1459.6812	1459.4788	-0.2024	-139	362	374	AEYDESGPSIVHR					Mascot
1515.7491	1515.5427	-0.2064	-136	87	97	IWHHTFYNELR	60	99.99			Mascot
1515.7491	1515.5427	-0.2064	-136	87	97	IWHHTFYNELR					Mascot
1747.8861	1747.6422	-0.2439	-140	241	256	SYELPDGQVITIGAER					Mascot
1932.8796	1932.6024	-0.2772	-143	71	86	YPIEHGIVSNWDDMEK					Mascot
1954.0643	1953.7786	-0.2857	-146	98	115	VAPEEHPVLLTEAPLNPK					Mascot
2608.2334	2607.9441	-0.2893	-111	218	240	LSYIALDYDQEMETAKTSSSVEK					Mascot
3151.6421	3151.1814	-0.4607	-146	150	179	TTGIVLDSGDGVSHTVPIYEGYALPHAILR					Mascot

Rank	Protein Name	Accession No	Protein MW	Protein PI	Pep. Count	Protein Score	Protein Score C. I. %	Total Ion Score	Total Ion C. I. %
9	Actin-85C (Fragment) OS=Solanum tuberosum GN=AC85C PE=3 SV=1	ACT10_SOLT	21681.1	5.89	6	95	99.999	60	99.99

Peptide Information

Calc. Mass	Obsrv. Mass	± da	± ppm	Start Seq.	End Seq.	Sequence	Ion Score	C. I. %	Modification	Rank	Result Type
976.4482	976.3551	-0.0931	-95	1	10	AGFAGDDAPR					Mascot
1198.7054	1198.5471	-0.1583	-132	11	21	AVFPSIVGRPR					Mascot
1515.7491	1515.5427	-0.2064	-136	67	77	IWHHTFYNELR	60	99.99			Mascot
1515.7491	1515.5427	-0.2064	-136	67	77	IWHHTFYNELR					Mascot
1932.8796	1932.6024	-0.2772	-143	51	66	YPIEHGIVSNWDDMEK					Mascot
	1932.6024										Mascot
1954.0643	1953.7786	-0.2857	-146	78	95	VAPEEHPVLLTEAPLNPK					Mascot
3151.6421	3151.1814	-0.4607	-146	130	159	TTGIVLDSGDGVSHTVPIYEGYALPHAILR					Mascot

Rank	Protein Name	Accession No	Protein MW	Protein PI	Pep. Count	Protein Score	Protein Score C. I. %	Total Ion Score	Total Ion C. I. %
10	Actin-11 OS=Arabidopsis thaliana GN=ACT11 PE SV=1	ACT11_ARAT	41647.9	5.23	8	95	99.999	60	99.99

Peptide Information

Calc. Mass	Obsrv. Mass	± da	± ppm	Start Seq.	End Seq.	Sequence	Ion Score	C. I. %	Modification	Rank	Result Type
976.4482	976.3551	-0.0931	-95	21	30	AGFAGDDAPR					Mascot
1198.7054	1198.5471	-0.1583	-132	31	41	AVFPSIVGRPR					Mascot
1459.6812	1459.4788	-0.2024	-139	362	374	AEYDESGPSIVHR					Mascot
1515.7491	1515.5427	-0.2064	-136	87	97	IWHHTFYNELR	60	99.99			Mascot
1515.7491	1515.5427	-0.2064	-136	87	97	IWHHTFYNELR					Mascot
1932.8796	1932.6024	-0.2772	-143	71	86	YPIEHGIVSNWDDMEK					Mascot
1954.0643	1953.7786	-0.2857	-146	98	115	VAPEEHPVLLTEAPLNPK					Mascot
2608.197	2607.9441	-0.2529	-97	218	240	LAYIALDYEQEMETANTSSSVEK			Oxidation (M)[12]		Mascot
3151.6421	3151.1814	-0.4607	-146	150	179	TTGIVLDSGDGVSHTVPIYEGYALPHAILR					Mascot

Gel Idx/Pos	57/C6	Instr./Gel Origin	BA2060/131017A	Process Sta	Analysis Succeeded
Plate [#] Name	[1] 31496	Instrument Sample Name		Spectra	11

Rank	Protein Name	Accession No	Protein MW	Protein PI	Pep. Count	Protein Score	Protein Score C. I. %	Total Ion Score	Total Ion C. I. %
1	Elongation factor Tu, chloroplastic OS=Glycine m GN=TUFA PE=3 SV=1	EFTU1_SOYE	52062.9	6.21	11	182	100	132	100

Peptide Information

Calc. Mass	Obsrv. Mass	± da	± ppm	Start Seq.	End Seq.	Sequence	Ion Score	C. I. %	Modification	Rank	Result Type
1157.6525	1157.5026	-0.1499	-129	319	329	VGETVDLVGLR					Mascot
1307.575	1307.5085	-0.0665	-51	117	127	YDEIDAAPEER					Mascot
1354.6671	1354.4913	-0.1758	-130	333	344	NTTVTGVEMFQK					Mascot
1435.6699	1435.4791	-0.1908	-133	116	127	KYDEIDAAPEER					Mascot
1435.6699	1435.4791	-0.1908	-133	116	127	KYDEIDAAPEER	53	99.953			Mascot
1681.9482	1681.7228	-0.2254	-134	345	360	ILDEALAGDNVGLLLR					Mascot
1805.8958	1805.655	-0.2408	-133	444	459	MVVELIVPVACEQGMR			Oxidation (M)[1,15]		Mascot
1810.8817	1810.6338	-0.2479	-137	130	145	GITINTATVEYETENR					Mascot
1810.8817	1810.6338	-0.2479	-137	130	145	GITINTATVEYETENR					Mascot
1830.9275	1830.6702	-0.2573	-141	444	459	MVVELIVPVACEQGMR			Carbamidomethyl (C)[11]		Mascot
1846.9224	1846.6615	-0.2609	-141	444	459	MVVELIVPVACEQGMR			Carbamidomethyl (C)[11], Oxidation (M)[1]		Mascot
2091.0579	2090.7764	-0.2815	-135	269	285	IFQLMDEVDNYIPIPQR					Mascot
2121.1226	2120.8174	-0.3052	-144	286	304	QTDLPFLLAVEDVFSITGR					Mascot
2170.0874	2169.7849	-0.3025	-139	208	225	QDQVDDEELLQLVEIEVR					Mascot
2170.0874	2169.7849	-0.3025	-139	208	225	QDQVDDEELLQLVEIEVR	79	100			Mascot
2253.0374	2252.7588	-0.2786	-124	422	441	VTSIMNDKDEESTMVLPGDR			Oxidation (M)[5]		Mascot

Rank	Protein Name	Accession No	Protein MW	Protein PI	Pep. Count	Protein Score	Protein Score C. I. %	Total Ion Score	Total Ion C. I. %
2	Glutamine synthetase leaf isozyme, chloroplastic OS=Phaseolus vulgaris PE=2 SV=1	GLNA4_PHAV	47216.9	6.77	5	160	100	147	100

Peptide Information

Calc. Mass	Obsrv. Mass	± da	± ppm	Start Seq.	End Seq.	Sequence	Ion Score	C. I. %	Modification	Rank	Result Type
899.5672	899.4523	-0.1149	-128	326	333	AILNLSLR					Mascot
899.5672	899.4523	-0.1149	-128	326	333	AILNLSLR	31	91.17			Mascot
1383.6572	1383.499	-0.1582	-114	313	324	SMREDGGFEVIK			Oxidation (M)[2]		Mascot
1688.9105	1688.678	-0.2325	-138	61	75	LEGLLNLDITPFTDK					Mascot
1688.9105	1688.678	-0.2325	-138	61	75	LEGLLNLDITPFTDK	23	44.412			Mascot
1775.969	1775.7277	-0.2413	-136	76	91	IIAEYIWIGGTGIDVR	94	100			Mascot
1775.969	1775.7277	-0.2413	-136	76	91	IIAEYIWIGGTGIDVR					Mascot
1789.8616	1789.6189	-0.2427	-136	353	368	HETASINTFSWGVANR					Mascot

Rank	Protein Name	Accession No	Protein MW	Protein PI	Pep. Count	Protein Score	Protein Score C. I. %	Total Ion Score	Total Ion C. I. %
3	Elongation factor Tu, chloroplastic OS=Glycine m GN=TUFB1 PE=3 SV=1	EFTU2_SOYE	52476.2	6.33	7	154	100	132	100

Peptide Information

Calc. Mass	Obsrv. Mass	± da	± ppm	Start Seq.	End Seq.	Sequence	Ion Score	C. I. %	Modification	Rank	Result Type
1307.575	1307.5085	-0.0665	-51	117	127	YDEIDAAPEER					Mascot
1354.6671	1354.4913	-0.1758	-130	333	344	NTTVTGVEMFQK					Mascot
1435.6699	1435.4791	-0.1908	-133	116	127	KYDEIDAAPEER					Mascot
1435.6699	1435.4791	-0.1908	-133	116	127	KYDEIDAAPEER	53	99.953			Mascot
1487.8064	1487.6212	-0.1852	-124	319	332	VGETVDIVGVKDTR					Mascot
1681.9482	1681.7228	-0.2254	-134	345	360	ILDEALAGDNVGLLLR					Mascot
1810.8817	1810.6338	-0.2479	-137	130	145	GITINTATVEYETENR					Mascot
1810.8817	1810.6338	-0.2479	-137	130	145	GITINTATVEYETENR					Mascot
2170.0874	2169.7849	-0.3025	-139	208	225	QDQVDDEELLQLVELEVR					Mascot
2170.0874	2169.7849	-0.3025	-139	208	225	QDQVDDEELLQLVELEVR	79	100			Mascot

Rank	Protein Name	Accession No	Protein MW	Protein PI	Pep. Count	Protein Score	Protein Score C. I. %	Total Ion Score	Total Ion C. I. %
4	Elongation factor Tu, chloroplastic OS=Nicotiana tabacum GN=TUFA PE=3 SV=1	EFTU_TOBA(	51923.7	6.34	4	141	100	132	100

Peptide Information

Calc. Mass	Obsrv. Mass	± da	± ppm	Start Seq.	End Seq.	Sequence	Ion Score	C. I. %	Modification	Rank	Result Type
1307.575	1307.5085	-0.0665	-51	116	126	YDEIDAAPEER					Mascot
1435.6699	1435.4791	-0.1908	-133	115	126	KYDEIDAAPEER					Mascot
1435.6699	1435.4791	-0.1908	-133	115	126	KYDEIDAAPEER	53	99.953			Mascot
1810.8817	1810.6338	-0.2479	-137	129	144	GITINTATVEYETENR					Mascot
1810.8817	1810.6338	-0.2479	-137	129	144	GITINTATVEYETENR					Mascot
2170.0874	2169.7849	-0.3025	-139	207	224	QDQVDDEELLQLVELEVR					Mascot
2170.0874	2169.7849	-0.3025	-139	207	224	QDQVDDEELLQLVELEVR	79	100			Mascot

Rank	Protein Name	Accession No	Protein MW	Protein PI	Pep. Count	Protein Score	Protein Score C. I. %	Total Ion Score	Total Ion C. I. %
5	Glutamine synthetase, chloroplastic OS=Daucus GN=GLN2 PE=2 SV=1	GLNA2_DAUC	47733.8	5.63	3	133	100	125	100

Peptide Information

Calc. Mass	Obsrv. Mass	± da	± ppm	Start Seq.	End Seq.	Sequence	Ion Score	C. I. %	Modification	Rank	Result Type
899.5672	899.4523	-0.1149	-128	329	336	AILNLSLR					Mascot
899.5672	899.4523	-0.1149	-128	329	336	AILNLSLR	31	91.17			Mascot
1775.969	1775.7277	-0.2413	-136	79	94	IIAEYIWIGGTGIDVR	94	100			Mascot
1775.969	1775.7277	-0.2413	-136	79	94	IIAEYIWIGGTGIDVR					Mascot
2958.3792	2957.9683	-0.4109	-139	113	139	WNYDGSSTGQAPGDDSEVILYPQAIFK					Mascot

Rank	Protein Name	Accession No	Protein MW	Protein PI	Pep. Count	Protein Score	Protein Score C. I. %	Total Ion Score	Total Ion C. I. %
6	Glutamine synthetase, chloroplastic OS=Zea may GN=GLN2 PE=2 SV=1	GLNAC_MAIZ	45988.2	6.42	6	101	100	81	100

Peptide Information

Calc. Mass	Obsrv. Mass	± da	± ppm	Start Seq.	End Seq.	Sequence	Ion Score	C. I. %	Modification	Rank	Result Type
899.5672	899.4523	-0.1149	-128	320	327	AILNLSLR					Mascot
899.5672	899.4523	-0.1149	-128	320	327	AILNLSLR	31	91.17			Mascot
976.5098	976.3799	-0.1299	-133	161	169	AAQIFSDPK					Mascot
1539.8264	1539.6184	-0.208	-135	90	103	TISKPVEDPSELPK					Mascot
1797.8575	1797.7074	-0.1501	-83	55	69	LEQLLNMDTTPYTDK			Oxidation (M)[7]		Mascot
2487.2183	2486.8774	-0.3409	-137	135	157	GGNNVLVICDTYTPQGEPLPTNK			Carbamidomethyl (C)[9]		Mascot
2487.2183	2486.8774	-0.3409	-137	135	157	GGNNVLVICDTYTPQGEPLPTNK	18	0	Carbamidomethyl (C)[9]		Mascot
2643.3196	2642.9502	-0.3694	-140	135	158	GGNNVLVICDTYTPQGEPLPTNKR	33	94.543	Carbamidomethyl (C)[9]		Mascot
2643.3196	2642.9502	-0.3694	-140	135	158	GGNNVLVICDTYTPQGEPLPTNKR			Carbamidomethyl (C)[9]		Mascot

Rank	Protein Name	Accession No	Protein MW	Protein PI	Pep. Count	Protein Score	Protein Score C. I. %	Total Ion Score	Total Ion C. I. %
7	Glutamine synthetase, chloroplastic OS=Brassica napus GN=GLN2 PE=2 SV=1	GLNAC_BRAN	47314.9	6.16	4	90	99.997	80	100

Peptide Information

Calc. Mass	Obsrv. Mass	± da	± ppm	Start Seq.	End Seq.	Sequence	Ion Score	C. I. %	Modification	Rank	Result Type
899.5672	899.4523	-0.1149	-128	325	332	AILNLSLR					Mascot
899.5672	899.4523	-0.1149	-128	325	332	AILNLSLR	31	91.17			Mascot
1383.6572	1383.499	-0.1582	-114	312	323	SMREDGGFEVIK			Oxidation (M)[2]		Mascot
1775.969	1775.7277	-0.2413	-136	75	90	IIAEYIWIGGSGIDLR	50	99.899			Mascot
1775.969	1775.7277	-0.2413	-136	75	90	IIAEYIWIGGSGIDLR					Mascot
1817.8566	1817.7124	-0.1442	-79	352	367	HETASIDQFSWGVANR					Mascot

Rank	Protein Name	Accession No	Protein MW	Protein PI	Pep. Count	Protein Score	Protein Score C. I. %	Total Ion Score	Total Ion C. I. %
8	Glutamine synthetase, chloroplastic/mitochondria OS=Arabidopsis thaliana GN=GLN2 PE=1 SV=1	GLNA2_ARAT	47381	6.43	3	87	99.994	80	100

Peptide Information

Calc. Mass	Obsrv. Mass	± da	± ppm	Start Seq.	End Seq.	Sequence	Ion Score	C. I. %	Modification	Rank	Result Type
899.5672	899.4523	-0.1149	-128	327	334	AILNLSLR					Mascot
899.5672	899.4523	-0.1149	-128	327	334	AILNLSLR	31	91.17			Mascot
1775.969	1775.7277	-0.2413	-136	77	92	IIAEYIWIGGSGIDLR	50	99.899			Mascot
1775.969	1775.7277	-0.2413	-136	77	92	IIAEYIWIGGSGIDLR					Mascot
1817.8566	1817.7124	-0.1442	-79	354	369	HETASIDQFSWGVANR					Mascot

Rank	Protein Name	Accession No	Protein MW	Protein PI	Pep. Count	Protein Score	Protein Score C. I. %	Total Ion Score	Total Ion C. I. %
9	Elongation factor Tu, chloroplastic OS=Arabidops thaliana GN=TUFA PE=1 SV=1	EFTU_ARATH	51597.6	5.84	8	82	99.98	53	99.953

Peptide Information

Calc. Mass	Obsrv. Mass	± da	± ppm	Start Seq.	End Seq.	Sequence	Ion Score	C. I. %	Modification	Rank	Result Type
1157.6525	1157.5026	-0.1499	-129	316	326	VGETVDLVGLR					Mascot
1307.575	1307.5085	-0.0665	-51	114	124	YDEIDAAPEER					Mascot
1354.609	1354.4913	-0.1177	-87	1	14	MAISAPAACSSSSR			Oxidation (M)[1]		Mascot
1435.6699	1435.4791	-0.1908	-133	113	124	KYDEIDAAPEER					Mascot
1435.6699	1435.4791	-0.1908	-133	113	124	KYDEIDAAPEER	53	99.953			Mascot
1681.9482	1681.7228	-0.2254	-134	342	357	ILDEALAGDNVGLLLR					Mascot
1775.8633	1775.7277	-0.1356	-76	327	341	ETRSYTVTGVEMFQK					Mascot
1775.8633	1775.7277	-0.1356	-76	327	341	ETRSYTVTGVEMFQK					Mascot
1810.8817	1810.6338	-0.2479	-137	127	142	GITINTATVEYETENR					Mascot
1810.8817	1810.6338	-0.2479	-137	127	142	GITINTATVEYETENR					Mascot
3457.6396	3457.1494	-0.4902	-142	2	35	AISAPAACSSSSRILCSYSSPSPSLCPAISTSGK			Carbamidomethyl (C)[8,16,26]		Mascot

Rank	Protein Name	Accession No	Protein MW	Protein PI	Pep. Count	Protein Score	Protein Score C. I. %	Total Ion Score	Total Ion C. I. %
10	Glutamine synthetase, chloroplastic OS=Oryza sa subsp. japonica GN=GLN2 PE=1 SV=1	GLNA2_ORYS	46613.4	5.96	4	75	99.889	65	99.997

Peptide Information

Calc. Mass	Obsrv. Mass	± da	± ppm	Start Seq.	End Seq.	Sequence	Ion Score	C. I. %	Modification	Rank	Result Type
899.5672	899.4523	-0.1149	-128	325	332	AILNLSLR					Mascot
899.5672	899.4523	-0.1149	-128	325	332	AILNLSLR	31	91.17			Mascot
1383.6572	1383.499	-0.1582	-114	312	323	SMREDGGFEVIK			Oxidation (M)[2]		Mascot
1539.8264	1539.6184	-0.208	-135	95	108	TISKPVEDPSELPK					Mascot
1775.969	1775.7277	-0.2413	-136	75	90	IIAEYIWVGGTGIDLR	34	96.436			Mascot
1775.969	1775.7277	-0.2413	-136	75	90	IIAEYIWVGGTGIDLR					Mascot

Gel Idx/Pos	58/C7	Instr./Gel Origin	BA2060/131017A	Process Sta	Analysis Succeeded
Plate [#] Name	[1] 31496	Instrument Sample Name		Spectra	11

Rank	Protein Name	Accession No	Protein MW	Protein PI	Pep. Count	Protein Score	Protein Score C. I. %	Total Ion Score	Total Ion C. I. %
1	Elongation factor Tu, chloroplastic OS=Glycine m GN=TUFA PE=3 SV=1	EFTU1_SOYE	52062.9	6.21	11	341	100	290	100

Peptide Information

Calc. Mass	Obsrv. Mass	± da	± ppm	Start Seq.	End Seq.	Sequence	Ion Score	C. I. %	Modification	Rank	Result Type
1118.5225	1118.3741	-0.1484	-133	260	268	RGDNEWVDK					Mascot
1157.6525	1157.4974	-0.1551	-134	319	329	VGETVDLVGLR					Mascot
1354.6671	1354.4711	-0.196	-145	333	344	NTTVTGVEMFQK					Mascot
1435.6699	1435.4751	-0.1948	-136	116	127	KYDEIDAAPEER					Mascot
1435.6699	1435.4751	-0.1948	-136	116	127	KYDEIDAAPEER	65	99.997			Mascot
1681.9482	1681.7134	-0.2348	-140	345	360	ILDEALAGDNVGLLLR	87	100			Mascot
1681.9482	1681.7134	-0.2348	-140	345	360	ILDEALAGDNVGLLLR					Mascot
1810.8817	1810.6307	-0.251	-139	130	145	GITINTATVEYETENR	9	0			Mascot
1810.8817	1810.6307	-0.251	-139	130	145	GITINTATVEYETENR					Mascot
1830.9275	1830.6691	-0.2584	-141	444	459	MVVELIVPVACEQGMR	29	85.814	Carbamidomethyl (C)[11]		Mascot
1830.9275	1830.6691	-0.2584	-141	444	459	MVVELIVPVACEQGMR			Carbamidomethyl (C)[11]		Mascot
1846.9224	1846.6613	-0.2611	-141	444	459	MVVELIVPVACEQGMR			Carbamidomethyl (C)[11], Oxidation (M)[1]		Mascot
1862.9172	1862.6689	-0.2483	-133	444	459	MVVELIVPVACEQGMR			Carbamidomethyl (C)[11], Oxidation (M)[1,15]		Mascot
2091.0579	2090.7585	-0.2994	-143	269	285	IFQLMDEVDNYIPIPQR					Mascot
2107.0527	2106.7732	-0.2795	-133	269	285	IFQLMDEVDNYIPIPQR			Oxidation (M)[5]		Mascot
2121.1226	2120.8152	-0.3074	-145	286	304	QTDLPFLLAVEDVFSITGR					Mascot
2170.0874	2169.7759	-0.3115	-144	208	225	QDQVDDEELLQLVEIEVR					Mascot
2170.0874	2169.7759	-0.3115	-144	208	225	QDQVDDEELLQLVEIEVR	99	100			Mascot
2237.0425	2236.7422	-0.3003	-134	422	441	VTSIMNDKDEESTMVLPGDR					Mascot

Rank	Protein Name	Accession No	Protein MW	Protein PI	Pep. Count	Protein Score	Protein Score C. I. %	Total Ion Score	Total Ion C. I. %
2	Elongation factor Tu, chloroplastic OS=Glycine m GN=TUFB1 PE=3 SV=1	EFTU2_SOYE	52476.2	6.33	7	284	100	261	100

Peptide Information

Calc. Mass	Obsrv. Mass	± da	± ppm	Start Seq.	End Seq.	Sequence	Ion Score	C. I. %	Modification	Rank	Result Type
965.4608	965.4061	-0.0547	-57	422	429	VTEIMNDK			Oxidation (M)[5]		Mascot
1354.6671	1354.4711	-0.196	-145	333	344	NTTVTGVEMFQK					Mascot
1435.6699	1435.4751	-0.1948	-136	116	127	KYDEIDAAPEER					Mascot
1435.6699	1435.4751	-0.1948	-136	116	127	KYDEIDAAPEER	65	99.997			Mascot
1487.8064	1487.6268	-0.1796	-121	319	332	VGETVDIVGVKDTR					Mascot
1487.8064	1487.6268	-0.1796	-121	319	332	VGETVDIVGVKDTR					Mascot
1681.9482	1681.7134	-0.2348	-140	345	360	ILDEALAGDNVGLLLR	87	100			Mascot
1681.9482	1681.7134	-0.2348	-140	345	360	ILDEALAGDNVGLLLR					Mascot
1810.8817	1810.6307	-0.251	-139	130	145	GITINTATVEYETENR	9	0			Mascot
1810.8817	1810.6307	-0.251	-139	130	145	GITINTATVEYETENR					Mascot
2170.0874	2169.7759	-0.3115	-144	208	225	QDQVDDEELLQLVELEVR					Mascot
2170.0874	2169.7759	-0.3115	-144	208	225	QDQVDDEELLQLVELEVR	99	100			Mascot

Rank	Protein Name	Accession No	Protein MW	Protein PI	Pep. Count	Protein Score	Protein Score C. I. %	Total Ion Score	Total Ion C. I. %
3	Elongation factor Tu, chloroplastic OS=Nicotiana tabacum GN=TUFA PE=3 SV=1	EFTU_TOBA(	51923.7	6.34	3	180	100	174	100

Peptide Information

Calc. Mass	Obsrv. Mass	± da	± ppm	Start Seq.	End Seq.	Sequence	Ion Score	C. I. %	Modification	Rank	Result Type
1435.6699	1435.4751	-0.1948	-136	115	126	KYDEIDAAPEER					Mascot
1435.6699	1435.4751	-0.1948	-136	115	126	KYDEIDAAPEER	65	99.997			Mascot
1810.8817	1810.6307	-0.251	-139	129	144	GITINTATVEYETENR	9	0			Mascot
1810.8817	1810.6307	-0.251	-139	129	144	GITINTATVEYETENR					Mascot
2170.0874	2169.7759	-0.3115	-144	207	224	QDQVDDEELLQLVELEVR					Mascot
2170.0874	2169.7759	-0.3115	-144	207	224	QDQVDDEELLQLVELEVR	99	100			Mascot

Rank	Protein Name	Accession No	Protein MW	Protein PI	Pep. Count	Protein Score	Protein Score C. I. %	Total Ion Score	Total Ion C. I. %
4	Elongation factor Tu, chloroplastic OS=Arabidops thaliana GN=TUFA PE=1 SV=1	EFTU_ARATH	51597.6	5.84	6	178	100	162	100

Peptide Information

Calc. Mass	Obsrv. Mass	± da	± ppm	Start Seq.	End Seq.	Sequence	Ion Score	C. I. %	Modification	Rank	Result Type
1157.6525	1157.4974	-0.1551	-134	316	326	VGETVDLVGLR					Mascot
1354.609	1354.4711	-0.1379	-102	1	14	MAISAPAACSSSSR			Oxidation (M)[1]		Mascot
1435.6699	1435.4751	-0.1948	-136	113	124	KYDEIDAAPEER					Mascot
1435.6699	1435.4751	-0.1948	-136	113	124	KYDEIDAAPEER	65	99.997			Mascot
1681.9482	1681.7134	-0.2348	-140	342	357	ILDEALAGDNVGLLLR	87	100			Mascot
1681.9482	1681.7134	-0.2348	-140	342	357	ILDEALAGDNVGLLLR					Mascot
1775.8633	1775.7213	-0.142	-80	327	341	ETRSYTVTGVEMFQK					Mascot
1775.8633	1775.7213	-0.142	-80	327	341	ETRSYTVTGVEMFQK					Mascot
1810.8817	1810.6307	-0.251	-139	127	142	GITINTATVEYETENR	9	0			Mascot
1810.8817	1810.6307	-0.251	-139	127	142	GITINTATVEYETENR					Mascot

Rank	Protein Name	Accession No	Protein MW	Protein PI	Pep. Count	Protein Score	Protein Score C. I. %	Total Ion Score	Total Ion C. I. %
5	Elongation factor TuB, chloroplastic OS=Nicotiar sylvestris GN=TUFB PE=2 SV=1	EFTUB_NICS	52655	5.95	4	84	99.985	75	100

Peptide Information

Calc. Mass	Obsrv. Mass	± da	± ppm	Start Seq.	End Seq.	Sequence	Ion Score	C. I. %	Modification	Rank	Result Type
1354.6671	1354.4711	-0.196	-145	339	350	NTTVTGVEMFQK					Mascot
1435.6699	1435.4751	-0.1948	-136	122	133	KYDEIDAAPEER					Mascot
1435.6699	1435.4751	-0.1948	-136	122	133	KYDEIDAAPEER	65	99.997			Mascot
1810.8817	1810.6307	-0.251	-139	136	151	GITINTATVEYETENR	9	0			Mascot
1810.8817	1810.6307	-0.251	-139	136	151	GITINTATVEYETENR					Mascot
2107.0527	2106.7732	-0.2795	-133	275	291	IYQLMDNVDEYIPIPQR					Mascot

Rank	Protein Name	Accession No	Protein MW	Protein PI	Pep. Count	Protein Score	Protein Score C. I. %	Total Ion Score	Total Ion C. I. %
6	Elongation factor Tu, chloroplastic OS=Pisum sa GN=tufA PE=2 SV=1	EFTU_PEA	53016.6	6.62	4	74	99.872	65	99.997

Peptide Information

Calc. Mass	Obsrv. Mass	± da	± ppm	Start Seq.	End Seq.	Sequence	Ion Score	C. I. %	Modification	Rank	Result Type
1354.6671	1354.4711	-0.196	-145	342	353	NTTVTGVEMFQK					Mascot
1435.6699	1435.4751	-0.1948	-136	125	136	KYDEIDAAPEER					Mascot
1435.6699	1435.4751	-0.1948	-136	125	136	KYDEIDAAPEER	65	99.997			Mascot
2121.105	2120.8152	-0.2898	-137	278	294	IYQLMDEVDKYIPIPQR					Mascot
2211.0386	2210.7966	-0.242	-109	270	287	GNNQWVDKIYQLMDEVDK			Oxidation (M)[13]		Mascot

Rank	Protein Name	Accession No	Protein MW	Protein PI	Pep. Count	Protein Score	Protein Score C. I. %	Total Ion Score	Total Ion C. I. %
7	Elongation factor Tu, chloroplastic OS=Chara vul GN=tufA PE=3 SV=1	EFTU_CHAVU	46419.2	5.89	1	66	99.052	65	99.997

Peptide Information

Calc. Mass	Obsrv. Mass	± da	± ppm	Start Seq.	End Seq.	Sequence	Ion Score	C. I. %	Modification	Rank	Result Type
1435.6699	1435.4751	-0.1948	-136	46	57	KYDEIDAAPEER					Mascot
1435.6699	1435.4751	-0.1948	-136	46	57	KYDEIDAAPEER	65	99.997			Mascot

Rank	Protein Name	Accession No	Protein MW	Protein PI	Pep. Count	Protein Score	Protein Score C. I. %	Total Ion Score	Total Ion C. I. %
8	Photosystem II 5 kDa protein, chloroplastic OS=P hybrida PE=1 SV=1	PST2_PETHY	11309.6	9.67	6	33	0		

Peptide Information

Calc. Mass	Obsrv. Mass	± da	± ppm	Start Seq.	End Seq.	Sequence	Ion Score	C. I. %	Modification	Rank	Result Type
1354.642	1354.4711	-0.1709	-126	91	102	YSSVCVTNPTAR			Carbamidomethyl (C)[5]		Mascot
1443.6896	1443.517	-0.1726	-120	34	46	AMHEGENNVVISK			Oxidation (M)[2]		Mascot
1482.7369	1482.5504	-0.1865	-126	90	102	KYSSVCVTNPTAR			Carbamidomethyl (C)[6]		Mascot
1616.7811	1616.5459	-0.2352	-145	57	72	ELFFAMAAAAACSVAK			Oxidation (M)[6]		Mascot
1813.9087	1813.7924	-0.1163	-64	56	72	RELFFAMAAAAACSVAK			Carbamidomethyl (C)[13]		Mascot
2643.2939	2642.9502	-0.3437	-130	1	26	MASITMMSSFLGGSTVAPAKVPSANR			Oxidation (M)[1,6]		Mascot

Rank	Protein Name	Accession No	Protein MW	Protein PI	Pep. Count	Protein Score	Protein Score C. I. %	Total Ion Score	Total Ion C. I. %
9	TPR repeat-containing thioredoxin TTL1 OS=Arabidopsis thaliana GN=TTL1 PE=1 SV=1	TTL1_ARATH	76067.4	9.03	11	33	0		

Peptide Information

Calc. Mass	Obsrv. Mass	± da	± ppm	Start Seq.	End Seq.	Sequence	Ion Score	C. I. %	Modification	Rank	Result Type
1081.5272	1081.382	-0.1452	-134	472	480	GNDLYKSER					Mascot
1148.5365	1148.4105	-0.126	-110	518	527	SIEDCNQALR					Mascot
1275.6791	1275.5557	-0.1234	-97	220	231	KAILGSDSEEVK					Mascot
1377.7267	1377.5697	-0.157	-114	149	160	IQITGMTQSRSR					Mascot
1451.677	1451.4955	-0.1815	-125	494	505	LDPCNAILYCNR			Carbamidomethyl (C)[4]		Mascot
1617.7688	1617.5438	-0.225	-139	89	103	SQSNSSSGNNNLRPR					Mascot
1789.8351	1789.605	-0.2301	-129	478	493	SERYTEASSAYAEGLR					Mascot
1810.8389	1810.6307	-0.2082	-115	161	178	SDVLGSGTGTYGHGSIMR			Oxidation (M)[17]		Mascot
1810.8389	1810.6307	-0.2082	-115	161	178	SDVLGSGTGTYGHGSIMR			Oxidation (M)[17]		Mascot
1882.9116	1882.7222	-0.1894	-101	546	561	MERWGAAVSDYEALIR			Oxidation (M)[1]		Mascot
1993.9219	1993.6862	-0.2357	-118	512	527	LGMWERSIEDCNQALR			Carbamidomethyl (C)[11], Oxidation (M)[3]		Mascot
2170.0928	2169.7759	-0.3169	-146	549	567	WGAAVSDYEALIRELPHDK					Mascot
2170.0928	2169.7759	-0.3169	-146	549	567	WGAAVSDYEALIRELPHDK					Mascot

Rank	Protein Name	Accession No	Protein MW	Protein PI	Pep. Count	Protein Score	Protein Score C. I. %	Total Ion Score	Total Ion C. I. %
10	Pentatricopeptide repeat-containing protein At1g7 mitochondrial OS=Arabidopsis thaliana GN=OTP	PP125_ARAT	55046.4	9.38	9	32	0		

Peptide Information

Calc. Mass	Obsrv. Mass	± da	± ppm	Start Seq.	End Seq.	Sequence	Ion Score	C. I. %	Modification	Rank	Result Type
1465.7296	1465.5571	-0.1725	-118	248	258	HAWEFFLEMKK					Mascot
1681.8346	1681.7134	-0.1212	-72	73	85	ALQFFHFLDNHHR					Mascot
1681.8346	1681.7134	-0.1212	-72	73	85	ALQFFHFLDNHHR					Mascot
1813.8094	1813.7924	-0.017	-9	314	328	DNVENAVVMFEEMVR			Oxidation (M)[9,13]		Mascot
1862.937	1862.6689	-0.2681	-144	243	257	AGQIRHAWEFFLEMK					Mascot
2256.0999	2255.8064	-0.2935	-130	220	238	EMVERGINPNLTTYNTMLK			Oxidation (M)[2,17]		Mascot
2256.0999	2255.8064	-0.2935	-130	220	238	EMVERGINPNLTTYNTMLK			Oxidation (M)[2,17]		Mascot
2263.9238	2263.6946	-0.2292	-101	361	378	MENEGCEPNFQTYNMMIR			Carbamidomethyl (C)[6]		Mascot
2733.3044	2733.0281	-0.2763	-101	396	419	MGSGDCLPNLDTYNILISGMFVRK			Carbamidomethyl (C)[6], Oxidation (M)[1,20]		Mascot
3274.6309	3274.2134	-0.4175	-127	389	418	ALGLFEKMGSGDCLPNLDTYNILISGMFVR					Mascot
3337.6167	3337.2727	-0.344	-103	148	175	LFLNMHEHGCFQDLASFNTILDVLCKSK			Carbamidomethyl (C)[10,25]		Mascot

Gel Idx/Pos	59/C8	Instr./Gel Origin	BA2060/131017A	Process State	Analysis Succeeded
Plate [#] Name	[1] 31496	Instrument Sample Name		Spectra	11

Rank	Protein Name	Accession No	Protein MW	Protein PI	Pep. Count	Protein Score	Protein Score C. I. %	Total Ion Score	Total Ion C. I. %
1	Ribulose bisphosphate carboxylase large chain (Fragment) OS=Streptopus lanceolatus GN=rbcL PE=3 SV=	RBL_STRLC	50649.4	6.18	13	66	99.052		

Peptide Information

Calc. Mass	Obsrv. Mass	± da	± ppm	Start Seq.	End Seq.	Sequence	Ion Score	C. I. %	Modification	Rank	Result Type
910.4451	910.335	-0.1101	-121	188	194	AVYECLR			Carbamidomethyl (C)[5]		Mascot
912.4719	912.3414	-0.1305	-143	296	303	AMHAVIDR					Mascot
921.4346	921.3862	-0.0484	-53	1	8	MSPQTETK					Mascot
1004.4506	1004.3907	-0.0599	-60	451	459	WSPELAAAC			Carbamidomethyl (C)[9]		Mascot
1021.5312	1021.3918	-0.1394	-136	33	41	DTDILAAFR					Mascot
1021.5312	1021.3918	-0.1394	-136	33	41	DTDILAAFR					Mascot
1116.583	1116.4401	-0.1429	-128	422	431	VALEACVQAR			Carbamidomethyl (C)[6]		Mascot
1187.6643	1187.4905	-0.1738	-146	286	295	DNGLLLHIHR					Mascot
1435.6344	1435.4783	-0.1561	-109	447	459	EACKWSPELAAAC			Carbamidomethyl (C)[3]		Mascot
1465.7216	1465.5457	-0.1759	-120	320	334	MSGGDHIHAGTVVGK					Mascot
1482.7257	1482.5446	-0.1811	-122	1	14	MSPOTETKASAGFK					Mascot
1810.8098	1810.6305	-0.1793	-99	237	252	GHYLNATAGTCEEMIK			Carbamidomethyl (C)[11], Oxidation (M)[14]		Mascot
1966.9109	1966.7991	-0.1118	-57	237	253	GHYLNATAGTCEEMIKR			Carbamidomethyl (C)[11], Oxidation (M)[14]		Mascot
2169.9868	2169.792	-0.1948	-90	195	213	GGLDFTKDDENVNSQPFMR					Mascot

Rank	Protein Name	Accession No	Protein MW	Protein PI	Pep. Count	Protein Score	Protein Score C. I. %	Total Ion Score	Total Ion C. I. %
2	Ribulose bisphosphate carboxylase large chain (Fragment) OS=Lupinus aureonitens GN=rbcL PE=3 SV=1	RBL_LUPAU	50255.4	6.32	12	58	94.017		

Peptide Information

Calc. Mass	Obsrv. Mass	± da	± ppm	Start Seq.	End Seq.	Sequence	Ion Score	C. I. %	Modification	Rank	Result Type
892.525	892.3983	-0.1267	-142	1	9	SVGFKAGVK					Mascot
910.4451	910.335	-0.1101	-121	179	185	AVYECLR			Carbamidomethyl (C)[5]		Mascot
912.4719	912.3414	-0.1305	-143	287	294	AMHAVIDR					Mascot
1021.5312	1021.3918	-0.1394	-136	24	32	DTDILAAFR					Mascot
1021.5312	1021.3918	-0.1394	-136	24	32	DTDILAAFR					Mascot
1116.583	1116.4401	-0.1429	-128	413	422	VALEACVQAR			Carbamidomethyl (C)[6]		Mascot
1187.6643	1187.4905	-0.1738	-146	277	286	DNGLLLHIHR					Mascot
1392.6682	1392.491	-0.1772	-127	13	23	LTYYTPDYQTK					Mascot
1465.7546	1465.5457	-0.2089	-143	138	150	TFQGPPHGIQVER					Mascot
1671.8408	1671.6123	-0.2285	-137	423	437	NEGRDLASEGNQIIR					Mascot
1810.8098	1810.6305	-0.1793	-99	228	243	GHYLNATAGTCEEMIK			Carbamidomethyl (C)[11], Oxidation (M)[14]		Mascot
1966.9109	1966.7991	-0.1118	-57	228	244	GHYLNATAGTCEEMIKR			Carbamidomethyl (C)[11], Oxidation (M)[14]		Mascot
2169.9868	2169.792	-0.1948	-90	186	204	GGLDFTKDDENVNSQPFMR					Mascot

Rank	Protein Name	Accession No	Protein MW	Protein PI	Pep. Count	Protein Score	Protein Score C. I. %	Total Ion Score	Total Ion C. I. %
3	Ribulose bisphosphate carboxylase large chain OS=Sesbania sesban GN=rbcL PE=3 SV=1	RBL_SESSE	52358.4	6.09	12	57	93.131		

Peptide Information

Calc. Mass	Obsrv. Mass	± da	± ppm	Start Seq.	End Seq.	Sequence	Ion Score	C. I. %	Modification	Rank	Result Type
856.3546	856.41	0.0554	65	467	473	FEFPAMD					Mascot
856.3546	856.41	0.0554	65	467	473	FEFPAMD					Mascot
910.4451	910.335	-0.1101	-121	188	194	AVYECLR			Carbamidomethyl (C)[5]		Mascot
912.4719	912.3414	-0.1305	-143	296	303	AMHAVIDR					Mascot
921.4346	921.3862	-0.0484	-53	1	8	MSPQTETK					Mascot
1021.5312	1021.3918	-0.1394	-136	33	41	DTDILAAFR					Mascot
1021.5312	1021.3918	-0.1394	-136	33	41	DTDILAAFR					Mascot
1039.4691	1039.3726	-0.0965	-93	351	358	DDYVEKDR					Mascot
1116.583	1116.4401	-0.1429	-128	422	431	VALEACVQAR			Carbamidomethyl (C)[6]		Mascot
1187.6643	1187.4905	-0.1738	-146	286	295	DNGLLLHIHR					Mascot
1465.7546	1465.5457	-0.2089	-143	147	159	TFQGPPHGIQVER					Mascot
1810.8098	1810.6305	-0.1793	-99	237	252	GHYLNATAGTCEEMIK			Carbamidomethyl (C)[11], Oxidation (M)[14]		Mascot
1966.9109	1966.7991	-0.1118	-57	237	253	GHYLNATAGTCEEMIKR			Carbamidomethyl (C)[11], Oxidation (M)[14]		Mascot
2169.9868	2169.792	-0.1948	-90	195	213	GGLDFTKDDENVNSQPFMR					Mascot

Rank	Protein Name	Accession No	Protein MW	Protein PI	Pep. Count	Protein Score	Protein Score C. I. %	Total Ion Score	Total Ion C. I. %
4	Ribulose bisphosphate carboxylase large chain (Fragment) OS=Sinapis alba GN=rbcL PE=3 SV=1	RBL_SINAL	52405.4	6.23	12	57	92.971		

Peptide Information

Calc. Mass	Obsrv. Mass	± da	± ppm	Start Seq.	End Seq.	Sequence	Ion Score	C. I. %	Modification	Rank	Result Type
856.3546	856.41	0.0554	65	467	473	FEFPAMD					Mascot
856.3546	856.41	0.0554	65	467	473	FEFPAMD					Mascot
910.4451	910.335	-0.1101	-121	188	194	AVYECLR			Carbamidomethyl (C)[5]		Mascot
912.4719	912.3414	-0.1305	-143	296	303	AMHAVIDR					Mascot
921.4346	921.3862	-0.0484	-53	1	8	MSPQTETK					Mascot
1021.5312	1021.3918	-0.1394	-136	33	41	DTDILAAFR					Mascot
1021.5312	1021.3918	-0.1394	-136	33	41	DTDILAAFR					Mascot
1116.583	1116.4401	-0.1429	-128	422	431	VALEACVQAR			Carbamidomethyl (C)[6]		Mascot
1187.6643	1187.4905	-0.1738	-146	286	295	DNGLLLHIHR					Mascot
1392.6682	1392.491	-0.1772	-127	22	32	LTYYTPDYQTK					Mascot
1465.7546	1465.5457	-0.2089	-143	147	159	TFQGPPHGIQVER					Mascot
1810.8098	1810.6305	-0.1793	-99	237	252	GHYLNATAGTCEEMIK			Carbamidomethyl (C)[11], Oxidation (M)[14]		Mascot
1966.9109	1966.7991	-0.1118	-57	237	253	GHYLNATAGTCEEMIKR			Carbamidomethyl (C)[11], Oxidation (M)[14]		Mascot
2169.9868	2169.792	-0.1948	-90	195	213	GGLDFTKDDENVNSQPFMR					Mascot

Rank	Protein Name	Accession No	Protein MW	Protein PI	Pep. Count	Protein Score	Protein Score C. I. %	Total Ion Score	Total Ion C. I. %
5	Ribulose bisphosphate carboxylase large chain OS=Manihot esculenta GN=rbcL PE=3 SV=1	RBL_MANES	52648.5	6.18	12	56	90.734		

Peptide Information

Calc. Mass	Obsrv. Mass	± da	± ppm	Start Seq.	End Seq.	Sequence	Ion Score	C. I. %	Modification	Rank	Result Type
910.4451	910.335	-0.1101	-121	188	194	AVYECLR			Carbamidomethyl (C)[5]		Mascot
912.4719	912.3414	-0.1305	-143	296	303	AMHAVIDR					Mascot
921.4346	921.3862	-0.0484	-53	1	8	MSPQTETK					Mascot
1021.5312	1021.3918	-0.1394	-136	33	41	DTDILAAFR					Mascot
1021.5312	1021.3918	-0.1394	-136	33	41	DTDILAAFR					Mascot
1116.583	1116.4401	-0.1429	-128	422	431	VALEACVQAR			Carbamidomethyl (C)[6]		Mascot
1187.6643	1187.4905	-0.1738	-146	286	295	DNGLLLHIHR					Mascot
1255.6205	1255.4624	-0.1581	-126	467	477	FEFAAVDTLDK					Mascot
1392.6682	1392.491	-0.1772	-127	22	32	LTYYTPDYQTK					Mascot
1465.7546	1465.5457	-0.2089	-143	147	159	TFQGPPHGIQVER					Mascot
1810.8098	1810.6305	-0.1793	-99	237	252	GHYLNATAGTCEEMIK			Carbamidomethyl (C)[11], Oxidation (M)[14]		Mascot
1966.9109	1966.7991	-0.1118	-57	237	253	GHYLNATAGTCEEMIKR			Carbamidomethyl (C)[11], Oxidation (M)[14]		Mascot
2169.9868	2169.792	-0.1948	-90	195	213	GGLDFTKDDENVNSQPFMR					Mascot

Rank	Protein Name	Accession No	Protein MW	Protein PI	Pep. Count	Protein Score	Protein Score C. I. %	Total Ion Score	Total Ion C. I. %
6	Putative cytochrome c oxidase subunit II PS17 (Fragments) OS=Pinus strobus PE=1 SV=1	PS17_PINST	1707	9.63	2	51	70.698	29	78.237

Peptide Information

Calc. Mass	Obsrv. Mass	± da	± ppm	Start Seq.	End Seq.	Sequence	Ion Score	C. I. %	Modification	Rank	Result Type
856.525	856.41	-0.115	-134	1	8	SPTVIALR					Mascot
856.525	856.41	-0.115	-134	1	8	SPTVIALR	8	0			Mascot
870.5043	870.4257	-0.0786	-90	9	16	VVEALSPR					Mascot
870.5043	870.4257	-0.0786	-90	9	16	VVEALSPR	21	0			Mascot

Rank	Protein Name	Accession No	Protein MW	Protein PI	Pep. Count	Protein Score	Protein Score C. I. %	Total Ion Score	Total Ion C. I. %
7	Ribulose bisphosphate carboxylase large chain (Fragment) OS=Lupinus albifrons GN=rbcL PE=3 SV=1	RBL_LUPAB	50269.4	6.44	11	50	63.951		

Peptide Information

Calc. Mass	Obsrv. Mass	± da	± ppm	Start Seq.	End Seq.	Sequence	Ion Score	C. I. %	Modification	Rank	Result Type
892.525	892.3983	-0.1267	-142	1	9	SVGFKAGVK					Mascot
910.4451	910.335	-0.1101	-121	179	185	AVYECLR			Carbamidomethyl (C)[5]		Mascot
912.4719	912.3414	-0.1305	-143	287	294	AMHAVIDR					Mascot
1021.5312	1021.3918	-0.1394	-136	24	32	DTDILAAFR					Mascot
1021.5312	1021.3918	-0.1394	-136	24	32	DTDILAAFR					Mascot
1116.583	1116.4401	-0.1429	-128	413	422	VALEACVQAR			Carbamidomethyl (C)[6]		Mascot
1187.6643	1187.4905	-0.1738	-146	277	286	DNGLLLHIHR					Mascot
1465.7546	1465.5457	-0.2089	-143	138	150	TFQGPPHGIQVER					Mascot
1671.8408	1671.6123	-0.2285	-137	423	437	NEGRDLASEGNQIIR					Mascot
1810.8098	1810.6305	-0.1793	-99	228	243	GHYLNATAGTCEEMIK			Carbamidomethyl (C)[11], Oxidation (M)[14]		Mascot
1966.9109	1966.7991	-0.1118	-57	228	244	GHYLNATAGTCEEMIKR			Carbamidomethyl (C)[11], Oxidation (M)[14]		Mascot
2169.9868	2169.792	-0.1948	-90	186	204	GGLDFTKDDENVNSQPFMR					Mascot

Rank	Protein Name	Accession No	Protein MW	Protein PI	Pep. Count	Protein Score	Protein Score C. I. %	Total Ion Score	Total Ion C. I. %
8	Ribulose bisphosphate carboxylase large chain (Fragment) OS=Corynocarpus laevigatus GN=rbcL PE=3 SV	RBL_CORLA	50423.4	6.32	11	50	63.111		

Peptide Information

Calc. Mass	Obsrv. Mass	± da	± ppm	Start Seq.	End Seq.	Sequence	Ion Score	C. I. %	Modification	Rank	Result Type
910.4451	910.335	-0.1101	-121	188	194	AVYECLR			Carbamidomethyl (C)[5]		Mascot
912.4719	912.3414	-0.1305	-143	296	303	AMHAVIDR					Mascot
921.4346	921.3862	-0.0484	-53	1	8	MSPQTETK					Mascot
1004.4506	1004.3907	-0.0599	-60	451	459	WSPELAAAC			Carbamidomethyl (C)[9]		Mascot
1021.5312	1021.3918	-0.1394	-136	33	41	DTDILAAFR					Mascot
1021.5312	1021.3918	-0.1394	-136	33	41	DTDILAAFR					Mascot
1116.583	1116.4401	-0.1429	-128	422	431	VALEACVQAR			Carbamidomethyl (C)[6]		Mascot
1187.6643	1187.4905	-0.1738	-146	286	295	DNGLLLHIHR					Mascot
1465.7216	1465.5457	-0.1759	-120	320	334	MSGGDHIHAGTVVGK					Mascot
1810.8098	1810.6305	-0.1793	-99	237	252	GHYLNATAGTCEEMIK			Carbamidomethyl (C)[11], Oxidation (M)[14]		Mascot
1966.9109	1966.7991	-0.1118	-57	237	253	GHYLNATAGTCEEMIKR			Carbamidomethyl (C)[11], Oxidation (M)[14]		Mascot
2169.9868	2169.792	-0.1948	-90	195	213	GGLDFTKDDENVNSQPFMR					Mascot

Rank	Protein Name	Accession No	Protein MW	Protein PI	Pep. Count	Protein Score	Protein Score C. I. %	Total Ion Score	Total Ion C. I. %
9	Ribulose bisphosphate carboxylase large chain (Fragment) OS=Aesculus pavia GN=rbcL PE=3 SV=2	RBL_AESPA	51619	6.18	11	49	61.373		

Peptide Information

Calc. Mass	Obsrv. Mass	± da	± ppm	Start Seq.	End Seq.	Sequence	Ion Score	C. I. %	Modification	Rank	Result Type
892.525	892.3983	-0.1267	-142	1	9	SVGFKAGVK					Mascot
910.4451	910.335	-0.1101	-121	179	185	AVYECLR			Carbamidomethyl (C)[5]		Mascot
912.4719	912.3414	-0.1305	-143	287	294	AMHAVIDR					Mascot
1021.5312	1021.3918	-0.1394	-136	24	32	DTDILAAFR					Mascot
1021.5312	1021.3918	-0.1394	-136	24	32	DTDILAAFR					Mascot
1116.583	1116.4401	-0.1429	-128	413	422	VALEACVQAR			Carbamidomethyl (C)[6]		Mascot
1187.6643	1187.4905	-0.1738	-146	277	286	DNGLLLHIHR					Mascot
1465.7216	1465.5457	-0.1759	-120	311	325	MSGGDHIHAGTVVGK					Mascot
1487.7086	1487.6149	-0.0937	-63	455	466	EIKFQFEAMDTL			Oxidation (M)[9]		Mascot
1487.7086	1487.6149	-0.0937	-63	455	466	EIKFQFEAMDTL			Oxidation (M)[9]		Mascot
1810.8098	1810.6305	-0.1793	-99	228	243	GHYLNATAGTCEEMIK			Carbamidomethyl (C)[11], Oxidation (M)[14]		Mascot
1966.9109	1966.7991	-0.1118	-57	228	244	GHYLNATAGTCEEMIKR			Carbamidomethyl (C)[11], Oxidation (M)[14]		Mascot
2169.9868	2169.792	-0.1948	-90	186	204	GGLDFTKDDENVNSQPFMR					Mascot

Rank	Protein Name	Accession No	Protein MW	Protein PI	Pep. Count	Protein Score	Protein Score C. I. %	Total Ion Score	Total Ion C. I. %
10	Ribulose bisphosphate carboxylase large chain (Fragment) OS=Poliothyrsis sinensis GN=rbcL PE=3 SV=1	RBL_POLSI	51642	5.99	11	49	61.373		

Peptide Information

Calc. Mass	Obsrv. Mass	± da	± ppm	Start Seq.	End Seq.	Sequence	Ion Score	C. I. %	Modification	Rank	Result Type
892.525	892.3983	-0.1267	-142	1	9	SVGFKAGVK					Mascot
910.4451	910.335	-0.1101	-121	179	185	AVYECLR			Carbamidomethyl (C)[5]		Mascot
912.4719	912.3414	-0.1305	-143	287	294	AMHAVIDR					Mascot
1021.5312	1021.3918	-0.1394	-136	24	32	DTDILAAFR					Mascot
1021.5312	1021.3918	-0.1394	-136	24	32	DTDILAAFR					Mascot
1116.583	1116.4401	-0.1429	-128	413	422	VALEACVQAR			Carbamidomethyl (C)[6]		Mascot
1187.6643	1187.4905	-0.1738	-146	277	286	DNGLLLHIHR					Mascot
1465.7546	1465.5457	-0.2089	-143	138	150	TFQGPPHGIQVER					Mascot
1487.7086	1487.6149	-0.0937	-63	455	466	EIKFEFQAMDTL			Oxidation (M)[9]		Mascot
1487.7086	1487.6149	-0.0937	-63	455	466	EIKFEFQAMDTL			Oxidation (M)[9]		Mascot
1810.8098	1810.6305	-0.1793	-99	228	243	GHYLNATAGTCEEMIK			Carbamidomethyl (C)[11], Oxidation (M)[14]		Mascot
1966.9109	1966.7991	-0.1118	-57	228	244	GHYLNATAGTCEEMIKR			Carbamidomethyl (C)[11], Oxidation (M)[14]		Mascot
2169.9868	2169.792	-0.1948	-90	186	204	GGLDFTKDDENVNSQPFMR					Mascot

Gel Idx/Pos	60/C9	Instr./Gel Origin	BA2060/131017A	Process State	Analysis Succeeded
Plate [#] Name	[1] 31496	Instrument Sample Name		Spectra	11

Rank	Protein Name	Accession No	Protein MW	Protein PI	Pep. Count	Protein Score	Protein Score C. I. %	Total Ion Score	Total Ion C. I. %
1	Glutamine synthetase leaf isozyme, chloroplastic OS=Phaseolus vulgaris PE=2 SV=1	GLNA4_PHAV	47216.9	6.77	8	191	100	158	100

Peptide Information

Calc. Mass	Obsrv. Mass	± da	± ppm	Start Seq.	End Seq.	Sequence	Ion Score	C. I. %	Modification	Rank	Result Type
899.5672	899.453	-0.1142	-127	326	333	AILNLSLR					Mascot
899.5672	899.453	-0.1142	-127	326	333	AILNLSLR	21	26.523			Mascot
948.4421	948.3303	-0.1118	-118	227	234	DISDAHYK					Mascot
1367.6624	1367.4923	-0.1701	-124	313	324	SMREDGGFEVIK					Mascot
1561.8584	1561.6479	-0.2105	-135	96	109	TISKPVEHPSELPK	40	98.957			Mascot
1561.8584	1561.6479	-0.2105	-135	96	109	TISKPVEHPSELPK					Mascot
1688.9105	1688.6758	-0.2347	-139	61	75	LEGLLNLDITPFTDK	35	96.579			Mascot
1688.9105	1688.6758	-0.2347	-139	61	75	LEGLLNLDITPFTDK					Mascot
1775.969	1775.7281	-0.2409	-136	76	91	IIAEYIWIGGTGIDVR					Mascot
1789.8616	1789.6182	-0.2434	-136	353	368	HETASINTFSWGVANR					Mascot
1789.8616	1789.6182	-0.2434	-136	353	368	HETASINTFSWGVANR	62	99.994			Mascot
3457.6692	3457.197	-0.4722	-137	281	312	ITEQAGVVLSLDPKPIEGDWNGAGCHTNYSTK			Carbamidomethyl (C)[25]		Mascot

Rank	Protein Name	Accession No	Protein MW	Protein PI	Pep. Count	Protein Score	Protein Score C. I. %	Total Ion Score	Total Ion C. I. %
2	Glutamine synthetase leaf isozyme, chloroplastic OS=Medicago sativa GN=GS2 PE=2 SV=1	GLNA2_MEDS	47085.9	6.29	6	142	100	123	100

Peptide Information

Calc. Mass	Obsrv. Mass	± da	± ppm	Start Seq.	End Seq.	Sequence	Ion Score	C. I. %	Modification	Rank	Result Type
899.5672	899.453	-0.1142	-127	325	332	AILNLSLR					Mascot
899.5672	899.453	-0.1142	-127	325	332	AILNLSLR	21	26.523			Mascot
948.4421	948.3303	-0.1118	-118	226	233	DISDAHYK					Mascot
976.5098	976.382	-0.1278	-131	166	174	AAEIFSNPK					Mascot
1367.6624	1367.4923	-0.1701	-124	312	323	SMREDGGFEVIK					Mascot
1561.8584	1561.6479	-0.2105	-135	95	108	TISKPVEHPSELPK	40	98.957			Mascot
1561.8584	1561.6479	-0.2105	-135	95	108	TISKPVEHPSELPK					Mascot
1789.8616	1789.6182	-0.2434	-136	352	367	HETASINTFSWGVANR					Mascot
1789.8616	1789.6182	-0.2434	-136	352	367	HETASINTFSWGVANR	62	99.994			Mascot

Rank	Protein Name	Accession No	Protein MW	Protein PI	Pep. Count	Protein Score	Protein Score C. I. %	Total Ion Score	Total Ion C. I. %
3	Glutamine synthetase, chloroplastic OS=Daucus GN=GLN2 PE=2 SV=1	GLNA2_DAUC	47733.8	5.63	6	82	99.979	61	99.992

Peptide Information

Calc. Mass	Obsrv. Mass	± da	± ppm	Start Seq.	End Seq.	Sequence	Ion Score	C. I. %	Modification	Rank	Result Type
899.5672	899.453	-0.1142	-127	329	336	AILNLSLR					Mascot
899.5672	899.453	-0.1142	-127	329	336	AILNLSLR	21	26.523			Mascot
948.4421	948.3303	-0.1118	-118	230	237	DISDAHYK					Mascot
1561.8584	1561.6479	-0.2105	-135	99	112	TISKPVEHPSELPK	40	98.957			Mascot
1561.8584	1561.6479	-0.2105	-135	99	112	TISKPVEHPSELPK					Mascot
1775.969	1775.7281	-0.2409	-136	79	94	IIAEYIWIGGTGIDVR					Mascot
2958.3792	2957.9612	-0.418	-141	113	139	WNYDGSSTGQAPGDDSEVILYPQAIFK					Mascot
3457.6692	3457.197	-0.4722	-137	284	315	ITEQAGVVLTLDPKPIDGDWNGAGCHTNYSTK			Carbamidomethyl (C)[25]		Mascot

Rank	Protein Name	Accession No	Protein MW	Protein PI	Pep. Count	Protein Score	Protein Score C. I. %	Total Ion Score	Total Ion C. I. %
4	Glutamine synthetase, chloroplastic OS=Zea may GN=GLN2 PE=2 SV=1	GLNAC_MAIZ	45988.2	6.42	7	77	99.939	52	99.932

Peptide Information

Calc. Mass	Obsrv. Mass	± da	± ppm	Start Seq.	End Seq.	Sequence	Ion Score	C. I. %	Modification	Rank	Result Type
899.5672	899.453	-0.1142	-127	320	327	AILNLSLR					Mascot
899.5672	899.453	-0.1142	-127	320	327	AILNLSLR	21	26.523			Mascot
948.4421	948.3303	-0.1118	-118	221	228	DISDAHYK					Mascot
976.5098	976.382	-0.1278	-131	161	169	AAQIFSDPK					Mascot
1377.6436	1377.5734	-0.0702	-51	1	12	MAQAVVPAMQCR			Carbamidomethyl (C)[11], Oxidation (M)[1]		Mascot
1539.8264	1539.6168	-0.2096	-136	90	103	TISKPVEDPSELPK					Mascot
2487.2183	2486.874	-0.3443	-138	135	157	GGNNVLVICDTYTPQGEPLPTNK	8	0	Carbamidomethyl (C)[9]		Mascot
2487.2183	2486.874	-0.3443	-138	135	157	GGNNVLVICDTYTPQGEPLPTNK			Carbamidomethyl (C)[9]		Mascot
2643.3196	2642.948	-0.3716	-141	135	158	GGNNVLVICDTYTPQGEPLPTNKR	22	36.004	Carbamidomethyl (C)[9]		Mascot
2643.3196	2642.948	-0.3716	-141	135	158	GGNNVLVICDTYTPQGEPLPTNKR			Carbamidomethyl (C)[9]		Mascot

Rank	Protein Name	Accession No	Protein MW	Protein PI	Pep. Count	Protein Score	Protein Score C. I. %	Total Ion Score	Total Ion C. I. %
5	Putative LRR disease resistance protein/transmembrane receptor kinase PS19 (Fragment) OS=Pinus stro	PS19_PINST	869.5	9.63	1	49	54.617	43	99.532

Peptide Information

Calc. Mass	Obsrv. Mass	± da	± ppm	Start Seq.	End Seq.	Sequence	Ion Score	C. I. %	Modification	Rank	Result Type
870.5406	870.4346	-0.106	-122	1	8	VVSLSIPR					Mascot
870.5406	870.4346	-0.106	-122	1	8	VVSLSIPR	43	99.532			Mascot

Rank	Protein Name	Accession No	Protein MW	Protein PI	Pep. Count	Protein Score	Protein Score C. I. %	Total Ion Score	Total Ion C. I. %
6	Putative cytochrome c oxidase subunit II PS17 (Fragments) OS=Pinus strobus PE=1 SV=1	PS17_PINST	1707	9.63	2	48	49.079	26	74.872

Peptide Information

Calc. Mass	Obsrv. Mass	± da	± ppm	Start Seq.	End Seq.	Sequence	Ion Score	C. I. %	Modification	Rank	Result Type
856.525	856.4147	-0.1103	-129	1	8	SPTVIALR					Mascot
870.5043	870.4346	-0.0697	-80	9	16	VVEALSPR					Mascot
870.5043	870.4346	-0.0697	-80	9	16	VVEALSPR	26	74.872			Mascot
	870.4346										Mascot

Rank	Protein Name	Accession No	Protein MW	Protein PI	Pep. Count	Protein Score	Protein Score C. I. %	Total Ion Score	Total Ion C. I. %
7	Glutamine synthetase root isozyme 1 OS=Zea ma GN=GLN6 PE=2 SV=1	GLNA1_MAIZ	39225.5	5.6	4	44	0	31	92.464

Peptide Information

Calc. Mass	Obsrv. Mass	± da	± ppm	Start Seq.	End Seq.	Sequence	Ion Score	C. I. %	Modification	Rank	Result Type
834.3951	834.3495	-0.0456	-55	320	326	DTEREGK					Mascot
1377.6909	1377.5734	-0.1175	-85	166	177	AFGRDVVDAHYK					Mascot
2487.1465	2486.874	-0.2725	-110	85	106	GNHILVMCDCYTPQGEPIPTNK	9	0	Carbamidomethyl (C)[10]		Mascot
2487.1465	2486.874	-0.2725	-110	85	106	GNHILVMCDCYTPQGEPIPTNK			Carbamidomethyl (C)[8]		Mascot
2643.2476	2642.948	-0.2996	-113	85	107	GNHILVMCDCYTPQGEPIPTNKR	22	34.814	Carbamidomethyl (C)[8]		Mascot
2643.2476	2642.948	-0.2996	-113	85	107	GNHILVMCDCYTPQGEPIPTNKR			Carbamidomethyl (C)[8]		Mascot

Rank	Protein Name	Accession No	Protein MW	Protein PI	Pep. Count	Protein Score	Protein Score C. I. %	Total Ion Score	Total Ion C. I. %
8	Glutamine synthetase, chloroplastic OS=Brassic napus GN=GLN2 PE=2 SV=1	GLNAC_BRAN	47314.9	6.16	6	40	0	21	26.523

Peptide Information

Calc. Mass	Obsrv. Mass	± da	± ppm	Start Seq.	End Seq.	Sequence	Ion Score	C. I. %	Modification	Rank	Result Type
899.5672	899.453	-0.1142	-127	325	332	AILNLSLR					Mascot
899.5672	899.453	-0.1142	-127	325	332	AILNLSLR	21	26.523			Mascot
948.4421	948.3303	-0.1118	-118	226	233	DISDAHYK					Mascot
1367.6624	1367.4923	-0.1701	-124	312	323	SMREDGGFEVIK					Mascot
1775.969	1775.7281	-0.2409	-136	75	90	IIAEYIWIGGSGIDLR					Mascot
1810.9116	1810.6432	-0.2684	-148	44	59	SFKVMALQSDNSTINR					Mascot
1817.8566	1817.7098	-0.1468	-81	352	367	HETASIDQFSWGVANR					Mascot

Rank	Protein Name	Accession No	Protein MW	Protein PI	Pep. Count	Protein Score	Protein Score C. I. %	Total Ion Score	Total Ion C. I. %
9	Cytochrome P450 71B21 OS=Arabidopsis thalian GN=CYP71B21 PE=3 SV=1	C71BL_ARAT	56947.6	6.45	10	37	0		

Peptide Information

Calc. Mass	Obsrv. Mass	± da	± ppm	Start Seq.	End Seq.	Sequence	Ion Score	C. I. %	Modification	Rank	Result Type
825.3658	825.3579	-0.0079	-10	374	380	ETMSDVK			Oxidation (M)[3]		Mascot
842.4995	842.4047	-0.0948	-113	237	243	LHKAFAR					Mascot
1045.5524	1045.4115	-0.1409	-135	333	341	ELLGDNKEK					Mascot
1495.7573	1495.5947	-0.1626	-109	389	401	NTMIEINTYAIGR					Mascot
1511.7522	1511.5667	-0.1855	-123	389	401	NTMIEINTYAIGR			Oxidation (M)[3]		Mascot
1688.9177	1688.6758	-0.2419	-143	154	168	KVTESAQTQTLVDLR					Mascot
1688.9177	1688.6758	-0.2419	-143	155	169	VTESAQTQTLVDLRK					Mascot
2088.8789	2088.8835	0.0046	2	182	198	LAFGQNFHECDFVDMDK			Carbamidomethyl (C)[10], Oxidation (M)[15]		Mascot
2170.0808	2169.7749	-0.3059	-141	79	97	EAAEEVLKTHDLETCTRPK					Mascot
2643.3235	2642.948	-0.3755	-142	87	109	THDLETCTRPKLSATGLFTYNFK					Mascot
2643.3235	2642.948	-0.3755	-142	87	109	THDLETCTRPKLSATGLFTYNFK					Mascot
3457.5649	3457.197	-0.3679	-106	170	198	ALFSYTASIVCRLAFGQNFHECDFVDMDK			Carbamidomethyl (C)[11,22], Oxidation (M)[27]		Mascot

Rank	Protein Name	Accession No	Protein MW	Protein PI	Pep. Count	Protein Score	Protein Score C. I. %	Total Ion Score	Total Ion C. I. %
10	Glutamine synthetase leaf isozyme, chloroplastic OS=Pisum sativum GN=GS2 PE=2 SV=2	GLNA2_PEA	47315.8	6.18	5	35	0	21	26.523

Peptide Information

Calc. Mass	Obsrv. Mass	± da	± ppm	Start Seq.	End Seq.	Sequence	Ion Score	C. I. %	Modification	Rank	Result Type
899.5672	899.453	-0.1142	-127	327	334	AILNLSLR					Mascot
899.5672	899.453	-0.1142	-127	327	334	AILNLSLR	21	26.523			Mascot
948.4421	948.3303	-0.1118	-118	228	235	DISDAHYK					Mascot
976.5098	976.382	-0.1278	-131	168	176	AAEIFSNPK					Mascot
1367.6624	1367.4923	-0.1701	-124	314	325	SMREDGGFEVIK					Mascot
1803.8408	1803.6493	-0.1915	-106	354	369	HETASINDFSWGVANR					Mascot

Gel Idx/Pos	61/C10	Instr./Gel Origin	BA2060/131017A	Process Sta	Analysis Succeeded
Plate [#] Name	[1] 31496	Instrument Sample Name		Spectra	11

Rank	Protein Name	Accession No	Protein MW	Protein PI	Pep. Count	Protein Score	Protein Score C. I. %	Total Ion Score	Total Ion C. I. %
1	S-adenosylmethionine synthase 4 OS=Vitis vinife GN=METK4 PE=3 SV=1	METK4_VITV	42903.7	5.5	17	480	100	356	100

Peptide Information

Calc. Mass	Obsrv. Mass	± da	± ppm	Start Seq.	End Seq.	Sequence	Ion Score	C. I. %	Modification	Rank	Result Type
979.4744	979.3508	-0.1236	-126	365	373	TAAYGHFGR					Mascot
979.4744	979.3508	-0.1236	-126	365	373	TAAYGHFGR	31	92.677			Mascot
1141.6113	1141.4637	-0.1476	-129	228	237	TIFHLNPSGR	17	0			Mascot
1141.6113	1141.4637	-0.1476	-129	228	237	TIFHLNPSGR					Mascot
1288.7623	1288.5753	-0.187	-145	212	222	EHVIKPIVPEK					Mascot
1400.674	1400.4852	-0.1888	-135	158	169	NGTCPWLRPDGK			Carbamidomethyl (C)[4]		Mascot
1400.674	1400.4852	-0.1888	-135	158	169	NGTCPWLRPDGK	12	0	Carbamidomethyl (C)[4]		Mascot
1453.7546	1453.5632	-0.1914	-132	238	252	FVIGGPHGDAGLTGR	52	99.939			Mascot
1453.7546	1453.5632	-0.1914	-132	238	252	FVIGGPHGDAGLTGR					Mascot
1528.7689	1528.5631	-0.2058	-135	157	169	KNGTCPWLRPDGK			Carbamidomethyl (C)[5]		Mascot
1688.853	1688.6241	-0.2289	-136	374	387	DDPDFTWEVVKPLK	37	97.988			Mascot
1688.853	1688.6241	-0.2289	-136	374	387	DDPDFTWEVVKPLK					Mascot
1963.9661	1963.6958	-0.2703	-138	254	273	IIIDTYGGWGAHGGGAFSGK					Mascot
1982.0051	1981.7402	-0.2649	-134	339	355	ESFDFRPGMIAINLDLK	26	79.408	Oxidation (M)[9]		Mascot
1982.0051	1981.7402	-0.2649	-134	339	355	ESFDFRPGMIAINLDLK			Oxidation (M)[9]		Mascot
2092.061	2091.7729	-0.2881	-138	253	273	KIIIDTYGGWGAHGGGAFSGK					Mascot
2138.1062	2137.8076	-0.2986	-140	339	356	ESFDFRPGMIAINLDLKR			Oxidation (M)[9]		Mascot
2383.2727	2382.9517	-0.321	-135	92	113	VLVNIEQQSPDIAQGVHGHLTK					Mascot
2383.2727	2382.9517	-0.321	-135	92	113	VLVNIEQQSPDIAQGVHGHLTK	100	100			Mascot
2392.0642	2391.7417	-0.3225	-135	19	39	LCDQISDAVLDACLEQDPDSK			Carbamidomethyl (C)[2,13]		Mascot
2405.1885	2404.8665	-0.322	-134	254	277	IIIDTYGGWGAHGGGAFSGKDPTK					Mascot
2649.3096	2648.9648	-0.3448	-130	365	387	TAAYGHFGRDDPDFTWEVVKPLK					Mascot
2649.3367	2648.9648	-0.3719	-140	188	211	VHTVLISTQHDETVTNDEIAADLK	83	100			Mascot
3697.7625	3697.238	-0.5245	-142	114	147	RPEEIGAGDQGHMFGYATDETPELMPLSHVLATK					Mascot
3713.7573	3713.26	-0.4973	-134	114	147	RPEEIGAGDQGHMFGYATDETPELMPLSHVLATK			Oxidation (M)[13]		Mascot

Rank	Protein Name	Accession No	Protein MW	Protein PI	Pep. Count	Protein Score	Protein Score C. I. %	Total Ion Score	Total Ion C. I. %
2	S-adenosylmethionine synthase OS=Medicago fa GN=SAMS PE=2 SV=1	METK_MEDF	43131.8	5.77	14	478	100	389	100

Peptide Information

Calc. Mass	Obsrv. Mass	± da	± ppm	Start Seq.	End Seq.	Sequence	Ion Score	C. I. %	Modification	Rank	Result Type
900.5261	900.4092	-0.1169	-130	294	302	SIVANGLAR					Mascot
979.4744	979.3508	-0.1236	-126	367	375	TAAYGHFGR					Mascot
979.4744	979.3508	-0.1236	-126	367	375	TAAYGHFGR	31	92.677			Mascot
1141.6113	1141.4637	-0.1476	-129	230	239	TIFHLNPSGR	17	0			Mascot
1141.6113	1141.4637	-0.1476	-129	230	239	TIFHLNPSGR					Mascot
1400.674	1400.4852	-0.1888	-135	160	171	NGTCPWLRPDGK			Carbamidomethyl (C)[4]		Mascot
1400.674	1400.4852	-0.1888	-135	160	171	NGTCPWLRPDGK	12	0	Carbamidomethyl (C)[4]		Mascot
1453.7546	1453.5632	-0.1914	-132	240	254	FVIGGPHGDAGLTGR	52	99.939			Mascot
1453.7546	1453.5632	-0.1914	-132	240	254	FVIGGPHGDAGLTGR					Mascot
1470.7332	1470.5389	-0.1943	-132	50	62	TNMVMVFGEITTK					Mascot
1528.7689	1528.5631	-0.2058	-135	159	171	KNGTCPWLRPDGK			Carbamidomethyl (C)[5]		Mascot
1838.8225	1838.577	-0.2455	-134	77	93	NIGFVSDDVGLDADNCK			Carbamidomethyl (C)[16]		Mascot
1838.8225	1838.577	-0.2455	-134	77	93	NIGFVSDDVGLDADNCK	95	100	Carbamidomethyl (C)[16]		Mascot
1963.9661	1963.6958	-0.2703	-138	256	275	IIIDTYGGWGAHGGGAFSGK					Mascot
2092.061	2091.7729	-0.2881	-138	255	275	KIIIDTYGGWGAHGGGAFSGK					Mascot
2383.2727	2382.9517	-0.321	-135	94	115	VLVNIEQQSPDIAQGVHGHLTK					Mascot
2383.2727	2382.9517	-0.321	-135	94	115	VLVNIEQQSPDIAQGVHGHLTK	100	100			Mascot
2392.0642	2391.7417	-0.3225	-135	21	41	LCDQISDAVLDACLEQDPDSK			Carbamidomethyl (C)[2,13]		Mascot
2405.1885	2404.8665	-0.322	-134	256	279	IIIDTYGGWGAHGGGAFSGKDPTK					Mascot
2649.3367	2648.9648	-0.3719	-140	190	213	VHTVLISTQHDETVTNDEIAADLK					Mascot
2649.3367	2648.9648	-0.3719	-140	190	213	VHTVLISTQHDETVTNDEIAADLK	83	100			Mascot

Rank	Protein Name	Accession No	Protein MW	Protein PI	Pep. Count	Protein Score	Protein Score C. I. %	Total Ion Score	Total Ion C. I. %
3	S-adenosylmethionine synthase 2 OS=Vitis vinife GN=METK2 PE=3 SV=1	METK2_VITV	43086.8	5.65	17	452	100	330	100

Peptide Information

Calc. Mass	Obsrv. Mass	± da	± ppm	Start Seq.	End Seq.	Sequence	Ion Score	C. I. %	Modification	Rank	Result Type
900.5261	900.4092	-0.1169	-130	292	300	SIVANGLAR					Mascot
979.4744	979.3508	-0.1236	-126	365	373	TAAYGHFGR					Mascot
979.4744	979.3508	-0.1236	-126	365	373	TAAYGHFGR	31	92.677			Mascot
1141.6113	1141.4637	-0.1476	-129	228	237	TIFHLNPSGR	17	0			Mascot
1141.6113	1141.4637	-0.1476	-129	228	237	TIFHLNPSGR					Mascot
1288.7623	1288.5753	-0.187	-145	212	222	EHVIKPVIPEK					Mascot
1400.674	1400.4852	-0.1888	-135	158	169	NGTCPWLRPDGK			Carbamidomethyl (C)[4]		Mascot
1400.674	1400.4852	-0.1888	-135	158	169	NGTCPWLRPDGK	12	0	Carbamidomethyl (C)[4]		Mascot
1453.7546	1453.5632	-0.1914	-132	238	252	FVIGGPHGDAGLTGR	52	99.939			Mascot
1453.7546	1453.5632	-0.1914	-132	238	252	FVIGGPHGDAGLTGR					Mascot
1470.7332	1470.5389	-0.1943	-132	48	60	TNMVMVFGEITTK					Mascot
1528.7689	1528.5631	-0.2058	-135	157	169	KNGTCPWLRPDGK			Carbamidomethyl (C)[5]		Mascot
1688.853	1688.6241	-0.2289	-136	374	387	DDPDFTWEVVKPLK	37	97.988			Mascot
1688.853	1688.6241	-0.2289	-136	374	387	DDPDFTWEVVKPLK					Mascot
1963.9661	1963.6958	-0.2703	-138	254	273	IIIDTYGGWGAHGGGAFSGK					Mascot
2025.011	2024.7406	-0.2704	-134	339	355	ENFDFRPGMISINLDLK			Oxidation (M)[9]		Mascot
2092.061	2091.7729	-0.2881	-138	253	273	KIIIDTYGGWGAHGGGAFSGK					Mascot
2383.2727	2382.9517	-0.321	-135	92	113	VLVNIEQQSPDIAQGVHGHLTK					Mascot
2383.2727	2382.9517	-0.321	-135	92	113	VLVNIEQQSPDIAQGVHGHLTK	100	100			Mascot
2405.1885	2404.8665	-0.322	-134	254	277	IIIDTYGGWGAHGGGAFSGKDPTK					Mascot
2649.3096	2648.9648	-0.3448	-130	365	387	TAAYGHFGRDDPDFTWEVVKPLK					Mascot
2649.3367	2648.9648	-0.3719	-140	188	211	VHTVLISTQHDETVTNDEIAADLK	83	100			Mascot
3697.7625	3697.238	-0.5245	-142	114	147	RPEEIGAGDQGHMFGYATDETPELMPLSHVLATK					Mascot
3713.7573	3713.26	-0.4973	-134	114	147	RPEEIGAGDQGHMFGYATDETPELMPLSHVLATK			Oxidation (M)[13]		Mascot

Rank	Protein Name	Accession No	Protein MW	Protein PI	Pep. Count	Protein Score	Protein Score C. I. %	Total Ion Score	Total Ion C. I. %
4	S-adenosylmethionine synthase 4 OS=Arabidopsi thaliana GN=METK4 PE=1 SV=1	METK4_ARAT	42768.7	5.51	15	428	100	328	100

Peptide Information

Calc. Mass	Obsrv. Mass	± da	± ppm	Start Seq.	End Seq.	Sequence	Ion Score	C. I. %	Modification	Rank	Result Type
979.4744	979.3508	-0.1236	-126	365	373	TAAYGHFGR					Mascot
979.4744	979.3508	-0.1236	-126	365	373	TAAYGHFGR	31	92.677			Mascot
1141.6113	1141.4637	-0.1476	-129	228	237	TIFHLNPSGR	17	0			Mascot
1141.6113	1141.4637	-0.1476	-129	228	237	TIFHLNPSGR					Mascot
1288.7623	1288.5753	-0.187	-145	212	222	EHVIKPVIPEK					Mascot
1400.674	1400.4852	-0.1888	-135	158	169	NGTCPWLRPDGK			Carbamidomethyl (C)[4]		Mascot
1400.674	1400.4852	-0.1888	-135	158	169	NGTCPWLRPDGK	12	0	Carbamidomethyl (C)[4]		Mascot
1453.7546	1453.5632	-0.1914	-132	238	252	FVIGGPHGDAGLTGR	52	99.939			Mascot
1453.7546	1453.5632	-0.1914	-132	238	252	FVIGGPHGDAGLTGR					Mascot
1470.7332	1470.5389	-0.1943	-132	48	60	TNMVMVFGEITTK					Mascot
1528.7689	1528.5631	-0.2058	-135	157	169	KNGTCPWLRPDGK			Carbamidomethyl (C)[5]		Mascot
1963.9661	1963.6958	-0.2703	-138	254	273	IIIDTYGGWGAHGGGAFSGK					Mascot
1982.0051	1981.7402	-0.2649	-134	339	355	ESFDFRPGMISINLDLK	33	95.128			Mascot
1982.0051	1981.7402	-0.2649	-134	339	355	ESFDFRPGMISINLDLK					Mascot
1998.0001	1997.7345	-0.2656	-133	339	355	ESFDFRPGMISINLDLK			Oxidation (M)[9]		Mascot
2092.061	2091.7729	-0.2881	-138	253	273	KIIIDTYGGWGAHGGGAFSGK					Mascot
2138.1062	2137.8076	-0.2986	-140	339	356	ESFDFRPGMISINLDLKR					Mascot
2154.1011	2153.7957	-0.3054	-142	339	356	ESFDFRPGMISINLDLKR			Oxidation (M)[9]		Mascot
2169.9902	2169.699	-0.2912	-134	72	91	TCREIGFVSADVGLDADNCK			Carbamidomethyl (C)[2]		Mascot
2383.2727	2382.9517	-0.321	-135	92	113	VLVNIEQQSPDIAQGVHGHLTK					Mascot
2383.2727	2382.9517	-0.321	-135	92	113	VLVNIEQQSPDIAQGVHGHLTK	100	100			Mascot
2405.1885	2404.8665	-0.322	-134	254	277	IIIDTYGGWGAHGGGAFSGKDPTK					Mascot
2649.3367	2648.9648	-0.3719	-140	188	211	VHTVLISTQHDETVTNDEIAADLK					Mascot
2649.3367	2648.9648	-0.3719	-140	188	211	VHTVLISTQHDETVTNDEIAADLK	83	100			Mascot

Rank	Protein Name	Accession No	Protein MW	Protein PI	Pep. Count	Protein Score	Protein Score C. I. %	Total Ion Score	Total Ion C. I. %
5	S-adenosylmethionine synthase 5 OS=Vitis vinife GN=METK5 PE=3 SV=1	METK5_VITV	42765.6	5.57	14	407	100	317	100

Peptide Information

Calc. Mass	Obsrv. Mass	± da	± ppm	Start Seq.	End Seq.	Sequence	Ion Score	C. I. %	Modification	Rank	Result Type
900.5261	900.4092	-0.1169	-130	292	300	SIVANGLAR					Mascot
979.4744	979.3508	-0.1236	-126	365	373	TAAYGHFGR					Mascot
979.4744	979.3508	-0.1236	-126	365	373	TAAYGHFGR	31	92.677			Mascot
1141.6113	1141.4637	-0.1476	-129	228	237	TIFHLNPSGR	17	0			Mascot
1141.6113	1141.4637	-0.1476	-129	228	237	TIFHLNPSGR					Mascot
1288.7623	1288.5753	-0.187	-145	212	222	EHVIKPVIPEK					Mascot
1453.7546	1453.5632	-0.1914	-132	238	252	FVIGGPHGDAGLTGR	52	99.939			Mascot
1453.7546	1453.5632	-0.1914	-132	238	252	FVIGGPHGDAGLTGR					Mascot
1470.7332	1470.5389	-0.1943	-132	48	60	TNMVMVFGEITTK					Mascot
1688.853	1688.6241	-0.2289	-136	374	387	DDPDFTWEVVKPLK	37	97.988			Mascot
1688.853	1688.6241	-0.2289	-136	374	387	DDPDFTWEVVKPLK					Mascot
1963.9661	1963.6958	-0.2703	-138	254	273	IIIDTYGGWGAHGGGAFSGK					Mascot
2092.061	2091.7729	-0.2881	-138	253	273	KIIIDTYGGWGAHGGGAFSGK					Mascot
2383.2727	2382.9517	-0.321	-135	92	113	VLVNIEQQSPDIAQGVHGHLTK					Mascot
2383.2727	2382.9517	-0.321	-135	92	113	VLVNIEQQSPDIAQGVHGHLTK	100	100			Mascot
2405.1885	2404.8665	-0.322	-134	254	277	IIIDTYGGWGAHGGGAFSGKDPTK					Mascot
2649.3096	2648.9648	-0.3448	-130	365	387	TAAYGHFGRDDPDFTWEVVKPLK					Mascot
2649.3367	2648.9648	-0.3719	-140	188	211	VHTVLISTQHDETVTNDEIAADLK	83	100			Mascot
3697.7625	3697.238	-0.5245	-142	114	147	RPEEIGAGDQGHMFGYATDETPELMPLSHVLATK					Mascot
3713.7573	3713.26	-0.4973	-134	114	147	RPEEIGAGDQGHMFGYATDETPELMPLSHVLATK			Oxidation (M)[13]		Mascot

Rank	Protein Name	Accession No	Protein MW	Protein PI	Pep. Count	Protein Score	Protein Score C. I. %	Total Ion Score	Total Ion C. I. %
6	S-adenosylmethionine synthase 2 OS=Pisum sat GN=SAMS2 PE=2 SV=1	METK2_PEA	40950.4	5.19	13	386	100	307	100

Peptide Information

Calc. Mass	Obsrv. Mass	± da	± ppm	Start Seq.	End Seq.	Sequence	Ion Score	C. I. %	Modification	Rank	Result Type
900.5261	900.4092	-0.1169	-130	294	302	SIVANGLAR					Mascot
1141.6113	1141.4637	-0.1476	-129	230	239	TIFHLNPSGR	17	0			Mascot
1141.6113	1141.4637	-0.1476	-129	230	239	TIFHLNPSGR					Mascot
1288.7623	1288.5753	-0.187	-145	214	224	EHVIKPVIPEK					Mascot
1359.6427	1359.4667	-0.176	-129	172	182	TQVTVEYYNDK					Mascot
1400.674	1400.4852	-0.1888	-135	160	171	NGTCPWLRPDGK			Carbamidomethyl (C)[4]		Mascot
1400.674	1400.4852	-0.1888	-135	160	171	NGTCPWLRPDGK	12	0	Carbamidomethyl (C)[4]		Mascot
1470.7332	1470.5389	-0.1943	-132	50	62	TNMVMVFGEITTK					Mascot
1528.7689	1528.5631	-0.2058	-135	159	171	KNGTCPWLRPDGK			Carbamidomethyl (C)[5]		Mascot
1838.8225	1838.577	-0.2455	-134	77	93	NIGFVSDDVGLDADNCK			Carbamidomethyl (C)[16]		Mascot
1838.8225	1838.577	-0.2455	-134	77	93	NIGFVSDDVGLDADNCK	95	100	Carbamidomethyl (C)[16]		Mascot
1963.9661	1963.6958	-0.2703	-138	256	275	IIIDTYGGWGAHGGGAFSGK					Mascot
2092.061	2091.7729	-0.2881	-138	255	275	KIIIDTYGGWGAHGGGAFSGK					Mascot
2383.2727	2382.9517	-0.321	-135	94	115	VLVNIEQQSPDIAQGVHGHLTK					Mascot
2383.2727	2382.9517	-0.321	-135	94	115	VLVNIEQQSPDIAQGVHGHLTK	100	100			Mascot
2405.1885	2404.8665	-0.322	-134	256	279	IIIDTYGGWGAHGGGAFSGKDPTK					Mascot
2649.3367	2648.9648	-0.3719	-140	190	213	VHTVLISTQHDETVTNDEIAADLK					Mascot
2649.3367	2648.9648	-0.3719	-140	190	213	VHTVLISTQHDETVTNDEIAADLK	83	100			Mascot

Rank	Protein Name	Accession No	Protein MW	Protein PI	Pep. Count	Protein Score	Protein Score C. I. %	Total Ion Score	Total Ion C. I. %
7	S-adenosylmethionine synthase 2 OS=Brassica j GN=MSAMS2 PE=2 SV=1	METK2_BRA	42852.6	5.37	12	385	100	315	100

Peptide Information

Calc. Mass	Obsrv. Mass	± da	± ppm	Start Seq.	End Seq.	Sequence	Ion Score	C. I. %	Modification	Rank	Result Type
979.4744	979.3508	-0.1236	-126	365	373	TAAYGHFGR					Mascot
979.4744	979.3508	-0.1236	-126	365	373	TAAYGHFGR	31	92.677			Mascot
1141.6113	1141.4637	-0.1476	-129	228	237	TIFHLNPSGR	17	0			Mascot
1141.6113	1141.4637	-0.1476	-129	228	237	TIFHLNPSGR					Mascot
1288.7623	1288.5753	-0.187	-145	212	222	EHVIKPVIPEK					Mascot
1453.7546	1453.5632	-0.1914	-132	238	252	FVIGGPHGDAGLTGR	52	99.939			Mascot
1453.7546	1453.5632	-0.1914	-132	238	252	FVIGGPHGDAGLTGR					Mascot
1470.7332	1470.5389	-0.1943	-132	48	60	TNMVMVFGEITTK					Mascot
1963.9661	1963.6958	-0.2703	-138	254	273	IIIDTYGGWGAHGGGAFSGK					Mascot
1982.0051	1981.7402	-0.2649	-134	339	355	ESFDFRPGMISINLDLK	33	95.128			Mascot
1982.0051	1981.7402	-0.2649	-134	339	355	ESFDFRPGMISINLDLK					Mascot
1998.0001	1997.7345	-0.2656	-133	339	355	ESFDFRPGMISINLDLK			Oxidation (M)[9]		Mascot
2092.061	2091.7729	-0.2881	-138	253	273	KIIIDTYGGWGAHGGGAFSGK					Mascot
2138.1062	2137.8076	-0.2986	-140	339	356	ESFDFRPGMISINLDLKR					Mascot
2154.1011	2153.7957	-0.3054	-142	339	356	ESFDFRPGMISINLDLKR			Oxidation (M)[9]		Mascot
2383.2727	2382.9517	-0.321	-135	92	113	VLVNIEQQSPDIAQGVHGHLTK					Mascot
2383.2727	2382.9517	-0.321	-135	92	113	VLVNIEQQSPDIAQGVHGHLTK	100	100			Mascot
2405.1885	2404.8665	-0.322	-134	254	277	IIIDTYGGWGAHGGGAFSGKDPTK					Mascot
2649.3367	2648.9648	-0.3719	-140	188	211	VHTVLISTQHDETVTNDEIAADLK					Mascot
2649.3367	2648.9648	-0.3719	-140	188	211	VHTVLISTQHDETVTNDEIAADLK	83	100			Mascot

Rank	Protein Name	Accession No	Protein MW	Protein PI	Pep. Count	Protein Score	Protein Score C. I. %	Total Ion Score	Total Ion C. I. %
8	S-adenosylmethionine synthase 2 OS=Catharanth roseus GN=SAMS2 PE=1 SV=1	METK2_CATF	42976.7	5.51	13	376	100	295	100

Peptide Information

Calc. Mass	Obsrv. Mass	± da	± ppm	Start Seq.	End Seq.	Sequence	Ion Score	C. I. %	Modification	Rank	Result Type
979.4744	979.3508	-0.1236	-126	365	373	TAAYGHFGR					Mascot
979.4744	979.3508	-0.1236	-126	365	373	TAAYGHFGR	31	92.677			Mascot
1141.6113	1141.4637	-0.1476	-129	228	237	TIFHLNPSGR	17	0			Mascot
1141.6113	1141.4637	-0.1476	-129	228	237	TIFHLNPSGR					Mascot
1400.674	1400.4852	-0.1888	-135	158	169	NGTCPWLRPDGK			Carbamidomethyl (C)[4]		Mascot
1400.674	1400.4852	-0.1888	-135	158	169	NGTCPWLRPDGK	12	0	Carbamidomethyl (C)[4]		Mascot
1453.7546	1453.5632	-0.1914	-132	238	252	FVIGGPHGDAGLTGR	52	99.939			Mascot
1453.7546	1453.5632	-0.1914	-132	238	252	FVIGGPHGDAGLTGR					Mascot
1470.7332	1470.5389	-0.1943	-132	48	60	TNMVMVFGEITTK					Mascot
1528.7689	1528.5631	-0.2058	-135	157	169	KNGTCPWLRPDGK			Carbamidomethyl (C)[5]		Mascot
1963.9661	1963.6958	-0.2703	-138	254	273	IIIDTYGGWGAHGGGAFSGK					Mascot
2092.061	2091.7729	-0.2881	-138	253	273	KIIIDTYGGWGAHGGGAFSGK					Mascot
2383.2727	2382.9517	-0.321	-135	92	113	VLVNIEQQSPDIAQGVHGHLTK					Mascot
2383.2727	2382.9517	-0.321	-135	92	113	VLVNIEQQSPDIAQGVHGHLTK	100	100			Mascot
2405.1885	2404.8665	-0.322	-134	254	277	IIIDTYGGWGAHGGGAFSGKDPTK					Mascot
2649.3367	2648.9648	-0.3719	-140	188	211	VHTVLISTQHDETVTNDEIAADLK					Mascot
2649.3367	2648.9648	-0.3719	-140	188	211	VHTVLISTQHDETVTNDEIAADLK	83	100			Mascot
2663.3252	2662.9893	-0.3359	-126	365	387	TAAYGHFGREDPDFTWEVVKPLK					Mascot
3697.7625	3697.238	-0.5245	-142	114	147	RPEEIGAGDQGHMFGYATDETPELMPLSHVLATK					Mascot
3713.7573	3713.26	-0.4973	-134	114	147	RPEEIGAGDQGHMFGYATDETPELMPLSHVLATK			Oxidation (M)[13]		Mascot

Rank	Protein Name	Accession No	Protein MW	Protein PI	Pep. Count	Protein Score	Protein Score C. I. %	Total Ion Score	Total Ion C. I. %
9	S-adenosylmethionine synthase OS=Mesembryanthemum crystallinum GN=METK PE=2 SV=1	METK_MESCI	42858.6	5.43	13	370	100	290	100

Peptide Information

Calc. Mass	Obsrv. Mass	± da	± ppm	Start Seq.	End Seq.	Sequence	Ion Score	C. I. %	Modification	Rank	Result Type
979.4744	979.3508	-0.1236	-126	364	372	TAAYGHFGR					Mascot
979.4744	979.3508	-0.1236	-126	364	372	TAAYGHFGR	31	92.677			Mascot
1141.6113	1141.4637	-0.1476	-129	228	237	TIFHLNPSGR	17	0			Mascot
1141.6113	1141.4637	-0.1476	-129	228	237	TIFHLNPSGR					Mascot
1288.7623	1288.5753	-0.187	-145	212	222	EHVIKPVIPEK					Mascot
1400.674	1400.4852	-0.1888	-135	158	169	NGTCPWLRPDGK			Carbamidomethyl (C)[4]		Mascot
1400.674	1400.4852	-0.1888	-135	158	169	NGTCPWLRPDGK	12	0	Carbamidomethyl (C)[4]		Mascot
1453.7546	1453.5632	-0.1914	-132	238	252	FVIGGPHGDAGLTGR	52	99.939			Mascot
1453.7546	1453.5632	-0.1914	-132	238	252	FVIGGPHGDAGLTGR					Mascot
1528.7689	1528.5631	-0.2058	-135	157	169	KNGTCPWLRPDGK			Carbamidomethyl (C)[5]		Mascot
1838.8225	1838.577	-0.2455	-134	75	91	NIGFVSDDVGLDADNCK			Carbamidomethyl (C)[16]		Mascot
1838.8225	1838.577	-0.2455	-134	75	91	NIGFVSDDVGLDADNCK	95	100	Carbamidomethyl (C)[16]		Mascot
1963.9661	1963.6958	-0.2703	-138	254	273	IIIDTYGGWGAHGGGAFSGK					Mascot
2092.061	2091.7729	-0.2881	-138	253	273	KIIIDTYGGWGAHGGGAFSGK					Mascot
2138.0698	2137.8076	-0.2622	-123	339	356	ETFDFRPGMIANNLDLLR			Oxidation (M)[9]		Mascot
2405.1885	2404.8665	-0.322	-134	254	277	IIIDTYGGWGAHGGGAFSGKDPTK					Mascot
2649.3367	2648.9648	-0.3719	-140	188	211	VHTVLISTQHDETVTNDEIAADLK					Mascot
2649.3367	2648.9648	-0.3719	-140	188	211	VHTVLISTQHDETVTNDEIAADLK	83	100			Mascot
3697.7625	3697.238	-0.5245	-142	114	147	RPEEIGAGDQGHMFGYATDETPELMPLSHVLATK					Mascot
3713.7573	3713.26	-0.4973	-134	114	147	RPEEIGAGDQGHMFGYATDETPELMPLSHVLATK			Oxidation (M)[13]		Mascot

Rank	Protein Name	Accession No	Protein MW	Protein PI	Pep. Count	Protein Score	Protein Score C. I. %	Total Ion Score	Total Ion C. I. %
10	S-adenosylmethionine synthase 3 (Fragment) OS=Actinidia chinensis GN=SAM3 PE=2 SV=1	METK3_ACT	39488.1	6.2	12	368	100	294	100

Peptide Information

Calc. Mass	Obsrv. Mass	± da	± ppm	Start Seq.	End Seq.	Sequence	Ion Score	C. I. %	Modification	Rank	Result Type
979.4744	979.3508	-0.1236	-126	332	340	TAAYGHFGR					Mascot
979.4744	979.3508	-0.1236	-126	332	340	TAAYGHFGR	31	92.677			Mascot
1141.6113	1141.4637	-0.1476	-129	195	204	TIFHLNPSGR	17	0			Mascot
1141.6113	1141.4637	-0.1476	-129	195	204	TIFHLNPSGR					Mascot
1401.658	1401.4824	-0.1756	-125	125	136	DGTCPWLRPDGK			Carbamidomethyl (C)[4]		Mascot
1453.7546	1453.5632	-0.1914	-132	205	219	FVIGGPHGDAGLTGR	52	99.939			Mascot
1453.7546	1453.5632	-0.1914	-132	205	219	FVIGGPHGDAGLTGR					Mascot
1470.7332	1470.5389	-0.1943	-132	15	27	TNMVMVFGEITTK					Mascot
1529.7529	1529.5604	-0.1925	-126	124	136	KDGTCPWLRPDGK			Carbamidomethyl (C)[5]		Mascot
1838.8225	1838.577	-0.2455	-134	42	58	NIGFVSDDVGLDADNCK			Carbamidomethyl (C)[16]		Mascot
1838.8225	1838.577	-0.2455	-134	42	58	NIGFVSDDVGLDADNCK	95	100	Carbamidomethyl (C)[16]		Mascot
1963.9661	1963.6958	-0.2703	-138	221	240	IIIDTYGGWGAHGGGAFSGK					Mascot
2092.061	2091.7729	-0.2881	-138	220	240	KIIIDTYGGWGAHGGGAFSGK					Mascot
2383.2727	2382.9517	-0.321	-135	59	80	VLVNIEQQSPDIAQGVHGHLTK					Mascot
2383.2727	2382.9517	-0.321	-135	59	80	VLVNIEQQSPDIAQGVHGHLTK	100	100			Mascot
2405.1885	2404.8665	-0.322	-134	221	244	IIIDTYGGWGAHGGGAFSGKDPTK					Mascot
3697.7625	3697.238	-0.5245	-142	81	114	RPEEIGAGDQGHMFGYATDETPELMPLSHVLATK					Mascot
3713.7573	3713.26	-0.4973	-134	81	114	RPEEIGAGDQGHMFGYATDETPELMPLSHVLATK			Oxidation (M)[13]		Mascot

Gel Idx/Pos	62/C11	Instr./Gel Origin	BA2060/131017A	Process Sta	Analysis Succeeded
Plate [#] Name	[1] 31496	Instrument Sample Name		Spectra	11

Rank	Protein Name	Accession No	Protein MW	Protein PI	Pep. Count	Protein Score	Protein Score C. I. %	Total Ion Score	Total Ion C. I. %
1	Probable calcium-binding protein CML33 OS=Arabidopsis thaliana GN=CML33 PE=2 SV=1	CML33_ARAT	15058.2	4.4	6	38	0		

Peptide Information

Calc. Mass	Obsrv. Mass	± da	± ppm	Start Seq.	End Seq.	Sequence	Ion Score	C. I. %	Modification	Rank	Result Type
850.4668	850.451	-0.0158	-19	80	86	ELKDAFK					Mascot
1341.6104	1341.5311	-0.0793	-59	2	12	NNMSLSDIFER			Oxidation (M)[3]		Mascot
1472.6509	1472.494	-0.1569	-107	1	12	MNNMSLSDIFER			Oxidation (M)[1]		Mascot
1488.6458	1488.4901	-0.1557	-105	1	12	MNNMSLSDIFER			Oxidation (M)[1,4]		Mascot
1917.7953	1917.7311	-0.0642	-33	62	79	FASCMDQTAQSSGGDVEK			Carbamidomethyl (C)[4]		Mascot
2403.0625	2402.8159	-0.2466	-103	112	133	CTVESCVGMVQAIDVDGDGYIR			Carbamidomethyl (C)[1], Oxidation (M)[9]		Mascot
2403.0625	2402.8159	-0.2466	-103	112	133	CTVESCVGMVQAIDVDGDGYIR			Carbamidomethyl (C)[1], Oxidation (M)[9]		Mascot
2748.2676	2747.957	-0.3106	-113	112	137	CTVESCVGMVQAIDVDGDGYIRFVGV			Oxidation (M)[9]		Mascot

Rank	Protein Name	Accession No	Protein MW	Protein PI	Pep. Count	Protein Score	Protein Score C. I. %	Total Ion Score	Total Ion C. I. %
2	Ribulose bisphosphate carboxylase large chain OS=Cucumis sativus GN=rbcL PE=1 SV=5	RBL_CUCSA	52608.4	6	9	34	0		

Peptide Information

Calc. Mass	Obsrv. Mass	± da	± ppm	Start Seq.	End Seq.	Sequence	Ion Score	C. I. %	Modification	Rank	Result Type
1021.5312	1021.4146	-0.1166	-114	33	41	DTDILAAFR					Mascot
1275.7307	1275.5737	-0.157	-123	340	350	EITLGFVDLLR					Mascot
1343.6525	1343.5164	-0.1361	-101	184	194	NYGRAVYECLR					Mascot
1407.6678	1407.4601	-0.2077	-148	22	32	LTYYTPEYETK					Mascot
1465.7216	1465.5837	-0.1379	-94	320	334	MSGGDHIHAGTVVGK					Mascot
1465.7546	1465.5837	-0.1709	-117	147	159	TFQGPPHGIQVER					Mascot
1472.6978	1472.494	-0.2038	-138	464	475	EIKFEFEAMDTL					Mascot
1488.6926	1488.4901	-0.2025	-136	464	475	EIKFEFEAMDTL			Oxidation (M)[9]		Mascot
2169.9868	2169.7346	-0.2522	-116	195	213	GGLDFTKDDENVNSQPFMR					Mascot
2953.397	2953.2537	-0.1433	-49	259	285	ELGAPIVMHDYLTGGFTANTSLAHYCR			Oxidation (M)[8]		Mascot

Rank	Protein Name	Accession No	Protein MW	Protein PI	Pep. Count	Protein Score	Protein Score C. I. %	Total Ion Score	Total Ion C. I. %
3	Phosphoribulokinase, chloroplastic OS=Mesembryanthemum crystallinum PE=2 SV=1	KPPR_MESC	44086.5	6.03	7	34	0	7	0

Peptide Information

Calc. Mass	Obsrv. Mass	± da	± ppm	Start Seq.	End Seq.	Sequence	Ion Score	C. I. %	Modification	Rank	Result Type
870.4679	870.4402	-0.0277	-32	211	218	GHSLESIK					Mascot
870.4679	870.4402	-0.0277	-32	211	218	GHSLESIK					Mascot
1343.6664	1343.5164	-0.15	-112	344	354	FYGEVTQQMLK					Mascot
1359.6613	1359.5009	-0.1604	-118	344	354	FYGEVTQQMLK			Oxidation (M)[9]		Mascot
1472.6726	1472.494	-0.1786	-121	125	136	ANDFDLMYEQVK					Mascot
1488.6675	1488.4901	-0.1774	-119	125	136	ANDFDLMYEQVK			Oxidation (M)[7]		Mascot
1626.8672	1626.6644	-0.2028	-125	167	180	ILVIEGLHPMFDSR					Mascot
1642.8621	1642.6566	-0.2055	-125	167	180	ILVIEGLHPMFDSR	7	0	Oxidation (M)[10]		Mascot
1642.8621	1642.6566	-0.2055	-125	167	180	ILVIEGLHPMFDSR			Oxidation (M)[10]		Mascot
1883.9636	1883.7227	-0.2409	-128	183	198	DLLDFSIYLDISNEVK					Mascot
1961.0226	1960.7926	-0.23	-117	327	343	LDELIYVESHLSNLSTK					Mascot
2298.1069	2297.916	-0.1909	-83	117	136	GVTALDPRANDFDLMYEQVK			Oxidation (M)[15]		Mascot

Rank	Protein Name	Accession No	Protein MW	Protein PI	Pep. Count	Protein Score	Protein Score C. I. %	Total Ion Score	Total Ion C. I. %
4	Phosphoribulokinase, chloroplastic OS=Arabidop thaliana GN=At1g32060 PE=1 SV=1	KPPR_ARAT	44435.6	5.71	8	34	0		

Peptide Information

Calc. Mass	Obsrv. Mass	± da	± ppm	Start Seq.	End Seq.	Sequence	Ion Score	C. I. %	Modification	Rank	Result Type
870.4679	870.4402	-0.0277	-32	210	217	GHSLESIK					Mascot
870.4679	870.4402	-0.0277	-32	210	217	GHSLESIK					Mascot
1343.6664	1343.5164	-0.15	-112	343	353	FYGEVTQQMLK					Mascot
1359.6613	1359.5009	-0.1604	-118	343	353	FYGEVTQQMLK			Oxidation (M)[9]		Mascot
1472.6726	1472.494	-0.1786	-121	124	135	ANDFDLMYEQVK					Mascot
1488.6675	1488.4901	-0.1774	-119	124	135	ANDFDLMYEQVK			Oxidation (M)[7]		Mascot
1883.9636	1883.7227	-0.2409	-128	182	197	DLLDFSIYLDISNEVK					Mascot
1961.0226	1960.7926	-0.23	-117	326	342	LDELIYVESHLSNLSTK					Mascot
2173.1035	2172.8254	-0.2781	-128	354	374	HADFPGSNNGTGLFQTIVGLK					Mascot
2225.0906	2224.8384	-0.2522	-113	117	135	VTALDPRANDFDLMYEQVK					Mascot
2664.2822	2663.9587	-0.3235	-121	1	25	MAVSTIYSTQALNSTHFLTSSSSSK			Oxidation (M)[1]		Mascot

Rank	Protein Name	Accession No	Protein MW	Protein PI	Pep. Count	Protein Score	Protein Score C. I. %	Total Ion Score	Total Ion C. I. %
5	Ribulose bisphosphate carboxylase large chain OS=Nandina domestica GN=rbcL PE=3 SV=3	RBL_NANDO	52643.5	6.22	9	33	0		

Peptide Information

Calc. Mass	Obsrv. Mass	± da	± ppm	Start Seq.	End Seq.	Sequence	Ion Score	C. I. %	Modification	Rank	Result Type
1021.5312	1021.4146	-0.1166	-114	33	41	DTDILAAFR					Mascot
1275.7307	1275.5737	-0.157	-123	340	350	EITLGFVDLLR					Mascot
1343.6525	1343.5164	-0.1361	-101	184	194	NYGRAVYECLR					Mascot
1465.7216	1465.5837	-0.1379	-94	320	334	MSGGDHIHAGTVVGK					Mascot
1465.7546	1465.5837	-0.1709	-117	147	159	TFQGPPHGIQVER					Mascot
1472.6978	1472.494	-0.2038	-138	464	475	EIKFEFEAMDTL					Mascot
1488.6926	1488.4901	-0.2025	-136	464	475	EIKFEFEAMDTL			Oxidation (M)[9]		Mascot
2169.9868	2169.7346	-0.2522	-116	195	213	GGLDFTKDDENVNSQPFMR					Mascot
2173.0996	2172.8254	-0.2742	-126	218	236	FLFCAEALFKAQAETGEIK			Carbamidomethyl (C)[4]		Mascot
2740.2375	2739.9788	-0.2587	-94	228	252	AQAETGEIKGHYLNATAGTCEEMMK			Carbamidomethyl (C)[20]		Mascot

Rank	Protein Name	Accession No	Protein MW	Protein PI	Pep. Count	Protein Score	Protein Score C. I. %	Total Ion Score	Total Ion C. I. %
6	Phosphoribulokinase, chloroplastic OS=Spinaci oleracea PE=1 SV=1	KPPR_SPIOL	44978.7	5.82	7	31	0	4	0

Peptide Information

Calc. Mass	Obsrv. Mass	± da	± ppm	Start Seq.	End Seq.	Sequence	Ion Score	C. I. %	Modification	Rank	Result Type
870.4679	870.4402	-0.0277	-32	216	223	GHSLESIK					Mascot
870.4679	870.4402	-0.0277	-32	216	223	GHSLESIK					Mascot
1273.6787	1273.5236	-0.1551	-122	76	88	LTSVFGGAAEPPK					Mascot
1343.6664	1343.5164	-0.15	-112	349	359	FYGEVTQQMLK					Mascot
1359.6613	1359.5009	-0.1604	-118	349	359	FYGEVTQQMLK			Oxidation (M)[9]		Mascot
1472.6726	1472.494	-0.1786	-121	130	141	ANDFDLMYEQVK					Mascot
1488.6675	1488.4901	-0.1774	-119	130	141	ANDFDLMYEQVK			Oxidation (M)[7]		Mascot
1626.8672	1626.6644	-0.2028	-125	172	185	ILVIEGLHPMYDAR					Mascot
1642.8621	1642.6566	-0.2055	-125	172	185	ILVIEGLHPMYDAR	4	0	Oxidation (M)[10]		Mascot
1642.8621	1642.6566	-0.2055	-125	172	185	ILVIEGLHPMYDAR			Oxidation (M)[10]		Mascot
1961.0226	1960.7926	-0.23	-117	332	348	LDELIYVESHLSNLSTK					Mascot
2808.1804	2807.9333	-0.2471	-88	308	331	FSYGPDTFYGNEVTVVEMDGMFDR			Oxidation (M)[18,21]		Mascot

Rank	Protein Name	Accession No	Protein MW	Protein PI	Pep. Count	Protein Score	Protein Score C. I. %	Total Ion Score	Total Ion C. I. %
7	CRS2-associated factor 2, chloroplastic OS=Zea GN=CAF2 PE=1 SV=1	CAF2P_MAIZ	68025.9	8.5	10	31	0		

Peptide Information

Calc. Mass	Obsrv. Mass	± da	± ppm	Start Seq.	End Seq.	Sequence	Ion Score	C. I. %	Modification	Rank	Result Type
1051.4473	1051.5801	0.1328	126	355	362	EEADEMRR			Oxidation (M)[6]		Mascot
1273.6317	1273.5236	-0.1081	-85	213	224	MDLARVSPGDGR					Mascot
1450.7173	1450.5415	-0.1758	-121	385	397	DVRDAFEGSDLVK					Mascot
1570.6115	1570.5919	-0.0196	-12	578	589	DDEHFDVDMFER			Oxidation (M)[9]		Mascot
1570.6115	1570.5919	-0.0196	-12	578	589	DDEHFDVDMFER			Oxidation (M)[9]		Mascot
1626.8711	1626.6644	-0.2067	-127	17	31	ANLFSASPPPLSNRR					Mascot
1880.8369	1880.7668	-0.0701	-37	53	69	HSQQPSQEEPTDSGPTR					Mascot
2300.1414	2299.8948	-0.2466	-107	417	435	DLVPCVLLSFDDEQILMHR			Carbamidomethyl (C)[5]		Mascot
2428.2363	2427.8953	-0.341	-140	417	437	DLVPCVLLSFDDEQILMHRGK					Mascot
2779.5005	2779.5762	0.0757	27	151	175	EPAFLPFAPPTMPRPWTGKAPLLTK			Oxidation (M)[12]		Mascot
2807.5161	2807.9333	0.4172	149	227	251	EEVLGEPLTPSEVRALVKPHISHNR					Mascot

Rank	Protein Name	Accession No	Protein MW	Protein PI	Pep. Count	Protein Score	Protein Score C. I. %	Total Ion Score	Total Ion C. I. %
8	Ribulose bisphosphate carboxylase small chain, chloroplastic OS=Oryza sativa subsp. japonica GN=RBC	RBS1_ORYS	19633.9	9.04	6	30	0		

Peptide Information

Calc. Mass	Obsrv. Mass	± da	± ppm	Start Seq.	End Seq.	Sequence	Ion Score	C. I. %	Modification	Rank	Result Type
1045.5571	1045.447	-0.1101	-105	21	30	STAGMPVARR					Mascot
1165.571	1165.6062	0.0352	30	85	93	WVPCLEFSK			Carbamidomethyl (C)[4]		Mascot
1450.6458	1450.5415	-0.1043	-72	100	111	ENHRSPGYYDGR					Mascot
1994.0085	1993.7228	-0.2857	-143	1	20	MAPSVMASSATTVAPFQGLK					Mascot
2239.0984	2238.8521	-0.2463	-110	118	137	LPMFGCTDATQVLKELEEAK			Oxidation (M)[3]		Mascot
2419.1648	2418.8284	-0.3364	-139	112	131	YWTMWKLPMFGCTDATQVLK					Mascot

Rank	Protein Name	Accession No	Protein MW	Protein PI	Pep. Count	Protein Score	Protein Score C. I. %	Total Ion Score	Total Ion C. I. %
9	Putative defensin-like protein 110 OS=Arabidops thaliana GN=At1g15757 PE=3 SV=1	DF110_ARAT	8990.4	8.09	5	30	0		

Peptide Information

Calc. Mass	Obsrv. Mass	± da	± ppm	Start Seq.	End Seq.	Sequence	Ion Score	C. I. %	Modification	Rank	Result Type
856.3803	856.4225	0.0422	49	58	65	GCIAFCSR					Mascot
1273.6093	1273.5236	-0.0857	-67	46	57	TSPCLPEVGGEK			Carbamidomethyl (C)[4]		Mascot
2110.9717	2110.6863	-0.2854	-135	46	65	TSPCLPEVGGEKGCIAFCSR			Carbamidomethyl (C)[4]		Mascot
2110.9717	2110.6863	-0.2854	-135	46	65	TSPCLPEVGGEKGCIAFCSR			Carbamidomethyl (C)[4]		Mascot
2138.0071	2137.8003	-0.2068	-97	26	45	STSDIVSGSGIKEDEHVCFK					Mascot
2172.9255	2172.8254	-0.1001	-46	66	83	MKFTTGLCLGSVVCCCYT			Carbamidomethyl (C)[8,14,15,16], Oxidation (M)[1]		Mascot
2225.0146	2224.8384	-0.1762	-79	46	65	TSPCLPEVGGEKGCIAFCSR			Carbamidomethyl (C)[4,14,18]		Mascot

Rank	Protein Name	Accession No	Protein MW	Protein PI	Pep. Count	Protein Score	Protein Score C. I. %	Total Ion Score	Total Ion C. I. %
10	Ribulose bisphosphate carboxylase large chain (Fragment) OS=Liriope muscari GN=rbcL PE=3 SV=1	RBL_LIRMU	49636.9	6.46	8	29	0		

Peptide Information

Calc. Mass	Obsrv. Mass	± da	± ppm	Start Seq.	End Seq.	Sequence	Ion Score	C. I. %	Modification	Rank	Result Type
1021.5312	1021.4146	-0.1166	-114	26	34	DTDILAAFR					Mascot
1275.7307	1275.5737	-0.157	-123	333	343	EITLGFVDLLR					Mascot
1343.6525	1343.5164	-0.1361	-101	177	187	NYGRAVYECLR					Mascot
1465.7216	1465.5837	-0.1379	-94	313	327	MSGGDHIHAGTVVGK					Mascot
1465.7546	1465.5837	-0.1709	-117	140	152	TFQGPPHGIQVER					Mascot
2169.9868	2169.7346	-0.2522	-116	188	206	GGLDFTKDDENVNSQPFMR					Mascot
2740.2375	2739.9788	-0.2587	-94	221	245	AQAETGEIKGHYLNATAGTCEEMMK			Carbamidomethyl (C)[20]		Mascot
2953.397	2953.2537	-0.1433	-49	252	278	ELGAPIVMHDYLTGGFTANTSLAHYCR			Oxidation (M)[8]		Mascot

Gel Idx/Pos	63/C12	Instr./Gel Origin	BA2060/131017A	Process Sta	Analysis Succeeded
Plate [#] Name	[1] 31496	Instrument Sample Name		Spectra	11

Rank	Protein Name	Accession No	Protein MW	Protein PI	Pep. Count	Protein Score	Protein Score C. I. %	Total Ion Score	Total Ion C. I. %
1	Ribulose bisphosphate carboxylase large chain OS=Liquidambar styraciflua GN=rbcL PE=3 SV=1	RBL_LIQST	52625.5	6.04	15	107	100	26	59.941

Peptide Information

Calc. Mass	Obsrv. Mass	± da	± ppm	Start Seq.	End Seq.	Sequence	Ion Score	C. I. %	Modification	Rank	Result Type
910.4451	910.3464	-0.0987	-108	188	194	AVYECLR			Carbamidomethyl (C)[5]		Mascot
1021.5312	1021.416	-0.1152	-113	33	41	DTDILAAFR					Mascot
1116.583	1116.4231	-0.1599	-143	422	431	VALEACVQAR			Carbamidomethyl (C)[6]		Mascot
1170.5585	1170.5072	-0.0513	-44	304	312	QKNHGMHFR			Oxidation (M)[6]		Mascot
1187.6643	1187.5364	-0.1279	-108	286	295	DNGLLLHIHR					Mascot
1245.6433	1245.4889	-0.1544	-124	440	450	EGNEIIREASK					Mascot
1275.7307	1275.5868	-0.1439	-113	340	350	EITLGFVDLLR					Mascot
1465.7216	1465.5914	-0.1302	-89	320	334	MSGGDHIHAGTVVGK					Mascot
1465.7546	1465.5914	-0.1632	-111	147	159	TFQGPPHGIQVER	27	72.809			Mascot
1472.6978	1472.5358	-0.162	-110	464	475	EIKFEFEAMDTL					Mascot
1502.8512	1502.6674	-0.1838	-122	165	177	YGRPLLGCTIKPK			Carbamidomethyl (C)[8]		Mascot
1546.7358	1546.5511	-0.1847	-119	451	463	WSPELAAACEVWK			Carbamidomethyl (C)[9]		Mascot
2169.9868	2169.7346	-0.2522	-116	195	213	GGLDFTKDDENVNSQPFMR					Mascot
2185.9819	2185.74	-0.2419	-111	195	213	GGLDFTKDDENVNSQPFMR			Oxidation (M)[18]		Mascot
3022.4548	3022.1138	-0.341	-113	259	285	ELGVPIVMHDYLTGGFTANTSLAHYCR			Carbamidomethyl (C)[26]		Mascot
3854.8718	3854.3596	-0.5122	-133	42	79	VTPQPGVPPEEAGAAVAAESSTGTWTTVWTDGLTSLDR					Mascot

Rank	Protein Name	Accession No	Protein MW	Protein PI	Pep. Count	Protein Score	Protein Score C. I. %	Total Ion Score	Total Ion C. I. %
2	Ribulose bisphosphate carboxylase large chain (Fragment) OS=Manilkara zapota GN=rbcL PE=3 SV=2	RBL_MANZA	51550	6.04	14	105	100	27	72.809

Peptide Information

Calc. Mass	Obsrv. Mass	± da	± ppm	Start Seq.	End Seq.	Sequence	Ion Score	C. I. %	Modification	Rank	Result Type
910.4451	910.3464	-0.0987	-108	178	184	AVYECLR			Carbamidomethyl (C)[5]		Mascot
1021.5312	1021.416	-0.1152	-113	23	31	DTDILAAFR					Mascot
1116.583	1116.4231	-0.1599	-143	412	421	VALEACVQAR			Carbamidomethyl (C)[6]		Mascot
1170.5585	1170.5072	-0.0513	-44	294	302	QKNHGMHFR			Oxidation (M)[6]		Mascot
1187.6643	1187.5364	-0.1279	-108	276	285	DNGLLLHIHR					Mascot
1245.6433	1245.4889	-0.1544	-124	430	440	EGNEIIREASK					Mascot
1275.7307	1275.5868	-0.1439	-113	330	340	EITLGFVDLLR					Mascot
1465.7546	1465.5914	-0.1632	-111	137	149	TFQGPPHGIQVER	27	72.809			Mascot
1465.7546	1465.5914	-0.1632	-111	137	149	TFQGPPHGIQVER					Mascot
1472.6978	1472.5358	-0.162	-110	454	465	EIKFEFEAMDTL					Mascot
1502.8512	1502.6674	-0.1838	-122	155	167	YGRPLLGCTIKPK			Carbamidomethyl (C)[8]		Mascot
1546.7358	1546.5511	-0.1847	-119	441	453	WSPELAAACEVWK			Carbamidomethyl (C)[9]		Mascot
2169.9868	2169.7346	-0.2522	-116	185	203	GGLDFTKDDENVNSQPFMR					Mascot
2185.9819	2185.74	-0.2419	-111	185	203	GGLDFTKDDENVNSQPFMR			Oxidation (M)[18]		Mascot
3022.4548	3022.1138	-0.341	-113	249	275	ELGVPIVMHDYLTGGFTANTSLAHYCR			Carbamidomethyl (C)[26]		Mascot
3854.8718	3854.3596	-0.5122	-133	32	69	VTPQPGVPPEEAGAAVAAESSTGTWTTVWTDGLTSLDR					Mascot

Rank	Protein Name	Accession No	Protein MW	Protein PI	Pep. Count	Protein Score	Protein Score C. I. %	Total Ion Score	Total Ion C. I. %
3	Ribulose bisphosphate carboxylase large chain OS=Cucumis sativus GN=rbcL PE=1 SV=5	RBL_CUCSA	52608.4	6	15	104	100	26	59.941

Peptide Information

Calc. Mass	Obsrv. Mass	± da	± ppm	Start Seq.	End Seq.	Sequence	Ion Score	C. I. %	Modification	Rank	Result Type
910.4451	910.3464	-0.0987	-108	188	194	AVYECLR			Carbamidomethyl (C)[5]		Mascot
1021.5312	1021.416	-0.1152	-113	33	41	DTDILAAFR					Mascot
1116.583	1116.4231	-0.1599	-143	422	431	VALEACVQAR			Carbamidomethyl (C)[6]		Mascot
1170.5585	1170.5072	-0.0513	-44	304	312	QKNHGMHFR			Oxidation (M)[6]		Mascot
1187.6643	1187.5364	-0.1279	-108	286	295	DNGLLLHIHR					Mascot
1245.6337	1245.4889	-0.1448	-116	218	227	FLFCAEAIFK			Carbamidomethyl (C)[4]		Mascot
1275.7307	1275.5868	-0.1439	-113	340	350	EITLGFVDLLR					Mascot
1465.7216	1465.5914	-0.1302	-89	320	334	MSGGDHIHAGTVVGK					Mascot
1465.7546	1465.5914	-0.1632	-111	147	159	TFQGPPHGIQVER	27	72.809			Mascot
1472.6978	1472.5358	-0.162	-110	464	475	EIKFEFEAMDTL					Mascot
1502.8512	1502.6674	-0.1838	-122	165	177	YGRPLLGCTIKPK			Carbamidomethyl (C)[8]		Mascot Mascot
1546.7358	1546.5511	-0.1847	-119	451	463	WSPELAAACEVWK			Carbamidomethyl (C)[9]		Mascot
2169.9868	2169.7346	-0.2522	-116	195	213	GGLDFTKDDENVNSQPFMR					Mascot
2185.9819	2185.74	-0.2419	-111	195	213	GGLDFTKDDENVNSQPFMR			Oxidation (M)[18]		Mascot
2953.397	2953.3186	-0.0784	-27	259	285	ELGAPIVMHDYLTGGFTANTSLAHYCR			Oxidation (M)[8]		Mascot
3854.8718	3854.3596	-0.5122	-133	42	79	VTPQPGVPPEEAGAAVAAESSTGTWTTVWTDGLTSLDR					Mascot

Rank	Protein Name	Accession No	Protein MW	Protein PI	Pep. Count	Protein Score	Protein Score C. I. %	Total Ion Score	Total Ion C. I. %
4	Ribulose bisphosphate carboxylase large chain (Fragment) OS=Geum quellyon GN=rbcL PE=3 SV=1	RBL_GEUQU	50910.8	6.23	14	103	100	26	59.941

Peptide Information

Calc. Mass	Obsrv. Mass	± da	± ppm	Start Seq.	End Seq.	Sequence	Ion Score	C. I. %	Modification	Rank	Result Type
910.4451	910.3464	-0.0987	-108	178	184	AVYECLR			Carbamidomethyl (C)[5]		Mascot
1021.5312	1021.416	-0.1152	-113	23	31	DTDILAAFR					Mascot
1116.583	1116.4231	-0.1599	-143	412	421	VALEACVQAR			Carbamidomethyl (C)[6]		Mascot
1170.5585	1170.5072	-0.0513	-44	294	302	QKNHGMHFR			Oxidation (M)[6]		Mascot
1187.6643	1187.5364	-0.1279	-108	276	285	DNGLLLHIHR					Mascot
1245.6337	1245.4889	-0.1448	-116	208	217	FLFCAEALFK			Carbamidomethyl (C)[4]		Mascot
1275.7307	1275.5868	-0.1439	-113	330	340	EITLGFVDLLR					Mascot
1465.7216	1465.5914	-0.1302	-89	310	324	MSGGDHIHAGTVVGK					Mascot
1465.7546	1465.5914	-0.1632	-111	137	149	TFQGPPHGIQVER	27	72.809			Mascot
1502.8512	1502.6674	-0.1838	-122	155	167	YGRPLLGCTIKPK			Carbamidomethyl (C)[8]		Mascot
1546.7358	1546.5511	-0.1847	-119	441	453	WSPELAAACEVWK			Carbamidomethyl (C)[9]		Mascot
2169.9868	2169.7346	-0.2522	-116	185	203	GGLDFTKDDENVNSQPFMR					Mascot
2185.9819	2185.74	-0.2419	-111	185	203	GGLDFTKDDENVNSQPFMR			Oxidation (M)[18]		Mascot
3022.4548	3022.1138	-0.341	-113	249	275	ELGVPIVMHDYLTGGFTANTSLAHYCR			Carbamidomethyl (C)[26]		Mascot
3854.8718	3854.3596	-0.5122	-133	32	69	VTPQPGVPPEEAGAAVAAESSTGTWTTVWTDGLTSLDR					Mascot

Rank	Protein Name	Accession No	Protein MW	Protein PI	Pep. Count	Protein Score	Protein Score C. I. %	Total Ion Score	Total Ion C. I. %
5	Ribulose bisphosphate carboxylase large chain (Fragment) OS=Prunus domestica GN=rbcL PE=3 SV=1	RBL_PRUDO	50914.6	6.23	14	102	100	26	59.941

Peptide Information

Calc. Mass	Obsrv. Mass	± da	± ppm	Start Seq.	End Seq.	Sequence	Ion Score	C. I. %	Modification	Rank	Result Type
910.4451	910.3464	-0.0987	-108	178	184	AVYECLR			Carbamidomethyl (C)[5]		Mascot
1021.5312	1021.416	-0.1152	-113	23	31	DTDILAAFR					Mascot
1116.583	1116.4231	-0.1599	-143	412	421	VALEACVQAR			Carbamidomethyl (C)[6]		Mascot
1170.5585	1170.5072	-0.0513	-44	294	302	QKNHGMHFR			Oxidation (M)[6]		Mascot
1187.6643	1187.5364	-0.1279	-108	276	285	DNGLLLHIHR					Mascot
1245.6433	1245.4889	-0.1544	-124	430	440	EGNEIIREASK					Mascot
1275.7307	1275.5868	-0.1439	-113	330	340	EITLGFVDLLR					Mascot
1465.7216	1465.5914	-0.1302	-89	310	324	MSGGDHIHAGTVVGK					Mascot
1465.7546	1465.5914	-0.1632	-111	137	149	TFQGPPHGIQVER	27	72.809			Mascot
1502.8512	1502.6674	-0.1838	-122	155	167	YGRPLLGCTIKPK			Carbamidomethyl (C)[8]		Mascot
1546.7358	1546.5511	-0.1847	-119	441	453	WSPELAAACEVWK			Carbamidomethyl (C)[9]		Mascot
2169.9868	2169.7346	-0.2522	-116	185	203	GGLDFTKDDENVNSQPFMR					Mascot
2185.9819	2185.74	-0.2419	-111	185	203	GGLDFTKDDENVNSQPFMR			Oxidation (M)[18]		Mascot
3022.4548	3022.1138	-0.341	-113	249	275	ELGVPIVMHDYLTGGFTANTSLAHYCR			Carbamidomethyl (C)[26]		Mascot
3854.8718	3854.3596	-0.5122	-133	32	69	VTPQPGVPPEEAGAAVAAESSTGTWTTVWTDGLTSLDR					Mascot

Rank	Protein Name	Accession No	Protein MW	Protein PI	Pep. Count	Protein Score	Protein Score C. I. %	Total Ion Score	Total Ion C. I. %
6	Ribulose bisphosphate carboxylase large chain (Fragment) OS=Quisqualis indica GN=rbcL PE=3 SV=1	RBL_QUIIN	51658	6.13	14	102	100	26	59.941

Peptide Information

Calc. Mass	Obsrv. Mass	± da	± ppm	Start Seq.	End Seq.	Sequence	Ion Score	C. I. %	Modification	Rank	Result Type
910.4451	910.3464	-0.0987	-108	178	184	AVYECLR			Carbamidomethyl (C)[5]		Mascot
1021.5312	1021.416	-0.1152	-113	23	31	DTDILAAFR					Mascot
1116.583	1116.4231	-0.1599	-143	412	421	VALEACVQAR			Carbamidomethyl (C)[6]		Mascot
1170.5585	1170.5072	-0.0513	-44	294	302	QKNHGMHFR			Oxidation (M)[6]		Mascot
1245.6433	1245.4889	-0.1544	-124	430	440	EGNEIIREASK					Mascot
1275.7307	1275.5868	-0.1439	-113	330	340	EITLGFVDLLR					Mascot
1284.7018	1284.5334	-0.1684	-131	426	436	NLAREGNEIIR					Mascot
1284.7018	1284.5334	-0.1684	-131	426	436	NLAREGNEIIR					Mascot
1465.7216	1465.5914	-0.1302	-89	310	324	MSGGDHIHAGTVVGK					Mascot
1465.7546	1465.5914	-0.1632	-111	137	149	TFQGPPHGIQVER	27	72.809			Mascot
1472.6978	1472.5358	-0.162	-110	454	465	EIKFEFEAMDTL					Mascot
1502.8512	1502.6674	-0.1838	-122	155	167	YGRPLLGCTIKPK			Carbamidomethyl (C)[8]		Mascot
1546.7358	1546.5511	-0.1847	-119	441	453	WSPELAAACEVWK			Carbamidomethyl (C)[9]		Mascot
2169.9868	2169.7346	-0.2522	-116	185	203	GGLDFTKDDENVNSQPFMR					Mascot
2185.9819	2185.74	-0.2419	-111	185	203	GGLDFTKDDENVNSQPFMR			Oxidation (M)[18]		Mascot
3854.8718	3854.3596	-0.5122	-133	32	69	VTPQPGVPPEEAGAAVAAESSTGTWTTVWTDGLTSLDR					Mascot

Rank	Protein Name	Accession No	Protein MW	Protein PI	Pep. Count	Protein Score	Protein Score C. I. %	Total Ion Score	Total Ion C. I. %
7	Ribulose bisphosphate carboxylase large chain (Fragment) OS=Coriaria myrtifolia GN=rbcL PE=3 SV=1	RBL_CORMY	51666.1	6	14	102	100	26	59.941

Peptide Information

Calc. Mass	Obsrv. Mass	± da	± ppm	Start Seq.	End Seq.	Sequence	Ion Score	C. I. %	Modification	Rank	Result Type
910.4451	910.3464	-0.0987	-108	179	185	AVYECLR			Carbamidomethyl (C)[5]		Mascot
1021.5312	1021.416	-0.1152	-113	24	32	DTDILAAFR					Mascot
1116.583	1116.4231	-0.1599	-143	413	422	VALEACVQAR			Carbamidomethyl (C)[6]		Mascot
1187.6643	1187.5364	-0.1279	-108	277	286	DNGLLLHIHR					Mascot
1245.6433	1245.4889	-0.1544	-124	431	441	EGNEIIREASK					Mascot
1275.7307	1275.5868	-0.1439	-113	331	341	EITLGFVDLLR					Mascot
1465.7216	1465.5914	-0.1302	-89	311	325	MSGGDHIHAGTVVGK					Mascot
1465.7546	1465.5914	-0.1632	-111	138	150	TFQGPPHGIQVER	27	72.809			Mascot
1472.6978	1472.5358	-0.162	-110	455	466	EIKFEFEAMDTL					Mascot
1502.8512	1502.6674	-0.1838	-122	156	168	YGRPLLGCTIKPK			Carbamidomethyl (C)[8]		Mascot
1546.7358	1546.5511	-0.1847	-119	442	454	WSPELAAACEVWK			Carbamidomethyl (C)[9]		Mascot
2169.9868	2169.7346	-0.2522	-116	186	204	GGLDFTKDDENVNSQPFMR					Mascot
2185.9819	2185.74	-0.2419	-111	186	204	GGLDFTKDDENVNSQPFMR			Oxidation (M)[18]		Mascot
3022.4548	3022.1138	-0.341	-113	250	276	ELGVPIVMHDYLTGGFTANTSLAHYCR			Carbamidomethyl (C)[26]		Mascot
3854.8718	3854.3596	-0.5122	-133	33	70	VTPQPGVPPEEAGAAVAAESSTGTWTTVWTDGLTSLDR					Mascot

Rank	Protein Name	Accession No	Protein MW	Protein PI	Pep. Count	Protein Score	Protein Score C. I. %	Total Ion Score	Total Ion C. I. %
8	Ribulose bisphosphate carboxylase large chain (Fragment) OS=Hamamelis mollis GN=rbcL PE=3 SV=1	RBL_HAMMO	51542.9	6.05	14	101	100	26	59.941

Peptide Information

Calc. Mass	Obsrv. Mass	± da	± ppm	Start Seq.	End Seq.	Sequence	Ion Score	C. I. %	Modification	Rank	Result Type
910.4451	910.3464	-0.0987	-108	178	184	AVYECLR			Carbamidomethyl (C)[5]		Mascot
1021.5312	1021.416	-0.1152	-113	23	31	DTDILAAFR					Mascot
1116.583	1116.4231	-0.1599	-143	412	421	VALEACVQAR			Carbamidomethyl (C)[6]		Mascot
1170.5585	1170.5072	-0.0513	-44	294	302	QKNHGMHFR			Oxidation (M)[6]		Mascot
1187.6643	1187.5364	-0.1279	-108	276	285	DNGLLLHIHR					Mascot
1245.6433	1245.4889	-0.1544	-124	430	440	EGNEIIREASK					Mascot
1275.7307	1275.5868	-0.1439	-113	330	340	EITLGFVDLLR					Mascot
1465.7216	1465.5914	-0.1302	-89	310	324	MSGGDHIHAGTVVGK					Mascot
1465.7546	1465.5914	-0.1632	-111	137	149	TFQGPPHGIQVER	27	72.809			Mascot
1472.6978	1472.5358	-0.162	-110	454	465	EIKFEFEAMDTL					Mascot
1502.8512	1502.6674	-0.1838	-122	155	167	YGRPLLGCTIKPK			Carbamidomethyl (C)[8]		Mascot
1546.7358	1546.5511	-0.1847	-119	441	453	WSPELAAACEVWK			Carbamidomethyl (C)[9]		Mascot
2169.9868	2169.7346	-0.2522	-116	185	203	GGLDFTKDDENVNSQPFMR					Mascot
2185.9819	2185.74	-0.2419	-111	185	203	GGLDFTKDDENVNSQPFMR			Oxidation (M)[18]		Mascot
3854.8718	3854.3596	-0.5122	-133	32	69	VTPQPGVPPEEAGAAVAAESSTGTWTTVWTDGLTSLDR					Mascot

Rank	Protein Name	Accession No	Protein MW	Protein PI	Pep. Count	Protein Score	Protein Score C. I. %	Total Ion Score	Total Ion C. I. %
9	Ribulose bisphosphate carboxylase large chain (Fragment) OS=Saururus cernuus GN=rbcL PE=3 SV=1	RBL_SAUCE	51663	6	14	101	100	26	59.941

Peptide Information

Calc. Mass	Obsrv. Mass	± da	± ppm	Start Seq.	End Seq.	Sequence	Ion Score	C. I. %	Modification	Rank	Result Type
910.4451	910.3464	-0.0987	-108	179	185	AVYECLR			Carbamidomethyl (C)[5]		Mascot
1021.5312	1021.416	-0.1152	-113	24	32	DTDILAAFR					Mascot
1116.583	1116.4231	-0.1599	-143	413	422	VALEACVQAR			Carbamidomethyl (C)[6]		Mascot
1170.5585	1170.5072	-0.0513	-44	295	303	QKNHGMHFR			Oxidation (M)[6]		Mascot
1187.6643	1187.5364	-0.1279	-108	277	286	DNGLLLHIHR					Mascot
1275.7307	1275.5868	-0.1439	-113	331	341	EITLGFVDLLR					Mascot
1465.7216	1465.5914	-0.1302	-89	311	325	MSGGDHIHAGTVVGK					Mascot
1465.7546	1465.5914	-0.1632	-111	138	150	TFQGPPHGIQVER	27	72.809			Mascot
1472.6978	1472.5358	-0.162	-110	455	466	EIKFEFEAMDTL					Mascot
1502.8512	1502.6674	-0.1838	-122	156	168	YGRPLLGCTIKPK			Carbamidomethyl (C)[8]		Mascot
1546.7358	1546.5511	-0.1847	-119	442	454	WSPELAAACEVWK			Carbamidomethyl (C)[9]		Mascot
2169.9868	2169.7346	-0.2522	-116	186	204	GGLDFTKDDENVNSQPFMR					Mascot
2185.9819	2185.74	-0.2419	-111	186	204	GGLDFTKDDENVNSQPFMR			Oxidation (M)[18]		Mascot
3022.4548	3022.1138	-0.341	-113	250	276	ELGVPIVMHDYLTGGFTANTSLAHYCR			Carbamidomethyl (C)[26]		Mascot
3854.8718	3854.3596	-0.5122	-133	33	70	VTPQPGVPPEEAGAAVAAESSTGTWTTVWTDGLTSLDR					Mascot

Rank	Protein Name	Accession No	Protein MW	Protein PI	Pep. Count	Protein Score	Protein Score C. I. %	Total Ion Score	Total Ion C. I. %
10	Ribulose bisphosphate carboxylase large chain OS=Glycine max GN=rbcL PE=1 SV=3	RBL_SOYBN	52576.4	6	14	98	100	27	72.809

Peptide Information

Calc. Mass	Obsrv. Mass	± da	± ppm	Start Seq.	End Seq.	Sequence	Ion Score	C. I. %	Modification	Rank	Result Type
910.4451	910.3464	-0.0987	-108	188	194	AVYECLR			Carbamidomethyl (C)[5]		Mascot
1021.5312	1021.416	-0.1152	-113	33	41	DTDILAAFR					Mascot
1116.583	1116.4231	-0.1599	-143	422	431	VALEACVQAR			Carbamidomethyl (C)[6]		Mascot
1170.5585	1170.5072	-0.0513	-44	304	312	QKNHGMHFR			Oxidation (M)[6]		Mascot
1187.6643	1187.5364	-0.1279	-108	286	295	DNGLLLHIHR					Mascot
1245.6337	1245.4889	-0.1448	-116	218	227	FLFCAEAIFK			Carbamidomethyl (C)[4]		Mascot
1275.7307	1275.5868	-0.1439	-113	340	350	EITLGFVDLLR					Mascot
1465.7546	1465.5914	-0.1632	-111	147	159	TFQGPPHGIQVER	27	72.809			Mascot
1465.7546	1465.5914	-0.1632	-111	147	159	TFQGPPHGIQVER					Mascot
1472.6978	1472.5358	-0.162	-110	464	475	EIKFEFEAMDTL					Mascot
1502.8512	1502.6674	-0.1838	-122	165	177	YGRPLLGCTIKPK			Carbamidomethyl (C)[8]		Mascot
1546.7358	1546.5511	-0.1847	-119	451	463	WSPELAAACEVWK			Carbamidomethyl (C)[9]		Mascot
2169.9868	2169.7346	-0.2522	-116	195	213	GGLDFTKDDENVNSQPFMR					Mascot
2185.9819	2185.74	-0.2419	-111	195	213	GGLDFTKDDENVNSQPFMR			Oxidation (M)[18]		Mascot
3022.4548	3022.1138	-0.341	-113	259	285	ELGVPIVMHDYLTGGFTANTSLAHYCR			Carbamidomethyl (C)[26]		Mascot
3854.8718	3854.3596	-0.5122	-133	42	79	VTPQPGVPPEEAGAAVAAESSTGTWTTVWTDGLTSLDR					Mascot

Gel Idx/Pos	64/C13	Instr./Gel Origin	BA2060/131017A	Process Sta	Analysis Succeeded
Plate [#] Name	[1] 31496	Instrument Sample Name		Spectra	11

Rank	Protein Name	Accession No	Protein MW	Protein PI	Pep. Count	Protein Score	Protein Score C. I. %	Total Ion Score	Total Ion C. I. %
1	Sedoheptulose-1,7-bisphosphatase, chloroplasti OS=Spinacia oleracea PE=2 SV=1	S17P_SPIOL	42054.4	5.87	6	118	100	100	100

Peptide Information

Calc. Mass	Obsrv. Mass	± da	± ppm	Start Seq.	End Seq.	Sequence	Ion Score	C. I. %	Modification	Rank	Result Type
908.5451	908.4473	-0.0978	-108	216	223	TTYVLALK					Mascot
1170.5138	1170.5167	0.0029	2	89	98	LMMCMGEALR			Oxidation (M)[2]		Mascot
1187.6055	1187.5389	-0.0666	-56	367	376	FEKTLYGSSR					Mascot
1207.421	1207.4952	0.0742	61	1	10	METSMACCSR			Carbamidomethyl (C)[7], Oxidation (M)[1,5]		Mascot
1588.9348	1588.7539	-0.1809	-114	317	330	LLFEVAPLGFLIEK					Mascot
1588.9348	1588.7539	-0.1809	-114	317	330	LLFEVAPLGFLIEK	70	99.999			Mascot
1633.8618	1633.6758	-0.186	-114	284	298	YTGGMVPDVNQIIVK	29	82.516			Mascot
1633.8618	1633.6758	-0.186	-114	284	298	YTGGMVPDVNQIIVK					Mascot
1649.8567	1649.6697	-0.187	-113	284	298	YTGGMVPDVNQIIVK			Oxidation (M)[5]		Mascot

Rank	Protein Name	Accession No	Protein MW	Protein PI	Pep. Count	Protein Score	Protein Score C. I. %	Total Ion Score	Total Ion C. I. %
2	Ribulose bisphosphate carboxylase large chain OS=Liquidambar styraciflua GN=rbcL PE=3 SV=1	RBL_LIQST	52625.5	6.04	17	110	100	6	0

Peptide Information

Calc. Mass	Obsrv. Mass	± da	± ppm	Start Seq.	End Seq.	Sequence	Ion Score	C. I. %	Modification	Rank	Result Type
898.41	898.31	-0.1	-111	306	312	NHGMHFR					Mascot
910.4451	910.349	-0.0961	-106	188	194	AVYECLR			Carbamidomethyl (C)[5]		Mascot
914.4049	914.3123	-0.0926	-101	306	312	NHGMHFR			Oxidation (M)[4]		Mascot
1021.5312	1021.4194	-0.1118	-109	33	41	DTDILAAFR					Mascot
1116.583	1116.4398	-0.1432	-128	422	431	VALEACVQAR			Carbamidomethyl (C)[6]		Mascot
1170.5585	1170.5167	-0.0418	-36	304	312	QKNHGMHFR			Oxidation (M)[6]		Mascot
1187.6643	1187.5389	-0.1254	-106	286	295	DNGLLLHIHR					Mascot
1245.6433	1245.4985	-0.1448	-116	440	450	EGNEIIREASK					Mascot
1275.7307	1275.5905	-0.1402	-110	340	350	EITLGFVDLLR					Mascot
1407.6678	1407.4768	-0.191	-136	22	32	LTYYTPEYETK					Mascot
1465.7216	1465.592	-0.1296	-88	320	334	MSGGDHIHAGTVVGK					Mascot
1465.7546	1465.592	-0.1626	-111	147	159	TFQGPPHGIQVER	7	0			Mascot
1488.6926	1488.5184	-0.1742	-117	464	475	EIKFEFEAMDTL			Oxidation (M)[9]		Mascot
1502.8512	1502.6764	-0.1748	-116	165	177	YGRPLLGCTIKPK			Carbamidomethyl (C)[8]		Mascot
1546.7358	1546.5664	-0.1694	-110	451	463	WSPELAAACEVWK			Carbamidomethyl (C)[9]		Mascot
2169.9868	2169.7385	-0.2483	-114	195	213	GGLDFTKDDENVNSQPFMR					Mascot
2185.9819	2185.7322	-0.2497	-114	195	213	GGLDFTKDDENVNSQPFMR			Oxidation (M)[18]		Mascot
3022.4548	3022.1033	-0.3515	-116	259	285	ELGVPIVMHDYLTGGFTANTSLAHYCR			Carbamidomethyl (C)[26]		Mascot
3038.45	3038.0874	-0.3626	-119	259	285	ELGVPIVMHDYLTGGFTANTSLAHYCR			Carbamidomethyl (C)[26], Oxidation (M)[8]		Mascot
3854.8718	3854.4312	-0.4406	-114	42	79	VTPQPGVPPEEAGAAVAAESSTGTWTTVWTDGLTSLDR					Mascot

Rank	Protein Name	Accession No	Protein MW	Protein PI	Pep. Count	Protein Score	Protein Score C. I. %	Total Ion Score	Total Ion C. I. %
3	Sedoheptulose-1,7-bisphosphatase, chloroplasti OS=Triticum aestivum PE=2 SV=1	S17P_WHEA	42034.1	6.04	2	105	100	100	100

Peptide Information

Calc. Mass	Obsrv. Mass	± da	± ppm	Start Seq.	End Seq.	Sequence	Ion Score	C. I. %	Modification	Rank	Result Type
1588.9348	1588.7539	-0.1809	-114	323	336	LLFEVAPLGFLIEK					Mascot
1588.9348	1588.7539	-0.1809	-114	323	336	LLFEVAPLGFLIEK	70	99.999			Mascot
1633.8618	1633.6758	-0.186	-114	290	304	YTGGMVPDVNQIIVK	29	82.516			Mascot
1633.8618	1633.6758	-0.186	-114	290	304	YTGGMVPDVNQIIVK					Mascot
1649.8567	1649.6697	-0.187	-113	290	304	YTGGMVPDVNQIIVK			Oxidation (M)[5]		Mascot

Rank	Protein Name	Accession No	Protein MW	Protein PI	Pep. Count	Protein Score	Protein Score C. I. %	Total Ion Score	Total Ion C. I. %
4	Ribulose bisphosphate carboxylase large chain (Fragment) OS=Hamamelis mollis GN=rbcL PE=3 SV=1	RBL_HAMMO	51542.9	6.05	16	100	100	6	0

Peptide Information

Calc. Mass	Obsrv. Mass	± da	± ppm	Start Seq.	End Seq.	Sequence	Ion Score	C. I. %	Modification	Rank	Result Type
898.41	898.31	-0.1	-111	296	302	NHGMHFR					Mascot
910.4451	910.349	-0.0961	-106	178	184	AVYECLR			Carbamidomethyl (C)[5]		Mascot
914.4049	914.3123	-0.0926	-101	296	302	NHGMHFR			Oxidation (M)[4]		Mascot
1021.5312	1021.4194	-0.1118	-109	23	31	DTDILAAFR					Mascot
1116.583	1116.4398	-0.1432	-128	412	421	VALEACVQAR			Carbamidomethyl (C)[6]		Mascot
1170.5585	1170.5167	-0.0418	-36	294	302	QKNHGMHFR			Oxidation (M)[6]		Mascot
1187.6643	1187.5389	-0.1254	-106	276	285	DNGLLLHIHR					Mascot
1245.6433	1245.4985	-0.1448	-116	430	440	EGNEIIREASK					Mascot
1275.7307	1275.5905	-0.1402	-110	330	340	EITLGFVDLLR					Mascot
1407.6678	1407.4768	-0.191	-136	12	22	LTYYTPEYETK					Mascot
1465.7216	1465.592	-0.1296	-88	310	324	MSGGDHIHAGTVVGK					Mascot
1465.7546	1465.592	-0.1626	-111	137	149	TFQGPPHGIQVER	7	0			Mascot
1488.6926	1488.5184	-0.1742	-117	454	465	EIKFEFEAMDTL			Oxidation (M)[9]		Mascot
1502.8512	1502.6764	-0.1748	-116	155	167	YGRPLLGCTIKPK			Carbamidomethyl (C)[8]		Mascot
1546.7358	1546.5664	-0.1694	-110	441	453	WSPELAAACEVWK			Carbamidomethyl (C)[9]		Mascot
2169.9868	2169.7385	-0.2483	-114	185	203	GGLDFTKDDENVNSQPFMR					Mascot
2185.9819	2185.7322	-0.2497	-114	185	203	GGLDFTKDDENVNSQPFMR			Oxidation (M)[18]		Mascot
3854.8718	3854.4312	-0.4406	-114	32	69	VTPQPGVPPEEAGAAVAAESSTGTWTTVWTDGLTSLDR					Mascot

Rank	Protein Name	Accession No	Protein MW	Protein PI	Pep. Count	Protein Score	Protein Score C. I. %	Total Ion Score	Total Ion C. I. %
5	Ribulose bisphosphate carboxylase large chain (Fragment) OS=Manilkara zapota GN=rbcL PE=3 SV=2	RBL_MANZA	51550	6.04	15	96	100	7	0

Peptide Information

Calc. Mass	Obsrv. Mass	± da	± ppm	Start Seq.	End Seq.	Sequence	Ion Score	C. I. %	Modification	Rank	Result Type
898.41	898.31	-0.1	-111	296	302	NHGMHFR					Mascot
910.4451	910.349	-0.0961	-106	178	184	AVYECLR			Carbamidomethyl (C)[5]		Mascot
914.4049	914.3123	-0.0926	-101	296	302	NHGMHFR			Oxidation (M)[4]		Mascot
1021.5312	1021.4194	-0.1118	-109	23	31	DTDILAAFR					Mascot
1116.583	1116.4398	-0.1432	-128	412	421	VALEACVQAR			Carbamidomethyl (C)[6]		Mascot
1170.5585	1170.5167	-0.0418	-36	294	302	QKNHGMHFR			Oxidation (M)[6]		Mascot
1187.6643	1187.5389	-0.1254	-106	276	285	DNGLLLHIHR					Mascot
1245.6433	1245.4985	-0.1448	-116	430	440	EGNEIIREASK					Mascot
1275.7307	1275.5905	-0.1402	-110	330	340	EITLGFVDLLR					Mascot
1465.7546	1465.592	-0.1626	-111	137	149	TFQGPPHGIQVER					Mascot
1465.7546	1465.592	-0.1626	-111	137	149	TFQGPPHGIQVER	7	0			Mascot
1488.6926	1488.5184	-0.1742	-117	454	465	EIKFEFEAMDTL			Oxidation (M)[9]		Mascot
1502.8512	1502.6764	-0.1748	-116	155	167	YGRPLLGCTIKPK			Carbamidomethyl (C)[8]		Mascot
1546.7358	1546.5664	-0.1694	-110	441	453	WSPELAAACEVWK			Carbamidomethyl (C)[9]		Mascot
2169.9868	2169.7385	-0.2483	-114	185	203	GGLDFTKDDENVNSQPFMR					Mascot
2185.9819	2185.7322	-0.2497	-114	185	203	GGLDFTKDDENVNSQPFMR			Oxidation (M)[18]		Mascot
3022.4548	3022.1033	-0.3515	-116	249	275	ELGVPIVMHDYLTGGFTANTSLAHYCR			Carbamidomethyl (C)[26]		Mascot
3038.45	3038.0874	-0.3626	-119	249	275	ELGVPIVMHDYLTGGFTANTSLAHYCR			Carbamidomethyl (C)[26], Oxidation (M)[8]		Mascot
3854.8718	3854.4312	-0.4406	-114	32	69	VTPQPGVPPEEAGAAVAAESSTGTWTTVWTDGLTSLDR					Mascot

Rank	Protein Name	Accession No	Protein MW	Protein PI	Pep. Count	Protein Score	Protein Score C. I. %	Total Ion Score	Total Ion C. I. %
6	Ribulose bisphosphate carboxylase large chain OS=Cucumis sativus GN=rbcL PE=1 SV=5	RBL_CUCSA	52608.4	6	16	94	99.999	6	0

Peptide Information

Calc. Mass	Obsrv. Mass	± da	± ppm	Start Seq.	End Seq.	Sequence	Ion Score	C. I. %	Modification	Rank	Result Type
898.41	898.31	-0.1	-111	306	312	NHGMHFR					Mascot
910.4451	910.349	-0.0961	-106	188	194	AVYECLR			Carbamidomethyl (C)[5]		Mascot
914.4049	914.3123	-0.0926	-101	306	312	NHGMHFR			Oxidation (M)[4]		Mascot
1021.5312	1021.4194	-0.1118	-109	33	41	DTDILAAFR					Mascot
1116.583	1116.4398	-0.1432	-128	422	431	VALEACVQAR			Carbamidomethyl (C)[6]		Mascot
1170.5585	1170.5167	-0.0418	-36	304	312	QKNHGMHFR			Oxidation (M)[6]		Mascot
1187.6643	1187.5389	-0.1254	-106	286	295	DNGLLLHIHR					Mascot
1245.6337	1245.4985	-0.1352	-109	218	227	FLFCAEAIFK			Carbamidomethyl (C)[4]		Mascot
1275.7307	1275.5905	-0.1402	-110	340	350	EITLGFVDLLR					Mascot
1407.6678	1407.4768	-0.191	-136	22	32	LTYYTPEYETK					Mascot
1465.7216	1465.592	-0.1296	-88	320	334	MSGGDHIHAGTVVGK					Mascot
1465.7546	1465.592	-0.1626	-111	147	159	TFQGPPHGIQVER	7	0			Mascot
1488.6926	1488.5184	-0.1742	-117	464	475	EIKFEFEAMDTL			Oxidation (M)[9]		Mascot
1502.8512	1502.6764	-0.1748	-116	165	177	YGRPLLGCTIKPK			Carbamidomethyl (C)[8]		Mascot
1546.7358	1546.5664	-0.1694	-110	451	463	WSPELAAACEVWK			Carbamidomethyl (C)[9]		Mascot
2169.9868	2169.7385	-0.2483	-114	195	213	GGLDFTKDDENVNSQPFMR					Mascot
2185.9819	2185.7322	-0.2497	-114	195	213	GGLDFTKDDENVNSQPFMR			Oxidation (M)[18]		Mascot
3854.8718	3854.4312	-0.4406	-114	42	79	VTPQPGVPPEEAGAAVAAESSTGTWTTVWTDGLTSLDR					Mascot

Rank	Protein Name	Accession No	Protein MW	Protein PI	Pep. Count	Protein Score	Protein Score C. I. %	Total Ion Score	Total Ion C. I. %
7	Ribulose bisphosphate carboxylase large chain (Fragment) OS=Geum quellyon GN=rbcL PE=3 SV=1	RBL_GEUQU	50910.8	6.23	15	94	99.999	6	0

Peptide Information

Calc. Mass	Obsrv. Mass	± da	± ppm	Start Seq.	End Seq.	Sequence	Ion Score	C. I. %	Modification	Rank	Result Type
898.41	898.31	-0.1	-111	296	302	NHGMHFR					Mascot
910.4451	910.349	-0.0961	-106	178	184	AVYECLR			Carbamidomethyl (C)[5]		Mascot
914.4049	914.3123	-0.0926	-101	296	302	NHGMHFR			Oxidation (M)[4]		Mascot
1021.5312	1021.4194	-0.1118	-109	23	31	DTDILAAFR					Mascot
1116.583	1116.4398	-0.1432	-128	412	421	VALEACVQAR			Carbamidomethyl (C)[6]		Mascot
1170.5585	1170.5167	-0.0418	-36	294	302	QKNHGMHFR			Oxidation (M)[6]		Mascot
1187.6643	1187.5389	-0.1254	-106	276	285	DNGLLLHIHR					Mascot
1245.6337	1245.4985	-0.1352	-109	208	217	FLFCAEALFK			Carbamidomethyl (C)[4]		Mascot
1275.7307	1275.5905	-0.1402	-110	330	340	EITLGFVDLLR					Mascot
1465.7216	1465.592	-0.1296	-88	310	324	MSGGDHIHAGTVVGK					Mascot
1465.7546	1465.592	-0.1626	-111	137	149	TFQGPPHGIQVER	7	0			Mascot
1502.8512	1502.6764	-0.1748	-116	155	167	YGRPLLGCTIKPK			Carbamidomethyl (C)[8]		Mascot
1546.7358	1546.5664	-0.1694	-110	441	453	WSPELAAACEVWK			Carbamidomethyl (C)[9]		Mascot
2169.9868	2169.7385	-0.2483	-114	185	203	GGLDFTKDDENVNSQPFMR					Mascot
2185.9819	2185.7322	-0.2497	-114	185	203	GGLDFTKDDENVNSQPFMR			Oxidation (M)[18]		Mascot
3022.4548	3022.1033	-0.3515	-116	249	275	ELGVPIVMHDYLTGGFTANTSLAHYCR			Carbamidomethyl (C)[26]		Mascot
3038.45	3038.0874	-0.3626	-119	249	275	ELGVPIVMHDYLTGGFTANTSLAHYCR			Carbamidomethyl (C)[26], Oxidation (M)[8]		Mascot
3854.8718	3854.4312	-0.4406	-114	32	69	VTPQPGVPPEEAGAAVAAESSTGTWTTVWTDGLTSLDR					Mascot

Rank	Protein Name	Accession No	Protein MW	Protein PI	Pep. Count	Protein Score	Protein Score C. I. %	Total Ion Score	Total Ion C. I. %
8	Ribulose bisphosphate carboxylase large chain (Fragment) OS=Prunus domestica GN=rbcL PE=3 SV=1	RBL_PRUDO	50914.6	6.23	15	94	99.998	6	0

Peptide Information

Calc. Mass	Obsrv. Mass	± da	± ppm	Start Seq.	End Seq.	Sequence	Ion Score	C. I. %	Modification	Rank	Result Type
898.41	898.31	-0.1	-111	296	302	NHGMHFR					Mascot
910.4451	910.349	-0.0961	-106	178	184	AVYECLR			Carbamidomethyl (C)[5]		Mascot
914.4049	914.3123	-0.0926	-101	296	302	NHGMHFR			Oxidation (M)[4]		Mascot
1021.5312	1021.4194	-0.1118	-109	23	31	DTDILAAFR					Mascot
1116.583	1116.4398	-0.1432	-128	412	421	VALEACVQAR			Carbamidomethyl (C)[6]		Mascot
1170.5585	1170.5167	-0.0418	-36	294	302	QKNHGMHFR			Oxidation (M)[6]		Mascot
1187.6643	1187.5389	-0.1254	-106	276	285	DNGLLLHIHR					Mascot
1245.6433	1245.4985	-0.1448	-116	430	440	EGNEIIREASK					Mascot
1275.7307	1275.5905	-0.1402	-110	330	340	EITLGFVDLLR					Mascot
1465.7216	1465.592	-0.1296	-88	310	324	MSGGDHIHAGTVVGK					Mascot
1465.7546	1465.592	-0.1626	-111	137	149	TFQGPPHGIQVER	7	0			Mascot
1502.8512	1502.6764	-0.1748	-116	155	167	YGRPLLGCTIKPK			Carbamidomethyl (C)[8]		Mascot
1546.7358	1546.5664	-0.1694	-110	441	453	WSPELAAACEVWK			Carbamidomethyl (C)[9]		Mascot
2169.9868	2169.7385	-0.2483	-114	185	203	GGLDFTKDDENVNSQPFMR					Mascot
2185.9819	2185.7322	-0.2497	-114	185	203	GGLDFTKDDENVNSQPFMR			Oxidation (M)[18]		Mascot
3022.4548	3022.1033	-0.3515	-116	249	275	ELGVPIVMHDYLTGGFTANTSLAHYCR			Carbamidomethyl (C)[26]		Mascot
3038.45	3038.0874	-0.3626	-119	249	275	ELGVPIVMHDYLTGGFTANTSLAHYCR			Carbamidomethyl (C)[26], Oxidation (M)[8]		Mascot
3854.8718	3854.4312	-0.4406	-114	32	69	VTPQPGVPPEEAGAAVA					Mascot
	3854.4312										Mascot
						AESSTGTWTTVWTDGLTSLDR					

Rank	Protein Name	Accession No	Protein MW	Protein PI	Pep. Count	Protein Score	Protein Score C. I. %	Total Ion Score	Total Ion C. I. %
9	Ribulose bisphosphate carboxylase large chain (Fragment) OS=Sinapis alba GN=rbcL PE=3 SV=1	RBL_SINAL	52405.4	6.23	15	93	99.998	7	0

Peptide Information

Calc. Mass	Obsrv. Mass	± da	± ppm	Start Seq.	End Seq.	Sequence	Ion Score	C. I. %	Modification	Rank	Result Type
856.3546	856.4339	0.0793	93	467	473	FEFPAMD					Mascot
898.41	898.31	-0.1	-111	306	312	NHGMHFR					Mascot
910.4451	910.349	-0.0961	-106	188	194	AVYECLR			Carbamidomethyl (C)[5]		Mascot
914.4049	914.3123	-0.0926	-101	306	312	NHGMHFR			Oxidation (M)[4]		Mascot
1021.5312	1021.4194	-0.1118	-109	33	41	DTDILAAFR					Mascot
1116.583	1116.4398	-0.1432	-128	422	431	VALEACVQAR			Carbamidomethyl (C)[6]		Mascot
1170.5585	1170.5167	-0.0418	-36	304	312	QKNHGMHFR			Oxidation (M)[6]		Mascot
1187.6643	1187.5389	-0.1254	-106	286	295	DNGLLLHIHR					Mascot
1245.6433	1245.4985	-0.1448	-116	440	450	EGNEIIREASK					Mascot
1275.7307	1275.5905	-0.1402	-110	340	350	EITLGFVDLLR					Mascot
1465.7546	1465.592	-0.1626	-111	147	159	TFQGPPHGIQVER					Mascot
1465.7546	1465.592	-0.1626	-111	147	159	TFQGPPHGIQVER	7	0			Mascot
1502.8512	1502.6764	-0.1748	-116	165	177	YGRPLLGCTIKPK			Carbamidomethyl (C)[8]		Mascot
1546.7358	1546.5664	-0.1694	-110	451	463	WSPELAAACEVWK			Carbamidomethyl (C)[9]		Mascot
2169.9868	2169.7385	-0.2483	-114	195	213	GGLDFTKDDENVNSQPFMR					Mascot
2185.9819	2185.7322	-0.2497	-114	195	213	GGLDFTKDDENVNSQPFMR			Oxidation (M)[18]		Mascot
3022.4548	3022.1033	-0.3515	-116	259	285	ELGVPIVMHDYLTGGFTANTSLAHYCR			Carbamidomethyl (C)[26]		Mascot
3038.45	3038.0874	-0.3626	-119	259	285	ELGVPIVMHDYLTGGFTANTSLAHYCR			Carbamidomethyl (C)[26], Oxidation (M)[8]		Mascot
3854.8718	3854.4312	-0.4406	-114	42	79	VTPQPGVPPEEAGAAVAAESSTGTWTTVWTDGLTSLDR					Mascot

Rank	Protein Name	Accession No	Protein MW	Protein PI	Pep. Count	Protein Score	Protein Score C. I. %	Total Ion Score	Total Ion C. I. %
10	Ribulose bisphosphate carboxylase large chain (Fragment) OS=Saururus cernuus GN=rbcL PE=3 SV=1	RBL_SAUCE	51663	6	15	92	99.998	6	0

Peptide Information

Calc. Mass	Obsrv. Mass	± da	± ppm	Start Seq.	End Seq.	Sequence	Ion Score	C. I. %	Modification	Rank	Result Type
898.41	898.31	-0.1	-111	297	303	NHGMHFR					Mascot
910.4451	910.349	-0.0961	-106	179	185	AVYECLR			Carbamidomethyl (C)[5]		Mascot
914.4049	914.3123	-0.0926	-101	297	303	NHGMHFR			Oxidation (M)[4]		Mascot
1021.5312	1021.4194	-0.1118	-109	24	32	DTDILAAFR					Mascot
1116.583	1116.4398	-0.1432	-128	413	422	VALEACVQAR			Carbamidomethyl (C)[6]		Mascot
1170.5585	1170.5167	-0.0418	-36	295	303	QKNHGMHFR			Oxidation (M)[6]		Mascot
1187.6643	1187.5389	-0.1254	-106	277	286	DNGLLLHIHR					Mascot
1275.7307	1275.5905	-0.1402	-110	331	341	EITLGFVDLLR					Mascot
1465.7216	1465.592	-0.1296	-88	311	325	MSGGDHIHAGTVVGK					Mascot
1465.7546	1465.592	-0.1626	-111	138	150	TFQGPPHGIQVER	7	0			Mascot
1488.6926	1488.5184	-0.1742	-117	455	466	EIKFEFEAMDTL			Oxidation (M)[9]		Mascot
1502.8512	1502.6764	-0.1748	-116	156	168	YGRPLLGCTIKPK			Carbamidomethyl (C)[8]		Mascot
1546.7358	1546.5664	-0.1694	-110	442	454	WSPELAAACEVWK			Carbamidomethyl (C)[9]		Mascot
2169.9868	2169.7385	-0.2483	-114	186	204	GGLDFTKDDENVNSQPFMR					Mascot
2185.9819	2185.7322	-0.2497	-114	186	204	GGLDFTKDDENVNSQPFMR			Oxidation (M)[18]		Mascot
3022.4548	3022.1033	-0.3515	-116	250	276	ELGVPIVMHDYLTGGFTANTSLAHYCR			Carbamidomethyl (C)[26]		Mascot
3038.45	3038.0874	-0.3626	-119	250	276	ELGVPIVMHDYLTGGFTANTSLAHYCR			Carbamidomethyl (C)[26], Oxidation (M)[8]		Mascot
3854.8718	3854.4312	-0.4406	-114	33	70	VTPQPGVPPEEAGAAVAAESSTGTWTTVWTDGLTSLDR					Mascot

Gel Idx/Pos	65/C14	Instr./Gel Origin	BA2060/131017A	Process Sta	Analysis Succeeded
Plate [#] Name	[1] 31496	Instrument Sample Name		Spectra	11

Rank	Protein Name	Accession No	Protein MW	Protein PI	Pep. Count	Protein Score	Protein Score C. I. %	Total Ion Score	Total Ion C. I. %
1	Ribulose bisphosphate carboxylase/oxygenase activase, chloroplastic OS=Vigna radiata var. radiata G	RCA_VIGRR	47871.3	7.57	9	276	100	243	100

Peptide Information

Calc. Mass	Obsrv. Mass	± da	± ppm	Start Seq.	End Seq.	Sequence	Ion Score	C. I. %	Modification	Rank	Result Type
940.4675	940.3726	-0.0949	-101	305	311	FYWAPTR	16	0			Mascot
940.4675	940.3726	-0.0949	-101	305	311	FYWAPTR					Mascot
1023.5105	1023.4305	-0.08	-78	354	361	VYDDEVRK					Mascot
1115.5367	1115.4215	-0.1152	-103	380	389	EGPPTFDQPK					Mascot
1152.7139	1152.5945	-0.1194	-104	159	169	VPLILGIWGGK					Mascot
1228.603	1228.4749	-0.1281	-104	174	183	SFQCELVFAK			Carbamidomethyl (C)[4]		Mascot
1623.7617	1623.5898	-0.1719	-106	224	238	MCALFINDLDAGAGR			Carbamidomethyl (C)[2]		Mascot
1709.7977	1709.618	-0.1797	-105	77	91	GLAYDISDDQQDITR	56	99.966			Mascot
1709.7977	1709.618	-0.1797	-105	77	91	GLAYDISDDQQDITR					Mascot
1882.9697	1882.766	-0.2037	-108	335	351	LVDTFPGQSIDFFGALR	67	99.997			Mascot
	1882.766										Mascot
1882.9697	1882.766	-0.2037	-108	335	351	LVDTFPGQSIDFFGALR					Mascot
2089.1692	2088.9438	-0.2254	-108	280	298	VPIIVTGNDFSTLYAPLIR	104	100			Mascot
2089.1692	2088.9438	-0.2254	-108	280	298	VPIIVTGNDFSTLYAPLIR					Mascot

Rank	Protein Name	Accession No	Protein MW	Protein PI	Pep. Count	Protein Score	Protein Score C. I. %	Total Ion Score	Total Ion C. I. %
2	Ribulose bisphosphate carboxylase/oxygenase a B, chloroplastic OS=Hordeum vulgare GN=RCAB PE	RCAB_HORVI	47197.8	7.59	9	271	100	243	100

Peptide Information

Calc. Mass	Obsrv. Mass	± da	± ppm	Start Seq.	End Seq.	Sequence	Ion Score	C. I. %	Modification	Rank	Result Type
940.4675	940.3726	-0.0949	-101	290	296	FYWAPTR	16	0			Mascot
940.4675	940.3726	-0.0949	-101	290	296	FYWAPTR					Mascot
1023.5105	1023.4305	-0.08	-78	339	346	VYDDEVRK					Mascot
1115.5367	1115.4215	-0.1152	-103	365	374	EGPPTFDQPK					Mascot
1152.7139	1152.5945	-0.1194	-104	145	155	VPLILGIWGGK					Mascot
1214.6012	1214.5046	-0.0966	-80	309	319	TDNVPDEAVVR					Mascot
1228.603	1228.4749	-0.1281	-104	160	169	SFQCELVFAK			Carbamidomethyl (C)[4]		Mascot
1709.7977	1709.618	-0.1797	-105	61	75	GLAYDISDDQQDITR	56	99.966			Mascot
1709.7977	1709.618	-0.1797	-105	61	75	GLAYDISDDQQDITR					Mascot
1882.9697	1882.766	-0.2037	-108	320	336	LVDTFPGQSIDFFGALR	67	99.997			Mascot
1882.9697	1882.766	-0.2037	-108	320	336	LVDTFPGQSIDFFGALR					Mascot
2089.1692	2088.9438	-0.2254	-108	265	283	VPIIVTGNDFSTLYAPLIR	104	100			Mascot
2089.1692	2088.9438	-0.2254	-108	265	283	VPIIVTGNDFSTLYAPLIR					Mascot

Rank	Protein Name	Accession No	Protein MW	Protein PI	Pep. Count	Protein Score	Protein Score C. I. %	Total Ion Score	Total Ion C. I. %
3	Ribulose bisphosphate carboxylase/oxygenase activase, chloroplastic (Fragments) OS=Arachis hypogaea	RCA_ARAHY	15036.4	4.35	5	264	100	232	100

Peptide Information

Calc. Mass	Obsrv. Mass	± da	± ppm	Start Seq.	End Seq.	Sequence	Ion Score	C. I. %	Modification	Rank	Result Type
1022.545	1022.4402	-0.1048	-102	115	123	IGVCTGIFR	5	0	Carbamidomethyl (C)[4]		Mascot
1022.545	1022.4402	-0.1048	-102	115	123	IGVCTGIFR			Carbamidomethyl (C)[4]		Mascot
1152.7139	1152.5945	-0.1194	-104	47	57	VPLILGIWGGK					Mascot
1709.7977	1709.618	-0.1797	-105	1	15	GLAYDISDDQQDITR	56	99.966			Mascot
1709.7977	1709.618	-0.1797	-105	1	15	GLAYDISDDQQDITR					Mascot
1882.9697	1882.766	-0.2037	-108	124	140	LVDTFPGQSIDFFGALR	67	99.997			Mascot
1882.9697	1882.766	-0.2037	-108	124	140	LVDTFPGQSIDFFGALR					Mascot
2089.1692	2088.9438	-0.2254	-108	96	114	VPIIVTGNDFSTLYAPLIR	104	100			Mascot
2089.1692	2088.9438	-0.2254	-108	96	114	VPIIVTGNDFSTLYAPLIR					Mascot
	2088.9438										Mascot

Rank	Protein Name	Accession No	Protein MW	Protein PI	Pep. Count	Protein Score	Protein Score C. I. %	Total Ion Score	Total Ion C. I. %
4	Ribulose bisphosphate carboxylase/oxygenase a 1, chloroplastic OS=Larrea tridentata GN=RCA1	RCA1_LARTF	52108	6.38	7	263	100	243	100

Peptide Information

Calc. Mass	Obsrv. Mass	± da	± ppm	Start Seq.	End Seq.	Sequence	Ion Score	C. I. %	Modification	Rank	Result Type
940.4675	940.3726	-0.0949	-101	303	309	FYWAPTR	16	0			Mascot
940.4675	940.3726	-0.0949	-101	303	309	FYWAPTR					Mascot
1115.5367	1115.4215	-0.1152	-103	378	387	EGPPSFEQPK					Mascot
1228.603	1228.4749	-0.1281	-104	172	181	SFQCELVFAK			Carbamidomethyl (C)[4]		Mascot
1709.7977	1709.618	-0.1797	-105	73	87	GLAYDISDDQQDITR	56	99.966			Mascot
1709.7977	1709.618	-0.1797	-105	73	87	GLAYDISDDQQDITR					Mascot
1882.9697	1882.766	-0.2037	-108	333	349	LVDTFPGQSIDFFGALR	67	99.997			Mascot
1882.9697	1882.766	-0.2037	-108	333	349	LVDTFPGQSIDFFGALR					Mascot
2089.1692	2088.9438	-0.2254	-108	278	296	VPIIVTGNDFSTLYAPLIR	104	100			Mascot
2089.1692	2088.9438	-0.2254	-108	278	296	VPIIVTGNDFSTLYAPLIR					Mascot
3654.7024	3655.1863	0.4839	132	88	120	GKGMVDTLFQAPMQSGTHYAVMSSYDYISQGLR			Oxidation (M)[4]		Mascot

Rank	Protein Name	Accession No	Protein MW	Protein PI	Pep. Count	Protein Score	Protein Score C. I. %	Total Ion Score	Total Ion C. I. %
5	Ribulose bisphosphate carboxylase/oxygenase activase, chloroplastic OS=Malus domestica GN=RCA PE=2	RCA_MALDO	48046.2	8.2	10	220	100	186	100

Peptide Information

Calc. Mass	Obsrv. Mass	± da	± ppm	Start Seq.	End Seq.	Sequence	Ion Score	C. I. %	Modification	Rank	Result Type
940.4675	940.3726	-0.0949	-101	303	309	FYWAPTR	16	0			Mascot
940.4675	940.3726	-0.0949	-101	303	309	FYWAPTR					Mascot
1023.5105	1023.4305	-0.08	-78	352	359	VYDDEVRK					Mascot
1129.5524	1129.4827	-0.0697	-62	378	387	EGPPTFEQPK					Mascot
1152.7139	1152.5945	-0.1194	-104	157	167	VPLILGIWGGK					Mascot
1228.603	1228.4749	-0.1281	-104	172	181	SFQCELVFAK			Carbamidomethyl (C)[4]		Mascot
1623.7617	1623.5898	-0.1719	-106	222	236	MCALFINDLDAGAGR			Carbamidomethyl (C)[2]		Mascot
1648.8839	1648.762	-0.1219	-74	313	327	IGVCIGIFRSDNVAK			Carbamidomethyl (C)[4]		Mascot
1648.8839	1648.762	-0.1219	-74	313	327	IGVCIGIFRSDNVAK			Carbamidomethyl (C)[4]		Mascot
1882.9697	1882.766	-0.2037	-108	333	349	LVDTFPGQSIDFFGALR	67	99.997			Mascot
1882.9697	1882.766	-0.2037	-108	333	349	LVDTFPGQSIDFFGALR					Mascot
2088.9939	2088.9438	-0.0501	-24	184	204	ISPIMMSAGELESGNAGEPAK					Mascot
2089.1692	2088.9438	-0.2254	-108	278	296	VPIIVTGNDFSTLYAPLIR	104	100			Mascot

Rank	Protein Name	Accession No	Protein MW	Protein PI	Pep. Count	Protein Score	Protein Score C. I. %	Total Ion Score	Total Ion C. I. %
6	Ribulose bisphosphate carboxylase/oxygenase a 2, chloroplastic OS=Nicotiana tabacum GN=RCA P	RCA2_TOBA(	48312.5	8.14	7	210	100	187	100

Peptide Information

Calc. Mass	Obsrv. Mass	± da	± ppm	Start Seq.	End Seq.	Sequence	Ion Score	C. I. %	Modification	Rank	Result Type
940.4675	940.3726	-0.0949	-101	307	313	FYWAPTR	16	0			Mascot
940.4675	940.3726	-0.0949	-101	307	313	FYWAPTR					Mascot
1021.5676	1021.4304	-0.1372	-134	40	49	VYGKGVSSPK					Mascot
1023.5105	1023.4305	-0.08	-78	356	363	VYDDEVRK					Mascot
1214.6263	1214.5046	-0.1217	-100	326	336	TDNVPEEAVIK					Mascot
1882.9697	1882.766	-0.2037	-108	337	353	IVDTFPGQSIDFFGALR	67	99.997			Mascot
1882.9697	1882.766	-0.2037	-108	337	353	IVDTFPGQSIDFFGALR					Mascot
2089.1692	2088.9438	-0.2254	-108	282	300	VPIIVTGNDFSTLYAPLIR	104	100			Mascot
2089.1692	2088.9438	-0.2254	-108	282	300	VPIIVTGNDFSTLYAPLIR					Mascot
2480.1465	2479.8799	-0.2666	-107	185	208	KMGINPIMMSAGELESGNAGEPAK			Oxidation (M)[2,8,9]		Mascot

Rank	Protein Name	Accession No	Protein MW	Protein PI	Pep. Count	Protein Score	Protein Score C. I. %	Total Ion Score	Total Ion C. I. %
7	Ribulose bisphosphate carboxylase/oxygenase activase, chloroplastic OS=Zea mays GN=RCA1 PE=2 SV=3	RCA_MAIZE	47908	6.29	9	209	100	176	100

Peptide Information

Calc. Mass	Obsrv. Mass	± da	± ppm	Start Seq.	End Seq.	Sequence	Ion Score	C. I. %	Modification	Rank	Result Type
940.4675	940.3726	-0.0949	-101	299	305	FYWAPTR	16	0			Mascot
940.4675	940.3726	-0.0949	-101	299	305	FYWAPTR					Mascot
1129.5524	1129.4827	-0.0697	-62	374	383	EGPPTFEQPK					Mascot
1152.7139	1152.5945	-0.1194	-104	153	163	VPLILGIWGGK					Mascot
1228.603	1228.4749	-0.1281	-104	168	177	SFQCELVFAK			Carbamidomethyl (C)[4]		Mascot
1389.7559	1389.5756	-0.1803	-130	132	143	GFMDKLVVHLSK			Oxidation (M)[3]		Mascot
1709.7977	1709.618	-0.1797	-105	69	83	GLAYDISDDQQDITR	56	99.966			Mascot
1709.7977	1709.618	-0.1797	-105	69	83	GLAYDISDDQQDITR					Mascot
1847.7615	1847.6964	-0.0651	-35	117	131	SYNFDNMMDGFYIAK			Oxidation (M)[7,8]		Mascot
1847.7615	1847.6964	-0.0651	-35	117	131	SYNFDNMMDGFYIAK			Oxidation (M)[7,8]		Mascot
2089.1692	2088.9438	-0.2254	-108	274	292	VPIIVTGNDFSTLYAPLIR	104	100			Mascot
2089.1692	2088.9438	-0.2254	-108	274	292	VPIIVTGNDFSTLYAPLIR					Mascot
2211.1404	2210.8584	-0.282	-128	2	24	AAAFSSTVGAPASTPTRSSFLGK					Mascot

Rank	Protein Name	Accession No	Protein MW	Protein PI	Pep. Count	Protein Score	Protein Score C. I. %	Total Ion Score	Total Ion C. I. %
8	Ribulose bisphosphate carboxylase/oxygenase activase, chloroplastic OS=Cucumis sativus PE=2 SV=1	RCA_CUCSA	45681.2	7.57	5	197	100	187	100

Peptide Information

Calc. Mass	Obsrv. Mass	± da	± ppm	Start Seq.	End Seq.	Sequence	Ion Score	C. I. %	Modification	Rank	Result Type
940.4675	940.3726	-0.0949	-101	300	306	FYWAPTR	16	0			Mascot
940.4675	940.3726	-0.0949	-101	300	306	FYWAPTR					Mascot
1023.5105	1023.4305	-0.08	-78	349	356	VYDDEVRK					Mascot
1228.603	1228.4749	-0.1281	-104	169	178	SFQCELVFAK			Carbamidomethyl (C)[4]		Mascot
1882.9697	1882.766	-0.2037	-108	330	346	LVDTFPGQSIDFFGALR	67	99.997			Mascot
1882.9697	1882.766	-0.2037	-108	330	346	LVDTFPGQSIDFFGALR					Mascot
2089.1692	2088.9438	-0.2254	-108	275	293	VPIIVTGNDFSTLYAPLIR	104	100			Mascot
2089.1692	2088.9438	-0.2254	-108	275	293	VPIIVTGNDFSTLYAPLIR					Mascot

Rank	Protein Name	Accession No	Protein MW	Protein PI	Pep. Count	Protein Score	Protein Score C. I. %	Total Ion Score	Total Ion C. I. %
9	Ribulose bisphosphate carboxylase/oxygenase activase, chloroplastic OS=Oryza sativa subsp. japonica	RCA_ORYSJ	51421.4	5.43	8	197	100	176	100

Peptide Information

Calc. Mass	Obsrv. Mass	± da	± ppm	Start Seq.	End Seq.	Sequence	Ion Score	C. I. %	Modification	Rank	Result Type
940.4675	940.3726	-0.0949	-101	294	300	FYWAPTR	16	0			Mascot
940.4675	940.3726	-0.0949	-101	294	300	FYWAPTR					Mascot
1023.5105	1023.4305	-0.08	-78	343	350	VYDDEVRK					Mascot
1152.7139	1152.5945	-0.1194	-104	148	158	VPLILGIWGGK					Mascot
1228.603	1228.4749	-0.1281	-104	163	172	SFQCELVFAK			Carbamidomethyl (C)[4]		Mascot
1709.7977	1709.618	-0.1797	-105	64	78	GLAYDISDDQQDITR	56	99.966			Mascot
1709.7977	1709.618	-0.1797	-105	64	78	GLAYDISDDQQDITR					Mascot
1775.8521	1775.7732	-0.0789	-44	369	383	EGPPEFEQPKMTIEK			Oxidation (M)[11]		Mascot
2089.1692	2088.9438	-0.2254	-108	269	287	VPIIVTGNDFSTLYAPLIR	104	100			Mascot
2089.1692	2088.9438	-0.2254	-108	269	287	VPIIVTGNDFSTLYAPLIR					Mascot
2480.2085	2479.8799	-0.3286	-132	400	422	RVQLAEQYLSEAALGDANSDAMK					Mascot

Rank	Protein Name	Accession No	Protein MW	Protein PI	Pep. Count	Protein Score	Protein Score C. I. %	Total Ion Score	Total Ion C. I. %
10	Ribulose bisphosphate carboxylase/oxygenase a 2, chloroplastic OS=Larrea tridentata GN=RCA2	RCA2_LARTF	48023.1	6.78	7	197	100	176	100

Peptide Information

Calc. Mass	Obsrv. Mass	± da	± ppm	Start Seq.	End Seq.	Sequence	Ion Score	C. I. %	Modification	Rank	Result Type
940.4675	940.3726	-0.0949	-101	303	309	FYWAPTR	16	0			Mascot
940.4675	940.3726	-0.0949	-101	303	309	FYWAPTR					Mascot
1023.5105	1023.4305	-0.08	-78	352	359	VYDDEVRK					Mascot
1129.5524	1129.4827	-0.0697	-62	378	387	EGPPTFEQPK					Mascot
1228.603	1228.4749	-0.1281	-104	172	181	SFQCELVFAK			Carbamidomethyl (C)[4]		Mascot
1709.7977	1709.618	-0.1797	-105	73	87	GLAYDISDDQQDITR	56	99.979			Mascot
1709.7977	1709.618	-0.1797	-105	73	87	GLAYDISDDQQDITR					Mascot
2089.1692	2088.9438	-0.2254	-108	278	296	VPIIVTGNDFSTLYAPLIR	104	100			Mascot
2089.1692	2088.9438	-0.2254	-108	278	296	VPIIVTGNDFSTLYAPLIR					Mascot
3654.7024	3655.1863	0.4839	132	88	120	GKGMVDTLFQAPMQSGTHYAVMSSYDYISQGLR			Oxidation (M)[4]		Mascot

Gel Idx/Pos	66/C15	Instr./Gel Origin	BA2060/131017A	Process Sta	Analysis Succeeded
Plate [#] Name	[1] 31496	Instrument Sample Name		Spectra	8

Rank	Protein Name	Accession No	Protein MW	Protein PI	Pep. Count	Protein Score	Protein Score C. I. %	Total Ion Score	Total Ion C. I. %
1	Ribulose bisphosphate carboxylase/oxygenase activase, chloroplastic OS=Vigna radiata var. radiata G	RCA_VIGRR	47871.3	7.57	9	222	100	186	100

Peptide Information

Calc. Mass	Obsrv. Mass	± da	± ppm	Start Seq.	End Seq.	Sequence	Ion Score	C. I. %	Modification	Rank	Result Type
940.4675	940.3771	-0.0904	-96	305	311	FYWAPTR	17	0			Mascot
940.4675	940.3771	-0.0904	-96	305	311	FYWAPTR					Mascot
1115.5367	1115.4242	-0.1125	-101	380	389	EGPPTFDQPK					Mascot
1152.7139	1152.6019	-0.112	-97	159	169	VPLILGIWGGK					Mascot
1228.603	1228.4756	-0.1274	-104	174	183	SFQCELVFAK			Carbamidomethyl (C)[4]		Mascot
1623.7617	1623.6044	-0.1573	-97	224	238	MCALFINDLDAGAGR			Carbamidomethyl (C)[2]		Mascot
1639.7567	1639.5924	-0.1643	-100	224	238	MCALFINDLDAGAGR			Carbamidomethyl (C)[2], Oxidation (M)[1]		Mascot
1709.7977	1709.627	-0.1707	-100	77	91	GLAYDISDDQQDITR					Mascot
1882.9697	1882.7795	-0.1902	-101	335	351	LVDTFPGQSIDFFGALR					Mascot
1882.9697	1882.7795	-0.1902	-101	335	351	LVDTFPGQSIDFFGALR	76	100			Mascot
2089.1692	2088.9587	-0.2105	-101	280	298	VPIIVTGNDFSTLYAPLIR					Mascot
2089.1692	2088.9587	-0.2105	-101	280	298	VPIIVTGNDFSTLYAPLIR	92	100			Mascot
2480.2524	2479.9041	-0.3483	-140	390	410	MSLDKLLQYGNMLVQEQENVK					Mascot
2480.2524	2479.9041	-0.3483	-140	390	410	MSLDKLLQYGNMLVQEQENVK					Mascot

Rank	Protein Name	Accession No	Protein MW	Protein PI	Pep. Count	Protein Score	Protein Score C. I. %	Total Ion Score	Total Ion C. I. %
2	Ribulose bisphosphate carboxylase/oxygenase a B, chloroplastic OS=Hordeum vulgare GN=RCAB PE	RCAB_HORVI	47197.8	7.59	7	206	100	186	100

Peptide Information

Calc. Mass	Obsrv. Mass	± da	± ppm	Start Seq.	End Seq.	Sequence	Ion Score	C. I. %	Modification	Rank	Result Type
Calc. Mass	Obsrv. Mass	± da	± ppm	Seq.	Seq.	Sequence	Score	C. I. %	Modification	Rank	Result Type
940.4675	940.3771	-0.0904	-96	290	296	FYWAPTR	17	0			Mascot
940.4675	940.3771	-0.0904	-96	290	296	FYWAPTR					Mascot
1115.5367	1115.4242	-0.1125	-101	365	374	EGPPTFDQPK					Mascot
1152.7139	1152.6019	-0.112	-97	145	155	VPLILGIWGGK					Mascot
1228.603	1228.4756	-0.1274	-104	160	169	SFQCELVFAK			Carbamidomethyl (C)[4]		Mascot
1709.7977	1709.627	-0.1707	-100	61	75	GLAYDISDDQQDITR					Mascot
1882.9697	1882.7795	-0.1902	-101	320	336	LVDTFPGQSIDFFGALR					Mascot
1882.9697	1882.7795	-0.1902	-101	320	336	LVDTFPGQSIDFFGALR	76	100			Mascot
2089.1692	2088.9587	-0.2105	-101	265	283	VPIIVTGNDFSTLYAPLIR					Mascot
2089.1692	2088.9587	-0.2105	-101	265	283	VPIIVTGNDFSTLYAPLIR	92	100			Mascot

Rank	Protein Name	Accession No	Protein MW	Protein PI	Pep. Count	Protein Score	Protein Score C. I. %	Total Ion Score	Total Ion C. I. %
3	Ribulose bisphosphate carboxylase/oxygenase activase, chloroplastic OS=Malus domestica GN=RCA PE=2	RCA_MALDO	48046.2	8.2	7	204	100	184	100

Peptide Information

Calc. Mass	Obsrv. Mass	± da	± ppm	Start Seq.	End Seq.	Sequence	Ion Score	C. I. %	Modification	Rank	Result Type
940.4675	940.3771	-0.0904	-96	303	309	FYWAPTR	17	0			Mascot
940.4675	940.3771	-0.0904	-96	303	309	FYWAPTR					Mascot
1152.7139	1152.6019	-0.112	-97	157	167	VPLILGIWGGK					Mascot
1228.603	1228.4756	-0.1274	-104	172	181	SFQCELVFAK			Carbamidomethyl (C)[4]		Mascot
1623.7617	1623.6044	-0.1573	-97	222	236	MCALFINDLDAGAGR			Carbamidomethyl (C)[2]		Mascot
1639.7567	1639.5924	-0.1643	-100	222	236	MCALFINDLDAGAGR			Carbamidomethyl (C)[2], Oxidation (M)[1]		Mascot
1882.9697	1882.7795	-0.1902	-101	333	349	LVDTFPGQSIDFFGALR					Mascot
1882.9697	1882.7795	-0.1902	-101	333	349	LVDTFPGQSIDFFGALR	76	100			Mascot
2088.9939	2088.9587	-0.0352	-17	184	204	ISPIMMSAGELESGNAGEPAK					Mascot
2089.1692	2088.9587	-0.2105	-101	278	296	VPIIVTGNDFSTLYAPLIR	92	100			Mascot

Rank	Protein Name	Accession No	Protein MW	Protein PI	Pep. Count	Protein Score	Protein Score C. I. %	Total Ion Score	Total Ion C. I. %
4	Ribulose bisphosphate carboxylase/oxygenase activase, chloroplastic (Fragments) OS=Arachis hypogaea	RCA_ARAHY	15036.4	4.35	5	201	100	168	100

Peptide Information

Calc. Mass	Obsrv. Mass	± da	± ppm	Start Seq.	End Seq.	Sequence	Ion Score	C. I. %	Modification	Rank	Result Type
1022.545	1022.4481	-0.0969	-95	115	123	IGVCTGIFR			Carbamidomethyl (C)[4]		Mascot
1152.7139	1152.6019	-0.112	-97	47	57	VPLILGIWGGK					Mascot
1709.7977	1709.627	-0.1707	-100	1	15	GLAYDISDDQQDITR					Mascot
1882.9697	1882.7795	-0.1902	-101	124	140	LVDTFPGQSIDFFGALR					Mascot
1882.9697	1882.7795	-0.1902	-101	124	140	LVDTFPGQSIDFFGALR	76	100			Mascot
2089.1692	2088.9587	-0.2105	-101	96	114	VPIIVTGNDFSTLYAPLIR					Mascot
2089.1692	2088.9587	-0.2105	-101	96	114	VPIIVTGNDFSTLYAPLIR	92	100			Mascot

Rank	Protein Name	Accession No	Protein MW	Protein PI	Pep. Count	Protein Score	Protein Score C. I. %	Total Ion Score	Total Ion C. I. %
5	Ribulose bisphosphate carboxylase/oxygenase a 1, chloroplastic OS=Larrea tridentata GN=RCA1	RCA1_LARTF	52108	6.38	6	199	100	186	100

Peptide Information

Calc. Mass	Obsrv. Mass	± da	± ppm	Start Seq.	End Seq.	Sequence	Ion Score	C. I. %	Modification	Rank	Result Type
940.4675	940.3771	-0.0904	-96	303	309	FYWAPTR	17	0			Mascot
940.4675	940.3771	-0.0904	-96	303	309	FYWAPTR					Mascot
1115.5367	1115.4242	-0.1125	-101	378	387	EGPPSFEQPK					Mascot
1228.603	1228.4756	-0.1274	-104	172	181	SFQCELVFAK			Carbamidomethyl (C)[4]		Mascot
1709.7977	1709.627	-0.1707	-100	73	87	GLAYDISDDQQDITR					Mascot
1882.9697	1882.7795	-0.1902	-101	333	349	LVDTFPGQSIDFFGALR					Mascot
1882.9697	1882.7795	-0.1902	-101	333	349	LVDTFPGQSIDFFGALR	76	100			Mascot
2089.1692	2088.9587	-0.2105	-101	278	296	VPIIVTGNDFSTLYAPLIR					Mascot
2089.1692	2088.9587	-0.2105	-101	278	296	VPIIVTGNDFSTLYAPLIR	92	100			Mascot

Rank	Protein Name	Accession No	Protein MW	Protein PI	Pep. Count	Protein Score	Protein Score C. I. %	Total Ion Score	Total Ion C. I. %
6	Ribulose bisphosphate carboxylase/oxygenase a 2, chloroplastic OS=Nicotiana tabacum GN=RCA P	RCA2_TOBA(	48312.5	8.14	4	195	100	186	100

Peptide Information

Calc. Mass	Obsrv. Mass	± da	± ppm	Start Seq.	End Seq.	Sequence	Ion Score	C. I. %	Modification	Rank	Result Type
940.4675	940.3771	-0.0904	-96	307	313	FYWAPTR	17	0			Mascot
940.4675	940.3771	-0.0904	-96	307	313	FYWAPTR					Mascot
1882.9697	1882.7795	-0.1902	-101	337	353	IVDTFPGQSIDFFGALR					Mascot
1882.9697	1882.7795	-0.1902	-101	337	353	IVDTFPGQSIDFFGALR	76	100			Mascot
2089.1692	2088.9587	-0.2105	-101	282	300	VPIIVTGNDFSTLYAPLIR					Mascot
2089.1692	2088.9587	-0.2105	-101	282	300	VPIIVTGNDFSTLYAPLIR	92	100			Mascot
2480.1465	2479.9041	-0.2424	-98	185	208	KMGINPIMMSAGELESGNAGEPAK			Oxidation (M)[2,8,9]		Mascot
2480.1465	2479.9041	-0.2424	-98	185	208	KMGINPIMMSAGELESGNAGEPAK			Oxidation (M)[2,8,9]		Mascot

Rank	Protein Name	Accession No	Protein MW	Protein PI	Pep. Count	Protein Score	Protein Score C. I. %	Total Ion Score	Total Ion C. I. %
7	Ribulose bisphosphate carboxylase/oxygenase activase, chloroplastic OS=Cucumis sativus PE=2 SV=1	RCA_CUCSA	45681.2	7.57	4	194	100	186	100

Peptide Information

Calc. Mass	Obsrv. Mass	± da	± ppm	Start Seq.	End Seq.	Sequence	Ion Score	C. I. %	Modification	Rank	Result Type
Calc. Mass	Obsrv. Mass	± da	± ppm	Start	Seq.	Sequence	Score	C. I. %	Modification	Rank	Result Type
940.4675	940.3771	-0.0904	-96	300	306	FYWAPTR	17	0			Mascot
940.4675	940.3771	-0.0904	-96	300	306	FYWAPTR					Mascot
1228.603	1228.4756	-0.1274	-104	169	178	SFQCELVFAK			Carbamidomethyl (C)[4]		Mascot
1882.9697	1882.7795	-0.1902	-101	330	346	LVDTFPGQSIDFFGALR					Mascot
1882.9697	1882.7795	-0.1902	-101	330	346	LVDTFPGQSIDFFGALR	76	100			Mascot
2089.1692	2088.9587	-0.2105	-101	275	293	VPIIVTGNDFSTLYAPLIR					Mascot
2089.1692	2088.9587	-0.2105	-101	275	293	VPIIVTGNDFSTLYAPLIR	92	100			Mascot

Rank	Protein Name	Accession No	Protein MW	Protein PI	Pep. Count	Protein Score	Protein Score C. I. %	Total Ion Score	Total Ion C. I. %
8	Phosphoribulokinase, chloroplastic OS=Mesembryanthemum crystallinum PE=2 SV=1	KPPR_MESCI	44086.5	6.03	8	183	100	148	100

Peptide Information

Calc. Mass	Obsrv. Mass	± da	± ppm	Start Seq.	End Seq.	Sequence	Ion Score	C. I. %	Modification	Rank	Result Type
828.4573	828.3846	-0.0727	-88	117	124	GVTALDPR					Mascot
870.4679	870.4583	-0.0096	-11	211	218	GHSLESIK					Mascot
1343.6664	1343.531	-0.1354	-101	344	354	FYGEVTQQMLK					Mascot
1359.6613	1359.527	-0.1343	-99	344	354	FYGEVTQQMLK			Oxidation (M)[9]		Mascot
1472.6726	1472.5287	-0.1439	-98	125	136	ANDFDLMYEQVK					Mascot
1488.6675	1488.5181	-0.1494	-100	125	136	ANDFDLMYEQVK			Oxidation (M)[7]		Mascot
1626.8672	1626.7056	-0.1616	-99	167	180	ILVIEGLHPMFDSR					Mascot
1642.8621	1642.6992	-0.1629	-99	167	180	ILVIEGLHPMFDSR			Oxidation (M)[10]		Mascot
1642.8621	1642.6992	-0.1629	-99	167	180	ILVIEGLHPMFDSR	31	92.638	Oxidation (M)[10]		Mascot
1961.0226	1960.8229	-0.1997	-102	327	343	LDELIYVESHLSNLSTK					Mascot
2139.1331	2138.9155	-0.2176	-102	181	198	VRDLLDFSIYLDISNEVK					Mascot
2979.3635	2979.0432	-0.3203	-108	84	110	GGNPDSNTLISDTTTVICLDDYHSLDR			Carbamidomethyl (C)[18]		Mascot
2979.3635	2979.0432	-0.3203	-108	84	110	GGNPDSNTLISDTTTVICLDDYHSLDR	117	100	Carbamidomethyl (C)[18]		Mascot

Rank	Protein Name	Accession No	Protein MW	Protein PI	Pep. Count	Protein Score	Protein Score C. I. %	Total Ion Score	Total Ion C. I. %
9	Phosphoribulokinase, chloroplastic OS=Triticum aestivum PE=2 SV=1	KPPR_WHEA	45112.9	5.72	9	159	100	117	100

Peptide Information

Calc. Mass	Obsrv. Mass	± da	± ppm	Start Seq.	End Seq.	Sequence	Ion Score	C. I. %	Modification	Rank	Result Type
870.4679	870.4583	-0.0096	-11	218	225	GHSLESIK					Mascot
1273.6787	1273.5487	-0.13	-102	78	90	LTSVFGGAAEPPK					Mascot
1343.6664	1343.531	-0.1354	-101	351	361	FYGEVTQQMLK					Mascot
1359.6613	1359.527	-0.1343	-99	351	361	FYGEVTQQMLK			Oxidation (M)[9]		Mascot
1429.7798	1429.6375	-0.1423	-100	77	90	RLTSVFGGAAEPPK					Mascot
1472.6726	1472.5287	-0.1439	-98	132	143	ANDFDLMYEQVK					Mascot
1488.6675	1488.5181	-0.1494	-100	132	143	ANDFDLMYEQVK			Oxidation (M)[7]		Mascot
1961.0226	1960.8229	-0.1997	-102	334	350	LDELIYVESHLSNLSTK					Mascot
2173.1035	2172.876	-0.2275	-105	362	382	HADFPGSNNGTGLFQTIVGLK					Mascot
2428.2241	2427.988	-0.2361	-97	244	265	QYADAVIEVLPTQLIPDDNEGK					Mascot
2979.3635	2979.0432	-0.3203	-108	91	117	GGNPDSNTLISDTTTVICLDDYHSLDR			Carbamidomethyl (C)[18]		Mascot
2979.3635	2979.0432	-0.3203	-108	91	117	GGNPDSNTLISDTTTVICLDDYHSLDR	117	100	Carbamidomethyl (C)[18]		Mascot

Rank	Protein Name	Accession No	Protein MW	Protein PI	Pep. Count	Protein Score	Protein Score C. I. %	Total Ion Score	Total Ion C. I. %
10	Phosphoribulokinase, chloroplastic OS=Arabidop thaliana GN=At1g32060 PE=1 SV=1	KPPR_ARATI	44435.6	5.71	8	152	100	117	100

Peptide Information

Calc. Mass	Obsrv. Mass	± da	± ppm	Start Seq.	End Seq.	Sequence	Ion Score	C. I. %	Modification	Rank	Result Type
870.4679	870.4583	-0.0096	-11	210	217	GHSLESIK					Mascot
1343.6664	1343.531	-0.1354	-101	343	353	FYGEVTQQMLK					Mascot
1359.6613	1359.527	-0.1343	-99	343	353	FYGEVTQQMLK			Oxidation (M)[9]		Mascot
1472.6726	1472.5287	-0.1439	-98	124	135	ANDFDLMYEQVK					Mascot
1488.6675	1488.5181	-0.1494	-100	124	135	ANDFDLMYEQVK			Oxidation (M)[7]		Mascot
1961.0226	1960.8229	-0.1997	-102	326	342	LDELIYVESHLSNLSTK					Mascot
2139.1331	2138.9155	-0.2176	-102	180	197	VRDLLDFSIYLDISNEVK					Mascot
2173.1035	2172.876	-0.2275	-105	354	374	HADFPGSNNGTGLFQTIVGLK					Mascot
2225.0906	2224.8877	-0.2029	-91	117	135	VTALDPRANDFDLMYEQVK					Mascot
2979.3635	2979.0432	-0.3203	-108	83	109	GGNPDSNTLISDTTTVICLDDYHSLDR			Carbamidomethyl (C)[18]		Mascot
2979.3635	2979.0432	-0.3203	-108	83	109	GGNPDSNTLISDTTTVICLDDYHSLDR	117	100	Carbamidomethyl (C)[18]		Mascot

Gel Idx/Pos	67/C16	Instr./Gel Origin	BA2060/131017A	Process Sta	Analysis Succeeded
Plate [#] Name	[1] 31496	Instrument Sample Name		Spectra	8

Rank	Protein Name	Accession No.	Protein MW	Protein PI	Pep. Count	Protein Score	Protein Score C. I. %	Total Ion Score	Total Ion C. I. %
1	Ribulose bisphosphate carboxylase/oxygenase activase, chloroplastic OS=Vigna radiata var. radiata G	RCA_VIGRR	47871.3	7.57	16	633	100	535	100

Peptide Information

Calc. Mass	Obsrv. Mass	± da	± ppm	Start Seq.	End Seq.	Sequence	Ion Score	C. I. %	Modification	Rank	Result Type
809.5243	809.4567	-0.0676	-84	143	149	LVVHITK					Mascot
829.489	829.4186	-0.0704	-85	411	417	RVQLADK					Mascot
895.4155	895.3469	-0.0686	-77	354	360	VYDDEVR					Mascot
940.4675	940.389	-0.0785	-83	305	311	FYWAPTR					Mascot
940.4675	940.389	-0.0785	-83	305	311	FYWAPTR	26	75.909			Mascot
1015.5781	1015.501	-0.0771	-76	214	223	EAADLIAKGK					Mascot
1077.5762	1077.4805	-0.0957	-89	150	158	NFMTLPNIK					Mascot
1093.571	1093.4717	-0.0993	-91	150	158	NFMTLPNIK			Oxidation (M)[3]		Mascot
1115.5367	1115.4391	-0.0976	-87	380	389	EGPPTFDQPK					Mascot
1152.7139	1152.6171	-0.0968	-84	159	169	VPLILGIWGGK					Mascot
1228.603	1228.4926	-0.1104	-90	174	183	SFQCELVFAK			Carbamidomethyl (C)[4]		Mascot
1326.6226	1326.5073	-0.1153	-87	305	314	FYWAPTRDDR					Mascot
1623.7617	1623.6115	-0.1502	-93	224	238	MCALFINDLDAGAGR	116	100	Carbamidomethyl (C)[2]		Mascot
1623.7617	1623.6115	-0.1502	-93	224	238	MCALFINDLDAGAGR			Carbamidomethyl (C)[2]		Mascot
1639.7567	1639.6095	-0.1472	-90	224	238	MCALFINDLDAGAGR			Carbamidomethyl (C)[2], Oxidation (M)[1]		Mascot
1709.7977	1709.6394	-0.1583	-93	77	91	GLAYDISDDQQDITR	120	100			Mascot
1709.7977	1709.6394	-0.1583	-93	77	91	GLAYDISDDQQDITR					Mascot
1882.9697	1882.7939	-0.1758	-93	335	351	LVDTFPGQSIDFFGALR					Mascot
1882.9697	1882.7939	-0.1758	-93	335	351	LVDTFPGQSIDFFGALR	152	100			Mascot
1894.9141	1894.7916	-0.1225	-65	77	93	GLAYDISDDQQDITRGK					Mascot
2089.1692	2088.9683	-0.2009	-96	280	298	VPIIVTGNDFSTLYAPLIR	121	100			Mascot
2089.1692	2088.9683	-0.2009	-96	280	298	VPIIVTGNDFSTLYAPLIR					Mascot
2304.0669	2303.8491	-0.2178	-95	184	206	MGINPIMMSAGELESGNAGEPAK					Mascot
2336.0566	2335.821	-0.2356	-101	184	206	MGINPIMMSAGELESGNAGEPAK			Oxidation (M)[1,7]		Mascot

Rank	Protein Name	Accession No.	Protein MW	Protein PI	Pep. Count	Protein Score	Protein Score C. I. %	Total Ion Score	Total Ion C. I. %
2	Ribulose bisphosphate carboxylase/oxygenase activase 1, chloroplastic OS=Larrea tridentata GN=RCA1	RCA1_LARTR	52108	6.38	14	487	100	419	100

Peptide Information

Calc. Mass	Obsrv. Mass	± da	± ppm	Start Seq.	End Seq.	Sequence	Ion Score	C. I. %	Modification	Rank	Result Type
809.5243	809.4567	-0.0676	-84	141	147	LVVHITK					Mascot
829.489	829.4186	-0.0704	-85	409	415	RVQLADK					Mascot
940.4675	940.389	-0.0785	-83	303	309	FYWAPTR					Mascot
940.4675	940.389	-0.0785	-83	303	309	FYWAPTR	26	75.909			Mascot
1045.5425	1045.48	-0.0625	-60	352	359	VYHDEVRK					Mascot
1078.5891	1078.4933	-0.0958	-89	210	218	YREAADIIK					Mascot
1115.5367	1115.4391	-0.0976	-87	378	387	EGPPSFEQPK					Mascot
1228.603	1228.4926	-0.1104	-90	172	181	SFQCELVFAK			Carbamidomethyl (C)[4]		Mascot
1326.647	1326.5073	-0.1397	-105	1	13	MAAAYSTVGAVNR			Oxidation (M)[1]		Mascot
1340.6382	1340.5099	-0.1283	-96	303	312	FYWAPTREDR					Mascot
1709.7977	1709.6394	-0.1583	-93	73	87	GLAYDISDDQQDITR	120	100			Mascot
1709.7977	1709.6394	-0.1583	-93	73	87	GLAYDISDDQQDITR					Mascot
1882.9697	1882.7939	-0.1758	-93	333	349	LVDTFPGQSIDFFGALR					Mascot
1882.9697	1882.7939	-0.1758	-93	333	349	LVDTFPGQSIDFFGALR	152	100			Mascot
1894.9141	1894.7916	-0.1225	-65	73	89	GLAYDISDDQQDITRGK					Mascot
2089.1692	2088.9683	-0.2009	-96	278	296	VPIIVTGNDFSTLYAPLIR	121	100			Mascot
2089.1692	2088.9683	-0.2009	-96	278	296	VPIIVTGNDFSTLYAPLIR					Mascot
2304.0669	2303.8491	-0.2178	-95	182	204	MGINPIMMSAGELESGNAGEPAK					Mascot
2336.0566	2335.821	-0.2356	-101	182	204	MGINPIMMSAGELESGNAGEPAK			Oxidation (M)[1,7]		Mascot

Rank	Protein Name	Accession No.	Protein MW	Protein PI	Pep. Count	Protein Score	Protein Score C. I. %	Total Ion Score	Total Ion C. I. %
3	Ribulose bisphosphate carboxylase/oxygenase activase, chloroplastic (Fragments) OS=Arachis hypogaea	RCA_ARAHY	15036.4	4.35	6	481	100	437	100

Peptide Information

Calc. Mass	Obsrv. Mass	± da	± ppm	Start Seq.	End Seq.	Sequence	Ion Score	C. I. %	Modification	Rank	Result Type
1022.545	1022.4594	-0.0856	-84	115	123	IGVCTGIFR			Carbamidomethyl (C)[4]		Mascot
1022.545	1022.4594	-0.0856	-84	115	123	IGVCTGIFR	44	99.629	Carbamidomethyl (C)[4]		Mascot
1152.7139	1152.6171	-0.0968	-84	47	57	VPLILGIWGGK					Mascot
1709.7977	1709.6394	-0.1583	-93	1	15	GLAYDISDDQQDITR	120	100			Mascot
1709.7977	1709.6394	-0.1583	-93	1	15	GLAYDISDDQQDITR					Mascot
1882.9697	1882.7939	-0.1758	-93	124	140	LVDTFPGQSIDFFGALR					Mascot
1882.9697	1882.7939	-0.1758	-93	124	140	LVDTFPGQSIDFFGALR	152	100			Mascot
2089.1692	2088.9683	-0.2009	-96	96	114	VPIIVTGNDFSTLYAPLIR	121	100			Mascot
2089.1692	2088.9683	-0.2009	-96	96	114	VPIIVTGNDFSTLYAPLIR					Mascot
2304.0669	2303.8491	-0.2178	-95	58	80	MGINPIMMSAGELESGNAGEPAK					Mascot
2336.0566	2335.821	-0.2356	-101	58	80	MGINPIMMSAGELESGNAGEPAK			Oxidation (M)[1,7]		Mascot

Rank	Protein Name	Accession No.	Protein MW	Protein PI	Pep. Count	Protein Score	Protein Score C. I. %	Total Ion Score	Total Ion C. I. %
4	Ribulose bisphosphate carboxylase/oxygenase activase B, chloroplastic OS=Hordeum vulgare GN=RCAB PE	RCAB_HORVU	47197.8	7.59	13	477	100	419	100

Peptide Information

Calc. Mass	Obsrv. Mass	± da	± ppm	Start Seq.	End Seq.	Sequence	Ion Score	C. I. %	Modification	Rank	Result Type
829.489	829.4186	-0.0704	-85	396	402	RVQLADK					Mascot
895.4155	895.3469	-0.0686	-77	339	345	VYDDEVR					Mascot
940.4675	940.389	-0.0785	-83	290	296	FYWAPTR					Mascot
940.4675	940.389	-0.0785	-83	290	296	FYWAPTR	26	75.909			Mascot
1077.5762	1077.4805	-0.0957	-89	136	144	NFMTLPNIK					Mascot
1093.571	1093.4717	-0.0993	-91	136	144	NFMTLPNIK			Oxidation (M)[3]		Mascot
1115.5367	1115.4391	-0.0976	-87	365	374	EGPPTFDQPK					Mascot
1152.7139	1152.6171	-0.0968	-84	145	155	VPLILGIWGGK					Mascot
1214.6012	1214.5192	-0.082	-68	309	319	TDNVPDEAVVR					Mascot
1228.603	1228.4926	-0.1104	-90	160	169	SFQCELVFAK			Carbamidomethyl (C)[4]		Mascot
1340.6382	1340.5099	-0.1283	-96	290	299	FYWAPTREDR					Mascot
1709.7977	1709.6394	-0.1583	-93	61	75	GLAYDISDDQQDITR	120	100			Mascot
1709.7977	1709.6394	-0.1583	-93	61	75	GLAYDISDDQQDITR					Mascot
1882.9697	1882.7939	-0.1758	-93	320	336	LVDTFPGQSIDFFGALR					Mascot
1882.9697	1882.7939	-0.1758	-93	320	336	LVDTFPGQSIDFFGALR	152	100			Mascot
1894.9141	1894.7916	-0.1225	-65	61	77	GLAYDISDDQQDITRGK					Mascot
2089.1692	2088.9683	-0.2009	-96	265	283	VPIIVTGNDFSTLYAPLIR	121	100			Mascot
2089.1692	2088.9683	-0.2009	-96	265	283	VPIIVTGNDFSTLYAPLIR					Mascot

Rank	Protein Name	Accession No.	Protein MW	Protein PI	Pep. Count	Protein Score	Protein Score C. I. %	Total Ion Score	Total Ion C. I. %
5	Ribulose bisphosphate carboxylase/oxygenase activase, chloroplastic OS=Malus domestica GN=RCA PE=2	RCA_MALDO	48046.2	8.2	12	464	100	413	100

Peptide Information

Calc. Mass	Obsrv. Mass	± da	± ppm	Start Seq.	End Seq.	Sequence	Ion Score	C. I. %	Modification	Rank	Result Type
809.5243	809.4567	-0.0676	-84	141	147	LVVHITK					Mascot
829.489	829.4186	-0.0704	-85	409	415	RVQLADK					Mascot
895.4155	895.3469	-0.0686	-77	352	358	VYDDEVR					Mascot
940.4675	940.389	-0.0785	-83	303	309	FYWAPTR					Mascot
940.4675	940.389	-0.0785	-83	303	309	FYWAPTR	26	75.909			Mascot
1152.7139	1152.6171	-0.0968	-84	157	167	VPLILGIWGGK					Mascot
1228.603	1228.4926	-0.1104	-90	172	181	SFQCELVFAK			Carbamidomethyl (C)[4]		Mascot
1340.6382	1340.5099	-0.1283	-96	303	312	FYWAPTREDR					Mascot
1623.7617	1623.6115	-0.1502	-93	222	236	MCALFINDLDAGAGR	116	100	Carbamidomethyl (C)[2]		Mascot
1623.7617	1623.6115	-0.1502	-93	222	236	MCALFINDLDAGAGR			Carbamidomethyl (C)[2]		Mascot
1639.7567	1639.6095	-0.1472	-90	222	236	MCALFINDLDAGAGR			Carbamidomethyl (C)[2], Oxidation (M)[1]		Mascot
1882.9697	1882.7939	-0.1758	-93	333	349	LVDTFPGQSIDFFGALR					Mascot
1882.9697	1882.7939	-0.1758	-93	333	349	LVDTFPGQSIDFFGALR	152	100			Mascot
2063.0591	2062.894	-0.1651	-80	393	409	LLEYGNMLVQEQENVKR					Mascot
	2062.894										Mascot
2088.9939	2088.9683	-0.0256	-12	184	204	ISPIMMSAGELESGNAGEPAK					Mascot
2089.1692	2088.9683	-0.2009	-96	278	296	VPIIVTGNDFSTLYAPLIR	121	100			Mascot

Rank	Protein Name	Accession No.	Protein MW	Protein PI	Pep. Count	Protein Score	Protein Score C. I. %	Total Ion Score	Total Ion C. I. %
6	Ribulose bisphosphate carboxylase/oxygenase activase, chloroplastic OS=Zea mays GN=RCA1 PE=2 SV=3	RCA_MAIZE	47908	6.29	15	348	100	267	100

Peptide Information

Calc. Mass	Obsrv. Mass	± da	± ppm	Start Seq.	End Seq.	Sequence	Ion Score	C. I. %	Modification	Rank	Result Type
829.489	829.4186	-0.0704	-85	405	411	RVQLADK					Mascot
895.4155	895.3469	-0.0686	-77	348	354	VYDDEVR					Mascot
940.4675	940.389	-0.0785	-83	299	305	FYWAPTR					Mascot
940.4675	940.389	-0.0785	-83	299	305	FYWAPTR	26	75.909			Mascot
1077.496	1077.4805	-0.0155	-14	61	68	QTDEDRWK					Mascot
1093.571	1093.4717	-0.0993	-91	144	152	NFMTLPNIK			Oxidation (M)[3]		Mascot
1152.7139	1152.6171	-0.0968	-84	153	163	VPLILGIWGGK					Mascot
1228.603	1228.4926	-0.1104	-90	168	177	SFQCELVFAK			Carbamidomethyl (C)[4]		Mascot
1340.6382	1340.5099	-0.1283	-96	299	308	FYWAPTREDR					Mascot
1373.761	1373.6029	-0.1581	-115	132	143	GFMDKLVVHLSK					Mascot
1639.7567	1639.6095	-0.1472	-90	218	232	MSCLFINDLDAGAGR			Carbamidomethyl (C)[3]		Mascot
1709.7977	1709.6394	-0.1583	-93	69	83	GLAYDISDDQQDITR	120	100			Mascot
1709.7977	1709.6394	-0.1583	-93	69	83	GLAYDISDDQQDITR					Mascot
1847.7615	1847.7159	-0.0456	-25	117	131	SYNFDNMMDGFYIAK			Oxidation (M)[7,8]		Mascot
1847.7615	1847.7159	-0.0456	-25	117	131	SYNFDNMMDGFYIAK			Oxidation (M)[7,8]		Mascot
1894.9141	1894.7916	-0.1225	-65	69	85	GLAYDISDDQQDITRGK					Mascot
2089.1692	2088.9683	-0.2009	-96	274	292	VPIIVTGNDFSTLYAPLIR	121	100			Mascot
2089.1692	2088.9683	-0.2009	-96	274	292	VPIIVTGNDFSTLYAPLIR					Mascot
2211.1404	2210.8948	-0.2456	-111	2	24	AAAFSSTVGAPASTPTRSSFLGK					Mascot

Rank	Protein Name	Accession No.	Protein MW	Protein PI	Pep. Count	Protein Score	Protein Score C. I. %	Total Ion Score	Total Ion C. I. %
7	Ribulose bisphosphate carboxylase/oxygenase activase 2, chloroplastic OS=Nicotiana tabacum GN=RCA P	RCA2_TOBAC	48312.5	8.14	10	339	100	299	100

Peptide Information

Calc. Mass	Obsrv. Mass	± da	± ppm	Start Seq.	End Seq.	Sequence	Ion Score	C. I. %	Modification	Rank	Result Type
809.5243	809.4567	-0.0676	-84	145	151	LVVHITK					Mascot
895.4155	895.3469	-0.0686	-77	356	362	VYDDEVR					Mascot
940.4675	940.389	-0.0785	-83	307	313	FYWAPTR					Mascot
940.4675	940.389	-0.0785	-83	307	313	FYWAPTR	26	75.909			Mascot
1214.6263	1214.5192	-0.1071	-88	326	336	TDNVPEEAVIK					Mascot
1340.6382	1340.5099	-0.1283	-96	307	316	FYWAPTREDR					Mascot
1555.7686	1555.5615	-0.2071	-133	172	184	GQGKSFQCELVFR			Carbamidomethyl (C)[8]		Mascot
1882.9697	1882.7939	-0.1758	-93	337	353	IVDTFPGQSIDFFGALR					Mascot
1882.9697	1882.7939	-0.1758	-93	337	353	IVDTFPGQSIDFFGALR	152	100			Mascot
2063.0591	2062.894	-0.1651	-80	397	413	LLEYGNMLVQEQENVKR					Mascot
2089.1692	2088.9683	-0.2009	-96	282	300	VPIIVTGNDFSTLYAPLIR	121	100			Mascot
2089.1692	2088.9683	-0.2009	-96	282	300	VPIIVTGNDFSTLYAPLIR					Mascot
2304.0669	2303.8491	-0.2178	-95	186	208	MGINPIMMSAGELESGNAGEPAK					Mascot
2336.0566	2335.821	-0.2356	-101	186	208	MGINPIMMSAGELESGNAGEPAK			Oxidation (M)[1,7]		Mascot

Rank	Protein Name	Accession No.	Protein MW	Protein PI	Pep. Count	Protein Score	Protein Score C. I. %	Total Ion Score	Total Ion C. I. %
8	Ribulose bisphosphate carboxylase/oxygenase activase 2, chloroplastic OS=Larrea tridentata GN=RCA2	RCA2_LARTR	48023.1	6.78	14	339	100	267	100

Peptide Information

Calc. Mass	Obsrv. Mass	± da	± ppm	Start Seq.	End Seq.	Sequence	Ion Score	C. I. %	Modification	Rank	Result Type
809.5243	809.4567	-0.0676	-84	141	147	LVVHITK					Mascot
829.489	829.4186	-0.0704	-85	409	415	RVQLADK					Mascot
895.4155	895.3469	-0.0686	-77	352	358	VYDDEVR					Mascot
940.4675	940.389	-0.0785	-83	303	309	FYWAPTR					Mascot
940.4675	940.389	-0.0785	-83	303	309	FYWAPTR	26	75.909			Mascot
1045.604	1045.48	-0.124	-119	148	156	NFLSLPNIK					Mascot
1078.5891	1078.4933	-0.0958	-89	210	218	YREAADIIK					Mascot
1228.603	1228.4926	-0.1104	-90	172	181	SFQCELVFAK			Carbamidomethyl (C)[4]		Mascot
1326.647	1326.5073	-0.1397	-105	1	13	MAAAYSTVGAVNR			Oxidation (M)[1]		Mascot
1340.6382	1340.5099	-0.1283	-96	303	312	FYWAPTREDR					Mascot
1709.7977	1709.6394	-0.1583	-93	73	87	GLAYDISDDQQDITR	120	100			Mascot
1709.7977	1709.6394	-0.1583	-93	73	87	GLAYDISDDQQDITR					Mascot
1894.9141	1894.7916	-0.1225	-65	73	89	GLAYDISDDQQDITRGK					Mascot
2063.0591	2062.894	-0.1651	-80	393	409	LLQYGNMLVEEQENVKR					Mascot
2089.1692	2088.9683	-0.2009	-96	278	296	VPIIVTGNDFSTLYAPLIR	121	100			Mascot
2089.1692	2088.9683	-0.2009	-96	278	296	VPIIVTGNDFSTLYAPLIR					Mascot
2304.0669	2303.8491	-0.2178	-95	182	204	MGINPIMMSAGELESGNAGEPAK					Mascot
2336.0566	2335.821	-0.2356	-101	182	204	MGINPIMMSAGELESGNAGEPAK			Oxidation (M)[1,7]		Mascot

Rank	Protein Name	Accession No.	Protein MW	Protein PI	Pep. Count	Protein Score	Protein Score C. I. %	Total Ion Score	Total Ion C. I. %
9	Ribulose bisphosphate carboxylase/oxygenase activase, chloroplastic OS=Cucumis sativus PE=2 SV=1	RCA_CUCSA	45681.2	7.57	9	331	100	299	100

Peptide Information

Calc. Mass	Obsrv. Mass	± da	± ppm	Start Seq.	End Seq.	Sequence	Ion Score	C. I. %	Modification	Rank	Result Type
809.5243	809.4567	-0.0676	-84	138	144	LTVHIVK					Mascot
895.4155	895.3469	-0.0686	-77	349	355	VYDDEVR					Mascot
940.4675	940.389	-0.0785	-83	300	306	FYWAPTR					Mascot
940.4675	940.389	-0.0785	-83	300	306	FYWAPTR	26	75.909			Mascot
1078.5891	1078.4933	-0.0958	-89	207	215	YREAADIIK					Mascot
1228.603	1228.4926	-0.1104	-90	169	178	SFQCELVFAK			Carbamidomethyl (C)[4]		Mascot
1340.6382	1340.5099	-0.1283	-96	300	309	FYWAPTREDR					Mascot
1882.9697	1882.7939	-0.1758	-93	330	346	LVDTFPGQSIDFFGALR					Mascot
1882.9697	1882.7939	-0.1758	-93	330	346	LVDTFPGQSIDFFGALR	152	100			Mascot
2089.1692	2088.9683	-0.2009	-96	275	293	VPIIVTGNDFSTLYAPLIR	121	100			Mascot
2089.1692	2088.9683	-0.2009	-96	275	293	VPIIVTGNDFSTLYAPLIR					Mascot
2304.0669	2303.8491	-0.2178	-95	179	201	MGINPIMMSAGELESGNAGEPAK					Mascot
2336.0566	2335.821	-0.2356	-101	179	201	MGINPIMMSAGELESGNAGEPAK			Oxidation (M)[1,7]		Mascot

Rank	Protein Name	Accession No.	Protein MW	Protein PI	Pep. Count	Protein Score	Protein Score C. I. %	Total Ion Score	Total Ion C. I. %
10	Ribulose bisphosphate carboxylase/oxygenase activase A, chloroplastic OS=Hordeum vulgare GN=RCAA PE	RCAA_HORVU	51041.4	8.04	9	326	100	298	100

Peptide Information

Calc. Mass	Obsrv. Mass	± da	± ppm	Start Seq.	End Seq.	Sequence	Ion Score	C. I. %	Modification	Rank	Result Type
895.4155	895.3469	-0.0686	-77	342	348	VYDDEVR					Mascot
940.4675	940.389	-0.0785	-83	293	299	FYWAPTR					Mascot
940.4675	940.389	-0.0785	-83	293	299	FYWAPTR	26	75.909			Mascot
1077.5762	1077.4805	-0.0957	-89	138	146	NFMTLPNIK					Mascot
1093.571	1093.4717	-0.0993	-91	138	146	NFMTLPNIK			Oxidation (M)[3]		Mascot
1228.603	1228.4926	-0.1104	-90	162	171	SFQCELVFAK			Carbamidomethyl (C)[4]		Mascot
1326.6226	1326.5073	-0.1153	-87	293	302	FYWAPTRDDR					Mascot
1709.7977	1709.6394	-0.1583	-93	63	77	GLAYDISDDQQDITR	120	100			Mascot
1709.7977	1709.6394	-0.1583	-93	63	77	GLAYDISDDQQDITR					Mascot
1882.9697	1882.7939	-0.1758	-93	323	339	IVDTFPGQSIDFFGALR					Mascot
1882.9697	1882.7939	-0.1758	-93	323	339	IVDTFPGQSIDFFGALR	152	100			Mascot
1894.9141	1894.7916	-0.1225	-65	63	79	GLAYDISDDQQDITRGK					Mascot
2304.0669	2303.8491	-0.2178	-95	172	194	MGINPIMMSAGELESGNAGEPAK					Mascot
2336.0566	2335.821	-0.2356	-101	172	194	MGINPIMMSAGELESGNAGEPAK			Oxidation (M)[1,7]		Mascot
	2335.821										Mascot

Gel Idx/Pos	68/C17	Instr./Gel Origin	BA2060/131017A	Process Sta	Analysis Succeeded
Plate [#] Name	[1] 31496	Instrument Sample Name		Spectra	8

Rank	Protein Name	Accession No.	Protein MW	Protein PI	Pep. Count	Protein Score	Protein Score C. I. %	Total Ion Score	Total Ion C. I. %
1	Ribulose bisphosphate carboxylase/oxygenase activase, chloroplastic OS=Vigna radiata var. radiata G	RCA_VIGRR	47871.3	7.57	13	621	100	551	100

Peptide Information

Calc. Mass	Obsrv. Mass	± da	± ppm	Start Seq.	End Seq.	Sequence	Ion Score	C. I. %	Modification	Rank	Result Type
895.4155	895.3477	-0.0678	-76	354	360	VYDDEVR					Mascot
940.4675	940.3981	-0.0694	-74	305	311	FYWAPTR	36	97.324			Mascot
940.4675	940.3981	-0.0694	-74	305	311	FYWAPTR					Mascot
1015.5781	1015.512	-0.0661	-65	214	223	EAADLIAKGK					Mascot
1077.5762	1077.4945	-0.0817	-76	150	158	NFMTLPNIK					Mascot
1093.571	1093.4827	-0.0883	-81	150	158	NFMTLPNIK			Oxidation (M)[3]		Mascot
1115.5367	1115.4507	-0.086	-77	380	389	EGPPTFDQPK					Mascot
1152.7139	1152.6263	-0.0876	-76	159	169	VPLILGIWGGK					Mascot
1203.574	1203.5422	-0.0318	-26	324	334	TDGVPEEDITK					Mascot
1228.603	1228.5083	-0.0947	-77	174	183	SFQCELVFAK			Carbamidomethyl (C)[4]		Mascot
1623.7617	1623.6337	-0.128	-79	224	238	MCALFINDLDAGAGR	96	100	Carbamidomethyl (C)[2]		Mascot
1623.7617	1623.6337	-0.128	-79	224	238	MCALFINDLDAGAGR			Carbamidomethyl (C)[2]		Mascot
1639.7567	1639.6273	-0.1294	-79	224	238	MCALFINDLDAGAGR			Carbamidomethyl (C)[2], Oxidation (M)[1]		Mascot
1709.7977	1709.6604	-0.1373	-80	77	91	GLAYDISDDQQDITR					Mascot
1709.7977	1709.6604	-0.1373	-80	77	91	GLAYDISDDQQDITR	140	100			Mascot
1882.9697	1882.8173	-0.1524	-81	335	351	LVDTFPGQSIDFFGALR	158	100			Mascot
1882.9697	1882.8173	-0.1524	-81	335	351	LVDTFPGQSIDFFGALR					Mascot
2089.1692	2088.9958	-0.1734	-83	280	298	VPIIVTGNDFSTLYAPLIR	121	100			Mascot
2089.1692	2088.9958	-0.1734	-83	280	298	VPIIVTGNDFSTLYAPLIR					Mascot
2304.0669	2303.8723	-0.1946	-84	184	206	MGINPIMMSAGELESGNAGEPAK					Mascot
2320.0618	2319.8677	-0.1941	-84	184	206	MGINPIMMSAGELESGNAGEPAK			Oxidation (M)[1]		Mascot
2336.0566	2335.8652	-0.1914	-82	184	206	MGINPIMMSAGELESGNAGEPAK			Oxidation (M)[1,7]		Mascot

Rank	Protein Name	Accession No.	Protein MW	Protein PI	Pep. Count	Protein Score	Protein Score C. I. %	Total Ion Score	Total Ion C. I. %
2	Ribulose bisphosphate carboxylase/oxygenase activase, chloroplastic (Fragments) OS=Arachis hypogaea	RCA_ARAHY	15036.4	4.35	7	512	100	456	100

Peptide Information

Calc. Mass	Obsrv. Mass	± da	± ppm	Start Seq.	End Seq.	Sequence	Ion Score	C. I. %	Modification	Rank	Result Type
1022.545	1022.4693	-0.0757	-74	115	123	IGVCTGIFR	37	97.951	Carbamidomethyl (C)[4]		Mascot
1022.545	1022.4693	-0.0757	-74	115	123	IGVCTGIFR			Carbamidomethyl (C)[4]		Mascot
1152.7139	1152.6263	-0.0876	-76	47	57	VPLILGIWGGK					Mascot
1655.7338	1655.6147	-0.1191	-72	81	95	MCCLFINDLDAGAGR			Carbamidomethyl (C)[2]		Mascot
1709.7977	1709.6604	-0.1373	-80	1	15	GLAYDISDDQQDITR					Mascot
1709.7977	1709.6604	-0.1373	-80	1	15	GLAYDISDDQQDITR	140	100			Mascot
1882.9697	1882.8173	-0.1524	-81	124	140	LVDTFPGQSIDFFGALR	158	100			Mascot
1882.9697	1882.8173	-0.1524	-81	124	140	LVDTFPGQSIDFFGALR					Mascot
2089.1692	2088.9958	-0.1734	-83	96	114	VPIIVTGNDFSTLYAPLIR	121	100			Mascot
2089.1692	2088.9958	-0.1734	-83	96	114	VPIIVTGNDFSTLYAPLIR					Mascot
2304.0669	2303.8723	-0.1946	-84	58	80	MGINPIMMSAGELESGNAGEPAK					Mascot
2320.0618	2319.8677	-0.1941	-84	58	80	MGINPIMMSAGELESGNAGEPAK			Oxidation (M)[1]		Mascot
2336.0566	2335.8652	-0.1914	-82	58	80	MGINPIMMSAGELESGNAGEPAK			Oxidation (M)[1,7]		Mascot

Rank	Protein Name	Accession No.	Protein MW	Protein PI	Pep. Count	Protein Score	Protein Score C. I. %	Total Ion Score	Total Ion C. I. %
3	Ribulose bisphosphate carboxylase/oxygenase activase B, chloroplastic OS=Hordeum vulgare GN=RCAB PE	RCAB_HORVU	47197.8	7.59	12	508	100	455	100

Peptide Information

Calc. Mass	Obsrv. Mass	± da	± ppm	Start Seq.	End Seq.	Sequence	Ion Score	C. I. %	Modification	Rank	Result Type
895.4155	895.3477	-0.0678	-76	339	345	VYDDEVR					Mascot
940.4675	940.3981	-0.0694	-74	290	296	FYWAPTR	36	97.324			Mascot
940.4675	940.3981	-0.0694	-74	290	296	FYWAPTR					Mascot
1077.5762	1077.4945	-0.0817	-76	136	144	NFMTLPNIK					Mascot
1093.571	1093.4827	-0.0883	-81	136	144	NFMTLPNIK			Oxidation (M)[3]		Mascot
1115.5367	1115.4507	-0.086	-77	365	374	EGPPTFDQPK					Mascot
1152.7139	1152.6263	-0.0876	-76	145	155	VPLILGIWGGK					Mascot
1214.6012	1214.5328	-0.0684	-56	309	319	TDNVPDEAVVR					Mascot
1228.603	1228.5083	-0.0947	-77	160	169	SFQCELVFAK			Carbamidomethyl (C)[4]		Mascot
1340.6382	1340.5334	-0.1048	-78	290	299	FYWAPTREDR					Mascot
1655.7338	1655.6147	-0.1191	-72	209	223	MCCLFINDLDAGAGR			Carbamidomethyl (C)[2]		Mascot
1709.7977	1709.6604	-0.1373	-80	61	75	GLAYDISDDQQDITR					Mascot
1709.7977	1709.6604	-0.1373	-80	61	75	GLAYDISDDQQDITR	140	100			Mascot
1882.9697	1882.8173	-0.1524	-81	320	336	LVDTFPGQSIDFFGALR	158	100			Mascot
1882.9697	1882.8173	-0.1524	-81	320	336	LVDTFPGQSIDFFGALR					Mascot
2089.1692	2088.9958	-0.1734	-83	265	283	VPIIVTGNDFSTLYAPLIR	121	100			Mascot
2089.1692	2088.9958	-0.1734	-83	265	283	VPIIVTGNDFSTLYAPLIR					Mascot

Rank	Protein Name	Accession No.	Protein MW	Protein PI	Pep. Count	Protein Score	Protein Score C. I. %	Total Ion Score	Total Ion C. I. %
4	Ribulose bisphosphate carboxylase/oxygenase activase 1, chloroplastic OS=Larrea tridentata GN=RCA1	RCA1_LARTR	52108	6.38	10	492	100	455	100

Peptide Information

Calc. Mass	Obsrv. Mass	± da	± ppm	Start Seq.	End Seq.	Sequence	Ion Score	C. I. %	Modification	Rank	Result Type
940.4675	940.3981	-0.0694	-74	303	309	FYWAPTR	36	97.324			Mascot
940.4675	940.3981	-0.0694	-74	303	309	FYWAPTR					Mascot
1045.5425	1045.4818	-0.0607	-58	352	359	VYHDEVRK					Mascot
1115.5367	1115.4507	-0.086	-77	378	387	EGPPSFEQPK					Mascot
1228.603	1228.5083	-0.0947	-77	172	181	SFQCELVFAK			Carbamidomethyl (C)[4]		Mascot
1340.6382	1340.5334	-0.1048	-78	303	312	FYWAPTREDR					Mascot
1655.7338	1655.6147	-0.1191	-72	222	236	MCCLFINDLDAGAGR			Carbamidomethyl (C)[2]		Mascot
1709.7977	1709.6604	-0.1373	-80	73	87	GLAYDISDDQQDITR					Mascot
1709.7977	1709.6604	-0.1373	-80	73	87	GLAYDISDDQQDITR	140	100			Mascot
1882.9697	1882.8173	-0.1524	-81	333	349	LVDTFPGQSIDFFGALR	158	100			Mascot
1882.9697	1882.8173	-0.1524	-81	333	349	LVDTFPGQSIDFFGALR					Mascot
2089.1692	2088.9958	-0.1734	-83	278	296	VPIIVTGNDFSTLYAPLIR	121	100			Mascot
2089.1692	2088.9958	-0.1734	-83	278	296	VPIIVTGNDFSTLYAPLIR					Mascot
2304.0669	2303.8723	-0.1946	-84	182	204	MGINPIMMSAGELESGNAGEPAK					Mascot
2320.0618	2319.8677	-0.1941	-84	182	204	MGINPIMMSAGELESGNAGEPAK			Oxidation (M)[1]		Mascot
2336.0566	2335.8652	-0.1914	-82	182	204	MGINPIMMSAGELESGNAGEPAK			Oxidation (M)[1,7]		Mascot

Rank	Protein Name	Accession No.	Protein MW	Protein PI	Pep. Count	Protein Score	Protein Score C. I. %	Total Ion Score	Total Ion C. I. %
5	Ribulose bisphosphate carboxylase/oxygenase activase, chloroplastic OS=Malus domestica GN=RCA PE=2	RCA_MALDO	48046.2	8.2	12	460	100	409	100

Peptide Information

Calc. Mass	Obsrv. Mass	± da	± ppm	Start Seq.	End Seq.	Sequence	Ion Score	C. I. %	Modification	Rank	Result Type
895.4155	895.3477	-0.0678	-76	352	358	VYDDEVR					Mascot
940.4675	940.3981	-0.0694	-74	303	309	FYWAPTR	36	97.324			Mascot
940.4675	940.3981	-0.0694	-74	303	309	FYWAPTR					Mascot
1152.7139	1152.6263	-0.0876	-76	157	167	VPLILGIWGGK					Mascot
1228.603	1228.5083	-0.0947	-77	172	181	SFQCELVFAK			Carbamidomethyl (C)[4]		Mascot
1340.6382	1340.5334	-0.1048	-78	303	312	FYWAPTREDR					Mascot
1623.7617	1623.6337	-0.128	-79	222	236	MCALFINDLDAGAGR	96	100	Carbamidomethyl (C)[2]		Mascot
1623.7617	1623.6337	-0.128	-79	222	236	MCALFINDLDAGAGR			Carbamidomethyl (C)[2]		Mascot
1639.7567	1639.6273	-0.1294	-79	222	236	MCALFINDLDAGAGR			Carbamidomethyl (C)[2], Oxidation (M)[1]		Mascot
1882.9697	1882.8173	-0.1524	-81	333	349	LVDTFPGQSIDFFGALR	158	100			Mascot
1882.9697	1882.8173	-0.1524	-81	333	349	LVDTFPGQSIDFFGALR					Mascot
1906.9579	1906.8262	-0.1317	-69	393	408	LLEYGNMLVQEQENVK					Mascot
1918.0288	1917.8319	-0.1969	-103	141	156	LVVHITKNFMTLPNMK			Oxidation (M)[10,15]		Mascot
2063.0591	2062.9231	-0.136	-66	393	409	LLEYGNMLVQEQENVKR					Mascot
2088.9939	2088.9958	0.0019	1	184	204	ISPIMMSAGELESGNAGEPAK					Mascot
2089.1692	2088.9958	-0.1734	-83	278	296	VPIIVTGNDFSTLYAPLIR	121	100			Mascot

Rank	Protein Name	Accession No.	Protein MW	Protein PI	Pep. Count	Protein Score	Protein Score C. I. %	Total Ion Score	Total Ion C. I. %
6	Ribulose bisphosphate carboxylase/oxygenase activase A, chloroplastic OS=Hordeum vulgare GN=RCAA PE	RCAA_HORVU	51041.4	8.04	12	385	100	334	100

Peptide Information

Calc. Mass	Obsrv. Mass	± da	± ppm	Start Seq.	End Seq.	Sequence	Ion Score	C. I. %	Modification	Rank	Result Type
895.4155	895.3477	-0.0678	-76	342	348	VYDDEVR					Mascot
940.4675	940.3981	-0.0694	-74	293	299	FYWAPTR	36	97.324			Mascot
940.4675	940.3981	-0.0694	-74	293	299	FYWAPTR					Mascot
1077.5762	1077.4945	-0.0817	-76	138	146	NFMTLPNIK					Mascot
1093.571	1093.4827	-0.0883	-81	138	146	NFMTLPNIK			Oxidation (M)[3]		Mascot
1096.5455	1096.4669	-0.0786	-72	200	208	YREAADMIK					Mascot
1203.6117	1203.5422	-0.0695	-58	27	37	QVTSAVNYHGK					Mascot
1228.603	1228.5083	-0.0947	-77	162	171	SFQCELVFAK			Carbamidomethyl (C)[4]		Mascot
1331.7067	1331.6285	-0.0782	-59	26	37	KQVTSAVNYHGK					Mascot
1505.7706	1505.5614	-0.2092	-139	27	40	QVTSAVNYHGKSSK					Mascot
1655.7338	1655.6147	-0.1191	-72	212	226	MCCLFINDLDAGAGR			Carbamidomethyl (C)[2]		Mascot
1709.7977	1709.6604	-0.1373	-80	63	77	GLAYDISDDQQDITR					Mascot
1709.7977	1709.6604	-0.1373	-80	63	77	GLAYDISDDQQDITR	140	100			Mascot
1882.9697	1882.8173	-0.1524	-81	323	339	IVDTFPGQSIDFFGALR	158	100			Mascot
1882.9697	1882.8173	-0.1524	-81	323	339	IVDTFPGQSIDFFGALR					Mascot
2304.0669	2303.8723	-0.1946	-84	172	194	MGINPIMMSAGELESGNAGEPAK					Mascot
2320.0618	2319.8677	-0.1941	-84	172	194	MGINPIMMSAGELESGNAGEPAK			Oxidation (M)[1]		Mascot
2336.0566	2335.8652	-0.1914	-82	172	194	MGINPIMMSAGELESGNAGEPAK			Oxidation (M)[1,7]		Mascot

Rank	Protein Name	Accession No.	Protein MW	Protein PI	Pep. Count	Protein Score	Protein Score C. I. %	Total Ion Score	Total Ion C. I. %
7	Ribulose bisphosphate carboxylase/oxygenase activase, chloroplastic OS=Zea mays GN=RCA1 PE=2 SV=3	RCA_MAIZE	47908	6.29	14	369	100	297	100

Peptide Information

Calc. Mass	Obsrv. Mass	± da	± ppm	Start Seq.	End Seq.	Sequence	Ion Score	C. I. %	Modification	Rank	Result Type
895.4155	895.3477	-0.0678	-76	348	354	VYDDEVR					Mascot
940.4675	940.3981	-0.0694	-74	299	305	FYWAPTR	36	97.324			Mascot
940.4675	940.3981	-0.0694	-74	299	305	FYWAPTR					Mascot
1077.496	1077.4945	-0.0015	-1	61	68	QTDEDRWK					Mascot
1093.571	1093.4827	-0.0883	-81	144	152	NFMTLPNIK			Oxidation (M)[3]		Mascot
1152.7139	1152.6263	-0.0876	-76	153	163	VPLILGIWGGK					Mascot
1228.603	1228.5083	-0.0947	-77	168	177	SFQCELVFAK			Carbamidomethyl (C)[4]		Mascot
1340.6382	1340.5334	-0.1048	-78	299	308	FYWAPTREDR					Mascot
1373.761	1373.6151	-0.1459	-106	132	143	GFMDKLVVHLSK					Mascot
1639.7567	1639.6273	-0.1294	-79	218	232	MSCLFINDLDAGAGR			Carbamidomethyl (C)[3]		Mascot
1655.7516	1655.6147	-0.1369	-83	218	232	MSCLFINDLDAGAGR			Carbamidomethyl (C)[3], Oxidation (M)[1]		Mascot
1709.7977	1709.6604	-0.1373	-80	69	83	GLAYDISDDQQDITR					Mascot
1709.7977	1709.6604	-0.1373	-80	69	83	GLAYDISDDQQDITR	140	100			Mascot
1847.7615	1847.7405	-0.021	-11	117	131	SYNFDNMMDGFYIAK			Oxidation (M)[7,8]		Mascot
2029.0535	2028.7841	-0.2694	-133	389	405	LLEYGHMLVAEQENVKR					Mascot
2045.0483	2044.9611	-0.0872	-43	389	405	LLEYGHMLVAEQENVKR			Oxidation (M)[7]		Mascot
2089.1692	2088.9958	-0.1734	-83	274	292	VPIIVTGNDFSTLYAPLIR	121	100			Mascot
2089.1692	2088.9958	-0.1734	-83	274	292	VPIIVTGNDFSTLYAPLIR					Mascot
2211.1404	2210.9199	-0.2205	-100	2	24	AAAFSSTVGAPASTPTRSSFLGK					Mascot

Rank	Protein Name	Accession No.	Protein MW	Protein PI	Pep. Count	Protein Score	Protein Score C. I. %	Total Ion Score	Total Ion C. I. %
8	Ribulose bisphosphate carboxylase/oxygenase activase 2, chloroplastic OS=Nicotiana tabacum GN=RCA P	RCA2_TOBAC	48312.5	8.14	9	350	100	315	100

Peptide Information

Calc. Mass	Obsrv. Mass	± da	± ppm	Start Seq.	End Seq.	Sequence	Ion Score	C. I. %	Modification	Rank	Result Type
895.4155	895.3477	-0.0678	-76	356	362	VYDDEVR					Mascot
940.4675	940.3981	-0.0694	-74	307	313	FYWAPTR	36	97.324			Mascot
940.4675	940.3981	-0.0694	-74	307	313	FYWAPTR					Mascot
1214.6263	1214.5328	-0.0935	-77	326	336	TDNVPEEAVIK					Mascot
1340.6382	1340.5334	-0.1048	-78	307	316	FYWAPTREDR					Mascot
1882.9697	1882.8173	-0.1524	-81	337	353	IVDTFPGQSIDFFGALR	158	100			Mascot
1882.9697	1882.8173	-0.1524	-81	337	353	IVDTFPGQSIDFFGALR					Mascot
1906.9579	1906.8262	-0.1317	-69	397	412	LLEYGNMLVQEQENVK					Mascot
2063.0591	2062.9231	-0.136	-66	397	413	LLEYGNMLVQEQENVKR					Mascot
2089.1692	2088.9958	-0.1734	-83	282	300	VPIIVTGNDFSTLYAPLIR	121	100			Mascot
2089.1692	2088.9958	-0.1734	-83	282	300	VPIIVTGNDFSTLYAPLIR					Mascot
2304.0669	2303.8723	-0.1946	-84	186	208	MGINPIMMSAGELESGNAGEPAK					Mascot
2320.0618	2319.8677	-0.1941	-84	186	208	MGINPIMMSAGELESGNAGEPAK			Oxidation (M)[1]		Mascot
2336.0566	2335.8652	-0.1914	-82	186	208	MGINPIMMSAGELESGNAGEPAK			Oxidation (M)[1,7]		Mascot

Rank	Protein Name	Accession No.	Protein MW	Protein PI	Pep. Count	Protein Score	Protein Score C. I. %	Total Ion Score	Total Ion C. I. %
9	Ribulose bisphosphate carboxylase/oxygenase activase, chloroplastic OS=Cucumis sativus PE=2 SV=1	RCA_CUCSA	45681.2	7.57	9	347	100	315	100

Peptide Information

Calc. Mass	Obsrv. Mass	± da	± ppm	Start Seq.	End Seq.	Sequence	Ion Score	C. I. %	Modification	Rank	Result Type
895.4155	895.3477	-0.0678	-76	349	355	VYDDEVR					Mascot
940.4675	940.3981	-0.0694	-74	300	306	FYWAPTR	36	97.324			Mascot
940.4675	940.3981	-0.0694	-74	300	306	FYWAPTR					Mascot
972.5261	972.3838	-0.1423	-146	357	365	WAVGVGVER					Mascot
1228.603	1228.5083	-0.0947	-77	169	178	SFQCELVFAK			Carbamidomethyl (C)[4]		Mascot
1340.6382	1340.5334	-0.1048	-78	300	309	FYWAPTREDR					Mascot
1655.7338	1655.6147	-0.1191	-72	219	233	MCCLFINDLDAGAGR			Carbamidomethyl (C)[2]		Mascot
1882.9697	1882.8173	-0.1524	-81	330	346	LVDTFPGQSIDFFGALR	158	100			Mascot
1882.9697	1882.8173	-0.1524	-81	330	346	LVDTFPGQSIDFFGALR					Mascot
2089.1692	2088.9958	-0.1734	-83	275	293	VPIIVTGNDFSTLYAPLIR	121	100			Mascot
2089.1692	2088.9958	-0.1734	-83	275	293	VPIIVTGNDFSTLYAPLIR					Mascot
2304.0669	2303.8723	-0.1946	-84	179	201	MGINPIMMSAGELESGNAGEPAK					Mascot
2320.0618	2319.8677	-0.1941	-84	179	201	MGINPIMMSAGELESGNAGEPAK			Oxidation (M)[1]		Mascot
2336.0566	2335.8652	-0.1914	-82	179	201	MGINPIMMSAGELESGNAGEPAK			Oxidation (M)[1,7]		Mascot

Rank	Protein Name	Accession No.	Protein MW	Protein PI	Pep. Count	Protein Score	Protein Score C. I. %	Total Ion Score	Total Ion C. I. %
10	Ribulose bisphosphate carboxylase/oxygenase activase 2, chloroplastic OS=Larrea tridentata GN=RCA2	RCA2_LARTR	48023.1	6.78	11	344	100	297	100

Peptide Information

Calc. Mass	Obsrv. Mass	± da	± ppm	Start Seq.	End Seq.	Sequence	Ion Score	C. I. %	Modification	Rank	Result Type
895.4155	895.3477	-0.0678	-76	352	358	VYDDEVR					Mascot
940.4675	940.3981	-0.0694	-74	303	309	FYWAPTR	36	97.324			Mascot
940.4675	940.3981	-0.0694	-74	303	309	FYWAPTR					Mascot
1045.604	1045.4818	-0.1222	-117	148	156	NFLSLPNIK					Mascot
1228.603	1228.5083	-0.0947	-77	172	181	SFQCELVFAK			Carbamidomethyl (C)[4]		Mascot
1340.6382	1340.5334	-0.1048	-78	303	312	FYWAPTREDR					Mascot
1655.7338	1655.6147	-0.1191	-72	222	236	MCCLFINDLDAGAGR			Carbamidomethyl (C)[2]		Mascot
1709.7977	1709.6604	-0.1373	-80	73	87	GLAYDISDDQQDITR					Mascot
1709.7977	1709.6604	-0.1373	-80	73	87	GLAYDISDDQQDITR	140	100			Mascot
1906.9579	1906.8262	-0.1317	-69	393	408	LLQYGNMLVEEQENVK					Mascot
2063.0591	2062.9231	-0.136	-66	393	409	LLQYGNMLVEEQENVKR					Mascot
2089.1692	2088.9958	-0.1734	-83	278	296	VPIIVTGNDFSTLYAPLIR	121	100			Mascot
2089.1692	2088.9958	-0.1734	-83	278	296	VPIIVTGNDFSTLYAPLIR					Mascot
2304.0669	2303.8723	-0.1946	-84	182	204	MGINPIMMSAGELESGNAGEPAK					Mascot
2320.0618	2319.8677	-0.1941	-84	182	204	MGINPIMMSAGELESGNAGEPAK			Oxidation (M)[1]		Mascot
2336.0566	2335.8652	-0.1914	-82	182	204	MGINPIMMSAGELESGNAGEPAK			Oxidation (M)[1,7]		Mascot

Gel Idx/Pos	69/C18	Instr./Gel Origin	BA2060/131017A	Process Sta	Analysis Succeeded
Plate [#] Name	[1] 31496	Instrument Sample Name		Spectra	8

Rank	Protein Name	Accession No.	Protein MW	Protein PI	Pep. Count	Protein Score	Protein Score C. I. %	Total Ion Score	Total Ion C. I. %
1	Phosphoglycerate kinase, chloroplastic OS=Tritic aestivum PE=2 SV=1	PGKH_WHEAT	49808.6	6.58	11	331	100	280	100

Peptide Information

Calc. Mass	Obsrv. Mass	± da	± ppm	Start Seq.	End Seq.	Sequence	Ion Score	C. I. %	Modification	Rank	Result Type
1005.5509	1005.5284	-0.0225	-22	66	75	GTRAVATMAK					Mascot
1022.5516	1022.4673	-0.0843	-82	403	412	FAVGTESIAK					Mascot
1100.6561	1100.5728	-0.0833	-76	278	287	IGVIESLLEK					Mascot
1102.6367	1102.5555	-0.0812	-74	263	273	RPFAAIVGGSK	61	99.991			Mascot
1102.6367	1102.5555	-0.0812	-74	263	273	RPFAAIVGGSK					Mascot
1275.6427	1275.6283	-0.0144	-11	77	89	SVGDLTAADLEGK					Mascot
1501.8835	1501.694	-0.1895	-126	274	287	VSSKIGVIESLLEK					Mascot
1573.8431	1573.7225	-0.1206	-77	421	437	GVTTIIGGGDSVAAVEK					Mascot
1780.0327	1779.8712	-0.1615	-91	177	193	LVADLANGAVLLLENVR					Mascot
1917.9817	1917.8296	-0.1521	-79	209	226	LASLADLFVNDAFGTAHR	87	100			Mascot
1917.9817	1917.8296	-0.1521	-79	209	226	LASLADLFVNDAFGTAHR					Mascot
2028.9469	2028.7855	-0.1614	-80	95	112	ADLNVPLDDNQNITDDTR	132	100			Mascot
2028.9469	2028.7855	-0.1614	-80	95	112	ADLNVPLDDNQNITDDTR					Mascot
2046.0767	2045.9131	-0.1636	-80	208	226	KLASLADLFVNDAFGTAHR					Mascot

Rank	Protein Name	Accession No.	Protein MW	Protein PI	Pep. Count	Protein Score	Protein Score C. I. %	Total Ion Score	Total Ion C. I. %
2	Phosphoglycerate kinase, chloroplastic OS=Nico tabacum PE=2 SV=1	PGKH_TOBAC	50145.9	8.48	14	309	100	233	100

Peptide Information

Calc. Mass	Obsrv. Mass	± da	± ppm	Start Seq.	End Seq.	Sequence	Ion Score	C. I. %	Modification	Rank	Result Type
834.3991	834.3939	-0.0052	-6	204	210	NEPEFAK					Mascot
1015.5934	1015.5208	-0.0726	-71	149	157	YSLAPLVPR					Mascot
1015.5934	1015.5208	-0.0726	-71	149	157	YSLAPLVPR	41	99.074			Mascot
1100.6561	1100.5728	-0.0833	-76	281	290	IGVIESLLEK					Mascot
1102.6367	1102.5555	-0.0812	-74	266	276	RPFAAIVGGSK	61	99.991			Mascot
1102.6367	1102.5555	-0.0812	-74	266	276	RPFAAIVGGSK					Mascot
1198.7405	1198.6501	-0.0904	-75	158	168	LSELLGIQVVK					Mascot
1206.7317	1206.6436	-0.0881	-73	133	143	VILSSHLGRPK					Mascot
1404.7369	1404.6289	-0.108	-77	253	265	ELDYLVGAVSNPK					Mascot
1501.8835	1501.694	-0.1895	-126	277	290	VSSKIGVIESLLEK					Mascot
1525.8835	1525.7612	-0.1223	-80	336	350	GVSLLLPSDVVIADK					Mascot
1573.8431	1573.7225	-0.1206	-77	424	440	GVTTIIGGGDSVAAVEK					Mascot
1933.9766	1933.8214	-0.1552	-80	212	229	LASLADLYVNDAFGTAHR					Mascot
2028.9469	2028.7855	-0.1614	-80	98	115	ADLNVPLDDNQNITDDTR	132	100			Mascot
2028.9469	2028.7855	-0.1614	-80	98	115	ADLNVPLDDNQNITDDTR					Mascot
2062.0715	2061.9036	-0.1679	-81	211	229	KLASLADLYVNDAFGTAHR					Mascot
2299.196	2299.0061	-0.1899	-83	441	464	VGVASVMSHISTGGGASLELLEGK					Mascot
2315.1912	2314.9998	-0.1914	-83	441	464	VGVASVMSHISTGGGASLELLEGK			Oxidation (M)[7]		Mascot

Rank	Protein Name	Accession No.	Protein MW	Protein PI	Pep. Count	Protein Score	Protein Score C. I. %	Total Ion Score	Total Ion C. I. %
3	Phosphoglycerate kinase 2, chloroplastic OS=Arabidopsis thaliana GN=At1g56190 PE=1 SV=3	PGKH2_ARATH	49907.8	8.27	10	236	100	193	100

Peptide Information

Calc. Mass	Obsrv. Mass	± da	± ppm	Start Seq.	End Seq.	Sequence	Ion Score	C. I. %	Modification	Rank	Result Type
1100.6561	1100.5728	-0.0833	-76	279	288	IGVIESLLEK					Mascot
1102.6367	1102.5555	-0.0812	-74	264	274	RPFAAIVGGSK	61	99.991			Mascot
1102.6367	1102.5555	-0.0812	-74	264	274	RPFAAIVGGSK					Mascot
1206.6589	1206.6436	-0.0153	-13	41	51	RLGFSAVVDSR					Mascot
1404.7369	1404.6289	-0.108	-77	251	263	ELDYLVGAVSNPK					Mascot
1416.723	1416.6736	-0.0494	-35	27	40	ASFGHIPSTSVSAR					Mascot
1501.8835	1501.694	-0.1895	-126	275	288	VSSKIGVIESLLEK					Mascot
1573.8431	1573.7225	-0.1206	-77	422	438	GVTTIIGGGDSVAAVEK					Mascot
1933.9766	1933.8214	-0.1552	-80	210	227	LASLADLYVNDAFGTAHR					Mascot
2028.9469	2028.7855	-0.1614	-80	96	113	ADLNVPLDDNQNITDDTR	132	100			Mascot
2028.9469	2028.7855	-0.1614	-80	96	113	ADLNVPLDDNQNITDDTR					Mascot
2062.0715	2061.9036	-0.1679	-81	209	227	KLASLADLYVNDAFGTAHR					Mascot

Rank	Protein Name	Accession No.	Protein MW	Protein PI	Pep. Count	Protein Score	Protein Score C. I. %	Total Ion Score	Total Ion C. I. %
4	Phosphoglycerate kinase, chloroplastic (Fragmen OS=Spinacia oleracea PE=2 SV=2	PGKH_SPIOL	45544.3	5.83	8	92	99.998	61	99.991

Peptide Information

Calc. Mass	Obsrv. Mass	± da	± ppm	Start Seq.	End Seq.	Sequence	Ion Score	C. I. %	Modification	Rank	Result Type
1100.6561	1100.5728	-0.0833	-76	234	243	IGVIESLLEK					Mascot
1102.6367	1102.5555	-0.0812	-74	219	229	RPFAAIVGGSK	61	99.991			Mascot
1102.6367	1102.5555	-0.0812	-74	219	229	RPFAAIVGGSK					Mascot
1198.7405	1198.6501	-0.0904	-75	111	121	LSELLGLQVVK					Mascot
1206.7317	1206.6436	-0.0881	-73	86	96	VILSSHLGRPK					Mascot
1404.7369	1404.6289	-0.108	-77	206	218	ELDYLVGAVSNPK					Mascot
1501.8835	1501.694	-0.1895	-126	230	243	VSSKIGVIESLLEK					Mascot
1933.9766	1933.8214	-0.1552	-80	165	182	LASLADLYVNDAFGTAHR					Mascot
2062.0715	2061.9036	-0.1679	-81	164	182	KLASLADLYVNDAFGTAHR					Mascot

Rank	Protein Name	Accession No.	Protein MW	Protein PI	Pep. Count	Protein Score	Protein Score C. I. %	Total Ion Score	Total Ion C. I. %
5	Phosphoglycerate kinase 1, chloroplastic OS=Arabidopsis thaliana GN=PGK1 PE=1 SV=1	PGKH1_ARATH	50080.7	5.91	8	89	99.996	61	99.991

Peptide Information

Calc. Mass	Obsrv. Mass	± da	± ppm	Start Seq.	End Seq.	Sequence	Ion Score	C. I. %	Modification	Rank	Result Type
1100.6561	1100.5728	-0.0833	-76	282	291	IGVIESLLEK					Mascot
1102.6367	1102.5555	-0.0812	-74	267	277	RPFAAIVGGSK	61	99.991			Mascot
1102.6367	1102.5555	-0.0812	-74	267	277	RPFAAIVGGSK					Mascot
1404.7369	1404.6289	-0.108	-77	254	266	ELDYLVGAVSNPK					Mascot
1501.8835	1501.694	-0.1895	-126	278	291	VSSKIGVIESLLEK					Mascot
1525.8835	1525.7612	-0.1223	-80	337	351	GVSLLLPTDVVVADK					Mascot
1573.8431	1573.7225	-0.1206	-77	425	441	GVTTIIGGGDSVAAVEK					Mascot
1933.9766	1933.8214	-0.1552	-80	213	230	LASLADLYVNDAFGTAHR					Mascot
2062.0715	2061.9036	-0.1679	-81	212	230	KLASLADLYVNDAFGTAHR					Mascot

Rank	Protein Name	Accession No.	Protein MW	Protein PI	Pep. Count	Protein Score	Protein Score C. I. %	Total Ion Score	Total Ion C. I. %
6	B3 domain-containing protein Os07g0183300/Os07g0183600 OS=Oryza sativa subsp. japonica GN=Os07g0183	Y7833_ORYS	84982.1	6.6	12	44	0		

Peptide Information

Calc. Mass	Obsrv. Mass	± da	± ppm	Start Seq.	End Seq.	Sequence	Ion Score	C. I. %	Modification	Rank	Result Type
811.4243	811.3831	-0.0412	-51	535	540	YMGLRR			Oxidation (M)[2]		Mascot
842.5094	842.4505	-0.0589	-70	224	230	TLDKPLR					Mascot
870.4501	870.4766	0.0265	30	714	721	MTPGVPPR	3	0	Oxidation (M)[1]		Mascot
870.4501	870.4766	0.0265	30	714	721	MTPGVPPR			Oxidation (M)[1]		Mascot
1100.5986	1100.5728	-0.0258	-23	148	157	DVFPPLVDAK					Mascot
1382.7638	1382.5997	-0.1641	-119	680	692	NLVNALQLPDGTK					Mascot
1404.6511	1404.6289	-0.0222	-16	19	31	AMWLACAAPNSGR			Carbamidomethyl (C)[6]		Mascot
1571.766	1571.553	-0.213	-136	2	18	AAAAAALADDGDGIVDR					Mascot
1709.8203	1709.6598	-0.1605	-94	123	136	YFVKTLMSSDAEYR					Mascot
1917.8469	1917.8296	-0.0173	-9	194	209	DDMDFVDGDSVIFMRR					Mascot
1917.8469	1917.8296	-0.0173	-9	194	209	DDMDFVDGDSVIFMRR					Mascot
1933.8419	1933.8214	-0.0205	-11	194	209	DDMDFVDGDSVIFMRR			Oxidation (M)[3]		Mascot
2028.9478	2028.7855	-0.1623	-80	517	534	LVTGDNVVFMRSMDSGER			Oxidation (M)[10]		Mascot
2028.9478	2028.7855	-0.1623	-80	517	534	LVTGDNVVFMRSMDSGER			Oxidation (M)[10]		Mascot
2211.0903	2210.9299	-0.1604	-73	591	609	AKFTPGMVVNFVWAVEEDR			Oxidation (M)[7]		Mascot
2225.113	2224.9512	-0.1618	-73	573	592	QDGDEFVVPCGIVHNALRAK			Carbamidomethyl (C)[10]		Mascot

Rank	Protein Name	Accession No.	Protein MW	Protein PI	Pep. Count	Protein Score	Protein Score C. I. %	Total Ion Score	Total Ion C. I. %
7	Subtilisin-chymotrypsin inhibitor CI-1C OS=Horde vulgare PE=1 SV=1	ICIC_HORVU	8252.8	6.78	5	43	0		

Peptide Information

Calc. Mass	Obsrv. Mass	± da	± ppm	Start Seq.	End Seq.	Sequence	Ion Score	C. I. %	Modification	Rank	Result Type
842.5458	842.4505	-0.0953	-113	29	35	AKEIILR					Mascot
933.4999	933.439	-0.0609	-65	67	75	ATTVAZVSR					Mascot
1089.5898	1089.4989	-0.0909	-83	67	77	ATTVAZVSRVG					Mascot
1463.7013	1463.749	0.0477	33	1	15	YPEPTEGSIGASGAK					Mascot
1619.8097	1619.6643	-0.1454	-90	16	30	TSWPEVVGMSAEKAK					Mascot

8	Putative cytochrome c oxidase subunit II PS17 (Fragments) OS=Pinus strobus PE=1 SV=1	PS17_PINST	1707	9.63	2	43	0	20	0

Peptide Information

Calc. Mass	Obsrv. Mass	± da	± ppm	Start Seq.	End Seq.	Sequence	Ion Score	C. I. %	Modification	Rank	Result Type
856.525	856.4641	-0.0609	-71	1	8	SPTVIALR					Mascot
870.5043	870.4766	-0.0277	-32	9	16	VVEALSPR	20	0			Mascot
870.5043	870.4766	-0.0277	-32	9	16	VVEALSPR					Mascot

9	Glyceraldehyde-3-phosphate dehydrogenase A chloroplastic (Fragment) OS=Nicotiana tabacum GN=GAPA P	G3PA_TOBAC	41836.7	6.6	9	41	0

Peptide Information

Calc. Mass	Obsrv. Mass	± da	± ppm	Start Seq.	End Seq.	Sequence	Ion Score	C. I. %	Modification	Rank	Result Type
811.442	811.3831	-0.0589	-73	244	250	LLDASHR					Mascot
833.4627	833.4026	-0.0601	-72	60	67	VAINGFGR					Mascot
1015.5894	1015.5208	-0.0686	-68	272	281	AVALSSQALR					Mascot
1015.5894	1015.5208	-0.0686	-68	272	281	AVALSSQALR					Mascot
1254.6113	1254.5179	-0.0934	-74	309	319	TFAEEVNAAFR					Mascot
1382.7063	1382.5997	-0.1066	-77	308	319	KTFAEEVNAAFR					Mascot
1555.6805	1555.5708	-0.1097	-71	230	243	GTMTTTHSYTGDQR					Mascot
1571.6755	1571.553	-0.1225	-78	230	243	GTMTTTHSYTGDQR			Oxidation (M)[3]		Mascot
1613.8381	1613.7397	-0.0984	-61	81	96	DSPLDVIAINDTGGVK					Mascot
1780.0215	1779.8712	-0.1503	-84	291	307	VPTPNVSVVDLVVQVSK					Mascot
1786.8184	1786.6786	-0.1398	-78	366	379	VIAWYDNEWGYSQR					Mascot

10	Subtilisin-chymotrypsin inhibitor CI-1B OS=Hordeum vulgare PE=1 SV=1	ICIB_HORVU	8957.7	5.33	5	41	0

Peptide Information

Calc. Mass	Obsrv. Mass	± da	± ppm	Start Seq.	End Seq.	Sequence	Ion Score	C. I. %	Modification	Rank	Result Type
834.4025	834.3939	-0.0086	-10	3	10	SMEGSVPK					Mascot
842.5458	842.4505	-0.0953	-113	39	45	AKEIILR					Mascot
1463.7013	1463.749	0.0477	33	11	25	YPEPTEGSIGASGAK					Mascot
1491.7261	1491.5789	-0.1472	-99	26	38	RSWPEVVGMSAEK			Oxidation (M)[9]		Mascot
1619.8024	1619.6643	-0.1381	-85	11	26	YPEPTEGSIGASGAKR					Mascot

Gel Idx/Pos	70/C19	Instr./Gel Origin	BA2060/131017A	Process Sta	Analysis Succeeded
Plate [#] Name	[1] 31496	Instrument Sample Name		Spectra	8

Rank	Protein Name	Accession No	Protein MW	Protein PI	Pep. Count	Protein Score	Protein Score C. I. %	Total Ion Score	Total Ion C. I. %
1	Phosphoglycerate kinase, chloroplastic OS=Tritic aestivum PE=2 SV=1	PGKH_WHEA	49808.6	6.58	11	335	100	283	100

Peptide Information

Calc. Mass	Obsrv. Mass	± da	± ppm	Start Seq.	End Seq.	Sequence	Ion Score	C. I. %	Modification	Rank	Result Type
1100.6561	1100.5819	-0.0742	-67	278	287	IGVIESLLEK					Mascot
1102.6367	1102.5651	-0.0716	-65	263	273	RPFAAIVGGSK	12	0			Mascot
1102.6367	1102.5651	-0.0716	-65	263	273	RPFAAIVGGSK					Mascot
1275.6427	1275.6295	-0.0132	-10	77	89	SVGDLTAADLEGK					Mascot
1501.8835	1501.7001	-0.1834	-122	274	287	VSSKIGVIESLLEK					Mascot
1573.8431	1573.7313	-0.1118	-71	421	437	GVTTIIGGGDSVAAVEK					Mascot
1780.0327	1779.8914	-0.1413	-79	177	193	LVADLANGAVLLLENVR					Mascot
1917.9817	1917.8456	-0.1361	-71	209	226	LASLADLFVNDAFGTAHR					Mascot
1917.9817	1917.8456	-0.1361	-71	209	226	LASLADLFVNDAFGTAHR	129	100			Mascot
2028.9469	2028.8069	-0.14	-69	95	112	ADLNVPLDDNQNITDDTR					Mascot
2028.9469	2028.8069	-0.14	-69	95	112	ADLNVPLDDNQNITDDTR	141	100			Mascot
2046.0767	2045.9303	-0.1464	-72	208	226	KLASLADLFVNDAFGTAHR					Mascot
2849.5447	2849.1362	-0.4085	-143	237	262	FLKPSVAGFLLQKELDYLDGAVSNPK					Mascot
3347.6902	3347.4038	-0.2864	-86	288	318	CDILLLGGGMIFTFYKAQGLSVGSSLVEEDK			Carbamidomethyl (C)[1]		Mascot

Rank	Protein Name	Accession No	Protein MW	Protein PI	Pep. Count	Protein Score	Protein Score C. I. %	Total Ion Score	Total Ion C. I. %
2	Phosphoglycerate kinase, chloroplastic OS=Nico tabacum PE=2 SV=1	PGKH_TOBA	50145.9	8.48	15	287	100	200	100

Peptide Information

Calc. Mass	Obsrv. Mass	± da	± ppm	Start Seq.	End Seq.	Sequence	Ion Score	C. I. %	Modification	Rank	Result Type
1006.5567	1006.4919	-0.0648	-64	406	415	FAVGTEAIAK					Mascot
1015.5934	1015.5285	-0.0649	-64	149	157	YSLAPLVPR					Mascot
1015.5934	1015.5285	-0.0649	-64	149	157	YSLAPLVPR	48	99.83			Mascot
1100.6561	1100.5819	-0.0742	-67	281	290	IGVIESLLEK					Mascot
1102.6367	1102.5651	-0.0716	-65	266	276	RPFAAIVGGSK	12	0			Mascot
1102.6367	1102.5651	-0.0716	-65	266	276	RPFAAIVGGSK					Mascot
1198.7405	1198.6613	-0.0792	-66	158	168	LSELLGIQVVK					Mascot
1206.7317	1206.6528	-0.0789	-65	133	143	VILSSHLGRPK					Mascot
1404.7369	1404.6404	-0.0965	-69	253	265	ELDYLVGAVSNPK					Mascot
1501.8835	1501.7001	-0.1834	-122	277	290	VSSKIGVIESLLEK					Mascot
1525.8835	1525.7784	-0.1051	-69	336	350	GVSLLLPSDVVIADK					Mascot
1573.8431	1573.7313	-0.1118	-71	424	440	GVTTIIGGGDSVAAVEK					Mascot
1933.9766	1933.8408	-0.1358	-70	212	229	LASLADLYVNDAFGTAHR					Mascot
2028.9469	2028.8069	-0.14	-69	98	115	ADLNVPLDDNQNITDDTR					Mascot
2028.9469	2028.8069	-0.14	-69	98	115	ADLNVPLDDNQNITDDTR	141	100			Mascot
2062.0715	2061.9255	-0.146	-71	211	229	KLASLADLYVNDAFGTAHR					Mascot
2299.196	2299.031	-0.165	-72	441	464	VGVASVMSHISTGGGASLELLEGK					Mascot
2315.1912	2315.0259	-0.1653	-71	441	464	VGVASVMSHISTGGGASLELLEGK			Oxidation (M)[7]		Mascot
3347.6902	3347.4038	-0.2864	-86	291	321	CDILLLGGGMIFTFYKAQGLSVGSSLVEEDK			Carbamidomethyl (C)[1]		Mascot

Rank	Protein Name	Accession No	Protein MW	Protein PI	Pep. Count	Protein Score	Protein Score C. I. %	Total Ion Score	Total Ion C. I. %
3	Phosphoglycerate kinase 2, chloroplastic OS=Arabidopsis thaliana GN=At1g56190 PE=1 SV=3	PGKH2_ARAT	49907.8	8.27	10	197	100	154	100

Peptide Information

Calc. Mass	Obsrv. Mass	± da	± ppm	Start Seq.	End Seq.	Sequence	Ion Score	C. I. %	Modification	Rank	Result Type
1100.6561	1100.5819	-0.0742	-67	279	288	IGVIESLLEK					Mascot
1102.6367	1102.5651	-0.0716	-65	264	274	RPFAAIVGGSK	12	0			Mascot
1102.6367	1102.5651	-0.0716	-65	264	274	RPFAAIVGGSK					Mascot
1206.6589	1206.6528	-0.0061	-5	41	51	RLGFSAVVDSR					Mascot
1404.7369	1404.6404	-0.0965	-69	251	263	ELDYLVGAVSNPK					Mascot
1501.8835	1501.7001	-0.1834	-122	275	288	VSSKIGVIESLLEK					Mascot
1573.8431	1573.7313	-0.1118	-71	422	438	GVTTIIGGGDSVAAVEK					Mascot
1933.9766	1933.8408	-0.1358	-70	210	227	LASLADLYVNDAFGTAHR					Mascot
2028.9469	2028.8069	-0.14	-69	96	113	ADLNVPLDDNQNITDDTR					Mascot
2028.9469	2028.8069	-0.14	-69	96	113	ADLNVPLDDNQNITDDTR	141	100			Mascot
2062.0715	2061.9255	-0.146	-71	209	227	KLASLADLYVNDAFGTAHR					Mascot
3347.6902	3347.4038	-0.2864	-86	289	319	CDILLLGGGMIFTFYKAQGLSVGSSLVEEDK			Carbamidomethyl (C)[1]		Mascot

Rank	Protein Name	Accession No	Protein MW	Protein PI	Pep. Count	Protein Score	Protein Score C. I. %	Total Ion Score	Total Ion C. I. %
4	Elongation factor Tu, chloroplastic (Fragment) OS=Derbesia marina GN=tufA PE=3 SV=1	EFTU_DERM	25747.3	4.53	8	51	70.698		

Peptide Information

Calc. Mass	Obsrv. Mass	± da	± ppm	Start Seq.	End Seq.	Sequence	Ion Score	C. I. %	Modification	Rank	Result Type
1045.5748	1045.4995	-0.0753	-72	146	155	GTVATGRVER					Mascot
1100.6058	1100.5819	-0.0239	-22	202	210	GIQKEEIQR					Mascot
1212.6809	1212.6733	-0.0076	-6	225	234	CFKAQVYILK					Mascot
1463.8151	1463.7651	-0.05	-34	211	224	GMVLAKPGSITPHR					Mascot
1463.8151	1463.7651	-0.05	-34	211	224	GMVLAKPGSITPHR					Mascot
1501.8108	1501.7001	-0.1107	-74	156	170	GSVDVGETVELVGLK					Mascot
1882.983	1882.8342	-0.1488	-79	174	189	ETIITGLEMFQKTLDK			Oxidation (M)[9]		Mascot
2705.2942	2704.9907	-0.3035	-112	2	28	NMITGAAQMDGAILVVSGADGPMPQTK			Oxidation (M)[2,9]		Mascot
2721.2891	2721.0786	-0.2105	-77	2	28	NMITGAAQMDGAILVVSGADGPMPQTK			Oxidation (M)[2,9,23]		Mascot
2833.3892	2833.1426	-0.2466	-87	1	28	KNMITGAAQMDGAILVVSGADGPMPQTK			Oxidation (M)[3,10]		Mascot
2849.384	2849.1362	-0.2478	-87	1	28	KNMITGAAQMDGAILVVSGADGPMPQTK			Oxidation (M)[3,10,24]		Mascot

Rank	Protein Name	Accession No	Protein MW	Protein PI	Pep. Count	Protein Score	Protein Score C. I. %	Total Ion Score	Total Ion C. I. %
5	Phosphoglycerate kinase, chloroplastic (Fragmen OS=Spinacia oleracea PE=2 SV=2	PGKH_SPIOL	45544.3	5.83	9	50	67.123	12	0

Peptide Information

Calc. Mass	Obsrv. Mass	± da	± ppm	Start Seq.	End Seq.	Sequence	Ion Score	C. I. %	Modification	Rank	Result Type
1100.6561	1100.5819	-0.0742	-67	234	243	IGVIESLLEK					Mascot
1102.6367	1102.5651	-0.0716	-65	219	229	RPFAAIVGGSK	12	0			Mascot
1102.6367	1102.5651	-0.0716	-65	219	229	RPFAAIVGGSK					Mascot
1198.7405	1198.6613	-0.0792	-66	111	121	LSELLGLQVVK					Mascot
1206.7317	1206.6528	-0.0789	-65	86	96	VILSSHLGRPK					Mascot
1404.7369	1404.6404	-0.0965	-69	206	218	ELDYLVGAVSNPK					Mascot
1501.8835	1501.7001	-0.1834	-122	230	243	VSSKIGVIESLLEK					Mascot
1933.9766	1933.8408	-0.1358	-70	165	182	LASLADLYVNDAFGTAHR					Mascot
2062.0715	2061.9255	-0.146	-71	164	182	KLASLADLYVNDAFGTAHR					Mascot
2313.1755	2313.0625	-0.113	-49	394	417	VGVAEAMSHISTGGGASLELLEGK					Mascot

Rank	Protein Name	Accession No	Protein MW	Protein PI	Pep. Count	Protein Score	Protein Score C. I. %	Total Ion Score	Total Ion C. I. %
6	Phosphoglycerate kinase 1, chloroplastic OS=Arabidopsis thaliana GN=PGK1 PE=1 SV=1	PGKH1_ARAT	50080.7	5.91	9	48	45.437	12	0

Peptide Information

Calc. Mass	Obsrv. Mass	± da	± ppm	Start Seq.	End Seq.	Sequence	Ion Score	C. I. %	Modification	Rank	Result Type
1100.6561	1100.5819	-0.0742	-67	282	291	IGVIESLLEK					Mascot
1102.6367	1102.5651	-0.0716	-65	267	277	RPFAAIVGGSK	12	0			Mascot
1102.6367	1102.5651	-0.0716	-65	267	277	RPFAAIVGGSK					Mascot
1404.7369	1404.6404	-0.0965	-69	254	266	ELDYLVGAVSNPK					Mascot
1501.8835	1501.7001	-0.1834	-122	278	291	VSSKIGVIESLLEK					Mascot
1525.8835	1525.7784	-0.1051	-69	337	351	GVSLLLPTDVVVADK					Mascot
1573.8431	1573.7313	-0.1118	-71	425	441	GVTTIIGGGDSVAAVEK					Mascot
1933.9766	1933.8408	-0.1358	-70	213	230	LASLADLYVNDAFGTAHR					Mascot
2062.0715	2061.9255	-0.146	-71	212	230	KLASLADLYVNDAFGTAHR					Mascot
3347.6902	3347.4038	-0.2864	-86	292	322	CDILLLGGGMIFTFYKAQGLSVGSSLVEEDK			Carbamidomethyl (C)[1]		Mascot

Rank	Protein Name	Accession No	Protein MW	Protein PI	Pep. Count	Protein Score	Protein Score C. I. %	Total Ion Score	Total Ion C. I. %
7	Lipoyl synthase, mitochondrial OS=Arabidopsis t GN=LIP1 PE=2 SV=1	LIAS_ARATH	41317.8	8.61	10	43	0		

Peptide Information

Calc. Mass	Obsrv. Mass	± da	± ppm	Start Seq.	End Seq.	Sequence	Ion Score	C. I. %	Modification	Rank	Result Type
1102.6116	1102.5651	-0.0465	-42	2	10	HSRSALLYR					Mascot
1102.6116	1102.5651	-0.0465	-42	2	10	HSRSALLYR					Mascot
1404.6763	1404.6404	-0.0359	-26	329	339	YRLLGMEMGFR			Oxidation (M)[6,8]		Mascot
1475.7853	1475.6594	-0.1259	-85	79	91	ESIPGGERYVQIK					Mascot
1882.9215	1882.8342	-0.0873	-46	18	35	CFSSSSAVTPVTVTQSPK			Carbamidomethyl (C)[1]		Mascot
1917.9276	1917.8456	-0.082	-43	313	328	RHMPVAEYVTPDAFER					Mascot
1918.0507	1917.8456	-0.2051	-107	199	214	FLKPEMLIEALVPDFR					Mascot
1933.9225	1933.8408	-0.0817	-42	313	328	RHMPVAEYVTPDAFER			Oxidation (M)[3]		Mascot
2045.9895	2045.9303	-0.0592	-29	296	313	AAGVDVMTFGQYMRPSKR			Oxidation (M)[7,13]		Mascot
2061.9917	2061.9255	-0.0662	-32	331	348	LLGMEMGFRYVASGPMVR			Oxidation (M)[4,6,16]		Mascot
2807.3845	2807.1116	-0.2729	-97	42	67	ARLANESPSLTDFIHGDTYSVEVGTK					Mascot
3347.458	3347.4038	-0.0542	-16	108	139	CPNLGECWSGGETGTATATIMILGDTCTRGCR			Carbamidomethyl (C)[1], Oxidation (M)[21]		Mascot

Rank	Protein Name	Accession No	Protein MW	Protein PI	Pep. Count	Protein Score	Protein Score C. I. %	Total Ion Score	Total Ion C. I. %
8	S-adenosylmethionine synthase 2 OS=Solanum tuberosum GN=METK2 PE=2 SV=1	METK2_SOLT	42674.6	5.67	8	43	0	8	0

Peptide Information

Calc. Mass	Obsrv. Mass	± da	± ppm	Start Seq.	End Seq.	Sequence	Ion Score	C. I. %	Modification	Rank	Result Type
1015.6006	1015.5285	-0.0721	-71	292	301	SVVASGLARR					Mascot
1015.6006	1015.5285	-0.0721	-71	292	301	SVVASGLARR					Mascot
1102.531	1102.5651	0.0341	31	178	187	NDNGAMVPIR	8	0	Oxidation (M)[6]		Mascot
1102.531	1102.5651	0.0341	31	178	187	NDNGAMVPIR			Oxidation (M)[6]		Mascot
1206.6477	1206.6528	0.0051	4	61	70	ANVDYEKIVR					Mascot
1525.9199	1525.7784	-0.1415	-93	325	338	TGTIPDKDILVLIK					Mascot
1917.9956	1917.8456	-0.15	-78	374	389	DDPDFTWETVKVLKPK					Mascot
1917.9956	1917.8456	-0.15	-78	374	389	DDPDFTWETVKVLKPK					Mascot
2299.033	2299.031	-0.002	-1	71	91	DTCREIGFISADVGLDADNCK			Carbamidomethyl (C)[3]		Mascot
2313.0571	2313.0625	0.0054	2	365	384	TAAYGHFGRDDPDFTWETVK					Mascot
2705.374	2704.9907	-0.3833	-142	188	211	VHTVLISTQHDETVTNDQIAQDLK					Mascot

Rank	Protein Name	Accession No	Protein MW	Protein PI	Pep. Count	Protein Score	Protein Score C. I. %	Total Ion Score	Total Ion C. I. %
9	U-box domain-containing protein 18 OS=Arabidop thaliana GN=PUB18 PE=2 SV=1	PUB18_ARAT	76946.8	8.91	12	39	0		

Peptide Information

Calc. Mass	Obsrv. Mass	± da	± ppm	Start Seq.	End Seq.	Sequence	Ion Score	C. I. %	Modification	Rank	Result Type
1089.5146	1089.509	-0.0056	-5	672	679	MIHEFQER					Mascot
1100.5695	1100.5819	0.0124	11	681	690	TGSVEPNLQR					Mascot
1198.6942	1198.6613	-0.0329	-27	94	103	EIHVGFQKLK					Mascot
1501.8043	1501.7001	-0.1042	-69	446	459	IQENAMAGILNLSK					Mascot
1525.8268	1525.7784	-0.0484	-32	354	367	HCKTNGIVLAGISR			Carbamidomethyl (C)[2]		Mascot
1763.8844	1763.6919	-0.1925	-109	567	584	SGEISGGLTADCLATLAK			Carbamidomethyl (C)[12]		Mascot
1779.7645	1779.8914	0.1269	71	116	130	LCMMMNSDQVSDHLR					Mascot
2210.9409	2210.9485	0.0076	3	112	130	DGARLCMMMNSDQVSDHLR			Oxidation (M)[7,8]		Mascot
2224.0083	2223.9688	-0.0395	-18	239	257	SSDQVELLSSLMGFICYCR			Carbamidomethyl (C)[16], Oxidation (M)[12]		Mascot
2230.1218	2230.0525	-0.0693	-31	533	551	RSALLAVMGLLMQSDNHWR			Oxidation (M)[8,12]		Mascot
2297.0505	2297.042	-0.0085	-4	116	134	LCMMMNSDQVSDHLRVLTR			Oxidation (M)[3,4,5]		Mascot
2313.2158	2313.0625	-0.1533	-66	388	407	LIAKFLTSELINGGEEMIYR			Oxidation (M)[17]		Mascot

Rank	Protein Name	Accession No	Protein MW	Protein PI	Pep. Count	Protein Score	Protein Score C. I. %	Total Ion Score	Total Ion C. I. %
10	Probable sucrose-phosphatase 3a OS=Arabidops thaliana GN=SPP3A PE=2 SV=2	SPP3A_ARAT	48613.2	5.06	9	39	0		

Peptide Information

Calc. Mass	Obsrv. Mass	± da	± ppm	Start Seq.	End Seq.	Sequence	Ion Score	C. I. %	Modification	Rank	Result Type
1006.4774	1006.4919	0.0145	14	299	306	WRCGEVEK					Mascot
1045.5775	1045.4995	-0.078	-75	107	115	DIVVEETLK					Mascot
1100.5847	1100.5819	-0.0028	-3	261	270	FNLGPNVSPR					Mascot
1173.6262	1173.4917	-0.1345	-115	374	383	FVKHELSEGK					Mascot
1198.6466	1198.6613	0.0147	12	116	125	FPKLEPQPDK					Mascot
1501.8009	1501.7001	-0.1008	-67	104	115	WNRDIVVEETLK					Mascot
2046.0007	2045.9303	-0.0704	-34	243	260	IFHASERCGAGMIEAIQR			Carbamidomethyl (C)[8]		Mascot
2061.9956	2061.9255	-0.0701	-34	243	260	IFHASERCGAGMIEAIQR			Carbamidomethyl (C)[8], Oxidation (M)[12]		Mascot
2230.1218	2230.0525	-0.0693	-31	250	270	CGAGMIEAIQRFNLGPNVSPR					Mascot
2721.3401	2721.0786	-0.2615	-96	10	33	LILVADLDCTLVDHDDPENNDLLR					Mascot

Gel Idx/Pos	71/C20	Instr./Gel Origin	BA2060/131017A	Process Sta	Analysis Succeeded
Plate [#] Name	[1] 31496	Instrument Sample Name		Spectra	11

Rank	Protein Name	Accession No	Protein MW	Protein PI	Pep. Count	Protein Score	Protein Score C. I. %	Total Ion Score	Total Ion C. I. %
1	Photosystem II stability/assembly factor HCF136, chloroplastic OS=Arabidopsis thaliana GN=HCF136 PE	P2SAF_ARAT	44076.4	6.79	15	288	100	192	100

Peptide Information

Calc. Mass	Obsrv. Mass	± da	± ppm	Start Seq.	End Seq.	Sequence	Ion Score	C. I. %	Modification	Rank	Result Type
842.4406	842.4651	0.0245	29	147	153	FNSISFK					Mascot
904.4457	904.4009	-0.0448	-50	291	297	IQNMGWR					Mascot
920.4406	920.3965	-0.0441	-48	291	297	IQNMGWR			Oxidation (M)[4]		Mascot
1060.5469	1060.4924	-0.0545	-51	290	297	RIQNMGWR					Mascot
1076.5419	1076.4736	-0.0683	-63	290	297	RIQNMGWR			Oxidation (M)[5]		Mascot
1096.5785	1096.519	-0.0595	-54	332	341	GFGILDVGYR					Mascot
1099.6259	1099.5626	-0.0633	-58	298	307	ADGGLWLLVR	27	74.772			Mascot
1099.6259	1099.5626	-0.0633	-58	298	307	ADGGLWLLVR					Mascot
1143.6382	1143.5203	-0.1179	-103	21	29	FLSQRISHR					Mascot
1259.7106	1259.6407	-0.0699	-55	389	400	GFVLGNDGVLLR					Mascot
1259.7106	1259.6407	-0.0699	-55	389	400	GFVLGNDGVLLR	28	76.726			Mascot
1387.8055	1387.7268	-0.0787	-57	388	400	KGFVLGNDGVLLR					Mascot
1387.8055	1387.7268	-0.0787	-57	388	400	KGFVLGNDGVLLR	26	67.5			Mascot
1644.903	1644.7616	-0.1414	-86	179	193	IPLSSQLPGDMVFIK					Mascot
1645.818	1645.7845	-0.0335	-20	342	357	SEEEAWAAGGSGILLR					Mascot
1777.8602	1777.7531	-0.1071	-60	316	331	GTGITEEFEEVPVQSR	110	100			Mascot
1777.8602	1777.7531	-0.1071	-60	316	331	GTGITEEFEEVPVQSR					Mascot
1842.8538	1842.7423	-0.1115	-61	199	215	SAEMVTDEGAIYVTSNR					Mascot
2225.1458	2224.9695	-0.1763	-79	1	20	MASLQLCDGYLLFKPSVSPR					Mascot
2374.1152	2373.9565	-0.1587	-67	267	285	GNFFLTWEPGQPYWQPHNR					Mascot
2374.1152	2373.9565	-0.1587	-67	267	285	GNFFLTWEPGQPYWQPHNR					Mascot
2574.3047	2574.0046	-0.3001	-117	30	55	LIPKASSSPPPSPSPSSSSSLSFSR					Mascot

Rank	Protein Name	Accession No	Protein MW	Protein PI	Pep. Count	Protein Score	Protein Score C. I. %	Total Ion Score	Total Ion C. I. %
2	Photosystem II stability/assembly factor HCF136, chloroplastic OS=Oryza sativa subsp. japonica GN=H	P2SAF_ORYS	45441.1	9.02	11	237	100	180	100

Peptide Information

Calc. Mass	Obsrv. Mass	± da	± ppm	Start Seq.	End Seq.	Sequence	Ion Score	C. I. %	Modification	Rank	Result Type
856.5185	856.4827	-0.0358	-42	22	28	CRLLVPR					Mascot
904.4457	904.4009	-0.0448	-50	282	288	IQNMGWR					Mascot
920.4406	920.3965	-0.0441	-48	282	288	IQNMGWR			Oxidation (M)[4]		Mascot
1060.5469	1060.4924	-0.0545	-51	281	288	RIQNMGWR					Mascot
1076.5419	1076.4736	-0.0683	-63	281	288	RIQNMGWR			Oxidation (M)[5]		Mascot
1096.5785	1096.519	-0.0595	-54	345	354	GFGILDVGYR					Mascot
1099.6259	1099.5626	-0.0633	-58	289	298	ADGGLWLLVR	27	74.772			Mascot
1099.6259	1099.5626	-0.0633	-58	289	298	ADGGLWLLVR					Mascot
1275.7056	1275.6511	-0.0545	-43	402	413	GYVLGNDGVLLR					Mascot
1359.7227	1359.6534	-0.0693	-51	212	224	AAVQETVSATLNR					Mascot
1542.6707	1542.5764	-0.0943	-61	125	137	SIPSAEDEDFNYR	64	99.995			Mascot
1542.6707	1542.5764	-0.0943	-61	125	137	SIPSAEDEDFNYR					Mascot
2104.1331	2103.8337	-0.2994	-142	1	19	MATTASLHLHLHLLLSSSR			Oxidation (M)[1]		Mascot
2182.041	2181.9041	-0.1369	-63	225	245	TVSSGISGASYYTGTFNTVNR					Mascot
2182.041	2181.9041	-0.1369	-63	225	245	TVSSGISGASYYTGTFNTVNR	70	99.999			Mascot
2374.1152	2373.9565	-0.1587	-67	258	276	GNFYLTWEPGQPFWQPHNR					Mascot
2374.1152	2373.9565	-0.1587	-67	258	276	GNFYLTWEPGQPFWQPHNR	19	0			Mascot

Rank	Protein Name	Accession No	Protein MW	Protein PI	Pep. Count	Protein Score	Protein Score C. I. %	Total Ion Score	Total Ion C. I. %
3	Photosystem II stability/assembly factor HCF136, chloroplastic (Fragment) OS=Populus euphratica PE=	P2SAF_POPE	2854.4	4.25	2	130	100	110	100

Peptide Information

Calc. Mass	Obsrv. Mass	± da	± ppm	Start Seq.	End Seq.	Sequence	Ion Score	C. I. %	Modification	Rank	Result Type
1096.5785	1096.519	-0.0595	-54	17	26	GFGILDVGYR					Mascot
1777.8602	1777.7531	-0.1071	-60	1	16	GTGITEEFEEVPVQSR	110	100			Mascot
1777.8602	1777.7531	-0.1071	-60	1	16	GTGITEEFEEVPVQSR					Mascot

Rank	Protein Name	Accession No	Protein MW	Protein PI	Pep. Count	Protein Score	Protein Score C. I. %	Total Ion Score	Total Ion C. I. %
4	DNA-directed RNA polymerase subunit beta" OS=Welwitschia mirabilis GN=rpoC2 PE=3 SV=1	RPOC2_WELI	149403.9	9.6	19	54	86.606		

Peptide Information

Calc. Mass	Obsrv. Mass	± da	± ppm	Start Seq.	End Seq.	Sequence	Ion Score	C. I. %	Modification	Rank	Result Type
973.5061	973.3962	-0.1099	-113	909	916	KNDNIQNK					Mascot
1099.5895	1099.5626	-0.0269	-24	1268	1276	KTNYSLFAR					Mascot
1099.5895	1099.5626	-0.0269	-24	1268	1276	KTNYSLFAR					Mascot
1116.587	1116.4694	-0.1176	-105	759	767	VPFCPDLLR			Carbamidomethyl (C)[4]		Mascot
1259.6995	1259.6407	-0.0588	-47	745	755	TAVEYKIPNPK					Mascot
1259.6995	1259.6407	-0.0588	-47	745	755	TAVEYKIPNPK					Mascot
1275.6315	1275.6511	0.0196	15	532	541	LEEEKLEEEK					Mascot
1387.7515	1387.7268	-0.0247	-18	590	600	NYPNCILKIPR			Carbamidomethyl (C)[5]		Mascot
1387.7515	1387.7268	-0.0247	-18	590	600	NYPNCILKIPR			Carbamidomethyl (C)[5]		Mascot
1465.7533	1465.6769	-0.0764	-52	496	508	DQDQVTIAFLSTK					Mascot
1542.7191	1542.5764	-0.1427	-92	295	306	SLSWICQLCYGR			Carbamidomethyl (C)[6,9]		Mascot
1542.7191	1542.5764	-0.1427	-92	295	306	SLSWICQLCYGR			Carbamidomethyl (C)[6,9]		Mascot
1603.7607	1603.7144	-0.0463	-29	889	901	NMFLILSTSDCFR			Carbamidomethyl (C)[11]		Mascot
1603.7607	1603.7144	-0.0463	-29	889	901	NMFLILSTSDCFR			Carbamidomethyl (C)[11]		Mascot
1619.7158	1619.6946	-0.0212	-13	607	619	NTTFCIWNDSQYK					Mascot
1764.9238	1764.9136	-0.0102	-6	188	204	GVVDTAVRTADAGYLTR					Mascot
1818.0306	1817.818	-0.2126	-117	279	294	NLLIQAISIRTPFTCK					Mascot
1821.8953	1821.8643	-0.031	-17	99	112	QLMERWHTTSEYLK					Mascot
2178.0869	2177.908	-0.1789	-82	26	43	LIDRFGMMCTSHILDQLK			Carbamidomethyl (C)[9]		Mascot
2239.1611	2238.9976	-0.1635	-73	85	103	QNLSGNLHTVETLRQLMER					Mascot
2374.1033	2373.9565	-0.1468	-62	601	619	SGFLHRNTTFCIWNDSQYK			Carbamidomethyl (C)[11]		Mascot
2374.1033	2373.9565	-0.1468	-62	601	619	SGFLHRNTTFCIWNDSQYK			Carbamidomethyl (C)[11]		Mascot
2402.333	2401.9824	-0.3506	-146	1171	1192	VLVSDDVIFNVFLPGELIGLSR					Mascot
3022.342	3022.272	-0.07	-23	113	137	EEMYPKFQLTNSFNPVYMMSFSGAR			Oxidation (M)[3,18,19]		Mascot
3339.7007	3339.5002	-0.2005	-60	407	434	SWVFVQNHQYVESEQLIGEFRTTTSLLK					Mascot

Rank	Protein Name	Accession No	Protein MW	Protein PI	Pep. Count	Protein Score	Protein Score C. I. %	Total Ion Score	Total Ion C. I. %
5	Ribulose bisphosphate carboxylase large chain (Fragment) OS=Prunus domestica GN=rbcL PE=3 SV=1	RBL_PRUDO	50914.6	6.23	10	44	0		

Peptide Information

Calc. Mass	Obsrv. Mass	± da	± ppm	Start Seq.	End Seq.	Sequence	Ion Score	C. I. %	Modification	Rank	Result Type
1116.583	1116.4694	-0.1136	-102	412	421	VALEACVQAR			Carbamidomethyl (C)[6]		Mascot
1245.6433	1245.5632	-0.0801	-64	430	440	EGNEIIREASK					Mascot
1275.7307	1275.6511	-0.0796	-62	330	340	EITLGFVDLLR					Mascot
1465.7216	1465.6769	-0.0447	-30	310	324	MSGGDHIHAGTVVGK					Mascot
1793.8024	1793.761	-0.0414	-23	192	205	DDENVNSQPFMRWR					Mascot
1799.8374	1799.7479	-0.0895	-50	9	22	DYKLTYYTPDYETK					Mascot
1821.9388	1821.8643	-0.0745	-41	307	324	ALRMSGGDHIHAGTVVGK			Oxidation (M)[4]		Mascot
2169.9868	2169.8674	-0.1194	-55	185	203	GGLDFTKDDENVNSQPFMR					Mascot
3022.4548	3022.272	-0.1828	-60	249	275	ELGVPIVMHDYLTGGFTANTSLAHYCR			Carbamidomethyl (C)[26]		Mascot
3854.8718	3854.563	-0.3088	-80	32	69	VTPQPGVPPEEAGAAVAAESSTGTWTTVWTDGLTSLDR					Mascot

Rank	Protein Name	Accession No	Protein MW	Protein PI	Pep. Count	Protein Score	Protein Score C. I. %	Total Ion Score	Total Ion C. I. %
6	Ribulose bisphosphate carboxylase large chain (Fragment) OS=Cornus oblonga GN=rbcL PE=3 SV=2	RBL_COROL	51605.1	6.14	10	44	0		

Peptide Information

Calc. Mass	Obsrv. Mass	± da	± ppm	Start Seq.	End Seq.	Sequence	Ion Score	C. I. %	Modification	Rank	Result Type
1116.583	1116.4694	-0.1136	-102	412	421	VALEACVQAR			Carbamidomethyl (C)[6]		Mascot
1245.6433	1245.5632	-0.0801	-64	430	440	EGNEIIREASK					Mascot
1275.7307	1275.6511	-0.0796	-62	330	340	EITLGFVDLLR					Mascot
1465.7546	1465.6769	-0.0777	-53	137	149	TFQGPPHGIQVER					Mascot
1793.8024	1793.761	-0.0414	-23	192	205	DDENVNSQPFMRWR					Mascot
1821.9388	1821.8643	-0.0745	-41	307	324	ALRMSGGDHIHSGTVVGK					Mascot
2169.9868	2169.8674	-0.1194	-55	185	203	GGLDFTKDDENVNSQPFMR					Mascot
2178.0786	2177.908	-0.1706	-78	208	226	FLFCTEALYKAQSETGEIK					Mascot
3022.4548	3022.272	-0.1828	-60	249	275	ELGVPIVMHDYLTGGFTANTSLAHYCR			Carbamidomethyl (C)[26]		Mascot
3854.8718	3854.563	-0.3088	-80	32	69	VTPQPGVPPEEAGAAVAAESSTGTWTTVWTDGLTSLDR					Mascot

Mascot

Rank	Protein Name	Accession No	Protein MW	Protein PI	Pep. Count	Protein Score	Protein Score C. I. %	Total Ion Score	Total Ion C. I. %
7	Ribulose bisphosphate carboxylase large chain (Fragment) OS=Platytheca verticillata GN=rbcL PE=3 SV	RBL_PLAVR	51588.1	6.04	10	44	0		

Peptide Information

Calc. Mass	Obsrv. Mass	± da	± ppm	Start Seq.	End Seq.	Sequence	Ion Score	C. I. %	Modification	Rank	Result Type
1116.583	1116.4694	-0.1136	-102	412	421	VALEACVQAR			Carbamidomethyl (C)[6]		Mascot
1245.6433	1245.5632	-0.0801	-64	430	440	EGNEIIREASK					Mascot
1275.7307	1275.6511	-0.0796	-62	330	340	EITLGFVDLLR					Mascot
1465.7546	1465.6769	-0.0777	-53	137	149	TFQGPPHGIQVER					Mascot
1793.8024	1793.761	-0.0414	-23	192	205	DDENVNSQPFMRWR					Mascot
1799.8374	1799.7479	-0.0895	-50	9	22	DYKLTYYTPDYETK					Mascot
1821.9388	1821.8643	-0.0745	-41	307	324	ALRMSGGDHIHSGTVVGK					Mascot
2169.9868	2169.8674	-0.1194	-55	185	203	GGLDFTKDDENVNSQPFMR					Mascot
3022.4548	3022.272	-0.1828	-60	249	275	ELGVPIVMHDYLTGGFTANTSLAHYCR			Carbamidomethyl (C)[26]		Mascot
3854.8718	3854.563	-0.3088	-80	32	69	VTPQPGVPPEEAGAAVAAESSTGTWTTVWTDGLTSLDR					Mascot

Rank	Protein Name	Accession No	Protein MW	Protein PI	Pep. Count	Protein Score	Protein Score C. I. %	Total Ion Score	Total Ion C. I. %
8	Ribulose bisphosphate carboxylase large chain (Fragment) OS=Oxalis dillenii GN=rbcL PE=3 SV=2	RBL_OXADI	51653.9	5.91	10	44	0		

Peptide Information

Calc. Mass	Obsrv. Mass	± da	± ppm	Start Seq.	End Seq.	Sequence	Ion Score	C. I. %	Modification	Rank	Result Type
1096.5197	1096.519	-0.0007	-1	458	466	FEFEPVDTI					Mascot
1116.583	1116.4694	-0.1136	-102	413	422	VALEACVQAR			Carbamidomethyl (C)[6]		Mascot
1245.6337	1245.5632	-0.0705	-57	209	218	FLFCAEAIFK			Carbamidomethyl (C)[4]		Mascot
1275.7307	1275.6511	-0.0796	-62	331	341	EITLGFVDLLR					Mascot
1465.7546	1465.6769	-0.0777	-53	138	150	TFQGPPHGIQVER					Mascot
1793.8024	1793.761	-0.0414	-23	193	206	DDENVNSQPFMRWR					Mascot
1821.9388	1821.8643	-0.0745	-41	308	325	ALRMSGGDHIHSGTVVGK					Mascot
2169.9868	2169.8674	-0.1194	-55	186	204	GGLDFTKDDENVNSQPFMR					Mascot
3022.4548	3022.272	-0.1828	-60	250	276	ELGVPIVMHDYLTGGFTANTSLAHYCR			Carbamidomethyl (C)[26]		Mascot
3854.8718	3854.563	-0.3088	-80	33	70	VTPQPGVPPEEAGAAVAAESSTGTWTTVWTDGLTSLDR					Mascot

Re Result Type

Rank	Protein Name	Accession No	Protein MW	Protein PI	Pep. Count	Protein Score	Protein Score C. I. %	Total Ion Score	Total Ion C. I. %
9	Ribulose bisphosphate carboxylase large chain OS=Phaseolus vulgaris GN=rbcL PE=3 SV=1	RBL_PHAVU	52767.6	5.96	10	44	0		

Peptide Information

Calc. Mass	Obsrv. Mass	± da	± ppm	Start Seq.	End Seq.	Sequence	Ion Score	C. I. %	Modification	Rank	Result Type
1116.583	1116.4694	-0.1136	-102	422	431	VALEACVQAR			Carbamidomethyl (C)[6]		Mascot
1245.6433	1245.5632	-0.0801	-64	440	450	EGNEIIREASK					Mascot
1275.7307	1275.6511	-0.0796	-62	340	350	EITLGFVDLLR					Mascot
1465.7546	1465.6769	-0.0777	-53	147	159	TFQGPPHGIQVER					Mascot
1603.7196	1603.7144	-0.0052	-3	464	476	EIKFEFEAMDTLD			Oxidation (M)[9]		Mascot
1603.7196	1603.7144	-0.0052	-3	464	476	EIKFEFEAMDTLD			Oxidation (M)[9]		Mascot
1793.8024	1793.761	-0.0414	-23	202	215	DDENVNSQPFMRWR					Mascot
1799.8374	1799.7479	-0.0895	-50	19	32	DYKLTYYTPDYETK					Mascot
2169.9868	2169.8674	-0.1194	-55	195	213	GGLDFTKDDENVNSQPFMR					Mascot
3022.4548	3022.272	-0.1828	-60	259	285	ELGVPIVMHDYLTGGFTANTSLAHYCR			Carbamidomethyl (C)[26]		Mascot
3854.8718	3854.563	-0.3088	-80	42	79	VTPQPGVPPEEAGAAVAAESSTGTWTTVWTDGLTSLDR					Mascot

Rank	Protein Name	Accession No	Protein MW	Protein PI	Pep. Count	Protein Score	Protein Score C. I. %	Total Ion Score	Total Ion C. I. %
10	Ribulose bisphosphate carboxylase large chain (Fragment) OS=Amorphophallus titanum GN=rbcL PE=3 SV=	RBL_AMOTI	52548.4	6.32	10	43	0		

Peptide Information

Calc. Mass	Obsrv. Mass	± da	± ppm	Start Seq.	End Seq.	Sequence	Ion Score	C. I. %	Modification	Rank	Result Type
1116.583	1116.4694	-0.1136	-102	416	425	VALEACVQAR			Carbamidomethyl (C)[6]		Mascot
1245.6433	1245.5632	-0.0801	-64	434	444	EGNEIIREASK					Mascot
1465.7216	1465.6769	-0.0447	-30	314	328	MSGGDHIHAGTVVGK					Mascot
1793.8024	1793.761	-0.0414	-23	196	209	DDENVNSQPFMRWR					Mascot
1799.8374	1799.7479	-0.0895	-50	13	26	DYKLTYYTPDYETK					Mascot
1821.9388	1821.8643	-0.0745	-41	311	328	ALRMSGGDHIHAGTVVGK			Oxidation (M)[4]		Mascot
2169.9868	2169.8674	-0.1194	-55	189	207	GGLDFTKDDENVNSQPFMR					Mascot
2211.1191	2210.9651	-0.154	-70	134	153	IPPAYSKTFEGPPHGIQSER					Mascot
3022.4548	3022.272	-0.1828	-60	253	279	ELGVPIVMHDYLTGGFTANTSLAHYCR			Carbamidomethyl (C)[26]		Mascot
3854.8718	3854.563	-0.3088	-80	36	73	VTPQPGVPPEEAGAAVAAESSTGTWTTVWTDGLTSLDR					Mascot

Gel Idx/Pos	72/C21	Instr./Gel Origin	BA2060/131017A	Process Sta	Analysis Succeeded
Plate [#] Name	[1] 31496	Instrument Sample Name		Spectra	11

Rank	Protein Name	Accession No	Protein MW	Protein PI	Pep. Count	Protein Score	Protein Score C. I. %	Total Ion Score	Total Ion C. I. %
1	Photosystem II stability/assembly factor HCF136, chloroplastic OS=Oryza sativa subsp. japonica GN=H	P2SAF_ORYS	45441.1	9.02	10	165	100	118	100

Peptide Information

Calc. Mass	Obsrv. Mass	± da	± ppm	Start Seq.	End Seq.	Sequence	Ion Score	C. I. %	Modification	Rank	Result Type
904.4457	904.4136	-0.0321	-35	282	288	IQNMGWR					Mascot
920.4406	920.3977	-0.0429	-47	282	288	IQNMGWR			Oxidation (M)[4]		Mascot
1096.5785	1096.5211	-0.0574	-52	345	354	GFGILDVGYR					Mascot
1099.6259	1099.5723	-0.0536	-49	289	298	ADGGLWLLVR					Mascot
1099.6259	1099.5723	-0.0536	-49	289	298	ADGGLWLLVR	27	67.489			Mascot
1275.7056	1275.6697	-0.0359	-28	402	413	GYVLGNDGVLLR					Mascot
1349.7059	1349.6449	-0.061	-45	383	395	AADNIAANLYSVK					Mascot
1359.7227	1359.6571	-0.0656	-48	212	224	AAVQETVSATLNR					Mascot
1373.7059	1373.6553	-0.0506	-37	357	370	DEAWAAGGSGVLLK					Mascot
1542.6707	1542.5895	-0.0812	-53	125	137	SIPSAEDEDFNYR					Mascot
1542.6707	1542.5895	-0.0812	-53	125	137	SIPSAEDEDFNYR	53	99.922			Mascot
2182.041	2181.9187	-0.1223	-56	225	245	TVSSGISGASYYTGTFNTVNR					Mascot
2182.041	2181.9187	-0.1223	-56	225	245	TVSSGISGASYYTGTFNTVNR	39	98.063			Mascot
2374.1152	2373.9841	-0.1311	-55	258	276	GNFYLTWEPGQPFWQPHNR					Mascot

Rank	Protein Name	Accession No	Protein MW	Protein PI	Pep. Count	Protein Score	Protein Score C. I. %	Total Ion Score	Total Ion C. I. %
2	Photosystem II stability/assembly factor HCF136, chloroplastic OS=Arabidopsis thaliana GN=HCF136 PE	P2SAF_ARAT	44076.4	6.79	13	127	100	52	99.906

Peptide Information

Calc. Mass	Obsrv. Mass	± da	± ppm	Start Seq.	End Seq.	Sequence	Ion Score	C. I. %	Modification	Rank	Result Type
842.4406	842.4694	0.0288	34	147	153	FNSISFK					Mascot
904.4457	904.4136	-0.0321	-35	291	297	IQNMGWR					Mascot
920.4406	920.3977	-0.0429	-47	291	297	IQNMGWR			Oxidation (M)[4]		Mascot
1096.5785	1096.5211	-0.0574	-52	332	341	GFGILDVGYR					Mascot
1099.6259	1099.5723	-0.0536	-49	298	307	ADGGLWLLVR					Mascot
1099.6259	1099.5723	-0.0536	-49	298	307	ADGGLWLLVR	27	67.489			Mascot
1143.6382	1143.5334	-0.1048	-92	21	29	FLSQRISHR					Mascot
1143.6382	1143.5334	-0.1048	-92	21	29	FLSQRISHR					Mascot
1259.7106	1259.6509	-0.0597	-47	389	400	GFVLGNDGVLLR					Mascot
1373.7383	1373.6553	-0.083	-60	221	233	AAIQETVSATLNR					Mascot
1387.8055	1387.7146	-0.0909	-65	388	400	KGFVLGNDGVLLR					Mascot
1387.8055	1387.7146	-0.0909	-65	388	400	KGFVLGNDGVLLR	1	0			Mascot
1645.818	1645.796	-0.022	-13	342	357	SEEEAWAAGGSGILLR					Mascot
1777.8602	1777.7628	-0.0974	-55	316	331	GTGITEEFEEVPVQSR					Mascot
1777.8602	1777.7628	-0.0974	-55	316	331	GTGITEEFEEVPVQSR	25	49.762			Mascot
1842.8538	1842.7423	-0.1115	-61	199	215	SAEMVTDEGAIYVTSNR					Mascot
2225.1458	2225.0061	-0.1397	-63	1	20	MASLQLCDGYLLFKPSVSPR					Mascot
2374.1152	2373.9841	-0.1311	-55	267	285	GNFFLTWEPGQPYWQPHNR					Mascot

Rank	Protein Name	Accession No	Protein MW	Protein PI	Pep. Count	Protein Score	Protein Score C. I. %	Total Ion Score	Total Ion C. I. %
3	Ribulose bisphosphate carboxylase large chain (Fragment) OS=Geum quellyon GN=rbcL PE=3 SV=1	RBL_GEUQU	50910.8	6.23	12	81	99.972	23	21.239

Peptide Information

Calc. Mass	Obsrv. Mass	± da	± ppm	Start Seq.	End Seq.	Sequence	Ion Score	C. I. %	Modification	Rank	Result Type
898.41	898.3665	-0.0435	-48	296	302	NHGMHFR					Mascot
910.4451	910.395	-0.0501	-55	178	184	AVYECLR			Carbamidomethyl (C)[5]		Mascot
914.4049	914.3558	-0.0491	-54	296	302	NHGMHFR			Oxidation (M)[4]		Mascot
1170.5585	1170.5684	0.0099	8	294	302	QKNHGMHFR			Oxidation (M)[6]		Mascot
1187.6643	1187.6082	-0.0561	-47	276	285	DNGLLLHIHR					Mascot
1245.6337	1245.5763	-0.0574	-46	208	217	FLFCAEALFK			Carbamidomethyl (C)[4]		Mascot
1275.7307	1275.6697	-0.061	-48	330	340	EITLGFVDLLR					Mascot
1413.8101	1413.6248	-0.1853	-131	125	136	LEDLRIPPAYVK					Mascot
1465.7216	1465.6792	-0.0424	-29	310	324	MSGGDHIHAGTVVGK					Mascot
1465.7546	1465.6792	-0.0754	-51	137	149	TFQGPPHGIQVER	24	46.54			Mascot
1793.8024	1793.7761	-0.0263	-15	192	205	DDENVNSQPFMRWR					Mascot
1809.7972	1809.7709	-0.0263	-15	192	205	DDENVNSQPFMRWR			Oxidation (M)[11]		Mascot
1821.9388	1821.8413	-0.0975	-54	307	324	ALRMSGGDHIHAGTVVGK			Oxidation (M)[4]		Mascot
3854.8718	3854.6709	-0.2009	-52	32	69	VTPQPGVPPEEAGAAVAAESSTGTWTTVWTDGLTSLDR					Mascot

Rank	Protein Name	Accession No	Protein MW	Protein PI	Pep. Count	Protein Score	Protein Score C. I. %	Total Ion Score	Total Ion C. I. %
4	Ribulose bisphosphate carboxylase large chain (Fragment) OS=Actinidia chinensis GN=rbcL PE=3 SV=1	RBL_ACTCH	51553	6.23	12	77	99.934	23	21.239

Peptide Information

Calc. Mass	Obsrv. Mass	± da	± ppm	Start Seq.	End Seq.	Sequence	Ion Score	C. I. %	Modification	Rank	Result Type
898.41	898.3665	-0.0435	-48	296	302	NHGMHFR					Mascot
910.4451	910.395	-0.0501	-55	178	184	AVYECLR			Carbamidomethyl (C)[5]		Mascot
914.4049	914.3558	-0.0491	-54	296	302	NHGMHFR			Oxidation (M)[4]		Mascot
962.4788	962.4544	-0.0244	-25	218	226	AQSETGEIK					Mascot
1170.5585	1170.5684	0.0099	8	294	302	QKNHGMHFR			Oxidation (M)[6]		Mascot
1187.6643	1187.6082	-0.0561	-47	276	285	DNGLLLHIHR					Mascot
1245.6337	1245.5763	-0.0574	-46	208	217	FLFCAEAIFK			Carbamidomethyl (C)[4]		Mascot
1387.7943	1387.7146	-0.0797	-57	125	136	LEDLRIPAAYVK					Mascot
1387.7943	1387.7146	-0.0797	-57	125	136	LEDLRIPAAYVK					Mascot
1465.7216	1465.6792	-0.0424	-29	310	324	MSGGDHIHAGTVVGK					Mascot
1465.7546	1465.6792	-0.0754	-51	137	149	TFQGPPHGIQVER	24	46.54			Mascot
1793.8024	1793.7761	-0.0263	-15	192	205	DDENVNSQPFMRWR					Mascot
1809.7972	1809.7709	-0.0263	-15	192	205	DDENVNSQPFMRWR			Oxidation (M)[11]		Mascot
1821.9388	1821.8413	-0.0975	-54	307	324	ALRMSGGDHIHAGTVVGK			Oxidation (M)[4]		Mascot
3854.8718	3854.6709	-0.2009	-52	32	69	VTPQPGVPPEEAGAAVAAESSTGTWTTVWTDGLTSLDR					Mascot

Rank	Protein Name	Accession No	Protein MW	Protein PI	Pep. Count	Protein Score	Protein Score C. I. %	Total Ion Score	Total Ion C. I. %
5	Ribulose bisphosphate carboxylase large chain (Fragment) OS=Apocynum cannabinum GN=rbcL PE=3 SV=1	RBL_APOCA	48701.5	6.03	11	77	99.93	23	21.239

Peptide Information

Calc. Mass	Obsrv. Mass	± da	± ppm	Start Seq.	End Seq.	Sequence	Ion Score	C. I. %	Modification	Rank	Result Type
898.41	898.3665	-0.0435	-48	272	278	NHGMHFR					Mascot
910.4451	910.395	-0.0501	-55	154	160	AVYECLR			Carbamidomethyl (C)[5]		Mascot
914.4049	914.3558	-0.0491	-54	272	278	NHGMHFR			Oxidation (M)[4]		Mascot
962.4788	962.4544	-0.0244	-25	194	202	SQAETGEIK					Mascot
1170.5585	1170.5684	0.0099	8	270	278	QKNHGMHFR			Oxidation (M)[6]		Mascot
1245.6337	1245.5763	-0.0574	-46	184	193	FLFCAEAIFK			Carbamidomethyl (C)[4]		Mascot
1413.8101	1413.6248	-0.1853	-131	101	112	LEDLRIPPAYVK					Mascot
1465.7216	1465.6792	-0.0424	-29	286	300	MSGGDHIHAGTVVGK					Mascot
1465.7546	1465.6792	-0.0754	-51	113	125	TFQGPPHGIQVER	24	46.54			Mascot
1793.8024	1793.7761	-0.0263	-15	168	181	DDENVNSQPFMRWR					Mascot
1809.7972	1809.7709	-0.0263	-15	168	181	DDENVNSQPFMRWR			Oxidation (M)[11]		Mascot
1821.9388	1821.8413	-0.0975	-54	283	300	ALRMSGGDHIHAGTVVGK			Oxidation (M)[4]		Mascot
3854.8718	3854.6709	-0.2009	-52	8	45	VTPQPGVPPEEAGAAVAAESSTGTWTTVWTDGLTSLDR					Mascot

Mascot

Rank	Protein Name	Accession No	Protein MW	Protein PI	Pep. Count	Protein Score	Protein Score C. I. %	Total Ion Score	Total Ion C. I. %
6	Ribulose bisphosphate carboxylase large chain (Fragment) OS=Vitis aestivalis GN=rbcL PE=3 SV=1	RBL_VITAE	51567	6.29	12	76	99.914	23	21.239

Peptide Information

Calc. Mass	Obsrv. Mass	± da	± ppm	Start Seq.	End Seq.	Sequence	Ion Score	C. I. %	Modification	Rank	Result Type
898.41	898.3665	-0.0435	-48	297	303	NHGMHFR					Mascot
910.4451	910.395	-0.0501	-55	179	185	AVYECLR			Carbamidomethyl (C)[5]		Mascot
914.4049	914.3558	-0.0491	-54	297	303	NHGMHFR			Oxidation (M)[4]		Mascot
962.4788	962.4544	-0.0244	-25	219	227	SQAETGEIK					Mascot
1170.5585	1170.5684	0.0099	8	295	303	QKNHGMHFR			Oxidation (M)[6]		Mascot
1187.6378	1187.6082	-0.0296	-25	431	441	EGNEIIRAASK					Mascot
1245.6337	1245.5763	-0.0574	-46	209	218	FLFCAEAIFK			Carbamidomethyl (C)[4]		Mascot
1275.7307	1275.6697	-0.061	-48	331	341	EITLGFVDLLR					Mascot
1465.7216	1465.6792	-0.0424	-29	311	325	MSGGDHIHAGTVVGK					Mascot
1465.7546	1465.6792	-0.0754	-51	138	150	TFQGPPHGIQVER	24	46.54			Mascot
1793.8024	1793.7761	-0.0263	-15	193	206	DDENVNSQPFMRWR					Mascot
1809.7972	1809.7709	-0.0263	-15	193	206	DDENVNSQPFMRWR			Oxidation (M)[11]		Mascot
1821.9388	1821.8413	-0.0975	-54	308	325	ALRMSGGDHIHAGTVVGK			Oxidation (M)[4]		Mascot
3854.8718	3854.6709	-0.2009	-52	33	70	VTPQPGVPPEEAGAAVAAESSTGTWTTVWTDGLTSLDR					Mascot

Rank	Protein Name	Accession No	Protein MW	Protein PI	Pep. Count	Protein Score	Protein Score C. I. %	Total Ion Score	Total Ion C. I. %
7	Ribulose bisphosphate carboxylase large chain (Fragment) OS=Polygala cruciata GN=rbcL PE=3 SV=2	RBL_POLCR	51580.1	6.23	11	76	99.912	24	46.54

Peptide Information

Calc. Mass	Obsrv. Mass	± da	± ppm	Start Seq.	End Seq.	Sequence	Ion Score	C. I. %	Modification	Rank	Result Type
860.4658	860.4363	-0.0295	-34	412	419	VALEACVR					Mascot
898.41	898.3665	-0.0435	-48	296	302	NHGMHFR					Mascot
910.4451	910.395	-0.0501	-55	178	184	AVYECLR			Carbamidomethyl (C)[5]		Mascot
914.4049	914.3558	-0.0491	-54	296	302	NHGMHFR			Oxidation (M)[4]		Mascot
1088.4604	1088.5513	0.0909	84	457	465	FDFEAMDTL					Mascot
1170.5585	1170.5684	0.0099	8	294	302	QKNHGMHFR			Oxidation (M)[6]		Mascot
1187.6643	1187.6082	-0.0561	-47	276	285	DNGLLLHIHR					Mascot
1245.6433	1245.5763	-0.067	-54	430	440	EGNEIIREASK					Mascot
1259.7722	1259.6509	-0.1213	-96	330	340	EITLGFVVLLR					Mascot
1465.7546	1465.6792	-0.0754	-51	137	149	TFQGPPHGIQVER					Mascot
1465.7546	1465.6792	-0.0754	-51	137	149	TFQGPPHGIQVER	24	46.54			Mascot
1793.8024	1793.7761	-0.0263	-15	192	205	DDENVNSQPFMRWR					Mascot
1809.7972	1809.7709	-0.0263	-15	192	205	DDENVNSQPFMRWR			Oxidation (M)[11]		Mascot
3854.8718	3854.6709	-0.2009	-52	32	69	VTPQPGVPPEEAGAAVAAESSTGTWTTVWTDGLTSLDR					Mascot

Rank	Protein Name	Accession No	Protein MW	Protein PI	Pep. Count	Protein Score	Protein Score C. I. %	Total Ion Score	Total Ion C. I. %
8	Ribulose bisphosphate carboxylase large chain (Fragment) OS=Sanguinaria canadensis GN=rbcL PE=3 SV=	RBL_SANCA	48270.4	6.46	11	76	99.907	23	21.239

Peptide Information

Calc. Mass	Obsrv. Mass	± da	± ppm	Start Seq.	End Seq.	Sequence	Ion Score	C. I. %	Modification	Rank	Result Type
898.41	898.3665	-0.0435	-48	296	302	NHGMHFR					Mascot
910.4451	910.395	-0.0501	-55	178	184	AVYECLR			Carbamidomethyl (C)[5]		Mascot
914.4049	914.3558	-0.0491	-54	296	302	NHGMHFR			Oxidation (M)[4]		Mascot
1170.5585	1170.5684	0.0099	8	294	302	QKNHGMHFR			Oxidation (M)[6]		Mascot
1187.6643	1187.6082	-0.0561	-47	276	285	DNGLLLHIHR					Mascot

Calc. Mass	Obsrv. Mass	± da	± ppm	Start Seq.	End Seq.	Sequence	Ion Score	C. I. %	Modification	Rank	Result Type
1275.7307	1275.6697	-0.061	-48	330	340	EITLGFVDLLR					Mascot
1413.8101	1413.6248	-0.1853	-131	125	136	LEDLRIPPAYVK					Mascot
1465.7216	1465.6792	-0.0424	-29	310	324	MSGGDHIHAGTVVGK					Mascot
1465.7546	1465.6792	-0.0754	-51	137	149	TFQGPPHGIQVER	24	46.54			Mascot
1793.8024	1793.7761	-0.0263	-15	192	205	DDENVNSQPFMRWR					Mascot
1809.7972	1809.7709	-0.0263	-15	192	205	DDENVNSQPFMRWR			Oxidation (M)[11]		Mascot
1821.9388	1821.8413	-0.0975	-54	307	324	ALRMSGGDHIHAGTVVG K			Oxidation (M)[4]		Mascot
3854.8718	3854.6709	-0.2009	-52	32	69	VTPQPGVPPEEAGAAVA AESSTGTWTTVWTDGLT SLDR					Mascot

9	Ribulose bisphosphate carboxylase large chain (Fragment) OS=Hamamelis mollis GN=rbcL PE=3 SV=1	RBL_HAMMO	51542.9	6.05	11	74	99.86	23	21.239

Peptide Information

Calc. Mass	Obsrv. Mass	± da	± ppm	Start Seq.	End Seq.	Sequence	Ion Score	C. I. %	Modification	Rank	Result Type
898.41	898.3665	-0.0435	-48	296	302	NHGMHFR					Mascot
910.4451	910.395	-0.0501	-55	178	184	AVYECLR			Carbamidomethyl (C)[5]		Mascot
914.4049	914.3558	-0.0491	-54	296	302	NHGMHFR			Oxidation (M)[4]		Mascot
1170.5585	1170.5684	0.0099	8	294	302	QKNHGMHFR			Oxidation (M)[6]		Mascot
1187.6643	1187.6082	-0.0561	-47	276	285	DNGLLLHIHR					Mascot
1245.6433	1245.5763	-0.067	-54	430	440	EGNEIIREASK					Mascot
1275.7307	1275.6697	-0.061	-48	330	340	EITLGFVDLLR					Mascot
1465.7216	1465.6792	-0.0424	-29	310	324	MSGGDHIHAGTVVGK					Mascot
1465.7546	1465.6792	-0.0754	-51	137	149	TFQGPPHGIQVER	24	46.54			Mascot
1793.8024	1793.7761	-0.0263	-15	192	205	DDENVNSQPFMRWR					Mascot
1809.7972	1809.7709	-0.0263	-15	192	205	DDENVNSQPFMRWR			Oxidation (M)[11]		Mascot
1821.9388	1821.8413	-0.0975	-54	307	324	ALRMSGGDHIHAGTVVG K			Oxidation (M)[4]		Mascot
3854.8718	3854.6709	-0.2009	-52	32	69	VTPQPGVPPEEAGAAVA AESSTGTWTTVWTDGLT SLDR					Mascot

10	Ribulose bisphosphate carboxylase large chain (Fragment) OS=Prunus domestica GN=rbcL PE=3 SV=1	RBL_PRUDO	50914.6	6.23	11	73	99.835	23	21.239

Peptide Information

Calc. Mass	Obsrv. Mass	± da	± ppm	Start Seq.	End Seq.	Sequence	Ion Score	C. I. %	Modification	Rank	Result Type
898.41	898.3665	-0.0435	-48	296	302	NHGMHFR					Mascot
910.4451	910.395	-0.0501	-55	178	184	AVYECLR			Carbamidomethyl (C)[5]		Mascot
914.4049	914.3558	-0.0491	-54	296	302	NHGMHFR			Oxidation (M)[4]		Mascot
1170.5585	1170.5684	0.0099	8	294	302	QKNHGMHFR			Oxidation (M)[6]		Mascot
1187.6643	1187.6082	-0.0561	-47	276	285	DNGLLLHIHR					Mascot
1245.6433	1245.5763	-0.067	-54	430	440	EGNEIIREASK					Mascot
1275.7307	1275.6697	-0.061	-48	330	340	EITLGFVDLLR					Mascot
1465.7216	1465.6792	-0.0424	-29	310	324	MSGGDHIHAGTVVGK					Mascot
1465.7546	1465.6792	-0.0754	-51	137	149	TFQGPPHGIQVER	24	45.3			Mascot
1793.8024	1793.7761	-0.0263	-15	192	205	DDENVNSQPFMRWR					Mascot
1809.7972	1809.7709	-0.0263	-15	192	205	DDENVNSQPFMRWR			Oxidation (M)[11]		Mascot
1821.9388	1821.8413	-0.0975	-54	307	324	ALRMSGGDHIHAGTVVG K			Oxidation (M)[4]		Mascot
3854.8718	3854.6709	-0.2009	-52	32	69	VTPQPGVPPEEAGAAVA AESSTGTWTTVWTDGLT SLDR					Mascot

Gel Idx/Pos	73/C22	Instr./Gel Origin	BA2060/131017A	Process Status	Analysis Succeeded
Plate [#] Name	[1] 31496	Instrument Sample Name		Spectra	11

Rank	Protein Name	Accession No.	Protein MW	Protein PI	Pep. Count	Protein Score	Protein Score C. I. %	Total Ion Score	Total Ion C. I. %
1	Fructose-bisphosphate aldolase 1, chloroplastic (Fragment) OS=Pisum sativum PE=1 SV=1	ALFC1_PEA	38633	5.83	4	157	100	145	100

Peptide Information

Calc. Mass	Obsrv. Mass	± da	± ppm	Start Seq.	End Seq.	Sequence	Ion Score	C. I. %	Modification	Rank	Result Type
873.4576	873.4239	-0.0337	-39	161	168	EAAWGLAR	14	0			Mascot
873.4576	873.4239	-0.0337	-39	161	168	EAAWGLAR					Mascot
1114.5276	1114.4794	-0.0482	-43	131	140	SAAYYQQGAR					Mascot
1114.5276	1114.4794	-0.0482	-43	131	140	SAAYYQQGAR	23	18.532			Mascot
1452.842	1452.7733	-0.0687	-47	146	160	TVVSIPNGPSALAVK					Mascot
2402.1404	2402.0183	-0.1221	-51	108	130	GLVPLAGSNDESWCQGLDGLASR	108	100	Carbamidomethyl (C)[14]		Mascot
2402.1404	2402.0183	-0.1221	-51	108	130	GLVPLAGSNDESWCQGLDGLASR			Carbamidomethyl (C)[14]		Mascot

Rank	Protein Name	Accession No.	Protein MW	Protein PI	Pep. Count	Protein Score	Protein Score C. I. %	Total Ion Score	Total Ion C. I. %
2	Fructose-bisphosphate aldolase, chloroplastic OS=Spinacia oleracea PE=1 SV=3	ALFC_SPIOL	42441.7	6.85	5	52	79.728	37	96.966

Peptide Information

Calc. Mass	Obsrv. Mass	± da	± ppm	Start Seq.	End Seq.	Sequence	Ion Score	C. I. %	Modification	Rank	Result Type
873.4576	873.4239	-0.0337	-39	201	208	EAAWGLAR	14	0			Mascot
873.4576	873.4239	-0.0337	-39	201	208	EAAWGLAR					Mascot
1114.5276	1114.4794	-0.0482	-43	171	180	SAAYYQQGAR					Mascot
1114.5276	1114.4794	-0.0482	-43	171	180	SAAYYQQGAR	23	18.532			Mascot
1387.7175	1387.6547	-0.0628	-45	81	93	LASIGLENTEANR					Mascot
1387.7175	1387.6547	-0.0628	-45	81	93	LASIGLENTEANR					Mascot
1452.842	1452.7733	-0.0687	-47	186	200	TVVSIPNGPSALAVK					Mascot
1969.1038	1968.9441	-0.1597	-81	130	147	MVDVLIEQGIVPGIKVDK			Oxidation (M)[1]		Mascot

Rank	Protein Name	Accession No.	Protein MW	Protein PI	Pep. Count	Protein Score	Protein Score C. I. %	Total Ion Score	Total Ion C. I. %
3	Fructose-bisphosphate aldolase 2, chloroplastic OS=Pisum sativum PE=1 SV=2	ALFC2_PEA	37803.4	5.48	4	49	57.646	37	96.966

Peptide Information

Calc. Mass	Obsrv. Mass	± da	± ppm	Start Seq.	End Seq.	Sequence	Ion Score	C. I. %	Modification	Rank	Result Type
873.4576	873.4239	-0.0337	-39	155	162	EAAWGLAR	14	0			Mascot
873.4576	873.4239	-0.0337	-39	155	162	EAAWGLAR					Mascot
1114.5276	1114.4794	-0.0482	-43	125	134	SAAYYQQGAR					Mascot
1114.5276	1114.4794	-0.0482	-43	125	134	SAAYYQQGAR	23	18.532			Mascot
1452.842	1452.7733	-0.0687	-47	140	154	TVVSIPNGPSALAVK					Mascot
1649.9836	1649.9072	-0.0764	-46	84	98	IVDVLVEQNIIPGIK					Mascot
1649.9836	1649.9072	-0.0764	-46	84	98	IVDVLVEQNIIPGIK					Mascot

Rank	Protein Name	Accession No.	Protein MW	Protein PI	Pep. Count	Protein Score	Protein Score C. I. %	Total Ion Score	Total Ion C. I. %
4	Alpha-1,4-glucan-protein synthase [UDP-forming] OS=Zea mays GN=UPTG PE=1 SV=2	UPTG_MAIZE	41177.6	5.75	8	48	50.238	15	0

Peptide Information

Calc. Mass	Obsrv. Mass	± da	± ppm	Start Seq.	End Seq.	Sequence	Ion Score	C. I. %	Modification	Rank	Result Type
839.4409	839.396	-0.0449	-53	159	165	GYPFSLR					Mascot
1223.5878	1223.5355	-0.0523	-43	31	39	NLDFLEMWR					Mascot
1239.5826	1239.561	-0.0216	-17	31	39	NLDFLEMWR			Oxidation (M)[7]		Mascot
1373.5653	1373.6549	0.0896	65	244	254	YDDMWAGWCVK					Mascot
1501.6958	1501.6293	-0.0665	-44	61	72	VPEGFDYELYNR					Mascot
1501.6958	1501.6293	-0.0665	-44	61	72	VPEGFDYELYNR	15	0			Mascot
1761.7756	1761.7234	-0.0522	-30	209	223	GTLFPMCGMNLAFDR			Carbamidomethyl (C)[7], Oxidation (M)[6,9]		Mascot
2089.0752	2089.0281	-0.0471	-23	40	57	AFFQPYHLIIVQDGDPTK					Mascot
2106.9688	2107.0854	0.1166	55	105	122	YIYTIDDDCFVAKDPSGK			Carbamidomethyl (C)[9]		Mascot
3342.7229	3342.5767	-0.1462	-44	166	195	EGAHTAVSHGLWLNIPDYDAPTQLVKPKER					Mascot

Rank	Protein Name	Accession No.	Protein MW	Protein PI	Pep. Count	Protein Score	Protein Score C. I. %	Total Ion Score	Total Ion C. I. %
5	Aminomethyltransferase, mitochondrial OS=Flave anomala GN=GDCST PE=3 SV=1	GCST_FLAAN	44250.8	8.82	10	46	5.181		

Peptide Information

Calc. Mass Calc. Mass	s rv. Mass	± da	± da ± ppm	± ppm Start Seq.	End S Seq.	Sequen	Score	C. I. % %	Modificat Modification	Rank	Rank
897.4498	897.469	0.0192	21	363	370	NIGMGYVK			Oxidation (M)[4]		Mascot
940.521	940.4308	-0.0902	-96	315	323	QIADGPAIR					Mascot
1254.5453	1254.5566	0.0113	9	65	75	DSIMDSTINCR					Mascot
1327.5616	1327.5424	-0.0192	-14	65	75	DSIMDSTINCR			Carbamidomethyl (C)[10], Oxidation (M)[4]		Mascot
1433.6444	1433.708	0.0636	44	169	180	GGDVSWHIYDER					Mascot
1465.7104	1465.6907	-0.0197	-13	152	163	DKDLAHIEEHMK					Mascot
1471.8129	1471.6957	-0.1172	-80	3	15	GGLWQLGQSITRR					Mascot
1786.9633	1786.7372	-0.2261	-127	363	379	NIGMGYVKSGLHKPGTK					Mascot
2211.2131	2210.9985	-0.2146	-97	302	323	AEGGFLGADVILKQIADGPAIR					Mascot
3315.7681	3315.582	-0.1861	-56	103	136	LVVADVAGLAPGTGSLTVFTNEKGGAIDDSVITK					Mascot
3342.6609	3342.5767	-0.0842	-25	270	299	LEAGLCLYGNDMEQHITPVEAGLTWAIGKR			Carbamidomethyl (C)[6]		Mascot

Rank	Protein Name	Accession No.	Protein MW	Protein PI	Pep. Count	Protein Score	Protein Score C. I. %	Total Ion Score	Total Ion C. I. %
6	Serine/threonine-protein phosphatase BSL2 OS=Arabidopsis thaliana GN=BSL2 PE=1 SV=2	BSL2_ARATH	108512.3	5.47	15	44	0		

Peptide Information

Calc. Mass	Obsrv. Mass	± da	± ppm	Start Seq.	End Seq.	Sequence	Ion Score	C. I. %	Modification	Rank	Result Type
839.4886	839.396	-0.0926	-110	675	681	GWKPPVR					Mascot
873.3737	873.4239	0.0502	57	481	487	YGFSDER					Mascot
873.3737	873.4239	0.0502	57	481	487	YGFSDER					Mascot
1080.5908	1080.4468	-0.144	-133	359	370	LHVSGGALGGGR					Mascot
1115.5844	1115.4745	-0.1099	-99	891	901	GPGLVTFGPDR					Mascot
1143.5177	1143.5417	0.024	21	481	489	YGFSDERNR					Mascot
1147.614	1147.5532	-0.0608	-53	656	665	QMSINSVPKK			Oxidation (M)[2]		Mascot
1147.614	1147.5532	-0.0608	-53	656	665	QMSINSVPKK			Oxidation (M)[2]		Mascot
1275.6593	1275.6702	0.0109	9	348	358	YQHAAVFVNAR					Mascot
1293.535	1293.6077	0.0727	56	917	927	AHECVMDGFER					Mascot
1465.7394	1465.6907	-0.0487	-33	637	650	RVSYGTPESATAAR					Mascot
1501.8413	1501.6293	-0.212	-141	821	833	LFNWLPLAASIEK					Mascot
1501.8413	1501.6293	-0.212	-141	821	833	LFNWLPLAASIEK					Mascot
1619.675	1619.7225	0.0475	29	84	97	CAPTYSVVDAMMDK			Carbamidomethyl (C)[1], Oxidation (M)[11,12]		Mascot
1629.9363	1629.728	-0.2083	-128	821	834	LFNWLPLAASIEKK					Mascot
1761.8085	1761.7234	-0.0851	-48	1003	1018	GRPQNSNDRGGSLAWM			Oxidation (M)[16]		Mascot
2088.9001	2089.0281	0.128	61	683	700	QFFLDCNEIADLCDSAER					Mascot
3342.3757	3342.5767	0.201	60	2	31	DEDSSMVADNDQDREFQSLDGGQSPSPMER					Mascot

Rank	Protein Name	Accession No.	Protein MW	Protein PI	Pep. Count	Protein Score	Protein Score C. I. %	Total Ion Score	Total Ion C. I. %
7	Protein transport Sec1a OS=Arabidopsis thaliana GN=SEC1A PE=2 SV=3	SEC1A_ARAT	76059.7	7.99	14	43	0		

Peptide Information

Calc. Mass	Obsrv. Mass	± da	± ppm	Start Seq.	End Seq.	Sequence	Ion Score	C. I. %	Modification	Rank	Result Type
839.3749	839.396	0.0211	25	57	63	VMSQSCK			Carbamidomethyl (C)[6]		Mascot
860.4472	860.4406	-0.0066	-8	136	143	SDSSVLPR					Mascot
897.4676	897.469	0.0014	2	596	603	SASTEFKK					Mascot
1015.608	1015.5417	-0.0663	-65	446	454	GVKLMQLAR					Mascot
1143.6302	1143.5417	-0.0885	-77	623	632	VCHKLTSSLR					Mascot
1187.718	1187.592	-0.126	-106	47	56	ILIMDRVTVK					Mascot
1471.7297	1471.6957	-0.034	-23	183	194	HFHICLNIMATR			Oxidation (M)[9]		Mascot
1501.823	1501.6293	-0.1937	-129	449	461	LMQLARLSPVDMK					Mascot
1501.823	1501.6293	-0.1937	-129	449	461	LMQLARLSPVDMK					Mascot
1725.9608	1725.7769	-0.1839	-107	427	440	LRLLMIYATVYPEK			Oxidation (M)[5]		Mascot
1829.983	1829.7893	-0.1937	-106	220	236	DLVPSKLAAAIWDCISK					Mascot
1891.7399	1891.8087	0.0688	36	1	19	MSFSDSESSSHGGGGGDYK					Mascot
2210.9373	2210.9985	0.0612	28	2	22	SFSDSESSSHGGGGGDYKFFR					Mascot
2316.2021	2316.0376	-0.1645	-71	654	673	LLSEKDIQGAPAQPFKPQYW					Mascot
2402.2834	2402.0183	-0.2651	-110	183	203	HFHICLNIMATRIATVFASLK			Oxidation (M)[9]		Mascot
2402.2834	2402.0183	-0.2651	-110	183	203	HFHICLNIMATRIATVFASLK			Oxidation (M)[9]		Mascot

Rank	Protein Name	Accession No.	Protein MW	Protein PI	Pep. Count	Protein Score	Protein Score C. I. %	Total Ion Score	Total Ion C. I. %
8	30S ribosomal protein S2, chloroplastic 2 OS=Scenedesmus obliquus GN=rps2-2 PE=3 SV=1	RR2B_SCEOE	43784.6	10.08	10	42	0		

Peptide Information

Calc. Mass	Obsrv. Mass	± da	± ppm	Start Seq.	End Seq.	Sequence	Ion Score	C. I. %	Modification	Rank	Result Type
839.4113	839.396	-0.0153	-18	280	286	MAKMNTK			Oxidation (M)[1]		Mascot
1115.5918	1115.4745	-0.1173	-105	273	282	YFGGITKMAK					Mascot
1254.6576	1254.5566	-0.101	-81	25	35	NTTVVTLSYEK					Mascot
1327.6926	1327.5424	-0.1502	-113	148	158	MLKELNSTFTK			Oxidation (M)[1]		Mascot
1387.729	1387.6547	-0.0743	-54	344	354	YILGEMLTYIR			Oxidation (M)[6]		Mascot
1387.729	1387.6547	-0.0743	-54	344	354	YILGEMLTYIR			Oxidation (M)[6]		Mascot
1822.0295	1821.8915	-0.138	-76	1	15	MKLVQFFEIGQQLLK					Mascot
1829.9829	1829.7893	-0.1936	-106	340	354	NSIKYILGEMLTYIR			Oxidation (M)[10]		Mascot
2088.9402	2089.0281	0.0879	42	75	93	EENGFFATNQMGTSSNRAK					Mascot
2170.0378	2169.885	-0.1528	-70	291	309	NVAIIIGQQEEMNAVHECR			Oxidation (M)[12]		Mascot
2211.0645	2210.9985	-0.066	-30	291	309	NVAIIIGQQEEMNAVHECR			Carbamidomethyl (C)[18]		Mascot
2339.1594	2338.9919	-0.1675	-72	291	310	NVAIIIGQQEEMNAVHECRK			Carbamidomethyl (C)[18]		Mascot

Rank	Protein Name	Accession No.	Protein MW	Protein PI	Pep. Count	Protein Score	Protein Score C. I. %	Total Ion Score	Total Ion C. I. %
9	Thioredoxin H-type OS=Oryza sativa subsp. indica GN=TRXH PE=1 SV=1	TRXH_ORYSI	13147.7	5.16	6	41	0		

Peptide Information

Calc. Mass	Obsrv. Mass	± da	± ppm	Start Seq.	End Seq.	Sequence	Ion Score	C. I. %	Modification	Rank	Result Type
1045.5525	1045.5262	-0.0263	-25	103	111	DDLQNTIVK					Mascot
1187.6014	1187.592	-0.0094	-8	90	101	DGAEADKVVGAR					Mascot
1373.7158	1373.6549	-0.0609	-44	65	76	VDVDELKEVAEK					Mascot
1471.7031	1471.6957	-0.0074	-5	1	14	MAAEEGVVIACHNK					Mascot
1968.9985	1968.9441	-0.0544	-28	103	122	DDLQNTIVKHVGATAASASA					Mascot
3017.5051	3017.3972	-0.1079	-36	30	55	VVIIDFTASWCGPCRFIAPVFAEYAK			Carbamidomethyl (C)[11,14]		Mascot
3017.5051	3017.3972	-0.1079	-36	30	55	VVIIDFTASWCGPCRFIAPVFAEYAK			Carbamidomethyl (C)[11,14]		Mascot

Rank	Protein Name	Accession No.	Protein MW	Protein PI	Pep. Count	Protein Score	Protein Score C. I. %	Total Ion Score	Total Ion C. I. %
10	Hexokinase-2 OS=Oryza sativa subsp. japonica GN=HXK2 PE=2 SV=1	HXK2_ORYSJ	53591.9	5.77	10	41	0		

Peptide Information

Calc. Mass	Obsrv. Mass	± da	± ppm	Start Seq.	End Seq.	Sequence	Ion Score	C. I. %	Modification	Rank	Result Type
940.5145	940.4308	-0.0837	-89	45	53	CAAPPGRLR					Mascot
1114.5851	1114.4794	-0.1057	-95	418	427	LGRDVPNTDK					Mascot
1114.5851	1114.4794	-0.1057	-95	418	427	LGRDVPNTDK					Mascot
1187.6631	1187.592	-0.0711	-60	377	387	DILGVQNTSLK					Mascot
1327.6423	1327.5424	-0.0999	-75	40	51	ELEERCAAPPGR					Mascot
1387.5262	1387.6547	0.1285	93	354	364	TPYMSMMHCDR			Oxidation (M)[4]		Mascot
1387.5262	1387.6547	0.1285	93	354	364	TPYMSMMHCDR			Oxidation (M)[4]		Mascot
1555.8267	1555.6636	-0.1631	-105	166	178	QRELGFTFSFPVK					Mascot
1619.7621	1619.7225	-0.0396	-24	331	345	MAEEASLFGDEVPPK					Mascot
2089.0886	2089.0281	-0.0605	-29	327	345	VLLKMAEEASLFGDEVPPK			Oxidation (M)[5]		Mascot
2316.1321	2316.0376	-0.0945	-41	54	76	QVADAMAVEMHAGLASEGGSKLK			Oxidation (M)[6]		Mascot
3017.4375	3017.3972	-0.0403	-13	287	313	SSHLPLTEFDQALDAESLNPGEQVYEK					Mascot
3017.4375	3017.3972	-0.0403	-13	287	313	SSHLPLTEFDQALDAESLNPGEQVYEK					Mascot

Gel Idx/Pos	74/C23	Instr./Gel Origin	BA2060/131017A	Process Status	Analysis Succeeded
Plate [#] Name	[1] 31496	Instrument Sample Name		Spectra	8

Rank	Protein Name	Accession No.	Protein MW	Protein PI	Pep. Count	Protein Score	Protein Score C. I. %	Total Ion Score	Total Ion C. I. %
1	Fructose-bisphosphate aldolase 1, chloroplastic (Fragment) OS=Pisum sativum PE=1 SV=1	ALFC1_PEA	38633	5.83	8	291	100	253	100

Peptide Information

Calc. Mass	Obsrv. Mass	± da	± ppm	Start Seq.	End Seq.	Sequence	Ion Score	C. I. %	Modification	Rank	Result Type
822.4355	822.4061	-0.0294	-36	197	203	TFEVAQK					Mascot
873.4576	873.4282	-0.0294	-34	161	168	EAAWGLAR	58	99.979			Mascot
873.4576	873.4282	-0.0294	-34	161	168	EAAWGLAR					Mascot
917.505	917.4742	-0.0308	-34	327	335	SNSLAQLGK					Mascot
1100.5735	1100.5328	-0.0407	-37	306	315	TWGGLPENVK					Mascot
1114.5276	1114.4893	-0.0383	-34	131	140	SAAYYQQGAR	35	96.186			Mascot
1114.5276	1114.4893	-0.0383	-34	131	140	SAAYYQQGAR					Mascot
1452.842	1452.7841	-0.0579	-40	146	160	TVVSIPNGPSALAVK					Mascot
1466.6614	1466.6503	-0.0111	-8	26	39	GILAMDESNATCGK			Carbamidomethyl (C)[12]		Mascot
1482.6562	1482.5923	-0.0639	-43	26	39	GILAMDESNATCGK			Carbamidomethyl (C)[12], Oxidation (M)[5]		Mascot
2402.1404	2402.0417	-0.0987	-41	108	130	GLVPLAGSNDESWCQGLDGLASR	160	100	Carbamidomethyl (C)[14]		Mascot
2402.1404	2402.0417	-0.0987	-41	108	130	GLVPLAGSNDESWCQGLDGLASR			Carbamidomethyl (C)[14]		Mascot

Rank	Protein Name	Accession No.	Protein MW	Protein PI	Pep. Count	Protein Score	Protein Score C. I. %	Total Ion Score	Total Ion C. I. %
2	Fructose-bisphosphate aldolase, chloroplastic OS=Spinacia oleracea PE=1 SV=3	ALFC_SPIOL	42441.7	6.85	9	242	100	200	100

Peptide Information

Calc. Mass	Obsrv. Mass	± da	± ppm	Start Seq.	End Seq.	Sequence	Ion Score	C. I. %	Modification	Rank	Result Type
873.4576	873.4282	-0.0294	-34	201	208	EAAWGLAR	58	99.979			Mascot
873.4576	873.4282	-0.0294	-34	201	208	EAAWGLAR					Mascot
910.4087	910.4205	0.0118	13	356	363	AQDFACAK			Carbamidomethyl (C)[6]		Mascot
917.505	917.4742	-0.0308	-34	364	372	SNSLAQLGK					Mascot
947.4978	947.4586	-0.0392	-41	338	345	ALQNTCLK			Carbamidomethyl (C)[6]		Mascot
1114.5276	1114.4893	-0.0383	-34	171	180	SAAYYQQGAR	35	96.186			Mascot
1114.5276	1114.4893	-0.0383	-34	171	180	SAAYYQQGAR					Mascot
1387.7175	1387.6666	-0.0509	-37	81	93	LASIGLENTEANR	107	100			Mascot
1387.7175	1387.6666	-0.0509	-37	81	93	LASIGLENTEANR					Mascot
1452.842	1452.7841	-0.0579	-40	186	200	TVVSIPNGPSALAVK					Mascot
1466.6614	1466.6503	-0.0111	-8	66	79	GILAMDESNATCGK			Carbamidomethyl (C)[12]		Mascot
1482.6562	1482.5923	-0.0639	-43	66	79	GILAMDESNATCGK			Carbamidomethyl (C)[12], Oxidation (M)[5]		Mascot
1543.8186	1543.7605	-0.0581	-38	80	93	RLASIGLENTEANR					Mascot

Rank	Protein Name	Accession No.	Protein MW	Protein PI	Pep. Count	Protein Score	Protein Score C. I. %	Total Ion Score	Total Ion C. I. %
3	Fructose-bisphosphate aldolase, chloroplastic OS=Oryza sativa subsp. japonica GN=Os11g0171300 PE=1	ALFC_ORYSJ	41979.5	6.38	9	207	100	165	100

Peptide Information

Calc. Mass	Obsrv. Mass	± da	± ppm	Start Seq.	End Seq.	Sequence	Ion Score	C. I. %	Modification	Rank	Result Type
822.4355	822.4061	-0.0294	-36	229	235	TFEVAQK					Mascot
873.4576	873.4282	-0.0294	-34	193	200	EAAWGLAR	58	99.979			Mascot
873.4576	873.4282	-0.0294	-34	193	200	EAAWGLAR					Mascot
947.4978	947.4586	-0.0392	-41	330	337	ALQNTCLK			Carbamidomethyl (C)[6]		Mascot
1100.6422	1100.5328	-0.1094	-99	357	367	AKANSLAQLGK					Mascot
1115.548	1115.4816	-0.0664	-60	338	347	TWGGQPENVK					Mascot
1387.7175	1387.6666	-0.0509	-37	73	85	LASIGLENTEANR	107	100			Mascot
1387.7175	1387.6666	-0.0509	-37	73	85	LASIGLENTEANR					Mascot
1466.6614	1466.6503	-0.0111	-8	58	71	GILAMDESNATCGK			Carbamidomethyl (C)[12]		Mascot
1482.6562	1482.5923	-0.0639	-43	58	71	GILAMDESNATCGK			Carbamidomethyl (C)[12], Oxidation (M)[5]		Mascot
1502.7386	1502.7775	0.0389	26	163	175	EAAYYQQGARFAK					Mascot
1543.8186	1543.7605	-0.0581	-38	72	85	RLASIGLENTEANR					Mascot

Rank	Protein Name	Accession No.	Protein MW	Protein PI	Pep. Count	Protein Score	Protein Score C. I. %	Total Ion Score	Total Ion C. I. %
4	Probable fructose-bisphosphate aldolase 1, chloroplastic OS=Arabidopsis thaliana GN=FBA1 PE=1 SV=2	ALFC1_ARAT	42903.9	6.18	7	192	100	165	100

Peptide Information

Calc. Mass	Obsrv. Mass	± da	± ppm	Start Seq.	End Seq.	Sequence	Ion Score	C. I. %	Modification	Rank	Result Type
873.4576	873.4282	-0.0294	-34	204	211	EAAWGLAR	58	99.979			Mascot
873.4576	873.4282	-0.0294	-34	204	211	EAAWGLAR					Mascot
947.4978	947.4586	-0.0392	-41	341	348	ALQNTCLK			Carbamidomethyl (C)[6]		Mascot
1100.6422	1100.5328	-0.1094	-99	368	378	AKANSLAQLGK					Mascot
1147.5742	1147.5615	-0.0127	-11	349	358	TWGGKEENVK					Mascot
1387.7175	1387.6666	-0.0509	-37	84	96	LASIGLENTEANR	107	100			Mascot
1387.7175	1387.6666	-0.0509	-37	84	96	LASIGLENTEANR					Mascot
1452.842	1452.7841	-0.0579	-40	189	203	TVVSIPNGPSALAVK					Mascot
1543.8186	1543.7605	-0.0581	-38	83	96	RLASIGLENTEANR					Mascot

Rank	Protein Name	Accession No.	Protein MW	Protein PI	Pep. Count	Protein Score	Protein Score C. I. %	Total Ion Score	Total Ion C. I. %
5	Fructose-bisphosphate aldolase 2, chloroplastic OS=Pisum sativum PE=1 SV=2	ALFC2_PEA	37803.4	5.48	10	147	100	93	100

Peptide Information

Calc. Mass	Obsrv. Mass	± da	± ppm	Start Seq.	End Seq.	Sequence	Ion Score	C. I. %	Modification	Rank	Result Type
822.4355	822.4061	-0.0294	-36	191	197	TFEVAQK					Mascot
873.4576	873.4282	-0.0294	-34	155	162	EAAWGLAR	58	99.979			Mascot
873.4576	873.4282	-0.0294	-34	155	162	EAAWGLAR					Mascot
917.505	917.4742	-0.0308	-34	321	329	SNSLAQLGK					Mascot
995.3233	995.4698	0.1465	147	342	349	DCCQGYSY			Carbamidomethyl (C)[2]		Mascot
1100.5735	1100.5328	-0.0407	-37	300	309	TWGGLPENVK					Mascot
1114.5276	1114.4893	-0.0383	-34	125	134	SAAYYQQGAR	35	96.186			Mascot
1114.5276	1114.4893	-0.0383	-34	125	134	SAAYYQQGAR					Mascot
1452.842	1452.7841	-0.0579	-40	140	154	TVVSIPNGPSALAVK					Mascot
1466.6614	1466.6503	-0.0111	-8	20	33	GILAMDESNATCGK			Carbamidomethyl (C)[12]		Mascot
1482.6562	1482.5923	-0.0639	-43	20	33	GILAMDESNATCGK			Carbamidomethyl (C)[12], Oxidation (M)[5]		Mascot
1649.9836	1649.9181	-0.0655	-40	84	98	IVDVLVEQNIIPGIK					Mascot
1778.0785	1778.0017	-0.0768	-43	83	98	KIVDVLVEQNIIPGIK					Mascot

Rank	Protein Name	Accession No.	Protein MW	Protein PI	Pep. Count	Protein Score	Protein Score C. I. %	Total Ion Score	Total Ion C. I. %
6	Probable fructose-bisphosphate aldolase 2, chloroplastic OS=Arabidopsis thaliana GN=FBA2 PE=1 SV=2	ALFC2_ARAT	42961.1	6.78	8	91	99.997	58	99.979

Peptide Information

Calc. Mass	Obsrv. Mass	± da	± ppm	Start Seq.	End Seq.	Sequence	Ion Score	C. I. %	Modification	Rank	Result Type
873.4576	873.4282	-0.0294	-34	203	210	EAAWGLAR	58	99.979			Mascot
873.4576	873.4282	-0.0294	-34	203	210	EAAWGLAR					Mascot
947.4978	947.4586	-0.0392	-41	340	347	ALQNTCLK			Carbamidomethyl (C)[6]		Mascot
1100.6422	1100.5328	-0.1094	-99	367	377	AKANSLAQLGK					Mascot
1358.7314	1358.6736	-0.0578	-43	9	20	ASPVLDKSEWVK					Mascot
1452.842	1452.7841	-0.0579	-40	188	202	TVVSIPNGPSALAVK					Mascot
1453.7533	1453.7784	0.0251	17	47	60	AASSYADELVKTAK					Mascot
1466.6614	1466.6503	-0.0111	-8	68	81	GILAMDESNATCGK			Carbamidomethyl (C)[12]		Mascot
1482.6562	1482.5923	-0.0639	-43	68	81	GILAMDESNATCGK			Carbamidomethyl (C)[12], Oxidation (M)[5]		Mascot
1562.8173	1562.7794	-0.0379	-24	279	292	DRATPEQVAAYTLK					Mascot

Rank	Protein Name	Accession No.	Protein MW	Protein PI	Pep. Count	Protein Score	Protein Score C. I. %	Total Ion Score	Total Ion C. I. %
7	Probable fructose-bisphosphate aldolase 3, chloroplastic OS=Arabidopsis thaliana GN=FBA3 PE=1 SV=1	ALFC3_ARAT	42300.6	8.19	3	66	99.073	58	99.979

Peptide Information

Calc. Mass	Obsrv. Mass	± da	± ppm	Start Seq.	End Seq.	Sequence	Ion Score	C. I. %	Modification	Rank	Result Type
873.4576	873.4282	-0.0294	-34	196	203	EAAWGLAR	58	99.979			Mascot
873.4576	873.4282	-0.0294	-34	196	203	EAAWGLAR					Mascot
910.4451	910.4205	-0.0246	-27	124	130	TFVDCLR			Carbamidomethyl (C)[5]		Mascot
1100.6422	1100.5328	-0.1094	-99	360	370	AKANSLAQLGK					Mascot

Rank	Protein Name	Accession No.	Protein MW	Protein PI	Pep. Count	Protein Score	Protein Score C. I. %	Total Ion Score	Total Ion C. I. %
8	S-adenosylmethionine synthase 2 OS=Dianthus caryophyllus GN=SAM2 PE=2 SV=1	METK2_DIAC	43161.8	5.57	7	53	84.622	26	66.246

Peptide Information

Calc. Mass	Obsrv. Mass	± da	± ppm	Start Seq.	End Seq.	Sequence	Ion Score	C. I. %	Modification	Rank	Result Type
842.5709	842.4809	-0.09	-107	336	342	EILKIVK					Mascot
873.5151	873.4282	-0.0869	-99	296	304	SIVASGLAR	26	66.246			Mascot
873.5151	873.4282	-0.0869	-99	296	304	SIVASGLAR					Mascot
1100.6099	1100.5328	-0.0771	-70	388	396	TLKWEKPQA					Mascot
1452.7766	1452.7841	0.0075	5	52	64	TNLVMVFGEITTK					Mascot
1453.7546	1453.7784	0.0238	16	242	256	FVIGGPHGDAGLTGR					Mascot
1502.7533	1502.7775	0.0242	16	161	173	KNGTCAWLRPDGK			Carbamidomethyl (C)[5]		Mascot
2402.1763	2402.0417	-0.1346	-56	44	64	VACETCTKTNLVMVFGEITTK			Carbamidomethyl (C)[3,6]		Mascot
2402.1763	2402.0417	-0.1346	-56	44	64	VACETCTKTNLVMVFGEITTK			Carbamidomethyl (C)[3,6]		Mascot
2418.1714	2418.0403	-0.1311	-54	44	64	VACETCTKTNLVMVFGEITTK			Carbamidomethyl (C)[3,6], Oxidation (M)[13]		Mascot

Rank	Protein Name	Accession No.	Protein MW	Protein PI	Pep. Count	Protein Score	Protein Score C. I. %	Total Ion Score	Total Ion C. I. %
9	Protein GIGANTEA OS=Oryza sativa subsp. japon GN=GI PE=2 SV=2	GIGAN_ORYS	126987	6.38	17	53	82.746		

Peptide Information

Calc. Mass	Obsrv. Mass	± da	± ppm	Start Seq.	End Seq.	Sequence	Ion Score	C. I. %	Modification	Rank	Result Type
856.5363	856.4949	-0.0414	-48	977	983	VNQRIVK					Mascot
910.3909	910.4205	0.0296	33	213	220	WCGGVMGK			Carbamidomethyl (C)[2], Oxidation (M)[6]		Mascot
1015.6047	1015.5336	-0.0711	-70	833	841	NGFARPLLK					Mascot
1100.631	1100.5328	-0.0982	-89	948	958	ASAAIVLQAEK					Mascot
1114.447	1114.4893	0.0423	38	354	363	AAEDYDSGMR					Mascot
1114.447	1114.4893	0.0423	38	354	363	AAEDYDSGMR					Mascot
1143.6997	1143.5516	-0.1481	-129	832	841	KNGFARPLLK					Mascot
1143.6997	1143.5516	-0.1481	-129	832	841	KNGFARPLLK	1	0			Mascot
1187.5262	1187.6156	0.0894	75	1129	1137	CIEWEAHSR			Carbamidomethyl (C)[1]		Mascot
1275.6539	1275.6796	0.0257	20	1086	1097	DILNSGQINSSK					Mascot
1348.7834	1348.7106	-0.0728	-54	1139	1152	ATGLTLAFLTAAAK					Mascot
1387.7844	1387.6666	-0.1178	-85	138	148	VLTHYNRPIFK					Mascot
1387.7844	1387.6666	-0.1178	-85	138	148	VLTHYNRPIFK					Mascot
1452.6787	1452.7841	0.1054	73	354	366	AAEDYDSGMRLPK					Mascot
1453.7798	1453.7784	-0.0014	-1	768	780	ASSLYHLIDLHGK					Mascot
1562.6879	1562.7794	0.0915	59	208	220	SDYFRWCGGVMGK			Carbamidomethyl (C)[7]		Mascot
1649.8242	1649.9181	0.0939	57	539	552	TFPSETSEQSRKPR					Mascot
1777.949	1778.0017	0.0527	30	1062	1077	LSTTIRCLSHPSAHVR					Mascot
2311.1897	2311.0515	-0.1382	-60	376	398	AIGTAMSMRAGIAADTSAALLFR			Oxidation (M)[6]		Mascot
3017.6709	3017.427	-0.2439	-81	326	353	LLFGLLEAPPSWAPDALDAAVQLVELLR					Mascot

Rank	Protein Name	Accession No.	Protein MW	Protein PI	Pep. Count	Protein Score	Protein Score C. I. %	Total Ion Score	Total Ion C. I. %
10	Maturase K OS=Cryptomeria japonica GN=matK [illegible] SV=2	MATK_CRYJ	60566.6	9.66	13	52	78.773		

Peptide Information

Calc. Mass	Obsrv. Mass	± da	± ppm	Start Seq.	End Seq.	Sequence	Ion Score	C. I. %	Modification	Rank	Result Type
842.528	842.4809	-0.0471	-56	337	343	MKPLVVR					Mascot
857.4661	857.5005	0.0344	40	437	443	TLACKHK			Carbamidomethyl (C)[4]		Mascot
859.5035	859.4131	-0.0904	-105	251	257	DIVLFPR					Mascot
910.5468	910.4205	-0.1263	-139	287	294	GTHLQVKK					Mascot
1004.4829	1004.5165	0.0336	33	500	507	MQNKQIEN					Mascot
1004.4829	1004.5165	0.0336	33	500	507	MQNKQIEN					Mascot
1021.5789	1021.4949	-0.084	-82	169	177	DAPSLHLLR					Mascot
1100.6826	1100.5328	-0.1498	-136	249	257	IKDIVLFPR					Mascot
1358.6772	1358.6736	-0.0036	-3	84	94	LIECNKNFYSK					Mascot
1621.7816	1621.886	0.1044	64	69	83	QTNSISLFGNSDPNK					Mascot
1749.8766	1749.9746	0.098	56	68	83	KQTNSISLFGNSDPNK					Mascot
1777.8544	1778.0017	0.1473	83	228	242	FFNSQSLLYGSFPDR					Mascot
2418.2891	2418.0403	-0.2488	-103	316	336	ICSLQLSKTSFSFLGYFLHVK					Mascot
3047.4324	3047.4387	0.0063	2	114	138	HFIEGMNGWNSLRSIHCLFPLMEDK			Carbamidomethyl (C)[17], Oxidation (M)[6]		Mascot

Gel Idx/Pos	75/C24	Instr./Gel Origin	BA2060/131017A	Process Status	Analysis Succeeded
Plate [#] Name	[1] 31496	Instrument Sample Name		Spectra	8

Rank	Protein Name	Accession No.	Protein MW	Protein PI	Pep. Count	Protein Score	Protein Score C. I. %	Total Ion Score	Total Ion C. I. %
1	Fructose-bisphosphate aldolase 1, chloroplastic (Fragment) OS=Pisum sativum PE=1 SV=1	ALFC1_PEA	38633	5.83	6	163	100	140	100

Peptide Information

Calc. Mass	Obsrv. Mass	± da	± ppm	Start Seq.	End Seq.	Sequence	Ion Score	C. I. %	Modification	Rank	Result Type
858.5043	858.4825	-0.0218	-25	298	305	ALQNTALK					Mascot
873.4576	873.4298	-0.0278	-32	161	168	EAAWGLAR	20	0			Mascot
873.4576	873.4298	-0.0278	-32	161	168	EAAWGLAR					Mascot
1114.5276	1114.4845	-0.0431	-39	131	140	SAAYYQQGAR					Mascot
1452.842	1452.7914	-0.0506	-35	146	160	TVVSIPNGPSALAVK					Mascot
1571.85	1571.7072	-0.1428	-91	40	53	RLASIGLENTEVNR					Mascot
2402.1404	2402.0549	-0.0855	-36	108	130	GLVPLAGSNDESWCQGLDGLASR			Carbamidomethyl (C)[14]		Mascot
2402.1404	2402.0549	-0.0855	-36	108	130	GLVPLAGSNDESWCQGLDGLASR	120	100	Carbamidomethyl (C)[14]		Mascot

Rank	Protein Name	Accession No.	Protein MW	Protein PI	Pep. Count	Protein Score	Protein Score C. I. %	Total Ion Score	Total Ion C. I. %
2	Fructose-bisphosphate aldolase 2, chloroplastic OS=Pisum sativum PE=1 SV=2	ALFC2_PEA	37803.4	5.48	7	64	98.75	34	95.968

Peptide Information

Calc. Mass	Obsrv. Mass	± da	± ppm	Start Seq.	End Seq.	Sequence	Ion Score	C. I. %	Modification	Rank	Result Type
858.5043	858.4825	-0.0218	-25	292	299	ALQNTALK					Mascot
873.4576	873.4298	-0.0278	-32	155	162	EAAWGLAR	20	0			Mascot
873.4576	873.4298	-0.0278	-32	155	162	EAAWGLAR					Mascot
1114.5276	1114.4845	-0.0431	-39	125	134	SAAYYQQGAR					Mascot
1452.842	1452.7914	-0.0506	-35	140	154	TVVSIPNGPSALAVK					Mascot
1649.9836	1649.9286	-0.055	-33	84	98	IVDVLVEQNIIPGIK					Mascot
1649.9836	1649.9286	-0.055	-33	84	98	IVDVLVEQNIIPGIK	14	0			Mascot
1778.0785	1778.0184	-0.0601	-34	83	98	KIVDVLVEQNIIPGIK					Mascot
2401.1565	2401.0356	-0.1209	-50	102	124	GLVPLAGSNNESWCQGLDGLASR			Carbamidomethyl (C)[14]		Mascot

Rank	Protein Name	Accession No.	Protein MW	Protein PI	Pep. Count	Protein Score	Protein Score C. I. %	Total Ion Score	Total Ion C. I. %
3	Probable fructose-bisphosphate aldolase 1, chloroplastic OS=Arabidopsis thaliana GN=FBA1 PE=1 SV=2	ALFC1_ARAT	42903.9	6.18	6	64	98.629	44	99.528

Peptide Information

Calc. Mass	Obsrv. Mass	± da	± ppm	Start Seq.	End Seq.	Sequence	Ion Score	C. I. %	Modification	Rank	Result Type
873.4576	873.4298	-0.0278	-32	204	211	EAAWGLAR	20	0			Mascot
873.4576	873.4298	-0.0278	-32	204	211	EAAWGLAR					Mascot
1147.5742	1147.5627	-0.0115	-10	349	358	TWGGKEENVK					Mascot
1387.7175	1387.6708	-0.0467	-34	84	96	LASIGLENTEANR					Mascot
1387.7175	1387.6708	-0.0467	-34	84	96	LASIGLENTEANR	24	54.655			Mascot
1452.842	1452.7914	-0.0506	-35	189	203	TVVSIPNGPSALAVK					Mascot
1543.8186	1543.7682	-0.0504	-33	83	96	RLASIGLENTEANR					Mascot
2012.1096	2012.0116	-0.098	-49	133	150	MVDVLVEQNIVPGIKVDK			Oxidation (M)[1]		Mascot

Rank	Protein Name	Accession No.	Protein MW	Protein PI	Pep. Count	Protein Score	Protein Score C. I. %	Total Ion Score	Total Ion C. I. %
4	Fructose-bisphosphate aldolase, chloroplastic OS=Oryza sativa subsp. japonica GN=Os11g0171300 PE=1	ALFC_ORYSJ	41979.5	6.38	5	60	96.395	44	99.528

Peptide Information

Calc. Mass	Obsrv. Mass	± da	± ppm	Start Seq.	End Seq.	Sequence	Ion Score	C. I. %	Modification	Rank	Result Type
873.4576	873.4298	-0.0278	-32	193	200	EAAWGLAR	20	0			Mascot
873.4576	873.4298	-0.0278	-32	193	200	EAAWGLAR					Mascot
1115.548	1115.4795	-0.0685	-61	338	347	TWGGQPENVK					Mascot
1115.548	1115.4795	-0.0685	-61	338	347	TWGGQPENVK					Mascot
1387.7175	1387.6708	-0.0467	-34	73	85	LASIGLENTEANR					Mascot
1387.7175	1387.6708	-0.0467	-34	73	85	LASIGLENTEANR	24	54.655			Mascot
1543.8186	1543.7682	-0.0504	-33	72	85	RLASIGLENTEANR					Mascot
2401.1565	2401.0356	-0.1209	-50	140	162	GLVPLAGSNNESWCQGLDGLASR			Carbamidomethyl (C)[14]		Mascot

Rank	Protein Name	Accession No.	Protein MW	Protein PI	Pep. Count	Protein Score	Protein Score C. I. %	Total Ion Score	Total Ion C. I. %
5	Fructose-bisphosphate aldolase, chloroplastic OS=Spinacia oleracea PE=1 SV=3	ALFC_SPIOL	42441.7	6.85	5	59	95.565	44	99.528

Peptide Information

Calc. Mass	Obsrv. Mass	± da	± ppm	Start Seq.	End Seq.	Sequence	Ion Score	C. I. %	Modification	Rank	Result Type
873.4576	873.4298	-0.0278	-32	201	208	EAAWGLAR	20	0			Mascot
873.4576	873.4298	-0.0278	-32	201	208	EAAWGLAR					Mascot
1114.5276	1114.4845	-0.0431	-39	171	180	SAAYYQQGAR					Mascot
1387.7175	1387.6708	-0.0467	-34	81	93	LASIGLENTEANR					Mascot
1387.7175	1387.6708	-0.0467	-34	81	93	LASIGLENTEANR	24	54.655			Mascot
1452.842	1452.7914	-0.0506	-35	186	200	TVVSIPNGPSALAVK					Mascot
1543.8186	1543.7682	-0.0504	-33	80	93	RLASIGLENTEANR					Mascot

Rank	Protein Name	Accession No.	Protein MW	Protein PI	Pep. Count	Protein Score	Protein Score C. I. %	Total Ion Score	Total Ion C. I. %
6	DEAD-box ATP-dependent RNA helicase 30 OS=Arabidopsis thaliana GN=RH30 PE=2 SV=2	RH30_ARATH	64580.9	9.66	12	48	42.866		

Peptide Information

Calc. Mass	Obsrv. Mass	± da	± ppm	Start Seq.	End Seq.	Sequence	Ion Score	C. I. %	Modification	Rank	Result Type
962.592	962.4749	-0.1171	-122	413	420	ILIFVETK					Mascot
1045.4558	1045.5375	0.0817	78	90	101	NGGGNWGGGGGR					Mascot
1143.6368	1143.556	-0.0808	-71	107	116	RELDSVSLPK					Mascot
1143.6368	1143.556	-0.0808	-71	107	116	RELDSVSLPK					Mascot
1175.595	1175.5358	-0.0592	-50	422	431	GCDQVTRQLR					Mascot
1293.6685	1293.6285	-0.04	-31	313	323	VTYLVLDEADR					Mascot
1358.7216	1358.6823	-0.0393	-29	345	355	QTLLWSATWPR					Mascot
1452.7263	1452.7914	0.0651	45	265	278	SGVRSTCIYGGAPK			Carbamidomethyl (C)[7]		Mascot
2011.9655	2012.0116	0.0461	23	432	449	MDGWPALAIHGDKTQSER					Mascot
2101.0786	2100.9685	-0.1101	-52	183	201	LGFTEPTPIQAQGWPMALK			Oxidation (M)[16]		Mascot
2298.2063	2298.1094	-0.0969	-42	183	203	LGFTEPTPIQAQGWPMALKGR					Mascot
2748.3735	2748.2092	-0.1643	-60	159	182	DVPKPMKMFQDANFPDNILEAIAK			Oxidation (M)[6]		Mascot
3808.7734	3808.6726	-0.1008	-26	117	148	QNFGNLVHFEKNFYVESPTVQAMTEQDVAMYR			Oxidation (M)[23]		Mascot

Rank	Protein Name	Accession No.	Protein MW	Protein PI	Pep. Count	Protein Score	Protein Score C. I. %	Total Ion Score	Total Ion C. I. %
7	Aspartate aminotransferase, chloroplastic OS=Arabidopsis thaliana GN=ASP3 PE=1 SV=1	AAT3_ARATH	48923.4	9.34	10	47	28.073		

Peptide Information

Calc. Mass	Obsrv. Mass	± da	± ppm	Start Seq.	End Seq.	Sequence	Ion Score	C. I. %	Modification	Rank	Result Type
962.5417	962.4749	-0.0668	-69	76	84	LNLGVGAYR					Mascot
1004.5887	1004.52	-0.0687	-68	372	379	KQLFEALR					Mascot
1163.6024	1163.6022	-0.0002	0	362	371	AMADRIISMR					Mascot
1387.5947	1387.6708	0.0761	55	413	423	EYHIYMTSDGR			Oxidation (M)[6]		Mascot
1387.5947	1387.6708	0.0761	55	413	423	EYHIYMTSDGR			Oxidation (M)[6]		Mascot
1453.8373	1453.7832	-0.0541	-37	85	97	TEEGKPLVLNVVR					Mascot
1571.8904	1571.7072	-0.1832	-117	433	447	TVPHLADAIHAVVTK					Mascot
1649.8533	1649.9286	0.0753	46	349	361	DKNLFNEWTLELK					Mascot
1649.8533	1649.9286	0.0753	46	349	361	DKNLFNEWTLELK					Mascot
2748.5593	2748.2092	-0.3501	-127	326	350	LVIRPMYSSPPIHGASIVAVILRDK			Oxidation (M)[6]		Mascot
2807.3345	2807.2068	-0.1277	-45	253	278	GLMPFFDSAYQGFASGSLDTDAKPIR			Oxidation (M)[3]		Mascot
3409.707	3409.479	-0.228	-67	382	412	GTPGDWSHIIKQIGMFTFTGLNPAQVSFMTK					Mascot

Rank	Protein Name	Accession No.	Protein MW	Protein PI	Pep. Count	Protein Score	Protein Score C. I. %	Total Ion Score	Total Ion C. I. %
8	Respiratory burst oxidase homolog protein C OS=Arabidopsis thaliana GN=RBOHC PE=2 SV=2	RBOHC_ARA	102452.9	9.5	14	42	0		

Peptide Information

Calc. Mass	Obsrv. Mass	± da	± ppm	Start Seq.	End Seq.	Sequence	Ion Score	C. I. %	Modification	Rank	Result Type
858.4427	858.4825	0.0398	46	745	751	EQAQLNR					Mascot
1004.5734	1004.52	-0.0534	-53	134	143	TKSAASQALK					Mascot
1143.5105	1143.556	0.0455	40	780	788	EQGSFDWFK					Mascot
1143.5105	1143.556	0.0455	40	780	788	EQGSFDWFK					Mascot
1358.6443	1358.6823	0.038	28	178	189	TKFGECIGMTSK			Carbamidomethyl (C)[6]		Mascot
1778.0394	1778.0184	-0.021	-12	113	130	LTSVSGGIGGRKPPRPAK					Mascot
2012.1394	2012.0116	-0.1278	-64	391	409	GAAETVKLNMALILLPVCR					Mascot
2225.2473	2225.0425	-0.2048	-92	663	684	ALKGVFSEVCKPPPAGVSGLLR					Mascot
2230.0393	2230.1248	0.0855	38	772	788	AYFYWVTREQGSFDWFK					Mascot
2297.2983	2297.1069	-0.1914	-83	398	416	LNMALILLPVCRNTITWLR			Carbamidomethyl (C)[11]		Mascot
2402.0857	2402.0549	-0.0308	-13	376	397	SPVYPVMGDCVCMAKGAAETVK			Carbamidomethyl (C)[10,12], Oxidation (M)[7,13]		Mascot
2402.2085	2402.0549	-0.1536	-64	51	72	TAPYVDLTVDVQDDTVSVHSLK					Mascot
2434.178	2434.0359	-0.1421	-58	180	201	FGECIGMTSKDFALELFDALAR					Mascot
2659.3574	2659.1082	-0.2492	-94	49	72	TRTAPYVDLTVDVQDDTVSVHSLK					Mascot
2780.3687	2780.3567	-0.012	-4	560	581	DWHNKTTWMYLVVPVVLYACER			Carbamidomethyl (C)[20]		Mascot

Rank	Protein Name	Accession No.	Protein MW	Protein PI	Pep. Count	Protein Score	Protein Score C. I. %	Total Ion Score	Total Ion C. I. %
9	Ribulose bisphosphate carboxylase/oxygenase a B, chloroplastic OS=Hordeum vulgare GN=RCAB PE	RCAB_HORVI	47197.8	7.59	8	42	0	9	0

Peptide Information

Calc. Mass	Obsrv. Mass	± da	± ppm	Start Seq.	End Seq.	Sequence	Ion Score	C. I. %	Modification	Rank	Result Type
873.4676	873.4298	-0.0378	-43	199	206	EAADIINK					Mascot
873.5338	873.4298	-0.104	-119	38	45	VVRVMAAK	10	0			Mascot
1093.571	1093.507	-0.064	-59	136	144	NFMTLPNIK			Oxidation (M)[3]		Mascot
1115.5367	1115.4795	-0.0572	-51	365	374	EGPPTFDQPK					Mascot
1115.5367	1115.4795	-0.0572	-51	365	374	EGPPTFDQPK					Mascot
2211.2722	2211.0281	-0.2441	-110	136	155	NFMTLPNIKVPLILGIWGGK					Mascot
2663.2837	2663.1414	-0.1423	-53	170	194	MGINPIMMSAGELESGNGEPAKLIR			Oxidation (M)[1,7,8]		Mascot
3017.4788	3017.4534	-0.0254	-8	110	135	YDFDNTMDGLYIAPAFMDKLIVHLAK			Oxidation (M)[7]		Mascot
3017.4788	3017.4534	-0.0254	-8	110	135	YDFDNTMDGLYIAPAFMDKLIVHLAK			Oxidation (M)[7]		Mascot
3354.6311	3354.5881	-0.043	-13	78	108	GIVDSLFQAPMGDGTHEAILSSYEYISQGLR					Mascot

Mascot

Rank	Protein Name	Accession No.	Protein MW	Protein PI	Pep. Count	Protein Score	Protein Score C. I. %	Total Ion Score	Total Ion C. I. %
10	S-adenosylmethionine synthase 2 (Fragment) OS=Petroselinum crispum GN=SMS-2 PE=2 SV=1	METK2_PET	15748.3	9.55	5	41	0		

Peptide Information

Calc. Mass	Obsrv. Mass	± da	± ppm	Start Seq.	End Seq.	Sequence	Ion Score	C. I. %	Modification	Rank	Result Type
842.5345	842.4855	-0.049	-58	86	92	EILQIVK					Mascot
873.5151	873.4298	-0.0853	-98	46	54	SIVASGLAR	9	0			Mascot
873.5151	873.4298	-0.0853	-98	46	54	SIVASGLAR					Mascot
1163.6531	1163.6022	-0.0509	-44	35	45	SGAYIVRQAAK					Mascot
2012.0157	2012.0116	-0.0041	-2	93	109	ETFDFRPGMISINLDLK			Oxidation (M)[9]		Mascot
2663.3252	2663.1414	-0.1838	-69	119	141	TAAYGHFGREDPDFTWEVVKPLK					Mascot

Gel Idx/Pos	76/D1	Instr./Gel Origin	BA2060/131017A	Process Sta	Analysis Succeeded
Plate [#] Name	[1] 31496	Instrument Sample Name		Spectra	11

Rank	Protein Name	Accession No	Protein MW	Protein PI	Pep. Count	Protein Score	Protein Score C. I. %	Total Ion Score	Total Ion C. I. %
1	Malate dehydrogenase (Fragments) OS=Pseudots menziesii PE=1 SV=1	MDH_PSEMZ	2518.4	4.43	2	84	99.985	63	99.993

fragment

Peptide Information

Calc. Mass	Obsrv. Mass	± da	± ppm	Start Seq.	End Seq.	Sequence	Ion Score	C. I. %	Modification	Rank	Result Type
1219.7045	1219.5333	-0.1712	-140	13	23	LFGVTTLDVVR	18	0			Mascot
1219.7045	1219.5333	-0.1712	-140	13	23	LFGVTTLDVVR					Mascot
1318.7001	1318.5142	-0.1859	-141	1	12	DDLFNINAGIVK	45	99.563			Mascot
1318.7001	1318.5142	-0.1859	-141	1	12	DDLFNINAGIVK					Mascot

Rank	Protein Name	Accession No	Protein MW	Protein PI	Pep. Count	Protein Score	Protein Score C. I. %	Total Ion Score	Total Ion C. I. %
2	Malate dehydrogenase, mitochondrial (Fragments OS=Capsicum annuum var. annuum PE=1 SV=1	MDHM_CAPA	3733	4.23	2	81	99.971	63	99.993

Peptide Information

Calc. Mass	Obsrv. Mass	± da	± ppm	Start Seq.	End Seq.	Sequence	Ion Score	C. I. %	Modification	Rank	Result Type
1219.7045	1219.5333	-0.1712	-140	13	23	LFGVTTLDVVR	18	0			Mascot
1219.7045	1219.5333	-0.1712	-140	13	23	LFGVTTLDVVR					Mascot
1318.7001	1318.5142	-0.1859	-141	1	12	DDLFNINAGIVK	45	99.563			Mascot
1318.7001	1318.5142	-0.1859	-141	1	12	DDLFNINAGIVK					Mascot

Rank	Protein Name	Accession No	Protein MW	Protein PI	Pep. Count	Protein Score	Protein Score C. I. %	Total Ion Score	Total Ion C. I. %
3	Malate dehydrogenase, mitochondrial OS=Citrullu lanatus GN=MMDH PE=1 SV=1	MDHM_CITLA	36178.2	8.88	4	75	99.889	63	99.993

Peptide Information

Calc. Mass	Obsrv. Mass	± da	± ppm	Start Seq.	End Seq.	Sequence	Ion Score	C. I. %	Modification	Rank	Result Type
834.4104	834.3402	-0.0702	-84	340	347	GIQFANAN					Mascot
1219.7045	1219.5333	-0.1712	-140	176	186	LFGVTTLDVVR	18	0			Mascot
1219.7045	1219.5333	-0.1712	-140	176	186	LFGVTTLDVVR					Mascot
1318.7001	1318.5142	-0.1859	-141	121	132	DDLFNINAGIVK	45	99.563			Mascot
1318.7001	1318.5142	-0.1859	-141	121	132	DDLFNINAGIVK					Mascot
1495.7274	1495.5764	-0.151	-101	85	98	SEVTGYVGEEQLGK					Mascot

Rank	Protein Name	Accession No	Protein MW	Protein PI	Pep. Count	Protein Score	Protein Score C. I. %	Total Ion Score	Total Ion C. I. %
4	Malate dehydrogenase 1, mitochondrial OS=Arabidopsis thaliana GN=At1g53240 PE=1 SV=1	MDHM1_ARAT	35781.9	8.54	3	72	99.783	63	99.993

Peptide Information

Calc. Mass	Obsrv. Mass	± da	± ppm	Start Seq.	End Seq.	Sequence	Ion Score	C. I. %	Modification	Rank	Result Type
1219.7045	1219.5333	-0.1712	-140	171	181	LFGVTTLDVVR	18	0			Mascot
1219.7045	1219.5333	-0.1712	-140	171	181	LFGVTTLDVVR					Mascot
1318.7001	1318.5142	-0.1859	-141	116	127	DDLFNINAGIVK	45	99.563			Mascot
1318.7001	1318.5142	-0.1859	-141	116	127	DDLFNINAGIVK					Mascot
3027.5793	3027.1692	-0.4101	-135	136	162	YCPHALINMISNPVNSTVPIAAEIFKK			Carbamidomethyl (C)[2]		Mascot

Rank	Protein Name	Accession No	Protein MW	Protein PI	Pep. Count	Protein Score	Protein Score C. I. %	Total Ion Score	Total Ion C. I. %
5	Malate dehydrogenase, mitochondrial OS=Brassi napus GN=MDH PE=2 SV=1	MDHM_BRAN	35688.9	8.81	2	68	99.525	63	99.993

Peptide Information

Calc. Mass	Obsrv. Mass	± da	± ppm	Start Seq.	End Seq.	Sequence	Ion Score	C. I. %	Modification	Rank	Result Type
1219.7045	1219.5333	-0.1712	-140	171	181	LFGVTTLDVVR	18	0			Mascot
1219.7045	1219.5333	-0.1712	-140	171	181	LFGVTTLDVVR					Mascot
1318.7001	1318.5142	-0.1859	-141	116	127	DDLFNINAGIVK	45	99.563			Mascot
1318.7001	1318.5142	-0.1859	-141	116	127	DDLFNINAGIVK					Mascot

Rank	Protein Name	Accession No	Protein MW	Protein PI	Pep. Count	Protein Score	Protein Score C. I. %	Total Ion Score	Total Ion C. I. %
6	Trypsin inhibitor 1 OS=Trichosanthes kirilowii PE SV=1	ITR1_TRIKI	6872.1	4.61	7	54	87.209		

Peptide Information

Calc. Mass	Obsrv. Mass	± da	± ppm	Start Seq.	End Seq.	Sequence	Ion Score	C. I. %	Modification	Rank	Result Type
834.3774	834.3402	-0.0372	-45	48	54	VNDDCLR					Mascot
850.4417	850.4253	-0.0164	-19	2	9	AAFVESAR					Mascot
873.3229	873.3503	0.0274	31	58	65	CLSNGYCG			Carbamidomethyl (C)[1]		Mascot
1165.5162	1165.5825	0.0663	57	32	41	MMEGVVACPR			Carbamidomethyl (C)[8], Oxidation (M)[1]		Mascot
1275.4915	1275.554	0.0625	49	55	65	GCKCLSNGYCG			Carbamidomethyl (C)[2,4,10]		Mascot
1576.7644	1576.6791	-0.0853	-54	42	54	ILMPCKVNDDCLR			Carbamidomethyl (C)[5]		Mascot
1576.7644	1576.6791	-0.0853	-54	42	54	ILMPCKVNDDCLR			Carbamidomethyl (C)[5]		Mascot
1882.8716	1882.6862	-0.1854	-98	32	47	MMEGVVACPRILMPCK			Carbamidomethyl (C)[8], Oxidation (M)[1,2,13]		Mascot

Rank	Protein Name	Accession No	Protein MW	Protein PI	Pep. Count	Protein Score	Protein Score C. I. %	Total Ion Score	Total Ion C. I. %
7	Malate dehydrogenase, mitochondrial (Fragments OS=Imperata cylindrica PE=1 SV=1	MDHM_IMPCY	3107.7	5.91	1	53	82.344	45	99.563

Peptide Information

Calc. Mass	Obsrv. Mass	± da	± ppm	Start Seq.	End Seq.	Sequence	Ion Score	C. I. %	Modification	Rank	Result Type
1318.7001	1318.5142	-0.1859	-141	20	31	DDLFNINAGIVK	45	99.563			Mascot
1318.7001	1318.5142	-0.1859	-141	20	31	DDLFNINAGIVK					Mascot

Rank	Protein Name	Accession No	Protein MW	Protein PI	Pep. Count	Protein Score	Protein Score C. I. %	Total Ion Score	Total Ion C. I. %
8	Malate dehydrogenase, glyoxysomal OS=Glycine PE=2 SV=2	MDHG_SOYBI	37370.7	7.55	3	53	81.933	45	99.563

Peptide Information

Calc. Mass	Obsrv. Mass	± da	± ppm	Start Seq.	End Seq.	Sequence	Ion Score	C. I. %	Modification	Rank	Result Type
850.478	850.4253	-0.0527	-62	35	43	AKGGVSGFK					Mascot
905.476	905.3565	-0.1195	-132	140	147	TLCEAIAK			Carbamidomethyl (C)[3]		Mascot
1318.7001	1318.5142	-0.1859	-141	128	139	DDLFNINAGIVK	45	99.563			Mascot
1318.7001	1318.5142	-0.1859	-141	128	139	DDLFNINAGIVK					Mascot

Rank	Protein Name	Accession No	Protein MW	Protein PI	Pep. Count	Protein Score	Protein Score C. I. %	Total Ion Score	Total Ion C. I. %
9	Ribulose bisphosphate carboxylase small chain, chloroplastic OS=Oryza sativa subsp. japonica GN=RBC	RBS1_ORYSJ	19633.9	9.04	8	47	29.71		

Peptide Information

Calc. Mass	Obsrv. Mass	± da	± ppm	Start Seq.	End Seq.	Sequence	Ion Score	C. I. %	Modification	Rank	Result Type
905.4509	905.3565	-0.0944	-104	21	29	STAGMPVAR			Oxidation (M)[5]		Mascot
914.4002	914.2737	-0.1265	-138	104	111	SPGYYDGR					Mascot
938.473	938.4205	-0.0525	-56	139	146	AYPDAFVR					Mascot
1045.5571	1045.4178	-0.1393	-133	21	30	STAGMPVARR					Mascot
1165.571	1165.5825	0.0115	10	85	93	WVPCLEFSK			Carbamidomethyl (C)[4]		Mascot
1495.6996	1495.5764	-0.1232	-82	30	44	RSGNSSFGNVSNGGR					Mascot
1886.9622	1886.6891	-0.2731	-145	85	99	WVPCLEFSKVGFVYR			Carbamidomethyl (C)[4]		Mascot
2239.0984	2238.8057	-0.2927	-131	118	137	LPMFGCTDATQVLKELEEAK			Oxidation (M)[3]		Mascot

Rank	Protein Name	Accession No	Protein MW	Protein PI	Pep. Count	Protein Score	Protein Score C. I. %	Total Ion Score	Total Ion C. I. %
10	Malate dehydrogenase, mitochondrial OS=Eucaly gunnii GN=MDH PE=2 SV=1	MDHM_EUCGI	36439.4	8.72	1	45	0	45	99.563

Peptide Information

Calc. Mass	Obsrv. Mass	± da	± ppm	Start Seq.	End Seq.	Sequence	Ion Score	C. I. %	Modification	Rank	Result Type
1318.7001	1318.5142	-0.1859	-141	121	132	DDLFNINAGIVK	45	99.563			Mascot
1318.7001	1318.5142	-0.1859	-141	121	132	DDLFNINAGIVK					Mascot

Gel Idx/Pos	77/D2	Instr./Gel Origin	BA2060/131017A	Process Sta	Analysis Succeeded
Plate [#] Name	[1] 31496	Instrument Sample Name		Spectra	11

Rank	Protein Name	Accession No	Protein MW	Protein PI	Pep. Count	Protein Score	Protein Score C. I. %	Total Ion Score	Total Ion C. I. %
1	Trypsin inhibitor 1 OS=Trichosanthes kirilowii PE SV=1	ITR1_TRIKI	6872.1	4.61	6	45	0		

Peptide Information

Calc. Mass	Obsrv. Mass	± da	± ppm	Start Seq.	End Seq.	Sequence	Ion Score	C. I. %	Modification	Rank	Result Type
850.4417	850.4357	-0.006	-7	2	9	AAFVESAR					Mascot
997.4771	997.4577	-0.0194	-19	1	9	MAAFVESAR			Oxidation (M)[1]		Mascot
1165.5162	1165.5919	0.0757	65	32	41	MMEGVVACPR			Carbamidomethyl (C)[8], Oxidation (M)[1]		Mascot
1165.5162	1165.5919	0.0757	65	32	41	MMEGVVACPR			Carbamidomethyl (C)[8], Oxidation (M)[1]		Mascot
1179.5245	1179.4321	-0.0924	-78	48	57	VNDDCLRGCK			Carbamidomethyl (C)[5]		Mascot
1275.4915	1275.5642	0.0727	57	55	65	GCKCLSNGYCG			Carbamidomethyl (C)[2,4,10]		Mascot
1882.8716	1882.6947	-0.1769	-94	32	47	MMEGVVACPRILMPCK			Carbamidomethyl (C)[8], Oxidation (M)[1,2,13]		Mascot

Rank	Protein Name	Accession No	Protein MW	Protein PI	Pep. Count	Protein Score	Protein Score C. I. %	Total Ion Score	Total Ion C. I. %
2	F-box/kelch-repeat protein At1g15670 OS=Arabido thaliana GN=At1g15670 PE=2 SV=1	FK126_ARAT	40152.6	5.46	9	40	0		

Peptide Information

Calc. Mass	Obsrv. Mass	± da	± ppm	Start Seq.	End Seq.	Sequence	Ion Score	C. I. %	Modification	Rank	Result Type
1180.5853	1180.4469	-0.1384	-117	23	32	QFPLMASVCK			Carbamidomethyl (C)[9]		Mascot
1275.5422	1275.5642	0.022	17	159	169	SFFACASDSQR			Carbamidomethyl (C)[5]		Mascot
1465.678	1465.537	-0.141	-96	347	359	YEGHVQAGCFLEI					Mascot
1604.7811	1604.5614	-0.2197	-137	19	32	SSYKQFPLMASVCK			Oxidation (M)[9]		Mascot
1604.7811	1604.5614	-0.2197	-137	19	32	SSYKQFPLMASVCK			Oxidation (M)[9]		Mascot
1684.767	1684.7117	-0.0553	-33	182	196	NAMMSALVYDVAEDR					Mascot
1684.767	1684.7117	-0.0553	-33	182	196	NAMMSALVYDVAEDR					Mascot
1779.9033	1779.7312	-0.1721	-97	23	36	QFPLMASVCKLWQR			Carbamidomethyl (C)[9], Oxidation (M)[5]		Mascot
1973.8593	1973.567	-0.2923	-148	152	169	SMPGGPRSFFACASDSQR			Carbamidomethyl (C)[12], Oxidation (M)[2]		Mascot
2121.0605	2120.8159	-0.2446	-115	1	18	MELIPDLPETVAYECLLR			Oxidation (M)[1]		Mascot
2162.0872	2161.8381	-0.2491	-115	1	18	MELIPDLPETVAYECLLR			Carbamidomethyl (C)[15]		Mascot
2758.2786	2757.9736	-0.305	-111	182	205	NAMMSALVYDVAEDRWAFLPDMGR					Mascot

Rank	Protein Name	Accession No	Protein MW	Protein PI	Pep. Count	Protein Score	Protein Score C. I. %	Total Ion Score	Total Ion C. I. %
3	ATP-dependent Clp protease proteolytic subunit-r protein 1, chloroplastic OS=Arabidopsis thal	CLPR1_ARAT	42600	8.81	7	28	0		

Peptide Information

Calc. Mass	Obsrv. Mass	± da	± ppm	Start Seq.	End Seq.	Sequence	Ion Score	C. I. %	Modification	Rank	Result Type
850.3876	850.4357	0.0481	57	52	59	SHCFASAK					Mascot
1180.5126	1180.4469	-0.0657	-56	146	154	YSMSVQMYR			Oxidation (M)[3]		Mascot
1465.7103	1465.537	-0.1733	-118	72	84	GDNLKDGVMQNFK					Mascot
1639.8326	1639.6514	-0.1812	-110	336	350	YLQAQAAIDYGIADK					Mascot
1993.8895	1993.7008	-0.1887	-95	52	68	SHCFASAKDMSFDHIPK			Carbamidomethyl (C)[3], Oxidation (M)[10]		Mascot
2126.0911	2125.8806	-0.2105	-99	2	21	ATALVSPLTSQLNHEAVCSK			Carbamidomethyl (C)[18]		Mascot
2126.0911	2125.8806	-0.2105	-99	2	21	ATALVSPLTSQLNHEAVCSK			Carbamidomethyl (C)[18]		Mascot
2298.0151	2297.8274	-0.1877	-82	226	246	METVGSETEAYAIADTISYCK			Oxidation (M)[1]		Mascot

Rank	Protein Name	Accession No	Protein MW	Protein PI	Pep. Count	Protein Score	Protein Score C. I. %	Total Ion Score	Total Ion C. I. %
4	Xanthine dehydrogenase 2 OS=Arabidopsis thali GN=XDH2 PE=2 SV=1	XDH2_ARATH	148667.2	6.27	13	27	0		

Peptide Information

Calc. Mass	Obsrv. Mass	± da	± ppm	Start Seq.	End Seq.	Sequence	Ion Score	C. I. %	Modification	Rank	Result Type
882.4203	882.4507	0.0304	34	579	585	QDYETVK					Mascot
1165.5306	1165.5919	0.0613	53	520	530	EGAPGGMVEFR			Oxidation (M)[7]		Mascot
1165.5306	1165.5919	0.0613	53	520	530	EGAPGGMVEFR			Oxidation (M)[7]		Mascot
1180.6208	1180.4469	-0.1739	-147	576	585	IGKQDYETVK					Mascot
1275.5997	1275.5642	-0.0355	-28	448	459	DDDIAIVNGGMR					Mascot
1670.6897	1670.6217	-0.068	-41	736	749	GDVELCFQSGQCDR			Carbamidomethyl (C)[6,12]		Mascot
1684.7417	1684.7117	-0.03	-18	735	749	KGDVELCFQSGQCDR					Mascot
1684.7417	1684.7117	-0.03	-18	735	749	KGDVELCFQSGQCDR					Mascot
1829.8785	1829.621	-0.2575	-141	851	866	DVDMMITGHRHSFVGK					Mascot
1886.9218	1886.7096	-0.2122	-112	917	933	IVGNVCFTNFPSNTAFR					Mascot
1886.9218	1886.7096	-0.2122	-112	917	933	IVGNVCFTNFPSNTAFR					Mascot
2108.9084	2108.8098	-0.0986	-47	1	17	MEQNEFMEAIMYVNGVR			Oxidation (M)[1,7,11]		Mascot
2540.093	2539.8557	-0.2373	-93	1332	1353	MACFDEFSAPFANSDFCPKLSV			Carbamidomethyl (C)[3,17]		Mascot
2660.3501	2660.0154	-0.3347	-126	934	957	GFGGPQGMLITENWIQRIAAELDK			Oxidation (M)[8]		Mascot
2780.377	2780.5684	0.1914	69	877	901	ILALDLEIYNNGGNSMDLSLSNLER			Oxidation (M)[16]		Mascot
2995.4136	2994.9792	-0.4344	-145	1092	1121	VPNASPTAASASSDMYGAAVLDACEQIIAR			Oxidation (M)[15]		Mascot

Rank	Protein Name	Accession No	Protein MW	Protein PI	Pep. Count	Protein Score	Protein Score C. I. %	Total Ion Score	Total Ion C. I. %
5	Viscotoxin-1-PS OS=Viscum album GN=THI2.4 P SV=1	THN1_VISAL	4900.8	9.55	4	26	0		

Peptide Information

Calc. Mass	Obsrv. Mass	± da	± ppm	Start Seq.	End Seq.	Sequence	Ion Score	C. I. %	Modification	Rank	Result Type
996.3873	996.4667	0.0794	80	2	10	SCCPBTTGR			Carbamidomethyl (C)[2]		Mascot
1179.5609	1179.4321	-0.1288	-109	24	33	ZVCARISGCK			Carbamidomethyl (C)[3,9]		Mascot
1180.5198	1180.4469	-0.0729	-62	1	10	KSCCPBTTGR			Carbamidomethyl (C)[3,4]		Mascot
1973.8374	1973.567	-0.2704	-137	2	17	SCCPBTTGRBIYBTCR			Carbamidomethyl (C)[2,3,15]		Mascot

Rank	Protein Name	Accession No	Protein MW	Protein PI	Pep. Count	Protein Score	Protein Score C. I. %	Total Ion Score	Total Ion C. I. %
6	Ribulose bisphosphate carboxylase small chain chloroplastic OS=Solanum tuberosum GN=RBCS-1 PE=3	RBS1_SOLTU	20543.2	8.23	5	24	0		

Peptide Information

Calc. Mass	Obsrv. Mass	± da	± ppm	Start Seq.	End Seq.	Sequence	Ion Score	C. I. %	Modification	Rank	Result Type
2240.0229	2239.8196	-0.2033	-91	94	111	NGWVPCLEFETEHGFVYR			Carbamidomethyl (C)[6]		Mascot
2368.1345	2367.958	-0.1765	-75	130	150	LPMFGCTDATQVLAEVQECKK			Carbamidomethyl (C)[6]		Mascot
2660.354	2660.0154	-0.3386	-127	159	181	IIGFDNVRQVQCISFIAYKPEGY					Mascot
2748.2622	2747.9248	-0.3374	-123	94	115	NGWVPCLEFETEHGFVYRENHK			Carbamidomethyl (C)[6]		Mascot
3078.4231	3078.1487	-0.2744	-89	124	149	YWTMWKLPMFGCTDATQVLAEVQECK					Mascot

Rank	Protein Name	Accession No	Protein MW	Protein PI	Pep. Count	Protein Score	Protein Score C. I. %	Total Ion Score	Total Ion C. I. %
7	Protein RALF-like 17 OS=Arabidopsis thaliana GN=RALFL17 PE=3 SV=1	RLF17_ARAT	8371.8	4.19	4	23	0		

Peptide Information

Calc. Mass	Obsrv. Mass	± da	± ppm	Start Seq.	End Seq.	Sequence	Ion Score	C. I. %	Modification	Rank	Result Type
1465.5608	1465.537	-0.0238	-16	35	50	GGCGGGDGSLGDDNER					Mascot
2283.1196	2282.8081	-0.3115	-136	6	23	EFIICCFLTLLLCNFFMR			Carbamidomethyl (C)[5]		Mascot
2684.322	2683.9263	-0.3957	-147	2	23	AASREFIICCFLTLLLCNFFMR			Carbamidomethyl (C)[9], Oxidation (M)[21]		Mascot
2758.3411	2757.9736	-0.3675	-133	1	23	MAASREFIICCFLTLLLCNFFMR			Oxidation (M)[1]		Mascot

Rank	Protein Name	Accession No	Protein MW	Protein PI	Pep. Count	Protein Score	Protein Score C. I. %	Total Ion Score	Total Ion C. I. %
8	Protein Brevis radix-like 3 OS=Arabidopsis thaliar GN=BRXL3 PE=2 SV=2	BRXL3_ARAT	41964.5	6.92	6	23	0		

Peptide Information

Calc. Mass	Obsrv. Mass	± da	± ppm	Start Seq.	End Seq.	Sequence	Ion Score	C. I. %	Modification	Rank	Result Type
1180.5198	1180.4469	-0.0729	-62	58	68	NCKPCSGTTNR					Mascot
1541.704	1541.6188	-0.0852	-55	128	140	TESIVFMEDDEVK					Mascot
1829.8831	1829.621	-0.2621	-143	349	362	FGETHARLWWEQNR					Mascot
2109.0176	2108.8098	-0.2078	-99	2	20	LTCIACTKQLNTNNGGSTR			Carbamidomethyl (C)[3,6]		Mascot
2126.0151	2125.8806	-0.1345	-63	1	20	MLTCIACTKQLNTNNGGSTR					Mascot
2126.0151	2125.8806	-0.1345	-63	1	20	MLTCIACTKQLNTNNGGSTR					Mascot
2240.0581	2239.8196	-0.2385	-106	1	20	MLTCIACTKQLNTNNGGSTR			Carbamidomethyl (C)[4,7]		Mascot
3018.3555	3018.0923	-0.2632	-87	113	140	GISSEEGTPTSMSGRTESIVFMEDDEVK					Mascot

Rank	Protein Name	Accession No	Protein MW	Protein PI	Pep. Count	Protein Score	Protein Score C. I. %	Total Ion Score	Total Ion C. I. %
9	Glycine-rich RNA-binding protein 6, mitochondrial OS=Arabidopsis thaliana GN=RBG6 PE=2 SV=1	RBG6_ARATH	15950	9.88	4	23	0		

Peptide Information

Calc. Mass	Obsrv. Mass	± da	± ppm	Start Seq.	End Seq.	Sequence	Ion Score	C. I. %	Modification	Rank	Result Type
842.4042	842.3956	-0.0086	-10	54	61	EAFGSFGK					Mascot
997.4961	997.4577	-0.0384	-38	134	143	GGYARGGFGR					Mascot
1604.7716	1604.5614	-0.2102	-131	139	155	GGFGRGGFGGGGYGFVR					Mascot
1604.7716	1604.5614	-0.2102	-131	139	155	GGFGRGGFGGGGYGFVR					Mascot
1684.8376	1684.7117	-0.1259	-75	2	15	HYMGLFSRAGNIFR			Oxidation (M)[3]		Mascot
1684.8376	1684.7117	-0.1259	-75	2	15	HYMGLFSRAGNIFR			Oxidation (M)[3]		Mascot

Rank	Protein Name	Accession No	Protein MW	Protein PI	Pep. Count	Protein Score	Protein Score C. I. %	Total Ion Score	Total Ion C. I. %
10	Hemoglobin-2 OS=Casuarina glauca GN=HBII PE SV=1	HBP2_CASGL	17834.3	8.93	4	22	0		

Peptide Information

Calc. Mass	Obsrv. Mass	± da	± ppm	Start Seq.	End Seq.	Sequence	Ion Score	C. I. %	Modification	Rank	Result Type
2109.0513	2108.8098	-0.2415	-115	100	118	LGASHFKHGVADEHFEVTK					Mascot
2140.0591	2139.9541	-0.105	-49	1	19	MSTLEGRGFTEEQEALVVK			Oxidation (M)[1]		Mascot
2211.0718	2210.7957	-0.2761	-125	68	86	SHAMSVFLMTCESAVQLRK			Carbamidomethyl (C)[11], Oxidation (M)[4]		Mascot
2211.0718	2210.7957	-0.2761	-125	68	86	SHAMSVFLMTCESAVQLRK			Carbamidomethyl (C)[11], Oxidation (M)[4]		Mascot
2283.1294	2282.8081	-0.3213	-141	66	85	LKSHAMSVFLMTCESAVQLR			Oxidation (M)[6,11]		Mascot

Gel Idx/Pos	78/D3	Instr./Gel Origin	BA2060/131017A	Process Sta	Analysis Succeeded
Plate [#] Name	[1] 31496	Instrument Sample Name		Spectra	11

Rank	Protein Name	Accession No	Protein MW	Protein PI	Pep. Count	Protein Score	Protein Score C. I. %	Total Ion Score	Total Ion C. I. %
1	Fructose-bisphosphate aldolase 1, chloroplastic (Fragment) OS=Pisum sativum PE=1 SV=1	ALFC1_PEA	38633	5.83	6	197	100	174	100

Peptide Information

Calc. Mass	Obsrv. Mass	± da	± ppm	Start Seq.	End Seq.	Sequence	Ion Score	C. I. %	Modification	Rank	Result Type
858.5043	858.4055	-0.0988	-115	298	305	ALQNTALK					Mascot
873.4576	873.3399	-0.1177	-135	161	168	EAAWGLAR	16	0			Mascot
873.4576	873.3399	-0.1177	-135	161	168	EAAWGLAR					Mascot
1114.5276	1114.3771	-0.1505	-135	131	140	SAAYYQQGAR					Mascot
1114.5276	1114.3771	-0.1505	-135	131	140	SAAYYQQGAR	51	99.911			Mascot
1452.842	1452.6327	-0.2093	-144	146	160	TVVSIPNGPSALAVK					Mascot
1466.6614	1466.5457	-0.1157	-79	26	39	GILAMDESNATCGK			Carbamidomethyl (C)[12]		Mascot
2402.1404	2401.7991	-0.3413	-142	108	130	GLVPLAGSNDESWCQGLDGLASR	107	100	Carbamidomethyl (C)[14]		Mascot
2402.1404	2401.7991	-0.3413	-142	108	130	GLVPLAGSNDESWCQGLDGLASR			Carbamidomethyl (C)[14]		Mascot

Rank	Protein Name	Accession No	Protein MW	Protein PI	Pep. Count	Protein Score	Protein Score C. I. %	Total Ion Score	Total Ion C. I. %
2	Fructose-bisphosphate aldolase 2, chloroplastic OS=Pisum sativum PE=1 SV=2	ALFC2_PEA	37803.4	5.48	8	120	100	83	100

Peptide Information

Calc. Mass	Obsrv. Mass	± da	± ppm	Start Seq.	End Seq.	Sequence	Ion Score	C. I. %	Modification	Rank	Result Type
858.5043	858.4055	-0.0988	-115	292	299	ALQNTALK					Mascot
873.4576	873.3399	-0.1177	-135	155	162	EAAWGLAR	16	0			Mascot
873.4576	873.3399	-0.1177	-135	155	162	EAAWGLAR					Mascot
938.3018	938.4258	0.124	132	342	349	DCCQGYSY					Mascot
1114.5276	1114.3771	-0.1505	-135	125	134	SAAYYQQGAR					Mascot
1114.5276	1114.3771	-0.1505	-135	125	134	SAAYYQQGAR	51	99.911			Mascot
1452.842	1452.6327	-0.2093	-144	140	154	TVVSIPNGPSALAVK					Mascot
1466.6614	1466.5457	-0.1157	-79	20	33	GILAMDESNATCGK			Carbamidomethyl (C)[12]		Mascot
1649.9836	1649.7563	-0.2273	-138	84	98	IVDVLVEQNIIPGIK					Mascot
1649.9836	1649.7563	-0.2273	-138	84	98	IVDVLVEQNIIPGIK	17	0			Mascot
1778.0785	1777.8284	-0.2501	-141	83	98	KIVDVLVEQNIIPGIK					Mascot

Rank	Protein Name	Accession No	Protein MW	Protein PI	Pep. Count	Protein Score	Protein Score C. I. %	Total Ion Score	Total Ion C. I. %
3	Fructose-bisphosphate aldolase, chloroplastic OS=Spinacia oleracea PE=1 SV=3	ALFC_SPIOL	42441.7	6.85	5	98	100	83	100

Peptide Information

Calc. Mass	Obsrv. Mass	± da	± ppm	Start Seq.	End Seq.	Sequence	Ion Score	C. I. %	Modification	Rank	Result Type
873.4576	873.3399	-0.1177	-135	201	208	EAAWGLAR	16	0			Mascot
873.4576	873.3399	-0.1177	-135	201	208	EAAWGLAR					Mascot
1114.5276	1114.3771	-0.1505	-135	171	180	SAAYYQQGAR					Mascot
1114.5276	1114.3771	-0.1505	-135	171	180	SAAYYQQGAR	51	99.911			Mascot
1387.7175	1387.5343	-0.1832	-132	81	93	LASIGLENTEANR	16	0			Mascot
1387.7175	1387.5343	-0.1832	-132	81	93	LASIGLENTEANR					Mascot
1452.842	1452.6327	-0.2093	-144	186	200	TVVSIPNGPSALAVK					Mascot
1466.6614	1466.5457	-0.1157	-79	66	79	GILAMDESNATCGK			Carbamidomethyl (C)[12]		Mascot

Rank	Protein Name	Accession No	Protein MW	Protein PI	Pep. Count	Protein Score	Protein Score C. I. %	Total Ion Score	Total Ion C. I. %
4	Outer envelope protein 61, chloroplastic OS=Arabidopsis thaliana GN=OEP61 PE=1 SV=1	OEP61_ARAT	60717.5	5.66	13	45	0		

Peptide Information

Calc. Mass	Obsrv. Mass	± da	± ppm	Start Seq.	End Seq.	Sequence	Ion Score	C. I. %	Modification	Rank	Result Type
938.4213	938.4258	0.0045	5	117	125	GNFSDAAEK					Mascot
940.4595	940.3464	-0.1131	-120	109	116	QGNELHSR					Mascot
984.5148	984.3933	-0.1215	-123	288	295	TFQNFISK					Mascot
1114.4543	1114.3771	-0.0772	-69	309	318	AGDMSPDMFK			Oxidation (M)[4]		Mascot
1114.4543	1114.3771	-0.0772	-69	309	318	AGDMSPDMFK			Oxidation (M)[4]		Mascot
1245.6321	1245.4724	-0.1597	-128	296	308	TDPDTLAALSGGK					Mascot
1453.6637	1453.6307	-0.033	-23	1	12	MFNGLMDPEMIR					Mascot
1536.7653	1536.5547	-0.2106	-137	168	181	EGSEVLGYDARNVK					Mascot
1882.9617	1882.7009	-0.2608	-139	271	287	TDVDGLQALRDNPEAIR					Mascot
1940.9456	1940.6735	-0.2721	-140	327	343	MSPEEIQKMVQTASSFK					Mascot
2311.1348	2310.8	-0.3348	-145	57	76	QAAEQLKHTRPEDMAEISEK					Mascot
2344.011	2343.7664	-0.2446	-104	29	48	IQQQMMSNPDLMNMATESMK			Oxidation (M)[5]		Mascot
3338.5378	3338.25	-0.2878	-86	344	375	GDNPFAPTAPSTENGFTPTPDMLKLASDMMGK					Mascot
3343.4917	3343.2769	-0.2148	-64	29	56	IQQQMMSNPDLMNMATESMKNMRPEDLK			Oxidation (M)[5,6]		Mascot

Rank	Protein Name	Accession No	Protein MW	Protein PI	Pep. Count	Protein Score	Protein Score C. I. %	Total Ion Score	Total Ion C. I. %
5	Trypsin inhibitor 1 OS=Trichosanthes kirilowii PE SV=1	ITR1_TRIKI	6872.1	4.61	6	43	0		

Peptide Information

Calc. Mass	Obsrv. Mass	± da	± ppm	Start Seq.	End Seq.	Sequence	Ion Score	C. I. %	Modification	Rank	Result Type
850.4417	850.4224	-0.0193	-23	2	9	AAFVESAR					Mascot
873.3229	873.3399	0.017	19	58	65	CLSNGYCG			Carbamidomethyl (C)[1]		Mascot
873.3229	873.3399	0.017	19	58	65	CLSNGYCG			Carbamidomethyl (C)[1]		Mascot
1165.5162	1165.5728	0.0566	49	32	41	MMEGVVACPR			Carbamidomethyl (C)[8], Oxidation (M)[1]		Mascot
1275.4915	1275.5416	0.0501	39	55	65	GCKCLSNGYCG			Carbamidomethyl (C)[2,4,10]		Mascot
1649.7808	1649.7563	-0.0245	-15	42	54	ILMPCKVNDDCLR			Carbamidomethyl (C)[5,11], Oxidation (M)[3]		Mascot
1649.7808	1649.7563	-0.0245	-15	42	54	ILMPCKVNDDCLR			Carbamidomethyl (C)[5,11], Oxidation (M)[3]		Mascot
1777.8654	1777.8284	-0.037	-21	32	47	MMEGVVACPRILMPCK					Mascot
1882.8716	1882.7009	-0.1707	-91	32	47	MMEGVVACPRILMPCK			Carbamidomethyl (C)[8], Oxidation (M)[1,2,13]		Mascot

Rank	Protein Name	Accession No	Protein MW	Protein PI	Pep. Count	Protein Score	Protein Score C. I. %	Total Ion Score	Total Ion C. I. %
6	Fructose-bisphosphate aldolase, chloroplastic OS=Oryza sativa subsp. japonica GN=Os11g0171300 PE=1	ALFC_ORYSJ	41979.5	6.38	4	43	0	33	94.356

Peptide Information

Calc. Mass	Obsrv. Mass	± da	± ppm	Start Seq.	End Seq.	Sequence	Ion Score	C. I. %	Modification	Rank	Result Type
850.4702	850.4224	-0.0478	-56	1	8	MASATLLK			Oxidation (M)[1]		Mascot
873.4576	873.3399	-0.1177	-135	193	200	EAAWGLAR	16	0			Mascot
873.4576	873.3399	-0.1177	-135	193	200	EAAWGLAR					Mascot
1387.7175	1387.5343	-0.1832	-132	73	85	LASIGLENTEANR	16	0			Mascot
1387.7175	1387.5343	-0.1832	-132	73	85	LASIGLENTEANR					Mascot
1466.6614	1466.5457	-0.1157	-79	58	71	GILAMDESNATCGK			Carbamidomethyl (C)[12]		Mascot

Rank	Protein Name	Accession No	Protein MW	Protein PI	Pep. Count	Protein Score	Protein Score C. I. %	Total Ion Score	Total Ion C. I. %
7	Probable fructose-bisphosphate aldolase 1, chloroplastic OS=Arabidopsis thaliana GN=FBA1 PE=1 SV=2	ALFC1_ARAT	42903.9	6.18	4	43	0	33	94.356

Peptide Information

Calc. Mass	Obsrv. Mass	± da	± ppm	Start Seq.	End Seq.	Sequence	Ion Score	C. I. %	Modification	Rank	Result Type
873.4576	873.3399	-0.1177	-135	204	211	EAAWGLAR	16	0			Mascot
873.4576	873.3399	-0.1177	-135	204	211	EAAWGLAR					Mascot
1147.5742	1147.4492	-0.125	-109	349	358	TWGGKEENVK					Mascot
1147.5742	1147.4492	-0.125	-109	349	358	TWGGKEENVK					Mascot
1387.7175	1387.5343	-0.1832	-132	84	96	LASIGLENTEANR	16	0			Mascot
1387.7175	1387.5343	-0.1832	-132	84	96	LASIGLENTEANR					Mascot
1452.842	1452.6327	-0.2093	-144	189	203	TVVSIPNGPSALAVK					Mascot

Rank	Protein Name	Accession No	Protein MW	Protein PI	Pep. Count	Protein Score	Protein Score C. I. %	Total Ion Score	Total Ion C. I. %
8	Putative beta-glucosidase 9 OS=Oryza sativa sub japonica GN=BGLU9 PE=5 SV=2	BGL09_ORYS	35525.5	6.3	7	32	0		

Peptide Information

Calc. Mass	Obsrv. Mass	± da	± ppm	Start Seq.	End Seq.	Sequence	Ion Score	C. I. %	Modification	Rank	Result Type
850.4417	850.4224	-0.0193	-23	193	199	EFGDRVK					Mascot
882.4468	882.4448	-0.002	-2	115	121	FSISWSR					Mascot
889.3906	889.335	-0.0556	-63	100	106	EDVHMMK					Mascot
905.3855	905.3275	-0.058	-64	100	106	EDVHMMK			Oxidation (M)[5]		Mascot
1004.3812	1004.4144	0.0332	33	107	114	EMGMDAYR			Oxidation (M)[2,4]		Mascot
1004.3812	1004.4144	0.0332	33	107	114	EMGMDAYR			Oxidation (M)[2,4]		Mascot
1649.7264	1649.7563	0.0299	18	185	197	DYAEICFKEFGDR			Carbamidomethyl (C)[6]		Mascot
1649.7264	1649.7563	0.0299	18	185	197	DYAEICFKEFGDR			Carbamidomethyl (C)[6]		Mascot
1828.7411	1828.625	-0.1161	-63	225	240	CSPWEMGKCSVGDSGR			Carbamidomethyl (C)[1,9], Oxidation (M)[6]		Mascot
2088.9265	2088.8909	-0.0356	-17	214	232	AYASGTYAPGRCSPWEMGK			Carbamidomethyl (C)[12]		Mascot

Rank	Protein Name	Accession No	Protein MW	Protein PI	Pep. Count	Protein Score	Protein Score C. I. %	Total Ion Score	Total Ion C. I. %
9	Probable fructose-bisphosphate aldolase 2, chloroplastic OS=Arabidopsis thaliana GN=FBA2 PE=1 SV=2	ALFC2_ARAT	42961.1	6.78	5	31	0	16	0

Peptide Information

Calc. Mass	Obsrv. Mass	± da	± ppm	Start Seq.	End Seq.	Sequence	Ion Score	C. I. %	Modification	Rank	Result Type
850.4702	850.4224	-0.0478	-56	1	8	MASTSLLK					Mascot
873.4576	873.3399	-0.1177	-135	203	210	EAAWGLAR	16	0			Mascot
873.4576	873.3399	-0.1177	-135	203	210	EAAWGLAR					Mascot
1452.842	1452.6327	-0.2093	-144	188	202	TVVSIPNGPSALAVK					Mascot
1453.7533	1453.6307	-0.1226	-84	47	60	AASSYADELVKTAK					Mascot
1466.6614	1466.5457	-0.1157	-79	68	81	GILAMDESNATCGK			Carbamidomethyl (C)[12]		Mascot

Rank	Protein Name	Accession No	Protein MW	Protein PI	Pep. Count	Protein Score	Protein Score C. I. %	Total Ion Score	Total Ion C. I. %
10	Protein argonaute 5 OS=Arabidopsis thaliana GN=AGO5 PE=1 SV=2	AGO5_ARATH	111019	9.51	10	30	0	13	0

Peptide Information

Calc. Mass	Obsrv. Mass	± da	± ppm	Start Seq.	End Seq.	Sequence	Ion Score	C. I. %	Modification	Rank	Result Type
842.4518	842.3959	-0.0559	-66	439	444	YNYRVK					Mascot
870.4468	870.4247	-0.0221	-25	332	338	GYFQSLR					Mascot
873.4611	873.3399	-0.1212	-139	548	554	EKMVNPR	13	0			Mascot
873.4611	873.3399	-0.1212	-139	548	554	EKMVNPR					Mascot
889.4559	889.335	-0.1209	-136	548	554	EKMVNPR			Oxidation (M)[3]		Mascot
938.5166	938.4258	-0.0908	-97	752	760	GLVSAQAHR					Mascot
1118.5588	1118.4082	-0.1506	-135	203	212	NYKDSHLGGK					Mascot
1466.7122	1466.5457	-0.1665	-114	85	99	GNIGDPTFSVASSSK					Mascot
1671.8231	1671.7292	-0.0939	-56	490	503	ATCQRPPDRENSIK			Carbamidomethyl (C)[3]		Mascot
2089.134	2088.8909	-0.2431	-116	936	954	SVSIVPPAYYAHLAAFRAR					Mascot
3338.5979	3338.25	-0.3479	-104	113	143	NTEVSETMSNLQITSTETKPEMTSLPPASSK					Mascot
3343.6853	3343.2769	-0.4084	-122	603	631	GMEFKPQPAIPFISCPPEHIEEALLDIHK			Carbamidomethyl (C)[15]		Mascot

Gel Idx/Pos	79/D4	Instr./Gel Origin	BA2060/131017A	Process Sta	Analysis Succeeded
Plate [#] Name	[1] 31496	Instrument Sample Name		Spectra	11

Rank	Protein Name	Accession No	Protein MW	Protein PI	Pep. Count	Protein Score	Protein Score C. I. %	Total Ion Score	Total Ion C. I. %
1	Chloroplast stem-loop binding protein of 41 kDa b chloroplastic OS=Arabidopsis thaliana GN=CSP41B	CP41B_ARAT	42593	8.19	7	170	100	143	100

Peptide Information

Calc. Mass	Obsrv. Mass	± da	± ppm	Start Seq.	End Seq.	Sequence	Ion Score	C. I. %	Modification	Rank	Result Type
860.5022	860.3859	-0.1163	-135	56	63	ILIMGGTR					Mascot
870.4355	870.4268	-0.0087	-10	306	312	EFDFGKK					Mascot
876.4971	876.3939	-0.1032	-118	56	63	ILIMGGTR			Oxidation (M)[4]		Mascot
1147.6205	1147.4513	-0.1692	-147	191	200	LETESLLQSK					Mascot
1187.6167	1187.4675	-0.1492	-126	76	85	EGHQVTLFTR					Mascot
1187.6167	1187.4675	-0.1492	-126	76	85	EGHQVTLFTR	30	87.728			Mascot
1207.5742	1207.413	-0.1612	-133	319	328	DQHFFASVEK					Mascot
1828.8711	1828.6156	-0.2555	-140	124	140	SSLSAEGFDVVYDINGR	113	100			Mascot
1828.8711	1828.6156	-0.2555	-140	124	140	SSLSAEGFDVVYDINGR					Mascot
2031.0433	2030.7479	-0.2954	-145	269	286	EIFNISGEKYVTFDGLAK					Mascot

Rank	Protein Name	Accession No	Protein MW	Protein PI	Pep. Count	Protein Score	Protein Score C. I. %	Total Ion Score	Total Ion C. I. %
2	Ribulose bisphosphate carboxylase large chain (Fragment) OS=Cornus florida GN=rbcL PE=3 SV=2	RBL_CORFO	51593.1	6.14	10	53	82.746	14	0

Peptide Information

Calc. Mass	Obsrv. Mass	± da	± ppm	Start Seq.	End Seq.	Sequence	Ion Score	C. I. %	Modification	Rank	Result Type
910.4451	910.3334	-0.1117	-123	178	184	AVYECLR			Carbamidomethyl (C)[5]		Mascot
1118.4711	1118.4696	-0.0015	-1	457	465	FEFEAMDTL			Oxidation (M)[6]		Mascot
1118.4711	1118.4696	-0.0015	-1	457	465	FEFEAMDTL			Oxidation (M)[6]		Mascot
1245.6433	1245.4674	-0.1759	-141	430	440	EGNEIIREASK					Mascot
1275.7307	1275.5616	-0.1691	-133	330	340	EITLGFVDLLR					Mascot
1465.7546	1465.5551	-0.1995	-136	137	149	TFQGPPHGIQVER					Mascot
1465.7546	1465.5551	-0.1995	-136	137	149	TFQGPPHGIQVER	14	0			Mascot
1475.7351	1475.5583	-0.1768	-120	206	217	DRFLFCAEALYK					Mascot
1828.7662	1828.6156	-0.1506	-82	227	242	GHYLNATAGTCEEMMK			Carbamidomethyl (C)[11], Oxidation (M)[14]		Mascot
1828.7662	1828.6156	-0.1506	-82	227	242	GHYLNATAGTCEEMMK			Carbamidomethyl (C)[11], Oxidation (M)[14]		Mascot
2009.059	2008.7769	-0.2821	-140	330	346	EITLGFVDLLRDDFVEK					Mascot
2185.9819	2185.668	-0.3139	-144	185	203	GGLDFTKDDENVNSQPFMR			Oxidation (M)[18]		Mascot
2683.2161	2682.9138	-0.3023	-113	218	242	AQAETGEIKGHYLNATAGTCEEMMK					Mascot

Rank	Protein Name	Accession No	Protein MW	Protein PI	Pep. Count	Protein Score	Protein Score C. I. %	Total Ion Score	Total Ion C. I. %
3	Ribulose bisphosphate carboxylase large chain (Fragment) OS=Fouquieria splendens GN=rbcL PE=3 SV=1	RBL_FOUSP	48841.6	6.09	9	49	56.659	13	0

Peptide Information

Calc. Mass	Obsrv. Mass	± da	± ppm	Start Seq.	End Seq.	Sequence	Ion Score	C. I. %	Modification	Rank	Result Type
910.4451	910.3334	-0.1117	-123	154	160	AVYECLR			Carbamidomethyl (C)[5]		Mascot
1118.4711	1118.4696	-0.0015	-1	433	441	FEFEAMDTL			Oxidation (M)[6]		Mascot
1118.4711	1118.4696	-0.0015	-1	433	441	FEFEAMDTL			Oxidation (M)[6]		Mascot
1245.6433	1245.4674	-0.1759	-141	406	416	EGNEIIREASK					Mascot
1465.7216	1465.5551	-0.1665	-114	286	300	MSGGDHIHAGTVVGK					Mascot
1465.7546	1465.5551	-0.1995	-136	113	125	TFQGPPHGIQVER	14	0			Mascot
1475.7351	1475.5583	-0.1768	-120	182	193	DRFLFCAEAIYK					Mascot
2009.059	2008.7769	-0.2821	-140	306	322	DITLGFVDLLRDDFLEK					Mascot
2185.9819	2185.668	-0.3139	-144	161	179	GGLDFTKDDENVNSQPFMR			Oxidation (M)[18]		Mascot
2681.2544	2680.9673	-0.2871	-107	194	218	AQAETGEIKGHYLNATAGTCEEMIK			Oxidation (M)[23]		Mascot

Rank	Protein Name	Accession No	Protein MW	Protein PI	Pep. Count	Protein Score	Protein Score C. I. %	Total Ion Score	Total Ion C. I. %
4	Inactive sesquithujene synthase B OS=Zea mays GN=TPS5B PE=2 SV=1	TPS5B_MAIZI	63770	5.17	11	48	49.079		

Peptide Information

Calc. Mass	Obsrv. Mass	± da	± ppm	Start Seq.	End Seq.	Sequence	Ion Score	C. I. %	Modification	Rank	Result Type
882.425	882.4439	0.0189	21	1	8	MASPPAHR			Oxidation (M)[1]		Mascot
1118.6165	1118.4696	-0.1469	-131	450	459	LSNDVVSTKR					Mascot
1118.6165	1118.4696	-0.1469	-131	450	459	LSNDVVSTKR	8	0			Mascot
1147.6028	1147.4513	-0.1515	-132	498	507	DMLEQSLALK					Mascot
1187.511	1187.4675	-0.0435	-37	523	531	TTDNMYRDR			Oxidation (M)[5]		Mascot
1187.511	1187.4675	-0.0435	-37	523	531	TTDNMYRDR			Oxidation (M)[5]		Mascot
1469.7271	1469.6119	-0.1152	-78	217	228	NYIPIYETEATR					Mascot
1475.6559	1475.5583	-0.0976	-66	253	264	HCSAWWNEMIAK					Mascot
1541.8362	1541.6115	-0.2247	-146	152	164	SLLSLYNAAYLWK					Mascot
1690.7828	1690.5822	-0.2006	-119	253	266	HCSAWWNEMIAKSK					Mascot
2540.126	2539.887	-0.239	-94	274	294	IVEEYFWMNGACYDPPYSLSR					Mascot
2807.1528	2806.8909	-0.2619	-93	464	487	DHSPSTVHCYMKEHGITMDDACEK			Carbamidomethyl (C)[9], Oxidation (M)[11]		Mascot
3283.5994	3283.1697	-0.4297	-131	20	48	ASSTFHPSLWGSFFLTYQPPTAPQRANMK			Oxidation (M)[28]		Mascot

Rank	Protein Name	Accession No	Protein MW	Protein PI	Pep. Count	Protein Score	Protein Score C. I. %	Total Ion Score	Total Ion C. I. %
5	Ribulose bisphosphate carboxylase large chain (Fragment) OS=Securidaca diversifolia GN=rbcL PE=3 SV	RBL_SECDI	51600.1	6.23	9	48	42.866	14	0

Peptide Information

Calc. Mass	Obsrv. Mass	± da	± ppm	Start Seq.	End Seq.	Sequence	Ion Score	C. I. %	Modification	Rank	Result Type
860.4658	860.3859	-0.0799	-93	412	419	VALEACVR					Mascot
910.4451	910.3334	-0.1117	-123	178	184	AVYECLR			Carbamidomethyl (C)[5]		Mascot
1245.6433	1245.4674	-0.1759	-141	430	440	EGNEIIREASK					Mascot
1275.7307	1275.5616	-0.1691	-133	330	340	EITLGFVDLLR					Mascot
1465.7546	1465.5551	-0.1995	-136	137	149	TFQGPPHGIQVER					Mascot
1465.7546	1465.5551	-0.1995	-136	137	149	TFQGPPHGIQVER	14	0			Mascot
1475.7351	1475.5583	-0.1768	-120	206	217	DRFLFCAEAIYK					Mascot
2009.059	2008.7769	-0.2821	-140	330	346	EITLGFVDLLRDDFVEK					Mascot
2185.9819	2185.668	-0.3139	-144	185	203	GGLDFTKDDENVNSQPFMR			Oxidation (M)[18]		Mascot
2681.2544	2680.9673	-0.2871	-107	218	242	AQAETGEIKGHYLNATAGTCEEMIK			Oxidation (M)[23]		Mascot

Rank	Protein Name	Accession No	Protein MW	Protein PI	Pep. Count	Protein Score	Protein Score C. I. %	Total Ion Score	Total Ion C. I. %
6	Ribulose bisphosphate carboxylase large chain (Fragment) OS=Quisqualis indica GN=rbcL PE=3 SV=1	RBL_QUIIN	51658	6.13	9	46	13.524	13	0

Peptide Information

Calc. Mass	Obsrv. Mass	± da	± ppm	Start Seq.	End Seq.	Sequence	Ion Score	C. I. %	Modification	Rank	Result Type
910.4451	910.3334	-0.1117	-123	178	184	AVYECLR			Carbamidomethyl (C)[5]		Mascot
1118.4711	1118.4696	-0.0015	-1	457	465	FEFEAMDTL			Oxidation (M)[6]		Mascot
1118.4711	1118.4696	-0.0015	-1	457	465	FEFEAMDTL			Oxidation (M)[6]		Mascot
1245.6433	1245.4674	-0.1759	-141	430	440	EGNEIIREASK					Mascot
1275.7307	1275.5616	-0.1691	-133	330	340	EITLGFVDLLR					Mascot
1465.7216	1465.5551	-0.1665	-114	310	324	MSGGDHIHAGTVVGK					Mascot
1465.7546	1465.5551	-0.1995	-136	137	149	TFQGPPHGIQVER	14	0			Mascot
1518.741	1518.6115	-0.1295	-85	206	217	DRFLFCAEGIYK			Carbamidomethyl (C)[6]		Mascot
2185.9819	2185.668	-0.3139	-144	185	203	GGLDFTKDDENVNSQPFMR			Oxidation (M)[18]		Mascot
2681.2544	2680.9673	-0.2871	-107	218	242	AQAETGEIKGHYLNATAGTCEEMIK			Oxidation (M)[23]		Mascot

Rank	Protein Name	Accession No	Protein MW	Protein PI	Pep. Count	Protein Score	Protein Score C. I. %	Total Ion Score	Total Ion C. I. %
7	Ribulose bisphosphate carboxylase large chain (Fragment) OS=Coriaria myrtifolia GN=rbcL PE=3 SV=1	RBL_CORMY	51666.1	6	9	46	7.339	13	0

Peptide Information

Calc. Mass	Obsrv. Mass	± da	± ppm	Start Seq.	End Seq.	Sequence	Ion Score	C. I. %	Modification	Rank	Result Type
910.4451	910.3334	-0.1117	-123	179	185	AVYECLR			Carbamidomethyl (C)[5]		Mascot
1118.4711	1118.4696	-0.0015	-1	458	466	FEFEAMDTL			Oxidation (M)[6]		Mascot
1118.4711	1118.4696	-0.0015	-1	458	466	FEFEAMDTL			Oxidation (M)[6]		Mascot
1245.6433	1245.4674	-0.1759	-141	431	441	EGNEIIREASK					Mascot
1275.7307	1275.5616	-0.1691	-133	331	341	EITLGFVDLLR					Mascot
1465.7216	1465.5551	-0.1665	-114	311	325	MSGGDHIHAGTVVGK					Mascot
1465.7546	1465.5551	-0.1995	-136	138	150	TFQGPPHGIQVER	14	0			Mascot
1475.7351	1475.5583	-0.1768	-120	207	218	DRFLFCAEAIYK					Mascot
2185.9819	2185.668	-0.3139	-144	186	204	GGLDFTKDDENVNSQPFMR			Oxidation (M)[18]		Mascot
2681.2544	2680.9673	-0.2871	-107	219	243	SQAETGEIKGHYLNATAGTCEEMIK					Mascot
2697.2495	2696.9565	-0.293	-109	219	243	SQAETGEIKGHYLNATAGTCEEMIK			Oxidation (M)[23]		Mascot
2697.2495	2696.9565	-0.293	-109	219	243	SQAETGEIKGHYLNATAGTCEEMIK			Oxidation (M)[23]		Mascot

Rank	Protein Name	Accession No	Protein MW	Protein PI	Pep. Count	Protein Score	Protein Score C. I. %	Total Ion Score	Total Ion C. I. %
8	Ribulose bisphosphate carboxylase large chain (Fragment) OS=Ajuga chamaepitys GN=rbcL PE=3 SV=1	RBL_AJUCH	52421.4	6.23	9	46	7.339	14	0

Peptide Information

Calc. Mass	Obsrv. Mass	± da	± ppm	Start Seq.	End Seq.	Sequence	Ion Score	C. I. %	Modification	Rank	Result Type
856.3546	856.4094	0.0548	64	467	473	FEFPAMD					Mascot
910.4451	910.3334	-0.1117	-123	188	194	AVYECLR			Carbamidomethyl (C)[5]		Mascot
1275.7307	1275.5616	-0.1691	-133	340	350	EITLGFVDLLR					Mascot
1344.6616	1344.5676	-0.094	-70	84	94	CYHIEPVLGEK			Carbamidomethyl (C)[1]		Mascot
1465.7546	1465.5551	-0.1995	-136	147	159	TFQGPPHGIQVER					Mascot
1465.7546	1465.5551	-0.1995	-136	147	159	TFQGPPHGIQVER	14	0			Mascot
1475.7351	1475.5583	-0.1768	-120	216	227	DRFLFCAEAIYK					Mascot
2009.059	2008.7769	-0.2821	-140	340	356	EITLGFVDLLRDDFVEK					Mascot
2185.9819	2185.668	-0.3139	-144	195	213	GGLDFTKDDENVNSQPFMR			Oxidation (M)[18]		Mascot
2681.2544	2680.9673	-0.2871	-107	228	252	AQAETGEIKGHYLNATAGTCEEMIK			Oxidation (M)[23]		Mascot

Rank	Protein Name	Accession No	Protein MW	Protein PI	Pep. Count	Protein Score	Protein Score C. I. %	Total Ion Score	Total Ion C. I. %
9	Ribulose bisphosphate carboxylase large chain (Fragment) OS=Adoxa moschatellina GN=rbcL PE=3 SV=1	RBL_ADOMO	51703	6.13	9	45	0	14	0

Peptide Information

Calc. Mass	Obsrv. Mass	± da	± ppm	Start Seq.	End Seq.	Sequence	Ion Score	C. I. %	Modification	Rank	Result Type
910.4451	910.3334	-0.1117	-123	179	185	AVYECLR			Carbamidomethyl (C)[5]		Mascot
1118.4711	1118.4696	-0.0015	-1	458	466	FEFEAMDTL			Oxidation (M)[6]		Mascot
1118.4711	1118.4696	-0.0015	-1	458	466	FEFEAMDTL			Oxidation (M)[6]		Mascot
1245.6433	1245.4674	-0.1759	-141	431	441	EGNEIIREASK					Mascot
1275.7307	1275.5616	-0.1691	-133	331	341	EITLGFVDLLR					Mascot
1465.7546	1465.5551	-0.1995	-136	138	150	TFQGPPHGIQVER					Mascot
1465.7546	1465.5551	-0.1995	-136	138	150	TFQGPPHGIQVER	14	0			Mascot
1475.7351	1475.5583	-0.1768	-120	207	218	DRFLFCAEALYK					Mascot
1828.7662	1828.6156	-0.1506	-82	228	243	GHYLNATAGTCEEMMK			Carbamidomethyl (C)[11], Oxidation (M)[14]		Mascot
1828.7662	1828.6156	-0.1506	-82	228	243	GHYLNATAGTCEEMMK			Carbamidomethyl (C)[11], Oxidation (M)[14]		Mascot
2185.9819	2185.668	-0.3139	-144	186	204	GGLDFTKDDENVNSQPFMR			Oxidation (M)[18]		Mascot
2683.2161	2682.9138	-0.3023	-113	219	243	AQAETGEIKGHYLNATAGTCEEMMK					Mascot

Rank	Protein Name	Accession No	Protein MW	Protein PI	Pep. Count	Protein Score	Protein Score C. I. %	Total Ion Score	Total Ion C. I. %
10	Ribulose bisphosphate carboxylase large chain (Fragment) OS=Polemonium reptans GN=rbcL PE=3 SV=1	RBL_POLRE	48777.7	6.04	8	45	0	14	0

Peptide Information

Calc. Mass	Obsrv. Mass	± da	± ppm	Start Seq.	End Seq.	Sequence	Ion Score	C. I. %	Modification	Rank	Result Type
910.4451	910.3334	-0.1117	-123	154	160	AVYECLR			Carbamidomethyl (C)[5]		Mascot
1118.4711	1118.4696	-0.0015	-1	433	441	FEFEAMDTL			Oxidation (M)[6]		Mascot
1118.4711	1118.4696	-0.0015	-1	433	441	FEFEAMDTL			Oxidation (M)[6]		Mascot
1245.6433	1245.4674	-0.1759	-141	406	416	EGNEIIREASK					Mascot
1275.7307	1275.5616	-0.1691	-133	306	316	EITLGFVDLLR					Mascot
1465.7546	1465.5551	-0.1995	-136	113	125	TFQGPPHGIQVER					Mascot

1465.7546	1465.5551	-0.1995	-136	113	125	TFQGPPHGIQVER	14	0		Mascot
1475.7351	1475.5583	-0.1768	-120	182	193	DRFLFCAEALYK				Mascot
2185.9819	2185.668	-0.3139	-144	161	179	GGLDFTKDDENVNSQPFMR			Oxidation (M)[18]	Mascot
2681.2544	2680.9673	-0.2871	-107	194	218	AQAETGEIKGHYLNATAGTCEEMIK			Oxidation (M)[23]	Mascot

Gel Idx/Pos	80/D5	Instr./Gel Origin	BA2060/131017A	Process Sta	Analysis Succeeded
Plate [#] Name	[1] 31496	Instrument Sample Name		Spectra	8

Rank	Protein Name	Accession No	Protein MW	Protein PI	Pep. Count	Protein Score	Protein Score C. I. %	Total Ion Score	Total Ion C. I. %
1	Ferredoxin--NADP reductase, leaf isozyme 1, chloroplastic OS=Arabidopsis thaliana GN=LFNR1 PE=1 SV=	FNRL1_ARAT	40300.6	8.32	9	280	100	244	100

Peptide Information

Calc. Mass	Obsrv. Mass	± da	± ppm	Start Seq.	End Seq.	Sequence	Ion Score	C. I. %	Modification	Rank	Result Type
807.4359	807.3295	-0.1064	-132	275	281	LDFAVSR					Mascot
827.408	827.296	-0.112	-135	291	296	MYIQTR			Oxidation (M)[1]		Mascot
856.4345	856.4113	-0.0232	-27	35	41	KSTVCYR					Mascot
1347.6072	1347.4249	-0.1823	-135	311	321	DNTFVYMCGLK	47	99.915	Carbamidomethyl (C)[8]		Mascot
1347.6072	1347.4249	-0.1823	-135	311	321	DNTFVYMCGLK			Carbamidomethyl (C)[8]		Mascot
1363.6021	1363.4185	-0.1836	-135	311	321	DNTFVYMCGLK			Carbamidomethyl (C)[8], Oxidation (M)[7]		Mascot
1379.71	1379.5309	-0.1791	-130	82	93	NPYTGRCLLNTK					Mascot
1475.7021	1475.5212	-0.1809	-123	310	321	KDNTFVYMCGLK			Carbamidomethyl (C)[9]		Mascot
1491.697	1491.4882	-0.2088	-140	310	321	KDNTFVYMCGLK			Carbamidomethyl (C)[9], Oxidation (M)[8]		Mascot
1630.7959	1630.5713	-0.2246	-138	140	155	LYSIASSAIGDFGDSK	73	100			Mascot
1630.7959	1630.5713	-0.2246	-138	140	155	LYSIASSAIGDFGDSK					Mascot
1751.7802	1751.656	-0.1242	-71	311	325	DNTFVYMCGLKGMEK			Oxidation (M)[7]		Mascot
1945.0211	1944.7522	-0.2689	-138	206	224	DPNATIIMLGTGTGIAPFR					Mascot
1945.0211	1944.7522	-0.2689	-138	206	224	DPNATIIMLGTGTGIAPFR	124	100			Mascot
1961.016	1960.7435	-0.2725	-139	206	224	DPNATIIMLGTGTGIAPFR			Oxidation (M)[8]		Mascot

Rank	Protein Name	Accession No	Protein MW	Protein PI	Pep. Count	Protein Score	Protein Score C. I. %	Total Ion Score	Total Ion C. I. %
2	Ferredoxin--NADP reductase, chloroplastic OS=V faba GN=PETH PE=2 SV=1	FENR_VICFA	40552.9	8.7	11	233	100	181	100

Peptide Information

Calc. Mass	Obsrv. Mass	± da	± ppm	Start Seq.	End Seq.	Sequence	Ion Score	C. I. %	Modification	Rank	Result Type
807.4359	807.3295	-0.1064	-132	278	284	LDFAVSR					Mascot
827.408	827.296	-0.112	-135	294	299	MYIQTR			Oxidation (M)[1]		Mascot
1265.6161	1265.4403	-0.1758	-139	354	363	KAEQWNVEVY					Mascot
1347.6072	1347.4249	-0.1823	-135	314	324	DNTFVYMCGLK	47	99.915	Carbamidomethyl (C)[8]		Mascot
1347.6072	1347.4249	-0.1823	-135	314	324	DNTFVYMCGLK			Carbamidomethyl (C)[8]		Mascot
1363.6021	1363.4185	-0.1836	-135	314	324	DNTFVYMCGLK			Carbamidomethyl (C)[8], Oxidation (M)[7]		Mascot
1378.6864	1378.5334	-0.153	-111	228	237	SFLWKMFFEK			Oxidation (M)[6]		Mascot
1427.7377	1427.5319	-0.2058	-144	121	134	EGQSIGIVPDGIDK					Mascot
1475.7021	1475.5212	-0.1809	-123	313	324	KDNTFVYMCGLK			Carbamidomethyl (C)[9]		Mascot
1491.697	1491.4882	-0.2088	-140	313	324	KDNTFVYMCGLK			Carbamidomethyl (C)[9], Oxidation (M)[8]		Mascot
1623.8087	1623.5828	-0.2259	-139	300	312	MAQYAEELWELLK					Mascot
1630.7959	1630.5713	-0.2246	-138	143	158	LYSIASSAIGDFGDSK	73	100			Mascot
1630.7959	1630.5713	-0.2246	-138	143	158	LYSIASSAIGDFGDSK					Mascot
1639.8036	1639.5774	-0.2262	-138	300	312	MAQYAEELWELLK			Oxidation (M)[1]		Mascot
1751.7802	1751.656	-0.1242	-71	314	328	DNTFVYMCGLKGMEK			Oxidation (M)[7]		Mascot
2708.2297	2707.8469	-0.3828	-141	97	120	ITGDDAPGETWHMVFTTEGEVPYR					Mascot
2708.2297	2707.8469	-0.3828	-141	97	120	ITGDDAPGETWHMVFTTEGEVPYR	61	99.997			Mascot
2724.2246	2723.8503	-0.3743	-137	97	120	ITGDDAPGETWHMVFTTEGEVPYR			Oxidation (M)[13]		Mascot

Rank	Protein Name	Accession No	Protein MW	Protein PI	Pep. Count	Protein Score	Protein Score C. I. %	Total Ion Score	Total Ion C. I. %
3	Ferredoxin--NADP reductase, leaf isozyme, chloroplastic OS=Pisum sativum GN=PETH PE=1 SV=1	FENR1_PEA	40168.6	8.56	10	163	100	120	100

Peptide Information

Calc. Mass	Obsrv. Mass	± da	± ppm	Start Seq.	End Seq.	Sequence	Ion Score	C. I. %	Modification	Rank	Result Type
807.4359	807.3295	-0.1064	-132	275	281	LDFAVSR					Mascot
827.408	827.296	-0.112	-135	291	296	MYIQTR			Oxidation (M)[1]		Mascot
1265.6161	1265.4403	-0.1758	-139	351	360	KAEQWNVEVY					Mascot
1347.6072	1347.4249	-0.1823	-135	311	321	DNTFVYMCGLK	47	99.915	Carbamidomethyl (C)[8]		Mascot
1347.6072	1347.4249	-0.1823	-135	311	321	DNTFVYMCGLK			Carbamidomethyl (C)[8]		Mascot
1363.6021	1363.4185	-0.1836	-135	311	321	DNTFVYMCGLK			Carbamidomethyl (C)[8], Oxidation (M)[7]		Mascot
1378.6864	1378.5334	-0.153	-111	225	234	SFLWKMFFEK			Oxidation (M)[6]		Mascot
1427.7377	1427.5319	-0.2058	-144	118	131	EGQSIGIVPDGIDK					Mascot
1475.7021	1475.5212	-0.1809	-123	310	321	KDNTFVYMCGLK			Carbamidomethyl (C)[9]		Mascot
1491.697	1491.4882	-0.2088	-140	310	321	KDNTFVYMCGLK			Carbamidomethyl (C)[9], Oxidation (M)[8]		Mascot
1623.8087	1623.5828	-0.2259	-139	297	309	MAQYAEELWELLK					Mascot
1630.7959	1630.5713	-0.2246	-138	140	155	LYSIASSAIGDFGDSK	73	100			Mascot
1630.7959	1630.5713	-0.2246	-138	140	155	LYSIASSAIGDFGDSK					Mascot
1639.8036	1639.5774	-0.2262	-138	297	309	MAQYAEELWELLK			Oxidation (M)[1]		Mascot
1751.7802	1751.656	-0.1242	-71	311	325	DNTFVYMCGLKGMEK			Oxidation (M)[7]		Mascot

Rank	Protein Name	Accession No	Protein MW	Protein PI	Pep. Count	Protein Score	Protein Score C. I. %	Total Ion Score	Total Ion C. I. %
4	Ferredoxin--NADP reductase, chloroplastic OS=Spinacia oleracea GN=PETH PE=1 SV=1	FENR_SPIOL	41161.9	8.67	6	138	100	124	100

Peptide Information

Calc. Mass	Obsrv. Mass	± da	± ppm	Start Seq.	End Seq.	Sequence	Ion Score	C. I. %	Modification	Rank	Result Type
807.4359	807.3295	-0.1064	-132	284	290	LDFAVSR					Mascot
827.408	827.296	-0.112	-135	300	305	MYIQTR			Oxidation (M)[1]		Mascot
1265.6161	1265.4403	-0.1758	-139	360	369	KAEQWNVEVY					Mascot
1378.6864	1378.5334	-0.153	-111	234	243	SFLWKMFFEK			Oxidation (M)[6]		Mascot
1427.597	1427.5319	-0.0651	-46	320	330	DNTYFYMCGLK			Carbamidomethyl (C)[8], Oxidation (M)[7]		Mascot
1945.0211	1944.7522	-0.2689	-138	215	233	DPNATIIMLGTGTGIAPFR					Mascot
1945.0211	1944.7522	-0.2689	-138	215	233	DPNATIIMLGTGTGIAPFR	124	100			Mascot
1961.016	1960.7435	-0.2725	-139	215	233	DPNATIIMLGTGTGIAPFR			Oxidation (M)[8]		Mascot

Rank	Protein Name	Accession No	Protein MW	Protein PI	Pep. Count	Protein Score	Protein Score C. I. %	Total Ion Score	Total Ion C. I. %
5	Ferredoxin--NADP reductase, chloroplastic (Fragn OS=Populus euphratica GN=PETH PE=1 SV=1	FENR_POPEU	1944	5.91	1	128	100	124	100

Peptide Information

Calc. Mass	Obsrv. Mass	± da	± ppm	Start Seq.	End Seq.	Sequence	Ion Score	C. I. %	Modification	Rank	Result Type
1945.0211	1944.7522	-0.2689	-138	1	19	DPNATIIMLGTGTGIAPFR					Mascot
1945.0211	1944.7522	-0.2689	-138	1	19	DPNATIIMLGTGTGIAPFR	124	100			Mascot
1961.016	1960.7435	-0.2725	-139	1	19	DPNATIIMLGTGTGIAPFR			Oxidation (M)[8]		Mascot

Rank	Protein Name	Accession No	Protein MW	Protein PI	Pep. Count	Protein Score	Protein Score C. I. %	Total Ion Score	Total Ion C. I. %
6	Ferredoxin--NADP reductase, leaf isozyme 2, chloroplastic OS=Arabidopsis thaliana GN=LFNR2 PE=1 SV=	FNRL2_ARAT	41141.7	8.51	7	125	100	102	100

Peptide Information

Calc. Mass	Obsrv. Mass	± da	± ppm	Start Seq.	End Seq.	Sequence	Ion Score	C. I. %	Modification	Rank	Result Type
827.408	827.296	-0.112	-135	300	305	MYIQTR			Oxidation (M)[1]		Mascot
1265.6161	1265.4403	-0.1758	-139	360	369	KAEQWNVEVY					Mascot
1347.6072	1347.4249	-0.1823	-135	320	330	DNTFVYMCGLK	47	99.915	Carbamidomethyl (C)[8]		Mascot
1347.6072	1347.4249	-0.1823	-135	320	330	DNTFVYMCGLK			Carbamidomethyl (C)[8]		Mascot
1363.6021	1363.4185	-0.1836	-135	320	330	DNTFVYMCGLK			Carbamidomethyl (C)[8], Oxidation (M)[7]		Mascot
1378.6864	1378.5334	-0.153	-111	234	243	SFLWKMFFEK			Oxidation (M)[6]		Mascot
1475.7021	1475.5212	-0.1809	-123	319	330	KDNTFVYMCGLK			Carbamidomethyl (C)[9]		Mascot
1491.697	1491.4882	-0.2088	-140	319	330	KDNTFVYMCGLK			Carbamidomethyl (C)[9], Oxidation (M)[8]		Mascot
1751.7802	1751.656	-0.1242	-71	320	334	DNTFVYMCGLKGMEK			Oxidation (M)[7]		Mascot
1945.0211	1944.7522	-0.2689	-138	215	233	DPNATVIMLATGTGIAPFR					Mascot
1945.0211	1944.7522	-0.2689	-138	215	233	DPNATVIMLATGTGIAPFR	55	99.986			Mascot
1961.016	1960.7435	-0.2725	-139	215	233	DPNATVIMLATGTGIAPFR			Oxidation (M)[8]		Mascot

Rank	Protein Name	Accession No	Protein MW	Protein PI	Pep. Count	Protein Score	Protein Score C. I. %	Total Ion Score	Total Ion C. I. %
7	Ferredoxin--NADP reductase, leaf isozyme, chloroplastic OS=Oryza sativa subsp. japonica GN=Os06g010	FENR1_ORYS	39982.3	8.72	6	88	99.995	73	100

Peptide Information

Calc. Mass	Obsrv. Mass	± da	± ppm	Start Seq.	End Seq.	Sequence	Ion Score	C. I. %	Modification	Rank	Result Type
827.408	827.296	-0.112	-135	293	298	MYIQTR			Oxidation (M)[1]		Mascot
1187.667	1187.5071	-0.1599	-135	304	312	EELWELLKK					Mascot
1378.6864	1378.5334	-0.153	-111	227	236	SFLWKMFFEK			Oxidation (M)[6]		Mascot
1465.6814	1465.5568	-0.1246	-85	293	303	MYIQTRMAEYK			Oxidation (M)[1,7]		Mascot
1534.8224	1534.6079	-0.2145	-140	165	177	RLVYTNDQGEIVK					Mascot
1630.7959	1630.5713	-0.2246	-138	142	157	LYSIASSALGDFGDSK	73	100			Mascot
1630.7959	1630.5713	-0.2246	-138	142	157	LYSIASSALGDFGDSK					Mascot

Rank	Protein Name	Accession No	Protein MW	Protein PI	Pep. Count	Protein Score	Protein Score C. I. %	Total Ion Score	Total Ion C. I. %
8	Ferredoxin--NADP reductase, leaf-type isozyme, chloroplastic OS=Nicotiana tabacum GN=PETH PE=2 SV=1	FENR1_TOBA	40419.5	8.37	5	67	99.264	55	99.986

Peptide Information

Calc. Mass	Obsrv. Mass	± da	± ppm	Start Seq.	End Seq.	Sequence	Ion Score	C. I. %	Modification	Rank	Result Type
807.4359	807.3295	-0.1064	-132	277	283	LDFAVSR					Mascot
827.408	827.296	-0.112	-135	293	298	MYIQTR			Oxidation (M)[1]		Mascot
1265.6161	1265.4403	-0.1758	-139	353	362	KAEQWNVEVY					Mascot
1378.6864	1378.5334	-0.153	-111	227	236	SFLWKMFFEK			Oxidation (M)[6]		Mascot
1945.0211	1944.7522	-0.2689	-138	208	226	DPNATVIMLATGTGIAPFR					Mascot
1945.0211	1944.7522	-0.2689	-138	208	226	DPNATVIMLATGTGIAPFR	55	99.986			Mascot
1961.016	1960.7435	-0.2725	-139	208	226	DPNATVIMLATGTGIAPFR			Oxidation (M)[8]		Mascot

Rank	Protein Name	Accession No	Protein MW	Protein PI	Pep. Count	Protein Score	Protein Score C. I. %	Total Ion Score	Total Ion C. I. %
9	Ribulose bisphosphate carboxylase large chain (Fragment) OS=Sinapis alba GN=rbcL PE=3 SV=1	RBL_SINAL	52405.4	6.23	13	65	98.911		

Peptide Information

Calc. Mass	Obsrv. Mass	± da	± ppm	Start Seq.	End Seq.	Sequence	Ion Score	C. I. %	Modification	Rank	Result Type
856.3546	856.4113	0.0567	66	467	473	FEFPAMD					Mascot
898.41	898.2878	-0.1222	-136	306	312	NHGMHFR					Mascot
910.4451	910.3186	-0.1265	-139	188	194	AVYECLR			Carbamidomethyl (C)[5]		Mascot
914.4049	914.2817	-0.1232	-135	306	312	NHGMHFR			Oxidation (M)[4]		Mascot
1116.583	1116.4259	-0.1571	-141	422	431	VALEACVQAR			Carbamidomethyl (C)[6]		Mascot
1170.5585	1170.4816	-0.0769	-66	304	312	QKNHGMHFR			Oxidation (M)[6]		Mascot
1187.6643	1187.5071	-0.1572	-132	286	295	DNGLLLHIHR					Mascot
1245.6433	1245.4607	-0.1826	-147	440	450	EGNEIIREASK					Mascot
1275.7307	1275.5573	-0.1734	-136	340	350	EITLGFVDLLR					Mascot
1379.7166	1379.5309	-0.1857	-135	2	14	SPQTETKASVGFK					Mascot
1465.7546	1465.5568	-0.1978	-135	147	159	TFQGPPHGIQVER					Mascot
1475.7351	1475.5212	-0.2139	-145	216	227	DRFLFCAEAIYK					Mascot
2169.9868	2169.6851	-0.3017	-139	195	213	GGLDFTKDDENVNSQPFMR					Mascot
2185.9819	2185.6763	-0.3056	-140	195	213	GGLDFTKDDENVNSQPFMR			Oxidation (M)[18]		Mascot
3022.4548	3022.0261	-0.4287	-142	259	285	ELGVPIVMHDYLTGGFTANTSLAHYCR			Carbamidomethyl (C)[26]		Mascot

Rank	Protein Name	Accession No	Protein MW	Protein PI	Pep. Count	Protein Score	Protein Score C. I. %	Total Ion Score	Total Ion C. I. %
10	Ribulose bisphosphate carboxylase large chain (Fragment) OS=Tamarindus indica GN=rbcL PE=3 SV=1	RBL_TAMIN	50497.5	6.13	12	60	97.002		

Peptide Information

Calc. Mass	Obsrv. Mass	± da	± ppm	Start Seq.	End Seq.	Sequence	Ion Score	C. I. %	Modification	Rank	Result Type
898.41	898.2878	-0.1222	-136	297	303	NHGMHFR					Mascot
910.4451	910.3186	-0.1265	-139	179	185	AVYECLR			Carbamidomethyl (C)[5]		Mascot
914.4049	914.2817	-0.1232	-135	297	303	NHGMHFR			Oxidation (M)[4]		Mascot
1116.583	1116.4259	-0.1571	-141	413	422	VALEACVQAR			Carbamidomethyl (C)[6]		Mascot
1170.5585	1170.4816	-0.0769	-66	295	303	QKNHGMHFR			Oxidation (M)[6]		Mascot
1187.6643	1187.5071	-0.1572	-132	277	286	DNGLLLHIHR					Mascot
1245.6433	1245.4607	-0.1826	-147	431	441	EGNEIIREASK					Mascot
1275.7307	1275.5573	-0.1734	-136	331	341	EITLGFVDLLR					Mascot
1465.7546	1465.5568	-0.1978	-135	138	150	TFQGPPHGIQVER					Mascot
1475.7351	1475.5212	-0.2139	-145	207	218	DRFLFCAEAIYK					Mascot
2169.9868	2169.6851	-0.3017	-139	186	204	GGLDFTKDDENVNSQPFMR					Mascot
2185.9819	2185.6763	-0.3056	-140	186	204	GGLDFTKDDENVNSQPFMR			Oxidation (M)[18]		Mascot
2748.3298	2747.9236	-0.4062	-148	219	243	AQAETGEIKGHYLNATAGTWEEMIK					Mascot
3022.4548	3022.0261	-0.4287	-142	250	276	ELGVPIVMHDYLTGGFTANTSLAHYCR			Carbamidomethyl (C)[26]		Mascot

Gel Idx/Pos	81/D6	Instr./Gel Origin	BA2060/131017A	Process Sta	Analysis Succeeded
Plate [#] Name	[1] 31496	Instrument Sample Name		Spectra	8

Rank	Protein Name	Accession No	Protein MW	Protein PI	Pep. Count	Protein Score	Protein Score C. I. %	Total Ion Score	Total Ion C. I. %
1	Ferredoxin--NADP reductase, leaf isozyme 1, chloroplastic OS=Arabidopsis thaliana GN=LFNR1 PE=1 SV=	FNRL1_ARAT	40300.6	8.32	9	249	100	213	100

Peptide Information

Calc. Mass	Obsrv. Mass	± da	± ppm	Start Seq.	End Seq.	Sequence	Ion Score	C. I. %	Modification	Rank	Result Type
807.4359	807.3297	-0.1062	-132	275	281	LDFAVSR					Mascot
827.408	827.2991	-0.1089	-132	291	296	MYIQTR			Oxidation (M)[1]		Mascot
856.4345	856.4114	-0.0231	-27	35	41	KSTVCYR					Mascot
1232.6555	1232.4863	-0.1692	-137	326	337	GIDDIMVSLAAK					Mascot
1248.6504	1248.4778	-0.1726	-138	326	337	GIDDIMVSLAAK			Oxidation (M)[6]		Mascot
1347.6072	1347.4222	-0.185	-137	311	321	DNTFVYMCGLK			Carbamidomethyl (C)[8]		Mascot
1363.6021	1363.4147	-0.1874	-137	311	321	DNTFVYMCGLK			Carbamidomethyl (C)[8], Oxidation (M)[7]		Mascot
1475.7021	1475.5079	-0.1942	-132	310	321	KDNTFVYMCGLK			Carbamidomethyl (C)[9]		Mascot
1491.697	1491.4922	-0.2048	-137	310	321	KDNTFVYMCGLK			Carbamidomethyl (C)[9], Oxidation (M)[8]		Mascot
1630.7959	1630.5697	-0.2262	-139	140	155	LYSIASSAIGDFGDSK					Mascot
1630.7959	1630.5697	-0.2262	-139	140	155	LYSIASSAIGDFGDSK	81	100			Mascot
1751.7802	1751.6619	-0.1183	-68	311	325	DNTFVYMCGLKGMEK			Oxidation (M)[7]		Mascot
1767.775	1767.6522	-0.1228	-69	311	325	DNTFVYMCGLKGMEK			Oxidation (M)[7,13]		Mascot
1945.0211	1944.749	-0.2721	-140	206	224	DPNATIIMLGTGTGIAPFR	131	100			Mascot
1945.0211	1944.749	-0.2721	-140	206	224	DPNATIIMLGTGTGIAPFR					Mascot
1961.016	1960.7418	-0.2742	-140	206	224	DPNATIIMLGTGTGIAPFR			Oxidation (M)[8]		Mascot

Rank	Protein Name	Accession No	Protein MW	Protein PI	Pep. Count	Protein Score	Protein Score C. I. %	Total Ion Score	Total Ion C. I. %
2	Ferredoxin--NADP reductase, chloroplastic OS=V faba GN=PETH PE=2 SV=1	FENR_VICFA	40552.9	8.7	11	242	100	190	100

Peptide Information

Calc. Mass	Obsrv. Mass	± da	± ppm	Start Seq.	End Seq.	Sequence	Ion Score	C. I. %	Modification	Rank	Result Type
807.4359	807.3297	-0.1062	-132	278	284	LDFAVSR					Mascot
827.408	827.2991	-0.1089	-132	294	299	MYIQTR			Oxidation (M)[1]		Mascot
1265.6161	1265.4435	-0.1726	-136	354	363	KAEQWNVEVY					Mascot
1265.6161	1265.4435	-0.1726	-136	354	363	KAEQWNVEVY	48	99.918			Mascot
1347.6072	1347.4222	-0.185	-137	314	324	DNTFVYMCGLK			Carbamidomethyl (C)[8]		Mascot
1363.6021	1363.4147	-0.1874	-137	314	324	DNTFVYMCGLK			Carbamidomethyl (C)[8], Oxidation (M)[7]		Mascot
1378.6864	1378.5302	-0.1562	-113	228	237	SFLWKMFFEK			Oxidation (M)[6]		Mascot
1427.7377	1427.5363	-0.2014	-141	121	134	EGQSIGIVPDGIDK					Mascot
1475.7021	1475.5079	-0.1942	-132	313	324	KDNTFVYMCGLK			Carbamidomethyl (C)[9]		Mascot
1491.697	1491.4922	-0.2048	-137	313	324	KDNTFVYMCGLK			Carbamidomethyl (C)[9], Oxidation (M)[8]		Mascot
1623.8087	1623.5801	-0.2286	-141	300	312	MAQYAEELWELLK					Mascot
1630.7959	1630.5697	-0.2262	-139	143	158	LYSIASSAIGDFGDSK					Mascot
1630.7959	1630.5697	-0.2262	-139	143	158	LYSIASSAIGDFGDSK	81	100			Mascot
1639.8036	1639.5745	-0.2291	-140	300	312	MAQYAEELWELLK			Oxidation (M)[1]		Mascot
1751.7802	1751.6619	-0.1183	-68	314	328	DNTFVYMCGLKGMEK			Oxidation (M)[7]		Mascot
1767.775	1767.6522	-0.1228	-69	314	328	DNTFVYMCGLKGMEK			Oxidation (M)[7,13]		Mascot
2708.2297	2707.8462	-0.3835	-142	97	120	ITGDDAPGETWHMVFTTEGEVPYR	61	99.996			Mascot
2708.2297	2707.8462	-0.3835	-142	97	120	ITGDDAPGETWHMVFTTEGEVPYR					Mascot
2724.2246	2723.8494	-0.3752	-138	97	120	ITGDDAPGETWHMVFTTEGEVPYR			Oxidation (M)[13]		Mascot

Rank	Protein Name	Accession No	Protein MW	Protein PI	Pep. Count	Protein Score	Protein Score C. I. %	Total Ion Score	Total Ion C. I. %
3	Ferredoxin--NADP reductase, chloroplastic OS=Spinacia oleracea GN=PETH PE=1 SV=1	FENR_SPIOL	41161.9	8.67	6	194	100	179	100

Peptide Information

Calc. Mass	Obsrv. Mass	± da	± ppm	Start Seq.	End Seq.	Sequence	Ion Score	C. I. %	Modification	Rank	Result Type
807.4359	807.3297	-0.1062	-132	284	290	LDFAVSR					Mascot
827.408	827.2991	-0.1089	-132	300	305	MYIQTR			Oxidation (M)[1]		Mascot
1265.6161	1265.4435	-0.1726	-136	360	369	KAEQWNVEVY					Mascot
1265.6161	1265.4435	-0.1726	-136	360	369	KAEQWNVEVY	48	99.918			Mascot
1378.6864	1378.5302	-0.1562	-113	234	243	SFLWKMFFEK			Oxidation (M)[6]		Mascot
1427.597	1427.5363	-0.0607	-43	320	330	DNTYFYMCGLK			Carbamidomethyl (C)[8], Oxidation (M)[7]		Mascot
1945.0211	1944.749	-0.2721	-140	215	233	DPNATIIMLGTGTGIAPFR	131	100			Mascot
1945.0211	1944.749	-0.2721	-140	215	233	DPNATIIMLGTGTGIAPFR					Mascot
	1944.749										Mascot
1961.016	1960.7418	-0.2742	-140	215	233	DPNATIIMLGTGTGIAPFR			Oxidation (M)[8]		Mascot

Rank	Protein Name	Accession No	Protein MW	Protein PI	Pep. Count	Protein Score	Protein Score C. I. %	Total Ion Score	Total Ion C. I. %
4	Ferredoxin--NADP reductase, leaf isozyme, chloroplastic OS=Pisum sativum GN=PETH PE=1 SV=1	FENR1_PEA	40168.6	8.56	11	181	100	129	100

Peptide Information

Calc. Mass	Obsrv. Mass	± da	± ppm	Start Seq.	End Seq.	Sequence	Ion Score	C. I. %	Modification	Rank	Result Type
807.4359	807.3297	-0.1062	-132	275	281	LDFAVSR					Mascot
827.408	827.2991	-0.1089	-132	291	296	MYIQTR			Oxidation (M)[1]		Mascot
1232.6555	1232.4863	-0.1692	-137	326	337	GIDDIMVSLAAK					Mascot
1248.6504	1248.4778	-0.1726	-138	326	337	GIDDIMVSLAAK			Oxidation (M)[6]		Mascot
1265.6161	1265.4435	-0.1726	-136	351	360	KAEQWNVEVY					Mascot
1265.6161	1265.4435	-0.1726	-136	351	360	KAEQWNVEVY	48	99.918			Mascot
1347.6072	1347.4222	-0.185	-137	311	321	DNTFVYMCGLK			Carbamidomethyl (C)[8]		Mascot
1363.6021	1363.4147	-0.1874	-137	311	321	DNTFVYMCGLK			Carbamidomethyl (C)[8], Oxidation (M)[7]		Mascot
1378.6864	1378.5302	-0.1562	-113	225	234	SFLWKMFFEK			Oxidation (M)[6]		Mascot
1427.7377	1427.5363	-0.2014	-141	118	131	EGQSIGIVPDGIDK					Mascot
1475.7021	1475.5079	-0.1942	-132	310	321	KDNTFVYMCGLK			Carbamidomethyl (C)[9]		Mascot
1491.697	1491.4922	-0.2048	-137	310	321	KDNTFVYMCGLK			Carbamidomethyl (C)[9], Oxidation (M)[8]		Mascot
1623.8087	1623.5801	-0.2286	-141	297	309	MAQYAEELWELLK					Mascot
1630.7959	1630.5697	-0.2262	-139	140	155	LYSIASSAIGDFGDSK					Mascot
1630.7959	1630.5697	-0.2262	-139	140	155	LYSIASSAIGDFGDSK	81	100			Mascot
1639.8036	1639.5745	-0.2291	-140	297	309	MAQYAEELWELLK			Oxidation (M)[1]		Mascot
1751.7802	1751.6619	-0.1183	-68	311	325	DNTFVYMCGLKGMEK			Oxidation (M)[7]		Mascot
1767.775	1767.6522	-0.1228	-69	311	325	DNTFVYMCGLKGMEK			Oxidation (M)[7,13]		Mascot

Rank	Protein Name	Accession No	Protein MW	Protein PI	Pep. Count	Protein Score	Protein Score C. I. %	Total Ion Score	Total Ion C. I. %
5	Ferredoxin--NADP reductase, chloroplastic (Fragn OS=Populus euphratica GN=PETH PE=1 SV=1	FENR_POPEU	1944	5.91	1	136	100	131	100

Peptide Information

Calc. Mass	Obsrv. Mass	± da	± ppm	Start Seq.	End Seq.	Sequence	Ion Score	C. I. %	Modification	Rank	Result Type
1945.0211	1944.749	-0.2721	-140	1	19	DPNATIIMLGTGTGIAPFR	131	100			Mascot
1945.0211	1944.749	-0.2721	-140	1	19	DPNATIIMLGTGTGIAPFR					Mascot
1961.016	1960.7418	-0.2742	-140	1	19	DPNATIIMLGTGTGIAPFR			Oxidation (M)[8]		Mascot

Rank	Protein Name	Accession No	Protein MW	Protein PI	Pep. Count	Protein Score	Protein Score C. I. %	Total Ion Score	Total Ion C. I. %
6	Ferredoxin--NADP reductase, leaf isozyme 2, chloroplastic OS=Arabidopsis thaliana GN=LFNR2 PE=1 SV=	FNRL2_ARAT	41141.7	8.51	7	120	100	97	100

Peptide Information

Calc. Mass	Obsrv. Mass	± da	± ppm	Start Seq.	End Seq.	Sequence	Ion Score	C. I. %	Modification	Rank	Result Type
827.408	827.2991	-0.1089	-132	300	305	MYIQTR			Oxidation (M)[1]		Mascot
1265.6161	1265.4435	-0.1726	-136	360	369	KAEQWNVEVY					Mascot
1265.6161	1265.4435	-0.1726	-136	360	369	KAEQWNVEVY	48	99.918			Mascot
1347.6072	1347.4222	-0.185	-137	320	330	DNTFVYMCGLK			Carbamidomethyl (C)[8]		Mascot
1363.6021	1363.4147	-0.1874	-137	320	330	DNTFVYMCGLK			Carbamidomethyl (C)[8], Oxidation (M)[7]		Mascot
1378.6864	1378.5302	-0.1562	-113	234	243	SFLWKMFFEK			Oxidation (M)[6]		Mascot
1475.7021	1475.5079	-0.1942	-132	319	330	KDNTFVYMCGLK			Carbamidomethyl (C)[9]		Mascot
1491.697	1491.4922	-0.2048	-137	319	330	KDNTFVYMCGLK			Carbamidomethyl (C)[9], Oxidation (M)[8]		Mascot
1751.7802	1751.6619	-0.1183	-68	320	334	DNTFVYMCGLKGMEK			Oxidation (M)[7]		Mascot
1767.775	1767.6522	-0.1228	-69	320	334	DNTFVYMCGLKGMEK			Oxidation (M)[7,13]		Mascot
1945.0211	1944.749	-0.2721	-140	215	233	DPNATVIMLATGTGIAPFR	50	99.947			Mascot
1945.0211	1944.749	-0.2721	-140	215	233	DPNATVIMLATGTGIAPFR					Mascot
1961.016	1960.7418	-0.2742	-140	215	233	DPNATVIMLATGTGIAPFR			Oxidation (M)[8]		Mascot

Rank	Protein Name	Accession No	Protein MW	Protein PI	Pep. Count	Protein Score	Protein Score C. I. %	Total Ion Score	Total Ion C. I. %
7	Ferredoxin--NADP reductase, leaf-type isozyme, chloroplastic OS=Nicotiana tabacum GN=PETH PE=2 SV=1	FENR1_TOBA	40419.5	8.37	5	109	100	97	100

Peptide Information

Calc. Mass	Obsrv. Mass	± da	± ppm	Start Seq.	End Seq.	Sequence	Ion Score	C. I. %	Modification	Rank	Result Type
807.4359	807.3297	-0.1062	-132	277	283	LDFAVSR					Mascot
827.408	827.2991	-0.1089	-132	293	298	MYIQTR			Oxidation (M)[1]		Mascot
1265.6161	1265.4435	-0.1726	-136	353	362	KAEQWNVEVY					Mascot
1265.6161	1265.4435	-0.1726	-136	353	362	KAEQWNVEVY	48	99.918			Mascot
1378.6864	1378.5302	-0.1562	-113	227	236	SFLWKMFFEK			Oxidation (M)[6]		Mascot
1945.0211	1944.749	-0.2721	-140	208	226	DPNATVIMLATGTGIAPFR	50	99.947			Mascot
1945.0211	1944.749	-0.2721	-140	208	226	DPNATVIMLATGTGIAPFR					Mascot
1961.016	1960.7418	-0.2742	-140	208	226	DPNATVIMLATGTGIAPFR			Oxidation (M)[8]		Mascot

Rank	Protein Name	Accession No	Protein MW	Protein PI	Pep. Count	Protein Score	Protein Score C. I. %	Total Ion Score	Total Ion C. I. %
8	Ferredoxin--NADP reductase, leaf isozyme, chloroplastic OS=Oryza sativa subsp. japonica GN=Os06g010	FENR1_ORYS	39982.3	8.72	7	101	100	81	100

Peptide Information

Calc. Mass	Obsrv. Mass	± da	± ppm	Start Seq.	End Seq.	Sequence	Ion Score	C. I. %	Modification	Rank	Result Type
Calc. Mass	Obsrv. Mass	± da	± ppm	Seq.	Seq.	Sequence	Score	C. I. %	Modification	Rank	Result Type
827.408	827.2991	-0.1089	-132	293	298	MYIQTR			Oxidation (M)[1]		Mascot
1187.667	1187.5001	-0.1669	-141	304	312	EELWELLKK					Mascot
1232.6555	1232.4863	-0.1692	-137	328	339	GIDDIMVSLAAK					Mascot
1248.6504	1248.4778	-0.1726	-138	328	339	GIDDIMVSLAAK			Oxidation (M)[6]		Mascot
1378.6864	1378.5302	-0.1562	-113	227	236	SFLWKMFFEK			Oxidation (M)[6]		Mascot
1465.6814	1465.5558	-0.1256	-86	293	303	MYIQTRMAEYK			Oxidation (M)[1,7]		Mascot
1534.8224	1534.6107	-0.2117	-138	165	177	RLVYTNDQGEIVK					Mascot
1630.7959	1630.5697	-0.2262	-139	142	157	LYSIASSALGDFGDSK					Mascot
1630.7959	1630.5697	-0.2262	-139	142	157	LYSIASSALGDFGDSK	81	100			Mascot

Rank	Protein Name	Accession No	Protein MW	Protein PI	Pep. Count	Protein Score	Protein Score C. I. %	Total Ion Score	Total Ion C. I. %
9	Ferredoxin--NADP reductase, chloroplastic OS=Mesembryanthemum crystallinum GN=PETH PE=2 SV=1	FENR_MESCR	41036.9	8.54	9	82	99.977	48	99.918

Peptide Information

Calc. Mass	Obsrv. Mass	± da	± ppm	Start Seq.	End Seq.	Sequence	Ion Score	C. I. %	Modification	Rank	Result Type
807.4359	807.3297	-0.1062	-132	280	286	LDFAVSR					Mascot
827.408	827.2991	-0.1089	-132	296	301	MYIQTR			Oxidation (M)[1]		Mascot
1265.6161	1265.4435	-0.1726	-136	356	365	KAEQWNVEVY					Mascot
1265.6161	1265.4435	-0.1726	-136	356	365	KAEQWNVEVY	48	99.918			Mascot
1363.6021	1363.4147	-0.1874	-137	316	326	DNTYVYMCGLK			Carbamidomethyl (C)[8]		Mascot
1378.6864	1378.5302	-0.1562	-113	230	239	SFLWKMFFEK			Oxidation (M)[6]		Mascot
1427.7377	1427.5363	-0.2014	-141	123	136	EGQSVGVIPEGIDK					Mascot
1491.697	1491.4922	-0.2048	-137	315	326	KDNTYVYMCGLK			Carbamidomethyl (C)[9]		Mascot
1534.8224	1534.6107	-0.2117	-138	168	180	RLIYTNDNGEIVK					Mascot
1751.7802	1751.6619	-0.1183	-68	316	330	DNTYVYMCGLKGMEK					Mascot
1767.775	1767.6522	-0.1228	-69	316	330	DNTYVYMCGLKGMEK			Oxidation (M)[7]		Mascot

Rank	Protein Name	Accession No	Protein MW	Protein PI	Pep. Count	Protein Score	Protein Score C. I. %	Total Ion Score	Total Ion C. I. %
10	18.1 kDa class I heat shock protein OS=Oryza sat subsp. japonica GN=HSP18.1 PE=2 SV=1	HS181_ORYS	18071.2	6.77	5	31	0		

Peptide Information

Calc. Mass	Obsrv. Mass	± da	± ppm	Start Seq.	End Seq.	Sequence	Ion Score	C. I. %	Modification	Rank	Result Type
842.4267	842.4009	-0.0258	-31	103	108	TDKWHR					Mascot
882.4692	882.449	-0.0202	-23	106	111	WHRVER					Mascot
1767.866	1767.6522	-0.2138	-121	43	59	GTSSETAAFAGARIDWK					Mascot
1944.9946	1944.749	-0.2456	-126	133	150	ASMENGVLTVTVPKEEPK			Oxidation (M)[3]		Mascot
1944.9946	1944.749	-0.2456	-126	133	150	ASMENGVLTVTVPKEEPK			Oxidation (M)[3]		Mascot
2723.4648	2723.8494	0.3846	141	122	146	LPENTKPEQIKASMENGVLTVTVPK					Mascot
2739.4597	2739.843	0.3833	140	122	146	LPENTKPEQIKASMENGVLTVTVPK			Oxidation (M)[14]		Mascot

Gel Idx/Pos	82/D7	Instr./Gel Origin	BA2060/131017A	Process Sta	Analysis Succeeded
Plate [#] Name	[1] 31496	Instrument Sample Name		Spectra	11

Re Result Type

Rank	Protein Name	Accession No	Protein MW	Protein PI	Pep. Count	Protein Score	Protein Score C. I. %	Total Ion Score	Total Ion C. I. %
1	ATP synthase subunit beta, chloroplastic OS=Gl max GN=atpB PE=3 SV=1	ATPB_SOYBI	53721.1	5.29	13	186	100	112	100

Peptide Information

Calc. Mass	Obsrv. Mass	± da	± ppm	Start Seq.	End Seq.	Sequence	Ion Score	C. I. %	Modification	Rank	Result Type
1245.5126	1245.4664	-0.0462	-37	208	217	EGNDLYMEMK			Oxidation (M)[7]		Mascot
1275.7129	1275.5601	-0.1528	-120	40	50	MPNIYNALIVK					Mascot
1275.7129	1275.5601	-0.1528	-120	40	50	MPNIYNALIVK					Mascot
1431.6863	1431.4924	-0.1939	-135	379	390	IVGEEHYETAQR					Mascot
1433.7747	1433.5828	-0.1919	-134	278	291	FVQAGSEVSALLGR					Mascot
1433.7747	1433.5828	-0.1919	-134	278	291	FVQAGSEVSALLGR	16	0			Mascot
1471.7614	1471.5635	-0.1979	-134	249	261	VGLTALTMAEYFR					Mascot
1487.7562	1487.559	-0.1972	-133	249	261	VGLTALTMAEYFR			Oxidation (M)[8]		Mascot
1487.7562	1487.559	-0.1972	-133	249	261	VGLTALTMAEYFR			Oxidation (M)[8]		Mascot
1502.6614	1502.5814	-0.08	-53	206	217	TREGNDLYMEMK			Oxidation (M)[9]		Mascot
1601.8104	1601.5908	-0.2196	-137	232	246	VALVYGQMNEPPGAR					Mascot
1601.8104	1601.5908	-0.2196	-137	232	246	VALVYGQMNEPPGAR	13	0			Mascot
1617.8053	1617.5864	-0.2189	-135	232	246	VALVYGQMNEPPGAR			Oxidation (M)[8]		Mascot
1949.9967	1949.734	-0.2627	-135	262	277	DVNEQDVLLFIDNIFR	50	99.893			Mascot
1949.9967	1949.734	-0.2627	-135	262	277	DVNEQDVLLFIDNIFR					Mascot
2061.0322	2060.7493	-0.2829	-137	360	378	GIYPAVDPLDSTSTMLQPR					Mascot
2077.0271	2076.7378	-0.2893	-139	360	378	GIYPAVDPLDSTSTMLQPR	14	0	Oxidation (M)[15]		Mascot
2077.0271	2076.7378	-0.2893	-139	360	378	GIYPAVDPLDSTSTMLQPR			Oxidation (M)[15]		Mascot
2282.0791	2281.7708	-0.3083	-135	292	312	MPSAVGYQPTLSTEMGSLQER					Mascot
2282.0791	2281.7708	-0.3083	-135	292	312	MPSAVGYQPTLSTEMGSLQER					Mascot
2298.074	2297.7598	-0.3142	-137	292	312	MPSAVGYQPTLSTEMGSLQER			Oxidation (M)[1]		Mascot
2314.0688	2313.79	-0.2788	-120	292	312	MPSAVGYQPTLSTEMGSLQER			Oxidation (M)[1,15]		Mascot
2462.2661	2461.9116	-0.3545	-144	398	418	YKELQDIIAILGLDELSEEDR					Mascot
2876.4927	2876.0928	-0.3999	-139	460	486	LILSGELDGLPEQAFYLVGNIDEATAK					Mascot
3843.9287	3843.354	-0.5747	-150	318	354	EGSITSIQAVYVPADDLTDPAPATTFAHLDATTVLSR	19	0			Mascot
3843.9287	3843.354	-0.5747	-150	318	354	EGSITSIQAVYVPADDLTDPAPATTFAHLDATTVLSR					Mascot

Rank	Protein Name	Accession No	Protein MW	Protein PI	Pep. Count	Protein Score	Protein Score C. I. %	Total Ion Score	Total Ion C. I. %
2	ATP synthase subunit beta, chloroplastic OS=Whiteheadia bifolia GN=atpB PE=3 SV=1	ATPB_WHIBI	53745	5.28	13	185	100	112	100

Peptide Information

Calc. Mass	Obsrv. Mass	± da	± ppm	Start Seq.	End Seq.	Sequence	Ion Score	C. I. %	Modification	Rank	Result Type
1245.5126	1245.4664	-0.0462	-37	208	217	EGNDLYMEMK			Oxidation (M)[7]		Mascot
1431.6863	1431.4924	-0.1939	-135	379	390	IVGEEHYETAQR					Mascot
1433.7747	1433.5828	-0.1919	-134	278	291	FVQAGSEVSALLGR					Mascot
1433.7747	1433.5828	-0.1919	-134	278	291	FVQAGSEVSALLGR	16	0			Mascot
1471.7614	1471.5635	-0.1979	-134	249	261	VGLTALTMAEYFR					Mascot
1475.7787	1475.5725	-0.2062	-140	40	52	MPNNYNALVVKGR					Mascot
1487.7562	1487.559	-0.1972	-133	249	261	VGLTALTMAEYFR			Oxidation (M)[8]		Mascot
1487.7562	1487.559	-0.1972	-133	249	261	VGLTALTMAEYFR			Oxidation (M)[8]		Mascot
1502.6614	1502.5814	-0.08	-53	206	217	TREGNDLYMEMK			Oxidation (M)[9]		Mascot
1601.8104	1601.5908	-0.2196	-137	232	246	VALVYGQMNEPPGAR					Mascot
1601.8104	1601.5908	-0.2196	-137	232	246	VALVYGQMNEPPGAR	13	0			Mascot
1617.8053	1617.5864	-0.2189	-135	232	246	VALVYGQMNEPPGAR			Oxidation (M)[8]		Mascot
1949.9967	1949.734	-0.2627	-135	262	277	DVNEQDVLLFIDNIFR	50	99.893			Mascot
1949.9967	1949.734	-0.2627	-135	262	277	DVNEQDVLLFIDNIFR					Mascot
2061.0322	2060.7493	-0.2829	-137	360	378	GIYPAVDPLDSTSTMLQPR					Mascot
2077.0271	2076.7378	-0.2893	-139	360	378	GIYPAVDPLDSTSTMLQPR	14	0	Oxidation (M)[15]		Mascot
2077.0271	2076.7378	-0.2893	-139	360	378	GIYPAVDPLDSTSTMLQPR			Oxidation (M)[15]		Mascot
2282.0791	2281.7708	-0.3083	-135	292	312	MPSAVGYQPTLSTEMGSLQER					Mascot
2282.0791	2281.7708	-0.3083	-135	292	312	MPSAVGYQPTLSTEMGSLQER					Mascot
2298.074	2297.7598	-0.3142	-137	292	312	MPSAVGYQPTLSTEMGSLQER			Oxidation (M)[1]		Mascot
2314.0688	2313.79	-0.2788	-120	292	312	MPSAVGYQPTLSTEMGSLQER			Oxidation (M)[1,15]		Mascot
2462.2661	2461.9116	-0.3545	-144	398	418	YKELQDIIAILGLDELSEEDR					Mascot
3022.7161	3023.0056	0.2895	96	23	50	IAQLIGPVLDVVFPLGKMPNNYNALVVK					Mascot
3843.9287	3843.354	-0.5747	-150	318	354	EGSITSIQAVYVPADDLTDPAPATTFAHLDATTVLSR	19	0			Mascot
3843.9287	3843.354	-0.5747	-150	318	354	EGSITSIQAVYVPADDLTDPAPATTFAHLDATTVLSR					Mascot

Rank	Protein Name	Accession No	Protein MW	Protein PI	Pep. Count	Protein Score	Protein Score C. I. %	Total Ion Score	Total Ion C. I. %
3	ATP synthase subunit beta, chloroplastic OS=Sa zalacca GN=atpB PE=3 SV=1	ATPB_SALZ	53753	5.16	13	183	100	110	100

Peptide Information

Calc. Mass	Obsrv. Mass	± da	± ppm	Start Seq.	End Seq.	Sequence	Ion Score	C. I. %	Modification	Rank	Result Type
1245.5126	1245.4664	-0.0462	-37	208	217	EGNDLYMEMK			Oxidation (M)[7]		Mascot
1431.6863	1431.4924	-0.1939	-135	379	390	IVGEEHYETAQR					Mascot
1433.7747	1433.5828	-0.1919	-134	278	291	FVQAGSEVSALLGR					Mascot
1433.7747	1433.5828	-0.1919	-134	278	291	FVQAGSEVSALLGR	16	0			Mascot
1471.7614	1471.5635	-0.1979	-134	249	261	VGLTALTMAEYFR					Mascot
1475.7675	1475.5725	-0.195	-132	40	52	MPNIYNALVVEGR					Mascot
1487.7562	1487.559	-0.1972	-133	249	261	VGLTALTMAEYFR			Oxidation (M)[8]		Mascot
1487.7562	1487.559	-0.1972	-133	249	261	VGLTALTMAEYFR			Oxidation (M)[8]		Mascot
1502.6614	1502.5814	-0.08	-53	206	217	TREGNDLYMEMK			Oxidation (M)[9]		Mascot
1601.8104	1601.5908	-0.2196	-137	232	246	VALVYGQMNEPPGAR					Mascot
1601.8104	1601.5908	-0.2196	-137	232	246	VALVYGQMNEPPGAR	13	0			Mascot
1617.8053	1617.5864	-0.2189	-135	232	246	VALVYGQMNEPPGAR			Oxidation (M)[8]		Mascot
1949.9967	1949.734	-0.2627	-135	262	277	DVNEQDVLLFIDNIFR	50	99.893			Mascot
1949.9967	1949.734	-0.2627	-135	262	277	DVNEQDVLLFIDNIFR					Mascot
2061.0322	2060.7493	-0.2829	-137	360	378	GIYPAVDPLDSTSTMLQPR					Mascot
2077.0271	2076.7378	-0.2893	-139	360	378	GIYPAVDPLDSTSTMLQPR	14	0	Oxidation (M)[15]		Mascot
2077.0271	2076.7378	-0.2893	-139	360	378	GIYPAVDPLDSTSTMLQPR			Oxidation (M)[15]		Mascot
2282.0791	2281.7708	-0.3083	-135	292	312	MPSAVGYQPTLSTEMGSLQER					Mascot
2282.0791	2281.7708	-0.3083	-135	292	312	MPSAVGYQPTLSTEMGSLQER					Mascot
2298.074	2297.7598	-0.3142	-137	292	312	MPSAVGYQPTLSTEMGSLQER			Oxidation (M)[1]		Mascot
2314.0688	2313.79	-0.2788	-120	292	312	MPSAVGYQPTLSTEMGSLQER			Oxidation (M)[1,15]		Mascot
2462.2661	2461.9116	-0.3545	-144	398	418	YKELQDIIAILGLDELSEEDR					Mascot
3842.9275	3843.354	0.4265	111	40	73	MPNIYNALVVEGRDTVGQQINVTCEVQQLLGNNR			Carbamidomethyl (C)[24]		Mascot
3843.9287	3843.354	-0.5747	-150	318	354	EGSITSIQAVYVPADDLTDPAPATTFAHLDATTVLSR	19	0			Mascot

Rank	Protein Name	Accession No	Protein MW	Protein PI	Pep. Count	Protein Score	Protein Score C. I. %	Total Ion Score	Total Ion C. I. %
4	ATP synthase subunit beta, chloroplastic OS=La sativa GN=atpB PE=3 SV=1	ATPB_LACS	53532.8	5.13	13	183	100	112	100

Peptide Information

Calc. Mass	Obsrv. Mass	± da	± ppm	Start Seq.	End Seq.	Sequence	Ion Score	C. I. %	Modification	Rank	Result Type
1245.5126	1245.4664	-0.0462	-37	208	217	EGNDLYMEMK			Oxidation (M)[7]		Mascot
1295.597	1295.4653	-0.1317	-102	487	497	AMNLEMESNLK			Oxidation (M)[2]		Mascot
1433.7747	1433.5828	-0.1919	-134	278	291	FVQAGSEVSALLGR					Mascot
1433.7747	1433.5828	-0.1919	-134	278	291	FVQAGSEVSALLGR	16	0			Mascot
1471.7614	1471.5635	-0.1979	-134	249	261	VGLTALTMAEYFR					Mascot
1487.7562	1487.559	-0.1972	-133	249	261	VGLTALTMAEYFR			Oxidation (M)[8]		Mascot
1487.7562	1487.559	-0.1972	-133	249	261	VGLTALTMAEYFR			Oxidation (M)[8]		Mascot
1502.6614	1502.5814	-0.08	-53	206	217	TREGNDLYMEMK			Oxidation (M)[9]		Mascot
1524.7211	1524.6193	-0.1018	-67	3	17	MNPTTSGSGVTTLDK			Oxidation (M)[1]		Mascot
1601.8104	1601.5908	-0.2196	-137	232	246	VALVYGQMNEPPGAR					Mascot
1601.8104	1601.5908	-0.2196	-137	232	246	VALVYGQMNEPPGAR	13	0			Mascot
1617.7755	1617.5864	-0.1891	-117	379	392	IVGEEHYDTAQEVK					Mascot
1949.9967	1949.734	-0.2627	-135	262	277	DVNEQDVLLFIDNIFR	50	99.893			Mascot
1949.9967	1949.734	-0.2627	-135	262	277	DVNEQDVLLFIDNIFR					Mascot
2061.0322	2060.7493	-0.2829	-137	360	378	GIYPAVDPLDSTSTMLQPR					Mascot
2077.0271	2076.7378	-0.2893	-139	360	378	GIYPAVDPLDSTSTMLQPR	14	0	Oxidation (M)[15]		Mascot
2077.0271	2076.7378	-0.2893	-139	360	378	GIYPAVDPLDSTSTMLQPR			Oxidation (M)[15]		Mascot
2282.0791	2281.7708	-0.3083	-135	292	312	MPSAVGYQPTLSTEMGSLQER					Mascot
2282.0791	2281.7708	-0.3083	-135	292	312	MPSAVGYQPTLSTEMGSLQER					Mascot
2298.074	2297.7598	-0.3142	-137	292	312	MPSAVGYQPTLSTEMGSLQER			Oxidation (M)[1]		Mascot
2314.0688	2313.79	-0.2788	-120	292	312	MPSAVGYQPTLSTEMGSLQER			Oxidation (M)[1,15]		Mascot
2462.2661	2461.9116	-0.3545	-144	398	418	YKELQDIIAILGLDELSEEDR					Mascot
3843.9287	3843.354	-0.5747	-150	318	354	EGSITSIQAVYVPADDLTDPAPATTFAHLDATTVLSR	19	0			Mascot
3843.9287	3843.354	-0.5747	-150	318	354	EGSITSIQAVYVPADDLTDPAPATTFAHLDATTVLSR					Mascot

Rank	Protein Name	Accession No	Protein MW	Protein PI	Pep. Count	Protein Score	Protein Score C. I. %	Total Ion Score	Total Ion C. I. %
5	ATP synthase subunit beta, chloroplastic OS=Sp oleracea GN=atpB PE=1 SV=2	ATPB_SPIOL	53710.9	5.22	12	176	100	112	100

Peptide Information

Calc. Mass	Obsrv. Mass	± da	± ppm	Start Seq.	End Seq.	Sequence	Ion Score	C. I. %	Modification	Rank	Result Type
1245.5126	1245.4664	-0.0462	-37	208	217	EGNDLYMEMK			Oxidation (M)[7]		Mascot
1275.7129	1275.5601	-0.1528	-120	40	50	MPNIYNALIVK					Mascot
1275.7129	1275.5601	-0.1528	-120	40	50	MPNIYNALIVK					Mascot
1433.7747	1433.5828	-0.1919	-134	278	291	FVQAGSEVSALLGR					Mascot
1433.7747	1433.5828	-0.1919	-134	278	291	FVQAGSEVSALLGR	16	0			Mascot
1471.7614	1471.5635	-0.1979	-134	249	261	VGLTALTMAEYFR					Mascot
1487.7562	1487.559	-0.1972	-133	249	261	VGLTALTMAEYFR			Oxidation (M)[8]		Mascot
1487.7562	1487.559	-0.1972	-133	249	261	VGLTALTMAEYFR			Oxidation (M)[8]		Mascot
1502.6614	1502.5814	-0.08	-53	206	217	TREGNDLYMEMK			Oxidation (M)[9]		Mascot
1601.8104	1601.5908	-0.2196	-137	232	246	VALVYGQMNEPPGAR					Mascot
1601.8104	1601.5908	-0.2196	-137	232	246	VALVYGQMNEPPGAR	13	0			Mascot
1617.8053	1617.5864	-0.2189	-135	232	246	VALVYGQMNEPPGAR			Oxidation (M)[8]		Mascot
1949.9967	1949.734	-0.2627	-135	262	277	DVNEQDVLLFIDNIFR	50	99.893			Mascot
1949.9967	1949.734	-0.2627	-135	262	277	DVNEQDVLLFIDNIFR					Mascot
2061.0322	2060.7493	-0.2829	-137	360	378	GIYPAVDPLDSTSTMLQPR					Mascot
2077.0271	2076.7378	-0.2893	-139	360	378	GIYPAVDPLDSTSTMLQPR	14	0	Oxidation (M)[15]		Mascot
2077.0271	2076.7378	-0.2893	-139	360	378	GIYPAVDPLDSTSTMLQPR			Oxidation (M)[15]		Mascot
2282.0791	2281.7708	-0.3083	-135	292	312	MPSAVGYQPTLSTEMGSLQER					Mascot
2282.0791	2281.7708	-0.3083	-135	292	312	MPSAVGYQPTLSTEMGSLQER					Mascot
2298.074	2297.7598	-0.3142	-137	292	312	MPSAVGYQPTLSTEMGSLQER			Oxidation (M)[1]		Mascot
2314.0688	2313.79	-0.2788	-120	292	312	MPSAVGYQPTLSTEMGSLQER			Oxidation (M)[1,15]		Mascot
2462.2661	2461.9116	-0.3545	-144	398	418	YKELQDIIAILGLDELSEEDR					Mascot
2543.2454	2542.9248	-0.3206	-126	53	75	DTAGQPMNVTCEVQQLLGNNRVR					Mascot
3843.9287	3843.354	-0.5747	-150	318	354	EGSITSIQAVYVPADDLTDPAPATTFAHLDATTVLSR	19	0			Mascot
3843.9287	3843.354	-0.5747	-150	318	354	EGSITSIQAVYVPADDLTDPAPATTFAHLDATTVLSR					Mascot

Rank	Protein Name	Accession No	Protein MW	Protein PI	Pep. Count	Protein Score	Protein Score C. I. %	Total Ion Score	Total Ion C. I. %
6	ATP synthase subunit beta, chloroplastic OS=Ca sativa GN=atpB PE=3 SV=1	ATPB_CASS	53794	5.37	12	176	100	112	100

Peptide Information

Calc. Mass	Obsrv. Mass	± da	± ppm	Start Seq.	End Seq.	Sequence	Ion Score	C. I. %	Modification	Rank	Result Type
1245.5126	1245.4664	-0.0462	-37	208	217	EGNDLYMEMK			Oxidation (M)[7]		Mascot
1431.6863	1431.4924	-0.1939	-135	379	390	IVGEEHYETAQR					Mascot
1433.7747	1433.5828	-0.1919	-134	278	291	FVQAGSEVSALLGR					Mascot
1433.7747	1433.5828	-0.1919	-134	278	291	FVQAGSEVSALLGR	16	0			Mascot
1471.7614	1471.5635	-0.1979	-134	249	261	VGLTALTMAEYFR					Mascot
1487.7562	1487.559	-0.1972	-133	249	261	VGLTALTMAEYFR			Oxidation (M)[8]		Mascot
1487.7562	1487.559	-0.1972	-133	249	261	VGLTALTMAEYFR			Oxidation (M)[8]		Mascot
1502.6614	1502.5814	-0.08	-53	206	217	TREGNDLYMEMK			Oxidation (M)[9]		Mascot
1601.8104	1601.5908	-0.2196	-137	232	246	VALVYGQMNEPPGAR					Mascot
1601.8104	1601.5908	-0.2196	-137	232	246	VALVYGQMNEPPGAR	13	0			Mascot
1617.8053	1617.5864	-0.2189	-135	232	246	VALVYGQMNEPPGAR			Oxidation (M)[8]		Mascot
1949.9967	1949.734	-0.2627	-135	262	277	DVNEQDVLLFIDNIFR	50	99.893			Mascot
1949.9967	1949.734	-0.2627	-135	262	277	DVNEQDVLLFIDNIFR					Mascot
2061.0322	2060.7493	-0.2829	-137	360	378	GIYPAVDPLDSTSTMLQPR					Mascot
2077.0271	2076.7378	-0.2893	-139	360	378	GIYPAVDPLDSTSTMLQPR	14	0	Oxidation (M)[15]		Mascot
2077.0271	2076.7378	-0.2893	-139	360	378	GIYPAVDPLDSTSTMLQPR			Oxidation (M)[15]		Mascot
2282.0791	2281.7708	-0.3083	-135	292	312	MPSAVGYQPTLSTEMGSLQER					Mascot
2282.0791	2281.7708	-0.3083	-135	292	312	MPSAVGYQPTLSTEMGSLQER					Mascot
2298.074	2297.7598	-0.3142	-137	292	312	MPSAVGYQPTLSTEMGSLQER			Oxidation (M)[1]		Mascot
2314.0688	2313.79	-0.2788	-120	292	312	MPSAVGYQPTLSTEMGSLQER			Oxidation (M)[1,15]		Mascot
2462.2661	2461.9116	-0.3545	-144	398	418	YKELQDIIAILGLDELSEEDR					Mascot
2876.4927	2876.0928	-0.3999	-139	460	486	LILSGELDGLPEQAFYLVGNIDEATAK					Mascot
3843.9287	3843.354	-0.5747	-150	318	354	EGSITSIQAVYVPADDLTDPAPATTFAHLDATTVLSR	19	0			Mascot
3843.9287	3843.354	-0.5747	-150	318	354	EGSITSIQAVYVPADDLTDPAPATTFAHLDATTVLSR					Mascot

Rank	Protein Name	Accession No	Protein MW	Protein PI	Pep. Count	Protein Score	Protein Score C. I. %	Total Ion Score	Total Ion C. I. %
7	ATP synthase subunit beta, chloroplastic OS=Lo japonicus GN=atpB PE=3 SV=1	ATPB_LOTJA	53779.2	5.37	12	176	100	112	100

Peptide Information

Calc. Mass	Obsrv. Mass	± da	± ppm	Start Seq.	End Seq.	Sequence	Ion Score	C. I. %	Modification	Rank	Result Type
1245.5126	1245.4664	-0.0462	-37	208	217	EGNDLYMEMK			Oxidation (M)[7]		Mascot
1275.7129	1275.5601	-0.1528	-120	40	50	MPNIYNALIVK					Mascot
1275.7129	1275.5601	-0.1528	-120	40	50	MPNIYNALIVK					Mascot
1431.6863	1431.4924	-0.1939	-135	379	390	IVGEEHYETAQR					Mascot
1433.7747	1433.5828	-0.1919	-134	278	291	FVQAGSEVSALLGR					Mascot
1433.7747	1433.5828	-0.1919	-134	278	291	FVQAGSEVSALLGR	16	0			Mascot
1471.7614	1471.5635	-0.1979	-134	249	261	VGLTALTMAEYFR					Mascot
1487.7562	1487.559	-0.1972	-133	249	261	VGLTALTMAEYFR			Oxidation (M)[8]		Mascot
1487.7562	1487.559	-0.1972	-133	249	261	VGLTALTMAEYFR			Oxidation (M)[8]		Mascot
1502.6614	1502.5814	-0.08	-53	206	217	TREGNDLYMEMK			Oxidation (M)[9]		Mascot
1601.8104	1601.5908	-0.2196	-137	232	246	VALVYGQMNEPPGAR					Mascot
1601.8104	1601.5908	-0.2196	-137	232	246	VALVYGQMNEPPGAR	13	0			Mascot
1617.8053	1617.5864	-0.2189	-135	232	246	VALVYGQMNEPPGAR			Oxidation (M)[8]		Mascot
1949.9967	1949.734	-0.2627	-135	262	277	DVNEQDVLLFIDNIFR	50	99.893			Mascot
1949.9967	1949.734	-0.2627	-135	262	277	DVNEQDVLLFIDNIFR					Mascot
2061.0322	2060.7493	-0.2829	-137	360	378	GIYPAVDPLDSTSTMLQPR					Mascot
2077.0271	2076.7378	-0.2893	-139	360	378	GIYPAVDPLDSTSTMLQPR	14	0	Oxidation (M)[15]		Mascot
2077.0271	2076.7378	-0.2893	-139	360	378	GIYPAVDPLDSTSTMLQPR			Oxidation (M)[15]		Mascot
2282.0791	2281.7708	-0.3083	-135	292	312	MPSAVGYQPTLSTEMGSLQER					Mascot
2282.0791	2281.7708	-0.3083	-135	292	312	MPSAVGYQPTLSTEMGSLQER					Mascot
2298.074	2297.7598	-0.3142	-137	292	312	MPSAVGYQPTLSTEMGSLQER			Oxidation (M)[1]		Mascot
2314.0688	2313.79	-0.2788	-120	292	312	MPSAVGYQPTLSTEMGSLQER			Oxidation (M)[1,15]		Mascot
2462.2661	2461.9116	-0.3545	-144	398	418	YKELQDIIAILGLDELSEEDR					Mascot
3843.9287	3843.354	-0.5747	-150	318	354	EGSITSIQAVYVPADDLTDPAPATTFAHLDATTVLSR	19	0			Mascot
3843.9287	3843.354	-0.5747	-150	318	354	EGSITSIQAVYVPADDLTDPAPATTFAHLDATTVLSR					Mascot

Rank	Protein Name	Accession No	Protein MW	Protein PI	Pep. Count	Protein Score	Protein Score C. I. %	Total Ion Score	Total Ion C. I. %
8	ATP synthase subunit beta, chloroplastic OS=Bo volubilis GN=atpB PE=3 SV=2	ATPB_BOWV	53341.8	5.35	12	176	100	112	100

Peptide Information

Calc. Mass	Obsrv. Mass	± da	± ppm	Start Seq.	End Seq.	Sequence	Ion Score	C. I. %	Modification	Rank	Result Type
1245.5126	1245.4664	-0.0462	-37	208	217	EGNDLYMEMK			Oxidation (M)[7]		Mascot
1431.6863	1431.4924	-0.1939	-135	379	390	IVGEEHYETAQR					Mascot
1433.7747	1433.5828	-0.1919	-134	278	291	FVQAGSEVSALLGR					Mascot
1433.7747	1433.5828	-0.1919	-134	278	291	FVQAGSEVSALLGR	16	0			Mascot
1471.7614	1471.5635	-0.1979	-134	249	261	VGLTALTMAEYFR					Mascot
1487.7562	1487.559	-0.1972	-133	249	261	VGLTALTMAEYFR			Oxidation (M)[8]		Mascot
1487.7562	1487.559	-0.1972	-133	249	261	VGLTALTMAEYFR			Oxidation (M)[8]		Mascot
1502.6614	1502.5814	-0.08	-53	206	217	TREGNDLYMEMK			Oxidation (M)[9]		Mascot
1524.7509	1524.6193	-0.1316	-86	76	90	AVAMSATDGLTRGMK			Oxidation (M)[4]		Mascot
1601.8104	1601.5908	-0.2196	-137	232	246	VALVYGQMNEPPGAR					Mascot
1601.8104	1601.5908	-0.2196	-137	232	246	VALVYGQMNEPPGAR	13	0			Mascot
1617.8053	1617.5864	-0.2189	-135	232	246	VALVYGQMNEPPGAR			Oxidation (M)[8]		Mascot
1949.9967	1949.734	-0.2627	-135	262	277	DVNEQDVLLFIDNIFR	50	99.893			Mascot
1949.9967	1949.734	-0.2627	-135	262	277	DVNEQDVLLFIDNIFR					Mascot
2061.0322	2060.7493	-0.2829	-137	360	378	GIYPAVDPLDSTSTMLQPR					Mascot
2077.0271	2076.7378	-0.2893	-139	360	378	GIYPAVDPLDSTSTMLQPR	14	0	Oxidation (M)[15]		Mascot
2077.0271	2076.7378	-0.2893	-139	360	378	GIYPAVDPLDSTSTMLQPR			Oxidation (M)[15]		Mascot
2282.0791	2281.7708	-0.3083	-135	292	312	MPSAVGYQPTLSTEMGSLQER					Mascot
2282.0791	2281.7708	-0.3083	-135	292	312	MPSAVGYQPTLSTEMGSLQER					Mascot
2298.074	2297.7598	-0.3142	-137	292	312	MPSAVGYQPTLSTEMGSLQER			Oxidation (M)[1]		Mascot
2314.0688	2313.79	-0.2788	-120	292	312	MPSAVGYQPTLSTEMGSLQER			Oxidation (M)[1,15]		Mascot
2462.2661	2461.9116	-0.3545	-144	398	418	YKELQDIIAILGLDELSEEDR					Mascot
3843.9287	3843.354	-0.5747	-150	318	354	EGSITSIQAVYVPADDLTDPAPATTFAHLDATTVLSR	19	0			Mascot
3843.9287	3843.354	-0.5747	-150	318	354	EGSITSIQAVYVPADDLTDPAPATTFAHLDATTVLSR					Mascot

Rank	Protein Name	Accession No	Protein MW	Protein PI	Pep. Count	Protein Score	Protein Score C. I. %	Total Ion Score	Total Ion C. I. %
9	ATP synthase subunit beta, chloroplastic OS=Galbulimima belgraveana GN=atpB PE=3 SV=1	ATPB_GALB	53610.9	5.44	12	175	100	112	100

Peptide Information

Calc. Mass	Obsrv. Mass	± da	± ppm	Start Seq.	End Seq.	Sequence	Ion Score	C. I. %	Modification	Rank	Result Type
1245.5126	1245.4664	-0.0462	-37	208	217	EGNDLYMEMK			Oxidation (M)[7]		Mascot
1431.6863	1431.4924	-0.1939	-135	379	390	IVGEEHYETAQR					Mascot
1433.7747	1433.5828	-0.1919	-134	278	291	FVQAGSEVSALLGR					Mascot
1433.7747	1433.5828	-0.1919	-134	278	291	FVQAGSEVSALLGR	16	0			Mascot
1471.7614	1471.5635	-0.1979	-134	249	261	VGLTALTMAEYFR					Mascot
1487.7562	1487.559	-0.1972	-133	249	261	VGLTALTMAEYFR			Oxidation (M)[8]		Mascot
1487.7562	1487.559	-0.1972	-133	249	261	VGLTALTMAEYFR			Oxidation (M)[8]		Mascot
1502.6614	1502.5814	-0.08	-53	206	217	TREGNDLYMEMK			Oxidation (M)[9]		Mascot
1601.8104	1601.5908	-0.2196	-137	232	246	VALVYGQMNEPPGAR					Mascot
1601.8104	1601.5908	-0.2196	-137	232	246	VALVYGQMNEPPGAR	13	0			Mascot
1612.8766	1612.6858	-0.1908	-118	2	17	RINPTTSGPGVSTLGR					Mascot
1617.8053	1617.5864	-0.2189	-135	232	246	VALVYGQMNEPPGAR			Oxidation (M)[8]		Mascot
1949.9967	1949.734	-0.2627	-135	262	277	DVNEQDVLLFIDNIFR	50	99.893			Mascot
1949.9967	1949.734	-0.2627	-135	262	277	DVNEQDVLLFIDNIFR					Mascot
2061.0322	2060.7493	-0.2829	-137	360	378	GIYPAVDPLDSTSTMLQPR					Mascot
2077.0271	2076.7378	-0.2893	-139	360	378	GIYPAVDPLDSTSTMLQPR	14	0	Oxidation (M)[15]		Mascot
2077.0271	2076.7378	-0.2893	-139	360	378	GIYPAVDPLDSTSTMLQPR			Oxidation (M)[15]		Mascot
2282.0791	2281.7708	-0.3083	-135	292	312	MPSAVGYQPTLSTEMGSLQER					Mascot
2282.0791	2281.7708	-0.3083	-135	292	312	MPSAVGYQPTLSTEMGSLQER					Mascot
2298.074	2297.7598	-0.3142	-137	292	312	MPSAVGYQPTLSTEMGSLQER			Oxidation (M)[1]		Mascot
2314.0688	2313.79	-0.2788	-120	292	312	MPSAVGYQPTLSTEMGSLQER			Oxidation (M)[1,15]		Mascot
2462.2661	2461.9116	-0.3545	-144	398	418	YKELQDIIAILGLDELSEEDR					Mascot
3843.9287	3843.354	-0.5747	-150	318	354	EGSITSIQAVYVPADDLTDPAPATTFAHLDATTVLSR	19	0			Mascot
3843.9287	3843.354	-0.5747	-150	318	354	EGSITSIQAVYVPADDLTDPAPATTFAHLDATTVLSR					Mascot

Rank	Protein Name	Accession No	Protein MW	Protein PI	Pep. Count	Protein Score	Protein Score C. I. %	Total Ion Score	Total Ion C. I. %
10	ATP synthase subunit beta, chloroplastic OS=Mo caryophyllacea GN=atpB PE=3 SV=1	ATPB_MONC	53551.7	5.05	12	175	100	112	100

Peptide Information

Calc. Mass	Obsrv. Mass	± da	± ppm	Start Seq.	End Seq.	Sequence	Ion Score	C. I. %	Modification	Rank	Result Type
1245.5126	1245.4664	-0.0462	-37	208	217	EGNDLYMEMK			Oxidation (M)[7]		Mascot
1295.597	1295.4653	-0.1317	-102	487	497	AMNLEMESNLK			Oxidation (M)[2]		Mascot
1431.6863	1431.4924	-0.1939	-135	379	390	IVGEEHYETAQR					Mascot
1433.7747	1433.5828	-0.1919	-134	278	291	FVQAGSEVSALLGR					Mascot
1433.7747	1433.5828	-0.1919	-134	278	291	FVQAGSEVSALLGR	16	0			Mascot
1471.7614	1471.5635	-0.1979	-134	249	261	VGLTALTMAEYFR					Mascot
1487.7562	1487.559	-0.1972	-133	249	261	VGLTALTMAEYFR			Oxidation (M)[8]		Mascot
1487.7562	1487.559	-0.1972	-133	249	261	VGLTALTMAEYFR			Oxidation (M)[8]		Mascot
1502.6614	1502.5814	-0.08	-53	206	217	TREGNDLYMEMK			Oxidation (M)[9]		Mascot
1601.8104	1601.5908	-0.2196	-137	232	246	VALVYGQMNEPPGAR					Mascot
1601.8104	1601.5908	-0.2196	-137	232	246	VALVYGQMNEPPGAR	13	0			Mascot
1617.8053	1617.5864	-0.2189	-135	232	246	VALVYGQMNEPPGAR			Oxidation (M)[8]		Mascot
1949.9967	1949.734	-0.2627	-135	262	277	DVNEQDVLLFIDNIFR	50	99.893			Mascot
1949.9967	1949.734	-0.2627	-135	262	277	DVNEQDVLLFIDNIFR					Mascot
2061.0322	2060.7493	-0.2829	-137	360	378	GIYPAVDPLDSTSTMLQPR					Mascot
2077.0271	2076.7378	-0.2893	-139	360	378	GIYPAVDPLDSTSTMLQPR	14	0	Oxidation (M)[15]		Mascot
2077.0271	2076.7378	-0.2893	-139	360	378	GIYPAVDPLDSTSTMLQPR			Oxidation (M)[15]		Mascot
2282.0791	2281.7708	-0.3083	-135	292	312	MPSAVGYQPTLSTEMGSLQER					Mascot
2282.0791	2281.7708	-0.3083	-135	292	312	MPSAVGYQPTLSTEMGSLQER					Mascot
2298.074	2297.7598	-0.3142	-137	292	312	MPSAVGYQPTLSTEMGSLQER			Oxidation (M)[1]		Mascot
2314.0688	2313.79	-0.2788	-120	292	312	MPSAVGYQPTLSTEMGSLQER			Oxidation (M)[1,15]		Mascot
2462.2661	2461.9116	-0.3545	-144	398	418	YKELQDIIAILGLDELSEEDR					Mascot
3843.9287	3843.354	-0.5747	-150	318	354	EGSITSIQAVYVPADDLTDPAPATTFAHLDATTVLSR	19	2.227			Mascot
3843.9287	3843.354	-0.5747	-150	318	354	EGSITSIQAVYVPADDLTDPAPATTFAHLDATTVLSR					Mascot

Gel Idx/Pos	83/D8	Instr./Gel Origin	BA2060/131017A	Process Status	Analysis Succeeded
Plate [#] Name	[1] 31496	Instrument Sample Name		Spectra	8

Rank	Protein Name	Accession No	Protein MW	Protein PI	Pep. Count	Protein Score	Protein Score C. I. %	Total Ion Score	Total Ion C. I. %
1	Oxygen-evolving enhancer protein 1, chloroplastic OS=Pisum sativum GN=PSBO PE=2 SV=1	PSBO_PEA	34871.8	6.25	10	433	100	375	100

Peptide Information

Calc. Mass	Obsrv. Mass	± da	± ppm	Start Seq.	End Seq.	Sequence	Ion Score	C. I. %	Modification	Rank	Result Type
850.4304	850.3168	-0.1136	-134	234	241	GSSFLDPK					Mascot
964.5865	964.4564	-0.1301	-135	205	212	VPFLFTIK	47	99.865			Mascot
964.5865	964.4564	-0.1301	-135	205	212	VPFLFTIK					Mascot
1080.5571	1080.4061	-0.151	-140	88	96	LTFDEIQSK					Mascot
1236.6583	1236.4913	-0.167	-135	87	96	RLTFDEIQSK	30	92.719			Mascot
1236.6583	1236.4913	-0.167	-135	87	96	RLTFDEIQSK					Mascot
1293.6473	1293.4675	-0.1798	-139	319	329	IQGVWYAQLES					Mascot
1294.6711	1294.4854	-0.1857	-143	132	142	LCLEPTSFTVK			Carbamidomethyl (C)[2]		Mascot
1576.7714	1576.5555	-0.2159	-137	244	260	GASTGYDNAVALPAGGR	74	100			Mascot
1576.7714	1576.5555	-0.2159	-137	244	260	GASTGYDNAVALPAGGR					Mascot
1760.8813	1760.6362	-0.2451	-139	188	204	DGIDYAAVTVQLPGGER	95	100			Mascot
1760.8813	1760.6362	-0.2451	-139	188	204	DGIDYAAVTVQLPGGER					Mascot
2284.1606	2283.8367	-0.3239	-142	213	233	QLVASGKPDSFSGEFLVPSYR					Mascot
2284.1606	2283.8367	-0.3239	-142	213	233	QLVASGKPDSFSGEFLVPSYR	129	100			Mascot
2294.1299	2293.803	-0.3269	-142	184	204	FEEKDGIDYAAVTVQLPGGER					Mascot

Rank	Protein Name	Accession No	Protein MW	Protein PI	Pep. Count	Protein Score	Protein Score C. I. %	Total Ion Score	Total Ion C. I. %
2	Oxygen-evolving enhancer protein 1, chloroplastic OS=Spinacia oleracea GN=PSBO PE=1 SV=1	PSBO_SPIOL	35148.9	5.58	6	232	100	206	100

Peptide Information

Calc. Mass	Obsrv. Mass	± da	± ppm	Start Seq.	End Seq.	Sequence	Ion Score	C. I. %	Modification	Rank	Result Type
850.4304	850.3168	-0.1136	-134	236	243	GSSFLDPK					Mascot
964.5865	964.4564	-0.1301	-135	207	214	VPFLFTIK	47	99.865			Mascot
964.5865	964.4564	-0.1301	-135	207	214	VPFLFTIK					Mascot
1760.8813	1760.6362	-0.2451	-139	190	206	DGIDYAAVTVQLPGGER	95	100			Mascot
1760.8813	1760.6362	-0.2451	-139	190	206	DGIDYAAVTVQLPGGER					Mascot
2284.1606	2283.8367	-0.3239	-142	215	235	QLVASGKPESFSGDFLVPSYR					Mascot
2284.1606	2283.8367	-0.3239	-142	215	235	QLVASGKPESFSGDFLVPSYR	64	99.997			Mascot
2294.1299	2293.803	-0.3269	-142	186	206	FEEKDGIDYAAVTVQLPGGER					Mascot
2300.1179	2299.7908	-0.3271	-142	165	185	LTYTLDEIEGPFEVSSDGTVK					Mascot

Rank	Protein Name	Accession No	Protein MW	Protein PI	Pep. Count	Protein Score	Protein Score C. I. %	Total Ion Score	Total Ion C. I. %
3	Oxygen-evolving enhancer protein 1, chloroplastic OS=Nicotiana tabacum GN=PSBO PE=2 SV=1	PSBO_TOBA(	35206	5.89	7	204	100	172	100

Peptide Information

Calc. Mass	Obsrv. Mass	± da	± ppm	Start Seq.	End Seq.	Sequence	Ion Score	C. I. %	Modification	Rank	Result Type
850.4304	850.3168	-0.1136	-134	238	245	GSSFLDPK					Mascot
964.5865	964.4564	-0.1301	-135	209	216	VPFLFTIK	47	99.865			Mascot
964.5865	964.4564	-0.1301	-135	209	216	VPFLFTIK					Mascot
1080.5571	1080.4061	-0.151	-140	92	100	LTFDEIQSK					Mascot
1236.6583	1236.4913	-0.167	-135	91	100	RLTFDEIQSK	30	92.719			Mascot
1236.6583	1236.4913	-0.167	-135	91	100	RLTFDEIQSK					Mascot
1760.8813	1760.6362	-0.2451	-139	192	208	DGIDYAAVTVQLPGGER	95	100			Mascot
1760.8813	1760.6362	-0.2451	-139	192	208	DGIDYAAVTVQLPGGER					Mascot
2294.1299	2293.803	-0.3269	-142	188	208	FEEKDGIDYAAVTVQLPGGER					Mascot
2300.1179	2299.7908	-0.3271	-142	167	187	LTYTLDEIEGPFEVSSDGTVK					Mascot

Rank	Protein Name	Accession No	Protein MW	Protein PI	Pep. Count	Protein Score	Protein Score C. I. %	Total Ion Score	Total Ion C. I. %
4	Oxygen-evolving enhancer protein 1, chloroplastic OS=Solanum tuberosum GN=PSBO PE=2 SV=1	PSBO_SOLTU	35367	5.84	6	197	100	172	100

Peptide Information

Calc. Mass Calc. Mass	s rv. Mass	± da	± da ± ppm	± ppm Seq.	Start Seq.	End S Sequen	Score	C. I. % %	Modificat Modification	Rank	Rank
850.4304	850.3168	-0.1136	-134	238	245	GSSFLDPK					Mascot
964.5865	964.4564	-0.1301	-135	209	216	VPFLFTIK	47	99.865			Mascot
964.5865	964.4564	-0.1301	-135	209	216	VPFLFTIK					Mascot
1080.5571	1080.4061	-0.151	-140	92	100	LTFDEIQSK					Mascot
1236.6583	1236.4913	-0.167	-135	91	100	RLTFDEIQSK	30	92.719			Mascot
1236.6583	1236.4913	-0.167	-135	91	100	RLTFDEIQSK					Mascot
1760.8813	1760.6362	-0.2451	-139	192	208	DGIDYAAVTVQLPGGER	95	100			Mascot
1760.8813	1760.6362	-0.2451	-139	192	208	DGIDYAAVTVQLPGGER					Mascot
2294.1299	2293.803	-0.3269	-142	188	208	FEEKDGIDYAAVTVQLPGGER					Mascot

Rank	Protein Name	Accession No	Protein MW	Protein PI	Pep. Count	Protein Score	Protein Score C. I. %	Total Ion Score	Total Ion C. I. %
5	Oxygen-evolving enhancer protein 1, chloroplastic OS=Helianthus annuus GN=PSBO PE=1 SV=1	PSBO_HELAN	34202.3	5.4	6	168	100	142	100

Peptide Information

Calc. Mass	Obsrv. Mass	± da	± ppm	Start Seq.	End Seq.	Sequence	Ion Score	C. I. %	Modification	Rank	Result Type
850.4304	850.3168	-0.1136	-134	230	237	GSSFLDPK					Mascot
964.5865	964.4564	-0.1301	-135	201	208	VPFLFTIK	47	99.865			Mascot
964.5865	964.4564	-0.1301	-135	201	208	VPFLFTIK					Mascot
1760.8813	1760.6362	-0.2451	-139	184	200	DGIDYAAVTVQLPGGER	95	100			Mascot
1760.8813	1760.6362	-0.2451	-139	184	200	DGIDYAAVTVQLPGGER					Mascot
2239.1572	2238.8289	-0.3283	-147	1	21	MAASLQAAATFMTPTSRVQLK			Oxidation (M)[1]		Mascot
2280.1494	2279.8274	-0.322	-141	159	179	LTYTLDEIEGPLEVSSDGTIK					Mascot
2294.1299	2293.803	-0.3269	-142	180	200	FEEKDGIDYAAVTVQLPGGER					Mascot

Rank	Protein Name	Accession No	Protein MW	Protein PI	Pep. Count	Protein Score	Protein Score C. I. %	Total Ion Score	Total Ion C. I. %
6	Oxygen-evolving enhancer protein 1, chloroplastic (Fragments) OS=Populus euphratica GN=PSBO PE=1 SV	PSBO_POPEU	10664.5	5.36	3	161	100	142	100

Peptide Information

Calc. Mass	Obsrv. Mass	± da	± ppm	Start Seq.	End Seq.	Sequence	Ion Score	C. I. %	Modification	Rank	Result Type
964.5865	964.4564	-0.1301	-135	48	55	VPFLFTIK	47	99.865			Mascot
964.5865	964.4564	-0.1301	-135	48	55	VPFLFTIK					Mascot
1161.5898	1161.4318	-0.158	-136	17	26	NSPPDFQKTK					Mascot
1760.8813	1760.6362	-0.2451	-139	31	47	DGIDYAAVTVQLPGGER	95	100			Mascot
1760.8813	1760.6362	-0.2451	-139	31	47	DGIDYAAVTVQLPGGER					Mascot

Rank	Protein Name	Accession No	Protein MW	Protein PI	Pep. Count	Protein Score	Protein Score C. I. %	Total Ion Score	Total Ion C. I. %
7	Oxygen-evolving enhancer protein 1, chloroplastic OS=Solanum lycopersicum GN=PSBO PE=2 SV=2	PSBO_SOLLC	34925.9	5.91	4	155	100	142	100

Peptide Information

Calc. Mass	Obsrv. Mass	± da	± ppm	Start Seq.	End Seq.	Sequence	Ion Score	C. I. %	Modification	Rank	Result Type
850.4304	850.3168	-0.1136	-134	235	242	GSSFLDPK					Mascot
964.5865	964.4564	-0.1301	-135	206	213	VPFLFTIK	47	99.865			Mascot
964.5865	964.4564	-0.1301	-135	206	213	VPFLFTIK					Mascot
1760.8813	1760.6362	-0.2451	-139	189	205	DGIDYAAVTVQLPGGER	95	100			Mascot
1760.8813	1760.6362	-0.2451	-139	189	205	DGIDYAAVTVQLPGGER					Mascot
2294.1299	2293.803	-0.3269	-142	185	205	FEEKDGIDYAAVTVQLPGGER					Mascot

Rank	Protein Name	Accession No	Protein MW	Protein PI	Pep. Count	Protein Score	Protein Score C. I. %	Total Ion Score	Total Ion C. I. %
8	Oxygen-evolving enhancer protein 1, chloroplastic OS=Triticum aestivum GN=PSBO PE=2 SV=1	PSBO_WHEA	34718.7	8.73	7	154	100	125	100

Peptide Information

Calc. Mass	Obsrv. Mass	± da	± ppm	Start Seq.	End Seq.	Sequence	Ion Score	C. I. %	Modification	Rank	Result Type
842.4076	842.406	-0.0016	-2	122	128	AGKYEMK			Oxidation (M)[6]		Mascot
850.4304	850.3168	-0.1136	-134	231	238	GSSFLDPK					Mascot
1080.5571	1080.4061	-0.151	-140	86	94	LTFDEIQSK					Mascot
1236.6583	1236.4913	-0.167	-135	85	94	RLTFDEIQSK	30	92.719			Mascot
1236.6583	1236.4913	-0.167	-135	85	94	RLTFDEIQSK					Mascot
1760.8813	1760.6362	-0.2451	-139	186	202	DGIDYAAVTVQLPGGER	95	100			Mascot
1760.8813	1760.6362	-0.2451	-139	186	202	DGIDYAAVTVQLPGGER					Mascot
2267.1411	2266.8152	-0.3259	-144	157	175	LMTRLTYTLDEMEGPLEVR					Mascot
2283.136	2282.822	-0.314	-138	157	175	LMTRLTYTLDEMEGPLEVR			Oxidation (M)[2]		Mascot
2294.1299	2293.803	-0.3269	-142	182	202	FEEKDGIDYAAVTVQLPGGER					Mascot

Rank	Protein Name	Accession No	Protein MW	Protein PI	Pep. Count	Protein Score	Protein Score C. I. %	Total Ion Score	Total Ion C. I. %
9	Oxygen-evolving enhancer protein 1, chloroplastic OS=Fritillaria agrestis GN=PSBO PE=2 SV=1	PSBO_FRIAG	34847.8	6.26	6	149	100	125	100

Peptide Information

Calc. Mass	Obsrv. Mass	± da	± ppm	Start Seq.	End Seq.	Sequence	Ion Score	C. I. %	Modification	Rank	Result Type
850.4304	850.3168	-0.1136	-134	235	242	GSSFLDPK					Mascot
1080.5571	1080.4061	-0.151	-140	89	97	LTFDEIQSK					Mascot
1236.6583	1236.4913	-0.167	-135	88	97	RLTFDEIQSK	30	92.719			Mascot
1236.6583	1236.4913	-0.167	-135	88	97	RLTFDEIQSK					Mascot
1294.6711	1294.4854	-0.1857	-143	133	143	LCLEPTSFTVK			Carbamidomethyl (C)[2]		Mascot
1760.8813	1760.6362	-0.2451	-139	189	205	DGIDYAAVTVQLPGGER	95	100			Mascot
1760.8813	1760.6362	-0.2451	-139	189	205	DGIDYAAVTVQLPGGER					Mascot
2294.1299	2293.803	-0.3269	-142	185	205	FEEKDGIDYAAVTVQLPGGER					Mascot

Rank	Protein Name	Accession No	Protein MW	Protein PI	Pep. Count	Protein Score	Protein Score C. I. %	Total Ion Score	Total Ion C. I. %
10	Putative oxygen-evolving enhancer protein 1 (Fragments) OS=Pinus strobus GN=PSBO PE=1 SV=1	PSBO_PINST	13643.7	4.46	2	104	100	95	100

Peptide Information

Calc. Mass	Obsrv. Mass	± da	± ppm	Start Seq.	End Seq.	Sequence	Ion Score	C. I. %	Modification	Rank	Result Type
850.3975	850.3168	-0.0807	-95	35	42	GSSMLDPK			Oxidation (M)[4]		Mascot
1760.8813	1760.6362	-0.2451	-139	1	17	DGIDYAAVTVQLPGGER	95	100			Mascot
1760.8813	1760.6362	-0.2451	-139	1	17	DGIDYAAVTVQLPGGER					Mascot

Gel Idx/Pos	84/D9	Instr./Gel Origin	BA2060/131017A	Process Status	Analysis Succeeded
Plate [#] Name	[1] 31496	Instrument Sample Name		Spectra	8

Rank	Protein Name	Accession No	Protein MW	Protein PI	Pep. Count	Protein Score	Protein Score C. I. %	Total Ion Score	Total Ion C. I. %
1	Ribulose bisphosphate carboxylase large chain (Fragment) OS=Prunus domestica GN=rbcL PE=3 SV=1	RBL_PRUDO	50914.6	6.23	14	84	99.985	7	0

Peptide Information

Calc. Mass	Obsrv. Mass	± da	± ppm	Start Seq.	End Seq.	Sequence	Ion Score	C. I. %	Modification	Rank	Result Type
830.4366	830.3275	-0.1091	-131	430	436	EGNEIIR					Mascot
898.41	898.2901	-0.1199	-133	296	302	NHGMHFR					Mascot
910.4451	910.325	-0.1201	-132	178	184	AVYECLR			Carbamidomethyl (C)[5]		Mascot
914.4049	914.2822	-0.1227	-134	296	302	NHGMHFR			Oxidation (M)[4]		Mascot
1116.583	1116.4386	-0.1444	-129	412	421	VALEACVQAR			Carbamidomethyl (C)[6]		Mascot
1170.5585	1170.4877	-0.0708	-60	294	302	QKNHGMHFR			Oxidation (M)[6]		Mascot
1187.6643	1187.5109	-0.1534	-129	276	285	DNGLLLHIHR					Mascot
1245.6433	1245.4706	-0.1727	-139	430	440	EGNEIIREASK					Mascot
1245.6433	1245.4706	-0.1727	-139	430	440	EGNEIIREASK					Mascot
1275.7307	1275.5624	-0.1683	-132	330	340	EITLGFVDLLR	7	0			Mascot
1275.7307	1275.5624	-0.1683	-132	330	340	EITLGFVDLLR					Mascot
1465.7216	1465.5588	-0.1628	-111	310	324	MSGGDHIHAGTVVGK					Mascot
1475.7351	1475.548	-0.1871	-127	206	217	DRFLFCAEAIYK					Mascot
1546.7358	1546.5234	-0.2124	-137	441	453	WSPELAAACEVWK			Carbamidomethyl (C)[9]		Mascot
2009.059	2008.7917	-0.2673	-133	330	346	EITLGFVDLLRDDFVEK					Mascot
2169.9868	2169.6772	-0.3096	-143	185	203	GGLDFTKDDENVNSQPFMR					Mascot
2185.9819	2185.7007	-0.2812	-129	185	203	GGLDFTKDDENVNSQPFMR			Oxidation (M)[18]		Mascot
3022.4548	3022.0066	-0.4482	-148	249	275	ELGVPIVMHDYLTGGFTANTSLAHYCR			Carbamidomethyl (C)[26]		Mascot
3038.45	3038.0183	-0.4317	-142	249	275	ELGVPIVMHDYLTGGFTANTSLAHYCR			Carbamidomethyl (C)[26], Oxidation (M)[8]		Mascot

Rank	Protein Name	Accession No	Protein MW	Protein PI	Pep. Count	Protein Score	Protein Score C. I. %	Total Ion Score	Total Ion C. I. %
2	Ribulose bisphosphate carboxylase large chain OS=Cajanus cajan GN=rbcL PE=3 SV=1	RBL_CAJCA	52358.5	6.45	14	81	99.975	7	0

Peptide Information

Calc. Mass	Obsrv. Mass	± da	± ppm	Start Seq.	End Seq.	Sequence	Ion Score	C. I. %	Modification	Rank	Result Type
830.4366	830.3275	-0.1091	-131	440	446	EGNEIIR					Mascot
856.3546	856.4183	0.0637	74	467	473	FEFPAMD					Mascot
898.41	898.2901	-0.1199	-133	306	312	NHGMHFR					Mascot
910.4451	910.325	-0.1201	-132	188	194	AVYECLR			Carbamidomethyl (C)[5]		Mascot
914.4049	914.2822	-0.1227	-134	306	312	NHGMHFR			Oxidation (M)[4]		Mascot
1116.583	1116.4386	-0.1444	-129	422	431	VALEACVQAR			Carbamidomethyl (C)[6]		Mascot
1170.5585	1170.4877	-0.0708	-60	304	312	QKNHGMHFR			Oxidation (M)[6]		Mascot
1187.6643	1187.5109	-0.1534	-129	286	295	DNGLLLHIHR					Mascot
1245.6337	1245.4706	-0.1631	-131	218	227	FLFCAEALFK			Carbamidomethyl (C)[4]		Mascot
1245.6337	1245.4706	-0.1631	-131	218	227	FLFCAEALFK			Carbamidomethyl (C)[4]		Mascot
1275.7307	1275.5624	-0.1683	-132	340	350	EITLGFVDLLR	7	0			Mascot
1275.7307	1275.5624	-0.1683	-132	340	350	EITLGFVDLLR					Mascot
1405.6998	1405.5753	-0.1245	-89	22	32	LTYYTPQYQTK					Mascot
1465.7546	1465.5588	-0.1958	-134	147	159	TFQGPPHGIQVER					Mascot
1546.7358	1546.5234	-0.2124	-137	451	463	WSPELAAACEVWK			Carbamidomethyl (C)[9]		Mascot
2009.059	2008.7917	-0.2673	-133	340	356	EITLGFVDLLRDDFVEK					Mascot
2169.9868	2169.6772	-0.3096	-143	195	213	GGLDFTKDDENVNSQPFMR					Mascot
2185.9819	2185.7007	-0.2812	-129	195	213	GGLDFTKDDENVNSQPFMR			Oxidation (M)[18]		Mascot

Rank	Protein Name	Accession No	Protein MW	Protein PI	Pep. Count	Protein Score	Protein Score C. I. %	Total Ion Score	Total Ion C. I. %
3	Ribulose bisphosphate carboxylase large chain (Fragment) OS=Sinapis alba GN=rbcL PE=3 SV=1	RBL_SINAL	52405.4	6.23	14	81	99.971	7	0

Peptide Information

Calc. Mass	Obsrv. Mass	± da	± ppm	Start Seq.	End Seq.	Sequence	Ion Score	C. I. %	Modification	Rank	Result Type
830.4366	830.3275	-0.1091	-131	440	446	EGNEIIR					Mascot
856.3546	856.4183	0.0637	74	467	473	FEFPAMD					Mascot
898.41	898.2901	-0.1199	-133	306	312	NHGMHFR					Mascot
910.4451	910.325	-0.1201	-132	188	194	AVYECLR			Carbamidomethyl (C)[5]		Mascot
914.4049	914.2822	-0.1227	-134	306	312	NHGMHFR			Oxidation (M)[4]		Mascot
1116.583	1116.4386	-0.1444	-129	422	431	VALEACVQAR			Carbamidomethyl (C)[6]		Mascot
1170.5585	1170.4877	-0.0708	-60	304	312	QKNHGMHFR			Oxidation (M)[6]		Mascot
1187.6643	1187.5109	-0.1534	-129	286	295	DNGLLLHIHR					Mascot
1245.6433	1245.4706	-0.1727	-139	440	450	EGNEIIREASK					Mascot
1245.6433	1245.4706	-0.1727	-139	440	450	EGNEIIREASK					Mascot
1275.7307	1275.5624	-0.1683	-132	340	350	EITLGFVDLLR	7	0			Mascot
1275.7307	1275.5624	-0.1683	-132	340	350	EITLGFVDLLR					Mascot
1465.7546	1465.5588	-0.1958	-134	147	159	TFQGPPHGIQVER					Mascot
1475.7351	1475.548	-0.1871	-127	216	227	DRFLFCAEAIYK					Mascot
1546.7358	1546.5234	-0.2124	-137	451	463	WSPELAAACEVWK			Carbamidomethyl (C)[9]		Mascot
2169.9868	2169.6772	-0.3096	-143	195	213	GGLDFTKDDENVNSQPFMR					Mascot
2185.9819	2185.7007	-0.2812	-129	195	213	GGLDFTKDDENVNSQPFMR			Oxidation (M)[18]		Mascot
3022.4548	3022.0066	-0.4482	-148	259	285	ELGVPIVMHDYLTGGFTANTSLAHYCR			Carbamidomethyl (C)[26]		Mascot
3038.45	3038.0183	-0.4317	-142	259	285	ELGVPIVMHDYLTGGFTANTSLAHYCR			Carbamidomethyl (C)[26], Oxidation (M)[8]		Mascot

Rank	Protein Name	Accession No	Protein MW	Protein PI	Pep. Count	Protein Score	Protein Score C. I. %	Total Ion Score	Total Ion C. I. %
4	Ribulose bisphosphate carboxylase large chain OS=Liquidambar styraciflua GN=rbcL PE=3 SV=1	RBL_LIQST	52625.5	6.04	14	81	99.971	7	0

Peptide Information

Calc. Mass	Obsrv. Mass	± da	± ppm	Start Seq.	End Seq.	Sequence	Ion Score	C. I. %	Modification	Rank	Result Type
830.4366	830.3275	-0.1091	-131	440	446	EGNEIIR					Mascot
898.41	898.2901	-0.1199	-133	306	312	NHGMHFR					Mascot
910.4451	910.325	-0.1201	-132	188	194	AVYECLR			Carbamidomethyl (C)[5]		Mascot
914.4049	914.2822	-0.1227	-134	306	312	NHGMHFR			Oxidation (M)[4]		Mascot
1116.583	1116.4386	-0.1444	-129	422	431	VALEACVQAR			Carbamidomethyl (C)[6]		Mascot
1170.5585	1170.4877	-0.0708	-60	304	312	QKNHGMHFR			Oxidation (M)[6]		Mascot
1187.6643	1187.5109	-0.1534	-129	286	295	DNGLLLHIHR					Mascot
1245.6433	1245.4706	-0.1727	-139	440	450	EGNEIIREASK					Mascot
1245.6433	1245.4706	-0.1727	-139	440	450	EGNEIIREASK					Mascot
1275.7307	1275.5624	-0.1683	-132	340	350	EITLGFVDLLR	7	0			Mascot
1275.7307	1275.5624	-0.1683	-132	340	350	EITLGFVDLLR					Mascot
1465.7216	1465.5588	-0.1628	-111	320	334	MSGGDHIHAGTVVGK					Mascot
1475.7351	1475.548	-0.1871	-127	216	227	DRFLFCAEAIYK					Mascot
1488.6926	1488.5103	-0.1823	-122	464	475	EIKFEFEAMDTL			Oxidation (M)[9]		Mascot
1546.7358	1546.5234	-0.2124	-137	451	463	WSPELAAACEVWK			Carbamidomethyl (C)[9]		Mascot
2169.9868	2169.6772	-0.3096	-143	195	213	GGLDFTKDDENVNSQPFMR					Mascot
2185.9819	2185.7007	-0.2812	-129	195	213	GGLDFTKDDENVNSQPFMR			Oxidation (M)[18]		Mascot
3022.4548	3022.0066	-0.4482	-148	259	285	ELGVPIVMHDYLTGGFTANTSLAHYCR			Carbamidomethyl (C)[26]		Mascot
3038.45	3038.0183	-0.4317	-142	259	285	ELGVPIVMHDYLTGGFTANTSLAHYCR			Carbamidomethyl (C)[26], Oxidation (M)[8]		Mascot

Rank	Protein Name	Accession No	Protein MW	Protein PI	Pep. Count	Protein Score	Protein Score C. I. %	Total Ion Score	Total Ion C. I. %
5	Ribulose bisphosphate carboxylase large chain (Fragment) OS=Ajuga chamaepitys GN=rbcL PE=3 SV=1	RBL_AJUCH	52421.4	6.23	14	80	99.969	7	0

Peptide Information

Calc. Mass	Obsrv. Mass	± da	± ppm	Start Seq.	End Seq.	Sequence	Ion Score	C. I. %	Modification	Rank	Result Type
856.3546	856.4183	0.0637	74	467	473	FEFPAMD					Mascot Mascot
898.41	898.2901	-0.1199	-133	306	312	NHGMHFR					Mascot
910.4451	910.325	-0.1201	-132	188	194	AVYECLR			Carbamidomethyl (C)[5]		Mascot
914.4049	914.2822	-0.1227	-134	306	312	NHGMHFR			Oxidation (M)[4]		Mascot
1116.583	1116.4386	-0.1444	-129	422	431	VALEACVQAR			Carbamidomethyl (C)[6]		Mascot
1170.5585	1170.4877	-0.0708	-60	304	312	QKNHGMHFR			Oxidation (M)[6]		Mascot
1187.6643	1187.5109	-0.1534	-129	286	295	DNGLLLHIHR					Mascot
1275.7307	1275.5624	-0.1683	-132	340	350	EITLGFVDLLR	7	0			Mascot
1275.7307	1275.5624	-0.1683	-132	340	350	EITLGFVDLLR					Mascot
1287.6401	1287.4926	-0.1475	-115	84	94	CYHIEPVLGEK					Mascot
1465.7546	1465.5588	-0.1958	-134	147	159	TFQGPPHGIQVER					Mascot
1475.7351	1475.548	-0.1871	-127	216	227	DRFLFCAEAIYK					Mascot
1546.7358	1546.5234	-0.2124	-137	451	463	WSPELAAACEVWK			Carbamidomethyl (C)[9]		Mascot
1827.8687	1827.6976	-0.1711	-94	19	32	EYKLTYYTPEYETK					Mascot
1827.8687	1827.6976	-0.1711	-94	19	32	EYKLTYYTPEYETK					Mascot
2009.059	2008.7917	-0.2673	-133	340	356	EITLGFVDLLRDDFVEK					Mascot
2169.9868	2169.6772	-0.3096	-143	195	213	GGLDFTKDDENVNSQPFMR					Mascot
2185.9819	2185.7007	-0.2812	-129	195	213	GGLDFTKDDENVNSQPFMR			Oxidation (M)[18]		Mascot

Rank	Protein Name	Accession No	Protein MW	Protein PI	Pep. Count	Protein Score	Protein Score C. I. %	Total Ion Score	Total Ion C. I. %
6	Ribulose bisphosphate carboxylase large chain OS=Glycine max GN=rbcL PE=1 SV=3	RBL_SOYBN	52576.4	6	14	78	99.951	7	0

Peptide Information

Calc. Mass	Obsrv. Mass	± da	± ppm	Start Seq.	End Seq.	Sequence	Ion Score	C. I. %	Modification	Rank	Result Type
830.4366	830.3275	-0.1091	-131	440	446	EGNEIIR					Mascot
898.41	898.2901	-0.1199	-133	306	312	NHGMHFR					Mascot
910.4451	910.325	-0.1201	-132	188	194	AVYECLR			Carbamidomethyl (C)[5]		Mascot
914.4049	914.2822	-0.1227	-134	306	312	NHGMHFR			Oxidation (M)[4]		Mascot
1116.583	1116.4386	-0.1444	-129	422	431	VALEACVQAR			Carbamidomethyl (C)[6]		Mascot
1170.5585	1170.4877	-0.0708	-60	304	312	QKNHGMHFR			Oxidation (M)[6]		Mascot
1187.6643	1187.5109	-0.1534	-129	286	295	DNGLLLHIHR					Mascot
1245.6337	1245.4706	-0.1631	-131	218	227	FLFCAEAIFK			Carbamidomethyl (C)[4]		Mascot
1245.6337	1245.4706	-0.1631	-131	218	227	FLFCAEAIFK			Carbamidomethyl (C)[4]		Mascot
1275.7307	1275.5624	-0.1683	-132	340	350	EITLGFVDLLR	7	0			Mascot
1275.7307	1275.5624	-0.1683	-132	340	350	EITLGFVDLLR					Mascot
1465.7546	1465.5588	-0.1958	-134	147	159	TFQGPPHGIQVER					Mascot
1488.6926	1488.5103	-0.1823	-122	464	475	EIKFEFEAMDTL			Oxidation (M)[9]		Mascot
1546.7358	1546.5234	-0.2124	-137	451	463	WSPELAAACEVWK			Carbamidomethyl (C)[9]		Mascot
2009.059	2008.7917	-0.2673	-133	340	356	EITLGFVDLLRDDFVEK					Mascot
2169.9868	2169.6772	-0.3096	-143	195	213	GGLDFTKDDENVNSQPFMR					Mascot
2185.9819	2185.7007	-0.2812	-129	195	213	GGLDFTKDDENVNSQPFMR			Oxidation (M)[18]		Mascot
3022.4548	3022.0066	-0.4482	-148	259	285	ELGVPIVMHDYLTGGFTANTSLAHYCR			Carbamidomethyl (C)[26]		Mascot
3038.45	3038.0183	-0.4317	-142	259	285	ELGVPIVMHDYLTGGFTANTSLAHYCR			Carbamidomethyl (C)[26], Oxidation (M)[8]		Mascot

Rank	Protein Name	Accession No	Protein MW	Protein PI	Pep. Count	Protein Score	Protein Score C. I. %	Total Ion Score	Total Ion C. I. %
7	Ribulose bisphosphate carboxylase large chain (Fragment) OS=Tamarindus indica GN=rbcL PE=3 SV=1	RBL_TAMIN	50497.5	6.13	13	78	99.944	7	0

Peptide Information

Calc. Mass	Obsrv. Mass	± da	± ppm	Start Seq.	End Seq.	Sequence	Ion Score	C. I. %	Modification	Rank	Result Type
830.4366	830.3275	-0.1091	-131	431	437	EGNEIIR					Mascot
898.41	898.2901	-0.1199	-133	297	303	NHGMHFR					Mascot
910.4451	910.325	-0.1201	-132	179	185	AVYECLR			Carbamidomethyl (C)[5]		Mascot
914.4049	914.2822	-0.1227	-134	297	303	NHGMHFR			Oxidation (M)[4]		Mascot
1116.583	1116.4386	-0.1444	-129	413	422	VALEACVQAR			Carbamidomethyl (C)[6]		Mascot
1170.5585	1170.4877	-0.0708	-60	295	303	QKNHGMHFR			Oxidation (M)[6]		Mascot
1187.6643	1187.5109	-0.1534	-129	277	286	DNGLLLHIHR					Mascot
1245.6433	1245.4706	-0.1727	-139	431	441	EGNEIIREASK					Mascot
1245.6433	1245.4706	-0.1727	-139	431	441	EGNEIIREASK					Mascot
1275.7307	1275.5624	-0.1683	-132	331	341	EITLGFVDLLR	7	0			Mascot
1275.7307	1275.5624	-0.1683	-132	331	341	EITLGFVDLLR					Mascot
1465.7546	1465.5588	-0.1958	-134	138	150	TFQGPPHGIQVER					Mascot
1475.7351	1475.548	-0.1871	-127	207	218	DRFLFCAEAIYK					Mascot
1546.7358	1546.5234	-0.2124	-137	442	454	WSPELAAACEVWK			Carbamidomethyl (C)[9]		Mascot
2169.9868	2169.6772	-0.3096	-143	186	204	GGLDFTKDDENVNSQPFMR					Mascot
2185.9819	2185.7007	-0.2812	-129	186	204	GGLDFTKDDENVNSQPFMR			Oxidation (M)[18]		Mascot
3022.4548	3022.0066	-0.4482	-148	250	276	ELGVPIVMHDYLTGGFTANTSLAHYCR			Carbamidomethyl (C)[26]		Mascot
3038.45	3038.0183	-0.4317	-142	250	276	ELGVPIVMHDYLTGGFTANTSLAHYCR			Carbamidomethyl (C)[26], Oxidation (M)[8]		Mascot

Rank	Protein Name	Accession No	Protein MW	Protein PI	Pep. Count	Protein Score	Protein Score C. I. %	Total Ion Score	Total Ion C. I. %
8	Ribulose bisphosphate carboxylase large chain (Fragment) OS=Manilkara zapota GN=rbcL PE=3 SV=2	RBL_MANZA	51550	6.04	13	78	99.942	7	0

Peptide Information

Calc. Mass	Obsrv. Mass	± da	± ppm	Start Seq.	End Seq.	Sequence	Ion Score	C. I. %	Modification	Rank	Result Type
830.4366	830.3275	-0.1091	-131	430	436	EGNEIIR					Mascot
898.41	898.2901	-0.1199	-133	296	302	NHGMHFR					Mascot
910.4451	910.325	-0.1201	-132	178	184	AVYECLR			Carbamidomethyl (C)[5]		Mascot
914.4049	914.2822	-0.1227	-134	296	302	NHGMHFR			Oxidation (M)[4]		Mascot
1116.583	1116.4386	-0.1444	-129	412	421	VALEACVQAR			Carbamidomethyl (C)[6]		Mascot
1170.5585	1170.4877	-0.0708	-60	294	302	QKNHGMHFR			Oxidation (M)[6]		Mascot
1187.6643	1187.5109	-0.1534	-129	276	285	DNGLLLHIHR					Mascot
1245.6433	1245.4706	-0.1727	-139	430	440	EGNEIIREASK					Mascot
1245.6433	1245.4706	-0.1727	-139	430	440	EGNEIIREASK					Mascot
1275.7307	1275.5624	-0.1683	-132	330	340	EITLGFVDLLR	7	0			Mascot
1275.7307	1275.5624	-0.1683	-132	330	340	EITLGFVDLLR					Mascot
1465.7546	1465.5588	-0.1958	-134	137	149	TFQGPPHGIQVER					Mascot
1488.6926	1488.5103	-0.1823	-122	454	465	EIKFEFEAMDTL			Oxidation (M)[9]		Mascot
1546.7358	1546.5234	-0.2124	-137	441	453	WSPELAAACEVWK			Carbamidomethyl (C)[9]		Mascot
2169.9868	2169.6772	-0.3096	-143	185	203	GGLDFTKDDENVNSQPFMR					Mascot
2185.9819	2185.7007	-0.2812	-129	185	203	GGLDFTKDDENVNSQPFMR			Oxidation (M)[18]		Mascot
3022.4548	3022.0066	-0.4482	-148	249	275	ELGVPIVMHDYLTGGFTANTSLAHYCR			Carbamidomethyl (C)[26]		Mascot
3038.45	3038.0183	-0.4317	-142	249	275	ELGVPIVMHDYLTGGFTANTSLAHYCR			Carbamidomethyl (C)[26], Oxidation (M)[8]		Mascot

Rank	Protein Name	Accession No	Protein MW	Protein PI	Pep. Count	Protein Score	Protein Score C. I. %	Total Ion Score	Total Ion C. I. %
9	Ribulose bisphosphate carboxylase large chain (Fragment) OS=Thespesia populnea GN=rbcL PE=3 SV=1	RBL_THEPO	51698	5.95	14	77	99.939	[illegible]	[illegible]

Peptide Information

Calc. Mass	Obsrv. Mass	± da	± ppm	Start Seq.	End Seq.	Sequence	Ion Score	C. I. %	Modification	Rank	Result Type
830.4366	830.3275	-0.1091	-131	431	437	EGNEIIR					Mascot
898.41	898.2901	-0.1199	-133	297	303	NHGMHFR					Mascot
910.4451	910.325	-0.1201	-132	179	185	AVYECLR			Carbamidomethyl (C)[5]		Mascot
914.4049	914.2822	-0.1227	-134	297	303	NHGMHFR			Oxidation (M)[4]		Mascot
1116.583	1116.4386	-0.1444	-129	413	422	VALEACVQAR			Carbamidomethyl (C)[6]		Mascot
1170.5585	1170.4877	-0.0708	-60	295	303	QKNHGMHFR			Oxidation (M)[6]		Mascot
1187.6643	1187.5109	-0.1534	-129	277	286	DNGLLLHIHR					Mascot
1245.6433	1245.4706	-0.1727	-139	431	441	EGNEIIREASK					Mascot
1245.6433	1245.4706	-0.1727	-139	431	441	EGNEIIREASK					Mascot
1405.6886	1405.5753	-0.1133	-81	13	23	LTYYTPEYEVK					Mascot
1465.7216	1465.5588	-0.1628	-111	311	325	MSGGDHIHAGTVVGK					Mascot
1475.7351	1475.548	-0.1871	-127	207	218	DRFLFCAEAIYK					Mascot
1546.7358	1546.5234	-0.2124	-137	442	454	WSPELAAACEVWK			Carbamidomethyl (C)[9]		Mascot
2009.059	2008.7917	-0.2673	-133	331	347	DITLGFVDLLRDDFIEK					Mascot
2169.9868	2169.6772	-0.3096	-143	186	204	GGLDFTKDDENVNSQPFMR					Mascot
2185.9819	2185.7007	-0.2812	-129	186	204	GGLDFTKDDENVNSQPFMR			Oxidation (M)[18]		Mascot
3022.4548	3022.0066	-0.4482	-148	250	276	ELGVPIVMHDYLTGGFTANTSLAHYCR			Carbamidomethyl (C)[26]		Mascot
3038.45	3038.0183	-0.4317	-142	250	276	ELGVPIVMHDYLTGGFTANTSLAHYCR			Carbamidomethyl (C)[26], Oxidation (M)[8]		Mascot

Rank	Protein Name	Accession No	Protein MW	Protein PI	Pep. Count	Protein Score	Protein Score C. I. %	Total Ion Score	Total Ion C. I. %
10	Ribulose bisphosphate carboxylase large chain (Fragment) OS=Cornus obliqua GN=rbcL PE=3 SV=2	RBL_COROB	51536.8	6	13	77	99.933	7	0

Peptide Information

Calc. Mass	Obsrv. Mass	± da	± ppm	Start Seq.	End Seq.	Sequence	Ion Score	C. I. %	Modification	Rank	Result Type
830.4366	830.3275	-0.1091	-131	430	436	EGNEIIR					Mascot
898.41	898.2901	-0.1199	-133	296	302	NHGMHFR					Mascot
910.4451	910.325	-0.1201	-132	178	184	AVYECLR			Carbamidomethyl (C)[5]		Mascot
914.4049	914.2822	-0.1227	-134	296	302	NHGMHFR			Oxidation (M)[4]		Mascot
1116.583	1116.4386	-0.1444	-129	412	421	VALEACVQAR			Carbamidomethyl (C)[6]		Mascot
1170.5585	1170.4877	-0.0708	-60	294	302	QKNHGMHFR			Oxidation (M)[6]		Mascot
1187.6643	1187.5109	-0.1534	-129	276	285	DNGLLLHIHR					Mascot
1245.6433	1245.4706	-0.1727	-139	430	440	EGNEIIREASK					Mascot
1245.6433	1245.4706	-0.1727	-139	430	440	EGNEIIREASK					Mascot
1275.7307	1275.5624	-0.1683	-132	330	340	EITLGFVDLLR	7	0			Mascot
1275.7307	1275.5624	-0.1683	-132	330	340	EITLGFVDLLR					Mascot
1465.7546	1465.5588	-0.1958	-134	137	149	TFQGPPHGIQVER					Mascot
1475.7351	1475.548	-0.1871	-127	206	217	DRFLFCAEAIYK					Mascot
1488.6926	1488.5103	-0.1823	-122	454	465	EIKFEFEAMDTL			Oxidation (M)[9]		Mascot
2169.9868	2169.6772	-0.3096	-143	185	203	GGLDFTKDDENVNSQPFMR					Mascot
2185.9819	2185.7007	-0.2812	-129	185	203	GGLDFTKDDENVNSQPFMR			Oxidation (M)[18]		Mascot
3022.4548	3022.0066	-0.4482	-148	249	275	ELGVPIVMHDYLTGGFTANTSLAHYCR			Carbamidomethyl (C)[26]		Mascot
3038.45	3038.0183	-0.4317	-142	249	275	ELGVPIVMHDYLTGGFTANTSLAHYCR			Carbamidomethyl (C)[26], Oxidation (M)[8]		Mascot

Gel Idx/Pos	85/D10	Instr./Gel Origin	BA2060/131017A	Process Sta	Analysis Succeeded
Plate [#] Name	[1] 31496	Instrument Sample Name		Spectra	8

Rank	Protein Name	Accession No	Protein MW	Protein PI	Pep. Count	Protein Score	Protein Score C. I. %	Total Ion Score	Total Ion C. I. %
1	Chlorophyll a-b binding protein of LHCII type I, chloroplastic (Fragment) OS=Cucumis sativus PE=2 S	CB21_CUCSA	27217.7	5.14	8	307	100	254	100

Peptide Information

Calc. Mass	Obsrv. Mass	± da	± ppm	Start Seq.	End Seq.	Sequence	Ion Score	C. I. %	Modification	Rank	Result Type
982.5316	982.4087	-0.1229	-125	86	93	ELEVIHSR					Mascot
983.4984	983.382	-0.1164	-118	115	122	FGEAVWFK	44	99.79			Mascot
983.4984	983.382	-0.1164	-118	115	122	FGEAVWFK					Mascot
1252.6757	1252.5199	-0.1558	-124	84	93	NRELEVIHSR					Mascot
1394.6335	1394.4331	-0.2004	-144	32	44	SVSSGSPWYGPDR					Mascot
1862.9656	1862.726	-0.2396	-129	94	110	WAMLGALGCVFPELLSR					Mascot
1919.9869	1919.7335	-0.2534	-132	94	110	WAMLGALGCVFPELLSR	114	100	Carbamidomethyl (C)[9]		Mascot
1919.9869	1919.7335	-0.2534	-132	94	110	WAMLGALGCVFPELLSR			Carbamidomethyl (C)[9]		Mascot
1935.9819	1935.723	-0.2589	-134	94	110	WAMLGALGCVFPELLSR			Carbamidomethyl (C)[9], Oxidation (M)[3]		Mascot
3108.5173	3108.1006	-0.4167	-134	227	255	GPLENLADHLADPVNNNAWAYATNFVPGK	96	100			Mascot
3108.5173	3108.1006	-0.4167	-134	227	255	GPLENLADHLADPVNNNAWAYATNFVPGK					Mascot
3528.7419	3528.2927	-0.4492	-127	166	200	IAGGPLGEVTDPIYPGGSFDPLGLADDPEAFAELK					Mascot
3939.791	3939.2524	-0.5386	-137	47	83	YLGPFSGEPPSYLTGEFPGDYGWDTAGLSADPETFAK					Mascot

Rank	Protein Name	Accession No	Protein MW	Protein PI	Pep. Count	Protein Score	Protein Score C. I. %	Total Ion Score	Total Ion C. I. %
2	Chlorophyll a-b binding protein 3, chloroplastic OS=Glycine max GN=CAB3 PE=3 SV=1	CB23_SOYBN	27844.1	5.46	7	304	100	262	100

Peptide Information

Calc. Mass	Obsrv. Mass	± da	± ppm	Start Seq.	End Seq.	Sequence	Ion Score	C. I. %	Modification	Rank	Result Type
982.5316	982.4087	-0.1229	-125	21	30	LGPSAPEVGR					Mascot
983.4984	983.382	-0.1164	-118	123	130	FGEAVWFK	44	99.79			Mascot
983.4984	983.382	-0.1164	-118	123	130	FGEAVWFK					Mascot
1252.6757	1252.5199	-0.1558	-124	92	101	NRELEVIHSR					Mascot
1435.66	1435.4767	-0.1833	-128	40	52	QVSSGSPWYGPDR					Mascot
1435.66	1435.4767	-0.1833	-128	40	52	QVSSGSPWYGPDR	104	100			Mascot
1862.9656	1862.726	-0.2396	-129	102	118	WAMLGALGCVFPELLSR					Mascot
1919.9869	1919.7335	-0.2534	-132	102	118	WAMLGALGCVFPELLSR	114	100	Carbamidomethyl (C)[9]		Mascot
1919.9869	1919.7335	-0.2534	-132	102	118	WAMLGALGCVFPELLSR			Carbamidomethyl (C)[9]		Mascot
1935.9819	1935.723	-0.2589	-134	102	118	WAMLGALGCVFPELLSR			Carbamidomethyl (C)[9], Oxidation (M)[3]		Mascot
3528.7419	3528.2927	-0.4492	-127	174	208	IAGGPLGEVTDPIYPGGSFDPLGLADDPEAFAELK					Mascot
3939.791	3939.2524	-0.5386	-137	55	91	YLGPFSGEPPSYLTGEFPGDYGWDTAGLSADPETFAK					Mascot

Rank	Protein Name	Accession No	Protein MW	Protein PI	Pep. Count	Protein Score	Protein Score C. I. %	Total Ion Score	Total Ion C. I. %
3	Chlorophyll a-b binding protein 1, chloroplastic O[illegible] mays GN=CAB1 PE=3 SV=1	CB21_MAIZE	27797.9	5.14	6	190	100	158	100

Peptide Information

Calc. Mass	Obsrv. Mass	± da	± ppm	Start Seq.	End Seq.	Sequence	Ion Score	C. I. %	Modification	Rank	Result Type
982.5316	982.4087	-0.1229	-125	93	100	ELEVIHSR					Mascot
983.4984	983.382	-0.1164	-118	122	129	FGEAVWFK	44	99.79			Mascot
983.4984	983.382	-0.1164	-118	122	129	FGEAVWFK					Mascot
1252.6757	1252.5199	-0.1558	-124	91	100	NRELEVIHSR					Mascot
1862.9656	1862.726	-0.2396	-129	101	117	WAMLGALGCVFPELLSR					Mascot
1871.8911	1871.7753	-0.1158	-62	1	19	MAASTMAISSTAMAGTPIK			Oxidation (M)[1,6]		Mascot
1887.886	1887.7323	-0.1537	-81	1	19	MAASTMAISSTAMAGTPIK			Oxidation (M)[1,6,13]		Mascot
1919.9869	1919.7335	-0.2534	-132	101	117	WAMLGALGCVFPELLSR	114	100	Carbamidomethyl (C)[9]		Mascot
1919.9869	1919.7335	-0.2534	-132	101	117	WAMLGALGCVFPELLSR			Carbamidomethyl (C)[9]		Mascot
1935.9819	1935.723	-0.2589	-134	101	117	WAMLGALGCVFPELLSR			Carbamidomethyl (C)[9], Oxidation (M)[3]		Mascot
3939.791	3939.2524	-0.5386	-137	54	90	YLGPFSGEPPSYLTGEFPGDYGWDTAGLSADPETFAK					Mascot

Rank	Protein Name	Accession No	Protein MW	Protein PI	Pep. Count	Protein Score	Protein Score C. I. %	Total Ion Score	Total Ion C. I. %
4	Chlorophyll a-b binding protein AB96 (Fragment) OS=Pisum sativum GN=AB96 PE=1 SV=1	CB21_PEA	24312.2	4.98	5	183	100	158	100

Peptide Information

Calc. Mass	Obsrv. Mass	± da	± ppm	Start Seq.	End Seq.	Sequence	Ion Score	C. I. %	Modification	Rank	Result Type
982.5316	982.4087	-0.1229	-125	59	66	ELEVIHSR					Mascot
983.4984	983.382	-0.1164	-118	88	95	FGEAVWFK	44	99.79			Mascot
983.4984	983.382	-0.1164	-118	88	95	FGEAVWFK					Mascot
1252.6757	1252.5199	-0.1558	-124	57	66	NRELEVIHSR					Mascot
1862.9656	1862.726	-0.2396	-129	67	83	WAMLGALGCVFPELLSR					Mascot
1919.9869	1919.7335	-0.2534	-132	67	83	WAMLGALGCVFPELLSR	114	100	Carbamidomethyl (C)[9]		Mascot
1919.9869	1919.7335	-0.2534	-132	67	83	WAMLGALGCVFPELLSR			Carbamidomethyl (C)[9]		Mascot
1935.9819	1935.723	-0.2589	-134	67	83	WAMLGALGCVFPELLSR			Carbamidomethyl (C)[9], Oxidation (M)[3]		Mascot
3124.5122	3124.0996	-0.4126	-132	200	228	GPLENLADHLADPVNNNAWSYATNFVPGK					Mascot

Rank	Protein Name	Accession No	Protein MW	Protein PI	Pep. Count	Protein Score	Protein Score C. I. %	Total Ion Score	Total Ion C. I. %
5	Chlorophyll a-b binding protein AB80, chloroplast OS=Pisum sativum GN=AB80 PE=1 SV=1	CB22_PEA	28635.4	5.47	5	180	100	158	100

Peptide Information

Calc. Mass	Obsrv. Mass	± da	± ppm	Start Seq.	End Seq.	Sequence	Ion Score	C. I. %	Modification	Rank	Result Type
982.5316	982.4087	-0.1229	-125	100	107	ELEVIHSR					Mascot
983.4984	983.382	-0.1164	-118	129	136	FGEAVWFK	44	99.79			Mascot
983.4984	983.382	-0.1164	-118	129	136	FGEAVWFK					Mascot
1252.6757	1252.5199	-0.1558	-124	98	107	NRELEVIHSR					Mascot
1862.9656	1862.726	-0.2396	-129	108	124	WAMLGALGCVFPELLSR					Mascot
1919.9869	1919.7335	-0.2534	-132	108	124	WAMLGALGCVFPELLSR	114	100	Carbamidomethyl (C)[9]		Mascot
1919.9869	1919.7335	-0.2534	-132	108	124	WAMLGALGCVFPELLSR			Carbamidomethyl (C)[9]		Mascot
1935.9819	1935.723	-0.2589	-134	108	124	WAMLGALGCVFPELLSR			Carbamidomethyl (C)[9], Oxidation (M)[3]		Mascot
3124.5122	3124.0996	-0.4126	-132	241	269	GPLENLADHLADPVNNNAWSYATNFVPGK					Mascot

Rank	Protein Name	Accession No	Protein MW	Protein PI	Pep. Count	Protein Score	Protein Score C. I. %	Total Ion Score	Total Ion C. I. %
6	Chlorophyll a-b binding protein 8, chloroplastic OS=Pisum sativum GN=CAB8 PE=1 SV=1	CB28_PEA	28508.2	5.16	4	173	100	158	100

Peptide Information

Calc. Mass	Obsrv. Mass	± da	± ppm	Start Seq.	End Seq.	Sequence	Ion Score	C. I. %	Modification	Rank	Result Type
982.5316	982.4087	-0.1229	-125	99	106	ELEVIHSR					Mascot
983.4984	983.382	-0.1164	-118	128	135	FGEAVWFK	44	99.79			Mascot
983.4984	983.382	-0.1164	-118	128	135	FGEAVWFK					Mascot
1252.6757	1252.5199	-0.1558	-124	97	106	NRELEVIHSR					Mascot
1862.9656	1862.726	-0.2396	-129	107	123	WAMLGALGCVFPELLSR					Mascot
1919.9869	1919.7335	-0.2534	-132	107	123	WAMLGALGCVFPELLSR	114	100	Carbamidomethyl (C)[9]		Mascot
	1919.7335										Mascot
1919.9869	1919.7335	-0.2534	-132	107	123	WAMLGALGCVFPELLSR			Carbamidomethyl (C)[9]		Mascot
1935.9819	1935.723	-0.2589	-134	107	123	WAMLGALGCVFPELLSR			Carbamidomethyl (C)[9], Oxidation (M)[3]		Mascot

Rank	Protein Name	Accession No	Protein MW	Protein PI	Pep. Count	Protein Score	Protein Score C. I. %	Total Ion Score	Total Ion C. I. %
7	Chlorophyll a-b binding protein 1, chloroplastic OS=Oryza sativa subsp. japonica GN=CAB1R PE=2 SV=2	CB21_ORYSJ	27996.2	5.14	5	164	100	140	100

Peptide Information

Calc. Mass	Obsrv. Mass	± da	± ppm	Start Seq.	End Seq.	Sequence	Ion Score	C. I. %	Modification	Rank	Result Type
982.5316	982.4087	-0.1229	-125	96	103	ELEVIHSR					Mascot
983.4984	983.382	-0.1164	-118	125	132	FGEAVWFK	44	99.79			Mascot
983.4984	983.382	-0.1164	-118	125	132	FGEAVWFK					Mascot
1252.6757	1252.5199	-0.1558	-124	94	103	NRELEVIHSR					Mascot
1862.9656	1862.726	-0.2396	-129	104	120	WAMLGALGCVFPELLAR			Oxidation (M)[3]		Mascot
1903.9921	1903.7274	-0.2647	-139	104	120	WAMLGALGCVFPELLAR			Carbamidomethyl (C)[9]		Mascot
1919.9869	1919.7335	-0.2534	-132	104	120	WAMLGALGCVFPELLAR			Carbamidomethyl (C)[9], Oxidation (M)[3]		Mascot
1919.9869	1919.7335	-0.2534	-132	104	120	WAMLGALGCVFPELLAR			Carbamidomethyl (C)[9], Oxidation (M)[3]		Mascot
3108.5173	3108.1006	-0.4167	-134	237	265	GPLENLADHLADPVNNNAWAYATNFVPGK	96	100			Mascot
3108.5173	3108.1006	-0.4167	-134	237	265	GPLENLADHLADPVNNNAWAYATNFVPGK					Mascot

Rank	Protein Name	Accession No	Protein MW	Protein PI	Pep. Count	Protein Score	Protein Score C. I. %	Total Ion Score	Total Ion C. I. %
8	Chlorophyll a-b binding protein 1A, chloroplastic OS=Pyrus pyrifolia PE=2 SV=1	CB2A_PYRPY	29476.7	5.25	5	163	100	140	100

Peptide Information

Calc. Mass	Obsrv. Mass	± da	± ppm	Start Seq.	End Seq.	Sequence	Ion Score	C. I. %	Modification	Rank	Result Type
982.5316	982.4087	-0.1229	-125	109	116	ELEVIHSR					Mascot
983.4984	983.382	-0.1164	-118	138	145	FGEAVWFK	44	99.79			Mascot
983.4984	983.382	-0.1164	-118	138	145	FGEAVWFK					Mascot
1252.6757	1252.5199	-0.1558	-124	107	116	NRELEVIHSR					Mascot
1862.9656	1862.726	-0.2396	-129	117	133	WAMLGALGCVFPELLAR			Oxidation (M)[3]		Mascot
1903.9921	1903.7274	-0.2647	-139	117	133	WAMLGALGCVFPELLAR			Carbamidomethyl (C)[9]		Mascot
1919.9869	1919.7335	-0.2534	-132	117	133	WAMLGALGCVFPELLAR			Carbamidomethyl (C)[9], Oxidation (M)[3]		Mascot
1919.9869	1919.7335	-0.2534	-132	117	133	WAMLGALGCVFPELLAR			Carbamidomethyl (C)[9], Oxidation (M)[3]		Mascot
3108.5173	3108.1006	-0.4167	-134	250	278	GPIENLADHLADPVNNNAWAYATNFVPGK	96	100			Mascot
3108.5173	3108.1006	-0.4167	-134	250	278	GPIENLADHLADPVNNNAWAYATNFVPGK					Mascot

Rank	Protein Name	Accession No	Protein MW	Protein PI	Pep. Count	Protein Score	Protein Score C. I. %	Total Ion Score	Total Ion C. I. %
9	Chlorophyll a-b binding protein 40, chloroplastic OS=Nicotiana tabacum GN=CAB40 PE=2 SV=1	CB24_TOBAC	28279.2	5.48	4	157	100	140	100

Peptide Information

Calc. Mass	Obsrv. Mass	± da	± ppm	Start Seq.	End Seq.	Sequence	Ion Score	C. I. %	Modification	Rank	Result Type
983.4984	983.382	-0.1164	-118	127	134	FGEAVWFK	44	99.79			Mascot
983.4984	983.382	-0.1164	-118	127	134	FGEAVWFK					Mascot
1408.6492	1408.4617	-0.1875	-133	44	56	TVSSGSPWYGPDR					Mascot
1862.9656	1862.726	-0.2396	-129	106	122	WAMLGALGCVFPELLAR			Oxidation (M)[3]		Mascot
1903.9921	1903.7274	-0.2647	-139	106	122	WAMLGALGCVFPELLAR			Carbamidomethyl (C)[9]		Mascot
1919.9869	1919.7335	-0.2534	-132	106	122	WAMLGALGCVFPELLAR			Carbamidomethyl (C)[9], Oxidation (M)[3]		Mascot
1919.9869	1919.7335	-0.2534	-132	106	122	WAMLGALGCVFPELLAR			Carbamidomethyl (C)[9], Oxidation (M)[3]		Mascot
3108.5173	3108.1006	-0.4167	-134	239	267	GPLENLADHLADPVNNNAWAYATNFVPGK	96	100			Mascot
3108.5173	3108.1006	-0.4167	-134	239	267	GPLENLADHLADPVNNNAWAYATNFVPGK					Mascot

Rank	Protein Name	Accession No	Protein MW	Protein PI	Pep. Count	Protein Score	Protein Score C. I. %	Total Ion Score	Total Ion C. I. %
10	Chlorophyll a-b binding protein of LHCII type 1 (Fragment) OS=Cucumis sativus PE=2 SV=1	CB22_CUCSA	21990.9	4.52	3	156	100	140	100

Peptide Information

Calc. Mass	Obsrv. Mass	± da	± ppm	Start Seq.	End Seq.	Sequence	Ion Score	C. I. %	Modification	Rank	Result Type
983.4984	983.382	-0.1164	-118	66	73	FGEAVWFK	44	99.79			Mascot
983.4984	983.382	-0.1164	-118	66	73	FGEAVWFK					Mascot
3108.5173	3108.1006	-0.4167	-134	178	206	GPLENLADHLADPVNNNAWAYATNFVPGK	96	100			Mascot
3108.5173	3108.1006	-0.4167	-134	178	206	GPLENLADHLADPVNNNAWAYATNFVPGK					Mascot
3528.7419	3528.2927	-0.4492	-127	117	151	IAGGPLGEVTDPIYPGGSFDPLGLADDPEAFAELK					Mascot

Gel Idx/Pos	86/D11	Instr./Gel Origin	BA2060/131017A	Process Sta	Analysis Succeeded
Plate [#] Name	[1] 31496	Instrument Sample Name		Spectra	8

Rank	Protein Name	Accession No	Protein MW	Protein PI	Pep. Count	Protein Score	Protein Score C. I. %	Total Ion Score	Total Ion C. I. %
1	Chlorophyll a-b binding protein 2, chloroplastic (Fragments) OS=Populus euphratica PE=1 SV=1	CB22_POPEU	3798.9	8.2	4	161	100	117	100

Peptide Information

Calc. Mass	Obsrv. Mass	± da	± ppm	Start Seq.	End Seq.	Sequence	Ion Score	C. I. %	Modification	Rank	Result Type
982.5316	982.4121	-0.1195	-122	17	24	ELEVIHSR					Mascot
983.4984	983.3867	-0.1117	-114	25	32	FGEAVWFK	46	99.796			Mascot
983.4984	983.3867	-0.1117	-114	25	32	FGEAVWFK					Mascot
1252.6757	1252.559	-0.1167	-93	15	24	NRELEVIHSR					Mascot
1601.8071	1601.6096	-0.1975	-123	1	14	SAPQSIWYGPDRPK	71	100			Mascot
1601.8071	1601.6096	-0.1975	-123	1	14	SAPQSIWYGPDRPK					Mascot

Rank	Protein Name	Accession No	Protein MW	Protein PI	Pep. Count	Protein Score	Protein Score C. I. %	Total Ion Score	Total Ion C. I. %
2	Chlorophyll a-b binding protein, chloroplastic OS[illegible] sativa subsp. japonica GN=RCABP89 PE=2 SV=1	CB23_ORYSJ	28477.4	5.62	4	132	100	117	100

Peptide Information

Calc. Mass	Obsrv. Mass	± da	± ppm	Start Seq.	End Seq.	Sequence	Ion Score	C. I. %	Modification	Rank	Result Type
982.5316	982.4121	-0.1195	-122	94	101	ELEVIHSR					Mascot
983.4984	983.3867	-0.1117	-114	123	130	FGEAVWFK	46	99.796			Mascot
983.4984	983.3867	-0.1117	-114	123	130	FGEAVWFK					Mascot
1252.6757	1252.559	-0.1167	-93	92	101	NRELEVIHSR					Mascot
1601.8071	1601.6096	-0.1975	-123	41	54	SAPQSIWYGPDRPK	71	100			Mascot
1601.8071	1601.6096	-0.1975	-123	41	54	SAPQSIWYGPDRPK					Mascot

Rank	Protein Name	Accession No	Protein MW	Protein PI	Pep. Count	Protein Score	Protein Score C. I. %	Total Ion Score	Total Ion C. I. %
3	Chlorophyll a-b binding protein 2, chloroplastic OS=Glycine max GN=CAB2 PE=3 SV=1	CB22_SOYBN	27104.8	5.84	6	76	99.915	46	99.796

Peptide Information

Calc. Mass	Obsrv. Mass	± da	± ppm	Start Seq.	End Seq.	Sequence	Ion Score	C. I. %	Modification	Rank	Result Type
982.5316	982.4121	-0.1195	-122	87	94	ELEVIHSR					Mascot
983.4984	983.3867	-0.1117	-114	116	123	FGEAVWFK	46	99.796			Mascot
983.4984	983.3867	-0.1117	-114	116	123	FGEAVWFK					Mascot
1252.6757	1252.559	-0.1167	-93	85	94	NRELEVIHSR					Mascot
1408.6492	1408.4691	-0.1801	-128	41	53	TVSSGSPWYGPDR					Mascot
1903.9921	1903.7186	-0.2735	-144	95	111	WAMLGALGCVFPELLAR			Carbamidomethyl (C)[9]		Mascot
1919.9869	1919.7451	-0.2418	-126	95	111	WAMLGALGCVFPELLAR			Carbamidomethyl (C)[9], Oxidation (M)[3]		Mascot
2594.2634	2593.9202	-0.3432	-132	228	251	GPLENLADHLADPVNNNAWAYATK					Mascot
2594.2634	2593.9202	-0.3432	-132	228	251	GPLENLADHLADPVNNNAWAYATK					Mascot

Rank	Protein Name	Accession No	Protein MW	Protein PI	Pep. Count	Protein Score	Protein Score C. I. %	Total Ion Score	Total Ion C. I. %
4	Chlorophyll a-b binding protein 3, chloroplastic OS=Glycine max GN=CAB3 PE=3 SV=1	CB23_SOYBN	27844.1	5.46	6	74	99.878	46	99.796

Peptide Information

Calc. Mass	Obsrv. Mass	± da	± ppm	Start Seq.	End Seq.	Sequence	Ion Score	C. I. %	Modification	Rank	Result Type
982.5316	982.4121	-0.1195	-122	21	30	LGPSAPEVGR					Mascot
983.4984	983.3867	-0.1117	-114	123	130	FGEAVWFK	46	99.796			Mascot
983.4984	983.3867	-0.1117	-114	123	130	FGEAVWFK					Mascot
1252.6757	1252.559	-0.1167	-93	92	101	NRELEVIHSR					Mascot
1435.66	1435.4834	-0.1766	-123	40	52	QVSSGSPWYGPDR					Mascot
1662.8234	1662.6746	-0.1488	-89	40	54	QVSSGSPWYGPDRVK					Mascot
1919.9869	1919.7451	-0.2418	-126	102	118	WAMLGALGCVFPELLSR			Carbamidomethyl (C)[9]		Mascot
1935.9819	1935.726	-0.2559	-132	102	118	WAMLGALGCVFPELLSR			Carbamidomethyl (C)[9], Oxidation (M)[3]		Mascot

Rank	Protein Name	Accession No	Protein MW	Protein PI	Pep. Count	Protein Score	Protein Score C. I. %	Total Ion Score	Total Ion C. I. %
5	Chlorophyll a-b binding protein of LHCII type I, chloroplastic (Fragment) OS=Cucumis sativus PE=2 S	CB21_CUCSA	27217.7	5.14	5	68	99.467	46	99.796

Peptide Information

Calc. Mass	Obsrv. Mass	± da	± ppm	Start Seq.	End Seq.	Sequence	Ion Score	C. I. %	Modification	Rank	Result Type
982.5316	982.4121	-0.1195	-122	86	93	ELEVIHSR					Mascot
983.4984	983.3867	-0.1117	-114	115	122	FGEAVWFK	46	99.796			Mascot
983.4984	983.3867	-0.1117	-114	115	122	FGEAVWFK					Mascot
1252.6757	1252.559	-0.1167	-93	84	93	NRELEVIHSR					Mascot
1781.8453	1781.7201	-0.1252	-70	28	44	TASKSVSSGSPWYGPDR					Mascot
1919.9869	1919.7451	-0.2418	-126	94	110	WAMLGALGCVFPELLSR			Carbamidomethyl (C)[9]		Mascot
1935.9819	1935.726	-0.2559	-132	94	110	WAMLGALGCVFPELLSR			Carbamidomethyl (C)[9], Oxidation (M)[3]		Mascot

Rank	Protein Name	Accession No	Protein MW	Protein PI	Pep. Count	Protein Score	Protein Score C. I. %	Total Ion Score	Total Ion C. I. %
6	Chlorophyll a-b binding protein 1, chloroplastic O[illegible] mays GN=CAB1 PE=3 SV=1	CB21_MAIZE	27797.9	5.14	5	68	99.429	46	99.796

Peptide Information

Calc. Mass	Obsrv. Mass	± da	± ppm	Start Seq.	End Seq.	Sequence	Ion Score	C. I. %	Modification	Rank	Result Type
982.5316	982.4121	-0.1195	-122	93	100	ELEVIHSR					Mascot
983.4984	983.3867	-0.1117	-114	122	129	FGEAVWFK	46	99.796			Mascot
983.4984	983.3867	-0.1117	-114	122	129	FGEAVWFK					Mascot
1252.6757	1252.559	-0.1167	-93	91	100	NRELEVIHSR					Mascot
1887.886	1887.7224	-0.1636	-87	1	19	MAASTMAISSTAMAGTPIK			Oxidation (M)[1,6,13]		Mascot
1919.9869	1919.7451	-0.2418	-126	101	117	WAMLGALGCVFPELLSR			Carbamidomethyl (C)[9]		Mascot
1935.9819	1935.726	-0.2559	-132	101	117	WAMLGALGCVFPELLSR			Carbamidomethyl (C)[9], Oxidation (M)[3]		Mascot

Rank	Protein Name	Accession No	Protein MW	Protein PI	Pep. Count	Protein Score	Protein Score C. I. %	Total Ion Score	Total Ion C. I. %
7	Chlorophyll a-b binding protein 8, chloroplastic OS=Pisum sativum GN=CAB8 PE=1 SV=1	CB28_PEA	28508.2	5.16	5	67	99.359	46	99.796

Peptide Information

Calc. Mass	Obsrv. Mass	± da	± ppm	Start Seq.	End Seq.	Sequence	Ion Score	C. I. %	Modification	Rank	Result Type
982.5316	982.4121	-0.1195	-122	99	106	ELEVIHSR					Mascot
983.4984	983.3867	-0.1117	-114	128	135	FGEAVWFK	46	99.796			Mascot
983.4984	983.3867	-0.1117	-114	128	135	FGEAVWFK					Mascot
1252.6757	1252.559	-0.1167	-93	97	106	NRELEVIHSR					Mascot
1605.802	1605.61	-0.192	-120	45	59	VASSGSPWYGPDRVK					Mascot
1919.9869	1919.7451	-0.2418	-126	107	123	WAMLGALGCVFPELLSR			Carbamidomethyl (C)[9]		Mascot
1935.9819	1935.726	-0.2559	-132	107	123	WAMLGALGCVFPELLSR			Carbamidomethyl (C)[9], Oxidation (M)[3]		Mascot

Rank	Protein Name	Accession No	Protein MW	Protein PI	Pep. Count	Protein Score	Protein Score C. I. %	Total Ion Score	Total Ion C. I. %
8	Chlorophyll a-b binding protein AB80, chloroplast OS=Pisum sativum GN=AB80 PE=1 SV=1	CB22_PEA	28635.4	5.47	5	67	99.344	46	99.796

Peptide Information

Calc. Mass	Obsrv. Mass	± da	± ppm	Start Seq.	End Seq.	Sequence	Ion Score	C. I. %	Modification	Rank	Result Type
982.5316	982.4121	-0.1195	-122	100	107	ELEVIHSR					Mascot
983.4984	983.3867	-0.1117	-114	129	136	FGEAVWFK	46	99.796			Mascot
983.4984	983.3867	-0.1117	-114	129	136	FGEAVWFK					Mascot
1252.6757	1252.559	-0.1167	-93	98	107	NRELEVIHSR					Mascot
1605.802	1605.61	-0.192	-120	46	60	VASSGSPWYGPDRVK					Mascot
1919.9869	1919.7451	-0.2418	-126	108	124	WAMLGALGCVFPELLSR			Carbamidomethyl (C)[9]		Mascot
1935.9819	1935.726	-0.2559	-132	108	124	WAMLGALGCVFPELLSR			Carbamidomethyl (C)[9], Oxidation (M)[3]		Mascot

Rank	Protein Name	Accession No	Protein MW	Protein PI	Pep. Count	Protein Score	Protein Score C. I. %	Total Ion Score	Total Ion C. I. %
9	Chlorophyll a-b binding protein 1A, chloroplastic OS=Pyrus pyrifolia PE=2 SV=1	CB2A_PYRPY	29476.7	5.25	5	66	99.247	46	99.796

Peptide Information

Calc. Mass	Obsrv. Mass	± da	± ppm	Start Seq.	End Seq.	Sequence	Ion Score	C. I. %	Modification	Rank	Result Type
982.5316	982.4121	-0.1195	-122	109	116	ELEVIHSR					Mascot
983.4984	983.3867	-0.1117	-114	138	145	FGEAVWFK	46	99.796			Mascot
983.4984	983.3867	-0.1117	-114	138	145	FGEAVWFK					Mascot
1170.5242	1170.4955	-0.0287	-25	2	13	ATTMASCGIGSR			Oxidation (M)[4]		Mascot
1252.6757	1252.559	-0.1167	-93	107	116	NRELEVIHSR					Mascot
1903.9921	1903.7186	-0.2735	-144	117	133	WAMLGALGCVFPELLAR			Carbamidomethyl (C)[9]		Mascot
1919.9869	1919.7451	-0.2418	-126	117	133	WAMLGALGCVFPELLAR			Carbamidomethyl (C)[9], Oxidation (M)[3]		Mascot

Rank	Protein Name	Accession No	Protein MW	Protein PI	Pep. Count	Protein Score	Protein Score C. I. %	Total Ion Score	Total Ion C. I. %
10	Chlorophyll a-b binding protein AB96 (Fragment) OS=Pisum sativum GN=AB96 PE=1 SV=1	CB21_PEA	24312.2	4.98	4	63	98.352	46	99.796

Peptide Information

Calc. Mass	Obsrv. Mass	± da	± ppm	Start Seq.	End Seq.	Sequence	Ion Score	C. I. %	Modification	Rank	Result Type
Calc. Mass	Obsrv. Mass	± da	± ppm	Seq.	Seq.	Sequence	Score	C. I. %	Modification	Rank	Result Type
982.5316	982.4121	-0.1195	-122	59	66	ELEVIHSR					Mascot
983.4984	983.3867	-0.1117	-114	88	95	FGEAVWFK	46	99.786			Mascot
983.4984	983.3867	-0.1117	-114	88	95	FGEAVWFK					Mascot
1252.6757	1252.559	-0.1167	-93	57	66	NRELEVIHSR					Mascot
1919.9869	1919.7451	-0.2418	-126	67	83	WAMLGALGCVFPELLSR			Carbamidomethyl (C)[9]		Mascot
1935.9819	1935.726	-0.2559	-132	67	83	WAMLGALGCVFPELLSR			Carbamidomethyl (C)[9], Oxidation (M)[3]		Mascot

Gel Idx/Pos	87/D12	Instr./Gel Origin	BA2060/131017A	Process Sta	Analysis Succeeded
Plate [#] Name	[1] 31496	Instrument Sample Name		Spectra	8

Rank	Protein Name	Accession No	Protein MW	Protein PI	Pep. Count	Protein Score	Protein Score C. I. %	Total Ion Score	Total Ion C. I. %
1	Chlorophyll a-b binding protein 13, chloroplastic OS=Solanum lycopersicum GN=CAB13 PE=1 SV=1	CB23_SOLLC	28604.4	5.09	6	255	100	226	100

Peptide Information

Calc. Mass	Obsrv. Mass	± da	± ppm	Start Seq.	End Seq.	Sequence	Ion Score	C. I. %	Modification	Rank	Result Type
805.4243	805.3296	-0.0947	-118	126	131	EPVWFK					Mascot
894.5155	894.4147	-0.1008	-113	94	101	ALEVIHGR	34	96.516			Mascot
894.5155	894.4147	-0.1008	-113	94	101	ALEVIHGR					Mascot
1294.683	1294.5317	-0.1513	-117	122	131	VDFKEPVWFK					Mascot
1294.683	1294.5317	-0.1513	-117	122	131	VDFKEPVWFK	64	99.997			Mascot
1601.7053	1601.6187	-0.0866	-54	40	52	FTMSNDLWYGPDR					Mascot
1994.0278	1993.7434	-0.2844	-143	219	236	LAMFSMFGFFVQAIVTGK					Mascot
2664.3416	2664.0232	-0.3184	-120	237	260	GPLENLLDHLDNPVANNAWVYATK	128	100			Mascot
2664.3416	2664.0232	-0.3184	-120	237	260	GPLENLLDHLDNPVANNAWVYATK					Mascot

Rank	Protein Name	Accession No	Protein MW	Protein PI	Pep. Count	Protein Score	Protein Score C. I. %	Total Ion Score	Total Ion C. I. %
2	Chlorophyll a-b binding protein of LHCII type III, chloroplastic OS=Hordeum vulgare GN=LHBC PE=2 SV	CB23_HORVU	28740.5	4.99	5	57	93.878	34	96.516

Peptide Information

Calc. Mass	Obsrv. Mass	± da	± ppm	Start Seq.	End Seq.	Sequence	Ion Score	C. I. %	Modification	Rank	Result Type
805.4243	805.3296	-0.0947	-118	129	134	EPVWFK					Mascot
894.5155	894.4147	-0.1008	-113	97	104	ALEVIHGR	34	96.516			Mascot
894.5155	894.4147	-0.1008	-113	97	104	ALEVIHGR					Mascot
994.4985	994.4012	-0.0973	-98	2	11	ASTIMAAASR			Oxidation (M)[5]		Mascot
1994.0278	1993.7434	-0.2844	-143	222	239	LAMFSMFGFFVQAIVTGK					Mascot
3766.8123	3766.3818	-0.4305	-114	178	213	INGLDGVGEGNDLYPGGQYFDPLGLADDPVTFAELK					Mascot

Rank	Protein Name	Accession No	Protein MW	Protein PI	Pep. Count	Protein Score	Protein Score C. I. %	Total Ion Score	Total Ion C. I. %
3	Polyphenol oxidase D, chloroplastic OS=Solanum lycopersicum PE=3 SV=2	PPOD_SOLLC	66486.6	6.76	9	33	0		

Peptide Information

Calc. Mass	Obsrv. Mass	± da	± ppm	Start Seq.	End Seq.	Sequence	Ion Score	C. I. %	Modification	Rank	Result Type
870.4791	870.4427	-0.0364	-42	383	389	RTDITHK					Mascot
882.3814	882.4666	0.0852	97	372	377	MWSEWK			Oxidation (M)[1]		Mascot
1189.5453	1189.4885	-0.0568	-48	291	300	QMVTNAPCPR			Carbamidomethyl (C)[8], Oxidation (M)[2]		Mascot
1192.5126	1192.4043	-0.1083	-91	419	427	MGYDYKPMR			Oxidation (M)[1,8]		Mascot
1192.5126	1192.4043	-0.1083	-91	419	427	MGYDYKPMR			Oxidation (M)[1,8]		Mascot
1243.571	1243.5242	-0.0468	-38	235	244	GMRFPAMYDR					Mascot
1275.5609	1275.5818	0.0209	16	235	244	GMRFPAMYDR			Oxidation (M)[2,7]		Mascot
1919.8801	1919.7322	-0.1479	-77	45	61	VTNTNGNQDETNSVDRR					Mascot
2807.3503	2806.9697	-0.3806	-136	161	185	TQPLNPIGFKQQANIHCAYCNGAYR					Mascot
3749.7739	3749.3318	-0.4421	-118	205	234	WYLYFYESNAGKLIDDPTFALPYWNWDHPK					Mascot
3793.9192	3794.3787	0.4595	121	301	335	MFFGDLMISGITLNSPGTIENIPHGPVHIWSGTVR					Mascot

Rank	Protein Name	Accession No	Protein MW	Protein PI	Pep. Count	Protein Score	Protein Score C. I. %	Total Ion Score	Total Ion C. I. %
4	COP9 signalosome complex subunit 7 (Fragment OS=Brassica oleracea GN=CSN7 PE=1 SV=1	CSN7_BRAOL	8080.1	4.84	4	33	0		

Peptide Information

Calc. Mass	Obsrv. Mass	± da	± ppm	Start Seq.	End Seq.	Sequence	Ion Score	C. I. %	Modification	Rank	Result Type
1208.5253	1208.3923	-0.133	-110	51	60	WADNMSEIDK					Mascot
1224.5201	1224.3909	-0.1292	-106	51	60	WADNMSEIDK			Oxidation (M)[5]		Mascot
1294.6215	1294.5317	-0.0898	-69	11	21	LFAHGTWGDYK					Mascot
1294.6215	1294.5317	-0.0898	-69	11	21	LFAHGTWGDYK					Mascot
1839.9745	1839.7411	-0.2334	-127	22	37	CNASRIPQLSPDQILK			Carbamidomethyl (C)[1]		Mascot
1882.8652	1882.7451	-0.1201	-64	11	26	LFAHGTWGDYKCNASR			Carbamidomethyl (C)[12]		Mascot

Rank	Protein Name	Accession No	Protein MW	Protein PI	Pep. Count	Protein Score	Protein Score C. I. %	Total Ion Score	Total Ion C. I. %
5	Putative beta-galactosidase OS=Dianthus caryop GN=CARSR12 PE=2 SV=1	BGAL_DIACA	82810.7	6.81	4	30	0	23	61.891

Peptide Information

Calc. Mass	Obsrv. Mass	± da	± ppm	Start Seq.	End Seq.	Sequence	Ion Score	C. I. %	Modification	Rank	Result Type
870.4389	870.4427	0.0038	4	155	161	MQVFTTK			Oxidation (M)[1]		Mascot
1192.5813	1192.4043	-0.177	-148	2	11	LCGKENNVMK			Carbamidomethyl (C)[2]		Mascot
1192.5813	1192.4043	-0.177	-148	2	11	LCGKENNVMK			Carbamidomethyl (C)[2]		Mascot
1571.7994	1571.6238	-0.1756	-112	155	167	MQVFTTKIVDMMK					Mascot
1571.7994	1571.6238	-0.1756	-112	155	167	MQVFTTKIVDMMK	23	61.891			Mascot
2748.3264	2747.9619	-0.3645	-133	69	92	AKDSQLDVIQTYVFWNGHEPSEGK					Mascot

Rank	Protein Name	Accession No	Protein MW	Protein PI	Pep. Count	Protein Score	Protein Score C. I. %	Total Ion Score	Total Ion C. I. %
6	Soluble inorganic pyrophosphatase 2 OS=Chlamydomonas reinhardtii GN=ppa2 PE=1 SV=1	IPYR2_CHLRI	22161.1	5.49	5	29	0		

Peptide Information

Calc. Mass	Obsrv. Mass	± da	± ppm	Start Seq.	End Seq.	Sequence	Ion Score	C. I. %	Modification	Rank	Result Type
894.493	894.4147	-0.0783	-88	37	43	VKYELDK					Mascot
894.493	894.4147	-0.0783	-88	37	43	VKYELDK					Mascot
1730.8054	1730.6578	-0.1476	-85	39	52	YELDKDTGLCFVDR			Carbamidomethyl (C)[10]		Mascot
1993.98	1993.7434	-0.2366	-119	170	185	VVKDSLNMYQEHYVPR			Oxidation (M)[8]		Mascot
3338.6123	3338.3379	-0.2744	-82	6	36	GTASHPWHDLHPGNDAPNFVSCVIEIPRGSK					Mascot
3766.7642	3766.3818	-0.3824	-102	1	33	MSFYRGTASHPWHDLHPGNDAPNFVSCVIEIPR			Oxidation (M)[1]		Mascot

Rank	Protein Name	Accession No	Protein MW	Protein PI	Pep. Count	Protein Score	Protein Score C. I. %	Total Ion Score	Total Ion C. I. %
7	Ras-related protein RABB1b OS=Arabidopsis tha GN=RABB1B PE=2 SV=1	RAB1B_ARAT	23160.6	6.52	5	28	0		

Peptide Information

Calc. Mass	Obsrv. Mass	± da	± ppm	Start Seq.	End Seq.	Sequence	Ion Score	C. I. %	Modification	Rank	Result Type
842.4301	842.4175	-0.0126	-15	121	127	CDLAHKR					Mascot
1045.5425	1045.4443	-0.0982	-94	70	77	SITRSYYR					Mascot
2663.3611	2663.0188	-0.3423	-129	47	69	MVTVDGRPIKLQIWDTAGQESFR			Oxidation (M)[1]		Mascot
3353.6152	3353.4277	-0.1875	-56	92	120	ETFNHLASWLEDARQHANPNMSIMLIGNK			Oxidation (M)[21]		Mascot
3765.8816	3766.3818	0.5002	133	152	186	TAQNVEEAFIETAAKILQNIQDGVFDVSNESSGIK					Mascot

Rank	Protein Name	Accession No	Protein MW	Protein PI	Pep. Count	Protein Score	Protein Score C. I. %	Total Ion Score	Total Ion C. I. %
8	ATP synthase epsilon chain, chloroplastic OS=Liriodendron tulipifera GN=atpE PE=3 SV=1	ATPE_LIRTU	14735.8	5.41	4	26	0		

Peptide Information

Calc. Mass	Obsrv. Mass	± da	± ppm	Start Seq.	End Seq.	Sequence	Ion Score	C. I. %	Modification	Rank	Result Type
1243.6827	1243.5242	-0.1585	-127	2	12	TLNLCVLTPNR					Mascot
1601.8492	1601.6187	-0.2305	-144	95	109	TLEIAEANLSRAEGK					Mascot
1839.8881	1839.7411	-0.147	-80	53	68	LNDQWLTMALMGGFAR			Oxidation (M)[8]		Mascot
3337.8953	3338.3379	0.4426	133	21	52	EIILSTNSGQIGVLPNHAPIATAVDIGILRVR					Mascot

Rank	Protein Name	Accession No	Protein MW	Protein PI	Pep. Count	Protein Score	Protein Score C. I. %	Total Ion Score	Total Ion C. I. %
9	Salt tolerance-like protein OS=Arabidopsis thalia GN=STH PE=1 SV=2	STH_ARATH	26639.7	4.9	6	25	0		

Peptide Information

Calc. Mass	Obsrv. Mass	± da	± ppm	Start Seq.	End Seq.	Sequence	Ion Score	C. I. %	Modification	Rank	Result Type
994.4332	994.4012	-0.032	-32	3	10	IQCDVCEK			Carbamidomethyl (C)[3]		Mascot
1179.5497	1179.4646	-0.0851	-72	2	10	KIQCDVCEK			Carbamidomethyl (C)[4,7]		Mascot
1196.5472	1196.4031	-0.1441	-120	1	10	MKIQCDVCEK					Mascot
1310.5901	1310.5242	-0.0659	-50	1	10	MKIQCDVCEK			Carbamidomethyl (C)[5,8]		Mascot
1326.5851	1326.5123	-0.0728	-55	1	10	MKIQCDVCEK			Carbamidomethyl (C)[5,8], Oxidation (M)[1]		Mascot
2239.019	2238.8577	-0.1613	-72	221	238	LEYRYDDEEEHFLVPDLG					Mascot
2283.1399	2282.8921	-0.2478	-109	45	63	LFLDSLSTKFPPCDICLEK			Carbamidomethyl (C)[13,16]		Mascot
2696.2219	2696.0088	-0.2131	-79	3	27	IQCDVCEKAPATLICCADEAALCAK			Carbamidomethyl (C)[3,6]		Mascot

Rank	Protein Name	Accession No	Protein MW	Protein PI	Pep. Count	Protein Score	Protein Score C. I. %	Total Ion Score	Total Ion C. I. %
10	Histone-lysine N-methyltransferase EZ2 OS=Zea GN=EZ2 PE=2 SV=1	EZ2_MAIZE	99915.8	8.47	11	25	0		

Peptide Information

Calc. Mass	Obsrv. Mass	± da	± ppm	Start Seq.	End Seq.	Sequence	Ion Score	C. I. %	Modification	Rank	Result Type
805.4301	805.3296	-0.1005	-125	485	491	ILDESTK					Mascot
894.4097	894.4147	0.005	6	16	23	RSDQGMGK			Oxidation (M)[6]		Mascot
894.4097	894.4147	0.005	6	16	23	RSDQGMGK			Oxidation (M)[6]		Mascot
1189.5969	1189.4885	-0.1084	-91	320	329	KPCGHLCYLR					Mascot
1882.8092	1882.7451	-0.0641	-34	496	512	KDMCGESPATTMENVGR			Carbamidomethyl (C)[4]		Mascot
1892.0065	1891.7615	-0.245	-129	150	164	IPPYTTWIFLDKNQR					Mascot
1903.957	1903.7399	-0.2171	-114	320	334	KPCGHLCYLRFPQWR					Mascot
2401.0032	2400.7756	-0.2276	-95	339	361	EMHDDGLAGGATYTMESGTASQR			Oxidation (M)[2]		Mascot
2401.0032	2400.7756	-0.2276	-95	339	361	EMHDDGLAGGATYTMESGTASQR			Oxidation (M)[2]		Mascot
2416.998	2416.7832	-0.2148	-89	339	361	EMHDDGLAGGATYTMESGTASQR			Oxidation (M)[2,15]		Mascot
2438.9712	2438.72	-0.2512	-103	587	607	SISGDFAENEQDYMEQDMAAR			Oxidation (M)[14,18]		Mascot
2663.3933	2663.0188	-0.3745	-141	17	42	SDQGMGKDAAAASVVPIHANLTQLIR					Mascot
2664.1301	2664.0232	-0.1069	-40	587	609	SISGDFAENEQDYMEQDMAARTR					Mascot
2664.1301	2664.0232	-0.1069	-40	587	609	SISGDFAENEQDYMEQDMAARTR					Mascot
2680.125	2680.0144	-0.1106	-41	587	609	SISGDFAENEQDYMEQDMAARTR			Oxidation (M)[14]		Mascot
2686.0459	2686.0149	-0.031	-12	636	657	TDDGKQCYTQYSPCACQQMCGK			Carbamidomethyl (C)[7,14,16,20]		Mascot
2696.1199	2696.0088	-0.1111	-41	587	609	SISGDFAENEQDYMEQDMAARTR			Oxidation (M)[14,18]		Mascot
2702.0408	2701.9802	-0.0606	-22	636	657	TDDGKQCYTQYSPCACQQMCGK			Carbamidomethyl (C)[7,14,16,20], Oxidation (M)[19]		Mascot

Gel Idx/Pos	88/D13	Instr./Gel Origin	BA2060/131017A	Process State	Analysis Succeeded
Plate [#] Name	[1] 31496	Instrument Sample Name		Spectra	8

Rank	Protein Name	Accession No.	Protein MW	Protein PI	Pep. Count	Protein Score	Protein Score C. I. %	Total Ion Score	Total Ion C. I. %
1	2-Cys peroxiredoxin BAS1-like, chloroplastic OS=Arabidopsis thaliana GN=At5g06290 PE=2 SV=3	BAS1B_ARAT	29761.2	5.55	5	159	100	137	100

Peptide Information

Calc. Mass	Obsrv. Mass	± da	± ppm	Start Seq.	End Seq.	Sequence	Ion Score	C. I. %	Modification	Rank	Result Type
805.4818	805.3957	-0.0861	-107	203	209	GLFIIDK					Mascot
844.3723	844.4271	0.0548	65	137	142	YEEFEK					Mascot
1748.9065	1748.7092	-0.1973	-113	168	184	SGGLGDLNYPLVSDITK					Mascot
1748.9065	1748.7092	-0.1973	-113	168	184	SGGLGDLNYPLVSDITK	47	99.882			Mascot
1877.0015	1876.7882	-0.2133	-114	167	184	KSGGLGDLNYPLVSDITK					Mascot
2857.3826	2857.0408	-0.3418	-120	233	257	TLQALQYVQENPDEVCPAGWKPGEK			Carbamidomethyl (C)[16]		Mascot
2857.3826	2857.0408	-0.3418	-120	233	257	TLQALQYVQENPDEVCPAGWKPGEK	90	100	Carbamidomethyl (C)[16]		Mascot

Rank	Protein Name	Accession No.	Protein MW	Protein PI	Pep. Count	Protein Score	Protein Score C. I. %	Total Ion Score	Total Ion C. I. %
2	Protein P21 OS=Glycine max PE=1 SV=1	P21_SOYBN	21448.6	4.84	5	117	100	91	100

Peptide Information

Calc. Mass	Obsrv. Mass	± da	± ppm	Start Seq.	End Seq.	Sequence	Ion Score	C. I. %	Modification	Rank	Result Type
1070.432	1070.3196	-0.1124	-105	49	58	TGCNFDGSGR	14	0	Carbamidomethyl (C)[3]		Mascot
1070.432	1070.3196	-0.1124	-105	49	58	TGCNFDGSGR			Carbamidomethyl (C)[3]		Mascot
1482.6464	1482.4808	-0.1656	-112	137	149	TQGGCNNPCTVFK			Carbamidomethyl (C)[5,9]		Mascot
1583.6246	1583.4438	-0.1808	-114	59	73	GGCQTGDCGGVLDCK			Carbamidomethyl (C)[3,8,14]		Mascot
1849.8418	1849.6346	-0.2072	-112	120	136	GISCTADINGQCPSELK			Carbamidomethyl (C)[4,12]		Mascot
2127.78	2127.531	-0.249	-117	150	167	TDQYCCNSGSCGPTDYSR			Carbamidomethyl (C)[5,6,11]		Mascot
2127.78	2127.531	-0.249	-117	150	167	TDQYCCNSGSCGPTDYSR	76	100	Carbamidomethyl (C)[5,6,11]		Mascot

Rank	Protein Name	Accession No.	Protein MW	Protein PI	Pep. Count	Protein Score	Protein Score C. I. %	Total Ion Score	Total Ion C. I. %
3	2-Cys peroxiredoxin BAS1, chloroplastic OS=Oryz sativa subsp. japonica GN=BAS1 PE=1 SV=1	BAS1_ORYS	28079.4	5.67	3	103	100	92	100

Peptide Information

Calc. Mass	Obsrv. Mass	± da	± ppm	Start Seq.	End Seq.	Sequence	Ion Score	C. I. %	Modification	Rank	Result Type
805.4818	805.3957	-0.0861	-107	191	197	GLFIIDK					Mascot
1721.9293	1721.7367	-0.1926	-112	198	213	EGVIQHSTINNLAIGR	92	100			Mascot
1721.9293	1721.7367	-0.1926	-112	198	213	EGVIQHSTINNLAIGR					Mascot
2508.3933	2508.0898	-0.3035	-121	191	213	GLFIIDKEGVIQHSTINNLAIGR					Mascot

Rank	Protein Name	Accession No.	Protein MW	Protein PI	Pep. Count	Protein Score	Protein Score C. I. %	Total Ion Score	Total Ion C. I. %
4	2-Cys peroxiredoxin BAS1, chloroplastic OS=Spir oleracea GN=BAS1 PE=2 SV=1	BAS1_SPIOL	28877.8	7.7	4	82	99.977	66	99.998

Peptide Information

Calc. Mass	Obsrv. Mass	± da	± ppm	Start Seq.	End Seq.	Sequence	Ion Score	C. I. %	Modification	Rank	Result Type
805.4818	805.3957	-0.0861	-107	196	202	GLFIIDK					Mascot
1748.9065	1748.7092	-0.1973	-113	161	177	SGGLGDLNYPLISDVTK					Mascot
1748.9065	1748.7092	-0.1973	-113	161	177	SGGLGDLNYPLISDVTK	66	99.998			Mascot
1877.0015	1876.7882	-0.2133	-114	160	177	KSGGLGDLNYPLISDVTK					Mascot
2297.1628	2296.9119	-0.2509	-109	1	22	MACVASSTTLISSPSSRVFPAK			Carbamidomethyl (C)[3]		Mascot
2313.1577	2312.9199	-0.2378	-103	1	22	MACVASSTTLISSPSSRVFPAK			Carbamidomethyl (C)[3], Oxidation (M)[1]		Mascot

Rank	Protein Name	Accession No.	Protein MW	Protein PI	Pep. Count	Protein Score	Protein Score C. I. %	Total Ion Score	Total Ion C. I. %
5	2-Cys peroxiredoxin BAS1, chloroplastic OS=Arabidopsis thaliana GN=BAS1 PE=1 SV=2	BAS1A_ARAT	29073.9	6.92	3	76	99.914	66	99.998

Peptide Information

Calc. Mass	Obsrv. Mass	± da	± ppm	Start Seq.	End Seq.	Sequence	Ion Score	C. I. %	Modification	Rank	Result Type
805.4818	805.3957	-0.0861	-107	196	202	GLFIIDK					Mascot
1748.9065	1748.7092	-0.1973	-113	161	177	SGGLGDLNYPLISDVTK					Mascot
1748.9065	1748.7092	-0.1973	-113	161	177	SGGLGDLNYPLISDVTK	66	99.998			Mascot
1877.0015	1876.7882	-0.2133	-114	160	177	KSGGLGDLNYPLISDVTK					Mascot

Rank	Protein Name	Accession No.	Protein MW	Protein PI	Pep. Count	Protein Score	Protein Score C. I. %	Total Ion Score	Total Ion C. I. %
6	17.8 kDa heat shock protein OS=Oryza sativa subs japonica GN=HSP17.8 PE=2 SV=2	HS178_ORYS	17753.9	6.97	6	36	0		

Peptide Information

Calc. Mass	Obsrv. Mass	± da	± ppm	Start Seq.	End Seq.	Sequence	Ion Score	C. I. %	Modification	Rank	Result Type
805.46	805.3957	-0.0643	-80	1	7	MSLVLSR					Mascot
1465.7865	1465.5931	-0.1934	-132	1	12	MSLVLSRMLLDR			Oxidation (M)[1,8]		Mascot
1539.7146	1539.6171	-0.0975	-63	66	81	AGGEHEDANNAAKAGK					Mascot
1678.7465	1678.603	-0.1435	-85	151	164	AAGDDKPVCCRFWP			Carbamidomethyl (C)[9,10]		Mascot
2089.0308	2088.9258	-0.105	-50	107	126	AAVTQFRLPEDAAADEASAR					Mascot
3384.762	3384.2749	-0.4871	-144	33	63	ETPVAHVFEMDLPGLAKDQVAEVEVVDGHILR					Mascot

Rank	Protein Name	Accession No.	Protein MW	Protein PI	Pep. Count	Protein Score	Protein Score C. I. %	Total Ion Score	Total Ion C. I. %
7	2-Cys peroxiredoxin BAS1, chloroplastic (Fragmen OS=Triticum aestivum GN=TSA PE=1 SV=2	BAS1_WHEA	23312.1	5.71	4	33	0	15	0

Peptide Information

Calc. Mass	Obsrv. Mass	± da	± ppm	Start Seq.	End Seq.	Sequence	Ion Score	C. I. %	Modification	Rank	Result Type
805.4818	805.3957	-0.0861	-107	141	147	GLFIIDK					Mascot Mascot
1189.6536	1189.5022	-0.1514	-127	164	173	SVDETLRTLR					Mascot
1748.9429	1748.7092	-0.2337	-134	106	122	SGGLGDLKYPLVSDVTK					Mascot
1748.9429	1748.7092	-0.2337	-134	106	122	SGGLGDLKYPLVSDVTK	15	0			Mascot
2508.3164	2508.0898	-0.2266	-90	148	170	EGVIQHSTINNLGIGRSVDETLR					Mascot

Rank	Protein Name	Accession No.	Protein MW	Protein PI	Pep. Count	Protein Score	Protein Score C. I. %	Total Ion Score	Total Ion C. I. %
8	Cytochrome P450 83B1 OS=Arabidopsis thaliana GN=CYP83B1 PE=1 SV=1	C83B1_ARAT	56809.6	8.52	9	30	0		

Peptide Information

Calc. Mass	Obsrv. Mass	± da	± ppm	Start Seq.	End Seq.	Sequence	Ion Score	C. I. %	Modification	Rank	Result Type
805.3661	805.3957	0.0296	37	415	420	FMNEHK					Mascot
834.4355	834.3452	-0.0903	-108	277	283	DQPFSIK					Mascot
882.4389	882.4716	0.0327	37	318	324	YPEAMKK			Oxidation (M)[5]		Mascot
1351.6464	1351.5684	-0.078	-58	415	425	FMNEHKGVDFK					Mascot
1465.7832	1465.5931	-0.1901	-130	35	47	GLPIIGNLHQMEK			Oxidation (M)[11]		Mascot
2272.2449	2271.9404	-0.3045	-134	378	398	IGGYDIPAKTIIQVNAWAVSR					Mascot
2297.1462	2296.9119	-0.2343	-102	128	146	MCMVNLFSPNRVASFRPVR			Carbamidomethyl (C)[2], Oxidation (M)[1]		Mascot
2313.1414	2312.9199	-0.2215	-96	128	146	MCMVNLFSPNRVASFRPVR			Carbamidomethyl (C)[2], Oxidation (M)[1,3]		Mascot
2541.2324	2540.8652	-0.3672	-144	160	183	AADQSGTVDLSELLLSFTNCVVCR					Mascot
2691.3643	2690.9707	-0.3936	-146	439	461	RMCPAMHLGIAMVEIPFANLLYK			Carbamidomethyl (C)[3], Oxidation (M)[2]		Mascot

Rank	Protein Name	Accession No.	Protein MW	Protein PI	Pep. Count	Protein Score	Protein Score C. I. %	Total Ion Score	Total Ion C. I. %
9	Nuclear cap-binding protein subunit 2 OS=Arabid thaliana GN=CBP20 PE=1 SV=1	NCBP2_ARAT	29591.2	6.86	6	30	0		

Peptide Information

Calc. Mass	Obsrv. Mass	± da	± ppm	Start Seq.	End Seq.	Sequence	Ion Score	C. I. %	Modification	Rank	Result Type
805.4487	805.3957	-0.053	-66	63	69	IIMGLDK			Oxidation (M)[3]		Mascot
1411.4728	1411.5337	0.0609	43	241	253	ESGDSDDDGEDDR					Mascot
1539.5677	1539.6171	0.0494	32	241	254	ESGDSDDDGEDDRK					Mascot
1882.8619	1882.761	-0.1009	-54	107	121	VDFDWGFQEGRQWGR					Mascot
2541.1423	2540.8652	-0.2771	-109	73	93	TPCGFCFVLFYSREDTEDAVK			Carbamidomethyl (C)[3,6]		Mascot
3416.6895	3416.2629	-0.4266	-125	32	61	ASTTVYIGNVSFYTTEEQLYELFSRAGEIK					Mascot

Rank	Protein Name	Accession No.	Protein MW	Protein PI	Pep. Count	Protein Score	Protein Score C. I. %	Total Ion Score	Total Ion C. I. %
10	Delta-selinene synthase OS=Abies grandis GN=a PE=1 SV=1	TPSD4_ABIG	67924.1	5.57	10	30	0		

Peptide Information

Calc. Mass	Obsrv. Mass	± da	± ppm	Start Seq.	End Seq.	Sequence	Ion Score	C. I. %	Modification	Rank	Result Type
805.4236	805.3957	-0.0279	-35	345	351	IMTEGVR					Mascot
1475.6511	1475.5887	-0.0624	-42	519	529	SLEEFNWEFMK			Oxidation (M)[10]		Mascot
1694.8344	1694.5933	-0.2411	-142	499	513	ENPESTVENALNHIK					Mascot
1703.9268	1703.7292	-0.1976	-116	540	553	FTFNIGRGLQFIYK					Mascot
1721.7291	1721.7367	0.0076	4	485	498	ERGEMASSLQCYMK			Carbamidomethyl (C)[11], Oxidation (M)[5,13]		Mascot
1721.7291	1721.7367	0.0076	4	485	498	ERGEMASSLQCYMK			Carbamidomethyl (C)[11], Oxidation (M)[5,13]		Mascot
1849.7809	1849.6346	-0.1463	-79	145	160	DENGKFFCNFTGEEGR					Mascot
2273.125	2272.9395	-0.1855	-82	232	249	WEARSFLEIYGHNHSWLK					Mascot
2297.0901	2296.9119	-0.1782	-78	51	69	HLLLTEMEMDDGDHDLIKR			Oxidation (M)[7]		Mascot
2313.085	2312.9199	-0.1651	-71	51	69	HLLLTEMEMDDGDHDLIKR			Oxidation (M)[7,9]		Mascot
2508.0703	2508.0898	0.0195	8	519	538	SLEEFNWEFMKQDSVPMCCK			Carbamidomethyl (C)[18]		Mascot
3224.522	3224.1838	-0.3382	-105	297	322	HVEYYSWVVMCIFEPEFSESRIAFAK			Carbamidomethyl (C)[11]		Mascot
3224.522	3224.1838	-0.3382	-105	297	322	HVEYYSWVVMCIFEPEFSESRIAFAK			Carbamidomethyl (C)[11]		Mascot

Gel Idx/Pos	89/D14	Instr./Gel Origin	BA2060/131017A	Process State	Analysis Succeeded
Plate [#] Name	[1] 31496	Instrument Sample Name		Spectra	11

Rank	Protein Name	Accession No.	Protein MW	Protein PI	Pep. Count	Protein Score	Protein Score C. I. %	Total Ion Score	Total Ion C. I. %
1	Ribulose bisphosphate carboxylase small chain chloroplastic OS=Triticum aestivum PE=3 SV=1	RBS2_WHEA	19441.7	8.52	7	40	0		

Peptide Information

Calc. Mass	Obsrv. Mass	± da	± ppm	Start Seq.	End Seq.	Sequence	Ion Score	C. I. %	Modification	Rank	Result Type
1165.571	1165.4768	-0.0942	-81	85	93	WVPCLEFSK			Carbamidomethyl (C)[4]		Mascot
1265.608	1265.5125	-0.0955	-75	31	44	SGSTGLSSVSNGGR					Mascot
1944.9272	1944.83	-0.0972	-50	139	154	EYPDAYVRVIGFDNMR					Mascot
1960.9221	1960.8153	-0.1068	-54	139	154	EYPDAYVRVIGFDNMR			Oxidation (M)[15]		Mascot
1994.0085	1993.7657	-0.2428	-122	1	20	MAPAVMASSATTVAPFQGLK			Oxidation (M)[1]		Mascot
1994.0085	1993.7657	-0.2428	-122	1	20	MAPAVMASSATTVAPFQGLK			Oxidation (M)[1]		Mascot
2367.1218	2367.0051	-0.1167	-49	155	175	QVQCVSFIAFRPPGCEESGKA			Carbamidomethyl (C)[4,15]		Mascot
2367.157	2367.0051	-0.1519	-64	118	138	LPMFGCTDATQVLNEVEEVKK			Oxidation (M)[3]		Mascot
3052.5264	3052.3071	-0.2193	-72	1	30	MAPAVMASSATTVAPFQGLKSTAGLPISCR			Carbamidomethyl (C)[29], Oxidation (M)[1,6]		Mascot

Rank	Protein Name	Accession No.	Protein MW	Protein PI	Pep. Count	Protein Score	Protein Score C. I. %	Total Ion Score	Total Ion C. I. %
2	50S ribosomal protein L33, chloroplastic OS=Fagopyrum esculentum subsp. ancestrale GN=rpl33 PE=3 SV	RK33_FAGE	7736.1	10.17	5	35	0		

Peptide Information

Calc. Mass	Obsrv. Mass	± da	± ppm	Start Seq.	End Seq.	Sequence	Ion Score	C. I. %	Modification	Rank	Result Type
1165.5168	1165.4768	-0.04	-34	50	57	KFCPYCYK			Carbamidomethyl (C)[3,6]		Mascot
1365.6864	1365.5007	-0.1857	-136	9	20	VSVILECTSCVR			Carbamidomethyl (C)[7]		Mascot
1493.7814	1493.5851	-0.1963	-131	9	21	VSVILECTSCVRK			Carbamidomethyl (C)[7]		Mascot
1707.8516	1707.5981	-0.2535	-148	6	20	DARVSVILECTSCVR			Carbamidomethyl (C)[10]		Mascot
1707.8516	1707.5981	-0.2535	-148	6	20	DARVSVILECTSCVR			Carbamidomethyl (C)[10]		Mascot
1838.8717	1838.7207	-0.151	-82	51	65	FCPYCYKHTIHGEIK					Mascot

Rank	Protein Name	Accession No.	Protein MW	Protein PI	Pep. Count	Protein Score	Protein Score C. I. %	Total Ion Score	Total Ion C. I. %
3	Ligatoxin-A OS=Phoradendron liga PE=1 SV=1	THNA_PHOL	4837.2	9.18	4	34	0		

Peptide Information

Calc. Mass	Obsrv. Mass	± da	± ppm	Start Seq.	End Seq.	Sequence	Ion Score	C. I. %	Modification	Rank	Result Type
1434.5955	1434.6293	0.0338	24	34	46	IISGSTCBSGWBH			Carbamidomethyl (C)[7]		Mascot
1434.5955	1434.6293	0.0338	24	34	46	IISGSTCBSGWBH			Carbamidomethyl (C)[7]		Mascot
1638.7938	1638.6782	-0.1156	-71	18	33	LTGTSRPTCASLSGCK			Carbamidomethyl (C)[9]		Mascot
1960.8422	1960.8153	-0.0269	-14	2	17	SCCPSTTARNIYNTCR			Carbamidomethyl (C)[2,3,15]		Mascot
3052.4033	3052.3071	-0.0962	-32	18	46	LTGTSRPTCASLSGCKIISGSTCBSGWBH			Carbamidomethyl (C)[9,15]		Mascot

Rank	Protein Name	Accession No.	Protein MW	Protein PI	Pep. Count	Protein Score	Protein Score C. I. %	Total Ion Score	Total Ion C. I. %
4	Ferredoxin--NADP reductase, leaf isozyme, chloroplastic OS=Pisum sativum GN=PETH PE=1 SV=1	FENR1_PEA	40168.6	8.56	9	34	0		

Peptide Information

Calc. Mass	Obsrv. Mass	± da	± ppm	Start Seq.	End Seq.	Sequence	Ion Score	C. I. %	Modification	Rank	Result Type
807.4359	807.3358	-0.1001	-124	275	281	LDFAVSR					Mascot
811.413	811.2996	-0.1134	-140	291	296	MYIOTR					Mascot
1265.6161	1265.5125	-0.1036	-82	351	360	KAEQWNVEVY					Mascot
1294.6426	1294.611	-0.0316	-24	338	347	DGIDWIEYKR					Mascot
1427.7377	1427.639	-0.0987	-69	118	131	EGQSIGIVPDGIDK					Mascot
1434.6755	1434.6293	-0.0462	-32	310	321	KDNTFVYMCGLK			Oxidation (M)[8]		Mascot
1434.6755	1434.6293	-0.0462	-32	310	321	KDNTFVYMCGLK			Oxidation (M)[8]		Mascot
1475.7021	1475.6058	-0.0963	-65	310	321	KDNTFVYMCGLK			Carbamidomethyl (C)[9]		Mascot
1520.7703	1520.5713	-0.199	-131	275	287	LDFAVSREOVNDK					Mascot
1520.7703	1520.5713	-0.199	-131	275	287	LDFAVSREOVNDK					Mascot
1521.7809	1521.5759	-0.205	-135	269	281	APENFRLDFAVSR					Mascot
2942.5945	2942.2732	-0.3213	-109	2	30	AAAVTAAVSLPYSNSTSLPIRTSIVAPER					Mascot

Rank	Protein Name	Accession No.	Protein MW	Protein PI	Pep. Count	Protein Score	Protein Score C. I. %	Total Ion Score	Total Ion C. I. %
5	Ferredoxin--NADP reductase, chloroplastic OS=V faba GN=PETH PE=2 SV=1	FENR_VICFA	40552.9	8.7	9	33	0		

Peptide Information

Calc. Mass	Obsrv. Mass	± da	± ppm	Start Seq.	End Seq.	Sequence	Ion Score	C. I. %	Modification	Rank	Result Type
807.4359	807.3358	-0.1001	-124	278	284	LDFAVSR					Mascot
811.413	811.2996	-0.1134	-140	294	299	MYIOTR					Mascot
1265.6161	1265.5125	-0.1036	-82	354	363	KAEQWNVEVY					Mascot
1294.6426	1294.611	-0.0316	-24	341	350	DGIDWIEYKR					Mascot
1427.7377	1427.639	-0.0987	-69	121	134	EGQSIGIVPDGIDK					Mascot
1434.6755	1434.6293	-0.0462	-32	313	324	KDNTFVYMCGLK			Oxidation (M)[8]		Mascot
1434.6755	1434.6293	-0.0462	-32	313	324	KDNTFVYMCGLK			Oxidation (M)[8]		Mascot
1475.7021	1475.6058	-0.0963	-65	313	324	KDNTFVYMCGLK			Carbamidomethyl (C)[9]		Mascot
1520.7703	1520.5713	-0.199	-131	278	290	LDFAVSREOVNDK					Mascot
1520.7703	1520.5713	-0.199	-131	278	290	LDFAVSREOVNDK					Mascot
1521.7809	1521.5759	-0.205	-135	272	284	APENFRLDFAVSR					Mascot
2942.5945	2942.2732	-0.3213	-109	2	30	AAAVTAAVSLPYSNSTSLPIRTSIVAPER					Mascot

Rank	Protein Name	Accession No.	Protein MW	Protein PI	Pep. Count	Protein Score	Protein Score C. I. %	Total Ion Score	Total Ion C. I. %
6	Caffeic acid 3-O-methyltransferase OS=Capsicum annuum GN=COMT PE=2 SV=2	COMT1_CAP.	39406.7	5.61	8	32	0		

Peptide Information

Calc. Mass	Obsrv. Mass	± da	± ppm	Start Seq.	End Seq.	Sequence	Ion Score	C. I. %	Modification	Rank	Result Type
807.4246	807.3358	-0.0888	-110	330	336	EFEALAK					Mascot
811.4018	811.2996	-0.1022	-126	255	261	ADAIFMK			Oxidation (M)[6]		Mascot
1165.6099	1165.4768	-0.1331	-114	327	336	TEKEFEALAK					Mascot
1179.5463	1179.4916	-0.0547	-46	278	288	NCYEALPANGK					Mascot
1179.5463	1179.4916	-0.0547	-46	278	288	NCYEALPANGK					Mascot
1434.7773	1434.6293	-0.148	-103	98	109	VERLYSLAPVCK			Carbamidomethyl (C)[11]		Mascot
1434.7773	1434.6293	-0.148	-103	98	109	VERLYSLAPVCK			Carbamidomethyl (C)[11]		Mascot
1716.7332	1716.6768	-0.0564	-33	346	359	ACCAYQTWVMEFHK					Mascot
1940.0455	1939.8628	-0.1827	-94	76	91	MLRLLATYSVLNCTLR			Carbamidomethyl (C)[13], Oxidation (M)[1]		Mascot
1940.0455	1939.8628	-0.1827	-94	76	91	MLRLLATYSVLNCTLR			Carbamidomethyl (C)[13], Oxidation (M)[1]		Mascot
2057.0708	2056.7979	-0.2729	-133	308	326	NAVHVDIVMLAHNPGGKER					Mascot

Rank	Protein Name	Accession No.	Protein MW	Protein PI	Pep. Count	Protein Score	Protein Score C. I. %	Total Ion Score	Total Ion C. I. %
7	Ribulose bisphosphate carboxylase small chain 512 (Fragment) OS=Triticum aestivum PE=3 SV=1	RBS3_WHEA	13046.5	5.84	5	32	0		

Peptide Information

Calc. Mass	Obsrv. Mass	± da	± ppm	Start Seq.	End Seq.	Sequence	Ion Score	C. I. %	Modification	Rank	Result Type
1165.571	1165.4768	-0.0942	-81	23	31	WVPCLEFSK			Carbamidomethyl (C)[4]		Mascot
1365.5818	1365.5007	-0.0811	-59	38	49	EHNASPGYYDGR					Mascot
2211.0571	2210.8606	-0.1965	-89	93	112	QVQCVSFIAFKPPGCEESGK			Carbamidomethyl (C)[4]		Mascot
2225.073	2224.894	-0.179	-80	93	113	QVQCVSFIAFKPPGCEESGKA					Mascot
2367.157	2367.0051	-0.1519	-64	56	76	LPMFGCTDATQVINEVEEVKK			Oxidation (M)[3]		Mascot
2367.157	2367.0051	-0.1519	-64	56	76	LPMFGCTDATQVINEVEEVKK			Oxidation (M)[3]		Mascot

Rank	Protein Name	Accession No.	Protein MW	Protein PI	Pep. Count	Protein Score	Protein Score C. I. %	Total Ion Score	Total Ion C. I. %
8	Beclin-1-like protein OS=Arabidopsis thaliana GN=At3g61710 PE=2 SV=2	BECN1_ARA	58467.1	5.64	9	32	0		

Peptide Information

Calc. Mass	Obsrv. Mass	± da	± ppm	Start Seq.	End Seq.	Sequence	Ion Score	C. I. %	Modification	Rank	Result Type
973.4948	973.4408	-0.054	-55	4	11	EEIPDKSR					Mascot
1193.5983	1193.5018	-0.0965	-81	69	78	MDNSFVVLPR			Oxidation (M)[1]		Mascot
1275.6146	1275.6045	-0.0101	-8	402	411	AMTLYLMCLK			Carbamidomethyl (C)[8], Oxidation (M)[2,7]		Mascot
1282.5732	1282.4852	-0.088	-69	193	203	DVEAYEACVQR					Mascot
1383.6288	1383.5466	-0.0822	-59	315	326	NDGEFGTINNFR					Mascot
1838.9041	1838.7207	-0.1834	-100	362	376	FQCQVKIQPMGSYPR			Carbamidomethyl (C)[3]		Mascot
1908.8612	1908.6248	-0.2364	-124	163	177	TQTQVEQPLCLECMR			Carbamidomethyl (C)[10,13], Oxidation (M)[14]		Mascot
2317.0852	2317.0049	-0.0803	-35	22	42	WVCQNCHHSLTIVGVDSYAGK					Mascot
3338.5542	3338.3774	-0.1768	-53	335	361	VEWDEINAAWGQACLLLHTMCNYFRPK			Carbamidomethyl (C)[14,21], Oxidation (M)[20]		Mascot

Rank	Protein Name	Accession No.	Protein MW	Protein PI	Pep. Count	Protein Score	Protein Score C. I. %	Total Ion Score	Total Ion C. I. %
9	Dynein 18 kDa light chain, flagellar outer arm OS=Chlamydomonas reinhardtii PE=1 SV=1	DYL3_CHLRE	17776.7	4.38	6	30	0		

Peptide Information

Calc. Mass	Obsrv. Mass	± da	± ppm	Start Seq.	End Seq.	Sequence	Ion Score	C. I. %	Modification	Rank	Result Type
856.5614	856.4445	-0.1169	-136	116	122	ILVDKLR					Mascot
973.4811	973.4408	-0.0403	-41	25	32	KAFAMFDK			Oxidation (M)[5]		Mascot
1227.619	1227.5566	-0.0624	-51	73	81	CIEFKEFVR			Carbamidomethyl (C)[1]		Mascot
1275.7089	1275.6045	-0.1044	-82	82	92	VIQINKQMSAK			Oxidation (M)[8]		Mascot
1475.6802	1475.6058	-0.0744	-50	144	155	DLNGFLSYDEFR					Mascot
1994.0262	1993.7657	-0.2605	-131	123	139	SICEEFELTVNVDRLVK					Mascot
1994.0262	1993.7657	-0.2605	-131	123	139	SICEEFELTVNVDRLVK					Mascot

Rank	Protein Name	Accession No.	Protein MW	Protein PI	Pep. Count	Protein Score	Protein Score C. I. %	Total Ion Score	Total Ion C. I. %
10	Putative protein ABIL2 OS=Oryza sativa subsp. japonica GN=Os01g0551800 PE=3 SV=1	ABIL2_ORYS	36099.5	9.69	7	30	0		

Peptide Information

Calc. Mass	Obsrv. Mass	± da	± ppm	Start Seq.	End Seq.	Sequence	Ion Score	C. I. %	Modification	Rank	Result Type
842.4301	842.4323	0.0022	3	112	118	VSCIHOR					Mascot
1165.6146	1165.4768	-0.1378	-118	1	10	MVEAVASFRR					Mascot
1390.5508	1390.5391	-0.0117	-8	313	323	EEPLPCYFDDY					Mascot
1707.8483	1707.5981	-0.2502	-147	173	189	AAQMQQVFSAAAASQAK					Mascot
1707.8483	1707.5981	-0.2502	-147	173	189	AAQMQQVFSAAAASQAK					Mascot
1796.9137	1796.8284	-0.0853	-47	213	229	SSSPAQRPPSENTIPTK					Mascot
1960.915	1960.8153	-0.0997	-51	112	128	VSCIHQRAQVSQACMDK			Carbamidomethyl (C)[3]		Mascot
1993.9872	1993.7657	-0.2215	-111	171	189	NRAAQMQQVFSAAAASQAK			Oxidation (M)[6]		Mascot
1993.9872	1993.7657	-0.2215	-111	171	189	NRAAQMQQVFSAAAASQAK			Oxidation (M)[6]		Mascot

Gel Idx/Pos	90/D15	Instr./Gel Origin	BA2060/131017A	Process Sta	Analysis Succeeded
Plate [#] Name	[1] 31496	Instrument Sample Name		Spectra	11

Rank	Protein Name	Accession No	Protein MW	Protein PI	Pep. Count	Protein Score	Protein Score C. I. %	Total Ion Score	Total Ion C. I. %
1	Ribulose bisphosphate carboxylase small chain ; chloroplastic OS=Solanum tuberosum GN=RBCS-2A PE=	RBSA SOLTU	20276	7.6	9	60	96.311		

Peptide Information

Calc. Mass	Obsrv. Mass	± da	± ppm	Start Seq.	End Seq.	Sequence	Ion Score	C. I. %	Modification	Rank	Result Type
914.4002	914.3223	-0.0779	-85	115	122	SPGYYDGR					Mascot
921.4788	921.4222	-0.0566	-61	31	39	SAASFPVSR					Mascot
933.5151	933.4591	-0.056	-60	158	165	IIGFDNVR					Mascot
933.5151	933.4591	-0.056	-60	158	165	IIGFDNVR					Mascot
1435.6531	1435.5992	-0.0539	-38	57	67	CMQVWPPINMK			Carbamidomethyl (C)[1], Oxidation (M)[2,10]		Mascot
1548.7838	1548.6451	-0.1387	-90	15	30	GNGAQASMVAPFTGLK					Mascot
2152.03	2151.8257	-0.2043	-95	129	148	LPMFGCTDATQVLAEVEEAK					Mascot
2183.0015	2182.8928	-0.1087	-50	93	110	NGWVPCLEFETEHGFVYR					Mascot
2225.0464	2224.8875	-0.1589	-71	129	148	LPMFGCTDATQVLAEVEEAK			Carbamidomethyl (C)[6], Oxidation (M)[3]		Mascot
2280.125	2279.9495	-0.1755	-77	129	149	LPMFGCTDATQVLAEVEEAKK					Mascot
2467.2397	2466.947	-0.2927	-119	15	39	GNGAQASMVAPFTGLKSAASFPVSR			Oxidation (M)[8]		Mascot

Rank	Protein Name	Accession No	Protein MW	Protein PI	Pep. Count	Protein Score	Protein Score C. I. %	Total Ion Score	Total Ion C. I. %
2	B3 domain-containing protein Os03g0120900 OS=Oryza sativa subsp. japonica GN=Os01g0723700 PE=2 SV=1	Y1237 ORYS	82608.3	5.81	16	58	94.154		

Peptide Information

Calc. Mass	Obsrv. Mass	± da	± ppm	Start Seq.	End Seq.	Sequence	Ion Score	C. I. %	Modification	Rank	Result Type
805.3798	805.4235	0.0437	54	359	364	ERSEER					Mascot
856.525	856.45	-0.075	-88	489	495	VLLDIQR					Mascot
933.4635	933.4591	-0.0044	-5	50	58	GTLQNSEGK					Mascot
933.4635	933.4591	-0.0044	-5	50	58	GTLQNSEGK					Mascot
1116.5724	1116.4835	-0.0889	-80	88	95	EYEFLLFR					Mascot
1444.7583	1444.6094	-0.1489	-103	67	79	LDGHVFLTTGWAK					Mascot
1560.6959	1560.5935	-0.1024	-66	365	378	QPSPDSMIDQAESR					Mascot
1605.882	1605.6864	-0.1956	-122	541	554	DAQRIVNALLEHAR					Mascot
1662.8634	1662.7095	-0.1539	-93	216	229	VTVELFCAMLVFYK					Mascot
1870.8496	1870.7411	-0.1085	-58	118	135	SSGSPQGKLPCDIFCCTK					Mascot
1870.8496	1870.7411	-0.1085	-58	118	135	SSGSPQGKLPCDIFCCTK					Mascot
1884.828	1884.7708	-0.0572	-30	184	200	DGLDEHLSLNGPMEDDK					Mascot
1891.8822	1891.8398	-0.0424	-22	453	469	GCIKSSPASCEVPACLR			Carbamidomethyl (C)[2,10,15]		Mascot
1962.9371	1962.7142	-0.2229	-114	295	310	LSNQPLQCDLTTEKCR			Carbamidomethyl (C)[8,15]		Mascot
1962.9371	1962.7142	-0.2229	-114	295	310	LSNQPLQCDLTTEKCR			Carbamidomethyl (C)[8,15]		Mascot
2061.9143	2061.6758	-0.2385	-116	361	378	SEERQPSPDSMIDQAESR					Mascot
2077.9092	2077.6694	-0.2398	-115	361	378	SEERQPSPDSMIDQAESR			Oxidation (M)[11]		Mascot
2225.1594	2224.8875	-0.2719	-122	618	636	LHNLQDEITGLQQSLEMKK					Mascot
2239.1289	2238.908	-0.2209	-99	642	660	LAHQVAEHESVFQKSLMER					Mascot
2333.0034	2332.9319	-0.0715	-31	96	114	YDDNMHFMVLPFGLNACEK			Carbamidomethyl (C)[17], Oxidation (M)[5,8]		Mascot

Rank	Protein Name	Accession No	Protein MW	Protein PI	Pep. Count	Protein Score	Protein Score C. I. %	Total Ion Score	Total Ion C. I. %
3	Ribulose bisphosphate carboxylase large chain (Fragment) OS=Cassia fistula GN=rbcL PE=3 SV=1	RBL CASFS	51606.1	6.14	10	58	94.154	16	0

Peptide Information

Calc. Mass	Obsrv. Mass	± da	± ppm	Start Seq.	End Seq.	Sequence	Ion Score	C. I. %	Modification	Rank	Result Type
805.4929	805.4235	-0.0694	-86	1	8	VGFKAGVK					Mascot
914.4049	914.3223	-0.0826	-90	296	302	NHGMHFR			Oxidation (M)[4]		Mascot
1116.583	1116.4835	-0.0995	-89	412	421	VALEACVQAR			Carbamidomethyl (C)[6]		Mascot
1194.5322	1194.4426	-0.0896	-75	208	217	FCFCAEALYK					Mascot
1275.7307	1275.6112	-0.1195	-94	330	340	EITLGFVDLLR	16	0			Mascot
1275.7307	1275.6112	-0.1195	-94	330	340	EITLGFVDLLR					Mascot
1465.6603	1465.6166	-0.0437	-30	206	217	DRFCFCAEALYK					Mascot
1893.8945	1893.8114	-0.0831	-44	227	243	GHYLNATAGTCEEMIKR					Mascot
1916.9574	1916.7963	-0.1611	-84	441	456	WSPELAAACEVWKEIK			Carbamidomethyl (C)[9]		Mascot
1916.9574	1916.7963	-0.1611	-84	441	456	WSPELAAACEVWKEIK			Carbamidomethyl (C)[9]		Mascot
1961.9425	1961.7245	-0.218	-111	437	453	EASKWSPELAAACEVWK			Carbamidomethyl (C)[13]		Mascot
2280.25	2279.9495	-0.3005	-132	130	149	IPISYIKTFQGPPHGIQVER					Mascot

Rank	Protein Name	Accession No	Protein MW	Protein PI	Pep. Count	Protein Score	Protein Score C. I. %	Total Ion Score	Total Ion C. I. %
4	Ribulose bisphosphate carboxylase large chain OS=Sesbania sesban GN=rbcL PE=3 SV=1	RBL SESSE	52358.4	6.09	10	57	93.44	16	0

Peptide Information

Calc. Mass	Obsrv. Mass	± da	± ppm	Start Seq.	End Seq.	Sequence	Ion Score	C. I. %	Modification	Rank	Result Type
856.3546	856.45	0.0954	111	467	473	FEFPAMD					Mascot
914.4049	914.3223	-0.0826	-90	306	312	NHGMHFR			Oxidation (M)[4]		Mascot
921.4346	921.4222	-0.0124	-13	1	8	MSPQTETK					Mascot
1039.4691	1039.4269	-0.0422	-41	351	358	DDYVEKDR					Mascot
1116.583	1116.4835	-0.0995	-89	422	431	VALEACVQAR			Carbamidomethyl (C)[6]		Mascot
1275.7307	1275.6112	-0.1195	-94	340	350	EITLGFVDLLR	16	0			Mascot
1275.7307	1275.6112	-0.1195	-94	340	350	EITLGFVDLLR					Mascot
1465.7546	1465.6166	-0.138	-94	147	159	TFQGPPHGIQVER					Mascot
1893.8945	1893.8114	-0.0831	-44	237	253	GHYLNATAGTCEEMIKR					Mascot
1916.9574	1916.7963	-0.1611	-84	451	466	WSPELAAACEVWKEIK			Carbamidomethyl (C)[9]		Mascot
1916.9574	1916.7963	-0.1611	-84	451	466	WSPELAAACEVWKEIK			Carbamidomethyl (C)[9]		Mascot
1961.9425	1961.7245	-0.218	-111	447	463	EASKWSPELAAACEVWK			Carbamidomethyl (C)[13]		Mascot

Rank	Protein Name	Accession No	Protein MW	Protein PI	Pep. Count	Protein Score	Protein Score C. I. %	Total Ion Score	Total Ion C. I. %
5	Ribulose bisphosphate carboxylase large chain (Fragment) OS=Sinapis alba GN=rbcL PE=3 SV=1	RBL_SINAL	52405.4	6.23	10	56	91.93	16	0

Peptide Information

Calc. Mass	Obsrv. Mass	± da	± ppm	Start Seq.	End Seq.	Sequence	Ion Score	C. I. %	Modification	Rank	Result Type
805.4818	805.4235	-0.0583	-72	140	146	IPTAYIK					Mascot
856.3546	856.45	0.0954	111	467	473	FEFPAMD					Mascot
914.4049	914.3223	-0.0826	-90	306	312	NHGMHFR			Oxidation (M)[4]		Mascot
921.4346	921.4222	-0.0124	-13	1	8	MSPQTETK					Mascot
1116.583	1116.4835	-0.0995	-89	422	431	VALEACVQAR			Carbamidomethyl (C)[6]		Mascot
1275.7307	1275.6112	-0.1195	-94	340	350	EITLGFVDLLR	16	0			Mascot
1275.7307	1275.6112	-0.1195	-94	340	350	EITLGFVDLLR					Mascot
1465.7546	1465.6166	-0.138	-94	147	159	TFQGPPHGIQVER					Mascot
1893.8945	1893.8114	-0.0831	-44	237	253	GHYLNATAGTCEEMIKR					Mascot
1916.9574	1916.7963	-0.1611	-84	451	466	WSPELAAACEVWKEIK			Carbamidomethyl (C)[9]		Mascot
1916.9574	1916.7963	-0.1611	-84	451	466	WSPELAAACEVWKEIK			Carbamidomethyl (C)[9]		Mascot
1961.9425	1961.7245	-0.218	-111	447	463	EASKWSPELAAACEVWK			Carbamidomethyl (C)[13]		Mascot

Rank	Protein Name	Accession No	Protein MW	Protein PI	Pep. Count	Protein Score	Protein Score C. I. %	Total Ion Score	Total Ion C. I. %
6	Ribulose bisphosphate carboxylase large chain (Fragment) OS=Manilkara zapota GN=rbcL PE=3 SV=2	RBL_MANZA	51550	6.04	9	51	70.698	16	0

Peptide Information

Calc. Mass	Obsrv. Mass	± da	± ppm	Start Seq.	End Seq.	Sequence	Ion Score	C. I. %	Modification	Rank	Result Type
805.4929	805.4235	-0.0694	-86	1	8	VGFKAGVK					Mascot
914.4049	914.3223	-0.0826	-90	296	302	NHGMHFR			Oxidation (M)[4]		Mascot
1039.4691	1039.4269	-0.0422	-41	341	348	DDYVEKDR					Mascot
1116.583	1116.4835	-0.0995	-89	412	421	VALEACVQAR			Carbamidomethyl (C)[6]		Mascot
1275.7307	1275.6112	-0.1195	-94	330	340	EITLGFVDLLR	16	0			Mascot
1275.7307	1275.6112	-0.1195	-94	330	340	EITLGFVDLLR					Mascot
1465.7546	1465.6166	-0.138	-94	137	149	TFQGPPHGIQVER					Mascot
1893.8945	1893.8114	-0.0831	-44	227	243	GHYLNATAGTCEEMIKR					Mascot
1916.9574	1916.7963	-0.1611	-84	441	456	WSPELAAACEVWKEIK			Carbamidomethyl (C)[9]		Mascot
1916.9574	1916.7963	-0.1611	-84	441	456	WSPELAAACEVWKEIK			Carbamidomethyl (C)[9]		Mascot
1961.9425	1961.7245	-0.218	-111	437	453	EASKWSPELAAACEVWK			Carbamidomethyl (C)[13]		Mascot

Rank	Protein Name	Accession No	Protein MW	Protein PI	Pep. Count	Protein Score	Protein Score C. I. %	Total Ion Score	Total Ion C. I. %
7	Ribulose bisphosphate carboxylase large chain (Fragment) OS=Morus rubra GN=rbcL PE=3 SV=1	RBL_MORRU	51574	6	9	50	69.317	16	0

Peptide Information

Calc. Mass	Obsrv. Mass	± da	± ppm	Start Seq.	End Seq.	Sequence	Ion Score	C. I. %	Modification	Rank	Result Type
805.4929	805.4235	-0.0694	-86	1	8	VGFKAGVK					Mascot
914.4049	914.3223	-0.0826	-90	296	302	NHGMHFR			Oxidation (M)[4]		Mascot
1116.6194	1116.4835	-0.1359	-122	412	421	VALEACVKAR			Carbamidomethyl (C)[6]		Mascot
1275.7307	1275.6112	-0.1195	-94	330	340	EITLGFVDLLR	16	0			Mascot
1275.7307	1275.6112	-0.1195	-94	330	340	EITLGFVDLLR					Mascot
1444.8158	1444.6094	-0.2064	-143	125	136	LEDLRIPNAYIK					Mascot
1465.7216	1465.6166	-0.105	-72	310	324	MSGGDHIHAGTVVGK					Mascot
1684.8612	1684.6335	-0.2277	-135	422	436	NEGRDLAVEGNEIIR					Mascot
1916.9574	1916.7963	-0.1611	-84	441	456	WSPELAAACEVWKEIK			Carbamidomethyl (C)[9]		Mascot
1916.9574	1916.7963	-0.1611	-84	441	456	WSPELAAACEVWKEIK			Carbamidomethyl (C)[9]		Mascot
1961.9425	1961.7245	-0.218	-111	437	453	EASKWSPELAAACEVWK			Carbamidomethyl (C)[13]		Mascot

Rank	Protein Name	Accession No	Protein MW	Protein PI	Pep. Count	Protein Score	Protein Score C. I. %	Total Ion Score	Total Ion C. I. %
8	Ribulose bisphosphate carboxylase large chain OS=Calycanthus floridus var. glaucus GN=rbcL PE=3 SV=	RBL_CALFG	52571.5	6.23	9	50	68.603	16	0

Peptide Information

Calc. Mass	Obsrv. Mass	± da	± ppm	Start Seq.	End Seq.	Sequence	Ion Score	C. I. %	Modification	Rank	Result Type
914.4049	914.3223	-0.0826	-90	306	312	NHGMHFR			Oxidation (M)[4]		Mascot
921.4709	921.4222	-0.0487	-53	1	8	MSPKTETK					Mascot
933.5515	933.4591	-0.0924	-99	9	18	AGVGFKAGVK					Mascot
933.5515	933.4591	-0.0924	-99	9	18	AGVGFKAGVK					Mascot
1116.583	1116.4835	-0.0995	-89	422	431	VALEACVQAR			Carbamidomethyl (C)[6]		Mascot
1275.7307	1275.6112	-0.1195	-94	340	350	EITLGFVDLLR	16	0			Mascot
1275.7307	1275.6112	-0.1195	-94	340	350	EITLGFVDLLR					Mascot
1465.7216	1465.6166	-0.105	-72	320	334	MSGGDHIHAGTVVGK					Mascot
1893.8945	1893.8114	-0.0831	-44	237	253	GHYLNATAGTCEEMIKR					Mascot
1916.9574	1916.7963	-0.1611	-84	451	466	WSPELAAACEVWKEIK			Carbamidomethyl (C)[9]		Mascot
1916.9574	1916.7963	-0.1611	-84	451	466	WSPELAAACEVWKEIK			Carbamidomethyl (C)[9]		Mascot
1961.9425	1961.7245	-0.218	-111	447	463	EASKWSPELAAACEVWK			Carbamidomethyl (C)[13]		Mascot

Rank	Protein Name	Accession No	Protein MW	Protein PI	Pep. Count	Protein Score	Protein Score C. I. %	Total Ion Score	Total Ion C. I. %
9	Ribulose bisphosphate carboxylase large chain OS=Morus indica GN=rbcL PE=3 SV=1	RBL_MORIN	52634.5	6	9	50	63.951	16	0

Peptide Information

Re Result Type

Calc. Mass	Obsrv. Mass	± da	± ppm	Start Seq.	End Seq.	Sequence	Ion Score	C. I. %	Modification	Rank	Result Type
914.4049	914.3223	-0.0826	-90	306	312	NHGMHFR			Oxidation (M)[4]		Mascot
921.4346	921.4222	-0.0124	-13	1	8	MSPQTETK					Mascot
1116.6194	1116.4835	-0.1359	-122	422	431	VALEACVKAR			Carbamidomethyl (C)[6]		Mascot
1275.7307	1275.6112	-0.1195	-94	340	350	EITLGFVDLLR	16	0			Mascot
1275.7307	1275.6112	-0.1195	-94	340	350	EITLGFVDLLR					Mascot
1444.8158	1444.6094	-0.2064	-143	135	146	LEDLRIPNAYIK					Mascot
1465.7216	1465.6166	-0.105	-72	320	334	MSGGDHIHAGTVVGK					Mascot
1684.8612	1684.6335	-0.2277	-135	432	446	NEGRDLAVEGNEIIR					Mascot
1916.9574	1916.7963	-0.1611	-84	451	466	WSPELAAACEVWKEIK			Carbamidomethyl (C)[9]		Mascot
1916.9574	1916.7963	-0.1611	-84	451	466	WSPELAAACEVWKEIK			Carbamidomethyl (C)[9]		Mascot
1961.9425	1961.7245	-0.218	-111	447	463	EASKWSPELAAACEVWK			Carbamidomethyl (C)[13]		Mascot

Rank	Protein Name	Accession No	Protein MW	Protein PI	Pep. Count	Protein Score	Protein Score C. I. %	Total Ion Score	Total Ion C. I. %
10	Ribulose bisphosphate carboxylase large chain OS=Phaseolus vulgaris GN=rbcL PE=3 SV=1	RBL_PHAVU	52767.6	5.96	9	49	56.659	16	0

Peptide Information

Calc. Mass	Obsrv. Mass	± da	± ppm	Start Seq.	End Seq.	Sequence	Ion Score	C. I. %	Modification	Rank	Result Type
805.4818	805.4235	-0.0583	-72	140	146	IPTAYIK					Mascot
914.4049	914.3223	-0.0826	-90	306	312	NHGMHFR			Oxidation (M)[4]		Mascot
921.4346	921.4222	-0.0124	-13	1	8	MSPQTETK					Mascot
1116.583	1116.4835	-0.0995	-89	422	431	VALEACVQAR			Carbamidomethyl (C)[6]		Mascot
1275.7307	1275.6112	-0.1195	-94	340	350	EITLGFVDLLR	16	0			Mascot
1275.7307	1275.6112	-0.1195	-94	340	350	EITLGFVDLLR					Mascot
1465.7546	1465.6166	-0.138	-94	147	159	TFQGPPHGIQVER					Mascot
1893.8945	1893.8114	-0.0831	-44	237	253	GHYLNATAGTCEEMIKR					Mascot
1916.9574	1916.7963	-0.1611	-84	451	466	WSPELAAACEVWKEIK			Carbamidomethyl (C)[9]		Mascot
1916.9574	1916.7963	-0.1611	-84	451	466	WSPELAAACEVWKEIK			Carbamidomethyl (C)[9]		Mascot
1961.9425	1961.7245	-0.218	-111	447	463	EASKWSPELAAACEVWK			Carbamidomethyl (C)[13]		Mascot

Gel Idx/Pos	91/D16	Instr./Gel Origin	BA2060/131017A	Process Sta	Analysis Succeeded
Plate [#] Name	[1] 31496	Instrument Sample Name		Spectra	8

Rank	Protein Name	Accession No	Protein MW	Protein PI	Pep. Count	Protein Score	Protein Score C. I. %	Total Ion Score	Total Ion C. I. %
1	Superoxide dismutase [Fe], chloroplastic OS=Gly max GN=SODB PE=2 SV=1	SODF_SOYB	27824.1	5.6	8	372	100	322	100

Peptide Information

Calc. Mass	Obsrv. Mass	± da	± ppm	Start Seq.	End Seq.	Sequence	Ion Score	C. I. %	Modification	Rank	Result Type
842.473	842.4437	-0.0293	-35	19	26	EGPKVNAK					Mascot
994.5567	994.4355	-0.1212	-122	58	65	TYVENLKK					Mascot
1045.5524	1045.4758	-0.0766	-73	66	75	QVVGTELDGK					Mascot
1119.5793	1119.4862	-0.0931	-83	228	237	LVSWDAVSSR	64	99.996			Mascot
1119.5793	1119.4862	-0.0931	-83	228	237	LVSWDAVSSR					Mascot
1370.6774	1370.5592	-0.1182	-86	217	227	RPDYISVFMDK					Mascot
1370.6774	1370.5592	-0.1182	-86	217	227	RPDYISVFMDK	65	99.997			Mascot
1386.6722	1386.5518	-0.1204	-87	217	227	RPDYISVFMDK			Oxidation (M)[9]		Mascot
1395.7366	1395.6182	-0.1184	-85	76	87	SLEEIIVTSYNK					Mascot
1395.7366	1395.6182	-0.1184	-85	76	87	SLEEIIVTSYNK	51	99.914			Mascot
1826.8972	1826.7338	-0.1634	-89	142	158	AAATQFGSGWAWLAYR					Mascot
1826.8972	1826.7338	-0.1634	-89	142	158	AAATQFGSGWAWLAYR	143	100			Mascot
3282.6655	3282.355	-0.3105	-95	27	54	FELKPPPYPLNGLEPVMSQQTLEFHWGK					Mascot
3298.6604	3298.3508	-0.3096	-94	27	54	FELKPPPYPLNGLEPVMSQQTLEFHWGK			Oxidation (M)[17]		Mascot

Rank	Protein Name	Accession No	Protein MW	Protein PI	Pep. Count	Protein Score	Protein Score C. I. %	Total Ion Score	Total Ion C. I. %
2	AP2/ERF and B3 domain-containing transcription repressor TEM1 OS=Arabidopsis thaliana GN=TEM1 PE=1	RAVL1 ARAT	40532.5	9.3	8	39	0		

Peptide Information

Calc. Mass	Obsrv. Mass	± da	± ppm	Start Seq.	End Seq.	Sequence	Ion Score	C. I. %	Modification	Rank	Result Type
994.4992	994.4355	-0.0637	-64	83	90	WGAQIYEK					Mascot
1435.7111	1435.5347	-0.1764	-123	279	290	NLRAGDVVCFER			Carbamidomethyl (C)[9]		Mascot
1566.7758	1566.6237	-0.1521	-97	175	189	SGLETATYGNDAVLR					Mascot
1665.7649	1665.6516	-0.1133	-68	282	296	AGDVVCFERSTGPDR			Carbamidomethyl (C)[6]		Mascot
2225.0349	2224.9197	-0.1152	-52	45	66	MGSGGSSVVLDSENGVETESRK					Mascot
2807.3384	2807.0664	-0.272	-97	91	114	HQRVWLGTFNEEEEAASYDIAVR					Mascot
3299.4666	3299.3679	-0.0987	-30	1	30	MEYSCVDDSSTTSESLSISTTPKPTTTTEK			Carbamidomethyl (C)[5], Oxidation (M)[1]		Mascot
3354.5452	3354.4175	-0.1277	-38	1	31	MEYSCVDDSSTTSESLSISTTPKPTTTTEKK					Mascot

Rank	Protein Name	Accession No	Protein MW	Protein PI	Pep. Count	Protein Score	Protein Score C. I. %	Total Ion Score	Total Ion C. I. %
3	Uncharacterized protein PAM68-like OS=Arabidop thaliana GN=At5g52780 PE=2 SV=1	Y5278 ARAT	18736.8	9.24	6	39	0		

Peptide Information

Calc. Mass	Obsrv. Mass	± da	± ppm	Start Seq.	End Seq.	Sequence	Ion Score	C. I. %	Modification	Rank	Result Type
856.4457	856.4542	0.0085	10	3	9	ALLCSHR			Carbamidomethyl (C)[4]		Mascot
955.5254	955.3832	-0.1422	-149	2	9	RALLCSHR					Mascot
1152.616	1152.446	-0.17	-147	39	48	WESKLHAGPK					Mascot
1632.8492	1632.7312	-0.118	-72	30	42	TLYPNNKPRWESK					Mascot
1665.882	1665.6516	-0.2304	-138	25	38	SHNPKTLYPNNKPR					Mascot
3354.5366	3354.4175	-0.1191	-36	55	83	TSEKPGRPDPDPEDDPPIPQEVFERMMGR			Oxidation (M)[26,27]		Mascot

Rank	Protein Name	Accession No	Protein MW	Protein PI	Pep. Count	Protein Score	Protein Score C. I. %	Total Ion Score	Total Ion C. I. %
4	Cytochrome P450 71B19 OS=Arabidopsis thalian GN=CYP71B19 PE=2 SV=1	C71BJ ARAT	57482.3	7.28	7	38	0	21	17.222

Peptide Information

Calc. Mass	Obsrv. Mass	± da	± ppm	Start Seq.	End Seq.	Sequence	Ion Score	C. I. %	Modification	Rank	Result Type
829.4236	829.3071	-0.1165	-140	377	383	ETMAHIK					Mascot
914.44	914.3317	-0.1083	-118	469	476	LPDGMTHK			Oxidation (M)[5]		Mascot
1632.869	1632.7312	-0.1378	-84	477	491	DIDTEEAGTLTIVKK					Mascot
1816.854	1816.6019	-0.2521	-139	109	123	DFKDIGFTPYGNEWK					Mascot
1826.9469	1826.7338	-0.2131	-117	377	392	ETMAHIKVQGYDIPPK					Mascot
1827.0023	1826.7338	-0.2685	-147	37	52	FPVIGNLHQIGELPHR	23	43.813			Mascot
1842.9418	1842.7288	-0.213	-116	377	392	ETMAHIKVQGYDIPPK			Oxidation (M)[3]		Mascot
2024.9957	2024.864	-0.1317	-65	264	280	SKEHEDIIDSMLDVIHK			Oxidation (M)[11]		Mascot

Rank	Protein Name	Accession No	Protein MW	Protein PI	Pep. Count	Protein Score	Protein Score C. I. %	Total Ion Score	Total Ion C. I. %
5	(3S,6E)-nerolidol synthase 2, chloroplastic/mitochondrial OS=Fragaria ananassa PE=1 SV=1	NES2_FRAAN	66160.6	6.27	11	35	0		

Peptide Information

Calc. Mass	Obsrv. Mass	± da	± ppm	Start Seq.	End Seq.	Sequence	Ion Score	C. I. %	Modification	Rank	Result Type
882.4501	882.4888	0.0387	44	233	240	SLASFMAR					Mascot
996.5359	996.4278	-0.1081	-108	118	125	EIDEILHK					Mascot
1045.5902	1045.4758	-0.1144	-109	35	42	QLPTFQRR					Mascot
1395.759	1395.6182	-0.1408	-101	269	280	SLHQNEIVQISK					Mascot
1395.759	1395.6182	-0.1408	-101	269	280	SLHQNEIVQISK					Mascot
1444.6447	1444.6149	-0.0298	-21	372	383	ALYDMTNEISCK			Carbamidomethyl (C)[11]		Mascot
1708.8727	1708.7675	-0.1052	-62	398	412	ISWASLCNAFLVEAK			Carbamidomethyl (C)[7]		Mascot
1816.8582	1816.6019	-0.2563	-141	66	80	HTRTMGDIFVQHCQK			Oxidation (M)[5]		Mascot
1824.7527	1824.7509	-0.0018	-1	496	510	CYLEEHEGCSIEEAR			Carbamidomethyl (C)[1]		Mascot
1951.8962	1951.9368	0.0406	21	368	383	ICFKALYDMTNEISCK			Carbamidomethyl (C)[2], Oxidation (M)[9]		Mascot
2024.8688	2024.864	-0.0048	-2	496	512	CYLEEHEGCSIEEAREK					Mascot
2239.0925	2238.9348	-0.1577	-70	354	371	WEITAIDHLPDYMKICFK			Oxidation (M)[13]		Mascot

Rank	Protein Name	Accession No	Protein MW	Protein PI	Pep. Count	Protein Score	Protein Score C. I. %	Total Ion Score	Total Ion C. I. %
6	Putative cysteine-rich repeat secretory protein 13 OS=Arabidopsis thaliana GN=CRRSP13 PE=3 SV=1	CRR13_ARAT	29416.6	9.27	7	34	0		

Peptide Information

Calc. Mass	Obsrv. Mass	± da	± ppm	Start Seq.	End Seq.	Sequence	Ion Score	C. I. %	Modification	Rank	Result Type
856.4999	856.4542	-0.0457	-53	49	55	NLNQVIR					Mascot
1123.5378	1123.4829	-0.0549	-49	153	162	NVSSNAEQFK					Mascot
1830.8486	1830.7369	-0.1117	-61	99	113	CHSCLHTAFSGLRER			Carbamidomethyl (C)[1,4]		Mascot
1952.0377	1951.9368	-0.1009	-52	1	17	MSSNILAMVAMQLLLIR			Oxidation (M)[1,8,11]		Mascot
2182.9756	2182.8596	-0.116	-53	92	111	GDSYGSKCHSCLHTAFSGLR			Carbamidomethyl (C)[8]		Mascot
3284.5933	3284.3574	-0.2359	-72	64	91	YGYTYNSNVEAYEVSKDPNIVFVLLQCR					Mascot
3354.5271	3354.4175	-0.1096	-33	197	224	NKMYAMMQCALDLTTNGCYVCLEWIIGR			Carbamidomethyl (C)[9,18]		Mascot

Rank	Protein Name	Accession No	Protein MW	Protein PI	Pep. Count	Protein Score	Protein Score C. I. %	Total Ion Score	Total Ion C. I. %
7	(3S,6E)-nerolidol synthase 1, chloroplastic OS=F vesca PE=1 SV=1	NES1 FRAVE	66489.8	6.31	10	33	0		

Peptide Information

Calc. Mass	Obsrv. Mass	± da	± ppm	Start Seq.	End Seq.	Sequence	Ion Score	C. I. %	Modification	Rank	Result Type
882.4501	882.4888	0.0387	44	235	242	SLASFMAR					Mascot
996.5359	996.4278	-0.1081	-108	120	127	EIDEILHK					Mascot
1045.5902	1045.4758	-0.1144	-109	37	44	QLPTFQRR					Mascot
1395.7954	1395.6182	-0.1772	-127	271	282	SVHQKEIVQISK					Mascot
1395.7954	1395.6182	-0.1772	-127	271	282	SVHQKEIVQISK					Mascot
1708.8727	1708.7675	-0.1052	-62	400	414	ISWASLCNAFLVEAK			Carbamidomethyl (C)[7]		Mascot
1824.7527	1824.7509	-0.0018	-1	498	512	CYLEEHEGCSIEEAR			Carbamidomethyl (C)[1]		Mascot
2024.8688	2024.864	-0.0048	-2	498	514	CYLEEHEGCSIEEAREK					Mascot
2057.0083	2056.8374	-0.1709	-83	550	567	MIPLMYSYDGNQSLPSLK					Mascot
2230.2112	2230.0042	-0.207	-93	455	475	KSVELLNETPAMISSSAAILR					Mascot
2239.0925	2238.9348	-0.1577	-70	356	373	WEITAIDHLPDYMKICFK			Oxidation (M)[13]		Mascot

Rank	Protein Name	Accession No	Protein MW	Protein PI	Pep. Count	Protein Score	Protein Score C. I. %	Total Ion Score	Total Ion C. I. %
8	30S ribosomal protein S4, chloroplastic OS=Taka lepidozioides GN=rps4 PE=3 SV=1	RR4_TAKLE	23380.7	10.7	1	33	0	29	86.707

Peptide Information

Calc. Mass	Obsrv. Mass	± da	± ppm	Start Seq.	End Seq.	Sequence	Ion Score	C. I. %	Modification	Rank	Result Type
1255.7004	1255.5842	-0.1162	-93	132	142	EPGDIITIRNK	29	86.707			Mascot
1255.7004	1255.5842	-0.1162	-93	132	142	EPGDIITIRNK					Mascot

Rank	Protein Name	Accession No	Protein MW	Protein PI	Pep. Count	Protein Score	Protein Score C. I. %	Total Ion Score	Total Ion C. I. %
9	Formate dehydrogenase, mitochondrial OS=Solar tuberosum GN=FDH1 PE=1 SV=2	FDH_SOLTU	42011.6	6.64	8	33	0		

Peptide Information

Calc. Mass	Obsrv. Mass	± da	± ppm	Start Seq.	End Seq.	Sequence	Ion Score	C. I. %	Modification	Rank	Result Type
842.4076	842.4437	0.0361	43	264	270	TKGMFDK			Oxidation (M)[4]		Mascot
882.4138	882.4888	0.075	85	266	272	GMFDKER					Mascot
1267.6245	1267.5817	-0.0428	-34	1	12	MAMSRVASTAAR			Oxidation (M)[1]		Mascot
1370.6508	1370.5592	-0.0916	-67	240	250	FEEDLDKMLSK			Oxidation (M)[8]		Mascot
1370.6508	1370.5592	-0.0916	-67	240	250	FEEDLDKMLSK			Oxidation (M)[8]		Mascot
1444.7716	1444.6149	-0.1567	-108	251	263	CDIVVINTPLTEK					Mascot
1632.8315	1632.7312	-0.1003	-61	213	225	LKPFNCNLLYHDR					Mascot
2211.0122	2210.905	-0.1072	-48	228	246	MDSELENQIGAKFEEDLDK					Mascot
2313.2649	2312.9788	-0.2861	-124	208	225	LLLQRLKPFNCNLLYHDR			Carbamidomethyl (C)[11]		Mascot

Rank	Protein Name	Accession No	Protein MW	Protein PI	Pep. Count	Protein Score	Protein Score C. I. %	Total Ion Score	Total Ion C. I. %
10	Cytochrome P450 71B20 OS=Arabidopsis thalian GN=CYP71B20 PE=2 SV=1	C71BK ARAT	57379.1	7.66	6	32	0	21	17.222

Peptide Information

Calc. Mass	Obsrv. Mass	± da	± ppm	Start Seq.	End Seq.	Sequence	Ion Score	C. I. %	Modification	Rank	Result Type
829.4236	829.3071	-0.1165	-140	377	383	ETMAHIK					Mascot
914.44	914.3317	-0.1083	-118	469	476	LPDGMTHK			Oxidation (M)[5]		Mascot
1119.4775	1119.4862	0.0087	8	420	428	FMDSSVDYR					Mascot
1119.4775	1119.4862	0.0087	8	420	428	FMDSSVDYR					Mascot
1632.869	1632.7312	-0.1378	-84	477	491	DIDTEEAGTLTIVKK					Mascot
1826.9469	1826.7338	-0.2131	-117	377	392	ETMAHIKVQGYDIPPK					Mascot
1827.0023	1826.7338	-0.2685	-147	37	52	FPVIGNLHQIGELPHR	23	43.813			Mascot
1842.9418	1842.7288	-0.213	-116	377	392	ETMAHIKVQGYDIPPK			Oxidation (M)[3]		Mascot

Gel Idx/Pos	92/D17	Instr./Gel Origin	BA2060/131017A	Process Sta	Analysis Succeeded
Plate [#] Name	[1] 31496	Instrument Sample Name		Spectra	8

Rank	Protein Name	Accession No.	Protein MW	Protein PI	Pep. Count	Protein Score	Protein Score C. I. %	Total Ion Score	Total Ion C. I. %
1	Superoxide dismutase [Fe], chloroplastic OS=Gly max GN=SODB PE=2 SV=1	SODB_SOYBN	27824.1	5.6	9	341	100	282	100

Peptide Information

Calc. Mass	Obsrv. Mass	± da	± ppm	Start Seq.	End Seq.	Sequence	Ion Score	C. I. %	Modification	Rank	Result Type
842.473	842.452	-0.021	-25	19	26	EGPKVNAK					Mascot
994.5567	994.4309	-0.1258	-126	58	65	TYVENLKK					Mascot
1045.5524	1045.4868	-0.0656	-63	66	75	QVVGTELDGK					Mascot
1119.5793	1119.4978	-0.0815	-73	228	237	LVSWDAVSSR	55	99.965			Mascot
1119.5793	1119.4978	-0.0815	-73	228	237	LVSWDAVSSR					Mascot
1370.6774	1370.5787	-0.0987	-72	217	227	RPDYISVFMDK	50	99.897			Mascot
1370.6774	1370.5787	-0.0987	-72	217	227	RPDYISVFMDK					Mascot
1386.6722	1386.567	-0.1052	-76	217	227	RPDYISVFMDK			Oxidation (M)[9]		Mascot
1395.7366	1395.6331	-0.1035	-74	76	87	SLEEIIVTSYNK					Mascot
1395.7366	1395.6331	-0.1035	-74	76	87	SLEEIIVTSYNK	52	99.936			Mascot
1826.8972	1826.7542	-0.143	-78	142	158	AAAATQFGSGWAWLAYR					Mascot
1826.8972	1826.7542	-0.143	-78	142	158	AAAATQFGSGWAWLAYR	125	100			Mascot
1972.8883	1972.7394	-0.1489	-75	161	178	KFDGENVANPPSPDEDNK					Mascot
3282.6655	3282.3589	-0.3066	-93	27	54	FELKPPPYPLNGLEPVMSQQTLEFHWGK					Mascot
3298.6604	3298.3794	-0.281	-85	27	54	FELKPPPYPLNGLEPVMSQQTLEFHWGK			Oxidation (M)[17]		Mascot

Rank	Protein Name	Accession No.	Protein MW	Protein PI	Pep. Count	Protein Score	Protein Score C. I. %	Total Ion Score	Total Ion C. I. %
2	(3S,6E)-nerolidol synthase 1, chloroplastic OS=Fr vesca PE=1 SV=1	NES1_FRAVE	66489.8	6.31	12	48	41.535		

Peptide Information

Calc. Mass	Obsrv. Mass	± da	± ppm	Start Seq.	End Seq.	Sequence	Ion Score	C. I. %	Modification	Rank	Result Type
882.4501	882.5007	0.0506	57	235	242	SLASFMAR					Mascot
996.5359	996.4201	-0.1158	-116	120	127	EIDEILHK					Mascot
1045.5902	1045.4868	-0.1034	-99	37	44	QLPTFQRR					Mascot
1236.5929	1236.5681	-0.0248	-20	515	524	TINMISDEWK					Mascot
1395.7954	1395.6331	-0.1623	-116	271	282	SVHQKEIVQISK					Mascot
1395.7954	1395.6331	-0.1623	-116	271	282	SVHQKEIVQISK					Mascot
1555.725	1555.8099	0.0849	55	303	315	WYTWSMAGLTDPK					Mascot
1708.8727	1708.7872	-0.0855	-50	400	414	ISWASLCNAFLVEAK			Carbamidomethyl (C)[7]		Mascot
1810.9116	1810.7739	-0.1377	-76	20	35	SISHIGQSDLMQLTHK			Oxidation (M)[11]		Mascot
1824.7527	1824.74	-0.0127	-7	498	512	CYLEEHEGCSIEEAR			Carbamidomethyl (C)[1]		Mascot
2057.0083	2056.8647	-0.1436	-70	550	567	MIPLMYSYDGNQSLPSLK					Mascot
2230.2112	2230.0383	-0.1729	-78	455	475	KSVELLNETPAMISSSAAILR					Mascot
2525.314	2525.0854	-0.2286	-91	88	110	NVLRNAAELDALEGLNMIDAVQR					Mascot
2525.314	2525.0854	-0.2286	-91	88	110	NVLRNAAELDALEGLNMIDAVQR					Mascot

Rank	Protein Name	Accession No.	Protein MW	Protein PI	Pep. Count	Protein Score	Protein Score C. I. %	Total Ion Score	Total Ion C. I. %
3	B3 domain-containing protein Os03g0120900 OS=Oryza sativa subsp. japonica GN=Os01g0723700 PE=2 SV=1	Y1237_ORYS	82608.3	5.81	15	45	0		

Peptide Information

Calc. Mass	Obsrv. Mass	± da	± ppm	Start Seq.	End Seq.	Sequence	Ion Score	C. I. %	Modification	Rank	Result Type
856.525	856.4602	-0.0648	-76	489	495	VLLDIQR					Mascot
862.3974	862.4198	0.0224	26	288	294	CSLEEPK			Carbamidomethyl (C)[1]		Mascot
1123.6582	1123.4954	-0.1628	-145	501	510	TISHVQGIIR					Mascot
1135.6582	1135.4905	-0.1677	-148	545	554	IVNALLEHAR					Mascot
1151.5084	1151.4885	-0.0199	-17	533	540	WNCCLKDR			Carbamidomethyl (C)[3,4]		Mascot
1236.579	1236.5681	-0.0109	-9	329	339	GSFCSPETPRR					Mascot
1433.6511	1433.5521	-0.099	-69	457	469	SSPASCEVPACLR			Carbamidomethyl (C)[6,11]		Mascot
1605.882	1605.713	-0.169	-105	541	554	DAQRIVNALLEHAR					Mascot
1662.8634	1662.7417	-0.1217	-73	216	229	VTVELFCAMLVFYK					Mascot
1772.9429	1772.8534	-0.0895	-50	705	719	EIELSKESLLNITFH					Mascot
1772.9429	1772.8534	-0.0895	-50	705	719	EIELSKESLLNITFH					Mascot
1830.9371	1830.7584	-0.1787	-98	585	599	EVETIYTSLELDYKK					Mascot
1834.8608	1834.7603	-0.1005	-55	453	469	GCIKSSPASCEVPACLR			Carbamidomethyl (C)[2,10]		Mascot
1891.8822	1891.8881	0.0059	3	453	469	GCIKSSPASCEVPACLR			Carbamidomethyl (C)[2,10,15]		Mascot
2225.1594	2224.9504	-0.209	-94	618	636	LHNLQDEITGLQQSLEMKK					Mascot
2525.1726	2525.0854	-0.0872	-35	161	183	TTQGHELISPQSFPDQHEVCGSK					Mascot
2525.1726	2525.0854	-0.0872	-35	161	183	TTQGHELISPQSFPDQHEVCGSK					Mascot
3353.6042	3353.467	-0.1372	-41	216	243	VTVELFCAMLVFYKWNVDAVAEDFDICR			Carbamidomethyl (C)[7]		Mascot
	3353.467										Mascot

Rank	Protein Name	Accession No.	Protein MW	Protein PI	Pep. Count	Protein Score	Protein Score C. I. %	Total Ion Score	Total Ion C. I. %
4	Non-specific lipid-transfer protein OS=Eleusine coracana PE=1 SV=1	NLTP_ELECO	9325.6	9.88	5	40	0		

Peptide Information

Calc. Mass	Obsrv. Mass	± da	± ppm	Start Seq.	End Seq.	Sequence	Ion Score	C. I. %	Modification	Rank	Result Type
1395.6467	1395.6331	-0.0136	-10	48	60	AACNCSLKSAASR			Carbamidomethyl (C)[3,5]		Mascot
1395.6467	1395.6331	-0.0136	-10	48	60	AACNCSLKSAASR			Carbamidomethyl (C)[3,5]		Mascot
1413.7808	1413.5898	-0.191	-135	61	75	VSGLNAGKASSIPGR					Mascot
1810.8938	1810.7739	-0.1199	-66	76	92	CGVRLPYAISASIDCSR					Mascot
2001.9883	2001.8589	-0.1294	-65	20	41	GAGAAPSASCQSGVRSLNAAAR					Mascot
3337.5833	3337.5112	-0.0721	-22	1	34	AISCGQVSSAIGPCLAYARGAGAAPSASCQSGVR			Carbamidomethyl (C)[4,14,29]		Mascot

Rank	Protein Name	Accession No.	Protein MW	Protein PI	Pep. Count	Protein Score	Protein Score C. I. %	Total Ion Score	Total Ion C. I. %
5	DNA-directed RNA polymerase subunit beta'' OS=Buxus microphylla GN=rpoC2 PE=3 SV=1	RPOC2_BUXM	158595.6	9.36	19	38	0		

Peptide Information

Calc. Mass	Obsrv. Mass	± da	± ppm	Start Seq.	End Seq.	Sequence	Ion Score	C. I. %	Modification	Rank	Result Type
800.397	800.3463	-0.0507	-63	658	663	SKYQMK			Oxidation (M)[5]		Mascot
842.5458	842.452	-0.0938	-111	1270	1277	AVLLGITR					Mascot
870.3444	870.4747	0.1303	150	236	242	NGMMTER			Oxidation (M)[3,4]		Mascot
955.4665	955.3976	-0.0689	-72	660	666	YQMKVDR			Oxidation (M)[3]		Mascot
994.4695	994.4309	-0.0386	-39	1	8	MEVLMAER			Oxidation (M)[1]		Mascot
995.4614	995.3962	-0.0652	-65	1350	1357	NIFEGEMR					Mascot
1045.5173	1045.4868	-0.0305	-29	1169	1176	RVEGWNER					Mascot
1123.5564	1123.4954	-0.061	-54	1349	1357	KNIFEGEMR					Mascot
1236.614	1236.5681	-0.0459	-37	1158	1168	SIDSISMNLEK					Mascot
1238.6965	1238.5354	-0.1611	-130	1366	1375	FKLLNYCIPK					Mascot
1255.6542	1255.5975	-0.0567	-45	1170	1179	VEGWNERIPR					Mascot
1255.6542	1255.5975	-0.0567	-45	1170	1179	VEGWNERIPR					Mascot
1413.65	1413.5898	-0.0602	-43	838	848	TCLVLNCDQYK			Carbamidomethyl (C)[2,7]		Mascot
1546.8159	1546.6584	-0.1575	-102	284	296	AQPISIRTPFTCR			Carbamidomethyl (C)[12]		Mascot
1639.7778	1639.7422	-0.0356	-22	939	953	ECQSLIILSSSNCSR					Mascot
1712.8649	1712.8016	-0.0633	-37	475	489	TSHLWVLSGGPCRSR			Carbamidomethyl (C)[12]		Mascot
1842.7566	1842.7483	-0.0083	-5	1376	1391	NFHDTPEQSFTGFNDS					Mascot
1862.8524	1862.7257	-0.1267	-68	125	141	MTDPSNPVHIMSFSGAR			Oxidation (M)[1]		Mascot
2028.0549	2027.8617	-0.1932	-95	101	116	LRQSIEIWYATSEYLR					Mascot
3282.5564	3282.3589	-0.1975	-60	125	154	MTDPSNPVHIMSFSGARGNPSQVHQLVGMR			Oxidation (M)[1,11]		Mascot
3298.5513	3298.3794	-0.1719	-52	125	154	MTDPSNPVHIMSFSGARGNPSQVHQLVGMR			Oxidation (M)[1,11,29]		Mascot

Rank	Protein Name	Accession No.	Protein MW	Protein PI	Pep. Count	Protein Score	Protein Score C. I. %	Total Ion Score	Total Ion C. I. %
6	Tubulin alpha-2 chain (Fragment) OS=Anemia ph GN=TUBA2 PE=2 SV=1	TBA2_ANEPH	40151	5.06	8	36	0		

Peptide Information

Calc. Mass	Obsrv. Mass	± da	± ppm	Start Seq.	End Seq.	Sequence	Ion Score	C. I. %	Modification	Rank	Result Type
1267.509	1267.6022	0.0932	74	228	236	YMACCLMYR			Carbamidomethyl (C)[4,5]		Mascot
1605.6793	1605.713	0.0337	21	225	236	HGKYMACCLMYR			Carbamidomethyl (C)[7,8], Oxidation (M)[5]		Mascot
1862.842	1862.7257	-0.1163	-62	228	242	YMACCLMYRGDVVPK			Carbamidomethyl (C)[4,5]		Mascot
1874.9429	1874.7307	-0.2122	-113	21	36	GHYTVGKEIVDLCLDR			Carbamidomethyl (C)[13]		Mascot
2001.772	2001.8589	0.0869	43	347	364	DYEEVAAEGVDEPEGDDY					Mascot
2211.1008	2210.9246	-0.1762	-80	287	306	VQRAVCMISNNTAVAEVFSR			Oxidation (M)[7]		Mascot
3337.7207	3337.5112	-0.2095	-63	39	71	KLSDNCTGLQGFLVFNAVGGGTGSGLGSLLLER			Carbamidomethyl (C)[6]		Mascot
3380.644	3380.2737	-0.3703	-110	256	286	TVQFVDWCPTGFKCGINYQPPSVVPGGDLAK			Carbamidomethyl (C)[8]		Mascot

Rank	Protein Name	Accession No.	Protein MW	Protein PI	Pep. Count	Protein Score	Protein Score C. I. %	Total Ion Score	Total Ion C. I. %
7	Oxygen-evolving enhancer protein 1, chloroplastic OS=Triticum aestivum GN=PSBO PE=2 SV=1	PSBO_WHEA	34718.7	8.73	8	36	0		

Peptide Information

Calc. Mass	Obsrv. Mass	± da	± ppm	Start Seq.	End Seq.	Sequence	Ion Score	C. I. %	Modification	Rank	Result Type
842.4076	842.452	0.0444	53	122	128	AGKYEMK			Oxidation (M)[6]		Mascot
1135.5776	1135.4905	-0.0871	-77	41	50	ITCSLQSDIR					Mascot
1236.6583	1236.5681	-0.0902	-73	85	94	RLTFDEIQSK					Mascot
1238.6488	1238.5354	-0.1134	-92	141	151	AEGIQKNEPPR					Mascot
1360.6591	1360.5656	-0.0935	-69	258	269	GDEEELAKENVK					Mascot
1605.7727	1605.713	-0.0597	-37	241	257	GGSTGYDNAGALPRGGR					Mascot
1712.9476	1712.8016	-0.146	-85	2	19	AASLQAAATVMPAKIGGR					Mascot
1760.8813	1760.7433	-0.138	-78	186	202	DGIDYAAVTVQLPGGER					Mascot

Rank	Protein Name	Accession No.	Protein MW	Protein PI	Pep. Count	Protein Score	Protein Score C. I. %	Total Ion Score	Total Ion C. I. %
8	UPF0496 protein At5g66670 OS=Arabidopsis tha GN=At5g66670 PE=2 SV=1	U496G_ARAT	45568	8.96	9	35	0		

Peptide Information

Calc. Mass	Obsrv. Mass	± da	± ppm	Start Seq.	End Seq.	Sequence	Ion Score	C. I. %	Modification	Rank	Result Type
882.4389	882.5007	0.0618	70	350	356	FAMQEIK			Oxidation (M)[3]		Mascot
	882.5007										Mascot
954.5294	954.4127	-0.1167	-122	120	127	ALVDVYFK					Mascot
994.5389	994.4309	-0.108	-109	350	357	FAMQEIKK					Mascot
995.4098	995.3962	-0.0136	-14	342	349	EENEMATR			Oxidation (M)[5]		Mascot
1255.6755	1255.5975	-0.078	-62	2	12	VFCGLFSELIK					Mascot
1255.6755	1255.5975	-0.078	-62	2	12	VFCGLFSELIK					Mascot
1386.7159	1386.567	-0.1489	-107	1	12	MVFCGLFSELIK					Mascot
1826.8412	1826.7542	-0.087	-48	342	356	EENEMATRFAMQEIK					Mascot
1826.8654	1826.7542	-0.1112	-61	159	174	QFETETVDTDLGESKK					Mascot
1842.8361	1842.7483	-0.0878	-48	342	356	EENEMATRFAMQEIK			Oxidation (M)[5]		Mascot
1858.8309	1858.7397	-0.0912	-49	342	356	EENEMATRFAMQEIK			Oxidation (M)[5,11]		Mascot
3347.77	3347.3452	-0.4248	-127	298	327	GLVLTMELGVQANNVTMVNIKFEVENLSIR			Oxidation (M)[6]		Mascot

Rank	Protein Name	Accession No.	Protein MW	Protein PI	Pep. Count	Protein Score	Protein Score C. I. %	Total Ion Score	Total Ion C. I. %
9	Major pollen allergen Ole e 1 OS=Olea europaea SV=2	ALL1_OLEEU	16319.1	6.18	6	35	0		

Peptide Information

Calc. Mass	Obsrv. Mass	± da	± ppm	Start Seq.	End Seq.	Sequence	Ion Score	C. I. %	Modification	Rank	Result Type
882.4138	882.5007	0.0869	98	131	137	CAQVYNK			Carbamidomethyl (C)[1]		Mascot
954.4059	954.4127	0.0068	7	138	145	LGMYPPNM			Oxidation (M)[3,8]		Mascot
1267.6351	1267.6022	-0.0329	-26	61	71	AEGLYSMLVER					Mascot
1760.7804	1760.7433	-0.0371	-21	131	145	CAQVYNKLGMYPPNM			Oxidation (M)[10,15]		Mascot
1830.8505	1830.7584	-0.0921	-50	45	60	DKENGDVTFTEVGYTR					Mascot
1972.9796	1972.7394	-0.2402	-122	88	104	KDCNEIPTEGWAKPSLK			Carbamidomethyl (C)[3]		Mascot

Rank	Protein Name	Accession No.	Protein MW	Protein PI	Pep. Count	Protein Score	Protein Score C. I. %	Total Ion Score	Total Ion C. I. %
10	V-type proton ATPase catalytic subunit A OS=Gossypium hirsutum GN=CVA69.24 PE=2 SV=1	VATA_GOSHI	68478.9	5.36	7	35	0	15	0

Peptide Information

Calc. Mass	Obsrv. Mass	± da	± ppm	Start Seq.	End Seq.	Sequence	Ion Score	C. I. %	Modification	Rank	Result Type
862.4152	862.4198	0.0046	5	502	509	DALAETDK					Mascot Mascot
1119.547	1119.4978	-0.0492	-44	133	141	DALWDFQPK	2	0			Mascot
1119.547	1119.4978	-0.0492	-44	133	141	DALWDFQPK					Mascot
1135.5742	1135.4905	-0.0837	-74	607	616	LNEDLTAGFR					Mascot
1255.7004	1255.5975	-0.1029	-82	223	234	LAADTPLLTGQR	15	0			Mascot
1255.7004	1255.5975	-0.1029	-82	223	234	LAADTPLLTGQR					Mascot
1712.656	1712.8016	0.1456	85	342	356	DMGYNVSMMADSTSR			Oxidation (M)[2,8,9]		Mascot
1772.9541	1772.8534	-0.1007	-57	116	132	SGDVYIPRGVSVPALDK					Mascot
1772.9541	1772.8534	-0.1007	-57	116	132	SGDVYIPRGVSVPALDK					Mascot
2057.0337	2056.8647	-0.169	-82	517	533	LLREDYLAQNAFTPYDK					Mascot

Gel Idx/Pos	93/D18	Instr./Gel Origin	BA2060/131017A	Process Sta	Analysis Succeeded
Plate [#] Name	[1] 31496	Instrument Sample Name		Spectra	11

Rank	Protein Name	Accession No.	Protein MW	Protein PI	Pep. Count	Protein Score	Protein Score C. I. %	Total Ion Score	Total Ion C. I. %
1	Carbonic anhydrase 1 OS=Flaveria linearis PE=2	CAH1_FLALI	35551.8	5.7	5	101	100	84	100

Peptide Information

Calc. Mass	Obsrv. Mass	± da	± ppm	Start Seq.	End Seq.	Sequence	Ion Score	C. I. %	Modification	Rank	Result Type
1219.5234	1219.4403	-0.0831	-68	156	165	FMVFACSDSR			Carbamidomethyl (C)[6]		Mascot
1235.5183	1235.4302	-0.0881	-71	156	165	FMVFACSDSR			Carbamidomethyl (C)[6], Oxidation (M)[2]		Mascot
1892.0276	1891.8778	-0.1498	-79	277	293	EAVNVSLGNLLTYPFVR					Mascot
1985.9233	1985.8511	-0.0722	-36	37	55	FTCNSSSSSSATPPSLIR			Carbamidomethyl (C)[3]		Mascot
2057.0273	2056.8735	-0.1538	-75	166	183	VCPSHVLDFQPGEAFVVR	84	100	Carbamidomethyl (C)[2]		Mascot
2057.0273	2056.8735	-0.1538	-75	166	183	VCPSHVLDFQPGEAFVVR			Carbamidomethyl (C)[2]		Mascot
2238.9866	2238.969	-0.0176	-8	258	276	VVAEHNGTHLDDQCVQCEK			Carbamidomethyl (C)[14,17]		Mascot

Rank	Protein Name	Accession No.	Protein MW	Protein PI	Pep. Count	Protein Score	Protein Score C. I. %	Total Ion Score	Total Ion C. I. %
2	Carbonic anhydrase OS=Flaveria bidentis PE=2 S	CAHX_FLABI	35522.9	5.85	4	96	100	84	100

Peptide Information

Calc. Mass	Obsrv. Mass	± da	± ppm	Start Seq.	End Seq.	Sequence	Ion Score	C. I. %	Modification	Rank	Result Type
1219.5234	1219.4403	-0.0831	-68	156	165	FMVFACSDSR			Carbamidomethyl (C)[6]		Mascot
1235.5183	1235.4302	-0.0881	-71	156	165	FMVFACSDSR			Carbamidomethyl (C)[6], Oxidation (M)[2]		Mascot
1892.0276	1891.8778	-0.1498	-79	277	293	EAVNVSLGNLLTYPFVR					Mascot
2057.0273	2056.8735	-0.1538	-75	166	183	VCPSHVLDFQPGEAFVVR	84	100	Carbamidomethyl (C)[2]		Mascot
2057.0273	2056.8735	-0.1538	-75	166	183	VCPSHVLDFQPGEAFVVR			Carbamidomethyl (C)[2]		Mascot
2246.0942	2245.9688	-0.1254	-56	1	22	MSAASAFAMNAPSFVNASSLKK			Oxidation (M)[1]		Mascot

Rank	Protein Name	Accession No.	Protein MW	Protein PI	Pep. Count	Protein Score	Protein Score C. I. %	Total Ion Score	Total Ion C. I. %
3	Carbonic anhydrase OS=Flaveria brownii PE=2 S	CAHX_FLABR	35524.8	5.7	4	96	100	84	100

Peptide Information

Calc. Mass	Obsrv. Mass	± da	± ppm	Start Seq.	End Seq.	Sequence	Ion Score	C. I. %	Modification	Rank	Result Type
1219.5234	1219.4403	-0.0831	-68	156	165	FMVFACSDSR			Carbamidomethyl (C)[6]		Mascot
1235.5183	1235.4302	-0.0881	-71	156	165	FMVFACSDSR			Carbamidomethyl (C)[6], Oxidation (M)[2]		Mascot
1892.0276	1891.8778	-0.1498	-79	277	293	EAVNVSLGNLLTYPFVR					Mascot
1985.9233	1985.8511	-0.0722	-36	37	55	FTCNSSSSSSATPPSLIR			Carbamidomethyl (C)[3]		Mascot
2057.0273	2056.8735	-0.1538	-75	166	183	VCPSHVLDFQPGEAFVVR	84	100	Carbamidomethyl (C)[2]		Mascot
2057.0273	2056.8735	-0.1538	-75	166	183	VCPSHVLDFQPGEAFVVR			Carbamidomethyl (C)[2]		Mascot

Rank	Protein Name	Accession No.	Protein MW	Protein PI	Pep. Count	Protein Score	Protein Score C. I. %	Total Ion Score	Total Ion C. I. %
4	Carbonic anhydrase OS=Flaveria pringlei PE=2 S	CAHX_FLAPR	35463.8	5.7	4	96	100	84	100

Peptide Information

Calc. Mass	Obsrv. Mass	± da	± ppm	Start Seq.	End Seq.	Sequence	Ion Score	C. I. %	Modification	Rank	Result Type
1219.5234	1219.4403	-0.0831	-68	155	164	FMVFACSDSR			Carbamidomethyl (C)[6]		Mascot
1235.5183	1235.4302	-0.0881	-71	155	164	FMVFACSDSR			Carbamidomethyl (C)[6], Oxidation (M)[2]		Mascot
1892.0276	1891.8778	-0.1498	-79	276	292	EAVNVSLGNLLTYPFVR					Mascot
1985.9233	1985.8511	-0.0722	-36	36	54	FTCNSSSSSSATPPSLIR			Carbamidomethyl (C)[3]		Mascot
2057.0273	2056.8735	-0.1538	-75	165	182	VCPSHVLDFQPGEAFVVR	84	100	Carbamidomethyl (C)[2]		Mascot
2057.0273	2056.8735	-0.1538	-75	165	182	VCPSHVLDFQPGEAFVVR			Carbamidomethyl (C)[2]		Mascot

Rank	Protein Name	Accession No.	Protein MW	Protein PI	Pep. Count	Protein Score	Protein Score C. I. %	Total Ion Score	Total Ion C. I. %
5	Carbonic anhydrase, chloroplastic OS=Pisum sat PE=1 SV=1	CAHC_PEA	35355.1	7.01	2	89	99.996	84	100

Peptide Information

Calc. Mass	Obsrv. Mass	± da	± ppm	Start Seq.	End Seq.	Sequence	Ion Score	C. I. %	Modification	Rank	Result Type
948.446	948.3774	-0.0686	-72	303	310	GGYYDFVK					Mascot
2057.0273	2056.8735	-0.1538	-75	164	181	VCPSHVLDFQPGEAFVVR	84	100	Carbamidomethyl (C)[2]		Mascot
2057.0273	2056.8735	-0.1538	-75	164	181	VCPSHVLDFQPGEAFVVR			Carbamidomethyl (C)[2]		Mascot

Rank	Protein Name	Accession No.	Protein MW	Protein PI	Pep. Count	Protein Score	Protein Score C. I. %	Total Ion Score	Total Ion C. I. %
6	Ribulose bisphosphate carboxylase small chain (512 (Fragment) OS=Triticum aestivum PE=3 SV=1	RBS3_WHEAT	13046.5	5.84	7	52	80.64		

Peptide Information

Calc. Mass	Obsrv. Mass	± da	± ppm	Start Seq.	End Seq.	Sequence	Ion Score	C. I. %	Modification	Rank	Result Type
914.4229	914.3405	-0.0824	-90	50	55	YWTMWK					Mascot
1974.9377	1974.8479	-0.0898	-45	77	92	EYPDAYVRIIGFDNMR			Oxidation (M)[15]		Mascot
2084.9937	2084.8896	-0.1041	-50	32	49	VGFIFREHNASPGYYDGR					Mascot
2084.9937	2084.8896	-0.1041	-50	32	49	VGFIFREHNASPGYYDGR					Mascot
2211.0571	2210.9387	-0.1184	-54	93	112	QVQCVSFIAFKPPGCEESGK			Carbamidomethyl (C)[4]		Mascot
2225.073	2224.9419	-0.1311	-59	93	113	QVQCVSFIAFKPPGCEESGKA					Mascot
2239.062	2238.969	-0.093	-42	56	75	LPMFGCTDATQVINEVEEVK			Oxidation (M)[3]		Mascot
3100.5051	3100.3477	-0.1574	-51	85	112	IIGFDNMRQVQCVSFIAFKPPGCEESGK					Mascot

Rank	Protein Name	Accession No.	Protein MW	Protein PI	Pep. Count	Protein Score	Protein Score C. I. %	Total Ion Score	Total Ion C. I. %
7	Ribulose bisphosphate carboxylase small chain, chloroplastic OS=Batophora oerstedii GN=RBCS PE=2 SV	RBS_BATOE	20096.8	7.57	6	42	0	6	0

Peptide Information

Calc. Mass	Obsrv. Mass	± da	± ppm	Start Seq.	End Seq.	Sequence	Ion Score	C. I. %	Modification	Rank	Result Type
914.4229	914.3405	-0.0824	-90	107	112	YWTMWK					Mascot
933.5073	933.4541	-0.0532	-57	9	17	TIVPCATTK					Mascot
994.4774	994.4086	-0.0688	-69	142	149	LVCFDANR			Carbamidomethyl (C)[3]		Mascot
994.4774	994.4086	-0.0688	-69	142	149	LVCFDANR	6	0	Carbamidomethyl (C)[3]		Mascot
1360.5256	1360.5702	0.0446	33	95	106	MGDCASTYQDNR					Mascot
1625.7562	1625.7449	-0.0113	-7	33	45	SRAMMVWEPFNNK			Oxidation (M)[4]		Mascot
1886.9351	1886.8292	-0.1059	-56	1	17	MSFATTNKTIVPCATTK			Carbamidomethyl (C)[13], Oxidation (M)[1]		Mascot

Rank	Protein Name	Accession No.	Protein MW	Protein PI	Pep. Count	Protein Score	Protein Score C. I. %	Total Ion Score	Total Ion C. I. %
8	Triosephosphate isomerase, cytosolic OS=Petun hybrida GN=TPIP1 PE=2 SV=1	TPIS_PETHY	27115.1	5.54	4	40	0	25	56.824

Peptide Information

Calc. Mass	Obsrv. Mass	± da	± ppm	Start Seq.	End Seq.	Sequence	Ion Score	C. I. %	Modification	Rank	Result Type
954.4832	954.416	-0.0672	-70	5	12	FFVGGNWK					Mascot
954.4832	954.416	-0.0672	-70	5	12	FFVGGNWK	25	56.824			Mascot
1082.5781	1082.501	-0.0771	-71	4	12	KFFVGGNWK					Mascot
1605.8344	1605.7161	-0.1183	-74	176	190	VASPAQAQEVHAELR					Mascot
1733.9293	1733.8015	-0.1278	-74	176	191	VASPAQAQEVHAELRK					Mascot

Rank	Protein Name	Accession No.	Protein MW	Protein PI	Pep. Count	Protein Score	Protein Score C. I. %	Total Ion Score	Total Ion C. I. %
9	Triosephosphate isomerase, cytosolic OS=Secal cereale PE=2 SV=3	TPIS_SECCE	26910	5.24	4	40	0	25	56.824

Peptide Information

Calc. Mass	Obsrv. Mass	± da	± ppm	Start Seq.	End Seq.	Sequence	Ion Score	C. I. %	Modification	Rank	Result Type
954.4832	954.416	-0.0672	-70	5	12	FFVGGNWK					Mascot
954.4832	954.416	-0.0672	-70	5	12	FFVGGNWK	25	56.824			Mascot
1082.5781	1082.501	-0.0771	-71	4	12	KFFVGGNWK					Mascot
1255.6351	1255.5992	-0.0359	-29	207	219	IIYGGSVTGASCK					Mascot
1395.7002	1395.6388	-0.0614	-44	101	113	SLLAESSEFVGEK					Mascot

Rank	Protein Name	Accession No.	Protein MW	Protein PI	Pep. Count	Protein Score	Protein Score C. I. %	Total Ion Score	Total Ion C. I. %
10	Triosephosphate isomerase, cytosolic OS=Stella longipes GN=TPI PE=2 SV=1	TPIS_STELP	27482.1	5.54	4	40	0	25	56.824

Peptide Information

Calc. Mass	Obsrv. Mass	± da	± ppm	Start Seq.	End Seq.	Sequence	Ion Score	C. I. %	Modification	Rank	Result Type
954.4832	954.416	-0.0672	-70	5	12	FFVGGNWK					Mascot
954.4832	954.416	-0.0672	-70	5	12	FFVGGNWK	25	56.824			Mascot
1082.5781	1082.501	-0.0771	-71	4	12	KFFVGGNWK					Mascot
1228.6394	1228.5531	-0.0863	-70	140	149	TFDVCFKQLK					Mascot
1605.8119	1605.7161	-0.0958	-60	192	205	DWLKTNVSEEVASK					Mascot

Gel Idx/Pos	94/D19	Instr./Gel Origin	BA2060/131017A	Process Sta	Analysis Succeeded
Plate [#] Name	[1] 31496	Instrument Sample Name		Spectra	11

Rank	Protein Name	Accession No	Protein MW	Protein PI	Pep. Count	Protein Score	Protein Score C. I. %	Total Ion Score	Total Ion C. I. %
1	RuBisCO-associated protein OS=Glycine max PE SV=1	RUAP_SOYBI	31239.2	6	8	136	100	88	100

Peptide Information

Calc. Mass	Obsrv. Mass	± da	± ppm	Start Seq.	End Seq.	Sequence	Ion Score	C. I. %	Modification	Rank	Result Type
849.4464	849.3861	-0.0603	-71	70	76	YEPTALR					Mascot
977.5414	977.4721	-0.0693	-71	69	76	KYEPTALR					Mascot
1057.5524	1057.4822	-0.0702	-66	198	207	ANPVASLEEK					Mascot
1198.5779	1198.5205	-0.0574	-48	49	57	FIPYWDTEK					Mascot
1198.5779	1198.5205	-0.0574	-48	49	57	FIPYWDTEK	5	0			Mascot
1353.8141	1353.7247	-0.0894	-66	272	283	WIPEILLLAASK					Mascot
2572.2202	2571.967	-0.2532	-98	89	112	QFPFTIGSDSNSEAWVSEATASLK					Mascot
3213.595	3213.3364	-0.2586	-80	10	37	EFTSDDSFLNQVIPENITEFQVTLSLAR					Mascot
3216.533	3216.3508	-0.1822	-57	117	146	TYNLDGIDVSYEDIAANEADFVNSVGGLVR	83	100			Mascot
3216.533	3216.3508	-0.1822	-57	117	146	TYNLDGIDVSYEDIAANEADFVNSVGGLVR					Mascot

2	Magnesium-protoporphyrin IX monomethyl ester [oxidative] cyclase 2, chloroplastic OS=Chlamydomonas	CTH1_CHLRE	47451	8.7	10	44	0		

Peptide Information

Calc. Mass	Obsrv. Mass	± da	± ppm	Start Seq.	End Seq.	Sequence	Ion Score	C. I. %	Modification	Rank	Result Type
849.4611	849.3861	-0.075	-88	2	8	ATMLNKR			Oxidation (M)[3]		Mascot
996.4965	996.5422	0.0457	46	1	8	MATMLNKR			Oxidation (M)[1,4]		Mascot
1157.671	1157.5066	-0.1644	-142	368	377	AIMKAPILER			Oxidation (M)[3]		Mascot
1198.5222	1198.5205	-0.0017	-1	392	402	ESGSYDLDANK					Mascot
1198.5222	1198.5205	-0.0017	-1	392	402	ESGSYDLDANK					Mascot
1381.7039	1381.5743	-0.1296	-94	209	219	FIFYATYLSEK					Mascot
1465.7031	1465.6666	-0.0365	-25	137	149	SCTAEFSGFLLYK					Mascot
1465.7031	1465.6666	-0.0365	-25	137	149	SCTAEFSGFLLYK					Mascot
1893.7704	1893.8486	0.0782	41	87	101	NMDMEEFEAMLNEFK			Oxidation (M)[2]		Mascot
1893.7704	1893.8486	0.0782	41	87	101	NMDMEEFEAMLNEFK			Oxidation (M)[2]		Mascot
2057.053	2056.8628	-0.1902	-92	209	224	FIFYATYLSEKIGYWR					Mascot
2150.0198	2149.9419	-0.0779	-36	283	299	FFCLSVYITMYLNDHQR					Mascot
3213.5811	3213.3364	-0.2447	-76	300	327	DAFYSSLGLNTTQFNQHVIIETNKSTER					Mascot

3	Ribonuclease S-4 OS=Pyrus pyrifolia PE=1 SV=2	RNS4_PYRPY	25840.8	9.17	7	42	0		

Peptide Information

Calc. Mass	Obsrv. Mass	± da	± ppm	Start Seq.	End Seq.	Sequence	Ion Score	C. I. %	Modification	Rank	Result Type
806.3937	806.4462	0.0525	65	183	188	CQKNTR			Carbamidomethyl (C)[1]		Mascot
910.4299	910.3985	-0.0314	-34	77	84	TTTMNSQK					Mascot
1157.5289	1157.5066	-0.0223	-19	75	84	CKTTTMNSQK			Oxidation (M)[6]		Mascot
1198.5555	1198.5205	-0.035	-29	75	84	CKTTTMNSQK			Carbamidomethyl (C)[1]		Mascot
1198.5555	1198.5205	-0.035	-29	75	84	CKTTTMNSQK			Carbamidomethyl (C)[1]		Mascot
1234.6538	1234.6108	-0.043	-35	172	182	SGNNNTKPKFK					Mascot
1234.6538	1234.6108	-0.043	-35	172	182	SGNNNTKPKFK					Mascot
1466.7321	1466.6628	-0.0693	-47	207	219	FINCPHGPPKGSR			Carbamidomethyl (C)[4]		Mascot
1993.9971	1993.8425	-0.1546	-78	186	202	NTRTTTELVEVTLCSNR			Carbamidomethyl (C)[14]		Mascot
1994.9099	1994.8409	-0.069	-35	116	132	HGTCGYPTIKDDMHYLK			Oxidation (M)[13]		Mascot

4	Disease susceptibility protein LOV1 OS=Arabido thaliana GN=LOV1 PE=1 SV=1	LOV1B_ARAT	104444	6.83	15	39	0		

Peptide Information

Calc. Mass	Obsrv. Mass	± da	± ppm	Start Seq.	End Seq.	Sequence	Ion Score	C. I. %	Modification	Rank	Result Type
842.4518	842.4614	0.0096	11	313	319	SFGFKTR					Mascot
849.3373	849.3861	0.0488	57	278	283	EEDWDR					Mascot
850.4338	850.4947	0.0609	72	481	487	NMITIDK			Oxidation (M)[2]		Mascot
1006.5349	1006.5571	0.0222	22	480	487	RNMITIDK			Oxidation (M)[3]		Mascot
1021.5281	1021.451	-0.0771	-75	852	860	ALTICNCRK					Mascot
1107.5979	1107.4517	-0.1462	-132	293	301	GWKMLLTSR			Oxidation (M)[4]		Mascot
1194.5637	1194.4985	-0.0652	-55	470	479	GEDYLEELAR					Mascot
1275.7307	1275.6602	-0.0705	-55	863	874	LPGGINYITSLK					Mascot
1275.7307	1275.6602	-0.0705	-55	863	874	LPGGINYITSLK					Mascot
1493.7161	1493.6582	-0.0579	-39	359	372	EMVTCCGGLPLAVK			Carbamidomethyl (C)[5], Oxidation (M)[2]		Mascot
1493.7161	1493.6582	-0.0579	-39	359	372	EMVTCCGGLPLAVK			Carbamidomethyl (C)[5], Oxidation (M)[2]		Mascot
1838.9442	1838.7734	-0.1708	-93	199	213	TTLARQVFHHDMVQR					Mascot
1993.8169	1993.8425	0.0256	13	496	510	HCQMHDMMREVCLSK			Carbamidomethyl (C)[2,12], Oxidation (M)[4,7]		Mascot
2053.0522	2052.8381	-0.2141	-104	673	690	LELSDLVNLESLMNFSTK					Mascot
2211.2107	2210.9893	-0.2214	-100	1	19	MAEGVVLFGVHKLWELLNR					Mascot
2211.9744	2211.9397	-0.0347	-16	339	358	DETGTLSEVRVDEDMEAMGK					Mascot
2240.1558	2239.9917	-0.1641	-73	513	532	EENFLEIFKVSTATSAINAR					Mascot

5	Inactive disease susceptibility protein LOV1 OS=Arabidopsis thaliana GN=LOV1 PE=3 SV=1	LOV1C_ARAT	104358.9	6.66	15	39	0		

Peptide Information

Calc. Mass	Obsrv. Mass	± da	± ppm	Start Seq.	End Seq.	Sequence	Ion Score	C. I. %	Modification	Rank	Result Type
842.4518	842.4614	0.0096	11	313	319	SFGFKTR					Mascot
849.3373	849.3861	0.0488	57	278	283	EEDWDR					Mascot
850.4338	850.4947	0.0609	72	481	487	NMITIDK			Oxidation (M)[2]		Mascot
1006.5349	1006.5571	0.0222	22	480	487	RNMITIDK			Oxidation (M)[3]		Mascot
1021.5281	1021.451	-0.0771	-75	852	860	ALTICNCRK					Mascot
1107.5979	1107.4517	-0.1462	-132	293	301	GWKMLLTSR			Oxidation (M)[4]		Mascot
1194.5637	1194.4985	-0.0652	-55	470	479	GEDYLEELAR					Mascot
1275.7307	1275.6602	-0.0705	-55	863	874	LPGGINYITSLK					Mascot
1275.7307	1275.6602	-0.0705	-55	863	874	LPGGINYITSLK					Mascot
1493.7161	1493.6582	-0.0579	-39	359	372	EMVTCCGGLPLAVK			Carbamidomethyl (C)[5], Oxidation (M)[2]		Mascot
1493.7161	1493.6582	-0.0579	-39	359	372	EMVTCCGGLPLAVK			Carbamidomethyl (C)[5], Oxidation (M)[2]		Mascot
1838.9442	1838.7734	-0.1708	-93	199	213	TTLARQVFHHDMVQR					Mascot
1993.8169	1993.8425	0.0256	13	496	510	HCQMHDMMREVCLSK			Carbamidomethyl (C)[2,12], Oxidation (M)[4,7]		Mascot
2053.0522	2052.8381	-0.2141	-104	673	690	LELSDLVNLESLMNFSTK					Mascot
2211.2107	2210.9893	-0.2214	-100	1	19	MAEGVVLFGVHKLWELLNR					Mascot
2211.9744	2211.9397	-0.0347	-16	339	358	DETGTLSEVRVDEDMEAMGK					Mascot
2240.1558	2239.9917	-0.1641	-73	513	532	EENFLEIFKVSTATSAINAR					Mascot

6	Ras-related protein RABA3 OS=Arabidopsis thali GN=RABA3 PE=2 SV=1	RABA3_ARAT	26233.2	5.52	7	38	0		

Peptide Information

Calc. Mass	Obsrv. Mass	± da	± ppm	Start Seq.	End Seq.	Sequence	Ion Score	C. I. %	Modification	Rank	Result Type
1006.5567	1006.5571	0.0004	0	186	193	LLEEIFSR					Mascot
1193.583	1193.5428	-0.0402	-34	199	210	KAMESDGGATVK					Mascot
1194.6664	1194.4985	-0.1679	-141	15	24	HVKKPTMPEK					Mascot
1353.7083	1353.7247	0.0164	12	100	112	GALGAMVVYDITK			Oxidation (M)[6]		Mascot
1493.8145	1493.6582	-0.1563	-105	100	113	GALGAMVVYDITKR					Mascot
1493.8145	1493.6582	-0.1563	-105	100	113	GALGAMVVYDITKR					Mascot
2211.1438	2210.9893	-0.1545	-70	128	149	AHADDSAVIMLVGNKADLSVGK					Mascot
2299.0427	2299.0227	-0.02	-9	216	237	IDVISGSDLETSNIKEQASCSC					Mascot

7	Ribulose bisphosphate carboxylase large chain OS=Pinus edulis GN=rbcL PE=3 SV=1	RBL_PINED	52663.5	6.19	9	37	0	3	0

Peptide Information

Calc. Mass	Obsrv. Mass	± da	± ppm	Start Seq.	End Seq.	Sequence	Ion Score	C. I. %	Modification	Rank	Result Type
910.4451	910.3985	-0.0466	-51	188	194	AVYECLR			Carbamidomethyl (C)[5]		Mascot
1021.5312	1021.451	-0.0802	-79	33	41	DTDILAAFR					Mascot
1198.5647	1198.5205	-0.0442	-37	304	312	QRNHGMHFR			Oxidation (M)[6]		Mascot
1198.5924	1198.5205	-0.0719	-60	218	227	FVFCAEAINK	5	0	Carbamidomethyl (C)[4]		Mascot
1465.7216	1465.6666	-0.055	-38	320	334	MSGGDHIHAGTVVGK					Mascot
1465.7546	1465.6666	-0.088	-60	147	159	TFQGPPHGIQVER					Mascot
1560.7515	1560.6548	-0.0967	-62	451	463	WSPELAAACEIWK			Carbamidomethyl (C)[9]		Mascot
1560.7515	1560.6548	-0.0967	-62	451	463	WSPELAAACEIWK			Carbamidomethyl (C)[9]		Mascot
1893.8945	1893.8486	-0.0459	-24	237	253	GHYLNATAGTCEEMIKR					Mascot
1893.8945	1893.8486	-0.0459	-24	237	253	GHYLNATAGTCEEMIKR					Mascot
1995.0433	1994.8409	-0.2024	-101	340	356	DVTLGFVDLLRDDFIEK					Mascot

8	Ribulose bisphosphate carboxylase large chain OS=Pinus pinea GN=rbcL PE=3 SV=1	RBL_PINPI	52708.5	6.19	9	36	0	3	0

Peptide Information

Calc. Mass	Obsrv. Mass	± da	± ppm	Start Seq.	End Seq.	Sequence	Ion Score	C. I. %	Modification	Rank	Result Type
910.4451	910.3985	-0.0466	-51	188	194	AVYECLR			Carbamidomethyl (C)[5]		Mascot
1021.5312	1021.451	-0.0802	-79	33	41	DTDILAAFR					Mascot
1198.5647	1198.5205	-0.0442	-37	304	312	QRNHGMHFR			Oxidation (M)[6]		Mascot
1198.5924	1198.5205	-0.0719	-60	218	227	FVFCAEAINK	5	0	Carbamidomethyl (C)[4]		Mascot
1465.7216	1465.6666	-0.055	-38	320	334	MSGGDHIHAGTVVGK					Mascot
1465.7546	1465.6666	-0.088	-60	147	159	TFQGPPHGIQVER					Mascot
1560.7515	1560.6548	-0.0967	-62	451	463	WSPELAAACEIWK			Carbamidomethyl (C)[9]		Mascot
1560.7515	1560.6548	-0.0967	-62	451	463	WSPELAAACEIWK			Carbamidomethyl (C)[9]		Mascot
1893.8945	1893.8486	-0.0459	-24	237	253	GHYLNATAGTCEEMIKR					Mascot
1893.8945	1893.8486	-0.0459	-24	237	253	GHYLNATAGTCEEMIKR					Mascot
1995.0433	1994.8409	-0.2024	-101	340	356	DVTLGFVDLLRDDFIEK					Mascot

9	Ribulose bisphosphate carboxylase large chain (Fragment) OS=Amorphophallus titanum GN=rbcL PE=3 SV=	RBL_AMOTI	52548.4	6.32	9	36	0		

Peptide Information

Calc. Mass	Obsrv. Mass	± da	± ppm	Start Seq.	End Seq.	Sequence	Ion Score	C. I. %	Modification	Rank	Result Type
1021.5312	1021.451	-0.0802	-79	27	35	DTDILAAFR					Mascot
1358.715	1358.623	-0.092	-68	300	310	YHGMHFRVLAK					Mascot
1461.6361	1461.6968	0.0607	42	178	188	NYGRACYECLR			Carbamidomethyl (C)[6,9]		Mascot
1465.7216	1465.6666	-0.055	-38	314	328	MSGGDHIHAGTVVGK					Mascot
1465.7216	1465.6666	-0.055	-38	314	328	MSGGDHIHAGTVVGK					Mascot
1475.7351	1475.663	-0.0721	-49	210	221	DRFLFCAEALYK					Mascot
1560.7515	1560.6548	-0.0967	-62	445	457	WSPELAAACEIWK			Carbamidomethyl (C)[9]		Mascot
1560.7515	1560.6548	-0.0967	-62	445	457	WSPELAAACEIWK			Carbamidomethyl (C)[9]		Mascot
1893.8945	1893.8486	-0.0459	-24	231	247	GHYLNATAGTCEEMIKR					Mascot
1893.8945	1893.8486	-0.0459	-24	231	247	GHYLNATAGTCEEMIKR					Mascot
2057.0259	2056.8628	-0.1631	-79	334	350	EMTLGFVDLLRDDYIEK					Mascot
2211.1191	2210.9893	-0.1298	-59	134	153	IPPAYSKTFEGPPHGIQSER					Mascot

10	Protein NIM1-INTERACTING 2 OS=Arabidopsis thaliana GN=NIMIN-2 PE=1 SV=1	NIMI2_ARATH	13499.9	5.93	5	35	0		

Peptide Information

Calc. Mass	Obsrv. Mass	± da	± ppm	Start Seq.	End Seq.	Sequence	Ion Score	C. I. %	Modification	Rank	Result Type
850.4451	850.4947	0.0496	58	1	7	MNNSLKK			Oxidation (M)[1]		Mascot
977.4468	977.4721	0.0253	26	82	90	NSLDCNGVR					Mascot
1501.7717	1501.6294	-0.1423	-95	18	31	SDGNRGKPSTEVVR					Mascot
2052.9038	2052.8381	-0.0657	-32	82	99	NSLDCNGVRDGEFDEINR					Mascot
2426.2085	2425.95	-0.2585	-107	23	43	GKPSTEVVRTVTEEEVDEFFK					Mascot

Gel Idx/Pos	95/D20	Instr./Gel Origin	BA2060/131017A	Process Sta	Analysis Succeeded
Plate [#] Name	[1] 31496	Instrument Sample Name		Spectra	11

Rank	Protein Name	Accession No	Protein MW	Protein PI	Pep. Count	Protein Score	Protein Score C. I. %	Total Ion Score	Total Ion C. I. %
1	Ribulose bisphosphate carboxylase large chain (Fragment) OS=Byrsonima crassifolia GN=rbcL PE=3 SV=1	RBL_BYRCR	51554.9	6.09	13	70	99.693	6	0

Peptide Information

Calc. Mass	Obsrv. Mass	± da	± ppm	Start Seq.	End Seq.	Sequence	Ion Score	C. I. %	Modification	Rank	Result Type
805.4929	805.4553	-0.0376	-47	1	8	VGFKAGVK					Mascot
910.4451	910.3904	-0.0547	-60	178	184	AVYECLR			Carbamidomethyl (C)[5]		Mascot
914.4049	914.3474	-0.0575	-63	296	302	NHGMHFR			Oxidation (M)[4]		Mascot
1021.5312	1021.4674	-0.0638	-62	23	31	DTDILAAFR					Mascot
1116.583	1116.5176	-0.0654	-59	412	421	VALEACVQAR			Carbamidomethyl (C)[6]		Mascot
1170.5585	1170.571	0.0125	11	294	302	QKNHGMHFR			Oxidation (M)[6]		Mascot
1187.6643	1187.5975	-0.0668	-56	276	285	DNGLLLHIHR					Mascot
1275.7307	1275.6504	-0.0803	-63	330	340	EITLGFVDLLR	1	0			Mascot
1275.7307	1275.6504	-0.0803	-63	330	340	EITLGFVDLLR					Mascot
1465.7216	1465.6561	-0.0655	-45	310	324	MSGGDHIHAGTVVGK					Mascot
1465.7546	1465.6561	-0.0985	-67	137	149	TFQGPPHGIQVER	7	0			Mascot
1502.8512	1502.7539	-0.0973	-65	155	167	YGRPLLGCTIKPK			Carbamidomethyl (C)[8]		Mascot
1961.9425	1961.7662	-0.1763	-90	437	453	EASKWSPELAAACEVWK			Carbamidomethyl (C)[13]		Mascot
1984.8673	1984.8652	-0.0021	-1	227	243	GHYLNATAGTCEEMMKR			Carbamidomethyl (C)[11], Oxidation (M)[14]		Mascot

2	Ribulose bisphosphate carboxylase large chain (Fragment) OS=Morus rubra GN=rbcL PE=3 SV=1	RBL_MORRU	51574	6	13	70	99.693	6	0

Peptide Information

Calc. Mass	Obsrv. Mass	± da	± ppm	Start Seq.	End Seq.	Sequence	Ion Score	C. I. %	Modification	Rank	Result Type
805.4929	805.4553	-0.0376	-47	1	8	VGFKAGVK					Mascot
910.4451	910.3904	-0.0547	-60	178	184	AVYECLR			Carbamidomethyl (C)[5]		Mascot
914.4049	914.3474	-0.0575	-63	296	302	NHGMHFR			Oxidation (M)[4]		Mascot
1021.5312	1021.4674	-0.0638	-62	23	31	DTDILAAFR					Mascot
1116.6194	1116.5176	-0.1018	-91	412	421	VALEACVKAR			Carbamidomethyl (C)[6]		Mascot
1170.5585	1170.571	0.0125	11	294	302	QKNHGMHFR			Oxidation (M)[6]		Mascot
1187.6643	1187.5975	-0.0668	-56	276	285	DNGLLLHIHR					Mascot
1275.7307	1275.6504	-0.0803	-63	330	340	EITLGFVDLLR	1	0			Mascot
1275.7307	1275.6504	-0.0803	-63	330	340	EITLGFVDLLR					Mascot
1465.7216	1465.6561	-0.0655	-45	310	324	MSGGDHIHAGTVVGK					Mascot
1465.7546	1465.6561	-0.0985	-67	137	149	TFQGPPHGIQVER	7	0			Mascot
1502.8512	1502.7539	-0.0973	-65	155	167	YGRPLLGCTIKPK			Carbamidomethyl (C)[8]		Mascot
1961.9425	1961.7662	-0.1763	-90	437	453	EASKWSPELAAACEVWK			Carbamidomethyl (C)[13]		Mascot
1984.8673	1984.8652	-0.0021	-1	227	243	GHYLNATAGTCEEMMKR			Carbamidomethyl (C)[11], Oxidation (M)[14]		Mascot

3	Ribulose bisphosphate carboxylase large chain (Fragment) OS=Cassia fistula GN=rbcL PE=3 SV=1	RBL_CASFS	51606.1	6.14	13	69	99.576	6	0

Peptide Information

Calc. Mass	Obsrv. Mass	± da	± ppm	Start Seq.	End Seq.	Sequence	Ion Score	C. I. %	Modification	Rank	Result Type
805.4929	805.4553	-0.0376	-47	1	8	VGFKAGVK					Mascot
910.4451	910.3904	-0.0547	-60	178	184	AVYECLR			Carbamidomethyl (C)[5]		Mascot
914.4049	914.3474	-0.0575	-63	296	302	NHGMHFR			Oxidation (M)[4]		Mascot
1021.5312	1021.4674	-0.0638	-62	23	31	DTDILAAFR					Mascot
1116.583	1116.5176	-0.0654	-59	412	421	VALEACVQAR			Carbamidomethyl (C)[6]		Mascot
1170.5585	1170.571	0.0125	11	294	302	QKNHGMHFR			Oxidation (M)[6]		Mascot
1187.6643	1187.5975	-0.0668	-56	276	285	DNGLLLHIHR					Mascot
1194.5322	1194.4813	-0.0509	-43	208	217	FCFCAEALYK					Mascot
1275.7307	1275.6504	-0.0803	-63	330	340	EITLGFVDLLR	1	0			Mascot
1275.7307	1275.6504	-0.0803	-63	330	340	EITLGFVDLLR					Mascot
1465.6603	1465.6561	-0.0042	-3	206	217	DRFCFCAEALYK					Mascot
1465.7546	1465.6561	-0.0985	-67	137	149	TFQGPPHGIQVER	7	0			Mascot
1502.8512	1502.7539	-0.0973	-65	155	167	YGRPLLGCTIKPK			Carbamidomethyl (C)[8]		Mascot
1961.9425	1961.7662	-0.1763	-90	437	453	EASKWSPELAAACEVWK			Carbamidomethyl (C)[13]		Mascot

4	Ribulose bisphosphate carboxylase large chain (Fragment) OS=Gladiolus quienzii GN=rbcL PE=3 SV=1	RBL_GLAGU	50712.3	6.23	13	69	99.576	5	0

Peptide Information

Calc. Mass	Obsrv. Mass	± da	± ppm	Start Seq.	End Seq.	Sequence	Ion Score	C. I. %	Modification	Rank	Result Type
910.4451	910.3904	-0.0547	-60	181	187	AVYECLR			Carbamidomethyl (C)[5]		Mascot
914.4049	914.3474	-0.0575	-63	299	305	NHGMHFR			Oxidation (M)[4]		Mascot
1021.5312	1021.4674	-0.0638	-62	26	34	DTDILAAFR					Mascot
1116.583	1116.5176	-0.0654	-59	415	424	VALEACVQAR			Carbamidomethyl (C)[6]		Mascot
1170.5585	1170.571	0.0125	11	297	305	QKNHGMHFR			Oxidation (M)[6]		Mascot
1187.6643	1187.5975	-0.0668	-56	279	288	DNGLLLHIHR					Mascot
1465.7216	1465.6561	-0.0655	-45	313	327	MSGGDHIHAGTVVGK					Mascot
1465.7546	1465.6561	-0.0985	-67	140	152	TFQGPPHGIQVER	7	0			Mascot
1502.8512	1502.7539	-0.0973	-65	158	170	YGRPLLGCTIKPK			Carbamidomethyl (C)[8]		Mascot
1891.8466	1891.8995	0.0529	28	440	456	EACNWSPGLAAACEVWK			Carbamidomethyl (C)[3]		Mascot
1984.8673	1984.8652	-0.0021	-1	230	246	GHYLNATAGTCEEMMKR			Carbamidomethyl (C)[11], Oxidation (M)[14]		Mascot
2057.0259	2056.8831	-0.1428	-69	333	349	EMTLGFVDLLRDDYIEK					Mascot
2057.0259	2056.8831	-0.1428	-69	333	349	EMTLGFVDLLRDDYIEK					Mascot
3855.8672	3855.6294	-0.2378	-62	35	72	VTPQPGVPAEEAGAAVAAESSTGTWTTVWTDGLTNLDR					Mascot

5	Ribulose bisphosphate carboxylase large chain (Fragment) OS=Manilkara zapota GN=rbcL PE=3 SV=2	RBL_MANZA	51550	6.04	12	65	98.86	7	0

Peptide Information

Calc. Mass	Obsrv. Mass	± da	± ppm	Start Seq.	End Seq.	Sequence	Ion Score	C. I. %	Modification	Rank	Result Type
805.4929	805.4553	-0.0376	-47	1	8	VGFKAGVK					Mascot
910.4451	910.3904	-0.0547	-60	178	184	AVYECLR			Carbamidomethyl (C)[5]		Mascot
914.4049	914.3474	-0.0575	-63	296	302	NHGMHFR			Oxidation (M)[4]		Mascot
1021.5312	1021.4674	-0.0638	-62	23	31	DTDILAAFR					Mascot
1039.4691	1039.4707	0.0016	2	341	348	DDYVEKDR					Mascot
1116.583	1116.5176	-0.0654	-59	412	421	VALEACVQAR			Carbamidomethyl (C)[6]		Mascot
1170.5585	1170.571	0.0125	11	294	302	QKNHGMHFR			Oxidation (M)[6]		Mascot
1187.6643	1187.5975	-0.0668	-56	276	285	DNGLLLHIHR					Mascot
1275.7307	1275.6504	-0.0803	-63	330	340	EITLGFVDLLR	1	0			Mascot
1275.7307	1275.6504	-0.0803	-63	330	340	EITLGFVDLLR					Mascot
1465.7546	1465.6561	-0.0985	-67	137	149	TFQGPPHGIQVER	7	0			Mascot
1465.7546	1465.6561	-0.0985	-67	137	149	TFQGPPHGIQVER					Mascot
1502.8512	1502.7539	-0.0973	-65	155	167	YGRPLLGCTIKPK			Carbamidomethyl (C)[8]		Mascot
1961.9425	1961.7662	-0.1763	-90	437	453	EASKWSPELAAACEVWK			Carbamidomethyl (C)[13]		Mascot

6	Ribulose bisphosphate carboxylase large chain (Fragment) OS=Acacia farnesiana GN=rbcL PE=3 SV=1	RBL_ACAFA	50346.3	6.22	12	63	98.352	7	0

Peptide Information

Calc. Mass	Obsrv. Mass	± da	± ppm	Start Seq.	End Seq.	Sequence	Ion Score	C. I. %	Modification	Rank	Result Type
910.4451	910.3904	-0.0547	-60	179	185	AVYECLR			Carbamidomethyl (C)[5]		Mascot
914.4049	914.3474	-0.0575	-63	297	303	NHGMHFR			Oxidation (M)[4]		Mascot
1021.5312	1021.4674	-0.0638	-62	24	32	DTDILAAFR					Mascot
1039.4691	1039.4707	0.0016	2	342	349	DDYVEKDR					Mascot
1116.583	1116.5176	-0.0654	-59	413	422	VALEACVQAR			Carbamidomethyl (C)[6]		Mascot
1170.5585	1170.571	0.0125	11	295	303	QKNHGMHFR			Oxidation (M)[6]		Mascot
1187.6643	1187.5975	-0.0668	-56	277	286	DNGLLLHIHR					Mascot
1275.7307	1275.6504	-0.0803	-63	331	341	EITLGFVDLLR	1	0			Mascot
1275.7307	1275.6504	-0.0803	-63	331	341	EITLGFVDLLR					Mascot
1465.7546	1465.6561	-0.0985	-67	138	150	TFQGPPHGIQVER	7	0			Mascot
1465.7546	1465.6561	-0.0985	-67	138	150	TFQGPPHGIQVER					Mascot
1502.8512	1502.7539	-0.0973	-65	156	168	YGRPLLGCTIKPK			Carbamidomethyl (C)[8]		Mascot
1961.9425	1961.7662	-0.1763	-90	438	454	EASKWSPELAAACEVWK			Carbamidomethyl (C)[13]		Mascot
1984.8673	1984.8652	-0.0021	-1	228	244	GHYLNATAGTCEEMMKR			Carbamidomethyl (C)[11], Oxidation (M)[14]		Mascot

7	Ribulose bisphosphate carboxylase large chain (Fragment) OS=Geum quellyon GN=rbcL PE=3 SV=1	RBL_GEUQU	50910.8	6.23	12	63	98.234	6	0

Peptide Information

Calc. Mass	Obsrv. Mass	± da	± ppm	Start Seq.	End Seq.	Sequence	Ion Score	C. I. %	Modification	Rank	Result Type
805.4929	805.4553	-0.0376	-47	1	8	VGFKAGVK					Mascot
910.4451	910.3904	-0.0547	-60	178	184	AVYECLR			Carbamidomethyl (C)[5]		Mascot
914.4049	914.3474	-0.0575	-63	296	302	NHGMHFR			Oxidation (M)[4]		Mascot
1021.5312	1021.4674	-0.0638	-62	23	31	DTDILAAFR					Mascot
1116.583	1116.5176	-0.0654	-59	412	421	VALEACVQAR			Carbamidomethyl (C)[6]		Mascot
1170.5585	1170.571	0.0125	11	294	302	QKNHGMHFR			Oxidation (M)[6]		Mascot
1187.6643	1187.5975	-0.0668	-56	276	285	DNGLLLHIHR					Mascot
1275.7307	1275.6504	-0.0803	-63	330	340	EITLGFVDLLR	1	0			Mascot
1275.7307	1275.6504	-0.0803	-63	330	340	EITLGFVDLLR					Mascot
1465.7216	1465.6561	-0.0655	-45	310	324	MSGGDHIHAGTVVGK					Mascot
1465.7546	1465.6561	-0.0985	-67	137	149	TFQGPPHGIQVER	7	0			Mascot
1502.8512	1502.7539	-0.0973	-65	155	167	YGRPLLGCTIKPK			Carbamidomethyl (C)[8]		Mascot
1984.8673	1984.8652	-0.0021	-1	227	243	GHYLNATAGTCEEMMKR			Carbamidomethyl (C)[11], Oxidation (M)[14]		Mascot

8	Ribulose bisphosphate carboxylase large chain (Fragment) OS=Prunus domestica GN=rbcL PE=3 SV=1	RBL_PRUDO	50914.6	6.23	12	63	98.234	6	0

Peptide Information

Calc. Mass	Obsrv. Mass	± da	± ppm	Start Seq.	End Seq.	Sequence	Ion Score	C. I. %	Modification	Rank	Result Type
805.4929	805.4553	-0.0376	-47	1	8	VGFKAGVK					Mascot
910.4451	910.3904	-0.0547	-60	178	184	AVYECLR			Carbamidomethyl (C)[5]		Mascot
914.4049	914.3474	-0.0575	-63	296	302	NHGMHFR			Oxidation (M)[4]		Mascot
1021.5312	1021.4674	-0.0638	-62	23	31	DTDILAAFR					Mascot
1116.583	1116.5176	-0.0654	-59	412	421	VALEACVQAR			Carbamidomethyl (C)[6]		Mascot
1170.5585	1170.571	0.0125	11	294	302	QKNHGMHFR			Oxidation (M)[6]		Mascot
1187.6643	1187.5975	-0.0668	-56	276	285	DNGLLLHIHR					Mascot
1275.7307	1275.6504	-0.0803	-63	330	340	EITLGFVDLLR	1	0			Mascot
1275.7307	1275.6504	-0.0803	-63	330	340	EITLGFVDLLR					Mascot
1465.7216	1465.6561	-0.0655	-45	310	324	MSGGDHIHAGTVVGK					Mascot
1465.7546	1465.6561	-0.0985	-67	137	149	TFQGPPHGIQVER	7	0			Mascot
1502.8512	1502.7539	-0.0973	-65	155	167	YGRPLLGCTIKPK			Carbamidomethyl (C)[8]		Mascot
1961.9425	1961.7662	-0.1763	-90	437	453	EASKWSPELAAACEVWK			Carbamidomethyl (C)[13]		Mascot

9	Ribulose bisphosphate carboxylase large chain (Fragment) OS=Nyssa ogeche GN=rbcL PE=3 SV=2	RBL_NYSOG	51012.7	6.13	12	63	98.193	6	0

Peptide Information

Calc. Mass	Obsrv. Mass	± da	± ppm	Start Seq.	End Seq.	Sequence	Ion Score	C. I. %	Modification	Rank	Result Type
805.4929	805.4553	-0.0376	-47	1	8	VGFKAGVK					Mascot
910.4451	910.3904	-0.0547	-60	178	184	AVYECLR			Carbamidomethyl (C)[5]		Mascot
914.4049	914.3474	-0.0575	-63	296	302	NHGMHFR			Oxidation (M)[4]		Mascot
1021.5312	1021.4674	-0.0638	-62	23	31	DTDILAAFR					Mascot
1116.583	1116.5176	-0.0654	-59	412	421	VALEACVQAR			Carbamidomethyl (C)[6]		Mascot
1170.5585	1170.571	0.0125	11	294	302	QKNHGMHFR			Oxidation (M)[6]		Mascot
1187.6643	1187.5975	-0.0668	-56	276	285	DNGLLLHIHR					Mascot
1275.7307	1275.6504	-0.0803	-63	330	340	EITLGFVDLLR	1	0			Mascot
1275.7307	1275.6504	-0.0803	-63	330	340	EITLGFVDLLR					Mascot
1465.7216	1465.6561	-0.0655	-45	310	324	MSGGDHIHAGTVVGK					Mascot
1465.7546	1465.6561	-0.0985	-67	137	149	TFQGPPHGIQVER	7	0			Mascot
1502.8512	1502.7539	-0.0973	-65	155	167	YGRPLLGCTIKPK			Carbamidomethyl (C)[8]		Mascot
1560.7515	1560.6594	-0.0921	-59	441	453	WSPELAAACEIWK			Carbamidomethyl (C)[9]		Mascot

10	Ribulose bisphosphate carboxylase large chain OS=Cucumis sativus GN=rbcL PE=1 SV=5	RBL_CUCSA	52608.4	6	13	63	98.151	6	0

Peptide Information

Calc. Mass	Obsrv. Mass	± da	± ppm	Start Seq.	End Seq.	Sequence	Ion Score	C. I. %	Modification	Rank	Result Type
805.4818	805.4553	-0.0265	-33	140	146	IPTAYIK					Mascot
910.4451	910.3904	-0.0547	-60	188	194	AVYECLR			Carbamidomethyl (C)[5]		Mascot
914.4049	914.3474	-0.0575	-63	306	312	NHGMHFR			Oxidation (M)[4]		Mascot
1021.5312	1021.4674	-0.0638	-62	33	41	DTDILAAFR					Mascot
1116.583	1116.5176	-0.0654	-59	422	431	VALEACVQAR			Carbamidomethyl (C)[6]		Mascot
1170.5585	1170.571	0.0125	11	304	312	QKNHGMHFR			Oxidation (M)[6]		Mascot
1187.6643	1187.5975	-0.0668	-56	286	295	DNGLLLHIHR					Mascot
1275.7307	1275.6504	-0.0803	-63	340	350	EITLGFVDLLR	1	0			Mascot
1275.7307	1275.6504	-0.0803	-63	340	350	EITLGFVDLLR					Mascot
1465.7216	1465.6561	-0.0655	-45	320	334	MSGGDHIHAGTVVGK					Mascot
1465.7546	1465.6561	-0.0985	-67	147	159	TFQGPPHGIQVER	7	0			Mascot
1502.8512	1502.7539	-0.0973	-65	165	177	YGRPLLGCTIKPK			Carbamidomethyl (C)[8]		Mascot
1961.9425	1961.7662	-0.1763	-90	447	463	EASKWSPELAAACEVWK			Carbamidomethyl (C)[13]		Mascot
1984.8673	1984.8652	-0.0021	-1	237	253	GHYLNATAGTCEEMMKR			Carbamidomethyl (C)[11], Oxidation (M)[14]		Mascot

Gel Idx/Pos	96/D21	Instr./Gel Origin	BA2060/131017A	Process Sta	Analysis Succeeded
Plate [#] Name	[1] 31496	Instrument Sample Name		Spectra	8

Rank	Protein Name	Accession No	Protein MW	Protein PI	Pep. Count	Protein Score	Protein Score C. I. %	Total Ion Score	Total Ion C. I. %
1	Ribulose bisphosphate carboxylase large chain (Fragment) OS=Manilkara zapota GN=rbcL PE=3 SV=2	RBL_MANZA	51550	6.04	10	77	99.933	33	94.923

Peptide Information

Calc. Mass	Obsrv. Mass	± da	± ppm	Start Seq.	End Seq.	Sequence	Ion Score	C. I. %	Modification	Rank	Result Type
898.41	898.3665	-0.0435	-48	296	302	NHGMHFR					Mascot
914.4049	914.3593	-0.0456	-50	296	302	NHGMHFR			Oxidation (M)[4]		Mascot
1039.4691	1039.474	0.0049	5	341	348	DDYVEKDR					Mascot
1116.583	1116.5302	-0.0528	-47	412	421	VALEACVQAR			Carbamidomethyl (C)[6]		Mascot
1170.5585	1170.5773	0.0188	16	294	302	QKNHGMHFR			Oxidation (M)[6]		Mascot
1187.6643	1187.6027	-0.0616	-52	276	285	DNGLLLHIHR					Mascot
1275.7307	1275.6674	-0.0633	-50	330	340	EITLGFVDLLR	33	94.923			Mascot
1275.7307	1275.6674	-0.0633	-50	330	340	EITLGFVDLLR					Mascot
1546.7358	1546.6567	-0.0791	-51	441	453	WSPELAAACEVWK			Carbamidomethyl (C)[9]		Mascot
1793.8024	1793.7729	-0.0295	-16	192	205	DDENVNSQPFMRWR					Mascot
1794.8149	1794.7448	-0.0701	-39	227	242	GHYLNATAGTCEEMIK			Carbamidomethyl (C)[11]		Mascot
3022.4548	3022.2896	-0.1652	-55	249	275	ELGVPIVMHDYLTGGFTA NTSLAHYCR			Carbamidomethyl (C)[26]		Mascot

Rank	Protein Name	Accession No	Protein MW	Protein PI	Pep. Count	Protein Score	Protein Score C. I. %	Total Ion Score	Total Ion C. I. %
2	Ribulose bisphosphate carboxylase large chain (Fragment) OS=Couroupita guianensis GN=rbcL PE=3 SV=1	RBL_COUGU	51807.1	6.46	10	76	99.919	33	94.923

Peptide Information

Calc. Mass	Obsrv. Mass	± da	± ppm	Start Seq.	End Seq.	Sequence	Ion Score	C. I. %	Modification	Rank	Result Type
898.41	898.3665	-0.0435	-48	299	305	NHGMHFR					Mascot
914.4049	914.3593	-0.0456	-50	299	305	NHGMHFR			Oxidation (M)[4]		Mascot
1116.583	1116.5302	-0.0528	-47	415	424	VALEACVQAR			Carbamidomethyl (C)[6]		Mascot
1170.5585	1170.5773	0.0188	16	297	305	QKNHGMHFR			Oxidation (M)[6]		Mascot
1187.6378	1187.6027	-0.0351	-30	433	443	EGNEIIRAASK					Mascot
1275.7307	1275.6674	-0.0633	-50	333	343	EITLGFVDLLR	33	94.923			Mascot
1275.7307	1275.6674	-0.0633	-50	333	343	EITLGFVDLLR					Mascot
1546.7358	1546.6567	-0.0791	-51	444	456	WSPELAAACEVWK			Carbamidomethyl (C)[9]		Mascot
1793.8024	1793.7729	-0.0295	-16	195	208	DDENVNSQPFMRWR					Mascot
1891.8789	1891.9324	0.0535	28	230	246	GHYLNAPAGTSEEMMKR					Mascot
2663.2439	2663.1528	-0.0911	-34	221	245	AQAETGEIKGHYLNAPAG TSEEMMK					Mascot
3022.4548	3022.2896	-0.1652	-55	252	278	ELGVPIVMHDYLTGGFTA NTSLAHYCR			Carbamidomethyl (C)[26]		Mascot

Rank	Protein Name	Accession No	Protein MW	Protein PI	Pep. Count	Protein Score	Protein Score C. I. %	Total Ion Score	Total Ion C. I. %
3	Ribulose bisphosphate carboxylase large chain OS=Cerastium glomeratum GN=rbcL PE=3 SV=1	RBL_CERGL	52632.5	6.22	10	76	99.905	33	94.923

Peptide Information

Calc. Mass	Obsrv. Mass	± da	± ppm	Start Seq.	End Seq.	Sequence	Ion Score	C. I. %	Modification	Rank	Result Type
898.41	898.3665	-0.0435	-48	306	312	NHGMHFR					Mascot
914.4049	914.3593	-0.0456	-50	306	312	NHGMHFR			Oxidation (M)[4]		Mascot
1116.583	1116.5302	-0.0528	-47	422	431	VALEACVQAR			Carbamidomethyl (C)[6]		Mascot
1170.5585	1170.5773	0.0188	16	304	312	QKNHGMHFR			Oxidation (M)[6]		Mascot
1187.6643	1187.6027	-0.0616	-52	286	295	DNGLLLHIHR					Mascot
1275.7307	1275.6674	-0.0633	-50	340	350	EITLGFVDLLR	33	94.923			Mascot
1275.7307	1275.6674	-0.0633	-50	340	350	EITLGFVDLLR					Mascot
1395.7114	1395.667	-0.0444	-32	2	14	SPQTETKSSVGFK					Mascot
1546.7358	1546.6567	-0.0791	-51	451	463	WSPELAAACEVWK			Carbamidomethyl (C)[9]		Mascot
1793.8024	1793.7729	-0.0295	-16	202	215	DDENVNSQPFMRWR					Mascot
1794.8149	1794.7448	-0.0701	-39	237	252	GHYLNATAGTCEEMIK			Carbamidomethyl (C)[11]		Mascot
3022.4548	3022.2896	-0.1652	-55	259	285	ELGVPIVMHDYITGGFTA NTSLAHYCR			Carbamidomethyl (C)[26]		Mascot

Rank	Protein Name	Accession No	Protein MW	Protein PI	Pep. Count	Protein Score	Protein Score C. I. %	Total Ion Score	Total Ion C. I. %
4	Ribulose bisphosphate carboxylase large chain (Fragment) OS=Saururus cernuus GN=rbcL PE=3 SV=1	RBL_SAUCE	51663	6	10	75	99.898	33	94.923

Peptide Information

Calc. Mass	Obsrv. Mass	± da	± ppm	Start Seq.	End Seq.	Sequence	Ion Score	C. I. %	Modification	Rank	Result Type
898.41	898.3665	-0.0435	-48	297	303	NHGMHFR					Mascot
914.4049	914.3593	-0.0456	-50	297	303	NHGMHFR			Oxidation (M)[4]		Mascot
1116.583	1116.5302	-0.0528	-47	413	422	VALEACVQAR			Carbamidomethyl (C)[6]		Mascot
1170.5585	1170.5773	0.0188	16	295	303	QKNHGMHFR			Oxidation (M)[6]		Mascot
1187.6643	1187.6027	-0.0616	-52	277	286	DNGLLLHIHR					Mascot
1229.6484	1229.5594	-0.089	-72	431	441	EGNEIIREAAK					Mascot
1229.6484	1229.5594	-0.089	-72	431	441	EGNEIIREAAK					Mascot
1275.7307	1275.6674	-0.0633	-50	331	341	EITLGFVDLLR	33	94.923			Mascot
1275.7307	1275.6674	-0.0633	-50	331	341	EITLGFVDLLR					Mascot
1546.7358	1546.6567	-0.0791	-51	442	454	WSPELAAACEVWK			Carbamidomethyl (C)[9]		Mascot
1793.8024	1793.7729	-0.0295	-16	193	206	DDENVNSQPFMRWR					Mascot
1794.8149	1794.7448	-0.0701	-39	228	243	GHYLNATAGTCEEMIK			Carbamidomethyl (C)[11]		Mascot
3022.4548	3022.2896	-0.1652	-55	250	276	ELGVPIVMHDYLTGGFTA NTSLAHYCR			Carbamidomethyl (C)[26]		Mascot

Rank	Protein Name	Accession No	Protein MW	Protein PI	Pep. Count	Protein Score	Protein Score C. I. %	Total Ion Score	Total Ion C. I. %
5	Ribulose bisphosphate carboxylase large chain OS=Calycanthus floridus var. glaucus GN=rbcL PE=3 SV=	RBL_CALFG	52571.5	6.23	10	75	99.894	33	94.923

Peptide Information

Calc. Mass	Obsrv. Mass	± da	± ppm	Start Seq.	End Seq.	Sequence	Ion Score	C. I. %	Modification	Rank	Result Type
898.41	898.3665	-0.0435	-48	306	312	NHGMHFR					Mascot
914.4049	914.3593	-0.0456	-50	306	312	NHGMHFR			Oxidation (M)[4]		Mascot
933.5515	933.468	-0.0835	-89	9	18	AGVGFKAGVK					Mascot
1116.583	1116.5302	-0.0528	-47	422	431	VALEACVQAR			Carbamidomethyl (C)[6]		Mascot
1170.5585	1170.5773	0.0188	16	304	312	QKNHGMHFR			Oxidation (M)[6]		Mascot
1187.6643	1187.6027	-0.0616	-52	286	295	DNGLLLHIHR					Mascot
1275.7307	1275.6674	-0.0633	-50	340	350	EITLGFVDLLR	33	94.923			Mascot
1275.7307	1275.6674	-0.0633	-50	340	350	EITLGFVDLLR					Mascot
1546.7358	1546.6567	-0.0791	-51	451	463	WSPELAAACEVWK			Carbamidomethyl (C)[9]		Mascot
1793.8024	1793.7729	-0.0295	-16	202	215	DDENVNSQPFMRWR					Mascot
1794.8149	1794.7448	-0.0701	-39	237	252	GHYLNATAGTCEEMIK			Carbamidomethyl (C)[11]		Mascot
3022.4548	3022.2896	-0.1652	-55	259	285	ELGVPIVMHDYLTGGFTA NTSLAHYCR			Carbamidomethyl (C)[26]		Mascot

Rank	Protein Name	Accession No	Protein MW	Protein PI	Pep. Count	Protein Score	Protein Score C. I. %	Total Ion Score	Total Ion C. I. %
6	Ribulose bisphosphate carboxylase large chain OS=Bartlettina sordida GN=rbcL PE=3 SV=1	RBL_BARSO	53814.9	6	11	73	99.846	33	94.923

Peptide Information

Calc. Mass	Obsrv. Mass	± da	± ppm	Start Seq.	End Seq.	Sequence	Ion Score	C. I. %	Modification	Rank	Result Type
898.41	898.3665	-0.0435	-48	306	312	NHGMHFR					Mascot
914.3858	914.3593	-0.0265	-29	188	194	ACYECLR			Carbamidomethyl (C)[2]		Mascot
1116.583	1116.5302	-0.0528	-47	422	431	VALEACVQAR			Carbamidomethyl (C)[6]		Mascot
1170.5585	1170.5773	0.0188	16	304	312	QKNHGMHFR			Oxidation (M)[6]		Mascot
1187.6643	1187.6027	-0.0616	-52	286	295	DNGLLLHIHR					Mascot
1275.7307	1275.6674	-0.0633	-50	340	350	EITLGFVDLLR	33	94.923			Mascot
1275.7307	1275.6674	-0.0633	-50	340	350	EITLGFVDLLR					Mascot
1546.7358	1546.6567	-0.0791	-51	451	463	WSPELAAACEVWK			Carbamidomethyl (C)[9]		Mascot
1689.7723	1689.6698	-0.1025	-61	188	201	ACYECLRGGLDFTK			Carbamidomethyl (C)[2,5]		Mascot
1793.8024	1793.7729	-0.0295	-16	202	215	DDENVNSQPFMRWR					Mascot
2225.1714	2224.9934	-0.178	-80	140	159	IPTAYVKTFDGPPHGIQV ER					Mascot
3022.4548	3022.2896	-0.1652	-55	259	285	ELGVPIVMHDYLTGGFTA NTSLAHYCR			Carbamidomethyl (C)[26]		Mascot

Rank	Protein Name	Accession No	Protein MW	Protein PI	Pep. Count	Protein Score	Protein Score C. I. %	Total Ion Score	Total Ion C. I. %
7	Ribulose bisphosphate carboxylase large chain OS=Sesbania sesban GN=rbcL PE=3 SV=1	RBL_SESSE	52358.4	6.09	10	73	99.843	33	94.923

Peptide Information

Calc. Mass	Obsrv. Mass	± da	± ppm	Start Seq.	End Seq.	Sequence	Ion Score	C. I. %	Modification	Rank	Result Type
898.41	898.3665	-0.0435	-48	306	312	NHGMHFR					Mascot
914.4049	914.3593	-0.0456	-50	306	312	NHGMHFR			Oxidation (M)[4]		Mascot
1039.4691	1039.474	0.0049	5	351	358	DDYVEKDR					Mascot
1116.583	1116.5302	-0.0528	-47	422	431	VALEACVQAR			Carbamidomethyl (C)[6]		Mascot
1170.5585	1170.5773	0.0188	16	304	312	QKNHGMHFR			Oxidation (M)[6]		Mascot
1187.6643	1187.6027	-0.0616	-52	286	295	DNGLLLHIHR					Mascot
1275.7307	1275.6674	-0.0633	-50	340	350	EITLGFVDLLR	33	94.923			Mascot
1275.7307	1275.6674	-0.0633	-50	340	350	EITLGFVDLLR					Mascot
1546.7358	1546.6567	-0.0791	-51	451	463	WSPELAAACEVWK			Carbamidomethyl (C)[9]		Mascot
1793.8024	1793.7729	-0.0295	-16	202	215	DDENVNSQPFMRWR					Mascot
1794.8149	1794.7448	-0.0701	-39	237	252	GHYLNATAGTCEEMIK			Carbamidomethyl (C)[11]		Mascot
3022.4548	3022.2896	-0.1652	-55	259	285	ELGVPIVMHDYLTGGFTA NTSLAHYCR			Carbamidomethyl (C)[26]		Mascot

Rank	Protein Name	Accession No	Protein MW	Protein PI	Pep. Count	Protein Score	Protein Score C. I. %	Total Ion Score	Total Ion C. I. %
8	Ribulose bisphosphate carboxylase large chain OS=Guizotia abyssinica GN=rbcL PE=3 SV=1	RBL_GUIAB	53825	6	11	73	99.815	33	94.923

Peptide Information

Calc. Mass	Obsrv. Mass	± da	± ppm	Start Seq.	End Seq.	Sequence	Ion Score	C. I. %	Modification	Rank	Result Type
898.41	898.3665	-0.0435	-48	306	312	NHGMHFR					Mascot
914.3858	914.3593	-0.0265	-29	188	194	ACYECLR			Carbamidomethyl (C)[2]		Mascot
1116.583	1116.5302	-0.0528	-47	422	431	VALEACVQAR			Carbamidomethyl (C)[6]		Mascot
1170.5585	1170.5773	0.0188	16	304	312	QKNHGMHFR			Oxidation (M)[6]		Mascot
1187.6643	1187.6027	-0.0616	-52	286	295	DNGLLLHIHR					Mascot
1275.7307	1275.6674	-0.0633	-50	340	350	EITLGFVDLLR	33	94.923			Mascot
1275.7307	1275.6674	-0.0633	-50	340	350	EITLGFVDLLR					Mascot
1546.7358	1546.6567	-0.0791	-51	451	463	WSPELAAACEVWK			Carbamidomethyl (C)[9]		Mascot
1689.7723	1689.6698	-0.1025	-61	188	201	ACYECLRGGLDFTK			Carbamidomethyl (C)[2,5]		Mascot
1793.8024	1793.7729	-0.0295	-16	202	215	DDENVNSQPFMRWR					Mascot
1794.8149	1794.7448	-0.0701	-39	237	252	GHYLNATAGTCEEMLK			Carbamidomethyl (C)[11]		Mascot
2225.1714	2224.9934	-0.178	-80	140	159	IPTAYVKTFDGPPHGIQV ER					Mascot

Rank	Protein Name	Accession No	Protein MW	Protein PI	Pep. Count	Protein Score	Protein Score C. I. %	Total Ion Score	Total Ion C. I. %
9	Ribulose bisphosphate carboxylase large chain OS=Ostrya virginiana GN=rbcL PE=3 SV=1	RBL_OSTVI	52442.4	6.16	10	73	99.815	33	94.923

Peptide Information

Calc. Mass	Obsrv. Mass	± da	± ppm	Start Seq.	End Seq.	Sequence	Ion Score	C. I. %	Modification	Rank	Result Type
898.41	898.3665	-0.0435	-48	306	312	NHGMHFR					Mascot
914.4049	914.3593	-0.0456	-50	306	312	NHGMHFR			Oxidation (M)[4]		Mascot
1116.583	1116.5302	-0.0528	-47	422	431	VALEACVQAR			Carbamidomethyl (C)[6]		Mascot
1170.5585	1170.5773	0.0188	16	304	312	QKNHGMHFR			Oxidation (M)[6]		Mascot
1187.6643	1187.6027	-0.0616	-52	286	295	DNGLLLHIHR					Mascot
1275.7307	1275.6674	-0.0633	-50	340	350	EITLGFVDLLR	33	94.923			Mascot
1275.7307	1275.6674	-0.0633	-50	340	350	EITLGFVDLLR					Mascot
1546.7358	1546.6567	-0.0791	-51	451	463	WSPELAAACEVWK			Carbamidomethyl (C)[9]		Mascot
1688.802	1688.6819	-0.1201	-71	432	446	NEGRDLACEGNEIIR					Mascot
1793.8024	1793.7729	-0.0295	-16	202	215	DDENVNSQPFMRWR					Mascot
1794.8149	1794.7448	-0.0701	-39	237	252	GHYLNATAGTCEEMIK			Carbamidomethyl (C)[11]		Mascot
3022.4548	3022.2896	-0.1652	-55	259	285	ELGVPIVMHDYLTGGFTA NTSLAHYCR			Carbamidomethyl (C)[26]		Mascot

Rank	Protein Name	Accession No	Protein MW	Protein PI	Pep. Count	Protein Score	Protein Score C. I. %	Total Ion Score	Total Ion C. I. %
10	Ribulose bisphosphate carboxylase large chain OS=Flaveria pringlei GN=rbcL PE=3 SV=1	RBL_FLAPR	53841	6	11	73	99.815	33	94.923

Peptide Information

Calc. Mass	Obsrv. Mass	± da	± ppm	Start Seq.	End Seq.	Sequence	Ion Score	C. I. %	Modification	Rank	Result Type
898.41	898.3665	-0.0435	-48	306	312	NHGMHFR					Mascot
914.3858	914.3593	-0.0265	-29	188	194	ACYECLR			Carbamidomethyl (C)[2]		Mascot
1116.583	1116.5302	-0.0528	-47	422	431	VALEACVQAR			Carbamidomethyl (C)[6]		Mascot
1170.5585	1170.5773	0.0188	16	304	312	QKNHGMHFR			Oxidation (M)[6]		Mascot
1187.6643	1187.6027	-0.0616	-52	286	295	DNGLLLHIHR					Mascot
1275.7307	1275.6674	-0.0633	-50	340	350	EITLGFVDLLR	33	94.923			Mascot
1275.7307	1275.6674	-0.0633	-50	340	350	EITLGFVDLLR					Mascot
1546.7358	1546.6567	-0.0791	-51	451	463	WSPELAAACEVWK			Carbamidomethyl (C)[9]		Mascot
1689.7723	1689.6698	-0.1025	-61	188	201	ACYECLRGGLDFTK			Carbamidomethyl (C)[2,5]		Mascot
1793.8024	1793.7729	-0.0295	-16	202	215	DDENVNSQPFMRWR					Mascot
1794.8149	1794.7448	-0.0701	-39	237	252	GHYLNATAGTCEEMLK			Carbamidomethyl (C)[11]		Mascot
2239.187	2239.0073	-0.1797	-80	140	159	IPTAYVKTFEGPPHGIQV ER					Mascot

Gel Idx/Pos	97/D22	Instr./Gel Origin	BA2060/131017A	Process Sta	Analysis Succeeded
Plate [#] Name	[1] 31496	Instrument Sample Name		Spectra	8

Rank	Protein Name	Accession No	Protein MW	Protein PI	Pep. Count	Protein Score	Protein Score C. I. %	Total Ion Score	Total Ion C. I. %
1	Ribulose bisphosphate carboxylase large chain OS=Glycine max GN=rbcL PE=1 SV=3	RBL_SOYBN	52576.4	6	20	630	100	484	100

Peptide Information

Calc. Mass	Obsrv. Mass	± da	± ppm	Start Seq.	End Seq.	Sequence	Ion Score	C. I. %	Modification	Rank	Result Type
830.4366	830.4017	-0.0349	-42	440	446	EGNEIIR					Mascot
898.41	898.371	-0.039	-43	306	312	NHGMHFR					Mascot
910.4451	910.4059	-0.0392	-43	188	194	AVYECLR	10	0	Carbamidomethyl (C)[5]		Mascot
910.4451	910.4059	-0.0392	-43	188	194	AVYECLR			Carbamidomethyl (C)[5]		Mascot
912.4719	912.4152	-0.0567	-62	296	303	AMHAVIDR					Mascot
914.4049	914.3646	-0.0403	-44	306	312	NHGMHFR			Oxidation (M)[4]		Mascot
928.4669	928.4258	-0.0411	-44	296	303	AMHAVIDR			Oxidation (M)[2]		Mascot
962.4788	962.4354	-0.0434	-45	228	236	SQAETGEIK					Mascot
1023.4741	1023.4259	-0.0482	-47	351	358	DDFVEKDR					Mascot
1116.583	1116.5344	-0.0486	-44	422	431	VALEACVQAR			Carbamidomethyl (C)[6]		Mascot
1116.583	1116.5344	-0.0486	-44	422	431	VALEACVQAR	51	99.894	Carbamidomethyl (C)[6]		Mascot
1154.5636	1154.5364	-0.0272	-24	304	312	QKNHGMHFR					Mascot
1170.5585	1170.5868	0.0283	24	304	312	QKNHGMHFR			Oxidation (M)[6]		Mascot
1170.5585	1170.5868	0.0283	24	304	312	QKNHGMHFR			Oxidation (M)[6]		Mascot
1187.6643	1187.6113	-0.053	-45	286	295	DNGLLLHIHR					Mascot
1245.6337	1245.5771	-0.0566	-45	218	227	FLFCAEAIFK	63	99.994	Carbamidomethyl (C)[4]		Mascot
1245.6337	1245.5771	-0.0566	-45	218	227	FLFCAEAIFK			Carbamidomethyl (C)[4]		Mascot
1275.7307	1275.6761	-0.0546	-43	340	350	EITLGFVDLLR	95	100			Mascot
1275.7307	1275.6761	-0.0546	-43	340	350	EITLGFVDLLR					Mascot
1433.7495	1433.6823	-0.0672	-47	320	334	LSGGDHVHAGTVVGK					Mascot
1451.6219	1451.5577	-0.0642	-44	202	213	DDENVNSQPFMR					Mascot
1467.6168	1467.5497	-0.0671	-46	202	213	DDENVNSQPFMR			Oxidation (M)[11]		Mascot
1472.6978	1472.6229	-0.0749	-51	464	475	EIKFEFEAMDTL					Mascot
1488.6926	1488.6216	-0.071	-48	464	475	EIKFEFEAMDTL			Oxidation (M)[9]		Mascot
1546.7358	1546.6625	-0.0733	-47	451	463	WSPELAAACEVWK			Carbamidomethyl (C)[9]		Mascot
1812.7714	1812.6842	-0.0872	-48	237	252	GHYLNATAGTCEEMMK			Carbamidomethyl (C)[11]		Mascot
1828.7662	1828.6816	-0.0846	-46	237	252	GHYLNATAGTCEEMMK			Carbamidomethyl (C)[11], Oxidation (M)[14]		Mascot
1844.7611	1844.6737	-0.0874	-47	237	252	GHYLNATAGTCEEMMK			Carbamidomethyl (C)[11], Oxidation (M)[14,15]		Mascot
1984.8673	1984.7748	-0.0925	-47	237	253	GHYLNATAGTCEEMMKR			Carbamidomethyl (C)[11], Oxidation (M)[14]		Mascot
2000.8623	2000.7628	-0.0995	-50	237	253	GHYLNATAGTCEEMMKR			Carbamidomethyl (C)[11], Oxidation (M)[14,15]		Mascot
2009.059	2008.9618	-0.0972	-48	340	356	EITLGFVDLLRDDFVEK					Mascot
2169.9868	2169.8826	-0.1042	-48	195	213	GGLDFTKDDENVNSQPFMR					Mascot
2169.9868	2169.8826	-0.1042	-48	195	213	GGLDFTKDDENVNSQPFMR	122	100			Mascot
2185.9819	2185.8743	-0.1076	-49	195	213	GGLDFTKDDENVNSQPFMR			Oxidation (M)[18]		Mascot
3022.4548	3022.3018	-0.153	-51	259	285	ELGVPIVMHDYLTGGFTANTSLAHYCR			Carbamidomethyl (C)[26]		Mascot
3022.4548	3022.3018	-0.153	-51	259	285	ELGVPIVMHDYLTGGFTANTSLAHYCR	144	100	Carbamidomethyl (C)[26]		Mascot
3038.45	3038.3064	-0.1436	-47	259	285	ELGVPIVMHDYLTGGFTANTSLAHYCR			Carbamidomethyl (C)[26], Oxidation (M)[8]		Mascot

Rank	Protein Name	Accession No	Protein MW	Protein PI	Pep. Count	Protein Score	Protein Score C. I. %	Total Ion Score	Total Ion C. I. %
2	Ribulose bisphosphate carboxylase large chain (Fragment) OS=Ilex crenata GN=rbcL PE=3 SV=1	RBL_ILECR	51518	6.1	18	615	100	484	100

Peptide Information

Calc. Mass	Obsrv. Mass	± da	± ppm	Start Seq.	End Seq.	Sequence	Ion Score	C. I. %	Modification	Rank	Result Type
830.4366	830.4017	-0.0349	-42	430	436	EGNEIIR					Mascot
898.41	898.371	-0.039	-43	296	302	NHGMHFR					Mascot
910.4451	910.4059	-0.0392	-43	178	184	AVYECLR	10	0	Carbamidomethyl (C)[5]		Mascot
910.4451	910.4059	-0.0392	-43	178	184	AVYECLR			Carbamidomethyl (C)[5]		Mascot
912.4719	912.4152	-0.0567	-62	286	293	AMHAVIDR					Mascot
914.4049	914.3646	-0.0403	-44	296	302	NHGMHFR			Oxidation (M)[4]		Mascot
928.4669	928.4258	-0.0411	-44	286	293	AMHAVIDR			Oxidation (M)[2]		Mascot
1023.4741	1023.4259	-0.0482	-47	341	348	DDFVEKDR					Mascot
1116.6194	1116.5344	-0.085	-76	412	421	VALEACVKAR			Carbamidomethyl (C)[6]		Mascot
1116.6194	1116.5344	-0.085	-76	412	421	VALEACVKAR	51	99.894	Carbamidomethyl (C)[6]		Mascot
1154.5636	1154.5364	-0.0272	-24	294	302	QKNHGMHFR					Mascot
1170.5585	1170.5868	0.0283	24	294	302	QKNHGMHFR			Oxidation (M)[6]		Mascot
1170.5585	1170.5868	0.0283	24	294	302	QKNHGMHFR			Oxidation (M)[6]		Mascot
1187.6643	1187.6113	-0.053	-45	276	285	DNGLLLHIHR					Mascot
1245.6337	1245.5771	-0.0566	-45	208	217	FLFCAEALFK	63	99.994	Carbamidomethyl (C)[4]		Mascot
1245.6337	1245.5771	-0.0566	-45	208	217	FLFCAEALFK			Carbamidomethyl (C)[4]		Mascot
1275.7307	1275.6761	-0.0546	-43	330	340	EITLGFVDLLR	95	100			Mascot
1275.7307	1275.6761	-0.0546	-43	330	340	EITLGFVDLLR					Mascot
1451.6219	1451.5577	-0.0642	-44	192	203	DDENVNSQPFMR					Mascot
1467.6168	1467.5497	-0.0671	-46	192	203	DDENVNSQPFMR			Oxidation (M)[11]		Mascot
1546.7358	1546.6625	-0.0733	-47	441	453	WSPELAAACEVWK			Carbamidomethyl (C)[9]		Mascot
1794.8149	1794.7372	-0.0777	-43	227	242	GHYLNATAGTCEEMIK			Carbamidomethyl (C)[11]		Mascot
1812.8326	1812.6842	-0.1484	-82	9	22	EYKLNYYTPDYDTK					Mascot
2009.059	2008.9618	-0.0972	-48	330	346	EITLGFVDLLRDDFVEK					Mascot
2050.0134	2049.9038	-0.1096	-53	310	329	MSGGDHIHAGTVVGKLEGER					Mascot
2169.9868	2169.8826	-0.1042	-48	185	203	GGLDFTKDDENVNSQPFMR					Mascot
2169.9868	2169.8826	-0.1042	-48	185	203	GGLDFTKDDENVNSQPFMR	122	100			Mascot
2185.9819	2185.8743	-0.1076	-49	185	203	GGLDFTKDDENVNSQPFMR			Oxidation (M)[18]		Mascot
3022.4548	3022.3018	-0.153	-51	249	275	ELGVPIVMHDYLTGGFTANTSLAHYCR			Carbamidomethyl (C)[26]		Mascot
3022.4548	3022.3018	-0.153	-51	249	275	ELGVPIVMHDYLTGGFTANTSLAHYCR	144	100	Carbamidomethyl (C)[26]		Mascot
3038.45	3038.3064	-0.1436	-47	249	275	ELGVPIVMHDYLTGGFTANTSLAHYCR			Carbamidomethyl (C)[26], Oxidation (M)[8]		Mascot

Rank	Protein Name	Accession No	Protein MW	Protein PI	Pep. Count	Protein Score	Protein Score C. I. %	Total Ion Score	Total Ion C. I. %
3	Ribulose bisphosphate carboxylase large chain OS=Vitis vinifera GN=rbcL PE=3 SV=2	RBL_VITVI	52485.5	6.33	17	594	100	486	100

Peptide Information

Calc. Mass	Obsrv. Mass	± da	± ppm	Start Seq.	End Seq.	Sequence	Ion Score	C. I. %	Modification	Rank	Result Type
830.4366	830.4017	-0.0349	-42	440	446	EGNEIIR					Mascot
898.41	898.371	-0.039	-43	306	312	NHGMHFR					Mascot
910.4451	910.4059	-0.0392	-43	188	194	AVYECLR	10	0	Carbamidomethyl (C)[5]		Mascot
910.4451	910.4059	-0.0392	-43	188	194	AVYECLR			Carbamidomethyl (C)[5]		Mascot
912.4719	912.4152	-0.0567	-62	296	303	AMHAVIDR					Mascot
914.4049	914.3646	-0.0403	-44	306	312	NHGMHFR			Oxidation (M)[4]		Mascot
928.4669	928.4258	-0.0411	-44	296	303	AMHAVIDR			Oxidation (M)[2]		Mascot
962.4788	962.4354	-0.0434	-45	228	236	SQAETGEIK					Mascot
1023.4741	1023.4259	-0.0482	-47	351	358	DDFVEKDR					Mascot
1116.583	1116.5344	-0.0486	-44	422	431	VALEACVQAR			Carbamidomethyl (C)[6]		Mascot
1116.583	1116.5344	-0.0486	-44	422	431	VALEACVQAR	51	99.894	Carbamidomethyl (C)[6]		Mascot
1154.5636	1154.5364	-0.0272	-24	304	312	QKNHGMHFR					Mascot
1170.5585	1170.5868	0.0283	24	304	312	QKNHGMHFR			Oxidation (M)[6]		Mascot
1170.5585	1170.5868	0.0283	24	304	312	QKNHGMHFR			Oxidation (M)[6]		Mascot
1187.6378	1187.6113	-0.0265	-22	440	450	EGNEIIRAASK					Mascot
1245.6337	1245.5771	-0.0566	-45	218	227	FLFCAEAIFK	63	99.994	Carbamidomethyl (C)[4]		Mascot
1245.6337	1245.5771	-0.0566	-45	218	227	FLFCAEAIFK			Carbamidomethyl (C)[4]		Mascot
1275.7307	1275.6761	-0.0546	-43	340	350	EITLGFVDLLR	95	100			Mascot
1275.7307	1275.6761	-0.0546	-43	340	350	EITLGFVDLLR					Mascot
1451.6219	1451.5577	-0.0642	-44	202	213	DDENVNSQPFMR					Mascot
1467.6168	1467.5497	-0.0671	-46	202	213	DDENVNSQPFMR			Oxidation (M)[11]		Mascot
1546.7358	1546.6625	-0.0733	-47	451	463	WSPELAAACEVWK			Carbamidomethyl (C)[9]		Mascot
1794.8149	1794.7372	-0.0777	-43	237	252	GHYLNATAGTCEEMIK			Carbamidomethyl (C)[11]		Mascot
2009.059	2008.9618	-0.0972	-48	340	356	EITLGFVDLLRDDFVEK					Mascot
2169.9868	2169.8826	-0.1042	-48	195	213	GGLDFTKDDENVNSQPFMR					Mascot
2169.9868	2169.8826	-0.1042	-48	195	213	GGLDFTKDDENVNSQPFMR	122	100			Mascot
2185.9819	2185.8743	-0.1076	-49	195	213	GGLDFTKDDENVNSQPFMR			Oxidation (M)[18]		Mascot
3022.4548	3022.3018	-0.153	-51	259	285	ELGVPIVMHDYLTGGFTANTSLAHYCR			Carbamidomethyl (C)[26]		Mascot
3022.4548	3022.3018	-0.153	-51	259	285	ELGVPIVMHDYLTGGFTANTSLAHYCR	144	100	Carbamidomethyl (C)[26]		Mascot
3038.45	3038.3064	-0.1436	-47	259	285	ELGVPIVMHDYLTGGFTANTSLAHYCR			Carbamidomethyl (C)[26], Oxidation (M)[8]		Mascot

Rank	Protein Name	Accession No	Protein MW	Protein PI	Pep. Count	Protein Score	Protein Score C. I. %	Total Ion Score	Total Ion C. I. %
4	Ribulose bisphosphate carboxylase large chain OS=Calycanthus floridus var. glaucus GN=rbcL PE=3 SV=	RBL_CALFG	52571.5	6.23	17	590	100	484	100

Peptide Information

Calc. Mass	Obsrv. Mass	± da	± ppm	Start Seq.	End Seq.	Sequence	Ion Score	C. I. %	Modification	Rank	Result Type
830.4366	830.4017	-0.0349	-42	440	446	EGNEIIR					Mascot
898.41	898.371	-0.039	-43	306	312	NHGMHFR					Mascot
910.4451	910.4059	-0.0392	-43	188	194	AVYECLR	10	0	Carbamidomethyl (C)[5]		Mascot
910.4451	910.4059	-0.0392	-43	188	194	AVYECLR			Carbamidomethyl (C)[5]		Mascot
912.4719	912.4152	-0.0567	-62	296	303	AMHAVIDR					Mascot
914.4049	914.3646	-0.0403	-44	306	312	NHGMHFR			Oxidation (M)[4]		Mascot
928.4669	928.4258	-0.0411	-44	296	303	AMHAVIDR			Oxidation (M)[2]		Mascot
962.4788	962.4354	-0.0434	-45	228	236	SQAETGEIK					Mascot
1116.583	1116.5344	-0.0486	-44	422	431	VALEACVQAR			Carbamidomethyl (C)[6]		Mascot
1116.583	1116.5344	-0.0486	-44	422	431	VALEACVQAR	51	99.894	Carbamidomethyl (C)[6]		Mascot
1154.5636	1154.5364	-0.0272	-24	304	312	QKNHGMHFR					Mascot
1170.5585	1170.5868	0.0283	24	304	312	QKNHGMHFR			Oxidation (M)[6]		Mascot
1170.5585	1170.5868	0.0283	24	304	312	QKNHGMHFR			Oxidation (M)[6]		Mascot
1187.6643	1187.6113	-0.053	-45	286	295	DNGLLLHIHR					Mascot
1245.6337	1245.5771	-0.0566	-45	218	227	FLFCAEAIFK	63	99.994	Carbamidomethyl (C)[4]		Mascot
1245.6337	1245.5771	-0.0566	-45	218	227	FLFCAEAIFK			Carbamidomethyl (C)[4]		Mascot
1275.7307	1275.6761	-0.0546	-43	340	350	EITLGFVDLLR	95	100			Mascot
1275.7307	1275.6761	-0.0546	-43	340	350	EITLGFVDLLR					Mascot
1451.6219	1451.5577	-0.0642	-44	202	213	DDENVNSQPFMR					Mascot
1467.6168	1467.5497	-0.0671	-46	202	213	DDENVNSQPFMR			Oxidation (M)[11]		Mascot
1472.6978	1472.6229	-0.0749	-51	464	475	EIKFEFEAMDTL					Mascot
1488.6926	1488.6216	-0.071	-48	464	475	EIKFEFEAMDTL			Oxidation (M)[9]		Mascot
1546.7358	1546.6625	-0.0733	-47	451	463	WSPELAAACEVWK			Carbamidomethyl (C)[9]		Mascot
1794.8149	1794.7372	-0.0777	-43	237	252	GHYLNATAGTCEEMIK			Carbamidomethyl (C)[11]		Mascot
2050.0134	2049.9038	-0.1096	-53	320	339	MSGGDHIHAGTVVGKLEGER					Mascot
2169.9868	2169.8826	-0.1042	-48	195	213	GGLDFTKDDENVNSQPFMR					Mascot
2169.9868	2169.8826	-0.1042	-48	195	213	GGLDFTKDDENVNSQPFMR	122	100			Mascot
2185.9819	2185.8743	-0.1076	-49	195	213	GGLDFTKDDENVNSQPFMR			Oxidation (M)[18]		Mascot
3022.4548	3022.3018	-0.153	-51	259	285	ELGVPIVMHDYLTGGFTANTSLAHYCR			Carbamidomethyl (C)[26]		Mascot
3022.4548	3022.3018	-0.153	-51	259	285	ELGVPIVMHDYLTGGFTANTSLAHYCR	144	100	Carbamidomethyl (C)[26]		Mascot
3038.45	3038.3064	-0.1436	-47	259	285	ELGVPIVMHDYLTGGFTANTSLAHYCR			Carbamidomethyl (C)[26], Oxidation (M)[8]		Mascot

Rank	Protein Name	Accession No	Protein MW	Protein PI	Pep. Count	Protein Score	Protein Score C. I. %	Total Ion Score	Total Ion C. I. %
5	Ribulose bisphosphate carboxylase large chain (Fragment) OS=Geum quellyon GN=rbcL PE=3 SV=1	RBL_GEUQU	50910.8	6.23	15	581	100	486	100

Peptide Information

Calc. Mass	Obsrv. Mass	± da	± ppm	Start Seq.	End Seq.	Sequence	Ion Score	C. I. %	Modification	Rank	Result Type
898.41	898.371	-0.039	-43	296	302	NHGMHFR					Mascot
910.4451	910.4059	-0.0392	-43	178	184	AVYECLR	10	0	Carbamidomethyl (C)[5]		Mascot
910.4451	910.4059	-0.0392	-43	178	184	AVYECLR			Carbamidomethyl (C)[5]		Mascot
912.4719	912.4152	-0.0567	-62	286	293	AMHAVIDR					Mascot
914.4049	914.3646	-0.0403	-44	296	302	NHGMHFR			Oxidation (M)[4]		Mascot
928.4669	928.4258	-0.0411	-44	286	293	AMHAVIDR			Oxidation (M)[2]		Mascot
1116.583	1116.5344	-0.0486	-44	412	421	VALEACVQAR			Carbamidomethyl (C)[6]		Mascot
1116.583	1116.5344	-0.0486	-44	412	421	VALEACVQAR	51	99.894	Carbamidomethyl (C)[6]		Mascot
1154.5636	1154.5364	-0.0272	-24	294	302	QKNHGMHFR					Mascot
1170.5585	1170.5868	0.0283	24	294	302	QKNHGMHFR			Oxidation (M)[6]		Mascot
1170.5585	1170.5868	0.0283	24	294	302	QKNHGMHFR			Oxidation (M)[6]		Mascot
1187.6643	1187.6113	-0.053	-45	276	285	DNGLLLHIHR					Mascot
1245.6337	1245.5771	-0.0566	-45	208	217	FLFCAEALFK	63	99.994	Carbamidomethyl (C)[4]		Mascot
1245.6337	1245.5771	-0.0566	-45	208	217	FLFCAEALFK			Carbamidomethyl (C)[4]		Mascot
1275.7307	1275.6761	-0.0546	-43	330	340	EITLGFVDLLR	95	100			Mascot
1275.7307	1275.6761	-0.0546	-43	330	340	EITLGFVDLLR					Mascot
1451.6219	1451.5577	-0.0642	-44	192	203	DDENVNSQPFMR					Mascot
1467.6168	1467.5497	-0.0671	-46	192	203	DDENVNSQPFMR			Oxidation (M)[11]		Mascot
1546.7358	1546.6625	-0.0733	-47	441	453	WSPELAAACEVWK			Carbamidomethyl (C)[9]		Mascot
1812.7714	1812.6842	-0.0872	-48	227	242	GHYLNATAGTCEEMMK			Carbamidomethyl (C)[11]		Mascot
1828.7662	1828.6816	-0.0846	-46	227	242	GHYLNATAGTCEEMMK			Carbamidomethyl (C)[11], Oxidation (M)[14]		Mascot
1844.7611	1844.6737	-0.0874	-47	227	242	GHYLNATAGTCEEMMK			Carbamidomethyl (C)[11], Oxidation (M)[14,15]		Mascot
1984.8673	1984.7748	-0.0925	-47	227	243	GHYLNATAGTCEEMMKR			Carbamidomethyl (C)[11], Oxidation (M)[14]		Mascot
2000.8623	2000.7628	-0.0995	-50	227	243	GHYLNATAGTCEEMMKR			Carbamidomethyl (C)[11], Oxidation (M)[14,15]		Mascot
2050.0134	2049.9038	-0.1096	-53	310	329	MSGGDHIHAGTVVGKLEGER					Mascot
2169.9868	2169.8826	-0.1042	-48	185	203	GGLDFTKDDENVNSQPFMR					Mascot
2169.9868	2169.8826	-0.1042	-48	185	203	GGLDFTKDDENVNSQPFMR	122	100			Mascot
2185.9819	2185.8743	-0.1076	-49	185	203	GGLDFTKDDENVNSQPFMR			Oxidation (M)[18]		Mascot
3022.4548	3022.3018	-0.153	-51	249	275	ELGVPIVMHDYLTGGFTANTSLAHYCR			Carbamidomethyl (C)[26]		Mascot
3022.4548	3022.3018	-0.153	-51	249	275	ELGVPIVMHDYLTGGFTANTSLAHYCR	144	100	Carbamidomethyl (C)[26]		Mascot
3038.45	3038.3064	-0.1436	-47	249	275	ELGVPIVMHDYLTGGFTANTSLAHYCR			Carbamidomethyl (C)[26], Oxidation (M)[8]		Mascot

Rank	Protein Name	Accession No	Protein MW	Protein PI	Pep. Count	Protein Score	Protein Score C. I. %	Total Ion Score	Total Ion C. I. %
6	Ribulose bisphosphate carboxylase large chain OS=Sesbania sesban GN=rbcL PE=3 SV=1	RBL_SESSE	52358.4	6.09	15	571	100	484	100

Peptide Information

Calc. Mass	Obsrv. Mass	± da	± ppm	Start Seq.	End Seq.	Sequence	Ion Score	C. I. %	Modification	Rank	Result Type
830.4366	830.4017	-0.0349	-42	440	446	EGNEIIR					Mascot
898.41	898.371	-0.039	-43	306	312	NHGMHFR					Mascot
910.4451	910.4059	-0.0392	-43	188	194	AVYECLR	10	0	Carbamidomethyl (C)[5]		Mascot
910.4451	910.4059	-0.0392	-43	188	194	AVYECLR			Carbamidomethyl (C)[5]		Mascot
912.4719	912.4152	-0.0567	-62	296	303	AMHAVIDR					Mascot
914.4049	914.3646	-0.0403	-44	306	312	NHGMHFR			Oxidation (M)[4]		Mascot
928.4669	928.4258	-0.0411	-44	296	303	AMHAVIDR			Oxidation (M)[2]		Mascot
962.4788	962.4354	-0.0434	-45	228	236	SQAETGEIK					Mascot
1116.583	1116.5344	-0.0486	-44	422	431	VALEACVQAR			Carbamidomethyl (C)[6]		Mascot
1116.583	1116.5344	-0.0486	-44	422	431	VALEACVQAR	51	99.894	Carbamidomethyl (C)[6]		Mascot
1154.5636	1154.5364	-0.0272	-24	304	312	QKNHGMHFR					Mascot
1170.5585	1170.5868	0.0283	24	304	312	QKNHGMHFR			Oxidation (M)[6]		Mascot
1170.5585	1170.5868	0.0283	24	304	312	QKNHGMHFR			Oxidation (M)[6]		Mascot
1187.6643	1187.6113	-0.053	-45	286	295	DNGLLLHIHR					Mascot
1245.6337	1245.5771	-0.0566	-45	218	227	FLFCAEAIFK	63	99.994	Carbamidomethyl (C)[4]		Mascot
1245.6337	1245.5771	-0.0566	-45	218	227	FLFCAEAIFK			Carbamidomethyl (C)[4]		Mascot
1275.7307	1275.6761	-0.0546	-43	340	350	EITLGFVDLLR	95	100			Mascot
1275.7307	1275.6761	-0.0546	-43	340	350	EITLGFVDLLR					Mascot
1451.6219	1451.5577	-0.0642	-44	202	213	DDENVNSQPFMR					Mascot
1467.6168	1467.5497	-0.0671	-46	202	213	DDENVNSQPFMR			Oxidation (M)[11]		Mascot
1546.7358	1546.6625	-0.0733	-47	451	463	WSPELAAACEVWK			Carbamidomethyl (C)[9]		Mascot
1794.8149	1794.7372	-0.0777	-43	237	252	GHYLNATAGTCEEMIK			Carbamidomethyl (C)[11]		Mascot
2169.9868	2169.8826	-0.1042	-48	195	213	GGLDFTKDDENVNSQPFMR					Mascot
2169.9868	2169.8826	-0.1042	-48	195	213	GGLDFTKDDENVNSQPFMR	122	100			Mascot
2185.9819	2185.8743	-0.1076	-49	195	213	GGLDFTKDDENVNSQPFMR			Oxidation (M)[18]		Mascot
3022.4548	3022.3018	-0.153	-51	259	285	ELGVPIVMHDYLTGGFTANTSLAHYCR			Carbamidomethyl (C)[26]		Mascot
3022.4548	3022.3018	-0.153	-51	259	285	ELGVPIVMHDYLTGGFTANTSLAHYCR	144	100	Carbamidomethyl (C)[26]		Mascot
3038.45	3038.3064	-0.1436	-47	259	285	ELGVPIVMHDYLTGGFTANTSLAHYCR			Carbamidomethyl (C)[26], Oxidation (M)[8]		Mascot

Rank	Protein Name	Accession No	Protein MW	Protein PI	Pep. Count	Protein Score	Protein Score C. I. %	Total Ion Score	Total Ion C. I. %
7	Ribulose bisphosphate carboxylase large chain (Fragment) OS=Oxalis dillenii GN=rbcL PE=3 SV=2	RBL_OXADI	51653.9	5.91	14	563	100	482	100

Peptide Information

Calc. Mass	Obsrv. Mass	± da	± ppm	Start Seq.	End Seq.	Sequence	Ion Score	C. I. %	Modification	Rank	Result Type
830.4366	830.4017	-0.0349	-42	431	437	EGNEIIR					Mascot
910.4451	910.4059	-0.0392	-43	179	185	AVYECLR	10	0	Carbamidomethyl (C)[5]		Mascot
910.4451	910.4059	-0.0392	-43	179	185	AVYECLR			Carbamidomethyl (C)[5]		Mascot
912.4719	912.4152	-0.0567	-62	287	294	AMHAVIDR					Mascot
928.4669	928.4258	-0.0411	-44	287	294	AMHAVIDR			Oxidation (M)[2]		Mascot
1023.4741	1023.4259	-0.0482	-47	342	349	DDFVEKDR					Mascot
1116.583	1116.5344	-0.0486	-44	413	422	VALEACVQAR			Carbamidomethyl (C)[6]		Mascot
1116.583	1116.5344	-0.0486	-44	413	422	VALEACVQAR	51	99.894	Carbamidomethyl (C)[6]		Mascot
1187.6643	1187.6113	-0.053	-45	277	286	DNGLLLHIHR					Mascot
1245.6337	1245.5771	-0.0566	-45	209	218	FLFCAEAIFK	63	99.994	Carbamidomethyl (C)[4]		Mascot
1245.6337	1245.5771	-0.0566	-45	209	218	FLFCAEAIFK			Carbamidomethyl (C)[4]		Mascot
1275.7307	1275.6761	-0.0546	-43	331	341	EITLGFVDLLR	95	100			Mascot
1275.7307	1275.6761	-0.0546	-43	331	341	EITLGFVDLLR					Mascot
1451.6219	1451.5577	-0.0642	-44	193	204	DDENVNSQPFMR					Mascot
1467.6168	1467.5497	-0.0671	-46	193	204	DDENVNSQPFMR			Oxidation (M)[11]		Mascot
1546.7358	1546.6625	-0.0733	-47	442	454	WSPELAAACEVWK			Carbamidomethyl (C)[9]		Mascot
1794.8149	1794.7372	-0.0777	-43	228	243	GHYLNATAGTCEEMIK			Carbamidomethyl (C)[11]		Mascot
2009.059	2008.9618	-0.0972	-48	331	347	EITLGFVDLLRDDFVEK					Mascot
2169.9868	2169.8826	-0.1042	-48	186	204	GGLDFTKDDENVNSQPFMR					Mascot
2169.9868	2169.8826	-0.1042	-48	186	204	GGLDFTKDDENVNSQPFMR	122	100			Mascot
2185.9819	2185.8743	-0.1076	-49	186	204	GGLDFTKDDENVNSQPFMR			Oxidation (M)[18]		Mascot
3022.4548	3022.3018	-0.153	-51	250	276	ELGVPIVMHDYLTGGFTANTSLAHYCR			Carbamidomethyl (C)[26]		Mascot
3022.4548	3022.3018	-0.153	-51	250	276	ELGVPIVMHDYLTGGFTANTSLAHYCR	144	100	Carbamidomethyl (C)[26]		Mascot
3038.45	3038.3064	-0.1436	-47	250	276	ELGVPIVMHDYLTGGFTANTSLAHYCR			Carbamidomethyl (C)[26], Oxidation (M)[8]		Mascot

Rank	Protein Name	Accession No	Protein MW	Protein PI	Pep. Count	Protein Score	Protein Score C. I. %	Total Ion Score	Total Ion C. I. %
8	Ribulose bisphosphate carboxylase large chain OS=Lactuca sativa GN=rbcL PE=3 SV=2	RBL_LACSA	52861.6	5.91	13	551	100	486	100

Peptide Information

Calc. Mass	Obsrv. Mass	± da	± ppm	Start Seq.	End Seq.	Sequence	Ion Score	C. I. %	Modification	Rank	Result Type
910.4451	910.4059	-0.0392	-43	188	194	AVYECLR	10	0	Carbamidomethyl (C)[5]		Mascot
910.4451	910.4059	-0.0392	-43	188	194	AVYECLR			Carbamidomethyl (C)[5]		Mascot
912.4719	912.4152	-0.0567	-62	296	303	AMHAVIDR					Mascot
928.4669	928.4258	-0.0411	-44	296	303	AMHAVIDR			Oxidation (M)[2]		Mascot
962.4788	962.4354	-0.0434	-45	228	236	SQAETGEIK					Mascot
1116.583	1116.5344	-0.0486	-44	422	431	VALEACVQAR			Carbamidomethyl (C)[6]		Mascot
1116.583	1116.5344	-0.0486	-44	422	431	VALEACVQAR	51	99.894	Carbamidomethyl (C)[6]		Mascot
1187.6643	1187.6113	-0.053	-45	286	295	DNGLLLHIHR					Mascot
1245.6337	1245.5771	-0.0566	-45	218	227	FLFCAEAIFK	63	99.994	Carbamidomethyl (C)[4]		Mascot
1245.6337	1245.5771	-0.0566	-45	218	227	FLFCAEAIFK			Carbamidomethyl (C)[4]		Mascot
1275.7307	1275.6761	-0.0546	-43	340	350	EITLGFVDLLR	95	100			Mascot
1275.7307	1275.6761	-0.0546	-43	340	350	EITLGFVDLLR					Mascot
1451.6219	1451.5577	-0.0642	-44	202	213	DDENVNSQPFMR					Mascot
1467.6168	1467.5497	-0.0671	-46	202	213	DDENVNSQPFMR			Oxidation (M)[11]		Mascot
1546.7358	1546.6625	-0.0733	-47	451	463	WSPELAAACEVWK			Carbamidomethyl (C)[9]		Mascot
1812.7714	1812.6842	-0.0872	-48	237	252	GHYLNATAGTCEEMMK			Carbamidomethyl (C)[11]		Mascot
1828.7662	1828.6816	-0.0846	-46	237	252	GHYLNATAGTCEEMMK			Carbamidomethyl (C)[11], Oxidation (M)[14]		Mascot
1844.7611	1844.6737	-0.0874	-47	237	252	GHYLNATAGTCEEMMK			Carbamidomethyl (C)[11], Oxidation (M)[14,15]		Mascot
1984.8673	1984.7748	-0.0925	-47	237	253	GHYLNATAGTCEEMMKR			Carbamidomethyl (C)[11], Oxidation (M)[14]		Mascot
2000.8623	2000.7628	-0.0995	-50	237	253	GHYLNATAGTCEEMMKR			Carbamidomethyl (C)[11], Oxidation (M)[14,15]		Mascot
2169.9868	2169.8826	-0.1042	-48	195	213	GGLDFTKDDENVNSQPFMR					Mascot
2169.9868	2169.8826	-0.1042	-48	195	213	GGLDFTKDDENVNSQPFMR	122	100			Mascot
2185.9819	2185.8743	-0.1076	-49	195	213	GGLDFTKDDENVNSQPFMR			Oxidation (M)[18]		Mascot
3022.4548	3022.3018	-0.153	-51	259	285	ELGVPIVMHDYLTGGFTANTSLAHYCR			Carbamidomethyl (C)[26]		Mascot
3022.4548	3022.3018	-0.153	-51	259	285	ELGVPIVMHDYLTGGFTANTSLAHYCR	144	100	Carbamidomethyl (C)[26]		Mascot
3038.45	3038.3064	-0.1436	-47	259	285	ELGVPIVMHDYLTGGFTANTSLAHYCR			Carbamidomethyl (C)[26], Oxidation (M)[8]		Mascot

Rank	Protein Name	Accession No	Protein MW	Protein PI	Pep. Count	Protein Score	Protein Score C. I. %	Total Ion Score	Total Ion C. I. %
9	Ribulose bisphosphate carboxylase large chain OS=Cichorium intybus GN=rbcL PE=3 SV=1	RBL_CICIN	52890.6	6.09	13	550	100	486	100

Peptide Information

Calc. Mass	Obsrv. Mass	± da	± ppm	Start Seq.	End Seq.	Sequence	Ion Score	C. I. %	Modification	Rank	Result Type
910.4451	910.4059	-0.0392	-43	188	194	AVYECLR	10	0	Carbamidomethyl (C)[5]		Mascot
910.4451	910.4059	-0.0392	-43	188	194	AVYECLR			Carbamidomethyl (C)[5]		Mascot
912.4719	912.4152	-0.0567	-62	296	303	AMHAVIDR					Mascot
928.4669	928.4258	-0.0411	-44	296	303	AMHAVIDR			Oxidation (M)[2]		Mascot
962.4788	962.4354	-0.0434	-45	228	236	SQAETGEIK					Mascot
1116.583	1116.5344	-0.0486	-44	422	431	VALEACVQAR			Carbamidomethyl (C)[6]		Mascot
1116.583	1116.5344	-0.0486	-44	422	431	VALEACVQAR	51	99.894	Carbamidomethyl (C)[6]		Mascot
1187.6643	1187.6113	-0.053	-45	286	295	DNGLLLHIHR					Mascot
1245.6337	1245.5771	-0.0566	-45	218	227	FLFCAEAIFK	63	99.994	Carbamidomethyl (C)[4]		Mascot
1245.6337	1245.5771	-0.0566	-45	218	227	FLFCAEAIFK			Carbamidomethyl (C)[4]		Mascot
1275.7307	1275.6761	-0.0546	-43	340	350	EITLGFVDLLR	95	100			Mascot
1275.7307	1275.6761	-0.0546	-43	340	350	EITLGFVDLLR					Mascot
1451.6219	1451.5577	-0.0642	-44	202	213	DDENVNSQPFMR					Mascot
1467.6168	1467.5497	-0.0671	-46	202	213	DDENVNSQPFMR			Oxidation (M)[11]		Mascot
1546.7358	1546.6625	-0.0733	-47	451	463	WSPELAAACEVWK			Carbamidomethyl (C)[9]		Mascot
1812.7714	1812.6842	-0.0872	-48	237	252	GHYLNATAGTCEEMMK			Carbamidomethyl (C)[11]		Mascot
1828.7662	1828.6816	-0.0846	-46	237	252	GHYLNATAGTCEEMMK			Carbamidomethyl (C)[11], Oxidation (M)[14]		Mascot
1844.7611	1844.6737	-0.0874	-47	237	252	GHYLNATAGTCEEMMK			Carbamidomethyl (C)[11], Oxidation (M)[14,15]		Mascot
1984.8673	1984.7748	-0.0925	-47	237	253	GHYLNATAGTCEEMMKR			Carbamidomethyl (C)[11], Oxidation (M)[14]		Mascot
2000.8623	2000.7628	-0.0995	-50	237	253	GHYLNATAGTCEEMMKR			Carbamidomethyl (C)[11], Oxidation (M)[14,15]		Mascot
2169.9868	2169.8826	-0.1042	-48	195	213	GGLDFTKDDENVNSQPFMR					Mascot
2169.9868	2169.8826	-0.1042	-48	195	213	GGLDFTKDDENVNSQPFMR	122	100			Mascot
2185.9819	2185.8743	-0.1076	-49	195	213	GGLDFTKDDENVNSQPFMR			Oxidation (M)[18]		Mascot
3022.4548	3022.3018	-0.153	-51	259	285	ELGVPIVMHDYLTGGFTANTSLAHYCR			Carbamidomethyl (C)[26]		Mascot
3022.4548	3022.3018	-0.153	-51	259	285	ELGVPIVMHDYLTGGFTANTSLAHYCR	144	100	Carbamidomethyl (C)[26]		Mascot
3038.45	3038.3064	-0.1436	-47	259	285	ELGVPIVMHDYLTGGFTANTSLAHYCR			Carbamidomethyl (C)[26], Oxidation (M)[8]		Mascot

Rank	Protein Name	Accession No	Protein MW	Protein PI	Pep. Count	Protein Score	Protein Score C. I. %	Total Ion Score	Total Ion C. I. %
10	Ribulose bisphosphate carboxylase large chain OS=Ipomoea purpurea GN=rbcL PE=3 SV=1	RBL_IPOPU	53353.9	6.41	18	548	100	422	100

Peptide Information

Calc. Mass	Obsrv. Mass	± da	± ppm	Start Seq.	End Seq.	Sequence	Ion Score	C. I. %	Modification	Rank	Result Type
830.4366	830.4017	-0.0349	-42	440	446	EGNEIIR					Mascot
898.41	898.371	-0.039	-43	306	312	NHGMHFR					Mascot
910.4451	910.4059	-0.0392	-43	188	194	AVYECLR	10	0	Carbamidomethyl (C)[5]		Mascot
910.4451	910.4059	-0.0392	-43	188	194	AVYECLR			Carbamidomethyl (C)[5]		Mascot
912.4719	912.4152	-0.0567	-62	296	303	AMHAVIDR					Mascot
914.4049	914.3646	-0.0403	-44	306	312	NHGMHFR			Oxidation (M)[4]		Mascot
928.4669	928.4258	-0.0411	-44	296	303	AMHAVIDR			Oxidation (M)[2]		Mascot
962.4788	962.4354	-0.0434	-45	228	236	SQAETGEIK					Mascot
1023.4377	1023.4259	-0.0118	-12	351	358	DDFVEQDR					Mascot
1116.583	1116.5344	-0.0486	-44	422	431	VALEACVQAR			Carbamidomethyl (C)[6]		Mascot
1116.583	1116.5344	-0.0486	-44	422	431	VALEACVQAR	51	99.919	Carbamidomethyl (C)[6]		Mascot
1154.5636	1154.5364	-0.0272	-24	304	312	QKNHGMHFR					Mascot
1170.5585	1170.5868	0.0283	24	304	312	QKNHGMHFR			Oxidation (M)[6]		Mascot
1170.5585	1170.5868	0.0283	24	304	312	QKNHGMHFR			Oxidation (M)[6]		Mascot
1187.6643	1187.6113	-0.053	-45	286	295	DNGLLLHIHR					Mascot
1275.7307	1275.6761	-0.0546	-43	340	350	EITLGFVDLLR	95	100			Mascot
1275.7307	1275.6761	-0.0546	-43	340	350	EITLGFVDLLR					Mascot
1451.6219	1451.5577	-0.0642	-44	202	213	DDENVNSQPFMR					Mascot
1467.6168	1467.5497	-0.0671	-46	202	213	DDENVNSQPFMR			Oxidation (M)[11]		Mascot
1546.7358	1546.6625	-0.0733	-47	451	463	WSPELAAACEVWK			Carbamidomethyl (C)[9]		Mascot
1812.7714	1812.6842	-0.0872	-48	237	252	GHYLNATAGTCEEMMK			Carbamidomethyl (C)[11]		Mascot
1828.7662	1828.6816	-0.0846	-46	237	252	GHYLNATAGTCEEMMK			Carbamidomethyl (C)[11], Oxidation (M)[14]		Mascot
1844.7611	1844.6737	-0.0874	-47	237	252	GHYLNATAGTCEEMMK			Carbamidomethyl (C)[11], Oxidation (M)[14,15]		Mascot
1984.8673	1984.7748	-0.0925	-47	237	253	GHYLNATAGTCEEMMKR			Carbamidomethyl (C)[11], Oxidation (M)[14]		Mascot
2000.8623	2000.7628	-0.0995	-50	237	253	GHYLNATAGTCEEMMKR			Carbamidomethyl (C)[11], Oxidation (M)[14,15]		Mascot
2033.9572	2033.8984	-0.0588	-29	447	463	QACKWSPELAAACEVWK			Carbamidomethyl (C)[3,13]		Mascot
2169.9868	2169.8826	-0.1042	-48	195	213	GGLDFTKDDENVNSQPFMR					Mascot
2169.9868	2169.8826	-0.1042	-48	195	213	GGLDFTKDDENVNSQPFMR	122	100			Mascot
2185.9819	2185.8743	-0.1076	-49	195	213	GGLDFTKDDENVNSQPFMR			Oxidation (M)[18]		Mascot
2280.1506	2280.0742	-0.0764	-34	340	358	EITLGFVDLLRDDFVEQDR					Mascot
3022.4548	3022.3018	-0.153	-51	259	285	ELGVPIVMHDYLTGGFTANTSLAHYCR			Carbamidomethyl (C)[26]		Mascot
3022.4548	3022.3018	-0.153	-51	259	285	ELGVPIVMHDYLTGGFTANTSLAHYCR	144	100	Carbamidomethyl (C)[26]		Mascot
3038.45	3038.3064	-0.1436	-47	259	285	ELGVPIVMHDYLTGGFTANTSLAHYCR			Carbamidomethyl (C)[26], Oxidation (M)[8]		Mascot

Gel Idx/Pos	98/D23	Instr./Gel Origin	BA2060/131017A	Process Sta	Analysis Succeeded
Plate [#] Name	[1] 31496	Instrument Sample Name		Spectra	8

Rank	Protein Name	Accession No	Protein MW	Protein PI	Pep Count	Protein Score	Protein Score C. I. %	Total Ion Score	Total Ion C. I. %
1	Stem 31 kDa glycoprotein OS=Glycine max GN=V PE=2 SV=1	VSPB_SOYBI	29261.8	6.72	14	615	100	499	100

Peptide Information

Calc. Mass	Obsrv. Mass	± da	± ppm	Start Seq.	End Seq.	Sequence	Ion Score	C. I. %	Modification	Rank	Result Type
976.5131	976.4814	-0.0317	-32	174	182	MAVTEANLK					Mascot
1022.5741	1022.5379	-0.0362	-35	48	56	LAVEAHNIR					Mascot
1022.5741	1022.5379	-0.0362	-35	48	56	LAVEAHNIR	10	0			Mascot
1111.5994	1111.5532	-0.0462	-42	141	151	GDAPALPETLK					Mascot
1139.5442	1139.5037	-0.0405	-36	246	254	LPNPMYYIE					Mascot
1141.5272	1141.4851	-0.0421	-37	71	79	DYINGEQFR					Mascot
1155.5391	1155.4921	-0.047	-41	246	254	LPNPMYYIE	41	99.137	Oxidation (M)[5]		Mascot
1155.5391	1155.4921	-0.047	-41	246	254	LPNPMYYIE			Oxidation (M)[5]		Mascot
1302.6246	1302.579	-0.0456	-35	60	70	TIPEECVEPTK			Carbamidomethyl (C)[6]		Mascot
1442.7791	1442.7229	-0.0562	-39	184	195	AGFHTWEQLILK					Mascot
1442.7791	1442.7229	-0.0562	-39	184	195	AGFHTWEQLILK	63	99.995			Mascot
1468.7794	1468.7207	-0.0587	-40	196	208	DPHLITPNALSYK					Mascot
1560.7441	1560.6829	-0.0612	-39	84	96	TVNQQAFFYASER	105	100			Mascot
1560.7441	1560.6829	-0.0612	-39	84	96	TVNQQAFFYASER					Mascot
1893.9817	1893.9064	-0.0753	-40	222	238	IVGIIGDQWSDLLGDHR					Mascot
1893.9817	1893.9064	-0.0753	-40	222	238	IVGIIGDQWSDLLGDHR	119	100			Mascot
2323.179	2323.0835	-0.0955	-41	222	242	IVGIIGDQWSDLLGDHRGESR					Mascot
2368.1125	2368.0759	-0.0366	-15	60	79	TIPEECVEPTKDYINGEQFR					Mascot
2425.134	2425.0339	-0.1001	-41	60	79	TIPEECVEPTKDYINGEQFR	62	99.993	Carbamidomethyl (C)[6]		Mascot
2425.134	2425.0339	-0.1001	-41	60	79	TIPEECVEPTKDYINGEQFR			Carbamidomethyl (C)[6]		Mascot
2892.5405	2892.4058	-0.1347	-47	184	208	AGFHTWEQLILKDPHLITPNALSYK					Mascot
2977.4729	2977.3528	-0.1201	-40	97	121	EVHHNDIFIFGIDNTVLSNIPYYEK	99	100			Mascot
2977.4729	2977.3528	-0.1201	-40	97	121	EVHHNDIFIFGIDNTVLSNIPYYEK					Mascot

Rank	Protein Name	Accession No	Protein MW	Protein PI	Pep Count	Protein Score	Protein Score C. I. %	Total Ion Score	Total Ion C. I. %
2	30S ribosomal protein S8, chloroplastic OS=Pseudendoclonium akinetum GN=rps8 PE=3 SV=1	RR8_PSEAK	14927.1	9.74	6	38	0		

Peptide Information

Calc. Mass	Obsrv. Mass	± da	± ppm	Start Seq.	End Seq.	Sequence	Ion Score	C. I. %	Modification	Rank	Result Type
870.5406	870.5085	-0.0321	-37	70	77	KPGITNLK					Mascot
976.5938	976.4814	-0.1124	-115	13	20	IRNAYLVK					Mascot
	976.4814										Mascot
1446.741	1446.7203	-0.0207	-14	119	131	ANKVGGELLCSIW			Carbamidomethyl (C)[10]		Mascot
1474.6917	1474.7126	0.0209	14	1	12	MIYDTISDMLTR			Oxidation (M)[1]		Mascot
1892.0276	1891.9137	-0.1139	-60	15	30	NAYLVKHETVYVLNTK					Mascot
1909.9865	1909.9065	-0.08	-42	41	57	KEGYIESVNLSTNSLQK					Mascot

Rank	Protein Name	Accession No	Protein MW	Protein PI	Pep Count	Protein Score	Protein Score C. I. %	Total Ion Score	Total Ion C. I. %
3	Probable beta-1,3-galactosyltransferase 16 OS=Arabidopsis thaliana GN=B3GALT16 PE=2 SV=1	B3GTG_ARA1	70590.1	6.65	10	37	0		

Peptide Information

Calc. Mass	Obsrv. Mass	± da	± ppm	Start Seq.	End Seq.	Sequence	Ion Score	C. I. %	Modification	Rank	Result Type
842.4995	842.4805	-0.019	-23	540	546	FVVKGHR					Mascot
882.4501	882.5316	0.0815	92	3	9	QFMSVVR			Oxidation (M)[3]		Mascot
976.5574	976.4814	-0.076	-78	547	554	QRDLGLFK					Mascot
1141.5857	1141.4851	-0.1006	-88	1	9	MKQFMSVVR			Oxidation (M)[1]		Mascot
1468.7253	1468.7207	-0.0046	-3	147	158	RICPDFVTAFDK			Carbamidomethyl (C)[3]		Mascot
1893.96	1893.9064	-0.0536	-28	248	263	NHLVDELPLCNKQTGR			Carbamidomethyl (C)[10]		Mascot
1893.96	1893.9064	-0.0536	-28	248	263	NHLVDELPLCNKQTGR			Carbamidomethyl (C)[10]		Mascot
2322.1084	2322.0962	-0.0122	-5	582	600	FHNSDCKSNYILVHYQTPR					Mascot
2368.1536	2368.0759	-0.0777	-33	240	259	CQYHGSLKNHLVDELPLCNK			Carbamidomethyl (C)[1]		Mascot
2425.175	2425.0339	-0.1411	-58	240	259	CQYHGSLKNHLVDELPLCNK			Carbamidomethyl (C)[1,18]		Mascot
2425.175	2425.0339	-0.1411	-58	240	259	CQYHGSLKNHLVDELPLCNK			Carbamidomethyl (C)[1,18]		Mascot
2807.48	2807.2002	-0.2798	-100	549	572	DLGLFKLEDVAMGIWIQQFNQTIK					Mascot
3809.0088	3808.6343	-0.3745	-98	98	131	MRPFLERPDALPETAQGIEEATLAMKGLVLEINR					Mascot

Rank	Protein Name	Accession No	Protein MW	Protein PI	Pep Count	Protein Score	Protein Score C. I. %	Total Ion Score	Total Ion C. I. %
4	F-box protein At2g16450 OS=Arabidopsis thaliana GN=At2g16450 PE=2 SV=1	FB108_ARAT	49548	8.64	9	36	0		

Peptide Information

Calc. Mass	Obsrv. Mass	± da	± ppm	Start Seq.	End Seq.	Sequence	Ion Score	C. I. %	Modification	Rank	Result Type
1302.6517	1302.579	-0.0727	-56	33	43	WASIFGSPYFK					Mascot
1474.7107	1474.7126	0.0019	1	174	187	ALSMVYPSGPGHSR			Oxidation (M)[4]		Mascot
1560.9108	1560.6829	-0.2279	-146	50	63	SSTKPRLLFAIAEK					Mascot
1560.9108	1560.6829	-0.2279	-146	50	63	SSTKPRLLFAIAEK					Mascot
1875.9922	1875.9205	-0.0717	-38	373	389	NSLQRVEIQGFGEAVTK					Mascot
1893.9276	1893.9064	-0.0212	-11	171	187	QYKALSMVYPSGPGHSR			Oxidation (M)[7]		Mascot
1893.9276	1893.9064	-0.0212	-11	171	187	QYKALSMVYPSGPGHSR			Oxidation (M)[7]		Mascot
1897.9153	1897.8987	-0.0166	-9	101	115	FSPDNLLICHYYDLK			Carbamidomethyl (C)[9]		Mascot
2230.1443	2230.1101	-0.0342	-15	116	134	YFSIGYAFGLIYIYGNRGR					Mascot
2313.0525	2313.1135	0.061	26	390	409	SCDVCTFVNHVEDLNVYDLK					Mascot
2334.0706	2334.1001	0.0295	13	211	231	SEGVCINGVLYYLGDTSDWSR					Mascot

Rank	Protein Name	Accession No	Protein MW	Protein PI	Pep Count	Protein Score	Protein Score C. I. %	Total Ion Score	Total Ion C. I. %
5	Photosystem I iron-sulfur center OS=Ostreococcu GN=psaC PE=3 SV=1	PSAC_OSTT/	8736	5.65	6	36	0		

Peptide Information

Calc. Mass	Obsrv. Mass	± da	± ppm	Start Seq.	End Seq.	Sequence	Ion Score	C. I. %	Modification	Rank	Result Type
870.4791	870.5085	0.0294	34	36	44	AGQIASAPR					Mascot
1468.6559	1468.7207	0.0648	44	54	66	CEAACPTDFLSVR			Carbamidomethyl (C)[1]		Mascot
1474.6487	1474.7126	0.0639	43	7	19	IYDTCIGCTQCVR					Mascot
1893.818	1893.9064	0.0884	47	20	35	ACPTDVLEMVPWDGCK			Carbamidomethyl (C)[2,15], Oxidation (M)[9]		Mascot
1893.818	1893.9064	0.0884	47	20	35	ACPTDVLEMVPWDGCK			Carbamidomethyl (C)[2,15], Oxidation (M)[9]		Mascot
2299.0449	2299.072	0.0271	12	1	19	MSHAVKIYDTCIGCTQCVR			Carbamidomethyl (C)[11,14,17]		Mascot
3349.4487	3349.4919	0.0432	13	7	35	IYDTCIGCTQCVRACPTDVLEMVPWDGCK			Carbamidomethyl (C)[5,8], Oxidation (M)[22]		Mascot

Rank	Protein Name	Accession No	Protein MW	Protein PI	Pep Count	Protein Score	Protein Score C. I. %	Total Ion Score	Total Ion C. I. %
6	Peptidyl-prolyl cis-trans isomerase FKBP20-2, chloroplastic OS=Arabidopsis thaliana GN=FKBP20-2 PE=	FK202_ARAT	27174.8	8.83	7	35	0		

Peptide Information

Calc. Mass	Obsrv. Mass	± da	± ppm	Start Seq.	End Seq.	Sequence	Ion Score	C. I. %	Modification	Rank	Result Type
1022.5516	1022.5379	-0.0137	-13	109	117	VLASDNYIK					Mascot
1022.5516	1022.5379	-0.0137	-13	109	117	VLASDNYIK					Mascot
1893.9673	1893.9064	-0.0609	-32	167	183	IRMGTNALVPGFEMGIR			Oxidation (M)[3,14]		Mascot
1893.9673	1893.9064	-0.0609	-32	167	183	IRMGTNALVPGFEMGIR			Oxidation (M)[3,14]		Mascot
1898.0018	1897.8987	-0.1031	-54	109	125	VLASDNYIKADSGLIYR					Mascot
2225.1018	2225.0266	-0.0752	-34	213	230	QFEVFDVELLSIQNCERR					Mascot
2239.2485	2239.0327	-0.2158	-96	192	212	RIIIPPELGPPVGPSTFFSSK					Mascot
2323.24	2323.0835	-0.1565	-67	1	20	MVTILSTPLSPRLTFLCETK			Carbamidomethyl (C)[17], Oxidation (M)[1]		Mascot
2334.1516	2334.1001	-0.0515	-22	169	190	MGTNALVPGFEMGIRDMKPGGR					Mascot

Rank	Protein Name	Accession No	Protein MW	Protein PI	Pep Count	Protein Score	Protein Score C. I. %	Total Ion Score	Total Ion C. I. %
7	ATP synthase subunit b, chloroplastic OS=Antho formosae GN=atpF PE=2 SV=1	ATPF_ANTFC	21244.2	6.36	6	35	0		

Peptide Information

Calc. Mass	Obsrv. Mass	± da	± ppm	Start Seq.	End Seq.	Sequence	Ion Score	C. I. %	Modification	Rank	Result Type
856.5363	856.495	-0.0413	-48	78	84	LKQAQIR					Mascot
1022.4822	1022.5379	0.0557	54	97	105	VNGLSEMEK			Oxidation (M)[7]		Mascot
1022.4822	1022.5379	0.0557	54	97	105	VNGLSEMEK			Oxidation (M)[7]		Mascot
1360.6591	1360.6113	-0.0478	-35	108	119	QDLINIADEDSK					Mascot
1909.9398	1909.9065	-0.0333	-17	168	184	MIDYHIGLLKTMGSTTE					Mascot
1925.9347	1925.8883	-0.0464	-24	168	184	MIDYHIGLLKTMGSTTE			Oxidation (M)[1]		Mascot
2322.2473	2322.0962	-0.1511	-65	3	21	NLIFFVIPFHFWLPAEGFK					Mascot
2368.2036	2368.0759	-0.1277	-54	147	167	LALERASEVLNNCLNSELHSR					Mascot
2425.2251	2425.0339	-0.1912	-79	147	167	LALERASEVLNNCLNSELHSR			Carbamidomethyl (C)[13]		Mascot
2425.2251	2425.0339	-0.1912	-79	147	167	LALERASEVLNNCLNSELHSR			Carbamidomethyl (C)[13]		Mascot

Rank	Protein Name	Accession No	Protein MW	Protein PI	Pep Count	Protein Score	Protein Score C. I. %	Total Ion Score	Total Ion C. I. %
8	Protein ROOT HAIR DEFECTIVE 3 homolog 1 OS=Arabidopsis thaliana GN=At1g72960 PE=2 SV=2	RHD31_ARAT	88763	5.41	11	34	0		

Peptide Information

Calc. Mass	Obsrv. Mass	± da	± ppm	Start Seq.	End Seq.	Sequence	Ion Score	C. I. %	Modification	Rank	Result Type
842.4406	842.4805	0.0399	47	387	393	FKASFDK					Mascot
996.5546	996.5546	0	0	166	173	TTMLFVIR			Oxidation (M)[3]		Mascot
1111.647	1111.5532	-0.0938	-84	142	151	EQAANKPLLK					Mascot
1442.7598	1442.7229	-0.0369	-26	442	454	DIEAHISSVRTSK					Mascot
1442.7598	1442.7229	-0.0369	-26	442	454	DIEAHISSVRTSK					Mascot
1894.028	1893.9064	-0.1216	-64	178	193	TPLENLEPVLREDIQK					Mascot
1894.028	1893.9064	-0.1216	-64	178	193	TPLENLEPVLREDIQK	1	0			Mascot
1897.8497	1897.8987	0.049	26	550	565	ERFGTIFSHDSDSMPR			Oxidation (M)[14]		Mascot
2322.0522	2322.0962	0.044	19	88	108	CAGIEPCTLVMDLEGTDGRER			Carbamidomethyl (C)[1]		Mascot
2425.2102	2425.0339	-0.1763	-73	32	55	DVKLADCGLSYAVVSIMGPQSSGK					Mascot
2425.2102	2425.0339	-0.1763	-73	32	55	DVKLADCGLSYAVVSIMGPQSSGK					Mascot
2691.1694	2691.1775	0.0081	3	1	25	MDADKSEGCCSVQLIDGDGIYNVSR			Oxidation (M)[1]		Mascot
2748.1909	2748.1936	0.0027	1	1	25	MDADKSEGCCSVQLIDGDGIYNVSR			Carbamidomethyl (C)[9], Oxidation (M)[1]		Mascot
3338.8008	3338.6255	-0.1753	-53	705	733	NPLYLGVMFVAFLLAKALWTQLDIPGEFR			Oxidation (M)[8]		Mascot
3795.0083	3794.6521	-0.3562	-94	118	151	QSALFALAISDIVLINMWCHDIGREQAANKPLLK			Oxidation (M)[17]		Mascot

Rank	Protein Name	Accession No	Protein MW	Protein PI	Pep Count	Protein Score	Protein Score C. I. %	Total Ion Score	Total Ion C. I. %
9	ABC transporter G family member 27 OS=Arabido thaliana GN=ABCG27 PE=2 SV=1	AB27G_ARAT	81718.5	9.12	11	33	0		

Peptide Information

Calc. Mass	Obsrv. Mass	± da	± ppm	Start Seq.	End Seq.	Sequence	Ion Score	C. I. %	Modification	Rank	Result Type
805.431	805.4644	0.0334	41	469	475	LMITCPK					Mascot
842.356	842.4805	0.1245	148	158	165	GMTSSSEK			Oxidation (M)[2]		Mascot
850.4451	850.5108	0.0657	77	561	567	AMLSKER			Oxidation (M)[2]		Mascot
1155.6377	1155.4921	-0.1456	-126	329	338	IVQMLHCIAK					Mascot
1155.6377	1155.4921	-0.1456	-126	329	338	IVQMLHCIAK					Mascot
1194.6221	1194.506	-0.1161	-97	447	456	TQIAVMEKMK			Oxidation (M)[6]		Mascot
1468.8126	1468.7207	-0.0919	-63	329	341	IVQMLHCIAKAGK			Carbamidomethyl (C)[7]		Mascot
2225.0959	2225.0266	-0.0693	-31	476	492	REWGLSWWEQYCLLSLR					Mascot
2299.0645	2299.072	0.0075	3	689	708	VQYEEIMESVNGEEIESGLK			Oxidation (M)[7]		Mascot
2334.2419	2334.1001	-0.1418	-61	135	153	FQAEPTFPIYLKFIDITYK					Mascot
2368.229	2368.0759	-0.1531	-65	644	665	ATTLASVTVMTFMLAGGYFVKK			Oxidation (M)[10,13]		Mascot
3347.7073	3347.5273	-0.18	-54	298	328	RVCIGNEIMTNPSLLLDEPTSSLDSTTALK			Oxidation (M)[9]		Mascot

Rank	Protein Name	Accession No	Protein MW	Protein PI	Pep Count	Protein Score	Protein Score C. I. %	Total Ion Score	Total Ion C. I. %
10	Ribulose bisphosphate carboxylase small chain (512 (Fragment) OS=Triticum aestivum PE=3 SV=1	RBS3_WHEA	13046.5	5.84	5	32	0		

Peptide Information

Calc. Mass	Obsrv. Mass	± da	± ppm	Start Seq.	End Seq.	Sequence	Ion Score	C. I. %	Modification	Rank	Result Type
1165.571	1165.6973	0.1263	108	23	31	WVPCLEFSK			Carbamidomethyl (C)[4]		Mascot
2211.0571	2211.0076	-0.0495	-22	93	112	QVQCVSFIAFKPPGCEESGK			Carbamidomethyl (C)[4]		Mascot
2225.073	2225.0266	-0.0464	-21	93	113	QVQCVSFIAFKPPGCEESGKA					Mascot
2239.062	2239.0327	-0.0293	-13	56	75	LPMFGCTDATQVINEVEEVK			Oxidation (M)[3]		Mascot
2408.1836	2408	-0.1836	-76	56	76	LPMFGCTDATQVINEVEEVKK			Carbamidomethyl (C)[6]		Mascot

Gel Idx/Pos	99/D24	Instr./Gel Origin	BA2060/131017A	Process Sta	Analysis Succeeded
Plate [#] Name	[1] 31496	Instrument Sample Name		Spectra	8

Rank	Protein Name	Accession No	Protein MW	Protein PI	Pep. Count	Protein Score	Protein Score C. I. %	Total Ion Score	Total Ion C. I. %
1	Carbonic anhydrase OS=Flaveria bidentis PE=2 S	CAHX_FLABI	35522.9	5.85	4	70	99.671	57	99.979

Peptide Information

Calc. Mass	Obsrv. Mass	± da	± ppm	Start Seq.	End Seq.	Sequence	Ion Score	C. I. %	Modification	Rank	Result Type
1219.5234	1219.4924	-0.031	-25	156	165	FMVFACSDSR			Carbamidomethyl (C)[6]		Mascot
1235.5183	1235.4862	-0.0321	-26	156	165	FMVFACSDSR			Carbamidomethyl (C)[6], Oxidation (M)[2]		Mascot
1892.0276	1891.9565	-0.0711	-38	277	293	EAVNVSLGNLLTYPFVR					Mascot
1986.9589	1986.7806	-0.1783	-90	2	21	SAASAFAMNAPSFVNASSLK			Oxidation (M)[8]		Mascot
2057.0273	2056.9592	-0.0681	-33	166	183	VCPSHVLDFQPGEAFVVR			Carbamidomethyl (C)[2]		Mascot
2057.0273	2056.9592	-0.0681	-33	166	183	VCPSHVLDFQPGEAFVVR	57	99.979	Carbamidomethyl (C)[2]		Mascot

Rank	Protein Name	Accession No	Protein MW	Protein PI	Pep. Count	Protein Score	Protein Score C. I. %	Total Ion Score	Total Ion C. I. %
2	Carbonic anhydrase 1 OS=Flaveria linearis PE=2	CAH1_FLALI	35551.8	5.7	4	69	99.596	57	99.979

Peptide Information

Calc. Mass	Obsrv. Mass	± da	± ppm	Start Seq.	End Seq.	Sequence	Ion Score	C. I. %	Modification	Rank	Result Type
1219.5234	1219.4924	-0.031	-25	156	165	FMVFACSDSR			Carbamidomethyl (C)[6]		Mascot
1235.5183	1235.4862	-0.0321	-26	156	165	FMVFACSDSR			Carbamidomethyl (C)[6], Oxidation (M)[2]		Mascot
1892.0276	1891.9565	-0.0711	-38	277	293	EAVNVSLGNLLTYPFVR					Mascot
2057.0273	2056.9592	-0.0681	-33	166	183	VCPSHVLDFQPGEAFVVR			Carbamidomethyl (C)[2]		Mascot
2057.0273	2056.9592	-0.0681	-33	166	183	VCPSHVLDFQPGEAFVVR	57	99.979	Carbamidomethyl (C)[2]		Mascot
2238.9866	2239.0571	0.0705	31	258	276	VVAEHNGTHLDDQCVQCEK			Carbamidomethyl (C)[14,17]		Mascot

Rank	Protein Name	Accession No	Protein MW	Protein PI	Pep. Count	Protein Score	Protein Score C. I. %	Total Ion Score	Total Ion C. I. %
3	Carbonic anhydrase OS=Flaveria brownii PE=2 S'	CAHX_FLABF	35524.8	5.7	3	66	99.094	57	99.979

Peptide Information

Calc. Mass	Obsrv. Mass	± da	± ppm	Start Seq.	End Seq.	Sequence	Ion Score	C. I. %	Modification	Rank	Result Type
1219.5234	1219.4924	-0.031	-25	156	165	FMVFACSDSR			Carbamidomethyl (C)[6]		Mascot
1235.5183	1235.4862	-0.0321	-26	156	165	FMVFACSDSR			Carbamidomethyl (C)[6], Oxidation (M)[2]		Mascot
1892.0276	1891.9565	-0.0711	-38	277	293	EAVNVSLGNLLTYPFVR					Mascot
2057.0273	2056.9592	-0.0681	-33	166	183	VCPSHVLDFQPGEAFVVR			Carbamidomethyl (C)[2]		Mascot
2057.0273	2056.9592	-0.0681	-33	166	183	VCPSHVLDFQPGEAFVVR	57	99.979	Carbamidomethyl (C)[2]		Mascot

Rank	Protein Name	Accession No	Protein MW	Protein PI	Pep. Count	Protein Score	Protein Score C. I. %	Total Ion Score	Total Ion C. I. %
4	Carbonic anhydrase, chloroplastic OS=Pisum sat PE=1 SV=1	CAHC_PEA	35355.1	7.01	1	57	93.589	57	99.979

Peptide Information

Calc. Mass	Obsrv. Mass	± da	± ppm	Start Seq.	End Seq.	Sequence	Ion Score	C. I. %	Modification	Rank	Result Type
2057.0273	2056.9592	-0.0681	-33	164	181	VCPSHVLDFQPGEAFVVR			Carbamidomethyl (C)[2]		Mascot
2057.0273	2056.9592	-0.0681	-33	164	181	VCPSHVLDFQPGEAFVVR	57	99.979	Carbamidomethyl (C)[2]		Mascot

Rank	Protein Name	Accession No	Protein MW	Protein PI	Pep. Count	Protein Score	Protein Score C. I. %	Total Ion Score	Total Ion C. I. %
5	Putative beta-glucosidase 9 OS=Oryza sativa sub japonica GN=BGLU9 PE=5 SV=2	BGL09_ORYS	35525.5	6.3	9	49	56.659		

Peptide Information

Calc. Mass	Obsrv. Mass	± da	± ppm	Start Seq.	End Seq.	Sequence	Ion Score	C. I. %	Modification	Rank	Result Type
805.3773	805.4684	0.0911	113	311	316	VCQYRH					Mascot
850.4417	850.5187	0.077	91	193	199	EFGDRVK					Mascot
882.4468	882.5405	0.0937	106	115	121	FSISWSR					Mascot
994.412	994.4458	0.0338	34	225	232	CSPWEMGK			Carbamidomethyl (C)[1]		Mascot
994.412	994.4458	0.0338	34	225	232	CSPWEMGK			Carbamidomethyl (C)[1]		Mascot
1045.4659	1045.5337	0.0678	65	185	192	DYAEICFK			Carbamidomethyl (C)[6]		Mascot
1665.8204	1665.7286	-0.0918	-55	273	285	QSQFIHDNDLHRR					Mascot
1665.8204	1665.7286	-0.0918	-55	273	285	QSQFIHDNDLHRR					Mascot
1851.8153	1851.8738	0.0585	32	107	121	EMGMDAYRFSISWSR			Oxidation (M)[2]		Mascot
2295.1292	2295.1267	-0.0025	-1	151	170	GVQSFVTLFHYDTPQALEDK					Mascot
2467.1016	2467.0781	-0.0235	-10	86	106	SNGDVADNTYHLYKEDVHMMK					Mascot

Rank	Protein Name	Accession No	Protein MW	Protein PI	Pep. Count	Protein Score	Protein Score C. I. %	Total Ion Score	Total Ion C. I. %
6	Tricyclene synthase TPS4, chloroplastic OS=Me truncatula GN=TPS4 PE=1 SV=1	TPS4_MEDTI	67076.9	5.71	11	43	0		

Peptide Information

Calc. Mass	Obsrv. Mass	± da	± ppm	Start Seq.	End Seq.	Sequence	Ion Score	C. I. %	Modification	Rank	Result Type
850.4529	850.5187	0.0658	77	292	298	LSFGRDR					Mascot
882.5294	882.5405	0.0111	13	573	580	ALILEPIN					Mascot
1229.5586	1229.5894	0.0308	25	242	250	WFIDSYENR					Mascot
1229.6749	1229.5894	-0.0855	-70	136	145	SLHETALRFR					Mascot
1665.8668	1665.7286	-0.1382	-83	401	414	VWSDMLKAFLQEAK					Mascot
1665.8668	1665.7286	-0.1382	-83	401	414	VWSDMLKAFLQEAK					Mascot
1957.9905	1957.9092	-0.0813	-42	146	161	LLREYGYDISPDIFEK					Mascot
1987.078	1986.7806	-0.2974	-150	86	102	MIKDENVNILELIDTVK					Mascot
2295.1768	2295.1267	-0.0501	-22	46	64	KSANYQPNIWNYDILQSLK					Mascot
2394.2195	2394.0278	-0.1917	-80	455	475	EGLGYLENCPMLLQTPSIIFR					Mascot
2467.2358	2467.0781	-0.1577	-64	455	475	EGLGYLENCPMLLQTPSIIFR			Carbamidomethyl (C)[9], Oxidation (M)[11]		Mascot
2807.3142	2807.2085	-0.1057	-38	299	321	LMECFFWAAGMTPFEPQFSNIRK			Carbamidomethyl (C)[4]		Mascot
2923.5056	2923.3154	-0.1902	-65	451	475	NITKEGLGYLENCPMLLQTPSIIFR			Carbamidomethyl (C)[13], Oxidation (M)[15]		Mascot

Rank	Protein Name	Accession No	Protein MW	Protein PI	Pep. Count	Protein Score	Protein Score C. I. %	Total Ion Score	Total Ion C. I. %
7	Homeobox-leucine zipper protein HOX18 OS=Oryz sativa subsp. indica GN=HOX18 PE=2 SV=1	HOX18_ORYS	27577.7	9.13	8	42	0		

Peptide Information

Calc. Mass	Obsrv. Mass	± da	± ppm	Start Seq.	End Seq.	Sequence	Ion Score	C. I. %	Modification	Rank	Result Type
882.5883	882.5405	-0.0478	-54	148	154	QLKLKPR					Mascot
994.5792	994.4458	-0.1334	-134	143	150	HELARQLK					Mascot
994.5792	994.4458	-0.1334	-134	143	150	HELARQLK					Mascot
1165.5518	1165.7134	0.1616	139	237	249	VTVMGGGGGETGK			Oxidation (M)[4]		Mascot
1638.7462	1638.803	0.0568	35	182	194	CCESLTEENKQLK			Carbamidomethyl (C)[1,2]		Mascot
1892.0024	1891.9565	-0.0459	-24	203	221	LASAAAAAAGSQLYVQFPR					Mascot
1908.0073	1907.9623	-0.045	-24	117	132	LQLTKEQSTLLEDSFR					Mascot
2705.1606	2705.061	-0.0996	-37	1	27	MEGEDLGSWLGLGIGGGGYAYGGDDCR					Mascot
3353.6438	3353.5562	-0.0876	-26	203	236	LASAAAAAAGSQLYVQFPRAAAAAMVNVCPSCEK			Oxidation (M)[25]		Mascot

Rank	Protein Name	Accession No	Protein MW	Protein PI	Pep. Count	Protein Score	Protein Score C. I. %	Total Ion Score	Total Ion C. I. %
8	Ribulose bisphosphate carboxylase small chain (512 (Fragment) OS=Triticum aestivum PE=3 SV=1	RBS3_WHEA	13046.5	5.84	6	42	0		

Peptide Information

Calc. Mass	Obsrv. Mass	± da	± ppm	Start Seq.	End Seq.	Sequence	Ion Score	C. I. %	Modification	Rank	Result Type
914.4229	914.3837	-0.0392	-43	50	55	YWTMWK					Mascot
1165.571	1165.7134	0.1424	122	23	31	WVPCLEFSK			Carbamidomethyl (C)[4]		Mascot
1974.9377	1974.9296	-0.0081	-4	77	92	EYPDAYVRIIGFDNMR			Oxidation (M)[15]		Mascot
2211.0571	2211.0281	-0.029	-13	93	112	QVQCVSFIAFKPPGCEESGK			Carbamidomethyl (C)[4]		Mascot
2225.073	2225.0454	-0.0276	-12	93	113	QVQCVSFIAFKPPGCEESGKA					Mascot
2239.062	2239.0571	-0.0049	-2	56	75	LPMFGCTDATQVINEVEEVK			Oxidation (M)[3]		Mascot

Rank	Protein Name	Accession No	Protein MW	Protein PI	Pep. Count	Protein Score	Protein Score C. I. %	Total Ion Score	Total Ion C. I. %
9	B3 domain-containing protein Os03g0120900 OS=Oryza sativa subsp. japonica GN=Os01g0723700 PE=2 SV=1	Y1237_ORYS	82608.3	5.81	13	40	0		

Peptide Information

Calc. Mass	Obsrv. Mass	± da	± ppm	Start Seq.	End Seq.	Sequence	Ion Score	C. I. %	Modification	Rank	Result Type
805.3798	805.4684	0.0886	110	359	364	ERSEER					Mascot
819.4141	819.4315	0.0174	21	1	6	MEEVRR					Mascot
856.525	856.5007	-0.0243	-28	489	495	VLLDIQR					Mascot
967.4223	967.4215	-0.0008	-1	697	704	VEMDNMTK					Mascot
999.4121	999.4196	0.0075	8	697	704	VEMDNMTK			Oxidation (M)[3,6]		Mascot
1199.5258	1199.4899	-0.0359	-30	126	135	LPCDIFCCTK			Carbamidomethyl (C)[3]		Mascot
1444.7583	1444.6976	-0.0607	-42	67	79	LDGHVFLTTGWAK					Mascot
1662.8634	1662.8309	-0.0325	-20	216	229	VTVELFCAMLVFYK					Mascot
1891.8822	1891.9565	0.0743	39	453	469	GCIKSSPASCEVPACLR			Carbamidomethyl (C)[2,10,15]		Mascot
2225.1594	2225.0454	-0.114	-51	618	636	LHNLQDEITGLQQSLEMKK					Mascot
2239.1289	2239.0571	-0.0718	-32	642	660	LAHQVAEHESVFQKSLMER					Mascot
2298.9966	2299.0991	0.1025	45	379	399	SEQACLCHDKTDSGSLFQDSK					Mascot
2691.1443	2691.1968	0.0525	20	365	388	QPSPDSMIDQAESRSEQACLCHDK			Oxidation (M)[7]		Mascot
2748.1658	2748.2236	0.0578	21	365	388	QPSPDSMIDQAESRSEQACLCHDK			Carbamidomethyl (C)[19], Oxidation (M)[7]		Mascot
3353.6042	3353.5562	-0.048	-14	216	243	VTVELFCAMLVFYKWNVDAVAEDFDICR			Carbamidomethyl (C)[7]		Mascot

Rank	Protein Name	Accession No	Protein MW	Protein PI	Pep. Count	Protein Score	Protein Score C. I. %	Total Ion Score	Total Ion C. I. %
10	Putative small RNA degrading nuclease 4 OS=Arabidopsis thaliana GN=SDN4 PE=3 SV=1	SDN4_ARATH	36191.8	8.74	8	38	0		

Peptide Information

Calc. Mass	Obsrv. Mass	± da	± ppm	Start Seq.	End Seq.	Sequence	Ion Score	C. I. %	Modification	Rank	Result Type
850.5219	850.5187	-0.0032	-4	15	21	LMIAKFK					Mascot
1045.5823	1045.5337	-0.0486	-46	11	19	EANRLMIAK					Mascot
1165.6146	1165.7134	0.0988	85	2	10	DYLQAMRLR					Mascot
1199.5402	1199.4899	-0.0503	-42	56	65	DWFVSDVGMK			Oxidation (M)[9]		Mascot
1229.5393	1229.5894	0.0501	41	23	33	ESPANDSPEQR					Mascot
1229.5393	1229.5894	0.0501	41	23	33	ESPANDSPEQR					Mascot
2307.2312	2307.0872	-0.144	-62	176	195	LRRPSLNTLCMSVLGYEVQK					Mascot
2313.2317	2313.1113	-0.1204	-52	196	218	AGVSHHCVHDAAAAMKLALAVIK					Mascot
3794.7712	3794.6836	-0.0876	-23	66	100	MSNVMIPNQMLALDCEMVLCEDGTEGVVRVGAVDR					Mascot

Gel Idx/Pos	102/E1	Instr./Gel Origin	BA2060/131017A	Process Sta	Analysis Succeeded
Plate [#] Name	[1] 31496	Instrument Sample Name		Spectra	8

Rank	Protein Name	Accession No	Protein MW	Protein PI	Pep. Count	Protein Score	Protein Score C. I. %	Total Ion Score	Total Ion C. I. %
1	Stem 28 kDa glycoprotein OS=Glycine max GN=V PE=2 SV=1	VSPA_SOYBI	29046.8	8.75	7	83	99.984	45	99.74

Peptide Information

Calc. Mass	Obsrv. Mass	± da	± ppm	Start Seq.	End Seq.	Sequence	Ion Score	C. I. %	Modification	Rank	Result Type
805.493	805.3817	-0.1113	-138	162	168	IIFLSGR	6	0			Mascot
805.493	805.3817	-0.1113	-138	162	168	IIFLSGR					Mascot
991.4631	991.3939	-0.0692	-70	183	190	AGYHTWEK					Mascot
1120.6038	1120.4524	-0.1514	-135	246	254	LPNPLYYIQ					Mascot
1194.5538	1194.3909	-0.1629	-136	70	78	EYIHGEQYR					Mascot
1194.5538	1194.3909	-0.1629	-136	70	78	EYIHGEQYR	13	0			Mascot
1360.6644	1360.4841	-0.1803	-133	83	93	TVNQQAYFYAR					Mascot
1360.6644	1360.4841	-0.1803	-133	83	93	TVNQQAYFYAR	27	80.975			Mascot
1986.0542	1985.7742	-0.28	-141	191	208	LILKDPQDPSTPNAVSYK					Mascot
2604.2649	2603.8792	-0.3857	-148	47	69	LAVEAHNIFGFETIPEECVEATK			Carbamidomethyl (C)[18]		Mascot

2	Stem 31 kDa glycoprotein (Fragment) OS=Glycine GN=VSP25 PE=2 SV=2	S25K_SOYBN	32861.8	8.59	7	80	99.966	45	99.74

Peptide Information

Calc. Mass	Obsrv. Mass	± da	± ppm	Start Seq.	End Seq.	Sequence	Ion Score	C. I. %	Modification	Rank	Result Type
805.493	805.3817	-0.1113	-138	159	165	IIFLSGR	6	0			Mascot
805.493	805.3817	-0.1113	-138	159	165	IIFLSGR					Mascot
991.4631	991.3939	-0.0692	-70	180	187	AGYHTWEK					Mascot
1194.5538	1194.3909	-0.1629	-136	67	75	EYIHGEQYR					Mascot
1194.5538	1194.3909	-0.1629	-136	67	75	EYIHGEQYR	13	0			Mascot
1360.6644	1360.4841	-0.1803	-133	80	90	TVNQQAYFYAR					Mascot
1360.6644	1360.4841	-0.1803	-133	80	90	TVNQQAYFYAR	27	80.975			Mascot
1986.0542	1985.7742	-0.28	-141	188	205	LILKDPQDPSTPNAVSYK					Mascot
2239.1284	2238.8057	-0.3227	-144	271	291	CVQIGALASLYVVSAVCQQVM			Carbamidomethyl (C)[1]		Mascot
2604.2649	2603.8792	-0.3857	-148	44	66	LAVEAHNIFGFETIPEECVEATK			Carbamidomethyl (C)[18]		Mascot

3	Ribulose bisphosphate carboxylase large chain (Fragment) OS=Ajuga chamaepitys GN=rbcL PE=3 SV=1	RBL_AJUCH	52421.4	6.23	13	75	99.898	15	0

Peptide Information

Calc. Mass	Obsrv. Mass	± da	± ppm	Start Seq.	End Seq.	Sequence	Ion Score	C. I. %	Modification	Rank	Result Type
856.3546	856.4055	0.0509	59	467	473	FEFPAMD					Mascot
910.4451	910.3134	-0.1317	-145	188	194	AVYECLR			Carbamidomethyl (C)[5]		Mascot
914.4049	914.2849	-0.12	-131	306	312	NHGMHFR			Oxidation (M)[4]		Mascot
1021.5312	1021.4023	-0.1289	-126	33	41	DTDILAAFR					Mascot
1023.4741	1023.3226	-0.1515	-148	351	358	DDFVEKDR					Mascot
1204.6071	1204.4578	-0.1493	-124	218	227	FLFCAEAIYK					Mascot
1226.5762	1226.4653	-0.1109	-90	464	473	EIKFEFPAMD					Mascot
1275.7307	1275.5613	-0.1694	-133	340	350	EITLGFVDLLR					Mascot
1407.6678	1407.5081	-0.1597	-113	22	32	LTYYTPEYETK					Mascot
1465.7546	1465.5535	-0.2011	-137	147	159	TFQGPPHGIQVER					Mascot
1465.7546	1465.5535	-0.2011	-137	147	159	TFQGPPHGIQVER	15	0			Mascot
1502.8512	1502.6492	-0.202	-134	165	177	YGRPLLGCTIKPK			Carbamidomethyl (C)[8]		Mascot
1794.8149	1794.5659	-0.249	-139	237	252	GHYLNATAGTCEEMIK			Carbamidomethyl (C)[11]		Mascot
2185.9819	2185.6653	-0.3166	-145	195	213	GGLDFTKDDENVNSQPFMR			Oxidation (M)[18]		Mascot

4	Ribulose bisphosphate carboxylase large chain (Fragment) OS=Sinapis alba GN=rbcL PE=3 SV=1	RBL_SINAL	52405.4	6.23	13	75	99.894	15	0

Peptide Information

Calc. Mass	Obsrv. Mass	± da	± ppm	Start Seq.	End Seq.	Sequence	Ion Score	C. I. %	Modification	Rank	Result Type
805.4818	805.3817	-0.1001	-124	140	146	IPTAYIK					Mascot
805.4818	805.3817	-0.1001	-124	140	146	IPTAYIK					Mascot
856.3546	856.4055	0.0509	59	467	473	FEFPAMD					Mascot
910.4451	910.3134	-0.1317	-145	188	194	AVYECLR			Carbamidomethyl (C)[5]		Mascot
914.4049	914.2849	-0.12	-131	306	312	NHGMHFR			Oxidation (M)[4]		Mascot
1021.5312	1021.4023	-0.1289	-126	33	41	DTDILAAFR					Mascot
1204.6071	1204.4578	-0.1493	-124	218	227	FLFCAEAIYK					Mascot
1226.5762	1226.4653	-0.1109	-90	464	473	EIKFEFPAMD					Mascot
1275.7307	1275.5613	-0.1694	-133	340	350	EITLGFVDLLR					Mascot
1465.7546	1465.5535	-0.2011	-137	147	159	TFQGPPHGIQVER					Mascot
1465.7546	1465.5535	-0.2011	-137	147	159	TFQGPPHGIQVER	15	0			Mascot
1502.8512	1502.6492	-0.202	-134	165	177	YGRPLLGCTIKPK			Carbamidomethyl (C)[8]		Mascot
1789.9667	1789.718	-0.2487	-139	317	334	ALRLSGGDHVHSGTVVGK					Mascot
1794.8149	1794.5659	-0.249	-139	237	252	GHYLNATAGTCEEMIK			Carbamidomethyl (C)[11]		Mascot
2185.9819	2185.6653	-0.3166	-145	195	213	GGLDFTKDDENVNSQPFMR			Oxidation (M)[18]		Mascot

5	Ribulose bisphosphate carboxylase large chain (Fragment) OS=Ulmus alata GN=rbcL PE=3 SV=1	RBL_ULMAL	51558	6.14	12	74	99.866	15	0

Peptide Information

Calc. Mass	Obsrv. Mass	± da	± ppm	Start Seq.	End Seq.	Sequence	Ion Score	C. I. %	Modification	Rank	Result Type
805.4929	805.3817	-0.1112	-138	1	8	VGFKAGVK					Mascot
805.4929	805.3817	-0.1112	-138	1	8	VGFKAGVK					Mascot
910.4451	910.3134	-0.1317	-145	178	184	AVYECLR			Carbamidomethyl (C)[5]		Mascot
914.4049	914.2849	-0.12	-131	296	302	NHGMHFR			Oxidation (M)[4]		Mascot
1021.5312	1021.4023	-0.1289	-126	23	31	DTDILAAFR					Mascot
1023.4741	1023.3226	-0.1515	-148	341	348	DDFVEKDR					Mascot
1204.6071	1204.4578	-0.1493	-124	208	217	FLFCAEAIYK					Mascot
1275.7307	1275.5613	-0.1694	-133	330	340	EITLGFVDLLR					Mascot
1407.6678	1407.5081	-0.1597	-113	12	22	LTYYTPEYETK					Mascot
1465.7546	1465.5535	-0.2011	-137	137	149	TFQGPPHGIQVER					Mascot
1465.7546	1465.5535	-0.2011	-137	137	149	TFQGPPHGIQVER	15	0			Mascot
1502.8512	1502.6492	-0.202	-134	155	167	YGRPLLGCTIKPK			Carbamidomethyl (C)[8]		Mascot
1794.8149	1794.5659	-0.249	-139	227	242	GHYLNATAGTCEEMIK			Carbamidomethyl (C)[11]		Mascot
2185.9819	2185.6653	-0.3166	-145	185	203	GGLDFTKDDENVNSQPFMR			Oxidation (M)[18]		Mascot

6	Ribulose bisphosphate carboxylase large chain (Fragment) OS=Clitoria ternatea GN=rbcL PE=3 SV=1	RBL_CLITE	51460	6.23	12	73	99.846	15	0

Peptide Information

Calc. Mass	Obsrv. Mass	± da	± ppm	Start Seq.	End Seq.	Sequence	Ion Score	C. I. %	Modification	Rank	Result Type
805.4929	805.3817	-0.1112	-138	1	8	VGFKAGVK					Mascot
805.4929	805.3817	-0.1112	-138	1	8	VGFKAGVK					Mascot
910.4451	910.3134	-0.1317	-145	178	184	AVYECLR			Carbamidomethyl (C)[5]		Mascot
914.4049	914.2849	-0.12	-131	296	302	NHGMHFR			Oxidation (M)[4]		Mascot
1021.5312	1021.4023	-0.1289	-126	23	31	DTDILAAFR					Mascot
1023.4741	1023.3226	-0.1515	-148	341	348	DDFVEKDR					Mascot
1204.6071	1204.4578	-0.1493	-124	208	217	FLFCAEAIYK					Mascot
1275.7307	1275.5613	-0.1694	-133	330	340	EITLGFVDLLR					Mascot
1407.6678	1407.5081	-0.1597	-113	12	22	LTYYTPEYETK					Mascot
1465.7546	1465.5535	-0.2011	-137	137	149	TFQGPPHGIQVER					Mascot
1465.7546	1465.5535	-0.2011	-137	137	149	TFQGPPHGIQVER	15	0			Mascot
1502.8512	1502.6492	-0.202	-134	155	167	YGRPLLGCTIKPK			Carbamidomethyl (C)[8]		Mascot
1794.8149	1794.5659	-0.249	-139	227	242	GHYLNATAGTCEEMIK			Carbamidomethyl (C)[11]		Mascot
2185.9819	2185.6653	-0.3166	-145	185	203	GGLDFTKDDENVNSQPFMR			Oxidation (M)[18]		Mascot

7	Ribulose bisphosphate carboxylase large chain (Fragment) OS=Sanguinaria canadensis GN=rbcL PE=3 SV=	RBL_SANCA	48270.4	6.46	12	73	99.839	13	0

Peptide Information

Calc. Mass	Obsrv. Mass	± da	± ppm	Start Seq.	End Seq.	Sequence	Ion Score	C. I. %	Modification	Rank	Result Type
805.4929	805.3817	-0.1112	-138	1	8	VGFKAGVK					Mascot
805.4929	805.3817	-0.1112	-138	1	8	VGFKAGVK					Mascot
910.4451	910.3134	-0.1317	-145	178	184	AVYECLR			Carbamidomethyl (C)[5]		Mascot
914.4049	914.2849	-0.12	-131	296	302	NHGMHFR			Oxidation (M)[4]		Mascot
1021.5312	1021.4023	-0.1289	-126	23	31	DTDILAAFR					Mascot
1023.4741	1023.3226	-0.1515	-148	341	348	DDFVEKDR					Mascot
1204.6071	1204.4578	-0.1493	-124	208	217	FLFCAEAIYK					Mascot
1275.7307	1275.5613	-0.1694	-133	330	340	EITLGFVDLLR					Mascot
1465.7216	1465.5535	-0.1681	-115	310	324	MSGGDHIHAGTVVGK					Mascot
1465.7546	1465.5535	-0.2011	-137	137	149	TFQGPPHGIQVER	15	0			Mascot
1502.8512	1502.6492	-0.202	-134	155	167	YGRPLLGCTIKPK			Carbamidomethyl (C)[8]		Mascot
1794.8149	1794.5659	-0.249	-139	227	242	GHYLNATAGTCEEMIK			Carbamidomethyl (C)[11]		Mascot
2185.9819	2185.6653	-0.3166	-145	185	203	GGLDFTKDDENVNSQPFMR			Oxidation (M)[18]		Mascot

8	Ribulose bisphosphate carboxylase large chain (Fragment) OS=Ilex crenata GN=rbcL PE=3 SV=1	RBL_ILECR	51518	6.1	12	72	99.762	13	0

Peptide Information

Calc. Mass	Obsrv. Mass	± da	± ppm	Start Seq.	End Seq.	Sequence	Ion Score	C. I. %	Modification	Rank	Result Type
805.4929	805.3817	-0.1112	-138	1	8	VGFKAGVK					Mascot
805.4929	805.3817	-0.1112	-138	1	8	VGFKAGVK					Mascot
910.4451	910.3134	-0.1317	-145	178	184	AVYECLR			Carbamidomethyl (C)[5]		Mascot
914.4049	914.2849	-0.12	-131	296	302	NHGMHFR			Oxidation (M)[4]		Mascot
1021.5312	1021.4023	-0.1289	-126	23	31	DTDILAAFR					Mascot
1023.4741	1023.3226	-0.1515	-148	341	348	DDFVEKDR					Mascot
1275.7307	1275.5613	-0.1694	-133	330	340	EITLGFVDLLR					Mascot
1465.7216	1465.5535	-0.1681	-115	310	324	MSGGDHIHAGTVVGK					Mascot
1465.7546	1465.5535	-0.2011	-137	137	149	TFQGPPHGIQVER	15	0			Mascot
1487.7086	1487.5371	-0.1715	-115	454	465	EIKFEFQAMDTL			Oxidation (M)[9]		Mascot
1502.8512	1502.6492	-0.202	-134	155	167	YGRPLLGCTIKPK			Carbamidomethyl (C)[8]		Mascot
1794.8149	1794.5659	-0.249	-139	227	242	GHYLNATAGTCEEMIK			Carbamidomethyl (C)[11]		Mascot
2185.9819	2185.6653	-0.3166	-145	185	203	GGLDFTKDDENVNSQPFMR			Oxidation (M)[18]		Mascot

9	Ribulose bisphosphate carboxylase large chain (Fragment) OS=Hamamelis mollis GN=rbcL PE=3 SV=1	RBL_HAMMO	51542.9	6.05	12	71	99.751	13	0

Peptide Information

Calc. Mass	Obsrv. Mass	± da	± ppm	Start Seq.	End Seq.	Sequence	Ion Score	C. I. %	Modification	Rank	Result Type
805.4929	805.3817	-0.1112	-138	1	8	VGFKAGVK					Mascot
805.4929	805.3817	-0.1112	-138	1	8	VGFKAGVK					Mascot
910.4451	910.3134	-0.1317	-145	178	184	AVYECLR			Carbamidomethyl (C)[5]		Mascot
914.4049	914.2849	-0.12	-131	296	302	NHGMHFR			Oxidation (M)[4]		Mascot
1021.5312	1021.4023	-0.1289	-126	23	31	DTDILAAFR					Mascot
1204.6071	1204.4578	-0.1493	-124	208	217	FLFCAEAIYK					Mascot
1275.7307	1275.5613	-0.1694	-133	330	340	EITLGFVDLLR					Mascot
1407.6678	1407.5081	-0.1597	-113	12	22	LTYYTPEYETK					Mascot
1465.7216	1465.5535	-0.1681	-115	310	324	MSGGDHIHAGTVVGK					Mascot
1465.7546	1465.5535	-0.2011	-137	137	149	TFQGPPHGIQVER	15	0			Mascot
1502.8512	1502.6492	-0.202	-134	155	167	YGRPLLGCTIKPK			Carbamidomethyl (C)[8]		Mascot
1794.8149	1794.5659	-0.249	-139	227	242	GHYLNATAGTCEEMIK			Carbamidomethyl (C)[11]		Mascot
2185.9819	2185.6653	-0.3166	-145	185	203	GGLDFTKDDENVNSQPFMR			Oxidation (M)[18]		Mascot

10	Ribulose bisphosphate carboxylase large chain (Fragment) OS=Nyssa ogeche GN=rbcL PE=3 SV=2	RBL_NYSOG	51012.7	6.13	12	71	99.72	13	0

Peptide Information

Calc. Mass	Obsrv. Mass	± da	± ppm	Start Seq.	End Seq.	Sequence	Ion Score	C. I. %	Modification	Rank	Result Type
805.4929	805.3817	-0.1112	-138	1	8	VGFKAGVK					Mascot
805.4929	805.3817	-0.1112	-138	1	8	VGFKAGVK					Mascot
910.4451	910.3134	-0.1317	-145	178	184	AVYECLR			Carbamidomethyl (C)[5]		Mascot
914.4049	914.2849	-0.12	-131	296	302	NHGMHFR			Oxidation (M)[4]		Mascot
1021.5312	1021.4023	-0.1289	-126	23	31	DTDILAAFR					Mascot
1204.6071	1204.4578	-0.1493	-124	208	217	FLFCAEAIYK					Mascot
1275.7307	1275.5613	-0.1694	-133	330	340	EITLGFVDLLR					Mascot
1465.7216	1465.5535	-0.1681	-115	310	324	MSGGDHIHAGTVVGK					Mascot
1465.7546	1465.5535	-0.2011	-137	137	149	TFQGPPHGIQVER	15	0			Mascot
1502.8512	1502.6492	-0.202	-134	155	167	YGRPLLGCTIKPK			Carbamidomethyl (C)[8]		Mascot
1560.7515	1560.5289	-0.2226	-143	441	453	WSPELAAACEIWK			Carbamidomethyl (C)[9]		Mascot
1741.7343	1741.6652	-0.0691	-40	227	242	GHYLNATGTCEDMMK					Mascot
2185.9819	2185.6653	-0.3166	-145	185	203	GGLDFTKDDENVNSQPFMR			Oxidation (M)[18]		Mascot

Gel Idx/Pos	103/E2	Instr./Gel Origin	BA2060/131017A	Process Sta	Analysis Succeeded
Plate [#] Name	[1] 31496	Instrument Sample Name		Spectra	8

Rank	Protein Name	Accession No	Protein MW	Protein PI	Pep. Count	Protein Score	Protein Score C. I. %	Total Ion Score	Total Ion C. I. %
1	Oxygen-evolving enhancer protein 2-1, chloroplast OS=Nicotiana tabacum GN=PSBP1 PE=3 SV=2	PSBP1_TOB/	28634.4	6.84	6	114	100	86	100

Peptide Information

Calc. Mass	Obsrv. Mass	± da	± ppm	Start Seq.	End Seq.	Sequence	Ion Score	C. I. %	Modification	Rank	Result Type
1590.707	1590.5004	-0.2066	-130	96	109	ENTDFLAYNGDGFK					Mascot
1703.7831	1703.5488	-0.2343	-138	45	60	QSSPQEDDGNSVVVSR					Mascot
1891.0217	1890.7507	-0.271	-143	29	44	YVSSLKPNQLVCRAQK			Carbamidomethyl (C)[12]		Mascot
1891.9548	1891.7573	-0.1975	-104	77	95	VSPADAAYGEAANVFGKPK					Mascot
2259.0664	2258.7749	-0.2915	-129	131	150	YEDNFDSTSNLIVTVTPTDK	32	96.335			Mascot
2259.0664	2258.7749	-0.2915	-129	131	150	YEDNFDSTSNLIVTVTPTDK					Mascot
2387.1614	2386.8528	-0.3086	-129	131	151	YEDNFDSTSNLIVTVTPTDKK	54	99.975			Mascot
2387.1614	2386.8528	-0.3086	-129	131	151	YEDNFDSTSNLIVTVTPTDKK					Mascot

2	Oxygen-evolving enhancer protein 2, chloroplastic OS=Pisum sativum GN=PSBP PE=1 SV=1	PSBP_PEA	28030.3	8.29	5	100	100	77	100

Peptide Information

Calc. Mass	Obsrv. Mass	± da	± ppm	Start Seq.	End Seq.	Sequence	Ion Score	C. I. %	Modification	Rank	Result Type
807.461	807.3584	-0.1026	-127	157	163	VDYLLGK					Mascot
1572.7428	1572.5383	-0.2045	-130	143	156	SITDYGSPEEFLSK					Mascot
1572.7428	1572.5383	-0.2045	-130	143	156	SITDYGSPEEFLSK	77	100			Mascot
1865.9391	1865.6659	-0.2732	-146	68	86	VSPADAAYGEAANVFGKAK					Mascot
2263.0747	2262.8096	-0.2651	-117	1	19	MASTQCFLHHQYAITTPTR			Carbamidomethyl (C)[6]		Mascot
2807.3716	2806.9622	-0.4094	-146	1	24	MASTQCFLHHQYAITTPTRTLSQR			Oxidation (M)[1]		Mascot

3	Oxygen-evolving enhancer protein 2, chloroplastic OS=Solanum tuberosum GN=PSBP PE=2 SV=1	PSBP_SOLTU	27987.1	8.27	5	99	100	77	100

Peptide Information

Calc. Mass	Obsrv. Mass	± da	± ppm	Start Seq.	End Seq.	Sequence	Ion Score	C. I. %	Modification	Rank	Result Type
807.461	807.3584	-0.1026	-127	158	164	VDYLLGK					Mascot
1391.6146	1391.5216	-0.093	-67	40	52	QDDANNTSNAVSR					Mascot
1572.7428	1572.5383	-0.2045	-130	144	157	SITDYGSPEEFLSK					Mascot
1572.7428	1572.5383	-0.2045	-130	144	157	SITDYGSPEEFLSK	77	100			Mascot
1891.9548	1891.7573	-0.1975	-104	69	87	VSPADAAYGEAANVFGKPK					Mascot
2263.0071	2262.8096	-0.1975	-87	123	142	YEDNFDSTSNLMVAVTPTDK			Oxidation (M)[12]		Mascot

4	Oxygen-evolving enhancer protein 2, chloroplastic OS=Solanum lycopersicum GN=PSBP PE=2 SV=1	PSBP_SOLLC	27775	8.26	5	99	100	77	100

Peptide Information

Calc. Mass	Obsrv. Mass	± da	± ppm	Start Seq.	End Seq.	Sequence	Ion Score	C. I. %	Modification	Rank	Result Type
807.461	807.3584	-0.1026	-127	156	162	VDYLLGK					Mascot
1133.5182	1133.478	-0.0402	-35	40	50	QDDASNAAVSR					Mascot
1189.6211	1189.4692	-0.1519	-128	111	120	EVEYPGQVLR					Mascot
1572.7428	1572.5383	-0.2045	-130	142	155	SITDYGSPEEFLSK					Mascot
1572.7428	1572.5383	-0.2045	-130	142	155	SITDYGSPEEFLSK	77	100			Mascot
1891.9548	1891.7573	-0.1975	-104	67	85	VSPADAAYGEAANVFGKPK					Mascot

5	ATP-dependent Clp protease proteolytic subunit : chloroplastic OS=Arabidopsis thaliana GN=CLPP3 PE	CLPP3_ARAT	33903.1	7.6	9	41	0		

Peptide Information

Calc. Mass	Obsrv. Mass	± da	± ppm	Start Seq.	End Seq.	Sequence	Ion Score	C. I. %	Modification	Rank	Result Type
807.4029	807.3584	-0.0445	-55	199	205	ATEMSIR					Mascot
842.3535	842.4042	0.0507	60	179	185	YCMPNSK					Mascot
870.3484	870.431	0.0826	95	208	213	EMMYHK			Oxidation (M)[2,3]		Mascot
998.4546	998.438	-0.0166	-17	178	185	RYCMPNSK					Mascot
1045.4908	1045.433	-0.0578	-55	290	298	DNTNLPSER					Mascot
1139.5336	1139.4044	-0.1292	-113	206	213	IREMMYHK			Oxidation (M)[4,5]		Mascot
1275.7242	1275.5637	-0.1605	-126	34	44	IPKTSKPFCVR					Mascot
2210.9983	2210.8132	-0.1851	-84	290	309	DNTNLPSERSMTQNGYAAIE					Mascot
2758.3572	2758.0754	-0.2818	-102	149	175	QCKADVSTVCLGLAASMGAFLLASGSK			Carbamidomethyl (C)[2,10], Oxidation (M)[17]		Mascot

6	Heat stress transcription factor C-1b OS=Oryza sa subsp. japonica GN=HSFC1B PE=2 SV=1	HFC1B_ORYS	27201.7	8.93	7	34	0		

Peptide Information

Calc. Mass	Obsrv. Mass	± da	± ppm	Start Seq.	End Seq.	Sequence	Ion Score	C. I. %	Modification	Rank	Result Type
882.5155	882.452	-0.0635	-72	103	110	GQAQLLPR					Mascot
998.5053	998.438	-0.0673	-67	79	86	QLNTYGFR					Mascot
1133.5963	1133.478	-0.1183	-104	70	78	HRNFASFVR					Mascot
1249.5365	1249.437	-0.0995	-80	126	135	ELCEEGEEVR			Carbamidomethyl (C)[3]		Mascot
1598.8934	1598.6614	-0.232	-145	179	193	LADEPGVVLRAMLAK			Oxidation (M)[12]		Mascot
2595.2805	2594.9106	-0.3699	-143	2	28	MGGECKVHQLQAAGDGGPGAVAPFVAK					Mascot
2758.3108	2758.0754	-0.2354	-85	1	28	MMGGECKVHQLQAAGDGGPGAVAPFVAK			Oxidation (M)[1,2]		Mascot

7	Acyl carrier protein 2, chloroplastic OS=Spinacia oleracea GN=ACL1.2 PE=1 SV=1	ACP2_SPIOL	14089.1	4.78	5	34	0		

Peptide Information

Calc. Mass	Obsrv. Mass	± da	± ppm	Start Seq.	End Seq.	Sequence	Ion Score	C. I. %	Modification	Rank	Result Type
856.4821	856.4174	-0.0647	-76	36	42	RTPVMPR					Mascot
882.4468	882.452	0.0052	6	29	35	SSVTFWR					Mascot
1045.5524	1045.433	-0.1194	-114	64	73	SQLALAEDAK					Mascot
1577.8025	1577.603	-0.1995	-126	43	57	GLSVSCAAKPEMVTK			Carbamidomethyl (C)[6]		Mascot
2434.1741	2433.9421	-0.232	-95	1	23	MASITGSSVSFKCAPLQSSFNSK			Carbamidomethyl (C)[13]		Mascot

8	Metallothionein-like protein type 2, MT2-4/MT2-25 OS=Brassica juncea PE=3 SV=1	MT21_BRAJU	8055.2	4.55	5	34	0		

Peptide Information

Calc. Mass	Obsrv. Mass	± da	± ppm	Start Seq.	End Seq.	Sequence	Ion Score	C. I. %	Modification	Rank	Result Type
882.3266	882.452	0.1254	142	74	80	CNPCTCK			Carbamidomethyl (C)[1,4]		Mascot
1292.3944	1292.5046	0.1102	85	2	15	SCCGGNCGCGSGCK			Carbamidomethyl (C)[2]		Mascot
1463.4589	1463.5648	0.1059	72	2	15	SCCGGNCGCGSGCK			Carbamidomethyl (C)[2,3,7,9]		Mascot
1589.5634	1589.5048	-0.0586	-37	68	80	CGSDCKCNPCTCK			Carbamidomethyl (C)[1,5,7,10]		Mascot
1589.5634	1589.5048	-0.0586	-37	68	80	CGSDCKCNPCTCK			Carbamidomethyl (C)[1,5,7,10]		Mascot
1594.4993	1594.5068	0.0075	5	1	15	MSCCGGNCGCGSGCK			Carbamidomethyl (C)[3,4,8,10]		Mascot
2056.6711	2056.7554	0.0843	41	2	23	SCCGGNCGCGSGCKCVGCGGCK			Carbamidomethyl (C)[2,3]		Mascot

9	F-box only protein 7 OS=Arabidopsis thaliana GN=FBX7 PE=2 SV=3	FBX7_ARATH	46788.7	9.23	9	33	0		

Peptide Information

Calc. Mass	Obsrv. Mass	± da	± ppm	Start Seq.	End Seq.	Sequence	Ion Score	C. I. %	Modification	Rank	Result Type
870.3696	870.431	0.0614	71	2	9	GMTTDSMK					Mascot
998.4645	998.438	-0.0265	-27	2	10	GMTTDSMKK					Mascot
1001.41	1001.4439	0.0339	34	1	9	MGMTTDSMK					Mascot
1133.468	1133.478	0.01	9	16	24	MSENHDWSK					Mascot
1161.4949	1161.4442	-0.0507	-44	1	10	MGMTTDSMKK			Oxidation (M)[1,3]		Mascot
1275.5674	1275.5637	-0.0037	-3	350	360	SGSICFVEYDR					Mascot
1590.7152	1590.5004	-0.2148	-135	157	169	SRYICFDNCNSIR					Mascot
2387.197	2386.8528	-0.3442	-144	190	208	TGDYFVAWIFNKHYLFTHK					Mascot
2387.197	2386.8528	-0.3442	-144	190	208	TGDYFVAWIFNKHYLFTHK					Mascot
2623.2498	2623.0581	-0.1917	-73	350	373	SGSICFVEYDRQPFSGVFDLATGK					Mascot

10	Serine carboxypeptidase-like 47 OS=Arabidopsis thaliana GN=SCPL47 PE=2 SV=1	SCP47_ARAT	56507.1	7.05	9	32	0		

Peptide Information

Calc. Mass	Obsrv. Mass	± da	± ppm	Start Seq.	End Seq.	Sequence	Ion Score	C. I. %	Modification	Rank	Result Type
836.4335	836.3246	-0.1089	-130	49	55	SFNLMPK					Mascot
1249.5339	1249.437	-0.0969	-78	344	354	CVGSLCYDFSR					Mascot
1515.7089	1515.6008	-0.1081	-71	113	123	MFYFFFESRNK					Mascot
1602.7404	1602.6553	-0.0851	-53	468	481	VYNAGHMVPMDQPK			Oxidation (M)[7]		Mascot
2239.0344	2238.8418	-0.1926	-86	344	361	CVGSLCYDFSRMEIFLNK			Carbamidomethyl (C)[1,6]		Mascot
2259.0454	2258.7749	-0.2705	-120	306	327	CSLGGGLVCDSAVEVCTSIFNK			Carbamidomethyl (C)[1]		Mascot
2259.0454	2258.7749	-0.2705	-120	306	327	CSLGGGLVCDSAVEVCTSIFNK			Carbamidomethyl (C)[1]		Mascot
2387.1404	2386.8528	-0.2876	-120	305	327	KCSLGGGLVCDSAVEVCTSIFNK			Carbamidomethyl (C)[2]		Mascot
2387.1404	2386.8528	-0.2876	-120	305	327	KCSLGGGLVCDSAVEVCTSIFNK			Carbamidomethyl (C)[2]		Mascot
2595.2263	2594.9106	-0.3157	-122	86	109	NASVGPSVQEFGHYAGYYSLPHSK					Mascot
2807.3345	2806.9622	-0.3723	-133	257	282	GFAIGNGLTNPEIQYGAYGDYALQMK			Oxidation (M)[25]		Mascot

Gel Idx/Pos	104/E3	Instr./Gel Origin	BA2060/131017A	Process State	Analysis Succeeded
Plate [#] Name	[1] 31496	Instrument Sample Name		Spectra	8

Rank	Protein Name	Accession No	Protein MW	Protein PI	Pep. Count	Protein Score	Protein Score C. I. %	Total Ion Score	Total Ion C. I. %
1	Chlorophyll a-b binding protein 3, chloroplastic OS=Pisum sativum GN=lhca3 PE=1 SV=1	CB23_PEA	29588.2	8.84	3	51	72.654	41	99.414

Peptide Information

Calc. Mass	Obsrv. Mass	± da	± ppm	Start Seq.	End Seq.	Sequence	Ion Score	C. I. %	Modification	Rank	Result Type
997.5352	997.4033	-0.1319	-132	185	192	QYFLGLEK	41	99.414			Mascot
997.5352	997.4033	-0.1319	-132	185	192	QYFLGLEK					Mascot
1351.6464	1351.4694	-0.177	-131	173	184	FQDWAKPGSMGK					Mascot
1367.6412	1367.4584	-0.1828	-134	173	184	FQDWAKPGSMGK			Oxidation (M)[10]		Mascot
1523.7423	1523.5425	-0.1998	-131	172	184	RFQDWAKPGSMGK			Oxidation (M)[11]		Mascot

Rank	Protein Name	Accession No	Protein MW	Protein PI	Pep. Count	Protein Score	Protein Score C. I. %	Total Ion Score	Total Ion C. I. %
2	Homeobox-leucine zipper protein HOX18 OS=Oryz sativa subsp. indica GN=HOX18 PE=2 SV=1	HOX18_ORYS	27577.7	9.13	8	45	0		

Peptide Information

Calc. Mass	Obsrv. Mass	± da	± ppm	Start Seq.	End Seq.	Sequence	Ion Score	C. I. %	Modification	Rank	Result Type
950.4061	950.361	-0.0451	-47	57	65	DEEQASGSK					Mascot
1105.5344	1105.3965	-0.1379	-125	80	90	SIRNSGGDGSR					Mascot
1165.5518	1165.5875	0.0357	31	237	249	VTVMGGGGGETGK			Oxidation (M)[4]		Mascot
1367.6624	1367.4584	-0.204	-149	171	181	QTEVDCEFLKR					Mascot
1892.0024	1891.7745	-0.2279	-120	203	221	LASAAAAAAGSQLYVQFPR					Mascot
1908.0073	1907.7709	-0.2364	-124	117	132	LQLTKEQSTLLEDSFR					Mascot
2595.2034	2594.9114	-0.292	-113	222	249	AAAAAMVNVCPSCEKVTVMGGGGGETGK					Mascot
2595.2034	2594.9114	-0.292	-113	222	249	AAAAAMVNVCPSCEKVTVMGGGGGETGK					Mascot
3337.6489	3337.3547	-0.2942	-88	203	236	LASAAAAAAGSQLYVQFPRAAAAAMVNVCPSCEK					Mascot

Rank	Protein Name	Accession No	Protein MW	Protein PI	Pep. Count	Protein Score	Protein Score C. I. %	Total Ion Score	Total Ion C. I. %
3	Probable UDP-arabinose 4-epimerase 3 OS=Oryz sativa subsp. japonica GN=UEL-3 PE=2 SV=1	ARAE3_ORYS	44799.1	9.11	8	34	0		

Peptide Information

Calc. Mass	Obsrv. Mass	± da	± ppm	Start Seq.	End Seq.	Sequence	Ion Score	C. I. %	Modification	Rank	Result Type
980.4903	980.3781	-0.1122	-114	233	240	DMAVMILR			Oxidation (M)[2,5]		Mascot
1045.4408	1045.4292	-0.0116	-11	13	21	GGMEYFDAR					Mascot
1295.6552	1295.5065	-0.1487	-115	219	229	MAEDIILDFTK					Mascot
1399.6158	1399.4553	-0.1605	-115	286	298	GTDYPTTDGTCIR					Mascot
1602.801	1602.6569	-0.1441	-90	299	312	DYIDVTDLVDAHVK					Mascot
1673.9003	1673.7048	-0.1955	-117	90	105	VTIVDNLSRGNMGAVK					Mascot
1689.8951	1689.699	-0.1961	-116	90	105	VTIVDNLSRGNMGAVK			Oxidation (M)[12]		Mascot
2299.1208	2298.8779	-0.2429	-106	233	252	DMAVMILRYFNVIGSDPEGR			Oxidation (M)[2]		Mascot
3510.7725	3511.2468	0.4743	135	129	160	TVNKIFAENAFDAVMHFAAVAYVGESTLEPLR					Mascot

Rank	Protein Name	Accession No	Protein MW	Protein PI	Pep. Count	Protein Score	Protein Score C. I. %	Total Ion Score	Total Ion C. I. %
4	Ribulose bisphosphate carboxylase small chain (512 (Fragment) OS=Triticum aestivum PE=3 SV=1	RBS3 WHEA	13046.5	5.84	5	33	0		

Peptide Information

Calc. Mass	Obsrv. Mass	± da	± ppm	Start Seq.	End Seq.	Sequence	Ion Score	C. I. %	Modification	Rank	Result Type
1165.571	1165.5875	0.0165	14	23	31	WVPCLEFSK			Carbamidomethyl (C)[4]		Mascot
2211.0571	2210.8098	-0.2473	-112	93	112	QVQCVSFIAFKPPGCEESGK			Carbamidomethyl (C)[4]		Mascot
2223.0671	2222.7935	-0.2736	-123	56	75	LPMFGCTDATQVINEVEEVK					Mascot
2223.0671	2222.7935	-0.2736	-123	56	75	LPMFGCTDATQVINEVEEVK					Mascot
2225.073	2224.8076	-0.2654	-119	93	113	QVQCVSFIAFKPPGCEESGKA					Mascot
2239.062	2238.8367	-0.2253	-101	56	75	LPMFGCTDATQVINEVEEVK			Oxidation (M)[3]		Mascot
2260.9868	2260.7285	-0.2583	-114	38	55	EHNASPGYYDGRYWTMWK					Mascot

Rank	Protein Name	Accession No	Protein MW	Protein PI	Pep. Count	Protein Score	Protein Score C. I. %	Total Ion Score	Total Ion C. I. %
5	Ras-related protein RABA2a OS=Arabidopsis tha GN=RABA2A PE=2 SV=1	RAA2A ARAT	24093.3	6.2	6	32	0		

Peptide Information

Calc. Mass	Obsrv. Mass	± da	± ppm	Start Seq.	End Seq.	Sequence	Ion Score	C. I. %	Modification	Rank	Result Type
882.5155	882.4489	-0.0666	-75	127	133	TDLKHLR					Mascot
1274.6124	1274.4846	-0.1278	-100	63	73	AQIWDTAGQER					Mascot
1373.6517	1373.481	-0.1707	-124	32	42	FTRNEFCLESK					Mascot
1602.8235	1602.6569	-0.1666	-104	60	73	TVKAQIWDTAGQER					Mascot
1620.7711	1620.6504	-0.1207	-74	195	210	EGQTIDVAATSESNAK					Mascot
2313.0332	2312.8875	-0.1457	-63	195	217	EGQTIDVAATSESNAKKPCCSSS					Mascot

Rank	Protein Name	Accession No	Protein MW	Protein PI	Pep. Count	Protein Score	Protein Score C. I. %	Total Ion Score	Total Ion C. I. %
6	Fructose-1,6-bisphosphatase, cytosolic OS=Spin oleracea PE=1 SV=2	F16P2 SPIOL	37174.7	5.52	7	32	0		

Peptide Information

Calc. Mass	Obsrv. Mass	± da	± ppm	Start Seq.	End Seq.	Sequence	Ion Score	C. I. %	Modification	Rank	Result Type
870.5294	870.4288	-0.1006	-116	305	312	ALDLIPTK					Mascot
950.4976	950.361	-0.1366	-144	10	17	TDLMTITR					Mascot
980.5046	980.3781	-0.1265	-129	18	25	YVLNEQSK					Mascot
1291.6641	1291.5123	-0.1518	-118	235	246	FPTDGSSPKSLR					Mascot
1291.6641	1291.5123	-0.1518	-118	235	246	FPTDGSSPKSLR					Mascot
1662.8221	1662.6622	-0.1599	-96	146	160	DFETATLEDVLQPGK					Mascot
2298.2385	2297.9026	-0.3359	-146	26	45	RPESRGDFTILLSHIVLGCK			Carbamidomethyl (C)[19]		Mascot
3511.6946	3511.2468	-0.4478	-128	114	145	GKYCVVFDPLDGSSNIDCGVSIGTIFGIYMVK			Carbamidomethyl (C)[4,18]		Mascot
3527.6895	3528.2102	0.5207	148	114	145	GKYCVVFDPLDGSSNIDCGVSIGTIFGIYMVK			Carbamidomethyl (C)[4,18], Oxidation (M)[30]		Mascot

Rank	Protein Name	Accession No	Protein MW	Protein PI	Pep. Count	Protein Score	Protein Score C. I. %	Total Ion Score	Total Ion C. I. %
7	Metallothionein-like protein type 2, MT2-4/MT2-25 OS=Brassica juncea PE=3 SV=1	MT21 BRAJU	8055.2	4.55	5	32	0		

Peptide Information

Calc. Mass	Obsrv. Mass	± da	± ppm	Start Seq.	End Seq.	Sequence	Ion Score	C. I. %	Modification	Rank	Result Type
882.3266	882.4489	0.1223	139	74	80	CNPCTCK			Carbamidomethyl (C)[1,4]		Mascot
1292.3944	1292.5057	0.1113	86	2	15	SCCGGNCGCGSGCK			Carbamidomethyl (C)[2]		Mascot
1406.4374	1406.4924	0.055	39	2	15	SCCGGNCGCGSGCK			Carbamidomethyl (C)[2,3,7]		Mascot
1423.4349	1423.5068	0.0719	51	1	15	MSCCGGNCGCGSGCK			Carbamidomethyl (C)[3]		Mascot
2056.6711	2056.7629	0.0918	45	2	23	SCCGGNCGCGSGCKCVGCGGCK			Carbamidomethyl (C)[2,3]		Mascot
2260.728	2260.7285	0.0005	0	1	23	MSCCGGNCGCGSGCKCVGCGGCK			Carbamidomethyl (C)[3,4,8], Oxidation (M)[1]		Mascot

Rank	Protein Name	Accession No	Protein MW	Protein PI	Pep. Count	Protein Score	Protein Score C. I. %	Total Ion Score	Total Ion C. I. %
8	Ras-related protein RABA4b OS=Arabidopsis tha GN=RABA4B PE=2 SV=1	RAA4B ARAT	24391.3	5.82	6	32	0		

Peptide Information

Calc. Mass	Obsrv. Mass	± da	± ppm	Start Seq.	End Seq.	Sequence	Ion Score	C. I. %	Modification	Rank	Result Type
1274.6124	1274.4846	-0.1278	-100	68	78	AQIWDTAGQER					Mascot
1602.8235	1602.6569	-0.1666	-104	65	78	SIKAQIWDTAGQER					Mascot
1630.7667	1630.6437	-0.123	-75	186	202	NLASEGDSNNPGSLAGK					Mascot
1673.7977	1673.7048	-0.0929	-56	132	146	SDLEDQRAVPTEDAK					Mascot
1689.823	1689.699	-0.124	-73	2	19	AGGGGYGGASGKVDYVFK					Mascot
2299.1096	2298.8779	-0.2317	-101	81	101	AVTSAYYRGAVGAMLVYDMTK			Oxidation (M)[14,19]		Mascot

Rank	Protein Name	Accession No	Protein MW	Protein PI	Pep. Count	Protein Score	Protein Score C. I. %	Total Ion Score	Total Ion C. I. %
9	Homeobox-leucine zipper protein HOX28 OS=Oryz sativa subsp. japonica GN=HOX28 PE=2 SV=1	HOX28_ORYS	26999.7	9.12	7	31	0		

Peptide Information

Calc. Mass	Obsrv. Mass	± da	± ppm	Start Seq.	End Seq.	Sequence	Ion Score	C. I. %	Modification	Rank	Result Type
1292.5325	1292.5057	-0.0268	-21	161	170	WCDQLADDNR			Carbamidomethyl (C)[2]		Mascot
1367.626	1367.4584	-0.1676	-123	55	69	FACSPGSPVSSGSGK					Mascot
1391.6121	1391.5261	-0.086	-62	160	170	RWCDQLADDNR					Mascot
1391.6121	1391.5261	-0.086	-62	161	171	WCDQLADDNRR					Mascot
1523.7271	1523.5425	-0.1846	-121	55	70	FACSPGSPVSSGSGKR					Mascot
2225.1018	2224.8076	-0.2942	-132	101	119	DQAAVLEECFKTHHTLTPK			Carbamidomethyl (C)[9]		Mascot
2299.1494	2298.8779	-0.2715	-118	184	205	ATPTPPAAAPPLTTLTMCLSCK			Carbamidomethyl (C)[18,21]		Mascot

Rank	Protein Name	Accession No	Protein MW	Protein PI	Pep. Count	Protein Score	Protein Score C. I. %	Total Ion Score	Total Ion C. I. %
10	(2Z,6Z)-farnesyl diphosphate synthase, chloroplas OS=Solanum habrochaites GN=ZFPS PE=1 SV=1	ZFPS SOLHA	34407.5	6.01	7	30	0		

Peptide Information

Calc. Mass	Obsrv. Mass	± da	± ppm	Start Seq.	End Seq.	Sequence	Ion Score	C. I. %	Modification	Rank	Result Type
882.5559	882.4489	-0.107	-121	105	111	HLFPKLK					Mascot
1165.5769	1165.5875	0.0106	9	179	188	CIALTEETTK			Carbamidomethyl (C)[1]		Mascot
1295.7001	1295.5065	-0.1936	-149	80	90	HIALIMDGNRR					Mascot
1407.7148	1407.5103	-0.2045	-145	179	191	CIALTEETTKGNK					Mascot
1620.8625	1620.6504	-0.2121	-131	48	62	GLNKISCSLSLQTEK					Mascot
3249.4497	3249.2485	-0.2012	-62	63	89	LCYEDNDNDLDEELMPKHIALIMDGNR			Carbamidomethyl (C)[2], Oxidation (M)[15]		Mascot
3354.7258	3354.3118	-0.414	-123	1	30	MSSLVLQCWKLSSPSLILQQNTSISMGAFK			Carbamidomethyl (C)[8]		Mascot

Gel Idx/Pos	105/E4	Instr./Gel Origin	BA2060/131017A	Process State	Analysis Succeeded
Plate [#] Name	[1] 31496	Instrument Sample Name		Spectra	8

Rank	Protein Name	Accession No	Protein MW	Protein PI	Pep. Count	Protein Score	Protein Score C. I. %	Total Ion Score	Total Ion C. I. %
1	Chlorophyll a-b binding protein 6A, chloroplastic OS=Solanum lycopersicum GN=CAB6A PE=2 SV=1	CB11 SOLLC	26557.4	5.82	5	233	100	209	100

Peptide Information

Calc. Mass	Obsrv. Mass	± da	± ppm	Start Seq.	End Seq.	Sequence	Ion Score	C. I. %	Modification	Rank	Result Type
1043.4939	1043.3601	-0.1338	-128	86	93	ESELIHCR	47	99.864	Carbamidomethyl (C)[7]		Mascot
1043.4939	1043.3601	-0.1338	-128	86	93	ESELIHCR			Carbamidomethyl (C)[7]		Mascot
1371.658	1371.4795	-0.1785	-130	168	180	YPGGAFDPLGYSK	79	100			Mascot
1371.658	1371.4795	-0.1785	-130	168	180	YPGGAFDPLGYSK					Mascot
1499.7528	1499.5541	-0.1987	-132	167	180	KYPGGAFDPLGYSK	83	100			Mascot
1499.7528	1499.5541	-0.1987	-132	167	180	KYPGGAFDPLGYSK					Mascot
2230.0552	2229.9089	-0.1463	-66	2	23	ASNTLMSCGIPAVCPSFLSSTK			Oxidation (M)[6]		Mascot
2334.3042	2333.9954	-0.3088	-132	94	115	WAMLAVPGIIVPEALGLGNWVK					Mascot
2350.2991	2349.9802	-0.3189	-136	94	115	WAMLAVPGIIVPEALGLGNWVK			Oxidation (M)[3]		Mascot

Rank	Protein Name	Accession No	Protein MW	Protein PI	Pep. Count	Protein Score	Protein Score C. I. %	Total Ion Score	Total Ion C. I. %
2	Chlorophyll a-b binding protein 6, chloroplastic OS=Arabidopsis thaliana GN=LHCA1 PE=1 SV=1	CAB6 ARATH	25979.3	6.21	3	220	100	209	100

Peptide Information

Calc. Mass	Obsrv. Mass	± da	± ppm	Start Seq.	End Seq.	Sequence	Ion Score	C. I. %	Modification	Rank	Result Type
1043.4939	1043.3601	-0.1338	-128	85	92	ESELIHCR	47	99.864	Carbamidomethyl (C)[7]		Mascot
1043.4939	1043.3601	-0.1338	-128	85	92	ESELIHCR			Carbamidomethyl (C)[7]		Mascot
1371.658	1371.4795	-0.1785	-130	167	179	YPGGAFDPLGYSK	79	100			Mascot
1371.658	1371.4795	-0.1785	-130	167	179	YPGGAFDPLGYSK					Mascot
1499.7528	1499.5541	-0.1987	-132	166	179	KYPGGAFDPLGYSK	83	100			Mascot
1499.7528	1499.5541	-0.1987	-132	166	179	KYPGGAFDPLGYSK					Mascot

Rank	Protein Name	Accession No	Protein MW	Protein PI	Pep. Count	Protein Score	Protein Score C. I. %	Total Ion Score	Total Ion C. I. %
3	Limonene/alpha-pinene synthase, chloroplastic OS=Abies grandis GN=ag11 PE=1 SV=1	TPSDB ABIG	73226.8	6.31	11	35	0		

Peptide Information

Calc. Mass	Obsrv. Mass	± da	± ppm	Start Seq.	End Seq.	Sequence	Ion Score	C. I. %	Modification	Rank	Result Type
842.3824	842.4016	0.0192	23	532	538	GDTRCYK					Mascot
1005.4781	1005.4725	-0.0056	-6	164	172	EKEGIGCGR			Carbamidomethyl (C)[7]		Mascot
1503.7954	1503.5972	-0.1982	-132	319	330	LEFNIFHSLQQK					Mascot
1531.6879	1531.5042	-0.1837	-120	542	555	ARGEEASAISCYMK			Oxidation (M)[13]		Mascot
1531.8447	1532.0046	0.1599	104	29	42	ALCISTAVPTLRMR					Mascot
1920.8176	1920.6671	-0.1505	-78	354	369	HVEYYTMASCISMEPK			Oxidation (M)[7,13]		Mascot
2230.1311	2229.9089	-0.2222	-100	242	260	VMEEAEIFSASYLKEVLQK			Oxidation (M)[2]		Mascot
2239.0093	2238.8279	-0.1814	-81	352	369	HRHVEYYTMASCISMEPK			Carbamidomethyl (C)[12]		Mascot
2350.1455	2349.9802	-0.1653	-70	166	187	EGIGCGRDSTFPDLNSTALALR			Carbamidomethyl (C)[5]		Mascot
3083.6663	3083.9458	0.2795	91	493	519	TATLQPILTLDVPLPNYILQEIDYPSR					Mascot
3111.4182	3110.9707	-0.4475	-144	417	442	CLPEYMKAVYMDLYQCLNELAQEAEK			Oxidation (M)[6]		Mascot

Rank	Protein Name	Accession No	Protein MW	Protein PI	Pep. Count	Protein Score	Protein Score C. I. %	Total Ion Score	Total Ion C. I. %
4	50S ribosomal protein L14, plastid OS=Cuscuta r GN=rpl14 PE=3 SV=1	RK14 CUSRE	13667.3	9.35	2	33	0	22	58.504

Peptide Information

Calc. Mass	Obsrv. Mass	± da	± ppm	Start Seq.	End Seq.	Sequence	Ion Score	C. I. %	Modification	Rank	Result Type
1155.5826	1155.4542	-0.1284	-111	45	54	EAVPNMPLER	22	58.504			Mascot
1155.5826	1155.4542	-0.1284	-111	45	54	EAVPNMPLER					Mascot
3337.6562	3337.3152	-0.341	-102	1	30	MIQPQTHLNVADNSGARELMCIQIIGASNR			Carbamidomethyl (C)[21]		Mascot

Rank	Protein Name	Accession No	Protein MW	Protein PI	Pep. Count	Protein Score	Protein Score C. I. %	Total Ion Score	Total Ion C. I. %
5	Metallothionein-like protein 1A OS=Oryza sativa su japonica GN=MT1A PE=2 SV=1	MT1A ORYSJ	7477.1	6.51	5	33	0		

Peptide Information

Calc. Mass	Obsrv. Mass	± da	± ppm	Start Seq.	End Seq.	Sequence	Ion Score	C. I. %	Modification	Rank	Result Type
824.2484	824.2896	0.0412	50	69	74	CNPCNC			Carbamidomethyl (C)[1,4,6]		Mascot
824.2484	824.2896	0.0412	50	69	74	CNPCNC			Carbamidomethyl (C)[1,4,6]		Mascot
1021.52	1021.4004	-0.1196	-117	20	27	KYPDLEEK					Mascot
1659.4994	1659.6737	0.1743	105	2	19	SCSCGSSCSCGSNCSCGK					Mascot
1920.5778	1920.6671	0.0893	46	1	19	MSCSCGSSCSCGSNCSCGK			Carbamidomethyl (C)[3,5], Oxidation (M)[1]		Mascot
3078.1321	3078.0012	-0.1309	-43	45	74	AQQFEAAAESGETAHGCSCGSSCRCNPCNC			Carbamidomethyl (C)[17]		Mascot

Rank	Protein Name	Accession No	Protein MW	Protein PI	Pep. Count	Protein Score	Protein Score C. I. %	Total Ion Score	Total Ion C. I. %
6	Thioredoxin F, chloroplastic OS=Oryza sativa sub japonica GN=Os01g0913000 PE=2 SV=1	TRXF ORYSJ	19734.3	8.81	5	28	0		

Peptide Information

Calc. Mass	Obsrv. Mass	± da	± ppm	Start Seq.	End Seq.	Sequence	Ion Score	C. I. %	Modification	Rank	Result Type
1005.5403	1005.4725	-0.0678	-67	88	95	DTFWPIVK					Mascot
1814.8274	1814.7021	-0.1253	-69	101	115	VVVLDMYTQWCGPCK			Carbamidomethyl (C)[11], Oxidation (M)[6]		Mascot
2239.0708	2238.8279	-0.2429	-108	96	115	SAGPKVVVLDMYTQWCGPCK			Carbamidomethyl (C)[16]		Mascot
2382.1477	2381.9629	-0.1848	-78	101	120	VVVLDMYTQWCGPCKVMAPK			Carbamidomethyl (C)[11,14]		Mascot
3000.4561	3000.0984	-0.3577	-119	1	29	MALRLSVSSSHGPASSPAISTCRPAACGR			Carbamidomethyl (C)[22,27], Oxidation (M)[1]		Mascot

Rank	Protein Name	Accession No	Protein MW	Protein PI	Pep. Count	Protein Score	Protein Score C. I. %	Total Ion Score	Total Ion C. I. %
7	Putative U-box domain-containing protein 55 OS=Arabidopsis thaliana GN=PUB55 PE=3 SV=1	PUB55 ARAT	65993.7	5.4	11	28	0		

Peptide Information

Calc. Mass	Obsrv. Mass	± da	± ppm	Start Seq.	End Seq.	Sequence	Ion Score	C. I. %	Modification	Rank	Result Type
1005.4921	1005.4725	-0.0196	-19	322	330	VEAEEMLGK					Mascot
1021.4869	1021.4004	-0.0865	-85	322	330	VEAEEMLGK			Oxidation (M)[6]		Mascot
1043.4384	1043.3601	-0.0783	-75	363	370	KEMEESMK			Oxidation (M)[3,7]		Mascot
1043.4384	1043.3601	-0.0783	-75	363	370	KEMEESMK			Oxidation (M)[3,7]		Mascot
1177.5881	1177.4287	-0.1594	-135	321	330	RVEAEEMLGK			Oxidation (M)[7]		Mascot
1335.6572	1335.4868	-0.1704	-128	232	242	QALMEVEESKR			Oxidation (M)[4]		Mascot
1814.8961	1814.7021	-0.194	-107	1	17	MAELMAMGNDVVHVAVK					Mascot
2211.021	2210.8052	-0.2158	-98	111	129	LVMGAASDHHYSEKMFDLK			Oxidation (M)[3,15]		Mascot
2225.1594	2224.8379	-0.3215	-144	461	480	DAASSVCSEILLLLQSYTRR					Mascot
2334.0125	2333.9954	-0.0171	-7	426	444	EEACSVGQNFMRLYEEEAR			Carbamidomethyl (C)[4], Oxidation (M)[11]		Mascot
2350.1318	2349.9802	-0.1516	-65	496	514	QPPSYFICPISQEVMREPR			Carbamidomethyl (C)[8], Oxidation (M)[15]		Mascot
3078.3979	3078.0012	-0.3967	-129	133	158	AKYVYQHAPSSCEVMFMCDGHLIYTK			Carbamidomethyl (C)[12]		Mascot
3094.3928	3094.011	-0.3818	-123	133	158	AKYVYQHAPSSCEVMFMCDGHLIYTK			Carbamidomethyl (C)[12], Oxidation (M)[15]		Mascot
3337.6494	3337.3152	-0.3342	-100	529	557	EWLDNGHETSPMTNLKLAHNNLVPNHALR			Oxidation (M)[12]		Mascot

Rank	Protein Name	Accession No	Protein MW	Protein PI	Pep. Count	Protein Score	Protein Score C. I. %	Total Ion Score	Total Ion C. I. %
8	Defensin-like protein 1 OS=Triticum aestivum PE SV=1	DEF1 WHEA	5235.4	9.49	3	27	0		

Peptide Information

Calc. Mass	Obsrv. Mass	± da	± ppm	Start Seq.	End Seq.	Sequence	Ion Score	C. I. %	Modification	Rank	Result Type
1283.587	1283.5266	-0.0604	-47	7	18	SAGFKGPCMSNK			Carbamidomethyl (C)[8]		Mascot
2381.9919	2381.9629	-0.029	-12	19	40	NCAQVCQQEGWGGGNCDGPFRR					Mascot
3000.2063	3000.0984	-0.1079	-36	12	39	GPCMSNKNCAQVCQQEGWGGGNCDGPFR			Carbamidomethyl (C)[3]		Mascot

Rank	Protein Name	Accession No	Protein MW	Protein PI	Pep. Count	Protein Score	Protein Score C. I. %	Total Ion Score	Total Ion C. I. %
9	E3 ubiquitin-protein ligase SINA-like 1 OS=Arabic thaliana GN=At1g66610 PE=2 SV=1	SINL1 ARATI	40761.6	5.3	8	27	0		

Peptide Information

Calc. Mass	Obsrv. Mass	± da	± ppm	Start Seq.	End Seq.	Sequence	Ion Score	C. I. %	Modification	Rank	Result Type
882.4501	882.4513	0.0012	1	346	352	GTYICIR			Carbamidomethyl (C)[5]		Mascot
1318.6174	1318.4863	-0.1311	-99	222	232	DLYSHYAANHK					Mascot
1318.6174	1318.4863	-0.1311	-99	222	232	DLYSHYAANHK					Mascot
1523.6399	1523.5199	-0.12	-79	73	87	GHIACSSCCTNVSNK					Mascot
1659.7729	1659.6737	-0.0992	-60	88	101	CPYCSLAIGNYRSR			Carbamidomethyl (C)[1]		Mascot
2225.1155	2224.8379	-0.2776	-125	24	44	QRVPSIVEEEGENGGGDVVVR					Mascot
2921.262	2920.9465	-0.3155	-108	73	99	GHIACSSCCTNVSNKCPYCSLAIGNYR			Carbamidomethyl (C)[5]		Mascot
3000.4531	3000.0984	-0.3547	-118	329	352	DFMFIPEYILCPMGLYKGTYICIR			Carbamidomethyl (C)[11,22]		Mascot
3028.4543	3028.1152	-0.3391	-112	321	345	VSFQTPEKDFMFIPEYILCPMGLYK			Oxidation (M)[11,21]		Mascot

Rank	Protein Name	Accession No	Protein MW	Protein PI	Pep. Count	Protein Score	Protein Score C. I. %	Total Ion Score	Total Ion C. I. %
10	50S ribosomal protein L14, chloroplastic OS=Atro belladonna GN=rpl14 PE=3 SV=1	RK14_ATRBE	13583.3	8.71	1	26	0	22	58.504

Peptide Information

Calc. Mass	Obsrv. Mass	± da	± ppm	Start Seq.	End Seq.	Sequence	Ion Score	C. I. %	Modification	Rank	Result Type
1155.5826	1155.4542	-0.1284	-111	45	54	EAVPNMPLER	22	58.504			Mascot
1155.5826	1155.4542	-0.1284	-111	45	54	EAVPNMPLER					Mascot

Gel Idx/Pos	106/E5	Instr./Gel Origin	BA2060/131017A	Process State	Analysis Succeeded
Plate [#] Name	[1] 31496	Instrument Sample Name		Spectra	8

Rank	Protein Name	Accession No	Protein MW	Protein PI	Pep. Count	Protein Score	Protein Score C. I. %	Total Ion Score	Total Ion C. I. %
1	Ribulose bisphosphate carboxylase large chain (Fragment) OS=Garrya elliptica GN=rbcL PE=3 SV=1	RBL GAREL	50884.7	6.34	9	501	100	462	100

Peptide Information

Calc. Mass	Obsrv. Mass	± da	± ppm	Start Seq.	End Seq.	Sequence	Ion Score	C. I. %	Modification	Rank	Result Type
805.4929	805.3777	-0.1152	-143	1	8	VGFKAGVK					Mascot
1021.5312	1021.4037	-0.1275	-125	23	31	DTDILAAFR					Mascot
1021.5312	1021.4037	-0.1275	-125	23	31	DTDILAAFR	71	100			Mascot
1155.5867	1155.45	-0.1367	-118	208	217	FLFCAEAINK					Mascot
1318.642	1318.4843	-0.1577	-120	430	440	EGNEIIREACK			Carbamidomethyl (C)[10]		Mascot
1393.6522	1393.4709	-0.1813	-130	12	22	LTYYTPDYETK					Mascot
1393.6522	1393.4709	-0.1813	-130	12	22	LTYYTPDYETK	48	99.809			Mascot
1445.8297	1445.6433	-0.1864	-129	155	167	YGRPLLGCTIKPK					Mascot
1465.7216	1465.5674	-0.1542	-105	310	324	MSGGDHIHAGTVVGK					Mascot
1465.7546	1465.5674	-0.1872	-128	137	149	TFQGPPHGIQVER	108	100			Mascot
1502.8512	1502.6565	-0.1947	-130	155	167	YGRPLLGCTIKPK			Carbamidomethyl (C)[8]		Mascot
1502.8512	1502.6565	-0.1947	-130	155	167	YGRPLLGCTIKPK	40	98.894	Carbamidomethyl (C)[8]		Mascot
3854.8718	3854.332	-0.5398	-140	32	69	VTPQPGVPPEEAGAAVAAESSTGTWTTVWTDGLTSLDR					Mascot
3854.8718	3854.332	-0.5398	-140	32	69	VTPQPGVPPEEAGAAVAAESSTGTWTTVWTDGLTSLDR	196	100			Mascot

Rank	Protein Name	Accession No	Protein MW	Protein PI	Pep. Count	Protein Score	Protein Score C. I. %	Total Ion Score	Total Ion C. I. %
2	Ribulose bisphosphate carboxylase large chain (Fragment) OS=Actinidia chinensis GN=rbcL PE=3 SV=1	RBL_ACTCH	51553	6.23	9	501	100	462	100

Peptide Information

Calc. Mass	Obsrv. Mass	± da	± ppm	Start Seq.	End Seq.	Sequence	Ion Score	C. I. %	Modification	Rank	Result Type
805.4929	805.3777	-0.1152	-143	1	8	VGFKAGVK					Mascot
1021.5312	1021.4037	-0.1275	-125	23	31	DTDILAAFR					Mascot
1021.5312	1021.4037	-0.1275	-125	23	31	DTDILAAFR	71	100			Mascot
1393.6522	1393.4709	-0.1813	-130	12	22	LTYYTPDYETK					Mascot
1393.6522	1393.4709	-0.1813	-130	12	22	LTYYTPDYETK	48	99.809			Mascot
1445.8297	1445.6433	-0.1864	-129	155	167	YGRPLLGCTIKPK					Mascot
1465.7216	1465.5674	-0.1542	-105	310	324	MSGGDHIHAGTVVGK					Mascot
1465.7546	1465.5674	-0.1872	-128	137	149	TFQGPPHGIQVER	108	100			Mascot
1487.7086	1487.545	-0.1636	-110	454	465	EIKFEFQAMDTL			Oxidation (M)[9]		Mascot
1502.8512	1502.6565	-0.1947	-130	155	167	YGRPLLGCTIKPK			Carbamidomethyl (C)[8]		Mascot
1502.8512	1502.6565	-0.1947	-130	155	167	YGRPLLGCTIKPK	40	98.894	Carbamidomethyl (C)[8]		Mascot
1516.7616	1516.6597	-0.1019	-67	206	217	DRFLFCAEAIFK			Carbamidomethyl (C)[6]		Mascot
3854.8718	3854.332	-0.5398	-140	32	69	VTPQPGVPPEEAGAAVAAESSTGTWTTVWTDGLTSLDR					Mascot
3854.8718	3854.332	-0.5398	-140	32	69	VTPQPGVPPEEAGAAVAAESSTGTWTTVWTDGLTSLDR	196	100			Mascot

Rank	Protein Name	Accession No	Protein MW	Protein PI	Pep. Count	Protein Score	Protein Score C. I. %	Total Ion Score	Total Ion C. I. %
3	Ribulose bisphosphate carboxylase large chain OS=Sesbania sesban GN=rbcL PE=3 SV=1	RBL_SESSE	52358.4	6.09	8	496	100	464	100

Peptide Information

Calc. Mass	Obsrv. Mass	± da	± ppm	Start Seq.	End Seq.	Sequence	Ion Score	C. I. %	Modification	Rank	Result Type
856.3546	856.4178	0.0632	74	467	473	FEFPAMD					Mascot
1021.5312	1021.4037	-0.1275	-125	33	41	DTDILAAFR					Mascot
1021.5312	1021.4037	-0.1275	-125	33	41	DTDILAAFR	71	100			Mascot
1393.6522	1393.4709	-0.1813	-130	22	32	LTYYTPDYETK					Mascot
1393.6522	1393.4709	-0.1813	-130	22	32	LTYYTPDYETK	48	99.809			Mascot
1445.8297	1445.6433	-0.1864	-129	165	177	YGRPLLGCTIKPK					Mascot
1447.7651	1447.5873	-0.1778	-123	320	334	LSGGDHIHAGTVVGK					Mascot
1465.7546	1465.5674	-0.1872	-128	147	159	TFQGPPHGIQVER	108	100			Mascot
1465.7546	1465.5674	-0.1872	-128	147	159	TFQGPPHGIQVER					Mascot
1502.8512	1502.6565	-0.1947	-130	165	177	YGRPLLGCTIKPK			Carbamidomethyl (C)[8]		Mascot
1502.8512	1502.6565	-0.1947	-130	165	177	YGRPLLGCTIKPK	40	98.894	Carbamidomethyl (C)[8]		Mascot
1516.7616	1516.6597	-0.1019	-67	216	227	DRFLFCAEAIFK			Carbamidomethyl (C)[6]		Mascot
3854.8718	3854.332	-0.5398	-140	42	79	VTPQPGVPPEEAGAAVAAESSTGTWTTVWTDGLTSLDR					Mascot
3854.8718	3854.332	-0.5398	-140	42	79	VTPQPGVPPEEAGAAVAAESSTGTWTTVWTDGLTSLDR	196	100			Mascot

Rank	Protein Name	Accession No	Protein MW	Protein PI	Pep. Count	Protein Score	Protein Score C. I. %	Total Ion Score	Total Ion C. I. %
4	Ribulose bisphosphate carboxylase large chain (Fragment) OS=Nyssa ogeche GN=rbcL PE=3 SV=2	RBL_NYSOG	51012.7	6.13	8	494	100	462	100

Peptide Information

Calc. Mass	Obsrv. Mass	± da	± ppm	Start Seq.	End Seq.	Sequence	Ion Score	C. I. %	Modification	Rank	Result Type
805.4929	805.3777	-0.1152	-143	1	8	VGFKAGVK					Mascot
1021.5312	1021.4037	-0.1275	-125	23	31	DTDILAAFR					Mascot
1021.5312	1021.4037	-0.1275	-125	23	31	DTDILAAFR	71	100			Mascot
1393.6522	1393.4709	-0.1813	-130	12	22	LTYYTPDYETK					Mascot
1393.6522	1393.4709	-0.1813	-130	12	22	LTYYTPDYETK	48	99.809			Mascot
1445.8297	1445.6433	-0.1864	-129	155	167	YGRPLLGCTIKPK					Mascot
1465.7216	1465.5674	-0.1542	-105	310	324	MSGGDHIHAGTVVGK					Mascot
1465.7546	1465.5674	-0.1872	-128	137	149	TFQGPPHGIQVER	108	100			Mascot
1502.8512	1502.6565	-0.1947	-130	155	167	YGRPLLGCTIKPK			Carbamidomethyl (C)[8]		Mascot
1502.8512	1502.6565	-0.1947	-130	155	167	YGRPLLGCTIKPK	40	98.894	Carbamidomethyl (C)[8]		Mascot
1814.7506	1814.6594	-0.0912	-50	227	242	GHYLNATAGTCEDMMK			Carbamidomethyl (C)[11], Oxidation (M)[14]		Mascot
3854.8718	3854.332	-0.5398	-140	32	69	VTPQPGVPPEEAGAAVAAESSTGTWTTVWTDGLTSLDR					Mascot
3854.8718	3854.332	-0.5398	-140	32	69	VTPQPGVPPEEAGAAVAAESSTGTWTTVWTDGLTSLDR	196	100			Mascot

Rank	Protein Name	Accession No	Protein MW	Protein PI	Pep. Count	Protein Score	Protein Score C. I. %	Total Ion Score	Total Ion C. I. %
5	Ribulose bisphosphate carboxylase large chain (Fragment) OS=Geum quellyon GN=rbcL PE=3 SV=1	RBL_GEUQU	50910.8	6.23	8	494	100	462	100

Peptide Information

Calc. Mass	Obsrv. Mass	± da	± ppm	Start Seq.	End Seq.	Sequence	Ion Score	C. I. %	Modification	Rank	Result Type
805.4929	805.3777	-0.1152	-143	1	8	VGFKAGVK					Mascot
1021.5312	1021.4037	-0.1275	-125	23	31	DTDILAAFR					Mascot
1021.5312	1021.4037	-0.1275	-125	23	31	DTDILAAFR	71	100			Mascot
1393.6522	1393.4709	-0.1813	-130	12	22	LTYYTPDYETK					Mascot
1393.6522	1393.4709	-0.1813	-130	12	22	LTYYTPDYETK	48	99.809			Mascot
1445.8297	1445.6433	-0.1864	-129	155	167	YGRPLLGCTIKPK					Mascot
1465.7216	1465.5674	-0.1542	-105	310	324	MSGGDHIHAGTVVGK					Mascot
1465.7546	1465.5674	-0.1872	-128	137	149	TFQGPPHGIQVER	108	100			Mascot
1502.8512	1502.6565	-0.1947	-130	155	167	YGRPLLGCTIKPK			Carbamidomethyl (C)[8]		Mascot
1502.8512	1502.6565	-0.1947	-130	155	167	YGRPLLGCTIKPK	40	98.894	Carbamidomethyl (C)[8]		Mascot
1516.7616	1516.6597	-0.1019	-67	206	217	DRFLFCAEALFK			Carbamidomethyl (C)[6]		Mascot
3854.8718	3854.332	-0.5398	-140	32	69	VTPQPGVPPEEAGAAVAAESSTGTWTTVWTDGLTSLDR					Mascot
3854.8718	3854.332	-0.5398	-140	32	69	VTPQPGVPPEEAGAAVAAESSTGTWTTVWTDGLTSLDR	196	100			Mascot

Rank	Protein Name	Accession No	Protein MW	Protein PI	Pep. Count	Protein Score	Protein Score C. I. %	Total Ion Score	Total Ion C. I. %
6	Ribulose bisphosphate carboxylase large chain (Fragment) OS=Cassia fistula GN=rbcL PE=3 SV=1	RBL_CASFS	51606.1	6.14	8	494	100	462	100

Peptide Information

Calc. Mass	Obsrv. Mass	± da	± ppm	Start Seq.	End Seq.	Sequence	Ion Score	C. I. %	Modification	Rank	Result Type
805.4929	805.3777	-0.1152	-143	1	8	VGFKAGVK					Mascot
1021.5312	1021.4037	-0.1275	-125	23	31	DTDILAAFR					Mascot
1021.5312	1021.4037	-0.1275	-125	23	31	DTDILAAFR	71	100			Mascot
1393.6522	1393.4709	-0.1813	-130	12	22	LTYYTPDYETK					Mascot
1393.6522	1393.4709	-0.1813	-130	12	22	LTYYTPDYETK	48	99.809			Mascot
1445.8297	1445.6433	-0.1864	-129	155	167	YGRPLLGCTIKPK					Mascot
1447.7651	1447.5873	-0.1778	-123	310	324	LSGGDHIHAGTVVGK					Mascot
1465.6603	1465.5674	-0.0929	-63	206	217	DRFCFCAEALYK					Mascot
1465.7546	1465.5674	-0.1872	-128	137	149	TFQGPPHGIQVER	108	100			Mascot
1502.8512	1502.6565	-0.1947	-130	155	167	YGRPLLGCTIKPK			Carbamidomethyl (C)[8]		Mascot
1502.8512	1502.6565	-0.1947	-130	155	167	YGRPLLGCTIKPK	40	98.894	Carbamidomethyl (C)[8]		Mascot
3854.8718	3854.332	-0.5398	-140	32	69	VTPQPGVPPEEAGAAVAAESSTGTWTTVWTDGLTSLDR					Mascot
3854.8718	3854.332	-0.5398	-140	32	69	VTPQPGVPPEEAGAAVAAESSTGTWTTVWTDGLTSLDR	196	100			Mascot

Rank	Protein Name	Accession No	Protein MW	Protein PI	Pep. Count	Protein Score	Protein Score C. I. %	Total Ion Score	Total Ion C. I. %
7	Ribulose bisphosphate carboxylase large chain (Fragment) OS=Drimys winteri GN=rbcL PE=3 SV=2	RBL_DRIWI	51726.1	6.23	8	494	100	462	100

Peptide Information

Calc. Mass	Obsrv. Mass	± da	± ppm	Start Seq.	End Seq.	Sequence	Ion Score	C. I. %	Modification	Rank	Result Type
805.4929	805.3777	-0.1152	-143	1	8	VGFKAGVK					Mascot
1021.5312	1021.4037	-0.1275	-125	23	31	DTDILAAFR					Mascot
1021.5312	1021.4037	-0.1275	-125	23	31	DTDILAAFR	71	100			Mascot
1318.642	1318.4843	-0.1577	-120	430	440	EGNEIIREACK			Carbamidomethyl (C)[10]		Mascot
1393.6522	1393.4709	-0.1813	-130	12	22	LTYYTPDYETK					Mascot
1393.6522	1393.4709	-0.1813	-130	12	22	LTYYTPDYETK	48	99.809			Mascot
1445.8297	1445.6433	-0.1864	-129	155	167	YGRPLLGCTIKPK					Mascot
1465.7216	1465.5674	-0.1542	-105	310	324	MSGGDHIHAGTVVGK					Mascot
1465.7546	1465.5674	-0.1872	-128	137	149	TFQGPPHGIQVER	108	100			Mascot
1502.8512	1502.6565	-0.1947	-130	155	167	YGRPLLGCTIKPK			Carbamidomethyl (C)[8]		Mascot
1502.8512	1502.6565	-0.1947	-130	155	167	YGRPLLGCTIKPK	40	98.894	Carbamidomethyl (C)[8]		Mascot
3854.8718	3854.332	-0.5398	-140	32	69	VTPQPGVPPEEAGAAVAAESSTGTWTTVWTDGLTSLDR					Mascot
3854.8718	3854.332	-0.5398	-140	32	69	VTPQPGVPPEEAGAAVAAESSTGTWTTVWTDGLTSLDR	196	100			Mascot

Rank	Protein Name	Accession No	Protein MW	Protein PI	Pep. Count	Protein Score	Protein Score C. I. %	Total Ion Score	Total Ion C. I. %
8	Ribulose bisphosphate carboxylase large chain OS=Lotus japonicus GN=rbcL PE=3 SV=1	RBL_LOTJA	52739.5	6.04	8	492	100	462	100

Peptide Information

Calc. Mass	Obsrv. Mass	± da	± ppm	Start Seq.	End Seq.	Sequence	Ion Score	C. I. %	Modification	Rank	Result Type
1021.5312	1021.4037	-0.1275	-125	33	41	DTDILAAFR					Mascot
1021.5312	1021.4037	-0.1275	-125	33	41	DTDILAAFR	71	100			Mascot
1393.6522	1393.4709	-0.1813	-130	22	32	LTYYTPDYETK					Mascot
1393.6522	1393.4709	-0.1813	-130	22	32	LTYYTPDYETK	48	99.809			Mascot
1445.8297	1445.6433	-0.1864	-129	165	177	YGRPLLGCTIKPK					Mascot
1465.7216	1465.5674	-0.1542	-105	320	334	MSGGDHIHAGTVVGK					Mascot
1465.7546	1465.5674	-0.1872	-128	147	159	TFQGPPHGIQVER	108	100			Mascot
1487.7086	1487.545	-0.1636	-110	464	475	EIKFEFQAMDTL			Oxidation (M)[9]		Mascot
1502.8512	1502.6565	-0.1947	-130	165	177	YGRPLLGCTIKPK			Carbamidomethyl (C)[8]		Mascot
1502.8512	1502.6565	-0.1947	-130	165	177	YGRPLLGCTIKPK	40	98.894	Carbamidomethyl (C)[8]		Mascot
1516.7616	1516.6597	-0.1019	-67	216	227	DRFLFCAEALFK			Carbamidomethyl (C)[6]		Mascot
3854.8718	3854.332	-0.5398	-140	42	79	VTPQPGVPPEEAGAAVAAESSTGTWTTVWTDGLTSLDR					Mascot
3854.8718	3854.332	-0.5398	-140	42	79	VTPQPGVPPEEAGAAVAAESSTGTWTTVWTDGLTSLDR	196	100			Mascot

Rank	Protein Name	Accession No	Protein MW	Protein PI	Pep. Count	Protein Score	Protein Score C. I. %	Total Ion Score	Total Ion C. I. %
9	Ribulose bisphosphate carboxylase large chain (Fragment) OS=Sedum rubrotinctum GN=rbcL PE=3 SV=1	RBL_SEDRU	49773.1	6.23	7	491	100	464	100

Peptide Information

Calc. Mass	Obsrv. Mass	± da	± ppm	Start Seq.	End Seq.	Sequence	Ion Score	C. I. %	Modification	Rank	Result Type
805.4929	805.3777	-0.1152	-143	1	8	VGFKAGVK					Mascot
1021.5312	1021.4037	-0.1275	-125	23	31	DTDILAAFR					Mascot
1021.5312	1021.4037	-0.1275	-125	23	31	DTDILAAFR	71	100			Mascot
1318.642	1318.4843	-0.1577	-120	430	440	EGNEIIREACK			Carbamidomethyl (C)[10]		Mascot
1393.6522	1393.4709	-0.1813	-130	12	22	LTYYTPDYETK					Mascot
1393.6522	1393.4709	-0.1813	-130	12	22	LTYYTPDYETK	48	99.809			Mascot
1445.8297	1445.6433	-0.1864	-129	155	167	YGRPLLGCTIKPK					Mascot
1465.7546	1465.5674	-0.1872	-128	137	149	TFQGPPHGIQVER	108	100			Mascot
1465.7546	1465.5674	-0.1872	-128	137	149	TFQGPPHGIQVER					Mascot
1502.8512	1502.6565	-0.1947	-130	155	167	YGRPLLGCTIKPK			Carbamidomethyl (C)[8]		Mascot
1502.8512	1502.6565	-0.1947	-130	155	167	YGRPLLGCTIKPK	40	98.894	Carbamidomethyl (C)[8]		Mascot
3854.8718	3854.332	-0.5398	-140	32	69	VTPQPGVPPEEAGAAVAAESSTGTWTTVWTDGLTSLDR					Mascot
3854.8718	3854.332	-0.5398	-140	32	69	VTPQPGVPPEEAGAAVAAESSTGTWTTVWTDGLTSLDR	196	100			Mascot

Rank	Protein Name	Accession No	Protein MW	Protein PI	Pep. Count	Protein Score	Protein Score C. I. %	Total Ion Score	Total Ion C. I. %
10	Ribulose bisphosphate carboxylase large chain (Fragment) OS=Senna didymobotrya GN=rbcL PE=3 SV=1	RBL_SENDI	50390.4	6.22	7	490	100	464	100

Peptide Information

Calc. Mass	Obsrv. Mass	± da	± ppm	Start Seq.	End Seq.	Sequence	Ion Score	C. I. %	Modification	Rank	Result Type
1021.5312	1021.4037	-0.1275	-125	24	32	DTDILAAFR					Mascot
1021.5312	1021.4037	-0.1275	-125	24	32	DTDILAAFR	71	100			Mascot
1393.6522	1393.4709	-0.1813	-130	13	23	LTYYTPDYETK					Mascot
1393.6522	1393.4709	-0.1813	-130	13	23	LTYYTPDYETK	48	99.809			Mascot
1445.8297	1445.6433	-0.1864	-129	156	168	YGRPLLGCTIKPK					Mascot
1447.7651	1447.5873	-0.1778	-123	311	325	LSGGDHIHAGTVVGK					Mascot
1465.7546	1465.5674	-0.1872	-128	138	150	TFQGPPHGIQVER	108	100			Mascot
1465.7546	1465.5674	-0.1872	-128	138	150	TFQGPPHGIQVER					Mascot
1502.8512	1502.6565	-0.1947	-130	156	168	YGRPLLGCTIKPK			Carbamidomethyl (C)[8]		Mascot
1502.8512	1502.6565	-0.1947	-130	156	168	YGRPLLGCTIKPK	40	98.894	Carbamidomethyl (C)[8]		Mascot
1516.7616	1516.6597	-0.1019	-67	207	218	DRFLFCAEAIFK			Carbamidomethyl (C)[6]		Mascot
3854.8718	3854.332	-0.5398	-140	33	70	VTPQPGVPPEEAGAAVAAESSTGTWTTVWTDGLTSLDR					Mascot
3854.8718	3854.332	-0.5398	-140	33	70	VTPQPGVPPEEAGAAVAAESSTGTWTTVWTDGLTSLDR	196	100			Mascot

Gel Idx/Pos	107/E6	Instr./Gel Origin	BA2060/131017A	Process Sta	Analysis Succeeded
Plate [#] Name	[1] 31496	Instrument Sample Name		Spectra	8

Rank	Protein Name	Accession No	Protein MW	Protein PI	Pep Count	Protein Score	Protein Score C. I. %	Total Ion Score	Total Ion C. I. %
1	Chlorophyll a-b binding protein 6, chloroplastic OS=Arabidopsis thaliana GN=LHCA1 PE=1 SV=1	CAB6_ARAT	25979.3	6.21	4	152	100	135	100

Peptide Information

Calc. Mass	Obsrv. Mass	± da	± ppm	Start Seq.	End Seq.	Sequence	Ion Score	C. I. %	Modification	Rank	Result Type
986.4724	986.3559	-0.1165	-118	85	92	ESELIHCR					Mascot
1043.4939	1043.3655	-0.1284	-123	85	92	ESELIHCR			Carbamidomethyl (C)[7]		Mascot
1043.4939	1043.3655	-0.1284	-123	85	92	ESELIHCR	47	99.839	Carbamidomethyl (C)[7]		Mascot
1334.6521	1334.5078	-0.1443	-108	83	92	YKESELIHCR			Carbamidomethyl (C)[9]		Mascot
1371.658	1371.4852	-0.1728	-126	167	179	YPGGAFDPLGYSK	88	100			Mascot
1371.658	1371.4852	-0.1728	-126	167	179	YPGGAFDPLGYSK					Mascot
1499.7528	1499.5592	-0.1936	-129	166	179	KYPGGAFDPLGYSK					Mascot

Rank	Protein Name	Accession No	Protein MW	Protein PI	Pep Count	Protein Score	Protein Score C. I. %	Total Ion Score	Total Ion C. I. %
2	Chlorophyll a-b binding protein 6A, chloroplastic OS=Solanum lycopersicum GN=CAB6A PE=2 SV=1	CB11_SOLLC	26557.4	5.82	4	151	100	135	100

Peptide Information

Calc. Mass	Obsrv. Mass	± da	± ppm	Start Seq.	End Seq.	Sequence	Ion Score	C. I. %	Modification	Rank	Result Type
986.4724	986.3559	-0.1165	-118	86	93	ESELIHCR					Mascot
1043.4939	1043.3655	-0.1284	-123	86	93	ESELIHCR			Carbamidomethyl (C)[7]		Mascot
1043.4939	1043.3655	-0.1284	-123	86	93	ESELIHCR	47	99.839	Carbamidomethyl (C)[7]		Mascot
1334.6521	1334.5078	-0.1443	-108	84	93	YKESELIHCR			Carbamidomethyl (C)[9]		Mascot
1371.658	1371.4852	-0.1728	-126	168	180	YPGGAFDPLGYSK	88	100			Mascot
1371.658	1371.4852	-0.1728	-126	168	180	YPGGAFDPLGYSK					Mascot
1499.7528	1499.5592	-0.1936	-129	167	180	KYPGGAFDPLGYSK					Mascot

Rank	Protein Name	Accession No	Protein MW	Protein PI	Pep Count	Protein Score	Protein Score C. I. %	Total Ion Score	Total Ion C. I. %
3	Putative cytochrome c oxidase subunit II PS17 (Fragments) OS=Pinus strobus PE=1 SV=1	PS17_PINST	1707	9.63	2	61	97.07	38	98.828

Peptide Information

Calc. Mass	Obsrv. Mass	± da	± ppm	Start Seq.	End Seq.	Sequence	Ion Score	C. I. %	Modification	Rank	Result Type
Calc. Mass	Obsrv. Mass	± da	± ppm	Start Seq.	End Seq.	Sequence	Ion Score	C. I. %	Modification	Rank	Result Type
856.525	856.4191	-0.1059	-124	1	8	SPTVIALR	11	0			Mascot
856.525	856.4191	-0.1059	-124	1	8	SPTVIALR					Mascot
870.5043	870.4335	-0.0708	-81	9	16	VVEALSPR	27	83.79			Mascot
870.5043	870.4335	-0.0708	-81	9	16	VVEALSPR					Mascot

Rank	Protein Name	Accession No	Protein MW	Protein PI	Pep Count	Protein Score	Protein Score C. I. %	Total Ion Score	Total Ion C. I. %
4	Putative LRR disease resistance protein/transmembrane receptor kinase PS19 (Fragment) OS=Pinus stro	PS19_PINST	869.5	9.63	1	49	59.552	44	99.656

Peptide Information

Calc. Mass	Obsrv. Mass	± da	± ppm	Start Seq.	End Seq.	Sequence	Ion Score	C. I. %	Modification	Rank	Result Type
870.5406	870.4335	-0.1071	-123	1	8	VVSLSIPR	44	99.656			Mascot
870.5406	870.4335	-0.1071	-123	1	8	VVSLSIPR					Mascot

Rank	Protein Name	Accession No	Protein MW	Protein PI	Pep Count	Protein Score	Protein Score C. I. %	Total Ion Score	Total Ion C. I. %
5	Serine/threonine protein phosphatase 2A 57 kDa regulatory subunit B' iota isoform OS=Arabidopsis th	2A5I_ARATH	56385	8.9	4	44	0	35	97.419

Peptide Information

Calc. Mass	Obsrv. Mass	± da	± ppm	Start Seq.	End Seq.	Sequence	Ion Score	C. I. %	Modification	Rank	Result Type
856.525	856.4191	-0.1059	-124	487	494	TAVLVTPR	35	97.419			Mascot
856.525	856.4191	-0.1059	-124	487	494	TAVLVTPR					Mascot
1010.491	1010.4702	-0.0208	-21	140	147	MCAVNLFR			Carbamidomethyl (C)[2]		Mascot
1026.486	1026.3729	-0.1131	-110	140	147	MCAVNLFR			Carbamidomethyl (C)[2], Oxidation (M)[1]		Mascot
1179.5601	1179.4536	-0.1065	-90	13	23	SSKSDSGELNR					Mascot
2210.8796	2210.8115	-0.0681	-31	436	452	KMFCEMDEALFMSCHAR			Carbamidomethyl (C)[4,14], Oxidation (M)[2,6,12]		Mascot

Rank	Protein Name	Accession No	Protein MW	Protein PI	Pep Count	Protein Score	Protein Score C. I. %	Total Ion Score	Total Ion C. I. %
6	Ribulose bisphosphate carboxylase large chain (Fragment) OS=Isophysis tasmanica GN=rbcL PE=3 SV=1	RBL_ISOTA	51734.8	5.87	10	42	0		

Peptide Information

Calc. Mass	Obsrv. Mass	± da	± ppm	Start Seq.	End Seq.	Sequence	Ion Score	C. I. %	Modification	Rank	Result Type
864.4937	864.3859	-0.1078	-125	1	9	SAGFKAGVK					Mascot
914.4049	914.2896	-0.1153	-126	297	303	NHGMHFR			Oxidation (M)[4]		Mascot
1010.4828	1010.4702	-0.0126	-12	458	465	FEFEPVDK					Mascot
1021.5312	1021.4025	-0.1287	-126	24	32	DTDILAAFR					Mascot
1111.5306	1111.4203	-0.1103	-99	458	466	FEFEPVDKT					Mascot
1116.583	1116.4412	-0.1418	-127	413	422	VALEACVQAR			Carbamidomethyl (C)[6]		Mascot
1393.6522	1393.4832	-0.169	-121	13	23	LTYYTPDYETK					Mascot
1465.7546	1465.5701	-0.1845	-126	138	150	TFQGPPHGIQVER					Mascot
1475.7351	1475.5643	-0.1708	-116	207	218	DRFLFCAEAIYK					Mascot
1794.8149	1794.5649	-0.25	-139	228	243	GHYLNATAGTCEEMIK			Carbamidomethyl (C)[11]		Mascot

Rank	Protein Name	Accession No	Protein MW	Protein PI	Pep Count	Protein Score	Protein Score C. I. %	Total Ion Score	Total Ion C. I. %
7	Ribulose bisphosphate carboxylase large chain (Fragment) OS=Streptopus lanceolatus GN=rbcL PE=3 SV=	RBL_STRLC	50649.4	6.18	9	36	0		

Peptide Information

Calc. Mass	Obsrv. Mass	± da	± ppm	Start Seq.	End Seq.	Sequence	Ion Score	C. I. %	Modification	Rank	Result Type
914.4049	914.2896	-0.1153	-126	306	312	NHGMHFR			Oxidation (M)[4]		Mascot
1021.5312	1021.4025	-0.1287	-126	33	41	DTDILAAFR					Mascot
1116.583	1116.4412	-0.1418	-127	422	431	VALEACVQAR			Carbamidomethyl (C)[6]		Mascot
1318.642	1318.4927	-0.1493	-113	440	450	EGNEIIREACK			Carbamidomethyl (C)[10]		Mascot
1318.642	1318.4927	-0.1493	-113	440	450	EGNEIIREACK			Carbamidomethyl (C)[10]		Mascot
1351.6852	1351.5255	-0.1597	-118	2	14	SPQTETKASAGFK					Mascot
1393.6522	1393.4832	-0.169	-121	22	32	LTYYTPDYETK					Mascot
1465.7216	1465.5701	-0.1515	-103	320	334	MSGGDHIHAGTVVGK					Mascot
1475.7351	1475.5643	-0.1708	-116	216	227	DRFLFCAEAIYK					Mascot
1794.8149	1794.5649	-0.25	-139	237	252	GHYLNATAGTCEEMIK			Carbamidomethyl (C)[11]		Mascot

Rank	Protein Name	Accession No	Protein MW	Protein PI	Pep Count	Protein Score	Protein Score C. I. %	Total Ion Score	Total Ion C. I. %
8	50S ribosomal protein L14, chloroplastic OS=Calycanthus floridus var. glaucus GN=rpl14 PE=3 SV=1	RK14_CALFG	13568.3	9.39	2	34	0	24	67.88

Peptide Information

Calc. Mass	Obsrv. Mass	± da	± ppm	Start Seq.	End Seq.	Sequence	Ion Score	C. I. %	Modification	Rank	Result Type
1155.5826	1155.4575	-0.1251	-108	45	54	EAVPNMPLER					Mascot
1155.5826	1155.4575	-0.1251	-108	45	54	EAVPNMPLER	24	67.88			Mascot
1475.7821	1475.5643	-0.2178	-148	18	30	ELMCIRIIGASNR					Mascot

Rank	Protein Name	Accession No	Protein MW	Protein PI	Pep Count	Protein Score	Protein Score C. I. %	Total Ion Score	Total Ion C. I. %
9	50S ribosomal protein L14, chloroplastic OS=Pip cenocladum GN=rpl14 PE=3 SV=1	RK14_PIPCE	13589.3	9.09	2	34	0	24	67.88

Peptide Information

Calc. Mass	Obsrv. Mass	± da	± ppm	Start Seq.	End Seq.	Sequence	Ion Score	C. I. %	Modification	Rank	Result Type
1155.5826	1155.4575	-0.1251	-108	45	54	EAVPNMPLER					Mascot
1155.5826	1155.4575	-0.1251	-108	45	54	EAVPNMPLER	24	67.88			Mascot
1475.7821	1475.5643	-0.2178	-148	18	30	ELMCIRILGASNR					Mascot

Rank	Protein Name	Accession No	Protein MW	Protein PI	Pep Count	Protein Score	Protein Score C. I. %	Total Ion Score	Total Ion C. I. %
10	Ribulose bisphosphate carboxylase large chain (Fragment) OS=Amorphophallus titanum GN=rbcL PE=3 SV=	RBL_AMOTI	52548.4	6.32	9	32	0		

Peptide Information

Calc. Mass	Obsrv. Mass	± da	± ppm	Start Seq.	End Seq.	Sequence	Ion Score	C. I. %	Modification	Rank	Result Type
914.3858	914.2896	-0.0962	-105	182	188	ACYECLR			Carbamidomethyl (C)[2]		Mascot
1010.4828	1010.4702	-0.0126	-12	461	468	FEFEPVDK					Mascot
1021.5312	1021.4025	-0.1287	-126	27	35	DTDILAAFR					Mascot
1116.583	1116.4412	-0.1418	-127	416	425	VALEACVQAR			Carbamidomethyl (C)[6]		Mascot
1393.6522	1393.4832	-0.169	-121	16	26	LTYYTPDYETK					Mascot
1465.7216	1465.5701	-0.1515	-103	314	328	MSGGDHIHAGTVVGK					Mascot
1475.7351	1475.5643	-0.1708	-116	210	221	DRFLFCAEALYK					Mascot
1794.8149	1794.5649	-0.25	-139	231	246	GHYLNATAGTCEEMIK			Carbamidomethyl (C)[11]		Mascot
2211.1191	2210.8115	-0.3076	-139	134	153	IPPAYSKTFEGPPHGIQSER					Mascot

Gel Idx/Pos	108/E7	Instr./Gel Origin	BA2060/131017A	Process Sta	Analysis Succeeded
Plate [#] Name	[1] 31496	Instrument Sample Name		Spectra	8

Rank	Protein Name	Accession No	Protein MW	Protein PI	Pep. Count	Protein Score	Protein Score C. I. %	Total Ion Score	Total Ion C. I. %
1	Ribulose bisphosphate carboxylase large chain OS=Glycine max GN=rbcL PE=1 SV=3	RBL_SOYBN	52576.4	6	6	531	100	511	100

Peptide Information

Calc. Mass	Obsrv. Mass	± da	± ppm	Start Seq.	End Seq.	Sequence	Ion Score	C. I. %	Modification	Rank	Result Type
805.4818	805.3775	-0.1043	-129	140	146	IPTAYIK	37	97.68			Mascot
805.4818	805.3775	-0.1043	-129	140	146	IPTAYIK					Mascot
1021.5312	1021.4022	-0.129	-126	33	41	DTDILAAFR					Mascot
1021.5312	1021.4022	-0.129	-126	33	41	DTDILAAFR	70	99.999			Mascot
1168.6255	1168.4816	-0.1439	-123	296	305	AMHAVIDRQK					Mascot
1393.6522	1393.4695	-0.1827	-131	22	32	LTYYTPDYETK					Mascot
1393.6522	1393.4695	-0.1827	-131	22	32	LTYYTPDYETK	57	99.974			Mascot
1465.7546	1465.5664	-0.1882	-128	147	159	TFQGPPHGIQVER	95	100			Mascot
1465.7546	1465.5664	-0.1882	-128	147	159	TFQGPPHGIQVER					Mascot
3854.8718	3854.3462	-0.5256	-136	42	79	VTPQPGVPPEEAGAAVAESSTGTWTTVWTDGLTSLDR	252	100			Mascot
3854.8718	3854.3462	-0.5256	-136	42	79	VTPQPGVPPEEAGAAVAESSTGTWTTVWTDGLTSLDR					Mascot

Rank	Protein Name	Accession No	Protein MW	Protein PI	Pep. Count	Protein Score	Protein Score C. I. %	Total Ion Score	Total Ion C. I. %
2	Ribulose bisphosphate carboxylase large chain (Fragment) OS=Roridula gorgonias GN=rbcL PE=3 SV=1	RBL_RORGO	50964.7	6.34	8	506	100	474	100

Peptide Information

Calc. Mass	Obsrv. Mass	± da	± ppm	Start Seq.	End Seq.	Sequence	Ion Score	C. I. %	Modification	Rank	Result Type
805.4929	805.3775	-0.1154	-143	1	8	VGFKAGVK					Mascot
805.4929	805.3775	-0.1154	-143	1	8	VGFKAGVK					Mascot
990.4738	990.4683	-0.0055	-6	218	226	AQDETGEIK					Mascot
1021.5312	1021.4022	-0.129	-126	23	31	DTDILAAFR					Mascot
1021.5312	1021.4022	-0.129	-126	23	31	DTDILAAFR	70	99.999			Mascot
1168.6255	1168.4816	-0.1439	-123	286	295	AMHAVIDRQK					Mascot
1393.6522	1393.4695	-0.1827	-131	12	22	LTYYTPDYETK					Mascot
1393.6522	1393.4695	-0.1827	-131	12	22	LTYYTPDYETK	57	99.974			Mascot
1465.7546	1465.5664	-0.1882	-128	137	149	TFQGPPHGIQVER	95	100			Mascot
1465.7546	1465.5664	-0.1882	-128	137	149	TFQGPPHGIQVER					Mascot
1481.7166	1481.5549	-0.1617	-109	310	324	MSGGDHIHSGTVVGK					Mascot
3854.8718	3854.3462	-0.5256	-136	32	69	VTPQPGVPPEEAGAAVAESSTGTWTTVWTDGLTSLDR	252	100			Mascot
3854.8718	3854.3462	-0.5256	-136	32	69	VTPQPGVPPEEAGAAVAESSTGTWTTVWTDGLTSLDR					Mascot

Rank	Protein Name	Accession No	Protein MW	Protein PI	Pep. Count	Protein Score	Protein Score C. I. %	Total Ion Score	Total Ion C. I. %
3	Ribulose bisphosphate carboxylase large chain (Fragment) OS=Idesia polycarpa GN=rbcL PE=3 SV=1	RBL_IDEPO	51555	6	8	506	100	474	100

Peptide Information

Calc. Mass	Obsrv. Mass	± da	± ppm	Start Seq.	End Seq.	Sequence	Ion Score	C. I. %	Modification	Rank	Result Type
805.4929	805.3775	-0.1154	-143	1	8	VGFKAGVK					Mascot
805.4929	805.3775	-0.1154	-143	1	8	VGFKAGVK					Mascot
1021.5312	1021.4022	-0.129	-126	23	31	DTDILAAFR					Mascot
1021.5312	1021.4022	-0.129	-126	23	31	DTDILAAFR	70	99.999			Mascot
1168.6255	1168.4816	-0.1439	-123	286	295	AMHAVIDRQK					Mascot
1393.6522	1393.4695	-0.1827	-131	12	22	LTYYTPDYETK					Mascot
1393.6522	1393.4695	-0.1827	-131	12	22	LTYYTPDYETK	57	99.974			Mascot
1465.7546	1465.5664	-0.1882	-128	137	149	TFQGPPHGIQVER	95	100			Mascot
1465.7546	1465.5664	-0.1882	-128	137	149	TFQGPPHGIQVER					Mascot
1481.7166	1481.5549	-0.1617	-109	310	324	MSGGDHIHSGTVVGK					Mascot
1487.7086	1487.5428	-0.1658	-111	454	465	EIKFEFQAMDTL			Oxidation (M)[9]		Mascot
3854.8718	3854.3462	-0.5256	-136	32	69	VTPQPGVPPEEAGAAVAESSTGTWTTVWTDGLTSLDR	252	100			Mascot
3854.8718	3854.3462	-0.5256	-136	32	69	VTPQPGVPPEEAGAAVAESSTGTWTTVWTDGLTSLDR					Mascot

Rank	Protein Name	Accession No	Protein MW	Protein PI	Pep. Count	Protein Score	Protein Score C. I. %	Total Ion Score	Total Ion C. I. %
4	Ribulose bisphosphate carboxylase large chain (Fragment) OS=Nyssa ogeche GN=rbcL PE=3 SV=2	RBL_NYSOG	51012.7	6.13	8	505	100	472	100

Peptide Information

Calc. Mass	Obsrv. Mass	± da	± ppm	Start Seq.	End Seq.	Sequence	Ion Score	C. I. %	Modification	Rank	Result Type
805.4929	805.3775	-0.1154	-143	1	8	VGFKAGVK					Mascot
805.4929	805.3775	-0.1154	-143	1	8	VGFKAGVK					Mascot
1021.5312	1021.4022	-0.129	-126	23	31	DTDILAAFR					Mascot
1021.5312	1021.4022	-0.129	-126	23	31	DTDILAAFR	70	99.999			Mascot
1168.6255	1168.4816	-0.1439	-123	286	295	AMHAVIDRQK					Mascot
1393.6522	1393.4695	-0.1827	-131	12	22	LTYYTPDYETK					Mascot
1393.6522	1393.4695	-0.1827	-131	12	22	LTYYTPDYETK	57	99.974			Mascot
1465.7216	1465.5664	-0.1552	-106	310	324	MSGGDHIHAGTVVGK					Mascot
1465.7546	1465.5664	-0.1882	-128	137	149	TFQGPPHGIQVER	95	100			Mascot
1481.7166	1481.5549	-0.1617	-109	310	324	MSGGDHIHAGTVVGK			Oxidation (M)[1]		Mascot
1503.7301	1503.5112	-0.2189	-146	441	453	WSPELAAACEIWK					Mascot
3854.8718	3854.3462	-0.5256	-136	32	69	VTPQPGVPPEEAGAAVAESSTGTWTTVWTDGLTSLDR	252	100			Mascot
3854.8718	3854.3462	-0.5256	-136	32	69	VTPQPGVPPEEAGAAVAESSTGTWTTVWTDGLTSLDR					Mascot

Rank	Protein Name	Accession No	Protein MW	Protein PI	Pep. Count	Protein Score	Protein Score C. I. %	Total Ion Score	Total Ion C. I. %
5	Ribulose bisphosphate carboxylase large chain (Fragment) OS=Cassia fistula GN=rbcL PE=3 SV=1	RBL_CASFS	51606.1	6.14	8	504	100	472	100

Peptide Information

Calc. Mass	Obsrv. Mass	± da	± ppm	Start Seq.	End Seq.	Sequence	Ion Score	C. I. %	Modification	Rank	Result Type
805.4929	805.3775	-0.1154	-143	1	8	VGFKAGVK					Mascot
805.4929	805.3775	-0.1154	-143	1	8	VGFKAGVK					Mascot
1021.5312	1021.4022	-0.129	-126	23	31	DTDILAAFR					Mascot
1021.5312	1021.4022	-0.129	-126	23	31	DTDILAAFR	70	99.999			Mascot
1168.6255	1168.4816	-0.1439	-123	286	295	AMHAVIDRQK					Mascot
1393.6522	1393.4695	-0.1827	-131	12	22	LTYYTPDYETK					Mascot
1393.6522	1393.4695	-0.1827	-131	12	22	LTYYTPDYETK	57	99.974			Mascot
1447.7651	1447.5518	-0.2133	-147	310	324	LSGGDHIHAGTVVGK					Mascot
1465.6603	1465.5664	-0.0939	-64	206	217	DRFCFCAEALYK					Mascot
1465.7546	1465.5664	-0.1882	-128	137	149	TFQGPPHGIQVER	95	100			Mascot
1522.6816	1522.5703	-0.1113	-73	206	217	DRFCFCAEALYK			Carbamidomethyl (C)[4]		Mascot
3854.8718	3854.3462	-0.5256	-136	32	69	VTPQPGVPPEEAGAAVAESSTGTWTTVWTDGLTSLDR	252	100			Mascot
3854.8718	3854.3462	-0.5256	-136	32	69	VTPQPGVPPEEAGAAVAESSTGTWTTVWTDGLTSLDR					Mascot

Rank	Protein Name	Accession No	Protein MW	Protein PI	Pep. Count	Protein Score	Protein Score C. I. %	Total Ion Score	Total Ion C. I. %
6	Ribulose bisphosphate carboxylase large chain (Fragment) OS=Actinidia chinensis GN=rbcL PE=3 SV=1	RBL_ACTCH	51553	6.23	8	504	100	472	100

Peptide Information

Calc. Mass	Obsrv. Mass	± da	± ppm	Start Seq.	End Seq.	Sequence	Ion Score	C. I. %	Modification	Rank	Result Type
805.4929	805.3775	-0.1154	-143	1	8	VGFKAGVK					Mascot
805.4929	805.3775	-0.1154	-143	1	8	VGFKAGVK					Mascot
1021.5312	1021.4022	-0.129	-126	23	31	DTDILAAFR					Mascot
1021.5312	1021.4022	-0.129	-126	23	31	DTDILAAFR	70	99.999			Mascot
1168.6255	1168.4816	-0.1439	-123	286	295	AMHAVIDRQK					Mascot
1393.6522	1393.4695	-0.1827	-131	12	22	LTYYTPDYETK					Mascot
1393.6522	1393.4695	-0.1827	-131	12	22	LTYYTPDYETK	57	99.974			Mascot
1465.7216	1465.5664	-0.1552	-106	310	324	MSGGDHIHAGTVVGK					Mascot
1465.7546	1465.5664	-0.1882	-128	137	149	TFQGPPHGIQVER	95	100			Mascot
1481.7166	1481.5549	-0.1617	-109	310	324	MSGGDHIHAGTVVGK			Oxidation (M)[1]		Mascot
1487.7086	1487.5428	-0.1658	-111	454	465	EIKFEFQAMDTL			Oxidation (M)[9]		Mascot
3854.8718	3854.3462	-0.5256	-136	32	69	VTPQPGVPPEEAGAAVAESSTGTWTTVWTDGLTSLDR	252	100			Mascot
3854.8718	3854.3462	-0.5256	-136	32	69	VTPQPGVPPEEAGAAVAESSTGTWTTVWTDGLTSLDR					Mascot

Rank	Protein Name	Accession No	Protein MW	Protein PI	Pep. Count	Protein Score	Protein Score C. I. %	Total Ion Score	Total Ion C. I. %
7	Ribulose bisphosphate carboxylase large chain OS=Lotus japonicus GN=rbcL PE=3 SV=1	RBL_LOTJA	52739.5	6.04	8	503	100	472	100

Peptide Information

Calc. Mass	Obsrv. Mass	± da	± ppm	Start Seq.	End Seq.	Sequence	Ion Score	C. I. %	Modification	Rank	Result Type
1021.5312	1021.4022	-0.129	-126	33	41	DTDILAAFR					Mascot
	1021.4022										Mascot
1021.5312	1021.4022	-0.129	-126	33	41	DTDILAAFR	70	99.999			Mascot
1168.6255	1168.4816	-0.1439	-123	296	305	AMHAVIDRQK					Mascot
1393.6522	1393.4695	-0.1827	-131	22	32	LTYYTPDYETK					Mascot
1393.6522	1393.4695	-0.1827	-131	22	32	LTYYTPDYETK	57	99.974			Mascot
1465.7216	1465.5664	-0.1552	-106	320	334	MSGGDHIHAGTVVGK					Mascot
1465.7546	1465.5664	-0.1882	-128	147	159	TFQGPPHGIQVER	95	100			Mascot
1481.7166	1481.5549	-0.1617	-109	320	334	MSGGDHIHAGTVVGK			Oxidation (M)[1]		Mascot
1487.7086	1487.5428	-0.1658	-111	464	475	EIKFEFQAMDTL			Oxidation (M)[9]		Mascot
1503.7301	1503.5112	-0.2189	-146	451	463	WSPELAAACEIWK					Mascot
3854.8718	3854.3462	-0.5256	-136	42	79	VTPQPGVPPEEAGAAVAESSTGTWTTVWTDGLTSLDR	252	100			Mascot
3854.8718	3854.3462	-0.5256	-136	42	79	VTPQPGVPPEEAGAAVAESSTGTWTTVWTDGLTSLDR					Mascot

Rank	Protein Name	Accession No	Protein MW	Protein PI	Pep. Count	Protein Score	Protein Score C. I. %	Total Ion Score	Total Ion C. I. %
8	Ribulose bisphosphate carboxylase large chain (Fragment) OS=Sedum rubrotinctum GN=rbcL PE=3 SV=1	RBL_SEDRU	49773.1	6.23	7	501	100	474	100

Peptide Information

Calc. Mass	Obsrv. Mass	± da	± ppm	Start Seq.	End Seq.	Sequence	Ion Score	C. I. %	Modification	Rank	Result Type
805.4929	805.3775	-0.1154	-143	1	8	VGFKAGVK					Mascot
805.4929	805.3775	-0.1154	-143	1	8	VGFKAGVK					Mascot
1021.5312	1021.4022	-0.129	-126	23	31	DTDILAAFR					Mascot
1021.5312	1021.4022	-0.129	-126	23	31	DTDILAAFR	70	99.999			Mascot
1168.6255	1168.4816	-0.1439	-123	286	295	AMHAVIDRQK					Mascot
1393.6522	1393.4695	-0.1827	-131	12	22	LTYYTPDYETK					Mascot
1393.6522	1393.4695	-0.1827	-131	12	22	LTYYTPDYETK	57	99.974			Mascot
1465.7546	1465.5664	-0.1882	-128	137	149	TFQGPPHGIQVER	95	100			Mascot
1465.7546	1465.5664	-0.1882	-128	137	149	TFQGPPHGIQVER					Mascot
1481.7166	1481.5549	-0.1617	-109	310	324	MSGGDHIHSGTVVGK					Mascot
3854.8718	3854.3462	-0.5256	-136	32	69	VTPQPGVPPEEAGAAVAESSTGTWTTVWTDGLTSLDR	252	100			Mascot
3854.8718	3854.3462	-0.5256	-136	32	69	VTPQPGVPPEEAGAAVAESSTGTWTTVWTDGLTSLDR					Mascot

Rank	Protein Name	Accession No	Protein MW	Protein PI	Pep. Count	Protein Score	Protein Score C. I. %	Total Ion Score	Total Ion C. I. %
9	Ribulose bisphosphate carboxylase large chain (Fragment) OS=Polygala cruciata GN=rbcL PE=3 SV=2	RBL_POLCR	51580.1	6.23	7	500	100	474	100

Peptide Information

Calc. Mass	Obsrv. Mass	± da	± ppm	Start Seq.	End Seq.	Sequence	Ion Score	C. I. %	Modification	Rank	Result Type
805.4929	805.3775	-0.1154	-143	1	8	VGFKAGVK					Mascot
805.4929	805.3775	-0.1154	-143	1	8	VGFKAGVK					Mascot
1021.5312	1021.4022	-0.129	-126	23	31	DTDILAAFR					Mascot
1021.5312	1021.4022	-0.129	-126	23	31	DTDILAAFR	70	99.999			Mascot
1168.6255	1168.4816	-0.1439	-123	286	295	AMHAVIDRQK					Mascot
1393.6522	1393.4695	-0.1827	-131	12	22	LTYYTPDYETK					Mascot
1393.6522	1393.4695	-0.1827	-131	12	22	LTYYTPDYETK	57	99.974			Mascot
1447.7651	1447.5518	-0.2133	-147	310	324	LSGGDHIHAGTVVGK					Mascot
1465.7546	1465.5664	-0.1882	-128	137	149	TFQGPPHGIQVER	95	100			Mascot
1465.7546	1465.5664	-0.1882	-128	137	149	TFQGPPHGIQVER					Mascot
3854.8718	3854.3462	-0.5256	-136	32	69	VTPQPGVPPEEAGAAVAESSTGTWTTVWTDGLTSLDR	252	100			Mascot
3854.8718	3854.3462	-0.5256	-136	32	69	VTPQPGVPPEEAGAAVAESSTGTWTTVWTDGLTSLDR					Mascot

Rank	Protein Name	Accession No	Protein MW	Protein PI	Pep. Count	Protein Score	Protein Score C. I. %	Total Ion Score	Total Ion C. I. %
10	Ribulose bisphosphate carboxylase large chain (Fragment) OS=Platytheca verticillata GN=rbcL PE=3 SV	RBL_PLAVR	51588.1	6.04	7	500	100	474	100

Peptide Information

Calc. Mass	Obsrv. Mass	± da	± ppm	Start Seq.	End Seq.	Sequence	Ion Score	C. I. %	Modification	Rank	Result Type
805.4929	805.3775	-0.1154	-143	1	8	VGFKAGVK					Mascot
805.4929	805.3775	-0.1154	-143	1	8	VGFKAGVK					Mascot
1021.5312	1021.4022	-0.129	-126	23	31	DTDILAAFR					Mascot
1021.5312	1021.4022	-0.129	-126	23	31	DTDILAAFR	70	99.999			Mascot
1168.6255	1168.4816	-0.1439	-123	286	295	AMHAVIDRQK					Mascot
1393.6522	1393.4695	-0.1827	-131	12	22	LTYYTPDYETK					Mascot
1393.6522	1393.4695	-0.1827	-131	12	22	LTYYTPDYETK	57	99.974			Mascot
1465.7546	1465.5664	-0.1882	-128	137	149	TFQGPPHGIQVER	95	100			Mascot
1465.7546	1465.5664	-0.1882	-128	137	149	TFQGPPHGIQVER					Mascot
1481.7166	1481.5549	-0.1617	-109	310	324	MSGGDHIHSGTVVGK					Mascot
3854.8718	3854.3462	-0.5256	-136	32	69	VTPQPGVPPEEAGAAVAESSTGTWTTVWTDGLTSLDR	252	100			Mascot
3854.8718	3854.3462	-0.5256	-136	32	69	VTPQPGVPPEEAGAAVAESSTGTWTTVWTDGLTSLDR					Mascot

Gel Idx/Pos	109/E8	Instr./Gel Origin	BA2060/131017A	Process Sta	Analysis Succeeded
Plate [#] Name	[1] 31496	Instrument Sample Name		Spectra	8

Rank	Protein Name	Accession No	Protein MW	Protein PI	Pep. Count	Protein Score	Protein Score C. I. %	Total Ion Score	Total Ion C. I. %
1	Chain B, Refined 1.8 Angstroms Resolution Cryst Structure Of Porcine Epsilon-Trypsin	gi|999627	8813.5	6.67	4	115	99.997	83	99.99

Peptide Information

Calc. Mass	Obsrv. Mass	± da	± ppm	Start Seq.	End Seq.	Sequence	Ion Score	C. I. %	Modification	Rank	Result Type
842.5094	842.4023	-0.1071	-127	57	64	VATVSLPR					Mascot
1045.5636	1045.4271	-0.1365	-131	47	56	LSSPATLNSR					Mascot
2211.104	2210.804	-0.3	-136	7	26	LGEHNIDVLEGNEQFINAK	83	99.99			Mascot
2211.104	2210.804	-0.3	-136	7	26	LGEHNIDVLEGNEQFINAK					Mascot
2299.175	2298.8679	-0.3071	-134	27	46	IITHPNFNGNTLDNDIMLIK			Oxidation (M)[17]		Mascot

Rank	Protein Name	Accession No	Protein MW	Protein PI	Pep. Count	Protein Score	Protein Score C. I. %	Total Ion Score	Total Ion C. I. %
2	Chain A, Crystal Structure Of The First Active Autolysate Form Of The Porcine Alpha Trypsin	gi|1942351	13283.6	7.83	4	108	99.986	83	99.99

Peptide Information

Calc. Mass	Obsrv. Mass	± da	± ppm	Start Seq.	End Seq.	Sequence	Ion Score	C. I. %	Modification	Rank	Result Type
842.5094	842.4023	-0.1071	-127	100	107	VATVSLPR					Mascot
1045.5636	1045.4271	-0.1365	-131	90	99	LSSPATLNSR					Mascot
2211.104	2210.804	-0.3	-136	50	69	LGEHNIDVLEGNEQFINAK	83	99.99			Mascot
2211.104	2210.804	-0.3	-136	50	69	LGEHNIDVLEGNEQFINAK					Mascot
2299.175	2298.8679	-0.3071	-134	70	89	IITHPNFNGNTLDNDIMLIK			Oxidation (M)[17]		Mascot

Rank	Protein Name	Accession No	Protein MW	Protein PI	Pep. Count	Protein Score	Protein Score C. I. %	Total Ion Score	Total Ion C. I. %
3	Chain E, Complex Of Ecti-Ii With Porcine Trypsin	gi|157878102	24156.7	8.26	5	108	99.986	83	99.99

Peptide Information

Calc. Mass	Obsrv. Mass	± da	± ppm	Start Seq.	End Seq.	Sequence	Ion Score	C. I. %	Modification	Rank	Result Type
842.5094	842.4023	-0.1071	-127	103	110	VATVSLPR					Mascot
1045.5636	1045.4271	-0.1365	-131	93	102	LSSPATLNSR					Mascot
2211.104	2210.804	-0.3	-136	53	72	LGEHNIDVLEGNEQFINAK	83	99.99			Mascot
2211.104	2210.804	-0.3	-136	53	72	LGEHNIDVLEGNEQFINAK					Mascot
2299.175	2298.8679	-0.3071	-134	73	92	IITHPNFNGNTLDNDIMLIK			Oxidation (M)[17]		Mascot
2736.3442	2736.092	-0.2522	-92	103	129	VATVSLPRSCAAAGTECSLISGWGNTK			Carbamidomethyl (C)[10]		Mascot
2736.3442	2736.092	-0.2522	-92	103	129	VATVSLPRSCAAAGTECSLISGWGNTK			Carbamidomethyl (C)[10]		Mascot

Rank	Protein Name	Accession No	Protein MW	Protein PI	Pep. Count	Protein Score	Protein Score C. I. %	Total Ion Score	Total Ion C. I. %
4	Chain A, The Refined 1.6 Angstroms Resolution Structure Of The Complex Formed Between Porci	gi|494360	23458.5	8.6	5	108	99.986	83	99.99

Peptide Information

Calc. Mass	Obsrv. Mass	± da	± ppm	Start Seq.	End Seq.	Sequence	Ion Score	C. I. %	Modification	Rank	Result Type
842.5094	842.4023	-0.1071	-127	100	107	VATVSLPR					Mascot
1045.5636	1045.4271	-0.1365	-131	90	99	LSSPATLNSR					Mascot
2211.104	2210.804	-0.3	-136	50	69	LGEHNIDVLEGNEQFINAK	83	99.99			Mascot
2211.104	2210.804	-0.3	-136	50	69	LGEHNIDVLEGNEQFINAK					Mascot
2230.063	2229.8923	-0.1707	-77	150	170	SSYPGQITGNMICVGFLQGGK			Carbamidomethyl (C)[13], Oxidation (M)[11]		Mascot
2299.175	2298.8679	-0.3071	-134	70	89	IITHPNFNGNTLDNDIMLIK			Oxidation (M)[17]		Mascot

Rank	Protein Name	Accession No	Protein MW	Protein PI	Pep. Count	Protein Score	Protein Score C. I. %	Total Ion Score	Total Ion C. I. %
5	Chain A, Trypsin In Complex With Borate	gi|110590762	23458.3	8.22	4	100	99.914	83	99.99

Peptide Information

Calc. Mass	Obsrv. Mass	± da	± ppm	Start Seq.	End Seq.	Sequence	Ion Score	C. I. %	Modification	Rank	Result Type
842.5094	842.4023	-0.1071	-127	100	107	VATVSLPR					Mascot
1045.5636	1045.4271	-0.1365	-131	90	99	LSSPATLNSR					Mascot
2211.104	2210.804	-0.3	-136	50	69	LGEHNIDVLEGNEQFINAK	83	99.99			Mascot
2211.104	2210.804	-0.3	-136	50	69	LGEHNIDVLEGNEQFINAK					Mascot
2299.175	2298.8679	-0.3071	-134	70	89	IITHPNFNGNTLDNDIMLIK			Oxidation (M)[17]		Mascot
	2298.8679										Mascot

Rank	Protein Name	Accession No	Protein MW	Protein PI	Pep. Count	Protein Score	Protein Score C. I. %	Total Ion Score	Total Ion C. I. %
6	RecName: Full=Trypsin; Flags: Precursor	gi|136429	24393.8	7	4	100	99.912	83	99.99

Peptide Information

Calc. Mass	Obsrv. Mass	± da	± ppm	Start Seq.	End Seq.	Sequence	Ion Score	C. I. %	Modification	Rank	Result Type
842.5094	842.4023	-0.1071	-127	108	115	VATVSLPR					Mascot
1045.5636	1045.4271	-0.1365	-131	98	107	LSSPATLNSR					Mascot
2211.104	2210.804	-0.3	-136	58	77	LGEHNIDVLEGNEQFINAK	83	99.99			Mascot
2211.104	2210.804	-0.3	-136	58	77	LGEHNIDVLEGNEQFINAK					Mascot
2299.175	2298.8679	-0.3071	-134	78	97	IITHPNFNGNTLDNDIMLIK			Oxidation (M)[17]		Mascot

Rank	Protein Name	Accession No	Protein MW	Protein PI	Pep. Count	Protein Score	Protein Score C. I. %	Total Ion Score	Total Ion C. I. %
7	Chain E, Leech-Derived Tryptase Inhibitor/TRYPS COMPLEX	gi|3318722	23457.4	8.26	3	95	99.701	83	99.99

Peptide Information

Calc. Mass	Obsrv. Mass	± da	± ppm	Start Seq.	End Seq.	Sequence	Ion Score	C. I. %	Modification	Rank	Result Type
842.5094	842.4023	-0.1071	-127	100	107	VATVSLPR					Mascot
2211.104	2210.804	-0.3	-136	50	69	LGEHNIDVLEGNEQFINAK	83	99.99			Mascot
2211.104	2210.804	-0.3	-136	50	69	LGEHNIDVLEGNEQFINAK					Mascot
2299.175	2298.8679	-0.3071	-134	70	89	IITHPNFNGNTLDNDIMLIK			Oxidation (M)[17]		Mascot

Rank	Protein Name	Accession No	Protein MW	Protein PI	Pep. Count	Protein Score	Protein Score C. I. %	Total Ion Score	Total Ion C. I. %
8	Ribosomal protein L7/L12 [Crocosphaera watsoni 8501]	gi|67924345	13803.2	4.73	2	80	91.188	71	99.829

Peptide Information

Calc. Mass	Obsrv. Mass	± da	± ppm	Start Seq.	End Seq.	Sequence	Ion Score	C. I. %	Modification	Rank	Result Type
845.4727	845.3618	-0.1109	-131	122	129	KLEEAGAK					Mascot
1704.869	1704.6167	-0.2523	-148	64	78	TEFDVVLEEVPGDKK	71	99.829			Mascot
1704.869	1704.6167	-0.2523	-148	64	78	TEFDVVLEEVPGDKK					Mascot

Rank	Protein Name	Accession No	Protein MW	Protein PI	Pep. Count	Protein Score	Protein Score C. I. %	Total Ion Score	Total Ion C. I. %
9	50S ribosomal protein L7/L12 [Cyanothece sp. P(8801]	gi|218246569	13750.2	4.65	2	58	0	49	76.309

Peptide Information

Calc. Mass	Obsrv. Mass	± da	± ppm	Start Seq.	End Seq.	Sequence	Ion Score	C. I. %	Modification	Rank	Result Type
845.4363	845.3618	-0.0745	-88	121	128	QLEEAGAK					Mascot
1704.869	1704.6167	-0.2523	-148	63	77	TEFDVVLEDVPADKK	49	76.309			Mascot
1704.869	1704.6167	-0.2523	-148	63	77	TEFDVVLEDVPADKK					Mascot

Rank	Protein Name	Accession No	Protein MW	Protein PI	Pep. Count	Protein Score	Protein Score C. I. %	Total Ion Score	Total Ion C. I. %
10	hypothetical protein CLK_0227 [Clostridium botul A3 str. Loch Maree]	gi|170759757	52691.2	8.63	11	54	0		

Peptide Information

Calc. Mass	Obsrv. Mass	± da	± ppm	Start Seq.	End Seq.	Sequence	Ion Score	C. I. %	Modification	Rank	Result Type
816.4573	816.4996	0.0423	52	120	127	NLANISGK					Mascot
845.425	845.3618	-0.0632	-75	204	210	TPEELEK					Mascot
1045.5823	1045.4271	-0.1552	-148	304	312	VQKAIQCAK			Carbamidomethyl (C)[7]		Mascot
1165.6034	1165.5938	-0.0096	-8	75	83	LPIYMDKNR			Oxidation (M)[5]		Mascot
1275.6176	1275.5607	-0.0569	-45	28	38	ENEAQVKENSK					Mascot
1275.6176	1275.5607	-0.0569	-45	28	38	ENEAQVKENSK					Mascot
2211.0535	2210.804	-0.2495	-113	139	156	EDIMYMSLFDFNKIMGLK			Oxidation (M)[4]		Mascot
2211.0535	2210.804	-0.2495	-113	139	156	EDIMYMSLFDFNKIMGLK			Oxidation (M)[4]		Mascot
2239.0662	2238.8394	-0.2268	-101	134	151	DTILKEDIMYMSLFDFNK			Oxidation (M)[9]		Mascot
2246.1704	2245.9143	-0.2561	-114	364	383	TVTYIPTPLDYVDGIKHADK					Mascot
2296.1106	2295.8074	-0.3032	-132	335	352	IIEQYFNYMYEPPKFASR					Mascot
3082.519	3082.2815	-0.2375	-77	307	334	AIQCAKILDIPVGFFESAHYSATNEETK					Mascot
3346.8523	3347.2144	0.3621	108	6	34	ILILILFCFLIGITSYFFVGAKENEAQVK			Carbamidomethyl (C)[8]		Mascot

Gel Idx/Pos	110/E9	Instr./Gel Origin	BA2060/131017A	Process Sta	Analysis Succeeded
Plate [#] Name	[1] 31496	Instrument Sample Name		Spectra	8

Rank	Protein Name	Accession No	Protein MW	Protein PI	Pep Count	Protein Score	Protein Score C. I. %	Total Ion Score	Total Ion C. I. %
1	Glycine cleavage system H protein 1, mitochondri OS=Arabidopsis thaliana GN=GDCSH PE=1 SV=1	GCSH1_ARAT	17935.9	5.24	4	68	99.502	46	99.853

Peptide Information

Calc. Mass	Obsrv. Mass	± da	± ppm	Start Seq.	End Seq.	Sequence	Ion Score	C. I. %	Modification	Rank	Result Type
1179.5826	1179.4495	-0.1331	-113	5	15	MWASSTANALK					Mascot
2211.0903	2210.811	-0.2793	-126	33	51	CFSTVLEGLKYANSHEWVK					Mascot
2211.0903	2210.811	-0.2793	-126	33	51	CFSTVLEGLKYANSHEWVK					Mascot
2237.0793	2236.7866	-0.2927	-131	117	136	LTESPGLINSSPYEDGWMIK					Mascot
2253.0745	2252.7791	-0.2954	-131	117	136	LTESPGLINSSPYEDGWMIK	46	99.853	Oxidation (M)[18]		Mascot
2253.0745	2252.7791	-0.2954	-131	117	136	LTESPGLINSSPYEDGWMIK			Oxidation (M)[18]		Mascot
2365.1743	2364.8491	-0.3252	-137	116	136	KLTESPGLINSSPYEDGWMIK					Mascot
2381.1692	2380.8564	-0.3128	-131	116	136	KLTESPGLINSSPYEDGWMIK			Oxidation (M)[19]		Mascot

Rank	Protein Name	Accession No	Protein MW	Protein PI	Pep Count	Protein Score	Protein Score C. I. %	Total Ion Score	Total Ion C. I. %
2	Probable glycine cleavage system H protein 2, mitochondrial OS=Arabidopsis thaliana GN=At1g32470 PE	GCSH2_ARAT	17885.9	5.11	2	54	87.5	46	99.853

Peptide Information

Calc. Mass	Obsrv. Mass	± da	± ppm	Start Seq.	End Seq.	Sequence	Ion Score	C. I. %	Modification	Rank	Result Type
1179.5826	1179.4495	-0.1331	-113	5	15	MWASSTANALK					Mascot
2237.0793	2236.7866	-0.2927	-131	118	137	LTESPGLINSSPYEDGWMIK					Mascot
2253.0745	2252.7791	-0.2954	-131	118	137	LTESPGLINSSPYEDGWMIK	46	99.853	Oxidation (M)[18]		Mascot
2253.0745	2252.7791	-0.2954	-131	118	137	LTESPGLINSSPYEDGWMIK			Oxidation (M)[18]		Mascot

Rank	Protein Name	Accession No	Protein MW	Protein PI	Pep Count	Protein Score	Protein Score C. I. %	Total Ion Score	Total Ion C. I. %
3	Glycine cleavage system H protein, mitochondrial OS=Oryza sativa subsp. japonica GN=GDCSH PE=1 SV=1	GCSH_ORYS	17356.6	4.92	2	36	0	27	88.668

Peptide Information

Calc. Mass	Obsrv. Mass	± da	± ppm	Start Seq.	End Seq.	Sequence	Ion Score	C. I. %	Modification	Rank	Result Type
2237.0793	2236.7866	-0.2927	-131	117	136	LSETPGLINSSPYEDGWMIK					Mascot
2246.1128	2245.9307	-0.1821	-81	33	51	YFSTVLDGLKYSSSHEWVK					Mascot
2253.0745	2252.7791	-0.2954	-131	117	136	LSETPGLINSSPYEDGWMIK	27	88.668	Oxidation (M)[18]		Mascot
2253.0745	2252.7791	-0.2954	-131	117	136	LSETPGLINSSPYEDGWMIK			Oxidation (M)[18]		Mascot

Rank	Protein Name	Accession No	Protein MW	Protein PI	Pep Count	Protein Score	Protein Score C. I. %	Total Ion Score	Total Ion C. I. %
4	Glycine cleavage system H protein, mitochondrial OS=Flaveria pringlei GN=GDCSH PE=2 SV=1	GCSH_FLAP	17341.5	5.04	2	36	0	27	88.668

Peptide Information

Calc. Mass	Obsrv. Mass	± da	± ppm	Start Seq.	End Seq.	Sequence	Ion Score	C. I. %	Modification	Rank	Result Type
1123.3997	1123.2555	-0.1442	-128	154	162	FCEEEDSAH			Carbamidomethyl (C)[2]		Mascot
2237.0793	2236.7866	-0.2927	-131	114	133	LSETPGLINSSPYEDGWMIK					Mascot
2253.0745	2252.7791	-0.2954	-131	114	133	LSETPGLINSSPYEDGWMIK	27	88.668	Oxidation (M)[18]		Mascot
2253.0745	2252.7791	-0.2954	-131	114	133	LSETPGLINSSPYEDGWMIK			Oxidation (M)[18]		Mascot

Rank	Protein Name	Accession No	Protein MW	Protein PI	Pep Count	Protein Score	Protein Score C. I. %	Total Ion Score	Total Ion C. I. %
5	Glycine cleavage system H protein, mitochondrial (Fragment) OS=Flaveria pubescens GN=GDCSH PE=2 SV=	GCSH_FLAP	16186	5.27	1	32	0	27	88.668

Peptide Information

Calc. Mass	Obsrv. Mass	± da	± ppm	Start Seq.	End Seq.	Sequence	Ion Score	C. I. %	Modification	Rank	Result Type
2237.0793	2236.7866	-0.2927	-131	114	133	LSETPGLINSSPYEDGWMIK					Mascot
2253.0745	2252.7791	-0.2954	-131	114	133	LSETPGLINSSPYEDGWMIK	27	88.668	Oxidation (M)[18]		Mascot
2253.0745	2252.7791	-0.2954	-131	114	133	LSETPGLINSSPYEDGWMIK			Oxidation (M)[18]		Mascot

Rank	Protein Name	Accession No	Protein MW	Protein PI	Pep Count	Protein Score	Protein Score C. I. %	Total Ion Score	Total Ion C. I. %
6	Metallothionein-like protein type 2, MT2-28 OS=Brassica juncea PE=3 SV=1	MT25_BRAJU	8053.2	4.55	5	32	0		

Peptide Information

Calc. Mass	Obsrv. Mass	± da	± ppm	Start Seq.	End Seq.	Sequence	Ion Score	C. I. %	Modification	Rank	Result Type
882.3266	882.4546	0.128	145	74	80	CNPCTCK			Carbamidomethyl (C)[1,4]		Mascot
1475.5204	1475.5743	0.0539	37	68	80	CGSDCKCNPCTCK			Carbamidomethyl (C)[1,5]		Mascot
1651.5208	1651.6531	0.1323	80	1	15	MSCCGGNCGCGAGCK			Carbamidomethyl (C)[3,4,8,10,14], Oxidation (M)[1]		Mascot
2268.7622	2268.7661	0.0039	2	2	23	SCCGGNCGCGAGCKCVGCGGCK			Carbamidomethyl (C)[2,3,7,9,13,15]		Mascot
2285.7598	2285.7756	0.0158	7	1	23	MSCCGGNCGCGAGCKCVGCGGCK			Carbamidomethyl (C)[3,4,8,10]		Mascot

Rank	Protein Name	Accession No	Protein MW	Protein PI	Pep Count	Protein Score	Protein Score C. I. %	Total Ion Score	Total Ion C. I. %
7	Glycine cleavage system H protein, mitochondrial OS=Flaveria anomala GN=GDCSH PE=2 SV=1	GCSH_FLAAN	17343.5	5.04	1	32	0	27	88.668

Peptide Information

Calc. Mass	Obsrv. Mass	± da	± ppm	Start Seq.	End Seq.	Sequence	Ion Score	C. I. %	Modification	Rank	Result Type
2237.0793	2236.7866	-0.2927	-131	114	133	LSETPGLINSSPYEDGWMIK					Mascot
2253.0745	2252.7791	-0.2954	-131	114	133	LSETPGLINSSPYEDGWMIK	27	88.668	Oxidation (M)[18]		Mascot
2253.0745	2252.7791	-0.2954	-131	114	133	LSETPGLINSSPYEDGWMIK			Oxidation (M)[18]		Mascot

Rank	Protein Name	Accession No	Protein MW	Protein PI	Pep Count	Protein Score	Protein Score C. I. %	Total Ion Score	Total Ion C. I. %
8	Putative small RNA degrading nuclease 4 OS=Arabidopsis thaliana GN=SDN4 PE=3 SV=1	SDN4_ARAT	36191.8	8.74	6	30	0		

Peptide Information

Calc. Mass	Obsrv. Mass	± da	± ppm	Start Seq.	End Seq.	Sequence	Ion Score	C. I. %	Modification	Rank	Result Type
850.5219	850.428	-0.0939	-110	15	21	LMIAKFK					Mascot
1027.47	1027.3887	-0.0813	-79	1	8	MDYLQAMR					Mascot
1045.5823	1045.4352	-0.1471	-141	11	19	EANRLMIAK					Mascot
3352.8779	3353.2517	0.3738	111	145	174	LRPFLSAGAILIDHPIVIDTSLVFKYPNSR					Mascot
3769.8027	3769.3748	-0.4279	-114	178	211	RPSLNTLCMSVLGYEVQKAGVSHHCVHDAAAAMK			Carbamidomethyl (C)[8,25], Oxidation (M)[9,33]		Mascot
3794.7712	3794.3394	-0.4318	-114	66	100	MSNVMIPNQMLALDCEMVLCEDGTEGVVRVGAVDR					Mascot

Rank	Protein Name	Accession No	Protein MW	Protein PI	Pep Count	Protein Score	Protein Score C. I. %	Total Ion Score	Total Ion C. I. %
9	Putative cytochrome c oxidase subunit II PS17 (Fragments) OS=Pinus strobus PE=1 SV=1	PS17_PINST	1707	9.63	2	28	0	6	0

Peptide Information

Calc. Mass	Obsrv. Mass	± da	± ppm	Start Seq.	End Seq.	Sequence	Ion Score	C. I. %	Modification	Rank	Result Type
856.525	856.4188	-0.1062	-124	1	8	SPTVIALR					Mascot
870.5043	870.4302	-0.0741	-85	9	16	VVEALSPR					Mascot
870.5043	870.4302	-0.0741	-85	9	16	VVEALSPR	6	0			Mascot

Rank	Protein Name	Accession No	Protein MW	Protein PI	Pep Count	Protein Score	Protein Score C. I. %	Total Ion Score	Total Ion C. I. %
10	Cytochrome P450 71D10 OS=Glycine max GN=CYP71D10 PE=2 SV=1	C71DA_SOYB	58425.4	8.59	8	27	0		

Peptide Information

Calc. Mass	Obsrv. Mass	± da	± ppm	Start Seq.	End Seq.	Sequence	Ion Score	C. I. %	Modification	Rank	Result Type
818.4254	818.3576	-0.0678	-83	297	303	LTDDNIK					Mascot
882.4904	882.4546	-0.0358	-41	251	257	VHRVTDR					Mascot
1045.5411	1045.4352	-0.1059	-101	162	170	EEEVAELVK					Mascot
1275.7341	1275.5721	-0.162	-127	142	152	KICTVELLTAK			Carbamidomethyl (C)[3]		Mascot
1820.9429	1821.1666	0.2237	123	350	364	GYVDETELHQLIYLK					Mascot Mascot
3263.6365	3264.0974	0.4609	141	304	333	AVIQDIFIGGGETSSSVVEWGMSELIRNPR			Oxidation (M)[22]		Mascot
3337.7522	3337.3416	-0.4106	-123	2	28	VMELHNHTPFSIYFITSILFIFFVFFK					Mascot
3353.7471	3353.2517	-0.4954	-148	2	28	VMELHNHTPFSIYFITSILFIFFVFFK			Oxidation (M)[2]		Mascot
3679.8523	3679.3164	-0.5359	-146	297	330	LTDDNIKAVIQDIFIGGGETSSSVVEWGMSELIR					Mascot

Gel Idx/Pos	111/E10	Instr./Gel Origin	BA2060/131017A	Process Sta	Analysis Succeeded
Plate [#] Name	[1] 31496	Instrument Sample Name		Spectra	8

Rank	Protein Name	Accession No	Protein MW	Protein PI	Pep. Count	Protein Score	Protein Score C. I. %	Total Ion Score	Total Ion C. I. %
1	Ribulose bisphosphate carboxylase large chain OS=Sesbania sesban GN=rbcL PE=3 SV=1	RBL_SESSE	52358.4	6.09	8	219	100	187	100

Peptide Information

Calc. Mass	Obsrv. Mass	± da	± ppm	Start Seq.	End Seq.	Sequence	Ion Score	C. I. %	Modification	Rank	Result Type
856.3546	856.4232	0.0686	80	467	473	FEFPAMD					Mascot
856.3546	856.4232	0.0686	80	467	473	FEFPAMD					Mascot
914.4049	914.2906	-0.1143	-125	306	312	NHGMHFR			Oxidation (M)[4]		Mascot
921.4346	921.3887	-0.0459	-50	1	8	MSPQTETK					Mascot
1021.5312	1021.4089	-0.1223	-120	33	41	DTDILAAFR					Mascot
1021.5312	1021.4089	-0.1223	-120	33	41	DTDILAAFR	63	99.995			Mascot
1039.4691	1039.3938	-0.0753	-72	351	358	DDYVEKDR					Mascot
1393.6522	1393.4775	-0.1747	-125	22	32	LTYYTPDYETK					Mascot
1393.6522	1393.4775	-0.1747	-125	22	32	LTYYTPDYETK	44	99.626			Mascot
2169.9868	2169.8855	-0.1013	-47	195	213	GGLDFTKDDENVNSQPFMR					Mascot
3854.8718	3854.3867	-0.4851	-126	42	79	VTPQPGVPPEEAGAAVAAESSTGTWTTVWTDGLTSLDR	79	100			Mascot
3854.8718	3854.3867	-0.4851	-126	42	79	VTPQPGVPPEEAGAAVAAESSTGTWTTVWTDGLTSLDR					Mascot

Rank	Protein Name	Accession No	Protein MW	Protein PI	Pep. Count	Protein Score	Protein Score C. I. %	Total Ion Score	Total Ion C. I. %
2	Ribulose bisphosphate carboxylase large chain (Fragment) OS=Salacia pallescens GN=rbcL PE=3 SV=2	RBL_SALPL	49365.3	6.25	7	214	100	187	100

Peptide Information

Calc. Mass	Obsrv. Mass	± da	± ppm	Start Seq.	End Seq.	Sequence	Ion Score	C. I. %	Modification	Rank	Result Type
914.4049	914.2906	-0.1143	-125	306	312	NHGMHFR			Oxidation (M)[4]		Mascot
921.4346	921.3887	-0.0459	-50	1	8	MSPQTETK					Mascot
1021.5312	1021.4089	-0.1223	-120	33	41	DTDILAAFR					Mascot
1021.5312	1021.4089	-0.1223	-120	33	41	DTDILAAFR	63	99.995			Mascot
1045.5459	1045.4387	-0.1072	-103	422	431	VAVEACVQAR					Mascot
1393.6522	1393.4775	-0.1747	-125	22	32	LTYYTPDYETK					Mascot
1393.6522	1393.4775	-0.1747	-125	22	32	LTYYTPDYETK	44	99.626			Mascot
2169.9868	2169.8855	-0.1013	-47	195	213	GGLDFTKDDENVNSQPFMR					Mascot
3854.8718	3854.3867	-0.4851	-126	42	79	VTPQPGVPPEEAGAAVAAESSTGTWTTVWTDGLTSLDR	79	100			Mascot
3854.8718	3854.3867	-0.4851	-126	42	79	VTPQPGVPPEEAGAAVAAESSTGTWTTVWTDGLTSLDR					Mascot

Rank	Protein Name	Accession No	Protein MW	Protein PI	Pep. Count	Protein Score	Protein Score C. I. %	Total Ion Score	Total Ion C. I. %
3	Ribulose bisphosphate carboxylase large chain OS=Lotus japonicus GN=rbcL PE=3 SV=1	RBL_LOTJA	52739.5	6.04	7	212	100	187	100

Peptide Information

Calc. Mass	Obsrv. Mass	± da	± ppm	Start Seq.	End Seq.	Sequence	Ion Score	C. I. %	Modification	Rank	Result Type
914.4049	914.2906	-0.1143	-125	306	312	NHGMHFR			Oxidation (M)[4]		Mascot
921.4346	921.3887	-0.0459	-50	1	8	MSPQTETK					Mascot
1021.5312	1021.4089	-0.1223	-120	33	41	DTDILAAFR					Mascot
1021.5312	1021.4089	-0.1223	-120	33	41	DTDILAAFR	63	99.995			Mascot
1393.6522	1393.4775	-0.1747	-125	22	32	LTYYTPDYETK					Mascot
1393.6522	1393.4775	-0.1747	-125	22	32	LTYYTPDYETK	44	99.626			Mascot
2169.9868	2169.8855	-0.1013	-47	195	213	GGLDFTKDDENVNSQPFMR					Mascot
2231.1052	2230.9077	-0.1975	-89	218	236	FLFCAEALFKAQEETGEIK			Carbamidomethyl (C)[4]		Mascot
3854.8718	3854.3867	-0.4851	-126	42	79	VTPQPGVPPEEAGAAVAAESSTGTWTTVWTDGLTSLDR	79	100			Mascot
3854.8718	3854.3867	-0.4851	-126	42	79	VTPQPGVPPEEAGAAVAAESSTGTWTTVWTDGLTSLDR					Mascot

Rank	Protein Name	Accession No	Protein MW	Protein PI	Pep. Count	Protein Score	Protein Score C. I. %	Total Ion Score	Total Ion C. I. %
4	Ribulose bisphosphate carboxylase large chain OS=Phalaenopsis aphrodite subsp. formosana GN=rbcL PE	RBL_PHAAO	53228.7	5.95	7	212	100	187	100

Peptide Information

Calc. Mass	Obsrv. Mass	± da	± ppm	Start Seq.	End Seq.	Sequence	Ion Score	C. I. %	Modification	Rank	Result Type
816.421	816.509	0.088	108	440	446	EGNDIIR					Mascot
914.4049	914.2906	-0.1143	-125	306	312	NHGMHFR			Oxidation (M)[4]		Mascot
921.4346	921.3887	-0.0459	-50	1	8	MSPQTETK					Mascot
1021.5312	1021.4089	-0.1223	-120	33	41	DTDILAAFR					Mascot
1021.5312	1021.4089	-0.1223	-120	33	41	DTDILAAFR	63	99.995			Mascot
1393.6522	1393.4775	-0.1747	-125	22	32	LTYYTPDYETK					Mascot
1393.6522	1393.4775	-0.1747	-125	22	32	LTYYTPDYETK	44	99.626			Mascot
2169.9868	2169.8855	-0.1013	-47	195	213	GGLDFTKDDENVNSQPFMR					Mascot
3854.8718	3854.3867	-0.4851	-126	42	79	VTPQPGVPPEEAGAAVAAESSTGTWTTVWTDGLTSLDR	79	100			Mascot
3854.8718	3854.3867	-0.4851	-126	42	79	VTPQPGVPPEEAGAAVAAESSTGTWTTVWTDGLTSLDR					Mascot

Rank	Protein Name	Accession No	Protein MW	Protein PI	Pep. Count	Protein Score	Protein Score C. I. %	Total Ion Score	Total Ion C. I. %
5	Ribulose bisphosphate carboxylase large chain (Fragment) OS=Acacia farnesiana GN=rbcL PE=3 SV=1	RBL_ACAFA	50346.3	6.22	6	208	100	187	100

Peptide Information

Calc. Mass	Obsrv. Mass	± da	± ppm	Start Seq.	End Seq.	Sequence	Ion Score	C. I. %	Modification	Rank	Result Type
914.4049	914.2906	-0.1143	-125	297	303	NHGMHFR			Oxidation (M)[4]		Mascot
1021.5312	1021.4089	-0.1223	-120	24	32	DTDILAAFR					Mascot
1021.5312	1021.4089	-0.1223	-120	24	32	DTDILAAFR	63	99.995			Mascot
1039.4691	1039.3938	-0.0753	-72	342	349	DDYVEKDR					Mascot
1393.6522	1393.4775	-0.1747	-125	13	23	LTYYTPDYETK					Mascot
1393.6522	1393.4775	-0.1747	-125	13	23	LTYYTPDYETK	44	99.626			Mascot
2169.9868	2169.8855	-0.1013	-47	186	204	GGLDFTKDDENVNSQPFMR					Mascot
3854.8718	3854.3867	-0.4851	-126	33	70	VTPQPGVPPEEAGAAVAAESSTGTWTTVWTDGLTSLDR	79	100			Mascot
3854.8718	3854.3867	-0.4851	-126	33	70	VTPQPGVPPEEAGAAVAAESSTGTWTTVWTDGLTSLDR					Mascot

Rank	Protein Name	Accession No	Protein MW	Protein PI	Pep. Count	Protein Score	Protein Score C. I. %	Total Ion Score	Total Ion C. I. %
6	Ribulose bisphosphate carboxylase large chain (Fragment) OS=Phelline comosa GN=rbcL PE=3 SV=2	RBL_PHECO	50393.5	6.58	6	208	100	187	100

Peptide Information

Calc. Mass	Obsrv. Mass	± da	± ppm	Start Seq.	End Seq.	Sequence	Ion Score	C. I. %	Modification	Rank	Result Type
816.421	816.509	0.088	108	440	446	EGNDIIR					Mascot
921.4346	921.3887	-0.0459	-50	1	8	MSPQTETK					Mascot
1021.5312	1021.4089	-0.1223	-120	33	41	DTDILAAFR					Mascot
1021.5312	1021.4089	-0.1223	-120	33	41	DTDILAAFR	63	99.995			Mascot
1393.6522	1393.4775	-0.1747	-125	22	32	LTYYTPDYETK					Mascot
1393.6522	1393.4775	-0.1747	-125	22	32	LTYYTPDYETK	44	99.626			Mascot
2169.9868	2169.8855	-0.1013	-47	195	213	GGLDFTKDDENVNSQPFMR					Mascot
3854.8718	3854.3867	-0.4851	-126	42	79	VTPQPGVPPEEAGAAVAAESSTGTWTTVWTDGLTSLDR	79	100			Mascot
3854.8718	3854.3867	-0.4851	-126	42	79	VTPQPGVPPEEAGAAVAAESSTGTWTTVWTDGLTSLDR					Mascot

Rank	Protein Name	Accession No	Protein MW	Protein PI	Pep. Count	Protein Score	Protein Score C. I. %	Total Ion Score	Total Ion C. I. %
7	Ribulose bisphosphate carboxylase large chain (Fragment) OS=Geum quellyon GN=rbcL PE=3 SV=1	RBL_GEUQU	50910.8	6.23	6	208	100	187	100

Peptide Information

Calc. Mass	Obsrv. Mass	± da	± ppm	Start Seq.	End Seq.	Sequence	Ion Score	C. I. %	Modification	Rank	Result Type
816.421	816.509	0.088	108	430	436	EGNEVIR					Mascot
914.4049	914.2906	-0.1143	-125	296	302	NHGMHFR			Oxidation (M)[4]		Mascot
1021.5312	1021.4089	-0.1223	-120	23	31	DTDILAAFR					Mascot
1021.5312	1021.4089	-0.1223	-120	23	31	DTDILAAFR	63	99.995			Mascot
1393.6522	1393.4775	-0.1747	-125	12	22	LTYYTPDYETK					Mascot
1393.6522	1393.4775	-0.1747	-125	12	22	LTYYTPDYETK	44	99.626			Mascot
2169.9868	2169.8855	-0.1013	-47	185	203	GGLDFTKDDENVNSQPFMR					Mascot
3854.8718	3854.3867	-0.4851	-126	32	69	VTPQPGVPPEEAGAAVAAESSTGTWTTVWTDGLTSLDR	79	100			Mascot
3854.8718	3854.3867	-0.4851	-126	32	69	VTPQPGVPPEEAGAAVAAESSTGTWTTVWTDGLTSLDR					Mascot

Rank	Protein Name	Accession No	Protein MW	Protein PI	Pep. Count	Protein Score	Protein Score C. I. %	Total Ion Score	Total Ion C. I. %
8	Ribulose bisphosphate carboxylase large chain (Fragment) OS=Streptopus lanceolatus GN=rbcL PE=3 SV=	RBL_STRLC	50649.4	6.18	6	208	100	187	100

Peptide Information

Calc. Mass	Obsrv. Mass	± da	± ppm	Start Seq.	End Seq.	Sequence	Ion Score	C. I. %	Modification	Rank	Result Type
914.4049	914.2906	-0.1143	-125	306	312	NHGMHFR			Oxidation (M)[4]		Mascot
921.4346	921.3887	-0.0459	-50	1	8	MSPQTETK					Mascot
1021.5312	1021.4089	-0.1223	-120	33	41	DTDILAAFR					Mascot
1021.5312	1021.4089	-0.1223	-120	33	41	DTDILAAFR	63	99.995			Mascot
1393.6522	1393.4775	-0.1747	-125	22	32	LTYYTPDYETK					Mascot
1393.6522	1393.4775	-0.1747	-125	22	32	LTYYTPDYETK	44	99.626			Mascot
2169.9868	2169.8855	-0.1013	-47	195	213	GGLDFTKDDENVNSQPFMR					Mascot
3854.8718	3854.3867	-0.4851	-126	42	79	VTPQPGVPPEEAGAAVAAESSTGTWTTVWTDGLTSLDR	79	100			Mascot
3854.8718	3854.3867	-0.4851	-126	42	79	VTPQPGVPPEEAGAAVAAESSTGTWTTVWTDGLTSLDR					Mascot

Rank	Protein Name	Accession No	Protein MW	Protein PI	Pep. Count	Protein Score	Protein Score C. I. %	Total Ion Score	Total Ion C. I. %
9	Ribulose bisphosphate carboxylase large chain (Fragment) OS=Fragaria ananassa GN=rbcL PE=3 SV=1	RBL_FRAAN	51628.9	6.04	6	207	100	187	100

Peptide Information

Calc. Mass	Obsrv. Mass	± da	± ppm	Start Seq.	End Seq.	Sequence	Ion Score	C. I. %	Modification	Rank	Result Type
816.421	816.509	0.088	108	430	436	EGNDIIR					Mascot
914.4049	914.2906	-0.1143	-125	296	302	NHGMHFR			Oxidation (M)[4]		Mascot
1021.5312	1021.4089	-0.1223	-120	23	31	DTDILAAFR					Mascot
1021.5312	1021.4089	-0.1223	-120	23	31	DTDILAAFR	63	99.995			Mascot
1393.6522	1393.4775	-0.1747	-125	12	22	LTYYTPDYETK					Mascot
1393.6522	1393.4775	-0.1747	-125	12	22	LTYYTPDYETK	44	99.626			Mascot
2169.9868	2169.8855	-0.1013	-47	185	203	GGLDFTKDDENVNSQPFMR					Mascot
3854.8718	3854.3867	-0.4851	-126	32	69	VTPQPGVPPEEAGAAVAAESSTGTWTTVWTDGLTSLDR	79	100			Mascot
3854.8718	3854.3867	-0.4851	-126	32	69	VTPQPGVPPEEAGAAVAAESSTGTWTTVWTDGLTSLDR					Mascot

Rank	Protein Name	Accession No	Protein MW	Protein PI	Pep. Count	Protein Score	Protein Score C. I. %	Total Ion Score	Total Ion C. I. %
10	Ribulose bisphosphate carboxylase large chain (Fragment) OS=Polygala cruciata GN=rbcL PE=3 SV=2	RBL_POLCR	51580.1	6.23	6	207	100	187	100

Peptide Information

Calc. Mass	Obsrv. Mass	± da	± ppm	Start Seq.	End Seq.	Sequence	Ion Score	C. I. %	Modification	Rank	Result Type
860.4658	860.5248	0.059	69	412	419	VALEACVR					Mascot
914.4049	914.2906	-0.1143	-125	296	302	NHGMHFR			Oxidation (M)[4]		Mascot
1021.5312	1021.4089	-0.1223	-120	23	31	DTDILAAFR					Mascot
1021.5312	1021.4089	-0.1223	-120	23	31	DTDILAAFR	63	99.995			Mascot
1393.6522	1393.4775	-0.1747	-125	12	22	LTYYTPDYETK					Mascot
1393.6522	1393.4775	-0.1747	-125	12	22	LTYYTPDYETK	44	99.607			Mascot
2169.9868	2169.8855	-0.1013	-47	185	203	GGLDFTKDDENVNSQPFMR					Mascot
3854.8718	3854.3867	-0.4851	-126	32	69	VTPQPGVPPEEAGAAVAAESSTGTWTTVWTDGLTSLDR	79	100			Mascot
3854.8718	3854.3867	-0.4851	-126	32	69	VTPQPGVPPEEAGAAVAAESSTGTWTTVWTDGLTSLDR					Mascot

Gel Idx/Pos	112/E11	Instr./Gel Origin	BA2060/131017A	Process Sta	Analysis Succeeded
Plate [#] Name	[1] 31496	Instrument Sample Name		Spectra	8

Rank	Protein Name	Accession No	Protein MW	Protein PI	Pep. Count	Protein Score	Protein Score C. I. %	Total Ion Score	Total Ion C. I. %
1	Ribulose bisphosphate carboxylase large chain OS=Sesbania sesban GN=rbcL PE=3 SV=1	RBL_SESSE	52358.4	6.09	9	333	100	295	100

Peptide Information

Calc. Mass	Obsrv. Mass	± da	± ppm	Start Seq.	End Seq.	Sequence	Ion Score	C. I. %	Modification	Rank	Result Type
856.3546	856.4217	0.0671	78	467	473	FEFPAMD					Mascot
914.4049	914.2933	-0.1116	-122	306	312	NHGMHFR			Oxidation (M)[4]		Mascot
921.4346	921.3905	-0.0441	-48	1	8	MSPQTETK					Mascot
1021.5312	1021.4114	-0.1198	-117	33	41	DTDILAAFR					Mascot
1021.5312	1021.4114	-0.1198	-117	33	41	DTDILAAFR	70	99.999			Mascot
1039.4691	1039.3998	-0.0693	-67	351	358	DDYVEKDR					Mascot
1168.6255	1168.4894	-0.1361	-116	296	305	AMHAVIDRQK					Mascot
1275.7307	1275.5737	-0.157	-123	340	350	EITLGFVDLLR					Mascot
1393.6522	1393.4844	-0.1678	-120	22	32	LTYYTPDYETK					Mascot
1393.6522	1393.4844	-0.1678	-120	22	32	LTYYTPDYETK	62	99.993			Mascot
3854.8718	3854.3855	-0.4863	-126	42	79	VTPQPGVPPEEAGAAVAAESSTGTWTTVWTDGLTSLDR	163	100			Mascot
3854.8718	3854.3855	-0.4863	-126	42	79	VTPQPGVPPEEAGAAVAAESSTGTWTTVWTDGLTSLDR					Mascot

Rank	Protein Name	Accession No	Protein MW	Protein PI	Pep. Count	Protein Score	Protein Score C. I. %	Total Ion Score	Total Ion C. I. %
2	Ribulose bisphosphate carboxylase large chain (Fragment) OS=Moringa oleifera GN=rbcL PE=3 SV=1	RBL_MOROL	51552.1	6.32	8	327	100	295	100

Peptide Information

Calc. Mass	Obsrv. Mass	± da	± ppm	Start Seq.	End Seq.	Sequence	Ion Score	C. I. %	Modification	Rank	Result Type
914.4049	914.2933	-0.1116	-122	297	303	NHGMHFR			Oxidation (M)[4]		Mascot
1021.5312	1021.4114	-0.1198	-117	24	32	DTDILAAFR					Mascot
1021.5312	1021.4114	-0.1198	-117	24	32	DTDILAAFR	70	99.999			Mascot
1038.5143	1038.4127	-0.1016	-98	458	466	FEFPAVDTL					Mascot
1168.6255	1168.4894	-0.1361	-116	287	296	AMHAVIDRQK					Mascot
1275.7307	1275.5737	-0.157	-123	331	341	EITLGFVDLLR					Mascot
1393.6522	1393.4844	-0.1678	-120	13	23	LTYYTPDYETK					Mascot
1393.6522	1393.4844	-0.1678	-120	13	23	LTYYTPDYETK	62	99.993			Mascot
1475.7351	1475.5814	-0.1537	-104	207	218	DRFLFCAEAIYK					Mascot
1475.7351	1475.5814	-0.1537	-104	207	218	DRFLFCAEAIYK					Mascot
3854.8718	3854.3855	-0.4863	-126	33	70	VTPQPGVPPEEAGAAVAAESSTGTWTTVWTDGLTSLDR	163	100			Mascot
											Mascot
3854.8718	3854.3855	-0.4863	-126	33	70	VTPQPGVPPEEAGAAVAAESSTGTWTTVWTDGLTSLDR					Mascot

Rank	Protein Name	Accession No	Protein MW	Protein PI	Pep. Count	Protein Score	Protein Score C. I. %	Total Ion Score	Total Ion C. I. %
3	Ribulose bisphosphate carboxylase large chain OS=Hyophorbe lagenicaulis GN=rbcL PE=3 SV=1	RBL_HYOLA	52928.5	6.13	8	326	100	295	100

Peptide Information

Calc. Mass	Obsrv. Mass	± da	± ppm	Start Seq.	End Seq.	Sequence	Ion Score	C. I. %	Modification	Rank	Result Type
914.4049	914.2933	-0.1116	-122	306	312	NHGMHFR			Oxidation (M)[4]		Mascot
921.4346	921.3905	-0.0441	-48	1	8	MSPQTETK					Mascot
996.4672	996.4753	0.0081	8	467	474	FEFDPVDK					Mascot
1021.5312	1021.4114	-0.1198	-117	33	41	DTDILAAFR					Mascot
1021.5312	1021.4114	-0.1198	-117	33	41	DTDILAAFR	70	99.999			Mascot
1168.6255	1168.4894	-0.1361	-116	296	305	AMHAVIDRQK					Mascot
1393.6522	1393.4844	-0.1678	-120	22	32	LTYYTPDYETK					Mascot
1393.6522	1393.4844	-0.1678	-120	22	32	LTYYTPDYETK	62	99.993			Mascot
1475.7351	1475.5814	-0.1537	-104	216	227	DRFLFCAEAIYK					Mascot
1475.7351	1475.5814	-0.1537	-104	216	227	DRFLFCAEAIYK					Mascot
3854.8718	3854.3855	-0.4863	-126	42	79	VTPQPGVPPEEAGAAVAAESSTGTWTTVWTDGLTSLDR	163	100			Mascot
3854.8718	3854.3855	-0.4863	-126	42	79	VTPQPGVPPEEAGAAVAAESSTGTWTTVWTDGLTSLDR					Mascot

Rank	Protein Name	Accession No	Protein MW	Protein PI	Pep. Count	Protein Score	Protein Score C. I. %	Total Ion Score	Total Ion C. I. %
4	Ribulose bisphosphate carboxylase large chain OS=Barnadesia caryophylla GN=rbcL PE=3 SV=1	RBL_BARCA	52672.5	6.29	8	326	100	295	100

Peptide Information

Calc. Mass	Obsrv. Mass	± da	± ppm	Start Seq.	End Seq.	Sequence	Ion Score	C. I. %	Modification	Rank	Result Type
914.4049	914.2933	-0.1116	-122	306	312	NHGMHFR			Oxidation (M)[4]		Mascot
921.4346	921.3905	-0.0441	-48	1	8	MSPQTETK					Mascot
1021.5312	1021.4114	-0.1198	-117	33	41	DTDILAAFR					Mascot
1021.5312	1021.4114	-0.1198	-117	33	41	DTDILAAFR	70	99.999			Mascot
1168.6255	1168.4894	-0.1361	-116	296	305	AMHAVIDRQK					Mascot
1275.7307	1275.5737	-0.157	-123	340	350	EITLGFVDLLR					Mascot
1393.6522	1393.4844	-0.1678	-120	22	32	LTYYTPDYETK					Mascot
1393.6522	1393.4844	-0.1678	-120	22	32	LTYYTPDYETK	62	99.993			Mascot
1475.7351	1475.5814	-0.1537	-104	216	227	DRFLFCAEAIYK					Mascot
1475.7351	1475.5814	-0.1537	-104	216	227	DRFLFCAEAIYK					Mascot
3854.8718	3854.3855	-0.4863	-126	42	79	VTPQPGVPPEEAGAAVAAESSTGTWTTVWTDGLTSLDR	163	100			Mascot
3854.8718	3854.3855	-0.4863	-126	42	79	VTPQPGVPPEEAGAAVAAESSTGTWTTVWTDGLTSLDR					Mascot

Rank	Protein Name	Accession No	Protein MW	Protein PI	Pep. Count	Protein Score	Protein Score C. I. %	Total Ion Score	Total Ion C. I. %
5	Ribulose bisphosphate carboxylase large chain OS=Gerbera jamesonii GN=rbcL PE=3 SV=1	RBL_GERJA	52895.7	6.29	8	326	100	295	100

Peptide Information

Calc. Mass	Obsrv. Mass	± da	± ppm	Start Seq.	End Seq.	Sequence	Ion Score	C. I. %	Modification	Rank	Result Type
914.4049	914.2933	-0.1116	-122	306	312	NHGMHFR			Oxidation (M)[4]		Mascot
921.4346	921.3905	-0.0441	-48	1	8	MSPQTETK					Mascot
1021.5312	1021.4114	-0.1198	-117	33	41	DTDILAAFR					Mascot
1021.5312	1021.4114	-0.1198	-117	33	41	DTDILAAFR	70	99.999			Mascot
1168.6255	1168.4894	-0.1361	-116	296	305	AMHAVIDRQK					Mascot
1275.6442	1275.5737	-0.0705	-55	218	227	FLFCTEALFK			Carbamidomethyl (C)[4]		Mascot
1393.6522	1393.4844	-0.1678	-120	22	32	LTYYTPDYETK					Mascot
1393.6522	1393.4844	-0.1678	-120	22	32	LTYYTPDYETK	62	99.993			Mascot
1837.9337	1837.7479	-0.1858	-101	317	334	ALRMSGGDHIHSGTVVGK			Oxidation (M)[4]		Mascot
3854.8718	3854.3855	-0.4863	-126	42	79	VTPQPGVPPEEAGAAVAAESSTGTWTTVWTDGLTSLDR	163	100			Mascot
3854.8718	3854.3855	-0.4863	-126	42	79	VTPQPGVPPEEAGAAVAAESSTGTWTTVWTDGLTSLDR					Mascot

Rank	Protein Name	Accession No	Protein MW	Protein PI	Pep. Count	Protein Score	Protein Score C. I. %	Total Ion Score	Total Ion C. I. %
6	Ribulose bisphosphate carboxylase large chain (Fragment) OS=Sanguinaria canadensis GN=rbcL PE=3 SV=	RBL_SANCA	48270.4	6.46	7	322	100	295	100

Peptide Information

Calc. Mass	Obsrv. Mass	± da	± ppm	Start Seq.	End Seq.	Sequence	Ion Score	C. I. %	Modification	Rank	Result Type
914.4049	914.2933	-0.1116	-122	296	302	NHGMHFR			Oxidation (M)[4]		Mascot
1021.5312	1021.4114	-0.1198	-117	23	31	DTDILAAFR					Mascot
1021.5312	1021.4114	-0.1198	-117	23	31	DTDILAAFR	70	99.999			Mascot
1168.6255	1168.4894	-0.1361	-116	286	295	AMHAVIDRQK					Mascot
1275.7307	1275.5737	-0.157	-123	330	340	EITLGFVDLLR					Mascot
1393.6522	1393.4844	-0.1678	-120	12	22	LTYYTPDYETK					Mascot
1393.6522	1393.4844	-0.1678	-120	12	22	LTYYTPDYETK	62	99.993			Mascot
1475.7351	1475.5814	-0.1537	-104	206	217	DRFLFCAEAIYK					Mascot
1475.7351	1475.5814	-0.1537	-104	206	217	DRFLFCAEAIYK					Mascot
3854.8718	3854.3855	-0.4863	-126	32	69	VTPQPGVPPEEAGAAVAAESSTGTWTTVWTDGLTSLDR	163	100			Mascot
3854.8718	3854.3855	-0.4863	-126	32	69	VTPQPGVPPEEAGAAVAAESSTGTWTTVWTDGLTSLDR					Mascot

Rank	Protein Name	Accession No	Protein MW	Protein PI	Pep. Count	Protein Score	Protein Score C. I. %	Total Ion Score	Total Ion C. I. %
7	Ribulose bisphosphate carboxylase large chain (Fragment) OS=Salacia pallescens GN=rbcL PE=3 SV=2	RBL_SALPL	49365.3	6.25	7	322	100	295	100

Peptide Information

Calc. Mass	Obsrv. Mass	± da	± ppm	Start Seq.	End Seq.	Sequence	Ion Score	C. I. %	Modification	Rank	Result Type
914.4049	914.2933	-0.1116	-122	306	312	NHGMHFR			Oxidation (M)[4]		Mascot
921.4346	921.3905	-0.0441	-48	1	8	MSPQTETK					Mascot
1021.5312	1021.4114	-0.1198	-117	33	41	DTDILAAFR					Mascot
1021.5312	1021.4114	-0.1198	-117	33	41	DTDILAAFR	70	99.999			Mascot
1045.5459	1045.4387	-0.1072	-103	422	431	VAVEACVQAR					Mascot
1393.6522	1393.4844	-0.1678	-120	22	32	LTYYTPDYETK					Mascot
1393.6522	1393.4844	-0.1678	-120	22	32	LTYYTPDYETK	62	99.993			Mascot
1475.7351	1475.5814	-0.1537	-104	216	227	DRFLFCAEALYK					Mascot
1475.7351	1475.5814	-0.1537	-104	216	227	DRFLFCAEALYK					Mascot
3854.8718	3854.3855	-0.4863	-126	42	79	VTPQPGVPPEEAGAAVAAESSTGTWTTVWTDGLTSLDR	163	100			Mascot
3854.8718	3854.3855	-0.4863	-126	42	79	VTPQPGVPPEEAGAAVAAESSTGTWTTVWTDGLTSLDR					Mascot

Rank	Protein Name	Accession No	Protein MW	Protein PI	Pep. Count	Protein Score	Protein Score C. I. %	Total Ion Score	Total Ion C. I. %
8	Ribulose bisphosphate carboxylase large chain (Fragment) OS=Lupinus albus GN=rbcL PE=3 SV=1	RBL_LUPAL	50283.4	6.22	7	321	100	295	100

Peptide Information

Calc. Mass	Obsrv. Mass	± da	± ppm	Start Seq.	End Seq.	Sequence	Ion Score	C. I. %	Modification	Rank	Result Type
914.4049	914.2933	-0.1116	-122	297	303	NHGMHFR			Oxidation (M)[4]		Mascot
1021.5312	1021.4114	-0.1198	-117	24	32	DTDILAAFR					Mascot
1021.5312	1021.4114	-0.1198	-117	24	32	DTDILAAFR	70	99.999			Mascot
1168.6255	1168.4894	-0.1361	-116	287	296	AMHAVIDRQK					Mascot
1275.7307	1275.5737	-0.157	-123	331	341	EITLGFVDLLR					Mascot
1393.6522	1393.4844	-0.1678	-120	13	23	LTYYTPDYETK					Mascot
1393.6522	1393.4844	-0.1678	-120	13	23	LTYYTPDYETK	62	99.993			Mascot
1475.7351	1475.5814	-0.1537	-104	207	218	DRFLFCAEALYK					Mascot
1475.7351	1475.5814	-0.1537	-104	207	218	DRFLFCAEALYK					Mascot
3854.8718	3854.3855	-0.4863	-126	33	70	VTPQPGVPPEEAGAAVAAESSTGTWTTVWTDGLTSLDR	163	100			Mascot
3854.8718	3854.3855	-0.4863	-126	33	70	VTPQPGVPPEEAGAAVAAESSTGTWTTVWTDGLTSLDR					Mascot

Rank	Protein Name	Accession No	Protein MW	Protein PI	Pep. Count	Protein Score	Protein Score C. I. %	Total Ion Score	Total Ion C. I. %
9	Ribulose bisphosphate carboxylase large chain (Fragment) OS=Serenoa repens GN=rbcL PE=3 SV=1	RBL_SERRE	51794	6.33	7	321	100	295	100

Peptide Information

Calc. Mass	Obsrv. Mass	± da	± ppm	Start Seq.	End Seq.	Sequence	Ion Score	C. I. %	Modification	Rank	Result Type
914.4049	914.2933	-0.1116	-122	306	312	NHGMHFR			Oxidation (M)[4]		Mascot
921.4346	921.3905	-0.0441	-48	1	8	MSPQTETK					Mascot
1021.5312	1021.4114	-0.1198	-117	33	41	DTDILAAFR					Mascot
1021.5312	1021.4114	-0.1198	-117	33	41	DTDILAAFR	70	99.999			Mascot
1168.6255	1168.4894	-0.1361	-116	296	305	AMHAVIDRQK					Mascot
1393.6522	1393.4844	-0.1678	-120	22	32	LTYYTPDYETK					Mascot
1393.6522	1393.4844	-0.1678	-120	22	32	LTYYTPDYETK	62	99.993			Mascot
1475.7351	1475.5814	-0.1537	-104	216	227	DRFLFCAEAIYK					Mascot
1475.7351	1475.5814	-0.1537	-104	216	227	DRFLFCAEAIYK					Mascot
3854.8718	3854.3855	-0.4863	-126	42	79	VTPQPGVPPEEAGAAVAAESSTGTWTTVWTDGLTSLDR	163	100			Mascot
3854.8718	3854.3855	-0.4863	-126	42	79	VTPQPGVPPEEAGAAVAAESSTGTWTTVWTDGLTSLDR					Mascot

Rank	Protein Name	Accession No	Protein MW	Protein PI	Pep. Count	Protein Score	Protein Score C. I. %	Total Ion Score	Total Ion C. I. %
10	Ribulose bisphosphate carboxylase large chain (Fragment) OS=Manilkara zapota GN=rbcL PE=3 SV=2	RBL_MANZA	51550	6.04	7	321	100	295	100

Peptide Information

Calc. Mass	Obsrv. Mass	± da	± ppm	Start Seq.	End Seq.	Sequence	Ion Score	C. I. %	Modification	Rank	Result Type
914.4049	914.2933	-0.1116	-122	296	302	NHGMHFR			Oxidation (M)[4]		Mascot
1021.5312	1021.4114	-0.1198	-117	23	31	DTDILAAFR					Mascot
1021.5312	1021.4114	-0.1198	-117	23	31	DTDILAAFR	70	99.999			Mascot
1039.4691	1039.3998	-0.0693	-67	341	348	DDYVEKDR					Mascot
1168.6255	1168.4894	-0.1361	-116	286	295	AMHAVIDRQK					Mascot
1275.7307	1275.5737	-0.157	-123	330	340	EITLGFVDLLR					Mascot
1393.6522	1393.4844	-0.1678	-120	12	22	LTYYTPDYETK					Mascot
1393.6522	1393.4844	-0.1678	-120	12	22	LTYYTPDYETK	62	99.993			Mascot
3854.8718	3854.3855	-0.4863	-126	32	69	VTPQPGVPPEEAGAAVAAESSTGTWTTVWTDGLTSLDR	163	100			Mascot
3854.8718	3854.3855	-0.4863	-126	32	69	VTPQPGVPPEEAGAAVAAESSTGTWTTVWTDGLTSLDR					Mascot

Gel Idx/Pos	113/E12	Instr./Gel Origin	BA2060/131017A	Process Sta	Analysis Succeeded
Plate [#] Name	[1] 31496	Instrument Sample Name		Spectra	8

Rank	Protein Name	Accession No	Protein MW	Protein PI	Pep. Count	Protein Score	Protein Score C. I. %	Total Ion Score	Total Ion C. I. %
1	Ribulose bisphosphate carboxylase large chain OS=Glycine max GN=rbcL PE=1 SV=3	RBL_SOYBN	52576.4	6	11	383	100	330	100

Peptide Information

Calc. Mass	Obsrv. Mass	± da	± ppm	Start Seq.	End Seq.	Sequence	Ion Score	C. I. %	Modification	Rank	Result Type
830.4366	830.3445	-0.0921	-111	440	446	EGNEIIR					Mascot
830.4366	830.3445	-0.0921	-111	440	446	EGNEIIR	30	89.768			Mascot
914.4049	914.3002	-0.1047	-115	306	312	NHGMHFR			Oxidation (M)[4]		Mascot
914.4049	914.3002	-0.1047	-115	306	312	NHGMHFR			Oxidation (M)[4]		Mascot
921.4346	921.4008	-0.0338	-37	1	8	MSPQTETK					Mascot
1023.4741	1023.3631	-0.111	-108	351	358	DDFVEKDR					Mascot
1116.583	1116.4614	-0.1216	-109	422	431	VALEACVQAR	46	99.728	Carbamidomethyl (C)[6]		Mascot
1116.583	1116.4614	-0.1216	-109	422	431	VALEACVQAR			Carbamidomethyl (C)[6]		Mascot
1275.7307	1275.5891	-0.1416	-111	340	350	EITLGFVDLLR	100	100			Mascot
1275.7307	1275.5891	-0.1416	-111	340	350	EITLGFVDLLR					Mascot
1433.7495	1433.5809	-0.1686	-118	320	334	LSGGDHVHAGTVVGK					Mascot
1472.6978	1472.5215	-0.1763	-120	464	475	EIKFEFEAMDTL					Mascot
1488.6926	1488.522	-0.1706	-115	464	475	EIKFEFEAMDTL			Oxidation (M)[9]		Mascot
1488.6926	1488.522	-0.1706	-115	464	475	EIKFEFEAMDTL	34	96.276	Oxidation (M)[9]		Mascot
1516.7616	1516.5952	-0.1664	-110	216	227	DRFLFCAEAIFK			Carbamidomethyl (C)[6]		Mascot
1546.7358	1546.5579	-0.1779	-115	451	463	WSPELAAACEVWK	85	100	Carbamidomethyl (C)[9]		Mascot
1546.7358	1546.5579	-0.1779	-115	451	463	WSPELAAACEVWK			Carbamidomethyl (C)[9]		Mascot
2009.059	2008.8201	-0.2389	-119	340	356	EITLGFVDLLRDDFVEK	35	96.994			Mascot
2009.059	2008.8201	-0.2389	-119	340	356	EITLGFVDLLRDDFVEK					Mascot

Rank	Protein Name	Accession No	Protein MW	Protein PI	Pep. Count	Protein Score	Protein Score C. I. %	Total Ion Score	Total Ion C. I. %
2	Ribulose bisphosphate carboxylase large chain OS=Cucumis sativus GN=rbcL PE=1 SV=5	RBL_CUCSA	52608.4	6	10	375	100	330	100

Peptide Information

Calc. Mass	Obsrv. Mass	± da	± ppm	Start Seq.	End Seq.	Sequence	Ion Score	C. I. %	Modification	Rank	Result Type
830.4366	830.3445	-0.0921	-111	440	446	EGNEIIR					Mascot
830.4366	830.3445	-0.0921	-111	440	446	EGNEIIR	30	89.768			Mascot
914.4049	914.3002	-0.1047	-115	306	312	NHGMHFR			Oxidation (M)[4]		Mascot
914.4049	914.3002	-0.1047	-115	306	312	NHGMHFR			Oxidation (M)[4]		Mascot
921.4346	921.4008	-0.0338	-37	1	8	MSPQTETK					Mascot
1023.4741	1023.3631	-0.111	-108	351	358	DDFVEKDR					Mascot
1116.583	1116.4614	-0.1216	-109	422	431	VALEACVQAR	46	99.728	Carbamidomethyl (C)[6]		Mascot
1116.583	1116.4614	-0.1216	-109	422	431	VALEACVQAR			Carbamidomethyl (C)[6]		Mascot
1275.7307	1275.5891	-0.1416	-111	340	350	EITLGFVDLLR	100	100			Mascot
1275.7307	1275.5891	-0.1416	-111	340	350	EITLGFVDLLR					Mascot
1472.6978	1472.5215	-0.1763	-120	464	475	EIKFEFEAMDTL					Mascot
1488.6926	1488.522	-0.1706	-115	464	475	EIKFEFEAMDTL			Oxidation (M)[9]		Mascot
1488.6926	1488.522	-0.1706	-115	464	475	EIKFEFEAMDTL	34	96.276	Oxidation (M)[9]		Mascot
1516.7616	1516.5952	-0.1664	-110	216	227	DRFLFCAEAIFK			Carbamidomethyl (C)[6]		Mascot
1546.7358	1546.5579	-0.1779	-115	451	463	WSPELAAACEVWK	85	100	Carbamidomethyl (C)[9]		Mascot
1546.7358	1546.5579	-0.1779	-115	451	463	WSPELAAACEVWK			Carbamidomethyl (C)[9]		Mascot
2009.059	2008.8201	-0.2389	-119	340	356	EITLGFVDLLRDDFVEK	35	96.994			Mascot
2009.059	2008.8201	-0.2389	-119	340	356	EITLGFVDLLRDDFVEK					Mascot

Rank	Protein Name	Accession No	Protein MW	Protein PI	Pep. Count	Protein Score	Protein Score C. I. %	Total Ion Score	Total Ion C. I. %
3	Ribulose bisphosphate carboxylase large chain (Fragment) OS=Cornus florida GN=rbcL PE=3 SV=2	RBL_CORFO	51593.1	6.14	7	355	100	330	100

Peptide Information

Calc. Mass	Obsrv. Mass	± da	± ppm	Start Seq.	End Seq.	Sequence	Ion Score	C. I. %	Modification	Rank	Result Type
830.4366	830.3445	-0.0921	-111	430	436	EGNEIIR					Mascot
830.4366	830.3445	-0.0921	-111	430	436	EGNEIIR	30	89.768			Mascot
1023.4741	1023.3631	-0.111	-108	341	348	DDFVEKDR					Mascot
1116.583	1116.4614	-0.1216	-109	412	421	VALEACVQAR	46	99.728	Carbamidomethyl (C)[6]		Mascot
1116.583	1116.4614	-0.1216	-109	412	421	VALEACVQAR			Carbamidomethyl (C)[6]		Mascot
1275.7307	1275.5891	-0.1416	-111	330	340	EITLGFVDLLR	100	100			Mascot
1275.7307	1275.5891	-0.1416	-111	330	340	EITLGFVDLLR					Mascot
1472.6978	1472.5215	-0.1763	-120	454	465	EIKFEFEAMDTL					Mascot
1488.6926	1488.522	-0.1706	-115	454	465	EIKFEFEAMDTL			Oxidation (M)[9]		Mascot
1488.6926	1488.522	-0.1706	-115	454	465	EIKFEFEAMDTL	34	96.276	Oxidation (M)[9]		Mascot
1546.7358	1546.5579	-0.1779	-115	441	453	WSPELAAACEVWK	85	100	Carbamidomethyl (C)[9]		Mascot
1546.7358	1546.5579	-0.1779	-115	441	453	WSPELAAACEVWK			Carbamidomethyl (C)[9]		Mascot
2009.059	2008.8201	-0.2389	-119	330	346	EITLGFVDLLRDDFVEK	35	96.994			Mascot
2009.059	2008.8201	-0.2389	-119	330	346	EITLGFVDLLRDDFVEK					Mascot

Rank	Protein Name	Accession No	Protein MW	Protein PI	Pep. Count	Protein Score	Protein Score C. I. %	Total Ion Score	Total Ion C. I. %
4	Ribulose bisphosphate carboxylase large chain OS=Cajanus cajan GN=rbcL PE=3 SV=1	RBL_CAJCA	52358.5	6.45	10	341	100	295	100

Peptide Information

Calc. Mass	Obsrv. Mass	± da	± ppm	Start Seq.	End Seq.	Sequence	Ion Score	C. I. %	Modification	Rank	Result Type
830.4366	830.3445	-0.0921	-111	440	446	EGNEIIR					Mascot
830.4366	830.3445	-0.0921	-111	440	446	EGNEIIR	30	89.768			Mascot
856.3546	856.4332	0.0786	92	467	473	FEFPAMD					Mascot
914.4049	914.3002	-0.1047	-115	306	312	NHGMHFR			Oxidation (M)[4]		Mascot
914.4049	914.3002	-0.1047	-115	306	312	NHGMHFR			Oxidation (M)[4]		Mascot
921.4346	921.4008	-0.0338	-37	1	8	MSPQTETK					Mascot
1023.4741	1023.3631	-0.111	-108	351	358	DDFVEKDR					Mascot
1116.583	1116.4614	-0.1216	-109	422	431	VALEACVQAR	46	99.728	Carbamidomethyl (C)[6]		Mascot
1116.583	1116.4614	-0.1216	-109	422	431	VALEACVQAR			Carbamidomethyl (C)[6]		Mascot
1275.7307	1275.5891	-0.1416	-111	340	350	EITLGFVDLLR	100	100			Mascot
1275.7307	1275.5891	-0.1416	-111	340	350	EITLGFVDLLR					Mascot
1516.7616	1516.5952	-0.1664	-110	216	227	DRFLFCAEALFK			Carbamidomethyl (C)[6]		Mascot
1546.7358	1546.5579	-0.1779	-115	451	463	WSPELAAACEVWK	85	100	Carbamidomethyl (C)[9]		Mascot
1546.7358	1546.5579	-0.1779	-115	451	463	WSPELAAACEVWK			Carbamidomethyl (C)[9]		Mascot
2009.059	2008.8201	-0.2389	-119	340	356	EITLGFVDLLRDDFVEK	35	96.994			Mascot
2009.059	2008.8201	-0.2389	-119	340	356	EITLGFVDLLRDDFVEK					Mascot

Rank	Protein Name	Accession No	Protein MW	Protein PI	Pep. Count	Protein Score	Protein Score C. I. %	Total Ion Score	Total Ion C. I. %
5	Ribulose bisphosphate carboxylase large chain OS=Vitis vinifera GN=rbcL PE=3 SV=2	RBL_VITVI	52485.5	6.33	9	333	100	295	100

Peptide Information

Calc. Mass	Obsrv. Mass	± da	± ppm	Start Seq.	End Seq.	Sequence	Ion Score	C. I. %	Modification	Rank	Result Type
830.4366	830.3445	-0.0921	-111	440	446	EGNEIIR					Mascot
830.4366	830.3445	-0.0921	-111	440	446	EGNEIIR	30	89.768			Mascot
914.4049	914.3002	-0.1047	-115	306	312	NHGMHFR			Oxidation (M)[4]		Mascot
914.4049	914.3002	-0.1047	-115	306	312	NHGMHFR			Oxidation (M)[4]		Mascot
921.4346	921.4008	-0.0338	-37	1	8	MSPQTETK					Mascot
1023.4741	1023.3631	-0.111	-108	351	358	DDFVEKDR					Mascot
1116.583	1116.4614	-0.1216	-109	422	431	VALEACVQAR	46	99.728	Carbamidomethyl (C)[6]		Mascot
1116.583	1116.4614	-0.1216	-109	422	431	VALEACVQAR			Carbamidomethyl (C)[6]		Mascot
1275.7307	1275.5891	-0.1416	-111	340	350	EITLGFVDLLR	100	100			Mascot
1275.7307	1275.5891	-0.1416	-111	340	350	EITLGFVDLLR					Mascot
1516.7616	1516.5952	-0.1664	-110	216	227	DRFLFCAEAIFK			Carbamidomethyl (C)[6]		Mascot
1546.7358	1546.5579	-0.1779	-115	451	463	WSPELAAACEVWK	85	100	Carbamidomethyl (C)[9]		Mascot
1546.7358	1546.5579	-0.1779	-115	451	463	WSPELAAACEVWK			Carbamidomethyl (C)[9]		Mascot
2009.059	2008.8201	-0.2389	-119	340	356	EITLGFVDLLRDDFVEK	35	96.994			Mascot
2009.059	2008.8201	-0.2389	-119	340	356	EITLGFVDLLRDDFVEK					Mascot

Rank	Protein Name	Accession No	Protein MW	Protein PI	Pep. Count	Protein Score	Protein Score C. I. %	Total Ion Score	Total Ion C. I. %
6	Ribulose bisphosphate carboxylase large chain (Fragment) OS=Moringa oleifera GN=rbcL PE=3 SV=1	RBL_MOROL	51552.1	6.32	8	327	100	295	100

Peptide Information

Calc. Mass	Obsrv. Mass	± da	± ppm	Start Seq.	End Seq.	Sequence	Ion Score	C. I. %	Modification	Rank	Result Type
830.4366	830.3445	-0.0921	-111	431	437	EGNEIIR					Mascot
830.4366	830.3445	-0.0921	-111	431	437	EGNEIIR	30	89.768			Mascot
914.4049	914.3002	-0.1047	-115	297	303	NHGMHFR			Oxidation (M)[4]		Mascot
914.4049	914.3002	-0.1047	-115	297	303	NHGMHFR			Oxidation (M)[4]		Mascot
1023.4741	1023.3631	-0.111	-108	342	349	DDFVEKDR					Mascot
1038.5143	1038.4189	-0.0954	-92	458	466	FEFPAVDTL					Mascot
1116.583	1116.4614	-0.1216	-109	413	422	VALEACVQAR	46	99.728	Carbamidomethyl (C)[6]		Mascot
1116.583	1116.4614	-0.1216	-109	413	422	VALEACVQAR			Carbamidomethyl (C)[6]		Mascot
1275.7307	1275.5891	-0.1416	-111	331	341	EITLGFVDLLR	100	100			Mascot
1275.7307	1275.5891	-0.1416	-111	331	341	EITLGFVDLLR					Mascot
1546.7358	1546.5579	-0.1779	-115	442	454	WSPELAAACEVWK	85	100	Carbamidomethyl (C)[9]		Mascot
1546.7358	1546.5579	-0.1779	-115	442	454	WSPELAAACEVWK			Carbamidomethyl (C)[9]		Mascot
2009.059	2008.8201	-0.2389	-119	331	347	EITLGFVDLLRDDFVEK	35	96.994			Mascot
2009.059	2008.8201	-0.2389	-119	331	347	EITLGFVDLLRDDFVEK					Mascot

Rank	Protein Name	Accession No	Protein MW	Protein PI	Pep. Count	Protein Score	Protein Score C. I. %	Total Ion Score	Total Ion C. I. %
7	Ribulose bisphosphate carboxylase large chain (Fragment) OS=Manilkara zapota GN=rbcL PE=3 SV=2	RBL_MANZA	51550	6.04	8	326	100	294	100

Peptide Information

Calc. Mass	Obsrv. Mass	± da	± ppm	Start Seq.	End Seq.	Sequence	Ion Score	C. I. %	Modification	Rank	Result Type
830.4366	830.3445	-0.0921	-111	430	436	EGNEIIR					Mascot
830.4366	830.3445	-0.0921	-111	430	436	EGNEIIR	30	89.768			Mascot
914.4049	914.3002	-0.1047	-115	296	302	NHGMHFR			Oxidation (M)[4]		Mascot
914.4049	914.3002	-0.1047	-115	296	302	NHGMHFR			Oxidation (M)[4]		Mascot
1039.4691	1039.4093	-0.0598	-58	341	348	DDYVEKDR					Mascot
1116.583	1116.4614	-0.1216	-109	412	421	VALEACVQAR	46	99.728	Carbamidomethyl (C)[6]		Mascot
1116.583	1116.4614	-0.1216	-109	412	421	VALEACVQAR			Carbamidomethyl (C)[6]		Mascot
1275.7307	1275.5891	-0.1416	-111	330	340	EITLGFVDLLR	100	100			Mascot
1275.7307	1275.5891	-0.1416	-111	330	340	EITLGFVDLLR					Mascot
1433.7495	1433.5809	-0.1686	-118	310	324	LSGGDHVHAGTVVGK					Mascot
1472.6978	1472.5215	-0.1763	-120	454	465	EIKFEFEAMDTL					Mascot
1488.6926	1488.522	-0.1706	-115	454	465	EIKFEFEAMDTL			Oxidation (M)[9]		Mascot
1488.6926	1488.522	-0.1706	-115	454	465	EIKFEFEAMDTL	34	96.276	Oxidation (M)[9]		Mascot
1546.7358	1546.5579	-0.1779	-115	441	453	WSPELAAACEVWK	85	100	Carbamidomethyl (C)[9]		Mascot
1546.7358	1546.5579	-0.1779	-115	441	453	WSPELAAACEVWK			Carbamidomethyl (C)[9]		Mascot

Rank	Protein Name	Accession No	Protein MW	Protein PI	Pep. Count	Protein Score	Protein Score C. I. %	Total Ion Score	Total Ion C. I. %
8	Ribulose bisphosphate carboxylase large chain (Fragment) OS=Ilex crenata GN=rbcL PE=3 SV=1	RBL_ILECR	51518	6.1	8	326	100	295	100

Peptide Information

Calc. Mass	Obsrv. Mass	± da	± ppm	Start Seq.	End Seq.	Sequence	Ion Score	C. I. %	Modification	Rank	Result Type
830.4366	830.3445	-0.0921	-111	430	436	EGNEIIR					Mascot
830.4366	830.3445	-0.0921	-111	430	436	EGNEIIR	30	89.768			Mascot
914.4049	914.3002	-0.1047	-115	296	302	NHGMHFR			Oxidation (M)[4]		Mascot
914.4049	914.3002	-0.1047	-115	296	302	NHGMHFR			Oxidation (M)[4]		Mascot
1023.4741	1023.3631	-0.111	-108	341	348	DDFVEKDR					Mascot
1116.6194	1116.4614	-0.158	-141	412	421	VALEACVKAR	46	99.728	Carbamidomethyl (C)[6]		Mascot
1116.6194	1116.4614	-0.158	-141	412	421	VALEACVKAR			Carbamidomethyl (C)[6]		Mascot
1275.7307	1275.5891	-0.1416	-111	330	340	EITLGFVDLLR	100	100			Mascot
1275.7307	1275.5891	-0.1416	-111	330	340	EITLGFVDLLR					Mascot
1516.7616	1516.5952	-0.1664	-110	206	217	DRFLFCAEALFK			Carbamidomethyl (C)[6]		Mascot
1546.7358	1546.5579	-0.1779	-115	441	453	WSPELAAACEVWK	85	100	Carbamidomethyl (C)[9]		Mascot
1546.7358	1546.5579	-0.1779	-115	441	453	WSPELAAACEVWK			Carbamidomethyl (C)[9]		Mascot
2009.059	2008.8201	-0.2389	-119	330	346	EITLGFVDLLRDDFVEK	35	96.994			Mascot
2009.059	2008.8201	-0.2389	-119	330	346	EITLGFVDLLRDDFVEK					Mascot

Rank	Protein Name	Accession No	Protein MW	Protein PI	Pep. Count	Protein Score	Protein Score C. I. %	Total Ion Score	Total Ion C. I. %
9	Ribulose bisphosphate carboxylase large chain (Fragment) OS=Vitis aestivalis GN=rbcL PE=3 SV=1	RBL_VITAE	51567	6.29	8	326	100	295	100

Peptide Information

Calc. Mass	Obsrv. Mass	± da	± ppm	Start Seq.	End Seq.	Sequence	Ion Score	C. I. %	Modification	Rank	Result Type
830.4366	830.3445	-0.0921	-111	431	437	EGNEIIR					Mascot
830.4366	830.3445	-0.0921	-111	431	437	EGNEIIR	30	89.768			Mascot
914.4049	914.3002	-0.1047	-115	297	303	NHGMHFR			Oxidation (M)[4]		Mascot
914.4049	914.3002	-0.1047	-115	297	303	NHGMHFR			Oxidation (M)[4]		Mascot
1023.4741	1023.3631	-0.111	-108	342	349	DDFVEKDR					Mascot
1116.583	1116.4614	-0.1216	-109	413	422	VALEACVQAR	46	99.728	Carbamidomethyl (C)[6]		Mascot
1116.583	1116.4614	-0.1216	-109	413	422	VALEACVQAR			Carbamidomethyl (C)[6]		Mascot
1275.7307	1275.5891	-0.1416	-111	331	341	EITLGFVDLLR	100	100			Mascot
1275.7307	1275.5891	-0.1416	-111	331	341	EITLGFVDLLR					Mascot
1516.7616	1516.5952	-0.1664	-110	207	218	DRFLFCAEAIFK			Carbamidomethyl (C)[6]		Mascot
1546.7358	1546.5579	-0.1779	-115	442	454	WSPELAAACEVWK	85	100	Carbamidomethyl (C)[9]		Mascot
1546.7358	1546.5579	-0.1779	-115	442	454	WSPELAAACEVWK			Carbamidomethyl (C)[9]		Mascot
2009.059	2008.8201	-0.2389	-119	331	347	EITLGFVDLLRDDFVEK	35	96.994			Mascot
2009.059	2008.8201	-0.2389	-119	331	347	EITLGFVDLLRDDFVEK					Mascot

Rank	Protein Name	Accession No	Protein MW	Protein PI	Pep. Count	Protein Score	Protein Score C. I. %	Total Ion Score	Total Ion C. I. %
10	Ribulose bisphosphate carboxylase large chain OS=Calycanthus floridus var. glaucus GN=rbcL PE=3 SV=	RBL_CALFG	52571.5	6.23	8	325	100	294	100

Peptide Information

Calc. Mass	Obsrv. Mass	± da	± ppm	Start Seq.	End Seq.	Sequence	Ion Score	C. I. %	Modification	Rank	Result Type
830.4366	830.3445	-0.0921	-111	440	446	EGNEIIR					Mascot
830.4366	830.3445	-0.0921	-111	440	446	EGNEIIR	30	87.611			Mascot
914.4049	914.3002	-0.1047	-115	306	312	NHGMHFR			Oxidation (M)[4]		Mascot
914.4049	914.3002	-0.1047	-115	306	312	NHGMHFR			Oxidation (M)[4]		Mascot
921.4709	921.4008	-0.0701	-76	1	8	MSPKTETK					Mascot
1116.583	1116.4614	-0.1216	-109	422	431	VALEACVQAR	46	99.671	Carbamidomethyl (C)[6]		Mascot
1116.583	1116.4614	-0.1216	-109	422	431	VALEACVQAR			Carbamidomethyl (C)[6]		Mascot
1275.7307	1275.5891	-0.1416	-111	340	350	EITLGFVDLLR	100	100			Mascot
1275.7307	1275.5891	-0.1416	-111	340	350	EITLGFVDLLR					Mascot

1472.6978	1472.5215	-0.1763	-120	464	475	EIKFEFEAMDTL				Mascot
1488.6926	1488.522	-0.1706	-115	464	475	EIKFEFEAMDTL			Oxidation (M)[9]	Mascot
1488.6926	1488.522	-0.1706	-115	464	475	EIKFEFEAMDTL	34	95.492	Oxidation (M)[9]	Mascot
1516.7616	1516.5952	-0.1664	-110	216	227	DRFLFCAEAIFK			Carbamidomethyl (C)[6]	Mascot
1546.7358	1546.5579	-0.1779	-115	451	463	WSPELAAACEVWK	85	100	Carbamidomethyl (C)[9]	Mascot
1546.7358	1546.5579	-0.1779	-115	451	463	WSPELAAACEVWK			Carbamidomethyl (C)[9]	Mascot

Gel Idx/Pos	114/E13	Instr./Gel Origin	BA2060/131017A	Process State	Analysis Succeeded
Plate [#] Name	[1] 31496	Instrument Sample Name		Spectra	8

Rank	Protein Name	Accession No.	Protein MW	Protein PI	Pep. Count	Protein Score	Protein Score C. I. %	Total Ion Score	Total Ion C. I. %
1	Ribulose bisphosphate carboxylase large chain OS=Glycine max GN=rbcL PE=1 SV=3	RBL_SOYBN	52576.4	6	11	276	100	222	100

Peptide Information

Calc. Mass	Obsrv. Mass	± da	± ppm	Start Seq.	End Seq.	Sequence	Ion Score	C. I. %	Modification	Rank	Result Type
830.4366	830.3543	-0.0823	-99	440	446	EGNEIIR					Mascot
830.4366	830.3543	-0.0823	-99	440	446	EGNEIIR	20	0			Mascot
914.4049	914.3083	-0.0966	-106	306	312	NHGMHFR			Oxidation (M)[4]		Mascot
914.4049	914.3083	-0.0966	-106	306	312	NHGMHFR			Oxidation (M)[4]		Mascot
921.4346	921.4094	-0.0252	-27	1	8	MSPQTETK					Mascot
1023.4741	1023.3729	-0.1012	-99	351	358	DDFVEKDR					Mascot
1116.583	1116.4695	-0.1135	-102	422	431	VALEACVQAR			Carbamidomethyl (C)[6]		Mascot
1116.583	1116.4695	-0.1135	-102	422	431	VALEACVQAR	36	96.719	Carbamidomethyl (C)[6]		Mascot
1275.7307	1275.6027	-0.128	-100	340	350	EITLGFVDLLR					Mascot
1275.7307	1275.6027	-0.128	-100	340	350	EITLGFVDLLR	88	100			Mascot
1433.7495	1433.5996	-0.1499	-105	320	334	LSGGDHVHAGTVVGK					Mascot
1472.6978	1472.5408	-0.157	-107	464	475	EIKFEFEAMDTL					Mascot
1488.6926	1488.5336	-0.159	-107	464	475	EIKFEFEAMDTL			Oxidation (M)[9]		Mascot
1546.7358	1546.573	-0.1628	-105	451	463	WSPELAAACEVWK	79	100	Carbamidomethyl (C)[9]		Mascot
1546.7358	1546.573	-0.1628	-105	451	463	WSPELAAACEVWK			Carbamidomethyl (C)[9]		Mascot
2009.059	2008.8429	-0.2161	-108	340	356	EITLGFVDLLRDDFVEK					Mascot
2788.2222	2788.1345	-0.0877	-31	228	252	SQAETGEIKGHYLNATAGTCEEMMK			Carbamidomethyl (C)[20], Oxidation (M)[23,24]		Mascot

Rank	Protein Name	Accession No.	Protein MW	Protein PI	Pep. Count	Protein Score	Protein Score C. I. %	Total Ion Score	Total Ion C. I. %
2	Ribulose bisphosphate carboxylase large chain OS=Cucumis sativus GN=rbcL PE=1 SV=5	RBL_CUCSA	52608.4	6	10	268	100	222	100

Peptide Information

Calc. Mass	Obsrv. Mass	± da	± ppm	Start Seq.	End Seq.	Sequence	Ion Score	C. I. %	Modification	Rank	Result Type
830.4366	830.3543	-0.0823	-99	440	446	EGNEIIR					Mascot
830.4366	830.3543	-0.0823	-99	440	446	EGNEIIR	20	0			Mascot
914.4049	914.3083	-0.0966	-106	306	312	NHGMHFR			Oxidation (M)[4]		Mascot
914.4049	914.3083	-0.0966	-106	306	312	NHGMHFR			Oxidation (M)[4]		Mascot
921.4346	921.4094	-0.0252	-27	1	8	MSPQTETK					Mascot
1023.4741	1023.3729	-0.1012	-99	351	358	DDFVEKDR					Mascot
1116.583	1116.4695	-0.1135	-102	422	431	VALEACVQAR			Carbamidomethyl (C)[6]		Mascot
1116.583	1116.4695	-0.1135	-102	422	431	VALEACVQAR	36	96.719	Carbamidomethyl (C)[6]		Mascot
1275.7307	1275.6027	-0.128	-100	340	350	EITLGFVDLLR					Mascot
1275.7307	1275.6027	-0.128	-100	340	350	EITLGFVDLLR	88	100			Mascot
1472.6978	1472.5408	-0.157	-107	464	475	EIKFEFEAMDTL					Mascot
1488.6926	1488.5336	-0.159	-107	464	475	EIKFEFEAMDTL			Oxidation (M)[9]		Mascot
1546.7358	1546.573	-0.1628	-105	451	463	WSPELAAACEVWK	79	100	Carbamidomethyl (C)[9]		Mascot
1546.7358	1546.573	-0.1628	-105	451	463	WSPELAAACEVWK			Carbamidomethyl (C)[9]		Mascot
2009.059	2008.8429	-0.2161	-108	340	356	EITLGFVDLLRDDFVEK					Mascot
2788.2222	2788.1345	-0.0877	-31	228	252	SQAETGEIKGHYLNATAGTCEEMMK			Carbamidomethyl (C)[20], Oxidation (M)[23,24]		Mascot

Rank	Protein Name	Accession No.	Protein MW	Protein PI	Pep. Count	Protein Score	Protein Score C. I. %	Total Ion Score	Total Ion C. I. %
3	Ribulose bisphosphate carboxylase large chain (Fragment) OS=Vitis aestivalis GN=rbcL PE=3 SV=1	RBL_VITAE	51567	6.29	9	261	100	222	100

Peptide Information

Calc. Mass	Obsrv. Mass	± da	± ppm	Start Seq.	End Seq.	Sequence	Ion Score	C. I. %	Modification	Rank	Result Type
830.4366	830.3543	-0.0823	-99	431	437	EGNEIIR					Mascot
830.4366	830.3543	-0.0823	-99	431	437	EGNEIIR	20	0			Mascot
892.525	892.4012	-0.1238	-139	1	9	SVGFKAGVK					Mascot
914.4049	914.3083	-0.0966	-106	297	303	NHGMHFR			Oxidation (M)[4]		Mascot
914.4049	914.3083	-0.0966	-106	297	303	NHGMHFR			Oxidation (M)[4]		Mascot
1023.4741	1023.3729	-0.1012	-99	342	349	DDFVEKDR					Mascot
1116.583	1116.4695	-0.1135	-102	413	422	VALEACVQAR			Carbamidomethyl (C)[6]		Mascot
1116.583	1116.4695	-0.1135	-102	413	422	VALEACVQAR	36	96.719	Carbamidomethyl (C)[6]		Mascot
1275.7307	1275.6027	-0.128	-100	331	341	EITLGFVDLLR					Mascot
1275.7307	1275.6027	-0.128	-100	331	341	EITLGFVDLLR	88	100			Mascot
1546.7358	1546.573	-0.1628	-105	442	454	WSPELAAACEVWK	79	100	Carbamidomethyl (C)[9]		Mascot
1546.7358	1546.573	-0.1628	-105	442	454	WSPELAAACEVWK			Carbamidomethyl (C)[9]		Mascot
2009.059	2008.8429	-0.2161	-108	331	347	EITLGFVDLLRDDFVEK					Mascot
2788.2222	2788.1345	-0.0877	-31	219	243	SQAETGEIKGHYLNATAGTCEEMMK			Carbamidomethyl (C)[20], Oxidation (M)[23,24]		Mascot

Rank	Protein Name	Accession No.	Protein MW	Protein PI	Pep. Count	Protein Score	Protein Score C. I. %	Total Ion Score	Total Ion C. I. %
4	Ribulose bisphosphate carboxylase large chain (Fragment) OS=Moringa oleifera GN=rbcL PE=3 SV=1	RBL_MOROL	51552.1	6.32	9	260	100	222	100

Peptide Information

Calc. Mass	Obsrv. Mass	± da	± ppm	Start Seq.	End Seq.	Sequence	Ion Score	C. I. %	Modification	Rank	Result Type
830.4366	830.3543	-0.0823	-99	431	437	EGNEIIR					Mascot
830.4366	830.3543	-0.0823	-99	431	437	EGNEIIR	20	0			Mascot
892.525	892.4012	-0.1238	-139	1	9	SVGFKAGVK					Mascot
914.4049	914.3083	-0.0966	-106	297	303	NHGMHFR			Oxidation (M)[4]		Mascot
914.4049	914.3083	-0.0966	-106	297	303	NHGMHFR			Oxidation (M)[4]		Mascot
1023.4741	1023.3729	-0.1012	-99	342	349	DDFVEKDR					Mascot
1038.5143	1038.4299	-0.0844	-81	458	466	FEFPAVDTL					Mascot
1116.583	1116.4695	-0.1135	-102	413	422	VALEACVQAR			Carbamidomethyl (C)[6]		Mascot
1116.583	1116.4695	-0.1135	-102	413	422	VALEACVQAR	36	96.719	Carbamidomethyl (C)[6]		Mascot
1275.7307	1275.6027	-0.128	-100	331	341	EITLGFVDLLR					Mascot
1275.7307	1275.6027	-0.128	-100	331	341	EITLGFVDLLR	88	100			Mascot
1546.7358	1546.573	-0.1628	-105	442	454	WSPELAAACEVWK	79	100	Carbamidomethyl (C)[9]		Mascot
1546.7358	1546.573	-0.1628	-105	442	454	WSPELAAACEVWK			Carbamidomethyl (C)[9]		Mascot
2009.059	2008.8429	-0.2161	-108	331	347	EITLGFVDLLRDDFVEK					Mascot

Rank	Protein Name	Accession No.	Protein MW	Protein PI	Pep. Count	Protein Score	Protein Score C. I. %	Total Ion Score	Total Ion C. I. %
5	Ribulose bisphosphate carboxylase large chain OS=Ipomoea purpurea GN=rbcL PE=3 SV=1	RBL_IPOPU	53353.9	6.41	9	260	100	222	100

Peptide Information

Calc. Mass	Obsrv. Mass	± da	± ppm	Start Seq.	End Seq.	Sequence	Ion Score	C. I. %	Modification	Rank	Result Type
830.4366	830.3543	-0.0823	-99	440	446	EGNEIIR					Mascot
830.4366	830.3543	-0.0823	-99	440	446	EGNEIIR	20	0			Mascot
914.4049	914.3083	-0.0966	-106	306	312	NHGMHFR			Oxidation (M)[4]		Mascot
914.4049	914.3083	-0.0966	-106	306	312	NHGMHFR			Oxidation (M)[4]		Mascot
921.4346	921.4094	-0.0252	-27	1	8	MSPQTETK					Mascot
1023.4377	1023.3729	-0.0648	-63	351	358	DDFVEQDR					Mascot
1116.583	1116.4695	-0.1135	-102	422	431	VALEACVQAR			Carbamidomethyl (C)[6]		Mascot
1116.583	1116.4695	-0.1135	-102	422	431	VALEACVQAR	36	96.719	Carbamidomethyl (C)[6]		Mascot
1275.7307	1275.6027	-0.128	-100	340	350	EITLGFVDLLR					Mascot
1275.7307	1275.6027	-0.128	-100	340	350	EITLGFVDLLR	88	100			Mascot
1546.7358	1546.573	-0.1628	-105	451	463	WSPELAAACEVWK	79	100	Carbamidomethyl (C)[9]		Mascot
1546.7358	1546.573	-0.1628	-105	451	463	WSPELAAACEVWK			Carbamidomethyl (C)[9]		Mascot
2280.1506	2279.9263	-0.2243	-98	340	358	EITLGFVDLLRDDFVEQDR					Mascot
2788.2222	2788.1345	-0.0877	-31	228	252	SQAETGEIKGHYLNATAGTCEEMMK			Carbamidomethyl (C)[20], Oxidation (M)[23,24]		Mascot

Rank	Protein Name	Accession No.	Protein MW	Protein PI	Pep. Count	Protein Score	Protein Score C. I. %	Total Ion Score	Total Ion C. I. %
6	Ribulose bisphosphate carboxylase large chain OS=Cajanus cajan GN=rbcL PE=3 SV=1	RBL_CAJCA	52358.5	6.45	9	260	100	222	100

Peptide Information

Calc. Mass	Obsrv. Mass	± da	± ppm	Start Seq.	End Seq.	Sequence	Ion Score	C. I. %	Modification	Rank	Result Type
830.4366	830.3543	-0.0823	-99	440	446	EGNEIIR					Mascot
830.4366	830.3543	-0.0823	-99	440	446	EGNEIIR	20	0			Mascot
856.3546	856.4392	0.0846	99	467	473	FEFPAMD					Mascot
914.4049	914.3083	-0.0966	-106	306	312	NHGMHFR			Oxidation (M)[4]		Mascot
914.4049	914.3083	-0.0966	-106	306	312	NHGMHFR			Oxidation (M)[4]		Mascot
921.4346	921.4094	-0.0252	-27	1	8	MSPQTETK					Mascot
1023.4741	1023.3729	-0.1012	-99	351	358	DDFVEKDR					Mascot
1116.583	1116.4695	-0.1135	-102	422	431	VALEACVQAR			Carbamidomethyl (C)[6]		Mascot
1116.583	1116.4695	-0.1135	-102	422	431	VALEACVQAR	36	96.719	Carbamidomethyl (C)[6]		Mascot
1275.7307	1275.6027	-0.128	-100	340	350	EITLGFVDLLR					Mascot
1275.7307	1275.6027	-0.128	-100	340	350	EITLGFVDLLR	88	100			Mascot
1546.7358	1546.573	-0.1628	-105	451	463	WSPELAAACEVWK	79	100	Carbamidomethyl (C)[9]		Mascot
1546.7358	1546.573	-0.1628	-105	451	463	WSPELAAACEVWK			Carbamidomethyl (C)[9]		Mascot
2009.059	2008.8429	-0.2161	-108	340	356	EITLGFVDLLRDDFVEK					Mascot

Rank	Protein Name	Accession No.	Protein MW	Protein PI	Pep. Count	Protein Score	Protein Score C. I. %	Total Ion Score	Total Ion C. I. %
7	Ribulose bisphosphate carboxylase large chain (Fragment) OS=Tamarindus indica GN=rbcL PE=3 SV=1	RBL_TAMIN	50497.5	6.13	8	254	100	222	100

Peptide Information

Calc. Mass	Obsrv. Mass	± da	± ppm	Start Seq.	End Seq.	Sequence	Ion Score	C. I. %	Modification	Rank	Result Type
830.4366	830.3543	-0.0823	-99	431	437	EGNEIIR					Mascot
830.4366	830.3543	-0.0823	-99	431	437	EGNEIIR	20	0			Mascot
892.525	892.4012	-0.1238	-139	1	9	SVGFKAGVK					Mascot
914.4049	914.3083	-0.0966	-106	297	303	NHGMHFR			Oxidation (M)[4]		Mascot
914.4049	914.3083	-0.0966	-106	297	303	NHGMHFR			Oxidation (M)[4]		Mascot
1116.583	1116.4695	-0.1135	-102	413	422	VALEACVQAR			Carbamidomethyl (C)[6]		Mascot
1116.583	1116.4695	-0.1135	-102	413	422	VALEACVQAR	36	96.719	Carbamidomethyl (C)[6]		Mascot
1275.7307	1275.6027	-0.128	-100	331	341	EITLGFVDLLR					Mascot
1275.7307	1275.6027	-0.128	-100	331	341	EITLGFVDLLR	88	100			Mascot
1546.7358	1546.573	-0.1628	-105	442	454	WSPELAAACEVWK	79	100	Carbamidomethyl (C)[9]		Mascot
1546.7358	1546.573	-0.1628	-105	442	454	WSPELAAACEVWK			Carbamidomethyl (C)[9]		Mascot
1618.757	1618.6925	-0.0645	-40	442	455	WSPELAAACEVWKE					Mascot
2748.3298	2748.0088	-0.321	-117	219	243	AQAETGEIKGHYLNATAGTWEEMIK					Mascot

Rank	Protein Name	Accession No.	Protein MW	Protein PI	Pep. Count	Protein Score	Protein Score C. I. %	Total Ion Score	Total Ion C. I. %
8	Ribulose bisphosphate carboxylase large chain (Fragment) OS=Acacia farnesiana GN=rbcL PE=3 SV=1	RBL_ACAFA	50346.3	6.22	8	254	100	222	100

Peptide Information

Calc. Mass	Obsrv. Mass	± da	± ppm	Start Seq.	End Seq.	Sequence	Ion Score	C. I. %	Modification	Rank	Result Type
830.4366	830.3543	-0.0823	-99	431	437	EGNEIIR					Mascot
830.4366	830.3543	-0.0823	-99	431	437	EGNEIIR	20	0			Mascot
892.525	892.4012	-0.1238	-139	1	9	SVGFKAGVK					Mascot
914.4049	914.3083	-0.0966	-106	297	303	NHGMHFR			Oxidation (M)[4]		Mascot
914.4049	914.3083	-0.0966	-106	297	303	NHGMHFR			Oxidation (M)[4]		Mascot
1039.4691	1039.4216	-0.0475	-46	342	349	DDYVEKDR					Mascot
1116.583	1116.4695	-0.1135	-102	413	422	VALEACVQAR			Carbamidomethyl (C)[6]		Mascot
1116.583	1116.4695	-0.1135	-102	413	422	VALEACVQAR	36	96.719	Carbamidomethyl (C)[6]		Mascot
1275.7307	1275.6027	-0.128	-100	331	341	EITLGFVDLLR					Mascot
1275.7307	1275.6027	-0.128	-100	331	341	EITLGFVDLLR	88	100			Mascot
1546.7358	1546.573	-0.1628	-105	442	454	WSPELAAACEVWK	79	100	Carbamidomethyl (C)[9]		Mascot
1546.7358	1546.573	-0.1628	-105	442	454	WSPELAAACEVWK			Carbamidomethyl (C)[9]		Mascot Mascot
1618.757	1618.6925	-0.0645	-40	442	455	WSPELAAACEVWKE					Mascot

Rank	Protein Name	Accession No.	Protein MW	Protein PI	Pep. Count	Protein Score	Protein Score C. I. %	Total Ion Score	Total Ion C. I. %
9	Ribulose bisphosphate carboxylase large chain (Fragment) OS=Manilkara zapota GN=rbcL PE=3 SV=2	RBL_MANZA	51550	6.04	8	254	100	222	100

Peptide Information

Calc. Mass	Obsrv. Mass	± da	± ppm	Start Seq.	End Seq.	Sequence	Ion Score	C. I. %	Modification	Rank	Result Type
830.4366	830.3543	-0.0823	-99	430	436	EGNEIIR					Mascot
830.4366	830.3543	-0.0823	-99	430	436	EGNEIIR	20	0			Mascot
914.4049	914.3083	-0.0966	-106	296	302	NHGMHFR			Oxidation (M)[4]		Mascot
914.4049	914.3083	-0.0966	-106	296	302	NHGMHFR			Oxidation (M)[4]		Mascot
1039.4691	1039.4216	-0.0475	-46	341	348	DDYVEKDR					Mascot
1116.583	1116.4695	-0.1135	-102	412	421	VALEACVQAR			Carbamidomethyl (C)[6]		Mascot
1116.583	1116.4695	-0.1135	-102	412	421	VALEACVQAR	36	96.719	Carbamidomethyl (C)[6]		Mascot
1275.7307	1275.6027	-0.128	-100	330	340	EITLGFVDLLR					Mascot
1275.7307	1275.6027	-0.128	-100	330	340	EITLGFVDLLR	88	100			Mascot
1433.7495	1433.5996	-0.1499	-105	310	324	LSGGDHVHAGTVVGK					Mascot
1472.6978	1472.5408	-0.157	-107	454	465	EIKFEFEAMDTL					Mascot
1488.6926	1488.5336	-0.159	-107	454	465	EIKFEFEAMDTL			Oxidation (M)[9]		Mascot
1546.7358	1546.573	-0.1628	-105	441	453	WSPELAAACEVWK	79	100	Carbamidomethyl (C)[9]		Mascot
1546.7358	1546.573	-0.1628	-105	441	453	WSPELAAACEVWK			Carbamidomethyl (C)[9]		Mascot

Rank	Protein Name	Accession No.	Protein MW	Protein PI	Pep. Count	Protein Score	Protein Score C. I. %	Total Ion Score	Total Ion C. I. %
10	Ribulose bisphosphate carboxylase large chain OS=Calycanthus floridus var. glaucus GN=rbcL PE=3 SV=	RBL_CALFG	52571.5	6.23	8	253	100	222	100

Peptide Information

Calc. Mass	Obsrv. Mass	± da	± ppm	Start Seq.	End Seq.	Sequence	Ion Score	C. I. %	Modification	Rank	Result Type
830.4366	830.3543	-0.0823	-99	440	446	EGNEIIR					Mascot
830.4366	830.3543	-0.0823	-99	440	446	EGNEIIR	20	0			Mascot
914.4049	914.3083	-0.0966	-106	306	312	NHGMHFR			Oxidation (M)[4]		Mascot
914.4049	914.3083	-0.0966	-106	306	312	NHGMHFR			Oxidation (M)[4]		Mascot
921.4709	921.4094	-0.0615	-67	1	8	MSPKTETK					Mascot
933.5515	933.4205	-0.131	-140	9	18	AGVGFKAGVK					Mascot
933.5515	933.4205	-0.131	-140	9	18	AGVGFKAGVK					Mascot
1116.583	1116.4695	-0.1135	-102	422	431	VALEACVQAR			Carbamidomethyl (C)[6]		Mascot
1116.583	1116.4695	-0.1135	-102	422	431	VALEACVQAR	36	96.719	Carbamidomethyl (C)[6]		Mascot
1275.7307	1275.6027	-0.128	-100	340	350	EITLGFVDLLR					Mascot
1275.7307	1275.6027	-0.128	-100	340	350	EITLGFVDLLR	88	100			Mascot
1472.6978	1472.5408	-0.157	-107	464	475	EIKFEFEAMDTL					Mascot
1488.6926	1488.5336	-0.159	-107	464	475	EIKFEFEAMDTL			Oxidation (M)[9]		Mascot
1546.7358	1546.573	-0.1628	-105	451	463	WSPELAAACEVWK	79	100	Carbamidomethyl (C)[9]		Mascot
	1546.573										Mascot
1546.7358	1546.573	-0.1628	-105	451	463	WSPELAAACEVWK			Carbamidomethyl (C)[9]		Mascot

Gel Idx/Pos	115/E14	Instr./Gel Origin	BA2060/131017A	Process State	Analysis Succeeded
Plate [#] Name	[1] 31496	Instrument Sample Name		Spectra	8

Rank	Protein Name	Accession No.	Protein MW	Protein PI	Pep. Count	Protein Score	Protein Score C. I. %	Total Ion Score	Total Ion C. I. %
1	Ribulose bisphosphate carboxylase large chain OS=Glycine max GN=rbcL PE=1 SV=3	RBL_SOYBN	52576.4	6	10	298	100	253	100

Peptide Information

Calc. Mass	Obsrv. Mass	± da	± ppm	Start Seq.	End Seq.	Sequence	Ion Score	C. I. %	Modification	Rank	Result Type
830.4366	830.356	-0.0806	-97	440	446	EGNEIIR	16	0			Mascot
830.4366	830.356	-0.0806	-97	440	446	EGNEIIR					Mascot
914.4049	914.311	-0.0939	-103	306	312	NHGMHFR			Oxidation (M)[4]		Mascot
914.4049	914.311	-0.0939	-103	306	312	NHGMHFR			Oxidation (M)[4]		Mascot
921.4346	921.4099	-0.0247	-27	1	8	MSPQTETK					Mascot
1023.4741	1023.3725	-0.1016	-99	351	358	DDFVEKDR					Mascot
1116.583	1116.4741	-0.1089	-98	422	431	VALEACVQAR			Carbamidomethyl (C)[6]		Mascot
1116.583	1116.4741	-0.1089	-98	422	431	VALEACVQAR	18	0	Carbamidomethyl (C)[6]		Mascot
1275.7307	1275.606	-0.1247	-98	340	350	EITLGFVDLLR	102	100			Mascot
1275.7307	1275.606	-0.1247	-98	340	350	EITLGFVDLLR					Mascot
1433.7495	1433.6105	-0.139	-97	320	334	LSGGDHVHAGTVVGK					Mascot
1472.6978	1472.5441	-0.1537	-104	464	475	EIKFEFEAMDTL					Mascot
1488.6926	1488.5378	-0.1548	-104	464	475	EIKFEFEAMDTL			Oxidation (M)[9]		Mascot
1488.6926	1488.5378	-0.1548	-104	464	475	EIKFEFEAMDTL	45	99.677	Oxidation (M)[9]		Mascot
1546.7358	1546.5753	-0.1605	-104	451	463	WSPELAAACEVWK			Carbamidomethyl (C)[9]		Mascot
1546.7358	1546.5753	-0.1605	-104	451	463	WSPELAAACEVWK	72	100	Carbamidomethyl (C)[9]		Mascot
2009.059	2008.8403	-0.2187	-109	340	356	EITLGFVDLLRDDFVEK					Mascot

Rank	Protein Name	Accession No.	Protein MW	Protein PI	Pep. Count	Protein Score	Protein Score C. I. %	Total Ion Score	Total Ion C. I. %
2	Ribulose bisphosphate carboxylase large chain OS=Cucumis sativus GN=rbcL PE=1 SV=5	RBL_CUCSA	52608.4	6	9	291	100	253	100

Peptide Information

Calc. Mass	Obsrv. Mass	± da	± ppm	Start Seq.	End Seq.	Sequence	Ion Score	C. I. %	Modification	Rank	Result Type
830.4366	830.356	-0.0806	-97	440	446	EGNEIIR	16	0			Mascot
830.4366	830.356	-0.0806	-97	440	446	EGNEIIR					Mascot
914.4049	914.311	-0.0939	-103	306	312	NHGMHFR			Oxidation (M)[4]		Mascot
914.4049	914.311	-0.0939	-103	306	312	NHGMHFR			Oxidation (M)[4]		Mascot
921.4346	921.4099	-0.0247	-27	1	8	MSPQTETK					Mascot
1023.4741	1023.3725	-0.1016	-99	351	358	DDFVEKDR					Mascot
1116.583	1116.4741	-0.1089	-98	422	431	VALEACVQAR			Carbamidomethyl (C)[6]		Mascot
1116.583	1116.4741	-0.1089	-98	422	431	VALEACVQAR	18	0	Carbamidomethyl (C)[6]		Mascot
1275.7307	1275.606	-0.1247	-98	340	350	EITLGFVDLLR	102	100			Mascot
1275.7307	1275.606	-0.1247	-98	340	350	EITLGFVDLLR					Mascot
1472.6978	1472.5441	-0.1537	-104	464	475	EIKFEFEAMDTL					Mascot
1488.6926	1488.5378	-0.1548	-104	464	475	EIKFEFEAMDTL			Oxidation (M)[9]		Mascot
1488.6926	1488.5378	-0.1548	-104	464	475	EIKFEFEAMDTL	45	99.677	Oxidation (M)[9]		Mascot
1546.7358	1546.5753	-0.1605	-104	451	463	WSPELAAACEVWK			Carbamidomethyl (C)[9]		Mascot
1546.7358	1546.5753	-0.1605	-104	451	463	WSPELAAACEVWK	72	100	Carbamidomethyl (C)[9]		Mascot
2009.059	2008.8403	-0.2187	-109	340	356	EITLGFVDLLRDDFVEK					Mascot

Rank	Protein Name	Accession No.	Protein MW	Protein PI	Pep. Count	Protein Score	Protein Score C. I. %	Total Ion Score	Total Ion C. I. %
3	Ribulose bisphosphate carboxylase large chain OS=Nandina domestica GN=rbcL PE=3 SV=3	RBL_NANDO	52643.5	6.22	9	290	100	253	100

Peptide Information

Calc. Mass	Obsrv. Mass	± da	± ppm	Start Seq.	End Seq.	Sequence	Ion Score	C. I. %	Modification	Rank	Result Type
830.4366	830.356	-0.0806	-97	440	446	EGNEIIR	16	0			Mascot
830.4366	830.356	-0.0806	-97	440	446	EGNEIIR					Mascot
921.4346	921.4099	-0.0247	-27	1	8	MSPQTETK					Mascot
1116.583	1116.4741	-0.1089	-98	422	431	VALEACVQAR			Carbamidomethyl (C)[6]		Mascot
1116.583	1116.4741	-0.1089	-98	422	431	VALEACVQAR	18	0	Carbamidomethyl (C)[6]		Mascot
1260.6365	1260.5852	-0.0513	-41	440	450	EGNEIIRAACK			Carbamidomethyl (C)[10]		Mascot
1275.7307	1275.606	-0.1247	-98	340	350	EITLGFVDLLR	102	100			Mascot
1275.7307	1275.606	-0.1247	-98	340	350	EITLGFVDLLR					Mascot
1433.7998	1433.6105	-0.1893	-132	135	146	LEDLRIPTSYVK					Mascot
1472.6978	1472.5441	-0.1537	-104	464	475	EIKFEFEAMDTL					Mascot
1488.6926	1488.5378	-0.1548	-104	464	475	EIKFEFEAMDTL			Oxidation (M)[9]		Mascot
1488.6926	1488.5378	-0.1548	-104	464	475	EIKFEFEAMDTL	45	99.677	Oxidation (M)[9]		Mascot
1546.7358	1546.5753	-0.1605	-104	451	463	WSPELAAACEVWK			Carbamidomethyl (C)[9]		Mascot
1546.7358	1546.5753	-0.1605	-104	451	463	WSPELAAACEVWK	72	100	Carbamidomethyl (C)[9]		Mascot
2116.0784	2115.7766	-0.3018	-143	218	236	FLFCAEALFKAQAETGEIK					Mascot

Rank	Protein Name	Accession No.	Protein MW	Protein PI	Pep. Count	Protein Score	Protein Score C. I. %	Total Ion Score	Total Ion C. I. %
4	Ribulose bisphosphate carboxylase large chain (Fragment) OS=Saururus cernuus GN=rbcL PE=3 SV=1	RBL_SAUCE	51663	6	9	290	100	253	100

Peptide Information

Calc. Mass	Obsrv. Mass	± da	± ppm	Start Seq.	End Seq.	Sequence	Ion Score	C. I. %	Modification	Rank	Result Type
830.4366	830.356	-0.0806	-97	431	437	EGNEIIR	16	0			Mascot
830.4366	830.356	-0.0806	-97	431	437	EGNEIIR					Mascot
914.4049	914.311	-0.0939	-103	297	303	NHGMHFR			Oxidation (M)[4]		Mascot
914.4049	914.311	-0.0939	-103	297	303	NHGMHFR			Oxidation (M)[4]		Mascot
1116.583	1116.4741	-0.1089	-98	413	422	VALEACVQAR			Carbamidomethyl (C)[6]		Mascot
1116.583	1116.4741	-0.1089	-98	413	422	VALEACVQAR	18	0	Carbamidomethyl (C)[6]		Mascot
1229.6484	1229.5646	-0.0838	-68	431	441	EGNEIIREAAK					Mascot
1275.7307	1275.606	-0.1247	-98	331	341	EITLGFVDLLR	102	100			Mascot
1275.7307	1275.606	-0.1247	-98	331	341	EITLGFVDLLR					Mascot
1472.6978	1472.5441	-0.1537	-104	455	466	EIKFEFEAMDTL					Mascot
1475.7351	1475.6086	-0.1265	-86	207	218	DRFLFCAEAIYK					Mascot
1488.6926	1488.5378	-0.1548	-104	455	466	EIKFEFEAMDTL			Oxidation (M)[9]		Mascot
1488.6926	1488.5378	-0.1548	-104	455	466	EIKFEFEAMDTL	45	99.677	Oxidation (M)[9]		Mascot
1546.7358	1546.5753	-0.1605	-104	442	454	WSPELAAACEVWK			Carbamidomethyl (C)[9]		Mascot
1546.7358	1546.5753	-0.1605	-104	442	454	WSPELAAACEVWK	72	100	Carbamidomethyl (C)[9]		Mascot
2148.0681	2147.7698	-0.2983	-139	209	227	FLFCAEAIYKSQAETGEIK					Mascot

Rank	Protein Name	Accession No.	Protein MW	Protein PI	Pep. Count	Protein Score	Protein Score C. I. %	Total Ion Score	Total Ion C. I. %
5	Ribulose bisphosphate carboxylase large chain (Fragment) OS=Manilkara zapota GN=rbcL PE=3 SV=2	RBL_MANZA	51550	6.04	8	284	100	253	100

Peptide Information

Calc. Mass	Obsrv. Mass	± da	± ppm	Start Seq.	End Seq.	Sequence	Ion Score	C. I. %	Modification	Rank	Result Type
830.4366	830.356	-0.0806	-97	430	436	EGNEIIR	16	0			Mascot
830.4366	830.356	-0.0806	-97	430	436	EGNEIIR					Mascot
914.4049	914.311	-0.0939	-103	296	302	NHGMHFR			Oxidation (M)[4]		Mascot
914.4049	914.311	-0.0939	-103	296	302	NHGMHFR			Oxidation (M)[4]		Mascot
1039.4691	1039.4204	-0.0487	-47	341	348	DDYVEKDR					Mascot
1116.583	1116.4741	-0.1089	-98	412	421	VALEACVQAR			Carbamidomethyl (C)[6]		Mascot
1116.583	1116.4741	-0.1089	-98	412	421	VALEACVQAR	18	0	Carbamidomethyl (C)[6]		Mascot
1275.7307	1275.606	-0.1247	-98	330	340	EITLGFVDLLR	102	100			Mascot
1275.7307	1275.606	-0.1247	-98	330	340	EITLGFVDLLR					Mascot
1433.7495	1433.6105	-0.139	-97	310	324	LSGGDHVHAGTVVGK					Mascot
1472.6978	1472.5441	-0.1537	-104	454	465	EIKFEFEAMDTL					Mascot
1488.6926	1488.5378 #	-0.1548	-104	454	465	EIKFEFEAMDTL			Oxidation (M)[9]		Mascot
1488.6926	1488.5378	-0.1548	-104	454	465	EIKFEFEAMDTL	45	99.677	Oxidation (M)[9]		Mascot
1546.7358	1546.5753	-0.1605	-104	441	453	WSPELAAACEVWK			Carbamidomethyl (C)[9]		Mascot
1546.7358	1546.5753	-0.1605	-104	441	453	WSPELAAACEVWK	72	100	Carbamidomethyl (C)[9]		Mascot

Rank	Protein Name	Accession No.	Protein MW	Protein PI	Pep. Count	Protein Score	Protein Score C. I. %	Total Ion Score	Total Ion C. I. %
6	Ribulose bisphosphate carboxylase large chain OS=Calycanthus floridus var. glaucus GN=rbcL PE=3 SV=	RBL_CALFG	52571.5	6.23	8	284	100	253	100

Peptide Information

Calc. Mass	Obsrv. Mass	± da	± ppm	Start Seq.	End Seq.	Sequence	Ion Score	C. I. %	Modification	Rank	Result Type
830.4366	830.356	-0.0806	-97	440	446	EGNEIIR	16	0			Mascot
830.4366	830.356	-0.0806	-97	440	446	EGNEIIR					Mascot
914.4049	914.311	-0.0939	-103	306	312	NHGMHFR			Oxidation (M)[4]		Mascot
914.4049	914.311	-0.0939	-103	306	312	NHGMHFR			Oxidation (M)[4]		Mascot
921.4709	921.4099	-0.061	-66	1	8	MSPKTETK					Mascot
933.5515	933.4146	-0.1369	-147	9	18	AGVGFKAGVK					Mascot
933.5515	933.4146	-0.1369	-147	9	18	AGVGFKAGVK					Mascot
1116.583	1116.4741	-0.1089	-98	422	431	VALEACVQAR			Carbamidomethyl (C)[6]		Mascot
1116.583	1116.4741	-0.1089	-98	422	431	VALEACVQAR	18	0	Carbamidomethyl (C)[6]		Mascot
1275.7307	1275.606	-0.1247	-98	340	350	EITLGFVDLLR	102	100			Mascot
1275.7307	1275.606	-0.1247	-98	340	350	EITLGFVDLLR					Mascot
1472.6978	1472.5441	-0.1537	-104	464	475	EIKFEFEAMDTL					Mascot
1488.6926	1488.5378	-0.1548	-104	464	475	EIKFEFEAMDTL			Oxidation (M)[9]		Mascot
1488.6926	1488.5378	-0.1548	-104	464	475	EIKFEFEAMDTL	45	99.677	Oxidation (M)[9]		Mascot
1546.7358	1546.5753	-0.1605	-104	451	463	WSPELAAACEVWK			Carbamidomethyl (C)[9]		Mascot
1546.7358	1546.5753	-0.1605	-104	451	463	WSPELAAACEVWK	72	100	Carbamidomethyl (C)[9]		Mascot

Rank	Protein Name	Accession No.	Protein MW	Protein PI	Pep. Count	Protein Score	Protein Score C. I. %	Total Ion Score	Total Ion C. I. %
7	Ribulose bisphosphate carboxylase large chain OS=Liquidambar styraciflua GN=rbcL PE=3 SV=1	RBL_LIQST	52625.5	6.04	8	284	100	253	100

Peptide Information

Calc. Mass	Obsrv. Mass	± da	± ppm	Start Seq.	End Seq.	Sequence	Ion Score	C. I. %	Modification	Rank	Result Type
830.4366	830.356	-0.0806	-97	440	446	EGNEIIR	16	0			Mascot
830.4366	830.356	-0.0806	-97	440	446	EGNEIIR					Mascot
914.4049	914.311	-0.0939	-103	306	312	NHGMHFR			Oxidation (M)[4]		Mascot
914.4049	914.311	-0.0939	-103	306	312	NHGMHFR			Oxidation (M)[4]		Mascot
921.4346	921.4099	-0.0247	-27	1	8	MSPQTETK					Mascot
1116.583	1116.4741	-0.1089	-98	422	431	VALEACVQAR			Carbamidomethyl (C)[6]		Mascot
1116.583	1116.4741	-0.1089	-98	422	431	VALEACVQAR	18	0	Carbamidomethyl (C)[6]		Mascot
1275.7307	1275.606	-0.1247	-98	340	350	EITLGFVDLLR	102	100			Mascot
1275.7307	1275.606	-0.1247	-98	340	350	EITLGFVDLLR					Mascot
1472.6978	1472.5441	-0.1537	-104	464	475	EIKFEFEAMDTL					Mascot
1475.7351	1475.6086	-0.1265	-86	216	227	DRFLFCAEAIYK					Mascot
1488.6926	1488.5378	-0.1548	-104	464	475	EIKFEFEAMDTL			Oxidation (M)[9]		Mascot
1488.6926	1488.5378	-0.1548	-104	464	475	EIKFEFEAMDTL	45	99.677	Oxidation (M)[9]		Mascot
1546.7358	1546.5753	-0.1605	-104	451	463	WSPELAAACEVWK			Carbamidomethyl (C)[9]		Mascot
1546.7358	1546.5753	-0.1605	-104	451	463	WSPELAAACEVWK	72	100	Carbamidomethyl (C)[9]		Mascot

Rank	Protein Name	Accession No.	Protein MW	Protein PI	Pep. Count	Protein Score	Protein Score C. I. %	Total Ion Score	Total Ion C. I. %
8	Ribulose bisphosphate carboxylase large chain (Fragment) OS=Cornus florida GN=rbcL PE=3 SV=2	RBL_CORFO	51593.1	6.14	8	284	100	253	100

Peptide Information

Calc. Mass	Obsrv. Mass	± da	± ppm	Start Seq.	End Seq.	Sequence	Ion Score	C. I. %	Modification	Rank	Result Type
830.4366	830.356	-0.0806	-97	430	436	EGNEIIR	16	0			Mascot
830.4366	830.356	-0.0806	-97	430	436	EGNEIIR					Mascot
1023.4741	1023.3725	-0.1016	-99	341	348	DDFVEKDR					Mascot
1116.583	1116.4741	-0.1089	-98	412	421	VALEACVQAR			Carbamidomethyl (C)[6]		Mascot
1116.583	1116.4741	-0.1089	-98	412	421	VALEACVQAR	18	0	Carbamidomethyl (C)[6]		Mascot
1275.7307	1275.606	-0.1247	-98	330	340	EITLGFVDLLR	102	100			Mascot
1275.7307	1275.606	-0.1247	-98	330	340	EITLGFVDLLR					Mascot
1472.6978	1472.5441	-0.1537	-104	454	465	EIKFEFEAMDTL					Mascot
1475.7351	1475.6086	-0.1265	-86	206	217	DRFLFCAEALYK					Mascot
1488.6926	1488.5378	-0.1548	-104	454	465	EIKFEFEAMDTL			Oxidation (M)[9]		Mascot
1488.6926	1488.5378	-0.1548	-104	454	465	EIKFEFEAMDTL	45	99.677	Oxidation (M)[9]		Mascot
1546.7358	1546.5753	-0.1605	-104	441	453	WSPELAAACEVWK			Carbamidomethyl (C)[9]		Mascot
1546.7358	1546.5753	-0.1605	-104	441	453	WSPELAAACEVWK	72	100	Carbamidomethyl (C)[9]		Mascot
2009.059	2008.8403	-0.2187	-109	330	346	EITLGFVDLLRDDFVEK					Mascot

Rank	Protein Name	Accession No.	Protein MW	Protein PI	Pep. Count	Protein Score	Protein Score C. I. %	Total Ion Score	Total Ion C. I. %
9	Ribulose bisphosphate carboxylase large chain (Fragment) OS=Hamamelis mollis GN=rbcL PE=3 SV=1	RBL_HAMMO	51542.9	6.05	7	278	100	253	100

Peptide Information

Calc. Mass	Obsrv. Mass	± da	± ppm	Start Seq.	End Seq.	Sequence	Ion Score	C. I. %	Modification	Rank	Result Type
830.4366	830.356	-0.0806	-97	430	436	EGNEIIR	16	0			Mascot
830.4366	830.356	-0.0806	-97	430	436	EGNEIIR					Mascot
	830.356										Mascot
914.4049	914.311	-0.0939	-103	296	302	NHGMHFR			Oxidation (M)[4]		Mascot
914.4049	914.311	-0.0939	-103	296	302	NHGMHFR			Oxidation (M)[4]		Mascot
1116.583	1116.4741	-0.1089	-98	412	421	VALEACVQAR			Carbamidomethyl (C)[6]		Mascot
1116.583	1116.4741	-0.1089	-98	412	421	VALEACVQAR	18	0	Carbamidomethyl (C)[6]		Mascot
1275.7307	1275.606	-0.1247	-98	330	340	EITLGFVDLLR	102	100			Mascot
1275.7307	1275.606	-0.1247	-98	330	340	EITLGFVDLLR					Mascot
1472.6978	1472.5441	-0.1537	-104	454	465	EIKFEFEAMDTL					Mascot
1475.7351	1475.6086	-0.1265	-86	206	217	DRFLFCAEAIYK					Mascot
1488.6926	1488.5378	-0.1548	-104	454	465	EIKFEFEAMDTL			Oxidation (M)[9]		Mascot
1488.6926	1488.5378	-0.1548	-104	454	465	EIKFEFEAMDTL	45	99.677	Oxidation (M)[9]		Mascot
1546.7358	1546.5753	-0.1605	-104	441	453	WSPELAAACEVWK			Carbamidomethyl (C)[9]		Mascot
1546.7358	1546.5753	-0.1605	-104	441	453	WSPELAAACEVWK	72	100	Carbamidomethyl (C)[9]		Mascot

Rank	Protein Name	Accession No.	Protein MW	Protein PI	Pep. Count	Protein Score	Protein Score C. I. %	Total Ion Score	Total Ion C. I. %
10	Ribulose bisphosphate carboxylase large chain (Fragment) OS=Coriaria myrtifolia GN=rbcL PE=3 SV=1	RBL_CORMY	51666.1	6	7	277	100	253	100

Peptide Information

Calc. Mass	Obsrv. Mass	± da	± ppm	Start Seq.	End Seq.	Sequence	Ion Score	C. I. %	Modification	Rank	Result Type
830.4366	830.356	-0.0806	-97	431	437	EGNEIIR	16	0			Mascot
830.4366	830.356	-0.0806	-97	431	437	EGNEIIR					Mascot
1116.583	1116.4741	-0.1089	-98	413	422	VALEACVQAR			Carbamidomethyl (C)[6]		Mascot
1116.583	1116.4741	-0.1089	-98	413	422	VALEACVQAR	18	0	Carbamidomethyl (C)[6]		Mascot
1275.7307	1275.606	-0.1247	-98	331	341	EITLGFVDLLR	102	100			Mascot
1275.7307	1275.606	-0.1247	-98	331	341	EITLGFVDLLR					Mascot
1472.6978	1472.5441	-0.1537	-104	455	466	EIKFEFEAMDTL					Mascot
1475.7351	1475.6086	-0.1265	-86	207	218	DRFLFCAEAIYK					Mascot
1488.6926	1488.5378	-0.1548	-104	455	466	EIKFEFEAMDTL			Oxidation (M)[9]		Mascot
1488.6926	1488.5378	-0.1548	-104	455	466	EIKFEFEAMDTL	45	99.677	Oxidation (M)[9]		Mascot
1546.7358	1546.5753	-0.1605	-104	442	454	WSPELAAACEVWK			Carbamidomethyl (C)[9]		Mascot
1546.7358	1546.5753	-0.1605	-104	442	454	WSPELAAACEVWK	72	100	Carbamidomethyl (C)[9]		Mascot
2148.0681	2147.7698	-0.2983	-139	209	227	FLFCAEAIYKSQAETGEIK					Mascot

Gel Idx/Pos	116/E15	Instr./Gel Origin	BA2060/131017A	Process State	Analysis Succeeded
Plate [#] Name	[1] 31496	Instrument Sample Name		Spectra	8

Rank	Protein Name	Accession No.	Protein MW	Protein PI	Pep. Count	Protein Score	Protein Score C. I. %	Total Ion Score	Total Ion C. I. %
1	Ribulose bisphosphate carboxylase small chain chloroplastic OS=Glycine max GN=RBCS-1 PE=3 SV=1	RBS1_SOYB	20060	8.87	12	521	100	429	100

Peptide Information

Calc. Mass	Obsrv. Mass	± da	± ppm	Start Seq.	End Seq.	Sequence	Ion Score	C. I. %	Modification	Rank	Result Type
866.4189	866.3433	-0.0756	-87	30	37	SMAGFPTR					Mascot
882.4138	882.4902	0.0764	87	30	37	SMAGFPTR			Oxidation (M)[2]		Mascot
914.4002	914.3235	-0.0767	-84	113	120	SPXYYDGR	54	99.966			Mascot
914.4229	914.3235	-0.0994	-109	121	126	YWTMWK					Mascot
921.504	921.4245	-0.0795	-86	84	90	EVEYLLR					Mascot
930.4178	930.334	-0.0838	-90	121	126	YWTMWK			Oxidation (M)[4]		Mascot
933.5151	933.435	-0.0801	-86	156	163	IIGFDNVR	46	99.757			Mascot
933.5151	933.435	-0.0801	-86	156	163	IIGFDNVR					Mascot
1038.5367	1038.4462	-0.0905	-87	147	155	TAYPNGFIR					Mascot
1754.8934	1754.729	-0.1644	-94	164	178	QVQCISFIAYKPPGF			Carbamidomethyl (C)[4]		Mascot
1754.8934	1754.729	-0.1644	-94	164	178	QVQCISFIAYKPPGF	79	100	Carbamidomethyl (C)[4]		Mascot
1938.9695	1938.7888	-0.1807	-93	67	83	FETLSYLPDLDDAQLAK					Mascot
2067.0513	2066.8684	-0.1829	-88	17	37	AGAGMVAPFTGLKSMAGFPTR					Mascot
2067.0645	2066.8684	-0.1961	-95	66	83	KFETLSYLPDLDDAQLAK	117	100			Mascot
2152.032	2151.8276	-0.2044	-95	92	108	GWIPCLEFELEHGFVYR	70	100	Carbamidomethyl (C)[5]		Mascot
2152.032	2151.8276	-0.2044	-95	92	108	GWIPCLEFELEHGFVYR			Carbamidomethyl (C)[5]		Mascot
2280.127	2279.9072	-0.2198	-96	91	108	KGWIPCLEFELEHGFVYR			Carbamidomethyl (C)[6]		Mascot
2280.127	2279.9072	-0.2198	-96	91	108	KGWIPCLEFELEHGFVYR	66	99.998	Carbamidomethyl (C)[6]		Mascot

Rank	Protein Name	Accession No.	Protein MW	Protein PI	Pep. Count	Protein Score	Protein Score C. I. %	Total Ion Score	Total Ion C. I. %
2	Ribulose bisphosphate carboxylase small chain chloroplastic OS=Glycine tomentella GN=RBCS PE=3 SV=	RBS_GLYTO	19936	8.87	10	425	100	352	100

Peptide Information

Calc. Mass	Obsrv. Mass	± da	± ppm	Start Seq.	End Seq.	Sequence	Ion Score	C. I. %	Modification	Rank	Result Type
866.4189	866.3433	-0.0756	-87	30	37	SMAGFPTR					Mascot
882.4138	882.4902	0.0764	87	30	37	SMAGFPTR			Oxidation (M)[2]		Mascot
914.4002	914.3235	-0.0767	-84	113	120	SPGYYDGR	54	99.966			Mascot
914.4002	914.3235	-0.0767	-84	113	120	SPGYYDGR					Mascot
921.504	921.4245	-0.0795	-86	84	90	EVEYLLR					Mascot
930.4178	930.334	-0.0838	-90	121	126	YWTMWK			Oxidation (M)[4]		Mascot
933.5151	933.435	-0.0801	-86	156	163	IIGFDNVR	46	99.757			Mascot
933.5151	933.435	-0.0801	-86	156	163	IIGFDNVR					Mascot
1038.5367	1038.4462	-0.0905	-87	147	155	TAYPNGFIR					Mascot
1938.9695	1938.7888	-0.1807	-93	67	83	FETLSYLPDLDDAQLAK					Mascot
2067.0645	2066.8684	-0.1961	-95	66	83	KFETLSYLPDLDDAQLAK	117	100			Mascot
2067.0645	2066.8684	-0.1961	-95	66	83	KFETLSYLPDLDDAQLAK					Mascot
2152.032	2151.8276	-0.2044	-95	92	108	GWIPCLEFELEHGFVYR	70	100	Carbamidomethyl (C)[5]		Mascot
2152.032	2151.8276	-0.2044	-95	92	108	GWIPCLEFELEHGFVYR			Carbamidomethyl (C)[5]		Mascot
2280.127	2279.9072	-0.2198	-96	91	108	KGWIPCLEFELEHGFVYR			Carbamidomethyl (C)[6]		Mascot
2280.127	2279.9072	-0.2198	-96	91	108	KGWIPCLEFELEHGFVYR	66	99.998	Carbamidomethyl (C)[6]		Mascot

Rank	Protein Name	Accession No.	Protein MW	Protein PI	Pep. Count	Protein Score	Protein Score C. I. %	Total Ion Score	Total Ion C. I. %
3	Ribulose bisphosphate carboxylase small chain, chloroplastic OS=Glycine tabacina GN=RBCS PE=3 SV=1	RBS_GLYTA	20005	8.87	9	412	100	352	100

Peptide Information

Calc. Mass	Obsrv. Mass	± da	± ppm	Start Seq.	End Seq.	Sequence	Ion Score	C. I. %	Modification	Rank	Result Type
866.4189	866.3433	-0.0756	-87	30	37	SMAGFPTR					Mascot
882.4138	882.4902	0.0764	87	30	37	SMAGFPTR			Oxidation (M)[2]		Mascot
914.4002	914.3235	-0.0767	-84	113	120	SPGYYDGR	54	99.966			Mascot
914.4002	914.3235	-0.0767	-84	113	120	SPGYYDGR					Mascot
921.504	921.4245	-0.0795	-86	84	90	EVEYLLR					Mascot
930.4178	930.334	-0.0838	-90	121	126	YWTMWK			Oxidation (M)[4]		Mascot
933.5151	933.435	-0.0801	-86	156	163	IIGFDNVR	46	99.757			Mascot
933.5151	933.435	-0.0801	-86	156	163	IIGFDNVR					Mascot
1938.9695	1938.7888	-0.1807	-93	67	83	FETLSYLPDLDDAQLAK					Mascot
2067.0645	2066.8684	-0.1961	-95	66	83	KFETLSYLPDLDDAQLAK	117	100			Mascot
2067.0645	2066.8684	-0.1961	-95	66	83	KFETLSYLPDLDDAQLAK					Mascot
2152.032	2151.8276	-0.2044	-95	92	108	GWIPCLEFELEHGFVYR	70	100	Carbamidomethyl (C)[5]		Mascot
2152.032	2151.8276	-0.2044	-95	92	108	GWIPCLEFELEHGFVYR			Carbamidomethyl (C)[5]		Mascot
2280.127	2279.9072	-0.2198	-96	91	108	KGWIPCLEFELEHGFVYR			Carbamidomethyl (C)[6]		Mascot
2280.127	2279.9072	-0.2198	-96	91	108	KGWIPCLEFELEHGFVYR	66	99.998	Carbamidomethyl (C)[6]		Mascot

Rank	Protein Name	Accession No.	Protein MW	Protein PI	Pep. Count	Protein Score	Protein Score C. I. %	Total Ion Score	Total Ion C. I. %
4	Ribulose bisphosphate carboxylase small chain chloroplastic OS=Glycine max GN=RBCS-4 PE=3 SV=1	RBS4_SOYB	20004	8.87	8	354	100	305	100

Peptide Information

Calc. Mass	Obsrv. Mass	± da	± ppm	Start Seq.	End Seq.	Sequence	Ion Score	C. I. %	Modification	Rank	Result Type
914.4229	914.3235	-0.0994	-109	121	126	YWTMWK	6	0			Mascot
	914.3235										Mascot
914.4229	914.3235	-0.0994	-109	121	126	YWTMWK					Mascot
921.504	921.4245	-0.0795	-86	84	90	EVEYLLR					Mascot
930.4178	930.334	-0.0838	-90	121	126	YWTMWK			Oxidation (M)[4]		Mascot
933.5151	933.435	-0.0801	-86	156	163	IIGFDNVR	46	99.757			Mascot
933.5151	933.435	-0.0801	-86	156	163	IIGFDNVR					Mascot
1038.5367	1038.4462	-0.0905	-87	147	155	TAYPNGFIR					Mascot
1938.9695	1938.7888	-0.1807	-93	67	83	FETLSYLPDLDDAQLAK					Mascot
2067.0645	2066.8684	-0.1961	-95	66	83	KFETLSYLPDLDDAQLAK	117	100			Mascot
2067.0645	2066.8684	-0.1961	-95	66	83	KFETLSYLPDLDDAQLAK					Mascot
2152.032	2151.8276	-0.2044	-95	92	108	GWIPCLEFELEHGFVYR	70	100	Carbamidomethyl (C)[5]		Mascot
2152.032	2151.8276	-0.2044	-95	92	108	GWIPCLEFELEHGFVYR			Carbamidomethyl (C)[5]		Mascot
2280.127	2279.9072	-0.2198	-96	91	108	KGWIPCLEFELEHGFVYR			Carbamidomethyl (C)[6]		Mascot
2280.127	2279.9072	-0.2198	-96	91	108	KGWIPCLEFELEHGFVYR	66	99.998	Carbamidomethyl (C)[6]		Mascot

Rank	Protein Name	Accession No.	Protein MW	Protein PI	Pep. Count	Protein Score	Protein Score C. I. %	Total Ion Score	Total Ion C. I. %
5	Ribulose bisphosphate carboxylase small chain chloroplastic OS=Flaveria pringlei GN=RBCS6 PE=2 S	RBS6_FLAPR	19694.8	9.07	6	193	100	163	100

Peptide Information

Calc. Mass	Obsrv. Mass	± da	± ppm	Start Seq.	End Seq.	Sequence	Ion Score	C. I. %	Modification	Rank	Result Type
914.4002	914.3235	-0.0767	-84	108	115	SPGYYDGR	54	99.966			Mascot
914.4002	914.3235	-0.0767	-84	108	115	SPGYYDGR					Mascot
930.4178	930.334	-0.0838	-90	116	121	YWTMWK			Oxidation (M)[4]		Mascot
933.5151	933.435	-0.0801	-86	151	158	IIGFDNVR	46	99.757			Mascot
933.5151	933.435	-0.0801	-86	151	158	IIGFDNVR					Mascot
1491.755	1491.614	-0.141	-95	34	47	KVNDFSTLPSNGGR					Mascot
1754.8418	1754.729	-0.1128	-64	159	173	QVQCISFIASKPDXF	35	97.263	Carbamidomethyl (C)[4]		Mascot
2067.1121	2066.8684	-0.2437	-118	61	78	KYXTLSYLPNLTEAQLAK	29	89.326			Mascot

Rank	Protein Name	Accession No.	Protein MW	Protein PI	Pep. Count	Protein Score	Protein Score C. I. %	Total Ion Score	Total Ion C. I. %
6	Ribulose bisphosphate carboxylase small chain, chloroplastic OS=Helianthus annuus GN=RBCS PE=2 SV=1	RBS_HELAN	20198.1	9.07	4	168	100	153	100

Peptide Information

Calc. Mass	Obsrv. Mass	± da	± ppm	Start Seq.	End Seq.	Sequence	Ion Score	C. I. %	Modification	Rank	Result Type
914.4002	914.3235	-0.0767	-84	113	120	SPGYYDGR	54	99.966			Mascot
914.4002	914.3235	-0.0767	-84	113	120	SPGYYDGR					Mascot
930.4178	930.334	-0.0838	-90	121	126	YWTMWK			Oxidation (M)[4]		Mascot
933.5151	933.435	-0.0801	-86	156	163	IIGFDNVR	46	99.757			Mascot
933.5151	933.435	-0.0801	-86	156	163	IIGFDNVR					Mascot
2152.0684	2151.8276	-0.2408	-112	92	108	KWVPCLEFELEHGFVYR	55	99.972			Mascot
2152.0684	2151.8276	-0.2408	-112	92	108	KWVPCLEFELEHGFVYR					Mascot

Rank	Protein Name	Accession No.	Protein MW	Protein PI	Pep. Count	Protein Score	Protein Score C. I. %	Total Ion Score	Total Ion C. I. %
7	Ribulose bisphosphate carboxylase small chain chloroplastic OS=Petunia hybrida GN=RBCS PE=3 S	RBS1_PETHY	20357.2	8.29	5	159	100	130	100

Peptide Information

Calc. Mass	Obsrv. Mass	± da	± ppm	Start Seq.	End Seq.	Sequence	Ion Score	C. I. %	Modification	Rank	Result Type
914.4229	914.3235	-0.0994	-109	123	128	YWTMWK	6	0			Mascot
914.4229	914.3235	-0.0994	-109	123	128	YWTMWK					Mascot
930.4178	930.334	-0.0838	-90	123	128	YWTMWK			Oxidation (M)[4]		Mascot
933.5151	933.435	-0.0801	-86	158	165	IIGFDNVR	46	99.757			Mascot
933.5151	933.435	-0.0801	-86	158	165	IIGFDNVR					Mascot
1021.5564	1021.4158	-0.1406	-138	86	93	EIEYLLNK					Mascot
1663.8472	1663.7256	-0.1216	-73	15	30	TNVAQASMVAPFNGLK			Oxidation (M)[8]		Mascot
1754.8934	1754.729	-0.1644	-94	166	180	QVQCISFIAYKPPGF			Carbamidomethyl (C)[4]		Mascot
1754.8934	1754.729	-0.1644	-94	166	180	QVQCISFIAYKPPGF	79	100	Carbamidomethyl (C)[4]		Mascot

Rank	Protein Name	Accession No.	Protein MW	Protein PI	Pep. Count	Protein Score	Protein Score C. I. %	Total Ion Score	Total Ion C. I. %
8	Ribulose bisphosphate carboxylase small chain, chloroplastic OS=Nicotiana tabacum GN=RBCS PE=1 SV=1	RBS_TOBAC	20298.1	7.6	7	147	100	98	100

Peptide Information

Calc. Mass	Obsrv. Mass	± da	± ppm	Start Seq.	End Seq.	Sequence	Ion Score	C. I. %	Modification	Rank	Result Type
914.4002	914.3235	-0.0767	-84	115	122	SPGYYDGR	54	99.966			Mascot
914.4002	914.3235	-0.0767	-84	115	122	SPGYYDGR					Mascot
921.4788	921.4245	-0.0543	-59	31	39	SAASFPVSR					Mascot
930.4178	930.334	-0.0838	-90	123	128	YWTMWK			Oxidation (M)[4]		Mascot
933.5151	933.435	-0.0801	-86	158	165	IIGFDNVR	46	99.757			Mascot
933.5151	933.435	-0.0801	-86	158	165	IIGFDNVR					Mascot
1663.8472	1663.7256	-0.1216	-73	15	30	SNVAQANMVAPFTGLK			Oxidation (M)[8]		Mascot
2152.03	2151.8276	-0.2024	-94	129	148	LPMFGCTDATQVLAEVEEAK					Mascot
2152.03	2151.8276	-0.2024	-94	129	148	LPMFGCTDATQVLAEVEEAK					Mascot
2168.0249	2167.8206	-0.2043	-94	129	148	LPMFGCTDATQVLAEVEEAK			Oxidation (M)[3]		Mascot
2225.0464	2224.915	-0.1314	-59	129	148	LPMFGCTDATQVLAEVEEAK			Carbamidomethyl (C)[6], Oxidation (M)[3]		Mascot
2280.125	2279.9072	-0.2178	-96	129	149	LPMFGCTDATQVLAEVEEAKK					Mascot
2280.125	2279.9072	-0.2178	-96	129	149	LPMFGCTDATQVLAEVEEAKK					Mascot
2296.1199	2295.9023	-0.2176	-95	129	149	LPMFGCTDATQVLAEVEEAKK			Oxidation (M)[3]		Mascot

Rank	Protein Name	Accession No.	Protein MW	Protein PI	Pep. Count	Protein Score	Protein Score C. I. %	Total Ion Score	Total Ion C. I. %
9	Ribulose bisphosphate carboxylase small chain chloroplastic OS=Solanum tuberosum GN=RBCS-2A PE=	RBSA_SOLT	20276	7.6	6	134	100	98	100

Peptide Information

Calc. Mass	Obsrv. Mass	± da	± ppm	Start Seq.	End Seq.	Sequence	Ion Score	C. I. %	Modification	Rank	Result Type
914.4002	914.3235	-0.0767	-84	115	122	SPGYYDGR	54	99.966			Mascot
914.4002	914.3235	-0.0767	-84	115	122	SPGYYDGR					Mascot
921.4788	921.4245	-0.0543	-59	31	39	SAASFPVSR					Mascot
930.4178	930.334	-0.0838	-90	123	128	YWTMWK			Oxidation (M)[4]		Mascot
933.5151	933.435	-0.0801	-86	158	165	IIGFDNVR	46	99.757			Mascot
933.5151	933.435	-0.0801	-86	158	165	IIGFDNVR					Mascot
2152.03	2151.8276	-0.2024	-94	129	148	LPMFGCTDATQVLAEVEEAK					Mascot
2152.03	2151.8276	-0.2024	-94	129	148	LPMFGCTDATQVLAEVEEAK					Mascot
2168.0249	2167.8206	-0.2043	-94	129	148	LPMFGCTDATQVLAEVEEAK			Oxidation (M)[3]		Mascot
2225.0464	2224.915	-0.1314	-59	129	148	LPMFGCTDATQVLAEVEEAK			Carbamidomethyl (C)[6], Oxidation (M)[3]		Mascot
2280.125	2279.9072	-0.2178	-96	129	149	LPMFGCTDATQVLAEVEEAKK					Mascot
2280.125	2279.9072	-0.2178	-96	129	149	LPMFGCTDATQVLAEVEEAKK					Mascot
2296.1199	2295.9023	-0.2176	-95	129	149	LPMFGCTDATQVLAEVEEAKK			Oxidation (M)[3]		Mascot

Rank	Protein Name	Accession No.	Protein MW	Protein PI	Pep. Count	Protein Score	Protein Score C. I. %	Total Ion Score	Total Ion C. I. %
10	Ribulose bisphosphate carboxylase small chain, chloroplastic OS=Oryza sativa subsp. japonica GN=RBC	RBS1_ORYSJ	19633.9	9.04	6	130	100	98	100

Peptide Information

Calc. Mass	Obsrv. Mass	± da	± ppm	Start Seq.	End Seq.	Sequence	Ion Score	C. I. %	Modification	Rank	Result Type

				Seq.	Seq.		Score			
914.4002	914.3235	-0.0767	-84	104	111	SPGYYDGR	54	99.966		Mascot
914.4002	914.3235	-0.0767	-84	104	111	SPGYYDGR				Mascot
930.4178	930.334	-0.0838	-90	112	117	YWTMWK			Oxidation (M)[4]	Mascot
933.5151	933.435	-0.0801	-86	147	154	IIGFDNVR	46	99.757		Mascot
933.5151	933.435	-0.0801	-86	147	154	IIGFDNVR				Mascot
1045.5571	1045.4766	-0.0805	-77	21	30	STAGMPVARR				Mascot
1165.571	1165.6375	0.0665	57	85	93	WVPCLEFSK			Carbamidomethyl (C)[4]	Mascot
2280.125	2279.9072	-0.2178	-96	118	137	LPMFGCTDATQVLKELEE AK			Carbamidomethyl (C)[6]	Mascot
2280.125	2279.9072	-0.2178	-96	118	137	LPMFGCTDATQVLKELEE AK			Carbamidomethyl (C)[6]	Mascot
2296.1199	2295.9023	-0.2176	-95	118	137	LPMFGCTDATQVLKELEE AK			Carbamidomethyl (C)[6], Oxidation (M)[3]	Mascot

Gel Idx/Pos	117/E16	Instr./Gel Origin	BA2060/131017A	Process Sta	Analysis Succeeded
Plate [#] Name	[1] 31496	Instrument Sample Name		Spectra	8

Rank	Protein Name	Accession No	Protein MW	Protein PI	Pep. Count	Protein Score	Protein Score C. I. %	Total Ion Score	Total Ion C. I. %
1	Ribulose bisphosphate carboxylase small chain chloroplastic OS=Glycine max GN=RBCS-1 PE=3 SV=1	RBS1_SOYBN	20060	8.87	12	561	100	468	100

Peptide Information

Calc. Mass	Obsrv. Mass	± da	± ppm	Start Seq.	End Seq.	Sequence	Ion Score	C. I. %	Modification	Rank	Result Type
866.4189	866.3524	-0.0665	-77	30	37	SMAGFPTR					Mascot
882.4138	882.4923	0.0785	89	30	37	SMAGFPTR			Oxidation (M)[2]		Mascot
914.4002	914.3317	-0.0685	-75	113	120	SPXYYDGR	48	99.858			Mascot
914.4229	914.3317	-0.0912	-100	121	126	YWTMWK					Mascot
921.504	921.4318	-0.0722	-78	84	90	EVEYLLR					Mascot
930.4178	930.3439	-0.0739	-79	121	126	YWTMWK			Oxidation (M)[4]		Mascot
933.5151	933.4431	-0.072	-77	156	163	IIGFDNVR	41	99.279			Mascot
933.5151	933.4431	-0.072	-77	156	163	IIGFDNVR					Mascot
1038.5367	1038.4554	-0.0813	-78	147	155	TAYPNGFIR					Mascot
1038.5367	1038.4554	-0.0813	-78	147	155	TAYPNGFIR	44	99.658			Mascot
1754.8934	1754.7472	-0.1462	-83	164	178	QVQCISFIAYKPPGF			Carbamidomethyl (C)[4]		Mascot
1754.8934	1754.7472	-0.1462	-83	164	178	QVQCISFIAYKPPGF	81	100	Carbamidomethyl (C)[4]		Mascot
1938.9695	1938.8055	-0.164	-85	67	83	FETLSYLPDLDDAQLAK					Mascot
2067.0513	2066.8894	-0.1619	-78	17	37	AGAGMVAPFTGLKSMAG FPTR					Mascot
2067.0645	2066.8894	-0.1751	-85	66	83	KFETLSYLPDLDDAQLAK	106	100			Mascot
2152.032	2151.8508	-0.1812	-84	92	108	GWIPCLEFELEHGFVYR			Carbamidomethyl (C)[5]		Mascot
2152.032	2151.8508	-0.1812	-84	92	108	GWIPCLEFELEHGFVYR	68	99.998	Carbamidomethyl (C)[5]		Mascot
2280.127	2279.9333	-0.1937	-85	91	108	KGWIPCLEFELEHGFVYR			Carbamidomethyl (C)[6]		Mascot
2280.127	2279.9333	-0.1937	-85	91	108	KGWIPCLEFELEHGFVYR	83	100	Carbamidomethyl (C)[6]		Mascot

Rank	Protein Name	Accession No	Protein MW	Protein PI	Pep. Count	Protein Score	Protein Score C. I. %	Total Ion Score	Total Ion C. I. %
2	Ribulose bisphosphate carboxylase small chain, chloroplastic OS=Glycine tomentella GN=RBCS PE=3 SV=	RBS_GLYTO	19936	8.87	10	462	100	389	100

Peptide Information

Calc. Mass	Obsrv. Mass	± da	± ppm	Start Seq.	End Seq.	Sequence	Ion Score	C. I. %	Modification	Rank	Result Type
866.4189	866.3524	-0.0665	-77	30	37	SMAGFPTR					Mascot
882.4138	882.4923	0.0785	89	30	37	SMAGFPTR			Oxidation (M)[2]		Mascot
914.4002	914.3317	-0.0685	-75	113	120	SPGYYDGR					Mascot
914.4002	914.3317	-0.0685	-75	113	120	SPGYYDGR	48	99.858			Mascot
921.504	921.4318	-0.0722	-78	84	90	EVEYLLR					Mascot
930.4178	930.3439	-0.0739	-79	121	126	YWTMWK			Oxidation (M)[4]		Mascot
933.5151	933.4431	-0.072	-77	156	163	IIGFDNVR	41	99.279			Mascot
933.5151	933.4431	-0.072	-77	156	163	IIGFDNVR					Mascot
1038.5367	1038.4554	-0.0813	-78	147	155	TAYPNGFIR					Mascot
1038.5367	1038.4554	-0.0813	-78	147	155	TAYPNGFIR	44	99.658			Mascot
1938.9695	1938.8055	-0.164	-85	67	83	FETLSYLPDLDDAQLAK					Mascot
2067.0645	2066.8894	-0.1751	-85	66	83	KFETLSYLPDLDDAQLAK					Mascot
2067.0645	2066.8894	-0.1751	-85	66	83	KFETLSYLPDLDDAQLAK	106	100			Mascot
2152.032	2151.8508	-0.1812	-84	92	108	GWIPCLEFELEHGFVYR			Carbamidomethyl (C)[5]		Mascot
2152.032	2151.8508	-0.1812	-84	92	108	GWIPCLEFELEHGFVYR	68	99.998	Carbamidomethyl (C)[5]		Mascot
2280.127	2279.9333	-0.1937	-85	91	108	KGWIPCLEFELEHGFVYR			Carbamidomethyl (C)[6]		Mascot
2280.127	2279.9333	-0.1937	-85	91	108	KGWIPCLEFELEHGFVYR	83	100	Carbamidomethyl (C)[6]		Mascot

Rank	Protein Name	Accession No	Protein MW	Protein PI	Pep. Count	Protein Score	Protein Score C. I. %	Total Ion Score	Total Ion C. I. %
3	Ribulose bisphosphate carboxylase small chain, chloroplastic OS=Glycine tabacina GN=RBCS PE=3 SV=1	RBS_GLYTA	20005	8.87	9	404	100	344	100

Peptide Information

Calc. Mass	Obsrv. Mass	± da	± ppm	Start Seq.	End Seq.	Sequence	Ion Score	C. I. %	Modification	Rank	Result Type
866.4189	866.3524	-0.0665	-77	30	37	SMAGFPTR					Mascot
882.4138	882.4923	0.0785	89	30	37	SMAGFPTR			Oxidation (M)[2]		Mascot
914.4002	914.3317	-0.0685	-75	113	120	SPGYYDGR					Mascot
914.4002	914.3317	-0.0685	-75	113	120	SPGYYDGR	48	99.858			Mascot
921.504	921.4318	-0.0722	-78	84	90	EVEYLLR					Mascot
930.4178	930.3439	-0.0739	-79	121	126	YWTMWK			Oxidation (M)[4]		Mascot
933.5151	933.4431	-0.072	-77	156	163	IIGFDNVR	41	99.279			Mascot
933.5151	933.4431	-0.072	-77	156	163	IIGFDNVR					Mascot
1938.9695	1938.8055	-0.164	-85	67	83	FETLSYLPDLDDAQLAK					Mascot
2067.0645	2066.8894	-0.1751	-85	66	83	KFETLSYLPDLDDAQLAK					Mascot
2067.0645	2066.8894	-0.1751	-85	66	83	KFETLSYLPDLDDAQLAK	106	100			Mascot
2152.032	2151.8508	-0.1812	-84	92	108	GWIPCLEFELEHGFVYR			Carbamidomethyl (C)[5]		Mascot
2152.032	2151.8508	-0.1812	-84	92	108	GWIPCLEFELEHGFVYR	68	99.998	Carbamidomethyl (C)[5]		Mascot
2280.127	2279.9333	-0.1937	-85	91	108	KGWIPCLEFELEHGFVYR			Carbamidomethyl (C)[6]		Mascot
2280.127	2279.9333	-0.1937	-85	91	108	KGWIPCLEFELEHGFVYR	83	100	Carbamidomethyl (C)[6]		Mascot

Rank	Protein Name	Accession No	Protein MW	Protein PI	Pep. Count	Protein Score	Protein Score C. I. %	Total Ion Score	Total Ion C. I. %
4	Ribulose bisphosphate carboxylase small chain chloroplastic OS=Glycine max GN=RBCS-4 PE=3 SV=1	RBS4_SOYBN	20004	8.87	8	403	100	353	100

Peptide Information

Calc. Mass	Obsrv. Mass	± da	± ppm	Start Seq.	End Seq.	Sequence	Ion Score	C. I. %	Modification	Rank	Result Type
914.4229	914.3317	-0.0912	-100	121	126	YWTMWK					Mascot
914.4229	914.3317	-0.0912	-100	121	126	YWTMWK	11	0			Mascot
921.504	921.4318	-0.0722	-78	84	90	EVEYLLR					Mascot
930.4178	930.3439	-0.0739	-79	121	126	YWTMWK			Oxidation (M)[4]		Mascot
933.5151	933.4431	-0.072	-77	156	163	IIGFDNVR	41	99.279			Mascot
933.5151	933.4431	-0.072	-77	156	163	IIGFDNVR					Mascot
1038.5367	1038.4554	-0.0813	-78	147	155	TAYPNGFIR					Mascot
1038.5367	1038.4554	-0.0813	-78	147	155	TAYPNGFIR	44	99.658			Mascot
1938.9695	1938.8055	-0.164	-85	67	83	FETLSYLPDLDDAQLAK					Mascot
2067.0645	2066.8894	-0.1751	-85	66	83	KFETLSYLPDLDDAQLAK					Mascot
2067.0645	2066.8894	-0.1751	-85	66	83	KFETLSYLPDLDDAQLAK	106	100			Mascot
2152.032	2151.8508	-0.1812	-84	92	108	GWIPCLEFELEHGFVYR			Carbamidomethyl (C)[5]		Mascot
2152.032	2151.8508	-0.1812	-84	92	108	GWIPCLEFELEHGFVYR	68	99.998	Carbamidomethyl (C)[5]		Mascot
2280.127	2279.9333	-0.1937	-85	91	108	KGWIPCLEFELEHGFVYR			Carbamidomethyl (C)[6]		Mascot
2280.127	2279.9333	-0.1937	-85	91	108	KGWIPCLEFELEHGFVYR	83	100	Carbamidomethyl (C)[6]		Mascot

Rank	Protein Name	Accession No	Protein MW	Protein PI	Pep. Count	Protein Score	Protein Score C. I. %	Total Ion Score	Total Ion C. I. %
5	Ribulose bisphosphate carboxylase small chain chloroplastic OS=Flaveria pringlei GN=RBCS6 PE=2 S	RBS6_FLAPR	19694.8	9.07	6	178	100	148	100

Peptide Information

Calc. Mass	Obsrv. Mass	± da	± ppm	Start Seq.	End Seq.	Sequence	Ion Score	C. I. %	Modification	Rank	Result Type
914.4002	914.3317	-0.0685	-75	108	115	SPGYYDGR					Mascot
914.4002	914.3317	-0.0685	-75	108	115	SPGYYDGR	48	99.858			Mascot
930.4178	930.3439	-0.0739	-79	116	121	YWTMWK			Oxidation (M)[4]		Mascot
933.5151	933.4431	-0.072	-77	151	158	IIGFDNVR	41	99.279			Mascot
933.5151	933.4431	-0.072	-77	151	158	IIGFDNVR					Mascot
1491.755	1491.6145	-0.1405	-94	34	47	KVNDFSTLPSNGGR					Mascot
1754.8418	1754.7472	-0.0946	-54	159	173	QVQCISFIASKPDXF	38	98.427	Carbamidomethyl (C)[4]		Mascot
2067.1121	2066.8894	-0.2227	-108	61	78	KYXTLSYLPNLTEAQLAK	22	42.75			Mascot

Rank	Protein Name	Accession No	Protein MW	Protein PI	Pep. Count	Protein Score	Protein Score C. I. %	Total Ion Score	Total Ion C. I. %
6	Ribulose bisphosphate carboxylase small chain chloroplastic OS=Petunia hybrida GN=RBCS PE=3 S	RBS1_PETHY	20357.2	8.29	5	162	100	133	100

Peptide Information

Calc. Mass	Obsrv. Mass	± da	± ppm	Start Seq.	End Seq.	Sequence	Ion Score	C. I. %	Modification	Rank	Result Type
914.4229	914.3317	-0.0912	-100	123	128	YWTMWK					Mascot
914.4229	914.3317	-0.0912	-100	123	128	YWTMWK	11	0			Mascot
930.4178	930.3439	-0.0739	-79	123	128	YWTMWK			Oxidation (M)[4]		Mascot
933.5151	933.4431	-0.072	-77	158	165	IIGFDNVR	41	99.279			Mascot
933.5151	933.4431	-0.072	-77	158	165	IIGFDNVR					Mascot
1021.5564	1021.4346	-0.1218	-119	86	93	EIEYLLNK					Mascot
1663.8472	1663.7413	-0.1059	-64	15	30	TNVAQASMVAPFNGLK			Oxidation (M)[8]		Mascot
1754.8934	1754.7472	-0.1462	-83	166	180	QVQCISFIAYKPPGF			Carbamidomethyl (C)[4]		Mascot
1754.8934	1754.7472	-0.1462	-83	166	180	QVQCISFIAYKPPGF	81	100	Carbamidomethyl (C)[4]		Mascot

Rank	Protein Name	Accession No	Protein MW	Protein PI	Pep. Count	Protein Score	Protein Score C. I. %	Total Ion Score	Total Ion C. I. %
7	Ribulose bisphosphate carboxylase small chain, chloroplastic OS=Helianthus annuus GN=RBCS PE=2 SV=1	RBS_HELAN	20198.1	9.07	4	153	100	138	100

Peptide Information

Calc. Mass	Obsrv. Mass	± da	± ppm	Start Seq.	End Seq.	Sequence	Ion Score	C. I. %	Modification	Rank	Result Type
914.4002	914.3317	-0.0685	-75	113	120	SPGYYDGR					Mascot
914.4002	914.3317	-0.0685	-75	113	120	SPGYYDGR	48	99.858			Mascot
930.4178	930.3439	-0.0739	-79	121	126	YWTMWK			Oxidation (M)[4]		Mascot
933.5151	933.4431	-0.072	-77	156	163	IIGFDNVR	41	99.279			Mascot
933.5151	933.4431	-0.072	-77	156	163	IIGFDNVR					Mascot
2152.0684	2151.8508	-0.2176	-101	92	108	KWVPCLEFELEHGFVYR					Mascot
2152.0684	2151.8508	-0.2176	-101	92	108	KWVPCLEFELEHGFVYR	51	99.916			Mascot

Rank	Protein Name	Accession No	Protein MW	Protein PI	Pep. Count	Protein Score	Protein Score C. I. %	Total Ion Score	Total Ion C. I. %
8	Ribulose bisphosphate carboxylase small chain, chloroplastic OS=Nicotiana tabacum GN=RBCS PE=1 SV=1	RBS_TOBAC	20298.1	7.6	7	137	100	88	100

Peptide Information

Calc. Mass	Obsrv. Mass	± da	± ppm	Start Seq.	End Seq.	Sequence	Ion Score	C. I. %	Modification	Rank	Result Type
914.4002	914.3317	-0.0685	-75	115	122	SPGYYDGR					Mascot
	914.3317										Mascot
914.4002	914.3317	-0.0685	-75	115	122	SPGYYDGR	48	99.858			Mascot
921.4788	921.4318	-0.047	-51	31	39	SAASFPVSR					Mascot
930.4178	930.3439	-0.0739	-79	123	128	YWTMWK			Oxidation (M)[4]		Mascot
933.5151	933.4431	-0.072	-77	158	165	IIGFDNVR	41	99.279			Mascot
933.5151	933.4431	-0.072	-77	158	165	IIGFDNVR					Mascot
1663.8472	1663.7413	-0.1059	-64	15	30	SNVAQANMVAPFTGLK			Oxidation (M)[8]		Mascot
2152.03	2151.8508	-0.1792	-83	129	148	LPMFGCTDATQVLAEVE EAK					Mascot
2152.03	2151.8508	-0.1792	-83	129	148	LPMFGCTDATQVLAEVE EAK					Mascot
2168.0249	2167.8442	-0.1807	-83	129	148	LPMFGCTDATQVLAEVE EAK			Oxidation (M)[3]		Mascot
2225.0464	2224.9336	-0.1128	-51	129	148	LPMFGCTDATQVLAEVE EAK			Carbamidomethyl (C)[6], Oxidation (M)[3]		Mascot
2280.125	2279.9333	-0.1917	-84	129	149	LPMFGCTDATQVLAEVE EAKK					Mascot
2280.125	2279.9333	-0.1917	-84	129	149	LPMFGCTDATQVLAEVE EAKK					Mascot
2296.1199	2295.917	-0.2029	-88	129	149	LPMFGCTDATQVLAEVE EAKK			Oxidation (M)[3]		Mascot

Rank	Protein Name	Accession No	Protein MW	Protein PI	Pep. Count	Protein Score	Protein Score C. I. %	Total Ion Score	Total Ion C. I. %
9	Ribulose bisphosphate carboxylase small chain chloroplastic OS=Solanum tuberosum GN=RBCS-2A PE=	RBSA_SOLTU	20276	7.6	6	124	100	88	100

Peptide Information

Calc. Mass	Obsrv. Mass	± da	± ppm	Start Seq.	End Seq.	Sequence	Ion Score	C. I. %	Modification	Rank	Result Type
914.4002	914.3317	-0.0685	-75	115	122	SPGYYDGR					Mascot
914.4002	914.3317	-0.0685	-75	115	122	SPGYYDGR	48	99.858			Mascot
921.4788	921.4318	-0.047	-51	31	39	SAASFPVSR					Mascot
930.4178	930.3439	-0.0739	-79	123	128	YWTMWK			Oxidation (M)[4]		Mascot
933.5151	933.4431	-0.072	-77	158	165	IIGFDNVR	41	99.279			Mascot
933.5151	933.4431	-0.072	-77	158	165	IIGFDNVR					Mascot
2152.03	2151.8508	-0.1792	-83	129	148	LPMFGCTDATQVLAEVE EAK					Mascot
2152.03	2151.8508	-0.1792	-83	129	148	LPMFGCTDATQVLAEVE EAK					Mascot
2168.0249	2167.8442	-0.1807	-83	129	148	LPMFGCTDATQVLAEVE EAK			Oxidation (M)[3]		Mascot
2225.0464	2224.9336	-0.1128	-51	129	148	LPMFGCTDATQVLAEVE EAK			Carbamidomethyl (C)[6], Oxidation (M)[3]		Mascot
2280.125	2279.9333	-0.1917	-84	129	149	LPMFGCTDATQVLAEVE EAKK					Mascot
2280.125	2279.9333	-0.1917	-84	129	149	LPMFGCTDATQVLAEVE EAKK					Mascot
2296.1199	2295.917	-0.2029	-88	129	149	LPMFGCTDATQVLAEVE EAKK			Oxidation (M)[3]		Mascot

Rank	Protein Name	Accession No	Protein MW	Protein PI	Pep. Count	Protein Score	Protein Score C. I. %	Total Ion Score	Total Ion C. I. %
10	Ribulose bisphosphate carboxylase small chain chloroplastic OS=Nicotiana sylvestris PE=3 SV=1	RBS2_NICSY	20461.2	6.59	5	118	100	88	100

Peptide Information

Calc. Mass	Obsrv. Mass	± da	± ppm	Start Seq.	End Seq.	Sequence	Ion Score	C. I. %	Modification	Rank	Result Type
914.4002	914.3317	-0.0685	-75	116	123	SPGYYDGR					Mascot
914.4002	914.3317	-0.0685	-75	116	123	SPGYYDGR	48	99.849			Mascot
930.4178	930.3439	-0.0739	-79	124	129	YWTMWK			Oxidation (M)[4]		Mascot
933.5151	933.4431	-0.072	-77	159	166	IIGFDNVR	41	99.237			Mascot
933.5151	933.4431	-0.072	-77	159	166	IIGFDNVR					Mascot
2152.03	2151.8508	-0.1792	-83	130	149	LPMFGCTDATQVLAEVE EAK					Mascot
2152.03	2151.8508	-0.1792	-83	130	149	LPMFGCTDATQVLAEVE EAK					Mascot
2168.0249	2167.8442	-0.1807	-83	130	149	LPMFGCTDATQVLAEVE EAK			Oxidation (M)[3]		Mascot
2225.0464	2224.9336	-0.1128	-51	130	149	LPMFGCTDATQVLAEVE EAK			Carbamidomethyl (C)[6], Oxidation (M)[3]		Mascot
2280.125	2279.9333	-0.1917	-84	130	150	LPMFGCTDATQVLAEVE EAKK					Mascot
2280.125	2279.9333	-0.1917	-84	130	150	LPMFGCTDATQVLAEVE EAKK					Mascot
2296.1199	2295.917	-0.2029	-88	130	150	LPMFGCTDATQVLAEVE EAKK			Oxidation (M)[3]		Mascot

Gel Idx/Pos	118/E17	Instr./Gel Origin	BA2060/131017A	Process Sta	Analysis Succeeded
Plate [#] Name	[1] 31496	Instrument Sample Name		Spectra	8

Rank	Protein Name	Accession No	Protein MW	Protein PI	Pep. Count	Protein Score	Protein Score C. I. %	Total Ion Score	Total Ion C. I. %
1	Ribulose bisphosphate carboxylase small chain chloroplastic OS=Glycine max GN=RBCS-1 PE=3 SV=1	RBS1_SOYBN	20060	8.87	11	478	100	398	100

Peptide Information

Calc. Mass	Obsrv. Mass	± da	± ppm	Start Seq.	End Seq.	Sequence	Ion Score	C. I. %	Modification	Rank	Result Type
866.4189	866.3678	-0.0511	-59	30	37	SMAGFPTR					Mascot
914.4002	914.3453	-0.0549	-60	113	120	SPXYYDGR	66	99.997			Mascot
914.4229	914.3453	-0.0776	-85	121	126	YWTMWK					Mascot
921.504	921.4462	-0.0578	-63	84	90	EVEYLLR					Mascot
930.4178	930.3547	-0.0631	-68	121	126	YWTMWK			Oxidation (M)[4]		Mascot
933.5151	933.4564	-0.0587	-63	156	163	IIGFDNVR	53	99.949			Mascot
933.5151	933.4564	-0.0587	-63	156	163	IIGFDNVR					Mascot
1038.5367	1038.4706	-0.0661	-64	147	155	TAYPNGFIR					Mascot
1038.5367	1038.4706	-0.0661	-64	147	155	TAYPNGFIR	44	99.611			Mascot
1754.8934	1754.7635	-0.1299	-74	164	178	QVQCISFIAYKPPGF			Carbamidomethyl (C)[4]		Mascot
1938.9695	1938.8278	-0.1417	-73	67	83	FETLSYLPDLDDAQLAK	117	100			Mascot
1938.9695	1938.8278	-0.1417	-73	67	83	FETLSYLPDLDDAQLAK					Mascot
2067.0513	2066.9131	-0.1382	-67	17	37	AGAGMVAPFTGLKSMAG FPTR					Mascot
2067.0645	2066.9131	-0.1514	-73	66	83	KFETLSYLPDLDDAQLAK	124	100			Mascot
2152.032	2151.8645	-0.1675	-78	92	108	GWIPCLEFELEHGFVYR			Carbamidomethyl (C)[5]		Mascot

Rank	Protein Name	Accession No	Protein MW	Protein PI	Pep. Count	Protein Score	Protein Score C. I. %	Total Ion Score	Total Ion C. I. %
2	Ribulose bisphosphate carboxylase small chain, chloroplastic OS=Glycine tomentella GN=RBCS PE=3 SV=	RBS_GLYTO	19936	8.87	9	463	100	402	100

Peptide Information

Calc. Mass	Obsrv. Mass	± da	± ppm	Start Seq.	End Seq.	Sequence	Ion Score	C. I. %	Modification	Rank	Result Type
866.4189	866.3678	-0.0511	-59	30	37	SMAGFPTR					Mascot
914.4002	914.3453	-0.0549	-60	113	120	SPGYYDGR					Mascot
914.4002	914.3453	-0.0549	-60	113	120	SPGYYDGR	66	99.997			Mascot
921.504	921.4462	-0.0578	-63	84	90	EVEYLLR					Mascot
930.4178	930.3547	-0.0631	-68	121	126	YWTMWK			Oxidation (M)[4]		Mascot
933.5151	933.4564	-0.0587	-63	156	163	IIGFDNVR	53	99.949			Mascot
933.5151	933.4564	-0.0587	-63	156	163	IIGFDNVR					Mascot
1038.5367	1038.4706	-0.0661	-64	147	155	TAYPNGFIR					Mascot
1038.5367	1038.4706	-0.0661	-64	147	155	TAYPNGFIR	44	99.611			Mascot
1938.9695	1938.8278	-0.1417	-73	67	83	FETLSYLPDLDDAQLAK	117	100			Mascot
1938.9695	1938.8278	-0.1417	-73	67	83	FETLSYLPDLDDAQLAK					Mascot
2067.0645	2066.9131	-0.1514	-73	66	83	KFETLSYLPDLDDAQLAK	124	100			Mascot
2067.0645	2066.9131	-0.1514	-73	66	83	KFETLSYLPDLDDAQLAK					Mascot
2152.032	2151.8645	-0.1675	-78	92	108	GWIPCLEFELEHGFVYR			Carbamidomethyl (C)[5]		Mascot

Rank	Protein Name	Accession No	Protein MW	Protein PI	Pep. Count	Protein Score	Protein Score C. I. %	Total Ion Score	Total Ion C. I. %
3	Ribulose bisphosphate carboxylase small chain, chloroplastic OS=Glycine tabacina GN=RBCS PE=3 SV=1	RBS_GLYTA	20005	8.87	8	407	100	357	100

Peptide Information

Calc. Mass	Obsrv. Mass	± da	± ppm	Start Seq.	End Seq.	Sequence	Ion Score	C. I. %	Modification	Rank	Result Type
866.4189	866.3678	-0.0511	-59	30	37	SMAGFPTR					Mascot
914.4002	914.3453	-0.0549	-60	113	120	SPGYYDGR					Mascot
914.4002	914.3453	-0.0549	-60	113	120	SPGYYDGR	66	99.997			Mascot
921.504	921.4462	-0.0578	-63	84	90	EVEYLLR					Mascot
930.4178	930.3547	-0.0631	-68	121	126	YWTMWK			Oxidation (M)[4]		Mascot
933.5151	933.4564	-0.0587	-63	156	163	IIGFDNVR	53	99.949			Mascot
933.5151	933.4564	-0.0587	-63	156	163	IIGFDNVR					Mascot
1938.9695	1938.8278	-0.1417	-73	67	83	FETLSYLPDLDDAQLAK	117	100			Mascot
1938.9695	1938.8278	-0.1417	-73	67	83	FETLSYLPDLDDAQLAK					Mascot
2067.0645	2066.9131	-0.1514	-73	66	83	KFETLSYLPDLDDAQLAK	124	100			Mascot
2067.0645	2066.9131	-0.1514	-73	66	83	KFETLSYLPDLDDAQLAK					Mascot
2152.032	2151.8645	-0.1675	-78	92	108	GWIPCLEFELEHGFVYR			Carbamidomethyl (C)[5]		Mascot

Rank	Protein Name	Accession No	Protein MW	Protein PI	Pep. Count	Protein Score	Protein Score C. I. %	Total Ion Score	Total Ion C. I. %
4	Ribulose bisphosphate carboxylase small chain chloroplastic OS=Glycine max GN=RBCS-4 PE=3 SV=1	RBS4_SOYBN	20004	8.87	7	381	100	337	100

Peptide Information

Calc. Mass	Obsrv. Mass	± da	± ppm	Start Seq.	End Seq.	Sequence	Ion Score	C. I. %	Modification	Rank	Result Type
914.4229	914.3453	-0.0776	-85	121	126	YWTMWK					Mascot
914.4229	914.3453	-0.0776	-85	121	126	YWTMWK	3	0			Mascot
921.504	921.4462	-0.0578	-63	84	90	EVEYLLR					Mascot
930.4178	930.3547	-0.0631	-68	121	126	YWTMWK			Oxidation (M)[4]		Mascot
933.5151	933.4564	-0.0587	-63	156	163	IIGFDNVR	53	99.949			Mascot
933.5151	933.4564	-0.0587	-63	156	163	IIGFDNVR					Mascot
1038.5367	1038.4706	-0.0661	-64	147	155	TAYPNGFIR					Mascot
1038.5367	1038.4706	-0.0661	-64	147	155	TAYPNGFIR	44	99.611			Mascot
1938.9695	1938.8278	-0.1417	-73	67	83	FETLSYLPDLDDAQLAK	117	100			Mascot
1938.9695	1938.8278	-0.1417	-73	67	83	FETLSYLPDLDDAQLAK					Mascot
2067.0645	2066.9131	-0.1514	-73	66	83	KFETLSYLPDLDDAQLAK	124	100			Mascot
2067.0645	2066.9131	-0.1514	-73	66	83	KFETLSYLPDLDDAQLAK					Mascot
2152.032	2151.8645	-0.1675	-78	92	108	GWIPCLEFELEHGFVYR			Carbamidomethyl (C)[5]		Mascot

Rank	Protein Name	Accession No	Protein MW	Protein PI	Pep. Count	Protein Score	Protein Score C. I. %	Total Ion Score	Total Ion C. I. %
5	Ribulose bisphosphate carboxylase small chain chloroplastic OS=Flaveria pringlei GN=RBCS6 PE=2 S	RBS6_FLAPR	19694.8	9.07	6	188	100	158	100

Peptide Information

Calc. Mass	Obsrv. Mass	± da	± ppm	Start Seq.	End Seq.	Sequence	Ion Score	C. I. %	Modification	Rank	Result Type
914.4002	914.3453	-0.0549	-60	108	115	SPGYYDGR					Mascot
914.4002	914.3453	-0.0549	-60	108	115	SPGYYDGR	66	99.997			Mascot
930.4178	930.3547	-0.0631	-68	116	121	YWTMWK			Oxidation (M)[4]		Mascot
933.5151	933.4564	-0.0587	-63	151	158	IIGFDNVR	53	99.949			Mascot
933.5151	933.4564	-0.0587	-63	151	158	IIGFDNVR					Mascot
1939.0171	1938.8278	-0.1893	-98	62	78	YXTLSYLPNLTEAQLAK	12	0			Mascot
1977.0341	1976.7773	-0.2568	-130	143	158	EYPQAWIRIIGFDNVR					Mascot
2067.1121	2066.9131	-0.199	-96	61	78	KYXTLSYLPNLTEAQLAK	29	86.523			Mascot

Rank	Protein Name	Accession No	Protein MW	Protein PI	Pep. Count	Protein Score	Protein Score C. I. %	Total Ion Score	Total Ion C. I. %
6	Ribulose bisphosphate carboxylase small chain, chloroplastic OS=Nicotiana tabacum GN=RBCS PE=1 SV=1	RBS_TOBAC	20298.1	7.6	5	146	100	117	100

Peptide Information

Calc. Mass	Obsrv. Mass	± da	± ppm	Start Seq.	End Seq.	Sequence	Ion Score	C. I. %	Modification	Rank	Result Type
914.4002	914.3453	-0.0549	-60	115	122	SPGYYDGR					Mascot
914.4002	914.3453	-0.0549	-60	115	122	SPGYYDGR	66	99.997			Mascot
921.4788	921.4462	-0.0326	-35	31	39	SAASFPVSR					Mascot
930.4178	930.3547	-0.0631	-68	123	128	YWTMWK			Oxidation (M)[4]		Mascot
933.5151	933.4564	-0.0587	-63	158	165	IIGFDNVR	53	99.949			Mascot
933.5151	933.4564	-0.0587	-63	158	165	IIGFDNVR					Mascot
2152.03	2151.8645	-0.1655	-77	129	148	LPMFGCTDATQVLAEVE EAK					Mascot
2168.0249	2167.8684	-0.1565	-72	129	148	LPMFGCTDATQVLAEVE EAK			Oxidation (M)[3]		Mascot

Rank	Protein Name	Accession No	Protein MW	Protein PI	Pep. Count	Protein Score	Protein Score C. I. %	Total Ion Score	Total Ion C. I. %
7	Ribulose bisphosphate carboxylase small chain chloroplastic OS=Solanum tuberosum GN=RBCS-2A PE=	RBSA_SOLTU	20276	7.6	5	143	100	117	100

Peptide Information

Calc. Mass	Obsrv. Mass	± da	± ppm	Start Seq.	End Seq.	Sequence	Ion Score	C. I. %	Modification	Rank	Result Type
914.4002	914.3453	-0.0549	-60	115	122	SPGYYDGR					Mascot
914.4002	914.3453	-0.0549	-60	115	122	SPGYYDGR	66	99.997			Mascot
921.4788	921.4462	-0.0326	-35	31	39	SAASFPVSR					Mascot
930.4178	930.3547	-0.0631	-68	123	128	YWTMWK			Oxidation (M)[4]		Mascot
933.5151	933.4564	-0.0587	-63	158	165	IIGFDNVR	53	99.949			Mascot
933.5151	933.4564	-0.0587	-63	158	165	IIGFDNVR					Mascot
2152.03	2151.8645	-0.1655	-77	129	148	LPMFGCTDATQVLAEVE EAK					Mascot
2168.0249	2167.8684	-0.1565	-72	129	148	LPMFGCTDATQVLAEVE EAK			Oxidation (M)[3]		Mascot

Rank	Protein Name	Accession No	Protein MW	Protein PI	Pep. Count	Protein Score	Protein Score C. I. %	Total Ion Score	Total Ion C. I. %
8	Ribulose bisphosphate carboxylase small chain chloroplastic OS=Nicotiana sylvestris PE=3 SV=1	RBS2_NICSY	20461.2	6.59	4	138	100	117	100

Peptide Information

Calc. Mass	Obsrv. Mass	± da	± ppm	Start Seq.	End Seq.	Sequence	Ion Score	C. I. %	Modification	Rank	Result Type
914.4002	914.3453	-0.0549	-60	116	123	SPGYYDGR					Mascot
914.4002	914.3453	-0.0549	-60	116	123	SPGYYDGR	66	99.997			Mascot Mascot
930.4178	930.3547	-0.0631	-68	124	129	YWTMWK			Oxidation (M)[4]		Mascot
933.5151	933.4564	-0.0587	-63	159	166	IIGFDNVR	53	99.949			Mascot
933.5151	933.4564	-0.0587	-63	159	166	IIGFDNVR					Mascot
2152.03	2151.8645	-0.1655	-77	130	149	LPMFGCTDATQVLAEVE EAK					Mascot
2168.0249	2167.8684	-0.1565	-72	130	149	LPMFGCTDATQVLAEVE EAK			Oxidation (M)[3]		Mascot

Rank	Protein Name	Accession No	Protein MW	Protein PI	Pep. Count	Protein Score	Protein Score C. I. %	Total Ion Score	Total Ion C. I. %
9	Ribulose bisphosphate carboxylase small chain, chloroplastic OS=Helianthus annuus GN=RBCS PE=2 SV=1	RBS_HELAN	20198.1	9.07	5	138	100	117	100

Peptide Information

Calc. Mass	Obsrv. Mass	± da	± ppm	Start Seq.	End Seq.	Sequence	Ion Score	C. I. %	Modification	Rank	Result Type
914.4002	914.3453	-0.0549	-60	113	120	SPGYYDGR					Mascot
914.4002	914.3453	-0.0549	-60	113	120	SPGYYDGR	66	99.997			Mascot
930.4178	930.3547	-0.0631	-68	121	126	YWTMWK			Oxidation (M)[4]		Mascot
933.5151	933.4564	-0.0587	-63	156	163	IIGFDNVR	53	99.949			Mascot
933.5151	933.4564	-0.0587	-63	156	163	IIGFDNVR					Mascot
1977.0341	1976.7773	-0.2568	-130	148	163	EYPQAWIRIIGFDNVR					Mascot
2152.0684	2151.8645	-0.2039	-95	92	108	KWVPCLEFELEHGFVYR					Mascot

Rank	Protein Name	Accession No	Protein MW	Protein PI	Pep. Count	Protein Score	Protein Score C. I. %	Total Ion Score	Total Ion C. I. %
10	Ribulose bisphosphate carboxylase small chain chloroplastic OS=Pisum sativum GN=RBCS-3A PE=1 SV	RBS3_PEA	20218.3	9.1	5	138	100	117	100

Peptide Information

Calc. Mass	Obsrv. Mass	± da	± ppm	Start Seq.	End Seq.	Sequence	Ion Score	C. I. %	Modification	Rank	Result Type
866.444	866.3678	-0.0762	-88	32	39	SMTGFPVK					Mascot
914.4002	914.3453	-0.0549	-60	115	122	SPGYYDGR					Mascot
914.4002	914.3453	-0.0549	-60	115	122	SPGYYDGR	66	99.997			Mascot
921.504	921.4462	-0.0578	-63	86	92	EVEYLLR					Mascot
930.4178	930.3547	-0.0631	-68	123	128	YWTMWK			Oxidation (M)[4]		Mascot
933.5151	933.4564	-0.0587	-63	158	165	IIGFDNVR	53	99.949			Mascot
933.5151	933.4564	-0.0587	-63	158	165	IIGFDNVR					Mascot

Gel Idx/Pos	119/E18	Instr./Gel Origin	BA2060/131017A	Process Sta	Analysis Succeeded
Plate [#] Name	[1] 31496	Instrument Sample Name		Spectra	8

Rank	Protein Name	Accession No	Protein MW	Protein PI	Pep. Count	Protein Score	Protein Score C. I. %	Total Ion Score	Total Ion C. I. %
1	Ribulose bisphosphate carboxylase small chain chloroplastic OS=Glycine max GN=RBCS-1 PE=3 SV=1	RBS1_SOYBN	20060	8.87	13	520	100	415	100

Peptide Information

Calc. Mass	Obsrv. Mass	± da	± ppm	Start Seq.	End Seq.	Sequence	Ion Score	C. I. %	Modification	Rank	Result Type
882.4138	882.5139	0.1001	113	30	37	SMAGFPTR			Oxidation (M)[2]		Mascot
914.4002	914.3481	-0.0521	-57	113	120	SPXYYDGR	53	99.943			Mascot
914.4229	914.3481	-0.0748	-82	121	126	YWTMWK					Mascot
921.504	921.4506	-0.0534	-58	84	90	EVEYLLR					Mascot
930.4178	930.3611	-0.0567	-61	121	126	YWTMWK			Oxidation (M)[4]		Mascot
933.5151	933.4612	-0.0539	-58	156	163	IIGFDNVR	45	99.693			Mascot
933.5151	933.4612	-0.0539	-58	156	163	IIGFDNVR					Mascot
1038.5367	1038.4755	-0.0612	-59	147	155	TAYPNGFIR					Mascot
1038.5367	1038.4755	-0.0612	-59	147	155	TAYPNGFIR	41	99.226			Mascot
1566.7655	1566.6604	-0.1051	-67	127	140	LPMFGCTDASQVLK	100	100	Carbamidomethyl (C)[6]		Mascot
1566.7655	1566.6604	-0.1051	-67	127	140	LPMFGCTDASQVLK			Carbamidomethyl (C)[6]		Mascot
1582.7604	1582.6575	-0.1029	-65	127	140	LPMFGCTDASQVLK			Carbamidomethyl (C)[6], Oxidation (M)[3]		Mascot
1754.8934	1754.7727	-0.1207	-69	164	178	QVQCISFIAYKPPGF	75	100	Carbamidomethyl (C)[4]		Mascot
1754.8934	1754.7727	-0.1207	-69	164	178	QVQCISFIAYKPPGF			Carbamidomethyl (C)[4]		Mascot
1938.9695	1938.833	-0.1365	-70	67	83	FETLSYLPDLDDAQLAK					Mascot
2067.0513	2066.9192	-0.1321	-64	17	37	AGAGMVAPFTGLKSMAG FPTR					Mascot
2067.0645	2066.9192	-0.1453	-70	66	83	KFETLSYLPDLDDAQLAK	104	100			Mascot
2152.032	2151.8818	-0.1502	-70	92	108	GWIPCLEFELEHGFVYR			Carbamidomethyl (C)[5]		Mascot
2223.1055	2222.936	-0.1695	-76	91	108	KGWIPCLEFELEHGFVYR					Mascot
2280.127	2279.9531	-0.1739	-76	91	108	KGWIPCLEFELEHGFVYR			Carbamidomethyl (C)[6]		Mascot

Rank	Protein Name	Accession No	Protein MW	Protein PI	Pep. Count	Protein Score	Protein Score C. I. %	Total Ion Score	Total Ion C. I. %
2	Ribulose bisphosphate carboxylase small chain, chloroplastic OS=Glycine tabacina GN=RBCS PE=3 SV=1	RBS_GLYTA	20005	8.87	10	371	100	300	100

Peptide Information

Calc. Mass	Obsrv. Mass	± da	± ppm	Start Seq.	End Seq.	Sequence	Ion Score	C. I. %	Modification	Rank	Result Type
882.4138	882.5139	0.1001	113	30	37	SMAGFPTR			Oxidation (M)[2]		Mascot
914.4002	914.3481	-0.0521	-57	113	120	SPGYYDGR	53	99.943			Mascot
914.4002	914.3481	-0.0521	-57	113	120	SPGYYDGR					Mascot
921.504	921.4506	-0.0534	-58	84	90	EVEYLLR					Mascot
930.4178	930.3611	-0.0567	-61	121	126	YWTMWK			Oxidation (M)[4]		Mascot
933.5151	933.4612	-0.0539	-58	156	163	IIGFDNVR	45	99.693			Mascot
933.5151	933.4612	-0.0539	-58	156	163	IIGFDNVR					Mascot
1566.7655	1566.6604	-0.1051	-67	127	140	LPMFGCTDASQVLK	100	100	Carbamidomethyl (C)[6]		Mascot Mascot
1566.7655	1566.6604	-0.1051	-67	127	140	LPMFGCTDASQVLK			Carbamidomethyl (C)[6]		Mascot
1582.7604	1582.6575	-0.1029	-65	127	140	LPMFGCTDASQVLK			Carbamidomethyl (C)[6], Oxidation (M)[3]		Mascot
1938.9695	1938.833	-0.1365	-70	67	83	FETLSYLPDLDDAQLAK					Mascot
2067.0645	2066.9192	-0.1453	-70	66	83	KFETLSYLPDLDDAQLAK	104	100			Mascot
2067.0645	2066.9192	-0.1453	-70	66	83	KFETLSYLPDLDDAQLAK					Mascot
2152.032	2151.8818	-0.1502	-70	92	108	GWIPCLEFELEHGFVYR			Carbamidomethyl (C)[5]		Mascot
2223.1055	2222.936	-0.1695	-76	91	108	KGWIPCLEFELEHGFVYR					Mascot
2280.127	2279.9531	-0.1739	-76	91	108	KGWIPCLEFELEHGFVYR			Carbamidomethyl (C)[6]		Mascot

Rank	Protein Name	Accession No	Protein MW	Protein PI	Pep. Count	Protein Score	Protein Score C. I. %	Total Ion Score	Total Ion C. I. %
3	Ribulose bisphosphate carboxylase small chain chloroplastic OS=Glycine max GN=RBCS-4 PE=3 SV=1	RBS4_SOYBN	20004	8.87	9	359	100	298	100

Peptide Information

Calc. Mass	Obsrv. Mass	± da	± ppm	Start Seq.	End Seq.	Sequence	Ion Score	C. I. %	Modification	Rank	Result Type
914.4229	914.3481	-0.0748	-82	121	126	YWTMWK	8	0			Mascot
914.4229	914.3481	-0.0748	-82	121	126	YWTMWK					Mascot
921.504	921.4506	-0.0534	-58	84	90	EVEYLLR					Mascot
930.4178	930.3611	-0.0567	-61	121	126	YWTMWK			Oxidation (M)[4]		Mascot
933.5151	933.4612	-0.0539	-58	156	163	IIGFDNVR	45	99.693			Mascot
933.5151	933.4612	-0.0539	-58	156	163	IIGFDNVR					Mascot
1038.5367	1038.4755	-0.0612	-59	147	155	TAYPNGFIR					Mascot
1038.5367	1038.4755	-0.0612	-59	147	155	TAYPNGFIR	41	99.226			Mascot
1566.7655	1566.6604	-0.1051	-67	127	140	LPMFGCTDASQVLK	100	100	Carbamidomethyl (C)[6]		Mascot
1566.7655	1566.6604	-0.1051	-67	127	140	LPMFGCTDASQVLK			Carbamidomethyl (C)[6]		Mascot
1582.7604	1582.6575	-0.1029	-65	127	140	LPMFGCTDASQVLK			Carbamidomethyl (C)[6], Oxidation (M)[3]		Mascot
1938.9695	1938.833	-0.1365	-70	67	83	FETLSYLPDLDDAQLAK					Mascot
2067.0645	2066.9192	-0.1453	-70	66	83	KFETLSYLPDLDDAQLAK	104	100			Mascot
2067.0645	2066.9192	-0.1453	-70	66	83	KFETLSYLPDLDDAQLAK					Mascot
2152.032	2151.8818	-0.1502	-70	92	108	GWIPCLEFELEHGFVYR			Carbamidomethyl (C)[5]		Mascot
2223.1055	2222.936	-0.1695	-76	91	108	KGWIPCLEFELEHGFVYR					Mascot
2280.127	2279.9531	-0.1739	-76	91	108	KGWIPCLEFELEHGFVYR			Carbamidomethyl (C)[6]		Mascot

Rank	Protein Name	Accession No	Protein MW	Protein PI	Pep. Count	Protein Score	Protein Score C. I. %	Total Ion Score	Total Ion C. I. %
4	Ribulose bisphosphate carboxylase small chain, chloroplastic OS=Glycine tomentella GN=RBCS PE=3 SV=	RBS_GLYTO	19936	8.87	10	314	100	242	100

Peptide Information

Calc. Mass	Obsrv. Mass	± da	± ppm	Start Seq.	End Seq.	Sequence	Ion Score	C. I. %	Modification	Rank	Result Type
882.4138	882.5139	0.1001	113	30	37	SMAGFPTR			Oxidation (M)[2]		Mascot
914.4002	914.3481	-0.0521	-57	113	120	SPGYYDGR	53	99.943			Mascot
914.4002	914.3481	-0.0521	-57	113	120	SPGYYDGR					Mascot
921.504	921.4506	-0.0534	-58	84	90	EVEYLLR					Mascot
930.4178	930.3611	-0.0567	-61	121	126	YWTMWK			Oxidation (M)[4]		Mascot
933.5151	933.4612	-0.0539	-58	156	163	IIGFDNVR	45	99.693			Mascot
933.5151	933.4612	-0.0539	-58	156	163	IIGFDNVR					Mascot
1038.5367	1038.4755	-0.0612	-59	147	155	TAYPNGFIR					Mascot
1038.5367	1038.4755	-0.0612	-59	147	155	TAYPNGFIR	41	99.226			Mascot
1938.9695	1938.833	-0.1365	-70	67	83	FETLSYLPDLDDAQLAK					Mascot
2067.0645	2066.9192	-0.1453	-70	66	83	KFETLSYLPDLDDAQLAK	104	100			Mascot
2067.0645	2066.9192	-0.1453	-70	66	83	KFETLSYLPDLDDAQLAK					Mascot
2152.032	2151.8818	-0.1502	-70	92	108	GWIPCLEFELEHGFVYR			Carbamidomethyl (C)[5]		Mascot
2223.1055	2222.936	-0.1695	-76	91	108	KGWIPCLEFELEHGFVYR					Mascot
2280.127	2279.9531	-0.1739	-76	91	108	KGWIPCLEFELEHGFVYR			Carbamidomethyl (C)[6]		Mascot

Rank	Protein Name	Accession No	Protein MW	Protein PI	Pep. Count	Protein Score	Protein Score C. I. %	Total Ion Score	Total Ion C. I. %
5	Ribulose bisphosphate carboxylase small chain chloroplastic OS=Flaveria pringlei GN=RBCS6 PE=2 S	RBS6_FLAPR	19694.8	9.07	6	180	100	150	100

Peptide Information

Calc. Mass	Obsrv. Mass	± da	± ppm	Start Seq.	End Seq.	Sequence	Ion Score	C. I. %	Modification	Rank	Result Type
914.4002	914.3481	-0.0521	-57	108	115	SPGYYDGR	53	99.943			Mascot
914.4002	914.3481	-0.0521	-57	108	115	SPGYYDGR					Mascot
930.4178	930.3611	-0.0567	-61	116	121	YWTMWK			Oxidation (M)[4]		Mascot
933.5151	933.4612	-0.0539	-58	151	158	IIGFDNVR	45	99.693			Mascot
933.5151	933.4612	-0.0539	-58	151	158	IIGFDNVR					Mascot
1491.755	1491.6359	-0.1191	-80	34	47	KVNDFSTLPSNGGR					Mascot
1754.8418	1754.7727	-0.0691	-39	159	173	QVQCISFIASKPDXF	32	94.117	Carbamidomethyl (C)[4]		Mascot
2067.1121	2066.9192	-0.1929	-93	61	78	KYXTLSYLPNLTEAQLAK	21	17.277			Mascot

Rank	Protein Name	Accession No	Protein MW	Protein PI	Pep. Count	Protein Score	Protein Score C. I. %	Total Ion Score	Total Ion C. I. %
6	Ribulose bisphosphate carboxylase small chain chloroplastic OS=Petunia hybrida GN=RBCS PE=3 S	RBS1_PETHY	20357.2	8.29	4	149	100	128	100

Peptide Information

Calc. Mass	Obsrv. Mass	± da	± ppm	Start Seq.	End Seq.	Sequence	Ion Score	C. I. %	Modification	Rank	Result Type
914.4229	914.3481	-0.0748	-82	123	128	YWTMWK	8	0			Mascot
914.4229	914.3481	-0.0748	-82	123	128	YWTMWK					Mascot
930.4178	930.3611	-0.0567	-61	123	128	YWTMWK			Oxidation (M)[4]		Mascot
933.5151	933.4612	-0.0539	-58	158	165	IIGFDNVR	45	99.693			Mascot
933.5151	933.4612	-0.0539	-58	158	165	IIGFDNVR					Mascot
1021.5564	1021.4496	-0.1068	-105	86	93	EIEYLLNK					Mascot
1754.8934	1754.7727	-0.1207	-69	166	180	QVQCISFIAYKPPGF	75	100	Carbamidomethyl (C)[4]		Mascot
1754.8934	1754.7727	-0.1207	-69	166	180	QVQCISFIAYKPPGF			Carbamidomethyl (C)[4]		Mascot

Rank	Protein Name	Accession No	Protein MW	Protein PI	Pep. Count	Protein Score	Protein Score C. I. %	Total Ion Score	Total Ion C. I. %
7	Ribulose bisphosphate carboxylase small chain, chloroplastic OS=Nicotiana tabacum GN=RBCS PE=1 SV=1	RBS_TOBAC	20298.1	7.6	6	134	100	96	100

Peptide Information

Calc. Mass	Obsrv. Mass	± da	± ppm	Start Seq.	End Seq.	Sequence	Ion Score	C. I. %	Modification	Rank	Result Type
914.4002	914.3481	-0.0521	-57	115	122	SPGYYDGR	53	99.943			Mascot
914.4002	914.3481	-0.0521	-57	115	122	SPGYYDGR					Mascot
921.4788	921.4506	-0.0282	-31	31	39	SAASFPVSR					Mascot
930.4178	930.3611	-0.0567	-61	123	128	YWTMWK			Oxidation (M)[4]		Mascot
933.5151	933.4612	-0.0539	-58	158	165	IIGFDNVR	45	99.693			Mascot
933.5151	933.4612	-0.0539	-58	158	165	IIGFDNVR					Mascot
2152.03	2151.8818	-0.1482	-69	129	148	LPMFGCTDATQVLAEVE EAK					Mascot
2168.0249	2167.8801	-0.1448	-67	129	148	LPMFGCTDATQVLAEVE EAK			Oxidation (M)[3]		Mascot
2280.125	2279.9531	-0.1719	-75	129	149	LPMFGCTDATQVLAEVE EAKK					Mascot
2296.1199	2295.9558	-0.1641	-71	129	149	LPMFGCTDATQVLAEVE EAKK			Oxidation (M)[3]		Mascot

Rank	Protein Name	Accession No	Protein MW	Protein PI	Pep. Count	Protein Score	Protein Score C. I. %	Total Ion Score	Total Ion C. I. %
8	Ribulose bisphosphate carboxylase small chain chloroplastic OS=Pisum sativum GN=RBCS-3A PE=1 SV	RBS3_PEA	20218.3	9.1	7	132	100	96	100

Peptide Information

Calc. Mass	Obsrv. Mass	± da	± ppm	Start Seq.	End Seq.	Sequence	Ion Score	C. I. %	Modification	Rank	Result Type
Calc. Mass	Obsrv. Mass	± da	± ppm	Seq.	Seq.	Sequence	Score	C. I. %	Modification	Rank	Result Type
882.4389	882.5139	0.075	85	32	39	SMTGFPVK			Oxidation (M)[2]		Mascot
914.4002	914.3481	-0.0521	-57	115	122	SPGYYDGR	53	99.943			Mascot
914.4002	914.3481	-0.0521	-57	115	122	SPGYYDGR					Mascot
921.504	921.4506	-0.0534	-58	86	92	EVEYLLR					Mascot
930.4178	930.3611	-0.0567	-61	123	128	YWTMWK			Oxidation (M)[4]		Mascot
933.5151	933.4612	-0.0539	-58	158	165	IIGFDNVR	45	99.693			Mascot
933.5151	933.4612	-0.0539	-58	158	165	IIGFDNVR					Mascot
1518.8527	1518.6647	-0.188	-124	81	92	DQLLKEVEYLLR					Mascot
1779.837	1779.7689	-0.0681	-38	166	180	QVQCISFIAHTPESY			Carbamidomethyl (C)[4]		Mascot

Rank	Protein Name	Accession No	Protein MW	Protein PI	Pep. Count	Protein Score	Protein Score C. I. %	Total Ion Score	Total Ion C. I. %
9	Ribulose bisphosphate carboxylase small chain chloroplastic OS=Solanum tuberosum GN=RBCS-2A PE=	RBSA_SOLTU	20276	7.6	6	131	100	96	100

Peptide Information

Calc. Mass	Obsrv. Mass	± da	± ppm	Start Seq.	End Seq.	Sequence	Ion Score	C. I. %	Modification	Rank	Result Type
914.4002	914.3481	-0.0521	-57	115	122	SPGYYDGR	53	99.943			Mascot
914.4002	914.3481	-0.0521	-57	115	122	SPGYYDGR					Mascot
921.4788	921.4506	-0.0282	-31	31	39	SAASFPVSR					Mascot
930.4178	930.3611	-0.0567	-61	123	128	YWTMWK			Oxidation (M)[4]		Mascot
933.5151	933.4612	-0.0539	-58	158	165	IIGFDNVR	45	99.693			Mascot
933.5151	933.4612	-0.0539	-58	158	165	IIGFDNVR					Mascot
2152.03	2151.8818	-0.1482	-69	129	148	LPMFGCTDATQVLAEVE EAK					Mascot
2168.0249	2167.8801	-0.1448	-67	129	148	LPMFGCTDATQVLAEVE EAK			Oxidation (M)[3]		Mascot
2280.125	2279.9531	-0.1719	-75	129	149	LPMFGCTDATQVLAEVE EAKK					Mascot
2296.1199	2295.9558	-0.1641	-71	129	149	LPMFGCTDATQVLAEVE EAKK			Oxidation (M)[3]		Mascot

Rank	Protein Name	Accession No	Protein MW	Protein PI	Pep. Count	Protein Score	Protein Score C. I. %	Total Ion Score	Total Ion C. I. %
10	Ribulose bisphosphate carboxylase small chain chloroplastic OS=Pisum sativum GN=RBCS-3C PE=2 SV	RBS2_PEA	20231.3	9.24	7	130	100	96	100

Peptide Information

Calc. Mass	Obsrv. Mass	± da	± ppm	Start Seq.	End Seq.	Sequence	Ion Score	C. I. %	Modification	Rank	Result Type
882.4389	882.5139	0.075	85	32	39	SMTGFPVK			Oxidation (M)[2]		Mascot
914.4002	914.3481	-0.0521	-57	115	122	SPGYYDGR	53	99.943			Mascot
914.4002	914.3481	-0.0521	-57	115	122	SPGYYDGR					Mascot
921.504	921.4506	-0.0534	-58	86	92	EVEYLLR					Mascot
930.4178	930.3611	-0.0567	-61	123	128	YWTMWK			Oxidation (M)[4]		Mascot
933.5151	933.4612	-0.0539	-58	158	165	IIGFDNVR	45	99.573			Mascot
933.5151	933.4612	-0.0539	-58	158	165	IIGFDNVR					Mascot
1518.8527	1518.6647	-0.188	-124	81	92	DQLLKEVEYLLR					Mascot
1779.837	1779.7689	-0.0681	-38	166	180	QVQCISFIAHTPESY			Carbamidomethyl (C)[4]		Mascot

Gel Idx/Pos	120/E19	Instr./Gel Origin	BA2060/131017A	Process Sta	Analysis Succeeded
Plate [#] Name	[1] 31496	Instrument Sample Name		Spectra	11

Rank	Protein Name	Accession No	Protein MW	Protein PI	Pep. Count	Protein Score	Protein Score C. I. %	Total Ion Score	Total Ion C. I. %
1	Metalloendoproteinase 1 OS=Glycine max PE=1 S	MEP1_SOYBI	34022.9	6.29	9	119	100	68	99.998

Peptide Information

Calc. Mass	Obsrv. Mass	± da	± ppm	Start Seq.	End Seq.	Sequence	Ion Score	C. I. %	Modification	Rank	Result Type
850.4669	850.4968	0.0299	35	100	106	FDINTLK					Mascot
1144.6184	1144.546	-0.0724	-63	274	283	AIMYPSIPPR					Mascot
1144.6184	1144.546	-0.0724	-63	274	283	AIMYPSIPPR					Mascot
1160.6133	1160.5476	-0.0657	-57	274	283	AIMYPSIPPR			Oxidation (M)[3]		Mascot
1160.6133	1160.5476	-0.0657	-57	274	283	AIMYPSIPPR			Oxidation (M)[3]		Mascot
1328.6804	1328.5935	-0.0869	-65	287	298	VNLAQDDIDGIR					Mascot
1456.7754	1456.6859	-0.0895	-61	286	298	KVNLAQDDIDGIR	13	0			Mascot
1456.7754	1456.6859	-0.0895	-61	287	299	VNLAQDDIDGIRK					Mascot
1952.9501	1952.8278	-0.1223	-63	143	159	WQAGTTQLTYAFSPEPR	12	0			Mascot
1952.9501	1952.8278	-0.1223	-63	143	159	WQAGTTQLTYAFSPEPR					Mascot
1999.849	1999.7377	-0.1113	-56	227	243	CHFDADEYWVASGDVTK	16	0	Carbamidomethyl (C)[1]		Mascot
1999.849	1999.7377	-0.1113	-56	227	243	CHFDADEYWVASGDVTK			Carbamidomethyl (C)[1]		Mascot
2412.2083	2412.0449	-0.1634	-68	175	195	WTPVVNIAFQETTSYETA NIK					Mascot
2412.2083	2412.0449	-0.1634	-68	175	195	WTPVVNIAFQETTSYETA NIK	21	0			Mascot
2565.1904	2565.0322	-0.1582	-62	202	226	NHGDPYPFDGPGGILGH AFAPTDGR					Mascot
2565.1904	2565.0322	-0.1582	-62	202	226	NHGDPYPFDGPGGILGH AFAPTDGR	8	0			Mascot

Rank	Protein Name	Accession No	Protein MW	Protein PI	Pep. Count	Protein Score	Protein Score C. I. %	Total Ion Score	Total Ion C. I. %
2	Ras-related protein RABB1b OS=Arabidopsis tha GN=RABB1B PE=2 SV=1	RAB1B_ARAT	23160.6	6.52	8	49	54.617		

Peptide Information

Calc. Mass	Obsrv. Mass	± da	± ppm	Start Seq.	End Seq.	Sequence	Ion Score	C. I. %	Modification	Rank	Result Type
842.4301	842.4646	0.0345	41	121	127	CDLAHKR					Mascot
1045.5425	1045.4895	-0.053	-51	70	77	SITRSYYR					Mascot
1167.6078	1167.5872	-0.0206	-18	20	29	SCLLLQFTDK					Mascot
1263.6552	1263.6287	-0.0265	-21	187	199	IGYGRTQGAAGGR					Mascot
1323.709	1323.5995	-0.1095	-83	20	30	SCLLLQFTDKR					Mascot
1475.8329	1475.6847	-0.1482	-100	78	91	GAAGALLVYDITRR					Mascot
2367.1365	2367.0898	-0.0467	-20	106	126	QHANPNMSIMLIGNKCDL AHK			Oxidation (M)[7,10]		Mascot
3337.6204	3337.5156	-0.1048	-31	92	120	ETFNHLASWLEDARQHA NPNMSIMLIGNK					Mascot

Rank	Protein Name	Accession No	Protein MW	Protein PI	Pep. Count	Protein Score	Protein Score C. I. %	Total Ion Score	Total Ion C. I. %
3	Uncharacterized protein At4g29660 OS=Arabidop thaliana GN=EMB2752 PE=4 SV=1	Y4966_ARAT	12264.2	9.48	6	49	54.617		

Peptide Information

Calc. Mass	Obsrv. Mass	± da	± ppm	Start Seq.	End Seq.	Sequence	Ion Score	C. I. %	Modification	Rank	Result Type
856.4311	856.473	0.0419	49	17	22	YNKFER					Mascot
856.4311	856.473	0.0419	49	17	22	YNKFER					Mascot
1064.5048	1064.5487	0.0439	41	12	19	YADYKYNK					Mascot
1434.7311	1434.6788	-0.0523	-36	1	11	MQGVWSQLWRK			Oxidation (M)[1]		Mascot
1456.693	1456.6859	-0.0071	-5	23	33	FAVWEMIEPYR			Oxidation (M)[6]		Mascot
1456.693	1456.6859	-0.0071	-5	23	33	FAVWEMIEPYR			Oxidation (M)[6]		Mascot
1837.9418	1837.8623	-0.0795	-43	23	36	FAVWEMIEPYRRPK			Oxidation (M)[6]		Mascot
3311.7812	3312.009	0.2278	69	37	66	TFTTLITIYVAAFYTGVIGA AVTEQLYKEK					Mascot

Rank	Protein Name	Accession No	Protein MW	Protein PI	Pep. Count	Protein Score	Protein Score C. I. %	Total Ion Score	Total Ion C. I. %
4	Ras-related protein Rab-2-B OS=Zea mays GN=R PE=2 SV=1	RAB2B_MAIZI	23046.5	6.96	8	48	49.079		

Peptide Information

Calc. Mass	Obsrv. Mass	± da	± ppm	Start Seq.	End Seq.	Sequence	Ion Score	C. I. %	Modification	Rank	Result Type
1045.5425	1045.4895	-0.053	-51	70	77	SITRSYYR					Mascot
1167.6078	1167.5872	-0.0206	-18	20	29	SCLLLQFTDK					Mascot
1323.709	1323.5995	-0.1095	-83	20	30	SCLLLQFTDKR					Mascot
1357.627	1357.6293	0.0023	2	128	139	AVSYEEGEQFAK					Mascot
1475.8329	1475.6847	-0.1482	-100	78	91	GAAGALLVYDITRR					Mascot
1657.8148	1657.6959	-0.1189	-72	106	120	QHANANMTIMLVGNK			Oxidation (M)[7]		Mascot
2565.2654	2565.0322	-0.2332	-91	140	162	EHGLIFMEASAKTAQNVE EAFVK			Oxidation (M)[7]		Mascot
2565.2654	2565.0322	-0.2332	-91	140	162	EHGLIFMEASAKTAQNVE EAFVK			Oxidation (M)[7]		Mascot
3311.6047	3312.009	0.4043	122	92	120	ETFNHLASWLEDARQHA NANMTIMLVGNK					Mascot

Rank	Protein Name	Accession No	Protein MW	Protein PI	Pep. Count	Protein Score	Protein Score C. I. %	Total Ion Score	Total Ion C. I. %
5	Disease resistance protein RFL1 OS=Arabidopsis thaliana GN=RFL1 PE=3 SV=2	RFL1_ARATH	101379.3	6.27	16	47	35.895		

Peptide Information

Calc. Mass	Obsrv. Mass	± da	± ppm	Start Seq.	End Seq.	Sequence	Ion Score	C. I. %	Modification	Rank	Result Type
834.4025	834.369	-0.0335	-40	750	757	IEGCDGLK					Mascot
973.4303	973.4791	0.0488	50	100	108	LCLCGFCSK					Mascot
1005.5662	1005.5543	-0.0119	-12	878	885	FLPTCRLR					Mascot
1033.5135	1033.4586	-0.0549	-53	112	119	MSYLYGKR			Oxidation (M)[1]		Mascot
1045.5612	1045.4895	-0.0717	-69	685	692	CIQHIYIR					Mascot
1072.5494	1072.5016	-0.0478	-45	51	59	DDVQGRINR					Mascot
1144.4948	1144.546	0.0512	45	100	108	LCLCGFCSK			Carbamidomethyl (C)[2,4,7]		Mascot
1144.4948	1144.546	0.0512	45	100	108	LCLCGFCSK			Carbamidomethyl (C)[2,4,7]		Mascot
1160.6521	1160.5476	-0.1045	-90	461	471	SSLLLEGAKDK					Mascot
1160.6521	1160.5476	-0.1045	-90	461	471	SSLLLEGAKDK					Mascot
1357.7521	1357.6293	-0.1228	-90	682	692	VGRCIQHIYIR					Mascot
1383.6508	1383.5955	-0.0553	-40	520	530	RMSLMNNNFEK					Mascot
1390.7617	1390.5719	-0.1898	-136	260	270	FVLLLDDIWEK					Mascot
1434.7158	1434.6788	-0.037	-26	289	301	VAFTTHSKEVCGR					Mascot
1584.8202	1584.7784	-0.0418	-26	773	785	VWGCKHLEDIISK			Carbamidomethyl (C)[4]		Mascot
1952.9642	1952.8278	-0.1364	-70	348	365	CCGLPLALNVIGETMSFK			Carbamidomethyl (C)[1]		Mascot
1952.9642	1952.8278	-0.1364	-70	348	365	CCGLPLALNVIGETMSFK			Carbamidomethyl (C)[1]		Mascot
2211.0249	2210.9673	-0.0576	-26	409	425	SCFLYCSLFPEDFEIRK			Carbamidomethyl (C)[2,6]		Mascot
2225.2439	2224.9797	-0.2642	-119	203	223	LGGGFDVVIWVVVSKNA TVHK					Mascot
2412.197	2412.0449	-0.1521	-63	344	365	VSEKCCGLPLALNVIGET MSFK			Carbamidomethyl (C)[5], Oxidation (M)[19]		Mascot
2412.197	2412.0449	-0.1521	-63	344	365	VSEKCCGLPLALNVIGET MSFK			Carbamidomethyl (C)[5], Oxidation (M)[19]		Mascot

Rank	Protein Name	Accession No	Protein MW	Protein PI	Pep. Count	Protein Score	Protein Score C. I. %	Total Ion Score	Total Ion C. I. %
6	Probable calcium-binding protein CML22 OS=Ory sativa subsp. japonica GN=CML22 PE=2 SV=1	CML22_ORYS	26438	4.58	8	43	0		

Peptide Information

Calc. Mass	Obsrv. Mass	± da	± ppm	Start Seq.	End Seq.	Sequence	Ion Score	C. I. %	Modification	Rank	Result Type
834.4138	834.369	-0.0448	-54	220	226	MIGQVDR			Oxidation (M)[1]		Mascot
996.4302	996.5389	0.1087	109	155	164	SIMAGGDDSK			Oxidation (M)[3]		Mascot
1106.5007	1106.4614	-0.0393	-36	210	218	QGRTAEECR			Carbamidomethyl (C)[8]		Mascot
1308.5848	1308.5815	-0.0033	-3	155	167	SIMAGGDDSKDGR					Mascot
1328.6553	1328.5935	-0.0618	-47	84	95	QQQQADAAELAR					Mascot
1383.4852	1383.5955	0.1103	80	170	180	EEEEEEDGDMR			Oxidation (M)[10]		Mascot
1456.7502	1456.6859	-0.0643	-44	83	95	KQQQQADAAELAR					Mascot
1456.7502	1456.6859	-0.0643	-44	83	95	KQQQQADAAELAR					Mascot
1952.8741	1952.8278	-0.0463	-24	227	242	DGDGRVDFHEFLQMMR					Mascot
1952.8741	1952.8278	-0.0463	-24	227	242	DGDGRVDFHEFLQMMR					Mascot

Rank	Protein Name	Accession No	Protein MW	Protein PI	Pep. Count	Protein Score	Protein Score C. I. %	Total Ion Score	Total Ion C. I. %
7	Ribulose bisphosphate carboxylase small chain (512 (Fragment) OS=Triticum aestivum PE=3 SV=1	RBS3_WHEA	13046.5	5.84	6	41	0		

Peptide Information

Calc. Mass	Obsrv. Mass	± da	± ppm	Start Seq.	End Seq.	Sequence	Ion Score	C. I. %	Modification	Rank	Result Type
1165.571	1165.5229	-0.0481	-41	23	31	WVPCLEFSK			Carbamidomethyl (C)[4]		Mascot
1165.571	1165.6879	0.1169	100	23	31	WVPCLEFSK			Carbamidomethyl (C)[4]		Mascot
1323.6765	1323.5995	-0.077	-58	21	31	SKWVPCLEFSK					Mascot
2211.0571	2210.9673	-0.0898	-41	93	112	QVQCVSFIAFKPPGCEES GK			Carbamidomethyl (C)[4]		Mascot
2225.073	2224.9797	-0.0933	-42	93	113	QVQCVSFIAFKPPGCEES GKA					Mascot
2239.062	2238.9792	-0.0828	-37	56	75	LPMFGCTDATQVINEVEE VK			Oxidation (M)[3]		Mascot
2367.157	2367.0898	-0.0672	-28	56	76	LPMFGCTDATQVINEVEE VKK			Oxidation (M)[3]		Mascot

Rank	Protein Name	Accession No	Protein MW	Protein PI	Pep. Count	Protein Score	Protein Score C. I. %	Total Ion Score	Total Ion C. I. %
8	Cryptochrome-2 OS=Arabidopsis thaliana GN=CR PE=1 SV=2	CRY2_ARATI	69413	5.69	11	40	0		

Peptide Information

Calc. Mass	Obsrv. Mass	± da	± ppm	Start Seq.	End Seq.	Sequence	Ion Score	C. I. %	Modification	Rank	Result Type
850.4669	850.4968	0.0299	35	228	234	QLIDYAK					Mascot
856.4523	856.473	0.0207	24	112	118	DHTVKEK					Mascot
856.4523	856.473	0.0207	24	112	118	DHTVKEK					Mascot
1005.5615	1005.5543	-0.0072	-7	220	227	LLNEFIEK					Mascot
1045.5975	1045.4895	-0.108	-103	267	274	MKQIIWAR					Mascot
1144.548	1144.546	-0.002	-2	543	551	VKPEEEEER					Mascot
1144.548	1144.546	-0.002	-2	543	551	VKPEEEEER					Mascot
1179.5826	1179.5281	-0.0545	-46	336	346	TGYPLVDAGMR					Mascot
1179.6368	1179.5281	-0.1087	-92	228	237	QLIDYAKNSK					Mascot
1434.7966	1434.6788	-0.1178	-82	369	379	FLLLPWKWGMK			Oxidation (M)[10]		Mascot
1456.7577	1456.6859	-0.0718	-49	79	91	THNTISAILDCIR					Mascot
1456.7577	1456.6859	-0.0718	-49	79	91	THNTISAILDCIR					Mascot
1657.906	1657.6959	-0.2101	-127	98	111	VVFNHLYDPVSLVR					Mascot
2888.3142	2888.2268	-0.0874	-30	563	590	ELFSTAESSSSSSVFFVS QSCSLASEGK					Mascot

Rank	Protein Name	Accession No	Protein MW	Protein PI	Pep. Count	Protein Score	Protein Score C. I. %	Total Ion Score	Total Ion C. I. %
9	Putative pentatricopeptide repeat-containing prote At5g37570 OS=Arabidopsis thaliana GN=PCMP-E37	PP403_ARAT	61611.8	6.71	12	39	0		

Peptide Information

Calc. Mass	Obsrv. Mass	± da	± ppm	Start Seq.	End Seq.	Sequence	Ion Score	C. I. %	Modification	Rank	Result Type
996.493	996.5389	0.0459	46	158	165	CKDLFSAR			Carbamidomethyl (C)[1]		Mascot
1033.4844	1033.4586	-0.0258	-25	119	127	VCSNNGQVR			Carbamidomethyl (C)[2]		Mascot
1045.4666	1045.4895	0.0229	22	353	361	CGHMDRAAK			Carbamidomethyl (C)[1]		Mascot
1051.524	1051.6495	0.1255	119	229	236	LFDEMPKR			Oxidation (M)[5]		Mascot
1157.4966	1157.5116	0.015	13	197	205	SMFDLMPER			Oxidation (M)[2,6]		Mascot
1165.5518	1165.5229	-0.0289	-25	532	541	DKMNENGITK			Oxidation (M)[3]		Mascot
1165.5518	1165.6879	0.1361	117	532	541	DKMNENGITK			Oxidation (M)[3]		Mascot
1390.6896	1390.5719	-0.1177	-85	326	336	VDSYLHQRMNK					Mascot
1584.8995	1584.7784	-0.1211	-76	5	18	LSHPSLLSLETLFK					Mascot
1837.9517	1837.8623	-0.0894	-49	103	118	TGLARPDEYTFPLVMK					Mascot
1952.9795	1952.8278	-0.1517	-78	88	102	FLFFETVSILMRMMR			Oxidation (M)[11,13]		Mascot
1952.9795	1952.8278	-0.1517	-78	88	102	FLFFETVSILMRMMR			Oxidation (M)[11,13]		Mascot
2565.0547	2565.0322	-0.0225	-9	370	392	DLVSYCSMMEGMAIHGC GSEAIR			Carbamidomethyl (C)[6], Oxidation (M)[8,9,12]		Mascot
2565.0547	2565.0322	-0.0225	-9	370	392	DLVSYCSMMEGMAIHGC GSEAIR			Carbamidomethyl (C)[6], Oxidation (M)[8,9,12]		Mascot
3354.5994	3354.5059	-0.0935	-28	467	498	SMPFEAHASAWGSLLGG CSLHGNTEIAEVVAR			Carbamidomethyl (C)[18]		Mascot

Rank	Protein Name	Accession No	Protein MW	Protein PI	Pep. Count	Protein Score	Protein Score C. I. %	Total Ion Score	Total Ion C. I. %
10	Hexokinase-4, chloroplastic OS=Oryza sativa sub: japonica GN=HXK4 PE=2 SV=1	HXK4_ORYS.	54724.2	6.49	10	38	0		

Peptide Information

Calc. Mass	Obsrv. Mass	± da	± ppm	Start Seq.	End Seq.	Sequence	Ion Score	C. I. %	Modification	Rank	Result Type
842.4743	842.4646	-0.0097	-12	436	442	GHIFGRR					Mascot
882.458	882.5198	0.0618	70	455	460	YPQYRR					Mascot
996.5658	996.5389	-0.0269	-27	58	66	CAAPLPVLR			Carbamidomethyl (C)[1]		Mascot
1045.5497	1045.4895	-0.0602	-58	26	37	SRGGGSGAAAVR					Mascot
1072.6514	1072.5016	-0.1498	-140	357	365	KLAEPFVLR					Mascot
1152.667	1152.8079	0.1409	122	58	67	CAAPLPVLRR			Carbamidomethyl (C)[1]		Mascot
1233.6508	1233.5276	-0.1232	-100	403	413	VTVEVSDCIIR					Mascot
1265.7511	1265.562	-0.1891	-149	56	66	LRCAAPLPVLR			Carbamidomethyl (C)[3]		Mascot
1277.7576	1277.629	-0.1286	-101	273	283	TEAIPKLQHLK					Mascot
1434.6543	1434.6788	0.0245	17	430	441	MENDSRGHIFGR			Oxidation (M)[1]		Mascot

Gel Idx/Pos	121/E20	Instr./Gel Origin	BA2060/131017A	Process Sta	Analysis Succeeded
Plate [#] Name	[1] 31496	Instrument Sample Name		Spectra	8

Rank	Protein Name	Accession No	Protein MW	Protein PI	Pep. Count	Protein Score	Protein Score C. I. %	Total Ion Score	Total Ion C. I. %
1	Ribulose bisphosphate carboxylase large chain OS=Glycine max GN=rbcL PE=1 SV=3	RBL_SOYBN	52576.4	6	22	451	100	279	100

Peptide Information

Calc. Mass	Obsrv. Mass	± da	± ppm	Start Seq.	End Seq.	Sequence	Ion Score	C. I. %	Modification	Rank	Result Type
830.4366	830.3947	-0.0419	-50	440	446	EGNEIIR					Mascot
830.4366	830.3947	-0.0419	-50	440	446	EGNEIIR	45	99.53			Mascot
898.41	898.3639	-0.0461	-51	306	312	NHGMHFR					Mascot
910.4451	910.3986	-0.0465	-51	188	194	AVYECLR	21	0	Carbamidomethyl (C)[5]		Mascot
910.4451	910.3986	-0.0465	-51	188	194	AVYECLR			Carbamidomethyl (C)[5]		Mascot
912.4719	912.4184	-0.0535	-59	296	303	AMHAVIDR					Mascot
914.4049	914.3662	-0.0387	-42	306	312	NHGMHFR			Oxidation (M)[4]		Mascot
928.4669	928.4195	-0.0474	-51	296	303	AMHAVIDR	18	0	Oxidation (M)[2]		Mascot
928.4669	928.4195	-0.0474	-51	296	303	AMHAVIDR			Oxidation (M)[2]		Mascot
962.4788	962.4269	-0.0519	-54	228	236	SQAETGEIK					Mascot
1021.5312	1021.4796	-0.0516	-51	33	41	DTDILAAFR					Mascot
1023.4741	1023.421	-0.0531	-52	351	358	DDFVEKDR					Mascot
1023.4741	1023.421	-0.0531	-52	351	358	DDFVEKDR	59	99.982			Mascot
1102.4762	1102.4115	-0.0647	-59	467	475	FEFEAMDTL					Mascot
1116.583	1116.5267	-0.0563	-50	422	431	VALEACVQAR			Carbamidomethyl (C)[6]		Mascot
1116.583	1116.5267	-0.0563	-50	422	431	VALEACVQAR	65	99.995	Carbamidomethyl (C)[6]		Mascot
1154.5636	1154.4729	-0.0907	-79	304	312	QKNHGMHFR					Mascot
1170.5585	1170.5757	0.0172	15	304	312	QKNHGMHFR			Oxidation (M)[6]		Mascot
1187.6643	1187.6018	-0.0625	-53	286	295	DNGLLLHIHR					Mascot
1245.6337	1245.5686	-0.0651	-52	218	227	FLFCAEAIFK			Carbamidomethyl (C)[4]		Mascot
1275.7307	1275.6642	-0.0665	-52	340	350	EITLGFVDLLR					Mascot
1433.7495	1433.672	-0.0775	-54	320	334	LSGGDHVHAGTVVGK	71	99.999			Mascot
1433.7495	1433.672	-0.0775	-54	320	334	LSGGDHVHAGTVVGK					Mascot
1451.6219	1451.5431	-0.0788	-54	202	213	DDENVNSQPFMR					Mascot
1465.7546	1465.6755	-0.0791	-54	147	159	TFQGPPHGIQVER					Mascot
1467.6168	1467.5345	-0.0823	-56	202	213	DDENVNSQPFMR			Oxidation (M)[11]		Mascot
1488.6926	1488.6111	-0.0815	-55	464	475	EIKFEFEAMDTL			Oxidation (M)[9]		Mascot
1546.7358	1546.65	-0.0858	-55	451	463	WSPELAAACEVWK			Carbamidomethyl (C)[9]		Mascot
1812.7714	1812.6766	-0.0948	-52	237	252	GHYLNATAGTCEEMMK			Carbamidomethyl (C)[11]		Mascot
1828.7662	1828.6584	-0.1078	-59	237	252	GHYLNATAGTCEEMMK			Carbamidomethyl (C)[11], Oxidation (M)[14]		Mascot
1844.7611	1844.6625	-0.0986	-53	237	252	GHYLNATAGTCEEMMK			Carbamidomethyl (C)[11], Oxidation (M)[14,15]		Mascot
1984.8673	1984.7566	-0.1107	-56	237	253	GHYLNATAGTCEEMMKR			Carbamidomethyl (C)[11], Oxidation (M)[14]		Mascot
2000.8623	2000.744	-0.1183	-59	237	253	GHYLNATAGTCEEMMKR			Carbamidomethyl (C)[11], Oxidation (M)[14,15]		Mascot
2169.9868	2169.8643	-0.1225	-56	195	213	GGLDFTKDDENVNSQPFMR					Mascot
2185.9819	2185.8582	-0.1237	-57	195	213	GGLDFTKDDENVNSQPFMR			Oxidation (M)[18]		Mascot
3038.45	3038.2954	-0.1546	-51	259	285	ELGVPIVMHDYLTGGFTANTSLAHYCR			Carbamidomethyl (C)[26], Oxidation (M)[8]		Mascot

Rank	Protein Name	Accession No	Protein MW	Protein PI	Pep. Count	Protein Score	Protein Score C. I. %	Total Ion Score	Total Ion C. I. %
2	Ribulose bisphosphate carboxylase large chain OS=Cucumis sativus GN=rbcL PE=1 SV=5	RBL_CUCSA	52608.4	6	21	365	100	208	100

Peptide Information

Calc. Mass	Obsrv. Mass	± da	± ppm	Start Seq.	End Seq.	Sequence	Ion Score	C. I. %	Modification	Rank	Result Type
830.4366	830.3947	-0.0419	-50	440	446	EGNEIIR					Mascot
830.4366	830.3947	-0.0419	-50	440	446	EGNEIIR	45	99.53			Mascot
898.41	898.3639	-0.0461	-51	306	312	NHGMHFR					Mascot
910.4451	910.3986	-0.0465	-51	188	194	AVYECLR	21	0	Carbamidomethyl (C)[5]		Mascot
910.4451	910.3986	-0.0465	-51	188	194	AVYECLR			Carbamidomethyl (C)[5]		Mascot
912.4719	912.4184	-0.0535	-59	296	303	AMHAVIDR					Mascot
914.4049	914.3662	-0.0387	-42	306	312	NHGMHFR			Oxidation (M)[4]		Mascot
928.4669	928.4195	-0.0474	-51	296	303	AMHAVIDR	18	0	Oxidation (M)[2]		Mascot
928.4669	928.4195	-0.0474	-51	296	303	AMHAVIDR			Oxidation (M)[2]		Mascot
962.4788	962.4269	-0.0519	-54	228	236	SQAETGEIK					Mascot
1021.5312	1021.4796	-0.0516	-51	33	41	DTDILAAFR					Mascot
1023.4741	1023.421	-0.0531	-52	351	358	DDFVEKDR					Mascot
1023.4741	1023.421	-0.0531	-52	351	358	DDFVEKDR	59	99.982			Mascot
1102.4762	1102.4115	-0.0647	-59	467	475	FEFEAMDTL					Mascot
1116.583	1116.5267	-0.0563	-50	422	431	VALEACVQAR			Carbamidomethyl (C)[6]		Mascot
1116.583	1116.5267	-0.0563	-50	422	431	VALEACVQAR	65	99.995	Carbamidomethyl (C)[6]		Mascot
1154.5636	1154.4729	-0.0907	-79	304	312	QKNHGMHFR					Mascot
1170.5585	1170.5757	0.0172	15	304	312	QKNHGMHFR			Oxidation (M)[6]		Mascot
1187.6643	1187.6018	-0.0625	-53	286	295	DNGLLLHIHR					Mascot
1245.6337	1245.5686	-0.0651	-52	218	227	FLFCAEAIFK			Carbamidomethyl (C)[4]		Mascot
1275.7307	1275.6642	-0.0665	-52	340	350	EITLGFVDLLR					Mascot
1451.6219	1451.5431	-0.0788	-54	202	213	DDENVNSQPFMR					Mascot
1465.7216	1465.6755	-0.0461	-31	320	334	MSGGDHIHAGTVVGK					Mascot
1467.6168	1467.5345	-0.0823	-56	202	213	DDENVNSQPFMR			Oxidation (M)[11]		Mascot
1488.6926	1488.6111	-0.0815	-55	464	475	EIKFEFEAMDTL			Oxidation (M)[9]		Mascot
1546.7358	1546.65	-0.0858	-55	451	463	WSPELAAACEVWK			Carbamidomethyl (C)[9]		Mascot
1812.7714	1812.6766	-0.0948	-52	237	252	GHYLNATAGTCEEMMK			Carbamidomethyl (C)[11]		Mascot
1828.7662	1828.6584	-0.1078	-59	237	252	GHYLNATAGTCEEMMK			Carbamidomethyl (C)[11], Oxidation (M)[14]		Mascot
1844.7611	1844.6625	-0.0986	-53	237	252	GHYLNATAGTCEEMMK			Carbamidomethyl (C)[11], Oxidation (M)[14,15]		Mascot
1984.8673	1984.7566	-0.1107	-56	237	253	GHYLNATAGTCEEMMKR			Carbamidomethyl (C)[11], Oxidation (M)[14]		Mascot
2000.8623	2000.744	-0.1183	-59	237	253	GHYLNATAGTCEEMMKR			Carbamidomethyl (C)[11], Oxidation (M)[14,15]		Mascot
2050.0134	2049.8789	-0.1345	-66	320	339	MSGGDHIHAGTVVGKLEGER					Mascot
2169.9868	2169.8643	-0.1225	-56	195	213	GGLDFTKDDENVNSQPFMR					Mascot
2185.9819	2185.8582	-0.1237	-57	195	213	GGLDFTKDDENVNSQPFMR			Oxidation (M)[18]		Mascot

Rank	Protein Name	Accession No	Protein MW	Protein PI	Pep. Count	Protein Score	Protein Score C. I. %	Total Ion Score	Total Ion C. I. %
3	Ribulose bisphosphate carboxylase large chain (Fragment) OS=Cornus florida GN=rbcL PE=3 SV=2	RBL_CORFO	51593.1	6.14	19	348	100	208	100

Peptide Information

Calc. Mass	Obsrv. Mass	± da	± ppm	Start Seq.	End Seq.	Sequence	Ion Score	C. I. %	Modification	Rank	Result Type
830.4366	830.3947	-0.0419	-50	430	436	EGNEIIR					Mascot
830.4366	830.3947	-0.0419	-50	430	436	EGNEIIR	45	99.53			Mascot
880.4536	880.4147	-0.0389	-44	296	302	NHGIHFR					Mascot
910.4451	910.3986	-0.0465	-51	178	184	AVYECLR	21	0	Carbamidomethyl (C)[5]		Mascot
910.4451	910.3986	-0.0465	-51	178	184	AVYECLR			Carbamidomethyl (C)[5]		Mascot
912.4719	912.4184	-0.0535	-59	286	293	AMHAVIDR					Mascot
928.4669	928.4195	-0.0474	-51	286	293	AMHAVIDR	18	0	Oxidation (M)[2]		Mascot
928.4669	928.4195	-0.0474	-51	286	293	AMHAVIDR			Oxidation (M)[2]		Mascot
1021.5312	1021.4796	-0.0516	-51	23	31	DTDILAAFR					Mascot
1023.4741	1023.421	-0.0531	-52	341	348	DDFVEKDR					Mascot
1023.4741	1023.421	-0.0531	-52	341	348	DDFVEKDR	59	99.982			Mascot
1102.4762	1102.4115	-0.0647	-59	457	465	FEFEAMDTL					Mascot
1116.583	1116.5267	-0.0563	-50	412	421	VALEACVQAR			Carbamidomethyl (C)[6]		Mascot
1116.583	1116.5267	-0.0563	-50	412	421	VALEACVQAR	65	99.995	Carbamidomethyl (C)[6]		Mascot
1187.6643	1187.6018	-0.0625	-53	276	285	DNGLLLHIHR					Mascot
1245.6433	1245.5686	-0.0747	-60	430	440	EGNEIIREASK					Mascot
1275.7307	1275.6642	-0.0665	-52	330	340	EITLGFVDLLR					Mascot
1451.6219	1451.5431	-0.0788	-54	192	203	DDENVNSQPFMR					Mascot
1465.7546	1465.6755	-0.0791	-54	137	149	TFQGPPHGIQVER					Mascot
1467.6168	1467.5345	-0.0823	-56	192	203	DDENVNSQPFMR			Oxidation (M)[11]		Mascot
1488.6926	1488.6111	-0.0815	-55	454	465	EIKFEFEAMDTL			Oxidation (M)[9]		Mascot
1546.7358	1546.65	-0.0858	-55	441	453	WSPELAAACEVWK			Carbamidomethyl (C)[9]		Mascot
1812.7714	1812.6766	-0.0948	-52	227	242	GHYLNATAGTCEEMMK			Carbamidomethyl (C)[11]		Mascot
1828.7662	1828.6584	-0.1078	-59	227	242	GHYLNATAGTCEEMMK			Carbamidomethyl (C)[11], Oxidation (M)[14]		Mascot
1844.7611	1844.6625	-0.0986	-53	227	242	GHYLNATAGTCEEMMK			Carbamidomethyl (C)[11], Oxidation (M)[14,15]		Mascot
1984.8673	1984.7566	-0.1107	-56	227	243	GHYLNATAGTCEEMMKR			Carbamidomethyl (C)[11], Oxidation (M)[14]		Mascot
2000.8623	2000.744	-0.1183	-59	227	243	GHYLNATAGTCEEMMKR			Carbamidomethyl (C)[11], Oxidation (M)[14,15]		Mascot
2169.9868	2169.8643	-0.1225	-56	185	203	GGLDFTKDDENVNSQPFMR					Mascot
2185.9819	2185.8582	-0.1237	-57	185	203	GGLDFTKDDENVNSQPFMR			Oxidation (M)[18]		Mascot
3038.45	3038.2954	-0.1546	-51	249	275	ELGVPIVMHDYLTGGFTANTSLAHYCR			Carbamidomethyl (C)[26], Oxidation (M)[8]		Mascot

Rank	Protein Name	Accession No	Protein MW	Protein PI	Pep. Count	Protein Score	Protein Score C. I. %	Total Ion Score	Total Ion C. I. %
4	Ribulose bisphosphate carboxylase large chain (Fragment) OS=Cornus canadensis GN=rbcL PE=3 SV=3	RBL_CORCA	51610.1	6.23	19	348	100	208	100

Peptide Information

Calc. Mass	Obsrv. Mass	± da	± ppm	Start Seq.	End Seq.	Sequence	Ion Score	C. I. %	Modification	Rank	Result Type
830.4366	830.3947	-0.0419	-50	430	436	EGNEIIR					Mascot
830.4366	830.3947	-0.0419	-50	430	436	EGNEIIR	45	99.53			Mascot
880.4536	880.4147	-0.0389	-44	296	302	NHGIHFR					Mascot
910.4451	910.3986	-0.0465	-51	178	184	AVYECLR	21	0	Carbamidomethyl (C)[5]		Mascot
910.4451	910.3986	-0.0465	-51	178	184	AVYECLR			Carbamidomethyl (C)[5]		Mascot
912.4719	912.4184	-0.0535	-59	286	293	AMHAVIDR					Mascot
928.4669	928.4195	-0.0474	-51	286	293	AMHAVIDR	18	0	Oxidation (M)[2]		Mascot
928.4669	928.4195	-0.0474	-51	286	293	AMHAVIDR			Oxidation (M)[2]		Mascot
962.4788	962.4269	-0.0519	-54	218	226	SQAETGEIK					Mascot
1021.5312	1021.4796	-0.0516	-51	23	31	DTDILAAFR					Mascot
1023.4741	1023.421	-0.0531	-52	341	348	DDFVEKDR					Mascot
1023.4741	1023.421	-0.0531	-52	341	348	DDFVEKDR	59	99.982			Mascot
1102.4762	1102.4115	-0.0647	-59	457	465	FEFEAMDTL					Mascot
1116.583	1116.5267	-0.0563	-50	412	421	VALEACVQAR			Carbamidomethyl (C)[6]		Mascot
1116.583	1116.5267	-0.0563	-50	412	421	VALEACVQAR	65	99.995	Carbamidomethyl (C)[6]		Mascot
1187.6643	1187.6018	-0.0625	-53	276	285	DNGLLLHIHR					Mascot
1245.6433	1245.5686	-0.0747	-60	430	440	EGNEIIREASK					Mascot
1451.6219	1451.5431	-0.0788	-54	192	203	DDENVNSQPFMR					Mascot
1465.7546	1465.6755	-0.0791	-54	137	149	TFQGPPHGIQVER					Mascot
1467.6168	1467.5345	-0.0823	-56	192	203	DDENVNSQPFMR			Oxidation (M)[11]		Mascot
1488.6926	1488.6111	-0.0815	-55	454	465	EIKFEFEAMDTL			Oxidation (M)[9]		Mascot
1546.7358	1546.65	-0.0858	-55	441	453	WSPELAAACEVWK			Carbamidomethyl (C)[9]		Mascot
1812.7714	1812.6766	-0.0948	-52	227	242	GHYLNATAGTCEEMMK			Carbamidomethyl (C)[11]		Mascot
1828.7662	1828.6584	-0.1078	-59	227	242	GHYLNATAGTCEEMMK			Carbamidomethyl (C)[11], Oxidation (M)[14]		Mascot
1844.7611	1844.6625	-0.0986	-53	227	242	GHYLNATAGTCEEMMK			Carbamidomethyl (C)[11], Oxidation (M)[14,15]		Mascot
1984.8673	1984.7566	-0.1107	-56	227	243	GHYLNATAGTCEEMMKR			Carbamidomethyl (C)[11], Oxidation (M)[14]		Mascot
2000.8623	2000.744	-0.1183	-59	227	243	GHYLNATAGTCEEMMKR			Carbamidomethyl (C)[11], Oxidation (M)[14,15]		Mascot
2169.9868	2169.8643	-0.1225	-56	185	203	GGLDFTKDDENVNSQPFMR					Mascot
2185.9819	2185.8582	-0.1237	-57	185	203	GGLDFTKDDENVNSQPFMR			Oxidation (M)[18]		Mascot
3038.45	3038.2954	-0.1546	-51	249	275	ELGVPIVMHDYLTGGFTANTSLAHYCR			Carbamidomethyl (C)[26], Oxidation (M)[8]		Mascot

Rank	Protein Name	Accession No	Protein MW	Protein PI	Pep. Count	Protein Score	Protein Score C. I. %	Total Ion Score	Total Ion C. I. %
5	Ribulose bisphosphate carboxylase large chain (Fragment) OS=Manilkara zapota GN=rbcL PE=3 SV=2	RBL_MANZA	51550	6.04	18	347	100	220	100

Peptide Information

Calc. Mass	Obsrv. Mass	± da	± ppm	Start Seq.	End Seq.	Sequence	Ion Score	C. I. %	Modification	Rank	Result Type
830.4366	830.3947	-0.0419	-50	430	436	EGNEIIR					Mascot
830.4366	830.3947	-0.0419	-50	430	436	EGNEIIR	45	99.53			Mascot
898.41	898.3639	-0.0461	-51	296	302	NHGMHFR					Mascot
910.4451	910.3986	-0.0465	-51	178	184	AVYECLR	21	0	Carbamidomethyl (C)[5]		Mascot
910.4451	910.3986	-0.0465	-51	178	184	AVYECLR			Carbamidomethyl (C)[5]		Mascot
912.4719	912.4184	-0.0535	-59	286	293	AMHAVIDR					Mascot
914.4049	914.3662	-0.0387	-42	296	302	NHGMHFR			Oxidation (M)[4]		Mascot
928.4669	928.4195	-0.0474	-51	286	293	AMHAVIDR	18	0	Oxidation (M)[2]		Mascot
928.4669	928.4195	-0.0474	-51	286	293	AMHAVIDR			Oxidation (M)[2]		Mascot
1021.5312	1021.4796	-0.0516	-51	23	31	DTDILAAFR					Mascot
1102.4762	1102.4115	-0.0647	-59	457	465	FEFEAMDTL					Mascot
1116.583	1116.5267	-0.0563	-50	412	421	VALEACVQAR			Carbamidomethyl (C)[6]		Mascot
1116.583	1116.5267	-0.0563	-50	412	421	VALEACVQAR	65	99.995	Carbamidomethyl (C)[6]		Mascot
1154.5636	1154.4729	-0.0907	-79	294	302	QKNHGMHFR					Mascot
1170.5585	1170.5757	0.0172	15	294	302	QKNHGMHFR			Oxidation (M)[6]		Mascot
1187.6643	1187.6018	-0.0625	-53	276	285	DNGLLLHIHR					Mascot
1245.6433	1245.5686	-0.0747	-60	430	440	EGNEIIREASK					Mascot
1275.7307	1275.6642	-0.0665	-52	330	340	EITLGFVDLLR					Mascot
1433.7495	1433.672	-0.0775	-54	310	324	LSGGDHVHAGTVVGK	71	99.999			Mascot
1433.7495	1433.672	-0.0775	-54	310	324	LSGGDHVHAGTVVGK					Mascot
1451.6219	1451.5431	-0.0788	-54	192	203	DDENVNSQPFMR					Mascot
1465.7546	1465.6755	-0.0791	-54	137	149	TFQGPPHGIQVER					Mascot
1467.6168	1467.5345	-0.0823	-56	192	203	DDENVNSQPFMR			Oxidation (M)[11]		Mascot
1488.6926	1488.6111	-0.0815	-55	454	465	EIKFEFEAMDTL			Oxidation (M)[9]		Mascot
1546.7358	1546.65	-0.0858	-55	441	453	WSPELAAACEVWK			Carbamidomethyl (C)[9]		Mascot
2169.9868	2169.8643	-0.1225	-56	185	203	GGLDFTKDDENVNSQPFMR					Mascot
2185.9819	2185.8582	-0.1237	-57	185	203	GGLDFTKDDENVNSQPFMR			Oxidation (M)[18]		Mascot
3038.45	3038.2954	-0.1546	-51	249	275	ELGVPIVMHDYLTGGFTANTSLAHYCR			Carbamidomethyl (C)[26], Oxidation (M)[8]		Mascot

Rank	Protein Name	Accession No	Protein MW	Protein PI	Pep. Count	Protein Score	Protein Score C. I. %	Total Ion Score	Total Ion C. I. %
6	Ribulose bisphosphate carboxylase large chain (Fragment) OS=Ilex crenata GN=rbcL PE=3 SV=1	RBL_ILECR	51518	6.1	19	347	100	208	100

Peptide Information

Calc. Mass	Obsrv. Mass	± da	± ppm	Start Seq.	End Seq.	Sequence	Ion Score	C. I. %	Modification	Rank	Result Type
830.4366	830.3947	-0.0419	-50	430	436	EGNEIIR					Mascot
830.4366	830.3947	-0.0419	-50	430	436	EGNEIIR	45	99.53			Mascot
898.41	898.3639	-0.0461	-51	296	302	NHGMHFR					Mascot
910.4451	910.3986	-0.0465	-51	178	184	AVYECLR	21	0	Carbamidomethyl (C)[5]		Mascot
910.4451	910.3986	-0.0465	-51	178	184	AVYECLR			Carbamidomethyl (C)[5]		Mascot
912.4719	912.4184	-0.0535	-59	286	293	AMHAVIDR					Mascot
914.4049	914.3662	-0.0387	-42	296	302	NHGMHFR			Oxidation (M)[4]		Mascot
928.4669	928.4195	-0.0474	-51	286	293	AMHAVIDR	18	0	Oxidation (M)[2]		Mascot
928.4669	928.4195	-0.0474	-51	286	293	AMHAVIDR			Oxidation (M)[2]		Mascot
1021.5312	1021.4796	-0.0516	-51	23	31	DTDILAAFR					Mascot
1023.4741	1023.421	-0.0531	-52	341	348	DDFVEKDR					Mascot
1023.4741	1023.421	-0.0531	-52	341	348	DDFVEKDR	59	99.982			Mascot
1116.6194	1116.5267	-0.0927	-83	412	421	VALEACVKAR			Carbamidomethyl (C)[6]		Mascot
1116.6194	1116.5267	-0.0927	-83	412	421	VALEACVKAR	65	99.995	Carbamidomethyl (C)[6]		Mascot
1154.5636	1154.4729	-0.0907	-79	294	302	QKNHGMHFR					Mascot
1170.5585	1170.5757	0.0172	15	294	302	QKNHGMHFR			Oxidation (M)[6]		Mascot
1187.6643	1187.6018	-0.0625	-53	276	285	DNGLLLHIHR					Mascot
1245.6337	1245.5686	-0.0651	-52	208	217	FLFCAEALFK			Carbamidomethyl (C)[4]		Mascot
1275.7307	1275.6642	-0.0665	-52	330	340	EITLGFVDLLR					Mascot
1451.6219	1451.5431	-0.0788	-54	192	203	DDENVNSQPFMR					Mascot
1465.7216	1465.6755	-0.0461	-31	310	324	MSGGDHIHAGTVVGK					Mascot
1467.6168	1467.5345	-0.0823	-56	192	203	DDENVNSQPFMR			Oxidation (M)[11]		Mascot
1471.7137	1471.6204	-0.0933	-63	454	465	EIKFEFQAMDTL					Mascot
1546.7358	1546.65	-0.0858	-55	441	453	WSPELAAACEVWK			Carbamidomethyl (C)[9]		Mascot
1812.8326	1812.6766	-0.156	-86	9	22	EYKLNYYTPDYDTK					Mascot
2050.0134	2049.8789	-0.1345	-66	310	329	MSGGDHIHAGTVVGKLEGER					Mascot
2169.9868	2169.8643	-0.1225	-56	185	203	GGLDFTKDDENVNSQPFMR					Mascot
2185.9819	2185.8582	-0.1237	-57	185	203	GGLDFTKDDENVNSQPFMR			Oxidation (M)[18]		Mascot
3038.45	3038.2954	-0.1546	-51	249	275	ELGVPIVMHDYLTGGFTANTSLAHYCR			Carbamidomethyl (C)[26], Oxidation (M)[8]		Mascot

Rank	Protein Name	Accession No	Protein MW	Protein PI	Pep. Count	Protein Score	Protein Score C. I. %	Total Ion Score	Total Ion C. I. %
7	Ribulose bisphosphate carboxylase large chain OS=Vigna unguiculata GN=rbcL PE=3 SV=1	RBL_VIGUN	52468.4	6.23	19	340	100	208	100

Peptide Information

Calc. Mass	Obsrv. Mass	± da	± ppm	Start Seq.	End Seq.	Sequence	Ion Score	C. I. %	Modification	Rank	Result Type
830.4366	830.3947	-0.0419	-50	440	446	EGNEIIR					Mascot
830.4366	830.3947	-0.0419	-50	440	446	EGNEIIR	45	99.53			Mascot
856.3546	856.4824	0.1278	149	467	473	FEFPAMD					Mascot
898.41	898.3639	-0.0461	-51	306	312	NHGMHFR					Mascot
910.4451	910.3986	-0.0465	-51	188	194	AVYECLR	21	0	Carbamidomethyl (C)[5]		Mascot
910.4451	910.3986	-0.0465	-51	188	194	AVYECLR			Carbamidomethyl (C)[5]		Mascot
912.4719	912.4184	-0.0535	-59	296	303	AMHAVIDR					Mascot
914.4049	914.3662	-0.0387	-42	306	312	NHGMHFR			Oxidation (M)[4]		Mascot
928.4669	928.4195	-0.0474	-51	296	303	AMHAVIDR	18	0	Oxidation (M)[2]		Mascot
928.4669	928.4195	-0.0474	-51	296	303	AMHAVIDR			Oxidation (M)[2]		Mascot
962.4788	962.4269	-0.0519	-54	228	236	SQAETGEIK					Mascot
1021.5312	1021.4796	-0.0516	-51	33	41	DTDILAAFR					Mascot
1023.4741	1023.421	-0.0531	-52	351	358	DDFVEKDR					Mascot
1023.4741	1023.421	-0.0531	-52	351	358	DDFVEKDR	59	99.982			Mascot
1116.6194	1116.5267	-0.0927	-83	422	431	VALEACVKAR			Carbamidomethyl (C)[6]		Mascot
1116.6194	1116.5267	-0.0927	-83	422	431	VALEACVKAR	65	99.995	Carbamidomethyl (C)[6]		Mascot
1154.5636	1154.4729	-0.0907	-79	304	312	QKNHGMHFR					Mascot
1170.5585	1170.5757	0.0172	15	304	312	QKNHGMHFR			Oxidation (M)[6]		Mascot
1187.6643	1187.6018	-0.0625	-53	286	295	DNGLLLHIHR					Mascot
1245.6337	1245.5686	-0.0651	-52	218	227	FLFCAEAIFK			Carbamidomethyl (C)[4]		Mascot
1451.6219	1451.5431	-0.0788	-54	202	213	DDENVNSQPFMR					Mascot
1465.7546	1465.6755	-0.0791	-54	147	159	TFQGPPHGIQVER					Mascot
1467.6168	1467.5345	-0.0823	-56	202	213	DDENVNSQPFMR			Oxidation (M)[11]		Mascot
1546.7358	1546.65	-0.0858	-55	451	463	WSPELAAACEVWK			Carbamidomethyl (C)[9]		Mascot
1812.7714	1812.6766	-0.0948	-52	237	252	GHYLNATAGTCEEMMK			Carbamidomethyl (C)[11]		Mascot
1828.7662	1828.6584	-0.1078	-59	237	252	GHYLNATAGTCEEMMK			Carbamidomethyl (C)[11], Oxidation (M)[14]		Mascot
1844.7611	1844.6625	-0.0986	-53	237	252	GHYLNATAGTCEEMMK			Carbamidomethyl (C)[11], Oxidation (M)[14,15]		Mascot
1984.8673	1984.7566	-0.1107	-56	237	253	GHYLNATAGTCEEMMKR			Carbamidomethyl (C)[11], Oxidation (M)[14]		Mascot
2000.8623	2000.744	-0.1183	-59	237	253	GHYLNATAGTCEEMMKR			Carbamidomethyl (C)[11], Oxidation (M)[14,15]		Mascot
2169.9868	2169.8643	-0.1225	-56	195	213	GGLDFTKDDENVNSQPFMR					Mascot
2185.9819	2185.8582	-0.1237	-57	195	213	GGLDFTKDDENVNSQPFMR			Oxidation (M)[18]		Mascot
3038.45	3038.2954	-0.1546	-51	259	285	ELGVPIVMHDYLTGGFTANTSLAHYCR			Carbamidomethyl (C)[26], Oxidation (M)[8]		Mascot

Rank	Protein Name	Accession No	Protein MW	Protein PI	Pep. Count	Protein Score	Protein Score C. I. %	Total Ion Score	Total Ion C. I. %
8	Ribulose bisphosphate carboxylase large chain (Fragment) OS=Deppea grandiflora GN=rbcL PE=3 SV=1	RBL_DEPGR	52289.5	6.33	16	335	100	234	100

Peptide Information

Calc. Mass	Obsrv. Mass	± da	± ppm	Start Seq.	End Seq.	Sequence	Ion Score	C. I. %	Modification	Rank	Result Type
898.41	898.3639	-0.0461	-51	297	303	NHGMHFR					Mascot
910.4451	910.3986	-0.0465	-51	179	185	AVYECLR	21	0	Carbamidomethyl (C)[5]		Mascot
910.4451	910.3986	-0.0465	-51	179	185	AVYECLR			Carbamidomethyl (C)[5]		Mascot
912.4719	912.4184	-0.0535	-59	287	294	AMHAVIDR					Mascot
914.4049	914.3662	-0.0387	-42	297	303	NHGMHFR			Oxidation (M)[4]		Mascot
928.4669	928.4195	-0.0474	-51	287	294	AMHAVIDR	18	0	Oxidation (M)[2]		Mascot
928.4669	928.4195	-0.0474	-51	287	294	AMHAVIDR			Oxidation (M)[2]		Mascot
1021.5312	1021.4796	-0.0516	-51	24	32	DTDILAAFR					Mascot
1023.4741	1023.421	-0.0531	-52	342	349	DDFVEKDR					Mascot
1023.4741	1023.421	-0.0531	-52	342	349	DDFVEKDR	59	99.982			Mascot
1116.6194	1116.5267	-0.0927	-83	413	422	VALEACVKAR			Carbamidomethyl (C)[6]		Mascot
1116.6194	1116.5267	-0.0927	-83	413	422	VALEACVKAR	65	99.995	Carbamidomethyl (C)[6]		Mascot
1154.5636	1154.4729	-0.0907	-79	295	303	QKNHGMHFR					Mascot
1170.5585	1170.5757	0.0172	15	295	303	QKNHGMHFR			Oxidation (M)[6]		Mascot
1187.6643	1187.6018	-0.0625	-53	277	286	DNGLLLHIHR					Mascot
1245.6337	1245.5686	-0.0651	-52	209	218	FLFCAEALFK			Carbamidomethyl (C)[4]		Mascot
1433.7495	1433.672	-0.0775	-54	311	325	LSGGDHVHAGTVVGK	71	99.999			Mascot
1433.7495	1433.672	-0.0775	-54	311	325	LSGGDHVHAGTVVGK					Mascot
1451.6219	1451.5431	-0.0788	-54	193	204	DDENVNSQPFMR					Mascot
1465.7546	1465.6755	-0.0791	-54	138	150	TFQGPPHGIQVER					Mascot
1467.6168	1467.5345	-0.0823	-56	193	204	DDENVNSQPFMR			Oxidation (M)[11]		Mascot
1546.7358	1546.65	-0.0858	-55	442	454	WSPELAAACEVWK			Carbamidomethyl (C)[9]		Mascot
1812.7714	1812.6766	-0.0948	-52	228	243	GHYLNATAGTCEEMMK			Carbamidomethyl (C)[11]		Mascot
1828.7662	1828.6584	-0.1078	-59	228	243	GHYLNATAGTCEEMMK			Carbamidomethyl (C)[11], Oxidation (M)[14]		Mascot
1844.7611	1844.6625	-0.0986	-53	228	243	GHYLNATAGTCEEMMK			Carbamidomethyl (C)[11], Oxidation (M)[14,15]		Mascot
1984.8673	1984.7566	-0.1107	-56	228	244	GHYLNATAGTCEEMMKR			Carbamidomethyl (C)[11], Oxidation (M)[14]		Mascot
2000.8623	2000.744	-0.1183	-59	228	244	GHYLNATAGTCEEMMKR			Carbamidomethyl (C)[11], Oxidation (M)[14,15]		Mascot
2169.9868	2169.8643	-0.1225	-56	186	204	GGLDFTKDDENVNSQPFMR					Mascot
2185.9819	2185.8582	-0.1237	-57	186	204	GGLDFTKDDENVNSQPFMR			Oxidation (M)[18]		Mascot

Rank	Protein Name	Accession No	Protein MW	Protein PI	Pep. Count	Protein Score	Protein Score C. I. %	Total Ion Score	Total Ion C. I. %
9	Ribulose bisphosphate carboxylase large chain OS=Ipomoea purpurea GN=rbcL PE=3 SV=1	RBL_IPOPU	53353.9	6.41	18	333	100	208	100

Peptide Information

Calc. Mass	Obsrv. Mass	± da	± ppm	Start Seq.	End Seq.	Sequence	Ion Score	C. I. %	Modification	Rank	Result Type
830.4366	830.3947	-0.0419	-50	440	446	EGNEIIR					Mascot
830.4366	830.3947	-0.0419	-50	440	446	EGNEIIR	45	99.53			Mascot
898.41	898.3639	-0.0461	-51	306	312	NHGMHFR					Mascot
910.4451	910.3986	-0.0465	-51	188	194	AVYECLR	21	0	Carbamidomethyl (C)[5]		Mascot
910.4451	910.3986	-0.0465	-51	188	194	AVYECLR			Carbamidomethyl (C)[5]		Mascot
912.4719	912.4184	-0.0535	-59	296	303	AMHAVIDR					Mascot
914.4049	914.3662	-0.0387	-42	306	312	NHGMHFR			Oxidation (M)[4]		Mascot
928.4669	928.4195	-0.0474	-51	296	303	AMHAVIDR	18	0	Oxidation (M)[2]		Mascot
928.4669	928.4195	-0.0474	-51	296	303	AMHAVIDR			Oxidation (M)[2]		Mascot
962.4788	962.4269	-0.0519	-54	228	236	SQAETGEIK					Mascot
1021.5312	1021.4796	-0.0516	-51	33	41	DTDILAAFR					Mascot
1023.4377	1023.421	-0.0167	-16	351	358	DDFVEQDR					Mascot
1023.4377	1023.421	-0.0167	-16	351	358	DDFVEQDR	59	99.982			Mascot
1116.583	1116.5267	-0.0563	-50	422	431	VALEACVQAR			Carbamidomethyl (C)[6]		Mascot
1116.583	1116.5267	-0.0563	-50	422	431	VALEACVQAR	65	99.995	Carbamidomethyl (C)[6]		Mascot
1154.5636	1154.4729	-0.0907	-79	304	312	QKNHGMHFR					Mascot
1170.5585	1170.5757	0.0172	15	304	312	QKNHGMHFR			Oxidation (M)[6]		Mascot
1187.6643	1187.6018	-0.0625	-53	286	295	DNGLLLHIHR					Mascot
1275.7307	1275.6642	-0.0665	-52	340	350	EITLGFVDLLR					Mascot
1451.6219	1451.5431	-0.0788	-54	202	213	DDENVNSQPFMR					Mascot
1465.7546	1465.6755	-0.0791	-54	147	159	TFQGPPHGIQVER					Mascot
1467.6168	1467.5345	-0.0823	-56	202	213	DDENVNSQPFMR			Oxidation (M)[11]		Mascot
1546.7358	1546.65	-0.0858	-55	451	463	WSPELAAACEVWK			Carbamidomethyl (C)[9]		Mascot
1812.7714	1812.6766	-0.0948	-52	237	252	GHYLNATAGTCEEMMK			Carbamidomethyl (C)[11]		Mascot
1828.7662	1828.6584	-0.1078	-59	237	252	GHYLNATAGTCEEMMK			Carbamidomethyl (C)[11], Oxidation (M)[14]		Mascot
1844.7611	1844.6625	-0.0986	-53	237	252	GHYLNATAGTCEEMMK			Carbamidomethyl (C)[11], Oxidation (M)[14,15]		Mascot
1984.8673	1984.7566	-0.1107	-56	237	253	GHYLNATAGTCEEMMKR			Carbamidomethyl (C)[11], Oxidation (M)[14]		Mascot
2000.8623	2000.744	-0.1183	-59	237	253	GHYLNATAGTCEEMMKR			Carbamidomethyl (C)[11], Oxidation (M)[14,15]		Mascot
2169.9868	2169.8643	-0.1225	-56	195	213	GGLDFTKDDENVNSQPFMR					Mascot
2185.9819	2185.8582	-0.1237	-57	195	213	GGLDFTKDDENVNSQPFMR			Oxidation (M)[18]		Mascot
3038.45	3038.2954	-0.1546	-51	259	285	ELGVPIVMHDYLTGGFTANTSLAHYCR			Carbamidomethyl (C)[26], Oxidation (M)[8]		Mascot

Rank	Protein Name	Accession No	Protein MW	Protein PI	Pep. Count	Protein Score	Protein Score C. I. %	Total Ion Score	Total Ion C. I. %
10	Ribulose bisphosphate carboxylase large chain OS=Populus tremuloides GN=rbcL PE=3 SV=1	RBL_POPTM	52703.5	5.91	18	332	100	208	100

Peptide Information

Calc. Mass	Obsrv. Mass	± da	± ppm	Start Seq.	End Seq.	Sequence	Ion Score	C. I. %	Modification	Rank	Result Type
830.4366	830.3947	-0.0419	-50	440	446	EGNEIIR					Mascot
830.4366	830.3947	-0.0419	-50	440	446	EGNEIIR	45	99.53			Mascot
880.4536	880.4147	-0.0389	-44	306	312	NHGIHFR					Mascot
910.4451	910.3986	-0.0465	-51	188	194	AVYECLR	21	0	Carbamidomethyl (C)[5]		Mascot
910.4451	910.3986	-0.0465	-51	188	194	AVYECLR			Carbamidomethyl (C)[5]		Mascot
912.4719	912.4184	-0.0535	-59	296	303	AMHAVIDR					Mascot
928.4669	928.4195	-0.0474	-51	296	303	AMHAVIDR	18	0	Oxidation (M)[2]		Mascot
928.4669	928.4195	-0.0474	-51	296	303	AMHAVIDR			Oxidation (M)[2]		Mascot
1021.5312	1021.4796	-0.0516	-51	33	41	DTDILAAFR					Mascot
1023.4741	1023.421	-0.0531	-52	351	358	DDFVEKDR					Mascot
1023.4741	1023.421	-0.0531	-52	351	358	DDFVEKDR	59	99.982			Mascot
1102.4762	1102.4115	-0.0647	-59	467	475	FEFEAMDTL					Mascot
1116.583	1116.5267	-0.0563	-50	422	431	VALEACVQAR			Carbamidomethyl (C)[6]		Mascot
1116.583	1116.5267	-0.0563	-50	422	431	VALEACVQAR	65	99.995	Carbamidomethyl (C)[6]		Mascot
	1116.5267										Mascot
1187.6643	1187.6018	-0.0625	-53	286	295	DNGLLLHIHR					Mascot
1245.6433	1245.5686	-0.0747	-60	440	450	EGNEIIREASK					Mascot
1465.7546	1465.6755	-0.0791	-54	147	159	TFQGPPHGIQVER					Mascot
1467.6168	1467.5345	-0.0823	-56	202	213	DDENVNSQPFMR			Oxidation (M)[11]		Mascot
1488.6926	1488.6111	-0.0815	-55	464	475	EIKFEFEAMDTL			Oxidation (M)[9]		Mascot
1546.7358	1546.65	-0.0858	-55	451	463	WSPELAAACEVWK			Carbamidomethyl (C)[9]		Mascot
1812.7714	1812.6766	-0.0948	-52	237	252	GHYLNATAGTCEEMMK			Carbamidomethyl (C)[11]		Mascot
1828.7662	1828.6584	-0.1078	-59	237	252	GHYLNATAGTCEEMMK			Carbamidomethyl (C)[11], Oxidation (M)[14]		Mascot
1844.7611	1844.6625	-0.0986	-53	237	252	GHYLNATAGTCEEMMK			Carbamidomethyl (C)[11], Oxidation (M)[14,15]		Mascot
1984.8673	1984.7566	-0.1107	-56	237	253	GHYLNATAGTCEEMMKR			Carbamidomethyl (C)[11], Oxidation (M)[14]		Mascot
2000.8623	2000.744	-0.1183	-59	237	253	GHYLNATAGTCEEMMKR			Carbamidomethyl (C)[11], Oxidation (M)[14,15]		Mascot
2169.9868	2169.8643	-0.1225	-56	195	213	GGLDFTKDDENVNSQPFMR					Mascot
2185.9819	2185.8582	-0.1237	-57	195	213	GGLDFTKDDENVNSQPFMR			Oxidation (M)[18]		Mascot
3038.45	3038.2954	-0.1546	-51	259	285	ELGVPIVMHDYLTGGFTANTSLAHYCR			Carbamidomethyl (C)[26], Oxidation (M)[8]		Mascot

Table S5

Table S5: Protein-protein network data

Spot no.	Protein name	Uniprot ID	Uniprot Accession no.	Arabidopsis homolog Accession no.	Protein identity
1	**Acyl-[acyl-carrier-protein] desaturase 6**	STAD6_ORYSJ	Q0J7E4	O22832	44.55%
2	**lipoxygenase**	Q43440_SOYBN	Q43440	Q06327	53.95%
3	**Ribulose bisphosphate carboxylase large chain**	RBL_LIQST	Q01873	O03042	94.78%
4	**Transketolase, chloroplastic**	TKTC_SPIOL	O20250	Q8RWV0	79.63%
7	**Stromal 70 kDa heat shock-related protein**	HSP7S_PEA	Q02028	Q9S732	13.06%
9	**ATP-dependent zinc metalloprotease FTSH 8**	FTSH8_ARATH	Q8W585	Q8W585	100%
13	**RuBisCO large subunit-binding protein subunit beta**	RUBB_PEA	P08927	P21240	84.91%
16	**ATP synthase subunit alpha**	ATPA_SOYBN	Q2PMS8	P92549	55.96%
19	**ATP synthase subunit beta**	ATPB_SOYBN	Q2PMV0	P19366	94.58%
28	**Ribulose bisphosphate carboxylase/oxygenase activase**	RCA_MALDO	Q40281	P10896	73.90%
29	**Elongation factor Tu, chloroplastic**	EFTU1_SOYBN	Q43467	P17745	82.33%
33	**Glutamine synthetase leaf isozyme**	GLNA4_PHAVU	P15102	Q8LCE1	67.60%
34	**S-adenosylmethionine synthase 4**	METK4_VITVI	A7PRJ6	Q9LUT2	91.35%
35	**Probable calcium-binding protein CML33**	CML33_ARATH	Q9SRP4	Q9SRP4	100%
37	**Sedoheptulose-1,7-bisphosphatase**	S17P_SPIOL	O20252	P46283	78.43%
42	**Phosphoglycerate kinase**	PGKH_WHEAT	P12782	Q9LD57	79.38%
44	**Photosystem II stability/assembly factor HCF136**	P2SAF_ARATH	O82660	O82660	100%
46	**Fructose-bisphosphate aldolase 1**	ALFC1_PEA	Q01516	Q9SJU4	75.69%
49	**Malate dehydrogenase 1**	MDHM1_ARATH	Q9ZP06	Q9ZP06	100.00%
50	**Trypsin inhibitor 1**	ITR1_TRIKI	Q43667	Q42328	13%
52	**Chloroplast stem-loop binding protein of 41 kDa b**	CP41B_ARATH	Q9SA52	Q9SA52	100.00%
53	**Ferredoxin--NADP reductase, leaf isozyme 1**	FNRL1_ARATH	Q9FKW6-1	Q9FKW6	100.00%
56	**Oxygen-evolving enhancer protein 1**	PSBO_PEA	P14226	P23321	77.48%
58	**Chlorophyll a-b binding protein of LHCII type I**	CB21_CUCSA	P08221	P04778	83.90%
59	**Chlorophyll a-b binding protein**	CB23_ORYSJ	Q10HD0	P0CJ48	76.12%
60	**Chlorophyll a-b binding protein 13**	CB23_SOLLC	P27489	Q8VZ87	64.36%
61	**2-Cys peroxiredoxin BAS1-like**	BAS1B_ARATH	Q9C5R8	Q9C5R8	100%
62	**Ribulose bisphosphate carboxylase small chain PW9**	RBS2_WHEAT	P26667	P10795	59.24%

**63**	**Ribulose bisphosphate carboxylase small chain 2A**	RBSA_SOLTU	P26575	P10796	74.32%
**64**	**Superoxide dismutase [Fe]**	SODF_SOYBN	P28759	P21276	61.69%
**66**	**Carbonic anhydrase 1**	CAH1_FLALI	P46512	P27140	65.81%
**67**	**RuBisCO-associated protein**	RUAP_SOYBN	P39657	P10798	X
**71**	**Stem 31 kDa glycoprotein**	VSPB_SOYBN	P10743	O49195	36.63%
**73**	**Stem 28 kDa glycoprotein**	VSPA_SOYBN	P15490	O82122	36.67%
**74**	**Oxygen-evolving enhancer protein 2-1**	PSBP1_TOBAC	Q7DM39	Q42029	78.73%
**76**	**Chlorophyll a-b binding protein 6A**	CB11_SOLLC	P12360	Q01667	82.52%
**77**	**Ribulose bisphosphate carboxylase large chain (Fragment)**	RBL_GAREL	P28416	O03042	94.78%
**80**	**Ribosomal protein L7/L12**	Q4BZ06_CROWT	Q4BZ06	O81061	33%
**81**	**Glycine cleavage system H protein 1**	GCSH1_ARATH	P25855	Q9LR30	100%
**87**	**Ribulose bisphosphate carboxylase small chain 1**	RBS1_SOYBN	P00865	P10797	76.92%
